# Supplementary material for: Bacterial Magnetosomes Release Iron Ions and Induce Regulation of Iron Homeostasis in Endothelial Cells
Source: Nanomaterials (Basel). 2022 Nov 13;12(22):3995. doi: 10.3390/nano12223995 (PMC9695978; doi:10.3390/nano12223995)
Supplement: Supplementary file 1 [file nanomaterials-12-03995-s001.zip › nanomaterials-1995717-supplementary.pdf]

Supplementary Materials

Bacterial Magnetosomes Release Iron Ions and Regulation of Iron Homeostasis in Endothelial Cells

Wenjia Lai[1,*], Dan Li[2], Qingsong Wang[3], Yan Ma[4], Jiesheng Tian[5], and Qiaojun Fang[2,*]

1 Division of Nanotechnology Development, National Center for Nanoscience and Technology, Chinese Academy of Sciences, Beijing 100190, China
2 Laboratory of Theoretical and Computational Nanoscience, National Center for Nanoscience andTechnology, Chinese Academy of Sciences, Beijing 100190, China
3 State Key Laboratory of Protein and Plant Gene Research, College of Life Sciences, Peking University, Beijing 100871, China
4 Aviation Service Department, Yantai Engineering & Technology College, Yantai 264006, China
5 State Key Laboratories for Agrobiotechnology, College of Biological Sciences, China Agricultural University, Beijing 100193, China
* Correspondence: laiwj@nanoctr.cn (W.L.); fangqj@nanoctr.cn (Q.F.); Tel.: +86-10-82545693 (W.L.); +86-10-82545562 (Q.F.)

1. EXPERIMENTAL DETAILS

1.1 Protein extraction, ion, TMT labelling, and high pH reversed-phase peptide fractionation

Cells treated with or without MAGs were collected and lysed using an ultrasonic cell disruptor (JY 92-IIN, Scientz, Ningbo, China), then boiled for 5 min to denature proteins and shear deoxyribonucleic acid in a buffer containing 6 M guanidine hydrochloride, 50 mM ris(hydroxymethyl)aminoethane (Tris), 20 mM tris(2-carboxyethyl)phosphine, and 50 mM chloroacetamide (Sigma, Saint Louis, MO, USA) at pH 8.0. Following centrifugation at 18,000 × g for 20 min, the supernatant was collected and precipitated overnight using cold acetone (Fisher, Waltham, MA, USA) at -20ºC. After centrifugation, the protein pellet was washed with cold acetone three times and dissolved in 8 M urea 25 mM NH_4HCO_3. Then, the protein concentration was estimated using a 2D Quant kit (GE Healthcare, Chicago, IL, USA), and samples with up to 100 µg protein from three biological repeats were diluted four folds for

digestion using 25 mM NH_4HCO_3 containing Trypsin/Lys-C Mix (Promega, Madison, WI, USA) at a ratio of 1:50 (micrograms of enzymes to micrograms of protein). Digestion was performed at 37°C overnight, the resulting peptides were acidified with trifluoroacetic acid and desalted using C18 (3M, Bracknell, UK) and dried as described previously [24].

Before mass spectrometry (MS) analysis, the peptides were labelled with Tandem Mass Tag (TMT) 10 plex™ Isobaric Label Reagent Set labelling kit (Thermo, Waltham, MA, USA) according to the manufacturer's instructions (Figure S1). The peptide mixture at each time point was fractionated separately using a reversed-phase C18 column (3M, Bracknell, UK) as described previously with some modifications [69]. Briefly, peptides were loaded at pH 8, and eight fractions were subsequently eluted with buffer solutions (pH 8) containing acetonitrile step gradients (7.5, 10, 12.5, 15, 17.5, 20, 22.5, and 50%). Then, the fractions were dried in a vacuum centrifuge and stored at -80 ºC until liquid chromatograph tandem mass spectrometry (LC-MS/MS) analysis.

1.2 LC-MS/MS

The high-pH fractionated peptides were dissolved in 10 μL 0.2% formic acid and centrifuged at 13000 × g for 10 min before being analysed via LC-MS/MS using Q Exactive mass spectrometer (Thermo Scientific, Odense, Denmark). About 3 μg of peptide was separated using the nEASY LC-1000 liquid chromatography system (Thermo Scientific, Odense, Denmark) with an in-house packed 75 μm inner diameter (ID) × 50 cm capillary column with 2.5 μm Venusil C18 beads (Agela Technologies, Tianjin, China) at a flow rate of 200 nL/min. The column temperature was maintained at 42ºC using an integrated column oven (PRSO-V1, Sonation GmbH, Biberach, Germany). A total of 240 min or 320 min gradient containing acetonitrile at 5 to 32% in 0.1% formic acid was used. The instrument was operated at positive ion mode. Raw data were acquired in profile mode using Xcalibur software (version 3.1) with

data-dependent MS/MS scans (TopN = 15). The target value for the full MS scan was $3e^6$ in the 300–1700 m/z range with a maximum injection time of 50 ms and a resolution of 70,000 at m/z of 200. The isolation window was 1.6 m/z and the normalized collision energy was 32. The MS/MS scan resolution was 35,000 at m/z 200 with an ion-target value of $1e^5$ and a maximum injection time of 100 ms. To avoid the repeated sequencing of a peptide, the exclusion time was set to 45 s. Each fraction was analyzed twice [22].

The peptides samples from purified MAGs were identified using the above method with a little modification. A 145 min gradient containing acetonitrile at 5 to 32% in 0.1% formic acid was used and the normalized collision energy was 28. The MS/MS scan resolution was 17,500 at m/z 200. To ensure data quality, three biological repeats were performed.

1.3 Protein quantification and data analysis

The MS raw data from TMT labeled peptides were identified, quantified using default processing and consensus workflow for tandem mass spectrometry (MS2) TMT quantification method with Thermo software Proteome Discoverer (version 2.2) against Homo sapiens (Human) database (Uniprot, 88725 entries) with reversed protein sequences and a common contaminants database (247 entries) using the SequestHT search engine. The standard searching parameters were used as follows: a 10 ppm MS1 error tolerance, with 0.02 Da error for MS2 product ions on the Q Exactive. Trypsin was set as the enzyme, allowing for two missed cleavages. Methionine oxidation was set as a variable modification, and carbamidomethyl on cysteines, TMT6 plex on lysine, and peptide N-terminus were set as static modifications for all searches. False discovery rate was set to 0.01 for peptide database search.

The MS raw data of peptides isolated from MAGs were submitted to the MaxQuant software with label-free quantification workflow using the Andromeda search engine by searching against

a *Magnetospirillum gryphiswaldense* MSR-1 database (Uniprot, 4399 entries). Proteins with fewer than two unique peptides in each biological repeat were eliminated and the relative abundance of each protein was estimated as described [22].

References in the Manuscript

24. Lai, W.; Wang, Q.; Li, L.; Hu, Z.; Chen, J.; Fang, Q. Interaction of gold and silver nanoparticles with human plasma: Analysis of protein corona reveals specific binding patterns. *Colloids Surf. B* **2017**, *152*, 317–325.

69. Xiong L, Zhang L, Yang Y, Li N, Lai W, Wang F, et al. ER complex proteins are required for rhodopsin biosynthesis and photoreceptor survival in Drosophila and mice. *Cell Death Differ*. **2020,** *27,* 646–661.

22. Lai, W.; Li, D.; Wang, Q.; Nan, X.; Xiang, Z.; Ma, Y.; Liu, Y.; Chen, J.; Tian, J.; Fang, Q. A Protein Corona Adsorbed to a Bacterial Magnetosome Affects Its Cellular Uptake. *Int. J Nanomed.* **2020**, *15*, 1481–1498.

2. SUPPLEMENTARY FIRUGRES AND TABLES

Figure S1. Experiment workflow for liquid chromatograph tandem mass spectrometry (LC-MS/MS) sample preparation of Tandem Mass Tag (TMT) labeled magnetosomes (MAGs) treated cells and control cells from different time points as indicated.

Figure S2. Quantitative comparison of Western blot results using densitometric analysis.

Figure S3. Representative transmission electron microscope (TEM) images showing the nanoparticles uptake by ECs.

Figure S4. Venn plot showing numbers of identified proteins overlap at different time points (on day 1, 4, 7, and 10).

Figure S5. Bioinformatic enrichment analyses of differentially expressed proteins of MAG-treated ECs.

Table S1. All identified proteins from isolated MAGs analyzed by LC-MS/MS.

Table S2. MAG intrinsic membrane proteins identified by proteomic analysis.

Table S3. Corona protein amount on the nanoparticles measured by BCA assay.

Table S4. Zeta potential characterization of MAGs and IONPs.

Table S5. Abundance ratios of all identified proteins from TMT-labeled quantitative proteomics of ECs treated with MAGs at 4 timepoints.

Table S6. The ratios of 158 differential expressed proteins identified by LC-MS/MS from MAG-treated ECs at 4 time points.

Table S7. GO Cellular Component enrichment of the differential expressed proteins from 4 time points of EC cells under MAGs treatment.

Table S8. Function and pathway enrichment of the differential expressed proteins from 4 time points of EC cells under MAGs treatment.

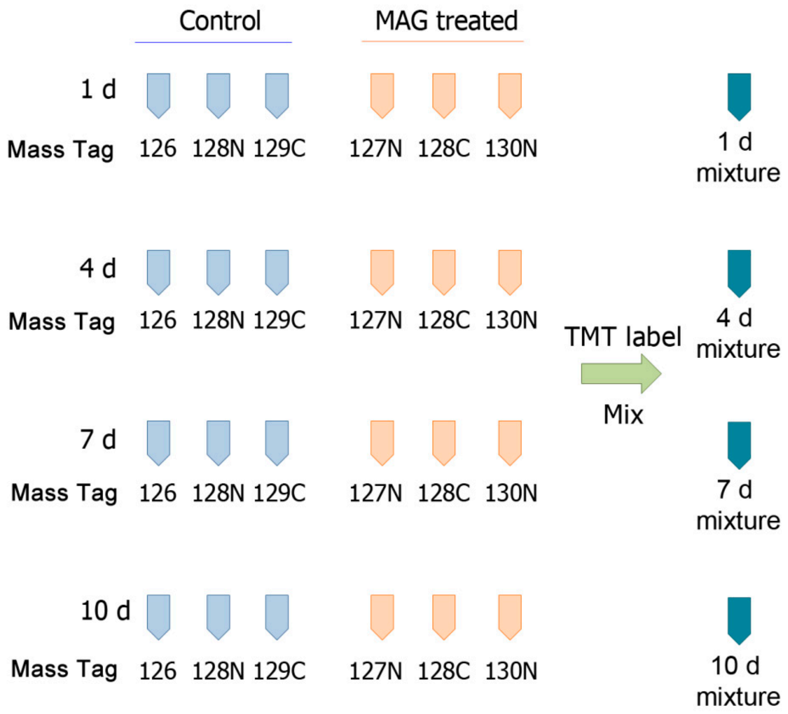

Figure S1. Experiment workflow for liquid chromatograph-tandem mass spectrometry (LC-MS/MS) sample preparation of Tandem Mass Tag (TMT) labeled magnetosomes (MAGs) treated cells and control cells from different timepoints, as indicated. The endothelial cells (ECs) were treated with or without MAGs at different timepoints (on days 1, 4, 7, and 10). For each timepoint, the peptide samples were labeled by mass tag label reagent 126, 128N, 129C, 127N, 128C, and 130N, according to the instruction by the manufacturer. The labeled samples were pooled into final four TMT sets according to timepoints for LC-MS/MS analysis. Control, ECs without MAGs treatment; MAG, ECs treated with MAGs at concentration of 10 µg Fe/mL.

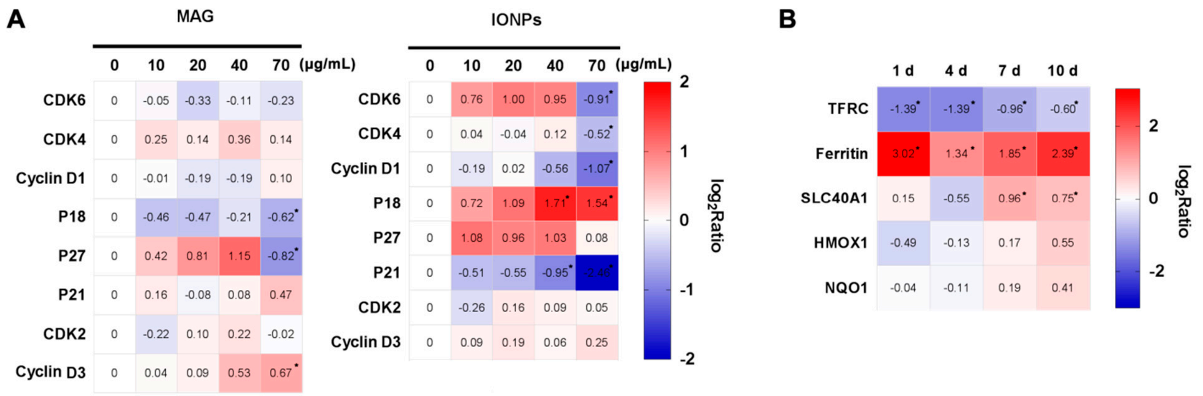

Figure S2. Quantitative comparison of Western blot results, using densitometric analysis. (A) Heatmap showing the logarithm of averaged expression ratios (log_2Ratio) of cell-cycle-related proteins in ECs treated with MAGs or amine magnetic iron oxide nanoparticles (IONPs) for 48 h over a dose range of 0-70 µg Fe/mL. The intensities of the bands from each protein were normalized with β-actin, and for each protein, the mean of ration from control (0 µg/mL) was set at 1. The log_2Ratios of proteins are labeled in each cell in the heatmap. CDK, cyclin-dependent kinase. * *p*-value < 0.05. (B) Heatmap showing the logarithm of averaged expression ratios (log_2Ratios) of proteins that are essential for limiting oxidative damage and iron metabolism in ECs treated with MAGs. The intensities of the bands from each protein were normalized with GAPDH. For each protein, the expression ratio was counted by comparing with its control (ECs without MAGs treatment) at days 1, 4, 7, and 10, respectively. * *p*-value < 0.05; GAPDH, human glyceraldehyde-3-phosphate dehydrogenase.

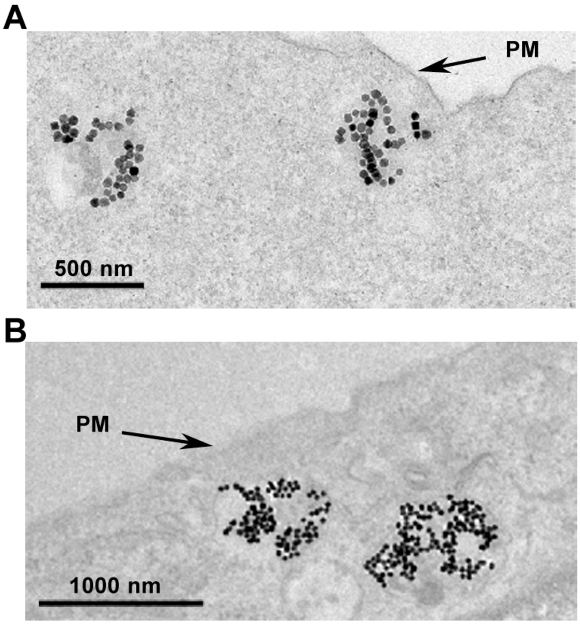

Figure S3. Representative transmission electron microscope (TEM) images showing the nanoparticles' uptake by ECs. After the incubation, the MAGs (A) and IONPs (B) were already internalized into vesicles near the plasma membrane. PM, plasma membrane.

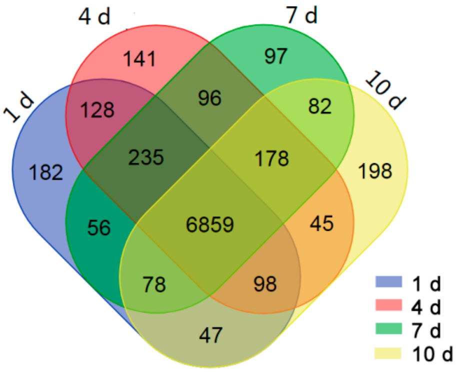

Figure S4. Venn plot showing numbers of identified proteins overlap at different timepoints (on days 1, 4, 7, and 10). The numbers of identified proteins from different timepoints are shown in different colors. A total of 8520 proteins were identified and quantified; 6859 proteins were shared at different timepoints (d, day).

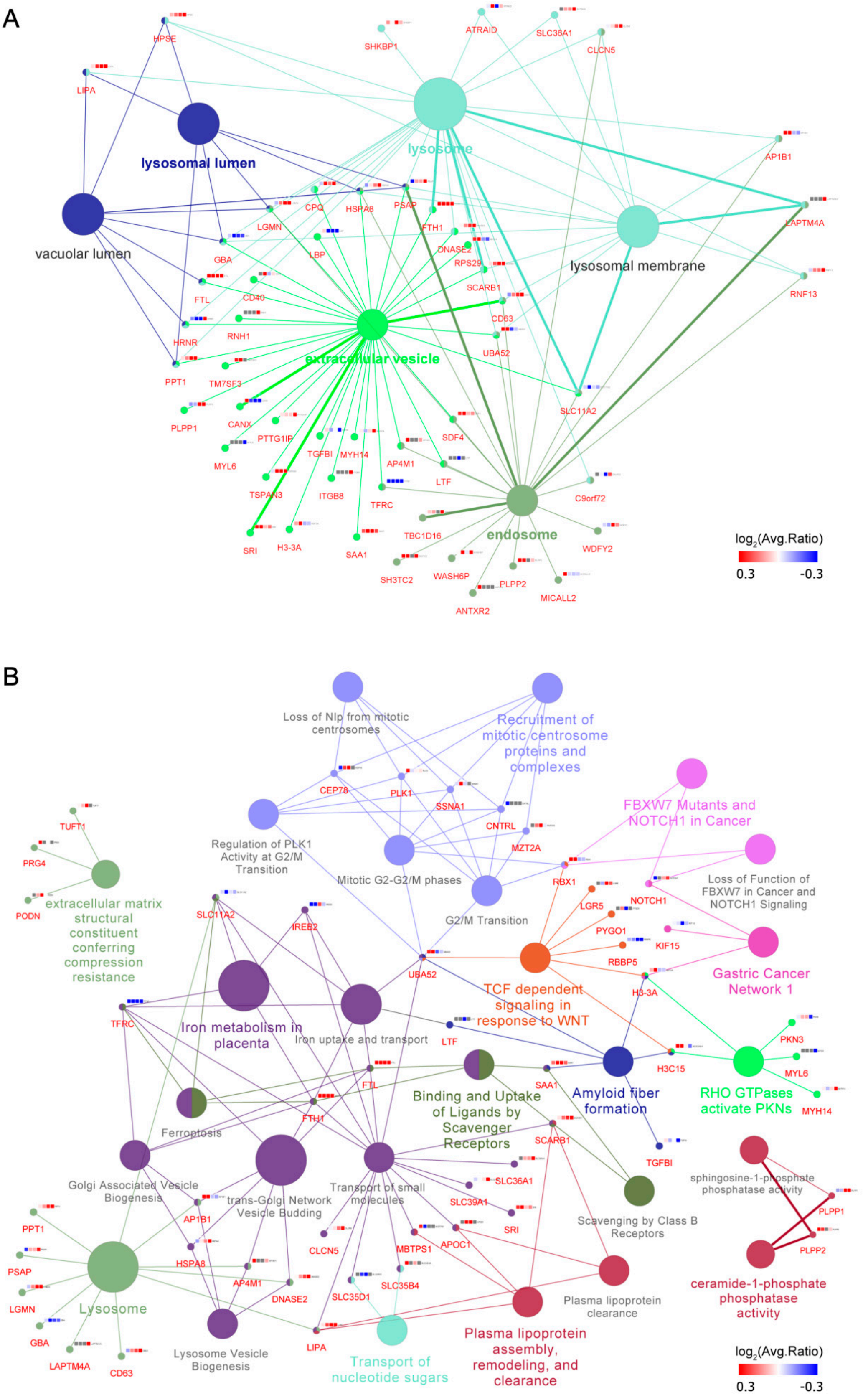
A
HPSE
LIPA
lysosomal lumen
vacuolar lumen
SHKBP1
ATRAID
SLC36A1
CLCN5
lysosome
AP1B1
LAPTM4A
lysosomal membrane
RNF13
CPQ
HSPA8
PSAP
LGMN
FTH1
DNASE2
LBP
GBA
RPS29
SCARB1
FTL
CD40
CD63
HRNR
RNH1
UBA52
PPT1
TM7SF3
extracellular vesicle
SLC11A2
PLPP1
CANX
PTTG1IP
SDF4
MYL6
TGFBI
MYH14
AP4M1
LTF
TSPAN3
ITGB8
TFRC
C9orf72
SRI
H3-3A
SAA1
TBC1D16
endosome
WDFY2
SH3TC2
WASH6P
PLPP2
MICALL2
ANTXR2
log2(Avg.Ratio)
0.3
-0.3
B
Loss of Nlp from mitotic centrosomes
Recruitment of mitotic centrosome proteins and complexes
CEP78
PLK1
SSNA1
CNTRL
MZT2A
Regulation of PLK1 Activity at G2/M Transition
Mitotic G2-G2/M phases
G2/M Transition
FBXW7 Mutants and NOTCH1 in Cancer
Loss of Function of FBXW7 in Cancer and NOTCH1 Signaling
RBX1
LGR5
NOTCH1
PYGO1
KIF15
RBBP5
H3-3A
Gastric Cancer Network 1
TUFT1
PRG4
PODN
extracellular matrix structural constituent conferring compression resistance
SLC11A2
IREB2
UBA52
TFRC
Iron metabolism in placenta
Iron uptake and transport
LTF
TCF dependent signaling in response to WNT
Amyloid fiber formation
H3C15
RHO GTPases activate PKNs
PKN3
MYL6
MYH14
FTL
SAA1
Binding and Uptake of Ligands by Scavenger Receptors
Ferroptosis
FTH1
SCARB1
TGFBI
Golgi Associated Vesicle Biogenesis
trans-Golgi Network Vesicle Budding
Transport of small molecules
SLC36A1
SLC39A1
SRI
Scavenging by Class B Receptors
sphingosine-1-phosphate phosphatase activity
PLPP1
PLPP2
ceramide-1-phosphate phosphatase activity
PPT1
PSAP
LGMN
GBA
LAPTM4A
CD63
Lysosome
AP1B1
HSPA8
AP4M1
DNASE2
CLCN5
MBTPS1
APOC1
SLC35B4
SLC35D1
LIPA
Lysosome Vesicle Biogenesis
Transport of nucleotide sugars
Plasma lipoprotein clearance
Plasma lipoprotein assembly, remodeling, and clearance
log2(Avg.Ratio)
0.3
-0.3

Figure S5. Bioinformatic enrichment analyses of differentially expressed proteins of MAG-treated ECs. Each protein is represented by a gene name; the superscript colors on the gene name show the protein expression ratios at days 1, 4, 7, and 10 (red, upregulated; blue, downregulated). The colors for enriched terms refer to the groups of terms. Terms with p-value < 0.05 are shown in the networks. The p-value and details of every enriched term are listed in Supplementary Table S7 and S8. (A) Network showing the enriched subcellular location terms. Enriched terms were obtained from the GO Cellular Component database. (B) Network showing the enriched functions and pathway terms. Enriched terms were obtained from four databases (KEGG pathways, GO Molecular Function, Wiki Pathways, and REACTOME Pathways).
