# Supplementary material for: Bacterial Magnetosomes Release Iron Ions and Induce Regulation of Iron Homeostasis in Endothelial Cells
Source: Nanomaterials (Basel). 2022 Nov 13;12(22):3995. doi: 10.3390/nano12223995 (PMC9695978; doi:10.3390/nano12223995)
Supplement: Supplementary file 1 [file nanomaterials-12-03995-s001.zip › Table S1.pdf]

Table S1. All identified proteins from isolated MAG analyzed by LC-MS/MS.

Protein IDs	Unique peptides	Unique sequence coverage [%]	Mol. weight [kDa]	Relative abundance (%)	iBAQ 1	iBAQ 2	iBAQ 3	MS/MS Count	Gene name	Protein description
V6F534_9PROT	13	68.8	12.39	18.96	1.3E+10	1.7E+10	1.5E+10	714	mamC	Magnetosome protein MamC
V6F5K9_9PROT	18	79.9	14.83	12.37	9.0E+09	1.0E+10	1.1E+10	1404	mms6	Magnetosome protein mms6
V6EVY5_9PROT	21	93.5	10.48	7.41	6.1E+09	5.6E+09	6.2E+09	143	rpsS	30S ribosomal protein S19
V6F244_9PROT	17	66.4	18.72	6.65	6.0E+09	5.1E+09	5.0E+09	80	mms5	Putative Magnetosome protein MamD-like
V6EZM6_9PROT	13	92.7	6.32	5.06	4.1E+09	3.6E+09	4.5E+09	80	rpmG	50S ribosomal protein L33
V6F2D1_9PROT	15	45.9	30.23	3.41	2.6E+09	2.8E+09	2.9E+09	158	mamD	Magnetosome protein MamD
V6F2K4_9PROT	4	13.3	16.81	3.36	2.7E+09	2.8E+09	2.6E+09	13	MGMSRv2__1301	Uncharacterized protein
V6F2H3_9PROT	14	41.9	28.36	2.64	2.2E+09	2.3E+09	1.8E+09	109	mamP	Magnetosome protein MamP, containing PDZ-like domain
V6F2B6_9PROT	32	37.8	78.04	2.29	1.9E+09	1.8E+09	1.8E+09	422	mamE	Magnetosome protein MamE, containing Serine/cysteine peptidase, trypsin-like domain and HrtA/DegP2/Q/S Peptidase S1C domain
V6EZS4_9PROT	10	80.6	8.20	1.92	1.5E+09	1.7E+09	1.5E+09	36	rpsU	30S ribosomal protein S21
V6F2I4_9PROT	37	82.4	23.52	1.91	1.6E+09	1.5E+09	1.5E+09	152	rpsD	30S ribosomal protein S4
V6EZ16_9PROT	16	71.3	11.53	1.89	1.5E+09	1.5E+09	1.6E+09	80	rpsN	30S ribosomal protein S14
V6EYF9_9PROT	16	78	13.94	1.27	1.1E+09	9.9E+08	1.0E+09	64	rpsL	30S ribosomal protein S12
V6EZ21_9PROT	10	80.3	6.91	1.26	1.1E+09	1.0E+09	9.6E+08	39	rpmD	50S ribosomal protein L30
V6F0N0_9PROT	12	60	17.09	1.13	1.1E+09	8.7E+08	7.5E+08	49	rpsI	30S ribosomal protein S9
V6EVK2_9PROT	14	73.7	15.43	1.08	9.0E+08	8.9E+08	8.4E+08	67	rplP	50S ribosomal protein L16
V6F2C2_9PROT	2	7.1	28.22	1.06	9.3E+08	7.8E+08	8.6E+08	10	mamX	Magnetosome protein MamX
V6F1K4_9PROT	6	32.5	14.08	1.03	8.7E+08	8.8E+08	7.5E+08	16	MGMSRv2__2190	Uncharacterized protein
V6EVN2_9PROT	24	88.5	17.75	1.03	8.2E+08	8.3E+08	8.6E+08	97	rpsG	30S ribosomal protein S7
V6F510_9PROT	10	19.5	31.94	0.88	7.5E+08	7.1E+08	6.7E+08	73	mamB	Magnetosome protein MamB, Cation efflux protein
V6F2Z8_9PROT	10	36.4	19.98	0.86	7.3E+08	6.8E+08	6.9E+08	51	MGMSRv2__1633	Uncharacterized protein
V6F2J8_9PROT	17	77.8	10.23	0.80	6.9E+08	6.0E+08	6.4E+08	60	rpsR	30S ribosomal protein S18
V6F631_9PROT	14	60.2	13.55	0.74	5.7E+08	6.4E+08	6.0E+08	49	rplT	50S ribosomal protein L20
V6EVQ3_9PROT	37	86	25.52	0.67	5.2E+08	5.6E+08	5.4E+08	142	rpsC	30S ribosomal protein S3
V6F8E8_9PROT	10	69.1	7.54	0.67	6.1E+08	5.1E+08	5.0E+08	35	rpmI	50S ribosomal protein L35

Protein IDs	Unique peptides	Unique sequence coverage [%]	Mol. weight [kDa]	Relative abundance (%)	iBAQ 1	iBAQ 2	iBAQ 3	MS/MS Count	Gene name	Protein description
V6F515_9PROT	10	19.8	65.38	0.64	4.7E+08	5.5E+08	5.2E+08	86	mamO	Probable membrane transporter protein
V6F401_9PROT	11	75	9.50	0.63	5.5E+08	3.7E+08	6.0E+08	43	hupB	HU, DNA-binding transcriptional regulator, beta subunit
V6F2G8_9PROT	6	28.9	18.71	0.61	6.2E+08	5.0E+08	3.7E+08	53	mamS	Magnetosome protein MamS
V6F519_9PROT	16	29.8	48.52	0.59	3.8E+08	4.4E+08	6.1E+08	69	mamJ	MamJ protein
V6F1F6_9PROT	37	55	39.11	0.58	4.9E+08	4.6E+08	4.5E+08	141	MGMSRv2__0921	Putative RND efflux membrane fusion protein
V6F3U0_9PROT	20	88.2	16.87	0.58	4.9E+08	4.7E+08	4.4E+08	89	rplM	50S ribosomal protein L13
V6EYI8_9PROT	16	66.7	19.37	0.54	4.6E+08	4.3E+08	4.2E+08	64	rplF	50S ribosomal protein L6
V6F1M7_9PROT	35	87.9	37.23	0.53	4.1E+08	4.3E+08	4.3E+08	126	MGMSRv2__1178	Putative Multidrug resistance protein mdtA
V6EW00_9PROT	15	74.4	9.22	0.53	4.5E+08	4.3E+08	3.9E+08	81	rpsQ	30S ribosomal protein S17
V6F5T4_9PROT	8	64.9	10.94	0.49	3.8E+08	3.9E+08	4.1E+08	34	rpsO	30S ribosomal protein S15
V6F8G3_9PROT	12	68.5	13.59	0.45	3.8E+08	3.5E+08	3.5E+08	49	rpsP	30S ribosomal protein S16
V6EYZ5_9PROT	7	56.5	7.71	0.43	3.7E+08	3.2E+08	3.4E+08	17	rpmC	50S ribosomal protein L29
V6EVR6_9PROT	19	90.6	11.29	0.42	3.7E+08	3.1E+08	3.3E+08	70	rplX	50S ribosomal protein L24
V6F235_9PROT	10	24.8	34.49	0.40	3.0E+08	3.5E+08	3.3E+08	81	mamM	Magnetosome protein MamM, containing Cation efflux protein
V6EYJ6_9PROT	15	64.7	15.37	0.40	3.3E+08	3.2E+08	3.2E+08	57	rplQ	50S ribosomal protein L17
V6EZ38_9PROT	24	46.2	28.32	0.39	3.4E+08	3.0E+08	3.1E+08	94	rpsB	30S ribosomal protein S2
V6F989_9PROT	11	62.2	9.72	0.36	3.3E+08	2.7E+08	2.7E+08	37	rpsT	30S ribosomal protein S20
V6F5I9_9PROT	12	35.6	18.88	0.35	3.0E+08	2.5E+08	2.9E+08	51	mamT	MamT protein
V6EVX0_9PROT	13	66.7	17.69	0.34	2.5E+08	2.7E+08	3.0E+08	40	rplJ	50S ribosomal protein L10
V6F742_9PROT	12	82.6	11.60	0.33	2.8E+08	2.8E+08	2.4E+08	40	MGMSRv2__3982	Uncharacterized protein
V6EW21_9PROT	14	69.7	14.35	0.32	2.6E+08	2.6E+08	2.6E+08	42	rpsH	30S ribosomal protein S8
V6EVL2_9PROT	23	84	14.08	0.32	2.8E+08	2.5E+08	2.5E+08	88	rpsM	30S ribosomal protein S13
V6F8M8_9PROT	23	79.9	19.18	0.32	2.5E+08	2.6E+08	2.6E+08	72	atpH	ATP synthase subunit delta
V6EVS7_9PROT	14	77.5	12.84	0.31	2.4E+08	2.5E+08	2.5E+08	45	rplR	50S ribosomal protein L18
V6EW25_9PROT	8	50.9	16.21	0.29	2.7E+08	2.0E+08	2.3E+08	26	rplO	50S ribosomal protein L15
V6F5J2_9PROT	24	84.8	24.01	0.26	2.1E+08	2.1E+08	2.1E+08	67	mamA	Magnetosome protein MamA

Protein IDs	Unique peptides	Unique sequence coverage [%]	Mol. weight [kDa]	Relative abundance (%)	iBAQ 1	iBAQ 2	iBAQ 3	MS/MS Count	Gene name	Protein description
V6EY28_9PROT	7	77.6	13.02	0.26	2.1E+08	2.1E+08	2.1E+08	23	MGMSRv2__0832	Putative NADH dehydrogenase [ubiquinone] 1 alpha subcomplex subunit 12
V6F0E4_9PROT	9	64	9.58	0.26	2.2E+08	2.2E+08	1.8E+08	23	rpmA	50S ribosomal protein L27
V6F797_9PROT	6	32.1	15.44	0.24	1.8E+08	2.1E+08	1.9E+08	27	mucR	Transcriptional regulatory protein mucR
V6EYH2_9PROT	10	73.4	14.24	0.23	2.0E+08	1.9E+08	1.8E+08	41	rplV	50S ribosomal protein L22
V6EVL0_9PROT	27	73.4	22.51	0.22	1.9E+08	1.8E+08	1.8E+08	94	rpsE	30S ribosomal protein S5
V6EVK6_9PROT	22	80.6	20.82	0.20	1.5E+08	1.5E+08	1.8E+08	66	rplE	50S ribosomal protein L5
V6F254_9PROT	3	27	12.34	0.20	1.6E+08	1.7E+08	1.5E+08	20	mamF	Magnetosome protein MamF
V6F1J6_9PROT	7	75	9.49	0.20	1.7E+08	1.5E+08	1.5E+08	34	hfq	RNA-binding protein Hfq
V6F6K6_9PROT	6	33.3	10.15	0.19	1.4E+08	1.5E+08	1.6E+08	22	MGMSRv2__3864	Uncharacterized protein
V6F389_9PROT	9	71	10.87	0.17	1.2E+08	1.4E+08	1.5E+08	22	rpmB	50S ribosomal protein L28
V6EYI4_9PROT	10	70.5	13.31	0.16	1.4E+08	1.3E+08	1.3E+08	30	rplN	50S ribosomal protein L14
V6EYY3_9PROT	28	78.2	30.47	0.16	1.3E+08	1.3E+08	1.3E+08	95	rplB	50S ribosomal protein L2
V6F0H0_9PROT	19	82.8	17.45	0.16	1.4E+08	1.3E+08	1.2E+08	50	omp	Outer membrane lipoprotein omp16 homolog
V6EW10_9PROT	11	61.5	11.40	0.15	1.2E+08	1.2E+08	1.3E+08	28	MGMSRv2__0255	Uncharacterized protein
V6EYW3_9PROT	18	62.3	24.40	0.15	1.3E+08	1.2E+08	1.2E+08	47	rplA	50S ribosomal protein L1
V6EZ26_9PROT	10	42.3	14.09	0.15	1.3E+08	1.2E+08	1.1E+08	32	rpsK	30S ribosomal protein S11
V6EVN7_9PROT	18	69.8	21.91	0.15	1.2E+08	1.1E+08	1.3E+08	49	rplD	50S ribosomal protein L4
V6F880_9PROT	10	74.1	12.44	0.15	1.3E+08	1.2E+08	1.1E+08	27	MGMSRv2__3518	cAMP-binding protein-catabolite gene activator and regulatory subunit of cAMP-dependent protein kinase
V6F7P4_9PROT	11	33.1	31.36	0.14	1.2E+08	1.2E+08	1.1E+08	48	MGMSRv2__4162	Putative Alpha/beta hydrolase fold
V6F329_9PROT	32	47.1	46.31	0.13	1.2E+08	1.1E+08	9.9E+07	122	cfa	Cyclopropane fatty acyl phospholipid synthase (Unsaturated-phospholipid methyltransferase)
V6F226_9PROT	6	62.2	9.08	0.12	1.0E+08	8.6E+07	9.2E+07	15	hupB	HU, DNA-binding transcriptional regulator, beta subunit

Protein IDs	Unique peptides	Unique sequence coverage [%]	Mol. weight [kDa]	Relative abundance (%)	iBAQ 1	iBAQ 2	iBAQ 3	MS/MS Count	Gene name	Protein description
V6F935_9PROT	14	32	33.69	0.11	8.8E+07	8.7E+07	8.7E+07	40	ompA	Outer membrane protein A (3aII*Gd)
V6F1E6_9PROT	6	39.2	8.29	0.11	8.2E+07	8.4E+07	9.5E+07	11	rpmE	50S ribosomal protein L31
V6F5M3_9PROT	9	40.6	15.09	0.11	8.1E+07	8.1E+07	9.5E+07	20	mamW	Magnetosome protein MamW
V6F780_9PROT	8	70	12.50	0.10	9.1E+07	8.0E+07	8.1E+07	23	MGMSRv2__4155	Cytochrome c2 iso-2
V6F7E5_9PROT	10	48.6	15.25	0.10	1.3E+08	4.3E+07	7.0E+07	28	ndk	Nucleoside diphosphate kinase
V6EYQ5_9PROT	11	39.1	17.29	0.10	7.9E+07	8.0E+07	7.7E+07	32	MGMSRv2__1175	Putative transcriptional regulatory protein, Ros/MucR family
V6EVY0_9PROT	12	64.7	11.71	0.10	6.2E+07	8.3E+07	8.9E+07	30	rpsJ	30S ribosomal protein S10
V6F5E0_9PROT	28	67.5	35.29	0.09	7.9E+07	7.7E+07	6.9E+07	79	MGMSRv2__2306	Uncharacterized protein
V6EZU0_9PROT	22	72.2	32.98	0.09	7.8E+07	7.0E+07	7.2E+07	57	MGMSRv2__1462	Protein HflC
V6EYJ0_9PROT	18	38	40.46	0.09	7.3E+07	7.3E+07	7.2E+07	64	MGMSRv2__1090	Putative TRAP-type mannitol/chloroaromatic compound transport system
V6EY16_9PROT	15	71.1	15.66	0.08	7.2E+07	6.2E+07	6.2E+07	40	MGMSRv2__0817	LWamide neuropeptides
V6F493_9PROT	11	61.9	13.45	0.08	7.1E+07	6.2E+07	6.1E+07	26	yhbH	Ribosome-associated, sigma 54 modulation protein
V6F6H9_9PROT	5	45.3	10.73	0.08	6.7E+07	5.8E+07	6.0E+07	13	ihfB	Integration host factor subunit beta
V6EYI6_9PROT	3	42.3	8.28	0.08	5.3E+07	5.7E+07	7.2E+07	12	tatA	Sec-independent protein translocase protein TatA
V6F3B8_9PROT	6	38	21.55	0.07	6.9E+07	5.8E+07	5.5E+07	22	MGMSRv2__2667	ABC-type polar amino acid transport system, ATPase component
V6EYG6_9PROT	15	57.1	24.56	0.07	5.6E+07	5.7E+07	6.1E+07	42	rplC	50S ribosomal protein L3
V6EZA2_9PROT	9	39.5	18.53	0.07	7.0E+07	4.9E+07	5.6E+07	25	nuoI	NADH-quinone oxidoreductase subunit I
V6F5X9_9PROT	6	44.2	14.40	0.07	5.9E+07	5.4E+07	5.8E+07	20	cheY	Chemotaxis regulator transmitting signal to flagellar motor component
V6F6Z3_9PROT	14	80.7	16.36	0.07	5.8E+07	5.0E+07	5.0E+07	31	mms16	Magnetic particle membrane specific GTPase P16

Protein IDs	Unique peptides	Unique sequence coverage [%]	Mol. weight [kDa]	Relative abundance (%)	iBAQ 1	iBAQ 2	iBAQ 3	MS/MS Count	Gene name	Protein description
V6F8Q1_9PROT	18	64.9	36.29	0.06	4.8E+07	5.4E+07	5.6E+07	54	gyaR	Glyoxylate reductase
V6F6F6_9PROT	19	55.6	31.84	0.06	5.2E+07	4.8E+07	5.0E+07	44	atpG	ATP synthase gamma chain
V6F627_9PROT	11	40.1	29.90	0.06	4.7E+07	4.5E+07	5.4E+07	31	ysgA	Putative carboxymethylenebutenolidase (Dienelactone hydrolase DLH)
V6EVI6_9PROT	8	44.1	10.99	0.06	5.2E+07	4.8E+07	4.4E+07	20	rplW	50S ribosomal protein L23
V6F2K8_9PROT	8	41.5	21.67	0.06	5.2E+07	5.1E+07	3.9E+07	26	MGMSRv2__1306	Uncharacterized protein
V6F476_9PROT	11	42.8	19.67	0.06	4.9E+07	4.6E+07	4.7E+07	36	infC	Translation initiation factor IF-3
V6EZW4_9PROT	7	48.6	16.40	0.05	4.8E+07	4.1E+07	4.0E+07	20	rpsF	30S ribosomal protein S6
V6F0Y5_9PROT	9	34.2	25.83	0.05	4.7E+07	4.0E+07	4.0E+07	25	phbB	Acetoacetyl-coA reductase
V6EZ95_9PROT	9	38.8	16.71	0.05	4.1E+07	3.9E+07	3.6E+07	24	MGMSRv2__1365	Uncharacterized protein
V6EZD1_9PROT	9	38.8	26.18	0.05	3.9E+07	3.9E+07	3.7E+07	32	scoA	Succinyl-CoA:3-ketoacid-coenzyme A transferase subunit A (Succinyl CoA:3-oxoacid CoA-transferase) (OXCT A)
V6F5H5_9PROT	3	29.7	10.63	0.05	4.5E+07	3.4E+07	3.4E+07	7	MGMSRv2__2346	Uncharacterized protein
V6F2A7_9PROT	5	61.9	9.33	0.05	4.2E+07	4.3E+07	2.8E+07	13	mamR	Magnetosome protein MamR, containing Excisionase/Xis, DNA-binding domain
V6F1W7_9PROT	3	20.7	12.30	0.04	3.4E+07	3.7E+07	3.8E+07	12	MGMSRv2__2310	Uncharacterized protein
V6EVW5_9PROT	16	40.7	43.08	0.04	3.6E+07	3.1E+07	3.3E+07	37	tufB	Elongation factor Tu
V6F289_9PROT	6	37	16.57	0.04	3.8E+07	3.0E+07	3.3E+07	16	iscR	DNA-binding transcriptional repressor
V6F7J2_9PROT	4	26.4	20.04	0.04	3.1E+07	3.3E+07	3.5E+07	11	ppa	Inorganic pyrophosphatase
V6F6N0_9PROT	31	48.3	54.64	0.04	3.4E+07	3.1E+07	2.9E+07	79	atpA	ATP synthase subunit alpha
V6EVH0_9PROT	5	24.6	15.00	0.04	3.3E+07	3.1E+07	2.9E+07	17	rplK	50S ribosomal protein L11
V6EZ52_9PROT	12	50.6	18.70	0.04	3.3E+07	2.7E+07	3.1E+07	37	pal	Peptidoglycan-associated outer membrane lipoprotein
V6F6I3_9PROT	9	46.4	17.08	0.04	3.5E+07	3.0E+07	2.6E+07	20	MGMSRv2__2888	Putative HTH-type transcriptional regulator iscR
V6F5R7_9PROT	11	63.9	17.17	0.04	3.0E+07	2.9E+07	3.1E+07	29	rplS	50S ribosomal protein L19
V6F1K7_9PROT	22	39.9	49.14	0.04	3.0E+07	2.9E+07	2.9E+07	51	lpD	Dihydrolipoyl dehydrogenase

Protein IDs	Unique peptides	Unique sequence coverage [%]	Mol. weight [kDa]	Relative abundance (%)	iBAQ 1	iBAQ 2	iBAQ 3	MS/MS Count	Gene name	Protein description
V6EXV3_9PROT	13	43.5	28.37	0.04	2.9E+07	3.3E+07	2.6E+07	31	MGMSRv2__0880	Putative thiol-disulfide oxidoreductase
V6F0D5_9PROT	12	45.1	23.84	0.04	2.9E+07	2.5E+07	3.1E+07	25	MGMSRv2__0541	Uncharacterized protein
V6F987_9PROT	6	37.2	18.42	0.03	2.7E+07	2.7E+07	2.9E+07	16	MGMSRv2__4251	Putative Ferritin and Dps family,DNA protection during starvation protein
V6EWF3_9PROT	7	38.2	29.82	0.03	3.1E+07	2.6E+07	2.5E+07	18	MGMSRv2__0304	Putative universal stress protein UspA
V6F6T8_9PROT	11	56.5	22.34	0.03	2.5E+07	2.8E+07	2.7E+07	23	MGMSRv2__3949	Putative major cold shock protein
V6F303_9PROT	32	49.9	57.69	0.03	2.8E+07	2.6E+07	2.3E+07	82	groL	60 kDa chaperonin
V6F8X1_9PROT	12	25.2	45.73	0.03	4.1E+07	1.8E+07	1.8E+07	29	icd	Isocitrate dehydrogenase [NADP]
V6EZ14_9PROT	7	73.9	12.12	0.03	2.6E+07	2.6E+07	2.3E+07	19	MGMSRv2__1290	Putative phasin
V6F410_9PROT	12	25.4	50.96	0.03	2.3E+07	2.6E+07	2.6E+07	40	MGMSRv2__1811	Putative amino acid dehydrogenase containing NAD(P)-binding domain and ferridoxin-like domain
V6F512_9PROT	17	46.6	36.53	0.03	2.6E+07	2.4E+07	2.3E+07	36	dctP	TRAP-Type C4-dicarboxylate transport system, binding periplasmic protein (DctP subunit)
V6F703_9PROT	8	25	31.69	0.03	2.4E+07	1.9E+07	2.2E+07	22	mmgB	3-hydroxybutyryl-CoA dehydrogenase (Beta-hydroxybutyryl-CoA dehydrogenase) (BHBD)
V6F1M2_9PROT	16	40.9	45.38	0.03	2.2E+07	2.1E+07	2.0E+07	36	glyA	Serine hydroxymethyltransferase
V6F630_9PROT	11	23.8	41.58	0.02	1.9E+07	2.0E+07	2.0E+07	32	sucB	Dihydrolipoyllysine-residue succinyltransferase component of 2-oxoglutarate dehydrogenase complex
V6F885_9PROT	12	30.7	38.44	0.02	2.0E+07	1.9E+07	1.9E+07	28	gapA	Glyceraldehyde-3-phosphate dehydrogenase

Protein IDs	Unique peptides	Unique sequence coverage [%]	Mol. weight [kDa]	Relative abundance (%)	iBAQ 1	iBAQ 2	iBAQ 3	MS/MS Count	Gene name	Protein description
V6F3J9_9PROT	11	27.2	40.38	0.02	1.9E+07	1.9E+07	1.8E+07	26	phbA	Acetyl-CoA acetyltransferase containing thiolase domain (Acetoacetyl-CoA thiolase)
V6EZY1_9PROT	11	25.5	44.01	0.02	2.1E+07	1.8E+07	1.6E+07	22	MGMSRv2__1575	Uncharacterized protein
V6EYF4_9PROT	5	38.7	12.79	0.02	2.1E+07	1.6E+07	1.7E+07	9	rplL	50S ribosomal protein L7/L12
V6F8R4_9PROT	13	63.3	25.36	0.02	2.0E+07	1.9E+07	1.4E+07	28	MGMSRv2__3968	Uncharacterized protein
V6EYH3_9PROT	14	18.1	47.30	0.02	1.5E+07	2.4E+07	1.4E+07	36	fixC	FixC protein flavoprotein-ubiquinone oxidoreductase
V6F5L5_9PROT	15	42.9	42.32	0.02	1.7E+07	1.8E+07	1.7E+07	45	pgk	Phosphoglycerate kinase
V6F384_9PROT	5	36.8	10.27	0.02	1.9E+07	1.5E+07	1.8E+07	12	groS	10 kDa chaperonin
V6F3X0_9PROT	5	52	10.75	0.02	1.7E+07	1.8E+07	1.6E+07	10	rplU	50S ribosomal protein L21
V6F5K0_9PROT	19	42.6	49.02	0.02	1.7E+07	1.6E+07	1.6E+07	51	ffh	Signal recognition particle protein
V6F2J4_9PROT	14	31.9	39.61	0.02	1.8E+07	1.6E+07	1.5E+07	38	MGMSRv2__1291	Putative multidrug efflux system, subunit A
V6EXW5_9PROT	7	50.3	17.23	0.02	1.7E+07	1.6E+07	1.6E+07	17	MGMSRv2__0890	Putative peroxiredoxin bcp
V6F2M8_9PROT	4	12.7	29.29	0.02	1.2E+07	1.7E+07	1.9E+07	10	UspA	Universal stress protein UspA
V6F1H5_9PROT	12	42.9	28.79	0.02	1.3E+07	1.8E+07	1.6E+07	19	purC	Phosphoribosylaminoimidazole-succinocarboxamide synthase
V6F8I4_9PROT	10	36.5	33.41	0.02	1.5E+07	1.5E+07	1.6E+07	19	mdh	Malate dehydrogenase
V6F3N9_9PROT	12	42	34.82	0.02	1.5E+07	1.5E+07	1.5E+07	30	MGMSRv2__2797	Putative alkanesulfonate transporter subunit periplasmic-binding component of ABC superfamily
V6EYD1_9PROT	14	33.9	38.23	0.02	1.6E+07	1.4E+07	1.5E+07	33	MGMSRv2__0989	Polyphosphate kinase 2
V6F0C8_9PROT	9	25.8	33.88	0.02	1.4E+07	1.3E+07	1.4E+07	29	accD	Acetyl-coenzyme A carboxylase carboxyl transferase subunit beta
V6F5E6_9PROT	14	31.4	49.84	0.02	1.4E+07	1.3E+07	1.4E+07	30	MGMSRv2__3520	Uncharacterized protein
V6F4V1_9PROT	11	52.3	19.97	0.02	1.3E+07	1.5E+07	1.2E+07	20	MGMSRv2__3269	Putative bacteriohemerythrin
V6EX21_9PROT	12	59.9	23.86	0.02	1.3E+07	1.3E+07	1.4E+07	26	MGMSRv2__0529	Uncharacterized protein
V6EYZ7_9PROT	5	30.8	11.29	0.02	1.4E+07	1.2E+07	1.3E+07	10	ihfA	Integration host factor subunit alpha

Protein IDs	Unique peptides	Unique sequence coverage [%]	Mol. weight [kDa]	Relative abundance (%)	iBAQ 1	iBAQ 2	iBAQ 3	MS/MS Count	Gene name	Protein description
V6EYM7_9PROT	19	41	49.66	0.02	1.3E+07	1.4E+07	1.2E+07	38	MGMSRv2__1074	Putative outer membrane efflux protein
V6F5P4_9PROT	14	33.9	42.31	0.02	1.4E+07	1.2E+07	1.2E+07	30	sucC	Succinate--CoA ligase [ADP-forming] subunit beta
V6F770_9PROT	10	27.7	37.97	0.02	1.2E+07	1.3E+07	1.3E+07	22	cbbA	Fructose-bisphosphate aldolase
V6F8S3_9PROT	3	11.5	27.52	0.02	1.3E+07	1.3E+07	1.2E+07	8	MGMSRv2__3846	Uncharacterized protein
V6EZA4_9PROT	4	11.5	52.20	0.02	1.4E+07	1.3E+07	9.1E+06	15	glnA	Glutamine synthetase
V6F637_9PROT	5	24	21.10	0.01	1.3E+07	1.0E+07	1.1E+07	14	ctc	50S ribosomal protein L25
V6F7D1_9PROT	15	23.2	74.53	0.01	1.2E+07	1.0E+07	1.1E+07	34	rpoD	RNA polymerase sigma factor RpoD
V6EY05_9PROT	15	37.2	35.98	0.01	1.1E+07	1.0E+07	1.1E+07	34	MGMSRv2__0925	Putative Secretion protein HlyD
V6EZP5_9PROT	12	24.1	51.92	0.01	1.3E+07	9.4E+06	1.0E+07	25	degP	Serine endoprotease (Protease Do), membrane-associated
V6F2B4_9PROT	12	42.5	32.09	0.01	1.3E+07	9.5E+06	1.0E+07	27	tsf	Elongation factor Ts
V6EXV2_9PROT	11	32.7	34.28	0.01	9.3E+06	1.2E+07	9.6E+06	24	cbbP	Phosphoribulokinase
V6F4T6_9PROT	5	55.9	10.68	0.01	8.7E+06	1.1E+07	1.1E+07	11	MGMSRv2__3254	Uncharacterized protein
V6F8T9_9PROT	8	23.6	50.48	0.01	1.6E+07	9.9E+06	4.8E+06	15	atpD	ATP synthase subunit beta
V6F5W3_9PROT	7	28.5	29.68	0.01	9.9E+06	1.0E+07	9.7E+06	11	sucD	Succinate--CoA ligase [ADP-forming] subunit alpha
V6F6W1_9PROT	9	28.8	25.66	0.01	9.9E+06	9.5E+06	9.5E+06	23	ftsE	Transporter subunit: ATP-binding component of ABC superfamily
V6F267_9PROT	6	31.8	18.94	0.01	9.2E+06	1.0E+07	9.7E+06	11	atpF	ATP synthase subunit b
V6F8Z9_9PROT	9	29.5	44.57	0.01	9.8E+06	9.4E+06	9.2E+06	19	hom	Homoserine dehydrogenase
V6EXY5_9PROT	17	27.7	49.94	0.01	1.0E+07	8.2E+06	9.3E+06	39	tolB	Protein TolB
V6F691_9PROT	9	23.3	49.01	0.01	9.6E+06	8.7E+06	9.5E+06	18	MGMSRv2__3749	Uncharacterized protein
V6EYK1_9PROT	6	13.1	48.06	0.01	9.7E+06	9.4E+06	8.4E+06	15	proS	Proline--tRNA ligase
V6EZG2_9PROT	8	47.6	18.81	0.01	9.4E+06	9.8E+06	8.2E+06	13	MGMSRv2__0211	Uncharacterized protein
V6F1K3_9PROT	8	27.9	36.33	0.01	8.3E+06	8.8E+06	9.0E+06	17	pdhA	Pyruvate dehydrogenase E1 component subunit alpha
V6F0I5_9PROT	9	41.8	22.16	0.01	9.4E+06	7.9E+06	8.5E+06	22	MGMSRv2__0798	Putative Glycerol metabolism activator

Protein IDs	Unique peptides	Unique sequence coverage [%]	Mol. weight [kDa]	Relative abundance (%)	iBAQ 1	iBAQ 2	iBAQ 3	MS/MS Count	Gene name	Protein description
V6F3Z2_9PROT	7	21.1	27.06	0.01	8.9E+06	8.3E+06	8.0E+06	14	MGMSRv2__3025	Glycosyl transferase, group 2 family protein
V6F1R4_9PROT	12	46.3	25.66	0.01	7.7E+06	8.7E+06	8.5E+06	15	MGMSRv2__2255	Putative Methyl-accepting chemotaxis sensory transducer
V6EZB2_9PROT	27	30.6	88.41	0.01	9.3E+06	7.5E+06	7.9E+06	49	lon	Lon protease
V6F5T3_9PROT	6	25.1	23.76	0.01	8.8E+06	8.2E+06	7.5E+06	12	agmR	Glycerol metabolism activator
V6F3S0_9PROT	5	21.2	19.45	0.01	8.9E+06	7.7E+06	7.7E+06	13	MGMSRv2__2827	Ubiquinol-cytochrome c reductase iron-sulfur subunit
V6F3W5_9PROT	7	22.8	22.45	0.01	9.0E+06	7.5E+06	7.8E+06	16	nadD	Probable nicotinate-nucleotide adenylyltransferase
V6F985_9PROT	9	36	29.28	0.01	8.4E+06	8.1E+06	7.7E+06	20	fabI	Enoyl-[acyl-carrier-protein] reductase [NADH]
V6EXU3_9PROT	6	22.6	34.61	0.01	8.6E+06	7.7E+06	7.8E+06	12	MGMSRv2__0804	Putative Translation initiation factor IF-2
V6F7K6_9PROT	4	31.1	15.97	0.01	8.4E+06	6.1E+06	7.3E+06	11	MGMSRv2__4239	Putative HTH-type transcriptional regulator CymR
V6EYS0_9PROT	6	22.6	23.28	0.01	7.4E+06	6.9E+06	7.3E+06	14	pcm	Protein-L-isoaspartate(D-aspartate) O-methyltransferase
V6F1M3_9PROT	4	49.6	13.25	0.01	8.5E+06	5.9E+06	7.2E+06	9	cheY	Chemotaxis protein cheY
V6F5Y9_9PROT	8	23.6	40.06	0.01	6.8E+06	7.6E+06	6.9E+06	12	leuB	3-isopropylmalate dehydrogenase
V6F1V1_9PROT	7	27.1	27.28	0.01	7.8E+06	6.3E+06	6.6E+06	13	MGMSRv2__2142	Putative amino acid transport protein, ATP-binding protein
V6F4R4_9PROT	10	24.5	55.93	0.01	6.4E+06	7.0E+06	7.2E+06	19	yqjD	Putative acyl-CoA carboxylase
V6F8S9_9PROT	14	27	51.75	0.01	7.1E+06	6.3E+06	7.1E+06	29	pepA	Probable cytosol aminopeptidase
V6F8K2_9PROT	17	30	61.47	0.01	7.1E+06	6.7E+06	6.8E+06	35	rpsA	30S ribosomal protein S1
V6F6T3_9PROT	6	23	26.43	0.01	7.6E+06	6.1E+06	6.5E+06	11	etfB	Electron transfer flavoprotein subunit beta
V6F110_9PROT	8	23.8	43.10	0.01	7.4E+06	6.5E+06	6.2E+06	17	pdhC	Acetyltransferase component of pyruvate dehydrogenase complex
V6F459_9PROT	5	9.6	58.72	0.01	7.7E+06	6.0E+06	6.3E+06	15	MGMSRv2__3029	Putative electron transfer flavoprotein dehydrogenases

Protein IDs	Unique peptides	Unique sequence coverage [%]	Mol. weight [kDa]	Relative abundance (%)	iBAQ 1	iBAQ 2	iBAQ 3	MS/MS Count	Gene name	Protein description
V6F7K2_9PROT	5	38.9	11.52	0.01	7.2E+06	6.4E+06	6.4E+06	11	MGMSRv2__4117	Anti-sigma factor antagonist
V6F883_9PROT	17	37.6	45.58	0.01	7.0E+06	6.6E+06	6.2E+06	27	argG	Argininosuccinate synthase
V6F106_9PROT	7	31.6	30.02	0.01	7.7E+06	5.7E+06	6.1E+06	14	MGMSRv2__1857	Putative ATPase MipZ
V6EZP7_9PROT	8	25	30.01	0.01	7.4E+06	6.0E+06	6.0E+06	14	MGMSRv2__1417	Uncharacterized protein
V6F0D7_9PROT	6	21.1	22.68	0.01	6.8E+06	6.1E+06	6.4E+06	7	MGMSRv2__1709	Uncharacterized protein
V6F6A5_9PROT	7	25.3	37.15	0.01	6.2E+06	6.1E+06	6.2E+06	13	rfaD	ADP-L-glycero-D-manno-heptose-6-epimerase
V6EWE4_9PROT	5	35.8	18.21	0.01	8.6E+06	3.1E+06	6.7E+06	7	MGMSRv2__0187	UPF0234 protein MGMSRv2__0187
V6EVH5_9PROT	34	22	165.76	0.01	6.3E+06	5.7E+06	6.0E+06	74	rpoC	DNA-directed RNA polymerase subunit beta
V6EVK4_9PROT	3	3.6	88.64	0.01	6.3E+06	6.0E+06	5.5E+06	7	gyrB	DNA gyrase subunit B
V6F2H8_9PROT	5	16.1	37.58	0.01	6.6E+06	5.1E+06	6.0E+06	15	mamK	Magnetosome cytoskeleton protein MamK
V6F8I3_9PROT	7	16.9	48.69	0.01	4.9E+06	6.7E+06	6.0E+06	12	lpd	Dihydrolipoyl dehydrogenase
V6EY88_9PROT	2	12.8	19.15	0.01	6.0E+06	5.8E+06	5.7E+06	6	ppiB	Peptidyl-prolyl cis-trans isomerase
V6EW92_9PROT	5	13.9	38.82	0.01	6.0E+06	5.5E+06	5.9E+06	11	MGMSRv2__0132	Putative UDP-glucose 4-epimerase
V6F8M7_9PROT	7	14.1	46.96	0.01	5.1E+06	5.5E+06	5.2E+06	13	rkpK	UDP-glucose 6-dehydrogenase (UDP-Glc dehydrogenase) (UDP-GlcDH) (UDPGDH)
V6F311_9PROT	4	19.2	11.31	0.01	5.9E+06	4.8E+06	5.0E+06	9	MGMSRv2__2705	Uncharacterized protein
V6F8F0_9PROT	7	21.7	27.51	0.01	6.3E+06	5.3E+06	4.2E+06	15	MGMSRv2__3688	Uncharacterized protein
V6F8Y3_9PROT	4	18.7	34.21	0.01	5.4E+06	5.5E+06	4.7E+06	10	MGMSRv2__4133	Putative NADH-ubiquinone oxidoreductase 40 kDa subunit
V6F3Y4_9PROT	9	42.8	32.51	0.01	5.3E+06	5.4E+06	4.9E+06	16	cysK	Pyridoxal-phosphate (PLP) dependent enzymes family subunit of cysteine synthase A (O-acetylserine sulfhydrolase A)
V6F016_9PROT	7	15	37.20	0.01	3.4E+06	5.8E+06	5.8E+06	17	ilvC	Ketol-acid reductoisomerase (NADP(+))

Protein IDs	Unique peptides	Unique sequence coverage [%]	Mol. weight [kDa]	Relative abundance (%)	iBAQ 1	iBAQ 2	iBAQ 3	MS/MS Count	Gene name	Protein description
V6F5E7_9PROT	5	29.6	26.80	0.01	4.6E+06	5.0E+06	5.3E+06	13	MGMSRv2__3382	Putative Chitooligosaccharide deacetylase
V6F0C3_9PROT	9	22.8	52.16	0.01	5.1E+06	4.7E+06	4.8E+06	21	der	GTPase Der
V6F1I6_9PROT	6	15.6	42.63	0.01	4.7E+06	5.1E+06	4.6E+06	17	MGMSRv2__1128	Putative Serine-type D-Ala-D-Ala carboxypeptidase
V6F5B6_9PROT	5	26.8	20.52	0.01	4.7E+06	4.8E+06	4.8E+06	7	MGMSRv2__2276	Uncharacterized protein
V6F7F4_9PROT	2	10.1	16.20	0.01	4.3E+06	4.8E+06	5.0E+06	7	omp	17 kDa surface antigen
V6F859_9PROT	10	26.1	46.81	0.01	4.6E+06	4.3E+06	4.7E+06	22	rho	Transcription termination factor Rho
V6F6J4_9PROT	11	15.7	68.26	0.01	4.4E+06	4.6E+06	4.7E+06	20	dnaK	Chaperone protein DnaK
V6F5Q1_9PROT	9	25.4	37.75	0.01	4.8E+06	4.8E+06	4.0E+06	20	MGMSRv2__3615	Uncharacterized protein
V6F3J4_9PROT	2	26	11.21	0.01	4.6E+06	5.0E+06	3.8E+06	6	trxA	Thioredoxin
V6EVM7_9PROT	26	19.4	155.65	0.01	4.6E+06	4.4E+06	4.4E+06	61	rpoB	DNA-directed RNA polymerase subunit beta
V6F4L5_9PROT	15	19.2	92.58	0.01	4.7E+06	4.8E+06	3.9E+06	29	acnB	Aconitate hydratase B
V6EZQ0_9PROT	11	14.4	74.20	0.01	4.6E+06	5.0E+06	3.7E+06	25	MGMSRv2__0483	Putative serine protein kinase
V6EWF5_9PROT	9	23.5	29.69	0.01	4.1E+06	4.7E+06	4.4E+06	15	MGMSRv2__0202	Putative high-affinity branched-chain amino acid ABC transporter, ATP-binding protein
V6F0J5_9PROT	7	7.7	67.17	0.01	3.9E+06	4.2E+06	4.9E+06	14	pckG	Phosphoenolpyruvate carboxykinase [GTP]
V6F8B9_9PROT	17	19.8	110.04	0.01	3.8E+06	4.8E+06	4.4E+06	30	sucA	2-oxoglutarate decarboxylase, component of the 2-oxoglutarate dehydrogenase complex, thiamin-binding
V6EZM7_9PROT	7	33.5	20.46	0.01	4.2E+06	4.9E+06	3.5E+06	9	rplI	50S ribosomal protein L9
V6F626_9PROT	2	14.4	13.54	0.01	4.8E+06	4.5E+06	3.2E+06	6	clpS	ATP-dependent Clp protease adapter protein ClpS
V6F2T2_9PROT	7	18.4	38.41	0.01	4.1E+06	3.9E+06	4.3E+06	14	murG	UDP-N-acetylglucosamine--N-acetylmuramyl-(pentapeptide) pyrophosphoryl-undecaprenol N-acetylglucosamine transferase

Protein IDs	Unique peptides	Unique sequence coverage [%]	Mol. weight [kDa]	Relative abundance (%)	iBAQ 1	iBAQ 2	iBAQ 3	MS/MS Count	Gene name	Protein description
V6F673_9PROT	11	12.1	94.56	0.01	4.3E+06	3.7E+06	4.2E+06	21	infB	Translation initiation factor IF-2
V6F6V4_9PROT	6	13	54.76	0.01	4.3E+06	4.1E+06	3.7E+06	10	aldB	Aldehyde dehydrogenase B
V6F0A4_9PROT	4	14.7	33.60	0.00	4.8E+06	3.7E+06	3.6E+06	9	yedY	Protein-methionine-sulfoxide reductase catalytic subunit MsrP
V6EWW8_9PROT	3	18.1	23.81	0.00	3.4E+06	5.1E+06	3.5E+06	6	MGMSRv2__0464	Uncharacterized protein
V6F678_9PROT	12	18.7	77.28	0.00	4.2E+06	3.9E+06	3.9E+06	26	pnp	Polyribonucleotide nucleotidyltransferase
V6EYW0_9PROT	7	22	43.82	0.00	3.7E+06	4.4E+06	3.9E+06	15	MGMSRv2__0198	Putative branched-chain amino acid ABC transporter
V6F8Q3_9PROT	5	25.1	31.40	0.00	4.5E+06	3.6E+06	3.5E+06	13	etfA	Electron transfer flavoprotein (Alpha subunit)
V6F417_9PROT	7	26.6	32.85	0.00	3.3E+06	3.5E+06	4.2E+06	13	cheR	Chemotaxis protein methyltransferase
V6EYK9_9PROT	4	10.7	46.51	0.00	3.2E+06	3.7E+06	3.8E+06	12	MGMSRv2__1120	Putative O-acetylhomoserine/O-acetylserine sulfhydrylase
V6EZR8_9PROT	4	20.4	25.44	0.00	3.2E+06	3.7E+06	3.7E+06	9	fabG	3-oxoacyl-[acyl-carrier-protein] reductase
V6F907_9PROT	5	13.8	52.63	0.00	4.1E+06	3.2E+06	3.4E+06	13	MGMSRv2__4046	Putative ATPase
V6F1W4_9PROT	3	24.2	15.91	0.00	4.0E+06	3.3E+06	3.3E+06	6	MGMSRv2__2157	Uncharacterized protein
V6EX06_9PROT	6	15	36.68	0.00	3.5E+06	3.6E+06	3.5E+06	14	mreB	Cell shape determining protein MreB/Mrl
V6EWZ2_9PROT	5	14.5	43.14	0.00	3.4E+06	3.2E+06	3.4E+06	8	MGMSRv2__0417	Putative ABC transport protein, substrate-binding component
V6F763_9PROT	2	8.9	31.55	0.00	3.6E+06	3.1E+06	3.1E+06	7	fixP	Cbb3-type cytochrome c oxidase subunit
V6F036_9PROT	7	31.5	28.93	0.00	4.3E+06	2.8E+06	2.7E+06	12	MGMSRv2__1599	Putative phosphate-binding protein pstS
V6F0Y4_9PROT	7	15.3	38.71	0.00	3.6E+06	3.0E+06	3.0E+06	8	livK	Leucine transporter subunit periplasmic-binding component of ABC superfamily
V6F067_9PROT	4	6.5	59.09	0.00	3.5E+06	3.0E+06	2.9E+06	10	MGMSRv2__1557	Putative acyl-CoA synthetase

Protein IDs	Unique peptides	Unique sequence coverage [%]	Mol. weight [kDa]	Relative abundance (%)	iBAQ 1	iBAQ 2	iBAQ 3	MS/MS Count	Gene name	Protein description
V6F7D9_9PROT	7	23.4	38.41	0.00	3.1E+06	2.8E+06	3.5E+06	9	mmeA	Magnetosome membrane associated protein MmeA
V6EZW3_9PROT	8	21.6	39.28	0.00	3.2E+06	3.0E+06	3.0E+06	17	pntA	Pyridine nucleotide transhydrogenase, alpha subunit
V6F477_9PROT	8	26.8	38.09	0.00	2.9E+06	2.9E+06	3.2E+06	13	recA	Protein RecA
V6F5Q2_9PROT	6	16.1	48.33	0.00	2.7E+06	2.9E+06	3.0E+06	12	hslU	ATP-dependent protease ATPase subunit HslU
V6F242_9PROT	6	16	48.60	0.00	2.7E+06	2.7E+06	3.0E+06	15	gltA	Citrate synthase
V6EZE1_9PROT	3	9.9	43.04	0.00	2.4E+06	2.8E+06	3.2E+06	8	MGMSRv2__1410	Uncharacterized protein
V6F189_9PROT	5	19.1	25.24	0.00	3.6E+06	2.2E+06	2.3E+06	9	MGMSRv2__0846	Transcriptional regulatory protein CusR
V6F4Y6_9PROT	4	8.7	48.74	0.00	2.6E+06	2.7E+06	2.8E+06	10	MGMSRv2__3222	Putative intracellular PHB depolymerase
V6F5W4_9PROT	4	24.6	23.20	0.00	2.6E+06	2.6E+06	2.4E+06	9	yrbC	Toluene transporter subunit: membrane component of ABC superfamily
V6F6Y9_9PROT	9	16.6	71.49	0.00	2.6E+06	2.5E+06	2.2E+06	15	acs	Acetyl-coenzyme A synthetase
V6F8X0_9PROT	7	22.9	40.17	0.00	2.5E+06	2.3E+06	2.5E+06	12	MGMSRv2__4093	Uncharacterized protein
V6F563_9PROT	7	16.7	64.52	0.00	2.9E+06	2.1E+06	2.2E+06	12	sdhA	Succinate dehydrogenase flavoprotein subunit
V6F1V9_9PROT	6	21.1	38.94	0.00	2.3E+06	2.6E+06	2.2E+06	11	fbpC	Sulfate/thiosulfate import ATP-binding protein CysA
V6F3C0_9PROT	4	8.6	50.60	0.00	2.4E+06	2.2E+06	2.4E+06	8	ahcY	Adenosylhomocysteinase
V6F793_9PROT	2	13.2	15.57	0.00	2.3E+06	2.3E+06	2.3E+06	6	MGMSRv2__4099	Cytochrome c
V6EXL6_9PROT	8	9.5	102.37	0.00	1.9E+06	1.8E+06	2.9E+06	13	rne	Ribonuclease E
V6EYH7_9PROT	5	16.8	31.19	0.00	2.1E+06	2.0E+06	2.3E+06	7	MGMSRv2__1039	Periplasmic protein TonB, links inner and outer membranes
V6F708_9PROT	5	9.2	56.36	0.00	1.8E+06	2.4E+06	2.1E+06	11	ubiB	Probable protein kinase UbiB
V6F4Q8_9PROT	4	12.6	24.18	0.00	2.8E+06	1.4E+06	1.9E+06	7	MGMSRv2__2051	Putative response regulator receiver (CheY-like protein)
V6EY51_9PROT	3	15.5	17.38	0.00	2.2E+06	1.9E+06	1.9E+06	6	MGMSRv2__0970	Uncharacterized protein
V6F2M1_9PROT	9	15.1	73.42	0.00	2.2E+06	1.9E+06	1.9E+06	15	nuoG	NADH-quinone oxidoreductase

Protein IDs	Unique peptides	Unique sequence coverage [%]	Mol. weight [kDa]	Relative abundance (%)	iBAQ 1	iBAQ 2	iBAQ 3	MS/MS Count	Gene name	Protein description
V6F7A2_9PROT	7	16.1	48.93	0.00	1.7E+06	1.8E+06	2.3E+06	15	degP	Putative serine protease (DegP periplasmic, membrane-associated serine endoprotease, protease Do), containing two PDZ domains
V6F2H5_9PROT	3	8.8	38.48	0.00	1.9E+06	1.9E+06	2.1E+06	8	pfkA	ATP-dependent 6-phosphofructokinase
V6EYD7_9PROT	9	12.6	79.10	0.00	2.1E+06	1.8E+06	1.8E+06	13	fusA	Elongation factor G
V6EZK7_9PROT	4	9.2	64.62	0.00	1.8E+06	2.1E+06	1.6E+06	9	ilvI	Acetolactate synthase
V6F7R4_9PROT	5	6	68.99	0.00	1.8E+06	2.0E+06	1.7E+06	8	htpG	Chaperone protein HtpG
V6F6Z7_9PROT	8	15.6	51.55	0.00	1.9E+06	1.7E+06	1.9E+06	12	guaB	Inosine-5-monophosphate dehydrogenase
V6EYE0_9PROT	4	10.1	45.27	0.00	1.8E+06	2.0E+06	1.6E+06	7	eno	Enolase
V6EWQ8_9PROT	4	9.2	48.96	0.00	1.8E+06	1.7E+06	1.8E+06	9	gltX	Glutamate--tRNA ligase
V6F3J1_9PROT	3	11.8	42.74	0.00	1.8E+06	1.6E+06	1.8E+06	8	dfp	Fused 4-phosphopantothenoylcysteine decarboxylase phosphopantothenoylcysteine synthetase, FMN-binding
V6EWF0_9PROT	4	17.5	30.44	0.00	1.8E+06	1.7E+06	1.6E+06	7	livF	High-affinity branched-chain amino acid transport ATP-binding protein
V6EZG7_9PROT	6	10.1	72.30	0.00	1.8E+06	1.6E+06	1.6E+06	14	MGMSRv2__0216	Uncharacterized protein
V6EWY1_9PROT	5	6.9	66.39	0.00	1.7E+06	1.7E+06	1.6E+06	7	aspS	Aspartate--tRNA(Asp/Asn) ligase
V6EYD6_9PROT	5	8	67.69	0.00	1.8E+06	1.6E+06	1.5E+06	9	MGMSRv2__0957	Peptidylprolyl isomerase
V6EZ58_9PROT	5	12.8	46.29	0.00	1.7E+06	1.8E+06	1.3E+06	8	clpX	ATP-dependent Clp protease ATP-binding subunit ClpX
V6EZ87_9PROT	3	18.3	25.43	0.00	1.5E+06	1.7E+06	1.5E+06	6	MGMSRv2__0126	Uncharacterized protein
V6F1G3_9PROT	4	11.2	36.32	0.00	1.7E+06	1.7E+06	1.2E+06	9	MGMSRv2__2145	Putative amino-acid ABC transporter, substrate-binding protein
V6F500_9PROT	2	7.1	29.18	0.00	1.5E+06	1.3E+06	1.4E+06	6	MGMSRv2__3380	Putative glycosyltransferase
V6F7Q6_9PROT	6	10.7	59.32	0.00	1.3E+06	1.4E+06	1.4E+06	10	MGMSRv2__3303	Uncharacterized protein

Protein IDs	Unique peptides	Unique sequence coverage [%]	Mol. weight [kDa]	Relative abundance (%)	iBAQ 1	iBAQ 2	iBAQ 3	MS/MS Count	Gene name	Protein description
V6F3L2_9PROT	4	11	45.81	0.00	1.6E+06	8.7E+05	1.5E+06	6	MGMSRv2__2910	Putative Glycosyltransferase
V6F1Z6_9PROT	6	7	100.54	0.00	1.3E+06	1.2E+06	1.3E+06	11	valS	Valine--tRNA ligase
V6F8F5_9PROT	4	25.8	18.31	0.00	1.3E+06	1.3E+06	1.3E+06	6	bfr	Bacterioferritin
V6EYE5_9PROT	2	5.9	48.84	0.00	1.2E+06	1.2E+06	1.3E+06	8	pdhB	Pyruvate dehydrogenase E1 component, beta subunit
V6EZV8_9PROT	3	9.1	43.48	0.00	1.2E+06	1.2E+06	1.2E+06	7	fabF	3-oxoacyl-[acyl-carrier-protein] synthase 2
V6F6X4_9PROT	4	11.3	47.54	0.00	1.2E+06	9.9E+05	1.2E+06	7	MGMSRv2__3989	Chaperone SurA
V6EY46_9PROT	2	5.2	47.12	0.00	1.1E+06	1.1E+06	1.2E+06	6	glgC	Glucose-1-phosphate adenylyltransferase
V6EW90_9PROT	2	5.7	46.02	0.00	1.1E+06	1.0E+06	1.1E+06	6	kdtA	3-deoxy-D-manno-octulosonic-acid transferase (KDO transferase)
V6F6Y7_9PROT	4	9.2	48.24	0.00	9.4E+05	1.1E+06	9.1E+05	7	ctpA	Carboxy-terminal processing protease
V6F0N7_9PROT	4	5.6	95.76	0.00	6.0E+05	5.7E+05	6.0E+05	6	MGMSRv2__1809	Oxidoreductase, FAD/iron-sulfur cluster-binding domain protein
V6F126_9PROT	6	7.9	86.33	0.00	5.5E+05	5.2E+05	5.5E+05	8	MGMSRv2__0983	Putative methyl-accepting chemotaxis protein
V6F7B3_9PROT	3	5.4	62.52	0.00	6.0E+05	4.9E+05	4.9E+05	7	MGMSRv2__4042	Putative transporter subunits of ABC superfamily: ATP-binding components
