# Supplementary material for: Bacterial Magnetosomes Release Iron Ions and Induce Regulation of Iron Homeostasis in Endothelial Cells
Source: Nanomaterials (Basel). 2022 Nov 13;12(22):3995. doi: 10.3390/nano12223995 (PMC9695978; doi:10.3390/nano12223995)
Supplement: Supplementary file 1 [file nanomaterials-12-03995-s001.zip › Table S2.pdf]

Table S2. MAG intrinsic membrane proteins identified by proteomic analysis.

Protein IDs	Unique peptides	Unique sequence coverage [%]	Mol. weight [kDa]	Relative abundance (%)	MS/MS Count	Gene name	Protein description
V6F5J2_9PROT	24	84.8	24.01	0.26	67	mamA	Magnetosome protein MamA
V6F510_9PROT	10	19.5	31.94	0.88	73	mamB	Magnetosome protein MamB, Cation efflux protein
V6F534_9PROT	13	68.8	12.39	18.96	714	mamC	Magnetosome protein MamC
V6F2D1_9PROT	15	45.9	30.23	3.41	158	mamD	Magnetosome protein MamD
V6F2B6_9PROT	32	37.8	78.04	2.29	422	mamE	Magnetosome protein MamE, containing Serine/cysteine peptidase, trypsin-like domain and HrtA/DegP2/Q/S Peptidase S1C domain
V6F254_9PROT	3	27	12.34	0.20	20	mamF	Magnetosome protein MamF
V6F519_9PROT	16	29.8	48.52	0.59	69	mamJ	MamJ protein
V6F2H8_9PROT	5	16.1	37.58	0.01	15	mamK	Magnetosome cytoskeleton protein MamK
V6F235_9PROT	10	24.8	34.49	0.40	81	mamM	Magnetosome protein MamM, containing Cation efflux protein
V6F515_9PROT	10	19.8	65.38	0.64	86	mamO	Probable membrane transporter protein
V6F2H3_9PROT	14	41.9	28.36	2.64	109	mamP	Magnetosome protein MamP, containing PDZ-like domain
V6F2A7_9PROT	5	61.9	9.33	0.05	13	mamR	Magnetosome protein MamR, containing Excisionase/Xis, DNA-binding domain
V6F2G8_9PROT	6	28.9	18.71	0.61	53	mamS	Magnetosome protein MamS
V6F5I9_9PROT	12	35.6	18.88	0.35	51	mamT	MamT protein
V6F5M3_9PROT	9	40.6	15.09	0.11	20	mamW	Magnetosome protein MamW
V6F2C2_9PROT	2	7.1	28.22	1.06	10	mamX	Magnetosome protein MamX
V6F7D9_9PROT	7	23.4	38.41	0.00	9	mmeA	Magnetosome membrane associated protein MmeA
V6F6Z3_9PROT	14	80.7	16.36	0.07	31	mms16	Magnetic particle membrane specific GTPase P16
V6F5K9_9PROT	18	79.9	14.83	12.37	1404	mms6	Magnetosome protein mms6
V6F244_9PROT	17	66.4	18.72	6.65	80	mms5	Magnetosome protein Mms5
