# Supplementary material for: Bacterial Magnetosomes Release Iron Ions and Induce Regulation of Iron Homeostasis in Endothelial Cells
Source: Nanomaterials (Basel). 2022 Nov 13;12(22):3995. doi: 10.3390/nano12223995 (PMC9695978; doi:10.3390/nano12223995)
Supplement: Supplementary file 1 [file nanomaterials-12-03995-s001.zip › Table S3.pdf]

Table S3. Corona protein amount on the nanoparticles measured by BCA assay.

	MAGs + plasma #	IONPs + plasma
Protein/Fe (µg/µg) *	0.018 ± 0.0087	0.098 ± 0.013

* Protein amount is normalized by Fe amount in each sample, data are shown in mean ± SD of three independent experiments. µg/µg, protein amount per microgramme Fe.

# The corona amount on MAGs was calculated by deducting the protein amount on MAGs from total protein amount of MAGs after plasma inclubation.
