# Supplementary material for: Bacterial Magnetosomes Release Iron Ions and Induce Regulation of Iron Homeostasis in Endothelial Cells
Source: Nanomaterials (Basel). 2022 Nov 13;12(22):3995. doi: 10.3390/nano12223995 (PMC9695978; doi:10.3390/nano12223995)
Supplement: Supplementary file 1 [file nanomaterials-12-03995-s001.zip › Table S4.pdf]

Table S4. Zeta potential characterization of MAGs and IONPs.

Nanoparticle	Zeta Potential (mV)	
	In H_2O	In Plasma*
MAGs	-18.6 ± 0.35	-21.7 ± 0.1
IONPs	34.7 ± 1.02	-15.4 ± 0.12

Data are shown in mean ± SD of three independent experiments.

*Zeta potential of nanoparticles in plasma were analyzed by magnet isolation after plasma exposure and resuspended in water by vortex.
