# Supplementary material for: Bacterial Magnetosomes Release Iron Ions and Induce Regulation of Iron Homeostasis in Endothelial Cells
Source: Nanomaterials (Basel). 2022 Nov 13;12(22):3995. doi: 10.3390/nano12223995 (PMC9695978; doi:10.3390/nano12223995)
Supplement: Supplementary file 1 [file nanomaterials-12-03995-s001.zip › Table S5.pdf]

Table S5. Abundance ratios of all identified proteins from TMT-labeled quantitative proteomics of ECs treated with MAGs at 4 timepoints.

Abundance Ratio														
1 d M1/C1	1 d M2/C2	1 d M3/C3	4 d M1/C1	4 d M2/C2	4 d M3/C3	7 d M1/C1	7 d M2/C2	7 d M3/C3	10 d M1/C1	10 d M2/C2	10 d M3/C3	Accession ID	Gene name	Protein Description
1.006	1.019	1.006	0.991	0.964	1.029	0.984	1.018	1.018	0.980	0.985	1.001	Q9C0C9	UBE2O	(E3-independent) E2 ubiquitin-conjugating enzyme
0.993	1.027	1.037	1.005	0.966	1.050	1.037	1.030	0.975	1.023	0.966	1.011	O14874	BCKDK	[3-methyl-2-oxobutanoate dehydrogenase [lipoamide]] kinase, mitochondrial
1.000	0.984	1.027	0.932	0.871	0.962	0.976	0.949	0.942	0.922	0.930	1.063	O94851	MICAL2	[F-actin]-methionine sulfoxide oxidase MICAL2
1.029	0.986	1.016	0.997	0.997	0.986	1.012	1.027	1.002	1.004	0.958	1.023	Q7RTP6	MICAL3	[F-actin]-methionine sulfoxide oxidase MICAL3
1.039	1.016	0.988	0.993	0.961	0.980	1.017	1.003	1.000	0.956	1.035	1.028	Q15118	PDK1	[Pyruvate dehydrogenase (acetyl-transferring)] kinase isozyme 1, mitochondrial
0.988	0.969	1.102	0.987	0.854	1.069	0.978	1.046	1.000				Q15119	PDK2	[Pyruvate dehydrogenase (acetyl-transferring)] kinase isozyme 2, mitochondrial
1.093	1.050	1.044	1.086	0.952	1.058	0.995	1.129	1.036	1.043	0.990	1.031	Q9BV57	ADI1	1,2-dihydroxy-3-keto-5-methylthiopentene dioxygenase
0.998	0.977	0.981	1.013	0.979	1.003	1.019	1.044	1.036	1.019	0.998	0.988	Q04446	GBE1	1,4-alpha-glucan-branching enzyme
0.972	0.986	0.949	0.992	0.959	0.995	0.956	0.963	0.961	0.996	0.966	0.971	P61604	HSPE1	10 kDa heat shock protein, mitochondrial
1.002	1.000	1.030	0.996	1.005	1.024	0.976	1.002	1.021	0.942	1.011	0.981	Q15029	EFTUD2	116 kDa U5 small nuclear ribonucleoprotein component
0.949	0.964	1.004	0.927	1.178	0.967	0.935	0.646	1.016	0.996	0.979	1.041	Q9NRX4	PHPT1	14 kDa phosphohistidine phosphatase
1.042	1.010	0.988	0.982	1.008	0.936	0.992	1.003	0.974	1.014	1.014	1.013	P31946	YWHAB	14-3-3 protein beta/alpha
0.995	1.017	0.993	1.030	1.125	1.000	1.053	0.975	1.026	1.010	1.015	0.983	P62258	YWHAE	14-3-3 protein epsilon
0.991	0.993	0.995	0.992	1.117	0.947	0.982	0.861	1.008	0.999	0.979	1.016	Q04917	YWHAH	14-3-3 protein eta
0.957	1.006	0.989	0.996	1.062	1.006	0.985	0.971	1.030	0.991	1.034	0.974	P61981	YWHAG	14-3-3 protein gamma
0.945	0.993	0.987	0.981	1.052	0.963	0.954	0.930	0.987	0.962	1.004	0.959	P31947	SFN	14-3-3 protein sigma
1.022	0.990	0.997	1.016	1.161	0.969	1.042	0.943	1.062	1.003	1.006	1.028	P27348	YWHAQ	14-3-3 protein theta
1.076	1.019	1.009	1.059	1.293	1.006	1.034	0.972	1.081	1.009	1.050	0.982	P63104	YWHAZ	14-3-3 protein zeta/delta
			0.937	1.118	1.089	0.985	1.010	1.068	1.008	0.881	1.050	Q9BPX1	HSD17B14	17-beta-hydroxysteroid dehydrogenase 14
1.025	0.994	1.031	1.000	0.961	0.972	0.999	0.953	0.983	1.009	0.944	1.043	Q9C0C2	TNKS1BP1	182 kDa tankyrase-1-binding protein
1.021	1.020	1.024	0.978	1.003	0.962	1.009	1.098	1.062	0.911	0.977	1.141	Q8WTS1	ABHD5	1-acylglycerol-3-phosphate O-acyltransferase ABHD5
1.004	0.982	1.007	0.965	0.899	1.050	1.068	1.087	1.029	1.067	1.034	1.112	Q99943	AGPAT1	1-acyl-sn-glycerol-3-phosphate acyltransferase alpha
0.989	1.033	0.977	1.041	0.921	1.065	1.144	0.887	1.146	1.140	1.153	1.044	O15120	AGPAT2	1-acyl-sn-glycerol-3-phosphate acyltransferase beta
1.057	0.936	0.962	0.993	0.970	1.014	1.040	1.008	1.027	1.029	0.964	1.007	Q9NRZ5	AGPAT4	1-acyl-sn-glycerol-3-phosphate acyltransferase delta
1.019	0.982	1.017	0.996	0.940	1.026	0.997	0.897	1.031	0.960	1.066	0.950	Q9NUQ2	AGPAT5	1-acyl-sn-glycerol-3-phosphate acyltransferase epsilon

Abundance Ratio														
1 d M1/C1	1 d M2/C2	1 d M3/C3	4 d M1/C1	4 d M2/C2	4 d M3/C3	7 d M1/C1	7 d M2/C2	7 d M3/C3	10 d M1/C1	10 d M2/C2	10 d M3/C3	Accession ID	Gene name	Protein Description
			1.070	0.872	0.941	0.939	1.165	1.028				Q96QU6	ACCS	1-aminocyclopropane-1-carboxylate synthase-like protein 1
1.062	1.023	1.121	1.007	0.942	1.041	1.094	0.985	0.998	1.008	1.029	1.036	Q9Y2I7	PIKFYVE	1-phosphatidylinositol 3-phosphate 5-kinase
1.009	0.998	1.019	1.009	1.004	1.009	1.006	1.067	1.027	1.016	1.035	1.005	Q01970	PLCB3	1-phosphatidylinositol 4,5-bisphosphate phosphodiesterase beta-3
0.938	0.937	1.110	1.007	0.972	1.037	0.999	1.143	0.989	0.942	1.064	1.131	Q8N3E9	PLCD3	1-phosphatidylinositol 4,5-bisphosphate phosphodiesterase delta-3
1.045	0.967	1.024	0.978	0.969	0.976	0.995	1.126	1.002	0.946	0.902	0.953	Q4KWH8	PLCH1	1-phosphatidylinositol 4,5-bisphosphate phosphodiesterase eta-1
1.000	0.998	1.022	1.007	0.959	1.006	0.989	1.053	0.990	0.932	0.960	0.972	P16885	PLCG2	1-phosphatidylinositol 4,5-bisphosphate phosphodiesterase gamma-2
1.033	1.002	1.052	0.998	0.931	1.064	1.000	0.863	1.027	0.975	0.987	1.021	Q9BZG8	DPH1	2-(3-amino-3-carboxypropyl)histidine synthase subunit 1
1.009	1.000	1.033	0.995	0.962	1.043	0.997	1.051	1.025	1.038	0.980	1.005	Q9BQC3	DPH2	2-(3-amino-3-carboxypropyl)histidine synthase subunit 2
0.986	0.973	0.985	1.012	0.974	1.015	0.986	1.014	0.972	1.008	0.982	0.971	P09543	CNP	2',3'-cyclic-nucleotide 3'-phosphodiesterase
1.017	0.990	0.974	0.982	0.956	0.971	0.993	1.030	0.977	0.993	1.008	0.966	Q16698	DECR1	2,4-dienoyl-CoA reductase, mitochondrial
1.013	0.977	1.033	0.998	0.975	1.020	1.001	1.083	1.004	1.004	0.994	1.021	Q6L8Q7	PDE12	2',5'-phosphodiesterase 12
1.000	1.027	1.012	0.992	1.027	1.003	0.998	1.069	1.034	0.970	0.977	1.006	Q9Y6K5	OAS3	2'-5'-oligoadenylate synthase 3
1.017	0.993	0.999	1.004	1.043	1.023	1.018	1.034	1.016	0.991	0.998	0.991	A0A087X2I1	PSMC6	26S protease regulatory subunit 10B
1.002	0.979	0.999	0.991	0.953	0.997	0.976	1.042	1.010	0.971	0.989	1.000	P62191	PSMC1	26S protease regulatory subunit 4
0.975	0.995	0.986	0.987	0.971	1.012	0.988	1.043	1.001	0.974	0.996	1.000	P17980	PSMC3	26S protease regulatory subunit 6A
1.004	1.001	1.005	0.992	0.969	1.026	1.002	1.035	1.014	0.999	1.014	1.016	P43686	PSMC4	26S protease regulatory subunit 6B
0.993	0.992	1.001	0.983	0.966	1.003	0.984	1.049	1.008	0.977	0.988	1.004	P35998	PSMC2	26S protease regulatory subunit 7
0.984	0.983	0.990	0.996	0.988	1.020	1.005	1.044	1.038	0.994	1.001	1.036	P62195	PSMC5	26S protease regulatory subunit 8
0.984	0.997	0.995	0.991	0.996	1.004	0.993	1.043	1.010	0.959	0.984	0.985	A0A087WW66	PSMD1	26S proteasome non-ATPase regulatory subunit 1
0.996	0.992	0.942	0.983	0.961	1.044	0.962	1.038	1.039	0.935	1.029	0.973	Q99460	PSMD1	26S proteasome non-ATPase regulatory subunit 1
1.014	0.978	1.006	0.995	0.943	0.995	0.998	1.034	0.987	1.013	0.997	0.974	O75832	PSMD10	26S proteasome non-ATPase regulatory subunit 10
0.992	1.013	1.016	1.001	1.018	0.994	0.971	1.035	1.014	0.948	0.993	0.983	O00231	PSMD11	26S proteasome non-ATPase regulatory subunit 11
0.977	0.992	1.003	1.004	0.972	1.018	0.995	1.017	1.027	0.948	0.990	0.999	O00232	PSMD12	26S proteasome non-ATPase regulatory subunit 12
0.995	0.984	1.005	0.997	1.015	0.997	1.000	1.036	0.998	0.983	0.983	0.983	Q9UNM6	PSMD13	26S proteasome non-ATPase regulatory subunit 13
0.923	0.967	0.967	1.001	0.967	0.989	0.959	0.914	0.971	0.956	1.010	0.968	O00487	PSMD14	26S proteasome non-ATPase regulatory subunit 14
1.000	1.011	1.002	1.019	0.988	0.986	0.989	1.021	0.984	0.982	0.994	0.961	Q13200	PSMD2	26S proteasome non-ATPase regulatory subunit 2

Abundance Ratio														
1 d M1/C1	1 d M2/C2	1 d M3/C3	4 d M1/C1	4 d M2/C2	4 d M3/C3	7 d M1/C1	7 d M2/C2	7 d M3/C3	10 d M1/C1	10 d M2/C2	10 d M3/C3	Accession ID	Gene name	Protein Description
0.995	1.009	0.995	0.991	0.968	1.004	0.977	1.019	0.984	0.974	0.983	0.976	O43242	PSMD3	26S proteasome non-ATPase regulatory subunit 3
1.070	1.138	1.001	0.976	0.980	0.864	0.953	1.367	1.049	0.969	1.020	1.068	Q5VWC4	PSMD4	26S proteasome non-ATPase regulatory subunit 4
0.999	0.992	0.991	0.993	1.022	0.996	0.988	0.978	0.986	0.993	0.993	0.985	P55036	PSMD4	26S proteasome non-ATPase regulatory subunit 4
0.987	1.017	1.011	1.007	1.020	1.026	0.988	1.066	1.028	0.990	0.990	1.011	Q16401	PSMD5	26S proteasome non-ATPase regulatory subunit 5
0.981	0.988	0.977	1.001	0.980	1.027	0.978	0.985	1.018	0.967	0.983	0.975	Q15008	PSMD6	26S proteasome non-ATPase regulatory subunit 6
0.984	1.007	0.998	1.001	1.015	0.983	0.948	1.013	0.977	0.931	0.979	0.917	P51665	PSMD7	26S proteasome non-ATPase regulatory subunit 7
1.011	0.993	0.992	1.016	0.987	0.973	0.984	1.059	0.975	0.953	0.991	0.942	P48556	PSMD8	26S proteasome non-ATPase regulatory subunit 8
1.044	0.977	1.017	1.016	1.018	1.010	1.016	0.916	1.026	1.013	0.994	0.993	O00233	PSMD9	26S proteasome non-ATPase regulatory subunit 9
0.969	0.947	1.007	0.924	0.964	1.001	0.857	0.638	0.924	0.981	0.993	0.982	Q13442	PDAP1	28 kDa heat- and acid-stable phosphoprotein
1.032	1.002	1.064	0.972	0.944	1.016	1.000	0.965	1.001	0.987	1.021	0.993	P82664	MRPS10	28S ribosomal protein S10, mitochondrial
1.079	1.080	1.016	1.019	0.973	0.995	1.011	1.052	0.978	1.146	1.037	1.072	P82912	MRPS11	28S ribosomal protein S11, mitochondrial
1.020	1.039	0.966	1.016	1.151	1.004	1.057	0.931	1.051	1.058	0.988	1.078	O15235	MRPS12	28S ribosomal protein S12, mitochondrial
0.961	0.958	1.112	1.000	0.947	1.142	1.006	0.980	1.052	0.941	1.025	1.101	O60783	MRPS14	28S ribosomal protein S14, mitochondrial
0.995	1.009	1.010	0.998	0.962	1.016	0.992	0.940	0.960	1.052	1.029	1.022	P82914	MRPS15	28S ribosomal protein S15, mitochondrial
1.068	0.981	1.032	1.008	1.003	0.959	0.985	0.910	1.006	1.008	1.028	1.003	Q9Y3D3	MRPS16	28S ribosomal protein S16, mitochondrial
1.053	0.984	1.008	1.011	0.973	0.994	1.018	0.987	0.983	1.079	1.031	1.055	Q9Y676	MRPS18B	28S ribosomal protein S18b, mitochondrial
0.975	1.115	0.944	1.025	0.987	0.876	0.982	1.086	1.025	1.023	0.998	0.929	Q9Y3D5	MRPS18C	28S ribosomal protein S18c, mitochondrial
0.998	1.010	1.003	0.980	0.977	1.021	1.014	1.024	1.001	1.050	1.013	1.032	Q9Y399	MRPS2	28S ribosomal protein S2, mitochondrial
0.912	0.986	0.971	0.967	1.023	1.108	1.010	1.004	1.061	1.021	1.043	1.038	A0A075B746	MRPS21	28S ribosomal protein S21, mitochondrial
0.983	1.021	0.977	0.991	0.978	1.000	1.007	1.006	1.011	1.019	0.992	0.987	P82650	MRPS22	28S ribosomal protein S22, mitochondrial
1.070	1.013	0.999	1.011	0.959	0.987	1.030	1.048	1.005	1.065	1.024	1.015	Q9Y3D9	MRPS23	28S ribosomal protein S23, mitochondrial
1.129	0.980	1.077	0.983	0.927	1.005	0.912	0.951	0.956	0.947	0.957	0.931	Q96EL2	MRPS24	28S ribosomal protein S24, mitochondrial
1.012	1.042	0.994	0.997	0.949	0.979	1.008	1.041	1.006	0.997	1.014	1.006	P82663	MRPS25	28S ribosomal protein S25, mitochondrial
1.018	0.989	1.023	0.989	0.967	0.990	1.010	0.964	0.981	1.031	1.022	1.029	Q9BYN8	MRPS26	28S ribosomal protein S26, mitochondrial
0.987	0.990	1.012	0.960	0.997	1.023	1.034	0.985	0.977	0.997	0.961	1.037	Q9Y2Q9	MRPS28	28S ribosomal protein S28, mitochondrial
1.025	1.007	0.986	0.988	0.988	0.976	1.010	0.995	1.011	1.026	1.013	0.979	P51398	DAP3	28S ribosomal protein S29, mitochondrial
0.999	0.981	0.986	0.994	0.974	1.001	0.981	1.022	1.006	0.993	0.982	0.985	Q92665	MRPS31	28S ribosomal protein S31, mitochondrial
1.008	0.979	0.996	1.004	0.984	0.979	0.979	1.072	0.996	0.974	1.017	1.000	Q9Y291	MRPS33	28S ribosomal protein S33, mitochondrial
0.983	1.004	1.009	0.992	1.044	0.985	1.018	1.027	0.990	1.049	1.009	1.062	C9JJ19	MRPS34	28S ribosomal protein S34, mitochondrial
0.952	1.011	1.002	0.975	0.996	1.033	1.022	0.937	1.011	1.054	1.014	1.066	P82673	MRPS35	28S ribosomal protein S35, mitochondrial
0.993	1.025	0.989	1.008	0.932	0.990	0.986	1.041	1.043	0.946	1.020	1.006	P82909	MRPS36	28S ribosomal protein S36, mitochondrial
0.978	0.994	1.017	0.980	0.998	1.036	1.013	0.968	0.985	1.027	1.005	1.059	P82675	MRPS5	28S ribosomal protein S5, mitochondrial
1.020	1.014	0.983	0.954	1.020	0.970	1.039	0.908	1.025	1.087	0.968	1.037	P82932	MRPS6	28S ribosomal protein S6, mitochondrial
1.024	1.020	1.005	0.981	0.994	0.978	1.012	1.053	0.999	0.970	0.970	0.980	J3QLS3	MRPS7	28S ribosomal protein S7, mitochondrial

Abundance Ratio												Accession ID	Gene name	Protein Description
1 d M1/C1	1 d M2/C2	1 d M3/C3	4 d M1/C1	4 d M2/C2	4 d M3/C3	7 d M1/C1	7 d M2/C2	7 d M3/C3	10 d M1/C1	10 d M2/C2	10 d M3/C3			
0.998	1.025	0.986	0.992	0.995	0.998	0.995	0.999	0.999	1.040	1.006	1.036	P82933	MRPS9	28S ribosomal protein S9, mitochondrial
0.909	1.032	0.960	1.006	1.048	1.046	1.180	1.183	1.026	1.322	1.078	1.326	Q8TDX5	ACMSD	2-amino-3-carboxymuconate-6-semialdehyde decarboxylase
0.968	0.985	1.026	0.969	0.972	1.014	1.026	1.027	1.000	1.006	1.001	1.059	Q96SZ5	ADO	2-aminoethanethiol dioxygenase
1.003	0.957	1.063	0.948	0.981	1.002	0.962	1.023	1.033	0.915	0.984	0.974	O43598	DNPH1	2'-deoxynucleoside 5'-phosphate N-hydrolase 1
1.019	1.012	1.025	0.948	0.920	1.020	0.982	1.049	1.012	0.962	0.958	1.019	Q9UJ83	HACL1	2-hydroxyacyl-CoA lyase 1
1.007	0.992	1.024	1.006	0.960	1.060	1.080	1.070	1.054	0.987	1.109	1.110	Q16880	UGT8	2-hydroxyacylsphingosine 1-beta-galactosyltransferase
1.013	1.008	0.980	1.002	1.012	1.012	1.042	0.876	1.018	1.055	0.984	1.050	Q5HYK3	COQ5	2-methoxy-6-polyprenyl-1,4-benzoquinol methylase, mitochondrial
0.911	1.012	1.086	1.025	0.955	1.016	0.991	0.904	1.092	0.810	0.911	1.200	Q6N063	OGFOD2	2-oxoglutarate and iron-dependent oxygenase domain-containing protein 2
1.008	1.012	1.016	1.005	1.056	1.008	1.019	0.978	1.031	1.029	0.991	1.034	Q02218	OGDH	2-oxoglutarate dehydrogenase, mitochondrial
0.926	0.980	1.061	0.987	1.129	1.026	0.998	0.725	1.043	0.985	0.969	1.006	P21953	BCKDHB	2-oxoisovalerate dehydrogenase subunit beta, mitochondrial
			0.982	0.909	0.934	0.957	0.986	1.069	0.956	0.877	1.001	Q9H2F3	HSD3B7	3 beta-hydroxysteroid dehydrogenase type 7
1.038	1.009	1.002	1.021	1.163	0.965	1.029	0.974	1.032	1.025	0.977	1.014	O95861	BPNT1	3'(2'),5'-bisphosphate nucleotidase 1
1.092	1.019	0.959	1.031	1.079	0.939	1.076	1.049	1.007	1.070	0.974	0.966	Q8IV48	ERI1	3'-5' exoribonuclease 1
1.023	0.988	1.009	0.995	0.957	0.989	1.010	1.030	1.000	1.017	0.987	1.028	Q9BYD6	MRPL1	39S ribosomal protein L1, mitochondrial
1.016	0.979	0.965	0.954	0.993	0.982	0.988	0.978	1.011	1.008	0.992	1.017	Q9Y3B7	MRPL11	39S ribosomal protein L11, mitochondrial
1.031	1.019	1.032	1.060	0.953	1.033	0.924	1.064	0.997	0.906	1.024	0.947	P52815	MRPL12	39S ribosomal protein L12, mitochondrial
1.026	1.014	0.993	0.977	0.986	0.975	1.006	1.040	1.002	1.014	0.986	1.016	Q9BYD1	MRPL13	39S ribosomal protein L13, mitochondrial
0.972	0.976	0.984	0.985	0.912	0.981	0.938	0.956	0.988	1.008	0.986	1.012	Q6P1L8	MRPL14	39S ribosomal protein L14, mitochondrial
1.007	1.002	0.973	0.991	0.955	0.976	0.974	1.048	0.983	0.963	0.990	0.917	Q9P015	MRPL15	39S ribosomal protein L15, mitochondrial
1.010	0.953	0.979	1.010	1.071	0.987	0.998	0.997	1.013	0.962	1.001	0.963	Q9NX20	MRPL16	39S ribosomal protein L16, mitochondrial
0.979	0.971	0.937	0.982	0.959	1.024	0.976	0.915	1.011	1.017	0.991	1.055	Q9NRX2	MRPL17	39S ribosomal protein L17, mitochondrial
1.043	1.001	1.064	1.000	1.023	1.013	1.013	1.025	0.976	1.000	0.980	1.060	Q9H0U6	MRPL18	39S ribosomal protein L18, mitochondrial
1.006	1.011	0.984	0.973	0.958	0.996	0.997	1.030	0.988	1.027	0.978	1.018	P49406	MRPL19	39S ribosomal protein L19, mitochondrial
1.000	1.027	0.943	1.000	0.987	1.006	1.003	0.980	0.998	1.048	0.990	1.007	Q5T653	MRPL2	39S ribosomal protein L2, mitochondrial
1.064	0.997	0.991	1.026	0.965	1.022	1.019	1.078	1.041	1.004	0.966	1.050	Q9BYC9	MRPL20	39S ribosomal protein L20, mitochondrial
1.034	1.013	0.986	0.987	0.970	0.999	0.983	0.980	0.989	1.064	1.006	1.032	Q7Z2W9	MRPL21	39S ribosomal protein L21, mitochondrial
0.989	0.986	0.998	0.957	0.990	1.000	0.993	0.886	1.015	1.023	1.002	1.026	J3KQY1	MRPL22	39S ribosomal protein L22, mitochondrial
1.075	1.054	1.017	0.989	0.932	0.944	0.953	1.162	0.940	0.946	1.007	1.002	Q16540	MRPL23	39S ribosomal protein L23, mitochondrial
0.972	0.993	0.990	0.993	1.032	0.998	1.042	0.951	1.038	1.016	1.011	1.005	Q96A35	MRPL24	39S ribosomal protein L24, mitochondrial
1.053	1.024	1.019	0.982	1.001	0.966	0.991	0.996	0.975	1.045	0.979	1.022	Q9P0M9	MRPL27	39S ribosomal protein L27, mitochondrial
1.066	0.994	1.037	1.040	0.999	1.039	1.000	1.015	1.022	1.048	1.002	1.020	Q13084	MRPL28	39S ribosomal protein L28, mitochondrial

Abundance Ratio												Accession ID	Gene name	Protein Description
1 d M1/C1	1 d M2/C2	1 d M3/C3	4 d M1/C1	4 d M2/C2	4 d M3/C3	7 d M1/C1	7 d M2/C2	7 d M3/C3	10 d M1/C1	10 d M2/C2	10 d M3/C3			
0.969	0.990	0.982	0.984	0.958	1.023	1.028	0.989	1.007	1.040	1.025	1.059	E7ETU7	MRPL3	39S ribosomal protein L3, mitochondrial
1.028	1.002	0.994	0.963	0.951	1.007	0.917	0.904	0.988	1.041	1.016	1.056	Q9BYC8	MRPL32	39S ribosomal protein L32, mitochondrial
1.022	1.002	0.975	1.006	1.024	0.922	1.012	0.973	1.037	1.005	0.990	1.036	O75394	MRPL33	39S ribosomal protein L33, mitochondrial
1.080	1.056	1.010	0.980	0.910	0.963	0.990	1.029	0.956	1.090	1.003	0.992	M0R226	MRPL34	39S ribosomal protein L34, mitochondrial
0.996	1.046	0.981	1.051	1.212	1.033	1.089	1.025	1.034	1.057	1.012	0.995	Q9NZE8	MRPL35	39S ribosomal protein L35, mitochondrial
1.002	0.998	1.006	0.987	0.973	0.971	1.017	1.041	0.988	1.019	0.989	1.018	Q9BZE1	MRPL37	39S ribosomal protein L37, mitochondrial
0.969	0.987	0.972	0.960	0.966	1.014	1.022	1.009	1.046	1.027	1.029	1.034	Q96DV4	MRPL38	39S ribosomal protein L38, mitochondrial
0.953	0.984	0.976	0.973	0.928	0.983	0.996	0.973	0.985	1.035	0.975	1.032	Q9NYK5	MRPL39	39S ribosomal protein L39, mitochondrial
1.016	1.015	0.994	0.955	0.944	0.996	0.999	1.070	1.002	0.991	1.008	0.980	Q9BYD3	MRPL4	39S ribosomal protein L4, mitochondrial
1.083	0.973	1.016	1.016	0.958	1.013	1.011	1.028	0.997	0.966	0.971	0.974	Q9NQ50	MRPL40	39S ribosomal protein L40, mitochondrial
0.959	0.969	0.926	1.015	0.977	1.017	1.008	0.998	1.024	1.014	0.971	0.988	Q8IXM3	MRPL41	39S ribosomal protein L41, mitochondrial
0.868	0.949	0.966	0.959	0.923	1.064	0.949	0.961	0.950	1.109	0.938	0.989	S4R2Z7	MRPL42	39S ribosomal protein L42, mitochondrial
0.967	1.010	1.039	0.973	1.004	1.001	0.982	0.988	1.011	0.985	1.006	0.969	Q9H9J2	MRPL44	39S ribosomal protein L44, mitochondrial
1.017	1.004	0.967	0.991	0.967	0.973	0.999	1.048	0.994	1.009	1.005	1.017	A0A087X2D5	MRPL45	39S ribosomal protein L45, mitochondrial
1.052	0.967	0.997	0.944	0.944	1.000	0.986	0.939	0.983	1.015	0.959	1.032	Q9H2W6	MRPL46	39S ribosomal protein L46, mitochondrial
1.048	0.989	1.006	0.995	1.124	0.990	1.022	0.992	1.077	1.062	0.974	1.015	Q9HD33	MRPL47	39S ribosomal protein L47, mitochondrial
0.984	1.051	1.000	0.957	0.939	1.024	1.005	0.982	0.953	1.056	1.008	1.073	Q96GC5	MRPL48	39S ribosomal protein L48, mitochondrial
1.025	0.983	1.015	0.939	0.966	1.016	1.019	1.019	1.030	1.041	0.962	1.062	Q13405	MRPL49	39S ribosomal protein L49, mitochondrial
1.034	1.003	1.021	0.976	0.909	1.008	0.994	1.038	1.002	1.031	1.006	1.010	Q8N5N7	MRPL50	39S ribosomal protein L50, mitochondrial
0.904	1.032	0.985	1.001	0.897	0.984	1.041	1.090	1.019	1.029	0.953	0.996	Q4U2R6	MRPL51	39S ribosomal protein L51, mitochondrial
1.070	0.923	1.147	0.893	0.948	1.012	0.871	0.878	0.958	0.872	0.966	0.962	Q86TS9	MRPL52	39S ribosomal protein L52, mitochondrial
1.030	1.045	0.986	1.005	1.053	1.004	0.990	0.995	1.004	1.069	1.016	1.071	Q96EL3	MRPL53	39S ribosomal protein L53, mitochondrial
0.964	0.928	0.955	0.963	0.939	0.961	0.946	0.989	1.002	1.101	1.001	1.140	Q6P161	MRPL54	39S ribosomal protein L54, mitochondrial
1.044	0.985	0.985	0.991	0.969	0.971	0.981	1.022	0.998	1.000	0.974	1.033	Q9BYD2	MRPL9	39S ribosomal protein L9, mitochondrial
1.089	1.020	1.005	1.011	1.030	0.971	0.937	1.012	0.927	0.955	0.997	0.864	Q9NVS2	MRPS18A	39S ribosomal protein S18a, mitochondrial
1.029	1.040	1.018	0.991	1.018	0.970	1.015	1.051	1.025	1.049	1.015	1.038	Q9NP92	MRPS30	39S ribosomal protein S30, mitochondrial
0.732	0.980	0.814	0.863	0.802	1.075	1.175	1.114	0.999				Q15125	EBP	3-beta-hydroxysteroid-Delta(8),Delta(7)-isomerase
1.006	1.011	0.991	0.982	1.027	0.969	0.989	1.002	1.030	0.969	1.006	0.963	Q99714	HSD17B10	3-hydroxyacyl-CoA dehydrogenase type-2
1.134	0.879	1.103	1.025	1.000	0.963	1.104	0.993	0.919	1.100	0.900	1.075	Q9BUT1	BDH2	3-hydroxybutyrate dehydrogenase type 2
0.988	1.017	0.984	1.028	1.015	0.993	1.037	1.052	1.001	1.064	0.970	0.997	P31937	HIBADH	3-hydroxyisobutyrate dehydrogenase, mitochondrial
0.965	0.999	0.996	0.976	0.995	1.021	1.016	0.975	0.999	1.007	0.979	1.000	Q6NVY1	HIBCH	3-hydroxyisobutyryl-CoA hydrolase, mitochondrial
0.988	0.991	0.984	0.978	1.008	0.967	0.947	0.949	0.960	0.954	0.975	0.956	P42765	ACAA2	3-ketoacyl-CoA thiolase, mitochondrial

Abundance Ratio														
1 d M1/C1	1 d M2/C2	1 d M3/C3	4 d M1/C1	4 d M2/C2	4 d M3/C3	7 d M1/C1	7 d M2/C2	7 d M3/C3	10 d M1/C1	10 d M2/C2	10 d M3/C3	Accession ID	Gene name	Protein Description
0.996	0.979	1.002	0.963	0.968	1.017	1.004	1.027	0.995	0.980	0.971	1.007	P09110	ACAA1	3-ketoacyl-CoA thiolase, peroxisomal
1.001	1.020	0.985	1.000	1.114	0.970	1.009	1.041	1.055	0.932	1.036	0.942	Q06136	KDSR	3-ketodihydrosphingosine reductase
1.055	0.980	0.991	1.018	0.929	1.081	1.099	1.113	0.996	1.048	1.017	1.006	P56937	HSD17B7	3-keto-steroid reductase
0.890	0.959	1.183										P18405	SRD5A1	3-oxo-5-alpha-steroid 4-dehydrogenase 1
1.021	0.997	1.056	0.995	0.997	1.008	1.006	0.996	1.034	1.022	0.973	1.043	Q9NWU1	OXSM	3-oxoacyl-[acyl-carrier-protein] synthase, mitochondrial
1.070	1.006	1.031	1.008	0.977	0.999	1.019	1.102	1.067	1.030	0.945	1.052	O15530	PDPK1	3-phosphoinositide-dependent protein kinase 1
1.043	1.001	1.002	1.021	0.977	1.024	1.015	1.012	0.995	1.038	1.033	1.019	P46783	RPS10	40S ribosomal protein S10
1.114	0.998	0.986	1.040	1.092	0.960	1.011	0.988	0.982	0.997	1.042	0.944	P62280	RPS11	40S ribosomal protein S11
1.241	1.014	1.054	1.065	1.678	0.951	1.122	0.807	1.087	0.987	0.993	0.953	P25398	RPS12	40S ribosomal protein S12
1.039	0.990	1.006	1.020	0.983	1.008	1.010	1.033	0.993	1.031	1.003	1.008	P62277	RPS13	40S ribosomal protein S13
0.996	1.007	0.980	1.008	0.967	0.968	0.973	1.033	0.976	0.975	1.012	0.993	P62263	RPS14	40S ribosomal protein S14
0.947	1.062	0.945	1.038	1.013	0.961	1.030	1.067	0.953	1.008	1.045	0.940	K7ELC2	RPS15	40S ribosomal protein S15
0.972	0.944	0.941							1.130	1.004	1.435	K7EJ78	RPS15	40S ribosomal protein S15
1.147	0.964	0.970	1.049	1.407	0.940	1.031	0.934	1.064	0.972	1.012	0.998	P62244	RPS15A	40S ribosomal protein S15a
1.072	0.989	0.999	1.015	1.037	1.011	0.988	0.990	1.007	0.985	0.999	0.929	P62249	RPS16	40S ribosomal protein S16
0.973	0.997	0.954	1.031	1.081	0.972	0.995	0.996	1.004	0.993	1.033	0.948	P08708	RPS17	40S ribosomal protein S17
1.012	1.034	0.936	0.899	0.957	0.998				1.064	1.069	1.183	A0A0G2JQH2	RPS18	40S ribosomal protein S18
1.024	1.015	0.990	1.028	0.983	1.008	0.989	1.030	0.990	1.042	1.046	1.026	P62269	RPS18	40S ribosomal protein S18
1.015	0.981	0.969	0.992	0.973	1.000	0.995	0.995	0.985	1.040	1.020	0.988	P39019	RPS19	40S ribosomal protein S19
1.085	1.000	0.999	1.018	1.074	1.019	1.024	1.025	1.034	0.976	1.018	0.965	P15880	RPS2	40S ribosomal protein S2
1.027	1.014	0.976	1.000	0.991	0.998	1.027	0.966	1.025	1.041	1.015	1.017	P60866	RPS20	40S ribosomal protein S20
1.077	0.983	0.987	1.053	1.209	0.988	1.052	0.913	0.997	1.041	1.014	1.024	P63220	RPS21	40S ribosomal protein S21
1.035	0.996	0.982	1.007	1.001	0.973	0.955	1.004	0.955	0.964	1.032	0.926	P62266	RPS23	40S ribosomal protein S23
1.042	0.988	1.001	0.972	0.999	1.040	0.958	0.952	0.989	1.025	1.041	1.032	P62851	RPS25	40S ribosomal protein S25
1.026	0.985	0.999	0.998	1.108	0.975	0.981	0.967	1.012	0.967	1.025	0.978	P62854	RPS26	40S ribosomal protein S26
1.014	0.920	1.013	1.022	0.977	1.080	0.950	0.949	0.994	1.058	1.027	1.004	P42677	RPS27	40S ribosomal protein S27
1.052	1.031	0.951	1.043	1.026	0.958	1.081	1.105	0.971	1.143	1.042	0.983	H0YMV8	RPS27L	40S ribosomal protein S27
1.054	1.023	1.019	1.044	1.179	1.017	0.995	0.964	1.122	1.051	1.056	1.068	P62857	RPS28	40S ribosomal protein S28
0.994	0.998	0.976	1.010	1.030	0.998	1.017	1.052	1.009	0.998	1.011	1.002	P23396	RPS3	40S ribosomal protein S3
0.954	0.903	0.907	0.892	0.989	0.976	0.987	0.870	0.965	0.994	0.903	0.923	E9PR30	FAU	40S ribosomal protein S30
1.050	1.006	1.012	1.023	1.085	0.981	1.007	0.996	1.023	0.988	1.029	0.997	P61247	RPS3A	40S ribosomal protein S3a
1.033	1.017	0.988	1.032	1.047	1.018	0.991	1.017	1.010	0.991	1.032	0.957	P62701	RPS4X	40S ribosomal protein S4, X isoform
1.097	0.998	1.031	1.043	1.293	0.980	1.091	0.868	1.111	1.018	1.027	1.024	P46782	RPS5	40S ribosomal protein S5

Abundance Ratio												Accession ID	Gene name	Protein Description
1 d M1/C1	1 d M2/C2	1 d M3/C3	4 d M1/C1	4 d M2/C2	4 d M3/C3	7 d M1/C1	7 d M2/C2	7 d M3/C3	10 d M1/C1	10 d M2/C2	10 d M3/C3			
1.056	1.003	1.006	1.003	1.094	1.013	0.984	0.859	1.018	1.040	1.045	1.001	P62753	RPS6	40S ribosomal protein S6
1.026	1.000	0.997	0.988	0.988	1.010	0.963	0.977	0.958	1.001	1.017	0.963	P62081	RPS7	40S ribosomal protein S7
1.049	1.004	1.007	1.022	1.047	0.950	0.958	0.952	0.991	0.903	1.035	0.872	P62241	RPS8	40S ribosomal protein S8
0.977	0.971	1.002	0.999	0.958	1.027	0.981	0.997	1.003	1.010	1.009	1.023	P46781	RPS9	40S ribosomal protein S9
1.118	1.040	1.085				1.144	1.018	1.088				F8WD59	RPSA	40S ribosomal protein SA (Fragment)
1.040	1.014	0.977	1.016	1.040	0.955	1.002	1.069	1.009	0.989	1.017	0.961	A0A0C4DG17	RPSA	40S ribosomal protein SA
1.126	1.171	1.184	1.270	1.195	1.294	1.062	0.992	1.099	1.123	1.030	1.093	Q9BRK5	SDF4	45 kDa calcium-binding protein
0.990	1.008	0.990	1.001	1.000	0.980	1.013	1.010	1.001	0.968	0.946	1.006	P49189	ALDH9A1	4-trimethylaminobutyraldehyde dehydrogenase
1.096	1.289	1.201										Q9H816	DCLRE1B	5' exonuclease Apollo
1.065	1.015	1.079	1.068	1.156	1.113	1.047	1.087	1.107	1.100	1.036	1.104	Q8TCD5	NT5C	5'(3')-deoxyribonucleotidase, cytosolic type
1.134	1.041	1.124	1.025	0.789	1.036	1.015	1.090	1.045	0.930	0.943	1.035	O43422	THAP12	52 kDa repressor of the inhibitor of the protein kinase
1.064	0.969	1.015	0.985	0.975	1.003	1.001	1.017	1.019	1.011	0.966	0.997	Q8IZH2	XRN1	5'-3' exoribonuclease 1
1.024	0.997	1.055	1.013	0.973	1.011	1.017	1.025	1.008	0.993	1.012	0.980	Q9H0D6	XRN2	5'-3' exoribonuclease 2
0.909	0.927	0.942	0.860	0.611	0.883				0.879	0.969	1.186	Q00013	MPP1	55 kDa erythrocyte membrane protein
0.999	1.375	1.152	1.044	0.931	1.059	1.140	0.955	0.975	1.006	1.052	1.116	P13196	ALAS1	5-aminolevulinate synthase, nonspecific, mitochondrial
1.082	1.088	0.969	1.016	1.052	1.012	1.053	1.048	1.047	1.157	0.994	1.053	P54646	PRKAA2	5'-AMP-activated protein kinase catalytic subunit alpha-2
1.072	1.052	1.026	1.035	0.958	0.964	0.976	1.096	1.029	0.984	0.978	0.936	Q9Y478	PRKAB1	5'-AMP-activated protein kinase subunit beta-1
0.984	0.989	0.991	0.978	1.009	0.965	0.982	0.995	0.922	0.867	1.024	1.065	O43741	PRKAB2	5'-AMP-activated protein kinase subunit beta-2
1.053	0.994	1.029	1.005	0.953	1.038	1.023	1.078	1.018	0.951	0.990	0.980	P54619	PRKAG1	5'-AMP-activated protein kinase subunit gamma-1
0.997	0.989	1.013	0.971	0.960	1.009	0.985	1.054	0.986	0.956	0.926	1.003	Q9UGJ0	PRKAG2	5'-AMP-activated protein kinase subunit gamma-2
0.927	0.988	0.995	1.048	0.973	1.077	0.966	0.908	0.893	1.045	1.047	0.957	Q9H6S1	AZI2	5-azacytidine-induced protein 2
1.019	1.003	0.999	1.011	0.947	0.994	0.988	1.020	0.964	1.005	1.002	1.030	Q99807	COQ7	5-demethoxyubiquinone hydroxylase, mitochondrial
1.054	1.013	1.028	0.992	0.967	1.005	1.024	0.994	1.015	1.066	1.010	1.024	P49914	MTHFS	5-formyltetrahydrofolate cyclo-ligase
1.046	0.969	1.033	0.994	0.961	1.068	1.085	0.948	0.987	1.057	0.978	0.979	Q96CB9	NSUN4	5-methylcytosine rRNA methyltransferase NSUN4
0.975	1.048	0.962	1.012	0.977	1.022	0.988	0.999	1.009	0.974	1.009	0.983	Q5TFE4	NT5DC1	5'-nucleotidase domain-containing protein 1
0.991	0.997	1.028	1.012	0.962	1.000	0.985	1.088	1.063	0.943	0.991	1.017	Q86UY8	NT5DC3	5'-nucleotidase domain-containing protein 3
1.011	1.014	1.024	1.093	0.985	1.034	1.121	1.133	1.095	1.128	1.062	1.175	P21589	NT5E	5'-nucleotidase
0.977	1.002	1.026	0.988	1.043	1.029	1.022	0.913	1.021	1.017	0.980	1.060	O14841	OPLAH	5-oxoprolinase
1.058	1.131	1.076	1.004	0.901	0.984	0.982	1.090	1.010	1.055	1.098	1.049	Q8IUZ5	PHYKPL	5-phosphohydroxy-L-lysine phospho-lyase
1.070	1.000	1.011	0.981	1.053	0.985	0.981	1.002	0.991	1.026	0.995	0.991	P56378	MP68	6.8 kDa mitochondrial proteolipid

Abundance Ratio														
1 d M1/C1	1 d M2/C2	1 d M3/C3	4 d M1/C1	4 d M2/C2	4 d M3/C3	7 d M1/C1	7 d M2/C2	7 d M3/C3	10 d M1/C1	10 d M2/C2	10 d M3/C3	Accession ID	Gene name	Protein Description
0.950	1.006	0.986	0.993	1.017	1.024	0.990	1.014	1.018	0.977	0.993	0.974	P10809	HSPD1	60 kDa heat shock protein, mitochondrial
1.008	1.000	1.014	1.017	1.003	1.004	1.014	0.949	1.006	1.007	0.976	0.970	P10155	TROVE2	60 kDa SS-A/Ro ribonucleoprotein
			0.880	0.861	1.064	0.890	1.070	1.026	1.007	0.849	1.038	F8VZS0	RPLP0	60S acidic ribosomal protein P0 (Fragment)
1.010	0.998	1.003	1.010	1.048	0.987	1.016	1.030	1.036	0.985	1.027	0.989	P05388	RPLP0	60S acidic ribosomal protein P0
1.090	0.957	0.954	0.951	1.088	0.991	0.948	1.011	1.331	0.932	0.996	0.977	P05386	RPLP1	60S acidic ribosomal protein P1
1.032	1.012	1.006	1.006	0.943	0.976	0.960	1.095	0.977	0.971	1.021	0.999	P05387	RPLP2	60S acidic ribosomal protein P2
0.994	1.050	0.985	1.014	0.999	1.018	0.971	1.098	0.974	1.008	1.039	1.022	C9JA08	NMD3	60S ribosomal export protein NMD3
1.131	0.987	1.077	1.071	0.923	0.805	0.746	0.911	0.843	0.641	1.181	0.643	F8W7C6	RPL10	60S ribosomal protein L10
1.036	0.977	1.011	1.020	1.101	0.957	0.958	0.874	0.995	0.936	1.011	0.922	P27635	RPL10	60S ribosomal protein L10
1.024	1.004	0.999	1.027	1.071	0.990	1.012	0.980	1.035	0.932	1.052	0.900	P62906	RPL10A	60S ribosomal protein L10a
1.025	1.009	0.997	1.012	1.133	1.017	0.997	0.946	1.076	1.020	1.037	1.016	P62913	RPL11	60S ribosomal protein L11
1.053	0.984	0.975	1.025	1.099	0.971	1.019	0.988	1.012	1.016	1.005	0.987	P30050	RPL12	60S ribosomal protein L12
0.956	0.988	0.954	1.004	0.967	1.014	0.991	0.905	0.997	1.063	1.022	1.028	P26373	RPL13	60S ribosomal protein L13
0.992	1.029	0.990	1.006	1.037	1.001	1.000	1.005	0.990	1.049	1.016	0.993	P40429	RPL13A	60S ribosomal protein L13a
1.068	0.961	1.063	0.991	1.109	0.976	0.882	0.775	0.982	0.866	1.035	0.898	P50914	RPL14	60S ribosomal protein L14
1.040	1.000	0.993	1.004	0.989	1.026	0.967	0.958	1.013	1.002	1.037	1.003	P61313	RPL15	60S ribosomal protein L15
1.034	1.012	1.020	0.982	1.022	1.012	0.986	1.017	1.017	0.998	1.040	1.019	G3V203	RPL18	60S ribosomal protein L18
1.013	1.020	0.980	1.040	1.136	0.985	1.016	0.905	0.982	1.020	1.026	0.956	Q02543	RPL18A	60S ribosomal protein L18a
0.949	1.044	0.971	0.996	0.994	1.007	0.969	0.999	0.983	1.031	1.059	0.975	P84098	RPL19	60S ribosomal protein L19
0.944	0.978	0.933	0.983	0.984	0.985	0.961	0.951	0.961	0.984	0.995	0.977	P46778	RPL21	60S ribosomal protein L21
1.003	0.971	0.981	1.009	1.000	0.995	0.966	1.047	1.035	0.930	1.010	0.971	P35268	RPL22	60S ribosomal protein L22
1.021	1.011	0.983	1.012	1.061	0.998	1.014	1.043	1.034	0.996	1.021	0.990	P62829	RPL23	60S ribosomal protein L23
0.997	0.994	0.978	0.993	0.954	1.008	0.989	1.023	0.986	1.028	1.013	0.999	P62750	RPL23A	60S ribosomal protein L23a
1.000	1.009	0.992	0.987	1.118	1.022	0.964	0.862	1.005	0.998	1.029	1.010	P83731	RPL24	60S ribosomal protein L24
1.043	1.007	1.007	0.986	0.936	1.053	0.996	0.999	0.961	0.942	1.000	0.877	P61254	RPL26	60S ribosomal protein L26
1.114	1.090	0.993	1.078	0.928	1.080	0.952	0.932	0.973	1.175	1.054	0.994	Q9UNX3	RPL26L1	60S ribosomal protein L26-like 1
1.015	1.010	0.978	0.994	0.958	0.973	0.992	1.060	0.969	0.977	1.012	0.997	P61353	RPL27	60S ribosomal protein L27
1.073	0.999	0.983	1.025	1.139	0.989	1.005	0.927	1.022	1.053	0.997	0.991	P46776	RPL27A	60S ribosomal protein L27a
1.100	1.011	0.996	1.010	0.998	0.997	0.993	0.957	0.950	0.973	1.014	0.919	P46779	RPL28	60S ribosomal protein L28
0.965	0.943	0.994	0.939	1.016	0.948	0.899	0.733	0.906	1.042	1.046	0.988	P47914	RPL29	60S ribosomal protein L29
0.935	1.006	0.943	1.016	1.060	1.010	1.025	0.961	0.990	1.039	1.040	1.031	P39023	RPL3	60S ribosomal protein L3
1.005	1.001	1.004	1.039	1.128	0.968	0.976	0.899	1.010	0.936	1.015	0.952	P62888	RPL30	60S ribosomal protein L30
0.996	1.013	0.956	0.980	0.995	1.032	0.975	0.959	0.946	1.091	1.036	1.021	P62899	RPL31	60S ribosomal protein L31
						1.026	1.011	1.055				D3YTI8	RPL32	60S ribosomal protein L32 (Fragment)

Abundance Ratio														
1 d M1/C1	1 d M2/C2	1 d M3/C3	4 d M1/C1	4 d M2/C2	4 d M3/C3	7 d M1/C1	7 d M2/C2	7 d M3/C3	10 d M1/C1	10 d M2/C2	10 d M3/C3	Accession ID	Gene name	Protein Description
1.000	1.026	0.974	1.041	1.107	0.968	0.985	0.949	0.995	0.950	1.023	0.908	F8W727	RPL32	60S ribosomal protein L32
1.213	1.014	0.995	1.020	1.315	0.952	1.056	0.794	0.980	1.046	1.029	0.969	P49207	RPL34	60S ribosomal protein L34
1.054	1.017	0.998	0.942	0.949	0.996	0.871	0.841	0.925	0.948	1.063	0.916	P42766	RPL35	60S ribosomal protein L35
1.060	1.002	1.001	1.037	1.076	0.994	1.019	0.900	0.993	1.015	1.039	0.968	P18077	RPL35A	60S ribosomal protein L35a
0.941	1.020	0.941	1.014	1.063	1.032	1.044	1.016	1.025	1.073	1.023	1.019	Q9Y3U8	RPL36	60S ribosomal protein L36
1.058	1.014	0.951	0.988	1.036	0.999	1.025	0.914	0.966	1.041	1.007	0.935	J3KQN4	RPL36A	60S ribosomal protein L36a
0.991	0.997	0.971	1.007	0.925	1.035	1.105	0.940	0.937	0.945	0.892	0.986	Q969Q0	RPL36AL	60S ribosomal protein L36a-like
0.901	0.900	1.040	0.833	1.106	0.941	0.720	0.509	0.910	0.900	1.119	1.105	P61927	RPL37	60S ribosomal protein L37
1.286	1.025	1.060	1.073	1.205	0.937	0.973	0.886	0.950	0.878	1.023	0.811	P61513	RPL37A	60S ribosomal protein L37a
1.051	1.025	1.006	1.017	0.991	1.014	0.996	1.098	1.046	1.007	1.010	0.987	P63173	RPL38	60S ribosomal protein L38
1.075	1.059	0.902	0.970	0.978	1.045	0.915	1.048	1.013	0.955	1.006	0.977	P62891	RPL39	60S ribosomal protein L39
1.009	1.001	1.006	1.012	1.085	1.006	0.976	0.950	1.032	0.969	1.011	0.952	P36578	RPL4	60S ribosomal protein L4
0.944	1.028	0.932	1.021	1.080	0.976	1.036	1.000	0.964	1.044	1.067	0.969	P46777	RPL5	60S ribosomal protein L5
			1.180	0.815	1.566							F8VR69	RPL6	60S ribosomal protein L6 (Fragment)
1.024	1.008	1.013	0.994	0.948	0.990	0.943	0.964	0.961	0.974	1.011	0.933	Q02878	RPL6	60S ribosomal protein L6
1.017	1.026	0.980	1.020	1.053	1.002	0.998	1.054	1.029	1.034	1.016	0.987	P18124	RPL7	60S ribosomal protein L7
1.021	0.999	0.951	1.002	1.064	0.962	1.001	1.013	0.993	1.007	1.022	0.963	P62424	RPL7A	60S ribosomal protein L7a
1.075	1.028	1.017	1.029	0.989	1.041	0.982	1.122	1.009	0.974	1.052	0.972	Q6DKI1	RPL7L1	60S ribosomal protein L7-like 1
1.054	0.992	0.943	1.022	0.984	0.943	0.975	0.970	0.936	0.980	0.991	0.913	P62917	RPL8	60S ribosomal protein L8
1.037	1.002	1.007	1.038	1.071	0.966	1.010	1.042	0.974	1.005	1.041	0.988	P32969	RPL9	60S ribosomal protein L9
1.088	0.987	0.979	1.047	1.215	0.977	1.082	0.979	1.084	1.063	0.961	0.988	Q9Y221	NIP7	60S ribosome subunit biogenesis protein NIP7 homolog
1.061	1.010	1.030	1.034	1.006	1.029	1.006	1.054	1.013	1.032	1.058	0.995	O60825	PFKFB2	6-phosphofructo-2-kinase/fructose-2,6-bisphosphatase 2
0.975	0.953	1.143	1.033	0.930	1.013	1.087	1.001	0.958	1.084	1.045	1.043	Q5VX20	PFKFB3	6-phosphofructo-2-kinase/fructose-2,6-bisphosphatase 3
0.901	0.911	0.923	1.084	0.891	1.012	1.065	1.144	0.880				Q16877	PFKFB4	6-phosphofructo-2-kinase/fructose-2,6-bisphosphatase 4
0.945	0.994	1.000	0.981	1.024	0.996	0.998	0.991	1.012	1.024	1.019	1.052	P52209	PGD	6-phosphogluconate dehydrogenase, decarboxylating
1.029	1.005	1.007	0.986	1.025	0.974	1.020	1.002	0.993	1.020	0.974	1.009	O95336	PGLS	6-phosphogluconolactonase
			1.075	0.863	1.129	0.986	1.105	1.013	1.048	1.023	1.074	Q03393	PTS	6-pyruvoyl tetrahydrobiopterin synthase
0.982	1.007	0.954	0.958	0.951	1.009	0.998	0.980	0.995	0.959	0.967	1.009	P36639	NUDT1	7,8-dihydro-8-oxoguanine triphosphatase
1.001	1.018	1.001	0.999	0.968	0.975	0.986	1.033	0.960	0.937	0.964	0.934	P11021	HSPA5	78 kDa glucose-regulated protein
0.899	1.005	0.957	0.958	0.935	1.027	1.129	1.098	1.042	1.274	1.094	1.132	Q9UBM7	DHCR7	7-dehydrocholesterol reductase

Abundance Ratio														
1 d M1/C1	1 d M2/C2	1 d M3/C3	4 d M1/C1	4 d M2/C2	4 d M3/C3	7 d M1/C1	7 d M2/C2	7 d M3/C3	10 d M1/C1	10 d M2/C2	10 d M3/C3	Accession ID	Gene name	Protein Description
1.016	0.991	1.025	0.997	1.037	0.930	1.016	1.066	1.008	0.957	0.992	0.972	Q969T7	NT5C3B	7-methylguanosine phosphate-specific 5'-nucleotidase
1.063	0.982	1.010	0.999	0.938	1.020	1.003	1.053	0.989	1.033	0.976	0.968	Q7L2J0	MEPCE	7SK snRNA methylphosphate capping enzyme
0.978	1.051	1.161										O60733	PLA2G6	85/88 kDa calcium-independent phospholipase A2
			1.066	0.919	0.946	0.953	1.163	1.106	0.928	1.013	0.790	Q6ZVK8	NUDT18	8-oxo-dGDP phosphatase NUDT18
1.363	0.758	0.976	1.091	0.974	0.917	0.864	1.193	1.136	0.989	1.043	1.061	Q9UHI8	ADAMTS1	A disintegrin and metalloproteinase with thrombospondin motifs 1
			1.112	0.861	0.762							Q9UKP4	ADAMTS7	A disintegrin and metalloproteinase with thrombospondin motifs 7
1.080	1.040	0.995	0.956	0.905	1.003	1.031	1.107	1.042	0.974	0.959	1.013	P42684	ABL2	Abelson tyrosine-protein kinase 2
1.028	1.052	0.975	1.018	0.992	0.978	1.013	1.035	0.970	1.043	0.984	1.006	Q8IZP0	ABI1	Abl interactor 1
1.004	1.004	1.018	1.012	0.975	1.001	0.926	0.923	1.005	1.026	1.052	0.983	F8WAL6	ABI2	Abl interactor 2
1.009	1.066	1.014	1.042	0.960	1.020	1.164	1.256	1.166				Q8IZT6	ASPM	Abnormal spindle-like microcephaly-associated protein
1.034	1.023	0.990	0.986	0.897	1.016	1.074	0.904	0.993	1.067	0.993	1.149	Q96K21	ZFYVE19	Abscission/NoCut checkpoint regulator
0.972	0.955	1.023	1.016	0.992	1.062	1.006	1.005	1.014	1.056	1.011	1.053	Q86V21	AACS	Acetoacetyl-CoA synthetase
1.022	1.018	1.062	1.022	0.968	0.988	1.017	1.062	1.029	1.003	1.016	1.005	A1L0T0	ILVBL	Acetolactate synthase-like protein
0.980	0.987	1.001	1.007	0.994	0.972	1.026	1.059	1.049	1.067	1.146	1.039	Q9BWD1	ACAT2	Acetyl-CoA acetyltransferase, cytosolic
1.003	0.986	0.962	1.005	0.970	0.996	1.015	0.959	0.975	1.039	0.956	0.978	P24752	ACAT1	Acetyl-CoA acetyltransferase, mitochondrial
0.762	1.131	1.193	0.986	0.816	1.240							O00763	ACACB	Acetyl-CoA carboxylase 2
1.026	1.001	1.032	1.023	0.923	0.964	1.008	1.044	0.995	1.081	0.814	1.023	Q9NUB1	ACSS1	Acetyl-coenzyme A synthetase 2-like, mitochondrial
1.284	0.845	0.977	1.268	0.742	1.045	1.251	0.925	0.957	1.186	0.976	1.063	O00400	SLC33A1	Acetyl-coenzyme A transporter 1
1.056	0.954	1.177	0.962	0.990	1.020	1.222	1.060	0.998				Q92484	SMPDL3A	Acid sphingomyelinase-like phosphodiesterase 3a
1.074	0.974	1.073	1.054	0.990	1.008	1.035	0.907	1.029	1.138	1.026	1.048	Q92485	SMPDL3B	Acid sphingomyelinase-like phosphodiesterase 3b
1.048	0.975	1.031	0.981	0.962	1.050	1.032	1.075	1.041	0.987	0.880	1.077	O43427	FIBP	Acidic fibroblast growth factor intracellular-binding protein
1.082	0.991	0.999	1.010	1.121	0.976	1.007	0.953	1.016	0.988	0.989	0.958	P39687	ANP32A	Acidic leucine-rich nuclear phosphoprotein 32 family member A
1.026	0.995	1.007	0.987	1.100	0.986	1.032	0.930	1.023	0.946	0.986	0.896	Q92688	ANP32B	Acidic leucine-rich nuclear phosphoprotein 32 family member B
1.057	0.980	1.030	1.010	1.075	1.027	1.039	1.058	1.051	0.975	0.996	1.006	Q9BTT0	ANP32E	Acidic leucine-rich nuclear phosphoprotein 32 family member E
1.044	1.046	1.032	1.068	1.073	1.048	1.078	1.111	1.079	1.071	1.097	1.057	A2A274	ACO2	Aconitate hydratase, mitochondrial
			1.034	1.045	1.002	0.962	1.116	1.040	0.988	0.993	0.985	Q8TED9	AFAP1L1	Actin filament-associated protein 1-like 1

Abundance Ratio												Accession ID	Gene name	Protein Description
1 d M1/C1	1 d M2/C2	1 d M3/C3	4 d M1/C1	4 d M2/C2	4 d M3/C3	7 d M1/C1	7 d M2/C2	7 d M3/C3	10 d M1/C1	10 d M2/C2	10 d M3/C3			
0.947	0.954	1.083	1.371	1.249	0.904	1.017	1.191	1.081				P68032	ACTC1	Actin, alpha cardiac muscle 1
0.888	1.020	0.944	1.035	0.985	0.960	1.030	1.034	0.990	0.995	1.034	0.969	P68133	ACTA1	Actin, alpha skeletal muscle
0.996	0.989	0.978	1.068	1.019	0.960	1.098	0.950	0.992	1.031	0.977	0.976	P60709	ACTB	Actin, cytoplasmic 1
1.135	1.013	0.967	0.959	0.958	1.035	0.934	1.044	0.951	0.940	0.922	1.001	K7EM38	ACTG1	Actin, cytoplasmic 2 (Fragment)
1.180	1.030	1.117	0.713	0.945	0.743							O14639	ABLIM1	Actin-binding LIM protein 1
0.971	0.983	1.000	0.984	1.030	1.005	0.988	0.906	1.021	1.004	0.985	1.044	O96019	ACTL6A	Actin-like protein 6A
1.047	0.961	0.988	1.021	0.982	1.057	1.025	1.018	1.017	0.994	1.034	1.011	Q9NZ32	ACTR10	Actin-related protein 10
0.966	0.994	0.974	1.006	1.038	1.008	1.009	1.038	1.016	0.996	0.996	0.989	P61160	ACTR2	Actin-related protein 2
1.025	1.005	0.951	1.028	1.049	1.005	1.036	0.970	1.021	1.028	1.011	0.980	Q92747	ARPC1A	Actin-related protein 2/3 complex subunit 1A
1.048	1.017	0.999	1.014	1.076	0.973	0.987	0.985	0.999	0.992	0.994	0.965	O15143	ARPC1B	Actin-related protein 2/3 complex subunit 1B
1.006	1.024	1.012	1.011	1.021	1.000	0.999	0.998	0.981	1.040	1.006	1.022	O15144	ARPC2	Actin-related protein 2/3 complex subunit 2
1.096	1.031	1.016	1.042	1.104	0.987	0.997	1.045	1.022	0.952	1.021	0.901	O15145	ARPC3	Actin-related protein 2/3 complex subunit 3
0.950	0.983	0.976	0.960	0.973	1.006	0.945	0.921	0.952	0.983	0.959	0.982	O15511	ARPC5	Actin-related protein 2/3 complex subunit 5
1.035	0.945	1.029	0.972	0.966	1.016	0.970	0.944	0.994	0.957	0.963	1.028	Q9BPX5	ARPC5L	Actin-related protein 2/3 complex subunit 5-like protein
1.029	1.007	1.017	1.000	1.107	1.007	0.997	0.932	1.041	0.988	1.000	0.992	P61158	ACTR3	Actin-related protein 3
									0.877	1.054	1.061	Q9P1U1	ACTR3B	Actin-related protein 3B
0.995	1.014	1.016	1.009	0.965	0.993	1.034	1.076	0.993	0.975	0.984	1.100	Q9H9F9	ACTR5	Actin-related protein 5
0.996	1.030	1.035	0.953	0.963	1.025	1.010	1.028	1.022	0.921	0.975	1.094	Q9GZN1	ACTR6	Actin-related protein 6
1.071	1.086	1.084	1.012	1.054	1.066				0.928	1.012	1.141	Q9H981	ACTR8	Actin-related protein 8
1.055	1.000	1.015	1.004	0.948	0.990	0.953	0.990	0.964	0.900	1.014	0.862	P53999	SUB1	Activated RNA polymerase II transcriptional coactivator p15
1.074	1.013	1.052	1.009	1.040	1.011	0.997	1.112	1.025	0.984	0.958	1.003	Q9H1I8	ASCC2	Activating signal cointegrator 1 complex subunit 2
1.019	1.025	1.017	0.990	0.961	0.982	1.007	1.037	1.005	1.025	0.975	1.041	Q8N3C0	ASCC3	Activating signal cointegrator 1 complex subunit 3
1.017	1.015	1.036	1.024	1.005	1.022	1.018	1.060	1.018	0.988	1.009	1.040	Q15650	TRIP4	Activating signal cointegrator 1
0.993	1.015	1.000	0.995	0.983	0.998	0.978	1.021	0.989	0.956	0.986	0.989	O95433	AHSA1	Activator of 90 kDa heat shock protein ATPase homolog 1
1.070	1.017	0.958	1.047	1.045	1.012	1.019	1.094	0.951	0.951	1.000	1.031	Q9ULW3	ABT1	Activator of basal transcription 1
0.910	0.952	0.956	0.997	0.933	1.003	0.985	1.033	0.997	1.047	1.032	1.032	Q86WX3	RPS19BP1	Active regulator of SIRT1
1.004	0.964	1.084	1.079	0.920	1.170				1.209	0.891	0.951	Q04771	ACVR1	Activin receptor type-1
1.019	0.995	0.997	1.006	0.995	0.987	1.027	0.962	1.016	1.031	1.015	1.021	Q9H2P0	ADNP	Activity-dependent neuroprotector homeobox protein
0.999	0.994	0.986	1.037	1.147	1.042	1.003	0.889	1.085	1.044	1.023	1.021	O14561	NDUFAB1	Acyl carrier protein, mitochondrial
0.955	0.977	0.998	0.979	1.035	0.999	0.966	0.933	1.002	0.998	1.015	1.042	C9JIF9	APEH	Acylamino-acid-releasing enzyme

Abundance Ratio														
1 d M1/C1	1 d M2/C2	1 d M3/C3	4 d M1/C1	4 d M2/C2	4 d M3/C3	7 d M1/C1	7 d M2/C2	7 d M3/C3	10 d M1/C1	10 d M2/C2	10 d M3/C3	Accession ID	Gene name	Protein Description
1.006	1.029	1.018	0.953	0.946	1.017	0.975	1.046	1.013	1.013	1.014	1.055	Q6JQN1	ACAD10	Acyl-CoA dehydrogenase family member 10
1.052	1.048	0.985	1.002	1.056	0.957	0.999	0.984	0.949	1.051	0.982	0.994	Q709F0	ACAD11	Acyl-CoA dehydrogenase family member 11
0.981	0.992	1.029	0.988	0.979	1.014	1.026	1.033	0.985	1.034	0.983	1.013	Q9H845	ACAD9	Acyl-CoA dehydrogenase family member 9, mitochondrial
0.890	0.903	1.010	0.934	0.830	0.894	1.055	1.104	1.021	1.120	1.163	1.161	O00767	SCD	Acyl-CoA desaturase
1.006	1.027	1.013	1.006	0.990	1.025	1.024	1.002	1.002	1.048	0.979	1.038	Q4G176	ACSF3	Acyl-CoA synthetase family member 3, mitochondrial
1.042	1.029	1.082	0.971	0.955	1.005	0.997	1.072	0.993	1.031	1.003	1.008	Q92604	LPGAT1	Acyl-CoA:lysophosphatidylglycerol acyltransferase 1
1.058	0.962	1.014	0.954	0.905	0.958	1.001	0.968	1.000	1.044	0.949	1.017	Q9BR61	ACBD6	Acyl-CoA-binding domain-containing protein 6
0.998	0.989	1.011	0.984	0.967	0.967	0.986	0.962	0.985	1.007	0.974	0.979	Q5T4U5	ACADM	Acyl-Coenzyme A dehydrogenase, C-4 to C-12 straight chain, isoform CRA_a
1.001	0.994	0.998	1.009	1.020	1.030	1.044	1.002	1.027	1.056	0.991	1.031	Q86TX2	ACOT1	Acyl-coenzyme A thioesterase 1
0.993	1.000	0.947	0.973	0.939	0.984	0.975	1.067	0.976	0.975	0.958	0.960	Q9NPJ3	ACOT13	Acyl-coenzyme A thioesterase 13
0.925	1.014	0.987	0.976	0.961	0.975	0.961	1.091	0.993	0.882	1.045	0.813	P49753	ACOT2	Acyl-coenzyme A thioesterase 2, mitochondrial
									0.854	0.941	1.068	Q8N9L9	ACOT4	Acyl-coenzyme A thioesterase 4
1.006	1.017	1.015	1.003	1.028	1.014	1.003	1.017	0.961	0.989	1.011	1.019	O14734	ACOT8	Acyl-coenzyme A thioesterase 8
1.026	0.996	0.991	1.014	0.968	0.984	1.021	1.019	0.979	0.997	0.979	0.972	Q9Y305	ACOT9	Acyl-coenzyme A thioesterase 9, mitochondrial
1.008	1.017	1.033	0.968	0.970	1.025	1.018	1.147	1.110	1.007	0.983	1.134	Q5T1C6	THEM4	Acyl-coenzyme A thioesterase THEM4
1.014	0.999	1.018	1.034	1.065	1.013	1.033	0.996	1.035	1.042	1.011	1.059	Q53H12	AGK	Acylglycerol kinase, mitochondrial
1.017	0.988	0.949	0.958	0.937	0.963	0.927	1.001	0.951	0.956	1.032	0.959	G3V2U7	ACYP1	Acylphosphatase
0.935	0.966	0.994	1.012	1.009	1.079	0.950	0.936	0.984	1.152	0.988	1.123	P14621	ACYP2	Acylphosphatase-2
1.020	0.996	1.011	0.988	0.994	0.985	0.989	1.005	0.978	1.005	1.025	0.999	O75608	LYPLA1	Acyl-protein thioesterase 1
0.985	1.016	1.002	0.991	1.018	1.049	0.982	1.006	1.013	0.993	1.003	1.029	O95372	LYPLA2	Acyl-protein thioesterase 2
0.982	1.000	1.019	1.010	1.266	0.982	0.999	0.867	1.068	1.040	1.005	1.018	Q6P587	FAHD1	Acylpyruvase FAHD1, mitochondrial
1.010	1.006	0.979	1.010	0.970	1.001	0.996	1.066	1.025	1.031	0.998	1.035	P46108	CRK	Adapter molecule crk
1.092	1.005	1.050	1.021	1.177	0.978	1.023	0.872	1.094	1.001	0.995	1.060	Q8NC96	NECAP1	Adaptin ear-binding coat-associated protein 1
0.976	0.996	1.025	0.982	1.050	1.036	0.955	0.895	1.016	0.992	0.975	1.057	Q9NVZ3	NECAP2	Adaptin ear-binding coat-associated protein 2
1.032	0.978	1.014	1.015	1.048	1.041	0.981	1.015	1.068	1.002	1.033	0.989	P07741	APRT	Adenine phosphoribosyltransferase
1.086	1.197	1.059	1.055	0.937	1.054							P25054	APC	Adenomatous polyposis coli protein
0.962	0.999	0.983	1.004	0.883	0.989	1.067	1.088	1.049	1.068	1.067	1.013	Q8TB61	SLC35B2	Adenosine 3'-phospho 5'-phosphosulfate transporter 1
0.830	1.216	1.014				0.904	1.092	1.076	1.007	1.039	0.950	Q9H1N7	SLC35B3	Adenosine 3'-phospho 5'-phosphosulfate transporter 2
0.993	0.999	1.001	0.999	0.984	1.014	0.985	1.020	0.970	1.007	0.953	0.996	P00813	ADA	Adenosine deaminase
0.889	0.839	0.916							1.355	0.891	1.100	Q6DHV7	ADAL	Adenosine deaminase-like protein

Abundance Ratio														
1 d M1/C1	1 d M2/C2	1 d M3/C3	4 d M1/C1	4 d M2/C2	4 d M3/C3	7 d M1/C1	7 d M2/C2	7 d M3/C3	10 d M1/C1	10 d M2/C2	10 d M3/C3	Accession ID	Gene name	Protein Description
1.056	1.026	1.035	1.037	1.096	0.995	0.980	0.954	1.006	0.943	0.955	0.919	P55263	ADK	Adenosine kinase
0.979	0.999	0.974	0.999	0.990	0.987	0.969	1.013	0.988	0.957	1.000	0.935	P23526	AHCY	Adenosylhomocysteinase
0.793	0.957	0.987										A0A0A0MSC1	ADCY3	Adenylate cyclase type 3
0.942	1.019	0.971	1.070	0.987	1.063	1.017	1.112	1.057	1.033	1.041	1.169	O43306	ADCY6	Adenylate cyclase type 6
0.723	0.953	0.962										P51828	ADCY7	Adenylate cyclase type 7
1.283	1.143	1.056	1.089	0.936	1.027							O60503	ADCY9	Adenylate cyclase type 9
0.971	1.003	0.980	1.003	1.033	1.006	1.013	0.981	1.004	0.989	1.016	0.977	P54819	AK2	Adenylate kinase 2, mitochondrial
1.027	0.992	0.994	0.967	0.955	0.964	0.949	1.017	0.947	0.956	0.980	0.921	P27144	AK4	Adenylate kinase 4, mitochondrial
0.999	0.978	0.982	0.983	1.028	1.000	1.021	0.949	0.982	1.036	0.973	1.061	Q5T9B7	AK1	Adenylate kinase isoenzyme 1
0.938	0.974	0.998	0.972	0.947	1.011	0.950	1.150	1.045	0.923	0.978	0.967	Q9Y3D8	AK6	Adenylate kinase isoenzyme 6
0.962	1.009	1.009	0.987	0.999	0.978	0.981	0.973	0.975	0.955	0.987	0.993	P30566	ADSL	Adenylosuccinate lyase
0.998	1.018	0.987	1.043	1.015	1.019	1.044	1.018	1.018	1.028	1.005	1.024	P30520	ADSS	Adenylosuccinate synthetase isozyme 2
0.982	1.149	0.861	1.116	1.175	0.988	1.071	1.290	1.168	1.063	1.047	0.986	Q01518	CAP1	Adenylyl cyclase-associated protein 1
1.015	1.002	0.983	0.989	1.007	0.975	0.961	0.911	0.992	1.012	0.985	1.025	P40123	CAP2	Adenylyl cyclase-associated protein 2
0.988	1.042	1.047	1.058	1.074	1.020	1.146	1.046	1.077	1.151	1.082	1.084	O95396	MOCS3	Adenylyltransferase and sulfurtransferase MOCS3
0.956	0.965	1.090	0.995	0.930	1.134	1.040	1.088	1.102	1.053	0.997	1.090	Q9UHX3	ADGRE2	Adhesion G protein-coupled receptor E2
1.057	1.091	1.017	1.065	1.019	0.946	1.036	1.010	0.929				Q9Y653	ADGRG1	Adhesion G-protein coupled receptor G1
0.963	0.981	0.983	0.982	0.964	1.005	0.979	1.009	0.976	0.988	0.962	0.981	Q9HDC9	APMAP	Adipocyte plasma membrane-associated protein
0.934	1.013	1.064	1.050	1.140	1.021				1.040	0.988	1.217	Q6IQ32	ADNP2	ADNP homeobox protein 2
1.055	1.019	1.104	1.000	1.017	0.977	1.023	1.052	1.015	0.942	0.986	0.947	P12235	SLC25A4	ADP/ATP translocase 1
0.970	1.002	1.003	0.992	1.013	1.005	0.975	1.023	1.012	0.933	0.987	0.926	P05141	SLC25A5	ADP/ATP translocase 2
1.005	1.000	1.046	0.978	0.961	1.006	1.006	1.059	1.009	1.010	1.043	1.016	P12236	SLC25A6	ADP/ATP translocase 3
0.947	1.013	1.055	0.976	0.982	1.012	1.025	1.114	1.056	0.956	1.021	1.035	Q9BRR6	ADPGK	ADP-dependent glucokinase
1.017	0.995	0.973	1.010	0.974	1.021	0.968	1.010	0.973	1.063	1.001	1.049	Q9BW91	NUDT9	ADP-ribose pyrophosphatase, mitochondrial
			0.659	0.733	1.280	0.901	1.091	1.297	1.139	1.022	1.105	P84077	ARF1	ADP-ribosylation factor 1
0.927	0.992	0.929	1.026	0.773	1.129	1.053	1.189	0.969	1.121	1.129	1.054	P61204	ARF3	ADP-ribosylation factor 3
0.948	0.951	0.950	0.991	0.713	1.123	1.116	1.123	0.932	1.055	1.057	1.101	P18085	ARF4	ADP-ribosylation factor 4
0.948	0.975	0.987	1.007	0.809	1.124	1.018	1.104	0.976	1.058	1.068	1.059	P84085	ARF5	ADP-ribosylation factor 5
1.028	1.038	1.029	1.003	0.854	1.079	1.048	1.128	1.016	1.183	1.065	1.055	P62330	ARF6	ADP-ribosylation factor 6
0.987	0.998	1.001	0.951	0.940	0.978	0.941	0.926	1.037	1.018	0.993	1.055	Q8N6T3	ARFGAP1	ADP-ribosylation factor GTPase-activating protein 1
1.023	0.995	1.048	1.037	1.009	1.035	1.039	1.014	1.015	1.064	1.037	1.089	Q8N6H7	ARFGAP2	ADP-ribosylation factor GTPase-activating protein 2

Abundance Ratio												Accession ID	Gene name	Protein Description
1 d M1/C1	1 d M2/C2	1 d M3/C3	4 d M1/C1	4 d M2/C2	4 d M3/C3	7 d M1/C1	7 d M2/C2	7 d M3/C3	10 d M1/C1	10 d M2/C2	10 d M3/C3			
1.037	0.996	1.018	0.967	1.011	0.983	1.001	0.914	0.983	1.046	0.995	1.026	Q9NP61	ARFGAP3	ADP-ribosylation factor GTPase-activating protein 3
1.070	1.026	1.052	0.986	0.968	1.073	1.027	1.085	1.067	1.023	1.014	1.043	Q9UJY5	GGA1	ADP-ribosylation factor-binding protein GGA1
1.055	1.033	0.999	1.072	1.023	1.044	1.066	1.027	1.009	1.087	1.037	1.067	Q9UJY4	GGA2	ADP-ribosylation factor-binding protein GGA2
1.097	0.977	1.009	1.007	0.973	0.962	0.982	1.013	0.999	1.076	1.025	1.098	Q9NZ52	GGA3	ADP-ribosylation factor-binding protein GGA3
0.944	1.025	0.933	0.950	0.818	1.068	0.988	1.075	1.037	1.056	1.052	1.077	P40616	ARL1	ADP-ribosylation factor-like protein 1
1.060	0.926	0.933										Q3SXY8	ARL13B	ADP-ribosylation factor-like protein 13B
0.941	0.968	1.053	1.019	0.963	0.994	0.970	0.983	0.974	0.957	0.899	1.007	A0A087WUW9	ARL15	ADP-ribosylation factor-like protein 15
1.002	1.004	0.987	0.986	0.965	1.007	0.990	1.085	1.010	1.005	0.994	1.014	P36404	ARL2	ADP-ribosylation factor-like protein 2
0.982	1.030	1.008	0.989	1.080	0.984	1.013	1.061	1.009	0.981	1.020	0.955	Q9Y2Y0	ARL2BP	ADP-ribosylation factor-like protein 2-binding protein
1.038	0.997	0.986	0.993	1.022	0.997	1.012	1.053	1.034	0.973	1.029	1.002	P36405	ARL3	ADP-ribosylation factor-like protein 3
						0.754	1.154	1.273				Q9Y689	ARL5A	ADP-ribosylation factor-like protein 5A
1.009	0.996	0.999	1.002	0.911	0.972	0.993	1.136	1.006	0.923	1.056	0.933	Q96KC2	ARL5B	ADP-ribosylation factor-like protein 5B
1.051	1.038	1.100	0.918	0.920	1.042	0.796	1.084	0.983	0.877	0.924	1.033	Q9H0F7	ARL6	ADP-ribosylation factor-like protein 6
			0.943	0.878	1.038	1.078	1.153	0.854	0.996	1.178	1.087	Q8N6S5	ARL6IP6	ADP-ribosylation factor-like protein 6-interacting protein 6
1.033	0.949	1.040	0.999	0.865	1.119	1.040	1.087	1.006	1.028	1.024	0.971	Q96BM9	ARL8A	ADP-ribosylation factor-like protein 8A
1.012	1.000	0.988	1.027	0.877	1.074	1.055	1.040	1.013	0.970	1.051	0.974	Q9NVJ2	ARL8B	ADP-ribosylation factor-like protein 8B
1.013	0.980	1.057	0.986	0.901	1.025	1.027	0.984	0.954	1.106	1.004	1.037	Q13795	ARFRP1	ADP-ribosylation factor-related protein 1
1.037	1.017	0.982	1.009	1.035	0.991	1.035	1.065	1.008	1.027	0.978	1.015	A6NFX8	NUDT5	ADP-sugar pyrophosphatase
1.044	0.975	1.023				1.084	1.176	1.132	1.138	0.981	1.067	Q96AP0	ACD	Adrenocortical dysplasia protein homolog
1.039	1.025	1.075	1.131	1.170	1.081	1.094	1.016	1.031	1.079	1.025	1.104	P10109	FDX1	Adrenodoxin, mitochondrial
0.986	1.081	0.996										Q9Y6U3	SCIN	Adseverin
0.984	0.979	1.026	0.932	0.905	0.938	0.955	0.945	0.999	0.974	0.942	1.083	Q9UHB7	AFF4	AF4/FMR2 family member 4
						1.093	1.066	1.041				Q9Y2D8	SSX2IP	Afadin- and alpha-actinin-binding protein
0.958	0.976	1.001	0.981	0.868	1.010	1.011	1.031	0.995	1.049	1.016	0.996	Q9Y4W6	AFG3L2	AFG3-like protein 2
1.064	1.020	0.987	1.000	1.009	0.989	0.991	1.038	0.977	1.014	0.986	1.006	O43488	AKR7A2	Aflatoxin B1 aldehyde reductase member 2
0.989	0.918	1.006	1.001	0.929	1.016	0.945	1.031	0.993	1.042	1.016	1.079	Q6ULP2	AFTPH	Aftiphilin
1.003	1.018	1.003	0.975	0.966	1.007	0.981	0.976	0.981	0.973	0.985	1.014	O00170	AIP	AH receptor-interacting protein
1.043	1.010	1.057	1.023	0.982	1.021	1.045	1.069	1.005	1.041	1.008	1.072	Q92667	AKAP1	A-kinase anchor protein 1, mitochondrial
0.995	0.938	1.082	0.967	0.980	1.030	1.019	0.955	1.035	1.017	0.996	1.114	O43572	AKAP10	A-kinase anchor protein 10, mitochondrial
0.928	0.978	0.980	0.941	0.977	0.936	0.997	0.896	0.981	0.992	0.988	1.044	Q02952	AKAP12	A-kinase anchor protein 12
0.987	1.006	1.051	0.962	0.934	1.071	0.980	1.020	1.016	1.032	0.983	1.130	Q02040	AKAP17A	A-kinase anchor protein 17A
0.986	0.957	0.991	0.958	0.882	1.019	0.953	0.951	1.027	1.043	0.983	1.080	O43823	AKAP8	A-kinase anchor protein 8

Abundance Ratio														
1 d M1/C1	1 d M2/C2	1 d M3/C3	4 d M1/C1	4 d M2/C2	4 d M3/C3	7 d M1/C1	7 d M2/C2	7 d M3/C3	10 d M1/C1	10 d M2/C2	10 d M3/C3	Accession ID	Gene name	Protein Description
0.959	1.005	1.013	1.016	1.044	1.017	1.023	0.860	0.985	1.012	1.029	1.030	Q9ULX6	AKAP8L	A-kinase anchor protein 8-like
1.024	1.028	1.005	0.966	0.978	1.014	1.007	1.101	1.028	0.968	1.010	0.985	Q9NRG9	AAAS	Aladin
0.970	0.885	1.071	0.934	0.982	0.951	1.082	1.000	1.015	0.966	0.985	1.133	Q8TD30	GPT2	Alanine aminotransferase 2
0.985	1.011	0.992	1.012	1.036	1.009	0.994	1.030	1.009	0.962	1.006	0.987	P49588	AARS	Alanine--tRNA ligase, cytoplasmic
0.976	0.986	1.042	0.995	0.978	1.039	1.019	1.007	1.015	1.056	0.987	1.090	Q5JTZ9	AARS2	Alanine--tRNA ligase, mitochondrial
0.946	1.002	0.990	0.971	0.994	0.965	0.976	0.989	0.982	0.979	0.970	0.996	P14550	AKR1A1	Alcohol dehydrogenase [NADP(+)]
0.989	0.993	0.998	0.977	1.054	0.998	1.028	0.967	1.022	0.962	0.961	0.956	P11766	ADH5	Alcohol dehydrogenase class-3
0.981	1.026	1.028	1.002	1.033	0.977	1.052	1.034	1.011	0.936	0.893	1.033	P47895	ALDH1A3	Aldehyde dehydrogenase family 1 member A3
0.996	0.994	1.057	1.057	1.018	1.048	1.062	0.997	0.993	1.063	1.003	1.090	Q8IZ83	ALDH16A1	Aldehyde dehydrogenase family 16 member A1
0.999	1.005	1.003	0.976	0.983	0.984	0.958	1.001	0.996	0.974	0.955	0.986	P30837	ALDH1B1	Aldehyde dehydrogenase X, mitochondrial
1.006	0.918	1.056	0.986	1.010	0.991	1.072	1.105	0.964				P05091	ALDH2	Aldehyde dehydrogenase, mitochondrial
1.072	1.102	1.129	1.072	1.013	1.091	1.067	1.043	1.022	0.966	0.945	1.168	Q06278	AOX1	Aldehyde oxidase
						1.114	1.153	0.861	0.896	1.019	1.154	A0A0A0MSS8	AKR1C3	Aldo-keto reductase family 1 member C3
0.972	0.989	0.993	1.014	0.935	1.012	0.976	0.992	0.959	1.059	0.928	0.991	Q96C23	GALM	Aldose 1-epimerase
0.968	1.006	1.000	0.977	1.094	0.886	0.951	0.856	0.948	0.979	0.785	0.999	P15121	AKR1B1	Aldose reductase
0.991	1.002	1.020	1.012	1.032	0.991	1.059	1.000	0.995	1.032	1.017	1.017	O00116	AGPS	Alkyldihydroxyacetonephosphate synthase, peroxisomal
0.948	1.015	1.049	1.012	0.936	0.950	1.025	1.011	0.966	1.122	1.014	1.139	Q6NUM9	RETSAT	All-trans-retinol 13,14-reductase
1.007	0.974	1.009	1.005	0.966	1.019	1.007	1.065	1.032	0.987	1.024	1.026	Q6PD74	AAGAB	Alpha- and gamma-adaptin-binding protein p34
1.183	0.821	1.066	0.929	0.936	0.974	0.937	1.066	1.117	0.984	0.954	0.948	Q495W5	FUT11	Alpha-(1,3)-fucosyltransferase 11
0.991	1.078	1.044	0.952	0.946	0.923	0.961	1.039	0.930	0.972	0.973	1.028	Q9BYC5	FUT8	Alpha-(1,6)-fucosyltransferase
0.982	0.959	1.092	1.012	0.957	1.002	1.021	1.026	1.020	1.059	0.996	1.026	Q9H553	ALG2	Alpha-1,3/1,6-mannosyltransferase ALG2
0.909	0.918	0.900	0.932	0.858	1.063	1.069	1.108	0.991	1.375	1.054	1.216	A2A2G4	ALG6	Alpha-1,3-glucosyltransferase
0.968	1.015	0.998	0.925	0.866	0.937	0.965	1.049	0.964	0.999	0.970	1.037	P26572	MGAT1	Alpha-1,3-mannosyl-glycoprotein 2-beta-N-acetylglucosaminyltransferase
1.007	0.960	0.994	0.961	1.001	0.943	0.888	1.103	0.910	0.968	0.971	0.922	Q10469	MGAT2	Alpha-1,6-mannosyl-glycoprotein 2-beta-N-acetylglucosaminyltransferase
0.856	1.077	1.133							0.874	1.148	1.097	Q09328	MGAT5	Alpha-1,6-mannosylglycoprotein 6-beta-N-acetylglucosaminyltransferase A
									1.127	1.017	0.985	A0A024R6I7	SERPINA1	Alpha-1-antitrypsin
1.035	1.002	1.046	0.989	0.959	1.064	0.993	1.071	1.074	1.049	1.062	1.061	Q13424	SNTA1	Alpha-1-syntrophin
1.018	0.985	1.010	0.964	0.957	1.014	0.955	1.006	0.971	0.973	0.994	0.936	P30533	LRPAP1	Alpha-2-macroglobulin receptor-associated protein
0.995	1.013	0.999	1.004	1.069	0.988	1.012	1.035	1.027	0.974	0.979	1.003	P12814	ACTN1	Alpha-actinin-1
0.886	0.934	1.003	1.039	0.909	1.068	1.127	1.058	1.054	1.008	0.974	0.899	K7EJH8	ACTN4	Alpha-actinin-4 (Fragment)
0.960	1.009	0.984	0.995	0.981	0.961	0.989	1.028	0.983	0.980	1.000	0.996	O43707	ACTN4	Alpha-actinin-4

Abundance Ratio														
1 d M1/C1	1 d M2/C2	1 d M3/C3	4 d M1/C1	4 d M2/C2	4 d M3/C3	7 d M1/C1	7 d M2/C2	7 d M3/C3	10 d M1/C1	10 d M2/C2	10 d M3/C3	Accession ID	Gene name	Protein Description
1.000	1.017	1.003	1.006	0.985	1.001	1.010	1.040	1.006	1.017	0.986	1.002	Q9UDR5	AASS	Alpha-aminoadipic semialdehyde synthase, mitochondrial
1.067	1.013	0.962	1.016	0.929	0.996	1.019	1.037	1.008	1.020	0.935	1.045	Q9UBT7	CTNNAL1	Alpha-catulin
1.056	1.027	1.012	1.045	1.047	0.969	0.997	1.014	1.019	0.988	0.996	0.974	P61163	ACTR1A	Alpha-centractin
			0.995	0.879	0.962	1.051	1.131	0.938	0.826	0.873	0.942	P02511	CRYAB	Alpha-crystallin B chain
0.978	1.009	0.972	0.984	0.971	0.982	1.034	0.985	0.976	1.029	1.020	1.005	Q5T5H1	ENSA	Alpha-endosulfine
1.039	0.983	0.987	1.027	0.982	0.979	0.975	0.990	1.012	0.966	0.973	0.942	P06733	ENO1	Alpha-enolase
0.999	0.995	0.984	1.041	0.997	1.023	1.070	1.080	1.033	1.133	1.091	1.055	P06280	GLA	Alpha-galactosidase A
1.043	1.002	1.021	1.029	0.955	1.010	1.011	1.034	1.044	0.980	0.982	1.007	Q12800	TFCP2	Alpha-globin transcription factor CP2
						1.190	1.464	0.892				Q96Q83	ALKBH3	Alpha-ketoglutarate-dependent dioxygenase alkB homolog 3
1.274	1.016	1.097	1.027	0.933	0.989	0.973	1.143	1.066	0.876	1.055	1.133	Q9NXW9	ALKBH4	Alpha-ketoglutarate-dependent dioxygenase alkB homolog 4
1.454	0.782	0.736	0.994	0.767	0.941				1.272	0.815	1.118	Q9BT30	ALKBH7	Alpha-ketoglutarate-dependent dioxygenase alkB homolog 7, mitochondrial
0.985	0.991	1.004	1.005	1.034	1.012	1.023	1.014	1.001	1.008	0.985	1.001	Q9C0B1	FTO	Alpha-ketoglutarate-dependent dioxygenase FTO
0.693	0.790	1.126	0.997	0.960	0.978	0.884	1.152	1.057	1.103	1.060	1.048	P35475	IDUA	Alpha-L-iduronidase
0.964	1.001	1.007	0.934	0.905	0.978	0.941	1.062	0.994	0.966	0.977	0.983	Q16706	MAN2A1	Alpha-mannosidase 2
						0.967	1.209	0.862	1.056	0.892	0.964	P49641	MAN2A2	Alpha-mannosidase 2x
1.015	1.035	1.005	0.988	0.959	0.957	0.952	1.048	0.954	1.011	1.018	0.958	P17050	NAGA	Alpha-N-acetylgalactosaminidase
1.005	0.893	1.003	0.975	0.994	1.031	0.941	1.036	1.032	1.033	0.970	1.005	P54802	NAGLU	Alpha-N-acetylglucosaminidase
1.008	0.989	1.018	0.988	0.944	1.004	1.008	1.040	0.993	0.966	0.985	0.972	J3KNQ4	PARVA	Alpha-parvin
						0.790	1.250	1.204	1.008	1.055	0.855	Q96QP1	ALPK1	Alpha-protein kinase 1
1.025	1.000	1.005	1.027	1.050	1.011	1.033	1.002	1.001	0.992	1.013	1.005	P54920	NAPA	Alpha-soluble NSF attachment protein
1.032	0.930	1.008	0.989	0.934	0.979	0.999	0.930	0.971	1.094	1.004	1.054	P37840	SNCA	Alpha-synuclein
0.976	1.000	1.014	1.010	0.962	1.023	1.015	0.971	1.014	1.061	1.017	1.082	P40222	TXLNA	Alpha-taxilin
1.025	0.985	0.994	0.980	0.959	0.985	0.981	1.129	1.087	0.941	0.985	1.085	Q9BTX7	TTPAL	Alpha-tocopherol transfer protein-like
1.123	1.052	1.014				1.015	1.024	1.128	0.882	1.231	1.195	Q60I27	ALS2CL	ALS2 C-terminal-like protein
1.091	0.936	1.066	0.994	0.896	1.006	0.929	1.017	0.960	0.934	1.021	1.020	Q96Q42	ALS2	Alsin
1.032	0.960	1.033	0.924	0.937	1.012	1.001	1.027	1.035	0.957	0.970	1.152	L0R819	ASNSD1	Alternative protein ASNSD1
0.945	0.956	1.015	0.974	1.051	1.011	0.969	0.975	0.993	1.017	1.049	1.009	Q9HB66	MKKS	Alternative protein MKKS
									1.002	1.359	0.987	L0R6P0	RPP14	Alternative protein RPP14
0.982	0.978	1.002	1.009	0.933	1.025	0.999	1.081	1.017	1.001	1.019	0.999	Q06203	PPAT	Amidophosphoribosyltransferase
1.013	0.981	0.981	1.004	0.953	1.007	1.013	1.015	1.010	1.028	0.994	1.017	P21397	MAOA	Amine oxidase [flavin-containing] A
						1.078	1.057	0.983				F8W950	AIMP2	Aminoacyl tRNA synthase complex-interacting multifunctional protein 2

Abundance Ratio														
1 d M1/C1	1 d M2/C2	1 d M3/C3	4 d M1/C1	4 d M2/C2	4 d M3/C3	7 d M1/C1	7 d M2/C2	7 d M3/C3	10 d M1/C1	10 d M2/C2	10 d M3/C3	Accession ID	Gene name	Protein Description
1.005	1.010	0.991	1.004	1.107	0.989	0.994	0.982	1.007	1.000	1.001	0.975	Q13155	AIMP2	Aminoacyl tRNA synthase complex-interacting multifunctional protein 2
1.009	0.995	1.015	1.018	1.017	1.023	1.008	1.024	1.008	0.986	0.994	0.979	Q03154	ACY1	Aminoacylase-1
1.019	1.003	0.993	1.006	1.018	0.985	0.988	1.028	0.987	1.012	0.978	0.988	Q9H4A4	RNPEP	Aminopeptidase B
1.082	0.960	0.846	1.116	0.987	0.927	1.034	1.036	1.005	1.051	1.422	0.829	Q9HAU8	RNPEPL1	Aminopeptidase RNPEPL1
0.904	0.973	1.041	1.061	0.938	0.954	1.088	1.118	1.017				K7ELE3	AES	Amino-terminal enhancer of split (Fragment)
1.183	1.077	0.968	1.157	0.591	1.230							Q6DCA0	AMMECR1L	AMMECR1-like protein
1.008	0.993	1.023	0.982	0.971	1.011	0.996	1.006	1.004	0.994	0.989	1.000	Q01433	AMPD2	AMP deaminase 2
0.834	0.979	1.165	0.872	0.917	0.960	0.964	0.752	1.058	0.875	1.049	1.275	O96018	APBA3	Amyloid beta A4 precursor protein-binding family A member 3
1.139	1.073	1.183	0.830	0.989	1.024	0.913	1.046	0.969	1.059	1.018	1.022	J3KPL8	APBB1	Amyloid beta A4 precursor protein-binding family B member 1
0.947	0.985	0.984	0.960	0.913	0.941	0.990	0.926	0.938	1.058	0.910	1.048	P05067	APP	Amyloid beta A4 protein
0.980	1.148	0.880										Q92624	APPBP2	Amyloid protein-binding protein 2
1.026	0.760	0.984	1.068	0.838	1.024	1.042	0.873	0.952	1.207	0.736	1.124	Q06481	APLP2	Amyloid-like protein 2
1.073	0.996	1.099	1.068	0.944	1.044	0.930	1.086	1.005	0.907	1.108	0.978	Q8TCF1	ZFAND1	AN1-type zinc finger protein 1
0.979	0.915	1.067	0.981	1.252	1.042	1.188	0.967	1.018	0.971	0.987	1.078	Q8WV99	ZFAND2B	AN1-type zinc finger protein 2B
1.055	1.023	1.027	1.031	0.900	1.017				0.962	1.022	1.101	O76080	ZFAND5	AN1-type zinc finger protein 5
1.103	0.975	0.941	0.627	1.160	0.919							Q6FIF0	ZFAND6	AN1-type zinc finger protein 6
1.000	1.006	1.036	0.967	0.957	0.987	0.973	0.920	0.954	0.982	1.001	0.998	Q6FI81	CIAPIN1	Anamorsin
1.003	1.033	1.028	1.003	0.942	1.039	0.983	1.024	0.967	0.973	0.993	1.032	Q9H1A4	ANAPC1	Anaphase-promoting complex subunit 1
1.048	1.018	0.983	0.988	1.071	1.040	0.977	0.842	1.012	1.215	0.991	1.115	Q9UM13	ANAPC10	Anaphase-promoting complex subunit 10
									1.055	1.027	1.000	Q9NYG5	ANAPC11	Anaphase-promoting complex subunit 11
1.108	0.928	0.886	0.962	1.221	1.008	1.066	0.978	1.062	1.065	0.958	1.166	Q9BS18	ANAPC13	Anaphase-promoting complex subunit 13
1.010	1.041	0.913	0.913	1.015	0.865	1.027	1.019	0.968	0.974	0.968	1.112	Q96DE5	ANAPC16	Anaphase-promoting complex subunit 16
1.026	0.990	1.049	1.001	0.903	1.019	1.081	1.056	1.133	1.105	1.020	1.013	Q9UJX6	ANAPC2	Anaphase-promoting complex subunit 2
1.034	0.985	1.014	1.016	0.938	1.090	0.983	1.123	1.037	1.017	1.030	1.060	F5H0F9	ANAPC5	Anaphase-promoting complex subunit 5
1.022	0.997	0.996	1.068	1.009	0.982	1.061	1.092	1.051	1.069	0.993	1.067	Q9UJX3	ANAPC7	Anaphase-promoting complex subunit 7
1.045	1.013	1.040	0.972	0.984	1.064	0.937	0.982	1.066	0.978	0.881	1.033	Q8NHZ8	CDC26	Anaphase-promoting complex subunit CDC26
0.988	1.022	1.017	0.994	0.935	1.001	1.008	1.004	0.989	1.010	1.028	1.041	Q9Y679	AUP1	Ancient ubiquitous protein 1
0.984	0.967	0.988	0.957	0.934	1.001	0.997	1.046	0.994	0.957	0.935	1.027	C9JEH3	AAMP	Angio-associated migratory cell protein
0.989	1.032	1.153	0.832	1.064	0.999	0.735	0.878	1.051	1.202	0.858	1.268	Q8IY63	AMOTL1	Angiomotin-like protein 1
0.997	1.004	0.976	0.938	0.964	0.988	0.934	1.024	0.952	0.923	0.932	0.884	Q9BY76	ANGPTL4	Angiopoietin-related protein 4
0.953	1.013	1.027	0.969	0.929	1.027	1.045	0.988	0.997	0.884	0.873	1.050	Q9NQW6	ANLN	Anillin
0.997	1.032	0.948	0.975	0.803	0.991	1.190	1.047	1.144	0.991	0.770	0.896	P04920	SLC4A2	Anion exchange protein 2

Abundance Ratio														
1 d M1/C1	1 d M2/C2	1 d M3/C3	4 d M1/C1	4 d M2/C2	4 d M3/C3	7 d M1/C1	7 d M2/C2	7 d M3/C3	10 d M1/C1	10 d M2/C2	10 d M3/C3	Accession ID	Gene name	Protein Description
1.019	0.974	1.040	1.016	0.932	1.135	0.965	0.909	1.055	1.052	1.017	1.120	Q969K4	ABTB1	Ankyrin repeat and BTB/POZ domain-containing protein 1
1.080	1.074	1.041	0.972	0.956	1.010	1.001	1.042	1.040	0.928	0.929	1.027	Q8N961	ABTB2	Ankyrin repeat and BTB/POZ domain-containing protein 2
1.033	1.058	1.130	1.046	1.062	1.111	1.228	1.079	1.118	1.167	1.034	1.159	Q495B1	ANKDD1A	Ankyrin repeat and death domain-containing protein 1A
1.094	0.868	1.031	1.147	0.898	0.951	1.099	0.972	1.016	1.093	0.923	1.021	Q9P2G1	ANKIB1	Ankyrin repeat and IBR domain-containing protein 1
0.898	0.886	0.999				1.085	1.015	1.080	0.942	1.027	1.131	E9PDP5	ANKHD1	Ankyrin repeat and KH domain-containing protein 1 (Fragment)
1.006	1.003	1.008	0.963	0.928	1.007	1.029	1.012	1.008	1.026	0.967	1.074	Q86XL3	ANKLE2	Ankyrin repeat and LEM domain-containing protein 2
1.032	1.022	0.998	1.025	1.021	1.016	1.058	1.061	1.158	1.005	0.978	1.065	Q8IV38	ANKMY2	Ankyrin repeat and MYND domain-containing protein 2
1.028	0.994	1.028	1.001	0.976	1.034	1.025	1.059	1.034	0.972	0.977	1.050	Q92625	ANKS1A	Ankyrin repeat and SAM domain-containing protein 1A
1.037	0.991	1.278	0.873	1.010	1.011	0.970	1.013	0.968	1.073	1.019	1.086	Q6ZW76	ANKS3	Ankyrin repeat and SAM domain-containing protein 3
						1.226	1.171	0.949	0.983	1.039	1.106	Q9Y576	ASB1	Ankyrin repeat and SOCS box protein 1
			1.189	0.981	1.284	0.937	1.194	1.172	1.073	0.717	0.956	Q9NWX5	ASB6	Ankyrin repeat and SOCS box protein 6
1.038	1.016	1.038	0.972	0.958	1.015	0.981	1.014	0.964	0.986	0.973	1.046	Q9H8Y5	ANKZF1	Ankyrin repeat and zinc finger domain-containing protein 1
									0.967	1.023	1.102	Q9NXR5	ANKRD10	Ankyrin repeat domain-containing protein 10
0.929	0.934	1.054	0.936	1.016	1.089	0.934	0.929	0.793				Q6UB99	ANKRD11	Ankyrin repeat domain-containing protein 11
0.985	1.038	1.011	0.983	1.021	0.970	0.961	1.148	1.014	1.029	0.999	1.008	Q8IZ07	ANKRD13A	Ankyrin repeat domain-containing protein 13A
1.114	0.996	0.964	1.091	0.884	0.972	1.089	1.175	0.937	0.941	0.943	0.998	Q6P6B7	ANKRD16	Ankyrin repeat domain-containing protein 16
1.021	0.994	1.069	0.995	0.957	1.026	0.992	1.040	1.026	1.015	0.993	1.030	O75179	ANKRD17	Ankyrin repeat domain-containing protein 17
			0.925	1.043	0.925	0.962	1.197	1.011	1.034	0.958	1.057	Q96NW4	ANKRD27	Ankyrin repeat domain-containing protein 27
			1.077	0.972	1.190	0.942	0.966	1.148				Q8N283	ANKRD35	Ankyrin repeat domain-containing protein 35
									1.047	0.967	0.913	Q6AI12	ANKRD40	Ankyrin repeat domain-containing protein 40
			0.998	0.945	0.869							Q8WVL7	ANKRD49	Ankyrin repeat domain-containing protein 49
1.075	1.189	0.999	0.988	0.958	0.960	0.967	1.133	1.022	1.051	0.955	0.964	Q9ULJ7	ANKRD50	Ankyrin repeat domain-containing protein 50
1.098	0.952	0.877	1.008	0.994	0.973	1.021	0.884	1.010	1.066	1.015	1.115	Q6NXT1	ANKRD54	Ankyrin repeat domain-containing protein 54
									1.166	1.084	1.211	B4E2M5	ANKRD66	Ankyrin repeat domain-containing protein 66
0.989	1.004	1.023	0.964	0.963	0.969	1.074	1.023	0.973	1.057	1.065	1.050	Q53LP3	SOWAHC	Ankyrin repeat domain-containing protein SOWAHC
1.027	0.985	1.038	1.010	0.971	1.039	1.012	0.978	1.052	1.075	1.006	1.036	Q01484	ANK2	Ankyrin-2
			1.131	1.279	1.133	0.958	1.107	0.931				H0YL33	ANXA2	Annexin (Fragment)

Abundance Ratio														
1 d M1/C1	1 d M2/C2	1 d M3/C3	4 d M1/C1	4 d M2/C2	4 d M3/C3	7 d M1/C1	7 d M2/C2	7 d M3/C3	10 d M1/C1	10 d M2/C2	10 d M3/C3	Accession ID	Gene name	Protein Description
1.035	1.021	1.013	1.002	1.066	1.031	0.998	1.075	1.045	0.963	1.020	0.997	P04083	ANXA1	Annexin A1
1.024	0.994	0.976	0.977	1.054	0.968	0.967	0.972	1.014	0.972	1.014	0.964	P50995	ANXA11	Annexin A11
1.017	1.011	0.993	0.998	0.996	0.965	1.019	0.995	0.999	1.002	0.940	1.004	P12429	ANXA3	Annexin A3
1.043	1.001	0.992	0.986	0.988	0.962	1.000	0.954	0.962	1.012	0.954	0.990	P09525	ANXA4	Annexin A4
1.016	0.999	1.012	1.010	0.992	1.002	0.967	0.969	0.998	0.963	0.995	0.969	P08758	ANXA5	Annexin A5
0.981	0.981	0.992	0.984	1.015	0.992	1.000	0.935	0.989	1.017	0.987	0.993	P08133	ANXA6	Annexin A6
0.970	1.007	1.000	1.009	1.051	0.998	1.024	0.965	0.999	0.999	0.998	0.999	P20073	ANXA7	Annexin A7
0.830	0.831	1.072	0.974	0.996	1.053	1.023	1.037	1.015	1.219	1.029	1.346	E5RK69	ANXA6	Annexin
0.952	0.953	1.075	0.980	0.970	0.977	1.006	1.029	1.022	1.082	0.977	1.074	Q9NW15	ANO10	Anoctamin-10
1.079	1.090	1.125	1.119	0.965	0.996	1.205	1.101	0.987	1.189	1.095	1.236	Q9H6X2	ANTXR1	Anthrax toxin receptor 1
1.231	1.171	1.213										P58335	ANTXR2	Anthrax toxin receptor 2
0.973	1.042	1.005	0.963	0.920	1.009	0.981	1.053	0.995	1.022	1.009	0.968	A0A140T9T7	TAP1	Antigen peptide transporter 1
0.961	1.037	1.046	0.962	0.921	0.944	0.956	0.989	0.964	0.988	1.023	1.004	X5CMH5	TAP2	Antigen peptide transporter 2
									1.066	1.042	1.225	P03973	SLPI	Antileukoproteinase
0.993	1.027	1.018	1.007	1.020	0.993	0.993	1.045	1.030	0.955	1.000	0.978	Q10567	AP1B1	AP-1 complex subunit beta-1
0.957	1.050	1.038	0.908	0.935	1.144	0.845	0.938	1.008	0.993	0.925	1.087	O75843	AP1G2	AP-1 complex subunit gamma-like 2
1.017	1.004	1.000	0.988	0.975	0.999	0.954	0.996	1.000	0.967	0.989	0.996	Q9BXS5	AP1M1	AP-1 complex subunit mu-1
1.006	0.979	0.979	0.995	0.892	1.019	1.023	1.044	1.028	0.878	0.947	0.935	P61966	AP1S1	AP-1 complex subunit sigma-1A
1.042	0.883	1.044	0.989	0.867	1.037	0.924	1.018	1.018	0.986	0.919	0.970	P56377	AP1S2	AP-1 complex subunit sigma-2
			0.920	0.833	1.132	1.043	1.077	0.941	0.531	1.047	0.995	Q63HQ0	AP1AR	AP-1 complex-associated regulatory protein
0.998	1.018	0.995	1.000	0.993	1.020	1.014	1.044	1.012	1.009	0.993	1.006	O95782	AP2A1	AP-2 complex subunit alpha-1
0.961	1.008	0.982	0.985	0.992	0.993	0.987	1.044	1.006	1.001	1.006	0.995	O94973	AP2A2	AP-2 complex subunit alpha-2
1.050	1.000	1.033	0.991	0.980	0.995	0.978	1.041	0.969	0.957	0.996	0.940	Q96CW1	AP2M1	AP-2 complex subunit mu
0.947	1.003	0.972	0.985	0.911	1.057	1.031	1.080	1.029	0.946	0.987	0.960	M0QYZ2	AP2S1	AP-2 complex subunit sigma
0.971	0.969	1.029	0.986	0.964	1.025	1.001	1.015	0.974	0.983	0.995	1.057	A0A096LP25	AAK1	AP2-associated protein kinase 1 (Fragment)
0.997	1.013	1.005	0.999	0.972	1.009	1.023	1.053	1.030	1.001	1.004	1.030	Q2M2I8	AAK1	AP2-associated protein kinase 1
1.012	1.009	1.004	1.009	0.969	1.003	1.012	1.027	0.997	1.024	1.001	1.015	O00203	AP3B1	AP-3 complex subunit beta-1
1.032	1.018	1.018	1.009	0.969	0.992	1.020	1.129	1.030	1.042	1.019	1.043	Q9Y2T2	AP3M1	AP-3 complex subunit mu-1
0.913	0.917	0.973	1.022	0.955	1.091	0.871	1.139	1.081				P53677	AP3M2	AP-3 complex subunit mu-2
1.021	0.989	1.004	1.020	0.982	1.029	1.059	1.091	1.057	0.999	1.002	0.988	Q92572	AP3S1	AP-3 complex subunit sigma-1
1.092	1.152	1.303	1.092	0.926	1.065	0.982	1.173	1.021	0.802	1.022	1.093	A0A1B0GV70	TEPSIN	AP-4 complex accessory subunit Tepsin
0.946	1.048	1.064	0.965	0.982	0.871	0.998	1.039	0.930	0.977	0.971	1.025	Q9Y6B7	AP4B1	AP-4 complex subunit beta-1
1.032	1.102	1.082	0.984	0.942	0.990	0.935	1.072	1.006	0.956	0.996	0.944	Q9UPM8	AP4E1	AP-4 complex subunit epsilon-1
1.362	1.384	1.051							1.605	0.936	0.816	C9JC87	AP4M1	AP-4 complex subunit mu-1

Abundance Ratio														
1 d M1/C1	1 d M2/C2	1 d M3/C3	4 d M1/C1	4 d M2/C2	4 d M3/C3	7 d M1/C1	7 d M2/C2	7 d M3/C3	10 d M1/C1	10 d M2/C2	10 d M3/C3	Accession ID	Gene name	Protein Description
						1.158	1.051	0.977				Q9Y587	AP4S1	AP-4 complex subunit sigma-1
0.880	1.028	1.016	0.934	0.934	0.999	1.073	0.993	1.056	0.868	0.831	1.059	Q2VPB7	AP5B1	AP-5 complex subunit beta-1
1.027	1.074	0.907	0.888	0.963	1.049	0.791	1.237	1.013	0.808	0.928	0.784	E7EQ45	AP5M1	AP-5 complex subunit mu-1
0.962	0.940	1.071	0.924	0.851	0.985	0.916	1.262	1.050	0.953	0.973	0.959	Q9NUS5	AP5S1	AP-5 complex subunit sigma-1
1.054	1.018	1.041	0.997	0.856	1.018	0.985	1.140	1.031	1.055	1.021	1.107	O43299	AP5Z1	AP-5 complex subunit zeta-1
			1.059	1.382	1.414	1.488	1.018	1.549				K7EPF9	APOC1	Apolipoprotein C-I
1.028	1.032	1.072	0.808	0.893	1.217							B0YIW2	APOC3	Apolipoprotein C-III
1.010	1.020	1.043	1.014	1.000	1.030	1.058	0.939	0.978	1.078	0.989	1.121	J3KQL8	APOL2	Apolipoprotein L2
1.016	1.002	0.994	0.991	0.951	1.020	0.976	1.049	1.012	0.944	1.001	0.942	Q9BZZ5	API5	Apoptosis inhibitor 5
			0.882	0.886	1.044				1.036	0.908	1.157	Q9ULZ3	PYCARD	Apoptosis-associated speck-like protein containing a CARD
0.972	0.989	0.995	0.972	0.982	0.964	0.986	0.976	0.981	1.013	0.996	1.006	O95831	AIFM1	Apoptosis-inducing factor 1, mitochondrial
1.079	1.036	1.017	1.080	0.986	1.023	1.026	1.102	0.965	1.116	0.984	1.019	Q9BRQ8	AIFM2	Apoptosis-inducing factor 2
1.313	1.147	1.354										O15033	AREL1	Apoptosis-resistant E3 ubiquitin protein ligase 1
0.988	0.977	0.991	0.984	0.969	1.011	0.983	0.934	0.995	1.017	1.007	1.010	Q9UKV3	ACIN1	Apoptotic chromatin condensation inducer in the nucleus
1.040	1.034	1.030	0.976	0.977	0.986	1.009	1.090	1.056	0.995	0.991	1.007	O14727	APAF1	Apoptotic protease-activating factor 1
0.918	1.028	1.095	1.033	0.948	1.003	0.946	0.952	1.059	1.149	0.960	1.100	Q8IW19	APLF	Aprataxin and PNK-like factor
1.029	0.956	0.995	0.995	0.884	1.026	0.987	1.116	1.010	1.007	0.976	0.973	Q7Z2E3	APTX	Aprataxin
									0.958	1.247	1.336	P16050	ALOX15	Arachidonate 15-lipoxygenase
1.012	1.030	1.063	1.016	0.985	1.036	0.995	1.081	1.024	1.034	1.001	1.066	Q96II5	ARAF	ARAF protein
1.208	1.123	1.030										Q86W34	AMZ2	Archaemetzincin-2
1.003	1.016	1.005	0.981	0.999	0.998	0.992	1.003	1.004	0.979	0.970	0.998	B0YIW6	ARCN1	Archain 1, isoform CRA_a
1.002	1.007	1.034	0.995	0.979	0.974	1.000	0.950	0.951	1.118	0.986	1.063	Q14161	GIT2	ARF GTPase-activating protein GIT2
1.053	0.964	1.017	1.028	0.998	0.996	1.000	1.043	0.963	1.026	0.987	1.004	P53367	ARFIP1	Arfaptin-1
0.997	0.992	1.019	0.940	0.911	0.973	0.978	1.050	0.985	1.025	1.096	1.037	A0A087X1E4	ARFIP2	Arfaptin-2
0.912	1.060	0.918	0.913	0.918	0.968	0.984	0.884	0.985	1.053	1.013	1.128	O95081	AGFG2	Arf-GAP domain and FG repeat-containing protein 2
1.044	0.995	1.021	1.019	0.997	1.026	1.002	0.935	0.984	0.991	1.010	1.010	Q15057	ACAP2	Arf-GAP with coiled-coil, ANK repeat and PH domain-containing protein 2
1.074	0.972	0.986	1.022	0.962	0.990							Q2V6Q1	CENTA2	Arf-GAP with dual PH domain-containing protein 2
0.923	0.970	0.993	0.986	0.980	1.058	0.936	0.878	1.058	0.976	0.975	1.079	Q9UPQ3	AGAP1	Arf-GAP with GTPase, ANK repeat and PH domain-containing protein 1
1.030	0.947	1.168	1.001	0.941	1.052	1.047	0.929	1.004	1.099	0.995	1.119	O43150	ASAP2	Arf-GAP with SH3 domain, ANK repeat and PH domain-containing protein 2

Abundance Ratio														
1 d M1/C1	1 d M2/C2	1 d M3/C3	4 d M1/C1	4 d M2/C2	4 d M3/C3	7 d M1/C1	7 d M2/C2	7 d M3/C3	10 d M1/C1	10 d M2/C2	10 d M3/C3	Accession ID	Gene name	Protein Description
1.012	0.947	1.022	0.991	0.965	1.016	0.946	0.808	0.958	1.053	1.008	1.056	Q9NWB6	ARGLU1	Arginine and glutamate-rich protein 1
1.006	0.983	0.971	1.023	0.932	1.037	1.011	1.053	0.958	1.036	1.068	1.025	Q7L4I2	RSRC2	Arginine/serine-rich coiled-coil protein 2
0.893	0.952	1.006	0.962	0.960	1.125	0.958	0.867	0.983	1.050	1.007	1.151	Q8TF01	PNISR	Arginine/serine-rich protein PNISR
1.055	0.936	1.046	0.986	0.877	0.950	0.945	0.986	1.011	0.951	1.024	0.966	Q5TEU4	NDUFAF5	Arginine-hydroxylase NDUFAF5, mitochondrial
1.011	1.004	1.010	1.002	0.962	0.984	1.000	1.038	1.001	0.963	1.010	0.958	P54136	RARS	Arginine--tRNA ligase, cytoplasmic
1.005	1.007	0.998	0.999	0.984	1.007	0.967	1.059	0.992	1.007	1.016	1.032	P04424	ASL	Argininosuccinate lyase
1.075	1.015	1.063	1.032	1.038	1.057	1.011	1.025	1.014	1.007	0.997	1.000	P00966	ASS1	Argininosuccinate synthase
1.090	0.948	0.935	0.919	0.944	1.131	1.029	1.122	1.085	1.025	0.913	1.092	F5GXE4	ATE1	Arginyl-tRNA--protein transferase 1
1.013	0.993	0.995	1.002	0.953	1.011	0.976	0.979	1.004	1.046	1.015	1.022	O95260	ATE1	Arginyl-tRNA--protein transferase 1
1.035	1.005	0.982	0.967	0.960	0.969	0.993	1.047	1.018	0.989	0.982	1.010	Q1RLN5	ARHGAP12	ARHGAP12 protein
0.953	1.049	1.036	1.042	0.944	1.043	1.038	1.131	1.120	0.950	1.005	1.028	Q8N8R7	ARL14EP	ARL14 effector protein
1.035	1.004	0.839										G5E9V6	ARMC8	Armadillo repeat containing 8, isoform CRA_e
			0.808	0.936	1.349	1.144	1.796	1.035	1.092	1.038	1.131	O00192	ARVCF	Armadillo repeat protein deleted in velo-cardio-facial syndrome
0.984	0.993	1.025	0.967	0.969	0.971	0.914	0.847	0.955	0.955	0.982	1.023	Q9NVT9	ARMC1	Armadillo repeat-containing protein 1
1.021	1.005	1.021	1.000	0.943	0.983	0.983	1.067	1.028	1.003	0.985	0.997	Q8N2F6	ARMC10	Armadillo repeat-containing protein 10
1.042	1.007	1.025	1.030	0.993	0.967	0.977	1.156	0.997	0.956	0.999	0.996	Q6NXE6	ARMC6	Armadillo repeat-containing protein 6
0.845	0.868	1.080	0.932	0.944	1.006	0.871	0.866	1.088	0.872	1.003	1.080	Q9H6L4	ARMC7	Armadillo repeat-containing protein 7
1.067	1.016	1.049	1.024	0.983	1.046	0.996	1.039	1.022	0.951	0.970	1.048	Q8IUR7	ARMC8	Armadillo repeat-containing protein 8
0.947	1.002	1.014	0.954	0.950	0.997	1.004	1.061	1.000	1.052	0.973	1.077	Q9UH62	ARMCX3	Armadillo repeat-containing X-linked protein 3
0.926	0.983	1.066							0.950	1.060	0.960	Q6P1M9	ARMCX5	Armadillo repeat-containing X-linked protein 5
0.949	1.059	1.269	1.057	1.096	1.064	1.056	0.974	1.032	1.116	1.046	1.052	Q8N5I2	ARRDC1	Arrestin domain-containing protein 1
1.363	0.962	1.090	1.159	1.101	1.067							Q96B67	ARRDC3	Arrestin domain-containing protein 3
0.998	1.066	1.034	1.008	1.188	1.003	0.957	1.098	1.052	1.056	1.049	1.110	P27540	ARNT	Aryl hydrocarbon receptor nuclear translocator
0.972	1.008	0.998	0.967	0.907	0.957	0.983	1.058	0.950	0.995	0.966	0.971	A0A0R4J2G3	NCEH1	Arylacetamide deacetylase-like 1
1.174	0.809	1.302	0.966	0.735	0.940	0.935	1.083	1.054				F5H5R8	NAT1	Arylamine N-acetyltransferase 1
0.936	0.996	0.972	1.009	0.976	0.987	0.970	1.035	1.035	1.053	0.990	1.044	A0A0C4DFZ2	ARSA	Arylsulfatase A
0.971	0.998	1.089	0.983	1.005	1.002	0.985	0.975	0.971	1.008	1.057	1.060	P15848	ARSB	Arylsulfatase B
1.026	1.250	1.013	0.981	0.898	0.939	0.992	1.426	0.905	0.907	0.953	1.174	P51689	ARSD	Arylsulfatase D
1.105	0.980	1.024	0.948	0.895	1.052	1.054	1.046	1.024	1.093	0.991	1.144	F5GYY5	ARSE	Arylsulfatase E
			1.003	0.919	0.996	0.922	1.037	0.997	1.083	0.964	1.115	Q6UWY0	ARSK	Arylsulfatase K
1.044	1.038	0.993	1.005	1.005	0.974	1.025	1.065	0.991	1.009	1.028	1.058	Q9BVC5	C2orf49	Ashwin
1.017	1.020	0.983	1.058	1.027	1.037	1.047	1.118	1.027	0.959	1.047	1.028	P08243	ASNS	Asparagine synthetase [glutamine-hydrolyzing]
1.011	0.806	0.912	0.835	0.610	1.052	1.341	0.855	0.848				Q9NWL6	ASNSD1	Asparagine synthetase domain-containing protein 1

Abundance Ratio														
1 d M1/C1	1 d M2/C2	1 d M3/C3	4 d M1/C1	4 d M2/C2	4 d M3/C3	7 d M1/C1	7 d M2/C2	7 d M3/C3	10 d M1/C1	10 d M2/C2	10 d M3/C3	Accession ID	Gene name	Protein Description
0.994	1.010	0.992	1.008	1.026	0.975	0.984	1.020	0.997	0.973	0.994	0.980	O43776	NARS	Asparagine--tRNA ligase, cytoplasmic
0.993	0.999	0.995	0.987	0.972	0.984	0.967	1.011	1.017	0.985	0.966	0.976	P17174	GOT1	Aspartate aminotransferase, cytoplasmic
1.024	1.008	1.000	1.010	1.162	0.956	1.020	0.962	1.017	0.993	0.988	0.974	P00505	GOT2	Aspartate aminotransferase, mitochondrial
1.013	1.018	0.991	1.007	1.014	0.970	0.993	1.009	0.977	0.982	1.005	0.969	P14868	DARS	Aspartate--tRNA ligase, cytoplasmic
1.020	1.003	1.015	0.977	0.997	0.994	0.995	0.942	1.003	0.974	0.979	1.009	Q6PI48	DARS2	Aspartate--tRNA ligase, mitochondrial
			1.016	0.943	0.894							B9ZVU2	DNPEP	Aspartyl aminopeptidase (Fragment)
0.980	1.013	1.009	0.975	0.994	0.997	0.986	1.000	0.973	0.988	0.992	0.999	E7ETB3	DNPEP	Aspartyl aminopeptidase
0.966	0.989	1.016	1.006	0.980	1.017	1.001	1.038	1.020	1.062	0.994	1.035	Q12797	ASPH	Aspartyl/asparaginyl beta-hydroxylase
0.998	1.003	1.016	1.009	0.985	1.022	1.028	1.039	1.038	0.997	0.976	1.018	Q9UBB4	ATXN10	Ataxin-10
1.003	0.995	1.038	1.014	1.039	1.029	0.991	0.972	0.948	1.072	1.068	1.252	P0C7T5	ATXN1L	Ataxin-1-like
1.008	0.999	1.043	0.990	0.979	0.974	1.036	0.950	1.008	1.061	1.009	1.066	Q99700	ATXN2	Ataxin-2
			0.919	0.981	0.698	0.937	1.530	0.929				H3BUF6	ATXN2L	Ataxin-2-like protein
0.988	1.003	0.992	0.996	0.975	1.000	0.999	0.981	0.997	1.016	1.063	1.016	Q8WWM7	ATXN2L	Ataxin-2-like protein
0.985	1.003	1.054	0.956	1.056	0.974	1.019	1.020	1.074	0.981	1.000	1.034	F5H211	ATXN3	Ataxin-3
1.095	1.077	1.048	1.098	1.031	1.005	0.974	0.776	1.086	1.040	0.982	1.027	Q6SPF0	SAMD1	Atherin
1.018	1.041	0.977	1.069	0.951	0.962	0.958	1.170	0.900	0.885	0.888	1.090	Q5TGY3	AHDC1	AT-hook DNA-binding motif-containing protein 1
0.980	0.964	1.047	1.030	0.941	1.017	1.076	0.965	1.036	1.029	0.864	1.063	Q8NHH9	ATL2	Atlastin-2
0.971	1.002	1.014	0.971	0.929	0.982	0.986	0.988	0.988	0.986	1.000	0.996	Q6DD88	ATL3	Atlastin-3
0.984	0.976	0.990	0.998	0.997	0.972	0.968	0.979	0.984	0.998	0.988	0.973	P24539	ATP5F1	ATP synthase F(0) complex subunit B1, mitochondrial
1.090	1.056	1.027	1.001	1.013	0.913	0.896	1.093	0.991	0.938	0.897	0.835	I3L448	ATPAF1	ATP synthase mitochondrial F1 complex assembly factor 1
0.993	1.000	1.005	0.964	0.941	1.025	0.992	1.009	1.029	1.060	0.942	1.065	Q8N5M1	ATPAF2	ATP synthase mitochondrial F1 complex assembly factor 2
						1.088	1.127	1.204	1.047	1.011	1.044	P03928	MT-ATP8	ATP synthase protein 8
0.979	0.952	0.936	0.908	0.715	0.923	0.902	1.112	0.953	0.983	1.020	0.853	P00846	MT-ATP6	ATP synthase subunit a
0.954	0.985	0.983	0.976	0.965	0.995	0.969	0.953	0.989	0.999	0.982	1.007	P25705	ATP5A1	ATP synthase subunit alpha, mitochondrial
1.135	0.934	1.628	1.023	0.987	0.884	0.952	1.067	1.074	1.021	0.972	1.145	F8W0P7	ATP5B	ATP synthase subunit beta, mitochondrial (Fragment)
0.948	1.014	0.981	0.984	1.004	1.021	1.001	1.047	1.018	0.996	0.999	1.007	P06576	ATP5B	ATP synthase subunit beta, mitochondrial
1.032	0.995	0.988	1.129	1.078	0.993	1.080	1.022	1.018	1.105	1.007	0.984	O75947	ATP5H	ATP synthase subunit d, mitochondrial
0.982	0.971	1.021	0.953	0.954	0.969	1.015	1.003	0.994	0.992	0.973	1.019	P30049	ATP5D	ATP synthase subunit delta, mitochondrial
0.937	1.010	0.991	0.923	1.014	0.951	0.950	1.026	0.966	0.978	0.986	0.963	P56385	ATP5I	ATP synthase subunit e, mitochondrial
1.247	1.009	1.012	1.146	1.826	0.925	1.123	0.774	1.140	1.027	0.959	0.894	P56381	ATP5E	ATP synthase subunit epsilon, mitochondrial
0.898	1.002	0.963	0.966	1.113	0.958	0.944	0.972	0.927	1.017	0.972	0.957	O75964	ATP5L	ATP synthase subunit g, mitochondrial

Abundance Ratio														
1 d M1/C1	1 d M2/C2	1 d M3/C3	4 d M1/C1	4 d M2/C2	4 d M3/C3	7 d M1/C1	7 d M2/C2	7 d M3/C3	10 d M1/C1	10 d M2/C2	10 d M3/C3	Accession ID	Gene name	Protein Description
0.900	0.873	0.980	0.966	0.851	1.090	0.928	0.985	1.138	0.946	0.815	1.269	P36542	ATP5C1	ATP synthase subunit gamma, mitochondrial
1.025	1.019	0.969	1.011	1.001	0.967	0.983	1.065	0.947	0.999	0.986	0.942	P48047	ATP5O	ATP synthase subunit O, mitochondrial
1.043	1.012	1.062	0.998	0.963	1.048	1.015	1.048	1.023	1.061	0.995	1.076	Q99766	ATP5S	ATP synthase subunit s, mitochondrial
0.928	0.989	0.989	0.973	0.894	1.019	0.966	1.069	1.041	1.022	0.944	1.014	G3V325	ATP5J2-PTCD1	ATP5J2-PTCD1 readthrough
0.973	0.994	1.005	0.993	1.023	0.982	0.984	1.086	1.023	0.986	0.989	0.989	O43681	ASNA1	ATPase ASNA1
0.977	0.973	1.037	0.995	1.012	1.015	0.989	1.074	1.030	1.017	1.002	0.988	Q8NBU5	ATAD1	ATPase family AAA domain-containing protein 1
0.921	0.977	0.937	1.058	1.006	1.006	1.010	1.071	1.046	0.981	0.980	1.065	Q6PL18	ATAD2	ATPase family AAA domain-containing protein 2
1.072	1.154	0.759							1.134	0.753	1.137	Q9ULI0	ATAD2B	ATPase family AAA domain-containing protein 2B
0.940	1.003	1.006	0.972	0.958	0.990	0.983	0.970	0.999	0.992	1.002	1.048	Q5T9A4	ATAD3B	ATPase family AAA domain-containing protein 3B
1.031	0.994	0.932	0.927	0.933	0.998	0.930	0.880	0.971	1.038	0.984	1.033	Q9UII2	ATPIF1	ATPase inhibitor, mitochondrial
1.023	0.981	1.015	1.030	0.966	1.006	1.004	1.053	0.982	1.021	0.988	1.009	Q96S55	WRNIP1	ATPase WRNIP1
0.987	0.998	1.076	0.962	0.885	1.056	1.023	1.010	1.123	0.993	0.941	1.013	Q99758	ABCA3	ATP-binding cassette sub-family A member 3
1.019	1.005	1.037	1.002	0.930	1.048	1.032	1.022	1.032	1.039	1.012	1.081	Q9NRK6	ABCB10	ATP-binding cassette sub-family B member 10, mitochondrial
1.062	0.968	1.058	0.993	0.920	1.028	1.028	1.039	1.017	1.248	0.919	1.096	Q9NUT2	ABCB8	ATP-binding cassette sub-family B member 8, mitochondrial
0.905	0.994	1.135	0.960	0.808	0.998	0.958	1.063	1.097	0.988	0.960	0.874	P33897	ABCD1	ATP-binding cassette sub-family D member 1
0.998	1.000	0.994	1.014	1.013	1.020	1.020	1.022	1.027	1.002	0.972	1.003	P28288	ABCD3	ATP-binding cassette sub-family D member 3
0.981	1.059	1.007	1.111	0.937	1.012	1.116	1.494	0.941				O14678	ABCD4	ATP-binding cassette sub-family D member 4
1.036	1.011	0.991	1.010	1.033	0.991	1.020	1.011	1.010	1.008	1.006	1.005	P61221	ABCE1	ATP-binding cassette sub-family E member 1
0.666	0.839	1.064	0.796	0.918	1.050	0.694	0.487	0.804	1.207	0.905	1.126	Q5STZ8	ABCF1	ATP-binding cassette sub-family F member 1 (Fragment)
0.954	0.998	0.975	0.958	1.002	0.985	0.981	0.974	0.993	0.977	0.990	1.007	Q8NE71	ABCF1	ATP-binding cassette sub-family F member 1
1.008	0.975	1.006	0.997	0.947	1.031	0.995	0.897	1.011	1.053	1.030	1.141	Q9NUQ8	ABCF3	ATP-binding cassette sub-family F member 3
0.942	0.994	0.977	1.019	1.034	0.994	1.030	1.034	1.030	1.032	1.024	1.014	P53396	ACLY	ATP-citrate synthase
1.048	1.042	1.001	0.989	0.974	0.979	1.044	1.038	1.010	0.988	1.014	1.004	Q8IW45	NAXD	ATP-dependent (S)-NAD(P)H-hydrate dehydratase
1.040	1.017	1.011	1.015	1.057	0.977	1.006	1.030	1.015	0.961	0.967	0.982	P17858	PFKL	ATP-dependent 6-phosphofructokinase, liver type
1.016	1.021	0.992	0.999	1.019	0.988	0.993	1.028	1.011	0.950	1.011	0.964	Q01813	PFKP	ATP-dependent 6-phosphofructokinase, platelet type
1.037	1.026	1.013	1.018	0.974	0.987	1.035	1.068	1.005	1.003	0.983	1.050	O76031	CLPX	ATP-dependent Clp protease ATP-binding subunit clpX-like, mitochondrial
1.011	1.018	0.976	1.002	0.954	1.014	1.003	1.023	0.990	1.020	1.023	0.986	Q16740	CLPP	ATP-dependent Clp protease proteolytic subunit, mitochondrial
0.972	1.004	0.991	0.988	1.022	0.980	0.969	0.978	0.975	0.960	0.998	0.951	P46063	RECQL	ATP-dependent DNA helicase Q1
0.998	1.038	0.987	1.324	0.847	1.058	1.092	1.155	0.961	0.966	0.939	0.861	A0A087WZ30	RECQL4	ATP-dependent DNA helicase Q4
			1.110	0.907	0.902	1.087	1.136	0.972	0.843	0.811	0.950	O94762	RECQL5	ATP-dependent DNA helicase Q5

Abundance Ratio														
1 d M1/C1	1 d M2/C2	1 d M3/C3	4 d M1/C1	4 d M2/C2	4 d M3/C3	7 d M1/C1	7 d M2/C2	7 d M3/C3	10 d M1/C1	10 d M2/C2	10 d M3/C3	Accession ID	Gene name	Protein Description
1.006	1.001	0.999	0.995	1.043	1.007	1.032	1.034	1.025	1.018	1.007	1.017	Q08211	DHX9	ATP-dependent RNA helicase A
0.989	1.014	0.991	0.998	1.021	0.996	0.984	1.028	1.010	1.006	1.002	1.003	Q92499	DDX1	ATP-dependent RNA helicase DDX1
0.978	1.007	0.994	1.003	0.978	1.018	0.987	1.014	0.989	1.001	1.061	0.994	Q9NVP1	DDX18	ATP-dependent RNA helicase DDX18
1.005	1.001	1.003	1.013	1.002	0.987	1.005	1.017	0.999	0.986	1.016	0.989	Q9NUU7	DDX19A	ATP-dependent RNA helicase DDX19A
0.983	1.068	0.942	0.939	1.003	1.003	1.059	1.136	1.004	0.918	1.038	1.074	H3BQK0	DDX19B	ATP-dependent RNA helicase DDX19B
0.996	1.007	0.996	1.008	0.984	1.004	1.001	0.986	0.984	1.043	1.049	1.017	Q9GZR7	DDX24	ATP-dependent RNA helicase DDX24
1.019	1.016	1.017	1.046	1.051	0.973	0.939	0.919	0.985	0.988	1.056	1.057	O00148	DDX39A	ATP-dependent RNA helicase DDX39A
1.019	1.000	0.990	1.018	1.022	1.016	1.026	1.019	1.021	1.021	0.995	1.025	O00571	DDX3X	ATP-dependent RNA helicase DDX3X
1.045	1.007	0.986	1.008	0.968	1.014	1.005	1.080	1.001	1.026	1.014	1.027	Q86XP3	DDX42	ATP-dependent RNA helicase DDX42
1.016	0.993	0.982	1.033	0.970	0.974	1.042	1.034	0.985	1.041	1.002	1.036	Q9BQ39	DDX50	ATP-dependent RNA helicase DDX50
1.010	1.007	0.992	0.995	0.983	1.040	1.001	1.072	1.013	1.016	0.980	1.035	Q8N8A6	DDX51	ATP-dependent RNA helicase DDX51
1.042	1.008	1.029	1.021	0.996	1.022	1.016	0.992	1.019	1.008	1.028	1.056	Q8TDD1	DDX54	ATP-dependent RNA helicase DDX54
1.082	0.989	1.086	0.996	1.003	0.997	0.996	1.091	1.031	1.005	1.029	1.022	Q8NHQ9	DDX55	ATP-dependent RNA helicase DDX55
1.022	1.006	1.031	0.995	0.986	1.020	0.959	0.987	1.001	1.015	0.998	1.034	A0A087WYN9	DHX29	ATP-dependent RNA helicase DHX29
1.011	0.996	1.010	0.976	0.946	1.009	1.015	1.068	1.070	1.023	1.003	1.051	Q9H2U1	DHX36	ATP-dependent RNA helicase DHX36
0.999	0.991	1.006	1.006	0.963	1.040	1.009	1.103	1.029	0.965	1.023	1.028	Q14562	DHX8	ATP-dependent RNA helicase DHX8
1.007	0.977	1.026	1.004	0.989	1.043	1.014	1.017	1.029	0.994	1.010	0.986	Q8IYB8	SUPV3L1	ATP-dependent RNA helicase SUPV3L1, mitochondrial
0.990	0.993	1.017	1.005	0.974	1.045	1.013	1.043	1.020	0.950	0.904	1.038	Q96TA2	YME1L1	ATP-dependent zinc metalloprotease YME1L1
1.143	0.958	1.107	1.010	1.055	1.102	0.998	1.076	0.953	1.137	1.052	0.972	P16066	NPR1	Atrial natriuretic peptide receptor 1
0.983	0.981	1.018	1.012	0.986	1.033	1.035	0.992	0.996	1.011	1.027	1.054	O14497	ARID1A	AT-rich interactive domain-containing protein 1A
0.983	0.994	1.001	0.975	0.920	1.022	0.974	0.955	1.011	1.024	1.020	1.041	Q68CP9	ARID2	AT-rich interactive domain-containing protein 2
1.040	1.059	1.054	1.140	0.794	1.066	0.929	0.971	1.054	0.980	0.929	1.190	Q99856	ARID3A	AT-rich interactive domain-containing protein 3A
1.057	1.012	0.983	1.007	0.899	0.964	1.024	1.046	0.967	1.082	0.969	1.070	P29374	ARID4A	AT-rich interactive domain-containing protein 4A
1.005	0.954	1.081	1.025	0.929	1.039	0.948	0.867	1.017	1.025	0.986	1.052	Q4LE39	ARID4B	AT-rich interactive domain-containing protein 4B
0.993	1.035	1.252	0.950	0.993	0.936	0.876	1.232	1.001	0.844	0.900	0.939	Q8WXE1	ATRIP	ATR-interacting protein
1.089	0.915	0.907	0.974	0.914	1.020	0.900	1.072	0.961	1.043	1.048	1.118	P54259	ATN1	Atrophin-1
1.073	1.067	1.045	1.025	1.022	1.089	0.964	1.154	1.091	1.023	1.049	1.031	O75882	ATRN	Attractin
1.010	1.020	0.994	0.977	0.958	0.991	0.954	1.029	0.994	0.980	1.004	0.993	Q8NI60	COQ8A	Atypical kinase COQ8A, mitochondrial
1.033	1.000	1.080	1.008	0.980	0.977	0.963	0.993	1.056	1.007	1.034	1.094	Q96D53	COQ8B	Atypical kinase COQ8B, mitochondrial
0.950	0.998	1.010	1.009	0.904	1.310	0.997	1.159	0.961	1.080	0.957	1.166	O14965	AURKA	Aurora kinase A
0.957	0.973	1.040	1.002	0.966	1.031	0.997	1.002	1.029	1.088	1.008	1.123	Q9NWT8	AURKAIP1	Aurora kinase A-interacting protein
1.004	1.042	1.015	0.974	0.972	0.960	0.959	1.066	0.982	0.982	0.986	0.982	Q9H1Y0	ATG5	Autophagy protein 5
1.040	1.010	1.116	0.922	0.968	1.121	1.023	0.846	1.062	0.965	1.006	1.077	Q9BSB4	ATG101	Autophagy-related protein 101

Abundance Ratio														
1 d M1/C1	1 d M2/C2	1 d M3/C3	4 d M1/C1	4 d M2/C2	4 d M3/C3	7 d M1/C1	7 d M2/C2	7 d M3/C3	10 d M1/C1	10 d M2/C2	10 d M3/C3	Accession ID	Gene name	Protein Description
0.975	0.977	1.026	0.975	1.007	1.031	1.001	0.949	1.012	0.993	0.973	1.079	Q676U5	ATG16L1	Autophagy-related protein 16-1
0.939	1.019	1.091	0.962	0.948	1.076	0.978	0.962	1.148	1.106	1.019	0.976	Q96BY7	ATG2B	Autophagy-related protein 2 homolog B
0.999	0.971	1.001	0.986	0.960	1.067	0.901	1.109	0.984	1.057	0.905	1.056	Q7Z3C6	ATG9A	Autophagy-related protein 9A
1.089	1.014	1.041	0.997	0.955	0.985	0.978	1.039	0.987	0.938	1.004	0.940	Q96BJ3	AIDA	Axin interactor, dorsalization-associated protein
1.129	1.125	1.045	0.842	1.507	1.099	0.956	0.849	1.087				O15169	AXIN1	Axin-1
1.087	0.976	1.002	1.171	1.126	1.071	1.017	0.743	0.903	1.007	0.853	1.166	Q9UPM9	B9D1	B9 domain-containing protein 1
0.965	0.977	1.015	0.976	0.964	1.024	1.018	1.061	1.015	1.007	1.015	1.034	Q13490	BIRC2	Baculoviral IAP repeat-containing protein 2
			1.025	0.691	1.056							Q13489	BIRC3	Baculoviral IAP repeat-containing protein 3
0.921	0.968	1.031	1.050	1.067	1.174	1.032	0.913	0.992	0.974	0.826	1.063	O15392	BIRC5	Baculoviral IAP repeat-containing protein 5
0.978	1.028	1.044	0.993	0.932	1.052	1.037	1.054	0.961	0.946	0.933	1.005	Q9NR09	BIRC6	Baculoviral IAP repeat-containing protein 6
1.011	0.939	1.010	1.044	0.984	1.048	1.121	0.996	1.072	1.115	1.027	1.108	J3QTA2	BAG1	BAG family molecular chaperone regulator 1
0.974	0.995	0.994	0.931	0.942	1.016	0.976	0.921	1.008	1.007	0.958	1.025	O95816	BAG2	BAG family molecular chaperone regulator 2
1.004	0.993	0.986	1.005	0.978	1.026	0.999	0.966	1.012	1.002	0.977	1.047	O95817	BAG3	BAG family molecular chaperone regulator 3
1.018	0.986	1.026	1.060	0.949	1.093	1.037	0.981	1.091	1.011	1.037	1.010	O95429	BAG4	BAG family molecular chaperone regulator 4
0.800	0.907	0.927	1.055	1.028	1.049	1.157	1.148	0.997	1.289	0.908	1.368	A0A075B747	BAHCC1	BAH and coiled-coil domain-containing protein 1
1.001	1.001	1.005	0.991	1.007	1.004	1.001	1.005	0.992	0.983	0.965	1.011	O43491	EPB41L2	Band 4.1-like protein 2
1.072	1.023	1.061	1.064	0.908	0.994	1.007	0.980	1.040	1.010	0.977	1.053	Q9BXC9	BBS2	Bardet-Biedl syndrome 2 protein
0.999	1.006	1.041	1.029	0.955	0.990	0.995	1.095	0.976	0.966	1.035	1.078	Q8IWZ6	BBS7	Bardet-Biedl syndrome 7 protein
1.160	1.119	1.135	1.075	1.130	0.915	0.828	0.885	0.978	0.896	1.021	0.837	O75531	BANF1	Barrier-to-autointegration factor
0.955	0.964	0.993	1.029	0.969	0.986	1.091	0.932	1.098	1.151	1.279	1.145	P50895	BCAM	Basal cell adhesion molecule
0.966	1.076	1.204	1.039	0.995	1.071							P98160	HSPG2	Basement membrane-specific heparan sulfate proteoglycan core protein
0.990	0.995	1.013	1.001	0.958	1.032	0.984	1.014	1.007	1.015	1.010	1.036	Q9Y6E2	BZW2	Basic leucine zipper and W2 domain-containing protein 2
0.966	0.993	0.981	0.995	0.965	1.025	1.004	1.000	0.990	0.963	1.016	0.959	P35613	BSG	Basigin
1.005	0.923	0.951	0.977	0.937	1.039	0.991	1.108	1.068	1.227	1.048	0.996	Q13286	CLN3	Battenin
1.034	0.983	1.010	1.056	0.889	0.990	1.071	1.068	0.997	1.064	0.983	1.090	Q9BQE9	BCL7B	B-cell CLL/lymphoma 7 protein family member B
1.012	1.003	1.028	1.021	0.918	1.002	0.946	1.030	1.003	1.058	1.010	1.127	I3L1Q2	BCL7C	B-cell CLL/lymphoma 7 protein family member C (Fragment)
0.986	0.990	1.032	0.984	0.994	1.075	1.015	0.910	0.987	0.973	1.029	1.105	Q86UU0	BCL9L	B-cell CLL/lymphoma 9-like protein
1.055	1.014	1.104	0.922	0.931	0.991	0.990	1.127	1.048	1.003	1.051	1.009	P20749	BCL3	B-cell lymphoma 3 protein
0.971	0.969	0.983	0.966	0.952	1.025	1.032	1.005	0.961	1.068	0.986	1.122	O95999	BCL10	B-cell lymphoma/leukemia 10
0.917	0.984	0.947	0.964	0.986	1.045	1.081	0.987	0.978	1.129	1.065	1.117	Q9UHQ4	BCAP29	B-cell receptor-associated protein 29
0.981	0.951	0.979	0.930	0.821	1.001	0.945	1.021	0.980	0.940	0.949	1.012	Q16611	BAK1	Bcl-2 homologous antagonist/killer

Abundance Ratio														
1 d M1/C1	1 d M2/C2	1 d M3/C3	4 d M1/C1	4 d M2/C2	4 d M3/C3	7 d M1/C1	7 d M2/C2	7 d M3/C3	10 d M1/C1	10 d M2/C2	10 d M3/C3	Accession ID	Gene name	Protein Description
1.066	1.111	1.135	0.987	1.054	1.038	0.976	0.942	1.040	1.000	1.006	0.916	J3KN59	BNIP2	BCL2/adenovirus E1B 19 kDa protein-interacting protein 2
0.949	0.926	0.980	1.012	0.948	0.993	0.988	1.080	1.049	1.086	1.008	1.152	A0A0J9YW18	BNIP3	BCL2/adenovirus E1B 19 kDa protein-interacting protein 3 (Fragment)
0.968	0.930	1.017										O60238	BNIP3L	BCL2/adenovirus E1B 19 kDa protein-interacting protein 3-like
1.004	1.024	0.986	1.008	0.956	0.992	1.051	1.017	0.983	1.099	1.042	1.122	Q92934	BAD	Bcl2-associated agonist of cell death
1.101	0.955	1.013	0.986	0.909	1.026	0.970	1.003	0.982				E9PK91	BCLAF1	Bcl-2-associated transcription factor 1
0.999	1.008	0.991	0.995	0.966	0.968	1.011	0.925	0.983	1.033	1.029	1.037	Q9NYF8	BCLAF1	Bcl-2-associated transcription factor 1
			1.002	1.163	0.958	1.056	1.015	0.990	1.156	0.995	1.119	Q9BXH1	BBC3	Bcl-2-binding component 3
0.990	1.012	1.014	0.957	0.919	1.092	1.074	1.076	1.020	1.032	0.984	1.054	Q07817	BCL2L1	Bcl-2-like protein 1
0.754	0.832	0.906	0.956	1.010	0.908	1.044	1.226	1.283				Q9HB09	BCL2L12	Bcl-2-like protein 12
0.961	0.987	1.042	0.972	0.940	1.033	0.969	0.945	0.987	1.078	1.017	1.036	Q9BXK5	BCL2L13	Bcl-2-like protein 13
1.069	1.128	1.055	0.841	0.670	1.093							Q96LC9	BMF	Bcl-2-modifying factor
			0.961	0.961	1.118	0.921	0.812	1.203	0.905	1.016	1.021	Q9UMX3	BOK	Bcl-2-related ovarian killer protein
1.241	0.898	0.849	0.873	0.826	0.850	1.036	1.093	0.990	0.965	1.001	1.113	Q6ZNE5	ATG14	Beclin 1-associated autophagy-related key regulator
0.968	1.008	1.010	1.009	0.955	1.002	0.973	1.037	1.023	1.052	0.975	1.012	Q14457	BECN1	Beclin-1
1.096	1.016	1.046	0.999	0.924	1.075	1.009	0.986	1.158	0.978	1.043	1.088	H7C1N3	BET1	BET1 homolog (Fragment)
			1.238	0.821	1.038	1.023	1.148	0.964				B8ZZS0	BET1L	BET1-like protein
0.711	0.886	1.056	0.858	0.683	1.005							Q96L58	B3GALT6	Beta-1,3-galactosyltransferase 6
1.057	1.025	1.020	0.977	0.986	1.008	0.974	1.029	0.984	1.006	1.015	0.998	Q6Y288	B3GLCT	Beta-1,3-glucosyltransferase
0.975	0.998	1.036	1.063	0.961	1.125	1.092	1.034	1.037				Q00973	B4GALNT1	Beta-1,4 N-acetylgalactosaminyltransferase 1
0.965	0.983	1.057	0.885	0.833	1.014	1.013	1.088	0.968	1.037	0.994	1.000	P15291	B4GALT1	Beta-1,4-galactosyltransferase 1
									1.610	1.010	1.049	O60512	B4GALT3	Beta-1,4-galactosyltransferase 3
1.030	0.992	0.916	0.985	0.890	0.983	0.989	0.883	0.968	0.971	0.992	1.080	O60513	B4GALT4	Beta-1,4-galactosyltransferase 4
1.191	0.977	1.109	1.014	0.805	0.934	1.233	1.096	0.870	1.091	1.192	1.042	O43286	B4GALT5	Beta-1,4-galactosyltransferase 5
			1.067	0.797	0.912				0.946	0.868	0.980	Q9UBX8	B4GALT6	Beta-1,4-galactosyltransferase 6
0.912	1.051	1.156	0.907	0.908	0.931	0.874	1.054	1.126	1.012	1.034	1.041	Q9UBV7	B4GALT7	Beta-1,4-galactosyltransferase 7
1.211	0.913	1.016	0.997	0.789	0.936	1.030	0.946	0.988				O43505	B4GAT1	Beta-1,4-glucuronyltransferase 1
0.994	1.047	1.063	0.963	1.002	0.971	0.927	0.988	0.943	0.940	0.943	1.007	P61769	B2M	Beta-2-microglobulin
0.950	0.993	0.999	0.961	0.941	0.994	1.008	1.017	0.997	0.997	0.974	1.020	Q13425	SNTB2	Beta-2-syntrophin
1.073	1.020	0.954	1.017	0.993	0.929	0.945	1.048	0.987	0.968	0.964	1.146	Q562R1	ACTBL2	Beta-actin-like protein 2
0.983	0.995	1.067	1.021	0.966	1.074	0.986	1.004	1.054	0.964	0.959	1.088	P25098	GRK2	Beta-adrenergic receptor kinase 1
			1.195	1.117	1.102	0.891	1.278	1.072				P35626	GRK3	Beta-adrenergic receptor kinase 2

Abundance Ratio														
1 d M1/C1	1 d M2/C2	1 d M3/C3	4 d M1/C1	4 d M2/C2	4 d M3/C3	7 d M1/C1	7 d M2/C2	7 d M3/C3	10 d M1/C1	10 d M2/C2	10 d M3/C3	Accession ID	Gene name	Protein Description
1.043	0.965	1.007	0.962	1.056	0.924	0.997	0.905	1.063	1.075	0.986	1.025	P49407	ARRB1	Beta-arrestin-1
0.877	1.184	1.073	0.989	0.994	1.065	0.964	0.936	1.069	0.900	0.941	0.894	Q9NSA3	CTNNBIP1	Beta-catenin-interacting protein 1
0.932	0.980	0.987	0.970	0.953	1.021	0.992	1.017	0.957	0.974	0.991	1.023	A0A087WUB9	CTNNBL1	Beta-catenin-like protein 1
0.958	1.000	1.064	0.992	0.964	0.992	0.957	0.921	0.950	1.038	0.986	1.047	P42025	ACTR1B	Beta-centractin
1.022	1.100	1.006	1.000	0.910	0.892	0.947	1.159	0.995	1.056	0.971	1.124	P13929	ENO3	Beta-enolase
1.013	1.009	0.999	1.003	1.028	0.985	1.025	1.052	0.999	1.014	0.981	0.995	P16278	GLB1	Beta-galactosidase
1.034	0.990	1.009	0.968	0.939	1.023	1.022	1.032	1.018	1.028	0.993	1.009	P08236	GUSB	Beta-glucuronidase
0.989	0.983	0.984	1.014	1.004	1.045	1.040	1.057	1.047	1.059	0.989	1.060	H3BP20	HEXA	Beta-hexosaminidase
1.011	0.993	0.997	1.015	0.996	1.026	1.012	1.050	1.013	1.043	1.036	0.970	P07686	HEXB	Beta-hexosaminidase subunit beta
0.978	1.005	0.987	1.035	0.935	1.002	1.026	1.193	1.076	1.058	1.045	1.076	O00462	MANBA	Beta-mannosidase
1.001	0.986	0.997	0.981	0.921	1.021	1.002	1.059	1.020	1.014	0.882	1.050	Q9HBI1	PARVB	Beta-parvin
									1.035	1.021	1.138	Q9H115	NAPB	Beta-soluble NSF attachment protein
1.031	1.036	0.996	1.025	1.033	0.988	1.023	1.035	0.994	1.024	1.016	0.993	O43252	PAPSS1	Bifunctional 3'-phosphoadenosine 5'-phosphosulfate synthase 1
0.989	1.003	0.986	1.007	1.012	0.987	0.982	0.995	0.994	0.975	1.004	0.962	P07814	EPRS	Bifunctional glutamate/proline--tRNA ligase
1.008	1.074	1.173	1.052	0.785	1.146	0.979	1.056	0.957	0.996	1.087	1.017	P52848	NDST1	Bifunctional heparan sulfate N-deacetylase/N-sulfotransferase 1
1.416	0.956	1.057	1.193	0.888	0.980							P52849	NDST2	Bifunctional heparan sulfate N-deacetylase/N-sulfotransferase 2
1.023	1.000	1.031	1.015	1.030	1.049	0.985	1.028	1.046	0.981	1.018	1.020	P13995	MTHFD2	Bifunctional methylenetetrahydrofolate dehydrogenase/cyclohydrolase, mitochondrial
1.012	1.026	1.024	1.004	0.977	1.042	0.989	1.000	1.010	1.046	1.023	1.066	Q96T60	PNKP	Bifunctional polynucleotide phosphatase/kinase
1.002	1.055	1.085	0.756	0.929	1.077	1.221	1.204	0.951				A0A0D9SFL2	PNKP	Bifunctional polynucleotide phosphatase/kinase
1.027	1.005	1.002	1.023	1.071	0.970	0.990	1.021	1.019	0.970	1.006	0.958	P31939	ATIC	Bifunctional purine biosynthesis protein PURH
0.850	1.017	1.008	1.015	1.061	1.126	1.051	0.926	1.060	0.972	1.034	1.187	O95342	ABCB11	Bile salt export pump
0.991	1.001	0.994	0.985	0.978	1.040	1.004	1.042	1.019	0.982	0.968	1.033	P53004	BLVRA	Biliverdin reductase A
1.081	1.034	0.959	0.975	1.015	0.957	0.966	1.066	0.997	0.996	0.973	0.976	P78537	BLOC1S1	Biogenesis of lysosome-related organelles complex 1 subunit 1
1.007	0.952	1.032	1.021	1.018	0.994	1.094	1.082	0.948	1.074	1.026	1.207	Q6QNY1	BLOC1S2	Biogenesis of lysosome-related organelles complex 1 subunit 2
1.017	0.954	1.045	1.039	1.007	1.022	1.035	0.991	1.022	1.063	0.987	1.027	Q6QNY0	BLOC1S3	Biogenesis of lysosome-related organelles complex 1 subunit 3
1.058	1.060	1.051	1.062	0.924	0.991	0.993	1.135	0.931	0.969	1.023	0.995	Q9NUP1	BLOC1S4	Biogenesis of lysosome-related organelles complex 1 subunit 4
1.130	1.104	1.175	1.013	0.959	1.075	1.047	0.966	1.005	1.055	1.032	1.084	Q8TDH9	BLOC1S5	Biogenesis of lysosome-related organelles complex 1 subunit 5

Abundance Ratio														
1 d M1/C1	1 d M2/C2	1 d M3/C3	4 d M1/C1	4 d M2/C2	4 d M3/C3	7 d M1/C1	7 d M2/C2	7 d M3/C3	10 d M1/C1	10 d M2/C2	10 d M3/C3	Accession ID	Gene name	Protein Description
1.078	0.970	1.102	1.018	1.075	1.090	0.930	0.976	1.066	0.974	1.153	1.074	H3BST1	BLOC1S6	Biogenesis of lysosome-related organelles complex 1 subunit 6
1.139	1.080	0.912	1.336	1.701	0.930				1.110	0.989	1.123	C9JNZ5	BOD1	Biorientation of chromosomes in cell division protein 1
0.994	0.898	0.994	0.930	0.966	1.086	0.963	1.011	1.006	0.893	1.040	0.993	Q96IK1	BOD1	Biorientation of chromosomes in cell division protein 1
1.007	1.001	1.029	0.978	0.949	1.003	0.972	1.000	0.970	1.044	1.034	1.089	Q8NFC6	BOD1L1	Biorientation of chromosomes in cell division protein 1-like 1
1.079	1.028	1.059	1.008	0.948	1.078	1.058	1.049	0.976	1.058	1.074	1.130	P50747	HLCS	Biotin--protein ligase
0.977	1.025	1.031	1.110	1.043	1.171	1.185	1.154	1.159	1.168	1.131	1.089	Q9Y6X5	ENPP4	Bis(5'-adenosyl)-triphosphatase ENPP4
						0.989	0.475	0.979				P49789	FHIT	Bis(5'-adenosyl)-triphosphatase
1.056	0.988	0.988	0.999	1.039	1.035	1.033	1.071	1.033	0.972	1.008	1.001	P50583	NUDT2	Bis(5'-nucleosyl)-tetraphosphatase [asymmetrical]
1.194	1.126	1.091	0.974	1.009	1.014	0.861	1.121	1.089	0.979	0.991	1.093	P07738	BPGM	Bisphosphoglycerate mutase
1.020	1.018	1.019	1.016	1.011	1.015	1.028	1.093	0.999	1.013	1.031	1.051	Q13867	BLMH	Bleomycin hydrolase
1.022	0.961	1.014	1.179	0.989	1.054	1.102	1.065	1.056	1.102	0.939	1.145	Q969J3	BORCS5	BLOC-1-related complex subunit 5
1.021	1.074	0.981	1.021	0.887	0.981	1.028	1.110	0.957	1.099	1.020	1.076	Q96GS4	BORCS6	BLOC-1-related complex subunit 6
1.012	0.944	1.070	0.922	0.880	1.026	0.912	1.275	0.979				Q96FH0	BORCS8	BLOC-1-related complex subunit 8
0.825	1.128	1.079	1.008	0.949	1.184	1.020	0.641	0.922	1.022	0.940	1.164	P54132	BLM	Bloom syndrome protein
1.020	1.011	1.018	1.012	0.956	1.032	1.031	1.016	1.003	1.006	1.020	1.092	Q9NSY1	BMP2K	BMP-2-inducible protein kinase
1.052	0.928	1.123	0.714	0.970	0.856	0.989	0.970	1.012	1.091	1.009	1.154	Q9Y3E2	BOLA1	BolA-like protein 1
1.041	0.989	0.985	0.987	1.065	0.994	1.031	1.029	1.002	0.994	0.984	1.012	A0A087WZT3	BOLA2	BolA-like protein 2
1.054	0.994	1.003	0.995	1.027	0.947	1.012	0.929	1.066	1.012	1.094	1.112	Q53S33	BOLA3	BolA-like protein 3
			0.992	1.273	0.959							P22004	BMP6	Bone morphogenetic protein 6
1.100	0.985	0.982				0.903	0.956	1.093				Q13873	BMPR2	Bone morphogenetic protein receptor type-2
1.055	1.049	1.056	1.108	0.987	0.959	0.939	1.163	0.919	1.039	1.178	1.173	A0A0B4J1R7	BORCS7-ASMT	BORCS7-ASMT readthrough (NMD candidate)
0.972	1.024	1.042	0.938	0.804	0.953				0.873	1.041	1.155	Q53HL2	CDCA8	Borealin
1.022	0.973	0.978	1.048	0.952	1.049	1.024	0.998	1.003	1.042	1.032	1.069	Q9NWK9	ZNHIT6	Box C/D snoRNA protein 1
			1.014	0.942	1.037							P59826	BPIFB3	BPI fold-containing family B member 3
1.013	0.985	0.956	0.859	0.952	0.994	0.979	0.768	0.885	1.201	1.037	1.019	P80723	BASP1	Brain acid soluble protein 1
						1.049	0.992	1.027	0.781	1.437	0.778	O95415	BRI3	Brain protein I3
1.020	1.015	0.995	1.027	0.977	1.032	1.005	1.043	0.988	0.960	1.031	1.022	Q9UQB8	BAIAP2	Brain-specific angiogenesis inhibitor 1-associated protein 2
0.992	1.008	0.991	0.995	0.973	0.976	1.037	1.020	0.984	1.011	0.952	1.077	Q9UHR4	BAIAP2L1	Brain-specific angiogenesis inhibitor 1-associated protein 2-like protein 1
1.004	1.015	0.927	1.025	1.022	0.987	1.060	1.069	1.008	1.057	0.991	0.999	O15382	BCAT2	Branched-chain-amino-acid aminotransferase, mitochondrial

Abundance Ratio												Accession ID	Gene name	Protein Description
1 d M1/C1	1 d M2/C2	1 d M3/C3	4 d M1/C1	4 d M2/C2	4 d M3/C3	7 d M1/C1	7 d M2/C2	7 d M3/C3	10 d M1/C1	10 d M2/C2	10 d M3/C3			
1.026	1.107	1.029	0.994	0.910	0.950	1.025	1.007	1.103	1.097	1.026	1.026	Q6UWZ7	FAM175A	BRCA1-A complex subunit Abraxas
1.045	1.016	1.013	1.021	1.012	1.005	0.982	1.051	0.980	0.997	0.999	0.990	Q9NXR7	BRE	BRCA1-A complex subunit BRE
0.942	0.878	1.078	0.918	0.928	1.032	0.964	0.921	1.093	1.035	1.065	1.165	Q96RL1	UIMC1	BRCA1-A complex subunit RAP80
1.019	0.970	1.025	1.114	1.024	0.845	0.992	0.984	1.123	0.991	0.899	1.095	Q6PJG6	BRAT1	BRCA1-associated ATM activator 1
1.077	0.938	1.022	1.046	0.894	1.068	1.155	0.912	0.962	1.136	0.867	1.110	Q7Z569	BRAP	BRCA1-associated protein
0.995	0.978	1.001	0.905	0.914	1.082	1.011	1.157	1.164				Q99728	BARD1	BRCA1-associated RING domain protein 1
1.035	1.009	1.016	1.015	0.955	1.061	0.955	1.094	1.034	0.900	0.998	0.952	Q9P287	BCCIP	BRCA2 and CDKN1A-interacting protein
1.037	0.984	0.993	1.009	0.927	0.987	0.972	0.991	1.037	1.002	0.963	1.056	P11274	BCR	Breakpoint cluster region protein
1.006	1.056	1.000										Q05GC8	BCAR2	Breast cancer anti-estrogen resistance 2
1.066	1.006	1.047	1.019	0.952	1.009	0.998	1.063	0.987	0.986	0.956	1.030	O75815	BCAR3	Breast cancer anti-estrogen resistance protein 3
1.162	1.069	1.108										G5E9I4	BRMS1	Breast cancer metastasis suppressor 1, isoform CRA_c
0.997	0.943	0.831	0.971	1.013	1.104				0.995	1.025	0.981	Q5PSV4	BRMS1L	Breast cancer metastasis-suppressor 1-like protein
1.032	1.008	0.982	0.996	0.944	0.989	1.011	1.058	0.996	1.017	1.019	1.004	Q9H6U6	BCAS3	Breast carcinoma-amplified sequence 3
1.025	1.004	1.029	1.000	0.956	1.031	1.024	1.023	1.056	1.006	0.976	1.072	Q9Y6D6	ARFGEF1	Brefeldin A-inhibited guanine nucleotide-exchange protein 1
0.988	1.001	1.001	1.000	0.983	1.046	1.021	1.036	1.011	0.994	1.013	1.024	Q9Y6D5	ARFGEF2	Brefeldin A-inhibited guanine nucleotide-exchange protein 2
1.007	1.054	1.013	0.954	0.957	0.999	1.036	1.166	1.024				Q5TH69	ARFGEF3	Brefeldin A-inhibited guanine nucleotide-exchange protein 3
0.927	0.940	0.934	0.900	0.946	1.000	1.072	1.130	1.028	1.037	1.053	1.014	Q8WY22	BRI3BP	BRI3-binding protein
0.771	0.899	1.562	0.943	0.896	1.043	1.004	0.839	1.021	0.968	0.885	1.021	Q9NQY0	BIN3	Bridging integrator 3
0.968	0.971	1.022	0.993	0.951	0.979	1.007	0.930	0.997	1.001	0.977	1.028	Q9NWV8	BABAM1	BRISC and BRCA1-A complex member 1
1.036	1.018	0.979	0.979	0.946	0.941	0.992	1.052	0.959	1.048	0.995	0.995	Q15018	FAM175B	BRISC complex subunit Abro1
1.014	1.004	1.029	1.011	0.994	0.977	1.000	1.064	1.007	1.045	1.013	1.042	Q5VW32	BROX	BRO1 domain-containing protein BROX
0.981	1.013	1.016	1.030	0.967	1.049	1.039	0.939	1.009	1.014	0.965	1.051	Q9NRL2	BAZ1A	Bromodomain adjacent to zinc finger domain protein 1A
1.012	1.001	1.004	0.975	0.912	1.008	1.055	1.076	1.021	1.111	1.004	1.042	Q9UIF9	BAZ2A	Bromodomain adjacent to zinc finger domain protein 2A
2.230	2.465	2.096	1.921	1.869	2.236	2.783	3.041	2.671				H7BXK5	BAZ2B	Bromodomain adjacent to zinc finger domain protein 2B (Fragment)
1.017	0.940	0.878				0.709	1.239	1.104				Q9UIF8	BAZ2B	Bromodomain adjacent to zinc finger domain protein 2B
1.022	1.127	0.990	1.155	0.693	0.937	1.342	1.096	0.825	1.179	0.940	1.123	Q9ULD4	BRPF3	Bromodomain and PHD finger-containing protein 3
1.027	1.009	0.979	1.143	0.905	1.010	1.121	1.041	1.034	0.982	0.992	1.029	A0A0G2JK44	BRD2	Bromodomain-containing protein 2

Abundance Ratio														
1 d M1/C1	1 d M2/C2	1 d M3/C3	4 d M1/C1	4 d M2/C2	4 d M3/C3	7 d M1/C1	7 d M2/C2	7 d M3/C3	10 d M1/C1	10 d M2/C2	10 d M3/C3	Accession ID	Gene name	Protein Description
0.979	0.980	1.030	0.982	0.977	1.043	1.001	1.005	1.122	1.006	0.933	1.110	Q15059	BRD3	Bromodomain-containing protein 3
1.048	1.012	1.023	0.990	0.961	0.993	1.054	1.087	1.020	0.970	0.973	1.008	O60885	BRD4	Bromodomain-containing protein 4
0.977	0.948	0.987	0.975	0.994	0.972	1.013	1.113	1.006	0.965	1.038	1.001	Q9H8M2	BRD9	Bromodomain-containing protein 9
1.072	1.013	0.981	1.043	1.118	0.922	0.991	0.991	1.021	0.953	0.971	1.006	Q9NW68	BSDC1	BSD domain-containing protein 1
									1.053	0.879	1.214	Q13829	TNFAIP1	BTB/POZ domain-containing adapter for CUL3-mediated RhoA degradation protein 2
1.022	0.976	1.038	1.004	0.920	1.043	1.030	1.100	0.986	1.103	0.986	1.064	Q9H3F6	KCTD10	BTB/POZ domain-containing adapter for CUL3-mediated RhoA degradation protein 3
1.301	0.988	0.999	1.043	1.006	0.985	0.968	0.878	0.921	1.453	0.979	1.081	Q9H0C5	BTBD1	BTB/POZ domain-containing protein 1
0.902	0.935	1.020	0.952	0.684	0.866	0.960	0.851	1.028				Q9BX70	BTBD2	BTB/POZ domain-containing protein 2
1.043	1.036	0.953	1.105	0.887	1.175	1.002	1.173	1.108	1.037	0.933	1.069	J3QRK1	KCTD1	BTB/POZ domain-containing protein KCTD1 (Fragment)
1.022	0.979	0.988	0.988	0.954	0.961	1.058	0.968	0.978	1.057	0.989	1.023	Q96CX2	KCTD12	BTB/POZ domain-containing protein KCTD12
1.067	1.059	0.952	1.027	0.959	1.010	1.007	1.098	0.997	1.068	0.984	1.022	Q9BQ13	KCTD14	BTB/POZ domain-containing protein KCTD14
0.849	1.000	1.653	0.846	0.870	0.965	0.929	1.004	1.251	1.054	1.065	1.006	Q96SI1	KCTD15	BTB/POZ domain-containing protein KCTD15
1.020	0.933	1.013	0.960	0.918	1.030	1.103	0.910	1.051	1.058	1.175	1.042	Q6PI47	KCTD18	BTB/POZ domain-containing protein KCTD18
1.206	1.008	0.960				1.058	1.188	0.996	0.950	0.939	0.925	Q14681	KCTD2	BTB/POZ domain-containing protein KCTD2
1.077	1.007	1.063	1.016	1.060	1.018	1.037	1.037	1.035	1.013	0.904	0.924	Q4G0X4	KCTD21	BTB/POZ domain-containing protein KCTD21
0.911	0.948	1.037	1.072	0.957	1.025							Q9Y597	KCTD3	BTB/POZ domain-containing protein KCTD3
1.086	1.051	0.951	1.043	1.039	0.958	1.021	1.099	0.943	0.895	1.101	0.942	Q9NXV2	KCTD5	BTB/POZ domain-containing protein KCTD5
1.035	1.010	1.053	0.799	0.746	1.032	1.012	1.135	1.046	1.008	0.987	1.026	Q7L273	KCTD9	BTB/POZ domain-containing protein KCTD9
1.011	1.012	0.991	1.014	1.173	1.020	1.035	0.940	0.999	1.047	0.982	1.038	X6R4W8	ZNF207	BUB3-interacting and GLEBS motif-containing protein ZNF207
1.004	0.961	0.988	1.032	0.816	1.102	1.030	1.018	1.007	1.086	0.988	1.022	Q9BRD0	BUD13	BUD13 homolog
1.015	0.989	1.014	0.985	0.982	1.016	1.030	1.076	1.043	1.041	1.051	1.017	Q13895	BYSL	Bystin
1.039	1.006	1.067	0.931	0.895	0.949	1.005	1.156	0.949	1.149	1.061	1.130	Q96EU7	C1GALT1C1	C1GALT1-specific chaperone 1
0.977	1.007	0.988	0.986	0.994	0.995	0.980	1.016	0.991	0.973	0.983	0.989	P11586	MTHFD1	C-1-tetrahydrofolate synthase, cytoplasmic
0.934	0.755	1.029	1.161	1.011	0.965				0.961	0.956	1.233	Q8TF44	C2CD4C	C2 calcium-dependent domain-containing protein 4C
0.856	0.983	0.956	0.975	0.928	1.135	0.936	1.046	1.037	0.995	1.109	0.949	Q9Y426	C2CD2	C2 domain-containing protein 2
0.764	1.054	0.973										Q4AC94	C2CD3	C2 domain-containing protein 3
0.930	0.987	0.977	0.973	0.899	1.018	1.014	1.105	1.020	1.063	1.048	1.019	O75844	ZMPSTE24	CAAX prenyl protease 1 homolog
						1.411	1.051	1.147	1.015	0.943	1.076	Q9Y256	RCE1	CAAX prenyl protease 2
0.973	0.997	1.008	0.995	0.961	0.994	0.957	0.994	1.010	1.027	1.009	0.984	P27708	CAD	CAD protein
0.785	1.035	1.235	0.896	0.840	1.176				0.913	1.000	1.014	Q9NYQ6	CELSR1	Cadherin EGF LAG seven-pass G-type receptor 1
1.053	0.955	1.011	1.049	0.991	0.997	1.199	0.967	1.006	0.991	0.977	1.030	P55283	CDH4	Cadherin-4

Abundance Ratio														
1 d M1/C1	1 d M2/C2	1 d M3/C3	4 d M1/C1	4 d M2/C2	4 d M3/C3	7 d M1/C1	7 d M2/C2	7 d M3/C3	10 d M1/C1	10 d M2/C2	10 d M3/C3	Accession ID	Gene name	Protein Description
1.011	0.961	1.015	1.043	0.961	1.016	0.986	0.990	1.010	1.020	1.002	1.082	Q99653	CHP1	Calcineurin B homologous protein 1
0.979	0.995	1.001	0.968	0.951	0.999	1.019	1.067	1.003	0.957	0.971	0.962	D3YTA9	PPP3R1	Calcineurin subunit B type 1
1.017	0.949	0.962	1.050	0.751	0.850							Q9Y6J0	CABIN1	Calcineurin-binding protein cabin-1
0.945	1.025	1.041				0.895	1.299	0.997				Q9UKA8	RCAN3	Calcipressin-3
0.932	0.897	0.950	0.825	0.971	1.055	0.792	0.987	0.809				Q9UGQ2	CACFD1	Calcium channel flower homolog
0.958	0.970	0.970	1.005	0.982	1.025	1.006	0.988	1.006	0.967	0.975	0.974	J3QK89	CHERP	Calcium homeostasis endoplasmic reticulum protein
			1.040	0.963	1.040	1.046	0.937	1.275	1.174	1.232	0.969	Q8IWX8	CHERP	Calcium homeostasis endoplasmic reticulum protein
0.963	0.921	0.944	1.005	1.030	1.006	1.208	1.004	0.976	1.031	1.066	1.010	J9JIE6	TMCO1	Calcium load-activated calcium channel
0.910	0.932	0.990	0.925	0.851	1.042	0.967	1.056	1.080	1.017	0.907	1.122	Q96D31	ORAI1	Calcium release-activated calcium channel protein 1
1.065	0.980	1.028	1.019	0.872	0.924	0.921	1.044	0.996	1.028	1.065	1.026	P49069	CAMLG	Calcium signal-modulating cyclophilin ligand
1.001	0.978	1.035	0.977	0.957	1.015	1.028	1.014	0.998	1.028	1.003	1.003	Q8NE86	MCU	Calcium uniporter protein, mitochondrial
0.983	0.988	1.004	1.000	0.899	0.992	1.012	1.101	0.980	1.052	1.014	1.040	Q9NWR8	MCUB	Calcium uniporter regulatory subunit MCUb, mitochondrial
0.994	1.004	1.013	0.947	0.957	0.996	1.052	1.037	1.030	1.088	0.948	1.087	A0A0U1RRK1	MICU1	Calcium uptake protein 1, mitochondrial
0.993	0.989	1.002	0.975	0.987	0.983	1.049	0.956	1.012	1.094	0.991	1.046	Q8IYU8	MICU2	Calcium uptake protein 2, mitochondrial
1.010	1.015	1.063	1.024	1.020	1.050	1.046	0.914	1.049	0.902	1.065	1.064	Q86XE3	MICU3	Calcium uptake protein 3, mitochondrial
			0.927	0.976	1.265							J3KPJ3	CAMKK1	Calcium/calmodulin-dependent protein kinase kinase 1
1.023	0.993	1.042	1.014	0.980	1.002	1.002	1.020	0.998	1.106	0.991	1.010	Q96RR4	CAMKK2	Calcium/calmodulin-dependent protein kinase kinase 2
1.005	0.986	0.980	0.953	0.906	0.950	0.979	1.059	0.988	0.984	0.956	1.059	Q14012	CAMK1	Calcium/calmodulin-dependent protein kinase type 1
1.015	1.012	0.975	1.007	0.968	1.006	0.982	1.029	0.977	0.999	0.985	0.975	E9PBG7	CAMK2D	Calcium/calmodulin-dependent protein kinase type II subunit delta
0.912	0.954	1.020	0.965	0.924	1.030	0.824	0.620	0.680	0.971	0.717	1.016	Q16566	CAMK4	Calcium/calmodulin-dependent protein kinase type IV
1.015	1.003	1.062	1.028	0.972	1.047	0.953	0.948	1.049				Q9P1Z2	CALCOCO1	Calcium-binding and coiled-coil domain-containing protein 1
1.012	1.001	1.022	0.993	0.975	1.004	1.041	1.053	1.037	1.024	0.990	1.001	O75746	SLC25A12	Calcium-binding mitochondrial carrier protein Aralar1
0.985	0.987	0.957	1.009	1.005	1.008	1.025	1.034	0.996	1.070	0.989	1.002	Q6NUK1	SLC25A24	Calcium-binding mitochondrial carrier protein SCaMC-1
0.997	1.054	1.081	0.971	0.907	1.028	0.978	1.006	0.993	0.999	0.964	1.115	Q9BV35	SLC25A23	Calcium-binding mitochondrial carrier protein SCaMC-3
1.011	1.002	0.992	0.994	0.972	1.034	1.024	1.037	1.001	1.007	0.951	1.055	Q9Y376	CAB39	Calcium-binding protein 39

Abundance Ratio														
1 d M1/C1	1 d M2/C2	1 d M3/C3	4 d M1/C1	4 d M2/C2	4 d M3/C3	7 d M1/C1	7 d M2/C2	7 d M3/C3	10 d M1/C1	10 d M2/C2	10 d M3/C3	Accession ID	Gene name	Protein Description
1.066	1.027	1.102	1.034	1.009	0.985	1.056	0.983	0.871	0.941	1.017	0.973	Q9H9S4	CAB39L	Calcium-binding protein 39-like
1.067	1.072	1.027	1.105	1.114	1.073	1.134	1.164	0.985	1.080	0.849	0.964	A0A087X1P3	CADPS2	Calcium-dependent secretion activator 2
1.053	1.041	1.094	1.027	0.969	1.003	1.043	1.102	0.974	1.072	0.994	1.007	Q9NP80	PNPLA8	Calcium-independent phospholipase A2-gamma
1.024	0.995	0.968	0.972	0.967	0.960	0.989	0.950	0.964	1.008	0.935	0.993	Q9Y2V2	CARHSP1	Calcium-regulated heat-stable protein 1
			0.914	0.954	1.079	0.994	0.910	1.038	1.099	1.030	1.102	O75177	SS18L1	Calcium-responsive transactivator
0.958	0.999	0.946	0.993	1.009	0.956	0.995	1.030	0.968	0.920	0.983	0.960	Q9HB71	CACYBP	Calcyclin-binding protein
			1.034	1.100	1.166	1.065	1.190	1.159	0.972	0.980	1.024	Q96CP7	TLCD1	Calfacilitin
0.938	1.049	0.993	1.070	0.912	1.007	1.143	0.887	1.190	1.001	0.911	1.058	O14967	CLGN	Calmegin
1.045	0.954	1.072	1.024	0.960	1.025	1.000	0.992	0.990	1.000	1.021	1.107	Q96JQ2	CLMN	Calmin
0.979	1.005	0.970	0.986	0.975	1.002	0.990	0.981	0.998	1.038	0.981	1.050	E7EMB3	CALM2	Calmodulin
0.973	0.976	0.966	0.816	0.624	0.979	1.222	1.081	1.120				P27482	CALML3	Calmodulin-like protein 3
									0.845	0.946	0.988	Q9NZT1	CALML5	Calmodulin-like protein 5
1.030	0.894	0.974	1.096	1.239	0.866				0.971	1.238	1.057	Q7Z624	CAMKMT	Calmodulin-lysine N-methyltransferase
1.025	1.042	1.022	1.010	0.940	1.007	1.003	1.022	1.001	0.962	0.960	0.996	Q08AD1	CAMSAP2	Calmodulin-regulated spectrin-associated protein 2
2.036	0.984	1.752	0.975	0.572	1.182	0.495	0.767	0.789	0.283	0.793	0.248	D6RB85	CANX	Calnexin (Fragment)
0.797	1.037	0.867	1.376	0.861	1.271	1.223	1.047	1.023	1.047	1.004	1.171	K7ES78	CAPNS1	Calpain small subunit 1 (Fragment)
0.955	0.989	0.972	1.014	0.994	0.964	0.950	1.010	0.958	0.961	0.992	0.955	A0A0C4DGQ5	CAPNS1	Calpain small subunit 1
0.990	0.991	1.008	0.993	0.966	0.997	0.974	0.959	0.992	0.989	0.981	1.004	P07384	CAPN1	Calpain-1 catalytic subunit
1.009	0.919	1.005	0.971	0.891	1.075	0.980	1.060	0.886	0.990	0.954	1.098	O75808	CAPN15	Calpain-15
1.008	1.005	0.994	1.007	1.051	1.007	1.002	1.011	1.061	0.971	0.973	1.005	P17655	CAPN2	Calpain-2 catalytic subunit
0.947	0.960	1.021	0.969	0.889	1.002	1.061	0.965	0.953	1.024	0.975	0.999	E7EV01	CAPN5	Calpain-5
1.049	1.010	1.003	1.004	0.995	1.014	1.009	1.046	0.985	1.060	1.037	1.067	Q9Y6W3	CAPN7	Calpain-7
0.969	1.013	0.906	0.974	0.990	0.911	1.112	1.215	0.983	1.136	1.080	1.228	D6RBR1	CAST	Calpastatin (Fragment)
			0.952	0.889	0.892				1.082	1.050	0.986	E7EVY3	CAST	Calpastatin
0.979	0.993	0.958	1.011	1.080	0.981	1.027	1.008	0.973	1.014	0.985	0.982	B4DUT8	CNN2	Calponin
0.951	0.995	0.944	0.999	1.064	0.977	1.030	0.993	0.987	1.023	1.001	0.976	Q15417	CNN3	Calponin-3
1.026	1.032	1.003	0.988	1.053	0.957	0.993	0.991	0.969	0.990	1.009	0.991	P27797	CALR	Calreticulin
1.174	1.124	0.829							0.973	0.991	0.828	O94985	CLSTN1	Calsyntenin-1
1.000	0.966	1.013	0.956	0.935	1.022	0.967	0.970	0.954	0.988	0.979	0.985	P17612	PRKACA	cAMP-dependent protein kinase catalytic subunit alpha
1.066	0.997	0.982	0.982	0.955	0.967	0.995	0.998	0.953	1.076	1.011	1.019	P22694	PRKACB	cAMP-dependent protein kinase catalytic subunit beta
1.030	1.211	0.780							1.203	0.980	1.083	P51817	PRKX	cAMP-dependent protein kinase catalytic subunit PRKX

Abundance Ratio														
1 d M1/C1	1 d M2/C2	1 d M3/C3	4 d M1/C1	4 d M2/C2	4 d M3/C3	7 d M1/C1	7 d M2/C2	7 d M3/C3	10 d M1/C1	10 d M2/C2	10 d M3/C3	Accession ID	Gene name	Protein Description
0.947	0.997	1.006	0.908	0.990	0.994							P61925	PKIA	cAMP-dependent protein kinase inhibitor alpha
0.746	0.801	1.121	0.808	0.886	0.964							Q9Y2B9	PKIG	cAMP-dependent protein kinase inhibitor gamma
1.011	1.031	1.037	1.011	0.999	1.021	1.019	1.064	1.011	1.017	1.014	1.062	P10644	PRKAR1A	cAMP-dependent protein kinase type I-alpha regulatory subunit
0.937	0.888	1.111	0.936	0.907	0.993	0.950	1.041	0.946	0.985	1.056	0.935	P31321	PRKAR1B	cAMP-dependent protein kinase type I-beta regulatory subunit
1.006	0.990	1.004	1.004	0.980	1.023	1.001	1.003	0.989	0.991	1.003	0.998	P13861	PRKAR2A	cAMP-dependent protein kinase type II-alpha regulatory subunit
1.013	0.979	0.965	0.953	0.975	1.041	1.061	0.963	0.957	1.059	0.958	1.036	H3BMD8	ARPP19	cAMP-regulated phosphoprotein 19
1.002	0.985	0.994	1.027	0.961	1.020	0.986	1.104	1.025	0.989	1.003	0.985	Q5TDF0	NTPCR	Cancer-related nucleoside-triphosphatase
0.975	1.004	1.014	1.012	0.966	1.004	0.999	0.963	1.002	1.055	1.002	1.038	Q9UDT6	CLIP2	CAP-Gly domain-containing linker protein 2
0.956	1.011	0.967	0.997	0.997	1.000	1.021	0.993	0.991	1.012	0.997	1.019	B1AK88	CAPZB	Capping protein (Actin filament) muscle Z-line, beta, isoform CRA_d
1.027	1.049	1.002	0.908	1.052	1.091	0.911	1.068	1.112	0.960	0.958	1.051	Q6F5E8	CARMIL2	Capping protein, Arp2/3 and myosin-I linker protein 2
0.968	1.013	0.963	1.015	0.986	1.010	1.020	1.018	1.023	0.989	1.037	0.965	Q14444	CAPRIN1	Caprin-1
						1.092	1.065	1.074	0.943	0.828	1.093	Q6IMN6	CAPRIN2	Caprin-2
0.977	0.986	1.025	0.985	0.951	1.011	0.999	1.034	1.047	1.012	0.996	1.039	Q8N1G2	CMTR1	Cap-specific mRNA (nucleoside-2'-O-)-methyltransferase 1
0.935	1.010	0.981	0.934	0.994	0.985	1.025	1.119	1.074	1.009	1.023	1.085	A0A0U1RRB3	CMTR2	Cap-specific mRNA (nucleoside-2'-O-)-methyltransferase 2
1.062	0.979	1.025										A8MPS7	YDJC	Carbohydrate deacetylase
1.006	1.063	1.284	0.911	0.765	1.009	0.907	1.025	1.052	0.949	1.063	1.159	Q9NPF2	CHST11	Carbohydrate sulfotransferase 11
									1.072	0.921	1.121	Q9NRB3	CHST12	Carbohydrate sulfotransferase 12
1.018	0.953	1.035	0.958	0.985	1.068							Q8NCH0	CHST14	Carbohydrate sulfotransferase 14
0.897	1.060	0.862	0.959	1.022	0.980	0.923	1.065	1.007				Q7LGC8	CHST3	Carbohydrate sulfotransferase 3
1.001	0.870	1.014	0.979	0.910	1.030	1.043	0.921	1.046	1.085	1.011	1.228	Q9NS84	CHST7	Carbohydrate sulfotransferase 7
1.057	1.027	1.027	1.033	0.999	1.043	1.041	0.991	0.999	1.094	1.005	1.012	Q8N1Q1	CA13	Carbonic anhydrase 13
0.956	0.997	1.005	0.947	0.911	0.936	0.944	0.991	0.985	0.950	0.892	1.044	P00918	CA2	Carbonic anhydrase 2
0.995	0.999	0.994	1.000	1.075	0.987	1.007	1.017	1.062	0.984	1.006	0.964	P16152	CBR1	Carbonyl reductase [NADPH] 1
1.053	0.950	1.000	1.064	1.002	1.009	0.949	1.111	1.003	1.018	0.950	1.050	O75828	CBR3	Carbonyl reductase [NADPH] 3
			1.004	0.915	0.915	1.011	1.055	1.025	0.907	0.461	1.009	A0A024R6X1	CES2	Carboxylic ester hydrolase
			0.967	0.973	1.015	1.068	0.922	1.052	1.031	0.946	1.126	O75052	NOS1AP	Carboxyl-terminal PDZ ligand of neuronal nitric oxide synthase protein
1.001	0.977	0.975	0.982	0.968	0.992	0.948	0.965	0.982	1.030	0.986	0.995	Q96DG6	CMBL	Carboxymethylenebutenolidase homolog
1.066	1.035	1.080	1.060	1.005	1.056	1.040	1.038	1.066	1.140	0.946	1.076	O75976	CPD	Carboxypeptidase D
0.994	1.037	0.946	1.081	1.088	1.005	1.090	1.039	1.004	1.128	1.065	0.990	X6R8A1	CTSA	Carboxypeptidase

Abundance Ratio														
1 d M1/C1	1 d M2/C2	1 d M3/C3	4 d M1/C1	4 d M2/C2	4 d M3/C3	7 d M1/C1	7 d M2/C2	7 d M3/C3	10 d M1/C1	10 d M2/C2	10 d M3/C3	Accession ID	Gene name	Protein Description
1.037	0.828	1.035	1.232	1.174	1.156	1.093	1.111	1.098	1.318	1.211	1.402	Q9Y646	CPQ	Carboxypeptidase Q
0.999	1.001	1.041	0.983	0.873	1.091	0.983	1.113	1.118	1.025	1.044	1.115	H7C1Z7	CTDSP1	Carboxy-terminal domain RNA polymerase II polypeptide A small phosphatase 1 (Fragment)
						1.043	1.303	0.931				P13688	CEACAM1	Carcinoembryonic antigen-related cell adhesion molecule 1
1.011	1.000	1.021	0.975	0.966	1.006	1.009	1.016	0.983	1.037	0.977	1.012	P43155	CRAT	Carnitine O-acetyltransferase
1.004	1.005	1.019	1.010	0.963	0.983	0.987	1.043	0.997	0.971	1.011	0.961	P50416	CPT1A	Carnitine O-palmitoyltransferase 1, liver isoform
0.983	0.996	1.027	1.017	1.027	1.034	1.002	0.946	1.004	1.056	0.979	1.061	P23786	CPT2	Carnitine O-palmitoyltransferase 2, mitochondrial
1.094	1.077	1.076	0.979	0.919	1.021	0.972	1.083	1.103	0.985	1.059	1.075	Q8N4J0	CARNMT1	Carnosine N-methyltransferase
1.050	1.006	0.987	1.012	0.980	0.981	1.014	1.073	1.013	0.997	0.932	0.982	O75718	CRTAP	Cartilage-associated protein
1.031	1.024	1.014	1.013	0.997	0.986	1.053	1.088	1.045	1.023	0.944	1.052	P48729	CSNK1A1	Casein kinase I isoform alpha
1.011	0.985	0.989	1.033	0.978	1.041	0.993	0.971	1.012	1.016	0.962	1.053	H7BYT1	CSNK1D	Casein kinase I isoform delta
0.994	1.057	0.929	0.892	0.949	0.926	1.176	0.877	0.766	1.122	1.054	1.000	P49674	CSNK1E	Casein kinase I isoform epsilon
1.286	1.351	0.993										P78368	CSNK1G2	Casein kinase I isoform gamma-2
1.036	0.988	1.007	1.059	0.963	0.993	1.034	1.039	0.994	1.001	0.986	1.013	P68400	CSNK2A1	Casein kinase II subunit alpha
1.015	1.005	1.015	1.004	1.061	1.029	1.042	1.147	1.049	1.022	0.999	1.019	P19784	CSNK2A2	Casein kinase II subunit alpha'
1.069	1.063	1.016	1.036	1.280	0.918	1.044	0.960	1.036	1.014	1.048	0.974	Q5SRQ6	CSNK2B	Casein kinase II subunit beta
0.951	0.926	1.027	0.989	0.965	1.047	0.989	0.991	1.021	0.999	0.903	1.142	Q8WXE0	CASKIN2	Caskin-2
1.056	0.966	1.062	0.868	0.842	0.965	0.868	0.773	0.969				O15519	CFLAR	CASP8 and FADD-like apoptosis regulator
1.061	1.061	1.008	0.992	0.940	1.085	1.082	1.093	1.078	1.097	1.022	1.061	Q9H8G2	CAAP1	Caspase activity and apoptosis inhibitor 1
1.180	0.906	1.135										Q9BWT7	CARD10	Caspase recruitment domain-containing protein 10
1.012	1.031	1.036	1.043	1.083	1.016	1.064	1.031	1.055	0.954	0.949	1.046	P29466	CASP1	Caspase-1
0.974	1.011	0.999	0.969	0.994	0.961	1.005	0.974	1.004	0.958	1.005	1.024	P42575	CASP2	Caspase-2
0.882	0.952	1.042	0.963	1.011	1.029	0.995	0.896	1.033	0.967	0.977	1.074	P42574	CASP3	Caspase-3
1.043	1.024	1.082	1.010	1.113	0.969	1.015	1.004	1.041	1.047	0.928	1.010	P49662	CASP4	Caspase-4
0.992	0.965	0.959	0.971	0.953	0.999	0.968	1.030	0.983	0.961	0.994	0.984	P55212	CASP6	Caspase-6
1.004	1.008	1.075	1.027	0.986	1.023				0.992	1.019	1.061	P55211	CASP9	Caspase-9
1.056	0.996	1.020	1.020	0.991	0.999	1.029	1.021	0.989	1.019	1.034	0.979	P04040	CAT	Catalase
0.910	1.015	1.010	1.043	1.001	1.064	1.050	1.109	1.042	0.981	0.995	1.031	Q86VU5	COMTD1	Catechol O-methyltransferase domain-containing protein 1
0.945	1.009	0.964	0.969	0.941	0.982	1.010	0.998	1.030	1.009	0.988	1.001	P21964	COMT	Catechol O-methyltransferase
0.991	0.983	1.007	0.994	0.954	1.011	1.031	1.022	1.005	0.976	0.975	1.067	P35221	CTNNA1	Catenin alpha-1
0.935	0.995	1.025	0.933	1.109	1.160				0.838	1.068	0.938	P26232	CTNNA2	Catenin alpha-2
1.032	0.994	1.022	1.034	0.915	1.016	1.056	1.056	0.995	1.094	0.928	1.101	P35222	CTNNB1	Catenin beta-1

Abundance Ratio														
1 d M1/C1	1 d M2/C2	1 d M3/C3	4 d M1/C1	4 d M2/C2	4 d M3/C3	7 d M1/C1	7 d M2/C2	7 d M3/C3	10 d M1/C1	10 d M2/C2	10 d M3/C3	Accession ID	Gene name	Protein Description
1.058	0.851	1.197	0.933	1.110	1.012							Q9UQB3	CTNND2	Catenin delta-2
			1.078	1.061	1.127	0.987	1.397	0.828	1.235	1.107	1.562	J3KNB4	CAMP	Cathelicidin antimicrobial peptide
1.091	1.014	0.992	1.047	1.165	0.960	1.059	0.944	1.045	1.050	0.966	0.978	P07858	CTSB	Cathepsin B
1.044	1.014	0.991	1.096	1.119	1.108	1.152	1.154	1.131	1.186	1.165	1.149	P07339	CTSD	Cathepsin D
0.927	0.964	0.943	1.088	1.022	1.060	1.150	1.122	1.051	1.173	1.048	1.144	P07711	CTSL	Cathepsin L1
1.047	1.000	0.935	1.035	1.001	0.973	1.107	0.966	0.931	1.181	1.097	1.047	Q9UBR2	CTSZ	Cathepsin Z
1.049	1.014	1.037	1.082	1.234	1.031	1.102	0.906	1.095	1.029	1.011	1.023	P20645	M6PR	Cation-dependent mannose-6-phosphate receptor
0.966	1.002	0.994	1.030	0.974	1.034	1.024	1.036	1.023	1.032	1.041	1.013	P11717	IGF2R	Cation-independent mannose-6-phosphate receptor
1.001	1.005	1.017	1.016	0.876	0.978	1.108	1.068	0.964	1.107	0.946	1.001	Q9NQ11	ATP13A2	Cation-transporting ATPase 13A2
0.933	1.029	1.000	1.001	0.917	0.996	1.039	1.001	1.001	1.196	1.001	1.057	Q03135	CAV1	Caveolin-1
0.877	1.009	1.008	0.951	0.875	1.060	1.071	1.014	1.067	1.141	1.022	1.115	P51636	CAV2	Caveolin-2
			0.895	0.942	1.046	1.096	1.224	1.036	1.146	1.085	1.109	Q96RK1	CITED4	Cbp/p300-interacting transactivator 4
						1.238	0.851	1.123	1.003	1.126	1.083	O43310	CTIF	CBP80/20-dependent translation initiation factor
0.933	1.058	1.160	1.141	1.376	1.200	1.079	1.123	1.086	1.002	1.082	1.009	P78556	CCL20	C-C motif chemokine 20
1.014	0.994	1.031	1.018	0.982	1.019	1.018	0.998	1.004	1.036	0.980	1.027	Q96Q11	TRNT1	CCA tRNA nucleotidyltransferase 1, mitochondrial
1.112	1.095	1.103	1.018	1.041	1.022	0.979	0.967	0.981	0.976	1.082	1.037	P17676	CEBPB	CCAAT/enhancer-binding protein beta
1.063	1.029	1.132	0.943	1.646	1.127							P49716	CEBPD	CCAAT/enhancer-binding protein delta
1.026	1.003	1.022	1.007	0.994	1.000	1.008	1.057	1.041	1.018	1.036	1.024	Q03701	CEBPZ	CCAAT/enhancer-binding protein zeta
0.987	0.996	1.016	0.988	0.957	1.020	0.982	1.051	1.024	0.982	0.996	1.006	A5YKK6	CNOT1	CCR4-NOT transcription complex subunit 1
1.036	0.994	1.019	0.969	0.917	1.006	1.000	1.063	1.016	0.951	0.956	1.064	Q9UKZ1	CNOT11	CCR4-NOT transcription complex subunit 11
0.963	0.992	1.008	0.962	1.034	0.986	0.960	0.859	0.971	0.987	1.044	1.065	Q9NZN8	CNOT2	CCR4-NOT transcription complex subunit 2
1.025	1.013	0.997	0.997	0.953	1.016	0.990	0.963	1.037	1.052	1.032	1.028	O75175	CNOT3	CCR4-NOT transcription complex subunit 3
0.949	1.256	1.012							0.800	1.237	0.909	Q9ULM6	CNOT6	CCR4-NOT transcription complex subunit 6
1.080	1.009	1.023	0.982	1.076	1.026	1.112	1.181	1.041	0.892	0.910	1.069	H0Y9Z5	CNOT6L	CCR4-NOT transcription complex subunit 6-like (Fragment)
1.067	1.036	0.977	0.945	0.880	0.980	0.974	1.126	1.033	0.987	0.955	0.959	Q9UIV1	CNOT7	CCR4-NOT transcription complex subunit 7
0.972	1.001	0.967	0.981	0.899	1.006	1.002	1.076	0.993	1.020	1.043	1.024	Q9UFF9	CNOT8	CCR4-NOT transcription complex subunit 8
1.029	1.005	1.004	0.990	0.934	1.027	1.038	1.099	1.033	0.987	0.998	0.988	Q92600	CNOT9	CCR4-NOT transcription complex subunit 9
1.008	1.018	1.004	1.050	1.026	1.035	1.044	1.096	1.053	0.995	0.960	1.074	Q6YHK3	CD109	CD109 antigen
1.162	0.957	1.009	1.025	1.224	1.151	1.293	0.789	1.296	1.205	1.076	1.168	P48509	CD151	CD151 antigen
0.989	1.021	0.986	1.039	0.942	1.004	1.037	0.954	0.992	1.074	1.028	1.060	Q13740	ALCAM	CD166 antigen
0.998	0.994	1.028	0.986	0.962	1.021	1.008	1.003	1.006	1.019	1.031	1.021	O95400	CD2BP2	CD2 antigen cytoplasmic tail-binding protein 2
0.995	0.953	0.962	1.062	0.951	1.098				1.028	1.034	1.149	Q5ZPR3	CD276	CD276 antigen
1.003	1.000	0.978	1.000	0.984	0.993	0.993	1.009	0.994	1.003	1.000	1.018	Q9Y5K6	CD2AP	CD2-associated protein

Abundance Ratio														
1 d M1/C1	1 d M2/C2	1 d M3/C3	4 d M1/C1	4 d M2/C2	4 d M3/C3	7 d M1/C1	7 d M2/C2	7 d M3/C3	10 d M1/C1	10 d M2/C2	10 d M3/C3	Accession ID	Gene name	Protein Description
									0.874	0.993	1.153	Q9NPF0	CD320	CD320 antigen
0.903	1.023	0.988	1.045	0.974	1.024	1.133	1.063	1.004	1.092	0.963	1.144	P16070	CD44	CD44 antigen
0.869	0.984	0.863	1.045	0.987	1.017	1.190	0.986	1.045	1.126	0.995	1.063	E9PR17	CD59	CD59 glycoprotein
0.815	1.049	0.926	1.119	0.979	1.198	1.288	1.145	1.057	1.306	1.267	1.130	P08962	CD63	CD63 antigen
0.973	1.006	1.024	0.960	0.905	1.024	0.969	1.104	0.986	0.984	0.965	1.040	P32970	CD70	CD70 antigen
						1.082	1.140	1.083				P27701	CD82	CD82 antigen
0.923	0.989	1.018	1.041	1.013	1.067	1.097	0.974	0.919	1.139	0.994	1.117	P48960	CD97	CD97 antigen
0.877	0.955	1.025	1.203	0.703	0.955	1.063	0.951	1.022	1.126	1.163	1.021	H7C2F2	CD99	CD99 antigen (Fragment)
1.012	1.037	1.118	0.968	0.935	1.190	1.131	0.988	0.988	1.207	1.101	1.168	P14209	CD99	CD99 antigen
1.073	0.967	1.024	1.001	0.968	0.983	1.005	1.022	0.991	1.025	1.006	1.024	Q00587	CDC42EP1	Cdc42 effector protein 1
0.962	0.931	1.189	0.909	0.995	0.956	0.963	1.234	0.952	1.090	0.959	1.295	O14613	CDC42EP2	Cdc42 effector protein 2
0.961	0.973	1.010	1.033	0.945	0.984	0.990	0.968	0.999	1.117	0.960	1.134	Q9H3Q1	CDC42EP4	Cdc42 effector protein 4
			0.990	0.790	0.913	1.209	1.077	0.978				Q9NRR3	CDC42SE2	CDC42 small effector protein 2
			0.985	1.161	0.999							Q15642	TRIP10	Cdc42-interacting protein 4
1.043	1.090	1.072	1.148	1.258	1.067	1.217	1.077	1.156	1.230	1.137	1.174	Q9NZ45	CISD1	CDGSH iron-sulfur domain-containing protein 1
1.019	1.031	1.033	1.074	1.091	1.053	1.185	1.140	1.112	1.124	0.937	1.087	Q8N5K1	CISD2	CDGSH iron-sulfur domain-containing protein 2
1.013	1.004	0.964	1.074	1.044	1.059	1.068	1.070	1.067	1.257	1.119	1.191	P0C7P0	CISD3	CDGSH iron-sulfur domain-containing protein 3, mitochondrial
1.132	0.997	1.110	1.045	1.053	0.950				1.117	1.104	1.120	Q86Y37	CACUL1	CDK2-associated and cullin domain-containing protein 1
									1.043	1.080	1.068	Q9BTV7	CABLES2	CDK5 and ABL1 enzyme substrate 2
0.969	0.912	1.060	1.039	1.039	0.976	1.036	1.021	1.011	0.993	1.035	1.040	Q96SN8	CDK5RAP2	CDK5 regulatory subunit-associated protein 2
1.014	0.946	1.027	0.957	0.914	1.080	1.044	1.033	1.004	1.043	1.008	1.083	P51948	MNAT1	CDK-activating kinase assembly factor MAT1
0.968	1.000	1.008	0.975	0.978	0.989	0.994	0.909	0.982	1.054	1.012	1.066	Q9NXV6	CDKN2AIP	CDKN2A-interacting protein
1.018	0.944	0.996	0.960	0.957	1.066	0.935	0.881	0.995	1.005	1.119	1.086	Q96HQ2	CDKN2AIPNL	CDKN2AIP N-terminal-like protein
						1.103	1.363	1.422	0.976	0.915	1.072	B4E1Z4	1 SV	cDNA FLJ55673, highly similar to Complement factor B (EC 3.4.21.47)
1.119	1.098	1.000	1.042	0.976	0.999	1.061	1.232	0.987	1.051	1.046	1.078	Q32NB8	PGS1	CDP-diacylglycerol--glycerol-3-phosphate 3-phosphatidyltransferase, mitochondrial
0.823	0.978	0.969	0.874	0.877	1.112	0.982	0.957	1.087	1.232	0.977	1.128	O14735	CDIPT	CDP-diacylglycerol--inositol 3-phosphatidyltransferase
0.994	0.925	1.119										Q8NFZ8	CADM4	Cell adhesion molecule 4
0.980	1.039	0.993	0.960	0.990	0.995	1.008	1.107	0.994	1.010	0.997	1.015	E5RFJ3	CCAR2	Cell cycle and apoptosis regulator protein 2 (Fragment)
1.009	1.005	1.004	0.974	0.980	0.991	0.993	0.994	1.007	0.988	0.989	1.009	Q8N163	CCAR2	Cell cycle and apoptosis regulator protein 2
1.001	0.939	1.194	1.009	0.949	1.009	0.963	1.024	1.185	0.980	0.995	1.012	Q99638	RAD9A	Cell cycle checkpoint control protein RAD9A

Abundance Ratio														
1 d M1/C1	1 d M2/C2	1 d M3/C3	4 d M1/C1	4 d M2/C2	4 d M3/C3	7 d M1/C1	7 d M2/C2	7 d M3/C3	10 d M1/C1	10 d M2/C2	10 d M3/C3	Accession ID	Gene name	Protein Description
1.053	0.969	1.055	1.222	0.994	1.049	1.028	0.965	0.962	0.973	0.985	1.097	O60671	RAD1	Cell cycle checkpoint protein RAD1
1.029	1.048	0.964	0.764	0.961	0.970	1.019	1.180	1.066	0.951	0.940	1.002	A0A0G2JPT5	RAD17	Cell cycle checkpoint protein RAD17
1.009	1.013	1.051	1.009	0.965	1.053	1.034	1.099	1.059	1.063	1.027	1.075	Q9NV96	TMEM30A	Cell cycle control protein 50A
1.031	1.013	1.075	1.025	0.963	1.051	0.992	1.022	1.043	1.053	1.002	1.115	Q9NQS1	AVEN	Cell death regulator Aven
1.083	0.979	1.007	1.027	1.156	0.999	1.052	0.944	1.004	1.044	1.048	0.961	P60953	CDC42	Cell division control protein 42 homolog
1.005	0.987	1.008	1.003	1.011	0.998	1.013	1.038	1.025	0.987	1.009	1.021	Q99459	CDC5L	Cell division cycle 5-like protein
0.806	1.141	0.932										O00311	CDC7	Cell division cycle 7-related protein kinase
1.004	0.995	1.006	0.982	0.994	0.994	0.985	0.974	0.965	1.033	1.037	1.060	Q8IX12	CCAR1	Cell division cycle and apoptosis regulator protein 1
1.057	1.022	0.976	1.036	1.034	1.118	1.058	1.115	1.068	0.945	1.008	1.032	O75794	CDC123	Cell division cycle protein 123 homolog
1.036	0.971	1.011	1.002	0.967	1.014	1.014	0.969	1.005	1.160	0.913	1.067	Q13042	CDC16	Cell division cycle protein 16 homolog
0.937	0.956	0.880	0.921	0.896	1.026	0.998	1.126	0.934	1.036	0.956	0.980	Q86Y33	CDC20B	Cell division cycle protein 20 homolog B
1.002	0.994	1.080	1.235	1.185	0.899	0.924	1.242	1.077	1.058	0.661	1.528	Q12834	CDC20	Cell division cycle protein 20 homolog
1.048	1.012	1.013	1.017	0.967	1.005	0.983	1.107	0.992	0.983	0.988	0.966	Q9UJX2	CDC23	Cell division cycle protein 23 homolog
0.898	0.959	1.038										Q69YH5	CDCA2	Cell division cycle-associated protein 2
0.951	1.040	1.057	1.090	1.107	0.988	1.290	0.958	0.943				Q99618	CDCA3	Cell division cycle-associated protein 3
1.270	0.913	1.073										Q99675	CGRRF1	Cell growth regulator with RING finger domain protein 1
0.987	0.978	1.018	0.970	0.972	1.027	0.962	0.988	0.957	1.017	0.993	1.051	Q9NX58	LYAR	Cell growth-regulating nucleolar protein
0.962	0.979	0.990	1.000	0.952	0.982	1.004	0.935	1.006	1.021	1.002	1.038	P43121	MCAM	Cell surface glycoprotein MUC18
1.035	1.075	1.177	1.156	1.809	1.045	1.148	0.555	1.072	1.043	1.052	1.045	P62633	CNBP	Cellular nucleic acid-binding protein
0.907	0.961	1.040	0.944	0.993	1.000	0.913	0.840	0.987	0.994	1.006	1.043	P41208	CETN2	Centrin-2
0.957	1.002	0.971	0.994	0.972	0.978	1.004	0.993	1.012	1.015	0.966	1.028	E5RJF8	CETN3	Centrin-3
1.033	0.966	1.094	0.837	0.742	1.186	0.887	1.357	1.228				O43303	CCP110	Centriolar coiled-coil protein of 110 kDa
0.866	0.857	0.691										Q7Z7A1	CNTRL	Centriolin
1.062	0.992	1.019	1.094	0.968	1.004	1.013	1.066	1.042	0.971	1.008	1.007	Q03188	CENPC	Centromere protein C
0.941	1.002	1.049	0.956	0.940	1.240	1.125	1.175	1.171	1.017	1.009	0.980	P49454	CENPF	Centromere protein F
0.979	1.028	0.995	0.915	0.898	1.025	0.968	1.210	0.981	0.997	0.825	0.994	Q9H3R5	CENPH	Centromere protein H
1.127	1.006	1.160	0.992	1.015	1.189	1.001	1.102	0.964	0.962	0.731	1.659	Q92674	CENPI	Centromere protein I
1.021	0.909	0.960	0.979	1.027	0.930							Q9BS16	CENPK	Centromere protein K
0.901	1.060	0.976	0.853	0.909	1.270	0.965	0.966	1.071	0.781	0.995	0.896	Q8N0S6	CENPL	Centromere protein L
1.080	1.040	1.285				0.916	1.210	1.073	1.023	0.962	1.122	Q9NSP4	CENPM	Centromere protein M
1.121	1.056	1.243										Q9BU64	CENPO	Centromere protein O
0.982	0.934	1.118	1.126	0.921	1.205	1.225	0.921	1.116				Q7L2Z9	CENPQ	Centromere protein Q
						1.095	0.758	0.957	1.162	0.920	1.218	Q8N2Z9	CENPS	Centromere protein S

Abundance Ratio														
1 d M1/C1	1 d M2/C2	1 d M3/C3	4 d M1/C1	4 d M2/C2	4 d M3/C3	7 d M1/C1	7 d M2/C2	7 d M3/C3	10 d M1/C1	10 d M2/C2	10 d M3/C3	Accession ID	Gene name	Protein Description
1.028	1.043	1.124	1.078	1.080	0.909	0.911	1.076	0.917	1.157	0.847	1.137	Q96BT3	CENPT	Centromere protein T
			0.954	1.085	0.877				1.233	1.052	1.171	Q71F23	CENPU	Centromere protein U
1.046	0.927	0.970	0.953	1.020	1.001	0.925	0.919	1.022	1.054	1.012	1.025	Q7Z7K6	CENPV	Centromere protein V
									0.758	0.803	1.018	A8MT69	CENPX	Centromere protein X
1.044	1.005	1.021	1.009	0.953	1.039	1.021	1.037	1.023	1.013	0.975	1.014	O43264	ZW10	Centromere/kinetochore protein zw10 homolog
0.909	0.850	0.903										Q02224	CENPE	Centromere-associated protein E
						0.906	1.133	1.071	0.884	0.778	1.454	O60308	CEP104	Centrosomal protein of 104 kDa
1.712	0.835	1.532				1.218	0.867	1.072	0.963	1.244	1.284	Q8N8E3	CEP112	Centrosomal protein of 112 kDa
2.161	0.935	1.019	0.993	0.826	1.006	0.993	1.180	1.071				Q8N960	CEP120	Centrosomal protein of 120 kDa
						0.999	0.920	0.836				Q6ZU80	CEP128	Centrosomal protein of 128 kDa
0.952	0.981	1.018	1.023	0.993	1.105	0.961	1.008	1.006	1.078	1.053	1.124	Q9UPN4	CEP131	Centrosomal protein of 131 kDa
1.085	0.955	1.110	0.820	1.063	0.932	0.949	0.986	0.981	1.016	0.792	1.140	Q66GS9	CEP135	Centrosomal protein of 135 kDa
0.936	0.921	1.121										Q5TB80	CEP162	Centrosomal protein of 162 kDa
									0.935	0.767	0.910	E7EWM2	CEP170	Centrosomal protein of 170 kDa
0.997	0.981	1.022	0.978	0.955	1.004	0.965	0.920	0.980	1.029	1.018	1.078	Q5SW79	CEP170	Centrosomal protein of 170 kDa
1.022	1.008	0.973	0.996	0.947	0.974	0.976	1.088	0.989	1.088	0.978	1.202	J3KQR7	CEP170B	Centrosomal protein of 170 kDa protein B
0.870	0.943	0.928	1.024	1.090	1.165	1.091	1.061	0.917	1.235	0.981	1.266	J3KNF5	CEP290	Centrosomal protein of 290 kDa
0.968	0.988	1.022	0.999	0.992	0.986	0.976	0.729	0.905	1.072	1.022	1.178	Q9BYV8	CEP41	Centrosomal protein of 41 kDa
1.199	1.021	0.935	1.021	0.892	1.082	0.954	0.955	1.138	0.973	0.920	1.041	Q9C0F1	CEP44	Centrosomal protein of 44 kDa
0.960	1.024	1.041	0.932	0.938	0.984	1.026	0.878	1.034	1.149	0.963	1.300	Q53EZ4	CEP55	Centrosomal protein of 55 kDa
0.958	0.871	1.053	0.908	0.950	1.067	1.085	0.884	1.106	1.075	1.060	1.461	Q86XR8	CEP57	Centrosomal protein of 57 kDa
0.920	0.904	1.068										Q76N32	CEP68	Centrosomal protein of 68 kDa
1.037	1.039	1.031	0.995	0.991	1.026	1.151	1.096	0.926	1.016	1.126	0.951	Q9P209	CEP72	Centrosomal protein of 72 kDa
1.331	1.031	1.258				0.949	0.864	0.885				Q8TAP6	CEP76	Centrosomal protein of 76 kDa
0.936	1.099	1.034				1.004	0.879	1.210	1.064	0.961	1.157	J3KNW7	CEP83	Centrosomal protein of 83 kDa
1.108	0.910	0.903	0.969	0.792	1.026	1.004	1.197	1.025	1.018	0.979	1.090	Q6P2H3	CEP85	Centrosomal protein of 85 kDa
0.909	0.898	0.817	1.142	0.832	0.864	1.273	0.894	0.912	1.539	1.363	0.998	Q96ST8	CEP89	Centrosomal protein of 89 kDa
0.883	1.012	0.964				0.965	1.020	0.963				Q5VT06	CEP350	Centrosome-associated protein 350
1.070	0.963	0.978	0.974	1.008	0.949	0.950	0.907	1.064	1.090	1.011	1.149	Q9BV73	CEP250	Centrosome-associated protein CEP250
1.041	1.023	1.017	1.094	1.022	1.092	1.078	1.213	1.055	1.003	1.076	1.051	Q16739	UGCG	Ceramide glucosyltransferase
0.968	1.000	0.991	1.041	0.937	0.999	0.982	0.966	0.999	1.088	1.083	1.078	Q96G23	CERS2	Ceramide synthase 2
						0.812	0.975	0.932				Q9HA82	CERS4	Ceramide synthase 4
1.091	1.072	1.101	0.997	0.848	1.049	1.070	1.108	1.040	1.011	1.074	1.087	Q8N5B7	CERS5	Ceramide synthase 5
1.037	0.971	0.978	1.026	0.989	1.058	1.130	1.056	0.958	1.015	1.001	0.950	Q5TA50	CPTP	Ceramide-1-phosphate transfer protein

Abundance Ratio														
1 d M1/C1	1 d M2/C2	1 d M3/C3	4 d M1/C1	4 d M2/C2	4 d M3/C3	7 d M1/C1	7 d M2/C2	7 d M3/C3	10 d M1/C1	10 d M2/C2	10 d M3/C3	Accession ID	Gene name	Protein Description
0.961	0.991	1.010	0.956	1.029	1.086	0.961	1.021	1.013	0.754	0.855	1.052	Q01850	CDR2	Cerebellar degeneration-related protein 2
0.971	1.021	0.835	0.952	0.961	0.998	1.047	1.129	0.948	1.023	0.987	1.183	Q86X02	CDR2L	Cerebellar degeneration-related protein 2-like
0.973	1.096	1.063	1.002	0.990	0.944	0.982	1.174	0.960	1.105	0.980	1.044	Q9BSQ5	CCM2	Cerebral cavernous malformations 2 protein
1.070	0.966	0.982	1.026	0.904	1.043	1.049	1.047	1.035	1.149	1.069	1.137	A0A024R644	CLN5	Ceroid-lipofuscinosis neuronal protein 5
0.972	1.020	1.100	0.990	0.838	0.837	0.961	1.132	1.001	0.859	1.133	0.838	Q9NWW5	CLN6	Ceroid-lipofuscinosis neuronal protein 6
			1.096	0.862	0.852							P00450	CP	Ceruloplasmin
1.021	0.983	1.026	0.960	0.876	1.026	0.999	0.908	1.014	1.010	0.873	1.085	Q9UFW8	CGGBP1	CGG triplet repeat-binding protein 1
0.977	0.989	0.987	0.988	0.996	1.023	0.998	0.942	0.962	1.027	1.007	1.021	Q9HD42	CHMP1A	Charged multivesicular body protein 1a
1.014	1.020	1.018	0.996	1.004	1.066	0.982	1.020	1.008	0.990	1.082	0.973	Q7LBR1	CHMP1B	Charged multivesicular body protein 1b
0.879	1.023	0.988	0.986	0.984	1.077	1.012	0.974	0.988	1.017	1.071	0.991	M0R1T5	CHMP2A	Charged multivesicular body protein 2a (Fragment)
0.911	0.999	0.951	0.986	1.032	1.063	1.003	0.922	0.980	1.009	1.002	1.020	Q9UQN3	CHMP2B	Charged multivesicular body protein 2b
1.040	1.006	0.999	0.967	0.983	1.011	0.993	0.918	0.970	1.032	0.957	1.082	Q9Y3E7	CHMP3	Charged multivesicular body protein 3
0.984	1.007	0.989	0.997	0.987	1.016	0.997	0.988	0.982	1.033	1.021	1.027	Q9H444	CHMP4B	Charged multivesicular body protein 4b
0.988	1.005	1.027	1.009	0.985	1.055	1.011	0.892	1.019	1.073	0.970	1.138	Q96CF2	CHMP4C	Charged multivesicular body protein 4c
0.993	1.002	0.994	0.966	0.953	1.002	0.976	0.905	0.981	0.979	1.004	0.966	Q9NZZ3	CHMP5	Charged multivesicular body protein 5
0.991	0.932	1.059	1.109	0.981	1.065	1.061	0.968	0.986	1.055	0.951	1.022	Q96FZ7	CHMP6	Charged multivesicular body protein 6
1.025	1.009	1.043	0.975	0.922	1.045	1.010	1.042	1.032	0.971	0.936	1.079	Q8WUX9	CHMP7	Charged multivesicular body protein 7
0.933	1.027	1.044	0.971	0.939	0.956	1.039	1.151	0.986	0.978	0.935	0.991	O60921	HUS1	Checkpoint protein HUS1
1.020	0.998	1.037	1.007	1.030	1.021	0.991	1.041	1.021	0.998	0.983	1.005	Q9BWS9	CHID1	Chitinase domain-containing protein 1
0.986	0.994	1.034	1.039	1.066	1.058	1.081	0.959	1.023	1.063	0.937	1.055	Q9BT22	ALG1	Chitobiosyldiphosphodolichol beta-mannosyltransferase
1.018	0.999	1.041	0.965	0.886	1.008	1.032	0.995	0.993	1.036	0.963	1.039	Q96S66	CLCC1	Chloride channel CLIC-like protein 1
1.140	1.027	1.003	1.052	1.323	0.949	1.047	0.943	1.063	1.003	1.001	0.997	O00299	CLIC1	Chloride intracellular channel protein 1
			1.200	1.546	0.999							O95833	CLIC3	Chloride intracellular channel protein 3
1.039	1.032	1.012	1.002	1.062	0.950	1.043	1.009	1.017	1.001	0.966	1.050	Q9Y696	CLIC4	Chloride intracellular channel protein 4
1.471	0.974	1.115	0.941	1.365	0.999	1.074	0.824	1.155	0.940	0.978	1.052	Q96NY7	CLIC6	Chloride intracellular channel protein 6
1.033	1.090	1.078	1.074	0.942	1.193	1.096	1.225	1.104	1.223	1.019	1.127	P51797	CLCN6	Chloride transport protein 6
0.994	1.134	0.984				1.146	1.136	0.932	0.880	1.041	1.087	P35790	CHKA	Choline kinase alpha
			0.915	0.819	0.909							Q9Y259	CHKB	Choline/ethanolamine kinase
1.020	1.021	1.072	0.978	0.887	1.029	0.981	1.088	1.056	1.072	1.026	1.098	Q9Y6K0	CEPT1	Choline/ethanolaminephosphotransferase 1
0.975	0.870	1.016	0.999	0.874	1.019	1.029	0.823	0.991	1.061	0.862	1.043	C9JEJ2	PCYT1A	Choline-phosphate cytidylyltransferase A
0.852	0.953	0.959	0.930	0.829	1.047	1.106	1.009	1.019	1.039	0.849	1.019	Q8WUD6	CHPT1	Cholinephosphotransferase 1
0.978	0.984	0.968										Q9P2E5	CHPF2	Chondroitin sulfate glucuronyltransferase

Abundance Ratio														
1 d M1/C1	1 d M2/C2	1 d M3/C3	4 d M1/C1	4 d M2/C2	4 d M3/C3	7 d M1/C1	7 d M2/C2	7 d M3/C3	10 d M1/C1	10 d M2/C2	10 d M3/C3	Accession ID	Gene name	Protein Description
1.054	1.087	1.082	1.055	0.822	0.937	0.988	1.136	1.018	1.000	0.945	1.110	Q8TDX6	CSGALNACT1	Chondroitin sulfate N-acetylgalactosaminyltransferase 1
1.286	0.899	1.005	1.026	0.870	1.137	0.894	1.123	1.191	2.556	1.069	1.291	Q8N6G5	CSGALNACT2	Chondroitin sulfate N-acetylgalactosaminyltransferase 2
			1.257	0.666	1.236	1.027	0.957	0.893				Q86X52	CHSY1	Chondroitin sulfate synthase 1
0.929	1.060	1.106										Q8IZ52	CHPF	Chondroitin sulfate synthase 2
									0.844	1.266	1.391	Q70JA7	CHSY3	Chondroitin sulfate synthase 3
1.026	1.029	1.024	1.022	1.015	1.034	0.986	1.102	1.043	0.933	1.010	1.014	Q9NRG0	CHRAC1	Chromatin accessibility complex protein 1
1.009	1.023	1.055	0.924	0.945	1.100	0.963	1.265	0.924	1.021	1.018	1.025	Q13111	CHAF1A	Chromatin assembly factor 1 subunit A
0.957	0.987	1.038	1.029	0.932	1.041	0.982	1.210	0.943	0.898	0.907	1.015	Q13112	CHAF1B	Chromatin assembly factor 1 subunit B
1.060	1.000	1.019	0.984	0.936	0.975	0.951	0.990	0.951	0.899	0.956	0.893	P83916	CBX1	Chromobox protein homolog 1
0.978	0.918	1.020	0.928	0.897	1.109	0.890	1.277	0.899	1.123	1.675	1.258	Q14781	CBX2	Chromobox protein homolog 2
1.019	1.006	0.982	0.978	1.000	1.005	0.955	0.941	0.982	0.956	1.008	0.948	Q13185	CBX3	Chromobox protein homolog 3
0.991	0.985	1.013	0.964	1.050	0.964	1.007	0.884	0.994	0.969	0.933	0.957	P45973	CBX5	Chromobox protein homolog 5
1.056	1.046	1.042	1.003	0.959	0.936	1.070	0.910	0.975	1.033	1.185	1.126	O95503	CBX6	Chromobox protein homolog 6
1.063	0.964	1.079	1.010	0.982	0.992	1.003	0.986	0.993	1.041	1.018	1.123	Q9HC52	CBX8	Chromobox protein homolog 8
1.024	0.969	1.023	0.920	0.906	1.010	0.955	1.019	1.038	0.939	0.963	0.995	Q9Y232	CDYL	Chromodomain Y-like protein
1.013	0.986	0.984	1.002	0.954	1.028	1.012	1.009	1.001	0.996	1.005	1.090	O14646	CHD1	Chromodomain-helicase-DNA-binding protein 1
1.014	0.991	1.023	0.985	0.958	1.012	1.010	1.075	1.014	0.989	0.974	1.031	Q86WJ1	CHD1L	Chromodomain-helicase-DNA-binding protein 1-like
1.008	1.008	1.016	1.009	0.932	1.031	1.009	0.976	1.006	0.952	0.970	1.012	O14647	CHD2	Chromodomain-helicase-DNA-binding protein 2
1.006	0.819	1.189	0.982	0.735	0.969				0.918	1.044	1.268	H7C0J3	CHD3	Chromodomain-helicase-DNA-binding protein 3 (Fragment)
			1.075	1.133	0.783							Q8TDI0	CHD5	Chromodomain-helicase-DNA-binding protein 5
1.061	0.972	1.002	0.865	0.969	1.067	0.912	1.181	0.988				Q8TD26	CHD6	Chromodomain-helicase-DNA-binding protein 6
0.998	1.008	1.006	0.995	0.917	0.960	0.917	1.070	0.980	0.905	0.964	0.993	Q9HCK8	CHD8	Chromodomain-helicase-DNA-binding protein 8
1.097	1.011	1.011	0.962	0.956	1.065	1.070	1.019	1.014	0.837	1.242	0.901	Q3L8U1	CHD9	Chromodomain-helicase-DNA-binding protein 9
			1.025	0.910	1.033	1.092	1.034	1.064				A0A0C4DFN5	TCTN3	Chromosome 10 open reading frame 61
0.999	1.035	1.001	0.985	0.942	1.024	0.991	1.058	1.036	1.054	0.972	0.972	A0A0B4J220	C11orf98	Chromosome 11 open reading frame 48, isoform CRA_c
0.947	1.070	0.997	0.991	0.935	1.017	1.006	1.084	1.106	1.349	1.130	1.207	F5H7W8	C12orf43	Chromosome 12 open reading frame 43, isoform CRA_a
0.876	0.970	1.029	1.011	0.981	0.960	1.058	0.964	0.959	1.204	1.018	1.219	F8VQD4	C12orf75	Chromosome 12 open reading frame 75 (Fragment)
1.031	0.979	0.996	0.956	0.947	0.952	1.007	1.101	1.007	0.896	0.922	1.090	Q5BIX2	ARKL1	Chromosome 18 open reading frame 25, isoform CRA_a
1.046	0.816	1.046	1.003	0.686	1.141	1.264	0.951	1.021	0.887	0.958	0.999	A0A087WXK8	FAM83D	Chromosome 20 open reading frame 129

Abundance Ratio														
1 d M1/C1	1 d M2/C2	1 d M3/C3	4 d M1/C1	4 d M2/C2	4 d M3/C3	7 d M1/C1	7 d M2/C2	7 d M3/C3	10 d M1/C1	10 d M2/C2	10 d M3/C3	Accession ID	Gene name	Protein Description
1.051	1.006	1.045	0.980	0.944	1.026	0.961	1.023	1.012	0.991	1.006	1.039	Q96JM3	CHAMP1	Chromosome alignment-maintaining phosphoprotein 1
1.060	1.040	1.072	0.962	0.844	0.846				0.958	1.047	1.031	H0YAV5	CHTF8	Chromosome transmission fidelity protein 8 homolog (Fragment)
0.996	0.990	0.979	0.958	0.944	1.049	0.932	1.087	0.988	0.970	1.027	0.997	P0CG12	CHTF8	Chromosome transmission fidelity protein 8 homolog isoform 2
0.976	0.993	1.016	1.037	0.878	0.990	0.973	1.067	1.054	0.954	0.885	1.129	O95239	KIF4A	Chromosome-associated kinesin KIF4A
0.917	0.932	0.863	1.014	1.102	1.048	1.258	1.079	0.992	1.648	1.068	1.614	P17538	CTRB1	Chymotrypsinogen B
1.055	0.996	1.005	1.007	0.942	1.016	0.944	1.031	0.956	0.960	1.016	0.924	Q9Y6A4	CFAP20	Cilia- and flagella-associated protein 20
1.014	1.026	0.982	1.020	0.962	1.064	1.092	1.159	1.099	0.975	0.987	0.996	Q96G28	CFAP36	Cilia- and flagella-associated protein 36
0.796	0.937	1.008							1.156	1.111	1.184	Q9UL16	CFAP45	Cilia- and flagella-associated protein 45
1.345	1.404	0.879	0.931	1.077	0.958	0.718	0.789	0.587				Q9P2B7	CFAP97	Cilia- and flagella-associated protein 97
0.964	0.951	1.009	0.955	0.908	1.021	0.838	0.620	1.024	1.227	1.004	1.102	Q9P2M7	CGN	Cingulin
0.974	0.989	0.984	1.043	1.050	1.027	1.039	0.945	0.984	1.050	1.033	1.071	J3KPP4	LUC7L3	Cisplatin resistance-associated overexpressed protein, isoform CRA_b
1.002	0.981	1.031	0.968	0.971	1.032	0.943	0.924	0.987	1.059	0.994	1.057	Q8N0X4	CLYBL	Citrate lyase subunit beta-like protein, mitochondrial
1.003	0.994	1.005	0.994	1.040	0.988	1.016	1.003	0.996	1.013	0.987	0.979	O75390	CS	Citrate synthase, mitochondrial
			0.917	1.023	0.658	0.921	0.953	1.186				A0A087WYG2	MAPK8IP3	C-Jun-amino-terminal kinase-interacting protein 3
1.014	1.013	1.012	1.010	1.013	1.018	1.012	1.022	1.014	1.022	1.007	1.040	O60271	SPAG9	C-Jun-amino-terminal kinase-interacting protein 4
0.772	0.952	0.947	0.938	0.907	1.031	0.964	0.742	0.975	1.004	0.984	1.085	Q96MX0	CMTM3	CKLF-like MARVEL transmembrane domain-containing protein 3
			0.941	1.059	1.024				1.328	1.072	1.140	Q8IZR5	CMTM4	CKLF-like MARVEL transmembrane domain-containing protein 4
0.923	0.949	0.906	1.010	0.863	1.141	1.071	0.807	0.986	1.317	1.114	1.279	Q9NX76	CMTM6	CKLF-like MARVEL transmembrane domain-containing protein 6
0.865	0.951	0.982							0.900	0.880	1.008	Q9HAW4	CLSPN	Claspin
1.093	1.040	1.098	0.974	0.979	0.947	1.048	0.978	0.952	1.024	0.989	0.985	O14503	BHLHE40	Class E basic helix-loop-helix protein 40
1.135	0.985	1.009	0.997	0.917	0.866	1.008	1.133	1.008	1.028	1.051	0.967	P53675	CLTCL1	Clathrin heavy chain 2
0.989	1.006	0.995	1.004	1.064	0.997	0.993	1.021	1.048	0.954	1.001	0.967	A0A087WVQ6	CLTC	Clathrin heavy chain
0.996	1.003	1.000	0.999	0.988	1.000	0.989	0.983	0.991	1.023	1.002	1.019	Q14677	CLINT1	Clathrin interactor 1
0.963	1.007	1.166	1.103	1.168	1.155	1.177	0.877	1.067	1.041	1.091	1.174	A0A0R4J2F2	CLDND1	Claudin domain-containing protein 1
1.187	0.811	0.988	1.250	0.835	1.076	1.181	0.900	1.104	1.221	1.100	1.095	O95832	CLDN1	Claudin-1
0.850	1.003	0.960	1.066	0.976	1.353	1.236	1.071	1.064				O75508	CLDN11	Claudin-11
1.019	1.010	1.029	1.001	0.963	1.021	1.020	1.012	1.000	0.988	0.989	1.023	Q10570	CPSF1	Cleavage and polyadenylation specificity factor subunit 1

Abundance Ratio														
1 d M1/C1	1 d M2/C2	1 d M3/C3	4 d M1/C1	4 d M2/C2	4 d M3/C3	7 d M1/C1	7 d M2/C2	7 d M3/C3	10 d M1/C1	10 d M2/C2	10 d M3/C3	Accession ID	Gene name	Protein Description
1.021	0.986	1.027	0.975	0.994	1.013	1.014	1.038	1.019	1.015	1.004	1.049	Q9P2I0	CPSF2	Cleavage and polyadenylation specificity factor subunit 2
1.041	1.020	1.021	0.995	0.964	1.003	1.002	1.043	0.971	0.982	1.003	1.013	Q9UKF6	CPSF3	Cleavage and polyadenylation specificity factor subunit 3
0.989	1.111	1.025	0.992	1.154	0.977	1.030	0.955	0.999	1.044	1.007	1.030	O95639	CPSF4	Cleavage and polyadenylation specificity factor subunit 4
1.010	0.986	0.977	0.978	0.956	1.009	0.998	1.005	0.968	1.033	1.004	0.972	O43809	NUDT21	Cleavage and polyadenylation specificity factor subunit 5
1.059	1.015	0.990	1.019	0.968	0.975	1.049	0.966	0.986	1.054	1.011	0.982	Q05048	CSTF1	Cleavage stimulation factor subunit 1
1.038	0.992	1.020	0.977	0.998	1.002	0.996	1.009	1.033	0.947	0.990	1.009	P33240	CSTF2	Cleavage stimulation factor subunit 2
1.033	0.952	1.113	1.045	1.026	1.010	1.032	1.057	1.010	0.979	0.974	1.056	Q9H0L4	CSTF2T	Cleavage stimulation factor subunit 2 tau variant
1.030	1.011	1.019	0.994	1.000	0.979	0.972	1.004	0.985	0.980	1.012	1.004	Q12996	CSTF3	Cleavage stimulation factor subunit 3
0.994	1.007	0.985	1.036	0.996	1.020	1.093	1.098	1.052	1.067	1.082	1.047	O96005	CLPTM1	Cleft lip and palate transmembrane protein 1
1.007	0.991	0.997	0.998	0.924	1.027	1.092	1.136	1.017	1.173	1.072	1.087	Q96KA5	CLPTM1L	Cleft lip and palate transmembrane protein 1-like protein
0.975	0.991	1.009	0.978	0.940	0.984	0.987	0.965	1.001	1.001	0.975	1.021	Q7Z460	CLASP1	CLIP-associating protein 1
0.878	0.930	0.985	0.994	0.967	1.033	0.954	1.347	1.135				E3W994	CLASP2	CLIP-associating protein 2
1.081	0.995	1.064	1.023	0.999	1.027	1.056	1.018	1.045	0.951	0.894	1.085	A0A0A0MQS2	CLASRP	CLK4-associating serine/arginine-rich protein
0.942	1.007	0.983	0.990	0.893	1.043	1.016	1.024	1.063	1.005	1.039	1.101	I3L2B0	CLUH	Clustered mitochondria protein homolog (Fragment)
0.982	1.001	1.024	1.025	0.981	1.037	1.025	1.028	1.026	0.987	0.994	1.075	O75153	CLUH	Clustered mitochondria protein homolog
1.143	0.900	1.011	1.081	1.053	1.037	1.107	1.067	1.003	1.020	0.987	1.007	J3KNW5	CLUAP1	Clusterin-associated protein 1
1.161	0.943	1.015	0.830	1.020	1.096	1.085	0.912	1.350	0.875	1.259	1.106	I3L2E1	CLUAP1	Clusterin-associated protein 1
0.978	1.006	0.999	0.958	0.950	1.013	1.060	1.123	1.083	1.028	1.001	1.109	Q8IY22	CMIP	C-Maf-inducing protein
			1.053	0.926	1.230	0.909	1.013	1.060	0.935	0.918	1.045	Q11201	ST3GAL1	CMP-N-acetylneuraminate-beta-galactosamide-alpha-2,3-sialyltransferase 1
						0.996	1.261	1.089				Q16842	ST3GAL2	CMP-N-acetylneuraminate-beta-galactosamide-alpha-2,3-sialyltransferase 2
1.157	1.070	0.964	1.053	1.014	1.104	1.053	1.184	1.044	1.092	1.130	0.965	P78382	SLC35A1	CMP-sialic acid transporter
0.985	0.931	0.991	0.928	1.090	1.033	1.077	1.011	1.043	1.169	1.077	1.108	A0A0U1RR27	DENND4A	C-myc promoter-binding protein
1.014	0.996	0.995	0.978	0.970	0.994	0.980	1.025	0.980	0.973	0.966	0.975	Q14019	COTL1	Coactosin-like protein
1.142	1.015	0.955	0.970	0.883	1.147	0.939	1.125	0.979	1.010	1.020	1.025	A0A0A0MRJ7	F5	Coagulation factor V
0.999	0.980	1.000	0.982	0.982	0.989	0.948	1.030	1.025	0.961	0.968	0.954	M0QXB4	COPE	Coatomer protein complex, subunit epsilon, isoform CRA_g
1.000	1.011	1.003	1.008	0.998	0.979	0.996	1.023	0.993	0.995	0.980	0.971	P53621	COPA	Coatomer subunit alpha
0.994	1.014	1.004	0.981	0.964	0.997	0.978	1.081	1.006	0.946	0.983	0.961	P53618	COPB1	Coatomer subunit beta
0.989	0.996	0.991	0.992	0.984	0.987	0.963	1.011	0.985	0.968	0.967	0.991	P35606	COPB2	Coatomer subunit beta'

Abundance Ratio												Accession ID	Gene name	Protein Description
1 d M1/C1	1 d M2/C2	1 d M3/C3	4 d M1/C1	4 d M2/C2	4 d M3/C3	7 d M1/C1	7 d M2/C2	7 d M3/C3	10 d M1/C1	10 d M2/C2	10 d M3/C3			
0.993	0.997	1.024	0.986	0.999	1.002	0.986	1.007	1.020	0.970	0.952	1.011	Q9Y678	COPG1	Coatomer subunit gamma-1
1.005	0.992	1.022	1.006	0.997	1.031	0.994	1.033	1.032	0.976	1.025	0.992	Q9UBF2	COPG2	Coatomer subunit gamma-2
1.013	0.997	0.972	0.978	0.903	0.966	0.974	1.076	0.980	0.958	1.003	0.943	F8VVA7	COPZ1	Coatomer subunit zeta-1
1.025	0.986	0.984	0.996	1.012	1.041	1.037	1.041	1.001	1.087	1.031	1.059	Q96EY8	MMAB	Cob(I)yrinic acid a,c-diamide adenosyltransferase, mitochondrial
1.101	1.018	1.051	1.035	0.969	1.030	1.005	1.067	0.957	0.990	1.007	1.001	Q8IUF1	CBWD2	COBW domain-containing protein 2
0.920	0.930	1.018	0.848	1.034	1.099	0.902	0.663	0.915	1.064	1.031	0.975	Q8IWY9	CDAN1	Codanin-1
0.992	0.954	1.046	0.936	0.894	0.926	0.968	1.149	1.048	0.876	0.918	1.091	Q9H8M1	COQ10B	Coenzyme Q-binding protein COQ10 homolog B, mitochondrial
1.110	1.002	0.958	1.048	1.149	1.000	1.021	0.967	1.060	0.986	0.984	0.935	E9PK25	CFL1	Cofilin-1
0.991	0.987	0.979	0.979	1.006	1.029	0.992	0.929	1.009	1.029	0.993	1.005	Q9Y281	CFL2	Cofilin-2
1.025	1.021	1.051	0.957	0.944	1.036	0.976	0.968	1.029	0.926	0.923	0.978	Q8WVM7	STAG1	Cohesin subunit SA-1
0.991	1.029	1.055	1.002	0.986	0.992	1.009	0.990	0.991	0.978	0.979	1.060	Q6P1N0	CC2D1A	Coiled-coil and C2 domain-containing protein 1A
1.204	1.078	0.861										Q9P2K1	CC2D2A	Coiled-coil and C2 domain-containing protein 2A
1.004	0.978	0.975	1.010	0.941	1.013	1.012	1.067	0.987	1.041	1.025	1.095	J3KR35	CCDC12	Coiled-coil domain containing 12, isoform CRA_a
1.025	1.041	1.121	1.009	0.894	0.959	1.019	1.092	1.058	1.014	0.919	1.120	Q96A19	CCDC102A	Coiled-coil domain-containing protein 102A
1.110	0.966	1.094	0.810	1.010	1.286							Q9BWC9	CCDC106	Coiled-coil domain-containing protein 106
1.039	0.970	0.993	1.084	1.024	1.057	1.186	0.937	0.952	1.064	0.987	1.050	Q96NT0	CCDC115	Coiled-coil domain-containing protein 115
1.004	0.953	1.113	1.130	1.113	1.006	0.945	0.897	0.885	1.340	1.025	1.411	Q8IWD4	CCDC117	Coiled-coil domain-containing protein 117
0.974	0.944	1.019	0.957	0.967	1.040	0.934	0.828	0.988	1.027	0.997	1.024	Q96CT7	CCDC124	Coiled-coil domain-containing protein 124
1.066	1.004	1.554				0.785	0.958	0.949	1.098	0.585	0.948	Q96EE4	CCDC126	Coiled-coil domain-containing protein 126
0.957	0.960	0.989	0.927	0.968	1.009	0.973	0.962	0.995	0.953	0.906	0.942	Q96BQ5	CCDC127	Coiled-coil domain-containing protein 127
						0.836	1.196	1.014	0.961	1.104	0.975	P13994	CCDC130	Coiled-coil domain-containing protein 130
1.036	0.949	1.032	0.997	0.959	0.997	1.025	1.017	0.989	0.998	0.963	0.986	Q9H6E4	CCDC134	Coiled-coil domain-containing protein 134
1.064	0.999	1.060	1.048	0.994	1.120	1.082	0.979	1.036	1.022	1.038	1.028	I3L0U5	CCDC137	Coiled-coil domain-containing protein 137 (Fragment)
0.831	1.076	0.884	1.005	1.480	1.042	1.165	0.998	0.935	1.256	1.048	1.131	Q5M9N0	CCDC158	Coiled-coil domain-containing protein 158
1.103	1.018	1.147	0.978	0.828	0.978	1.039	1.179	0.972	0.973	1.028	1.077	Q6PII3	CCDC174	Coiled-coil domain-containing protein 174
0.916	0.914	1.034	1.013	0.924	0.968	1.054	0.852	0.945	1.267	0.915	1.104	Q7Z3E2	CCDC186	Coiled-coil domain-containing protein 186
1.020	0.992	0.999	1.010	0.977	1.034	1.002	1.038	1.046	1.006	0.980	1.053	O60826	CCDC22	Coiled-coil domain-containing protein 22
0.966	1.000	0.994	1.035	0.982	1.013	1.022	1.023	0.986	1.039	1.028	1.017	Q86WR0	CCDC25	Coiled-coil domain-containing protein 25
0.980	1.037	1.165	0.956	0.953	0.959	0.940	0.955	1.021	0.978	1.062	1.025	Q8IWP9	CCDC28A	Coiled-coil domain-containing protein 28A
1.010	1.012	1.002	0.996	0.983	1.008	0.981	0.927	1.009	1.010	0.992	1.030	Q96MW1	CCDC43	Coiled-coil domain-containing protein 43
0.968	1.005	1.007	1.012	1.014	0.986	1.005	1.068	0.982	1.023	1.061	1.018	Q96A33	CCDC47	Coiled-coil domain-containing protein 47
0.992	1.002	1.102	1.020	0.963	1.028	0.971	0.982	1.020	1.057	1.024	1.027	Q96ER9	CCDC51	Coiled-coil domain-containing protein 51

Abundance Ratio														
1 d M1/C1	1 d M2/C2	1 d M3/C3	4 d M1/C1	4 d M2/C2	4 d M3/C3	7 d M1/C1	7 d M2/C2	7 d M3/C3	10 d M1/C1	10 d M2/C2	10 d M3/C3	Accession ID	Gene name	Protein Description
1.010	1.052	1.057	1.013	1.013	0.961	1.003	0.978	1.019	0.978	1.017	1.018	Q4VC31	CCDC58	Coiled-coil domain-containing protein 58
0.998	0.945	1.013	1.004	0.932	1.014	1.025	1.005	1.010	1.029	1.022	1.074	Q16204	CCDC6	Coiled-coil domain-containing protein 6
1.033	1.022	0.980	1.002	0.907	0.990	1.069	0.967	0.889	1.051	1.031	0.984	Q9Y6R9	CCDC61	Coiled-coil domain-containing protein 61
1.013	1.101	0.900	1.041	0.802	0.883	0.962	1.255	0.990				Q8N9Z2	CCDC71L	Coiled-coil domain-containing protein 71L
			1.551	0.720	1.262							Q9BR77	CCDC77	Coiled-coil domain-containing protein 77
1.000	1.015	1.020	0.952	1.020	0.937	1.049	0.930	1.024	1.026	0.954	1.064	Q8N4S0	CCDC82	Coiled-coil domain-containing protein 82
			0.973	0.910	1.141							Q86UT8	CCDC84	Coiled-coil domain-containing protein 84
1.391	1.038	1.056	1.000	1.016	1.052	1.039	1.122	1.142				Q15834	CCDC85B	Coiled-coil domain-containing protein 85B
1.010	0.974	1.025	1.033	0.969	1.031	0.969	0.917	0.997	1.025	1.026	1.058	Q9H6F5	CCDC86	Coiled-coil domain-containing protein 86
0.988	0.984	1.001	0.970	0.953	1.059	1.001	0.882	0.979	1.049	1.030	1.122	Q9Y3X0	CCDC9	Coiled-coil domain-containing protein 9
0.984	0.981	1.035	0.984	0.947	1.028	1.000	1.013	1.017	1.036	1.003	1.042	Q9GZT6	CCDC90B	Coiled-coil domain-containing protein 90B, mitochondrial
0.978	0.940	0.971	1.013	0.999	1.015	1.060	0.999	1.096	1.097	0.976	1.066	Q7Z6B0	CCDC91	Coiled-coil domain-containing protein 91
1.018	1.015	0.976	1.000	1.004	0.992	1.036	1.029	1.011	1.036	0.991	1.045	Q567U6	CCDC93	Coiled-coil domain-containing protein 93
0.948	1.018	1.046	1.010	0.997	0.987	0.952	1.113	1.033	1.000	1.006	1.091	Q9BW85	CCDC94	Coiled-coil domain-containing protein 94
1.045	0.930	1.146	0.983	0.965	1.081	0.988	0.834	0.977	0.973	0.968	0.989	Q96F63	CCDC97	Coiled-coil domain-containing protein 97
1.113	0.957	1.237										A0A087X0M0	R3HCC1L	Coiled-coil domain-containing protein R3HCC1L
0.978	1.010	1.007	0.970	0.928	1.086	1.009	1.092	0.976	0.996	1.004	1.024	Q96BP2	CHCHD1	Coiled-coil-helix-coiled-coil-helix domain-containing protein 1
									0.619	0.913	0.933	B5MBW9	CHCHD10	Coiled-coil-helix-coiled-coil-helix domain-containing protein 10, mitochondrial
1.077	1.098	0.891	0.923	0.945	1.329	1.049	1.045	0.975	0.894	0.985	0.967	Q9Y6H1	CHCHD2	Coiled-coil-helix-coiled-coil-helix domain-containing protein 2
1.003	0.999	1.014	0.991	1.036	1.045	1.018	1.069	1.126	1.091	0.985	1.115	P38432	COIL	Coilin
0.989	0.976	0.993	0.977	0.957	0.976	0.963	0.928	0.999	0.994	0.960	1.015	Q14011	CIRBP	Cold-inducible RNA-binding protein
			1.146	0.985	1.249							P12109	COL6A1	Collagen alpha-1(VI) chain
			1.094	1.006	0.816							Q99715	COL12A1	Collagen alpha-1(XII) chain
1.034	1.004	1.034	0.979	1.002	1.011	1.007	1.024	0.995	0.999	0.944	1.009	P39060	COL18A1	Collagen alpha-1(XVIII) chain
1.013	1.114	1.059	1.027	1.011	0.949	0.995	1.159	0.952	1.053	0.973	1.076	P08572	COL4A2	Collagen alpha-2(IV) chain
1.120	1.003	1.048	1.431	0.989	1.193	1.081	1.248	1.024	0.951	0.905	1.068	P12110	COL6A2	Collagen alpha-2(VI) chain
0.969	1.014	1.026	0.971	0.971	1.043	0.966	1.063	1.056	0.996	0.885	1.062	Q8N668	COMMD1	COMM domain-containing protein 1
0.988	0.964	1.072	1.015	0.989	1.023	0.982	1.068	1.030	1.008	0.955	1.030	Q9Y6G5	COMMD10	COMM domain-containing protein 10
1.069	1.055	1.041	0.986	0.976	1.052	0.966	1.063	1.012	1.017	0.945	1.079	Q86X83	COMMD2	COMM domain-containing protein 2
1.016	1.004	1.015	0.974	0.980	1.020	0.994	1.112	1.065	0.959	0.930	0.996	Q9UBI1	COMMD3	COMM domain-containing protein 3
1.026	1.003	1.034	1.014	1.051	1.022	0.997	1.053	1.094	0.959	0.975	0.980	Q9H0A8	COMMD4	COMM domain-containing protein 4

Abundance Ratio														
1 d M1/C1	1 d M2/C2	1 d M3/C3	4 d M1/C1	4 d M2/C2	4 d M3/C3	7 d M1/C1	7 d M2/C2	7 d M3/C3	10 d M1/C1	10 d M2/C2	10 d M3/C3	Accession ID	Gene name	Protein Description
1.040	0.985	1.054	0.995	0.918	1.049	1.015	1.009	1.024	0.988	0.905	1.011	Q9GZQ3	COMMD5	COMM domain-containing protein 5
0.959	0.945	1.025	0.969	1.088	1.044	0.931	0.888	1.044	1.017	0.972	1.074	Q7Z4G1	COMMD6	COMM domain-containing protein 6
0.961	0.961	1.018	0.966	0.933	1.005	1.012	1.044	1.047	0.922	0.908	0.947	Q86VX2	COMMD7	COMM domain-containing protein 7
1.012	0.982	1.027	1.004	0.973	1.067	0.988	1.101	1.076	1.026	0.946	0.969	Q9NX08	COMMD8	COMM domain-containing protein 8
1.004	1.001	1.043	0.971	0.960	1.042	0.938	0.968	1.023	0.987	1.018	1.041	Q9P000	COMMD9	COMM domain-containing protein 9
1.036	1.026	1.050	1.009	0.953	0.992	1.079	1.077	1.015	1.022	0.836	1.073	P01024	C3	Complement C3
0.993	0.979	1.026	0.983	0.995	1.029	1.001	1.060	1.068	0.991	1.010	1.036	Q07021	C1QBP	Complement component 1 Q subcomponent-binding protein, mitochondrial
0.893	0.984	1.026										P10643	C7	Complement component C7
1.018	0.984	1.033	1.128	1.000	1.055	1.052	1.153	1.002	1.046	1.106	1.020	B1AP13	CD55	Complement decay-accelerating factor
1.051	0.979	1.040	1.045	1.146	1.056	1.304	1.097	1.160	1.019	1.081	1.149	Q9NPL8	TIMMDC1	Complex I assembly factor TIMMDC1, mitochondrial
1.007	0.940	0.811	1.025	1.159	0.956	0.991	1.057	1.106	0.997	1.050	1.053	Q8IUX1	TMEM126B	Complex I assembly factor TMEM126B, mitochondrial
1.071	0.966	1.145	1.060	0.873	1.024	1.069	1.049	1.009	1.037	0.929	1.094	Q9Y375	NDUFAF1	Complex I intermediate-associated protein 30, mitochondrial
0.766	0.999	0.961	0.944	0.819	0.802	1.113	0.961	1.393	1.017	0.962	0.998	Q5U5X0	LYRM7	Complex III assembly factor LYRM7
						1.280	0.594	1.120				O14810	CPLX1	Complexin-1
0.966	1.011	1.014	1.017	0.913	0.993	1.019	1.086	1.064	0.942	0.923	1.045	Q15021	NCAPD2	Condensin complex subunit 1
0.942	1.009	0.981	1.001	0.960	0.991	1.018	0.974	1.024	0.985	0.977	1.091	Q15003	NCAPH	Condensin complex subunit 2
0.993	1.000	1.006	0.988	0.904	1.018	1.032	1.022	1.020	1.009	0.857	1.116	Q9BPX3	NCAPG	Condensin complex subunit 3
1.033	1.041	1.049	0.951	0.855	1.002	1.001	1.152	0.977	0.946	0.983	1.056	P42695	NCAPD3	Condensin-2 complex subunit D3
1.330	1.027	0.988	0.958	1.052	1.285				1.006	1.063	0.987	P29279	CTGF	Connective tissue growth factor
0.934	1.019	0.879	0.982	1.009	1.041	0.997	1.170	0.977	1.190	1.126	1.317	Q8WXI2	CNKSR2	Connector enhancer of kinase suppressor of ras 2
			0.852	0.908	1.044	0.892	1.040	0.978	1.211	0.908	1.275	Q6P9H4	CNKSR3	Connector enhancer of kinase suppressor of ras 3
1.022	0.979	1.051	1.023	0.952	0.995	0.958	0.989	0.980	1.036	0.964	1.043	Q8WTW3	COG1	Conserved oligomeric Golgi complex subunit 1
1.071	1.020	1.032	0.972	0.986	1.001	0.996	1.030	1.004	0.974	0.987	1.087	Q14746	COG2	Conserved oligomeric Golgi complex subunit 2
0.984	1.013	1.037	0.984	0.975	1.000	0.947	1.095	0.993	0.976	1.024	0.996	Q96JB2	COG3	Conserved oligomeric Golgi complex subunit 3
1.025	1.017	1.001	1.016	0.959	1.046	0.996	0.995	0.978	1.006	0.997	1.023	J3KNI1	COG4	Conserved oligomeric Golgi complex subunit 4
0.992	0.970	1.043	0.982	0.965	1.009	0.979	1.073	1.027	0.974	0.975	1.020	Q9Y2V7	COG6	Conserved oligomeric Golgi complex subunit 6
1.044	1.006	1.013	1.001	0.969	0.977	0.963	1.064	1.006	0.958	0.990	1.010	P83436	COG7	Conserved oligomeric Golgi complex subunit 7
1.038	1.022	1.039	1.006	0.971	1.034	0.975	1.041	1.037	0.989	0.974	1.009	Q96MW5	COG8	Conserved oligomeric Golgi complex subunit 8
0.954	1.003	1.025	0.989	0.937	0.982	0.929	1.050	1.061	0.874	0.966	0.993	F5GY05	FAM120B	Constitutive coactivator of peroxisome proliferator-activated receptor gamma
1.074	1.026	1.022	0.978	0.932	1.006	1.072	1.068	0.961	1.012	1.032	1.031	Q9NX05	FAM120C	Constitutive coactivator of PPAR-gamma-like protein 2

Abundance Ratio														
1 d M1/C1	1 d M2/C2	1 d M3/C3	4 d M1/C1	4 d M2/C2	4 d M3/C3	7 d M1/C1	7 d M2/C2	7 d M3/C3	10 d M1/C1	10 d M2/C2	10 d M3/C3	Accession ID	Gene name	Protein Description
1.078	1.044	1.001	1.030	1.015	1.021	0.992	0.939	0.867	1.108	1.170	1.152	Q8IWV2	CNTN4	Contactin-4
1.035	1.018	1.002	1.000	0.946	1.013	1.056	1.086	0.991	1.077	1.050	0.998	P78357	CNTNAP1	Contactin-associated protein 1
1.036	1.025	1.008	1.018	0.963	0.979	0.981	1.128	0.977	0.932	0.969	1.079	Q9BZ76	CNTNAP3	Contactin-associated protein-like 3
0.950	0.945	0.994	0.946	0.999	1.047	1.060	0.941	1.006	1.052	0.985	1.075	Q9NQ92	COPRS	Coordinator of PRMT5 and differentiation stimulator
1.001	0.996	0.993	1.037	1.008	1.020	1.040	1.029	1.037	1.036	1.003	1.035	A8K070	GPS1	COP9 signalosome complex subunit 1
1.017	0.983	1.010	0.994	0.964	1.005	0.986	1.063	1.021	0.968	0.976	0.976	P61201	COPS2	COP9 signalosome complex subunit 2
1.009	0.952	1.010	1.002	1.002	1.020	1.029	0.986	0.985	1.023	1.024	1.035	Q9UNS2	COPS3	COP9 signalosome complex subunit 3
1.013	0.992	1.007	1.008	0.996	0.978	0.994	1.041	0.996	0.956	1.002	0.975	Q9BT78	COPS4	COP9 signalosome complex subunit 4
1.014	0.997	0.985	1.009	1.039	1.020	0.990	1.060	1.036	0.976	1.012	0.991	Q92905	COPS5	COP9 signalosome complex subunit 5
0.994	0.983	1.018	1.004	0.998	0.965	0.985	1.047	1.029	0.957	1.031	0.987	Q7L5N1	COPS6	COP9 signalosome complex subunit 6
1.020	1.015	0.992	1.014	1.055	1.033	1.045	0.999	1.055	1.021	0.991	1.029	Q9UBW8	COPS7A	COP9 signalosome complex subunit 7a
0.974	0.942	1.048	0.994	0.942	1.062	1.069	0.987	0.977	1.009	0.964	1.071	Q9H9Q2	COPS7B	COP9 signalosome complex subunit 7b
1.019	1.012	0.998	0.986	0.949	0.991	1.006	1.067	1.016	0.983	1.019	0.973	Q99627	COPS8	COP9 signalosome complex subunit 8
1.309	0.980	1.183	1.367	1.177	0.986	0.859	1.007	1.159	1.018	0.830	0.967	Q8WXC6	COPS9	COP9 signalosome complex subunit 9
1.047	1.010	1.025	1.022	1.067	0.999	0.998	0.997	1.028	0.958	0.978	0.935	B0QZ18	CPNE1	Copine-1
1.032	1.046	1.023	0.988	0.958	0.992	0.985	0.981	0.999	1.022	0.978	1.024	Q96FN4	CPNE2	Copine-2
0.994	1.007	0.954	1.035	1.128	1.020	1.044	1.003	1.079	1.071	1.010	1.017	O75131	CPNE3	Copine-3
1.000	1.009	1.002	0.998	0.958	0.981	1.006	1.028	0.973	1.025	1.029	1.042	Q9UBL6	CPNE7	Copine-7
0.995	1.018	1.015	0.983	0.969	0.982	1.001	1.096	0.997	1.006	0.969	1.021	Q86YQ8	CPNE8	Copine-8
0.988	0.985	1.017	1.041	1.004	1.082	1.007	1.041	1.024	0.995	0.980	1.017	O14618	CCS	Copper chaperone for superoxide dismutase
1.029	0.910	1.058	0.991	0.934	1.035	0.938	1.035	0.992	0.955	0.991	1.026	Q9NTM9	CUTC	Copper homeostasis protein cutC homolog
1.017	1.244	1.129	1.018	1.371	1.263	1.020	1.160	1.180	1.034	1.059	1.085	E5RIM7	ATOX1	Copper transport protein ATOX1
									0.974	0.959	1.179	P35670	ATP7B	Copper-transporting ATPase 2
1.095	1.075	0.934	1.017	1.014	0.931	0.882	1.154	1.137	0.942	1.031	0.911	A0A0D9SG04	COBLL1	Cordon-bleu protein-like 1
1.049	1.002	1.011	1.007	1.000	0.977	0.988	1.036	1.011	0.947	0.969	0.962	O75367	H2AFY	Core histone macro-H2A.1
			1.004	0.971	1.243	1.065	1.057	1.074	1.118	0.866	1.323	Q9P0M6	H2AFY2	Core histone macro-H2A.2
0.971	1.004	0.997	0.986	1.019	1.016	1.007	1.060	1.011	1.026	1.061	1.081	Q13951	CBFB	Core-binding factor subunit beta
0.852	0.984	1.282	0.964	0.989	1.078	0.931	0.731	0.952	1.011	0.982	1.110	Q86X95	CIR1	Corepressor interacting with RBPJ 1
1.026	1.047	1.066	1.009	1.090	0.971	1.050	1.142	0.924	1.188	1.119	1.109	P31146	CORO1A	Coronin-1A
0.985	0.961	0.983	0.995	0.993	1.008	1.002	0.964	1.001	1.007	0.996	0.973	Q9BR76	CORO1B	Coronin-1B
0.942	1.285	1.153	1.000	0.874	0.933	0.961	0.914	0.964	1.197	1.055	1.103	Q92828	CORO2A	Coronin-2A
1.266	1.011	1.041	1.102	1.371	0.908	1.016	0.784	1.297	0.921	0.956	0.935	Q9P1F3	ABRACL	Costars family protein ABRACL
1.034	0.906	0.962	0.981	0.967	1.144	1.190	0.936	0.944	1.064	0.965	1.071	P24468	NR2F2	COUP transcription factor 2

Abundance Ratio														
1 d M1/C1	1 d M2/C2	1 d M3/C3	4 d M1/C1	4 d M2/C2	4 d M3/C3	7 d M1/C1	7 d M2/C2	7 d M3/C3	10 d M1/C1	10 d M2/C2	10 d M3/C3	Accession ID	Gene name	Protein Description
1.016	1.008	1.015	0.938	0.966	1.037	0.996	0.877	0.964	1.039	0.999	0.987	Q9NRP2	CMC2	COX assembly mitochondrial protein 2 homolog
0.872	0.994	1.001	1.087	1.180	0.977	1.033	0.970	1.002	1.073	1.016	1.024	H7C3D8	CMC1	COX assembly mitochondrial protein homolog (Fragment)
1.076	0.914	1.008	1.012	0.927	1.006	1.029	0.962	1.007	1.165	0.940	1.162	P78310	CXADR	Coxsackievirus and adenovirus receptor
0.975	0.973	0.988	0.951	0.957	0.980	0.930	0.869	0.961	0.990	1.000	1.037	Q9UEE9	CFDP1	Craniofacial development protein 1
0.996	0.938	1.031	0.997	0.946	1.030	0.984	1.074	1.036	0.966	1.099	0.999	P12277	CKB	Creatine kinase B-type
0.982	0.978	1.018	0.984	0.948	1.032	0.954	0.900	0.977	1.053	1.000	1.074	Q92793	CREBBP	CREB-binding protein
1.220	0.880	0.974				0.723	0.945	1.104				H0YDQ8	CRTC2	CREB-regulated transcription coactivator 2 (Fragment)
0.931	0.960	0.987	0.996	0.937	1.046	0.989	0.929	0.926	1.010	1.099	1.015	Q53ET0	CRTC2	CREB-regulated transcription coactivator 2
0.976	0.968	1.166	1.023	1.063	1.022	0.994	1.001	0.921	1.201	1.200	1.048	Q6UUV7	CRTC3	CREB-regulated transcription coactivator 3
0.986	1.013	0.953	1.015	1.059	0.987	0.986	1.053	1.016	0.976	0.996	0.972	P46109	CRKL	Crk-like protein
1.042	1.004	1.029	1.000	0.977	1.058	1.029	1.057	1.016	0.991	0.999	1.030	Q9BZJ0	CRNKL1	Crooked neck-like protein 1
1.197	0.986	1.096	1.025	1.283	1.003	1.016	1.043	1.055	1.044	1.072	1.016	O94886	TMEM63A	CSC1-like protein 1
1.034	0.990	0.978	1.049	0.972	0.864	1.029	1.066	0.958	1.040	1.078	0.950	Q5T3F8	TMEM63B	CSC1-like protein 2
									0.990	1.022	0.966	Q2NKJ3	CTC1	CST complex subunit CTC1
1.067	0.991	1.046	0.992	0.932	1.113	1.089	1.079	1.024	1.093	1.166	1.166	Q05D32	CTDSPL2	CTD small phosphatase-like protein 2
0.811	0.987	1.018	1.000	0.929	1.013	0.869	0.977	1.116	0.772	0.992	1.344	O15194	CTDSPL	CTD small phosphatase-like protein
1.033	1.012	0.991	1.012	0.961	0.955	0.996	1.082	1.005	0.989	0.993	1.011	Q13363	CTBP1	C-terminal-binding protein 1
1.003	1.011	0.994	1.004	1.036	0.973	0.999	1.013	0.994	1.001	0.994	1.002	P17812	CTPS1	CTP synthase 1
1.051	0.975	1.027	0.983	0.975	1.042	1.031	1.070	1.012	1.008	0.969	1.027	Q9NRF8	CTPS2	CTP synthase 2
1.029	0.995	1.039	0.985	0.954	1.036	1.016	0.994	0.968	0.982	1.006	1.018	Q9P2B4	CTTNBP2NL	CTTNBP2 N-terminal-like protein
						1.248	0.468	0.914	1.082	0.911	1.158	Q9ULY5	CLEC4E	C-type lectin domain family 4 member E
0.969	0.985	1.003	0.969	0.949	1.022	1.028	0.946	0.974	1.055	0.972	1.058	Q9UBG0	MRC2	C-type mannose receptor 2
0.981	1.014	1.043	1.032	0.993	1.026	1.052	1.022	0.976	1.050	0.984	1.052	Q9H5V8	CDCP1	CUB domain-containing protein 1
1.031	1.068	1.140	0.882	0.995	1.071	0.974	0.959	1.048	1.045	1.130	1.316	Q9NWM3	CUEDC1	CUE domain-containing protein 1
1.059	1.070	1.102										Q9H467	CUEDC2	CUE domain-containing protein 2
0.937	1.006	1.016	1.005	1.084	1.025	0.995	0.911	1.001	0.964	1.065	0.987	G5EA30	CELF1	CUG triplet repeat, RNA binding protein 1, isoform CRA_c
0.862	1.019	0.958	1.034	1.040	0.979	1.027	1.090	0.921	1.090	1.022	0.895	E9PSH0	CELF1	CUGBP Elav-like family member 1 (Fragment)
0.978	1.033	1.012	1.053	0.987	0.994	1.073	1.071	1.042	1.019	1.034	1.041	A0A0J9YXJ0	CELF2	CUGBP Elav-like family member 2
1.010	1.003	0.988	0.990	0.999	0.986	0.999	1.044	1.002	0.969	0.980	1.011	Q13616	CUL1	Cullin-1
1.009	0.991	1.006	0.995	1.018	1.029	0.990	1.010	1.001	0.993	0.973	1.026	Q13618	CUL3	Cullin-3
1.025	1.001	1.019	0.998	1.004	1.014	0.963	0.976	0.983	0.983	1.010	1.017	Q13619	CUL4A	Cullin-4A
0.994	0.986	1.024	1.019	1.068	1.018	0.995	0.962	1.031	0.994	1.007	1.020	Q13620	CUL4B	Cullin-4B

Abundance Ratio														
1 d M1/C1	1 d M2/C2	1 d M3/C3	4 d M1/C1	4 d M2/C2	4 d M3/C3	7 d M1/C1	7 d M2/C2	7 d M3/C3	10 d M1/C1	10 d M2/C2	10 d M3/C3	Accession ID	Gene name	Protein Description
1.004	1.000	1.015	0.994	0.970	1.026	0.981	1.009	0.996	0.991	0.981	1.004	Q93034	CUL5	Cullin-5
1.081	0.946	1.051	1.010	0.945	0.978	0.976	1.098	0.985				Q8IWT3	CUL9	Cullin-9
0.988	0.999	0.995	0.999	1.024	1.000	0.993	1.015	1.024	0.974	0.986	0.983	Q86VP6	CAND1	Cullin-associated NEDD8-dissociated protein 1
0.980	0.964	0.955	1.014	0.942	1.059	1.016	1.050	1.038	1.017	0.970	1.053	O75155	CAND2	Cullin-associated NEDD8-dissociated protein 2
1.019	0.981	1.006	1.076	0.977	1.050	1.047	1.017	1.024	0.997	1.000	1.034	Q69YN2	CWF19L1	CWF19-like protein 1
1.025	1.032	1.004	1.077	1.107	1.140	1.062	0.953	0.904	1.151	0.894	1.119	Q2TBE0	CWF19L2	CWF19-like protein 2
0.999	0.907	1.072	0.960	1.014	1.025	1.071	0.794	0.888	1.082	1.050	1.018	P56277	CMC4	Cx9C motif-containing protein 4
									0.980	0.838	1.004	P80162	CXCL6	C-X-C motif chemokine 6
1.127	0.897	0.982	1.161	0.816	1.025	1.106	0.919	0.985	1.108	0.940	1.033	P18846	ATF1	Cyclic AMP-dependent transcription factor ATF-1
0.982	1.003	1.044	1.008	0.852	0.967	0.945	1.146	0.952	1.046	0.976	1.035	P15336	ATF2	Cyclic AMP-dependent transcription factor ATF-2
						1.057	1.236	0.948	1.088	0.926	0.836	P18847	ATF3	Cyclic AMP-dependent transcription factor ATF-3
0.974	0.996	1.019	0.987	0.943	1.010	1.000	0.939	0.949	1.110	1.039	1.071	P17544	ATF7	Cyclic AMP-dependent transcription factor ATF-7
0.978	0.997	1.066	0.919	0.962	0.998	0.915	0.976	1.082	0.964	1.034	1.080	P16220	CREB1	Cyclic AMP-responsive element-binding protein 1
1.050	0.977	1.046	0.967	0.985	1.003	0.988	1.009	1.048	1.016	0.963	1.087	Q8N884	MB21D1	Cyclic GMP-AMP synthase
0.911	1.045	0.988	0.989	0.904	1.034	1.069	1.103	0.975	0.922	0.898	1.080	P20248	CCNA2	Cyclin-A2
0.844	0.930	0.575	1.135	1.173	1.078	0.904	1.140	0.747	0.668	0.760	0.950	P24863	CCNC	Cyclin-C
0.900	1.045	1.012	0.706	0.955	1.069	0.812	1.077	1.056	0.993	1.069	0.979	O95273	CCNDBP1	Cyclin-D1-binding protein 1
0.958	0.998	0.994	0.983	0.943	0.976	1.043	1.072	0.990	0.918	0.860	1.012	P06493	CDK1	Cyclin-dependent kinase 1
0.827	1.050	1.026	0.709	0.968	1.191	0.943	0.601	1.256	1.300	0.980	1.306	Q15131	CDK10	Cyclin-dependent kinase 10
1.100	1.096	0.948	1.047	0.957	0.870	0.846	1.102	0.947	0.990	0.884	1.049	Q9UQ88	CDK11A	Cyclin-dependent kinase 11A
									1.196	0.950	0.978	A0A087X1I0	CDK11B	Cyclin-dependent kinase 11B
0.995	0.999	0.997	0.983	0.954	1.016	1.037	1.034	0.990	1.028	0.988	1.013	J3QR44	CDK11B	Cyclin-dependent kinase 11B
1.011	1.021	1.000	1.007	0.947	1.026	1.031	1.053	0.976	1.009	1.008	1.030	Q9NYV4	CDK12	Cyclin-dependent kinase 12
1.049	1.036	1.020	1.040	0.941	1.173	0.710	0.796	1.256				Q14004	CDK13	Cyclin-dependent kinase 13
1.013	0.992	1.029	1.010	0.932	1.098	1.066	1.048	0.995	1.073	1.018	1.097	Q00537	CDK17	Cyclin-dependent kinase 17
0.966	1.055	1.066	0.970	0.932	1.018	0.896	1.124	1.055	0.932	1.044	1.017	Q9BWU1	CDK19	Cyclin-dependent kinase 19
0.964	1.003	0.963	1.014	0.926	0.995	1.015	1.076	0.979	0.954	0.935	0.967	G3V5T9	CDK2	Cyclin-dependent kinase 2
1.016	0.992	1.033	0.993	1.018	1.049	1.106	1.075	1.065	1.120	1.042	1.082	O14519	CDK2AP1	Cyclin-dependent kinase 2-associated protein 1
1.008	0.952	1.000	1.001	0.978	1.016	0.975	1.048	1.113	0.979	0.994	0.974	P42773	CDKN2C	Cyclin-dependent kinase 4 inhibitor C
1.013	1.026	1.032	1.049	1.037	1.045	0.991	1.016	0.967	1.061	0.968	1.026	P11802	CDK4	Cyclin-dependent kinase 4
1.080	0.983	1.034	1.033	1.047	1.017	0.969	1.022	1.055	0.942	0.997	1.046	Q00534	CDK6	Cyclin-dependent kinase 6
1.017	0.956	1.021	0.998	0.995	1.021	1.037	1.067	1.020	0.992	0.944	0.976	P50613	CDK7	Cyclin-dependent kinase 7
1.025	1.122	1.084	0.949	0.913	1.011				1.103	0.906	0.981	P49336	CDK8	Cyclin-dependent kinase 8
						1.087	0.798	0.941	1.138	0.972	1.243	P38936	CDKN1A	Cyclin-dependent kinase inhibitor 1

Abundance Ratio														
1 d M1/C1	1 d M2/C2	1 d M3/C3	4 d M1/C1	4 d M2/C2	4 d M3/C3	7 d M1/C1	7 d M2/C2	7 d M3/C3	10 d M1/C1	10 d M2/C2	10 d M3/C3	Accession ID	Gene name	Protein Description
1.091	0.968	1.076	1.077	1.055	1.064	0.958	0.883	0.921	1.111	1.008	1.182	P46527	CDKN1B	Cyclin-dependent kinase inhibitor 1B
1.006	1.037	0.951	0.944	0.980	0.876	1.071	1.057	0.908	1.037	0.830	1.101	P61024	CKS1B	Cyclin-dependent kinases regulatory subunit 1
1.057	0.986	1.139	0.995	0.954	1.003	1.006	1.062	0.993	0.914	0.907	1.154	P33552	CKS2	Cyclin-dependent kinases regulatory subunit 2
1.000	1.026	0.992	0.994	0.988	0.998	0.947	1.012	0.996	0.961	0.974	0.951	Q00535	CDK5	Cyclin-dependent-like kinase 5
1.019	1.001	1.052	1.000	0.949	1.090	1.005	1.024	1.040	1.054	1.012	1.091	O14976	GAK	Cyclin-G-associated kinase
0.994	0.970	0.948	1.137	1.006	1.023	1.091	0.977	1.010	0.974	0.940	1.001	P51946	CCNH	Cyclin-H
1.088	1.032	1.028	1.023	0.972	0.969	0.982	0.966	0.983	0.938	1.041	0.979	Q9UK58	CCNL1	Cyclin-L1
0.962	0.955	1.163	0.979	0.878	1.001	0.925	1.587	1.462	0.945	1.027	1.074	Q96S94	CCNL2	Cyclin-L2
1.074	0.878	0.915				0.789	0.635	0.984	0.897	0.903	1.052	Q8N1B3	FAM58A	Cyclin-related protein FAM58A
1.024	1.016	1.015	0.986	0.991	1.033	1.032	0.939	0.977	1.064	1.059	1.051	O60563	CCNT1	Cyclin-T1
1.000	0.988	1.015	0.928	0.926	0.912	0.912	1.048	1.017	0.835	1.011	0.836	Q8ND76	CCNY	Cyclin-Y
1.041	1.038	1.024	0.959	0.963	1.104	1.062	1.095	1.032	1.103	1.044	1.029	Q8N7R7	CCNYL1	Cyclin-Y-like protein 1
1.018	0.934	1.042	0.985	0.919	0.976	0.974	1.095	1.061	1.040	1.014	1.074	P32929	CTH	Cystathionine gamma-lyase
						0.968	1.118	0.933	0.972	1.030	0.739	P01040	CSTA	Cystatin-A
1.073	1.017	0.974	1.114	0.987	0.920	1.016	1.042	0.929	1.086	0.948	0.881	P04080	CSTB	Cystatin-B
1.021	0.985	1.024	0.857	0.910	1.038	1.052	1.088	1.021	1.029	1.048	1.190	P01034	CST3	Cystatin-C
1.093	1.033	0.961	1.032	1.325	0.912	1.110	0.870	0.975	1.026	1.011	1.003	P21291	CSRP1	Cysteine and glycine-rich protein 1
1.016	0.958	1.018	0.981	1.037	0.975	1.019	0.889	0.961	0.980	0.982	0.976	Q16527	CSRP2	Cysteine and glycine-rich protein 2
0.971	0.996	1.018	0.995	1.104	0.953	0.960	0.877	0.986	0.944	0.998	0.979	Q9UHD1	CHORDC1	Cysteine and histidine-rich domain-containing protein 1
1.032	0.966	1.026	0.970	0.980	1.022	0.994	1.032	1.010	0.995	1.018	1.015	E9PJD7	CYHR1	Cysteine and histidine-rich protein 1
1.018	0.928	1.044	0.995	0.965	0.995	1.024	1.101	1.079	0.965	0.989	0.986	B7Z4W5	KYAT1	Cysteine conjugate-beta lyase cytoplasmic (Glutamine transaminase K, kyneurenine aminotransferase), isoform CRA b
0.957	1.001	0.986	0.966	0.947	0.987	1.019	1.045	1.041	1.055	1.040	1.064	Q9Y697	NFS1	Cysteine desulfurase, mitochondrial
									0.980	0.658	0.916	Q8WYN0	ATG4A	Cysteine protease ATG4A
1.173	1.066	1.095	1.051	1.107	0.968	0.952	1.254	0.986	0.971	0.853	1.164	Q96DT6	ATG4C	Cysteine protease ATG4C
1.058	1.120	1.119	0.916	0.895	1.203	1.233	0.983	0.904	1.323	0.983	1.185	Q9H1C7	CYSTM1	Cysteine-rich and transmembrane domain-containing protein 1
			1.097	1.013	1.047				0.975	1.069	0.997	Q9NZV1	CRIM1	Cysteine-rich motor neuron 1 protein
0.871	0.984	0.870	0.992	0.943	0.921	1.110	1.041	0.953	1.048	1.023	0.949	Q9P021	CRIPT	Cysteine-rich PDZ-binding protein
1.153	1.024	1.025	1.053	1.457	0.976	1.022	0.802	1.020	0.987	0.914	0.926	P50238	CRIP1	Cysteine-rich protein 1
1.001	0.981	0.962	0.988	0.989	0.896	0.961	0.993	0.907	0.966	1.024	0.984	P52943	CRIP2	Cysteine-rich protein 2
0.929	1.028	1.068	0.991	0.967	1.075	1.001	1.039	0.977	1.103	1.085	1.046	Q96HD1	CRELD1	Cysteine-rich with EGF-like domain protein 1
1.028	0.986	0.982				1.159	1.009	0.754				Q9UPY5	SLC7A11	Cystine/glutamate transporter

Abundance Ratio														
1 d M1/C1	1 d M2/C2	1 d M3/C3	4 d M1/C1	4 d M2/C2	4 d M3/C3	7 d M1/C1	7 d M2/C2	7 d M3/C3	10 d M1/C1	10 d M2/C2	10 d M3/C3	Accession ID	Gene name	Protein Description
0.913	0.974	0.740	0.878	0.825	1.180	1.085	1.092	0.993				P00156	MT-CYB	Cytochrome b
1.002	1.038	1.054	0.953	0.860	0.921	1.082	0.990	0.960	1.141	0.972	1.148	P13498	CYBA	Cytochrome b-245 light chain
0.981	0.989	1.019	0.987	0.949	0.977	1.016	1.009	0.967	1.058	0.935	1.075	P00167	CYB5A	Cytochrome b5
0.991	1.007	1.073	0.960	0.958	1.108	1.081	1.132	0.986	0.998	1.003	1.138	Q7L1T6	CYB5R4	Cytochrome b5 reductase 4
0.935	0.989	1.013	0.950	0.889	0.917	0.992	1.043	0.984	1.076	1.019	1.087	J3KNF8	CYB5B	Cytochrome b5 type B
1.003	0.999	0.987	0.991	1.028	0.975	1.011	1.004	1.012	1.027	1.019	1.018	P31930	UQCRC1	Cytochrome b-c1 complex subunit 1, mitochondrial
0.962	0.945	0.925	1.179	1.328	0.847	0.995	1.013	1.079				O14957	UQCR11	Cytochrome b-c1 complex subunit 10
1.015	0.999	1.006	0.983	0.977	0.979	0.987	1.006	0.996	1.025	1.012	0.984	P22695	UQCRC2	Cytochrome b-c1 complex subunit 2, mitochondrial
1.674	0.982	1.184	1.077	2.108	1.000	1.123	0.523	1.205	1.076	0.994	1.088	P07919	UQCRH	Cytochrome b-c1 complex subunit 6, mitochondrial
0.995	0.984	0.969	0.981	0.980	0.956	0.957	0.861	0.971	1.041	1.019	0.975	P14927	UQCRB	Cytochrome b-c1 complex subunit 7
0.943	1.007	0.987	1.008	0.961	1.009	0.997	0.965	0.971	1.144	1.040	1.043	O14949	UQCRQ	Cytochrome b-c1 complex subunit 8
0.979	1.032	0.925	0.997	1.096	1.132	1.070	1.063	1.072	1.336	1.041	1.080	Q9UDW1	UQCR10	Cytochrome b-c1 complex subunit 9
0.981	1.022	1.018	0.997	0.994	1.015	0.989	1.049	1.015	1.030	1.033	1.040	P47985	UQCRFS1	Cytochrome b-c1 complex subunit Rieske, mitochondrial
0.905	1.086	0.989	1.016	1.044	1.020	1.025	1.031	0.967	1.004	0.971	0.958	P99999	CYCS	Cytochrome c
1.053	0.968	0.992	1.032	0.948	1.097	1.083	0.984	1.018	1.055	1.016	1.056	Q9GZY4	COA1	Cytochrome c oxidase assembly factor 1 homolog
0.863	0.983	0.972	0.933	1.085	1.028	0.993	0.875	0.991	1.073	0.998	1.052	Q9Y2R0	COA3	Cytochrome c oxidase assembly factor 3 homolog, mitochondrial
1.003	1.062	1.118	0.925	1.185	1.102	1.019	0.783	0.817	1.033	1.120	0.960	Q86WW8	COA5	Cytochrome c oxidase assembly factor 5
0.990	1.000	0.937	1.013	0.979	1.034	1.005	0.995	1.005	1.036	0.989	0.998	Q96BR5	COA7	Cytochrome c oxidase assembly factor 7
1.096	1.106	1.008	1.008	1.124	0.990	1.063	1.194	1.008	1.044	1.106	0.981	Q9Y6N1	COX11	Cytochrome c oxidase assembly protein COX11, mitochondrial
1.014	1.037	1.003	0.991	0.945	0.978	0.977	1.029	1.013	1.039	0.859	1.007	Q96I36	COX14	Cytochrome c oxidase assembly protein COX14
0.974	1.010	1.005	1.153	0.806	0.926	0.985	0.992	1.065	1.090	0.928	1.013	Q7KZN9	COX15	Cytochrome c oxidase assembly protein COX15 homolog
0.917	0.929	0.895	1.012	0.885	1.010	0.970	1.011	1.044	1.098	0.964	1.072	Q49B96	COX19	Cytochrome c oxidase assembly protein COX19
						1.280	0.757	1.021	1.019	1.000	0.926	Q14061	COX17	Cytochrome c oxidase copper chaperone
0.987	0.986	1.008	1.003	0.806	1.023	0.953	1.163	0.866	0.962	0.911	1.017	P00395	MT-CO1	Cytochrome c oxidase subunit 1
0.903	0.965	0.976	0.971	0.837	1.024	1.024	1.028	1.045	1.012	0.985	0.971	P00403	MT-CO2	Cytochrome c oxidase subunit 2
0.942	1.005	0.985	0.981	1.006	0.989	0.986	0.913	0.968	1.033	1.010	1.011	P13073	COX4I1	Cytochrome c oxidase subunit 4 isoform 1, mitochondrial
1.040	0.993	0.980	1.017	1.010	0.973	1.030	1.084	1.008	1.007	1.005	0.955	P20674	COX5A	Cytochrome c oxidase subunit 5A, mitochondrial
1.032	0.994	0.980	0.996	1.032	0.950	1.014	0.927	0.975	1.053	1.032	1.037	P10606	COX5B	Cytochrome c oxidase subunit 5B, mitochondrial

Abundance Ratio														
1 d M1/C1	1 d M2/C2	1 d M3/C3	4 d M1/C1	4 d M2/C2	4 d M3/C3	7 d M1/C1	7 d M2/C2	7 d M3/C3	10 d M1/C1	10 d M2/C2	10 d M3/C3	Accession ID	Gene name	Protein Description
1.055	1.045	0.993	0.978	0.948	0.882	0.982	1.157	1.004	0.918	1.085	0.936	P12074	COX6A1	Cytochrome c oxidase subunit 6A1, mitochondrial
1.028	0.989	1.031	0.979	1.054	1.018	1.078	0.929	1.029	1.026	1.014	1.006	P14854	COX6B1	Cytochrome c oxidase subunit 6B1
1.032	0.965	0.963	0.990	1.080	0.953	0.867	0.890	0.957	0.990	0.946	0.915	P09669	COX6C	Cytochrome c oxidase subunit 6C
1.032	1.000	1.007	0.983	1.145	1.034	1.027	1.003	1.042	1.046	1.010	0.991	H0UI06	COX7A2	Cytochrome c oxidase subunit 7A2, mitochondrial
1.024	1.002	0.949	0.977	0.957	0.951	0.996	1.004	0.946	1.023	1.018	0.944	O14548	COX7A2L	Cytochrome c oxidase subunit 7A-related protein, mitochondrial
0.939	0.974	0.992	0.977	1.001	1.027	1.012	0.883	0.982	1.175	1.061	1.114	P24311	COX7B	Cytochrome c oxidase subunit 7B, mitochondrial
0.902	0.961	0.995	0.963	0.981	1.046	1.021	0.904	0.947	0.867	0.969	1.132	P15954	COX7C	Cytochrome c oxidase subunit 7C, mitochondrial
1.005	0.983	1.026	1.016	1.135	0.989	1.136	0.973	1.020	1.170	1.121	1.086	O00483	NDUFA4	Cytochrome c oxidase subunit NDUFA4
1.015	1.011	1.017	1.021	1.149	1.005	1.037	1.029	1.060	1.053	0.984	1.057	P08574	CYC1	Cytochrome c1, heme protein, mitochondrial
1.017	0.985	1.032	1.034	0.977	1.052	1.116	0.983	1.001	1.108	1.029	1.091	P53701	HCCS	Cytochrome c-type heme lyase
0.973	0.973	1.019	0.984	0.888	1.044	0.989	0.977	1.021	1.006	1.045	1.061	E9PHG5	CYP20A1	Cytochrome P450 20A1
1.120	0.940	1.207	0.982	0.992	1.107	0.867	1.033	1.136	0.958	0.905	1.058	Q15438	CYTH1	Cytohesin-1
						0.976	1.187	1.106				A0A0D9SFG6	CYTH2	Cytohesin-2
0.992	0.956	1.089	1.005	0.933	1.020	1.043	1.112	1.080	1.025	0.969	1.063	O43739	CYTH3	Cytohesin-3
0.966	1.060	0.977	1.025	0.957	1.026	1.040	0.979	0.956	1.030	1.041	1.009	Q8IUI8	CRLF3	Cytokine receptor-like factor 3
1.002	1.026	1.021	1.031	1.054	1.031	1.045	1.004	1.057	1.039	0.994	1.059	P21399	ACO1	Cytoplasmic aconitate hydratase
0.985	0.995	1.011	0.998	0.976	1.007	0.992	1.037	1.014	0.956	0.988	0.976	Q14204	DYNC1H1	Cytoplasmic dynein 1 heavy chain 1
1.011	1.018	0.989	1.000	1.036	0.989	1.012	1.055	1.018	0.988	0.986	0.995	Q9Y6G9	DYNC1LI1	Cytoplasmic dynein 1 light intermediate chain 1
0.987	0.989	1.005	0.983	1.000	0.989	0.994	0.976	1.007	0.988	0.998	0.996	O43237	DYNC1LI2	Cytoplasmic dynein 1 light intermediate chain 2
1.035	0.984	1.017	0.979	0.944	0.982	1.004	0.941	0.980	1.012	0.915	0.970	Q8TCX1	DYNC2LI1	Cytoplasmic dynein 2 light intermediate chain 1
0.985	0.998	1.001	0.990	0.974	1.017	0.969	1.065	1.024	0.958	1.012	0.979	Q7L576	CYFIP1	Cytoplasmic FMR1-interacting protein 1
1.014	1.046	1.062	0.929	1.002	1.015	0.971	1.070	1.027	0.976	0.959	1.095	Q9UKF7	PITPNC1	Cytoplasmic phosphatidylinositol transfer protein 1
0.918	1.173	0.962	0.862	0.838	1.155	0.989	1.456	0.995				Q17RY0	CPEB4	Cytoplasmic polyadenylation element-binding protein 4
0.994	1.030	0.973	1.006	1.046	0.975	1.025	1.062	1.027	1.007	0.967	1.007	P16333	NCK1	Cytoplasmic protein NCK1
1.025	0.998	0.978	1.020	0.984	1.019	1.044	1.039	1.089	1.027	0.990	1.052	O43639	NCK2	Cytoplasmic protein NCK2
1.020	1.033	1.104	0.980	0.901	1.064	0.975	1.009	1.015	1.044	1.052	1.087	Q7Z7A3	CTU1	Cytoplasmic tRNA 2-thiolation protein 1
0.968	0.981	0.993	1.052	0.962	1.057	1.000	1.005	1.043	1.043	1.021	1.059	H3BSW6	CTU2	Cytoplasmic tRNA 2-thiolation protein 2
0.956	1.057	1.024	0.987	0.907	0.997	0.947	1.172	0.914	0.867	0.954	0.921	Q8WWK9	CKAP2	Cytoskeleton-associated protein 2
1.047	1.018	0.985										Q8IYA6	CKAP2L	Cytoskeleton-associated protein 2-like
0.966	0.977	1.013	0.992	0.936	1.011	0.993	0.987	0.983	0.996	0.994	1.026	Q07065	CKAP4	Cytoskeleton-associated protein 4
0.971	0.989	1.008	0.979	0.955	0.993	0.977	1.036	1.008	0.954	0.953	0.991	Q14008	CKAP5	Cytoskeleton-associated protein 5

Abundance Ratio														
1 d M1/C1	1 d M2/C2	1 d M3/C3	4 d M1/C1	4 d M2/C2	4 d M3/C3	7 d M1/C1	7 d M2/C2	7 d M3/C3	10 d M1/C1	10 d M2/C2	10 d M3/C3	Accession ID	Gene name	Protein Description
1.009	0.996	1.004	1.004	1.064	0.974	1.003	0.985	1.009	1.007	0.991	1.023	P28838	LAP3	Cytosol aminopeptidase
0.986	1.001	0.980	0.995	0.957	1.005	0.973	1.057	1.057	0.991	0.957	1.031	Q9H0P0	NT5C3A	Cytosolic 5'-nucleotidase 3A
0.964	0.992	1.003	0.978	0.990	0.965	0.961	0.953	0.963	0.976	0.987	1.012	O00154	ACOT7	Cytosolic acyl coenzyme A thioester hydrolase
			0.933	0.974	1.039	1.060	1.161	1.130	0.896	1.015	0.937	Q8WTX7	CASTOR1	Cytosolic arginine sensor for mTORC1 subunit 1
1.099	1.001	1.001	1.017	0.993	0.971	0.974	1.081	1.037	1.005	1.043	1.041	A6NHX0	CASTOR2	Cytosolic arginine sensor for mTORC1 subunit 2
1.085	1.024	1.176	1.017	1.119	1.144	1.099	0.707	0.837				J3KNS1	AGTPBP1	Cytosolic carboxypeptidase 1
1.040	0.965	1.023	0.991	0.931	1.059	0.995	1.123	1.022	0.991	1.007	1.061	Q8NFI3	ENGASE	Cytosolic endo-beta-N-acetylglucosaminidase
1.097	1.096	1.023	1.073	1.008	0.967	1.074	1.156	0.993	1.113	1.038	1.112	Q9H6Q4	NARFL	Cytosolic Fe-S cluster assembly factor NARFL
1.204	0.947	1.028	0.964	0.933	0.938	1.054	0.956	1.000	0.950	0.943	1.002	P53384	NUBP1	Cytosolic Fe-S cluster assembly factor NUBP1
0.987	0.994	1.019	0.975	0.929	0.979	0.951	1.088	1.009	0.961	0.984	1.026	Q9Y5Y2	NUBP2	Cytosolic Fe-S cluster assembly factor NUBP2
0.987	0.994	0.997	0.998	1.012	1.002	0.980	1.032	1.025	0.984	1.016	0.969	Q96KP4	CNDP2	Cytosolic non-specific dipeptidase
						1.082	1.072	1.161				P47712	PLA2G4A	Cytosolic phospholipase A2
0.989	1.008	1.020	0.998	0.998	1.038	0.984	1.095	1.013	0.991	0.958	0.989	P49902	NT5C2	Cytosolic purine 5'-nucleotidase
1.000	1.008	1.026	0.969	0.941	0.989	1.049	0.970	1.016	1.086	0.973	1.131	Q69YQ0	SPECC1L	Cytospin-A
0.912	0.957	1.053	0.956	0.964	1.052	1.004	0.948	0.996	1.029	0.908	1.087	Q5M775	SPECC1	Cytospin-B
0.933	1.051	1.029	1.000	0.954	1.009	1.029	1.013	1.024	1.084	1.008	1.009	Q8N465	D2HGDH	D-2-hydroxyglutarate dehydrogenase, mitochondrial
0.930	1.005	0.965	0.993	1.008	0.996	0.990	1.015	1.013	0.920	0.962	0.981	O43175	PHGDH	D-3-phosphoglycerate dehydrogenase
0.900	0.867	0.973	0.998	0.872	0.995	0.916	1.095	1.174	0.932	0.967	1.095	Q5D0E6	DALRD3	DALR anticodon-binding domain-containing protein 3
0.961	0.989	0.990	0.994	1.001	0.990	1.005	1.049	1.027	0.916	0.976	0.935	Q96EP5	DAZAP1	DAZ-associated protein 1
0.936	0.957	0.990	0.970	0.953	0.947	0.976	1.072	0.978	1.021	0.917	1.046	Q02338	BDH1	D-beta-hydroxybutyrate dehydrogenase, mitochondrial
0.972	1.005	0.979	1.028	1.039	0.951	1.021	0.976	0.966	0.935	1.001	0.969	Q5BKZ1	ZNF326	DBIRD complex subunit ZNF326
1.001	0.974	1.034	1.018	1.008	0.992	1.026	1.025	1.028	0.996	1.024	0.997	Q9UKG1	APPL1	DCC-interacting protein 13-alpha
1.026	0.981	0.996	1.001	1.032	1.011	0.994	1.019	1.052	0.987	0.980	0.992	Q96GG9	DCUN1D1	DCN1-like protein 1
0.947	1.033	1.005	0.907	0.985	1.042	0.931	1.025	0.972	1.077	1.157	1.087	Q8IWE4	DCUN1D3	DCN1-like protein 3
1.030	0.987	1.000	0.998	0.990	0.996	1.045	1.014	1.003	0.996	0.970	1.016	Q9BTE7	DCUN1D5	DCN1-like protein 5
0.972	0.975	1.045	0.972	1.003	1.035	0.987	1.000	1.070	0.978	0.986	1.110	Q9H773	DCTPP1	dCTP pyrophosphatase 1
1.020	0.974	1.024	1.016	0.966	0.995	1.043	1.027	1.053	0.960	1.003	1.012	Q9Y4B6	DCAF1	DDB1- and CUL4-associated factor 1
1.217	1.055	0.982							0.894	1.082	1.063	Q5QP82	DCAF10	DDB1- and CUL4-associated factor 10
1.011	0.981	1.066	0.957	0.921	1.052	1.115	1.061	1.056	1.015	0.971	1.061	Q8TEB1	DCAF11	DDB1- and CUL4-associated factor 11
1.047	0.979	1.012	1.031	1.013	1.030	1.033	1.003	1.011	1.035	1.030	1.041	A0A087WT20	DCAF13	DDB1- and CUL4-associated factor 13
0.988	1.047	0.936	1.023	0.917	0.940	0.980	1.078	1.327				Q9NXF7	DCAF16	DDB1- and CUL4-associated factor 16
			1.025	0.808	0.835	0.811	1.210	0.928				Q8WV16	DCAF4	DDB1- and CUL4-associated factor 4

Abundance Ratio														
1 d M1/C1	1 d M2/C2	1 d M3/C3	4 d M1/C1	4 d M2/C2	4 d M3/C3	7 d M1/C1	7 d M2/C2	7 d M3/C3	10 d M1/C1	10 d M2/C2	10 d M3/C3	Accession ID	Gene name	Protein Description
1.021	1.010	1.041	0.981	0.933	1.070	1.008	0.962	1.023	0.959	0.979	1.102	P61962	DCAF7	DDB1- and CUL4-associated factor 7
1.037	0.971	0.971	0.993	0.951	1.014	0.987	1.061	1.011	0.964	0.955	0.983	J3KQ18	DDT	D-dopachrome decarboxylase
0.959	0.972	1.035	0.966	0.926	1.065	0.963	0.968	1.023	1.026	1.023	1.072	Q96HY6	DDRGK1	DDRGK domain-containing protein 1
1.014	0.946	0.944	0.962	0.981	0.991	0.984	1.031	1.042	0.983	0.993	1.075	Q9UER7	DAXX	Death domain-associated protein 6
1.005	1.088	1.186	0.957	0.944	1.073	0.975	1.089	0.953	0.956	1.016	1.002	P78560	CRADD	Death domain-containing protein CRADD
1.027	0.933	1.004	0.894	0.991	0.969	0.878	0.689	0.982	1.018	0.927	1.002	P51397	DAP	Death-associated protein 1
0.974	0.971	1.024	0.984	0.944	1.024	1.024	1.041	0.968	0.982	0.961	1.017	O43293	DAPK3	Death-associated protein kinase 3
1.011	1.000	1.013	0.984	0.943	0.972	1.001	0.955	0.981	1.040	1.038	1.065	Q9BTC0	DIDO1	Death-inducer obliterator 1
1.106	1.124	1.262	1.001	0.775	0.980	0.935	0.821	0.949	0.711	0.908	1.087	O77932	DXO	Decapping and exoribonuclease protein
1.062	1.083	0.947	0.946	1.010	1.037	0.961	0.968	0.987				Q86YH6	PDSS2	Decaprenyl-diphosphate synthase subunit 2
1.020	1.019	1.029	1.011	0.936	0.995	1.016	1.044	1.008	0.981	0.962	1.018	Q14185	DOCK1	Dedicator of cytokinesis protein 1
1.007	1.012	0.966	1.109	0.872	1.029							Q96BY6	DOCK10	Dedicator of cytokinesis protein 10
1.079	0.982	1.031	1.003	0.957	0.988	1.089	0.953	1.008	1.043	0.937	1.035	A6NIW2	DOCK11	Dedicator of cytokinesis protein 11
0.995	1.173	1.092	0.966	1.064	1.169							Q8IZD9	DOCK3	Dedicator of cytokinesis protein 3
1.026	0.988	1.047	1.018	0.970	1.032	0.999	1.059	1.023	1.006	0.949	1.008	Q8N1I0	DOCK4	Dedicator of cytokinesis protein 4
0.991	1.019	1.042	1.008	0.931	1.021	1.020	1.137	0.995	0.959	1.012	0.999	Q9H7D0	DOCK5	Dedicator of cytokinesis protein 5
0.999	0.995	1.022	1.013	0.951	1.013	0.996	1.022	1.042	0.993	0.990	1.026	Q96N67	DOCK7	Dedicator of cytokinesis protein 7
1.055	1.024	1.038	1.038	0.936	1.031	1.055	1.038	1.085	1.031	0.984	1.113	A0A0A0MSY4	DOCK9	Dedicator of cytokinesis protein 9
1.243	1.063	1.042	0.795	0.981	1.239	0.992	1.330	1.163				Q96E22	NUS1	Dehydrodolichyl diphosphate synthase complex subunit NUS1
0.984	1.003	0.968	0.968	0.934	0.989	0.995	0.968	0.973	1.023	0.920	1.027	Q96LJ7	DHRS1	Dehydrogenase/reductase SDR family member 1
1.046	0.981	0.940	1.028	1.108	0.915	1.015	1.067	1.037	1.017	1.040	0.982	Q6UWP2	DHRS11	Dehydrogenase/reductase SDR family member 11
1.143	1.047	1.112	1.136	0.973	1.001	1.082	1.138	1.063	1.082	1.002	1.034	Q6UX07	DHRS13	Dehydrogenase/reductase SDR family member 13
1.027	1.073	1.032	0.977	0.872	0.957	1.022	1.216	0.947	0.896	1.058	0.964	Q13268	DHRS2	Dehydrogenase/reductase SDR family member 2, mitochondrial
1.045	1.018	1.053	1.016	1.114	1.028	1.045	1.016	1.022	0.997	1.001	1.039	Q9BTZ2	DHRS4	Dehydrogenase/reductase SDR family member 4
0.962	1.054	0.983	0.992	0.927	0.990	0.982	1.058	0.978	1.002	1.039	0.973	Q9Y394	DHRS7	Dehydrogenase/reductase SDR family member 7
1.107	0.905	1.010	1.097	0.803	1.014	1.152	1.009	0.987	1.072	0.944	1.020	Q6IAN0	DHRS7B	Dehydrogenase/reductase SDR family member 7B
0.936	1.060	1.051	0.996	1.040	0.881	0.898	1.273	0.967	0.913	0.954	0.864	Q8N5I4	DHRSX	Dehydrogenase/reductase SDR family member on chromosome X
0.999	1.101	1.039				1.011	1.128	0.936	0.893	0.897	0.997	Q8NDZ4	C3orf58	Deleted in autism protein 1
0.947	1.049	0.986	0.951	0.822	1.040	0.881	1.011	1.034	1.218	1.014	1.230	O76062	TM7SF2	Delta(14)-sterol reductase
0.969	0.971	1.042	1.049	1.004	1.001	1.146	1.018	1.130	1.241	1.062	1.120	Q15392	DHCR24	Delta(24)-sterol reductase
1.030	0.995	1.007	1.020	1.097	0.984	1.001	0.933	0.977	1.016	0.995	0.978	Q13011	ECH1	Delta(3,5)-Delta(2,4)-dienoyl-CoA isomerase, mitochondrial

Abundance Ratio														
1 d M1/C1	1 d M2/C2	1 d M3/C3	4 d M1/C1	4 d M2/C2	4 d M3/C3	7 d M1/C1	7 d M2/C2	7 d M3/C3	10 d M1/C1	10 d M2/C2	10 d M3/C3	Accession ID	Gene name	Protein Description
1.024	1.004	0.995	0.999	0.977	0.998	0.992	0.990	0.990	1.052	0.979	1.013	P30038	ALDH4A1	Delta-1-pyrroline-5-carboxylate dehydrogenase, mitochondrial
0.967	0.986	0.990	0.980	0.982	1.013	0.975	1.009	1.006	1.009	0.993	1.013	P54886	ALDH18A1	Delta-1-pyrroline-5-carboxylate synthase
0.999	0.998	1.020	1.015	1.034	0.994	1.019	1.026	0.974	1.010	0.942	1.017	P13716	ALAD	Delta-aminolevulinic acid dehydratase
1.019	1.022	1.004	0.985	0.982	1.087	1.083	1.015	0.998	1.064	0.987	1.085	Q8TEH3	DENND1A	DENN domain-containing protein 1A
0.980	1.120	0.882	1.065	0.988	1.021	0.686	1.038	1.069	1.173	0.747	1.097	Q9ULE3	DENND2A	DENN domain-containing protein 2A
1.007	1.009	1.032	1.018	1.019	1.027	1.018	1.025	1.021	1.019	0.987	1.059	E9PF32	DENND3	DENN domain-containing protein 3
			0.944	0.901	1.022				1.033	1.087	1.009	O75064	DENND4B	DENN domain-containing protein 4B
1.043	1.043	1.024	1.000	0.983	0.989	0.995	1.082	0.984	0.951	0.972	1.004	R4GN35	DENND4C	DENN domain-containing protein 4C
			1.037	1.008	0.892	0.957	1.024	1.055				Q6IQ26	DENND5A	DENN domain-containing protein 5A
0.972	0.981	1.014	0.992	1.019	1.063	1.246	1.174	1.192	1.054	1.016	1.009	G3V1S3	DENND5B	DENN domain-containing protein 5B
1.013	0.973	1.008	0.980	0.965	1.013	1.011	1.039	1.003	0.999	0.985	1.000	O43583	DENR	Density-regulated protein
									1.151	0.947	1.330	Q9NZJ0	DTL	Denticleless protein homolog
1.088	0.988	1.061	1.029	0.924	1.040	1.010	0.990	1.021	1.030	1.120	0.947	D6RFG8	DCK	Deoxycytidine kinase
1.025	1.002	0.991	0.951	0.953	0.971	0.971	1.038	1.030	0.991	0.988	0.928	Q16854	DGUOK	Deoxyguanosine kinase, mitochondrial
0.975	0.966	0.990	0.973	0.959	1.039	0.966	1.049	1.044	0.990	0.989	1.007	Q9BU89	DOHH	Deoxyhypusine hydroxylase
1.086	1.024	1.056	0.946	0.957	1.038	0.948	1.082	1.021	0.910	0.998	0.996	P49366	DHPS	Deoxyhypusine synthase
0.988	0.997	0.987	1.011	0.982	0.999	1.031	1.005	0.979	1.037	0.965	1.029	Q9Y3Z3	SAMHD1	Deoxynucleoside triphosphate triphosphohydrolase SAMHD1
0.994	0.991	1.015	1.000	0.961	1.021	0.972	1.031	0.949	0.970	0.980	0.989	Q9H147	DNTTIP1	Deoxynucleotidyltransferase terminal-interacting protein 1
0.991	0.982	1.004	0.968	0.977	1.029	0.971	0.945	0.991	1.000	1.026	1.021	Q5QJE6	DNTTIP2	Deoxynucleotidyltransferase terminal-interacting protein 2
0.945	0.855	1.205	0.866	0.967	0.719	1.190	1.027	0.958	0.974	1.058	0.937	P49184	DNASE1L1	Deoxyribonuclease-1-like 1
1.030	0.985	1.005	1.119	1.064	1.078	1.200	1.152	1.135	1.265	1.166	1.228	O00115	DNASE2	Deoxyribonuclease-2-alpha
1.045	0.973	1.025	1.020	0.984	1.004	0.979	1.005	1.003	0.920	1.015	0.970	Q9Y315	DERA	Deoxyribose-phosphate aldolase
0.926	0.996	0.985	0.971	1.017	0.983	0.978	0.934	1.019	1.042	0.933	1.096	P33316	DUT	Deoxyuridine 5'-triphosphate nucleotidohydrolase, mitochondrial
1.086	1.090	1.073	0.992	1.044	0.977	1.015	0.929	1.027	1.134	1.019	1.095	Q8TB45	DEPTOR	DEP domain-containing mTOR-interacting protein
0.820	0.995	1.001										Q8WUY9	DEPDC1B	DEP domain-containing protein 1B
1.083	1.128	1.110	1.032	0.978	1.021	0.975	1.094	1.108	0.917	0.991	1.075	O75140	DEPDC5	DEP domain-containing protein 5
0.975	0.952	1.094	0.995	0.825	1.041	1.052	1.050	0.987	0.998	0.926	1.025	Q8WVC6	DCAKD	Dephospho-CoA kinase domain-containing protein
1.001	1.041	0.978	0.960	0.936	0.996	1.000	1.102	1.047	1.054	1.036	1.061	Q9BUN8	DERL1	Derlin-1
1.019	0.933	0.992	1.070	1.063	1.143	1.101	1.178	1.194	1.027	1.000	1.029	Q9GZP9	DERL2	Derlin-2
1.163	1.132	1.190							1.107	1.049	0.869	Q9UL01	DSE	Dermatan-sulfate epimerase

Abundance Ratio														
1 d M1/C1	1 d M2/C2	1 d M3/C3	4 d M1/C1	4 d M2/C2	4 d M3/C3	7 d M1/C1	7 d M2/C2	7 d M3/C3	10 d M1/C1	10 d M2/C2	10 d M3/C3	Accession ID	Gene name	Protein Description
1.101	1.206	1.158	0.991	1.082	0.959	0.924	0.842	0.771	0.941	1.063	0.842	P81605	DCD	Dermcidin
1.104	0.975	0.986	0.914	0.855	0.939	0.875	0.941	0.953	1.026	1.012	0.941	P17661	DES	Desmin
0.966	0.944	1.007	0.962	0.916	0.985	1.002	1.031	0.923	0.997	0.823	0.957	Q02487	DSC2	Desmocollin-2
0.975	1.007	1.015	1.128	1.066	1.041	1.122	0.957	1.074	0.985	0.920	0.948	Q14574	DSC3	Desmocollin-3
			0.838	0.959	1.101	0.931	0.741	0.875	0.951	1.242	1.015	Q02413	DSG1	Desmoglein-1
0.949	0.959	0.998	0.981	0.918	0.991	1.027	1.009	1.014	1.015	1.006	1.059	Q14126	DSG2	Desmoglein-2
0.968	0.978	1.015	0.990	0.986	1.007	0.974	0.968	0.988	1.015	1.014	1.011	P15924	DSP	Desmoplakin
0.992	1.008	0.970	0.997	1.045	0.998	1.058	0.960	1.033	0.982	0.971	0.986	P60981	DSTN	Destrin
									1.073	0.949	1.176	Q6ICB0	DESI1	Desumoylating isopeptidase 1
						1.023	1.111	1.092	1.253	0.988	1.094	Q9BSY9	DESI2	Desumoylating isopeptidase 2
1.036	0.985	1.040	0.961	0.893	1.035	1.023	1.050	0.980	1.005	0.959	1.005	Q9BW61	DDA1	DET1- and DDB1-associated protein 1
1.024	1.007	1.018	0.998	0.962	1.006	1.028	1.059	1.054	1.025	0.981	1.083	Q96JH7	VCPIP1	Deubiquitinating protein VCIP135
1.012	0.994	0.991	1.018	1.041	0.996	1.004	1.047	1.052	0.986	0.984	1.018	Q9Y295	DRG1	Developmentally-regulated GTP-binding protein 1
0.974	0.992	1.033	1.005	0.999	1.034	0.997	1.056	1.039	0.984	1.004	1.015	P55039	DRG2	Developmentally-regulated GTP-binding protein 2
1.036	1.043	0.902	1.016	1.079	0.989	1.083	0.823	0.779	0.997	0.895	0.991	O94923	GLCE	D-glucuronyl C5-epimerase
1.047	1.021	1.028	0.994	0.966	1.013	0.981	0.950	0.966	1.001	1.015	1.057	Q9NR28	DIABLO	Diablo homolog, mitochondrial
1.014	1.003	1.006	1.007	0.958	0.982	1.007	1.013	1.029	0.957	0.953	1.007	P23743	DGKA	Diacylglycerol kinase alpha
1.173	0.998	1.023	1.082	0.999	1.088	1.015	1.089	1.011	0.983	0.997	1.021	P52429	DGKE	Diacylglycerol kinase epsilon
1.498	0.613	1.062	1.009	1.030	1.027	0.930	1.122	0.930	1.108	0.915	1.000	Q86XP1	DGKH	Diacylglycerol kinase eta
1.030	0.995	1.178										P52824	DGKQ	Diacylglycerol kinase theta
1.016	0.941	1.105	1.046	0.859	1.086	0.898	1.005	1.123	1.107	0.944	1.077	Q13574	DGKZ	Diacylglycerol kinase zeta
									1.310	1.379	1.019	O75907	DGAT1	Diacylglycerol O-acyltransferase 1
1.102	1.052	1.179				0.984	1.072	1.053	1.033	1.002	1.011	Q96F10	SAT2	Diamine acetyltransferase 2
1.018	1.002	1.012	0.996	0.992	1.032	1.012	1.007	1.031	0.998	0.955	1.044	K4DI95	DIAPH2	Diaphanous homolog 2 (Drosophila), isoform CRA_a
1.068	0.880	1.076	0.994	0.928	1.126				0.954	0.960	1.111	Q6ZN54	DEF8	Differentially expressed in FDCP 8 homolog
1.013	1.044	1.031	0.984	0.936	0.992	0.989	1.068	1.024	0.985	0.977	1.024	Q68CQ4	DIEXF	Digestive organ expansion factor homolog
0.976	0.993	0.966	1.048	0.984	0.992	1.175	1.073	1.158	0.916	0.852	1.047	P00374	DHFR	Dihydrofolate reductase
0.942	0.987	1.008	0.953	1.006	1.006	0.960	0.872	0.985	0.978	1.013	1.015	P09622	DLD	Dihydrolipoyl dehydrogenase, mitochondrial
1.039	1.002	1.044	1.014	0.994	0.983	1.000	1.004	1.010	0.990	0.961	0.957	P10515	DLAT	Dihydrolipoyllysine-residue acetyltransferase component of pyruvate dehydrogenase complex, mitochondrial
1.004	0.982	1.005	0.968	0.944	0.977	0.968	0.985	0.975	0.967	1.008	0.985	P36957	DLST	Dihydrolipoyllysine-residue succinyltransferase component of 2-oxoglutarate dehydrogenase complex, mitochondrial

Abundance Ratio														
1 d M1/C1	1 d M2/C2	1 d M3/C3	4 d M1/C1	4 d M2/C2	4 d M3/C3	7 d M1/C1	7 d M2/C2	7 d M3/C3	10 d M1/C1	10 d M2/C2	10 d M3/C3	Accession ID	Gene name	Protein Description
1.016	0.995	0.991	1.022	0.993	1.010	1.012	1.058	1.035	0.999	0.995	1.007	Q02127	DHODH	Dihydroorotate dehydrogenase (quinone), mitochondrial
0.983	1.020	0.986	1.014	0.973	0.986	1.032	1.073	0.988	0.987	1.029	1.068	P09417	QDPR	Dihydropteridine reductase
0.862	0.980	0.923	1.146	1.028	1.043							Q14117	DPYS	Dihydropyrimidinase
0.987	0.992	0.993	1.011	1.032	0.990	1.015	1.019	1.010	1.011	0.996	1.002	A0A1C7CYX9	DPYSL2	Dihydropyrimidinase-related protein 2
0.742	1.008	0.803	0.954	0.952	1.000							O14531	DPYSL4	Dihydropyrimidinase-related protein 4
2.577	0.593	1.069				1.029	0.982	0.964	0.922	1.208	0.902	Q9BPU6	DPYSL5	Dihydropyrimidinase-related protein 5
1.190	1.072	1.057	1.104	1.076	1.085	1.103	1.179	1.136	1.239	1.212	1.217	Q12882	DPYD	Dihydropyrimidine dehydrogenase [NADP(+)]
0.984	0.990	1.024	0.985	0.941	1.022	0.981	1.065	1.016	0.967	0.984	0.975	O15228	GNPAT	Dihydroxyacetone phosphate acyltransferase
1.000	0.990	0.986	0.997	0.945	0.972	1.036	1.000	1.073	1.037	0.990	1.045	Q8WVM0	TFB1M	Dimethyladenosine transferase 1, mitochondrial
0.980	1.033	1.044	1.018	0.996	1.080	1.113	1.130	1.059	1.109	1.043	1.173	Q9H5Q4	TFB2M	Dimethyladenosine transferase 2, mitochondrial
1.037	1.029	1.005	1.059	1.093	1.016	1.005	0.983	1.005	1.066	1.003	1.079	Q01459	CTBS	Di-N-acetylchitobiase
1.069	0.995	0.956	0.998	1.059	0.949	0.986	0.992	1.002	0.931	0.951	0.921	P53634	CTSC	Dipeptidyl peptidase 1
0.986	0.999	1.012	0.984	0.957	1.025	0.985	1.034	1.019	0.989	1.011	0.988	Q9UHL4	DPP7	Dipeptidyl peptidase 2
0.998	0.982	1.001	0.981	0.978	1.013	0.987	1.035	1.018	1.016	0.989	1.018	G3V180	DPP3	Dipeptidyl peptidase 3
0.779	0.906	1.147	0.956	0.917	0.952	0.965	0.991	0.990	0.968	0.857	1.086	P27487	DPP4	Dipeptidyl peptidase 4
1.095	0.973	0.983	0.995	0.844	0.999	1.023	1.062	1.019	0.930	1.095	0.999	Q6V1X1	DPP8	Dipeptidyl peptidase 8
1.043	0.920	1.019	0.974	0.942	1.017	1.050	0.978	0.997	1.059	1.006	1.076	O95989	NUDT3	Diphosphoinositol polyphosphate phosphohydrolase 1
1.084	0.938	1.025	0.988	0.940	1.135	0.989	1.015	1.076	1.169	0.962	1.059	Q9NZJ9	NUDT4	Diphosphoinositol polyphosphate phosphohydrolase 2
0.964	1.025	1.000	1.007	0.981	1.031	1.000	1.052	1.022	1.050	1.073	1.055	P53602	MVD	Diphosphomevalonate decarboxylase
1.080	0.935	1.003	1.082	1.112	0.961	0.947	1.224	0.994	1.222	0.964	1.085	Q9BTV6	DPH7	Diphthine methyltransferase
0.957	1.005	1.022	1.010	0.956	0.997	0.953	1.098	1.088	1.107	1.037	1.031	Q7L8W6	DPH6	Diphthine--ammonia ligase
0.756	0.749	0.937				0.976	1.214	1.164				Q8TF46	DIS3L	DIS3-like exonuclease 1
1.028	1.013	1.034	1.008	0.971	1.026	1.067	0.999	1.018	1.047	0.994	1.083	Q8IYB7	DIS3L2	DIS3-like exonuclease 2
0.984	0.910	0.970	1.039	0.995	1.033	0.989	1.050	1.132	0.868	0.854	1.064	P98082	DAB2	Disabled homolog 2
1.084	1.157	1.036	1.059	0.948	0.877	0.900	1.264	0.969				Q5VWQ8	DAB2IP	Disabled homolog 2-interacting protein
1.014	1.027	1.022	1.014	0.939	1.001	1.004	1.181	1.021	0.988	0.992	1.042	Q14689	DIP2A	Disco-interacting protein 2 homolog A
1.039	1.008	1.040	1.025	0.995	1.025	1.040	1.083	1.010	1.016	1.019	1.061	Q9P265	DIP2B	Disco-interacting protein 2 homolog B
1.109	0.821	1.177	0.956	1.063	1.053	0.990	1.000	1.047				Q5JUW8	DLG3	Discs, large homolog 3 (Neuroendocrine-dlg, Drosophila), isoform CRA_b
			0.860	0.861	1.220							Q9Y4D1	DAAM1	Disheveled-associated activator of morphogenesis 1
1.065	1.001	1.041	1.048	0.941	1.048	1.071	1.063	1.050	1.041	1.072	1.111	O14672	ADAM10	Disintegrin and metalloproteinase domain-containing protein 10

Abundance Ratio														
1 d M1/C1	1 d M2/C2	1 d M3/C3	4 d M1/C1	4 d M2/C2	4 d M3/C3	7 d M1/C1	7 d M2/C2	7 d M3/C3	10 d M1/C1	10 d M2/C2	10 d M3/C3	Accession ID	Gene name	Protein Description
0.935	0.998	1.029	1.136	0.983	1.209	1.120	1.065	0.980	1.034	1.222	1.208	Q13444	ADAM15	Disintegrin and metalloproteinase domain-containing protein 15
1.056	1.062	1.015	0.890	0.914	0.994	1.088	1.008	0.999				P78536	ADAM17	Disintegrin and metalloproteinase domain-containing protein 17
1.009	1.043	1.018	1.056	0.945	0.912	1.068	0.969	0.916	1.018	1.003	1.030	Q13443	ADAM9	Disintegrin and metalloproteinase domain-containing protein 9
0.963	0.940	1.062	0.944	0.871	1.064	0.984	0.920	1.072	1.144	0.842	1.182	Q8TDM6	DLG5	Disks large homolog 5
0.989	0.970	0.975	1.017	0.970	1.018	1.001	1.042	1.019	1.026	0.992	1.022	Q9Y2H0	DLGAP4	Disks large-associated protein 4
0.964	1.012	1.008	0.918	0.957	0.939	0.869	0.731	1.026	0.900	0.898	1.056	Q15398	DLGAP5	Disks large-associated protein 5
1.011	0.913	1.146				1.055	1.204	1.013	1.065	1.353	1.046	Q96SL1	DIRC2	Disrupted in renal carcinoma protein 2
1.081	1.002	1.027	1.023	0.986	1.029	0.985	1.001	1.010	0.968	0.969	1.021	F5H269	DMXL1	DmX-like protein 1
0.981	0.999	1.008	0.999	0.953	0.993	0.995	1.087	1.023	0.976	0.981	1.052	P26358	DNMT1	DNA (cytosine-5)-methyltransferase 1
			0.977	1.311	0.968	1.023	1.026	1.610				Q9Y6K1	DNMT3A	DNA (cytosine-5)-methyltransferase 3A
0.989	0.988	0.993	0.991	0.970	1.009	0.988	1.003	1.025	0.971	0.959	0.997	Q16531	DDB1	DNA damage-binding protein 1
1.000	1.037	1.002	1.005	0.922	1.005	1.038	1.102	1.037	0.956	0.912	1.050	Q92466	DDB2	DNA damage-binding protein 2
0.903	0.908	0.985	0.993	0.846	1.023	0.995	0.931	0.954	1.051	1.006	1.137	Q6UX65	DRAM2	DNA damage-regulated autophagy modulator protein 2
0.954	1.039	1.013	0.999	1.033	0.996	1.094	1.153	1.061	0.966	0.946	0.953	B0QYD3	APOBEC3B	DNA dC->dU-editing enzyme APOBEC-3B
0.970	1.059	0.952	1.021	1.006	0.887	0.992	1.050	0.993	0.895	0.982	0.886	Q9NRW3	APOBEC3C	DNA dC->dU-editing enzyme APOBEC-3C
1.023	1.011	1.032	0.973	0.959	1.033	0.975	1.100	1.004	0.960	0.931	0.965	Q8IUX4	APOBEC3F	DNA dC->dU-editing enzyme APOBEC-3F
1.036	1.029	1.021	0.993	0.971	0.991	0.971	0.953	1.000	0.984	0.937	0.997	Q9HC16	APOBEC3G	DNA dC->dU-editing enzyme APOBEC-3G
1.035	0.870	1.034	0.963	0.929	1.101	0.962	0.947	1.113	0.966	1.023	1.034	P07992	ERCC1	DNA excision repair protein ERCC-1
0.988	0.892	0.990										Q03468	ERCC6	DNA excision repair protein ERCC-6
1.026	0.976	1.020	0.962	0.975	0.929	0.962	1.110	1.187	0.776	0.814	1.114	Q2NKX8	ERCC6L	DNA excision repair protein ERCC-6-like
1.044	1.036	1.039	1.020	1.102	1.030	1.001	0.991	1.037	1.010	1.040	1.027	O00273	DFFA	DNA fragmentation factor subunit alpha
0.972	1.018	1.075	0.923	0.915	1.163	1.006	1.191	1.166	0.951	1.020	0.968	B4DZS0	DFFB	DNA fragmentation factor subunit beta
						0.723	0.798	0.921				Q9NXL9	MCM9	DNA helicase MCM9
0.956	1.000	0.992	1.008	0.975	1.006	0.996	0.997	1.067	0.952	0.921	1.086	P18858	LIG1	DNA ligase 1
1.003	1.000	1.035	0.967	0.949	1.013	0.970	0.988	1.001	1.005	1.016	1.017	P49916	LIG3	DNA ligase 3
1.013	0.999	1.008	0.990	0.946	1.017	0.988	1.011	1.003	1.001	0.995	1.085	P49917	LIG4	DNA ligase 4
1.035	0.973	1.023	0.998	0.983	1.034	0.989	0.983	1.011	1.061	0.950	1.000	Q9NPF5	DMAP1	DNA methyltransferase 1-associated protein 1
1.029	1.017	1.026	1.026	0.975	1.032	1.015	1.053	0.969	0.963	0.994	1.042	P40692	MLH1	DNA mismatch repair protein Mlh1
0.974	0.996	0.989	0.985	0.964	0.992	0.987	1.054	1.006	0.955	0.962	0.981	P43246	MSH2	DNA mismatch repair protein Msh2
1.016	0.986	1.025	0.978	0.973	1.007	0.963	1.030	1.018	0.953	0.959	1.020	P20585	MSH3	DNA mismatch repair protein Msh3
0.966	0.983	0.997	0.988	0.978	1.018	1.035	1.080	0.998	0.957	0.943	1.026	P52701	MSH6	DNA mismatch repair protein Msh6

Abundance Ratio														
1 d M1/C1	1 d M2/C2	1 d M3/C3	4 d M1/C1	4 d M2/C2	4 d M3/C3	7 d M1/C1	7 d M2/C2	7 d M3/C3	10 d M1/C1	10 d M2/C2	10 d M3/C3	Accession ID	Gene name	Protein Description
0.741	0.753	1.155	0.919	0.952	0.913	1.019	0.996	1.062	0.934	1.194	1.285	Q6NS38	ALKBH2	DNA oxidative demethylase ALKBH2
1.004	0.947	1.012	1.010	0.940	1.027	1.284	0.945	1.205	1.128	0.889	1.045	Q14181	POLA2	DNA polymerase alpha subunit B
1.010	0.996	1.029	0.990	0.982	1.027	0.986	0.963	0.960	0.956	1.025	0.990	P06746	POLB	DNA polymerase beta
0.998	1.026	0.980	1.015	0.953	0.982	1.031	1.073	1.037	0.961	0.982	1.050	A0A087WWF6	POLD2	DNA polymerase delta subunit 2
0.997	0.969	1.022	0.977	0.974	0.984	1.036	1.013	1.002	0.998	0.984	1.074	Q15054	POLD3	DNA polymerase delta subunit 3
1.012	0.968	1.058	1.195	0.820	1.002	1.092	1.045	0.944				Q9HCU8	POLD4	DNA polymerase delta subunit 4
0.980	1.009	0.993	0.970	0.939	1.082	0.970	1.107	1.015	0.893	0.896	1.018	Q07864	POLE	DNA polymerase epsilon catalytic subunit A
1.002	0.995	1.026	1.024	1.369	0.939	1.057	1.073	1.200	0.934	0.903	1.273	P56282	POLE2	DNA polymerase epsilon subunit 2
0.984	1.020	1.014	1.003	0.991	1.041	1.048	1.021	1.031	0.989	0.976	1.044	Q9NRF9	POLE3	DNA polymerase epsilon subunit 3
0.992	0.949	1.062	0.911	0.927	1.098	1.053	0.995	1.052	0.800	0.935	0.954	Q9NR33	POLE4	DNA polymerase epsilon subunit 4
1.007	0.794	1.292	0.942	1.006	0.975							Q9UNA4	POLI	DNA polymerase iota
0.894	0.948	1.117							0.966	0.829	1.110	Q9UBT6	POLK	DNA polymerase kappa
0.949	1.073	1.021	0.971	0.910	0.945	0.935	1.329	1.046	0.916	0.990	0.999	A6NMQ1	POLA1	DNA polymerase
0.976	1.024	1.012	0.994	0.958	0.982	1.022	1.104	1.005	0.960	0.941	1.019	M0R2B7	POLD1	DNA polymerase
0.977	0.956	1.080	0.947	0.937	1.010	0.988	0.968	0.988	1.003	1.006	1.074	P54098	POLG	DNA polymerase subunit gamma-1
1.015	1.021	1.015	0.990	0.958	1.034	1.010	1.062	1.085	0.985	0.950	1.029	Q9UHN1	POLG2	DNA polymerase subunit gamma-2, mitochondrial
0.938	0.960	0.965	1.027	1.043	0.953	1.013	1.011	1.021	1.070	0.942	1.138	O60673	REV3L	DNA polymerase zeta catalytic subunit
0.985	0.945	0.988	1.045	1.130	1.157	1.078	1.104	1.122	0.941	0.910	1.115	P49643	PRIM2	DNA primase large subunit
1.029	1.014	0.992	1.087	0.957	0.949	1.071	1.201	0.948	0.906	0.825	1.138	P49642	PRIM1	DNA primase small subunit
1.069	1.004	1.088	1.012	1.035	1.067	1.042	1.083	0.990	0.978	0.947	0.999	Q92889	ERCC4	DNA repair endonuclease XPF
1.246	0.911	1.103	1.365	0.630	1.196	0.974	0.983	0.966	1.025	1.087	1.314	P23025	XPA	DNA repair protein complementing XP-A cells
1.077	0.995	1.069	0.995	0.986	0.994	0.978	0.964	0.997	1.015	0.974	0.961	Q01831	XPC	DNA repair protein complementing XP-C cells
						0.890	0.864	1.080	0.921	1.024	0.956	P28715	ERCC5	DNA repair protein complementing XP-G cells
1.061	0.973	0.975				0.976	1.091	0.991	0.801	0.951	1.099	C9JYJ0	RAD51B	DNA repair protein RAD51 homolog 2
1.074	0.796	0.788										O43502	RAD51C	DNA repair protein RAD51 homolog 3
1.046	1.027	1.013	1.022	1.079	0.995	1.031	0.982	1.000	0.987	1.011	0.989	F5H8D7	XRCC1	DNA repair protein XRCC1
1.023	0.986	1.018	0.984	0.962	0.996	1.006	1.043	0.987	1.046	0.981	1.031	Q13426	XRCC4	DNA repair protein XRCC4
1.030	1.023	0.995	1.037	0.937	1.088	0.966	1.044	1.076	1.116	1.262	1.051	Q14691	GINS1	DNA replication complex GINS protein PSF1
0.987	1.073	1.018	0.922	1.030	1.062	1.134	1.147	0.959	0.873	0.981	1.013	Q9Y248	GINS2	DNA replication complex GINS protein PSF2
1.005	1.021	1.009	1.020	0.939	0.993	1.096	1.021	1.041	0.973	0.959	1.054	Q9BRX5	GINS3	DNA replication complex GINS protein PSF3
1.060	1.006	1.020	0.973	0.949	0.967	0.990	1.189	0.988	0.933	0.924	1.004	Q9BRT9	GINS4	DNA replication complex GINS protein SLD5
1.225	1.050	1.381	0.883	1.128	0.987							Q9H211	CDT1	DNA replication factor Cdt1
0.974	0.999	0.986	0.999	0.993	1.011	1.025	1.070	1.027	0.996	0.989	1.051	P49736	MCM2	DNA replication licensing factor MCM2

Abundance Ratio														
1 d M1/C1	1 d M2/C2	1 d M3/C3	4 d M1/C1	4 d M2/C2	4 d M3/C3	7 d M1/C1	7 d M2/C2	7 d M3/C3	10 d M1/C1	10 d M2/C2	10 d M3/C3	Accession ID	Gene name	Protein Description
0.994	0.994	0.983	1.001	0.971	0.965	1.051	1.083	1.037	0.998	0.977	1.042	P33991	MCM4	DNA replication licensing factor MCM4
0.992	1.001	0.975	1.010	1.061	0.963	1.063	1.048	1.036	1.037	0.974	1.054	P33992	MCM5	DNA replication licensing factor MCM5
1.006	0.985	0.998	1.009	1.072	0.992	1.078	1.000	1.024	1.000	0.975	1.093	Q14566	MCM6	DNA replication licensing factor MCM6
1.027	0.991	1.011	0.988	0.986	0.998	1.025	1.058	1.013	0.997	0.991	1.056	P33993	MCM7	DNA replication licensing factor MCM7
0.980	1.008	0.976	1.022	1.027	1.002	1.010	1.015	0.988	0.982	1.027	0.996	P11387	TOP1	DNA topoisomerase 1
1.063	1.007	1.076				0.773	1.067	1.095	0.967	1.018	0.966	Q92547	TOPBP1	DNA topoisomerase 2-binding protein 1
1.112	0.934	1.083	1.053	0.926	0.954	1.104	0.920	1.024	1.111	0.899	1.066	Q13472	TOP3A	DNA topoisomerase 3-alpha
1.083	1.051	1.020	1.066	0.975	0.985	1.036	1.178	1.003	1.008	1.014	0.973	O95985	TOP3B	DNA topoisomerase 3-beta-1
0.912	1.105	1.102	1.116	1.005	1.017	1.252	0.949	1.142	0.922	1.053	0.962	G3V5Q1	APEX1	DNA-(apurinic or apyrimidinic site) lyase (Fragment)
1.057	1.002	1.028	1.009	1.094	0.969	0.964	0.951	1.009	0.966	1.018	0.911	P27695	APEX1	DNA-(apurinic or apyrimidinic site) lyase
1.056	1.028	1.000	1.039	0.955	1.014	0.970	1.008	1.004	1.003	0.967	1.111	O60870	KIN	DNA/RNA-binding protein KIN17
1.061	0.986	1.021	1.032	1.004	1.001	0.975	1.050	1.029	1.034	1.010	1.026	P29372	MPG	DNA-3-methyladenine glycosylase
0.946	0.948	0.999	0.877	1.103	1.001	0.817	0.815	0.611				P41134	ID1	DNA-binding protein inhibitor ID-1
1.022	1.010	1.046	0.978	0.926	1.043	0.932	0.872	0.968	1.048	1.038	1.102	P48382	RFX5	DNA-binding protein RFX5
0.998	0.926	0.951	0.950	0.899	1.051	0.960	1.029	1.151	0.904	0.934	1.020	O14593	RFXANK	DNA-binding protein RFXANK
0.970	1.047	1.040	0.974	1.067	1.079	1.018	1.065	1.068	0.976	0.992	1.064	Q9UPW6	SATB2	DNA-binding protein SATB2
0.975	1.001	1.145	1.006	1.031	1.081	0.978	1.055	1.068	1.006	0.976	1.064	P38935	IGHMBP2	DNA-binding protein SMUBP-2
0.987	0.995	0.996	0.987	0.992	1.012	0.999	1.027	1.019	0.973	1.001	0.959	P78527	PRKDC	DNA-dependent protein kinase catalytic subunit
1.062	0.972	1.053	1.034	1.010	1.195	1.163	1.052	1.039	1.208	1.060	1.071	Q9NP87	POLM	DNA-directed DNA/RNA polymerase mu
1.053	1.029	1.030	1.079	0.971	1.030	1.043	1.068	1.007	1.066	1.013	1.023	O95602	POLR1A	DNA-directed RNA polymerase I subunit RPA1
									1.230	1.009	1.059	Q9P1U0	ZNRD1	DNA-directed RNA polymerase I subunit RPA12
1.008	0.971	0.989	1.002	0.984	1.017	1.036	1.145	1.041	0.962	1.027	1.010	Q3B726	TWISTNB	DNA-directed RNA polymerase I subunit RPA43
1.082	1.007	0.971	0.890	0.997	0.947	1.154	0.995	1.061	0.993	0.941	1.204	Q6EEV4	POLR2M	DNA-directed RNA polymerase II subunit GRINL1A, isoforms 4/5
1.010	1.004	0.996	1.010	0.973	1.024	1.006	1.039	0.996	0.986	0.992	1.021	P24928	POLR2A	DNA-directed RNA polymerase II subunit RPB1
1.029	1.011	0.967	1.015	1.035	1.008	1.053	1.051	0.991	1.001	0.994	0.992	E2QRJ6	POLR2J3	DNA-directed RNA polymerase II subunit RPB11-b2
1.005	1.019	0.986	1.010	0.961	0.983	1.042	1.065	0.994	1.014	0.991	1.004	P30876	POLR2B	DNA-directed RNA polymerase II subunit RPB2
0.974	0.992	0.992	0.998	0.963	1.022	0.988	1.049	1.075	0.998	1.002	1.085	P19387	POLR2C	DNA-directed RNA polymerase II subunit RPB3
0.976	0.980	1.050	0.957	0.965	1.008	0.952	0.871	0.948	1.049	0.996	1.105	O15514	POLR2D	DNA-directed RNA polymerase II subunit RPB4
1.048	1.033	1.017	0.989	0.982	1.013	0.989	1.121	1.010	0.979	0.973	0.968	P62487	POLR2G	DNA-directed RNA polymerase II subunit RPB7
1.051	0.985	1.095	0.953	0.860	0.980	0.983	1.039	1.201	1.003	1.044	1.090	P36954	POLR2I	DNA-directed RNA polymerase II subunit RPB9
1.005	0.997	1.027	1.035	0.974	0.992	1.033	1.041	1.033	1.033	0.999	1.058	O14802	POLR3A	DNA-directed RNA polymerase III subunit RPC1
			1.256	0.799	1.080	0.969	1.007	1.112	0.957	0.938	1.025	Q9Y2Y1	POLR3K	DNA-directed RNA polymerase III subunit RPC10

Abundance Ratio														
1 d M1/C1	1 d M2/C2	1 d M3/C3	4 d M1/C1	4 d M2/C2	4 d M3/C3	7 d M1/C1	7 d M2/C2	7 d M3/C3	10 d M1/C1	10 d M2/C2	10 d M3/C3	Accession ID	Gene name	Protein Description
1.049	0.938	1.072	0.983	0.969	1.046	0.970	1.078	1.017	0.995	0.997	1.054	Q9NW08	POLR3B	DNA-directed RNA polymerase III subunit RPC2
0.983	0.977	1.019	1.035	0.975	1.080	1.048	1.051	1.020	0.976	0.987	1.042	Q9BUI4	POLR3C	DNA-directed RNA polymerase III subunit RPC3
1.024	0.984	1.076	1.003	0.993	1.017	1.027	1.106	0.996	0.975	0.920	1.117	P05423	POLR3D	DNA-directed RNA polymerase III subunit RPC4
0.970	1.018	1.003	1.030	1.003	1.043	1.056	1.087	1.009	1.046	1.026	1.075	Q9NVU0	POLR3E	DNA-directed RNA polymerase III subunit RPC5
0.987	0.960	1.064	1.014	1.014	0.996	1.020	1.131	1.051	0.962	0.913	1.045	Q9H1D9	POLR3F	DNA-directed RNA polymerase III subunit RPC6
1.027	1.004	0.970	0.956	0.947	1.015	1.021	1.110	1.022	0.918	0.997	1.030	O15318	POLR3G	DNA-directed RNA polymerase III subunit RPC7
1.176	1.078	1.034	1.077	0.993	0.931	0.983	1.243	0.976				Q9BT43	POLR3GL	DNA-directed RNA polymerase III subunit RPC7-like
			0.967	1.063	1.068	0.959	1.017	1.047	1.008	1.053	1.065	Q9Y535	POLR3H	DNA-directed RNA polymerase III subunit RPC8
1.004	1.019	1.047	1.017	0.974	1.049	0.985	1.046	0.999	0.934	0.956	0.980	O00411	POLRMT	DNA-directed RNA polymerase, mitochondrial
1.035	0.996	0.995	1.005	0.973	0.996	1.030	1.068	1.019	0.976	1.020	1.040	O15160	POLR1C	DNA-directed RNA polymerases I and III subunit RPAC1
1.008	0.993	0.989	0.998	1.016	1.122	0.984	0.959	0.969	1.005	1.048	1.088	Q9Y2S0	POLR1D	DNA-directed RNA polymerases I and III subunit RPAC2
0.977	0.995	0.996	0.982	0.993	0.995	1.013	0.976	1.021	1.018	1.020	1.036	P19388	POLR2E	DNA-directed RNA polymerases I, II, and III subunit RPABC1
0.927	0.989	0.948	1.054	1.034	0.988	1.077	1.065	0.863				B0QYL9	POLR2F	DNA-directed RNA polymerases I, II, and III subunit RPABC2
1.011	1.001	1.039	1.040	1.003	1.019	1.064	0.971	0.995	1.074	1.031	1.077	P52434	POLR2H	DNA-directed RNA polymerases I, II, and III subunit RPABC3
1.114	1.016	1.060	0.996	1.010	0.970	1.009	1.057	1.031	0.948	1.025	0.970	P53803	POLR2K	DNA-directed RNA polymerases I, II, and III subunit RPABC4
1.038	0.923	0.932	0.945	1.176	0.981	1.001	0.901	0.962	1.018	0.984	1.001	P62875	POLR2L	DNA-directed RNA polymerases I, II, and III subunit RPABC5
0.982	0.979	1.037	1.043	0.969	0.990	1.118	0.969	0.993	1.050	1.010	1.127	J3KPS0	DNAJB12	DnaJ (Hsp40) homolog, subfamily B, member 12, isoform CRA_c
1.012	1.027	0.989	0.953	1.023	0.923	0.977	0.962	0.973	0.938	0.974	0.971	P31689	DNAJA1	DnaJ homolog subfamily A member 1
1.004	0.978	0.995	0.989	1.085	1.010	1.016	0.935	1.069	0.972	0.983	1.005	O60884	DNAJA2	DnaJ homolog subfamily A member 2
1.028	0.967	0.988	0.994	0.993	0.980	1.014	0.984	1.002	0.970	0.939	0.982	Q96EY1	DNAJA3	DnaJ homolog subfamily A member 3, mitochondrial
1.005	0.992	1.016	1.000	0.957	1.008	0.968	0.971	1.002	0.989	0.961	1.043	P25685	DNAJB1	DnaJ homolog subfamily B member 1
0.976	1.015	0.978	0.986	0.927	0.986	0.987	1.028	0.968	1.038	1.033	1.009	Q9UBS4	DNAJB11	DnaJ homolog subfamily B member 11
0.926	0.934	0.965	1.038	0.810	1.105	1.064	1.190	0.932	1.207	1.060	1.121	Q8TBM8	DNAJB14	DnaJ homolog subfamily B member 14
1.053	0.987	0.992	0.987	1.002	1.042	1.051	0.949	0.933	1.005	1.016	1.070	P25686	DNAJB2	DnaJ homolog subfamily B member 2
1.018	1.005	0.984	1.001	0.987	1.010	0.988	1.003	1.006	0.973	1.006	1.020	Q9UDY4	DNAJB4	DnaJ homolog subfamily B member 4
1.018	1.007	1.031	0.978	0.974	0.987	1.012	0.936	0.984	1.009	0.994	1.054	O75190	DNAJB6	DnaJ homolog subfamily B member 6
			0.991	0.880	0.911	1.293	1.048	0.868	1.092	0.986	1.113	Q9UBS3	DNAJB9	DnaJ homolog subfamily B member 9

Abundance Ratio														
1 d M1/C1	1 d M2/C2	1 d M3/C3	4 d M1/C1	4 d M2/C2	4 d M3/C3	7 d M1/C1	7 d M2/C2	7 d M3/C3	10 d M1/C1	10 d M2/C2	10 d M3/C3	Accession ID	Gene name	Protein Description
0.918	1.018	1.084	1.080	1.039	1.002	1.062	0.920	0.897	1.105	1.099	1.047	Q96KC8	DNAJC1	DnaJ homolog subfamily C member 1
1.016	1.018	1.015	1.014	0.981	0.989	1.019	0.967	1.007	1.045	0.984	1.039	Q8IXB1	DNAJC10	DnaJ homolog subfamily C member 10
0.986	1.002	1.028	1.023	0.942	0.982	1.020	1.010	1.009	1.052	1.025	1.045	Q9NVH1	DNAJC11	DnaJ homolog subfamily C member 11
1.007	1.008	1.014	1.003	1.011	1.025	0.994	1.068	1.027	1.011	0.976	1.039	O75165	DNAJC13	DnaJ homolog subfamily C member 13
0.752	0.696	1.200	1.003	0.895	1.170							Q6Y2X3	DNAJC14	DnaJ homolog subfamily C member 14
1.041	0.987	1.048	1.133	0.983	0.991	1.068	1.059	1.102	1.096	0.992	1.060	Q9Y2G8	DNAJC16	DnaJ homolog subfamily C member 16
0.961	0.977	1.018	1.029	0.993	1.010	0.972	1.043	1.039	1.006	1.062	1.091	Q9NVM6	DNAJC17	DnaJ homolog subfamily C member 17
1.004	0.967	0.992	0.976	0.978	1.037	0.998	0.993	1.024	0.964	0.903	1.060	Q99543	DNAJC2	DnaJ homolog subfamily C member 2
			0.905	0.709	1.168	0.929	1.205	1.033				A0A0A0MT77	DNAJC24	DnaJ homolog subfamily C member 24
1.002	1.347	1.022	0.994	0.919	1.057	1.070	1.095	0.973	1.107	0.988	1.105	Q9H1X3	DNAJC25	DnaJ homolog subfamily C member 25
0.987	0.985	1.021	0.997	0.975	1.033	0.981	0.976	1.018	1.017	1.020	1.051	Q13217	DNAJC3	DnaJ homolog subfamily C member 3
1.002	0.967	1.070	0.934	0.917	0.968	1.030	1.204	1.118	0.986	0.973	1.040	Q96LL9	DNAJC30	DnaJ homolog subfamily C member 30
0.915	0.995	0.995	0.949	0.862	0.950	0.979	0.893	0.956	1.047	1.042	1.105	Q9H3Z4	DNAJC5	DnaJ homolog subfamily C member 5
0.994	1.010	0.989	1.032	0.974	0.995	1.066	1.021	1.008	1.065	1.000	1.071	Q99615	DNAJC7	DnaJ homolog subfamily C member 7
1.013	0.979	0.986	0.970	0.949	0.978	0.966	0.954	0.975	0.982	1.023	0.991	O75937	DNAJC8	DnaJ homolog subfamily C member 8
1.017	0.980	1.023	1.018	0.938	0.998	0.992	1.027	1.007	0.950	1.018	1.024	Q8WXX5	DNAJC9	DnaJ homolog subfamily C member 9
0.924	0.990	1.029	0.850	0.887	1.017	0.941	0.853	0.960	0.959	1.072	1.030	Q5SXM8	DNLZ	DNL-type zinc finger protein
0.881	0.982	1.006	1.124	0.937	1.126	1.035	1.088	1.008				Q99704	DOK1	Docking protein 1
			1.048	1.131	1.057	1.028	0.989	0.899				Q9UPQ8	DOLK	Dolichol kinase
1.001	0.975	0.971	1.071	0.960	1.078	1.086	1.065	1.062	1.175	0.993	1.048	H0Y368	DPM1	Dolichol-phosphate mannosyltransferase subunit 1 (Fragment)
0.968	1.017	1.038	0.998	0.772	1.165	1.045	0.999	0.958	1.211	1.040	1.108	Q86YN1	DOLPP1	Dolichyldiphosphatase 1
0.989	0.982	0.994	0.978	0.974	0.998	0.983	1.061	1.014	0.997	0.987	0.983	P39656	DDOST	Dolichyl-diphosphooligosaccharide--protein glycosyltransferase 48 kDa subunit
0.972	0.996	1.013	0.984	0.978	1.008	0.979	1.024	0.990	1.041	1.012	1.000	P04843	RPN1	Dolichyl-diphosphooligosaccharide--protein glycosyltransferase subunit 1
1.249	1.385	1.305				0.555	1.027	0.975	0.878	1.085	0.860	P04844	RPN2	Dolichyl-diphosphooligosaccharide--protein glycosyltransferase subunit 2
0.960	1.052	0.956	1.009	0.920	1.067	1.120	1.080	1.114	1.273	1.143	1.158	P61803	DAD1	Dolichyl-diphosphooligosaccharide--protein glycosyltransferase subunit DAD1
0.939	0.973	0.962	0.952	0.878	1.011	0.982	0.908	0.979	1.098	1.008	1.007	P46977	STT3A	Dolichyl-diphosphooligosaccharide--protein glycosyltransferase subunit STT3A
0.962	0.968	0.985	0.996	0.919	1.020	1.039	1.031	1.018	1.111	1.059	1.047	Q8TCJ2	STT3B	Dolichyl-diphosphooligosaccharide--protein glycosyltransferase subunit STT3B
0.979	0.970	1.000	0.982	0.898	1.030	1.032	0.982	0.993	1.012	1.001	1.015	Q9Y673	ALG5	Dolichyl-phosphate beta-glucosyltransferase
0.827	0.872	1.098	0.700	0.901	1.071	0.744	0.547	0.946	0.991	1.356	0.893	Q5BKT4	ALG10	Dol-P-Glc:Glc(2)Man(9)GlcNAc(2)-PP-Dol alpha-1,2-glucosyltransferase

Abundance Ratio														
1 d M1/C1	1 d M2/C2	1 d M3/C3	4 d M1/C1	4 d M2/C2	4 d M3/C3	7 d M1/C1	7 d M2/C2	7 d M3/C3	10 d M1/C1	10 d M2/C2	10 d M3/C3	Accession ID	Gene name	Protein Description
1.047	0.984	0.980	0.960	0.925	0.986	1.012	1.162	1.056	1.016	1.032	0.947	Q9BV10	ALG12	Dol-P-Man:Man(7)GlcNAc(2)-PP-Dol alpha-1,6-mannosyltransferase
0.805	0.968	0.934	0.981	0.987	0.889	1.058	0.937	0.985	1.054	0.995	1.217	M0R2J8	DCDC1	Doublecortin domain-containing protein 1
1.039	1.021	1.014	1.010	1.022	0.991	0.989	1.026	1.038	0.949	0.971	0.998	O60216	RAD21	Double-strand-break repair protein rad21 homolog
1.022	0.990	1.020	0.981	0.976	1.013	0.971	0.950	0.998	0.973	0.992	1.038	O95793	STAU1	Double-stranded RNA-binding protein Staufen homolog 1
1.005	1.020	1.029	1.007	0.975	1.009	1.019	1.068	1.005	1.031	1.052	1.004	A0A0A0MTC5	STAU2	Double-stranded RNA-binding protein Staufen homolog 2
1.057	1.119	0.936	0.931	1.037	0.960	0.973	1.097	0.981	0.978	0.920	0.951	Q9NS39	ADARB2	Double-stranded RNA-specific editase B2
1.024	0.975	1.044	0.988	0.937	1.001	0.998	1.120	1.045	0.976	1.009	0.985	O14972	DSCR3	Down syndrome critical region protein 3
0.967	1.013	0.998	1.000	0.946	1.026	1.024	0.989	0.963	0.993	1.037	1.005	C9JCC6	DRAP1	Dr1-associated corepressor
0.993	0.983	1.010	0.990	0.989	0.992	1.007	0.957	0.998	1.034	1.012	1.035	Q9UJU6	DBNL	Drebrin-like protein
0.933	1.307	1.041										Q8NBA8	DTWD2	DTW domain-containing protein 2
0.975	0.956	0.990	1.000	0.962	0.995	1.009	1.056	0.973	1.069	1.053	0.990	Q8TEA8	DTD1	D-tyrosyl-tRNA(Tyr) deacylase 1
1.166	1.180	1.198	0.928	1.305	1.497	0.956	0.919	0.903	0.925	0.814	0.778	Q6XUX3	DSTYK	Dual serine/threonine and tyrosine protein kinase
1.011	1.027	0.990	0.983	0.955	1.016	1.000	1.076	0.993	0.992	0.975	1.011	Q02750	MAP2K1	Dual specificity mitogen-activated protein kinase kinase 1
0.959	0.996	0.977	0.996	0.959	0.990	0.991	1.033	0.992	0.995	1.004	1.004	P36507	MAP2K2	Dual specificity mitogen-activated protein kinase kinase 2
0.946	1.077	1.172	0.977	0.893	1.012							Q13163	MAP2K5	Dual specificity mitogen-activated protein kinase kinase 5
1.118	1.143	0.980	0.941	0.925	1.178	0.844	1.009	1.124				P49760	CLK2	Dual specificity protein kinase CLK2
0.959	1.165	1.063	0.974	0.937	0.995	0.994	1.017	1.136	0.981	0.966	1.151	P49761	CLK3	Dual specificity protein kinase CLK3
0.868	0.578	0.916	0.992	0.928	1.052	0.923	1.089	1.020	0.984	1.000	1.038	Q9HAZ1	CLK4	Dual specificity protein kinase CLK4
1.025	1.021	0.980	0.931	0.910	0.972	1.005	1.232	1.002	0.950	0.925	1.034	P33981	TTK	Dual specificity protein kinase TTK
0.999	0.998	0.983	0.979	0.942	1.026	0.986	1.047	1.017	0.982	0.982	0.987	Q9UNI6	DUSP12	Dual specificity protein phosphatase 12
1.035	1.006	1.010	0.996	0.936	1.018	1.041	1.106	0.987	1.025	1.016	1.024	Q9BVJ7	DUSP23	Dual specificity protein phosphatase 23
1.032	1.007	0.969	1.033	0.964	0.976	1.013	1.022	1.017	1.042	0.990	1.013	P51452	DUSP3	Dual specificity protein phosphatase 3
									1.112	0.927	1.173	Q16690	DUSP5	Dual specificity protein phosphatase 5
			1.269	0.924	1.052							Q16828	DUSP6	Dual specificity protein phosphatase 6
									0.972	0.939	1.128	Q16829	DUSP7	Dual specificity protein phosphatase 7
1.089	1.031	1.011	1.076	1.010	0.963	0.953	1.151	0.957	1.056	1.031	0.998	Q13627	DYRK1A	Dual specificity tyrosine-phosphorylation-regulated kinase 1A
0.886	0.913	0.886				1.037	1.292	0.909	0.806	0.761	0.980	V9GY92	DUSP12	Dual-specificity protein phosphatase 12

Abundance Ratio														
1 d M1/C1	1 d M2/C2	1 d M3/C3	4 d M1/C1	4 d M2/C2	4 d M3/C3	7 d M1/C1	7 d M2/C2	7 d M3/C3	10 d M1/C1	10 d M2/C2	10 d M3/C3	Accession ID	Gene name	Protein Description
0.989	1.040	0.928	0.869	1.044	1.107	0.935	0.975	1.094	0.984	1.085	1.060	Q5JU07	CDC14B	Dual-specificity protein phosphatase CDC14B (Fragment)
0.923	0.992	1.103	0.981	0.966	1.050	0.971	0.919	1.068	0.911	1.005	0.905	Q7RTS9	DYM	Dymeclin
0.979	0.968	0.995	0.988	1.017	1.007	0.995	0.980	1.020	1.018	0.989	1.022	E7EX90	DCTN1	Dynactin subunit 1
1.002	0.977	1.006	0.993	0.939	1.007	1.005	1.010	0.979	1.003	0.970	0.995	O75935	DCTN3	Dynactin subunit 3
0.996	1.013	1.016	1.017	0.928	1.006	1.006	1.049	1.036	0.988	0.994	1.010	Q9UJW0	DCTN4	Dynactin subunit 4
0.998	1.013	0.993	0.961	0.967	0.999	0.983	0.939	1.023	1.042	1.016	1.037	Q9BTE1	DCTN5	Dynactin subunit 5
1.036	1.039	0.992	1.043	1.010	0.981	1.015	1.020	0.984	1.003	1.037	1.000	O00399	DCTN6	Dynactin subunit 6
0.929	1.002	1.129	0.986	1.020	1.044	1.052	0.972	0.958	1.024	0.946	1.082	A0A0D9SFE4	DNM1	Dynamin-1
1.017	1.000	1.011	1.017	1.002	1.019	1.020	1.028	1.019	1.026	0.981	1.009	O00429	DNM1L	Dynamin-1-like protein
0.974	1.000	0.976	0.954	0.976	0.991	0.961	1.026	0.991	0.945	0.955	1.000	P50570	DNM2	Dynamin-2
1.002	0.991	1.036	0.949	0.973	0.993	0.935	0.981	0.997	0.956	0.941	1.097	Q6XZF7	DNMBP	Dynamin-binding protein
1.009	1.002	1.046	0.995	0.998	1.041	1.031	1.075	1.050	1.032	1.002	1.093	Q86Y56	DNAAF5	Dynein assembly factor 5, axonemal
0.914	1.149	0.903	0.984	0.929	0.978	0.875	0.720	0.986	1.086	1.023	1.178	Q4LDG9	DNAL1	Dynein light chain 1, axonemal
1.092	0.961	0.974	1.014	1.308	0.930	1.010	0.922	0.985	0.986	1.007	0.968	P63167	DYNLL1	Dynein light chain 1, cytoplasmic
1.066	0.995	1.010	0.982	0.969	0.945	1.033	1.059	0.951	1.070	1.009	1.003	Q96FJ2	DYNLL2	Dynein light chain 2, cytoplasmic
1.197	0.949	1.136	0.874	1.031	0.966	1.030	1.082	1.108				O96015	DNAL4	Dynein light chain 4, axonemal
0.959	0.975	1.019	0.939	0.996	0.993	0.945	0.859	0.970	0.949	1.051	0.969	B1AKR6	DYNLRB1	Dynein light chain roadblock-type 1
0.973	0.983	1.024	1.011	1.215	0.991	0.973	1.043	0.988	0.922	1.020	0.995	P63172	DYNLT1	Dynein light chain Tctex-type 1
1.379	1.048	1.153	1.113	1.694	0.912	1.107	0.936	1.068	0.919	0.969	0.871	A6NGJ0	DYNLT3	Dynein light chain Tctex-type 3
0.979	0.980	1.002	1.017	0.974	1.008	1.059	1.045	1.096	1.040	1.028	1.105	Q96EV8	DTNBP1	Dysbindin
1.012	1.003	0.989	1.003	0.876	0.973	0.905	1.151	1.025	0.971	0.963	1.173	Q8IZA0	KIAA0319L	Dyslexia-associated protein KIAA0319-like protein
0.996	1.038	1.053	0.999	0.921	1.036	1.023	1.097	1.039	0.984	0.988	1.047	Q03001	DST	Dystonin
			0.887	0.861	1.073	0.833	1.129	1.131	0.822	0.836	1.050	O60941	DTNB	Dystrobrevin beta
0.932	0.957	0.962	0.938	0.925	0.946	1.031	0.942	1.028	1.138	1.001	1.115	Q14118	DAG1	Dystroglycan
0.927	0.959	0.986	0.982	0.931	0.990	1.023	1.023	1.016	0.981	0.861	1.107	A0A075B6G3	DMD	Dystrophin
1.046	1.014	1.015	0.994	0.986	0.981	0.998	1.094	0.973	1.050	1.018	1.066	Q96L91	EP400	E1A-binding protein p400
0.993	0.985	1.014	0.917	1.078	1.043	0.976	0.906	0.949	0.973	1.018	1.180	Q56P03	EAPP	E2F-associated phosphoprotein
1.022	1.001	1.031	1.000	0.924	1.060	0.982	1.015	1.003	0.973	0.964	0.958	Q9UII4	HERC5	E3 ISG15--protein ligase HERC5
1.066	1.022	1.065	0.988	1.019	1.057	1.042	0.927	1.011	1.009	0.941	1.068	O00257	CBX4	E3 SUMO-protein ligase CBX4
1.040	0.981	0.998	0.992	0.943	0.965	1.046	1.039	1.001	0.978	0.996	1.136	Q96MF7	NSMCE2	E3 SUMO-protein ligase NSE2
1.136	0.934	1.046	1.097	0.995	1.054	1.192	1.067	1.094	0.954	0.979	1.057	O75928	PIAS2	E3 SUMO-protein ligase PIAS2
						0.785	0.900	1.057				Q9Y6X2	PIAS3	E3 SUMO-protein ligase PIAS3
1.073	1.001	1.010	0.997	1.030	0.910	0.969	1.162	1.012	0.668	1.056	1.011	Q8N2W9	PIAS4	E3 SUMO-protein ligase PIAS4

Abundance Ratio														
1 d M1/C1	1 d M2/C2	1 d M3/C3	4 d M1/C1	4 d M2/C2	4 d M3/C3	7 d M1/C1	7 d M2/C2	7 d M3/C3	10 d M1/C1	10 d M2/C2	10 d M3/C3	Accession ID	Gene name	Protein Description
0.981	0.989	1.004	0.987	1.000	1.001	1.014	0.959	1.014	0.999	0.992	1.035	P49792	RANBP2	E3 SUMO-protein ligase RanBP2
1.089	1.034	0.979	1.045	0.978	1.030	1.081	1.009	0.976	1.137	1.033	1.096	Q9Y4E5	ZNF451	E3 SUMO-protein ligase ZNF451
1.041	1.029	1.011	1.010	0.999	0.976	1.012	1.031	0.995	1.006	0.987	0.992	Q14258	TRIM25	E3 ubiquitin/ISG15 ligase TRIM25
1.091	1.058	1.023	0.963	0.982	1.025	1.062	1.113	0.981	1.133	1.035	1.115	Q9UKV5	AMFR	E3 ubiquitin-protein ligase AMFR
1.010	0.978	1.010	0.991	1.024	0.977	1.043	1.022	1.017	1.051	1.021	1.074	Q9Y4X5	ARIH1	E3 ubiquitin-protein ligase ARIH1
1.003	0.998	1.093	0.949	1.139	1.009	1.030	0.916	1.005	1.013	1.013	1.075	O95376	ARIH2	E3 ubiquitin-protein ligase ARIH2
0.959	0.970	0.994	0.992	0.946	1.033	1.001	0.952	1.011	1.051	0.991	1.083	Q5VTR2	RNF20	E3 ubiquitin-protein ligase BRE1A
0.993	0.997	1.031	1.030	0.960	1.035	0.996	0.981	0.980	1.033	1.027	1.077	O75150	RNF40	E3 ubiquitin-protein ligase BRE1B
0.975	1.003	1.000	0.998	1.016	0.983	1.036	1.088	1.026	1.004	1.016	1.073	P22681	CBL	E3 ubiquitin-protein ligase CBL
1.516	0.780	0.701										Q13191	CBLB	E3 ubiquitin-protein ligase CBL-B
0.965	0.985	0.975	1.000	0.971	1.028	1.015	0.966	1.015	1.038	1.015	1.056	Q9UNE7	STUB1	E3 ubiquitin-protein ligase CHIP
0.992	0.991	0.997	1.006	1.000	1.058	1.125	0.962	0.982	1.053	0.929	0.945	Q8TDB6	DTX3L	E3 ubiquitin-protein ligase DTX3L
1.059	1.105	1.072	0.991	0.858	1.032	0.888	1.284	1.005	1.144	0.965	1.025	Q8IYU2	HACE1	E3 ubiquitin-protein ligase HACE1
0.987	0.974	1.129	1.016	1.116	1.030	1.021	0.773	0.806	0.895	1.004	1.192	Q75N03	CBLL1	E3 ubiquitin-protein ligase Hakai
0.982	1.014	1.030	0.995	1.032	1.015	1.016	0.992	1.051	0.990	0.950	1.055	A0A087X2H1	HECTD1	E3 ubiquitin-protein ligase HECTD1
1.044	1.023	1.023	1.018	1.017	1.027	1.033	1.094	1.036	1.049	0.981	1.044	Q5T447	HECTD3	E3 ubiquitin-protein ligase HECTD3
1.073	0.992	1.012	0.991	0.939	1.007	1.048	1.079	1.011	0.987	0.997	1.017	O95714	HERC2	E3 ubiquitin-protein ligase HERC2
0.978	0.995	1.007	0.984	0.973	1.009	0.976	0.984	1.002	0.972	0.985	0.992	Q7Z6Z7	HUWE1	E3 ubiquitin-protein ligase HUWE1
1.051	1.011	1.001	1.009	1.008	1.001	0.984	1.111	1.027	0.953	0.970	1.008	Q96J02	ITCH	E3 ubiquitin-protein ligase Itchy homolog
0.956	0.992	1.047	0.996	0.968	1.017	0.947	1.019	1.031	0.977	0.983	1.042	Q9P0J7	KCMF1	E3 ubiquitin-protein ligase KCMF1
0.981	0.997	1.026	0.983	0.980	1.006	1.047	1.036	1.045	1.054	0.995	1.092	Q6UWE0	LRSAM1	E3 ubiquitin-protein ligase LRSAM1
0.948	0.863	1.016	0.839	0.851	1.019	1.187	0.737	0.922	1.186	1.037	0.856	Q9UHC7	MKRN1	E3 ubiquitin-protein ligase makorin-1
0.936	1.010	0.993	1.150	0.971	0.886	0.993	1.061	0.921	1.040	0.982	1.046	Q9NX47	5-Mar	E3 ubiquitin-protein ligase MARCH5
1.012	0.974	0.981				1.139	0.943	1.076	1.077	1.030	0.986	Q9H992	7-Mar	E3 ubiquitin-protein ligase MARCH7
1.045	1.026	0.994	1.011	1.223	1.053	1.033	1.025	1.019	1.141	1.130	1.141	K7EPJ5	MGRN1	E3 ubiquitin-protein ligase MGRN1 (Fragment)
1.007	0.979	1.025	0.909	0.932	1.035	0.991	1.077	0.961	1.012	0.831	0.973	Q86YT6	MIB1	E3 ubiquitin-protein ligase MIB1
0.992	1.004	1.017	1.006	0.922	1.028	1.038	1.186	1.119	0.985	0.970	1.007	O75592	MYCBP2	E3 ubiquitin-protein ligase MYCBP2
1.012	1.001	1.009	0.999	0.966	1.013	1.028	1.107	0.984	0.881	0.896	1.000	Q96PU5	NEDD4L	E3 ubiquitin-protein ligase NEDD4-like
0.990	0.979	0.920	0.978	0.939	1.115	1.023	0.946	1.050	1.148	1.079	1.142	Q9H4P4	RNF41	E3 ubiquitin-protein ligase NRDP1
			1.067	1.156	1.085	1.112	0.845	1.106	1.518	0.822	1.077	O43164	PJA2	E3 ubiquitin-protein ligase Praja-2
									0.980	0.851	1.038	Q9NS91	RAD18	E3 ubiquitin-protein ligase RAD18
1.022	1.014	0.976	1.004	0.982	1.026	0.997	0.998	1.005	0.999	1.005	1.022	Q7Z6E9	RBBP6	E3 ubiquitin-protein ligase RBBP6
1.690	1.189	1.336	1.150	1.996	0.848	1.010	0.734	1.104	0.932	0.991	0.921	P62877	RBX1	E3 ubiquitin-protein ligase RBX1
			0.954	0.860	0.920	0.947	0.950	1.059				Q8NHY2	RFWD2	E3 ubiquitin-protein ligase RFWD2

Abundance Ratio														
1 d M1/C1	1 d M2/C2	1 d M3/C3	4 d M1/C1	4 d M2/C2	4 d M3/C3	7 d M1/C1	7 d M2/C2	7 d M3/C3	10 d M1/C1	10 d M2/C2	10 d M3/C3	Accession ID	Gene name	Protein Description
1.076	0.998	1.027	0.976	0.874	1.034	1.015	1.006	0.945	1.084	1.019	0.976	Q06587	RING1	E3 ubiquitin-protein ligase RING1
1.018	0.997	1.023	1.004	0.960	0.983	1.012	1.039	1.057	0.996	0.945	1.042	Q99496	RNF2	E3 ubiquitin-protein ligase RING2
1.009	0.969	1.194	1.042	0.928	1.304	1.088	1.006	0.995	1.132	0.880	0.902	Q9NVW2	RLIM	E3 ubiquitin-protein ligase RLIM
1.010	0.992	0.973	1.018	1.567	0.983	1.054	0.764	0.986	1.008	1.016	1.000	Q9Y508	RNF114	E3 ubiquitin-protein ligase RNF114
0.886	0.924	1.403	1.011	1.088	0.987	1.038	0.797	1.079	1.076	0.977	1.424	Q9Y4L5	RNF115	E3 ubiquitin-protein ligase RNF115
0.981	1.023	0.988	0.995	0.888	0.971	0.988	1.107	0.941	1.029	0.945	0.953	Q5XPI4	RNF123	E3 ubiquitin-protein ligase RNF123
1.302	0.921	1.111				1.099	0.918	1.064	1.001	1.009	1.120	Q9BV68	RNF126	E3 ubiquitin-protein ligase RNF126
0.952	0.963	0.995	1.105	0.969	1.221	1.005	1.180	1.145	1.326	1.171	1.226	O43567	RNF13	E3 ubiquitin-protein ligase RNF13
						0.987	1.161	1.097	1.005	1.009	1.174	E5RI87	RNF130	E3 ubiquitin-protein ligase RNF130
0.888	0.976	0.937										Q8WVD3	RNF138	E3 ubiquitin-protein ligase RNF138
0.960	1.028	0.954	0.994	0.940	1.030	1.054	1.082	0.979	1.123	1.068	1.116	Q8WU17	RNF139	E3 ubiquitin-protein ligase RNF139
0.969	0.990	1.015	0.969	0.918	0.976	0.988	0.965	1.053	1.038	0.921	1.103	Q9UBS8	RNF14	E3 ubiquitin-protein ligase RNF14
0.926	1.088	1.050	1.116	1.091	1.129	1.146	1.049	1.089	0.929	0.879	1.081	Q8NC42	RNF149	E3 ubiquitin-protein ligase RNF149
			0.999	0.997	1.160				1.105	0.867	1.250	Q9H6Y7	RNF167	E3 ubiquitin-protein ligase RNF167
1.010	1.061	0.985	1.047	0.921	1.039	0.987	1.020	1.091	1.131	0.998	1.055	Q8NCN4	RNF169	E3 ubiquitin-protein ligase RNF169
0.922	0.887	0.926	0.944	0.988	1.102	1.105	1.038	0.957	1.192	0.951	1.267	Q96K19	RNF170	E3 ubiquitin-protein ligase RNF170
1.450	0.766	1.080	0.976	1.103	1.105	0.952	0.834	1.175	0.979	0.987	1.192	Q9P0P0	RNF181	E3 ubiquitin-protein ligase RNF181
0.955	1.016	1.037	0.957	1.135	0.969	1.056	0.852	1.021	1.149	1.056	1.111	Q96GF1	RNF185	E3 ubiquitin-protein ligase RNF185
0.869	0.888	0.817	1.088	0.961	0.924	1.248	1.240	1.104	1.178	0.977	1.136	Q5TA31	RNF187	E3 ubiquitin-protein ligase RNF187
0.991	1.004	1.032	0.986	0.951	1.015	0.999	1.019	1.021	1.007	0.981	1.034	A0A0A0MTR7	RNF213	E3 ubiquitin-protein ligase RNF213
1.049	1.063	0.962	0.950	0.880	1.062	1.052	1.157	0.974	1.043	1.044	1.056	Q5VTB9	RNF220	E3 ubiquitin-protein ligase RNF220
1.068	0.917	1.143	1.025	0.926	1.053	1.039	0.834	1.027	1.108	1.020	1.159	Q96BH1	RNF25	E3 ubiquitin-protein ligase RNF25
1.103	1.062	1.059	1.002	0.922	0.979	1.009	1.016	1.014	1.073	0.911	1.041	Q96EP0	RNF31	E3 ubiquitin-protein ligase RNF31
0.937	0.905	0.921	0.859	0.829	0.846	0.837	0.995	0.921	1.140	1.133	1.263	H7BYJ1	RNF34	E3 ubiquitin-protein ligase RNF34
									1.248	0.886	1.155	P78317	RNF4	E3 ubiquitin-protein ligase RNF4
1.055	1.059	1.034	0.952	0.925	0.845	0.993	0.883	0.929	0.942	1.233	1.209	Q99942	RNF5	E3 ubiquitin-protein ligase RNF5
			1.010	0.871	0.973	1.260	1.210	0.952	1.081	1.130	1.316	Q7Z6J0	SH3RF1	E3 ubiquitin-protein ligase SH3RF1
1.052	0.950	1.161	1.012	1.085	1.010	1.004	1.168	1.026	1.009	0.972	0.918	A0A0D9SFM0	SHPRH	E3 ubiquitin-protein ligase SHPRH
1.027	1.028	1.065	1.006	0.928	1.004	0.966	1.097	1.035	0.882	0.920	1.097	Q9HAU4	SMURF2	E3 ubiquitin-protein ligase SMURF2
1.012	0.962	0.950	0.993	1.072	1.053	1.007	0.879	1.017	1.121	1.006	1.105	Q86TM6	SYVN1	E3 ubiquitin-protein ligase synoviolin
						0.940	1.085	0.905	1.142	1.032	1.020	Q6Q0C0	TRAF7	E3 ubiquitin-protein ligase TRAF7
						1.282	0.889	1.150	0.966	0.950	1.130	Q96F44	TRIM11	E3 ubiquitin-protein ligase TRIM11
0.963	1.014	1.047	0.984	1.003	1.022	1.041	0.967	1.087	1.028	0.964	1.047	P19474	TRIM21	E3 ubiquitin-protein ligase TRIM21
0.846	1.083	1.370										Q8IYM9	TRIM22	E3 ubiquitin-protein ligase TRIM22

Abundance Ratio														
1 d M1/C1	1 d M2/C2	1 d M3/C3	4 d M1/C1	4 d M2/C2	4 d M3/C3	7 d M1/C1	7 d M2/C2	7 d M3/C3	10 d M1/C1	10 d M2/C2	10 d M3/C3	Accession ID	Gene name	Protein Description
1.015	1.019	1.032	1.030	1.008	1.079	1.019	1.027	1.037	1.045	1.087	1.003	Q13049	TRIM32	E3 ubiquitin-protein ligase TRIM32
0.983	1.009	1.003	0.990	0.977	0.991	1.047	1.061	1.020	0.996	1.021	1.048	Q9UPN9	TRIM33	E3 ubiquitin-protein ligase TRIM33
1.115	0.981	0.906	1.203	0.938	0.918	0.827	0.914	0.902	1.403	1.077	1.396	Q9NQ86	TRIM36	E3 ubiquitin-protein ligase TRIM36
1.118	0.941	0.904							1.092	1.054	1.276	O94972	TRIM37	E3 ubiquitin-protein ligase TRIM37
1.008	1.001	1.056	0.973	0.945	0.952	0.988	1.041	1.002	1.016	0.984	1.037	O00635	TRIM38	E3 ubiquitin-protein ligase TRIM38
1.037	1.056	1.051	1.001	0.982	1.032	0.997	1.054	1.008	1.054	1.037	1.015	Q9C037	TRIM4	E3 ubiquitin-protein ligase TRIM4
1.037	1.037	1.074	1.001	0.957	1.072	1.005	0.954	1.044	1.008	0.987	1.072	Q9BRZ2	TRIM56	E3 ubiquitin-protein ligase TRIM56
1.219	0.971	1.080										Q6AZZ1	TRIM68	E3 ubiquitin-protein ligase TRIM68
1.101	0.957	1.043										P53804	TTC3	E3 ubiquitin-protein ligase TTC3
0.991	0.992	0.995	1.003	0.939	0.998	1.037	1.122	1.022	0.980	0.957	1.002	Q8IWV7	UBR1	E3 ubiquitin-protein ligase UBR1
1.056	0.985	1.037	1.030	0.881	0.965	1.013	0.991	0.980	0.936	0.944	0.975	Q8IWV8	UBR2	E3 ubiquitin-protein ligase UBR2
1.007	1.011	1.018	0.993	0.956	1.024	0.985	1.040	1.005	0.957	0.996	0.980	Q5T4S7	UBR4	E3 ubiquitin-protein ligase UBR4
0.997	0.980	1.017	0.990	0.963	1.008	1.026	1.047	1.018	0.958	0.962	1.024	O95071	UBR5	E3 ubiquitin-protein ligase UBR5
1.057	0.896	1.046	1.046	0.946	0.959	1.032	1.022	1.012	0.875	0.883	1.037	A0A087WTW0	UHRF1	E3 ubiquitin-protein ligase UHRF1
1.179	1.035	1.086										Q96PU4	UHRF2	E3 ubiquitin-protein ligase UHRF2
0.977	1.001	1.074	1.010	1.014	1.164	1.051	0.979	1.069	1.059	1.018	1.240	P98170	XIAP	E3 ubiquitin-protein ligase XIAP
1.035	0.987	1.046	1.013	1.004	0.989	0.978	0.972	0.945	1.037	1.074	1.002	Q96JP5	ZFP91	E3 ubiquitin-protein ligase ZFP91
			1.063	0.870	0.894							H3BQQ2	ZNF598	E3 ubiquitin-protein ligase ZNF598
0.973	1.019	1.045	0.948	1.005	1.059	0.988	1.001	1.011	0.882	0.973	1.136	Q86UK7	ZNF598	E3 ubiquitin-protein ligase ZNF598
0.953	0.950	1.044	1.023	0.992	1.082	1.042	1.057	1.058	1.024	1.010	0.955	Q8NHG8	ZNRF2	E3 ubiquitin-protein ligase ZNRF2
1.015	1.009	1.017	1.004	0.970	1.010	0.986	1.004	1.021	1.002	1.038	1.011	O94874	UFL1	E3 UFM1-protein ligase 1
1.014	0.990	1.034	0.994	0.980	1.030	1.008	0.973	0.997	1.030	0.978	1.038	Q15075	EEA1	Early endosome antigen 1
0.962	1.003	0.984	1.009	0.964	1.053	1.020	1.058	1.002	1.062	1.039	1.057	H7C2Q8	EBNA1BP2	EBNA1 binding protein 2, isoform CRA_d
1.048	1.005	1.027	0.988	1.011	1.021	0.989	0.974	0.982	1.007	0.993	1.051	B7WPE2	EML3	Echinoderm microtubule associated protein like 3, isoform CRA_e
1.050	1.040	0.950	0.976	1.015	0.936	1.019	1.075	0.946	1.007	1.031	0.970	Q9HC35	EML4	Echinoderm microtubule-associated protein-like 4
0.983	0.984	1.013	1.001	1.050	1.008	1.000	0.951	0.993	1.028	1.005	1.040	B5MBZ0	EML4	Echinoderm microtubule-associated protein-like 4
1.296	1.194	0.955										Q16206	ENOX2	Ecto-NOX disulfide-thiol exchanger 2
1.009	0.986	0.983	1.003	0.977	0.995							Q9Y5L3	ENTPD2	Ectonucleoside triphosphate diphosphohydrolase 2
1.064	0.920	1.054										Q9Y227	ENTPD4	Ectonucleoside triphosphate diphosphohydrolase 4
1.125	1.013	1.078	1.018	0.920	1.062	0.922	1.132	0.928	1.042	1.011	1.044	O75356	ENTPD5	Ectonucleoside triphosphate diphosphohydrolase 5

Abundance Ratio														
1 d M1/C1	1 d M2/C2	1 d M3/C3	4 d M1/C1	4 d M2/C2	4 d M3/C3	7 d M1/C1	7 d M2/C2	7 d M3/C3	10 d M1/C1	10 d M2/C2	10 d M3/C3	Accession ID	Gene name	Protein Description
0.994	0.990	0.995	1.045	1.015	1.012	1.079	1.103	1.149				O75354	ENTPD6	Ectonucleoside triphosphate diphosphohydrolase 6
			0.971	0.996	1.017				0.971	0.928	1.165	Q9HCE0	EPG5	Ectopic P granules protein 5 homolog
									1.517	0.789	0.923	Q5JPI9	EEF1AKMT2	EEF1A lysine methyltransferase 2
0.809	1.095	0.958										O75071	EFCAB14	EF-hand calcium-binding domain-containing protein 14
						1.036	1.135	1.169				A8K855	EFCAB7	EF-hand calcium-binding domain-containing protein 7
0.965	0.916	0.953	1.046	1.067	1.176	0.831	1.144	1.066				Q9BUP0	EFHD1	EF-hand domain-containing protein D1
0.997	1.005	0.967	1.000	0.981	1.005	0.988	1.061	0.982	0.960	0.986	0.989	Q96C19	EFHD2	EF-hand domain-containing protein D2
1.072	1.007	1.022	1.005	0.948	1.015	1.007	1.018	1.010	1.013	0.986	1.013	Q5NDL2	EOGT	EGF domain-specific O-linked N-acetylglucosamine transferase
1.070	0.944	1.058	0.966	0.982	1.040	1.018	0.966	1.041	1.028	0.968	1.035	Q9GZT9	EGLN1	Egl nine homolog 1
0.993	0.978	1.046	0.992	0.919	1.010	1.005	0.915	1.039	1.002	0.947	1.078	Q8N3D4	EHBP1L1	EH domain-binding protein 1-like protein 1
0.973	1.000	1.010	0.973	0.980	0.986	0.973	1.038	0.997	0.946	0.974	0.975	A0A024R571	EHD1	EH domain-containing protein 1
0.962	0.995	1.015	0.976	0.944	1.022	0.994	1.010	1.030	1.022	0.955	0.961	Q9NZN4	EHD2	EH domain-containing protein 2
0.989	0.979	0.980	0.975	0.929	1.003	0.997	1.067	1.007	0.982	0.942	1.007	Q9H223	EHD4	EH domain-containing protein 4
0.976	0.989	1.020	0.988	0.979	1.013	0.986	0.995	1.014	0.971	0.990	0.998	Q92616	GCN1	eIF-2-alpha kinase activator GCN1
0.969	0.986	1.038	0.951	0.956	1.033	1.000	1.079	1.027	1.033	0.991	1.010	Q9P2K8	EIF2AK4	eIF-2-alpha kinase GCN2
1.025	0.983	0.953	0.924	0.947	1.008	1.112	1.028	1.085	1.096	0.965	1.042	Q9BXV9	GON7	EKC/KEOPS complex subunit GON7
1.027	0.945	1.048	1.022	0.938	1.036	1.029	0.955	0.995	1.140	0.998	1.214	Q14657	LAGE3	EKC/KEOPS complex subunit LAGE3
0.950	0.957	1.077	0.986	0.976	1.092	0.930	1.047	1.055	0.893	1.007	1.062	Q9Y3C4	TPRKB	EKC/KEOPS complex subunit TPRKB
1.019	0.997	1.010	0.987	1.096	0.999	1.003	0.954	1.012	0.984	0.988	0.990	P13804	ETFA	Electron transfer flavoprotein subunit alpha, mitochondrial
1.030	1.007	1.017	0.984	0.946	0.998	0.967	1.019	0.964	0.979	0.969	0.949	P38117	ETFB	Electron transfer flavoprotein subunit beta
0.993	0.979	1.022	0.983	0.986	1.039	1.018	1.017	1.032	1.078	0.989	1.101	Q16134	ETFDH	Electron transfer flavoprotein-ubiquinone oxidoreductase, mitochondrial
0.991	0.986	1.011	0.981	0.956	1.010	1.012	0.929	1.014	1.020	0.971	1.043	X6RLX0	ERC1	ELKS/Rab6-interacting/CAST family member 1
			0.979	0.922	1.039	1.062	1.131	1.091	1.051	1.062	1.097	Q96JC9	EAF1	ELL-associated factor 1
0.998	0.995	1.020	0.980	0.931	1.020	0.969	0.960	1.002	1.050	0.980	1.044	A0A1C7CYX1	ELMSAN1	ELM2 and SANT domain-containing protein 1
0.970	0.950	1.012	0.952	0.887	0.975	0.944	0.950	0.945	0.923	0.996	0.946	Q8IZ81	ELMOD2	ELMO domain-containing protein 2
1.101	0.895	1.031	0.946	1.357	0.954	0.976	1.006	1.128	0.816	1.047	0.923	A0A087WVQ9	EEF1A1	Elongation factor 1-alpha 1
1.029	0.955	0.976	1.037	1.022	0.995	1.017	0.915	0.990	1.064	0.998	0.997	Q05639	EEF1A2	Elongation factor 1-alpha 2
0.991	1.000	0.932	1.013	1.006	0.967	1.017	1.074	0.989	1.021	1.037	0.965	P24534	EEF1B2	Elongation factor 1-beta
0.990	0.987	0.984	0.993	1.056	0.976	0.982	0.971	0.978	0.958	1.006	0.969	E9PRY8	EEF1D	Elongation factor 1-delta
0.987	1.014	0.998	1.037	1.168	1.010	1.010	0.988	1.059	0.946	1.025	0.948	P13639	EEF2	Elongation factor 2

Abundance Ratio														
1 d M1/C1	1 d M2/C2	1 d M3/C3	4 d M1/C1	4 d M2/C2	4 d M3/C3	7 d M1/C1	7 d M2/C2	7 d M3/C3	10 d M1/C1	10 d M2/C2	10 d M3/C3	Accession ID	Gene name	Protein Description
1.019	1.009	1.004	1.008	0.991	0.978	0.995	1.004	0.993	1.024	0.987	0.977	Q96RP9	GFM1	Elongation factor G, mitochondrial
0.994	0.979	0.992	0.984	1.065	0.942	1.016	0.876	1.012	1.011	0.939	0.988	P43897	TSFM	Elongation factor Ts, mitochondrial
1.018	0.990	0.991	1.012	1.058	0.978	1.030	1.011	1.024	1.011	0.989	1.018	P49411	TUFM	Elongation factor Tu, mitochondrial
1.005	0.998	0.998	1.010	0.995	0.970	1.024	1.028	1.002	1.029	0.984	1.048	Q7Z2Z2	EFL1	Elongation factor-like GTPase 1
0.972	1.050	0.947	0.934	0.850	0.903	1.062	1.067	0.923	1.012	1.038	1.145	Q9BW60	ELOVL1	Elongation of very long chain fatty acids protein 1
1.044	0.946	0.906	0.995	0.908	0.979	1.072	1.148	0.998	1.044	0.972	1.007	A1L3X0	ELOVL7	Elongation of very long chain fatty acids protein 7
0.997	0.992	1.014	1.010	0.987	0.999	1.015	1.049	1.039	1.000	0.975	1.058	O95163	IKBKAP	Elongator complex protein 1
1.033	1.042	0.996	1.051	1.085	1.115	1.061	1.109	1.058	1.037	1.010	1.098	Q9H9T3	ELP3	Elongator complex protein 3
1.023	1.012	1.009	0.990	0.992	0.992	0.987	1.004	0.986	1.004	0.974	1.005	Q96EB1	ELP4	Elongator complex protein 4
1.066	1.068	1.015	0.990	0.942	0.977	1.027	1.035	1.003	1.005	0.976	1.032	Q0PNE2	ELP6	Elongator complex protein 6
1.004	0.985	1.045	1.021	0.980	1.049	1.025	0.999	1.003	1.004	1.045	1.034	Q14241	ELOA	Elongin-A
1.028	0.997	0.978	1.017	0.996	1.018	1.007	1.033	1.016	1.008	0.993	1.012	Q15370	ELOB	Elongin-B
0.990	0.999	1.046	1.037	1.024	1.010	1.007	0.957	0.996	0.995	1.037	0.924	Q15369	ELOC	Elongin-C
0.979	0.978	1.006	1.057	0.989	1.007				1.013	0.916	1.046	Q6PCB8	EMB	Embigin
1.012	0.967	0.986	0.970	0.979	0.982	0.997	1.011	1.043	0.977	0.953	1.010	Q96FZ2	HMCES	Embryonic stem cell-specific 5-hydroxymethylcytosine-binding protein
0.965	1.006	0.984	1.016	0.923	0.988	1.050	1.029	0.947	1.119	1.075	1.069	P50402	EMD	Emerin
0.867	0.928	0.918	0.948	0.899	0.909	1.023	1.111	1.001	0.981	1.026	0.908	A0A0U1RQV1	ERVK3-1	Endogenous retrovirus group K3 member 1 (Fragment)
0.964	1.012	1.158	0.958	0.946	1.000	1.024	0.892	0.965	0.939	0.936	1.062	P17813	ENG	Endoglin
1.166	1.053	1.089	0.958	0.940	0.960	0.982	1.222	0.992	0.946	1.012	0.998	Q969S2	NEIL2	Endonuclease 8-like 2
1.020	1.039	1.000	1.065	1.070	1.042	0.984	1.003	1.021	1.062	1.124	1.075	O94919	ENDOD1	Endonuclease domain-containing 1 protein
0.999	0.984	0.980	0.944	1.040	0.978	0.963	0.998	1.013	1.002	0.981	0.986	Q14249	ENDOG	Endonuclease G, mitochondrial
1.090	1.031	1.031	1.061	1.048	1.043	1.063	1.061	1.079	1.043	1.024	1.092	P78549	NTHL1	Endonuclease III-like protein 1
			0.996	0.958	1.096	0.966	1.126	1.024	1.106	0.808	0.979	Q8N8Q3	ENDOV	Endonuclease V
1.015	1.078	0.916	1.034	0.842	1.108				1.089	1.032	1.154	Q7L9B9	EEPD1	Endonuclease/exonuclease/phosphatase family domain-containing protein 1
1.000	1.000	1.012	0.993	0.985	1.009	0.978	1.017	0.960	0.981	1.009	0.987	Q99961	SH3GL1	Endophilin-A2
1.016	1.022	1.008	0.986	0.972	0.980	0.975	1.049	0.990	1.022	0.979	0.998	Q9Y371	SH3GLB1	Endophilin-B1
0.991	1.001	1.001	1.009	1.009	1.011	1.024	1.002	1.026	1.048	0.992	1.030	B7ZC38	SH3GLB2	Endophilin-B2
1.038	1.046	0.997	0.992	0.999	1.033	1.022	1.021	1.022	1.043	1.021	1.030	Q96DZ1	ERLEC1	Endoplasmic reticulum lectin 1
0.995	0.991	1.042	0.991	0.917	1.034	1.010	0.996	1.037	1.027	1.021	1.041	Q9UKM7	MAN1B1	Endoplasmic reticulum mannosyl-oligosaccharide 1,2-alpha-mannosidase
0.929	0.982	0.984	0.956	0.944	0.989	1.028	0.989	1.009	1.121	1.058	1.081	Q7Z2K6	ERMP1	Endoplasmic reticulum metallopeptidase 1

Abundance Ratio														
1 d M1/C1	1 d M2/C2	1 d M3/C3	4 d M1/C1	4 d M2/C2	4 d M3/C3	7 d M1/C1	7 d M2/C2	7 d M3/C3	10 d M1/C1	10 d M2/C2	10 d M3/C3	Accession ID	Gene name	Protein Description
0.976	1.028	0.951	0.992	1.010	0.993	0.978	1.026	0.990	0.985	0.997	0.964	P30040	ERP29	Endoplasmic reticulum resident protein 29
1.011	1.022	0.970	1.014	1.017	0.994	1.025	1.042	1.010	1.001	1.021	1.009	Q9BS26	ERP44	Endoplasmic reticulum resident protein 44
0.994	1.008	0.975	0.980	0.914	0.934	1.008	1.019	0.961	1.103	1.011	0.971	Q969X5	ERGIC1	Endoplasmic reticulum-Golgi intermediate compartment protein 1
1.011	0.996	0.991	1.058	0.965	1.080	1.086	1.042	0.985	0.979	0.994	1.004	A0A087WU02	ERGIC2	Endoplasmic reticulum-Golgi intermediate compartment protein 2
0.975	1.008	1.016	0.977	0.947	0.986	1.002	0.989	0.983	1.024	1.020	1.074	H0Y5K5	ERGIC3	Endoplasmic reticulum-Golgi intermediate compartment protein 3 (Fragment)
0.970	1.013	0.972	0.999	1.004	1.007	0.979	0.987	0.971	0.986	0.990	0.960	P14625	HSP90B1	Endoplasmin
1.157	1.090	1.192	0.936	0.900	1.139	0.939	1.032	0.895	0.984	0.958	0.935	Q9UPY3	DICER1	Endoribonuclease Dicer
1.041	1.008	1.020	1.041	0.997	1.000	1.053	1.102	1.014	1.022	0.994	0.991	Q53H82	LACTB2	Endoribonuclease LACTB2
									0.891	0.971	1.207	Q5D1E8	ZC3H12A	Endoribonuclease ZC3H12A
1.042	1.068	1.042	1.015	0.987	1.057	1.107	1.118	1.000	1.068	1.039	1.105	Q96AP7	ESAM	Endothelial cell-selective adhesion molecule
0.967	0.956	0.987	0.942	0.999	1.008	0.961	0.800	0.953	1.031	0.960	1.013	O60869	EDF1	Endothelial differentiation-related factor 1
0.972	0.972	1.002	0.998	0.966	1.036	1.042	1.085	1.072	1.013	1.037	1.096	Q9UNN8	PROCR	Endothelial protein C receptor
0.970	1.034	1.023	1.046	0.978	1.025	1.043	1.028	1.013	1.072	1.030	1.058	P42892	ECE1	Endothelin-converting enzyme 1
			0.830	0.924	1.191							O60344	ECE2	Endothelin-converting enzyme 2
1.022	0.993	1.057	0.992	0.955	1.026	0.996	1.028	1.002	0.947	0.966	1.009	Q96JJ3	ELMO2	Engulfment and cell motility protein 2
0.958	0.959	1.046	1.005	1.016	1.017	1.014	0.940	1.022	1.021	1.000	1.042	Q96F86	EDC3	Enhancer of mRNA-decapping protein 3
0.961	0.977	1.009	0.998	0.938	1.035	0.973	0.977	1.029	0.983	0.992	1.024	Q6P2E9	EDC4	Enhancer of mRNA-decapping protein 4
1.008	0.950	1.000	1.020	0.967	0.867	0.922	0.655	0.969	0.985	0.793	1.724	Q52LR7	EPC2	Enhancer of polycomb homolog 2
0.984	1.016	0.934	1.021	1.285	0.955	1.072	0.860	0.958	1.081	1.054	1.003	P84090	ERH	Enhancer of rudimentary homolog
1.016	0.975	1.006	1.012	0.983	0.992	0.978	1.048	1.004	0.957	1.024	0.977	Q9UHY7	ENOPH1	Enolase-phosphatase E1
1.010	1.040	0.988	1.010	1.127	1.004	1.006	0.984	1.016	1.044	1.036	0.987	Q9BV79	MECR	Enoyl-[acyl-carrier-protein] reductase, mitochondrial
0.988	1.007	0.992	0.981	1.008	1.010	0.984	0.994	0.996	1.007	1.039	0.972	P42126	ECI1	Enoyl-CoA delta isomerase 1, mitochondrial
0.933	1.007	0.999	0.951	0.967	1.016	1.007	1.012	1.004	0.990	0.981	1.034	O75521	ECI2	Enoyl-CoA delta isomerase 2, mitochondrial
			1.068	1.179	0.829	1.158	1.162	0.742	1.256	0.895	1.002	Q86YB7	ECHDC2	Enoyl-CoA hydratase domain-containing protein 2, mitochondrial
0.995	0.987	0.992	1.003	1.193	0.980	1.016	0.894	1.066	1.014	0.996	1.015	P30084	ECHS1	Enoyl-CoA hydratase, mitochondrial
0.978	1.023	1.087	1.041	1.041	1.065	1.044	0.881	1.020	1.079	1.024	1.147	Q14244	MAP7	Ensconsin
1.008	0.986	1.025	0.964	0.967	0.990	0.981	0.960	0.971	1.066	0.976	1.077	Q92817	EVPL	Envoplakin
1.018	0.991	1.000	1.058	0.965	1.064	1.022	1.074	1.016	0.962	1.051	0.999	P29317	EPHA2	Ephrin type-A receptor 2
1.173	0.714	1.181	1.076	0.828	1.105				1.436	0.901	1.589	P54753	EPHB3	Ephrin type-B receptor 3
1.342	1.016	1.050	1.097	1.247	1.003	1.126	0.955	1.092	1.114	1.057	1.027	P20827	EFNA1	Ephrin-A1
1.200	1.294	1.328	0.986	0.864	1.231	0.914	1.206	0.840				P52803	EFNA5	Ephrin-A5

Abundance Ratio												Accession ID	Gene name	Protein Description
1 d M1/C1	1 d M2/C2	1 d M3/C3	4 d M1/C1	4 d M2/C2	4 d M3/C3	7 d M1/C1	7 d M2/C2	7 d M3/C3	10 d M1/C1	10 d M2/C2	10 d M3/C3			
0.900	1.043	1.106	0.898	0.947	0.896	1.023	1.121	1.030	0.896	0.974	1.113	P98172	EFNB1	Ephrin-B1
			1.129	1.230	0.784							Q15768	EFNB3	Ephrin-B3
1.033	0.994	1.017	1.025	1.001	1.020	1.046	1.068	1.017	1.056	0.959	1.072	Q12929	EPS8	Epidermal growth factor receptor kinase substrate 8
1.000	1.009	1.023	1.012	1.012	0.993	1.012	1.022	1.000	1.011	0.990	1.014	Q9H6S3	EPS8L2	Epidermal growth factor receptor kinase substrate 8-like protein 2
1.003	0.990	1.022	1.006	1.012	1.006	1.078	1.016	1.039	1.057	1.012	1.083	P00533	EGFR	Epidermal growth factor receptor
0.985	0.998	1.020	0.987	0.979	1.007	0.983	1.048	1.021	0.990	0.983	1.016	P42566	EPS15	Epidermal growth factor receptor substrate 15
0.951	1.033	1.019	1.097	1.007	1.200	0.978	1.095	1.131				M0QY01	EPS15L1	Epidermal growth factor receptor substrate 15-like 1 (Fragment)
0.898	0.932	1.036	1.059	0.747	1.257	1.058	0.992	1.042	1.070	0.958	1.036	Q9UHF1	EGFL7	Epidermal growth factor-like protein 7
0.925	0.937	0.978	1.008	0.899	1.135	1.092	1.024	1.011	0.996	0.992	1.245	G3V145	SDR16C5	Epidermal retinol dehydrogenase 2
1.111	0.980	0.949	1.044	1.183	1.004	1.164	0.870	1.071	1.163	1.111	1.077	P61916	NPC2	Epididymal secretory protein E1
1.125	0.958	1.033	1.052	0.998	1.040	1.026	1.062	1.038	1.096	1.036	0.980	Q9Y2E5	MAN2B2	Epididymis-specific alpha-mannosidase
						0.892	1.199	0.888				A0A087X1U6	EPPK1	Epiplakin
0.833	0.927	0.882	1.075	0.911	1.010	1.110	1.151	1.076	1.194	1.278	1.212	P54852	EMP3	Epithelial membrane protein 3
1.131	1.020	0.970	1.066	0.978	1.028	1.013	1.031	1.021	1.034	1.049	1.024	Q7L775	EPM2AIP1	EPM2A-interacting protein 1
0.969	0.981	1.003	0.982	0.896	0.974	0.990	0.981	0.984	1.033	0.947	0.987	P07099	EPHX1	Epoxide hydrolase 1
0.970	1.006	1.160	1.009	0.976	1.316	1.202	1.006	1.119	0.821	0.957	1.075	Q8IUS5	EPHX4	Epoxide hydrolase 4
1.036	1.003	1.023	1.005	1.019	1.015	0.970	1.022	1.029	0.979	1.008	0.996	O95208	EPN2	Epsin-2
1.005	1.059	0.864	1.053	1.134	0.917	0.819	1.276	1.347				Q9BV94	EDEM2	ER degradation-enhancing alpha-mannosidase-like protein 2
1.040	1.016	0.989	1.039	0.939	0.976	1.094	1.141	1.205	1.020	0.898	1.065	Q9BZQ6	EDEM3	ER degradation-enhancing alpha-mannosidase-like protein 3
			0.952	1.238	1.403				1.714	1.147	1.290	P33947	KDELR2	ER lumen protein-retaining receptor 2
1.151	0.991	0.869	1.093	0.907	1.193	1.009	1.311	1.009				O43731	KDELR3	ER lumen protein-retaining receptor 3
0.985	1.012	1.019	0.995	0.962	1.016	0.983	1.075	1.006	0.964	1.019	0.984	Q8N766	EMC1	ER membrane protein complex subunit 1
0.995	0.995	0.960	0.998	0.930	0.946	1.019	1.037	0.971	1.008	0.998	1.049	M0R2A0	EMC10	ER membrane protein complex subunit 10
1.025	1.028	0.998	1.039	0.983	1.008	0.983	1.052	1.009	0.962	1.055	0.912	Q15006	EMC2	ER membrane protein complex subunit 2
1.080	0.974	1.007	1.031	0.917	1.062	1.042	1.173	1.008	1.033	1.117	1.040	Q9P0I2	EMC3	ER membrane protein complex subunit 3
0.979	0.967	1.019	0.954	0.921	1.059	0.995	1.046	1.041	1.039	1.061	1.008	Q5J8M3	EMC4	ER membrane protein complex subunit 4
									1.169	1.104	1.153	Q9BV81	EMC6	ER membrane protein complex subunit 6
1.054	0.874	0.956	0.990	0.977	1.017	0.999	1.000	0.980	1.014	0.916	0.956	Q9NPA0	EMC7	ER membrane protein complex subunit 7
1.020	1.012	0.969	1.015	1.102	1.026	1.032	0.970	1.041	1.052	1.031	1.041	O43402	EMC8	ER membrane protein complex subunit 8
			0.795	0.807	0.919	0.950	1.406	0.985				Q9Y3B6	EMC9	ER membrane protein complex subunit 9
0.979	1.056	1.009	1.166	1.162	1.145	1.180	1.190	1.150	0.966	1.019	1.105	Q9UJM3	ERRFI1	ERBB receptor feedback inhibitor 1

Abundance Ratio														
1 d M1/C1	1 d M2/C2	1 d M3/C3	4 d M1/C1	4 d M2/C2	4 d M3/C3	7 d M1/C1	7 d M2/C2	7 d M3/C3	10 d M1/C1	10 d M2/C2	10 d M3/C3	Accession ID	Gene name	Protein Description
						0.558	1.101	1.596				H0Y4B0	ERI3	ERI1 exoribonuclease 3 (Fragment)
0.915	0.952	0.926	0.966	1.027	1.048	1.012	1.058	1.025	0.989	1.021	1.020	O43414	ERI3	ERI1 exoribonuclease 3
0.963	0.978	0.994	0.998	0.935	0.985	1.011	1.029	1.009	1.049	0.968	1.034	O75477	ERLIN1	Erlin-1
0.983	0.993	0.992	0.997	0.985	0.999	0.986	1.025	0.971	1.043	1.006	0.975	O94905	ERLIN2	Erlin-2
0.993	1.006	1.002	0.982	0.962	1.017	0.971	0.999	0.996	0.923	0.967	0.921	Q96HE7	ERO1A	ERO1-like protein alpha
1.019	0.976	1.053	0.982	0.971	1.059	0.963	1.115	1.028	0.929	1.016	1.002	Q86YB8	ERO1B	ERO1-like protein beta
0.965	0.998	0.989	1.072	0.942	1.084	1.116	1.158	1.124	1.136	1.131	1.131	P27105	STOM	Erythrocyte band 7 integral membrane protein
1.037	1.001	1.043	1.013	0.970	1.055	0.965	1.033	1.003	0.916	1.029	0.941	Q9H501	ESF1	ESF1 homolog
			1.019	1.033	1.163	0.924	0.909	1.110	1.332	0.817	1.028	Q9H4I9	SMDT1	Essential MCU regulator, mitochondrial
1.042	1.038	0.977	1.021	1.060	0.959	0.984	1.066	1.040	0.956	0.975	0.959	Q9H0W9	C11orf54	Ester hydrolase C11orf54
1.068	1.096	1.101	0.977	0.795	0.967	1.122	1.067	0.970	1.042	0.955	1.044	Q8WZ82	OVCA2	Esterase OVCA2
0.946	0.986	0.956	0.966	0.864	0.983	0.966	1.022	0.963	0.909	0.977	0.929	Q8NBQ5	HSD17B11	Estradiol 17-beta-dehydrogenase 11
1.013	0.983	1.009	0.956	0.919	1.014	1.026	1.057	0.970	0.942	0.896	1.081	Q9HBU6	ETNK1	Ethanolamine kinase 1
1.024	0.995	0.997	0.997	0.966	1.017	1.020	1.052	1.027	1.040	1.030	1.044	Q9NTX5	ECHDC1	Ethylmalonyl-CoA decarboxylase
0.991	1.003	1.090	1.036	0.955	1.057	1.003	0.932	1.058	1.056	1.075	1.129	O14681	EI24	Etoposide-induced protein 2.4 homolog
1.044	1.029	1.012	1.005	0.917	1.107	0.976	1.027	0.994	1.034	1.119	1.050	P19419	ELK1	ETS domain-containing protein Elk-1
						1.056	1.065	1.099				P41970	ELK3	ETS domain-containing protein Elk-3
1.071	1.003	1.016	1.031	1.059	0.924	1.034	1.128	1.048	1.046	0.985	0.987	P50548	ERF	ETS domain-containing transcription factor ERF
0.988	1.021	0.980	0.942	0.969	0.898	0.898	1.213	1.071	1.190	0.871	1.212	P32519	ELF1	ETS-related transcription factor Elf-1
0.920	1.191	1.720	1.367	0.608	0.783							Q15723	ELF2	ETS-related transcription factor Elf-2
1.108	1.012	1.001	1.013	0.840	0.970	0.901	1.239	1.136				P78545	ELF3	ETS-related transcription factor Elf-3
1.060	0.950	1.013	0.932	0.985	1.023	0.935	0.991	1.313	0.866	0.934	1.224	O00418	EEF2K	Eukaryotic elongation factor 2 kinase
0.981	1.002	1.002	1.015	1.008	1.029	1.011	1.075	1.052	0.966	0.993	0.983	P60842	EIF4A1	Eukaryotic initiation factor 4A-I
0.996	0.981	0.979	0.985	0.965	1.010	1.015	1.053	0.995	0.966	1.015	0.992	P38919	EIF4A3	Eukaryotic initiation factor 4A-III
0.990	0.996	1.003	0.991	1.006	1.002	1.001	1.034	1.041	0.990	0.964	1.015	P62495	ETF1	Eukaryotic peptide chain release factor subunit 1
1.007	0.976	1.016	0.961	0.965	1.022	0.969	1.017	0.994	0.989	0.982	0.986	O43324	EEF1E1	Eukaryotic translation elongation factor 1 epsilon-1
1.152	1.032	0.988	1.172	1.220	1.041	1.137	0.910	1.241	1.030	1.198	1.002	K7EM18	EIF1	Eukaryotic translation initiation factor 1
1.010	0.997	0.987	0.979	0.996	0.993	1.003	0.954	0.965	1.002	0.960	0.998	P47813	EIF1AX	Eukaryotic translation initiation factor 1A, X-chromosomal
			1.289	2.089	0.874	1.314	0.613	1.458	0.954	1.097	1.049	O60739	EIF1B	Eukaryotic translation initiation factor 1b
0.995	1.016	0.989	1.011	0.993	0.993	1.013	1.050	0.980	1.001	1.003	0.999	P05198	EIF2S1	Eukaryotic translation initiation factor 2 subunit 1
1.006	0.988	0.992	1.015	1.090	0.973	1.033	0.928	0.990	1.016	0.994	1.016	P20042	EIF2S2	Eukaryotic translation initiation factor 2 subunit 2

Abundance Ratio														
1 d M1/C1	1 d M2/C2	1 d M3/C3	4 d M1/C1	4 d M2/C2	4 d M3/C3	7 d M1/C1	7 d M2/C2	7 d M3/C3	10 d M1/C1	10 d M2/C2	10 d M3/C3	Accession ID	Gene name	Protein Description
1.013	1.008	0.969	1.018	1.146	0.974	1.020	0.979	1.035	1.023	1.011	0.994	P41091	EIF2S3	Eukaryotic translation initiation factor 2 subunit 3
1.017	1.004	0.997	1.003	0.982	0.957	0.996	1.073	0.995	0.994	0.989	1.016	Q9BY44	EIF2A	Eukaryotic translation initiation factor 2A
									1.116	0.911	0.963	Q9NZJ5	EIF2AK3	Eukaryotic translation initiation factor 2-alpha kinase 3
1.020	0.999	1.044	1.087	0.965	1.061	1.065	0.956	1.044	1.116	1.024	1.131	P41214	EIF2D	Eukaryotic translation initiation factor 2D
0.969	1.009	1.011	1.003	1.032	1.024	1.002	0.941	1.010	1.020	1.018	1.037	Q14152	EIF3A	Eukaryotic translation initiation factor 3 subunit A
0.994	1.007	1.023	1.013	1.055	1.027	1.017	0.961	1.022	1.035	1.025	1.051	Q99613	EIF3C	Eukaryotic translation initiation factor 3 subunit C
0.972	1.008	0.987	1.031	1.013	1.001	1.028	1.050	0.982	1.032	1.042	0.997	O15371	EIF3D	Eukaryotic translation initiation factor 3 subunit D
0.985	1.011	0.973	1.002	0.988	1.024	1.012	1.088	1.005	0.993	1.011	1.003	P60228	EIF3E	Eukaryotic translation initiation factor 3 subunit E
1.017	1.028	0.999	1.027	1.011	1.033	1.061	1.083	1.019	0.999	1.029	1.005	O00303	EIF3F	Eukaryotic translation initiation factor 3 subunit F
1.009	1.006	1.014	1.020	1.034	1.022	1.001	1.026	1.043	1.004	1.031	1.026	O75821	EIF3G	Eukaryotic translation initiation factor 3 subunit G
0.983	1.000	1.006	1.004	0.978	1.014	0.995	1.021	1.010	0.996	1.045	1.068	B3KS98	EIF3S3	Eukaryotic translation initiation factor 3 subunit H
0.972	0.985	0.986	1.013	0.993	1.034	1.004	1.024	1.018	0.987	0.996	1.020	Q13347	EIF3I	Eukaryotic translation initiation factor 3 subunit I
1.002	0.994	0.974	0.984	0.965	0.963	1.019	0.992	0.972	1.057	1.057	1.073	O75822	EIF3J	Eukaryotic translation initiation factor 3 subunit J
1.021	0.982	1.009	1.006	1.047	1.044	0.989	0.987	1.019	0.984	1.019	1.031	Q9UBQ5	EIF3K	Eukaryotic translation initiation factor 3 subunit K
1.016	1.008	1.010	1.009	0.976	1.019	1.018	1.085	1.011	0.995	1.052	0.972	B0QY89	EIF3L	Eukaryotic translation initiation factor 3 subunit L
1.077	1.029	1.047	1.045	1.163	1.008	0.991	1.040	1.091	0.923	1.024	0.929	Q7L2H7	EIF3M	Eukaryotic translation initiation factor 3 subunit M
0.992	0.993	1.010	1.006	0.975	1.017	1.013	1.022	1.009	0.981	1.006	1.015	D3DQV9	EIF4G2	Eukaryotic translation initiation factor 4 gamma 2 (Fragment)
1.467	1.100	1.413										A0A0A0MSA7	EIF4G3	Eukaryotic translation initiation factor 4 gamma 3
1.015	0.994	1.030	1.000	0.959	1.015	0.989	0.971	0.994	1.043	0.960	1.050	A0A0U1RQK7	EIF4G3	Eukaryotic translation initiation factor 4 gamma 3
0.986	0.997	0.984	0.989	0.998	0.991	0.988	0.904	0.986	1.058	1.047	1.084	E7EX17	EIF4B	Eukaryotic translation initiation factor 4B
1.004	1.009	0.976	0.995	1.024	0.993	0.988	1.016	0.988	0.987	1.008	0.963	D6RBW1	EIF4E	Eukaryotic translation initiation factor 4E
1.007	0.992	0.990	0.983	0.972	0.969	0.952	1.056	1.015	1.031	1.005	1.077	O60573	EIF4E2	Eukaryotic translation initiation factor 4E type 2

Abundance Ratio														
1 d M1/C1	1 d M2/C2	1 d M3/C3	4 d M1/C1	4 d M2/C2	4 d M3/C3	7 d M1/C1	7 d M2/C2	7 d M3/C3	10 d M1/C1	10 d M2/C2	10 d M3/C3	Accession ID	Gene name	Protein Description
			0.721	1.000	1.314							Q13541	EIF4EBP1	Eukaryotic translation initiation factor 4E-binding protein 1
1.066	1.039	1.045										Q13542	EIF4EBP2	Eukaryotic translation initiation factor 4E-binding protein 2
1.023	1.022	1.018	1.023	1.087	1.024	1.038	0.984	1.034	0.944	0.958	1.021	P55010	EIF5	Eukaryotic translation initiation factor 5
0.914	0.962	0.992	0.933	0.913	0.994	1.037	0.984	1.063	1.078	0.954	1.079	Q9GZV4	EIF5A2	Eukaryotic translation initiation factor 5A-2
1.011	1.013	1.010	1.017	1.023	0.994	1.031	1.007	1.007	1.024	1.009	1.058	A0A087WUT6	EIF5B	Eukaryotic translation initiation factor 5B
0.995	0.980	1.013	1.010	1.228	0.991	1.059	0.941	1.073	0.993	1.005	0.996	P56537	EIF6	Eukaryotic translation initiation factor 6
0.982	1.018	1.014	1.052	0.993	1.028	1.004	1.146	0.995	1.030	1.008	0.995	Q9BQ95	ECSIT	Evolutionarily conserved signaling intermediate in Toll pathway, mitochondrial
1.043	1.024	1.025	1.046	1.181	1.018	1.060	0.960	1.077	0.976	0.994	1.033	Q9NV70	EXOC1	Exocyst complex component 1
1.007	1.017	1.023	0.985	0.979	1.039	0.997	1.044	1.018	0.967	0.991	1.002	Q96KP1	EXOC2	Exocyst complex component 2
1.004	1.011	1.056	1.030	0.980	1.025	1.031	1.040	1.013	1.051	0.987	1.026	O60645	EXOC3	Exocyst complex component 3
1.025	1.010	1.017	1.004	0.970	1.040	1.016	1.023	1.034	1.015	1.007	1.048	Q96A65	EXOC4	Exocyst complex component 4
1.009	1.012	1.013	1.002	1.028	1.023	1.005	0.988	0.984	1.032	0.986	1.030	O00471	EXOC5	Exocyst complex component 5
1.074	1.012	1.053	1.005	0.955	1.035	1.049	1.067	1.026	1.001	1.016	0.976	Q8TAG9	EXOC6	Exocyst complex component 6
1.005	1.012	1.010	0.999	0.985	0.966	0.975	1.080	1.000	1.007	0.991	0.999	Q9UPT5	EXOC7	Exocyst complex component 7
1.016	1.003	1.014	1.013	0.931	1.030	1.003	0.973	1.027	1.070	0.979	1.035	Q8IYI6	EXOC8	Exocyst complex component 8
1.029	0.991	1.028	1.022	0.953	1.041	1.016	0.996	1.019	1.009	0.997	1.026	A0A0U1RRB6	EXOC6B	Exocyst complex component
0.946	0.966	1.048	0.967	1.012	1.011	1.017	0.992	1.008	1.037	0.973	1.078	Q9NVH0	EXD2	Exonuclease 3'-5' domain-containing protein 2
0.930	0.928	1.067	0.984	0.931	1.015	1.000	0.986	1.004	0.983	1.004	1.074	Q86TP1	PRUNE1	Exopolyphosphatase PRUNE1
1.060	0.992	1.017	0.978	0.986	0.988	0.959	0.982	0.999	0.964	1.018	1.010	Q9Y3B2	EXOSC1	Exosome complex component CSL4
0.959	0.995	0.987	1.031	1.031	1.038	1.009	0.954	1.027	1.035	0.989	1.024	Q5RKV6	EXOSC6	Exosome complex component MTR3
0.984	0.992	0.979	1.009	0.988	0.999	0.987	1.081	0.959	0.974	1.010	1.080	Q13868	EXOSC2	Exosome complex component RRP4
1.048	0.990	0.987	0.981	0.942	0.995	1.004	1.058	1.004	0.967	0.965	0.988	Q9NQT5	EXOSC3	Exosome complex component RRP40
1.004	1.020	1.030	1.005	0.891	1.043	1.001	1.084	0.993	0.967	0.999	0.971	Q9NPD3	EXOSC4	Exosome complex component RRP41
0.964	0.977	0.995	0.974	0.955	1.046	0.960	0.921	1.004	0.995	0.975	1.042	Q15024	EXOSC7	Exosome complex component RRP42
1.012	0.984	0.984	0.983	0.965	1.007	0.993	1.026	1.032	0.996	0.994	1.040	Q96B26	EXOSC8	Exosome complex component RRP43
1.000	0.953	0.979	0.979	0.982	1.012	0.978	1.014	1.022	0.976	0.994	1.016	Q9NQT4	EXOSC5	Exosome complex component RRP46
1.011	1.002	1.002	1.000	0.949	0.981	0.992	1.037	0.990	0.968	0.971	1.032	Q9Y2L1	DIS3	Exosome complex exonuclease RRP44
0.977	0.994	1.003	0.999	0.970	1.019	1.000	1.021	1.006	0.986	0.995	1.029	Q01780	EXOSC10	Exosome component 10
1.028	1.034	0.993	0.885	0.912	1.006	0.882	1.194	1.107	1.002	0.963	1.081	Q16394	EXT1	Exostosin-1
1.015	1.046	1.029	1.216	0.948	1.068	1.022	0.780	1.052	1.101	0.975	1.049	Q9UBQ6	EXTL2	Exostosin-like 2
1.131	1.087	1.138	0.894	0.958	0.950	0.978	1.166	0.911				O43909	EXTL3	Exostosin-like 3
0.994	1.014	0.992	0.997	0.974	1.016	0.979	1.065	1.018	0.941	1.013	0.935	O14980	XPO1	Exportin-1

Abundance Ratio														
1 d M1/C1	1 d M2/C2	1 d M3/C3	4 d M1/C1	4 d M2/C2	4 d M3/C3	7 d M1/C1	7 d M2/C2	7 d M3/C3	10 d M1/C1	10 d M2/C2	10 d M3/C3	Accession ID	Gene name	Protein Description
1.004	0.999	1.003	1.009	0.981	1.020	1.047	1.086	1.047	0.973	1.016	1.013	P55060	CSE1L	Exportin-2
1.026	1.007	1.035	1.025	0.941	1.046	1.005	1.055	1.037	0.952	1.021	0.981	Q9C0E2	XPO4	Exportin-4
0.989	0.998	1.027	0.991	0.989	1.073	1.013	1.097	1.059	0.961	1.050	0.998	Q9HAV4	XPO5	Exportin-5
1.059	0.948	1.132	0.991	1.001	1.150	1.162	1.024	1.207	0.904	0.916	1.154	Q96QU8	XPO6	Exportin-6
1.029	0.994	0.990	1.018	0.940	1.055	1.005	1.045	0.996	1.003	1.056	1.003	E7ESC6	XPO7	Exportin-7
1.068	0.984	0.993	1.107	0.945	1.055	1.041	1.074	1.060	0.927	0.986	0.953	O43592	XPOT	Exportin-T
0.978	0.993	0.998	0.997	0.973	1.009	1.030	1.052	1.023	1.035	0.983	0.996	Q9BSJ8	ESYT1	Extended synaptotagmin-1
1.028	1.084	1.005	1.032	1.012	0.957	1.019	1.118	0.984	1.036	1.066	1.086	Q99504	EYA3	Eyes absent homolog 3
0.988	1.008	1.000	1.005	0.997	1.003	0.977	0.995	1.001	0.984	1.026	0.984	E7EQR4	EZR	Ezrin
0.976	1.009	0.983	1.006	1.019	0.999	1.016	1.029	0.999	0.995	1.002	1.005	Q9Y5B9	SUPT16H	FACT complex subunit SPT16
1.024	1.004	1.012	0.994	0.990	0.988	0.981	1.023	1.004	0.961	0.979	0.974	Q08945	SSRP1	FACT complex subunit SSRP1
1.029	1.034	1.003	1.010	1.144	0.950	1.005	1.008	1.031	0.973	1.013	0.993	P52907	CAPZA1	F-actin-capping protein subunit alpha-1
1.013	0.985	0.986	0.985	0.974	0.948	0.983	1.054	1.010	0.964	1.006	0.977	P47755	CAPZA2	F-actin-capping protein subunit alpha-2
0.977	1.012	1.028	0.959	0.966	1.026	0.997	0.987	1.027	0.986	0.973	1.044	Q5VZK9	CARMIL1	F-actin-uncapping protein LRRC16A
1.087	1.060	1.053	0.996	0.970	1.022	0.976	1.045	1.006	0.957	0.936	1.025	P23610	F8A1	Factor VIII intron 22 protein
1.053	1.010	1.060	1.003	1.001	1.038	1.028	1.130	1.086	1.041	0.963	1.050	Q8NFF5	FLAD1	FAD synthase
0.985	1.022	1.010	1.011	0.983	1.042	1.039	1.024	1.035	1.055	1.008	1.032	Q96CU9	FOXRED1	FAD-dependent oxidoreductase domain-containing protein 1
0.988	1.031	1.039	1.132	0.863	0.946	1.058	0.840	0.993	1.003	1.005	1.002	P55789	GFER	FAD-linked sulfhydryl oxidase ALR
0.987	1.021	1.071				0.976	1.000	0.926	1.084	1.210	0.980	D3DWX8	FAM3A	Family with sequence similarity 3, member A, isoform CRA_c
0.841	1.031	1.379	0.974	0.954	1.067							Q0VG06	FAAP100	Fanconi anemia core complex-associated protein 100
1.012	0.993	1.116	0.832	0.975	1.022	0.886	0.954	0.857	0.814	0.934	0.944	O15360	FANCA	Fanconi anemia group A protein
0.971	0.950	0.932	0.855	1.024	0.983							O15287	FANCG	Fanconi anemia group G protein
1.022	1.032	1.027	1.033	0.950	0.947	1.051	1.207	1.064	0.882	0.941	0.868	Q9NVI1	FANCI	Fanconi anemia group I protein
0.986	0.997	0.984	1.004	0.998	0.989	0.999	0.958	0.962	1.006	1.005	1.019	Q96AE4	FUBP1	Far upstream element-binding protein 1
1.004	0.990	1.006	0.968	1.003	1.006	0.991	0.927	0.996	1.002	1.025	1.047	Q92945	KHSRP	Far upstream element-binding protein 2
1.005	1.004	1.009	0.996	1.010	1.024	0.994	0.996	1.012	1.005	1.046	1.034	Q96I24	FUBP3	Far upstream element-binding protein 3
0.972	0.997	0.974	0.997	0.967	0.990	1.060	1.080	1.024	1.108	1.093	1.062	P14324	FDPS	Farnesyl pyrophosphate synthase
0.915	1.017	0.948	0.957	0.951	1.010	1.044	0.990	0.906	0.813	1.041	0.912	Q13158	FADD	FAS-associated death domain protein
1.006	0.980	1.022	0.979	0.984	1.024	0.994	1.004	1.035	1.007	1.006	1.042	Q9UNN5	FAF1	FAS-associated factor 1
0.980	0.962	1.016	1.032	0.939	1.005	0.999	0.954	0.962	0.989	1.013	0.966	Q96CS3	FAF2	FAS-associated factor 2
0.999	1.078	1.099	1.102	0.881	1.382	1.196	1.210	1.251	0.895	1.042	1.126	Q99689	FEZ1	Fasciculation and elongation protein zeta-1
1.013	0.994	0.984	0.976	1.010	0.959	0.979	1.002	0.989	0.958	0.974	0.970	Q16658	FSCN1	Fascin

Abundance Ratio														
1 d M1/C1	1 d M2/C2	1 d M3/C3	4 d M1/C1	4 d M2/C2	4 d M3/C3	7 d M1/C1	7 d M2/C2	7 d M3/C3	10 d M1/C1	10 d M2/C2	10 d M3/C3	Accession ID	Gene name	Protein Description
1.082	0.987	1.058	0.994	0.910	1.018	0.992	1.020	1.012	0.967	0.994	0.996	Q53R41	FASTKD1	FAST kinase domain-containing protein 1, mitochondrial
0.985	1.026	0.978	1.019	0.897	1.041	0.982	0.928	1.050	0.934	0.955	1.083	Q9NYY8	FASTKD2	FAST kinase domain-containing protein 2, mitochondrial
1.011	1.059	1.085	1.045	0.883	1.060	1.147	1.102	1.271	0.989	1.049	1.153	Q14CZ7	FASTKD3	FAST kinase domain-containing protein 3, mitochondrial
1.023	0.989	0.997	0.961	0.914	1.006	1.016	1.007	0.988	0.989	0.944	1.005	Q7L8L6	FASTKD5	FAST kinase domain-containing protein 5, mitochondrial
0.872	1.083	0.942	1.025	0.863	0.984	1.024	1.066	0.976	0.919	1.032	1.042	Q8N6M3	FITM2	Fat storage-inducing transmembrane protein 2
1.040	1.003	1.062	1.099	1.028	0.926	1.059	1.001	1.041	1.038	1.080	1.118	A0A0A0MR51	FADS1	Fatty acid desaturase 1
0.908	0.984	1.015	1.002	0.879	0.969	1.075	1.096	0.969	1.059	1.052	1.009	Q9Y5Q0	FADS3	Fatty acid desaturase 3
0.953	1.006	1.012	1.014	1.022	1.000	1.053	1.063	1.051	1.025	1.032	1.063	P49327	FASN	Fatty acid synthase
1.105	0.992	1.019	1.046	1.296	0.921	1.020	0.918	1.025	0.924	1.037	0.931	Q01469	FABP5	Fatty acid-binding protein, epidermal
1.091	0.965	1.000	1.011	1.005	1.049	1.049	1.197	1.081	1.054	1.008	1.120	Q8WVX9	FAR1	Fatty acyl-CoA reductase 1
			2.702	5.240	1.934	4.825	9.136	1.301				J3KTD9	ALDH3A2	Fatty aldehyde dehydrogenase (Fragment)
1.028	0.995	1.015	0.980	0.962	1.014	1.005	0.923	1.026	0.977	0.964	1.099	Q86WN1	FCHSD1	F-BAR and double SH3 domains protein 1
0.995	0.975	1.025	1.026	1.024	1.044	1.069	1.093	1.038	1.056	0.994	1.071	E9PG19	FCHSD2	F-BAR and double SH3 domains protein 2
1.007	0.998	1.015	1.023	0.976	0.979	0.989	1.023	0.988	1.023	0.970	1.052	Q0JRZ9	FCHO2	F-BAR domain only protein 2
0.941	0.990	0.982	0.953	0.908	1.066	1.004	1.044	1.023	1.035	1.068	1.054	J3KTA1	FBXL20	F-box and leucine-rich repeat protein 20, isoform CRA_a
1.191	0.987	1.017	1.128	0.904	1.015	1.001	1.086	0.932	1.496	0.889	1.089	Q96EF6	FBXO17	F-box only protein 17
0.947	0.964	0.999	0.996	1.025	0.995	1.011	1.024	1.075	0.985	0.973	0.980	Q9UK22	FBXO2	F-box only protein 2
0.990	1.150	1.120	1.005	1.018	1.019	1.130	1.343	1.032	1.033	0.986	1.048	O94952	FBXO21	F-box only protein 21
0.962	0.974	0.983	0.979	0.951	0.994	1.005	1.155	1.001	0.997	0.983	1.042	Q8NEZ5	FBXO22	F-box only protein 22
1.202	1.115	1.312				1.262	0.948	0.965	1.056	1.049	1.109	Q9NVF7	FBXO28	F-box only protein 28
1.123	1.026	1.042	0.996	1.060	0.979	0.920	1.077	1.204	1.014	0.965	1.006	Q9UK99	FBXO3	F-box only protein 3
0.972	0.992	1.033	0.919	1.062	1.062	1.072	1.013	1.029	0.957	0.976	1.032	Q8TB52	FBXO30	F-box only protein 30
									0.900	1.015	0.917	Q8NEA4	FBXO36	F-box only protein 36
0.990	0.969	0.983	1.000	0.970	0.922	1.054	1.080	0.910	1.150	0.999	0.970	Q9UKT5	FBXO4	F-box only protein 4
1.075	1.108	0.876	0.633	1.265	1.057	1.167	0.765	2.203				B7Z1P2	FBXO44	F-box only protein 44
0.836	1.193	1.145	0.981	0.988	1.105	1.046	0.931	1.044				Q6PJ61	FBXO46	F-box only protein 46
1.025	0.904	0.734										Q9UKT4	FBXO5	F-box only protein 5
1.035	1.074	0.981	1.018	0.974	1.011	0.978	1.040	0.971	1.068	1.051	1.032	Q9NRD1	FBXO6	F-box only protein 6
1.011	1.016	1.020	0.947	0.942	1.019	1.034	1.113	1.072	0.985	1.012	1.009	Q9Y3I1	FBXO7	F-box only protein 7
1.084	1.101	1.069	1.056	1.077	1.043	1.035	1.144	0.933	1.125	1.017	1.077	Q9NXK8	FBXL12	F-box/LRR-repeat protein 12
			1.079	0.821	0.862	0.936	1.045	1.064	1.030	1.046	1.039	Q9H469	FBXL15	F-box/LRR-repeat protein 15

Abundance Ratio														
1 d M1/C1	1 d M2/C2	1 d M3/C3	4 d M1/C1	4 d M2/C2	4 d M3/C3	7 d M1/C1	7 d M2/C2	7 d M3/C3	10 d M1/C1	10 d M2/C2	10 d M3/C3	Accession ID	Gene name	Protein Description
1.139	0.995	1.050	0.968	0.886	1.006	1.026	1.113	0.925	1.068	1.100	1.000	Q8N461	FBXL16	F-box/LRR-repeat protein 16
0.799	0.955	1.025										Q9UKT7	FBXL3	F-box/LRR-repeat protein 3
0.922	1.226	1.088	1.084	0.949	0.948	0.734	1.194	1.259				Q8N531	FBXL6	F-box/LRR-repeat protein 6
1.142	1.090	1.048	1.060	0.937	0.936	0.959	1.117	0.971	0.927	1.033	0.936	Q96CD0	FBXL8	F-box/LRR-repeat protein 8
									1.089	1.084	1.222	P0C2W1	FBXO45	F-box/SPRY domain-containing protein 1
1.069	0.973	1.090	1.262	1.163	1.034	0.925	1.061	1.214	1.062	0.865	0.897	Q9UKB1	FBXW11	F-box/WD repeat-containing protein 11
			1.226	1.175	0.857	1.009	0.915	1.096	0.994	1.103	1.072	Q9UKT8	FBXW2	F-box/WD repeat-containing protein 2
			0.927	0.921	0.966							Q969U6	FBXW5	F-box/WD repeat-containing protein 5
0.996	0.984	0.986	0.980	0.965	0.954	0.994	1.019	0.985	1.076	1.043	1.077	Q9BZK7	TBL1XR1	F-box-like/WD repeat-containing protein TBL1XR1
1.103	0.949	0.971	0.969	1.023	0.990	1.007	1.064	1.063	1.016	0.856	1.035	Q9P2Q2	FRMD4A	FERM domain-containing protein 4A
0.987	0.999	1.001	1.008	0.996	1.138	1.083	0.999	1.022	1.041	0.998	1.073	Q9BZ67	FRMD8	FERM domain-containing protein 8
0.999	1.009	1.019	0.987	0.979	0.988	0.987	0.988	0.990	1.037	0.997	1.040	Q9Y4F1	FARP1	FERM, RhoGEF and pleckstrin domain-containing protein 1
0.907	1.167	1.085	1.024	1.028	1.027				1.032	0.922	1.137	H7C3M7	FARP2	FERM, RhoGEF and pleckstrin domain-containing protein 2 (Fragment)
1.022	1.065	1.033	1.044	0.942	0.998	1.100	1.010	1.087	1.001	0.953	1.097	O94887	FARP2	FERM, RhoGEF and pleckstrin domain-containing protein 2
1.001	1.011	1.009	1.000	0.969	1.008	1.006	1.047	0.996	0.961	0.975	1.005	Q9BQL6	FERMT1	Fermitin family homolog 1
1.080	1.001	0.958	1.084	0.973	1.076	1.011	1.139	1.117	1.071	1.046	1.070	Q6P4F2	FDX2	Ferredoxin-2, mitochondrial
2.395	2.405	2.294	3.035	3.329	2.707	3.112	2.866	3.000	2.724	2.618	2.629	P02794	FTH1	Ferritin heavy chain
2.440	2.264	2.250	2.887	3.215	2.724	2.926	2.848	2.807	2.627	2.482	2.533	P02792	FTL	Ferritin light chain
			1.054	1.009	1.101	1.056	0.971	0.981	0.986	0.934	1.018	Q9NVK5	FGFR1OP2	FGFR1 oncogene partner 2
0.989	0.962	1.000	0.995	0.975	0.946	0.995	0.990	0.945	0.998	0.927	1.129	O95684	FGFR1OP	FGFR1 oncogene partner
0.964	1.026	1.015	0.994	0.955	0.987	1.035	0.970	1.053	0.972	0.961	1.119	Q9Y613	FHOD1	FH1/FH2 domain-containing protein 1
0.987	1.003	0.934	1.033	0.948	1.015	0.994	1.025	0.943	0.916	0.961	0.976	A0A087WUF6	FGF2	Fibroblast growth factor
			1.043	0.827	0.864							Q8WU20	FRS2	Fibroblast growth factor receptor substrate 2
1.039	0.964	1.019	1.072	1.014	1.006	1.065	1.193	0.919	1.167	0.940	1.279	P02751	FN1	Fibronectin
1.037	1.020	1.051	0.974	0.921	0.995	0.985	0.957	1.065	0.971	0.979	1.102	Q9BTV5	FSD1	Fibronectin type III and SPRY domain-containing protein 1
0.974	0.997	1.048	1.004	0.905	0.997	1.002	1.123	1.032	0.966	0.956	0.930	Q53EP0	FNDC3B	Fibronectin type III domain-containing protein 3B
1.069	1.037	1.016	1.026	0.937	1.112	1.059	0.992	0.999	1.111	1.019	1.126	Q9Y2H6	FNDC3A	Fibronectin type-III domain-containing protein 3A
0.908	0.967	0.859	0.941	0.888	1.151	1.239	1.153	1.047	1.332	0.972	1.345	Q9HCM7	FBRSL1	Fibrosin-1-like protein
									0.912	1.562	1.274	O75636	FCN3	Ficolin-3
1.233	0.813	1.005				1.002	1.220	0.934				Q5HY92	FIGN	Fidgetin
0.909	0.975	0.875				0.982	0.951	1.053	1.271	0.889	0.849	Q5D862	FLG2	Filaggrin-2

Abundance Ratio														
1 d M1/C1	1 d M2/C2	1 d M3/C3	4 d M1/C1	4 d M2/C2	4 d M3/C3	7 d M1/C1	7 d M2/C2	7 d M3/C3	10 d M1/C1	10 d M2/C2	10 d M3/C3	Accession ID	Gene name	Protein Description
						0.991	0.674	0.902				Q4L180	FILIP1L	Filamin A-interacting protein 1-like
0.898	0.999	0.872	0.747	1.106	1.084	0.981	0.972	1.126	1.374	1.087	1.370	H0Y5F3	FLNA	Filamin-A (Fragment)
0.981	1.006	0.983	0.997	1.044	0.947	0.995	0.968	0.981	0.984	0.976	0.976	P21333	FLNA	Filamin-A
0.841	0.993	0.882	0.922	0.940	0.841	0.954	1.151	0.918	0.828	0.986	0.978	O75369	FLNB	Filamin-B
0.990	1.018	0.968	0.984	1.021	0.916	1.046	0.964	0.919	0.993	0.967	1.017	Q8WUP2	FBLIM1	Filamin-binding LIM protein 1
1.107	1.114	1.193	1.081	0.881	0.959				0.922	0.982	1.056	Q9UIM3	FKBPL	FK506-binding protein-like
0.994	0.995	1.004	0.962	0.948	0.958	0.967	1.026	0.973	0.942	0.970	1.016	P39748	FEN1	Flap endonuclease 1
0.970	0.982	0.998	0.999	0.963	0.991	0.998	1.059	1.043	0.994	1.004	1.031	P30043	BLVRB	Flavin reductase (NADPH)
0.986	0.966	0.989	1.019	0.942	1.021	1.123	0.978	1.084	1.088	1.053	1.252	O75955	FLOT1	Flotillin-1
0.999	1.000	1.002	1.024	0.923	1.028	1.099	1.052	1.106	1.093	1.089	1.080	E7EMK3	FLOT2	Flotillin-2
1.032	1.029	0.988	0.991	0.939	0.979	0.974	0.942	0.922	0.967	1.022	0.985	Q96CP2	FLYWCH2	FLYWCH family member 2
1.016	1.042	1.018	1.064	1.000	0.986	0.989	1.148	1.010	1.012	1.045	1.002	Q5VW36	FOCAD	Focadhesin
1.009	1.000	1.006	1.008	1.021	0.995	1.022	1.003	0.990	1.007	1.010	0.987	E7ESA6	PTK2	Focal adhesion kinase 1
			0.799	1.524	1.623	1.253	1.081	1.206				P41440	SLC19A1	Folate transporter 1
0.925	0.976	1.069	1.007	0.944	1.043	0.980	1.085	1.020	0.982	0.995	0.955	Q8NFG4	FLCN	Folliculin
						0.767	1.279	1.240				Q8TF40	FNIP1	Folliculin-interacting protein 1
									1.131	0.996	1.089	O95633	FSTL3	Follistatin-related protein 3
1.044	1.007	1.008	0.954	0.901	1.057	1.005	1.100	1.026	0.987	1.028	1.015	Q05932	FPGS	Folylpolyglutamate synthase, mitochondrial
0.986	0.979	0.946	1.011	1.187	1.077	1.064	0.907	1.143	1.096	1.063	0.900	Q99958	FOXC2	Forkhead box protein C2
0.789	1.096	0.970	1.002	1.089	1.244	0.988	1.280	1.071	1.194	1.519	1.547	O75593	FOXH1	Forkhead box protein H1
1.012	0.903	0.975	0.998	1.621	1.079	1.049	0.949	1.079	1.184	1.003	1.181	Q9UPW0	FOXJ3	Forkhead box protein J3
1.002	1.019	1.011	1.009	0.980	1.011	0.981	0.990	1.008	0.989	1.024	1.048	P85037	FOXK1	Forkhead box protein K1
1.048	0.960	1.040	0.974	0.926	0.956	1.011	1.076	1.000	0.991	1.097	1.040	Q01167	FOXK2	Forkhead box protein K2
1.056	1.036	0.905							0.932	1.035	1.250	O43524	FOXO3	Forkhead box protein O3
1.033	1.009	0.908										A0A087X299	FOXP1	Forkhead box protein P1
			1.121	0.960	1.233							Q8IVH2	FOXP4	Forkhead box protein P4
0.997	0.969	1.027	0.967	0.944	0.977	1.022	0.992	0.964	1.070	0.934	1.080	Q96RU3	FNBP1	Formin-binding protein 1
0.974	0.981	1.000	1.016	0.957	1.054	1.047	0.969	0.976	1.042	1.011	1.060	Q8IVF7	FMNL3	Formin-like protein 3
						1.091	1.073	0.929	0.987	0.964	0.995	P15407	FOSL1	Fos-related antigen 1
0.973	0.967	1.008	0.919	0.944	0.955	0.954	0.919	0.931	0.877	1.097	1.025	P15408	FOSL2	Fos-related antigen 2
1.076	1.050	1.010	1.027	1.125	0.911	1.161	0.821	0.983	1.044	0.936	1.030	A0A0A0MSG2	FHL2	Four and a half LIM domains protein 2
1.160	1.096	1.039	1.036	0.984	0.992	0.988	0.994	0.950	1.043	0.925	1.031	Q13643	FHL3	Four and a half LIM domains protein 3
1.022	0.787	0.945							0.920	1.105	1.032	Q86VR8	FJX1	Four-jointed box protein 1

Abundance Ratio														
1 d M1/C1	1 d M2/C2	1 d M3/C3	4 d M1/C1	4 d M2/C2	4 d M3/C3	7 d M1/C1	7 d M2/C2	7 d M3/C3	10 d M1/C1	10 d M2/C2	10 d M3/C3	Accession ID	Gene name	Protein Description
1.019	1.002	0.996	1.003	0.975	1.002	1.003	1.004	1.006	1.014	1.032	1.019	P51114	FXR1	Fragile X mental retardation syndrome-related protein 1
0.987	1.005	1.013	0.990	0.966	0.968	0.979	0.979	0.968	1.071	1.056	1.081	P51116	FXR2	Fragile X mental retardation syndrome-related protein 2
1.036	0.982	0.990	1.120	0.986	1.007	1.055	1.028	0.954	1.175	0.983	1.030	Q16595	FXN	Frataxin, mitochondrial
0.875	0.927	0.786	1.050	1.041	1.047	1.232	1.110	1.042	1.311	1.038	1.105	F5H450	FZD10	Frizzled-10
0.943	0.974	1.101	0.857	0.924	1.153	1.196	0.924	0.997	1.114	1.116	1.162	O60353	FZD6	Frizzled-6
1.002	0.989	1.069	1.006	1.008	1.013	0.968	0.916	1.035	1.016	0.996	1.004	Q9H479	FN3K	Fructosamine-3-kinase
1.049	0.997	0.967	0.972	0.947	1.010	0.994	1.094	1.004	0.994	0.979	1.003	Q9NQ88	TIGAR	Fructose-2,6-bisphosphatase TIGAR
1.033	1.010	1.025	0.953	0.913	1.007	0.999	1.073	1.020	0.974	0.999	1.070	P09972	ALDOC	Fructose-bisphosphate aldolase C
1.055	1.204	1.183	0.955	0.972	1.092	1.053	0.967	1.050	0.941	0.932	1.095	A2VDF0	FUOM	Fucose mutarotase
1.031	0.985	1.028	0.959	0.983	1.003	1.044	1.083	1.032	0.983	1.005	0.938	A0A0A0MRP2	FPGT	Fucose-1-phosphate guanylyltransferase
0.925	1.068	0.984							1.063	0.990	0.924	O75072	FKTN	Fukutin
1.054	0.943	1.107	0.950	0.752	0.791	1.019	1.121	0.959	0.948	0.848	1.099	Q9H9S5	FKRP	Fukutin-related protein
0.973	1.003	0.994	0.984	0.971	0.993	0.992	1.033	0.981	1.008	1.010	1.003	P07954	FH	Fumarate hydratase, mitochondrial
1.042	1.040	0.966	1.006	1.025	1.011	1.012	1.067	1.046	0.978	0.980	0.979	P16930	FAH	Fumarylacetoacetase
1.018	0.998	0.994	1.014	1.138	0.982	1.009	0.976	1.049	1.077	0.976	0.982	Q96GK7	FAHD2A	Fumarylacetoacetate hydrolase domain-containing protein 2A
1.104	0.959	1.126	0.920	0.994	1.129	0.922	1.150	1.047	1.024	0.999	1.000	Q6P2I3	FAHD2B	Fumarylacetoacetate hydrolase domain-containing protein 2B
0.984	0.943	1.054	1.055	1.084	1.139	1.108	1.113	1.080	1.041	1.071	1.090	Q8IVP5	FUNDC1	FUN14 domain-containing protein 1
0.966	0.968	1.011	0.996	0.925	1.061	1.023	1.000	1.006	1.033	1.036	1.049	Q9BWH2	FUNDC2	FUN14 domain-containing protein 2
0.987	0.995	1.226				0.989	0.958	0.939				P98174	FGD1	FYVE, RhoGEF and PH domain-containing protein 1
0.940	1.091	1.027	1.029	0.947	1.022	1.125	1.030	1.028	1.078	0.991	1.042	F8VWL3	FGD4	FYVE, RhoGEF and PH domain-containing protein 4
1.036	1.036	1.036	1.006	0.926	0.999	1.022	1.130	1.011	0.981	0.966	1.080	Q6ZV73	FGD6	FYVE, RhoGEF and PH domain-containing protein 6
1.050	1.022	0.989	1.024	0.969	0.952	1.015	1.066	0.969	1.019	1.020	1.060	Q92917	GPKOW	G patch domain and KOW motifs-containing protein
1.070	1.007	1.083	1.010	0.930	1.088	0.989	0.863	0.949	0.999	0.929	1.156	Q9BRR8	GPATCH1	G patch domain-containing protein 1
1.075	0.963	1.030	0.860	0.999	0.909	0.972	1.185	1.078	0.959	1.044	0.983	A0A0A0MSF9	GPATCH11	G patch domain-containing protein 11
1.005	1.029	0.945				0.987	0.895	0.930				Q9NW75	GPATCH2	G patch domain-containing protein 2
1.026	1.007	1.031	1.014	0.980	0.962	0.963	0.988	0.977	0.995	1.010	1.052	Q9UKJ3	GPATCH8	G patch domain-containing protein 8
1.071	0.994	1.014	0.999	0.917	0.985	1.043	1.014	1.052	0.974	0.954	1.011	I3L3Y9	GPS2	G protein pathway suppressor 2 (Fragment)
0.945	0.979	1.013	1.010	0.978	1.045	1.031	0.937	0.978	1.081	1.033	1.156	Q7Z2K8	GPRIN1	G protein-regulated inducer of neurite outgrowth 1

Abundance Ratio														
1 d M1/C1	1 d M2/C2	1 d M3/C3	4 d M1/C1	4 d M2/C2	4 d M3/C3	7 d M1/C1	7 d M2/C2	7 d M3/C3	10 d M1/C1	10 d M2/C2	10 d M3/C3	Accession ID	Gene name	Protein Description
1.265	1.059	0.954	1.110	0.942	1.118	1.028	1.177	0.978	1.067	0.974	0.988	P24385	CCND1	G1/S-specific cyclin-D1
0.903	0.965	1.054				0.831	1.280	0.811	0.978	0.793	1.134	Q9NYZ3	GTSE1	G2 and S phase-expressed protein 1
0.973	1.043	1.039	1.001	0.991	0.975	0.987	1.136	1.005	0.860	0.822	1.068	P14635	CCNB1	G2/mitotic-specific cyclin-B1
0.876	1.034	1.112	1.044	0.805	0.898	0.970	1.329	1.045	0.707	1.148	0.724	O95067	CCNB2	G2/mitotic-specific cyclin-B2
0.999	1.002	1.008	1.049	1.018	1.001	1.046	1.034	0.928	1.035	1.017	0.982	Q06546	GABPA	GA-binding protein alpha chain
0.962	0.929	1.023	0.960	1.054	0.999	0.928	0.813	0.919	0.901	1.062	0.952	Q06547	GABPB1	GA-binding protein subunit beta-1
1.076	0.931	1.000	1.117	0.885	1.010	1.407	0.785	0.975	1.426	0.850	1.104	P07902	GALT	Galactose-1-phosphate uridylyltransferase
0.969	1.061	1.060	0.965	0.930	1.018	0.972	1.065	1.052	1.010	1.034	1.091	O94766	B3GAT3	Galactosylgalactosylxylosylprotein 3-beta-glucuronosyltransferase 3
1.023	1.028	0.983	1.007	1.047	0.992	1.009	0.989	1.010	1.011	1.031	1.021	P09382	LGALS1	Galectin-1
1.093	1.015	1.065	1.057	1.029	1.002	0.929	0.919	0.999	0.869	1.016	0.856	P17931	LGALS3	Galectin-3
0.974	1.090	1.032	1.010	0.932	1.025	1.194	0.993	1.066	1.048	0.971	1.218	Q08380	LGALS3BP	Galectin-3-binding protein
1.133	1.009	1.013	1.019	1.010	0.980	1.107	0.945	1.078	0.998	0.954	0.981	Q3ZCW2	LGALSL	Galectin-related protein
			0.980	0.902	0.980				1.006	1.094	1.114	Q9H3C7	GGNBP2	Gametogenetin-binding protein 2
0.978	1.008	0.980	1.001	0.995	1.009	1.067	0.954	0.955	1.132	1.016	1.078	Q9UEY8	ADD3	Gamma-adducin
1.059	1.038	0.999	1.048	1.014	0.953	1.017	1.023	0.967	1.052	1.083	0.957	O95166	GABARAP	Gamma-aminobutyric acid receptor-associated protein
1.003	0.997	0.935	1.048	0.969	1.016	1.050	1.108	1.035	1.065	1.044	1.071	P60520	GABARAPL2	Gamma-aminobutyric acid receptor-associated protein-like 2
1.120	1.008	1.074	1.002	1.005	0.971	1.001	0.983	0.999	0.956	0.933	0.982	P09104	ENO2	Gamma-enolase
1.056	1.005	1.001	1.001	0.944	0.936	0.964	1.018	0.962	0.993	1.008	0.954	Q92820	GGH	Gamma-glutamyl hydrolase
0.690	0.975	1.159				0.909	1.233	0.989	0.992	1.010	0.945	Q9BVM4	GGACT	Gamma-glutamylaminecyclotransferase
0.989	1.003	0.986	0.972	1.002	0.987	0.988	0.970	1.010	0.988	1.005	0.994	O75223	GGCT	Gamma-glutamylcyclotransferase
0.970	1.072	0.988	1.040	1.013	0.994	1.091	1.084	1.045	1.099	0.997	1.059	Q9UJ14	GGT7	Gamma-glutamyltransferase 7
1.134	1.041	1.083	0.967	0.994	1.088	1.084	1.071	1.043				P19440	GGT1	Gamma-glutamyltranspeptidase 1
0.976	1.084	0.979	0.947	0.931	0.973	1.048	0.651	0.964	1.100	0.937	1.080	P13284	IFI30	Gamma-interferon-inducible lysosomal thiol reductase
1.053	0.998	1.032	1.013	1.025	1.053	0.944	0.936	1.048	0.991	0.917	1.065	Q16666	IFI16	Gamma-interferon-inducible protein 16
1.085	0.947	1.126				1.214	0.896	1.022	1.548	1.084	1.102	Q96BI3	APH1A	Gamma-secretase subunit APH-1A
									1.094	0.954	0.947	Q8WW43	APH1B	Gamma-secretase subunit APH-1B
			0.841	0.637	1.410	0.976	0.988	1.390				Q9NZ42	PSENEN	Gamma-secretase subunit PEN-2
0.965	1.004	1.018	1.049	0.991	1.020	1.023	1.006	1.009	1.017	0.986	1.031	Q99747	NAPG	Gamma-soluble NSF attachment protein
1.003	1.014	1.019	0.992	1.009	1.051	1.022	1.018	1.005	1.034	1.024	1.077	Q9NUQ3	TXLNG	Gamma-taxilin
0.992	0.980	1.009	1.026	0.962	1.016	1.040	1.109	1.028	0.970	0.964	0.989	Q96CW5	TUBGCP3	Gamma-tubulin complex component 3
1.080	0.978	1.002	1.028	0.946	0.959	0.965	1.132	1.097	0.863	1.052	0.905	Q9UGJ1	TUBGCP4	Gamma-tubulin complex component 4

Abundance Ratio														
1 d M1/C1	1 d M2/C2	1 d M3/C3	4 d M1/C1	4 d M2/C2	4 d M3/C3	7 d M1/C1	7 d M2/C2	7 d M3/C3	10 d M1/C1	10 d M2/C2	10 d M3/C3	Accession ID	Gene name	Protein Description
1.181	1.134	0.900	0.948	0.859	1.019	0.946	0.974	1.068	0.837	0.797	1.088	Q96RT7	TUBGCP6	Gamma-tubulin complex component 6
			1.049	0.905	1.288							A0A087X1Z1	TUBGCP5	Gamma-tubulin complex component
1.005	0.980	0.959	0.982	0.951	0.988	0.997	1.000	0.948	1.032	0.996	0.998	P17900	GM2A	Ganglioside GM2 activator
0.984	0.998	1.072	0.998	0.915	1.079	1.060	0.962	0.974	0.987	0.999	1.008	Q8TB36	GDAP1	Ganglioside-induced differentiation-associated protein 1
1.076	0.979	1.018	0.989	0.943	1.049	1.065	1.074	0.979	1.044	1.000	1.044	Q9NXN4	GDAP2	Ganglioside-induced differentiation-associated protein 2
			0.976	0.933	1.023							P17302	GJA1	Gap junction alpha-1 protein
			0.947	0.891	1.265	1.178	1.546	0.902				P36383	GJC1	Gap junction gamma-1 protein
0.931	1.061	1.162	1.001	1.003	1.116	1.060	0.999	0.901	1.019	1.101	1.034	A0A5E8	GAS2L1	GAS2-like protein 1
1.092	0.989	1.059	0.972	0.903	0.971	1.002	1.121	1.004	1.107	0.985	1.130	G3V1A6	GSDMD	Gasdermin domain containing 1, isoform CRA_d
1.058	1.084	1.096	0.955	0.947	1.005	0.825	1.138	1.061	0.916	0.998	1.040	Q8WUU5	GATAD1	GATA zinc finger domain-containing protein 1
0.936	0.964	1.032	0.962	0.984	1.067	1.060	1.024	0.884	1.046	0.983	0.984	D4PHA4	GPBP1	GC-rich promoter binding protein 1, isoform CRA_c
1.004	1.017	0.998	0.960	0.955	0.982	1.036	1.046	1.015	0.986	0.987	1.054	P16383	GCFC2	GC-rich sequence DNA-binding factor 2
1.046	0.987	1.003	1.045	0.950	1.041	1.038	1.082	0.973	1.001	0.990	1.016	R4GMU1	H6PD	GDH/6PGL endoplasmic bifunctional protein
1.030	0.994	1.045	1.073	0.929	0.999	1.017	0.977	1.007	0.925	1.144	0.897	Q6ZNW5	GDPGP1	GDP-D-glucose phosphorylase 1
0.984	1.009	0.986	1.001	0.983	0.998	0.994	1.060	0.976	0.948	1.007	0.958	Q9H488	POFUT1	GDP-fucose protein O-fucosyltransferase 1
1.001	0.996	1.008	0.983	0.940	1.008	0.974	1.021	1.011	0.970	0.955	1.006	Q9Y2G5	POFUT2	GDP-fucose protein O-fucosyltransferase 2
1.020	1.008	0.992	0.997	1.013	0.991	1.019	1.059	1.000	0.999	0.994	1.017	Q13630	TSTA3	GDP-L-fucose synthase
1.038	0.992	1.045	0.996	0.906	0.985	1.013	0.948	0.967	1.001	0.924	1.028	Q2TAA5	ALG11	GDP-Man:Man(3)GlcNAc(2)-PP-Dol alpha-1,2-mannosyltransferase
0.966	0.995	1.001	1.026	1.070	1.013	1.038	1.013	0.997	1.009	0.988	1.011	O60547	GMDS	GDP-mannose 4,6 dehydratase
0.994	0.982	0.996	0.991	0.991	0.964	0.983	0.902	0.974	1.026	0.930	0.996	P06396	GSN	Gelsolin
0.897	1.059	0.996	0.949	0.853	1.083	1.038	0.989	1.000	1.147	1.001	1.090	O14893	GEMIN2	Gem-associated protein 2
1.005	1.003	0.989	0.991	1.006	1.053	1.005	1.069	1.019	0.976	0.972	1.018	P57678	GEMIN4	Gem-associated protein 4
0.980	0.989	1.012	0.990	0.983	1.019	0.987	1.028	1.043	1.024	1.008	1.016	Q8TEQ6	GEMIN5	Gem-associated protein 5
1.058	1.019	0.940	0.967	0.947	1.047	0.952	0.836	1.014	1.048	0.980	1.112	Q8WXD5	GEMIN6	Gem-associated protein 6
									1.105	0.905	1.120	Q9H840	GEMIN7	Gem-associated protein 7
1.022	0.939	0.872	0.962	1.007	1.024	0.968	1.031	0.995	1.085	1.048	1.195	Q9NWZ8	GEMIN8	Gem-associated protein 8
0.876	1.052	0.969										O75496	GMNN	Geminin
1.054	0.994	0.979	0.974	0.956	1.066	0.998	1.077	0.945	1.031	0.946	1.058	Q9P107	GMIP	GEM-interacting protein
1.020	1.022	1.021	1.015	0.991	1.026	1.033	1.064	1.036	1.002	0.999	1.041	Q12789	GTF3C1	General transcription factor 3C polypeptide 1
1.063	0.972	1.003	0.950	0.977	0.977	1.034	0.986	1.054	0.967	1.019	0.952	Q8WUA4	GTF3C2	General transcription factor 3C polypeptide 2
1.018	0.994	1.034	1.032	0.937	1.018	1.035	1.022	1.006	1.005	1.048	1.021	Q9Y5Q9	GTF3C3	General transcription factor 3C polypeptide 3

Abundance Ratio														
1 d M1/C1	1 d M2/C2	1 d M3/C3	4 d M1/C1	4 d M2/C2	4 d M3/C3	7 d M1/C1	7 d M2/C2	7 d M3/C3	10 d M1/C1	10 d M2/C2	10 d M3/C3	Accession ID	Gene name	Protein Description
0.987	1.014	0.992	1.008	0.966	1.011	1.032	1.094	1.016	0.972	0.973	1.043	Q9UKN8	GTF3C4	General transcription factor 3C polypeptide 4
0.955	0.944	0.938	0.968	0.962	1.063	1.013	1.059	1.065	1.058	1.020	1.043	Q969F1	GTF3C6	General transcription factor 3C polypeptide 6
1.010	1.001	0.998	0.967	0.969	1.064	1.065	1.078	1.009	0.990	1.016	1.024	P29083	GTF2E1	General transcription factor IIE subunit 1
0.979	1.003	1.026	0.954	0.964	0.994	0.956	0.967	0.987	1.034	1.010	1.070	P35269	GTF2F1	General transcription factor IIF subunit 1
1.045	1.026	1.001	0.986	0.993	1.023	1.017	1.039	0.993	1.043	1.008	1.008	P13984	GTF2F2	General transcription factor IIF subunit 2
0.985	0.997	1.004	1.016	0.996	1.048	1.069	1.089	0.969	1.065	0.951	1.085	P32780	GTF2H1	General transcription factor IIH subunit 1
0.993	0.997	1.085	0.971	0.950	1.056	0.990	0.937	1.002	0.975	0.981	1.051	Q13888	GTF2H2	General transcription factor IIH subunit 2
1.001	0.977	1.033	0.994	0.912	1.032	1.055	1.096	1.024	0.982	1.038	0.976	Q13889	GTF2H3	General transcription factor IIH subunit 3
1.057	0.991	1.042	1.065	0.945	1.102	1.020	1.061	1.055	0.897	0.921	1.006	Q92759	GTF2H4	General transcription factor IIH subunit 4
0.954	0.954	0.990	1.064	0.931	1.078	0.991	0.989	1.012	0.973	1.039	0.992	Q6ZYL4	GTF2H5	General transcription factor IIH subunit 5
0.986	0.996	0.980	0.989	0.979	0.981	0.998	1.016	1.001	0.975	0.977	0.995	P78347	GTF2I	General transcription factor II-I
0.989	0.998	1.025	0.981	0.948	1.017	0.963	1.010	0.990	0.986	0.999	0.996	O60763	USO1	General vesicular transport factor p115
0.936	1.011	1.198	0.964	1.148	1.183	0.933	0.946	1.151				Q14687	GSE1	Genetic suppressor element 1
1.000	1.034	0.998	1.043	0.971	0.970	1.002	1.072	1.006	1.033	0.984	1.053	F5H039	GPHN	Gephyrin
0.995	0.996	1.006	1.014	1.014	1.006	0.999	0.995	1.012	1.030	0.988	1.033	O95749	GGPS1	Geranylgeranyl pyrophosphate synthase
1.013	1.017	1.005	1.014	1.030	0.983	1.016	1.071	0.973	1.054	0.979	1.040	P53609	PGGT1B	Geranylgeranyl transferase type-1 subunit beta
0.999	1.002	1.038	0.982	0.979	1.069	0.980	1.046	1.050	1.007	1.002	1.028	Q92696	RABGGTA	Geranylgeranyl transferase type-2 subunit alpha
0.975	0.966	0.978	0.978	1.006	1.002	0.961	1.016	1.028	0.968	0.986	1.003	P53611	RABGGTB	Geranylgeranyl transferase type-2 subunit beta
1.100	1.002	1.026	1.006	0.960	0.997	1.002	1.064	0.999	0.955	1.014	1.027	O60318	MCM3AP	Germinal-center associated nuclear protein
0.896	0.893	1.055	0.980	0.962	1.009	0.977	1.008	1.080	1.072	1.010	0.978	Q8N2G8	GHDC	GH3 domain-containing protein
0.925	0.929	1.080	1.002	1.033	1.025	0.933	0.942	0.994	0.990	1.027	0.980	Q9H2C0	GAN	Gigaxonin
0.992	0.978	1.016	0.998	0.970	1.016	1.035	0.967	0.987	1.066	0.975	1.067	Q3V6T2	CCDC88A	Girdin
1.005	1.005	1.029	0.992	0.956	0.963	0.935	0.978	0.955	0.939	0.977	0.916	P60983	GMFB	Glia maturation factor beta
1.142	0.992	1.088	0.977	0.756	1.107	1.055	0.959	0.904	1.066	1.043	1.053	Q9NZM4	GLTSCR1	Glioma tumor suppressor candidate region gene 1 protein
1.040	1.011	1.059	1.109	1.018	1.098	1.074	0.924	1.014	1.144	1.040	1.088	Q9NZM5	GLTSCR2	Glioma tumor suppressor candidate region gene 2 protein
1.013	0.967	1.011	0.975	0.889	1.053	0.984	1.073	1.042	0.934	1.012	1.052	Q92990	GLMN	Glomulin
1.053	1.007	1.012	0.956	1.024	1.067	1.054	1.024	1.035	1.016	1.047	1.038	Q9Y692	GMEB1	Glucocorticoid modulatory element-binding protein 1
1.022	1.059	1.016	1.016	0.996	0.998	0.972	1.259	0.970	1.136	1.116	1.014	Q9UKD1	GMEB2	Glucocorticoid modulatory element-binding protein 2
0.963	1.035	1.009	0.986	1.220	1.010	1.014	1.012	1.014	0.959	1.016	0.992	Q96EK6	GNPNAT1	Glucosamine 6-phosphate N-acetyltransferase
1.017	1.001	0.998	1.010	0.997	1.034	0.989	1.080	1.026	0.946	0.993	0.933	P46926	GNPDA1	Glucosamine-6-phosphate isomerase 1
0.947	0.982	0.975	0.962	0.967	1.019	1.013	1.030	0.993	0.996	1.004	1.003	Q8TDQ7	GNPDA2	Glucosamine-6-phosphate isomerase 2

Abundance Ratio												Accession ID	Gene name	Protein Description
1 d M1/C1	1 d M2/C2	1 d M3/C3	4 d M1/C1	4 d M2/C2	4 d M3/C3	7 d M1/C1	7 d M2/C2	7 d M3/C3	10 d M1/C1	10 d M2/C2	10 d M3/C3			
1.004	1.035	0.987	0.968	0.956	0.973	0.955	0.999	0.995	0.971	0.956	0.985	Q6PCE3	PGM2L1	Glucose 1,6-bisphosphate synthase
1.026	1.013	1.017	0.970	1.013	0.972	0.913	0.981	0.971	0.905	0.998	0.925	A0A0A0MTS2	GPI	Glucose-6-phosphate isomerase (Fragment)
1.080	0.935	1.076	0.912	1.031	0.967	1.114	0.951	0.983	0.959	1.048	0.937	Q3B7J2	GFOD2	Glucose-fructose oxidoreductase domain-containing protein 2
1.055	1.097	0.958	0.996	1.024	0.976	1.004	1.189	0.985	1.033	1.019	1.022	Q8IVV7	GID4	Glucose-induced degradation protein 4 homolog
0.975	0.949	0.999	0.989	1.010	1.068	0.975	1.085	1.124	0.973	0.967	1.021	Q9NWU2	GID8	Glucose-induced degradation protein 8 homolog
1.034	0.997	0.997	0.985	0.932	0.991	0.943	0.962	0.959	0.960	0.967	0.942	K7ELL7	PRKCSH	Glucosidase 2 subunit beta
1.033	0.933	0.933	0.984	0.974	1.086	0.900	1.002	1.020	1.039	1.066	1.036	Q4G148	GXYLT1	Glucoside xylosyltransferase 1
0.992	0.950	0.951	0.812	0.802	0.831	0.845	0.877	0.861	0.903	0.864	0.866	P04062	GBA	Glucosylceramidase
0.981	1.001	0.978	1.024	1.025	0.996	1.003	1.003	0.991	0.985	0.999	0.956	P00367	GLUD1	Glutamate dehydrogenase 1, mitochondrial
1.074	0.741	1.679										P49448	GLUD2	Glutamate dehydrogenase 2, mitochondrial
1.015	1.020	1.022	0.993	0.970	1.019	0.973	1.086	0.993	1.010	1.025	1.007	P48506	GCLC	Glutamate--cysteine ligase catalytic subunit
1.013	1.005	1.001	1.022	0.967	1.002	1.013	1.049	1.053	1.028	0.978	1.035	P48507	GCLM	Glutamate--cysteine ligase regulatory subunit
0.894	1.127	1.043	0.865	0.985	1.125	1.116	1.055	1.087				Q86X53	ERICH1	Glutamate-rich protein 1
0.974	0.966	0.978	1.001	1.032	1.029	1.021	1.034	1.084	1.023	0.994	1.030	Q9BQ67	GRWD1	Glutamate-rich WD repeat-containing protein 1
0.951	1.009	0.962	0.937	0.973	0.919	0.976	0.843	0.944	0.983	0.801	0.998	O94925	GLS	Glutaminase kidney isoform, mitochondrial
0.947	0.938	1.052	0.966	0.899	1.033	1.130	1.058	1.055	0.965	0.914	1.051	Q2KHR3	QSER1	Glutamine and serine-rich protein 1
1.081	1.035	1.057	1.087	1.112	1.090	0.978	0.961	0.998	1.044	1.065	1.065	P15104	GLUL	Glutamine synthetase
1.181	1.048	1.084	1.035	1.046	1.046	1.010	1.054	0.973	1.019	0.995	1.032	Q6IA69	NADSYN1	Glutamine-dependent NAD(+) synthetase
0.982	0.995	1.013	1.013	1.002	1.014	0.988	1.044	1.020	0.974	1.002	1.005	Q06210	GFPT1	Glutamine--fructose-6-phosphate aminotransferase [isomerizing] 1
1.012	1.038	1.059	0.993	0.997	1.013	0.993	0.976	1.002	0.984	0.885	1.042	O94808	GFPT2	Glutamine--fructose-6-phosphate aminotransferase [isomerizing] 2
0.992	1.021	0.974	1.022	1.032	1.011	1.027	1.055	1.014	0.984	0.977	1.004	Q2TAL8	QRICH1	Glutamine-rich protein 1
1.305	0.855	0.940	1.658	0.667	0.994	1.604	0.668	0.937	1.433	0.911	0.961	A0A1B0GVU9	QARS	Glutamine--tRNA ligase (Fragment)
1.000	1.013	1.017	0.997	0.991	0.980	1.002	1.032	0.986	0.996	0.992	1.007	P47897	QARS	Glutamine--tRNA ligase
1.004	0.962	1.061	0.872	0.860	0.988	0.982	1.078	1.464	0.993	0.928	1.035	Q9NXS2	QPCTL	Glutaminyl-peptide cyclotransferase-like protein
0.997	1.001	1.015	0.990	0.999	1.029	1.030	1.031	0.993	1.065	0.990	1.035	Q9H0R6	QRSL1	Glutamyl-tRNA(Gln) amidotransferase subunit A, mitochondrial
0.999	1.006	1.023	1.026	0.964	1.000	1.026	0.958	0.984	1.015	0.983	1.087	O75879	GATB	Glutamyl-tRNA(Gln) amidotransferase subunit B, mitochondrial
0.944	1.001	0.963	1.036	0.934	1.023	0.999	1.083	1.095	1.009	0.930	0.987	P35754	GLRX	Glutaredoxin-1
1.005	1.007	1.001	0.996	0.986	0.982	0.978	1.087	1.019	0.923	0.960	0.969	O76003	GLRX3	Glutaredoxin-3
			1.017	1.713	1.422	0.806	1.255	1.056				A6NC05	C5orf63	Glutaredoxin-like protein C5orf63
0.991	0.974	0.909	1.054	0.940	0.974	1.008	1.019	1.038	1.063	1.006	1.052	Q86SX6	GLRX5	Glutaredoxin-related protein 5, mitochondrial
0.981	0.984	1.040	1.024	1.128	1.017	0.962	0.966	1.033	0.973	1.056	1.021	Q92947	GCDH	Glutaryl-CoA dehydrogenase, mitochondrial

Abundance Ratio														
1 d M1/C1	1 d M2/C2	1 d M3/C3	4 d M1/C1	4 d M2/C2	4 d M3/C3	7 d M1/C1	7 d M2/C2	7 d M3/C3	10 d M1/C1	10 d M2/C2	10 d M3/C3	Accession ID	Gene name	Protein Description
1.045	0.999	0.996	0.983	1.022	0.978	0.973	1.008	1.027	0.949	0.955	0.948	P07203	GPX1	Glutathione peroxidase 1
1.088	0.988	0.963	1.007	1.036	0.968	1.031	0.965	0.992	0.970	1.016	0.915	A0A087WT12	GPX4	Glutathione peroxidase
0.989	0.999	0.965	1.024	0.974	0.996	0.995	0.930	0.999	1.037	0.976	1.020	P00390	GSR	Glutathione reductase, mitochondrial
0.991	1.004	1.042	0.951	1.005	0.986	1.011	0.979	1.027	1.086	0.939	1.075	Q8NEC7	GSTCD	Glutathione S-transferase C-terminal domain-containing protein
0.931	1.014	0.956	1.007	1.020	1.024	1.024	1.039	1.012	1.037	0.997	1.021	Q9Y2Q3	GSTK1	Glutathione S-transferase kappa 1
1.046	0.961	0.933	1.180	0.984	1.194	0.957	1.074	0.973	0.943	0.930	1.080	P21266	GSTM3	Glutathione S-transferase Mu 3
0.982	1.006	0.983	1.012	1.014	1.034	0.995	1.073	1.012	0.959	0.999	0.981	P78417	GSTO1	Glutathione S-transferase omega-1
1.077	1.017	0.969	1.026	1.005	0.971	1.012	1.064	0.984	0.972	1.006	0.914	P09211	GSTP1	Glutathione S-transferase P
1.053	0.985	1.040	0.982	0.887	0.967	1.031	1.074	1.003	1.001	0.982	0.998	P0CG30	GSTT2B	Glutathione S-transferase theta-2B
1.001	0.989	0.998	0.994	1.034	1.003	0.967	0.983	0.996	0.994	1.004	0.981	P48637	GSS	Glutathione synthetase
0.965	1.007	0.950	0.994	1.053	0.990	0.950	0.983	0.999	0.935	1.004	0.938	P04406	GAPDH	Glyceraldehyde-3-phosphate dehydrogenase
1.021	0.956	1.050	1.030	0.947	1.008	1.029	1.058	0.999	1.009	1.014	1.014	P32189	GK	Glycerol kinase
0.986	0.976	1.079	1.078	0.968	1.083	0.914	1.076	1.021	1.016	0.927	1.074	Q9HCL2	GPAM	Glycerol-3-phosphate acyltransferase 1, mitochondrial
0.959	0.906	1.018	1.046	0.888	1.144	0.868	1.049	1.055	0.882	0.969	0.943	Q53EU6	GPAT3	Glycerol-3-phosphate acyltransferase 3
0.978	0.920	1.026	0.928	0.923	0.950	1.070	1.083	1.025	1.054	0.988	1.064	Q86UL3	GPAT4	Glycerol-3-phosphate acyltransferase 4
1.004	0.998	0.990	1.008	1.048	1.018	1.054	0.975	1.044	1.004	0.954	1.050	Q8N335	GPD1L	Glycerol-3-phosphate dehydrogenase 1-like protein
0.988	1.001	1.004	1.007	1.003	1.013	0.987	1.038	0.997	1.012	0.997	0.981	P43304	GPD2	Glycerol-3-phosphate dehydrogenase, mitochondrial
0.977	0.997	0.996	0.978	0.998	0.991	1.015	1.013	1.007	1.043	1.005	1.054	A6NDG6	PGP	Glycerol-3-phosphate phosphatase
1.092	1.021	1.029	1.128	0.952	0.994	1.047	1.249	0.992	1.047	1.025	1.074	Q9NPB8	GPCPD1	Glycerophosphocholine phosphodiesterase GPCPD1
			0.997	1.013	0.960	1.140	1.038	1.016				Q9NZC3	GDE1	Glycerophosphodiester phosphodiesterase 1
			1.011	0.891	0.996	0.996	1.071	0.968	0.933	1.033	0.914	Q8N9F7	GDPD1	Glycerophosphodiester phosphodiesterase domain-containing protein 1
1.120	0.949	1.010	1.042	1.687	0.956	1.073	0.858	1.166	1.010	1.015	0.966	P23434	GCSH	Glycine cleavage system H protein, mitochondrial
0.986	1.009	0.995	0.990	0.994	0.994	0.974	1.011	0.989	0.990	0.993	0.998	P41250	GARS	Glycine--tRNA ligase
0.992	1.004	0.985	0.977	0.964	1.021	0.948	1.006	0.993	0.991	0.972	0.986	P13807	GYS1	Glycogen [starch] synthase, muscle
1.023	0.982	1.021	1.008	0.973	1.045	1.018	1.033	1.018	1.017	1.011	1.014	P35573	AGL	Glycogen debranching enzyme
0.986	0.995	0.986	1.000	1.002	0.996	0.981	0.983	1.005	0.970	0.962	0.971	P11216	PYGB	Glycogen phosphorylase, brain form
0.978	1.002	0.981	1.006	1.006	1.000	0.996	1.034	0.995	0.997	1.022	0.988	P06737	PYGL	Glycogen phosphorylase, liver form
0.863	1.017	0.902	0.844	0.881	0.844	0.820	1.106	0.987	0.984	0.990	0.896	P11217	PYGM	Glycogen phosphorylase, muscle form
1.072	1.026	1.000	1.012	0.974	0.924	0.992	1.096	1.001	0.997	0.993	0.966	P49840	GSK3A	Glycogen synthase kinase-3 alpha
1.042	1.013	1.014	0.982	0.944	0.972	0.971	1.107	0.981	0.976	1.030	0.921	P46976	GYG1	Glycogenin-1

Abundance Ratio												Accession ID	Gene name	Protein Description
1 d M1/C1	1 d M2/C2	1 d M3/C3	4 d M1/C1	4 d M2/C2	4 d M3/C3	7 d M1/C1	7 d M2/C2	7 d M3/C3	10 d M1/C1	10 d M2/C2	10 d M3/C3			
1.091	0.995	1.017	1.067	0.942	1.020	1.155	1.105	1.014	1.015	0.944	1.077	Q9NZD2	GLTP	Glycolipid transfer protein
1.057	1.065	1.022	0.994	1.023	0.984	0.987	1.214	1.032	0.784	0.852	0.959	Q5SRI9	MANEA	Glycoprotein endo-alpha-1,2-mannosidase
			0.911	1.002	1.051							Q5VSG8	MANEAL	Glycoprotein endo-alpha-1,2-mannosidase-like protein
0.954	1.029	1.044	0.989	0.956	1.039	1.053	1.069	0.972	1.050	1.054	1.018	Q9NS00	C1GALT1	Glycoprotein-N-acetylgalactosamine 3-beta-galactosyltransferase 1
1.059	0.996	0.983	0.965	0.925	1.009	0.982	1.002	1.031	0.997	0.992	1.015	O75063	FAM20B	Glycosaminoglycan xylosylkinase
0.993	1.037	1.001	1.037	0.910	1.015	1.041	1.087	1.000	1.090	1.040	0.966	O43292	GPAA1	Glycosylphosphatidylinositol anchor attachment 1 protein
1.114	1.166	1.147	1.261	1.179	1.259	1.207	1.289	1.150	1.158	1.084	1.188	Q68CQ7	GLT8D1	Glycosyltransferase 8 domain-containing protein 1
1.615	1.201	1.076	0.815	0.770	0.938							Q4AE62	GTDC1	Glycosyltransferase-like domain-containing protein 1
0.990	1.016	0.968	1.005	1.005	0.991	0.978	0.998	0.984	0.993	1.026	0.979	P30419	NMT1	Glycylpeptide N-tetradecanoyltransferase 1
0.993	0.990	0.944	0.983	0.950	0.988	0.976	1.014	0.943	1.024	1.048	0.956	O60551	NMT2	Glycylpeptide N-tetradecanoyltransferase 2
0.985	1.011	0.982	0.955	0.974	1.001	0.987	1.035	1.009	1.027	0.968	1.008	Q9UBQ7	GRHPR	Glyoxylate reductase/hydroxypyruvate reductase
			1.055	0.942	1.070	1.076	1.031	1.060	1.519	1.424	0.843	P35052	GPC1	Glypican-1
0.980	0.959	1.005	0.917	0.950	1.044	0.984	1.036	1.097	1.011	0.983	1.048	O75487	GPC4	Glypican-4
1.033	1.010	1.014	1.018	1.123	0.994	1.058	0.980	1.028	1.067	1.018	1.013	H0YNJ6	GMPR2	GMP reductase
0.994	0.985	0.992	1.004	1.001	1.003	1.027	1.004	1.015	0.983	0.964	1.015	P49915	GMPS	GMP synthase [glutamine-hydrolyzing]
0.859	0.932	1.630	0.982	0.912	1.248	1.057	1.077	1.321	1.143	0.988	1.068	Q96ET8	TVP23C	Golgi apparatus membrane protein TVP23 homolog C
0.969	0.986	1.012	0.960	0.904	1.006	1.013	0.907	0.963	1.016	0.961	1.103	O00461	GOLIM4	Golgi integral membrane protein 4
0.938	0.953	1.013	0.928	0.904	0.962	1.005	0.904	0.965	1.054	0.934	1.122	Q8NBJ4	GOLM1	Golgi membrane protein 1
0.893	0.973	1.008	0.941	0.860	1.061	0.889	1.132	1.162	1.073	0.987	1.055	P0CG08	GPR89B	Golgi pH regulator B
0.966	1.007	1.027	0.973	1.030	1.028	0.968	0.975	1.031	0.979	0.981	1.013	Q9H4A6	GOLPH3	Golgi phosphoprotein 3
0.988	0.907	1.115	0.967	0.936	0.875	0.947	1.173	1.101	0.886	0.720	0.975	Q9H4A5	GOLPH3L	Golgi phosphoprotein 3-like
0.899	1.103	0.951	0.945	0.953	1.009	1.094	1.146	1.025	1.035	1.024	1.044	Q9BQQ3	GORASP1	Golgi reassembly-stacking protein 1
1.015	1.013	0.994	1.054	0.998	1.000	1.094	0.993	0.988	1.074	0.948	1.021	Q9H8Y8	GORASP2	Golgi reassembly-stacking protein 2
0.989	1.018	0.993	1.014	0.990	1.006	0.976	1.069	0.987	0.977	0.998	0.951	Q9H3P7	ACBD3	Golgi resident protein GCP60
0.955	0.952	1.014	0.958	0.949	0.997	0.980	0.964	0.966	1.033	1.016	1.026	G5E9T8	GOSR1	Golgi SNAP receptor complex member 1 (Fragment)
1.029	1.033	0.885	1.161	0.829	1.026	1.157	0.987	1.127	1.054	0.959	1.057	E7EQ34	GOSR2	Golgi SNAP receptor complex member 2
0.994	0.954	1.030	0.913	0.914	1.054	0.903	0.826	1.032	0.995	0.979	1.099	Q7L5D6	GET4	Golgi to ER traffic protein 4 homolog
0.968	1.004	0.997	0.975	0.970	0.982	0.959	0.927	1.002	1.109	0.943	1.017	Q9HD26	GOPC	Golgi-associated PDZ and coiled-coil motif-containing protein

Abundance Ratio														
1 d M1/C1	1 d M2/C2	1 d M3/C3	4 d M1/C1	4 d M2/C2	4 d M3/C3	7 d M1/C1	7 d M2/C2	7 d M3/C3	10 d M1/C1	10 d M2/C2	10 d M3/C3	Accession ID	Gene name	Protein Description
0.969	0.940	1.095	1.085	0.997	1.027	0.983	0.943	0.973	1.041	1.230	1.130	Q9H4G4	GLIPR2	Golgi-associated plant pathogenesis-related protein 1
0.996	0.974	1.082	0.985	0.992	1.030	0.955	1.083	1.086	1.052	0.924	1.172	Q92805	GOLGA1	Golgin subfamily A member 1
0.972	0.972	1.042	0.981	0.967	1.019	0.989	0.923	0.988	1.054	1.012	1.081	Q08379	GOLGA2	Golgin subfamily A member 2
0.998	0.984	1.016	0.979	0.951	1.014	0.990	1.008	1.011	0.985	0.972	1.051	Q08378	GOLGA3	Golgin subfamily A member 3
1.040	0.893	1.035	1.009	0.960	1.055	1.027	1.000	1.022	1.031	1.004	1.093	Q13439	GOLGA4	Golgin subfamily A member 4
0.994	0.981	1.076	1.031	0.932	0.996	0.993	1.014	1.007	1.044	0.999	1.032	Q8TBA6	GOLGA5	Golgin subfamily A member 5
0.941	1.006	0.994	0.992	0.948	1.003	1.038	1.020	1.018	1.072	1.019	1.106	Q7Z5G4	GOLGA7	Golgin subfamily A member 7
						0.920	0.767	0.923	0.886	1.011	1.119	Q9H2G9	BLZF1	Golgin-45
0.961	1.025	1.016	0.996	0.936	1.039	1.014	0.983	1.027	1.012	1.014	1.071	Q92538	GBF1	Golgi-specific brefeldin A-resistance guanine nucleotide exchange factor 1
0.999	1.024	0.938	1.100	0.991	1.015	0.975	1.019	1.022	0.977	1.122	1.080	Q8IXQ4	GPALPP1	GPALPP motifs-containing protein 1
0.960	0.932	1.060	0.924	0.902	0.996	1.015	1.054	1.027	1.148	0.948	0.989	O95427	PIGN	GPI ethanolamine phosphate transferase 1
1.027	1.105	1.009	1.006	0.907	1.028	1.034	1.104	0.966	0.954	1.020	1.004	Q5H8A4	PIGG	GPI ethanolamine phosphate transferase 2
0.965	0.941	0.961	1.056	0.898	1.011	0.926	1.064	1.009	1.017	1.010	0.992	Q8TEQ8	PIGO	GPI ethanolamine phosphate transferase 3
						1.121	0.878	1.187				Q9H3S5	PIGM	GPI mannosyltransferase 1
1.012	1.022	1.020	1.020	0.907	1.042	0.958	1.086	0.997	1.000	0.983	0.971	Q96S52	PIGS	GPI transamidase component PIG-S
0.997	1.012	0.980	1.005	0.949	1.007	0.973	1.052	1.004	0.948	0.990	0.961	Q969N2	PIGT	GPI transamidase component PIG-T
0.996	0.992	1.017	0.968	0.959	1.005	0.952	1.014	1.021	0.971	1.041	1.024	Q92643	PIGK	GPI-anchor transamidase
1.033	1.046	0.834	1.036	0.881	1.094	1.074	1.044	0.954	0.951	0.961	0.988	Q9H9Y4	GPN2	GPN-loop GTPase 2
1.114	0.969	1.103				1.117	1.118	0.995	1.039	1.058	1.044	O43194	GPR39	G-protein coupled receptor 39
1.029	1.034	0.962	0.890	0.927	0.901	0.902	0.849	0.902	1.170	1.103	1.187	Q96D09	GPRASP2	G-protein coupled receptor-associated sorting protein 2
			1.050	0.978	1.089	1.078	1.030	1.007	1.030	1.024	1.079	A0A0C4DFY5	GPRC5C	G-protein-coupled receptor family C group 5 member C
1.040	1.027	1.035	0.962	0.953	1.002	0.972	1.052	0.973	1.003	0.995	1.079	A0A0A0MSK4	GPSM1	G-protein-signaling modulator 1
1.062	1.011	0.956	1.013	1.069	0.912	1.046	1.046	1.197	1.044	1.094	1.091	M0QZ12	GRAMD1A	GRAM domain-containing protein 1A
1.386	0.998	0.969	0.983	0.894	0.933	0.987	0.839	1.190	1.138	0.829	1.168	Q6IC98	GRAMD4	GRAM domain-containing protein 4
0.997	0.996	0.961	1.021	0.939	0.996	1.006	1.064	0.989	0.976	0.963	0.943	P28676	GCA	Grancalcin
1.032	1.042	1.019	0.963	0.958	1.031	1.041	1.055	0.993	1.232	1.118	1.240	P28799	GRN	Granulins
0.982	0.943	0.977	1.020	0.928	1.077	1.062	1.150	1.022	1.034	1.039	0.996	P16260	SLC25A16	Graves disease carrier protein
1.003	0.943	1.050	1.011	0.973	1.074	1.000	1.049	1.040	0.900	0.954	1.090	O75420	GIGYF1	GRB10-interacting GYF protein 1
0.945	0.999	1.047	0.986	1.005	1.044	0.990	0.918	1.009	1.058	1.042	1.119	Q6Y7W6	GIGYF2	GRB10-interacting GYF protein 2
0.976	1.008	0.998	1.022	1.102	1.001	1.055	0.966	0.993	1.077	1.002	1.067	Q12849	GRSF1	G-rich sequence factor 1
1.046	0.974	1.013	0.976	0.923	1.035	0.983	1.029	0.984	1.010	0.998	1.052	Q96CN9	GCC1	GRIP and coiled-coil domain-containing protein 1

Abundance Ratio														
1 d M1/C1	1 d M2/C2	1 d M3/C3	4 d M1/C1	4 d M2/C2	4 d M3/C3	7 d M1/C1	7 d M2/C2	7 d M3/C3	10 d M1/C1	10 d M2/C2	10 d M3/C3	Accession ID	Gene name	Protein Description
1.002	0.999	1.020	1.000	0.961	1.030	1.035	1.057	1.018	0.988	0.981	1.048	Q8IWJ2	GCC2	GRIP and coiled-coil domain-containing protein 2
1.010	1.002	1.025	1.009	0.983	1.017	1.007	0.991	0.993	1.053	0.992	1.086	Q4V328	GRIPAP1	GRIP1-associated protein 1
1.013	1.023	0.991	0.967	0.990	0.974	0.995	1.078	1.059	1.028	0.976	1.073	Q8NCC3	PLA2G15	Group XV phospholipase A2
1.008	0.975	1.017	0.958	0.942	1.013	0.984	1.027	1.023	1.014	0.969	1.054	Q8TAE8	GADD45GIP1	Growth arrest and DNA damage-inducible proteins-interacting protein 1
1.127	1.027	1.032	1.038	1.033	0.994	1.102	1.173	1.000	1.003	1.057	1.054	Q13322	GRB10	Growth factor receptor-bound protein 10
1.037	0.933	0.901	0.912	0.893	1.030	0.971	0.832	1.039				Q14449	GRB14	Growth factor receptor-bound protein 14
0.979	1.004	1.001	0.979	1.001	1.030	0.973	1.028	0.980	1.007	1.001	0.981	P62993	GRB2	Growth factor receptor-bound protein 2
0.946	0.988	1.013	0.978	0.874	1.024	1.016	1.037	0.942	1.042	0.996	0.951	Q9H3K2	GHITM	Growth hormone-inducible transmembrane protein
0.918	0.912	0.927	0.918	0.914	1.033	0.938	0.983	0.912	0.923	1.003	0.853	Q99988	GDF15	Growth/differentiation factor 15
1.150	1.135	1.148	1.008	0.950	1.145	1.074	1.046	0.828	0.985	0.877	0.956	P09341	CXCL1	Growth-regulated alpha protein
0.999	0.987	0.979	0.969	0.997	0.953	0.978	0.949	0.956	0.983	0.976	1.022	Q9HAV7	GRPEL1	GrpE protein homolog 1, mitochondrial
0.977	1.148	1.163							0.813	0.815	0.933	Q8TAA5	GRPEL2	GrpE protein homolog 2, mitochondrial
1.029	0.916	0.927	0.952	0.933	0.837	1.129	1.042	0.975	1.095	0.963	1.096	Q9P0R6	GSKIP	GSK3-beta interaction protein
0.951	1.017	0.958	1.060	1.003	0.954	1.010	0.966	1.043	1.066	0.987	1.081	P30047	GCHFR	GTP cyclohydrolase 1 feedback regulatory protein
						1.044	1.291	0.903				P30793	GCH1	GTP cyclohydrolase 1
1.021	0.997	0.993	0.996	0.945	1.009	0.997	1.027	0.994	1.032	0.990	1.013	Q9UIJ7	AK3	GTP:AMP phosphotransferase AK3, mitochondrial
1.006	1.009	0.986	0.981	0.945	0.961	0.997	1.008	0.981	1.012	0.959	1.003	O75616	ERAL1	GTPase Era, mitochondrial
1.032	0.993	1.015	1.014	1.035	0.970	1.046	1.002	0.913	1.081	1.094	0.997	P01112	HRAS	GTPase HRas
0.957	0.970	0.998	1.063	0.901	1.029	1.055	0.933	1.022	1.077	0.976	1.039	P01111	NRAS	GTPase NRas
1.067	1.003	0.994	1.025	1.042	0.975	1.007	1.019	0.996	0.999	0.986	0.952	J3KQE5	RAN	GTP-binding nuclear protein Ran (Fragment)
0.974	1.017	1.021	0.984	0.955	1.033	0.998	1.065	1.017	0.982	0.974	1.023	O00178	GTPBP1	GTP-binding protein 1
1.036	1.003	0.978	0.989	0.952	0.963	1.005	1.056	0.977	1.059	0.965	1.035	A4D1E9	GTPBP10	GTP-binding protein 10
0.898	0.929	0.787	0.849	1.034	1.023	0.983	1.146	1.065	0.884	1.050	0.959	Q9BX10	GTPBP2	GTP-binding protein 2
			1.009	0.868	1.072							A0A087WUP3	GTPBP8	GTP-binding protein 8
						0.857	0.888	0.922	0.952	0.712	0.912	P55042	RRAD	GTP-binding protein RAD
0.995	0.972	0.998	1.016	0.995	1.035	1.015	1.048	1.014	0.944	0.980	1.020	Q15382	RHEB	GTP-binding protein Rheb
1.020	0.995	0.986	1.013	0.864	1.051	1.046	1.130	0.930	1.045	1.001	0.970	Q9NR31	SAR1A	GTP-binding protein SAR1a
1.123	1.033	1.054	1.100	0.924	0.997	1.056	1.014	0.954	0.941	1.034	1.011	Q9Y6B6	SAR1B	GTP-binding protein SAR1b
1.031	1.083	1.005	0.965	1.031	0.930	1.001	1.107	0.973	0.948	1.082	0.978	Q14353	GAMT	Guanidinoacetate N-methyltransferase
			0.888	0.930	1.176	0.820	0.745	0.900	1.152	1.139	1.113	Q96LT7	C9orf72	Guanine nucleotide exchange C9orf72
0.986	0.915	0.996	1.044	0.999	1.017	1.055	1.020	1.060	1.050	1.049	1.023	Q8TBN0	RAB3IL1	Guanine nucleotide exchange factor for Rab-3A
0.973	1.031	0.957	0.807	0.760	0.717	0.885	1.100	0.923	1.020	0.974	1.015	P47224	RABIF	Guanine nucleotide exchange factor MSS4
0.940	1.101	1.063	1.008	1.040	1.048	0.935	0.961	1.012	1.025	1.005	0.991	Q8TEV9	SMCR8	Guanine nucleotide exchange protein SMCR8

Abundance Ratio														
1 d M1/C1	1 d M2/C2	1 d M3/C3	4 d M1/C1	4 d M2/C2	4 d M3/C3	7 d M1/C1	7 d M2/C2	7 d M3/C3	10 d M1/C1	10 d M2/C2	10 d M3/C3	Accession ID	Gene name	Protein Description
0.962	1.017	0.983	1.046	0.895	0.922	1.016	1.093	1.055	1.091	1.171	1.059	P63096	GNAI1	Guanine nucleotide-binding protein G(i) subunit alpha-1
0.947	0.999	1.018	0.979	0.946	0.990	0.999	1.036	1.006	1.002	0.975	1.003	P04899	GNAI2	Guanine nucleotide-binding protein G(i) subunit alpha-2
0.946	0.974	0.993	0.942	0.952	1.068	0.918	0.831	0.894	0.988	1.024	1.008	Q9UBI6	GNG12	Guanine nucleotide-binding protein G(I)/G(S)/G(O) subunit gamma-12
0.990	1.018	0.991	0.958	0.835	1.007	0.968	0.934	0.800	1.035	1.003	1.010	P63218	GNG5	Guanine nucleotide-binding protein G(I)/G(S)/G(O) subunit gamma-5
1.006	0.996	0.973	0.991	1.028	0.950	1.016	0.912	1.005	0.994	0.982	0.979	P62873	GNB1	Guanine nucleotide-binding protein G(I)/G(S)/G(T) subunit beta-1
1.180	1.024	1.093	1.029	1.510	1.017	1.090	0.890	1.100	0.955	0.971	1.003	P62879	GNB2	Guanine nucleotide-binding protein G(I)/G(S)/G(T) subunit beta-2
0.989	1.051	0.956	1.058	1.047	0.950	0.953	0.991	0.970	1.133	1.012	1.046	P08754	GNAI3	Guanine nucleotide-binding protein G(k) subunit alpha
0.969	0.979	0.984	0.969	0.923	1.036	1.078	1.037	1.026	1.055	1.048	1.050	P50148	GNAQ	Guanine nucleotide-binding protein G(q) subunit alpha
0.903	0.882	1.022							0.940	1.003	1.036	P63211	GNGT1	Guanine nucleotide-binding protein G(T) subunit gamma-T1
1.090	1.183	0.944	0.937	0.975	0.879							P19086	GNAZ	Guanine nucleotide-binding protein G(z) subunit alpha
0.979	1.000	0.996	0.994	0.912	1.022	1.003	1.089	1.020	1.005	1.035	1.000	P29992	GNA11	Guanine nucleotide-binding protein subunit alpha-11
1.092	1.011	0.946	1.012	0.952	1.012	1.151	1.098	1.103	0.966	1.035	1.045	Q03113	GNA12	Guanine nucleotide-binding protein subunit alpha-12
0.971	0.992	1.028	1.006	0.902	1.035	1.000	1.044	1.009	1.034	1.030	1.029	Q14344	GNA13	Guanine nucleotide-binding protein subunit alpha-13
0.905	1.001	1.050	0.984	1.082	1.010	1.001	0.796	1.033	1.009	0.996	1.028	Q9HAV0	GNB4	Guanine nucleotide-binding protein subunit beta-4
0.915	0.864	1.071	1.120	0.863	0.892	1.035	1.262	0.914	1.008	1.010	1.083	O14775	GNB5	Guanine nucleotide-binding protein subunit beta-5
1.048	1.077	1.052	0.972	0.941	1.084	1.029	1.086	0.981	1.094	0.967	1.060	Q9BYB4	GNB1L	Guanine nucleotide-binding protein subunit beta-like protein 1
1.009	0.997	1.030	1.090	0.979	1.033	1.049	1.003	1.036	1.039	0.967	1.058	P36915	GNL1	Guanine nucleotide-binding protein-like 1
0.972	0.984	1.009	0.977	0.960	1.023	0.952	1.045	1.000	0.968	1.031	1.020	Q9BVP2	GNL3	Guanine nucleotide-binding protein-like 3
1.000	1.019	0.977	1.017	1.007	1.016	1.035	1.104	1.024	1.027	1.009	1.079	Q9NVN8	GNL3L	Guanine nucleotide-binding protein-like 3-like protein
1.009	1.049	1.042	1.054	1.030	1.036	1.060	1.039	0.965	0.997	0.901	1.031	P32455	GBP1	Guanylate-binding protein 1
0.993	1.043	1.078	1.053	0.973	1.027	1.105	1.036	0.980	1.049	0.896	1.093	P32456	GBP2	Guanylate-binding protein 2
			0.818	0.958	1.144	1.038	0.988	1.065				Q9H0R5	GBP3	Guanylate-binding protein 3

Abundance Ratio												Accession ID	Gene name	Protein Description
1 d M1/C1	1 d M2/C2	1 d M3/C3	4 d M1/C1	4 d M2/C2	4 d M3/C3	7 d M1/C1	7 d M2/C2	7 d M3/C3	10 d M1/C1	10 d M2/C2	10 d M3/C3			
0.956	1.012	1.040	0.847	1.223	0.985	1.182	1.052	1.130	0.896	0.894	1.098	Q96PP9	GBP4	Guanylate-binding protein 4
1.069	1.047	1.059	0.984	0.811	1.049	0.973	0.995	1.012				Q8N8V2	GBP7	Guanylate-binding protein 7
0.994	0.925	0.973	1.012	0.901	0.983	1.109	1.009	1.016	1.156	1.102	1.192	P51790	CLCN3	H(+)/Cl(-) exchange transporter 3
1.048	1.058	1.028	1.031	0.912	1.089	1.025	1.060	1.049	1.076	1.060	0.984	P51798	CLCN7	H(+)/Cl(-) exchange transporter 7
1.029	0.750	1.104	1.142	0.661	0.905	0.598	1.257	1.248	0.917	0.946	1.088	Q96HR8	NAF1	H/ACA ribonucleoprotein complex non-core subunit NAF1
1.047	1.021	1.001	0.984	1.074	0.996	1.015	0.984	1.049	1.011	0.992	0.966	Q9NY12	GAR1	H/ACA ribonucleoprotein complex subunit 1
1.007	1.029	0.961	1.004	1.049	0.999	1.033	0.977	1.010	1.014	1.002	0.982	Q9NX24	NHP2	H/ACA ribonucleoprotein complex subunit 2
1.033	1.016	0.999	1.044	0.992	0.939	0.994	1.052	0.964	0.938	1.061	0.912	Q9NPE3	NOP10	H/ACA ribonucleoprotein complex subunit 3
1.007	0.996	1.002	0.983	0.998	1.000	0.990	0.990	0.998	0.972	1.021	0.963	O60832	DKC1	H/ACA ribonucleoprotein complex subunit 4
0.986	0.989	1.009	0.965	0.970	1.010	1.005	1.020	1.002	1.008	0.981	0.988	Q9BXW7	HDHD5	Haloacid dehalogenase-like hydrolase domain-containing 5
0.989	1.007	0.986	0.997	0.949	1.037	1.004	1.015	1.003	1.033	0.999	1.029	Q9H0R4	HDHD2	Haloacid dehalogenase-like hydrolase domain-containing protein 2
1.083	1.033	1.033	0.941	0.980	1.019	0.956	1.068	0.996	1.035	0.982	1.028	Q9BSH5	HDHD3	Haloacid dehalogenase-like hydrolase domain-containing protein 3
1.005	1.008	1.049	1.003	1.114	1.045	1.026	1.112	0.950	1.029	1.018	1.028	Q92574	TSC1	Hamartin
1.085	0.996	1.019	0.952	0.896	1.167	0.973	1.021	1.040	0.950	0.994	0.968	Q96CS2	HAUS1	HAUS augmin-like complex subunit 1
0.987	0.928	1.017	1.084	1.079	0.835	1.280	1.028	0.846	0.948	0.934	0.967	Q9NVX0	HAUS2	HAUS augmin-like complex subunit 2
0.933	1.008	1.070	0.989	0.877	1.040	0.992	1.092	1.089	0.967	0.962	1.041	Q68CZ6	HAUS3	HAUS augmin-like complex subunit 3
1.080	0.990	1.024	0.996	1.060	1.043	1.028	1.107	1.056	1.051	1.052	1.049	Q9H6D7	HAUS4	HAUS augmin-like complex subunit 4
1.020	0.983	1.029	0.943	0.995	0.990	1.029	1.046	1.038	0.989	0.951	1.048	O94927	HAUS5	HAUS augmin-like complex subunit 5
1.054	0.966	1.023	1.066	0.970	1.016	0.967	1.102	0.993	0.913	0.942	1.083	Q7Z4H7	HAUS6	HAUS augmin-like complex subunit 6
0.778	0.996	1.028	0.818	0.986	0.971	0.749	0.582	1.061	0.935	0.928	1.016	Q9BT25	HAUS8	HAUS augmin-like complex subunit 8
0.994	1.024	1.024	1.018	0.983	1.002	1.029	1.021	1.032	1.025	1.035	1.051	Q9Y450	HBS1L	HBS1-like protein
1.140	1.115	1.040	0.994	0.992	1.102	0.991	0.976	1.028	1.009	1.095	0.992	A0A0C4DFM2	ZNF574	HCG1643764, isoform CRA_b
0.650	1.269	1.495				1.441	0.693	1.135				J3KR51	ZNF525	HCG1787564
1.084	1.009	0.987	1.004	0.951	1.045	0.972	1.086	0.987	0.988	1.081	0.917	I3L4Q0	MCRIP1	HCG1818442, isoform CRA_a
1.310	1.170	1.301	0.931	1.011	1.013				0.915	1.322	0.993	A4D104	LOC401307	HCG19535
1.072	0.993	1.007	0.993	0.962	0.997	1.015	1.032	1.047	0.946	0.981	0.957	I3L0E3	hCG_1984214	HCG1984214, isoform CRA_a
1.057	1.020	0.982	1.060	1.067	0.985	1.019	1.129	0.977	1.028	1.023	1.065	G3XAH0	5-Sep	HCG2002594, isoform CRA_c
0.934	0.989	0.990	0.982	1.007	0.943	1.021	1.008	0.949				J3KQ70	INO80B-WBP1	HCG2039827, isoform CRA_e
1.003	1.006	1.000	0.984	0.965	1.017	1.010	1.009	1.002	0.999	1.042	1.021	A0A0B4J1V8	PPAN-P2RY11	HCG2039996
1.025	0.994	0.984	1.012	0.935	1.007	0.969	1.061	0.981	0.981	0.994	0.967	I3L0A0	TMEM189-UBE2V1	HCG2044781
0.957	0.986	1.095	0.951	1.013	0.989							D6W601	ADAT3	HCG22695, isoform CRA_a

Abundance Ratio														
1 d M1/C1	1 d M2/C2	1 d M3/C3	4 d M1/C1	4 d M2/C2	4 d M3/C3	7 d M1/C1	7 d M2/C2	7 d M3/C3	10 d M1/C1	10 d M2/C2	10 d M3/C3	Accession ID	Gene name	Protein Description
1.003	0.996	1.045	0.986	0.965	1.008	0.984	1.069	1.037	0.977	1.002	1.021	Q53T59	HS1BP3	HCLS1-binding protein 3
1.087	1.026	0.998	1.032	0.952	1.030	0.983	0.994	0.993	1.014	1.053	0.997	Q7Z4H3	HDDC2	HD domain-containing protein 2
			1.039	0.883	0.957	0.888	1.233	1.196	0.983	1.029	0.910	Q9UBI9	HECA	Headcase protein homolog
0.994	1.004	1.022	0.992	0.950	1.047	0.988	1.119	1.036	0.991	1.035	0.995	Q9H583	HEATR1	HEAT repeat-containing protein 1
0.996	0.988	1.028	0.990	0.940	1.017	1.011	1.106	1.008	0.931	0.967	1.007	Q7Z4Q2	HEATR3	HEAT repeat-containing protein 3
1.070	1.041	1.050	1.014	0.938	1.028	1.040	1.205	1.063	1.008	1.010	1.032	F5H619	HEATR5A	HEAT repeat-containing protein 5A
1.076	1.036	1.073	1.308	0.802	1.037	0.944	1.042	1.039	0.952	1.074	1.066	Q9P2D3	HEATR5B	HEAT repeat-containing protein 5B
1.014	0.984	1.052	0.962	0.979	1.027	0.956	1.013	1.087	0.930	0.965	1.022	Q6AI08	HEATR6	HEAT repeat-containing protein 6
0.959	1.003	1.111	1.061	0.887	0.977	0.998	1.065	1.025	0.973	0.943	1.038	A0A1B0GTF3	HSPA12A	Heat shock 70 kDa protein 12A
1.055	1.068	1.027	1.044	0.958	1.004	0.995	1.075	0.984	0.983	1.007	0.977	P48723	HSPA13	Heat shock 70 kDa protein 13
0.994	1.006	1.010	0.986	0.957	1.049	0.980	1.022	1.011	1.002	1.020	1.072	Q0VDF9	HSPA14	Heat shock 70 kDa protein 14
0.994	0.993	0.976	0.986	1.008	0.985	0.985	1.009	1.005	0.965	0.984	0.970	A0A0G2JIW1	HSPA1B	Heat shock 70 kDa protein 1B
						0.684	1.004	1.057	0.870	0.996	1.128	P34931	HSPA1L	Heat shock 70 kDa protein 1-like
0.957	0.994	0.987	0.988	1.030	1.002	1.003	0.965	1.008	0.996	1.024	0.987	P34932	HSPA4	Heat shock 70 kDa protein 4
0.986	1.013	0.990	1.005	0.963	1.026	1.007	0.998	0.999	0.997	0.992	1.011	O95757	HSPA4L	Heat shock 70 kDa protein 4L
0.859	1.031	0.894	0.971	1.002	1.085	1.157	1.081	1.056	1.230	1.089	1.376	E9PN89	HSPA8	Heat shock cognate 71 kDa protein (Fragment)
0.987	0.923	1.042	0.936	0.830	1.045	1.019	1.189	1.401	1.287	0.984	1.299	E9PK54	HSPA8	Heat shock cognate 71 kDa protein (Fragment)
0.999	1.017	0.990	1.013	1.064	0.997	1.016	1.001	1.027	0.976	1.013	0.972	P11142	HSPA8	Heat shock cognate 71 kDa protein
0.988	1.022	1.020	1.013	0.927	1.046	1.075	0.978	1.011	1.025	0.983	1.085	Q00613	HSF1	Heat shock factor protein 1
0.971	1.036	1.059	0.970	1.060	1.041	0.967	0.884	0.955	0.925	0.948	1.034	O75506	HSBP1	Heat shock factor-binding protein 1
0.949	0.997	0.996	0.969	0.970	0.974	0.975	0.984	1.013	0.948	0.930	1.014	Q92598	HSPH1	Heat shock protein 105 kDa
1.013	0.979	0.997	1.011	0.950	0.989	0.992	0.999	0.975	0.961	0.994	0.947	Q12931	TRAP1	Heat shock protein 75 kDa, mitochondrial
1.022	0.990	0.988	0.970	0.954	0.952	0.958	1.013	0.981	0.951	0.946	0.980	P04792	HSPB1	Heat shock protein beta-1
0.979	1.015	0.966	1.009	1.020	0.984	0.994	1.013	0.990	0.977	0.990	0.970	P08238	HSP90AB1	Heat shock protein HSP 90-beta
0.961	0.954	0.978	0.968	0.931	1.024	0.861	1.006	0.889	0.964	1.067	1.068	P54652	HSPA2	Heat shock-related 70 kDa protein 2
0.882	0.942	1.110	0.915	0.897	0.920	0.941	0.960	1.009	1.010	1.016	1.031	Q9Y4B4	RAD54L2	Helicase ARIP4
0.971	0.990	1.013	0.953	0.984	1.012	1.018	1.032	1.036	1.019	0.965	1.021	Q15477	SKIV2L	Helicase SKI2W
1.090	1.029	1.002	0.990	1.035	0.989	1.031	0.925	0.962	1.036	1.038	1.097	Q6ZRS2	SRCAP	Helicase SRCAP
0.997	0.996	1.053	0.972	0.916	1.013	0.989	0.985	0.993	1.011	0.958	1.052	Q9BYK8	HELZ2	Helicase with zinc finger domain 2
0.958	1.025	1.003	1.020	0.961	0.977	1.094	1.070	1.026	1.029	0.921	1.004	A0A0B4J1V9	HELLS	Helicase, lymphoid-specific, isoform CRA_b
0.984	1.029	1.009	0.996	0.978	0.995	1.012	1.093	1.012	1.003	0.951	0.995	Q14527	HLTF	Helicase-like transcription factor
0.945	0.955	0.998	0.936	0.959	0.904	0.964	0.895	1.002	0.943	1.000	1.022	Q9UK76	HN1	Hematological and neurological expressed 1 protein
1.008	1.007	1.021	0.985	0.992	1.006	1.067	1.070	0.990	1.081	1.141	1.057	P09601	HMOX1	Heme oxygenase 1

Abundance Ratio														
1 d M1/C1	1 d M2/C2	1 d M3/C3	4 d M1/C1	4 d M2/C2	4 d M3/C3	7 d M1/C1	7 d M2/C2	7 d M3/C3	10 d M1/C1	10 d M2/C2	10 d M3/C3	Accession ID	Gene name	Protein Description
0.904	0.990	0.988	0.985	0.959	1.022	1.034	1.024	0.997	1.016	1.020	1.035	A0A087WT44	HMOX2	Heme oxygenase 2
0.984	0.936	0.912	0.891	0.923	1.323	1.325	1.038	1.179	1.187	0.834	1.318	Q6P1K1	SLC48A1	Heme transporter HRG1
1.007	1.006	0.992	1.047	1.047	1.052	1.019	1.043	1.037	1.028	1.017	1.096	Q9NRV9	HEBP1	Heme-binding protein 1
1.071	1.006	1.005	1.035	0.975	0.973	0.991	1.047	0.999	1.038	1.072	1.004	Q9Y5Z4	HEBP2	Heme-binding protein 2
0.983	1.025	1.067	0.968	0.982	1.046	1.008	1.009	0.979	0.972	0.978	1.195	B2RA97	HEMK2	HemK methyltransferase family member 2
1.020	1.046	0.978	1.062	0.952	1.001	1.024	1.096	1.000	1.020	1.012	0.944	Q7LGA3	HS2ST1	Heparan sulfate 2-O-sulfotransferase 1
			0.854	0.764	1.065	1.159	1.054	0.966				Q68CP4	HGSNAT	Heparan-alpha-glucosaminide N-acetyltransferase
1.044	1.055	1.050	1.030	1.103	1.201	1.043	1.244	1.164	1.185	1.290	1.378	Q9Y251	HPSE	Heparanase
0.963	1.033	1.130	0.977	0.946	1.052	1.169	0.970	1.019	1.183	0.969	1.044	Q96D42	HAVCR1	Hepatitis A virus cellular receptor 1
0.946	1.017	1.037	1.016	0.895	1.049	1.036	1.003	1.019	0.956	0.972	1.022	P08581	MET	Hepatocyte growth factor receptor
1.001	1.011	0.994	1.027	1.001	0.999	1.032	1.039	0.996	1.030	1.021	1.001	O14964	HGS	Hepatocyte growth factor-regulated tyrosine kinase substrate
1.168	1.055	1.134	0.996	1.003	0.941	0.947	1.101	1.034	0.948	0.929	0.940	P35680	HNF1B	Hepatocyte nuclear factor 1-beta
0.972	0.985	0.985	0.966	1.039	0.999	0.995	0.871	1.003	1.031	0.995	1.037	P51858	HDGF	Hepatoma-derived growth factor
1.013	0.989	0.996	0.938	0.932	1.011	0.969	0.901	0.966	1.003	0.985	1.043	A0A024R216	HDGFRP3	Hepatoma-derived growth factor, related protein 3, isoform CRA_a
1.017	1.001	1.013	0.978	0.998	0.988	1.006	0.941	0.974	1.087	1.028	1.097	Q7Z4V5	HDGFRP2	Hepatoma-derived growth factor-related protein 2
1.243	0.937	1.062				0.886	1.146	1.048	0.835	0.993	0.945	Q30201	HFE	Hereditary hemochromatosis protein
0.859	1.195	1.194				1.003	1.218	1.102	0.960	1.079	1.001	Q92902	HPS1	Hermansky-Pudlak syndrome 1 protein
1.155	0.892	1.044	0.979	0.948	1.067	0.998	1.083	1.029	0.984	0.969	1.053	Q969F9	HPS3	Hermansky-Pudlak syndrome 3 protein
1.025	1.016	1.025	1.034	0.988	1.082	1.068	1.089	1.036	1.005	0.990	1.063	Q9UPZ3	HPS5	Hermansky-Pudlak syndrome 5 protein
0.991	0.971	1.037	1.024	1.001	0.999	0.982	1.013	1.066	0.995	0.989	1.096	Q86YV9	HPS6	Hermansky-Pudlak syndrome 6 protein
1.031	1.006	0.983	0.990	1.051	0.988	0.986	0.945	0.966	1.004	1.003	0.952	Q5SSJ5	HP1BP3	Heterochromatin protein 1-binding protein 3
0.987	0.999	0.982	1.000	0.970	0.996	1.012	0.978	0.969	1.071	1.046	1.039	Q13151	HNRNPA0	Heterogeneous nuclear ribonucleoprotein A0
1.019	1.000	1.013	1.003	1.017	0.966	0.961	0.991	0.997	0.939	1.048	0.974	P09651	HNRNPA1	Heterogeneous nuclear ribonucleoprotein A1
						0.959	0.349	1.443				Q32P51	HNRNPA1L2	Heterogeneous nuclear ribonucleoprotein A1-like 2
1.027	1.003	0.997	1.014	1.045	0.926	0.957	0.905	0.949	0.987	1.036	0.946	P51991	HNRNPA3	Heterogeneous nuclear ribonucleoprotein A3
0.931	0.984	0.955	0.964	1.044	0.988	0.983	0.954	1.005	0.999	1.002	0.974	O14979	HNRNPDL	Heterogeneous nuclear ribonucleoprotein D-like
1.029	0.996	0.993	1.021	1.093	1.007	1.014	0.987	1.035	1.024	1.050	0.995	P52597	HNRNPF	Heterogeneous nuclear ribonucleoprotein F
0.996	0.993	0.980	0.989	1.007	1.003	0.999	0.971	1.019	0.982	1.003	1.000	G8JLB6	HNRNPH1	Heterogeneous nuclear ribonucleoprotein H
0.972	1.000	0.980	0.994	0.990	1.015	1.012	1.015	1.016	0.996	1.020	1.015	P55795	HNRNPH2	Heterogeneous nuclear ribonucleoprotein H2
1.018	0.970	1.000	0.972	0.955	1.011	0.986	1.017	1.003	0.967	0.986	0.975	P31942	HNRNPH3	Heterogeneous nuclear ribonucleoprotein H3
0.997	0.995	0.982	1.001	1.086	0.938	1.004	0.938	0.977	0.957	1.023	0.931	P14866	HNRNPL	Heterogeneous nuclear ribonucleoprotein L

Abundance Ratio														
1 d M1/C1	1 d M2/C2	1 d M3/C3	4 d M1/C1	4 d M2/C2	4 d M3/C3	7 d M1/C1	7 d M2/C2	7 d M3/C3	10 d M1/C1	10 d M2/C2	10 d M3/C3	Accession ID	Gene name	Protein Description
1.001	0.999	0.981	1.005	1.024	0.973	1.030	0.931	0.990	1.032	1.023	1.053	D6W592	HNRPLL	Heterogeneous nuclear ribonucleoprotein L-like
0.884	0.925	0.927	1.018	0.988	0.987	1.096	0.959	0.993	1.120	1.122	0.984	M0QZM1	HNRNPM	Heterogeneous nuclear ribonucleoprotein M (Fragment)
0.944	1.005	0.954	0.989	1.007	1.002	0.990	0.987	1.005	0.999	1.007	0.996	P52272	HNRNPM	Heterogeneous nuclear ribonucleoprotein M
1.016	1.008	0.999	1.000	1.030	0.994	0.982	1.064	1.031	0.994	1.025	0.981	O60506	SYNCRIP	Heterogeneous nuclear ribonucleoprotein Q
1.014	0.991	0.993	0.992	1.024	0.981	0.975	0.996	1.009	0.972	1.011	0.965	O43390	HNRNPR	Heterogeneous nuclear ribonucleoprotein R
1.036	0.996	0.995	0.986	1.147	0.958	0.994	0.859	0.987	0.971	1.009	0.977	Q00839	HNRNPU	Heterogeneous nuclear ribonucleoprotein U
1.021	1.011	0.990	1.005	1.040	0.964	1.020	0.959	1.000	1.021	0.999	0.980	Q9BUJ2	HNRNPUL1	Heterogeneous nuclear ribonucleoprotein U-like protein 1
1.022	1.051	1.021	0.994	1.056	0.996	0.994	0.932	0.989	1.009	1.002	1.016	Q1KMD3	HNRNPUL2	Heterogeneous nuclear ribonucleoprotein U-like protein 2
1.016	0.996	0.998	0.993	1.001	0.955	0.966	0.981	0.979	0.979	1.011	0.973	P22626	HNRNPA2B1	Heterogeneous nuclear ribonucleoproteins A2/B1
			0.945	0.718	1.093	1.006	0.940	0.999				G3V5V7	HNRNPC	Heterogeneous nuclear ribonucleoproteins C1/C2 (Fragment)
1.049	0.987	1.075	1.079	0.918	1.082	1.015	0.980	0.980	0.920	0.962	1.036	P52789	HK2	Hexokinase-2
0.947	1.226	1.145	1.042	0.890	1.128	0.695	1.126	0.710	0.969	1.019	0.739	Q9BW72	HIGD2A	HIG1 domain family member 2A, mitochondrial
0.969	1.002	0.955	1.004	0.887	1.146	1.046	1.141	1.136	1.086	1.041	1.087	P30825	SLC7A1	High affinity cationic amino acid transporter 1
0.986	0.990	1.001	1.005	0.974	1.021	1.011	1.022	1.016	1.023	1.002	1.059	A0A024R4E5	HDLBP	High density lipoprotein binding protein (Vigilin), isoform CRA_a
0.933	0.899	1.028	0.827	0.951	1.109	0.855	0.609	0.901	1.091	0.895	0.810	A0A087WZE9	HMGN3	High mobility group nucleosome-binding domain-containing protein 3
1.019	0.779	0.925	0.525	0.696	1.010	0.791	0.385	0.583	1.537	0.653	0.491	O00479	HMGN4	High mobility group nucleosome-binding domain-containing protein 4
			1.021	0.838	0.919							Q5JSK9	HMGN5	High mobility group nucleosome-binding domain-containing protein 5 (Fragment)
0.988	0.986	0.967	0.913	0.929	1.010	0.957	0.800	0.917	1.089	0.960	1.065	P82970	HMGN5	High mobility group nucleosome-binding domain-containing protein 5
1.054	1.037	1.015	1.009	0.889	0.990	1.142	1.034	1.004	1.092	1.060	1.070	Q9NP66	HMG20A	High mobility group protein 20A
0.991	1.023	0.973	0.983	0.932	0.943	0.904	1.008	0.939	0.863	0.999	0.818	P09429	HMGB1	High mobility group protein B1
1.036	1.004	1.002	0.972	1.034	0.994	0.899	0.813	0.936	0.917	1.038	0.896	P26583	HMGB2	High mobility group protein B2
1.025	1.025	0.974	1.003	0.997	0.969	0.967	1.027	0.972	0.939	1.017	0.899	O15347	HMGB3	High mobility group protein B3
1.017	0.981	0.968	0.898	0.920	0.917	0.999	0.894	0.820	1.161	0.911	0.893	P17096	HMGA1	High mobility group protein HMG-I/HMG-Y
1.043	0.887	0.900	0.745	0.924	0.891	1.046	0.777	0.802	1.260	0.711	0.779	F5H2A4	HMGA2	High mobility group protein HMGI-C
0.949	0.978	0.995	0.971	0.935	0.985	1.015	0.977	0.963	1.005	0.918	1.040	P37235	HPCAL1	Hippocalcin-like protein 1
0.975	1.029	1.026	0.932	0.934	0.970	1.042	0.974	1.015				Q9BW71	HIRIP3	HIRA-interacting protein 3
									0.996	1.239	0.932	O95568	METTL18	Histidine protein methyltransferase 1 homolog
1.088	0.991	1.009	1.026	1.290	0.945	1.045	0.935	1.055	0.949	0.976	0.945	P49773	HINT1	Histidine triad nucleotide-binding protein 1

Abundance Ratio														
1 d M1/C1	1 d M2/C2	1 d M3/C3	4 d M1/C1	4 d M2/C2	4 d M3/C3	7 d M1/C1	7 d M2/C2	7 d M3/C3	10 d M1/C1	10 d M2/C2	10 d M3/C3	Accession ID	Gene name	Protein Description
1.074	1.029	1.023	0.999	0.977	0.987	0.989	1.017	1.041	1.030	1.019	0.979	Q9BX68	HINT2	Histidine triad nucleotide-binding protein 2, mitochondrial
0.954	0.996	1.120	0.982	0.999	0.993	1.033	1.097	1.073	0.986	1.018	1.016	Q9NQE9	HINT3	Histidine triad nucleotide-binding protein 3
1.259	1.092	0.844	0.979	1.054	1.053							P04196	HRG	Histidine-rich glycoprotein
0.983	0.977	1.016	1.004	0.981	1.019	0.991	1.013	1.031	1.010	1.005	1.014	P12081	HARS	Histidine--tRNA ligase, cytoplasmic
1.005	1.018	1.017	0.925	0.971	1.017	0.994	1.136	1.033	0.960	1.021	1.053	Q92830	KAT2A	Histone acetyltransferase KAT2A
			0.981	1.283	1.239	0.683	1.086	0.758	1.061	0.774	0.769	Q92794	KAT6A	Histone acetyltransferase KAT6A
1.152	1.144	1.233										Q8WYB5	KAT6B	Histone acetyltransferase KAT6B
0.970	0.975	1.014	0.991	0.979	1.005	0.978	0.983	0.975	0.971	1.045	1.001	O95251	KAT7	Histone acetyltransferase KAT7
1.048	0.995	0.954	0.957	0.996	1.042	1.036	0.945	1.020	1.013	0.965	1.081	Q09472	EP300	Histone acetyltransferase p300
0.970	0.974	0.987	0.958	0.920	0.984	0.947	1.090	1.091	0.917	0.909	1.000	O14929	HAT1	Histone acetyltransferase type B catalytic subunit
1.008	0.972	1.021	1.053	0.970	1.066	1.129	1.173	0.971	0.957	1.073	0.947	Q9Y294	ASF1A	Histone chaperone ASF1A
0.986	1.039	0.987	1.020	1.099	0.975	1.029	0.957	0.985	1.053	0.984	1.046	Q13547	HDAC1	Histone deacetylase 1
			0.952	0.930	1.247							Q969S8	HDAC10	Histone deacetylase 10
1.001	1.079	0.955	1.012	1.107	0.977	1.031	1.011	1.011	0.984	1.013	1.019	Q92769	HDAC2	Histone deacetylase 2
0.961	0.981	1.014	0.959	0.938	0.990	1.017	0.998	0.988	1.015	0.891	1.000	Q9UBN7	HDAC6	Histone deacetylase 6
1.043	1.062	1.017	0.970	0.936	0.974	0.977	1.096	0.957	0.957	0.960	0.987	Q9BY41	HDAC8	Histone deacetylase 8
1.026	0.991	1.000	0.989	0.966	1.014	0.978	0.933	0.955	0.993	1.024	1.025	X6RAL5	SAP18	Histone deacetylase complex subunit SAP18
1.068	1.053	0.977	1.063	0.931	1.003	1.061	1.093	0.998	1.038	0.968	1.000	O75446	SAP30	Histone deacetylase complex subunit SAP30
1.091	1.015	1.067	1.019	0.881	1.036	1.031	0.941	1.005	1.013	1.027	1.077	Q9HAJ7	SAP30L	Histone deacetylase complex subunit SAP30L
0.974	0.952	1.048	1.017	0.993	1.016	1.013	0.997	0.983	1.026	0.971	1.083	J3KPH8	HDAC7	Histone deacetylase
0.965	0.988	0.956	0.941	0.929	0.989	0.913	0.879	0.929	0.938	0.937	0.912	P07305	H1F0	Histone H1.0
1.033	0.875	0.969	0.671	0.994	0.963	0.864	0.456	0.795	1.072	1.003	0.801	P16403	HIST1H1C	Histone H1.2
1.145	0.959	0.986	1.015	0.913	1.007	0.973	0.831	0.943	1.050	0.987	0.851	P16402	HIST1H1D	Histone H1.3
1.092	0.809	1.050	0.524	1.032	0.994	0.743	0.272	0.769	0.859	0.897	0.519	P10412	HIST1H1E	Histone H1.4
1.044	0.953	0.986	0.860	0.951	0.961	0.888	0.736	0.883	0.988	0.984	0.845	P16401	HIST1H1B	Histone H1.5
0.486	0.789	1.568	0.439	0.959	1.910	0.420	1.000	1.459	0.765	0.603	1.291	Q8IZA3	H1FOO	Histone H1oo
1.056	0.983	1.013	0.966	1.003	1.057	0.896	0.865	0.919	0.948	1.042	0.926	Q92522	H1FX	Histone H1x
0.831	0.900	0.896				0.909	1.159	1.048	1.048	1.009	1.148	Q5VVJ2	MYSM1	Histone H2A deubiquitinase MYSM1
1.271	0.994	1.100	1.078	1.000	0.977	0.943	0.900	1.005	0.966	0.957	0.845	P04908	HIST1H2AB	Histone H2A type 1-B/E
1.011	1.051	0.871	0.976	0.998	1.168	0.935	0.958	1.316	0.856	0.893	1.168	Q6FI13	HIST2H2AA3	Histone H2A type 2-A
0.978	0.957	1.053	0.858	0.986	1.065	0.775	0.542	0.857	0.928	0.998	0.924	Q8IUE6	HIST2H2AB	Histone H2A type 2-B
1.050	0.998	1.003	0.999	0.946	0.988	1.000	1.046	0.998	0.934	0.958	0.948	P0C0S5	H2AFZ	Histone H2A.Z
1.139	1.062	1.081	1.016	1.005	1.073	0.961	1.025	1.000	1.032	0.989	0.969	P0C5Z0	H2AFB2	Histone H2A-Bbd type 2/3

Abundance Ratio														
1 d M1/C1	1 d M2/C2	1 d M3/C3	4 d M1/C1	4 d M2/C2	4 d M3/C3	7 d M1/C1	7 d M2/C2	7 d M3/C3	10 d M1/C1	10 d M2/C2	10 d M3/C3	Accession ID	Gene name	Protein Description
1.149	0.928	1.031	1.095	0.850	0.919	0.876	0.771	0.920	0.976	0.922	0.891	P16104	H2AFX	Histone H2AX
0.974	0.981	1.137	0.920	0.908	1.097	0.923	1.116	0.940	1.008	1.090	1.033	Q96A08	HIST1H2BA	Histone H2B type 1-A
1.151	1.024	0.901	1.070	0.961	0.913	0.992	0.955	0.869	1.049	1.020	0.840	P06899	HIST1H2BJ	Histone H2B type 1-J
1.031	0.966	0.976	1.041	0.987	1.034	1.021	0.912	0.968	1.004	1.021	0.948	Q99880	HIST1H2BL	Histone H2B type 1-L
1.202	0.931	1.001	0.932	0.848	0.997	0.973	0.935	0.972	0.985	0.949	0.907	Q8N257	HIST3H2BB	Histone H2B type 3-B
1.050	1.023	0.984	0.985	1.092	0.984	0.985	0.977	1.003	0.956	0.990	0.951	Q5TEC6	HIST2H3PS2	Histone H3
1.129	0.933	1.007	1.145	1.043	0.990	1.105	0.808	0.994	1.026	0.956	0.885	P68431	HIST1H3A	Histone H3.1
									0.795	0.956	1.002	Q16695	HIST3H3	Histone H3.1t
1.493	1.088	1.199	1.164	2.038	1.135	1.024	0.745	1.347	0.839	1.056	0.795	Q71DI3	HIST2H3A	Histone H3.2
1.123	1.012	1.057	1.157	2.191	0.980	0.971	0.750	1.164	0.926	0.984	0.966	P84243	H3F3A	Histone H3.3
1.258	0.719	1.230	0.957	0.727	0.928	1.099	0.550	0.948	0.988	0.345	1.195	P49450	CENPA	Histone H3-like centromeric protein A
1.043	0.998	0.976	1.054	0.940	0.984	1.011	1.019	0.952	0.931	0.988	0.870	P62805	HIST1H4A	Histone H4
1.020	1.007	1.070	1.058	0.929	1.036	1.035	1.027	0.943	1.059	1.029	1.039	Q9UPP1	PHF8	Histone lysine demethylase PHF8
1.028	1.024	1.048	0.997	1.023	1.021	1.035	1.109	1.022	1.024	0.984	1.061	Q9NWY4	HPF1	Histone PARylation factor 1
			1.091	0.914	1.040	1.202	1.062	0.980	0.985	1.042	1.143	F8W8D3	SLBP	Histone RNA hairpin-binding protein
1.014	0.984	1.020	0.981	0.963	0.992	1.003	0.997	1.028	0.963	0.995	1.006	Q86X55	CARM1	Histone-arginine methyltransferase CARM1
0.993	0.992	0.993	0.976	0.967	0.976	1.024	1.050	1.006	0.997	0.989	1.013	Q09028	RBBP4	Histone-binding protein RBBP4
0.935	1.014	0.970	1.033	0.947	0.983	1.111	1.104	0.961	1.068	0.932	1.126	Q16576	RBBP7	Histone-binding protein RBBP7
1.002	1.107	1.082	0.994	0.948	0.918	0.828	1.191	0.935	0.967	1.025	1.065	Q9UMN6	KMT2B	Histone-lysine N-methyltransferase 2B
1.001	0.965	1.007	0.860	0.910	1.255	1.086	0.840	0.896	1.090	1.106	1.370	A0A1B0GU48	EHMT1	Histone-lysine N-methyltransferase EHMT1 (Fragment)
1.036	1.094	1.041	1.042	1.025	1.059	1.024	0.964	1.011	1.080	0.970	1.098	Q9H9B1	EHMT1	Histone-lysine N-methyltransferase EHMT1
0.994	0.970	0.971	1.050	1.089	1.060	1.068	0.893	1.024	1.097	0.993	1.113	A0A0G2JK64	EHMT2	Histone-lysine N-methyltransferase EHMT2
0.931	0.947	1.099	0.950	0.928	1.063	0.936	1.119	1.064	0.892	1.013	0.941	Q86Y97	KMT5C	Histone-lysine N-methyltransferase KMT5C
1.021	1.035	1.005	0.907	1.001	0.959	0.997	1.064	1.005	1.047	0.979	1.164	O96028	NSD2	Histone-lysine N-methyltransferase NSD2
1.085	1.000	1.233	1.024	0.978	1.100				0.866	0.830	1.294	Q9BZ95	NSD3	Histone-lysine N-methyltransferase NSD3
0.996	0.945	1.010	1.029	0.893	1.031	1.029	1.044	0.951	1.050	1.037	1.175	O15047	SETD1A	Histone-lysine N-methyltransferase SETD1A
									1.078	0.980	0.862	Q9UPS6	SETD1B	Histone-lysine N-methyltransferase SETD1B
1.011	0.994	1.023	1.003	1.028	1.030	1.092	0.955	0.979	1.049	1.108	1.273	Q9BYW2	SETD2	Histone-lysine N-methyltransferase SETD2
0.924	0.969	0.994	0.989	0.974	1.041	1.019	1.024	1.016	1.058	0.978	1.208	Q86TU7	SETD3	Histone-lysine N-methyltransferase setd3
1.069	1.076	1.057	1.032	0.906	1.050	1.046	1.013	1.052	1.008	0.976	1.056	Q8WTS6	SETD7	Histone-lysine N-methyltransferase SETD7
1.043	1.022	1.014	0.986	1.007	1.064	1.023	1.023	1.001	0.956	1.011	1.084	Q15047	SETDB1	Histone-lysine N-methyltransferase SETDB1
1.209	0.968	1.090	0.949	1.074	1.071	1.159	1.204	1.016	1.074	1.031	1.155	Q53H47	SETMAR	Histone-lysine N-methyltransferase SETMAR
0.964	0.982	1.040	0.955	0.962	1.098	0.977	1.060	1.019	1.071	1.003	1.191	Q9H7B4	SMYD3	Histone-lysine N-methyltransferase SMYD3

Abundance Ratio														
1 d M1/C1	1 d M2/C2	1 d M3/C3	4 d M1/C1	4 d M2/C2	4 d M3/C3	7 d M1/C1	7 d M2/C2	7 d M3/C3	10 d M1/C1	10 d M2/C2	10 d M3/C3	Accession ID	Gene name	Protein Description
									1.011	1.028	1.194	Q9H5I1	SUV39H2	Histone-lysine N-methyltransferase SUV39H2
1.401	1.063	1.340										Q96L73	NSD1	Histone-lysine N-methyltransferase, H3 lysine-36 and H4 lysine-20 specific
0.972	0.968	1.006	0.959	0.991	1.005	0.940	0.760	1.002	1.020	0.989	1.069	O43719	HTATSF1	HIV Tat-specific factor 1
0.921	1.006	1.004	1.009	0.941	1.005	0.994	1.010	0.994	1.012	1.018	1.037	A0A0G2JIF2	HLA-A	HLA class I histocompatibility antigen, A-3 alpha chain
0.999	0.960	1.002	1.000	0.931	1.006	1.076	1.045	1.024	1.063	0.978	1.074	P13747	HLA-E	HLA class I histocompatibility antigen, alpha chain E
1.007	1.024	1.003	1.017	0.950	1.001	1.058	0.938	0.999	1.082	1.016	1.067	P30466	HLA-B	HLA class I histocompatibility antigen, B-18 alpha chain
0.999	1.033	1.017	1.034	1.045	1.081	1.034	0.958	0.969	1.030	1.039	1.049	A0A0G2JH50	HLA-C	HLA class I histocompatibility antigen, Cw-6 alpha chain
1.063	1.082	0.982	0.987	0.952	0.995	1.007	1.055	1.019	0.982	1.055	0.983	A0A0G2JJD3	ABHD16A	HLA-B associated transcript 5, isoform CRA_b
0.960	1.054	1.046	0.929	1.032	1.006	0.961	0.930	1.088				Q8WY36	BBX	HMG box transcription factor BBX
1.078	1.010	1.050	0.962	0.946	1.006	0.931	1.059	1.027	0.912	0.984	1.029	Q9UGU5	HMGXB4	HMG domain-containing protein 4
			1.044	0.908	1.033							Q8NCD3	HJURP	Holliday junction recognition protein
1.050	0.875	1.034	1.109	1.167	1.147	1.014	0.887	1.099	1.427	1.120	1.356	Q00056	HOXA4	Homeobox protein Hox-A4
			1.006	0.799	0.963	0.941	0.918	0.974	0.918	0.896	1.130	P17483	HOXB4	Homeobox protein Hox-B4
0.756	0.943	0.829	0.812	0.980	0.845							F5GYS8	MEIS1	Homeobox protein Meis1
						0.833	1.112	0.851	1.010	0.986	1.002	P32242	OTX1	Homeobox protein OTX1
0.982	0.981	0.955	0.975	0.935	1.117	1.072	0.781	1.021	1.040	1.042	1.186	P55347	PKNOX1	Homeobox protein PKNOX1
1.071	1.261	1.325	1.048	0.891	1.057	1.011	1.409	1.078	0.803	1.001	0.978	Q9UIU6	SIX4	Homeobox protein SIX4
1.047	1.068	1.238	0.953	0.899	0.899							Q8N196	SIX5	Homeobox protein SIX5
0.967	0.891	1.062	0.973	0.900	1.016	0.997	0.999	0.980	1.006	1.072	0.944	Q15583	TGIF1	Homeobox protein TGIF1
			0.937	0.720	1.443							Q86YM7	HOMER1	Homer protein homolog 1
1.075	1.040	1.029	1.005	0.959	0.974	0.945	1.068	0.955	0.977	1.115	0.962	Q9NSB8	HOMER2	Homer protein homolog 2
1.000	0.957	1.004	1.026	0.988	1.026	1.032	1.019	1.035	1.042	0.969	1.092	Q9NSC5	HOMER3	Homer protein homolog 3
			0.800	0.966	1.301	0.917	0.805	1.259	0.808	0.943	1.325	Q15011	HERPUD1	Homocysteine-responsive endoplasmic reticulum-resident ubiquitin-like domain member 1 protein
0.811	0.932	0.991	1.057	1.026	1.103	1.051	1.081	0.959				Q9BSE4	HERPUD2	Homocysteine-responsive endoplasmic reticulum-resident ubiquitin-like domain member 2 protein
1.036	1.022	0.974	1.002	0.900	1.144				0.783	0.977	1.020	Q9P2W1	PSMC3IP	Homologous-pairing protein 2 homolog
									1.317	1.067	1.149	Q05469	LIPE	Hormone-sensitive lipase
1.004	0.756	1.025	1.131	0.947	0.513	0.970	0.666	0.592	1.017	2.231	0.765	Q86YZ3	HRNR	Hornerin
1.016	1.013	1.001	1.007	1.040	0.982	1.008	1.010	1.011	1.005	0.992	1.000	A6NEM2	HCFC1	Host cell factor 1
0.922	0.927	1.033	1.037	1.124	1.058	1.002	1.218	1.011	1.073	1.083	1.140	P51610	HCFC1	Host cell factor 1

Abundance Ratio														
1 d M1/C1	1 d M2/C2	1 d M3/C3	4 d M1/C1	4 d M2/C2	4 d M3/C3	7 d M1/C1	7 d M2/C2	7 d M3/C3	10 d M1/C1	10 d M2/C2	10 d M3/C3	Accession ID	Gene name	Protein Description
			0.726	1.195	0.905							Q9Y5Z7	HCFC2	Host cell factor 2
0.978	0.985	0.973	1.001	1.013	0.994	0.978	0.994	0.967	0.969	1.006	0.945	P50502	ST13	Hsc70-interacting protein
0.959	1.006	0.983	0.998	1.002	0.988	1.003	0.973	0.985	1.011	0.989	1.045	Q16543	CDC37	Hsp90 co-chaperone Cdc37
0.951	1.099	1.026	1.078	0.933	1.022	1.021	1.196	1.033	1.025	1.071	1.075	Q96EW2	HSPBAP1	HSPB1-associated protein 1
1.011	1.004	1.012	1.000	0.984	1.016	1.017	1.068	1.017	0.980	0.993	1.041	P42858	HTT	Huntingtin
0.949	0.981	1.023	0.940	0.947	0.986	0.972	0.953	0.968	1.010	0.948	1.042	O00291	HIP1	Huntingtin-interacting protein 1
1.014	0.953	1.038	0.993	0.963	1.033	0.973	0.998	0.982	1.017	0.971	1.059	O75146	HIP1R	Huntingtin-interacting protein 1-related protein
0.989	0.992	0.967	1.027	0.956	1.023	0.999	0.993	0.963	0.954	1.030	0.959	Q9NX55	HYPK	Huntingtin-interacting protein K
1.126	1.040	1.141	1.157	1.101	1.043				1.154	0.759	1.300	O00219	HAS3	Hyaluronan synthase 3
1.025	0.996	1.037	0.973	0.958	1.006	1.043	1.015	0.981	1.020	0.989	1.050	Q14520	HABP2	Hyaluronan-binding protein 2
			0.897	0.991	1.259	1.032	0.988	0.885	1.297	1.046	1.354	Q9UJM8	HAO1	Hydroxyacid oxidase 1
0.965	0.987	0.950	0.978	0.955	0.988	0.979	1.007	0.977	1.005	0.997	0.962	A0A0A0MSE2	HADH	Hydroxyacyl-coenzyme A dehydrogenase, mitochondrial
0.980	0.957	1.000	1.026	0.966	1.041	1.028	0.976	1.037	1.063	0.964	1.043	Q16775	HAGH	Hydroxyacylglutathione hydrolase, mitochondrial
1.027	0.984	1.021	0.986	1.030	0.985	0.972	0.913	1.008	0.963	0.991	0.955	P35914	HMGCL	Hydroxymethylglutaryl-CoA lyase, mitochondrial
1.004	1.045	1.026	1.090	1.035	1.037	1.133	1.143	1.062	1.158	1.233	1.015	Q01581	HMGCS1	Hydroxymethylglutaryl-CoA synthase, cytoplasmic
1.022	0.995	1.004	1.012	1.047	0.994	0.993	0.950	0.997	0.986	0.997	0.975	Q6YN16	HSDL2	Hydroxysteroid dehydrogenase-like protein 2
1.041	1.012	0.998	0.997	1.039	0.995	0.998	1.065	1.010	0.974	0.992	0.978	P00492	HPRT1	Hypoxanthine-guanine phosphoribosyltransferase
0.987	1.008	0.997	1.007	1.004	0.994	1.005	0.988	0.996	0.982	0.980	1.006	Q9Y4L1	HYOU1	Hypoxia up-regulated protein 1
1.039	0.981	1.030	0.994	0.945	0.957	1.011	1.045	0.995	0.993	0.949	0.894	Q9NWT6	HIF1AN	Hypoxia-inducible factor 1-alpha inhibitor
0.991	1.004	1.306	0.918	0.841	1.074				0.991	1.047	1.345	K4DIA0	ICOSLG	ICOS ligand
1.059	0.974	1.066	0.993	0.913	1.033	1.011	1.078	1.089	1.094	1.087	1.087	P22304	IDS	Iduronate 2-sulfatase
0.931	0.968	0.955	1.013	1.029	1.112	0.940	1.049	1.075	1.088	0.951	1.020	P55899	FCGRT	IgG receptor FcRn large subunit p51
0.942	1.000	0.948	0.873	0.855	0.913	0.891	1.056	0.974	0.950	0.946	0.917	Q9Y5U9	IER3IP1	Immediate early response 3-interacting protein 1
0.867	0.906	0.982	0.890	0.978	0.964	0.829	0.712	0.972	1.016	0.926	1.082	Q9GZP8	IMUP	Immortalization up-regulated protein
1.018	1.024	1.022	1.023	0.976	1.054	1.048	1.008	0.996	1.052	1.007	1.084	Q8WZA9	IRGQ	Immunity-related GTPase family Q protein
0.928	1.006	0.972	0.940	0.924	1.051	1.048	1.222	1.028	0.906	0.943	1.000	O75054	IGSF3	Immunoglobulin superfamily member 3
						1.199	1.013	1.104				Q969P0	IGSF8	Immunoglobulin superfamily member 8
1.001	0.976	0.998	0.970	0.977	1.021	1.004	1.015	1.013	1.047	1.022	1.083	P78318	IGBP1	Immunoglobulin-binding protein 1
0.933	1.038	1.017	1.023	0.944	1.007	1.029	1.140	1.042	0.928	0.934	1.036	P52292	KPNA2	Importin subunit alpha-1
1.003	1.008	1.057	0.997	1.009	0.986	1.014	0.998	1.016	0.965	1.002	1.029	O00629	KPNA4	Importin subunit alpha-3
0.956	0.996	0.947	0.961	0.987	1.008	0.975	1.114	1.036	0.959	0.973	1.008	O00505	KPNA3	Importin subunit alpha-4

Abundance Ratio														
1 d M1/C1	1 d M2/C2	1 d M3/C3	4 d M1/C1	4 d M2/C2	4 d M3/C3	7 d M1/C1	7 d M2/C2	7 d M3/C3	10 d M1/C1	10 d M2/C2	10 d M3/C3	Accession ID	Gene name	Protein Description
0.933	1.011	0.993	1.064	1.040	1.010	1.012	0.988	1.014	0.984	1.032	1.056	P52294	KPNA1	Importin subunit alpha-5
			0.981	0.887	1.023	0.907	1.035	1.063				O15131	KPNA5	Importin subunit alpha-6
1.006	0.986	1.053	1.023	1.077	0.995	0.991	1.041	1.064	0.979	0.984	1.019	O60684	KPNA6	Importin subunit alpha-7
			1.105	1.156	0.868	0.817	1.126	1.054	0.975	1.052	1.143	J3QKQ5	KPNB1	Importin subunit beta-1 (Fragment)
1.005	1.020	1.027	0.998	1.018	1.002	1.009	1.040	1.032	0.982	1.006	0.982	Q14974	KPNB1	Importin subunit beta-1
			1.004	0.869	1.006				0.968	1.099	0.840	O94829	IPO13	Importin-13
1.000	1.005	0.994	0.997	0.968	1.022	1.000	1.099	1.046	0.947	1.011	0.969	O95373	IPO7	Importin-7
1.023	1.017	1.014	1.072	1.001	1.008	0.991	1.063	0.975	0.918	0.984	1.117	O15397	IPO8	Importin-8
0.980	0.993	0.973	0.968	0.944	1.032	1.012	1.067	1.029	1.022	1.021	1.015	Q96P70	IPO9	Importin-9
0.969	0.937	0.956	0.985	0.897	0.957	1.052	1.018	0.976	1.012	1.014	1.010	Q3SXM5	HSDL1	Inactive hydroxysteroid dehydrogenase-like protein 1
			1.039	0.940	1.090	0.920	0.964	1.013	0.999	1.131	1.051	Q6PJF5	RHBDF2	Inactive rhomboid protein 2
0.962	1.014	1.205	0.893	0.863	0.990				0.897	0.943	1.007	Q8IV63	VRK3	Inactive serine/threonine-protein kinase VRK3
0.998	0.984	1.095	0.999	0.961	1.030	1.076	1.015	1.048	1.017	0.946	1.206	Q01973	ROR1	Inactive tyrosine-protein kinase transmembrane receptor ROR1
1.145	1.144	0.955	0.965	0.849	1.027	1.049	1.018	1.085	1.688	1.035	0.962	Q70EK8	USP53	Inactive ubiquitin carboxyl-terminal hydrolase 53
			1.053	0.909	1.102							Q70EL1	USP54	Inactive ubiquitin carboxyl-terminal hydrolase 54
1.052	1.000	0.942	0.990	0.920	0.905	1.002	1.028	0.911	0.916	0.977	0.937	Q9NUU6	FAM105A	Inactive ubiquitin thioesterase FAM105A
1.004	0.971	1.071	0.972	0.997	1.054	0.988	1.071	0.993	0.978	1.000	1.054	Q8NI35	PATJ	InaD-like protein
						1.103	1.139	1.065				P14902	IDO1	Indoleamine 2,3-dioxygenase 1
1.024	0.977	1.003	1.068	1.059	1.114	1.072	1.104	1.112	1.091	1.005	1.065	Q07820	MCL1	Induced myeloid leukemia cell differentiation protein Mcl-1
1.082	1.039	0.918	1.046	0.968	1.008	0.981	1.054	0.992	0.977	0.935	1.026	Q9Y6Y0	IVNS1ABP	Influenza virus NS1A-binding protein
1.053	0.994	1.065	0.987	0.980	1.028	0.998	1.042	1.000	1.062	1.001	1.045	Q9P2D0	IBTK	Inhibitor of Bruton tyrosine kinase
			1.058	1.209	1.535	0.793	1.026	0.930				Q9H160	ING2	Inhibitor of growth protein 2
0.979	0.910	1.053	0.961	0.959	0.972	0.996	1.119	0.978	1.086	1.127	1.045	Q9NXR8	ING3	Inhibitor of growth protein 3
0.984	0.914	1.028	1.101	1.072	1.063	1.128	1.020	0.855				Q9UNL4	ING4	Inhibitor of growth protein 4
1.043	1.005	1.014	0.989	0.944	0.966	1.023	1.024	1.012	0.932	0.981	1.015	A0A1B0GW41	ING5	Inhibitor of growth protein 5
0.953	1.049	1.025	1.004	0.986	1.123	1.255	0.819	1.063	0.804	0.986	1.017	A0A0C4DFW2	ING1	Inhibitor of growth protein
1.007	0.983	1.020	1.016	0.998	0.985	1.014	1.064	1.014	1.005	1.042	1.007	O15111	CHUK	Inhibitor of nuclear factor kappa-B kinase subunit alpha
1.122	1.000	0.990	1.059	0.979	0.999	1.403	1.072	1.017	2.473	0.991	0.982	O14920	IKBKB	Inhibitor of nuclear factor kappa-B kinase subunit beta
0.922	1.041	0.923										Q14164	IKBKE	Inhibitor of nuclear factor kappa-B kinase subunit epsilon

Abundance Ratio														
1 d M1/C1	1 d M2/C2	1 d M3/C3	4 d M1/C1	4 d M2/C2	4 d M3/C3	7 d M1/C1	7 d M2/C2	7 d M3/C3	10 d M1/C1	10 d M2/C2	10 d M3/C3	Accession ID	Gene name	Protein Description
0.973	0.982	1.003	0.974	0.913	1.020	0.985	1.020	0.991	0.965	0.949	0.964	Q70UQ0	IKBIP	Inhibitor of nuclear factor kappa-B kinase-interacting protein
0.993	0.999	1.072	0.909	0.978	1.122	1.050	0.957	1.054	0.824	0.823	1.085	Q9NQS7	INCENP	Inner centromere protein
1.029	0.995	1.001	1.030	0.951	0.998	1.010	1.047	1.009	1.092	1.055	1.061	Q9Y2U8	LEMD3	Inner nuclear membrane protein Man1
1.094	1.037	1.050	0.993	0.924	0.908	0.978	1.201	0.988				Q8NBZ0	INO80E	INO80 complex subunit E
									1.105	1.009	1.298	A0A0C4DGB9	PPA2	Inorganic pyrophosphatase 2, mitochondrial
1.092	1.006	1.004	1.010	1.029	0.967	0.994	1.019	1.003	0.997	0.997	0.957	Q9H2U2	PPA2	Inorganic pyrophosphatase 2, mitochondrial
1.000	1.001	0.997	1.006	1.026	1.010	1.028	1.035	1.026	0.986	1.017	1.016	Q15181	PPA1	Inorganic pyrophosphatase
1.070	0.990	0.973	1.021	1.108	0.991	1.005	1.047	1.041	0.991	1.057	0.987	Q9BY32	ITPA	Inosine triphosphate pyrophosphatase
1.035	1.011	0.999	1.044	1.058	0.987	1.015	1.034	1.040	0.965	1.037	0.946	P12268	IMPDH2	Inosine-5'-monophosphate dehydrogenase 2
1.053	0.978	0.984	0.992	0.926	1.011	0.991	1.017	1.098	1.054	0.996	1.083	Q14643	ITPR1	Inositol 1,4,5-trisphosphate receptor type 1
1.012	1.003	1.031	0.993	0.958	1.065	1.033	1.040	1.004	1.061	1.002	1.121	Q14571	ITPR2	Inositol 1,4,5-trisphosphate receptor type 2
0.962	0.969	0.982	0.994	0.940	1.014	1.021	0.986	0.994	1.098	1.004	1.070	Q14573	ITPR3	Inositol 1,4,5-trisphosphate receptor type 3
1.087	1.024	1.100	0.984	0.919	1.081	0.997	0.936	0.995	1.042	0.964	1.046	Q8IWB1	ITPRIP	Inositol 1,4,5-trisphosphate receptor-interacting protein
1.124	0.940	1.103	1.044	0.807	1.110	0.475	1.043	0.159	1.174	1.089	1.255	Q3MIP1	ITPRIPL2	Inositol 1,4,5-trisphosphate receptor-interacting protein-like 2
1.208	0.993	1.221	1.106	1.099	1.280	0.950	1.049	1.051	1.331	1.026	1.449	Q6PFW1	PPIP5K1	Inositol hexakisphosphate and diphosphoinositol-pentakisphosphate kinase 1
1.040	0.998	1.079	1.063	0.995	0.952	0.916	1.013	1.131	0.995	1.035	0.966	H0Y9S9	PPIP5K2	Inositol hexakisphosphate and diphosphoinositol-pentakisphosphate kinase 2 (Fragment)
1.003	1.017	0.987	1.019	0.996	0.996	0.983	1.045	0.982	0.987	1.002	1.017	A0A087WZV0	PPIP5K2	Inositol hexakisphosphate and diphosphoinositol-pentakisphosphate kinase 2
1.064	0.968	0.996	0.913	0.929	0.995	1.028	0.997	1.107	1.029	1.066	1.032	Q92551	IP6K1	Inositol hexakisphosphate kinase 1
1.053	0.989	1.026	0.855	0.947	1.359	0.923	0.948	1.079	1.150	1.009	1.197	O14732	IMPA2	Inositol monophosphatase 2
0.934	0.963	1.004	0.956	0.975	1.048	0.963	0.922	0.982	0.985	1.051	0.955	Q9NX62	IMPAD1	Inositol monophosphatase 3
0.974	0.978	0.935	0.955	0.990	1.074	0.997	0.975	1.037	1.027	0.925	1.016	P49441	INPP1	Inositol polyphosphate 1-phosphatase
1.040	1.009	1.013	0.977	0.963	1.078	1.062	1.069	1.062	1.034	1.022	1.068	Q9BT40	INPP5K	Inositol polyphosphate 5-phosphatase K
1.047	0.992	1.009	0.998	1.003	1.024	1.009	1.042	1.062	1.007	1.017	1.032	Q01968	OCRL	Inositol polyphosphate 5-phosphatase OCRL-1
0.594	0.715	1.480	0.448	0.830	1.534				0.425	0.835	1.296	Q8NFU5	IPMK	Inositol polyphosphate multikinase
0.962	0.992	0.980	0.912	0.947	0.935	0.921	0.931	0.926	0.960	0.982	0.959	Q9NPH2	ISYNA1	Inositol-3-phosphate synthase 1
1.041	0.999	0.989	0.993	0.910	1.069	0.970	1.207	0.946	0.942	1.066	0.846	Q13572	ITPK1	Inositol-tetrakisphosphate 1-kinase
0.951	0.987	1.022	0.938	0.996	1.037	0.993	0.921	0.987	1.050	0.985	1.136	P27987	ITPKB	Inositol-trisphosphate 3-kinase B
1.042	1.008	1.038	0.887	0.972	1.055	1.037	0.805	1.148	0.966	0.875	0.919	Q96DU7	ITPKC	Inositol-trisphosphate 3-kinase C
0.928	0.921	0.974	0.957	0.899	1.072	0.902	1.161	1.078				P06213	INSR	Insulin receptor

Abundance Ratio														
1 d M1/C1	1 d M2/C2	1 d M3/C3	4 d M1/C1	4 d M2/C2	4 d M3/C3	7 d M1/C1	7 d M2/C2	7 d M3/C3	10 d M1/C1	10 d M2/C2	10 d M3/C3	Accession ID	Gene name	Protein Description
0.934	0.981	0.996	0.991	1.004	0.948	0.896	0.799	0.977	0.960	0.999	1.062	P35568	IRS1	Insulin receptor substrate 1
1.045	0.987	1.014	0.991	0.925	1.054	1.032	1.059	1.008	1.032	1.014	1.042	Q9Y4H2	IRS2	Insulin receptor substrate 2
1.006	1.014	1.005	1.011	1.005	0.984	1.012	1.063	1.019	1.001	0.998	1.057	P14735	IDE	Insulin-degrading enzyme
2.431	0.745	1.113	1.511	0.957	1.069	1.799	0.703	1.511	1.023	0.972	0.986	P08069	IGF1R	Insulin-like growth factor 1 receptor
0.998	1.006	0.993	0.996	0.981	1.020	0.975	1.005	1.007	0.994	1.021	1.002	Q9NZI8	IGF2BP1	Insulin-like growth factor 2 mRNA-binding protein 1
1.011	0.994	1.012	0.991	0.978	0.995	0.970	1.008	0.994	1.001	1.000	1.012	F8W930	IGF2BP2	Insulin-like growth factor 2 mRNA-binding protein 2
1.017	1.003	0.998	1.001	0.999	1.007	1.003	0.981	1.009	0.997	0.991	1.004	O00425	IGF2BP3	Insulin-like growth factor 2 mRNA-binding protein 3
									0.967	0.991	1.010	P08833	IGFBP1	Insulin-like growth factor-binding protein 1
									1.060	0.984	1.312	Q86XT9	TMEM219	Insulin-like growth factor-binding protein 3 receptor
0.892	0.966	1.051	1.027	1.024	1.193	0.862	0.744	1.021	0.996	1.011	1.150	P22692	IGFBP4	Insulin-like growth factor-binding protein 4
0.986	0.984	0.958	0.976	0.922	0.903	1.072	0.946	0.978	1.076	1.010	1.072	Q16270	IGFBP7	Insulin-like growth factor-binding protein 7
1.013	1.058	1.071	1.126	1.073	1.062	1.157	1.100	1.044	1.202	1.082	1.083	Q9Y287	ITM2B	Integral membrane protein 2B
1.037	1.110	1.054	0.998	0.987	1.079	1.034	1.362	1.009	0.991	1.060	1.031	Q9NQX7	ITM2C	Integral membrane protein 2C
									1.017	1.042	1.164	F6SYP7	DGCR2	Integral membrane protein DGCR2/IDD
1.122	1.024	1.018	1.075	0.937	0.992	0.987	1.238	1.011	0.818	1.629	0.933	Q7Z3F1	GPR155	Integral membrane protein GPR155
0.996	0.953	0.947	0.956	0.842	0.994	0.996	1.199	1.011	0.983	1.025	0.999	Q86V85	GPR180	Integral membrane protein GPR180
1.012	1.022	1.066	0.983	0.961	1.014	0.996	1.077	1.033	1.009	1.002	1.036	Q8N201	INTS1	Integrator complex subunit 1
0.976	1.037	1.004	1.036	0.989	1.027	0.992	1.091	1.028	1.006	0.987	1.037	Q9NVR2	INTS10	Integrator complex subunit 10
1.013	0.988	1.039	0.986	0.945	1.018	1.011	1.090	0.949	0.927	0.952	1.034	Q5TA45	INTS11	Integrator complex subunit 11
1.000	0.951	1.001	0.961	0.928	1.019	0.994	0.843	0.988	1.003	0.980	1.103	Q96CB8	INTS12	Integrator complex subunit 12
1.033	1.022	0.980	0.986	0.969	1.024	0.958	1.032	1.007	1.031	1.001	1.026	B4DJL6	INTS14	Integrator complex subunit 14
0.979	1.073	1.005	0.983	0.945	1.013	1.038	1.137	0.990	1.014	1.012	1.056	Q9H0H0	INTS2	Integrator complex subunit 2
1.040	1.001	1.049	0.999	0.961	1.008	1.002	1.060	1.038	1.020	1.034	1.002	Q96HW7	INTS4	Integrator complex subunit 4
0.998	0.997	1.029	0.991	0.927	1.077	1.050	1.092	1.064	1.050	1.010	1.070	Q6P9B9	INTS5	Integrator complex subunit 5
1.059	1.013	1.017	1.006	0.950	0.994	1.013	0.995	1.013	1.031	0.964	0.976	Q9UL03	INTS6	Integrator complex subunit 6
1.067	1.085	0.976										Q5JSJ4	INTS6L	Integrator complex subunit 6-like
1.064	1.032	1.047	0.988	0.955	1.021	1.050	1.040	1.026	0.970	0.983	0.998	Q9NVH2	INTS7	Integrator complex subunit 7
1.063	1.029	1.041	0.979	0.965	1.034	0.960	0.936	1.001	0.937	0.994	1.026	Q75QN2	INTS8	Integrator complex subunit 8
1.043	1.035	1.018	1.007	0.972	0.961	0.989	1.108	1.007	1.009	1.027	1.007	Q9NV88	INTS9	Integrator complex subunit 9
1.095	0.979	1.044	0.947	1.029	1.162	1.081	1.003	1.206				P56199	ITGA1	Integrin alpha-1
			0.773	0.823	1.057	0.928	1.099	1.364	0.995	1.117	1.146	E7ESP4	ITGA2	Integrin alpha-2

Abundance Ratio														
1 d M1/C1	1 d M2/C2	1 d M3/C3	4 d M1/C1	4 d M2/C2	4 d M3/C3	7 d M1/C1	7 d M2/C2	7 d M3/C3	10 d M1/C1	10 d M2/C2	10 d M3/C3	Accession ID	Gene name	Protein Description
0.996	0.991	1.001	1.017	0.938	1.034	1.033	1.132	0.999	0.980	1.016	1.064	P17301	ITGA2	Integrin alpha-2
0.946	1.002	0.990	1.022	1.010	1.012	1.088	1.016	1.040	1.077	1.031	1.094	P26006	ITGA3	Integrin alpha-3
0.990	1.015	0.995	1.055	0.973	1.036	1.052	1.073	0.991	0.981	0.991	1.010	P08648	ITGA5	Integrin alpha-5
0.967	0.994	1.012	1.067	1.011	1.035	1.052	1.086	1.014	0.955	0.966	1.025	P23229	ITGA6	Integrin alpha-6
			1.046	0.843	1.157	0.968	0.787	1.057				P38570	ITGAE	Integrin alpha-E
0.984	0.995	0.994	1.007	0.985	1.022	1.033	1.041	1.020	1.024	0.982	1.033	P06756	ITGAV	Integrin alpha-V
0.931	0.981	0.951	1.031	0.966	1.001	1.087	1.056	1.015	1.078	1.052	1.061	P05556	ITGB1	Integrin beta-1
1.012	0.938	1.217	0.900	0.814	1.140	0.883	1.112	0.993				O14713	ITGB1BP1	Integrin beta-1-binding protein 1
0.998	0.986	0.981	0.980	0.917	0.975	1.033	0.980	0.974	1.063	1.001	1.048	P05106	ITGB3	Integrin beta-3
0.966	1.001	0.977	0.990	0.927	1.057	1.086	1.095	1.074	1.078	1.004	1.114	P18084	ITGB5	Integrin beta-5
									1.359	1.188	1.215	P26012	ITGB8	Integrin beta-8
1.090	1.062	0.991	0.946	0.941	0.979	1.124	1.148	1.094	1.020	1.002	1.175	Q969R8	ITFG2	Integrin-alpha FG-GAP repeat-containing protein 2
1.012	1.012	1.017	0.987	1.061	1.015	0.999	0.920	1.030	1.018	1.028	1.051	Q9H0C8	ILKAP	Integrin-linked kinase-associated serine/threonine phosphatase 2C
0.941	0.995	0.992	0.968	0.997	0.989	0.989	0.958	0.986	0.992	0.977	1.055	A0A0A0MTH3	ILK	Integrin-linked protein kinase
0.983	1.008	1.023	0.971	0.945	0.939	1.007	0.971	0.952	0.991	0.974	0.979	P05362	ICAM1	Intercellular adhesion molecule 1
						0.913	0.903	0.929				P17181	IFNAR1	Interferon alpha/beta receptor 1
0.968	1.005	1.138	1.161	1.063	1.057	1.294	1.102	1.015				P15260	IFNGR1	Interferon gamma receptor 1
			0.875	0.815	1.254							E7EUY1	IFNGR2	Interferon gamma receptor 2
1.082	1.002	1.019	0.960	0.947	1.100	1.024	0.998	0.978	1.026	1.126	1.103	P14316	IRF2	Interferon regulatory factor 2
1.027	0.981	1.027	1.017	0.975	1.016	1.003	1.001	1.015	1.076	1.028	1.105	Q8IU81	IRF2BP1	Interferon regulatory factor 2-binding protein 1
0.996	1.018	1.013	1.017	0.981	0.997	1.044	0.980	0.955	1.050	0.975	1.094	Q7Z5L9	IRF2BP2	Interferon regulatory factor 2-binding protein 2
0.988	0.993	0.993	0.950	0.985	0.983	0.975	0.972	1.008	1.009	1.014	1.091	Q9H1B7	IRF2BPL	Interferon regulatory factor 2-binding protein-like
1.027	1.016	0.971	1.003	0.961	1.002	0.924	0.860	1.030	1.024	0.981	0.998	Q14653	IRF3	Interferon regulatory factor 3
1.052	1.054	1.040	0.994	0.950	0.950	1.000	1.094	0.988	1.001	0.951	1.039	Q00978	IRF9	Interferon regulatory factor 9
1.013	1.062	1.045	0.936	0.848	1.084	1.059	1.080	1.032	0.962	0.961	1.083	Q9BYX4	IFIH1	Interferon-induced helicase C domain-containing protein 1
			0.895	0.894	1.019	0.877	0.842	1.096				P09914	IFIT1	Interferon-induced protein with tetratricopeptide repeats 1
1.087	0.982	1.003	0.960	0.836	0.870	0.862	1.133	0.858	1.009	0.833	0.883	A0A087X279	IFIT2	Interferon-induced protein with tetratricopeptide repeats 2
1.049	1.018	1.123	0.968	0.967	0.937	1.104	1.056	0.923	0.951	0.931	1.157	O14879	IFIT3	Interferon-induced protein with tetratricopeptide repeats 3
0.998	1.031	1.033	1.045	1.029	0.958	1.046	1.021	0.938	1.071	1.002	1.106	Q13325	IFIT5	Interferon-induced protein with tetratricopeptide repeats 5

Abundance Ratio														
1 d M1/C1	1 d M2/C2	1 d M3/C3	4 d M1/C1	4 d M2/C2	4 d M3/C3	7 d M1/C1	7 d M2/C2	7 d M3/C3	10 d M1/C1	10 d M2/C2	10 d M3/C3	Accession ID	Gene name	Protein Description
0.998	1.004	0.933	1.067	0.936	1.040	1.104	1.112	1.043	1.225	1.079	1.033	Q01628	IFITM3	Interferon-induced transmembrane protein 3
0.992	1.002	0.996	0.981	0.960	1.012	1.004	1.054	1.020	0.950	0.975	0.988	P19525	EIF2AK2	Interferon-induced, double-stranded RNA-activated protein kinase
0.969	1.001	0.986	1.038	1.023	1.023	1.010	1.045	0.993	1.012	1.018	1.012	O75569	PRKRA	Interferon-inducible double-stranded RNA-dependent protein kinase activator A
0.860	0.896	0.990	0.971	0.964	1.055	0.906	1.110	1.065	0.981	0.958	1.072	Q12894	IFRD2	Interferon-related developmental regulator 2
1.004	1.027	0.992	0.903	1.005	1.032	1.008	1.115	1.036	0.998	0.989	1.000	Q9H9L3	ISG20L2	Interferon-stimulated 20 kDa exonuclease-like 2
1.093	1.090	1.086	0.955	0.949	1.034	1.010	1.194	1.047	0.832	0.935	1.039	Q96AZ6	ISG20	Interferon-stimulated gene 20 kDa protein
1.043	0.994	1.009	0.980	0.938	0.994	0.970	1.029	0.980	0.925	0.987	0.913	Q12905	ILF2	Interleukin enhancer-binding factor 2
1.316	1.070	1.215	0.996	0.874	1.002	1.011	1.178	1.036	1.172	0.998	1.124	Q9NPH3	IL1RAP	Interleukin-1 receptor accessory protein
1.008	1.078	0.815	1.033	1.079	1.092	0.917	1.160	1.042	0.967	1.030	0.907	P51617	IRAK1	Interleukin-1 receptor-associated kinase 1
1.074	1.042	0.977										Q9Y616	IRAK3	Interleukin-1 receptor-associated kinase 3
1.015	0.971	0.965	1.010	0.994	1.042	0.962	1.015	0.990	1.026	1.061	1.025	Q9NWZ3	IRAK4	Interleukin-1 receptor-associated kinase 4
0.999	1.112	1.108	1.003	0.975	1.033	1.065	1.076	1.047	1.123	0.984	1.132	O43187	IRAK2	Interleukin-1 receptor-associated kinase-like 2
						0.982	0.997	0.792				P78552	IL13RA1	Interleukin-13 receptor subunit alpha-1
			0.797	0.901	1.258							Q8NAC3	IL17RC	Interleukin-17 receptor C
0.975	1.016	1.018	1.006	1.001	1.068	0.995	0.946	1.009	1.093	1.048	1.047	Q14116	IL18	Interleukin-18
1.429	0.881	0.848										Q13478	IL18R1	Interleukin-18 receptor 1
0.986	0.990	1.057	0.899	0.982	0.978	1.009	1.144	0.990	0.900	0.885	1.050	Q14213	EBI3	Interleukin-27 subunit beta
1.067	1.014	1.062	1.028	0.978	1.056	1.038	1.132	1.011	1.003	0.974	1.061	P40189	IL6ST	Interleukin-6 receptor subunit beta
1.045	1.019	1.152	1.162	0.926	1.128	1.111	1.116	1.195	1.034	0.998	1.123	O15554	KCNN4	Intermediate conductance calcium-activated potassium channel protein 4
1.025	1.008	0.967	1.012	0.948	0.991	0.997	1.036	0.989	1.031	1.005	1.043	Q15811	ITSN1	Intersectin-1
1.028	1.008	1.053	1.023	0.968	1.024	1.001	1.014	1.025	0.996	0.979	1.006	Q9NZM3	ITSN2	Intersectin-2
1.055	1.036	1.014	1.008	0.831	0.970	0.939	1.018	1.019	0.996	1.004	1.124	B4DUA7	IXL	Intersex-like (Drosophila)
0.999	0.970	1.049	1.000	0.997	1.006	1.028	0.942	0.960	1.099	1.089	1.081	Q5JVS0	HABP4	Intracellular hyaluronan-binding protein 4
0.986	1.008	1.025	1.033	0.950	1.025	1.036	1.029	0.990	0.999	0.982	1.064	Q96RY7	IFT140	Intraflagellar transport protein 140 homolog
1.048	1.052	1.017	1.074	0.970	0.988	1.005	1.078	1.080	0.799	0.968	0.883	Q9UG01	IFT172	Intraflagellar transport protein 172 homolog
0.967	0.928	1.175							0.955	0.979	1.020	Q9H7X7	IFT22	Intraflagellar transport protein 22 homolog
1.059	0.986	0.999	0.967	0.920	0.930	0.914	1.055	0.996	0.863	0.890	1.007	Q9Y547	HSPB11	Intraflagellar transport protein 25 homolog
1.021	0.990	0.990	0.997	0.993	0.962	0.968	1.030	1.010	1.018	0.922	1.030	Q9BW83	IFT27	Intraflagellar transport protein 27 homolog
1.091	1.034	0.990	0.959	0.904	0.942	0.982	1.174	1.003	0.895	1.025	1.058	Q9Y366	IFT52	Intraflagellar transport protein 52 homolog
0.985	1.014	1.010	1.015	0.999	1.098	1.059	1.055	1.051	0.997	1.017	1.079	A0AVF1	TTC26	Intraflagellar transport protein 56
1.182	1.049	1.106	1.038	0.836	0.964							Q9NWB7	IFT57	Intraflagellar transport protein 57 homolog
1.058	1.008	1.052	1.089	0.932	0.984	1.050	1.159	1.001	1.066	1.076	1.087	Q96LB3	IFT74	Intraflagellar transport protein 74 homolog

Abundance Ratio														
1 d M1/C1	1 d M2/C2	1 d M3/C3	4 d M1/C1	4 d M2/C2	4 d M3/C3	7 d M1/C1	7 d M2/C2	7 d M3/C3	10 d M1/C1	10 d M2/C2	10 d M3/C3	Accession ID	Gene name	Protein Description
0.908	0.932	0.945	0.980	0.902	1.080	1.077	0.853	0.989	1.036	0.876	1.168	Q8WYA0	IFT81	Intraflagellar transport protein 81 homolog
1.100	1.087	1.014	1.081	1.016	0.887	1.145	1.052	0.933	1.052	0.957	0.994	Q13099	IFT88	Intraflagellar transport protein 88 homolog
1.013	1.026	1.009	0.990	0.968	1.014	0.980	1.067	1.012	0.957	0.986	1.006	O60306	AQR	Intron-binding protein aquarius
1.116	0.826	0.929	1.105	0.968	1.054	0.956	1.123	0.932	1.057	1.142	0.976	Q8N2Y8	RUSC2	Iporin
						0.947	1.044	1.041				Q15051	IQCB1	IQ calmodulin-binding motif-containing protein 1
1.075	1.031	1.060	1.054	0.831	0.990	0.996	0.986	0.948	0.948	0.880	1.066	Q96DY2	IQCD	IQ domain-containing protein D
1.015	1.028	1.017	0.998	0.927	1.028	1.031	1.022	0.912	0.959	0.998	1.070	A0A087WWK8	IQSEC1	IQ motif and SEC7 domain-containing protein 1
0.706	0.818	0.833	0.974	0.646	0.890	1.053	1.099	1.340	0.888	1.084	0.898	A0A0A6YY96	IREB2	Iron-responsive element-binding protein 2
0.979	0.964	1.034	1.045	1.020	1.093	1.062	1.144	1.111	1.051	1.030	1.122	Q86U28	ISCA2	Iron-sulfur cluster assembly 2 homolog, mitochondrial
0.966	0.943	1.018	0.995	0.956	1.031	1.050	0.972	1.029	1.073	1.044	1.033	Q9H1K1	ISCU	Iron-sulfur cluster assembly enzyme ISCU, mitochondrial
1.057	1.001	1.058	1.017	0.893	1.058	1.017	1.043	1.020	1.026	0.994	1.048	Q8IWL3	HSCB	Iron-sulfur cluster co-chaperone protein HscB, mitochondrial
1.008	0.989	1.026	0.995	1.007	1.002	0.965	1.068	0.998	0.976	0.966	1.004	Q8TB37	NUBPL	Iron-sulfur protein NUBPL
1.047	1.021	0.996	1.051	1.006	1.002	1.037	1.084	0.949	1.021	1.010	0.987	Q2TAA2	IAH1	Isoamyl acetate-hydrolyzing esterase 1 homolog
0.993	1.002	0.997	1.051	1.080	0.928	1.013	1.063	1.008	1.002	0.947	0.994	Q7L266	ASRGL1	Isoaspartyl peptidase/L-asparaginase
0.949	0.994	0.953	0.989	0.993	1.007	1.036	0.994	0.968	1.041	0.945	1.037	Q9UKU7	ACAD8	Isobutyryl-CoA dehydrogenase, mitochondrial
0.992	1.024	0.996	1.011	0.992	1.008	1.004	1.064	1.026	0.951	1.000	0.963	Q96CN7	ISOC1	Isochorismatase domain-containing protein 1
1.030	1.049	1.008	1.003	1.049	1.005	0.985	1.124	1.059	0.929	1.038	0.955	Q96AB3	ISOC2	Isochorismatase domain-containing protein 2
0.970	0.987	0.981	0.991	0.989	1.032	0.990	0.980	0.993	1.022	1.002	0.993	P50213	IDH3A	Isocitrate dehydrogenase [NAD] subunit alpha, mitochondrial
1.025	1.003	1.074	0.965	1.061	1.049	0.928	0.970	0.996	0.929	1.043	1.003	P51553	IDH3G	Isocitrate dehydrogenase [NAD] subunit gamma, mitochondrial
0.971	0.985	0.984	1.017	1.041	0.994	0.992	1.015	0.993	1.003	0.971	0.991	A0A087WZN1	IDH3B	Isocitrate dehydrogenase [NAD] subunit, mitochondrial
0.975	0.976	1.011	0.979	1.045	0.998	0.966	0.907	0.984	1.028	1.008	1.026	O75874	IDH1	Isocitrate dehydrogenase [NADP] cytoplasmic
1.034	0.979	1.008	1.003	1.167	1.020	1.023	0.979	1.035	0.947	0.977	0.977	P48735	IDH2	Isocitrate dehydrogenase [NADP], mitochondrial
0.968	1.063	1.044	0.951	1.022	1.103	1.061	1.118	1.110	1.073	0.939	1.082	P55196-2	AFDN	Isoform 1 of Afadin
1.033	1.001	0.992	0.983	0.958	1.046	0.982	1.078	1.082	1.000	1.040	1.032	Q9ULH1-2	ASAP1	Isoform 1 of Arf-GAP with SH3 domain, ANK repeat and PH domain-containing protein 1
0.847	0.777	0.986	0.908	0.878	1.194	0.846	0.832	1.041	1.303	0.995	1.278	Q9UI08-2	EVL	Isoform 1 of Ena/VASP-like protein
1.028	1.007	1.076	0.809	1.024	0.914	1.140	1.003	1.021	1.109	0.774	1.205	Q9BXW9-1	FANCD2	Isoform 1 of Fanconi anemia group D2 protein
			0.966	1.167	1.067	0.946	0.898	0.929	1.140	0.954	1.290	O43189-2	PHF1	Isoform 1 of PHD finger protein 1
0.978	1.023	1.018	0.987	0.979	1.026	0.995	0.988	1.017	1.042	1.011	1.017	Q99959-2	PKP2	Isoform 1 of Plakophilin-2
0.909	0.922	1.098	0.902	0.903	1.023	0.899	1.034	1.304	0.985	0.926	1.090	Q9P2E9-2	RRBP1	Isoform 1 of Ribosome-binding protein 1

Abundance Ratio														
1 d M1/C1	1 d M2/C2	1 d M3/C3	4 d M1/C1	4 d M2/C2	4 d M3/C3	7 d M1/C1	7 d M2/C2	7 d M3/C3	10 d M1/C1	10 d M2/C2	10 d M3/C3	Accession ID	Gene name	Protein Description
1.051	0.973	1.095	0.969	0.940	1.018	0.960	0.961	0.981	0.987	0.976	1.024	Q6P6C2-1	ALKBH5	Isoform 1 of RNA demethylase ALKBH5
0.931	0.994	0.990	0.920	0.925	0.988	0.948	1.007	0.900	0.932	0.962	0.956	Q96A57-2	TMEM230	Isoform 1 of Transmembrane protein 230
1.016	0.991	0.986	0.999	0.987	0.939	0.988	0.962	0.994	1.022	1.033	0.992	P45880-1	VDAC2	Isoform 1 of Voltage-dependent anion-selective channel protein 2
0.988	1.001	1.016	0.956	0.968	0.997	1.037	1.009	0.964	0.955	0.934	0.984	P09493-10	TPM1	Isoform 10 of Tropomyosin alpha-1 chain
1.057	1.108	1.191				0.822	1.150	1.003	1.006	0.713	0.801	Q9BX66-12	SORBS1	Isoform 12 of Sorbin and SH3 domain-containing protein 1
1.005	1.041	1.091	1.017	0.940	0.997	1.050	1.113	1.065	1.022	1.010	1.095	O75923-13	DYSF	Isoform 13 of Dysferlin
1.085	1.071	1.024	0.946	0.976	0.789	1.058	0.993	1.098	0.786	0.917	0.778	Q9ULU4-13	ZMYND8	Isoform 13 of Protein kinase C-binding protein 1
0.817	0.983	1.067	0.941	0.993	1.088	0.967	0.953	0.997				Q96AX9-14	MIB2	Isoform 14 of E3 ubiquitin-protein ligase MIB2
1.037	1.013	1.022	0.992	0.967	0.963	1.025	1.107	0.982	0.953	1.003	0.961	Q9ULU4-19	ZMYND8	Isoform 19 of Protein kinase C-binding protein 1
1.072	1.037	1.092	1.025	0.953	1.016	1.008	1.043	1.092	1.046	1.005	0.971	O43318-2	MAP3K7	Isoform 1A of Mitogen-activated protein kinase kinase kinase 7
0.985	1.016	1.004	0.999	1.002	1.011	1.016	0.960	1.009	1.021	0.974	1.033	O60716-3	CTNND1	Isoform 1AC of Catenin delta-1
1.054	1.080	1.010	0.997	0.968	1.006	0.937	0.928	0.982	1.004	0.960	0.965	Q15120-2	PDK3	Isoform 2 of [Pyruvate dehydrogenase (acetyl-transferring)] kinase isozyme 3, mitochondrial
1.029	1.014	1.017	1.004	0.995	0.997	0.970	1.025	1.012	0.989	0.949	0.978	Q9P0J1-2	PDP1	Isoform 2 of [Pyruvate dehydrogenase [acetyl-transferring]]-phosphatase 1, mitochondrial
0.970	0.902	0.980	0.980	0.951	0.965	0.949	1.006	0.944	0.999	0.977	0.993	P51178-2	PLCD1	Isoform 2 of 1-phosphatidylinositol 4,5-bisphosphate phosphodiesterase delta-1
0.967	0.983	1.006	1.017	0.967	1.018	0.984	1.010	1.038	0.996	0.985	1.026	P19174-2	PLCG1	Isoform 2 of 1-phosphatidylinositol 4,5-bisphosphate phosphodiesterase gamma-1
1.009	0.991	0.991	0.986	0.971	0.987	0.986	0.972	0.974	1.031	0.973	0.999	Q92552-2	MRPS27	Isoform 2 of 28S ribosomal protein S27, mitochondrial
0.993	0.998	0.993	1.015	0.936	0.996	0.983	0.985	1.000	1.025	1.025	1.016	O75600-2	GCAT	Isoform 2 of 2-amino-3-ketobutyrate coenzyme A ligase, mitochondrial
1.050	0.966	1.053	1.018	0.949	1.045	1.039	0.976	0.992	1.217	1.054	1.002	Q6PK18-2	OGFOD3	Isoform 2 of 2-oxoglutarate and iron-dependent oxygenase domain-containing protein 3
0.996	1.020	0.934	0.989	0.952	0.985	0.964	1.005	0.985	0.968	0.990	0.915	Q7Z7H8-2	MRPL10	Isoform 2 of 39S ribosomal protein L10, mitochondrial
0.998	1.009	0.989	0.941	0.977	1.001	0.962	0.935	0.940	1.038	1.020	1.045	Q8TCC3-2	MRPL30	Isoform 2 of 39S ribosomal protein L30, mitochondrial
1.061	1.017	1.078	1.090	1.104	1.131	1.045	0.913	1.060	1.089	1.030	1.040	Q7Z7F7-2	MRPL55	Isoform 2 of 39S ribosomal protein L55, mitochondrial
0.989	0.976	0.990	0.982	0.972	1.004	0.983	1.008	1.011	1.029	0.944	1.025	P25325-2	MPST	Isoform 2 of 3-mercaptopyruvate sulfurtransferase
1.409	1.015	1.086	1.164	1.862	0.951	1.029	0.771	1.006	0.870	0.988	0.805	P62273-2	RPS29	Isoform 2 of 40S ribosomal protein S29
0.968	1.009	0.976	1.019	1.007	1.015	1.024	1.016	0.994	1.034	0.996	1.020	Q13131-2	PRKAA1	Isoform 2 of 5'-AMP-activated protein kinase catalytic subunit alpha-1

Abundance Ratio														
1 d M1/C1	1 d M2/C2	1 d M3/C3	4 d M1/C1	4 d M2/C2	4 d M3/C3	7 d M1/C1	7 d M2/C2	7 d M3/C3	10 d M1/C1	10 d M2/C2	10 d M3/C3	Accession ID	Gene name	Protein Description
1.049	1.027	1.024	0.975	0.950	1.005	0.986	1.118	0.961	1.046	1.042	0.913	Q9H857-2	NT5DC2	Isoform 2 of 5'-nucleotidase domain-containing protein 2
0.977	1.010	0.990	0.993	0.977	1.000	1.046	1.083	1.034	1.109	1.076	1.060	Q9NR19-2	ACSS2	Isoform 2 of Acetyl-coenzyme A synthetase, cytoplasmic
0.994	0.964	0.986	0.981	0.949	1.005	1.027	0.997	0.968	1.027	0.969	0.951	Q13510-2	ASAH1	Isoform 2 of Acid ceramidase
1.027	1.015	1.018	0.978	1.010	1.001	0.958	0.956	0.930	0.928	0.951	0.957	Q8N556-2	AFAP1	Isoform 2 of Actin filament-associated protein 1
1.001	0.997	1.005	0.960	0.963	0.947	0.977	1.077	1.011	0.938	0.967	0.960	Q8N9N2-2	ASCC1	Isoform 2 of Activating signal cointegrator 1 complex subunit 1
1.036	0.998	0.997	0.977	0.962	0.945	0.964	1.006	0.933	1.024	0.970	0.983	Q96CM8-2	ACSF2	Isoform 2 of Acyl-CoA synthetase family member 2, mitochondrial
0.996	0.970	0.996	1.023	1.001	1.057	1.027	1.063	1.078	1.039	1.005	1.044	Q96HN2-2	AHCYL2	Isoform 2 of Adenosylhomocysteinase 3
1.021	1.011	1.016	1.002	1.094	0.988	0.985	0.962	1.021	0.993	0.995	0.978	Q01518-2	CAP1	Isoform 2 of Adenylyl cyclase-associated protein 1
1.078	0.986	0.919	0.928	0.887	1.034	1.038	1.069	1.012	1.103	0.981	1.083	P56559-2	ARL4C	Isoform 2 of ADP-ribosylation factor-like protein 4C
1.033	0.960	1.031	0.971	0.920	1.049	0.993	0.910	1.010	1.133	1.009	1.102	Q66PJ3-2	ARL6IP4	Isoform 2 of ADP-ribosylation factor-like protein 6-interacting protein 4
1.017	0.888	1.001										P51825-2	AFF1	Isoform 2 of AF4/FMR2 family member 1
0.995	0.920	0.994	0.965	0.872	1.045	1.055	0.926	1.031	0.979	0.936	1.092	Q12802-2	AKAP13	Isoform 2 of A-kinase anchor protein 13
1.045	1.003	0.991	0.995	0.904	1.007	1.022	1.230	1.004	0.841	0.830	0.986	Q9H8T0-2	AKTIP	Isoform 2 of AKT-interacting protein
0.793	1.007	0.915	0.987	1.078	1.037	0.974	0.912	0.940	1.177	0.738	0.981	C9JRZ8-2	AKR1B15	Isoform 2 of Aldo-keto reductase family 1 member B15
									1.111	0.802	1.011	Q9BQI0-2	AIF1L	Isoform 2 of Allograft inflammatory factor 1-like
1.098	1.052	1.006	1.166	0.835	0.999	1.040	1.111	1.117	1.064	0.904	1.070	P35609-2	ACTN2	Isoform 2 of Alpha-actinin-2
1.014	0.997	0.976	0.997	1.015	0.966	0.995	0.993	0.985	0.985	0.980	0.962	P49419-2	ALDH7A1	Isoform 2 of Alpha-aminoadipic semialdehyde dehydrogenase
1.058	0.957	0.878										Q3KRA9-2	ALKBH6	Isoform 2 of Alpha-ketoglutarate-dependent dioxygenase alkB homolog 6
1.008	0.998	0.994	0.982	1.033	0.985	0.985	0.982	0.990	1.001	1.013	1.017	Q12904-2	AIMP1	Isoform 2 of Aminoacyl tRNA synthase complex-interacting multifunctional protein 1
1.050	0.868	1.033	0.952	0.916	1.001	1.058	0.987	1.038	1.012	0.993	1.101	Q96FJ0-2	STAMBPL1	Isoform 2 of AMSH-like protease
1.027	1.006	0.991	1.008	0.975	0.954	0.985	1.012	0.969	0.980	1.003	0.941	P07355-2	ANXA2	Isoform 2 of Annexin A2
						0.992	1.216	0.953				P09525-2	ANXA4	Isoform 2 of Annexin A4
0.983	0.993	0.994	0.971	0.918	0.981	0.990	1.020	0.995	1.031	1.016	1.005	Q4KMQ2-2	ANO6	Isoform 2 of Anoctamin-6
1.050	1.008	1.022	1.031	1.125	1.004	1.016	0.981	1.033	0.994	0.982	0.995	O43747-2	AP1G1	Isoform 2 of AP-1 complex subunit gamma-1
1.034	0.956	0.971	0.933	0.803	1.085	1.000	1.113	0.991	0.909	1.041	0.930	Q96PC3-2	AP1S3	Isoform 2 of AP-1 complex subunit sigma-3
0.979	1.007	0.986	0.991	0.988	0.978	0.972	1.012	1.008	0.986	0.982	0.982	P63010-2	AP2B1	Isoform 2 of AP-2 complex subunit beta
1.035	0.958	0.950	0.919	0.931	0.891				1.050	0.798	0.924	O14791-2	APOL1	Isoform 2 of Apolipoprotein L1

Abundance Ratio														
1 d M1/C1	1 d M2/C2	1 d M3/C3	4 d M1/C1	4 d M2/C2	4 d M3/C3	7 d M1/C1	7 d M2/C2	7 d M3/C3	10 d M1/C1	10 d M2/C2	10 d M3/C3	Accession ID	Gene name	Protein Description
1.060	1.028	0.935	1.039	1.014	0.910	1.042	1.111	0.938	1.088	1.074	0.933	Q9BZZ5-2	API5	Isoform 2 of Apoptosis inhibitor 5
			0.821	0.813	1.387	1.040	1.045	0.938				Q8N2F6-2	ARMC10	Isoform 2 of Armadillo repeat-containing protein 10
0.983	0.988	1.009	1.011	0.981	1.013	1.007	1.065	1.023	1.000	1.008	1.018	Q15121-2	PEA15	Isoform 2 of Astrocytic phosphoprotein PEA-15
0.975	0.883	0.951	1.073	1.116	0.921				0.918	0.979	0.882	Q14CW9-2	ATXN7L3	Isoform 2 of Ataxin-7-like protein 3
0.896	0.985	1.082	0.797	0.817	0.934	0.744	0.603	0.910	1.093	1.099	1.056	Q06055-2	ATP5G2	Isoform 2 of ATP synthase F(0) complex subunit C2, mitochondrial
0.978	1.015	1.004	1.041	0.951	0.919	0.979	0.985	0.961	0.977	1.003	0.930	P18859-2	ATP5J	Isoform 2 of ATP synthase-coupling factor 6, mitochondrial
0.971	0.994	1.010	0.979	0.976	1.004	0.982	0.942	0.942	1.012	1.019	1.033	Q9NVI7-2	ATAD3A	Isoform 2 of ATPase family AAA domain-containing protein 3A
1.010	0.992	1.040	1.009	0.876	1.020	1.006	1.023	1.014	1.087	0.928	0.996	O75027-2	ABCB7	Isoform 2 of ATP-binding cassette sub-family B member 7, mitochondrial
0.974	1.013	0.988	1.019	1.039	1.014	1.012	0.999	1.005	1.015	1.057	1.013	Q9UG63-2	ABCF2	Isoform 2 of ATP-binding cassette sub-family F member 2
			1.073	0.878	1.041				0.922	1.005	0.880	P53396-2	ACLY	Isoform 2 of ATP-citrate synthase
0.984	0.904	0.926	1.066	1.138	1.060	1.110	0.948	1.074	0.661	1.028	1.201	Q2TAZ0-3	ATG2A	Isoform 2 of Autophagy-related protein 2 homolog A
1.014	0.958	1.055	0.982	0.907	1.095	0.952	0.960	0.953	1.013	0.958	1.130	Q9UL15-2	BAG5	Isoform 2 of BAG family molecular chaperone regulator 5
1.058	1.040	1.000	0.949	0.966	0.957	1.037	1.046	0.964	1.024	1.008	1.057	Q9H4G0-2	EPB41L1	Isoform 2 of Band 4.1-like protein 1
0.773	0.688	1.706	1.094	0.889	0.885				0.949	1.083	0.847	Q96RK4-2	BBS4	Isoform 2 of Bardet-Biedl syndrome 4 protein
1.047	1.044	1.082	1.030	0.941	1.014	1.053	1.070	1.051	1.047	1.085	1.076	P55061-2	TMBIM6	Isoform 2 of Bax inhibitor 1
0.993	1.005	0.912	0.891	0.990	1.023	1.013	1.112	1.021	1.029	1.025	1.088	Q4VC05-2	BCL7A	Isoform 2 of B-cell CLL/lymphoma 7 protein family member A
0.916	0.976	1.039	0.960	0.943	0.989	0.956	0.885	0.954	1.030	1.054	1.006	P51572-2	BCAP31	Isoform 2 of B-cell receptor-associated protein 31
1.046	1.017	1.055	0.977	0.987	1.050	0.982	1.074	0.984	1.013	0.939	1.056	P55957-2	BID	Isoform 2 of BH3-interacting domain death agonist
0.962	1.007	0.986	0.980	0.986	1.071	1.030	1.106	1.032	1.066	1.029	1.034	Q13057-2	COASY	Isoform 2 of Bifunctional coenzyme A synthase
1.028	1.042	1.017	0.994	0.958	0.992	1.009	1.058	0.999	1.015	0.988	1.000	Q9Y223-2	GNE	Isoform 2 of Bifunctional UDP-N-acetylglucosamine 2-epimerase/N-acetylmannosamine kinase
1.035	1.020	1.021	0.995	0.987	0.950	1.033	1.090	1.012	1.104	0.946	1.045	P43251-2	BTD	Isoform 2 of Biotinidase
0.946	1.080	0.945	0.873	0.855	0.996	1.025	1.085	1.199	0.960	0.998	1.094	Q6UWU4-2	C6orf89	Isoform 2 of Bombesin receptor-activated protein C6orf89
			1.000	0.900	1.046	0.937	1.183	0.976	0.925	0.923	1.038	Q9P287-2	BCCIP	Isoform 2 of BRCA2 and CDKN1A-interacting protein
1.093	0.994	0.995	1.010	0.931	0.981	1.073	1.063	1.034	0.985	0.979	0.971	O95696-2	BRD1	Isoform 2 of Bromodomain-containing protein 1

Abundance Ratio														
1 d M1/C1	1 d M2/C2	1 d M3/C3	4 d M1/C1	4 d M2/C2	4 d M3/C3	7 d M1/C1	7 d M2/C2	7 d M3/C3	10 d M1/C1	10 d M2/C2	10 d M3/C3	Accession ID	Gene name	Protein Description
1.031	0.974	0.991	0.948	0.942	1.007	1.013	0.952	0.985	0.963	1.106	1.063	Q9NPI1-2	BRD7	Isoform 2 of Bromodomain-containing protein 7
0.976	1.008	1.026	1.009	0.905	0.983	1.031	1.007	1.084	1.068	0.988	1.108	Q9H0E9-2	BRD8	Isoform 2 of Bromodomain-containing protein 8
0.815	0.808	1.365										Q9BSF8-2	BTBD10	Isoform 2 of BTB/POZ domain-containing protein 10
1.018	1.047	0.987	0.986	0.962	1.019	0.972	0.909	0.917	1.002	0.993	1.076	Q7KYR7-1	BTN2A1	Isoform 2 of Butyrophilin subfamily 2 member A1
1.101	0.968	1.011	0.995	1.044	1.036	0.994	1.123	1.134	1.021	1.091	1.018	Q8WUQ7-2	CACTIN	Isoform 2 of Cactin
			1.397	1.196	0.879	0.965	0.944	1.059				Q99828-2	CIB1	Isoform 2 of Calcium and integrin-binding protein 1
0.992	1.009	1.005	1.005	0.955	0.980	0.995	1.063	0.993	0.982	0.981	0.959	Q9UJS0-2	SLC25A13	Isoform 2 of Calcium-binding mitochondrial carrier protein Aralar2
1.073	0.972	1.054	0.995	0.969	1.066	0.971	0.954	0.977	0.976	0.900	1.140	Q9P1Y5-2	CAMSAP3	Isoform 2 of Calmodulin-regulated spectrin-associated protein 3
0.922	1.007	0.973	0.989	0.936	0.973	1.006	1.010	0.962	1.052	1.051	1.054	P27824-2	CANX	Isoform 2 of Calnexin
0.994	1.017	1.079	0.961	0.918	1.008	0.951	1.041	0.943	1.000	1.003	1.005	Q9UDT6-2	CLIP2	Isoform 2 of CAP-Gly domain-containing linker protein 2
1.016	1.050	1.037	1.093	0.953	1.105	1.054	1.164	1.230	1.107	1.100	1.128	Q9Y6M4-2	CSNK1G3	Isoform 2 of Casein kinase I isoform gamma-3
0.980	0.972	1.017	0.980	0.930	1.033	0.982	0.978	1.000	1.004	0.994	1.048	Q9H078-2	CLPB	Isoform 2 of Caseinolytic peptidase B protein homolog
1.000	1.020	1.035	0.998	0.971	1.048	1.023	1.005	1.052	1.024	0.958	1.049	Q96LW7-2	CARD19	Isoform 2 of Caspase recruitment domain-containing protein 19
0.985	1.019	0.989	0.993	1.000	1.056	0.993	1.047	1.017	1.000	1.019	1.033	O43310-2	CTIF	Isoform 2 of CBP80/20-dependent translation initiation factor
0.967	0.998	0.991	0.980	0.972	1.001	0.972	0.986	1.006	1.029	1.000	1.037	Q15642-2	TRIP10	Isoform 2 of Cdc42-interacting protein 4
0.998	1.009	0.987	0.986	0.943	1.006	1.024	1.025	1.017	1.028	1.007	1.119	P30260-2	CDC27	Isoform 2 of Cell division cycle protein 27 homolog
						0.933	0.835	0.947				Q5SW79-2	CEP170	Isoform 2 of Centrosomal protein of 170 kDa
1.016	0.825	0.688	1.086	1.311	1.130	1.114	1.323	1.280				Q5JTW2-2	CEP78	Isoform 2 of Centrosomal protein of 78 kDa
1.039	1.005	1.035	1.047	0.885	0.975	0.968	1.099	1.035	1.025	0.933	1.134	Q8IW35-2	CEP97	Isoform 2 of Centrosomal protein of 97 kDa
0.959	1.022	1.053	1.004	0.898	1.006	1.053	1.077	1.013	1.034	1.077	0.988	Q6ZMG9-2	CERS6	Isoform 2 of Ceramide synthase 6
1.031	0.977	0.977	0.965	1.101	1.084	1.038	1.107	1.075	1.055	1.044	1.064	Q9BY43-2	CHMP4A	Isoform 2 of Charged multivesicular body protein 4a
0.944	0.977	1.017	1.154	0.752	0.934	1.126	0.896	1.029	1.031	1.103	1.031	Q8WWI5-2	SLC44A1	Isoform 2 of Choline transporter-like protein 1
1.000	0.973	1.054	1.093	0.982	0.981	0.979	1.036	1.132	0.999	1.063	0.963	Q8IWA5-2	SLC44A2	Isoform 2 of Choline transporter-like protein 2
0.901	1.042	0.957	0.980	0.967	0.952	0.894	0.976	0.960	0.951	1.152	0.911	Q9Y3Y2-3	CHTOP	Isoform 2 of Chromatin target of PRMT1 protein
0.992	1.006	1.004	1.000	0.979	1.013	1.039	0.997	1.023	0.994	0.995	1.029	Q14839-2	CHD4	Isoform 2 of Chromodomain-helicase-DNA-binding protein 4
1.015	1.058	1.040	0.998	0.940	1.070	1.021	1.041	1.022	0.944	0.989	1.023	Q8WVB6-2	CHTF18	Isoform 2 of Chromosome transmission fidelity protein 18 homolog

Abundance Ratio														
1 d M1/C1	1 d M2/C2	1 d M3/C3	4 d M1/C1	4 d M2/C2	4 d M3/C3	7 d M1/C1	7 d M2/C2	7 d M3/C3	10 d M1/C1	10 d M2/C2	10 d M3/C3	Accession ID	Gene name	Protein Description
1.093	1.004	1.035	1.039	0.971	1.003	0.971	0.970	1.019	1.017	1.053	1.010	Q9ULV3-2	CIZ1	Isoform 2 of Cip1-interacting zinc finger protein
0.963	0.992	0.978	0.974	1.051	0.996	0.985	0.887	0.976	1.018	1.010	1.033	Q16630-2	CPSF6	Isoform 2 of Cleavage and polyadenylation specificity factor subunit 6
1.027	1.057	1.107	0.984	0.917	1.109	1.063	1.047	1.021	1.177	1.046	1.114	P10909-2	CLU	Isoform 2 of Clusterin
0.997	0.998	0.997	0.986	0.918	1.013	0.991	1.004	1.014	0.947	0.963	0.981	Q8N3U4-2	STAG2	Isoform 2 of Cohesin subunit SA-2
						1.455	0.708	1.005				Q8TD31-2	CCHCR1	Isoform 2 of Coiled-coil alpha-helical rod protein 1
1.009	0.975	1.035	0.993	0.961	1.010	0.987	0.994	1.013	1.037	1.037	1.058	Q5T0F9-2	CC2D1B	Isoform 2 of Coiled-coil and C2 domain-containing protein 1B
1.092	0.803	1.519										Q6ZP82-2	CCDC141	Isoform 2 of Coiled-coil domain-containing protein 141
1.001	0.963	1.057	0.984	0.985	1.021	0.960	0.837	0.975	1.023	0.990	1.089	Q8IVM0-2	CCDC50	Isoform 2 of Coiled-coil domain-containing protein 50
									1.159	0.943	1.214	Q9BSY4-2	CHCHD5	Isoform 2 of Coiled-coil-helix-coiled-coil-helix domain-containing protein 5
1.002	0.959	1.029	0.994	0.900	0.985	1.121	1.025	0.963	0.818	0.846	1.093	Q86XI2-2	NCAPG2	Isoform 2 of Condensin-2 complex subunit G2
1.002	1.021	1.041	0.983	0.955	1.025	0.959	1.029	0.998	0.969	0.999	1.057	Q9UP83-2	COG5	Isoform 2 of Conserved oligomeric Golgi complex subunit 5
0.929	0.995	1.023	0.923	0.981	1.095	0.965	0.904	1.021	1.014	1.005	1.063	Q13098-7	GPS1	Isoform 2 of COP9 signalosome complex subunit 1
1.010	1.067	1.043										Q04656-3	ATP7A	Isoform 2 of Copper-transporting ATPase 1
0.992	0.967	0.935	0.957	1.007	0.976	1.011	0.891	0.922	1.043	0.991	1.121	Q13951-2	CBFB	Isoform 2 of Core-binding factor subunit beta
0.987	0.899	1.137	1.025	0.976	0.988	1.082	0.865	0.978	0.901	0.925	0.883	P12532-2	CKMT1A	Isoform 2 of Creatine kinase U-type, mitochondrial
1.036	1.028	0.974	0.932	0.949	0.997	1.024	1.029	1.018	1.032	1.061	0.994	Q6UUV9-2	CRTC1	Isoform 2 of CREB-regulated transcription coactivator 1
0.968	1.066	1.067										Q96AY2-2	EME1	Isoform 2 of Crossover junction endonuclease EME1
0.983	1.001	1.001	0.988	0.996	1.028	0.962	0.997	1.009	0.989	1.026	1.059	P56545-2	CTBP2	Isoform 2 of C-terminal-binding protein 2
0.983	1.014	0.996	1.010	1.074	0.985	1.013	0.994	0.987	0.994	1.016	0.997	Q13617-2	CUL2	Isoform 2 of Cullin-2
0.987	0.977	1.111	1.002	0.951	0.986	1.052	1.147	1.030	0.925	1.075	1.045	Q14999-2	CUL7	Isoform 2 of Cullin-7
1.101	1.066	0.944	1.006	0.936	0.913	1.099	1.063	0.975	1.143	1.053	1.098	Q9P0U4-2	CXXC1	Isoform 2 of CXXC-type zinc finger protein 1
0.998	1.013	0.942	1.022	0.964	1.058	0.990	1.071	1.039	1.030	1.031	1.072	Q00536-2	CDK16	Isoform 2 of Cyclin-dependent kinase 16
1.003	1.000	1.013	0.972	0.961	1.033	1.039	1.053	1.019	1.013	1.005	1.073	P50750-2	CDK9	Isoform 2 of Cyclin-dependent kinase 9
1.063	0.979	1.032	0.943	0.949	0.994	0.891	0.974	1.070	1.005	0.974	0.958	O60583-2	CCNT2	Isoform 2 of Cyclin-T2
0.941	0.980	1.003	1.026	0.965	1.036	1.067	0.962	1.013	1.080	0.995	1.142	P35520-2	CBS	Isoform 2 of Cystathionine beta-synthase
1.055	1.022	0.962	0.985	0.952	1.009	0.995	1.062	1.031	0.977	1.011	0.983	Q9Y4P1-2	ATG4B	Isoform 2 of Cysteine protease ATG4B

Abundance Ratio												Accession ID	Gene name	Protein Description
1 d M1/C1	1 d M2/C2	1 d M3/C3	4 d M1/C1	4 d M2/C2	4 d M3/C3	7 d M1/C1	7 d M2/C2	7 d M3/C3	10 d M1/C1	10 d M2/C2	10 d M3/C3			
0.992	0.980	0.987	0.976	0.854	1.129	0.938	0.889	1.018	1.083	1.084	1.009	Q9NYJ1-2	COA4	Isoform 2 of Cytochrome c oxidase assembly factor 4 homolog, mitochondrial
1.006	0.969	1.010	1.011	0.949	0.946	1.008	0.969	0.985	1.048	1.043	1.023	Q5JTJ3-2	COA6	Isoform 2 of Cytochrome c oxidase assembly factor 6 homolog
1.014	1.026	1.024	1.058	1.071	0.963	1.058	1.020	1.002	1.070	1.035	1.037	Q5RI15-2	COX20	Isoform 2 of Cytochrome c oxidase protein 20 homolog
1.018	0.994	1.041	0.991	0.931	1.010	1.002	1.060	1.028	0.978	0.958	1.022	Q8NCM8-2	DYNC2H1	Isoform 2 of Cytoplasmic dynein 2 heavy chain 1
1.165	0.843	1.047	0.735	0.934	1.058	0.860	1.253	1.166	0.930	0.984	1.128	Q96F07-2	CYFIP2	Isoform 2 of Cytoplasmic FMR1-interacting protein 2
0.900	0.987	1.164										Q15038-2	DAZAP2	Isoform 2 of DAZ-associated protein 2
1.010	0.805	0.741	1.019	0.862	3.006				0.898	1.021	1.054	Q86SQ9-2	DHDDS	Isoform 2 of Dehydrodolichyl diphosphate synthase complex subunit DHDDS
1.004	1.030	0.972	1.002	0.981	0.961	0.962	1.006	1.004	1.019	1.051	0.978	P32321-2	DCTD	Isoform 2 of Deoxycytidylate deaminase
1.000	1.020	1.020	0.990	0.982	1.022	0.990	1.093	1.022	0.959	0.999	1.018	Q86TI2-2	DPP9	Isoform 2 of Dipeptidyl peptidase 9
			1.034	0.934	1.124							Q9NZJ9-2	NUDT4	Isoform 2 of Diphosphoinositol polyphosphate phosphohydrolase 2
1.034	1.034	1.026	1.079	1.041	1.070	1.211	1.184	1.109	1.123	1.073	1.180	Q96PD2-2	DCBLD2	Isoform 2 of Discoidin, CUB and LCCL domain-containing protein 2
0.985	1.000	1.026	0.978	0.954	0.947	0.935	0.828	0.989	0.977	1.027	1.058	Q12959-2	DLG1	Isoform 2 of Disks large homolog 1
1.005	0.915	0.905	0.808	0.920	1.024	1.148	1.054	1.280				Q16531-2	DDB1	Isoform 2 of DNA damage-binding protein 1
0.988	0.972	1.019	0.983	0.987	1.007	0.985	0.866	1.000	1.013	1.001	1.037	Q92878-2	RAD50	Isoform 2 of DNA repair protein RAD50
0.986	0.996	0.996	0.986	1.028	1.001	1.043	1.020	1.037	0.986	0.985	1.044	P25205-2	MCM3	Isoform 2 of DNA replication licensing factor MCM3
1.100	0.992	1.026	0.999	1.036	0.968	1.015	1.062	1.097	0.989	1.024	1.043	O15446-2	CD3EAP	Isoform 2 of DNA-directed RNA polymerase I subunit RPA34
1.001	1.008	1.007	0.977	0.963	1.041	1.015	1.013	0.993	0.988	1.037	1.008	Q9GZS1-2	POLR1E	Isoform 2 of DNA-directed RNA polymerase I subunit RPA49
1.158	1.085	1.012	1.039	0.917	1.167							Q9Y2S0-2	POLR1D	Isoform 2 of DNA-directed RNA polymerases I and III subunit RPAC2
0.903	0.984	0.911	0.928	0.850	1.329	1.049	0.881	0.992	1.201	1.103	1.096	Q9P2X0-2	DPM3	Isoform 2 of Dolichol-phosphate mannosyltransferase subunit 3
0.933	0.977	0.994	0.977	0.946	0.999	0.951	1.057	1.013	0.986	1.030	0.966	P04844-2	RPN2	Isoform 2 of Dolichyl-diphosphooligosaccharide--protein glycosyltransferase subunit 2
1.003	1.065	1.033	1.048	1.011	0.988	1.101	0.777	1.150	1.087	1.040	1.272	Q9UJU6-2	DBNL	Isoform 2 of Drebrin-like protein
0.984	1.040	1.016	0.989	0.959	1.016	1.006	1.080	0.995	0.979	0.976	1.020	P46734-3	MAP2K3	Isoform 2 of Dual specificity mitogen-activated protein kinase kinase 3
1.018	1.024	1.022	0.977	0.976	1.037	0.994	1.092	1.048	1.018	1.015	1.044	P45985-2	MAP2K4	Isoform 2 of Dual specificity mitogen-activated protein kinase kinase 4

Abundance Ratio												Accession ID	Gene name	Protein Description
1 d M1/C1	1 d M2/C2	1 d M3/C3	4 d M1/C1	4 d M2/C2	4 d M3/C3	7 d M1/C1	7 d M2/C2	7 d M3/C3	10 d M1/C1	10 d M2/C2	10 d M3/C3			
1.056	0.944	1.053	0.798	1.091	1.146	1.038	1.330	0.903	1.285	0.898	1.010	Q9NRW4-2	DUSP22	Isoform 2 of Dual specificity protein phosphatase 22
0.989	1.006	0.997	0.984	0.960	0.995	1.013	1.053	0.998	1.003	0.973	1.014	Q13561-2	DCTN2	Isoform 2 of Dynactin subunit 2
0.994	0.980	1.030	1.009	0.984	1.007	1.004	1.015	1.039	1.012	0.999	1.040	O60313-2	OPA1	Isoform 2 of Dynamin-like 120 kDa protein, mitochondrial
									0.882	1.060	0.891	O75925-2	PIAS1	Isoform 2 of E3 SUMO-protein ligase PIAS1
			1.001	0.946	1.182	1.096	0.912	1.140	0.996	0.874	1.133	Q9NWF9-1	RNF216	Isoform 2 of E3 ubiquitin-protein ligase RNF216
			0.999	1.014	1.065	1.073	0.876	0.915	0.979	1.100	1.065	Q8IUQ4-2	SIAH1	Isoform 2 of E3 ubiquitin-protein ligase SIAH1
1.102	1.156	1.110							0.965	0.863	1.328	Q14669-2	TRIP12	Isoform 2 of E3 ubiquitin-protein ligase TRIP12
1.117	1.044	1.051	0.969	0.936	1.036	0.994	0.967	1.082	0.975	1.043	1.042	O60447-2	EVI5	Isoform 2 of Ecotropic viral integration site 5 protein homolog
1.133	1.055	0.811	0.962	1.004	0.940	1.087	1.249	1.071	0.960	0.922	1.210	Q9BSW2-2	CRACR2A	Isoform 2 of EF-hand calcium-binding domain-containing protein 4B
1.024	0.961	1.013	1.035	0.963	1.039	1.033	1.068	1.031	1.071	0.965	1.027	Q8NDI1-2	EHBP1	Isoform 2 of EH domain-binding protein 1
1.049	1.007	0.997	1.007	0.920	0.916	0.942	1.009	0.968	0.933	1.032	0.911	Q15717-2	ELAVL1	Isoform 2 of ELAV-like protein 1
1.244	0.990	1.117				1.078	1.001	0.934				P68104-2	EEF1A1	Isoform 2 of Elongation factor 1-alpha 1
1.025	1.012	1.016	1.012	1.005	1.025	0.992	1.073	1.010	0.964	1.024	0.957	P26641-2	EEF1G	Isoform 2 of Elongation factor 1-gamma
0.831	0.934	1.001	0.788	0.973	1.014	1.069	0.683	1.034	1.132	1.130	1.080	Q9NYP7-2	ELOVL5	Isoform 2 of Elongation of very long chain fatty acids protein 5
0.988	1.018	1.001	0.995	1.018	0.999	0.994	1.034	0.996	0.990	0.949	0.990	Q9NZ08-2	ERAP1	Isoform 2 of Endoplasmic reticulum aminopeptidase 1
0.991	0.970	1.030	0.998	0.960	1.024	1.021	1.017	0.987	1.000	0.981	1.011	Q9UBC2-2	EPS15L1	Isoform 2 of Epidermal growth factor receptor substrate 15-like 1
1.005	1.002	1.042	1.072	0.885	0.991	1.012	0.971	0.997	0.999	0.959	0.937	Q9NRG7-2	SDR39U1	Isoform 2 of Epimerase family protein SDR39U1
1.015	1.029	1.024	1.008	0.979	1.038	0.976	1.020	1.053	0.964	1.069	0.998	Q9Y6I3-1	EPN1	Isoform 2 of Epsin-1
0.868	0.965	0.895	1.028	0.789	1.147	1.185	0.956	1.057	1.223	0.990	1.218	Q99808-2	SLC29A1	Isoform 2 of Equilibrative nucleoside transporter 1
0.983	1.001	1.002	0.996	0.980	0.970	0.971	1.021	1.019	1.023	1.032	1.020	Q14240-2	EIF4A2	Isoform 2 of Eukaryotic initiation factor 4A-II
0.983	1.004	1.001	1.005	1.033	1.009	1.007	0.991	1.015	0.984	0.990	1.006	P55884-2	EIF3B	Isoform 2 of Eukaryotic translation initiation factor 3 subunit B
0.990	0.990	0.966	1.015	0.991	1.021	1.020	1.088	1.021	0.979	0.991	1.022	P63241-2	EIF5A	Isoform 2 of Eukaryotic translation initiation factor 5A-1
1.086	1.071	1.018										Q96CN4-2	EVI5L	Isoform 2 of EVI5-like protein
1.015	0.962	1.031	0.960	0.955	1.010	0.936	1.066	1.045	0.921	0.983	0.968	Q06265-2	EXOSC9	Isoform 2 of Exosome complex component RRP45
			1.312	0.864	1.347				0.995	0.950	1.014	Q9BSJ8-2	ESYT1	Isoform 2 of Extended synaptotagmin-1
0.977	1.010	0.989	0.985	0.962	1.019	1.009	1.037	0.992	1.027	0.999	0.989	A0FGR8-2	ESYT2	Isoform 2 of Extended synaptotagmin-2
									0.922	1.143	0.863	Q86XX4-2	FRAS1	Isoform 2 of Extracellular matrix protein FRAS1

Abundance Ratio												Accession ID	Gene name	Protein Description
1 d M1/C1	1 d M2/C2	1 d M3/C3	4 d M1/C1	4 d M2/C2	4 d M3/C3	7 d M1/C1	7 d M2/C2	7 d M3/C3	10 d M1/C1	10 d M2/C2	10 d M3/C3			
1.060	1.023	0.989	1.123	1.126	1.069	1.000	1.172	0.910	1.074	0.991	1.184	Q96AE4-2	FUBP1	Isoform 2 of Far upstream element-binding protein 1
1.099	0.979	0.929	1.221	0.778	1.137	1.515	0.805	0.993	1.055	0.900	1.050	Q9NVQ4-2	FAIM	Isoform 2 of Fas apoptotic inhibitory molecule 1
0.891	0.883	0.944	0.908	0.817	0.935	0.830	0.779	1.013	0.889	0.846	1.040	Q9UHY8-2	FEZ2	Isoform 2 of Fasciculation and elongation protein zeta-2
0.990	0.994	1.001	1.051	1.081	1.035	1.111	0.990	1.057	1.094	1.030	1.032	P51648-2	ALDH3A2	Isoform 2 of Fatty aldehyde dehydrogenase
0.945	0.951	1.216										Q8NFZ0-2	FBXO18	Isoform 2 of F-box DNA helicase 1
1.005	1.036	1.035	1.017	0.964	1.039	0.995	1.057	0.987	0.972	0.992	1.089	Q96NE9-2	FRMD6	Isoform 2 of FERM domain-containing protein 6
			1.050	0.804	1.048	1.320	1.142	0.973				Q6ZNA5-2	FRRS1	Isoform 2 of Ferric-chelate reductase 1
0.974	0.986	0.991	0.998	0.972	1.012	1.035	1.047	1.037	1.059	1.011	1.038	P22830-2	FECH	Isoform 2 of Ferrochelatase, mitochondrial
1.026	1.052	1.079	1.123	1.039	0.913							Q9UM11-2	FZR1	Isoform 2 of Fizzy-related protein homolog
0.761	1.095	0.871	0.934	1.026	1.089	1.014	0.958	0.886				Q5T1M5-2	FKBP15	Isoform 2 of FK506-binding protein 15
0.985	1.009	1.003	0.989	0.959	1.033	1.006	1.027	0.991	1.031	1.002	1.075	Q8N3X1-2	FNBP4	Isoform 2 of Formin-binding protein 4
0.992	1.018	1.043	0.991	0.937	1.002	0.976	0.967	0.954	0.977	0.889	1.036	Q96PY5-3	FMNL2	Isoform 2 of Formin-like protein 2
1.069	1.007	0.995	0.980	1.060	0.980	0.961	0.968	0.987	0.934	0.932	0.950	P04075-2	ALDOA	Isoform 2 of Fructose-bisphosphate aldolase A
1.039	1.000	1.085	0.943	0.831	1.091	0.941	1.093	0.947	0.960	1.058	1.064	Q8N612-2	FAM160A2	Isoform 2 of FTS and Hook-interacting protein
1.031	0.972	1.025	0.997	0.971	0.996	0.969	1.017	0.989	1.031	0.980	1.074	P51570-2	GALK1	Isoform 2 of Galactokinase
0.979	1.006	1.011	1.083	0.959	1.014	1.015	0.935	0.998	0.991	1.005	1.014	O00214-2	LGALS8	Isoform 2 of Galectin-8
0.954	0.917	0.997										P06396-2	GSN	Isoform 2 of Gelsolin
0.987	0.893	0.929	1.010	0.867	1.113	1.049	1.056	1.005	1.059	0.898	1.121	O43826-2	SLC37A4	Isoform 2 of Glucose-6-phosphate exchanger SLC37A4
0.892	0.881	0.883	0.949	0.805	0.881	1.006	1.185	1.136	0.928	0.951	1.031	Q9NS18-2	GLRX2	Isoform 2 of Glutaredoxin-2, mitochondrial
1.016	1.037	0.971	1.015	1.024	0.944	1.030	1.055	1.013	1.019	1.013	1.040	P49841-2	GSK3B	Isoform 2 of Glycogen synthase kinase-3 beta
1.013	0.997	1.001	1.007	1.133	0.981	1.012	0.926	1.019	0.971	0.977	0.957	Q9HC38-2	GLOD4	Isoform 2 of Glyoxalase domain-containing protein 4
0.960	0.971	0.984	0.983	0.940	0.983	1.006	0.894	0.933	1.032	1.007	1.027	Q92896-2	GLG1	Isoform 2 of Golgi apparatus protein 1
0.970	0.975	1.035	0.975	0.919	1.010	0.998	0.981	1.000	1.040	0.994	1.081	Q14789-2	GOLGB1	Isoform 2 of Golgin subfamily B member 1
0.905	0.741	0.928	1.106	1.055	0.857	0.891	1.142	0.794	0.964	0.784	1.112	Q9UHW5-2	GPN3	Isoform 2 of GPN-loop GTPase 3
0.985	0.930	0.943	1.074	0.932	0.969	1.185	1.186	0.970	1.078	1.115	1.175	Q13480-2	GAB1	Isoform 2 of GRB2-associated-binding protein 1
1.005	0.956	1.005	1.007	0.991	1.019	1.030	1.017	0.997	0.999	0.968	1.031	P52735-2	VAV2	Isoform 2 of Guanine nucleotide exchange factor VAV2
1.033	1.045	1.020	1.008	0.951	1.032	1.016	1.018	1.019	1.008	0.977	1.051	Q16774-2	GUK1	Isoform 2 of Guanylate kinase
1.007	0.996	0.969	1.036	1.001	1.038	1.063	1.149	1.159	1.273	1.200	1.155	P51795-2	CLCN5	Isoform 2 of H(+)/Cl(-) exchange transporter 5
1.016	1.023	1.017	0.875	0.920	1.142	0.989	1.038	1.055	1.041	1.001	1.271	Q99871-2	HAUS7	Isoform 2 of HAUS augmin-like complex subunit 7
									1.032	1.028	0.912	Q9Y450-2	HBS1L	Isoform 2 of HBS1-like protein
1.037	0.966	1.019	1.087	1.236	1.115	1.117	1.040	1.112	1.062	0.980	1.175	O00165-2	HAX1	Isoform 2 of HCLS1-associated protein X-1

Abundance Ratio														
1 d M1/C1	1 d M2/C2	1 d M3/C3	4 d M1/C1	4 d M2/C2	4 d M3/C3	7 d M1/C1	7 d M2/C2	7 d M3/C3	10 d M1/C1	10 d M2/C2	10 d M3/C3	Accession ID	Gene name	Protein Description
0.983	1.017	0.963	1.012	1.038	0.980	1.002	1.018	0.998	0.967	0.980	0.956	P07900-2	HSP90AA1	Isoform 2 of Heat shock protein HSP 90-alpha
1.027	1.036	0.941	1.020	1.091	0.942	1.133	1.159	0.980				Q9H910-2	HN1L	Isoform 2 of Hematological and neurological expressed 1-like protein
1.042	1.021	1.032	1.002	0.999	1.042	1.012	1.025	1.036	0.997	1.023	1.006	P61978-2	HNRNPK	Isoform 2 of Heterogeneous nuclear ribonucleoprotein K
1.014	0.994	0.993	0.989	0.987	1.031	0.946	1.189	1.042	0.974	0.971	0.974	O43390-2	HNRNPR	Isoform 2 of Heterogeneous nuclear ribonucleoprotein R
1.049	0.963	0.961	0.977	0.937	0.939	0.911	0.898	0.760	1.094	1.038	0.943	Q8WVB3-2	HEXDC	Isoform 2 of Hexosaminidase D
1.413	1.252	1.379	1.125	1.088	0.837	0.559	1.071	0.943	0.818	1.028	0.577	Q9Y241-2	HIGD1A	Isoform 2 of HIG1 domain family member 1A, mitochondrial
0.986	0.944	0.972	0.975	0.949	1.008	1.012	0.954	1.004	1.000	0.989	1.051	Q9H7Z6-2	KAT8	Isoform 2 of Histone acetyltransferase KAT8
1.079	1.008	1.013	1.089	0.957	1.128	1.134	1.157	0.810	1.025	1.007	0.865	O15379-2	HDAC3	Isoform 2 of Histone deacetylase 3
1.060	0.969	1.008	0.848	0.821	0.914	0.893	1.106	1.122	0.961	0.920	1.241	Q92800-2	EZH1	Isoform 2 of Histone-lysine N-methyltransferase EZH1
1.020	0.978	1.033	0.945	0.981	0.981	0.977	1.169	0.943	0.953	0.893	1.155	Q15910-2	EZH2	Isoform 2 of Histone-lysine N-methyltransferase EZH2
0.986	1.118	1.003										O43463-2	SUV39H1	Isoform 2 of Histone-lysine N-methyltransferase SUV39H1
			1.083	1.021	1.199	1.067	1.036	1.087	1.097	1.120	1.037	Q6PII5-2	HAGHL	Isoform 2 of Hydroxyacylglutathione hydrolase-like protein
1.016	0.971	1.024	1.013	0.919	1.074	1.002	1.124	1.028	0.979	1.017	0.996	Q9UI26-2	IPO11	Isoform 2 of Importin-11
0.978	1.016	1.030	0.998	0.963	1.025	1.017	1.105	1.063	1.012	1.017	1.022	Q8TEX9-2	IPO4	Isoform 2 of Importin-4
1.002	1.005	1.003	0.980	0.993	1.038	1.076	1.049	1.021	1.033	1.019	1.095	Q68E01-2	INTS3	Isoform 2 of Integrator complex subunit 3
1.181	1.089	1.034				1.005	1.072	1.041	1.076	0.966	1.058	Q7Z5L9-2	IRF2BP2	Isoform 2 of Interferon regulatory factor 2-binding protein 2
1.024	0.972	1.023	0.979	0.931	0.998	0.998	1.045	1.027	0.904	0.918	0.984	P80217-2	IFI35	Isoform 2 of Interferon-induced 35 kDa protein
1.025	0.943	0.950	0.958	1.095	1.038	1.070	1.057	1.060	1.058	1.039	1.040	Q8IY31-2	IFT20	Isoform 2 of Intraflagellar transport protein 20 homolog
1.094	0.852	1.080	1.152	0.978	1.192	1.008	0.997	1.086				Q96FT9-2	IFT43	Isoform 2 of Intraflagellar transport protein 43 homolog
0.961	0.834	1.348	0.905	0.932	1.553	0.930	1.044	0.963	0.952	0.975	1.153	Q9NQC8-2	IFT46	Isoform 2 of Intraflagellar transport protein 46 homolog
0.993	1.010	1.035	0.989	0.990	1.066	1.004	1.010	1.056	1.075	1.020	1.056	Q27J81-2	INF2	Isoform 2 of Inverted formin-2
1.003	1.007	1.051	0.956	0.980	1.022	0.984	0.986	1.041	1.000	1.007	1.033	Q5JU85-2	IQSEC2	Isoform 2 of IQ motif and SEC7 domain-containing protein 2
0.988	1.003	0.999	0.979	0.957	0.943	1.015	1.010	0.911	1.026	0.983	1.037	Q9BUE6-2	ISCA1	Isoform 2 of Iron-sulfur cluster assembly 1 homolog, mitochondrial
1.001	0.830	1.021	0.851	1.281	0.893	0.926	1.001	0.951	0.957	0.851	1.243	Q05084-2	ICA1	Isoform 2 of Islet cell autoantigen 1

Abundance Ratio														
1 d M1/C1	1 d M2/C2	1 d M3/C3	4 d M1/C1	4 d M2/C2	4 d M3/C3	7 d M1/C1	7 d M2/C2	7 d M3/C3	10 d M1/C1	10 d M2/C2	10 d M3/C3	Accession ID	Gene name	Protein Description
0.991	1.035	1.034	1.082	1.386	1.000	1.063	1.094	1.124	1.077	1.074	1.075	Q13907-2	IDI1	Isoform 2 of Isopentenyl-diphosphate Delta-isomerase 1
1.074	0.965	0.988	1.047	0.973	1.009	1.090	0.982	0.996	1.084	1.010	1.044	P53990-2	IST1	Isoform 2 of IST1 homolog
			0.931	0.872	0.941	0.862	1.075	0.996				Q96N16-2	JAKMIP1	Isoform 2 of Janus kinase and microtubule-interacting protein 1
0.946	1.009	0.992	0.997	0.980	1.002	0.983	1.033	1.015	0.981	1.066	0.962	P05787-2	KRT8	Isoform 2 of Keratin, type II cytoskeletal 8
1.005	0.969	0.937	0.996	0.933	0.971	1.050	1.211	1.112	0.987	1.214	0.935	Q8N6L1-2	KRTCAP2	Isoform 2 of Keratinocyte-associated protein 2
0.991	1.012	1.028	1.032	0.929	1.003	1.044	1.075	1.044	1.065	1.029	1.100	Q96J84-2	KIRREL	Isoform 2 of Kin of IRRE-like protein 1
1.002	1.005	1.018	0.990	0.978	1.051	1.034	0.985	1.009	1.030	0.973	1.044	O60333-2	KIF1B	Isoform 2 of Kinesin-like protein KIF1B
0.871	1.121	1.171										Q96Q89-2	KIF20B	Isoform 2 of Kinesin-like protein KIF20B
1.027	1.068	1.082	1.121	1.034	1.083	1.030	1.041	1.053	1.007	1.021	1.114	Q02241-2	KIF23	Isoform 2 of Kinesin-like protein KIF23
1.004	1.025	1.032	1.038	1.006	1.015	1.046	1.038	0.999	1.060	1.001	1.033	O00139-2	KIF2A	Isoform 2 of Kinesin-like protein KIF2A
1.037	0.999	1.017	0.918	1.017	0.868	0.986	1.054	1.176				Q8N5Z0-2	AADAT	Isoform 2 of Kynurenine/alpha-aminoadipate aminotransferase, mitochondrial
			1.258	0.846	0.904	1.012	1.338	0.948	1.141	0.907	1.331	Q96RQ9-2	IL4I1	Isoform 2 of L-amino-acid oxidase
1.014	0.971	1.011	0.989	0.925	1.021	1.032	1.038	1.042	1.032	1.008	1.069	Q9UIC8-2	LCMT1	Isoform 2 of Leucine carboxyl methyltransferase 1
1.004	0.933	1.063	0.958	1.003	1.062	1.009	0.973	1.025	0.961	0.959	0.976	Q9Y2L9-2	LRCH1	Isoform 2 of Leucine-rich repeat and calponin homology domain-containing protein 1
1.061	1.016	1.043	0.995	0.976	1.059	0.908	0.974	1.017	0.950	1.037	1.010	Q96PV6-2	LENG8	Isoform 2 of Leukocyte receptor cluster member 8
1.042	1.050	1.082	1.024	0.933	1.066	0.925	1.126	1.077	1.080	0.970	1.076	Q8N0W3-2	FUK	Isoform 2 of L-fucose kinase
1.012	1.016	1.028	1.001	0.931	0.982	1.023	1.008	1.005	1.015	1.006	1.000	P50851-2	LRBA	Isoform 2 of Lipopolysaccharide-responsive and beige-like anchor protein
			0.954	0.900	0.868							Q86W92-2	PPFIBP1	Isoform 2 of Liprin-beta-1
1.011	0.978	1.028	1.014	0.971	1.017	0.981	1.049	0.975	1.023	0.974	1.034	Q9P260-2	KIAA1468	Isoform 2 of LisH domain and HEAT repeat-containing protein KIAA1468
0.988	1.009	1.034	1.025	0.970	1.001	0.892	0.858	0.947	1.165	1.128	1.020	P24666-2	ACP1	Isoform 2 of Low molecular weight phosphotyrosine protein phosphatase
0.991	0.966	0.999	1.049	1.092	0.992	1.031	1.098	1.095	0.822	0.901	1.149	Q96KB5-2	PBK	Isoform 2 of Lymphokine-activated killer T-cell-originated protein kinase
1.000	1.051	1.653	1.064	0.997	1.184	1.064	0.907	1.169	1.118	1.145	1.081	Q92633-2	LPAR1	Isoform 2 of Lysophosphatidic acid receptor 1
0.971	1.056	1.129	1.038	0.935	1.073	0.903	1.138	1.028	0.935	1.020	1.001	Q15013-3	MAD2L1BP	Isoform 2 of MAD2L1-binding protein
1.013	1.005	1.014	0.989	1.003	0.980	1.022	0.998	1.016	1.007	0.980	1.018	Q96IJ6-2	GMPPA	Isoform 2 of Mannose-1-phosphate guanyltransferase alpha
1.101	1.032	1.062	1.012	1.039	0.983	1.000	1.017	1.019	0.991	0.957	1.017	Q9Y5P6-2	GMPPB	Isoform 2 of Mannose-1-phosphate guanyltransferase beta
1.144	0.972	1.046	1.019	0.975	1.002	1.018	0.986	1.002	0.980	1.002	1.074	Q8WXG6-2	MADD	Isoform 2 of MAP kinase-activating death domain protein

Abundance Ratio														
1 d M1/C1	1 d M2/C2	1 d M3/C3	4 d M1/C1	4 d M2/C2	4 d M3/C3	7 d M1/C1	7 d M2/C2	7 d M3/C3	10 d M1/C1	10 d M2/C2	10 d M3/C3	Accession ID	Gene name	Protein Description
			1.020	0.787	0.983							Q13495-3	MAMLD1	Isoform 2 of Mastermind-like domain-containing protein 1
0.941	0.944	1.026	0.934	0.898	1.036	1.030	0.946	0.978	1.130	1.049	1.156	P84157-2	MXRA7	Isoform 2 of Matrix-remodeling-associated protein 7
1.002	0.985	1.028	0.983	0.946	1.031	0.993	1.131	1.053	0.990	0.980	1.026	Q93074-2	MED12	Isoform 2 of Mediator of RNA polymerase II transcription subunit 12
1.057	0.993	1.009	1.039	0.939	1.072	1.014	1.074	0.972	1.030	0.970	1.028	Q96G25-2	MED8	Isoform 2 of Mediator of RNA polymerase II transcription subunit 8
1.040	0.989	1.027	0.994	0.974	0.978	1.039	0.977	0.941	1.006	0.965	1.080	Q9Y5V3-2	MAGED1	Isoform 2 of Melanoma-associated antigen D1
1.035	1.009	0.928	1.012	0.611	1.324	1.033	1.135	1.013	1.103	0.948	1.096	Q8N565-2	MREG	Isoform 2 of Melanoregulin
1.056	0.971	1.002	1.029	1.043	0.990	1.078	1.011	1.041	1.093	0.985	0.955	O15173-2	PGRMC2	Isoform 2 of Membrane-associated progesterone receptor component 2
1.008	0.950	1.051	0.949	0.903	1.037	1.029	1.049	1.032	1.003	0.968	1.044	Q8N108-12	MIER1	Isoform 2 of Mesoderm induction early response protein 1
			0.888	0.868	1.067	0.953	1.267	1.124				Q7Z3K6-2	MIER3	Isoform 2 of Mesoderm induction early response protein 3
0.996	0.970	0.940	1.004	0.934	1.087	1.078	1.112	1.026	1.004	1.041	1.097	Q658P3-2	STEAP3	Isoform 2 of Metalloreductase STEAP3
1.020	1.044	0.989	1.046	0.888	0.950	1.028	0.990	0.967	1.024	1.102	0.970	Q8IXL7-2	MSRB3	Isoform 2 of Methionine-R-sulfoxide reductase B3
1.055	1.025	1.102	1.080	0.854	0.974	1.014	0.951	1.077	0.991	1.179	1.061	P42898-2	MTHFR	Isoform 2 of Methylenetetrahydrofolate reductase
0.980	0.991	0.994	0.972	0.961	0.988	0.974	0.944	0.969	1.021	1.007	1.013	Q16891-2	IMMT	Isoform 2 of MICOS complex subunit MIC60
1.018	1.035	1.025	1.022	1.039	1.037	1.215	1.026	0.973	1.077	1.072	1.070	Q96EZ8-2	MCRS1	Isoform 2 of Microspherule protein 1
0.862	0.969	0.997	1.005	1.057	1.121	1.103	1.130	1.067	1.037	1.142	0.938	Q9H492-2	MAP1LC3A	Isoform 2 of Microtubule-associated proteins 1A/1B light chain 3A
			0.998	0.800	1.066	0.979	0.901	0.894				Q5VT66-2	1-Mar	Isoform 2 of Mitochondrial amidoxime-reducing component 1
0.938	1.006	0.985	0.990	0.931	0.998	1.007	1.020	0.989	0.992	0.995	0.989	Q9UBX3-2	SLC25A10	Isoform 2 of Mitochondrial dicarboxylate carrier
1.052	0.977	0.992	1.028	0.948	0.982	0.997	1.040	1.033	1.005	1.039	0.986	Q9GZY8-2	MFF	Isoform 2 of Mitochondrial fission factor
0.983	1.006	1.013	0.999	1.046	1.039	1.084	1.054	1.080	1.099	1.009	1.156	Q3ZCQ8-2	TIMM50	Isoform 2 of Mitochondrial import inner membrane translocase subunit TIM50
1.105	0.973	0.973	1.042	0.927	0.881	1.036	0.990	0.922	1.043	1.003	1.024	Q8N4Q1-2	CHCHD4	Isoform 2 of Mitochondrial intermembrane space import and assembly protein 40
0.960	0.973	0.986	0.936	0.956	0.984	1.074	1.189	0.971	0.935	0.981	1.042	Q9NYL2-2	MAP3K20	Isoform 2 of Mitogen-activated protein kinase kinase kinase 20
0.978	0.989	0.967	0.983	0.950	0.974	0.993	1.010	1.002	0.959	0.986	0.960	Q7L9L4-2	MOB1B	Isoform 2 of MOB kinase activator 1B
0.887	0.867	1.170	0.964	1.022	1.197	0.839	0.992	1.073	1.032	1.070	0.822	Q6N075-2	MFSD5	Isoform 2 of Molybdate-anion transporter
0.957	1.037	0.988	0.951	0.937	1.029	0.997	1.042	1.014	1.021	0.997	1.051	Q8N2K0-2	ABHD12	Isoform 2 of Monoacylglycerol lipase ABHD12

Abundance Ratio														
1 d M1/C1	1 d M2/C2	1 d M3/C3	4 d M1/C1	4 d M2/C2	4 d M3/C3	7 d M1/C1	7 d M2/C2	7 d M3/C3	10 d M1/C1	10 d M2/C2	10 d M3/C3	Accession ID	Gene name	Protein Description
1.013	0.996	0.988	0.979	0.972	0.997	1.009	1.053	0.995	0.987	0.974	1.006	O43148-2	RNMT	Isoform 2 of mRNA cap guanine-N7 methyltransferase
1.078	1.167	0.971	1.091	0.987	0.971							P15941-2	MUC1	Isoform 2 of Mucin-1
0.987	1.006	1.023	0.983	0.969	1.052	0.997	1.060	1.002	0.968	1.033	0.957	Q9BQG0-2	MYBBP1A	Isoform 2 of Myb-binding protein 1A
1.086	0.947	1.045	1.015	1.166	1.235	1.152	0.834	1.046	1.045	0.999	1.015	P56270-2	MAZ	Isoform 2 of Myc-associated zinc finger protein
0.987	0.987	0.990	1.002	0.969	1.000	0.990	1.000	0.996	1.013	1.009	1.042	Q6WCQ1-2	MPRIP	Isoform 2 of Myosin phosphatase Rho-interacting protein
1.585	1.040	1.196				0.542	0.882	0.936	0.836	0.947	0.915	P35749-2	MYH11	Isoform 2 of Myosin-11
1.083	0.960	0.996	0.960	0.868	1.025	1.064	0.985	1.038	1.202	1.130	1.431	Q7Z406-2	MYH14	Isoform 2 of Myosin-14
						0.896	0.966	1.067				P35579-2	MYH9	Isoform 2 of Myosin-9
0.961	0.974	0.991	1.000	0.973	1.011	1.004	1.031	1.031	1.003	0.958	0.996	Q9UJ70-2	NAGK	Isoform 2 of N-acetyl-D-glucosamine kinase
1.028	0.931	0.942	1.128	0.851	1.092				1.048	0.968	1.053	Q93015-2	NAT6	Isoform 2 of N-acetyltransferase 6
1.003	0.798	1.028	0.962	0.873	0.979	1.071	1.075	1.019	1.011	0.971	1.116	O95544-2	NADK	Isoform 2 of NAD kinase
1.006	1.019	1.008	0.947	0.932	0.960	1.021	1.079	1.034	1.023	1.005	0.987	Q86Y39-2	NDUFA11	Isoform 2 of NADH dehydrogenase [ubiquinone] 1 alpha subcomplex subunit 11
1.005	0.985	0.987	1.028	0.956	1.046	0.989	1.048	1.036	1.038	0.986	1.031	Q9P0J0-2	NDUFA13	Isoform 2 of NADH dehydrogenase [ubiquinone] 1 alpha subcomplex subunit 13
0.948	0.970	0.977	0.953	0.882	0.948	1.024	1.009	0.946	1.154	0.982	1.128	Q9NX14-2	NDUFB11	Isoform 2 of NADH dehydrogenase [ubiquinone] 1 beta subcomplex subunit 11, mitochondrial
1.029	1.010	1.004	0.987	0.971	1.023	1.021	0.994	0.990	1.061	1.001	1.050	P56181-2	NDUFV3	Isoform 2 of NADH dehydrogenase [ubiquinone] flavoprotein 3, mitochondrial
0.997	1.011	1.011	1.006	1.008	0.988	1.001	1.021	1.013	1.018	1.003	1.011	P28331-2	NDUFS1	Isoform 2 of NADH-ubiquinone oxidoreductase 75 kDa subunit, mitochondrial
1.425	1.514	0.936	0.603	0.979	1.278	1.218	1.691	0.904	0.978	1.125	1.082	Q9UHB4-2	NDOR1	Isoform 2 of NADPH-dependent diflavin oxidoreductase 1
1.105	1.007	1.110	1.044	0.936	0.993	1.015	1.053	1.041	0.945	0.986	0.918	Q9BRA0-2	NAA38	Isoform 2 of N-alpha-acetyltransferase 38, NatC auxiliary subunit
1.016	1.007	1.001	1.001	0.976	1.019	0.978	1.004	0.996	0.958	1.004	0.948	Q9Y2A7-2	NCKAP1	Isoform 2 of Nck-associated protein 1
			0.847	0.912	0.919	0.995	1.027	0.902	1.137	1.038	1.162	O76041-2	NEBL	Isoform 2 of Nebulette
0.968	1.102	1.069	1.057	0.683	1.006	0.914	0.648	0.970	1.237	0.946	1.268	Q969M7-2	UBE2F	Isoform 2 of NEDD8-conjugating enzyme UBE2F
0.961	0.964	0.997	0.968	0.934	0.992	1.011	1.001	0.994	1.066	1.003	1.065	Q8WXH0-2	SYNE2	Isoform 2 of Nesprin-2
0.982	1.010	1.007	1.192	1.138	1.154	1.146	1.152	1.063	1.100	1.113	1.137	P32004-2	L1CAM	Isoform 2 of Neural cell adhesion molecule L1
									0.679	1.057	0.830	P08949-2	NMB	Isoform 2 of Neuromedin-B
0.989	1.025	1.025	1.034	1.020	0.990	1.037	1.048	1.032	1.024	0.933	1.066	Q14697-2	GANAB	Isoform 2 of Neutral alpha-glucosidase AB
1.011	0.964	1.022	0.984	0.947	1.054	0.990	0.927	1.001	1.039	1.039	1.115	Q9Y6K9-2	IKBKG	Isoform 2 of NF-kappa-B essential modulator
0.960	0.992	0.985	1.004	0.995	1.015	1.015	0.998	0.989	1.087	1.011	1.061	O15226-2	NKRF	Isoform 2 of NF-kappa-B-repressing factor
						0.891	1.099	1.190	0.889	1.025	0.933	Q8NBF2-2	NHLRC2	Isoform 2 of NHL repeat-containing protein 2

Abundance Ratio														
1 d M1/C1	1 d M2/C2	1 d M3/C3	4 d M1/C1	4 d M2/C2	4 d M3/C3	7 d M1/C1	7 d M2/C2	7 d M3/C3	10 d M1/C1	10 d M2/C2	10 d M3/C3	Accession ID	Gene name	Protein Description
1.482	1.024	1.232										Q9UNZ2-4	NSFL1C	Isoform 2 of NSFL1 cofactor p47
			0.928	0.919	1.098	1.005	0.902	0.951	1.128	0.904	0.940	Q9GZM8-2	NDEL1	Isoform 2 of Nuclear distribution protein nudE-like 1
1.072	0.951	1.033	1.029	0.942	1.055	0.946	1.031	1.004	1.045	1.003	1.051	P19838-2	NFKB1	Isoform 2 of Nuclear factor NF-kappa-B p105 subunit
0.977	1.045	0.947	0.979	0.981	1.012	1.095	1.080	1.064	1.102	1.180	1.145	Q6P4R8-2	NFRKB	Isoform 2 of Nuclear factor related to kappa-B-binding protein
0.976	0.991	1.008	1.016	0.918	1.029	1.040	1.085	0.945	1.043	0.905	1.081	Q14980-2	NUMA1	Isoform 2 of Nuclear mitotic apparatus protein 1
			0.814	0.867	0.860	0.957	0.610	0.672				O60356-2	NUPR1	Isoform 2 of Nuclear protein 1
0.974	0.998	1.002	1.007	1.029	1.001	1.017	1.007	1.026	1.041	1.041	1.044	Q8TAT6-2	NPLOC4	Isoform 2 of Nuclear protein localization protein 4 homolog
1.046	0.977	1.070	0.993	0.985	1.047	1.081	1.056	1.034	0.984	0.938	1.045	Q86WQ0-2	NR2C2AP	Isoform 2 of Nuclear receptor 2C2-associated protein
1.022	1.043	1.006	1.009	0.969	1.047	1.022	1.016	1.052	1.063	0.968	1.051	Q8NI08-2	NCOA7	Isoform 2 of Nuclear receptor coactivator 7
1.049	1.004	1.084	1.016	0.998	1.017	1.001	1.078	0.987	0.920	0.899	1.045	P49116-2	NR2C2	Isoform 2 of Nuclear receptor subfamily 2 group C member 2
1.018	1.020	0.982	0.996	1.016	0.952	0.999	1.105	1.060	0.987	1.004	0.970	Q01085-2	TIAL1	Isoform 2 of Nucleolysin TIAR
0.879	0.940	0.979	0.951	0.965	1.091	0.727	0.613	0.950	0.975	0.974	0.857	P06748-2	NPM1	Isoform 2 of Nucleophosmin
1.033	1.003	1.118	0.972	0.992	1.019	0.964	0.972	0.869	0.945	0.971	1.006	P15531-2	NME1	Isoform 2 of Nucleoside diphosphate kinase A
1.022	0.992	1.008	0.999	0.973	0.968	0.966	0.961	0.997	0.975	0.965	0.987	Q99733-2	NAP1L4	Isoform 2 of Nucleosome assembly protein 1-like 4
0.843	1.019	0.981	1.117	1.148	1.141	1.165	1.003	1.133	1.070	1.053	1.104	Q9NRP0-2	OSTC	Isoform 2 of Oligosaccharyltransferase complex subunit OSTC
			0.811	1.439	0.988				0.979	1.095	1.091	Q96CV9-2	OPTN	Isoform 2 of Optineurin
1.016	0.918	1.049	0.913	0.850	1.008							Q9UBD5-2	ORC3	Isoform 2 of Origin recognition complex subunit 3
0.994	0.988	1.009				1.126	1.074	1.076				Q8N573-2	OXR1	Isoform 2 of Oxidation resistance protein 1
1.106	1.014	0.896	1.032	0.955	0.968	1.052	0.946	1.004	1.100	0.989	1.062	Q99571-2	P2RX4	Isoform 2 of P2X purinoceptor 4
0.995	1.003	1.045	0.991	0.964	0.991	1.030	1.061	1.054	1.011	1.089	1.078	O75182-2	SIN3B	Isoform 2 of Paired amphipathic helix protein Sin3b
0.924	0.937	0.965	0.958	0.889	1.013	0.938	0.910	0.981	1.018	0.928	0.997	Q02962-2	PAX2	Isoform 2 of Paired box protein Pax-2
						1.701	1.234	0.665	1.032	0.966	1.043	Q9NXF8-2	ZDHHC7	Isoform 2 of Palmitoyltransferase ZDHHC7
1.059	0.927	1.007	0.978	0.946	1.037	0.961	0.988	1.013	1.048	0.988	1.040	Q8NB37-2	PDDC1	Isoform 2 of Parkinson disease 7 domain-containing protein 1
0.986	1.020	1.013	1.125	0.932	1.080	0.890	1.112	1.028	0.962	0.990	0.947	Q96RG2-2	PASK	Isoform 2 of PAS domain-containing serine/threonine-protein kinase
1.014	1.000	1.012	0.964	0.991	0.970	0.968	1.047	0.955	1.029	1.065	1.033	Q5EBL8-2	PDZD11	Isoform 2 of PDZ domain-containing protein 11

Abundance Ratio												Accession ID	Gene name	Protein Description
1 d M1/C1	1 d M2/C2	1 d M3/C3	4 d M1/C1	4 d M2/C2	4 d M3/C3	7 d M1/C1	7 d M2/C2	7 d M3/C3	10 d M1/C1	10 d M2/C2	10 d M3/C3			
0.996	1.030	1.012	0.941	0.923	0.930	0.952	0.874	0.957	1.005	0.956	1.067	Q9Y680-2	FKBP7	Isoform 2 of Peptidyl-prolyl cis-trans isomerase FKBP7
0.969	0.993	1.034	1.022	1.107	1.035	1.073	1.020	1.084	1.039	1.092	1.072	Q14318-2	FKBP8	Isoform 2 of Peptidyl-prolyl cis-trans isomerase FKBP8
1.038	1.065	0.967	1.038	1.108	1.028	1.020	0.961	1.024	1.090	1.085	1.009	Q9Y237-2	PIN4	Isoform 2 of Peptidyl-prolyl cis-trans isomerase NIMA-interacting 4
1.031	0.997	0.998	0.975	0.955	0.987	1.019	1.080	0.992	1.039	1.070	1.066	Q9Y5Y5-2	PEX16	Isoform 2 of Peroxisomal membrane protein PEX16
0.986	1.015	1.025	0.975	0.916	1.012	0.970	1.020	0.965	0.978	1.000	1.068	Q8IZ21-2	PHACTR4	Isoform 2 of Phosphatase and actin regulator 4
0.950	0.953	1.082	0.977	0.894	0.981	1.060	1.075	1.044	0.991	0.987	0.995	Q9BQK8-2	LPIN3	Isoform 2 of Phosphatidate phosphatase LPIN3
0.984	1.035	1.044	0.898	0.803	0.956	1.106	1.124	1.061	1.111	1.094	1.176	Q9BRB3-2	PIGQ	Isoform 2 of Phosphatidylinositol N-acetylglucosaminyltransferase subunit Q
1.181	1.077	1.101	1.014	1.017	1.030	0.965	1.097	1.040	0.889	1.076	1.060	Q86VP3-2	PACS2	Isoform 2 of Phosphofurin acidic cluster sorting protein 2
1.151	1.515	1.486	1.224	0.890	1.501				0.979	1.047	1.111	O43688-2	PLPP2	Isoform 2 of Phospholipid phosphatase 2
0.933	0.985	1.414	0.969	0.957	1.282	0.644	1.279	1.012	0.893	1.072	1.025	O14523-2	C2CD2L	Isoform 2 of Phospholipid transfer protein C2CD2L
1.015	1.012	0.998	0.993	0.939	0.996	0.997	1.077	0.982	0.970	0.992	0.970	Q14558-2	PRPSAP1	Isoform 2 of Phosphoribosyl pyrophosphate synthase-associated protein 1
1.000	0.983	1.023	0.956	0.962	0.983	1.010	0.912	0.975	1.054	1.025	1.078	Q86UU1-2	PHLDB1	Isoform 2 of Pleckstrin homology-like domain family B member 1
0.984	0.997	0.965	0.988	0.989	0.998	1.007	1.029	1.011	0.970	1.037	1.014	Q9UHX1-2	PUF60	Isoform 2 of Poly(U)-binding-splicing factor PUF60
1.008	0.999	0.984	1.005	0.977	1.027	1.038	1.095	1.003	0.951	0.952	1.010	O75530-2	EED	Isoform 2 of Polycomb protein EED
0.987	0.956	1.032	0.977	0.918	1.045	1.000	1.087	0.965	0.992	0.959	1.038	Q9ULR0-1	ISY1	Isoform 2 of Pre-mRNA-splicing factor ISY1 homolog
0.936	0.977	1.004	0.932	0.922	0.962	0.915	0.856	0.937	0.986	0.929	0.996	Q5JRX3-2	PITRM1	Isoform 2 of Presequence protease, mitochondrial
0.831	0.755	1.117	0.776	1.184	1.089	0.762	0.445	0.977	0.928	1.127	1.107	P46087-2	NOP2	Isoform 2 of Probable 28S rRNA (cytosine(4447)-C(5))-methyltransferase
1.027	1.009	1.044	1.026	0.981	1.030	1.032	1.093	1.044	1.012	1.038	1.014	Q96P11-2	NSUN5	Isoform 2 of Probable 28S rRNA (cytosine-C(5))-methyltransferase
			0.922	1.970	1.148							Q5T6J7-2	IDNK	Isoform 2 of Probable gluconokinase
0.989	1.001	1.043	0.959	0.979	0.975	0.977	0.891	0.990	1.039	0.990	1.031	Q5JPH6-2	EARS2	Isoform 2 of Probable glutamate--tRNA ligase, mitochondrial
1.067	1.020	0.999	1.040	1.382	1.054	1.125	0.848	1.131	1.133	1.018	1.251	Q8N490-2	PNKD	Isoform 2 of Probable hydrolase PNKD
						1.149	1.220	0.940				Q9C0D7-2	ZC3H12C	Isoform 2 of Probable ribonuclease ZC3H12C
1.006	1.017	1.006	0.992	0.956	1.005	0.993	1.023	0.994	0.987	0.987	1.009	Q02809-2	PLOD1	Isoform 2 of Procollagen-lysine,2-oxoglutarate 5-dioxygenase 1

Abundance Ratio														
1 d M1/C1	1 d M2/C2	1 d M3/C3	4 d M1/C1	4 d M2/C2	4 d M3/C3	7 d M1/C1	7 d M2/C2	7 d M3/C3	10 d M1/C1	10 d M2/C2	10 d M3/C3	Accession ID	Gene name	Protein Description
1.010	1.031	1.069	0.997	0.974	1.030	0.969	1.072	1.004	0.956	0.960	0.962	O00469-2	PLOD2	Isoform 2 of Procollagen-lysine,2-oxoglutarate 5-dioxygenase 2
			1.154	0.990	1.194	0.925	1.180	1.181	1.018	1.003	0.920	Q8WUM4-2	PDCD6IP	Isoform 2 of Programmed cell death 6-interacting protein
0.940	1.013	0.960	1.003	0.972	1.013	0.972	1.059	1.023	0.987	0.989	0.986	O75340-2	PDCD6	Isoform 2 of Programmed cell death protein 6
1.061	1.002	1.017	0.953	1.013	0.994	0.935	0.964	0.941	0.910	0.965	0.939	P13674-2	P4HA1	Isoform 2 of Prolyl 4-hydroxylase subunit alpha-1
0.927	0.997	1.091	0.972	0.854	1.009	1.013	0.927	0.926	1.009	0.859	1.113	Q96B96-2	TMEM159	Isoform 2 of Promethin
1.013	0.995	1.017	0.976	0.985	1.043	1.023	1.078	1.038	0.980	0.955	1.069	P61289-2	PSME3	Isoform 2 of Proteasome activator complex subunit 3
0.876	1.004	0.965	0.950	0.964	1.039	0.972	0.989	1.093	0.989	1.023	0.988	Q5JS54-2	PSMG4	Isoform 2 of Proteasome assembly chaperone 4
0.904	0.954	1.167	0.987	1.009	1.182	1.029	1.039	1.116	0.991	0.979	1.041	Q5VST6-2	ABHD17B	Isoform 2 of Protein ABHD17B
0.977	0.969	1.010	1.000	0.956	1.053	1.028	0.976	1.020	1.044	0.986	1.075	Q8TD16-2	BICD2	Isoform 2 of Protein bicaudal D homolog 2
0.942	0.978	0.960	0.987	1.088	1.049	1.022	0.805	0.944	1.042	0.998	1.073	Q8WUW1-2	BRK1	Isoform 2 of Protein BRICK1
1.256	0.897	1.029	0.937	0.954	1.089	1.056	1.094	1.007	1.072	1.050	1.111	Q14201-2	BTG3	Isoform 2 of Protein BTG3
0.993	1.019	0.994	0.984	0.982	1.014	0.984	1.045	1.004	1.002	0.996	1.012	Q15084-2	PDIA6	Isoform 2 of Protein disulfide-isomerase A6
1.020	0.993	1.030	0.989	0.966	1.018	1.005	1.010	1.005	0.985	0.985	1.022	Q8WYP5-2	AHCTF1	Isoform 2 of Protein ELYS
1.151	0.900	0.888	0.927	0.988	1.039	1.187	0.950	1.093	0.971	0.900	1.110	Q9H098-2	FAM107B	Isoform 2 of Protein FAM107B
1.001	1.024	0.981	0.981	0.975	1.003	0.978	1.106	1.083	0.972	1.026	1.033	Q8N128-2	FAM177A1	Isoform 2 of Protein FAM177A1
1.002	0.992	0.991	0.992	0.994	1.015	0.998	0.993	1.025	0.974	1.022	0.998	Q52LJ0-2	FAM98B	Isoform 2 of Protein FAM98B
1.010	1.021	1.038	0.982	0.966	0.982	0.975	1.054	1.036	1.058	0.924	1.024	Q5VW38-2	GPR107	Isoform 2 of Protein GPR107
0.961	0.876	1.004	0.964	1.029	1.060	0.981	1.060	1.005	1.025	0.928	0.971	Q8IX03-2	WWC1	Isoform 2 of Protein KIBRA
0.951	0.917	1.004	0.904	1.042	1.042	0.913	0.832	0.991	0.987	0.998	1.000	Q3MHD2-2	LSM12	Isoform 2 of Protein LSM12 homolog
1.006	0.970	0.977	0.971	0.970	0.988	0.992	0.976	1.004	0.962	0.946	1.007	Q8WZA0-2	LZIC	Isoform 2 of Protein LZIC
1.017	0.986	1.008	1.006	0.968	1.010	1.010	1.048	1.049	1.027	1.040	1.049	Q96PU8-3	QKI	Isoform 2 of Protein quaking
1.192	0.889	1.201	0.976	0.971	1.092	0.888	1.104	0.957	1.083	1.103	1.087	Q8N9R8-2	SCAI	Isoform 2 of Protein SCAI
0.996	1.017	1.048	0.933	1.009	1.052	1.081	0.811	1.002	1.109	0.961	1.210	Q01105-2	SET	Isoform 2 of Protein SET
0.984	1.062	1.043	1.782	1.387	1.757	0.916	1.054	1.126	0.943	0.879	0.938	Q9Y2Z0-2	SUGT1	Isoform 2 of Protein SGT1 homolog
0.996	0.990	1.030	0.956	0.985	1.039	1.027	0.953	1.036	1.026	0.980	1.098	Q8ND04-2	SMG8	Isoform 2 of Protein SMG8
1.052	0.958	1.023	1.119	1.009	1.188	0.969	0.994	1.009	1.293	1.268	1.445	O94964-2	SOGA1	Isoform 2 of Protein SOGA1
1.008	0.952	1.050	1.232	1.122	1.121	1.072	1.010	0.878	1.051	1.035	1.084	Q08AE8-2	SPIRE1	Isoform 2 of Protein spire homolog 1
1.068	0.915	0.950	1.050	0.929	0.983	1.030	1.267	1.034	0.948	0.898	1.133	Q9HCD6-2	TANC2	Isoform 2 of Protein TANC2
1.045	0.992	1.018	1.002	0.952	0.973	0.936	0.973	0.999	0.999	0.959	0.968	O94855-2	SEC24D	Isoform 2 of Protein transport protein Sec24D
0.850	1.049	1.117	0.968	0.952	1.818	0.931	1.227	1.312	1.210	0.840	1.599	Q9Y2J8-2	PADI2	Isoform 2 of Protein-arginine deiminase type-2
1.028	1.001	1.018	1.011	1.044	1.000	1.021	1.086	1.034	0.957	1.013	0.990	P12931-2	SRC	Isoform 2 of Proto-oncogene tyrosine-protein kinase Src

Abundance Ratio														
1 d M1/C1	1 d M2/C2	1 d M3/C3	4 d M1/C1	4 d M2/C2	4 d M3/C3	7 d M1/C1	7 d M2/C2	7 d M3/C3	10 d M1/C1	10 d M2/C2	10 d M3/C3	Accession ID	Gene name	Protein Description
0.982	1.020	1.008	0.990	0.983	0.999	0.997	1.038	1.010	0.990	0.977	1.000	Q7L2E3-2	DHX30	Isoform 2 of Putative ATP-dependent RNA helicase DHX30
0.987	0.975	1.005	1.004	1.010	1.002	0.970	0.984	1.012	1.013	1.013	0.983	Q49A26-2	GLYR1	Isoform 2 of Putative oxidoreductase GLYR1
1.215	0.934	1.106	1.049	0.980	0.995	0.996	1.126	1.094	0.884	0.976	0.908	O75061-2	DNAJC6	Isoform 2 of Putative tyrosine-protein phosphatase auxilin
1.233	1.136	1.126	1.120	0.877	1.073	0.988	1.116	1.039				Q7L804-2	RAB11FIP2	Isoform 2 of Rab11 family-interacting protein 2
1.038	1.025	1.002	0.934	0.946	1.037	0.967	1.024	1.023	1.023	0.959	1.048	Q9UNT1-2	RABL2B	Isoform 2 of Rab-like protein 2B
1.012	1.037	0.932	0.993	0.942	1.000	1.000	1.056	1.026	1.014	1.024	1.033	P04049-2	RAF1	Isoform 2 of RAF proto-oncogene serine/threonine-protein kinase
1.134	1.172	1.142	0.864	0.949	1.184	0.959	1.048	1.158	1.335	0.966	1.228	Q3MIN7-2	RGL3	Isoform 2 of Ral guanine nucleotide dissociation stimulator-like 3
1.016	0.993	1.014	1.001	0.964	0.976	0.992	0.979	0.974	1.017	1.002	1.018	Q9H6Z4-2	RANBP3	Isoform 2 of Ran-binding protein 3
0.750	1.269	1.599	0.830	1.028	1.257							Q8WYP3-2	RIN2	Isoform 2 of Ras and Rab interactor 2
						0.996	0.700	1.033				Q9H2L5-2	RASSF4	Isoform 2 of Ras association domain-containing protein 4
0.990	1.006	0.997	0.992	0.994	1.027	0.976	1.052	1.028	0.957	1.012	1.018	Q9UJF2-2	RASAL2	Isoform 2 of Ras GTPase-activating protein nGAP
1.070	0.998	1.006	1.022	1.093	0.970	0.969	0.954	1.019	0.963	0.972	0.924	Q5VZM2-2	RRAGB	Isoform 2 of Ras-related GTP-binding protein B
1.020	1.033	1.119	0.984	0.865	0.987	1.013	1.122	1.003	0.933	0.910	1.059	A4D1S5-2	RAB19	Isoform 2 of Ras-related protein Rab-19
			1.085	0.910	0.964	0.980	1.195	1.004	0.951	1.044	0.994	P61018-2	RAB4B	Isoform 2 of Ras-related protein Rab-4B
0.947	0.994	0.964	0.997	0.909	1.031	0.999	1.071	1.027	1.031	1.065	1.071	P51148-2	RAB5C	Isoform 2 of Ras-related protein Rab-5C
0.988	0.980	0.972	0.990	0.958	1.001	0.993	1.015	0.981	1.015	1.014	1.034	P11234-2	RALB	Isoform 2 of Ras-related protein Ral-B
1.050	0.976	1.083	0.899	0.942	1.075	1.026	0.986	1.007	0.961	0.970	1.004	Q92766-2	RREB1	Isoform 2 of Ras-responsive element-binding protein 1
1.029	1.137	0.997	0.994	0.895	0.898	0.939	1.137	1.088	1.040	1.079	0.993	O00559-2	EBAG9	Isoform 2 of Receptor-binding cancer antigen expressed on SiSo cells
0.992	1.020	1.011	1.046	0.969	1.025	1.126	1.039	1.016	1.038	0.995	1.073	P28827-2	PTPRM	Isoform 2 of Receptor-type tyrosine-protein phosphatase mu
0.948	1.015	0.961	0.998	1.049	1.007	1.027	1.061	1.013	1.021	1.046	1.035	P18754-2	RCC1	Isoform 2 of Regulator of chromosome condensation
1.038	0.981	1.026	0.998	0.975	1.110	1.100	1.025	1.065	1.087	1.134	1.140	Q9BWE0-4	REPIN1	Isoform 2 of Replication initiator 1
1.003	0.999	0.992	1.001	0.974	0.999	0.962	1.010	1.017	1.029	1.037	1.027	Q14257-2	RCN2	Isoform 2 of Reticulocalbin-2
0.928	1.005	0.957	0.931	0.917	0.983	1.010	1.085	1.035	1.073	0.984	1.103	Q9NQC3-2	RTN4	Isoform 2 of Reticulon-4
0.758	1.004	1.078	0.828	0.811	1.116	0.839	0.565	0.892	0.833	0.796	0.736	Q15291-2	RBBP5	Isoform 2 of Retinoblastoma-binding protein 5
0.956	0.969	1.041	0.965	0.927	1.020	0.977	1.005	1.038	0.897	1.001	0.997	P28702-3	RXRB	Isoform 2 of Retinoic acid receptor RXR-beta
1.103	1.048	0.977										Q14CB8-2	ARHGAP19	Isoform 2 of Rho GTPase-activating protein 19
			0.855	0.952	0.813	1.210	1.042	1.104				Q9C0H5-2	ARHGAP39	Isoform 2 of Rho GTPase-activating protein 39

Abundance Ratio														
1 d M1/C1	1 d M2/C2	1 d M3/C3	4 d M1/C1	4 d M2/C2	4 d M3/C3	7 d M1/C1	7 d M2/C2	7 d M3/C3	10 d M1/C1	10 d M2/C2	10 d M3/C3	Accession ID	Gene name	Protein Description
1.074	1.027	1.005	1.036	1.022	1.038	1.045	1.054	0.997	0.956	0.977	0.993	O15085-2	ARHGEF11	Isoform 2 of Rho guanine nucleotide exchange factor 11
0.886	0.985	1.370				0.936	0.935	1.006				Q9NR81-2	ARHGEF3	Isoform 2 of Rho guanine nucleotide exchange factor 3
0.957	1.060	1.066	1.116	0.980	1.077	1.025	0.987	1.022	1.145	1.187	1.073	Q6NTF9-2	RHBDD2	Isoform 2 of Rhomboid domain-containing protein 2
			0.970	1.010	1.082	1.059	1.044	0.971	1.268	1.128	1.127	Q9BST9-2	RTKN	Isoform 2 of Rhotekin
1.042	1.005	1.012	1.030	1.026	0.981	1.026	1.072	1.008	0.990	1.005	1.035	P78346-2	RPP30	Isoform 2 of Ribonuclease P protein subunit p30
0.924	0.997	0.991	0.959	0.965	1.020	1.073	1.160	1.029	0.902	0.919	1.051	P31350-2	RRM2	Isoform 2 of Ribonucleoside-diphosphate reductase subunit M2
0.974	1.009	1.010	1.007	1.002	1.012	0.991	1.016	0.995	1.025	0.998	0.989	Q15418-2	RPS6KA1	Isoform 2 of Ribosomal protein S6 kinase alpha-1
0.975	0.953	1.022	0.992	0.982	0.955	1.034	1.066	0.940	1.104	0.987	1.149	Q9UK32-2	RPS6KA6	Isoform 2 of Ribosomal protein S6 kinase alpha-6
			1.049	0.983	1.027	0.919	1.069	1.079	0.971	0.964	1.149	Q8N5U6-2	RNF10	Isoform 2 of RING finger protein 10
0.946	0.933	0.986	0.972	1.056	0.994	0.979	0.825	1.013	0.993	1.006	1.041	O00442-2	RTCA	Isoform 2 of RNA 3'-terminal phosphate cyclase
1.338	1.416	1.161	0.948	1.030	1.046	1.359	1.154	1.350				Q5T8P6-2	RBM26	Isoform 2 of RNA-binding protein 26
1.002	0.995	0.959	1.001	1.048	0.994	1.006	1.001	1.011	1.022	1.025	1.009	Q14498-2	RBM39	Isoform 2 of RNA-binding protein 39
1.029	1.021	1.008	0.883	0.907	1.103	1.014	1.018	1.008	1.113	0.955	1.111	Q9Y5S9-2	RBM8A	Isoform 2 of RNA-binding protein 8A
1.041	0.964	1.019	1.076	0.921	1.015	1.159	1.118	1.074	1.114	1.038	1.080	Q9Y6N7-2	ROBO1	Isoform 2 of Roundabout homolog 1
1.305	1.097	0.956	1.111	0.964	0.908	1.144	1.081	1.091	1.169	1.120	0.956	Q92736-2	RYR2	Isoform 2 of Ryanodine receptor 2
1.078	0.981	1.052	1.037	0.936	1.036	1.076	1.074	1.104	0.894	1.013	1.008	Q92503-2	SEC14L1	Isoform 2 of SEC14-like protein 1
1.007	0.973	0.981	1.013	1.055	0.998	1.034	0.927	1.029	1.016	1.004	1.006	Q12765-2	SCRN1	Isoform 2 of Secernin-1
0.979	0.992	0.990	0.978	0.993	0.979	0.988	0.991	0.980	0.986	0.998	0.975	Q9NVA2-2	11-Sep	Isoform 2 of Septin-11
1.013	1.001	0.994	0.984	0.970	0.982	0.978	1.007	0.999	1.005	1.010	0.988	Q15019-2	2-Sep	Isoform 2 of Septin-2
1.008	1.012	0.984	0.982	0.998	0.965	0.990	0.939	0.982	1.000	1.016	1.008	Q16181-2	7-Sep	Isoform 2 of Septin-7
			1.053	0.906	0.848							O95084-2	PRSS23	Isoform 2 of Serine protease 23
0.986	0.979	1.014	0.977	0.958	1.040	0.946	1.035	1.042	0.946	0.970	0.986	Q13188-2	STK3	Isoform 2 of Serine/threonine-protein kinase 3
1.013	1.040	1.000	1.008	0.968	1.034	1.008	1.069	1.069	0.877	0.887	1.107	Q16512-2	PKN1	Isoform 2 of Serine/threonine-protein kinase N1
0.915	1.150	0.947	1.069	0.961	0.991	1.036	0.972	1.024	0.834	1.017	1.035	Q9HC98-2	NEK6	Isoform 2 of Serine/threonine-protein kinase Nek6
1.000	0.953	1.011	0.993	0.974	1.070	1.014	1.018	1.025	1.036	0.971	1.139	Q9UKI8-2	TLK1	Isoform 2 of Serine/threonine-protein kinase tousled-like 1
1.024	1.019	1.050	1.027	0.972	0.993	1.080	1.172	0.965	1.067	0.983	1.027	Q86UE8-2	TLK2	Isoform 2 of Serine/threonine-protein kinase tousled-like 2
0.996	0.985	1.018	0.986	1.010	0.990	0.988	0.927	0.971	1.010	0.952	1.050	P63151-2	PPP2R2A	Isoform 2 of Serine/threonine-protein phosphatase 2A 55 kDa regulatory subunit B alpha isoform

Abundance Ratio														
1 d M1/C1	1 d M2/C2	1 d M3/C3	4 d M1/C1	4 d M2/C2	4 d M3/C3	7 d M1/C1	7 d M2/C2	7 d M3/C3	10 d M1/C1	10 d M2/C2	10 d M3/C3	Accession ID	Gene name	Protein Description
1.004	1.131	1.119	0.987	1.135	0.988	1.016	1.151	1.083				P30154-2	PPP2R1B	Isoform 2 of Serine/threonine-protein phosphatase 2A 65 kDa regulatory subunit A beta isoform
0.956	1.042	1.032	0.985	0.989	1.027	1.018	1.046	1.007	1.051	0.994	1.056	Q08209-2	PPP3CA	Isoform 2 of Serine/threonine-protein phosphatase 2B catalytic subunit alpha isoform
1.012	1.009	1.017	0.986	0.939	0.996	1.008	1.091	0.987	0.935	0.984	0.989	Q5MIZ7-2	PPP4R3B	Isoform 2 of Serine/threonine-protein phosphatase 4 regulatory subunit 3B
0.989	0.954	1.035	1.001	1.012	1.087	1.021	0.978	1.033	1.023	1.005	1.181	Q8N8A2-2	ANKRD44	Isoform 2 of Serine/threonine-protein phosphatase 6 regulatory ankyrin repeat subunit B
1.038	1.008	0.985	1.027	1.051	0.976	1.031	1.046	0.995	1.014	1.000	0.995	Q9Y3F4-2	STRAP	Isoform 2 of Serine-threonine kinase receptor-associated protein
			0.837	0.808	1.088	0.876	1.179	1.067				Q96P63-2	SERPINB12	Isoform 2 of Serpin B12
0.985	1.380	1.022	1.025	0.936	1.087	0.765	1.206	1.141				Q9UQ49-2	NEU3	Isoform 2 of Sialidase-3
1.019	0.984	1.070	1.035	0.955	0.988	1.039	1.074	0.998	0.986	0.957	1.060	Q9UGK3-2	STAP2	Isoform 2 of Signal-transducing adaptor protein 2
1.005	1.002	0.924	0.958	0.890	1.090	1.279	1.001	0.943	1.124	1.108	1.051	Q96HU1-2	SGSM3	Isoform 2 of Small G protein signaling modulator 3
0.882	1.019	1.157				0.916	0.885	0.968	0.980	0.956	0.991	Q8N5G0-2	SMIM20	Isoform 2 of Small integral membrane protein 20
						1.324	1.026	1.097	1.144	1.063	1.084	Q92581-2	SLC9A6	Isoform 2 of Sodium/hydrogen exchanger 6
			0.945	0.710	1.018	0.982	1.224	1.134				P78383-2	SLC35B1	Isoform 2 of Solute carrier family 35 member B1
1.070	1.020	1.011	0.980	1.002	1.036	0.991	1.066	1.035	1.049	0.959	1.115	Q96RF0-2	SNX18	Isoform 2 of Sorting nexin-18
0.982	0.991	0.994	1.008	0.961	1.033	1.019	0.966	1.005	0.997	0.958	1.025	Q9NUQ6-2	SPATS2L	Isoform 2 of SPATS2-like protein
1.079	0.981	1.067	1.192	1.066	1.030	0.943	0.782	0.982	0.828	0.906	0.996	Q01082-3	SPTBN1	Isoform 2 of Spectrin beta chain, non-erythrocytic 1
0.985	1.009	0.964	0.942	0.917	0.946	0.963	0.940	0.984	1.007	0.934	1.020	Q8TB22-2	SPATA20	Isoform 2 of Spermatogenesis-associated protein 20
						0.919	1.312	0.995	0.959	0.945	0.892	Q9NYA1-2	SPHK1	Isoform 2 of Sphingosine kinase 1
1.032	1.002	1.000	0.998	0.938	0.982	1.008	1.077	0.996	0.999	0.980	1.000	Q12872-2	SFSWAP	Isoform 2 of Splicing factor, suppressor of white-apricot homolog
0.988	0.977	1.009	0.973	1.017	1.034	0.976	0.970	0.998	1.041	0.996	1.032	Q8WXA9-2	SREK1	Isoform 2 of Splicing regulatory glutamine/lysine-rich protein 1
1.059	1.039	1.109	0.940	1.054	1.009	0.947	1.019	0.980	0.960	1.057	1.094	P78362-2	SRPK2	Isoform 2 of SRSF protein kinase 2
0.984	1.000	1.016	0.991	1.015	1.043	0.985	0.914	1.005	1.048	1.033	1.048	Q9H2G2-2	SLK	Isoform 2 of STE20-like serine/threonine-protein kinase
0.987	1.007	0.960	1.004	1.056	0.973	0.983	0.929	0.986	0.975	1.017	0.972	P31948-2	STIP1	Isoform 2 of Stress-induced-phosphoprotein 1
			1.277	1.029	0.842							Q9P246-2	STIM2	Isoform 2 of Stromal interaction molecule 2

Abundance Ratio														
1 d M1/C1	1 d M2/C2	1 d M3/C3	4 d M1/C1	4 d M2/C2	4 d M3/C3	7 d M1/C1	7 d M2/C2	7 d M3/C3	10 d M1/C1	10 d M2/C2	10 d M3/C3	Accession ID	Gene name	Protein Description
1.008	0.997	1.027	0.959	0.985	1.013	1.017	0.856	1.037	1.060	1.012	1.075	Q8IYB5-2	SMAP1	Isoform 2 of Stromal membrane-associated protein 1
1.052	1.021	1.035	1.007	1.004	1.024	1.024	0.978	0.999	1.034	1.010	1.067	Q96SB8-2	SMC6	Isoform 2 of Structural maintenance of chromosomes protein 6
1.051	0.999	1.050	0.989	0.965	1.009	0.984	0.970	0.970	1.062	0.996	1.012	P51649-2	ALDH5A1	Isoform 2 of Succinate-semialdehyde dehydrogenase, mitochondrial
0.810	0.890	0.897	1.015	0.949	0.963	0.918	0.855	0.993	1.092	1.004	1.100	Q9BRV8-2	SIKE1	Isoform 2 of Suppressor of IKBKE 1
1.056	1.009	1.018	1.019	0.925	0.998	1.003	1.063	1.020	0.926	0.954	0.941	Q9H4L7-2	SMARCAD1	Isoform 2 of SWI/SNF-related matrix-associated actin-dependent regulator of chromatin subfamily A containing DEAD/H box 1
0.915	1.004	0.943	1.013	0.919	1.024	1.148	0.929	1.060	1.257	1.048	1.140	O43760-2	SYNGR2	Isoform 2 of Synaptogyrin-2
1.027	1.006	1.074	0.973	0.984	1.003	0.923	0.882	0.963	0.900	0.989	0.998	Q8N3V7-2	SYNPO	Isoform 2 of Synaptopodin
0.975	1.020	1.000	1.008	1.000	1.026	1.017	1.020	1.025	1.048	1.026	1.075	P61764-2	STXBP1	Isoform 2 of Syntaxin-binding protein 1
1.049	0.987	1.016	1.018	0.950	0.986	0.980	0.927	0.982	1.026	0.981	1.044	P82094-2	TMF1	Isoform 2 of TATA element modulatory factor
1.037	0.997	1.007	0.997	0.977	1.008	1.047	1.025	1.047	1.029	0.979	1.040	Q92609-2	TBC1D5	Isoform 2 of TBC1 domain family member 5
1.128	1.069	1.036	0.989	1.003	1.080	1.049	1.117	0.972	1.066	1.099	1.056	Q7Z6L1-2	TECPR1	Isoform 2 of Tectonin beta-propeller repeat-containing protein 1
0.949	0.883	0.864	0.970	0.992	1.106	1.103	1.041	1.181	0.950	1.008	1.149	Q86YL5-2	TDRP	Isoform 2 of Testis development-related protein
1.143	1.076	0.799	1.035	0.993	1.050	0.999	1.097	1.027	1.008	0.972	1.036	Q8IWB9-2	TEX2	Isoform 2 of Testis-expressed protein 2
1.060	1.012	1.030	0.966	0.946	0.993	1.000	1.069	0.987	0.957	0.980	1.052	Q9BZE9-2	ASPSCR1	Isoform 2 of Tether containing UBX domain for GLUT4
0.983	1.002	1.071	0.986	0.884	1.004	0.986	0.962	0.997				Q9H892-2	TTC12	Isoform 2 of Tetratricopeptide repeat protein 12
1.019	1.026	1.188	1.083	0.909	1.051	1.080	1.092	1.079	1.052	0.943	1.070	Q86TV6-2	TTC7B	Isoform 2 of Tetratricopeptide repeat protein 7B
1.044	1.022	1.034										P36897-2	TGFBR1	Isoform 2 of TGF-beta receptor type-1
1.332	1.000	1.337	1.258	1.026	1.389							P37173-2	TGFBR2	Isoform 2 of TGF-beta receptor type-2
1.035	1.012	1.058	1.039	1.069	1.015	1.004	0.950	1.024	0.980	1.018	0.973	Q86XR7-2	TICAM2	Isoform 2 of TIR domain-containing adapter molecule 2
1.215	0.901	1.093	1.247	0.880	1.064	1.088	0.837	1.008	1.094	1.000	1.046	Q12933-2	TRAF2	Isoform 2 of TNF receptor-associated factor 2
1.019	1.168	0.998	0.987	0.779	1.485	0.869	0.755	1.633				O00463-2	TRAF5	Isoform 2 of TNF receptor-associated factor 5
0.981	0.975	1.008	0.983	0.959	0.990	1.022	0.965	0.969	1.062	0.981	1.077	Q12888-2	TP53BP1	Isoform 2 of TP53-binding protein 1
			1.080	0.878	1.023				0.914	1.209	1.087	O75865-2	TRAPPC6A	Isoform 2 of Trafficking protein particle complex subunit 6A
1.036	1.053	1.066	1.024	0.949	1.019	1.024	1.069	1.049	1.012	0.998	1.052	Q96Q05-2	TRAPPC9	Isoform 2 of Trafficking protein particle complex subunit 9
0.882	0.969	0.953	1.105	1.110	1.054	1.016	0.931	1.016	1.032	1.012	1.211	Q15170-2	TCEAL1	Isoform 2 of Transcription elongation factor A protein-like 1
0.946	1.004	1.027	1.018	1.007	1.105	1.033	0.836	0.996	1.054	1.004	1.076	Q96EI5-2	TCEAL4	Isoform 2 of Transcription elongation factor A protein-like 4

Abundance Ratio														
1 d M1/C1	1 d M2/C2	1 d M3/C3	4 d M1/C1	4 d M2/C2	4 d M3/C3	7 d M1/C1	7 d M2/C2	7 d M3/C3	10 d M1/C1	10 d M2/C2	10 d M3/C3	Accession ID	Gene name	Protein Description
0.993	1.101	0.983	1.008	0.875	0.984	1.057	0.928	1.046	0.886	1.178	1.223	Q7Z7C8-2	TAF8	Isoform 2 of Transcription initiation factor TFIID subunit 8
0.983	1.016	0.970	0.992	1.036	0.996	0.973	1.046	0.989	0.927	1.002	0.919	P37802-2	TAGLN2	Isoform 2 of Transgelin-2
1.012	1.011	0.988	1.006	1.010	0.987	0.962	1.030	0.991	0.961	1.018	0.944	P29401-2	TKT	Isoform 2 of Transketolase
0.897	0.955	1.028	0.900	0.908	0.965	0.846	0.649	1.029	1.026	1.046	1.066	Q9UNL2-2	SSR3	Isoform 2 of Translocon-associated protein subunit gamma
1.255	1.140	1.357	0.925	0.990	0.826	0.882	1.230	0.892	1.118	1.253	0.974	Q96DC7-2	TMCO6	Isoform 2 of Transmembrane and coiled-coil domain-containing protein 6
1.113	1.031	0.926	0.974	0.789	0.955	0.942	0.980	1.076				Q9Y3A6-2	TMED5	Isoform 2 of Transmembrane emp24 domain-containing protein 5
1.331	1.008	1.331	0.696	0.675	0.863	1.027	1.290	0.946				Q24JP5-2	TMEM132A	Isoform 2 of Transmembrane protein 132A
			0.969	0.897	1.159	0.888	1.165	1.365				Q9H813-2	TMEM206	Isoform 2 of Transmembrane protein 206
1.049	0.994	0.968	0.981	0.883	0.986	1.003	1.026	1.002	1.146	0.968	0.998	Q9H330-2	TMEM245	Isoform 2 of Transmembrane protein 245
			0.884	0.777	1.134							Q12767-2	TMEM94	Isoform 2 of Transmembrane protein 94
0.965	1.009	0.998	1.042	1.009	1.045	1.004	1.105	1.015	1.020	0.973	1.034	O14787-2	TNPO2	Isoform 2 of Transportin-2
0.981	0.971	1.039	1.021	0.929	1.013	1.019	1.012	0.948	1.069	1.002	1.072	Q9C040-2	TRIM2	Isoform 2 of Tripartite motif-containing protein 2
1.108	0.955	1.081	0.952	0.979	1.080	0.937	1.092	1.034	1.005	0.996	0.956	Q969Y2-2	GTPBP3	Isoform 2 of tRNA modification GTPase GTPBP3, mitochondrial
1.358	1.513	1.476	0.988	1.059	1.043	1.052	1.194	1.048	1.230	1.257	1.318	P17752-2	TPH1	Isoform 2 of Tryptophan 5-hydroxylase 1
1.033	0.928	1.020	1.012	0.997	0.997	0.932	1.103	0.981	1.055	1.050	1.332	O75386-2	TULP3	Isoform 2 of Tubby-related protein 3
0.969	1.086	0.986	1.050	1.139	1.032	1.129	0.794	1.039	1.048	1.059	0.961	Q86T03-2	TMEM55B	Isoform 2 of Type 1 phosphatidylinositol 4,5-bisphosphate 4-phosphatase
0.971	0.930	1.067	0.997	0.948	1.003	0.994	0.921	1.009	0.895	0.993	0.980	Q6RW13-2	AGTRAP	Isoform 2 of Type-1 angiotensin II receptor-associated protein
			1.188	1.237	0.983							P07948-2	LYN	Isoform 2 of Tyrosine-protein kinase Lyn
0.962	1.020	0.995	0.994	0.987	1.014	0.987	0.997	0.997	1.008	1.016	1.035	Q06124-2	PTPN11	Isoform 2 of Tyrosine-protein phosphatase non-receptor type 11
1.102	0.961	1.143	1.035	0.952	1.068	1.013	0.966	1.070	1.012	1.008	1.065	O95551-2	TDP2	Isoform 2 of Tyrosyl-DNA phosphodiesterase 2
0.967	0.999	0.982	0.970	0.980	0.990	1.004	0.961	0.943	1.065	1.022	1.037	P08621-2	SNRNP70	Isoform 2 of U1 small nuclear ribonucleoprotein 70 kDa
1.132	1.040	1.160	0.973	0.980	1.168	0.943	1.120	1.015				Q16560-2	SNRNP35	Isoform 2 of U11/U12 small nuclear ribonucleoprotein 35 kDa protein
1.073	1.026	1.006	1.048	1.007	0.975	1.024	1.062	1.003	1.025	1.013	1.015	Q14694-2	USP10	Isoform 2 of Ubiquitin carboxyl-terminal hydrolase 10
0.986	1.023	1.112	1.001	0.888	0.947	0.993	1.102	1.040	1.034	1.028	1.068	Q9P275-2	USP36	Isoform 2 of Ubiquitin carboxyl-terminal hydrolase 36
1.000	1.008	1.038	1.018	0.991	1.030	1.022	1.059	0.995	1.011	1.037	1.002	Q14139-2	UBE4A	Isoform 2 of Ubiquitin conjugation factor E4 A
0.980	1.177	1.008										Q14157-1	UBAP2L	Isoform 2 of Ubiquitin-associated protein 2-like

Abundance Ratio														
1 d M1/C1	1 d M2/C2	1 d M3/C3	4 d M1/C1	4 d M2/C2	4 d M3/C3	7 d M1/C1	7 d M2/C2	7 d M3/C3	10 d M1/C1	10 d M2/C2	10 d M3/C3	Accession ID	Gene name	Protein Description
1.070	0.950	1.049	0.967	0.943	0.978	1.057	0.965	1.068	1.122	0.964	1.100	Q9BTM9-2	URM1	Isoform 2 of Ubiquitin-related modifier 1
0.979	1.006	1.033	0.951	0.948	1.051	0.986	1.074	1.005	0.991	0.947	1.034	Q8NBZ7-2	UXS1	Isoform 2 of UDP-glucuronic acid decarboxylase 1
1.200	1.108	0.838	0.994	0.820	1.068							Q9Y2D2-2	SLC35A3	Isoform 2 of UDP-N-acetylglucosamine transporter
0.862	0.924	1.071	1.018	0.901	1.104	1.000	0.850	0.982	1.094	1.069	1.125	Q96D05-2	C10orf35	Isoform 2 of Uncharacterized protein C10orf35
1.052	0.898	0.975	0.878	0.919	1.189	1.014	0.982	1.080	0.866	0.905	1.029	Q6ZUT1-2	C11orf57	Isoform 2 of Uncharacterized protein C11orf57
0.943	1.115	1.062	0.999	0.917	1.032	1.047	1.017	1.114	1.062	1.054	1.066	Q9BV29-2	C15orf57	Isoform 2 of Uncharacterized protein C15orf57
0.897	0.957	0.961	1.004	0.979	1.079	1.094	1.011	0.854	1.562	1.035	1.199	Q5SNV9-2	C1orf167	Isoform 2 of Uncharacterized protein C1orf167
2.798	3.001	2.739	5.166	4.952	4.623	6.679	4.357	4.313	5.310	3.574	5.205	Q17RF5-2	C4orf26	Isoform 2 of Uncharacterized protein C4orf26
1.069	1.120	0.919	0.838	0.844	0.900	1.043	0.888	0.915	1.000	1.198	1.043	Q8WVX3-2	C4orf3	Isoform 2 of Uncharacterized protein C4orf3
1.072	0.957	1.029	0.962	0.942	0.987	1.014	0.966	0.980	1.032	0.997	1.065	Q9P206-2	KIAA1522	Isoform 2 of Uncharacterized protein KIAA1522
0.962	1.027	1.016	1.022	0.981	1.147	1.002	1.108	1.072	1.033	1.039	1.087	Q8IYS2-2	KIAA2013	Isoform 2 of Uncharacterized protein KIAA2013
1.148	1.104	0.879										O43795-2	MYO1B	Isoform 2 of Unconventional myosin-Ib
0.998	0.998	1.025	1.012	0.991	1.032	1.062	0.951	0.935	0.990	0.986	0.992	Q7Z3D6-2	C14orf159	Isoform 2 of UPF0317 protein C14orf159, mitochondrial
0.965	1.030	1.015	0.989	0.938	0.973	1.001	1.143	0.992	0.901	1.038	0.973	Q9BPX7-2	C7orf25	Isoform 2 of UPF0415 protein C7orf25
						1.146	1.133	1.023				Q7Z6I8-2	C5orf24	Isoform 2 of UPF0461 protein C5orf24
1.013	1.039	0.987	1.019	0.914	1.022	0.999	1.134	0.967	1.019	1.029	0.967	Q9GZN8-2	C20orf27	Isoform 2 of UPF0687 protein C20orf27
1.015	0.907	0.987	0.952	0.926	0.965	0.909	0.871	1.002	1.042	0.958	1.029	Q15906-2	VPS72	Isoform 2 of Vacuolar protein sorting-associated protein 72 homolog
0.970	0.989	0.998	0.992	0.944	0.992	1.047	1.137	1.029	1.050	1.081	1.005	Q9H0V9-2	LMAN2L	Isoform 2 of VIP36-like protein
									1.140	0.576	1.146	P11473-2	VDR	Isoform 2 of Vitamin D3 receptor
1.078	1.008	1.043	0.999	0.983	1.020	1.012	1.027	1.039	1.011	1.054	1.015	Q13303-2	KCNAB2	Isoform 2 of Voltage-gated potassium channel subunit beta-2
0.941	0.984	1.020	1.034	1.007	1.050	0.984	1.057	1.024	0.987	0.975	1.038	Q16864-2	ATP6V1F	Isoform 2 of V-type proton ATPase subunit F
1.093	1.011	1.105	1.008	1.028	1.001	1.009	1.008	1.008	1.028	1.039	1.021	Q9Y2I8-2	WDR37	Isoform 2 of WD repeat-containing protein 37
						1.308	0.939	1.128				P16989-2	YBX3	Isoform 2 of Y-box-binding protein 3
1.377	1.269	0.940	1.018	1.127	0.749	1.070	1.370	0.892	0.753	1.088	0.853	Q8IY57-5	YAF2	Isoform 2 of YY1-associated factor 2
1.213	1.026	1.041										O15156-2	ZBTB7B	Isoform 2 of Zinc finger and BTB domain-containing protein 7B
			1.000	1.062	0.937	1.007	0.937	1.329				Q5T200-2	ZC3H13	Isoform 2 of Zinc finger CCCH domain-containing protein 13
0.946	0.985	1.042	0.982	0.923	1.043	1.010	1.050	1.020	1.000	1.004	1.020	Q8N5A5-2	ZGPAT	Isoform 2 of Zinc finger CCCH-type with G patch domain-containing protein
0.920	0.933	1.094	1.051	0.889	1.070	0.882	1.126	1.112				P37275-2	ZEB1	Isoform 2 of Zinc finger E-box-binding homeobox 1

Abundance Ratio														
1 d M1/C1	1 d M2/C2	1 d M3/C3	4 d M1/C1	4 d M2/C2	4 d M3/C3	7 d M1/C1	7 d M2/C2	7 d M3/C3	10 d M1/C1	10 d M2/C2	10 d M3/C3	Accession ID	Gene name	Protein Description
0.987	0.834	1.043	0.974	0.893	0.984	1.038	1.045	0.979	1.043	1.015	1.031	Q9BQ24-2	ZFYVE21	Isoform 2 of Zinc finger FYVE domain-containing protein 21
0.949	0.903	1.054	0.904	0.995	1.095	0.994	0.861	0.955	1.073	0.957	1.226	Q9Y6Q3-2	ZFP37	Isoform 2 of Zinc finger protein 37 homolog
1.019	1.006	0.987	1.044	0.982	0.966	1.050	0.895	0.990	0.995	0.996	0.986	Q8TF68-2	ZNF384	Isoform 2 of Zinc finger protein 384
1.191	0.920	0.813	1.057	0.861	1.119	1.650	0.628	1.092	2.414	0.710	0.932	Q96IQ9-2	ZNF414	Isoform 2 of Zinc finger protein 414
			0.946	1.008	1.101	0.889	1.486	0.820				Q8TAQ5-2	ZNF420	Isoform 2 of Zinc finger protein 420
			1.008	1.025	0.948							Q96K58-2	ZNF668	Isoform 2 of Zinc finger protein 668
1.025	1.086	1.006	1.099	1.065	1.056	0.930	0.712	0.988				P17097-2	ZNF7	Isoform 2 of Zinc finger protein 7
0.960	1.043	0.881										Q9UHF7-2	TRPS1	Isoform 2 of Zinc finger transcription factor Trps1
1.071	1.111	1.018	0.944	0.841	0.972	0.775	1.025	0.901	1.084	0.937	1.003	Q6NXT4-2	SLC30A6	Isoform 2 of Zinc transporter 6
						0.983	1.206	1.291	0.904	1.011	1.008	O15527-4	OGG1	Isoform 2A of N-glycosylase/DNA lyase
0.962	0.992	0.998	0.985	0.959	1.006	1.009	0.986	0.947	1.023	0.993	1.027	Q13409-2	DYNC1I2	Isoform 2B of Cytoplasmic dynein 1 intermediate chain 2
0.992	0.970	0.954	1.004	0.988	0.952	1.001	1.046	0.980	1.003	1.001	0.981	P01116-2	KRAS	Isoform 2B of GTPase KRas
1.118	0.970	1.009	1.070	0.923	1.006	1.123	1.077	1.028	1.128	0.998	1.045	Q9NRZ7-3	AGPAT3	Isoform 3 of 1-acyl-sn-glycerol-3-phosphate acyltransferase gamma
0.996	1.014	1.007	0.996	0.949	0.967	1.048	1.070	1.050	0.967	1.013	1.033	Q15147-4	PLCB4	Isoform 3 of 1-phosphatidylinositol 4,5-bisphosphate phosphodiesterase beta-4
0.919	0.890	1.309										P04035-3	HMGCR	Isoform 3 of 3-hydroxy-3-methylglutaryl-coenzyme A reductase
0.996	1.019	0.988	0.998	0.987	1.006	0.969	1.008	0.970	0.982	1.028	0.949	P18621-3	RPL17	Isoform 3 of 60S ribosomal protein L17
			0.902	0.898	1.008	0.987	1.037	0.790				Q9BTT0-3	ANP32E	Isoform 3 of Acidic leucine-rich nuclear phosphoprotein 32 family member E
0.976	0.999	1.008	0.977	0.964	0.990	0.990	1.034	1.004	0.973	0.998	1.023	P59998-3	ARPC4	Isoform 3 of Actin-related protein 2/3 complex subunit 4
0.996	0.977	1.010	1.001	0.927	0.974	0.921	1.064	1.156	0.829	1.026	1.018	Q6VMQ6-4	ATF7IP	Isoform 3 of Activating transcription factor 7-interacting protein 1
1.017	1.006	1.035	0.988	0.921	0.995	0.967	0.966	0.977	0.946	0.995	1.053	Q5T8D3-3	ACBD5	Isoform 3 of Acyl-CoA-binding domain-containing protein 5
1.094	1.005	1.064	1.104	0.820	1.013	1.000	1.126	1.172	1.211	1.146	1.081	Q6UY14-3	ADAMTSL4	Isoform 3 of ADAMTS-like protein 4
0.993	1.000	0.981	1.046	1.020	1.023	1.062	1.100	1.053	1.027	0.963	1.125	Q86SQ4-3	ADGRG6	Isoform 3 of Adhesion G-protein coupled receptor G6
									1.071	0.953	1.010	Q66PJ3-3	ARL6IP4	Isoform 3 of ADP-ribosylation factor-like protein 6-interacting protein 4
1.006	1.006	0.982	1.012	0.982	1.043	1.039	1.175	1.079	1.027	1.020	1.052	Q9BTE6-3	AARSD1	Isoform 3 of Alanyl-tRNA editing protein Aarsd1
1.037	0.970	0.948	1.254	1.212	1.225	1.063	0.817	0.682	0.991	1.147	1.026	Q6UW56-3	ATRAID	Isoform 3 of All-trans retinoic acid-induced differentiation factor

Abundance Ratio														
1 d M1/C1	1 d M2/C2	1 d M3/C3	4 d M1/C1	4 d M2/C2	4 d M3/C3	7 d M1/C1	7 d M2/C2	7 d M3/C3	10 d M1/C1	10 d M2/C2	10 d M3/C3	Accession ID	Gene name	Protein Description
0.990	1.007	1.016	0.890	0.754	0.901	0.945	0.954	0.898	1.095	0.912	1.036	Q9UQ53-3	MGAT4B	Isoform 3 of Alpha-1,3-mannosyl-glycoprotein 4-beta-N-acetylglucosaminyltransferase B
1.034	1.025	1.029	1.007	0.999	1.012	1.005	0.937	1.056	1.083	0.980	1.070	P35611-3	ADD1	Isoform 3 of Alpha-adducin
1.004	1.063	1.046	0.965	0.918	1.011	1.058	1.157	0.997	0.934	0.986	1.038	Q9UJX5-3	ANAPC4	Isoform 3 of Anaphase-promoting complex subunit 4
1.017	0.895	1.010										Q68DC2-4	ANKS6	Isoform 3 of Ankyrin repeat and SAM domain-containing protein 6
1.042	1.141	0.846	1.009	0.872	1.215	0.923	1.147	1.058	1.006	0.916	0.943	Q9Y575-3	ASB3	Isoform 3 of Ankyrin repeat and SOCS box protein 3
0.942	1.233	1.197	0.811	1.055	1.097	1.015	1.196	1.153				Q6ZTN6-3	ANKRD13D	Isoform 3 of Ankyrin repeat domain-containing protein 13D
1.051	1.114	0.987	0.998	0.963	1.002	1.000	1.122	0.999	0.983	0.989	1.018	Q13625-3	TP53BP2	Isoform 3 of Apoptosis-stimulating of p53 protein 2
1.007	1.012	1.024	1.004	1.006	1.024	0.992	1.036	0.981	1.007	1.031	1.056	Q9Y2X7-3	GIT1	Isoform 3 of ARF GTPase-activating protein GIT1
1.022	1.004	1.030	1.014	0.952	1.025	1.025	1.009	1.019	1.014	0.939	1.038	Q96P48-3	ARAP1	Isoform 3 of Arf-GAP with Rho-GAP domain, ANK repeat and PH domain-containing protein 1
0.989	1.004	0.987	0.994	0.975	1.004	0.995	1.075	1.005	1.004	0.989	1.008	P08237-3	PFKM	Isoform 3 of ATP-dependent 6-phosphofructokinase, muscle type
1.073	0.989	1.024	1.020	0.898	0.999	1.042	0.992	1.006	1.077	1.027	1.044	Q8NFD5-3	ARID1B	Isoform 3 of AT-rich interactive domain-containing protein 1B
1.016	1.076	1.014	1.062	0.969	0.981	1.005	1.126	1.026	1.042	1.112	0.973	Q8NFJ9-2	BBS1	Isoform 3 of Bardet-Biedl syndrome 1 protein
1.000	0.983	1.004	0.952	0.943	1.000	0.953	0.965	0.951	0.947	0.973	0.948	Q7L1Q6-3	BZW1	Isoform 3 of Basic leucine zipper and W2 domain-containing protein 1
0.986	0.975	0.976	0.972	0.933	1.001	1.015	0.979	0.990	1.023	1.018	1.013	Q92843-2	BCL2L2	Isoform 3 of Bcl-2-like protein 2
						1.174	1.151	0.899	1.200	1.015	1.035	Q5H9F3-3	BCORL1	Isoform 3 of BCL-6 corepressor-like protein 1
1.000	0.987	0.980	1.031	0.934	0.924	0.992	1.113	1.032	0.988	0.972	0.993	Q6NYC1-3	JMJD6	Isoform 3 of Bifunctional arginine demethylase and lysyl-hydroxylase JMJD6
1.058	0.956	1.061	0.952	0.918	0.970	0.934	1.227	1.112	0.915	0.822	0.925	Q8TDM0-3	BCAS4	Isoform 3 of Breast carcinoma-amplified sequence 4
1.020	0.985	1.008	0.962	0.964	1.010	1.008	1.038	1.045	1.043	0.993	1.032	Q86YS7-3	C2CD5	Isoform 3 of C2 domain-containing protein 5
1.002	0.933	1.043							1.079	1.004	1.109	Q6KCM7-3	SLC25A25	Isoform 3 of Calcium-binding mitochondrial carrier protein SCaMC-2
0.985	1.001	1.060	1.032	0.912	1.008	1.047	1.083	1.044	1.081	1.061	1.080	P98194-3	ATP2C1	Isoform 3 of Calcium-transporting ATPase type 2C member 1
			1.153	1.030	1.185	0.877	0.795	0.987	1.097	1.008	1.340	Q05682-3	CALD1	Isoform 3 of Caldesmon
0.990	0.975	1.012	0.982	0.990	1.046	1.023	1.031	0.984	1.039	1.057	1.067	Q5T5Y3-3	CAMSAP1	Isoform 3 of Calmodulin-regulated spectrin-associated protein 1
0.985	1.016	0.978	0.990	0.976	0.991	0.955	1.013	0.983	0.962	0.992	0.972	O43852-3	CALU	Isoform 3 of Calumenin

Abundance Ratio														
1 d M1/C1	1 d M2/C2	1 d M3/C3	4 d M1/C1	4 d M2/C2	4 d M3/C3	7 d M1/C1	7 d M2/C2	7 d M3/C3	10 d M1/C1	10 d M2/C2	10 d M3/C3	Accession ID	Gene name	Protein Description
0.969	0.996	1.010	0.995	0.960	1.029	1.007	1.016	1.023	1.036	1.000	1.066	P30622-2	CLIP1	Isoform 3 of CAP-Gly domain-containing linker protein 1
0.896	0.886	1.070	0.936	0.827	1.035	1.015	1.022	0.955	0.983	0.946	1.107	P31327-3	CPS1	Isoform 3 of Carbamoyl-phosphate synthase [ammonia], mitochondrial
			1.057	0.875	1.224							P48729-3	CSNK1A1	Isoform 3 of Casein kinase I isoform alpha
1.016	0.993	1.022	0.973	0.938	1.059	1.031	1.045	1.007	1.017	1.008	1.058	Q96SZ6-3	CDK5RAP1	Isoform 3 of CDK5 regulatory subunit-associated protein 1
0.863	1.055	1.068				1.112	1.016	1.006				O75419-3	CDC45	Isoform 3 of Cell division control protein 45 homolog
0.990	1.062	0.973				1.004	1.136	1.023				Q96H22-3	CENPN	Isoform 3 of Centromere protein N
1.087	1.018	1.134	0.910	0.923	1.022	0.844	1.056	0.689	0.981	0.901	0.955	Q8TEP8-3	CEP192	Isoform 3 of Centrosomal protein of 192 kDa
1.018	0.966	0.992	0.986	0.970	1.054	1.022	1.094	1.048	0.980	1.079	0.986	Q8IXM2-3	BAP18	Isoform 3 of Chromatin complexes subunit BAP18
1.069	0.948	1.019	1.015	0.965	0.940	0.953	0.891	0.966	1.424	0.711	0.968	Q9HAF1-3	MEAF6	Isoform 3 of Chromatin modification-related protein MEAF6
0.996	0.984	0.980	1.004	0.984	1.019	1.036	1.071	1.045	1.015	0.983	1.042	Q12873-3	CHD3	Isoform 3 of Chromodomain-helicase-DNA-binding protein 3
1.045	1.006	1.015	1.020	0.999	0.988	1.014	1.009	0.983	1.004	0.996	1.001	Q8N684-3	CPSF7	Isoform 3 of Cleavage and polyadenylation specificity factor subunit 7
0.995	0.978	1.019	0.985	0.963	0.987	1.008	1.002	1.008	0.992	1.042	1.041	O75122-3	CLASP2	Isoform 3 of CLIP-associating protein 2
1.041	1.021	1.004	1.021	1.013	1.022	1.047	0.997	1.027	1.075	1.003	1.086	Q9Y5P4-3	COL4A3BP	Isoform 3 of Collagen type IV alpha-3-binding protein
0.968	0.992	0.968	0.984	1.065	0.974	0.998	0.965	0.998	0.987	1.003	0.992	Q9ULV4-3	CORO1C	Isoform 3 of Coronin-1C
1.048	1.005	1.002	1.001	0.963	0.992	1.028	0.971	0.963	1.031	0.993	1.034	P57737-3	CORO7	Isoform 3 of Coronin-7
1.131	1.094	0.982	0.987	0.986	0.949	1.032	1.147	0.998	0.974	1.007	0.988	Q9BW66-3	CINP	Isoform 3 of Cyclin-dependent kinase 2-interacting protein
1.094	1.029	1.008	1.006	0.975	1.086	0.998	1.007	1.014	0.925	0.913	0.989	Q9Y600-3	CSAD	Isoform 3 of Cysteine sulfinic acid decarboxylase
0.999	1.011	1.028	1.001	0.985	1.007	0.973	1.070	1.027	0.963	0.996	0.990	P49589-3	CARS	Isoform 3 of Cysteine--tRNA ligase, cytoplasmic
1.027	1.007	1.004	0.984	0.984	1.039	0.993	1.068	1.043	1.060	1.024	1.059	Q8NEU8-3	APPL2	Isoform 3 of DCC-interacting protein 13-beta
1.232	0.960	1.107	1.092	0.910	1.182	1.129	0.988	1.159	0.822	1.021	1.052	Q92564-3	DCUN1D4	Isoform 3 of DCN1-like protein 4
1.012	1.004	1.055	1.011	0.898	1.005	0.898	1.032	0.989	1.028	0.998	1.002	Q58WW2-3	DCAF6	Isoform 3 of DDB1- and CUL4-associated factor 6
			0.628	0.553	1.082							P53355-3	DAPK1	Isoform 3 of Death-associated protein kinase 1
0.963	0.990	1.010	1.018	1.084	0.985	0.987	1.071	1.042	1.018	0.953	1.037	Q8TDJ6-3	DMXL2	Isoform 3 of DmX-like protein 2
0.904	0.943	1.196				1.081	0.873	1.193				P18858-3	LIG1	Isoform 3 of DNA ligase 1
1.008	0.979	1.061	0.992	1.015	1.036	0.990	1.025	1.008	0.969	0.998	1.019	Q9H9Y6-3	POLR1B	Isoform 3 of DNA-directed RNA polymerase I subunit RPA2
0.986	0.974	1.038	1.005	0.985	1.070	0.996	1.019	1.011	1.037	1.024	1.128	Q5F1R6-3	DNAJC21	Isoform 3 of DnaJ homolog subfamily C member 21

Abundance Ratio														
1 d M1/C1	1 d M2/C2	1 d M3/C3	4 d M1/C1	4 d M2/C2	4 d M3/C3	7 d M1/C1	7 d M2/C2	7 d M3/C3	10 d M1/C1	10 d M2/C2	10 d M3/C3	Accession ID	Gene name	Protein Description
0.996	0.987	1.004	0.988	1.005	1.022	0.995	0.961	1.000	1.013	1.008	1.028	P49959-3	MRE11	Isoform 3 of Double-strand break repair protein MRE11
									0.999	1.105	1.064	O95793-3	STAU1	Isoform 3 of Double-stranded RNA-binding protein Staufen homolog 1
0.959	0.969	1.006	0.986	1.014	0.974	0.990	0.938	0.977	1.018	0.983	1.034	Q16643-3	DBN1	Isoform 3 of Drebrin
1.104	1.049	1.009	1.000	0.934	0.996	1.073	0.996	1.021	1.115	1.040	1.051	O14733-3	MAP2K7	Isoform 3 of Dual specificity mitogen-activated protein kinase kinase 7
0.874	0.920	0.939	1.088	0.947	1.130	1.030	1.099	0.986	0.962	0.976	0.985	P49759-3	CLK1	Isoform 3 of Dual specificity protein kinase CLK1
1.089	0.865	1.095	0.755	0.974	0.929	0.931	0.945	1.002	1.050	0.917	1.287	Q9H9R9-3	DBNDD1	Isoform 3 of Dysbindin domain-containing protein 1
1.039	0.992	1.015	1.017	0.973	1.057	0.992	1.018	1.028	0.948	0.990	1.016	O94822-3	LTN1	Isoform 3 of E3 ubiquitin-protein ligase listerin
			0.998	0.986	0.944							O60858-3	TRIM13	Isoform 3 of E3 ubiquitin-protein ligase TRIM13
1.001	1.010	1.027	0.988	0.989	0.994	0.985	1.040	1.019	0.941	0.925	1.056	Q14669-3	TRIP12	Isoform 3 of E3 ubiquitin-protein ligase TRIP12
0.974	0.939	1.097	1.038	0.993	1.148	0.962	1.129	1.120	1.011	1.001	1.007	O94874-3	UFL1	Isoform 3 of E3 UFM1-protein ligase 1
1.034	1.035	1.012	1.030	1.008	1.018	1.041	0.954	0.998	1.000	0.971	0.998	O95834-3	EML2	Isoform 3 of Echinoderm microtubule-associated protein-like 2
1.042	0.964	1.023	1.001	0.996	1.020	1.039	0.998	1.095	1.106	1.058	1.124	Q99447-3	PCYT2	Isoform 3 of Ethanolamine-phosphate cytidylyltransferase
1.006	0.999	1.015	0.996	1.006	0.969	0.987	0.964	0.976	0.971	0.918	0.986	P15170-3	GSPT1	Isoform 3 of Eukaryotic peptide chain release factor GTP-binding subunit ERF3A
1.147	0.796	1.221	0.943	0.872	1.001	0.999	0.986	1.058	1.076	0.984	1.055	Q9NRA8-3	EIF4ENIF1	Isoform 3 of Eukaryotic translation initiation factor 4E transporter
1.002	1.053	0.979	1.061	0.977	0.961	1.069	1.156	0.896	0.914	0.922	0.988	Q93063-3	EXT2	Isoform 3 of Exostosin-2
1.006	1.014	1.024	1.022	1.055	0.989	1.035	1.014	1.005	0.982	0.972	0.992	Q96AC1-3	FERMT2	Isoform 3 of Fermitin family homolog 2
1.109	1.075	1.017	0.990	0.872	1.118	0.961	1.107	0.975	1.020	0.952	0.971	O95466-3	FMNL1	Isoform 3 of Formin-like protein 1
0.979	1.013	1.071	1.016	1.040	1.059	1.023	1.020	1.056	1.071	1.036	1.129	Q5T3I0-3	GPATCH4	Isoform 3 of G patch domain-containing protein 4
1.023	1.032	1.013	1.053	0.955	1.014	1.041	1.083	1.007	1.001	1.008	1.030	Q9BSJ2-4	TUBGCP2	Isoform 3 of Gamma-tubulin complex component 2
1.061	1.033	0.996	1.033	0.988	1.010	1.098	1.044	1.030	1.051	1.024	1.091	Q9Y5Q8-3	GTF3C5	Isoform 3 of General transcription factor 3C polypeptide 5
1.084	1.043	1.121	1.159	1.122	1.161	1.144	1.213	1.102	1.074	1.074	1.171	P07093-3	SERPINE2	Isoform 3 of Glia-derived nexin
0.938	1.005	0.976	0.959	0.897	0.930	0.977	0.881	0.920	0.975	0.790	0.953	O94925-3	GLS	Isoform 3 of Glutaminase kidney isoform, mitochondrial
0.973	0.995	0.987	0.973	0.944	1.001	0.974	0.986	1.017	1.010	0.974	1.003	Q14451-3	GRB7	Isoform 3 of Growth factor receptor-bound protein 7
0.978	0.963	1.031	1.094	1.019	1.125	1.038	1.302	1.038	0.937	0.963	1.045	Q92963-3	RIT1	Isoform 3 of GTP-binding protein Rit1
			0.895	0.978	1.034							P63092-3	GNAS	Isoform 3 of Guanine nucleotide-binding protein G(s) subunit alpha isoforms short

Abundance Ratio														
1 d M1/C1	1 d M2/C2	1 d M3/C3	4 d M1/C1	4 d M2/C2	4 d M3/C3	7 d M1/C1	7 d M2/C2	7 d M3/C3	10 d M1/C1	10 d M2/C2	10 d M3/C3	Accession ID	Gene name	Protein Description
0.990	0.996	1.005	0.968	0.962	0.961	1.002	0.931	0.939	1.026	0.953	1.061	Q9H910-3	HN1L	Isoform 3 of Hematological and neurological expressed 1-like protein
1.020	1.010	0.986	0.973	0.978	1.006	0.916	0.909	0.949	0.961	0.984	0.921	Q99729-3	HNRNPAB	Isoform 3 of Heterogeneous nuclear ribonucleoprotein A/B
1.050	0.996	1.019	0.988	1.190	0.984	0.969	0.860	1.059	0.943	0.930	0.943	Q14103-3	HNRNPD	Isoform 3 of Heterogeneous nuclear ribonucleoprotein D0
1.000	1.064	1.008	1.026	1.032	0.938	1.020	1.050	1.023	0.980	0.938	0.955	P61978-3	HNRNPK	Isoform 3 of Heterogeneous nuclear ribonucleoprotein K
0.927	1.015	1.223	0.984	0.842	1.123	0.916	1.021	1.159	1.070	1.004	1.026	O60506-3	SYNCRIP	Isoform 3 of Heterogeneous nuclear ribonucleoprotein Q
0.948	0.991	1.011	0.964	0.982	1.006	0.961	0.899	0.968	1.005	0.952	1.034	P19367-3	HK1	Isoform 3 of Hexokinase-1
0.963	0.904	1.218	1.018	0.930	1.067	0.923	1.049	1.340	1.013	0.884	1.371	Q92993-3	KAT5	Isoform 3 of Histone acetyltransferase KAT5
1.208	1.058	0.873	0.945	1.014	1.101	0.956	1.125	1.055	1.026	0.828	0.823	Q9UQL6-3	HDAC5	Isoform 3 of Histone deacetylase 5
1.023	1.013	0.972	1.030	0.966	1.003	0.994	0.959	1.009	1.078	1.007	1.080	Q9H0E3-3	SAP130	Isoform 3 of Histone deacetylase complex subunit SAP130
1.015	0.987	1.035	0.972	0.960	1.013	1.032	0.977	0.987	1.017	1.043	1.036	Q03164-3	KMT2A	Isoform 3 of Histone-lysine N-methyltransferase 2A
1.062	0.792	0.847	1.067	0.958	0.979	1.008	1.134	1.063	1.123	0.946	1.056	O14686-3	KMT2D	Isoform 3 of Histone-lysine N-methyltransferase 2D
1.135	1.075	1.077	0.988	1.472	0.823	0.975	1.006	1.035	0.858	1.123	0.868	P39880-3	CUX1	Isoform 3 of Homeobox protein cut-like 1
0.980	1.018	1.001	0.994	1.041	1.028	1.010	1.000	1.038	0.972	0.999	1.018	Q9NZL4-3	HSPBP1	Isoform 3 of Hsp70-binding protein 1
0.922	0.947	1.038				0.947	1.080	0.937	0.974	0.872	1.031	O75330-3	HMMR	Isoform 3 of Hyaluronan mediated motility receptor
1.043	1.015	1.013	1.038	0.977	1.026	0.966	1.124	1.005	0.981	0.972	0.997	Q9BYI3-3	FAM126A	Isoform 3 of Hyccin
0.988	1.006	1.009	1.000	1.028	1.022	1.009	1.038	1.040	0.965	0.999	0.993	O00410-3	IPO5	Isoform 3 of Importin-5
			0.895	0.967	0.889	1.055	1.195	0.857	1.175	1.181	1.048	Q6PI98-4	INO80C	Isoform 3 of INO80 complex subunit C
1.019	0.986	1.025	0.966	1.033	0.963	0.964	1.004	1.000	0.970	1.009	1.001	P29218-3	IMPA1	Isoform 3 of Inositol monophosphatase 1
1.055	1.023	1.024	0.991	1.064	1.031	0.884	0.978	0.997	1.030	1.033	0.974	P01344-3	IGF2	Isoform 3 of Insulin-like growth factor II
1.033	1.009	1.032	1.006	0.977	1.028	1.022	1.095	0.974	1.018	1.000	1.042	Q6P597-3	KLC3	Isoform 3 of Kinesin light chain 3
1.076	0.978	1.047	1.009	0.843	1.029	1.008	0.927	0.982	1.045	1.023	1.001	Q9NSK0-3	KLC4	Isoform 3 of Kinesin light chain 4
1.020	0.972	1.055	0.937	0.936	1.102	1.014	1.024	1.008	0.972	1.061	1.086	Q9H1H9-3	KIF13A	Isoform 3 of Kinesin-like protein KIF13A
0.953	0.992	1.069	0.981	0.947	1.022	1.002	1.028	1.012	1.002	0.913	1.027	O60333-3	KIF1B	Isoform 3 of Kinesin-like protein KIF1B
1.032	1.009	1.004	1.025	0.988	1.017	1.054	1.026	1.010	1.066	1.029	1.062	Q4G0J3-3	LARP7	Isoform 3 of La-related protein 7
0.998	1.024	1.021	0.984	0.940	1.012	1.027	1.052	1.004	0.982	0.965	1.053	P46379-3	BAG6	Isoform 3 of Large proline-rich protein BAG6
0.955	0.998	0.988	0.991	0.983	1.008	1.008	0.935	0.986	1.045	0.983	1.068	Q32MZ4-3	LRRFIP1	Isoform 3 of Leucine-rich repeat flightless-interacting protein 1
1.061	0.973	1.040	1.008	1.027	1.017	1.072	0.880	1.003	0.995	0.955	1.030	P48059-3	LIMS1	Isoform 3 of LIM and senescent cell antigen-like-containing domain protein 1

Abundance Ratio														
1 d M1/C1	1 d M2/C2	1 d M3/C3	4 d M1/C1	4 d M2/C2	4 d M3/C3	7 d M1/C1	7 d M2/C2	7 d M3/C3	10 d M1/C1	10 d M2/C2	10 d M3/C3	Accession ID	Gene name	Protein Description
1.160	0.894	0.764	0.975	0.912	0.895	0.998	1.356	1.054	0.980	0.907	0.966	P53671-3	LIMK2	Isoform 3 of LIM domain kinase 2
0.987	1.121	0.984										Q8WWI1-3	LMO7	Isoform 3 of LIM domain only protein 7
1.051	1.011	1.024	1.022	1.074	1.017	0.998	0.990	1.007	0.998	0.978	0.981	P00338-3	LDHA	Isoform 3 of L-lactate dehydrogenase A chain
			0.880	0.817	0.901	0.894	1.171	1.005	0.927	0.831	1.031	Q6NUT3-3	MFSD12	Isoform 3 of Major facilitator superfamily domain-containing protein 12
1.009	0.988	1.007	1.006	1.082	0.975	0.998	0.934	0.990	1.013	0.974	0.984	P40925-3	MDH1	Isoform 3 of Malate dehydrogenase, cytoplasmic
0.953	1.012	0.987	1.091	1.103	1.103	1.019	1.070	1.013	1.032	1.069	1.052	Q9ULC4-3	MCTS1	Isoform 3 of Malignant T-cell-amplified sequence 1
1.001	1.033	1.022	0.986	0.969	1.003	0.998	1.023	1.075	1.009	0.995	1.049	Q9Y2X0-3	MED16	Isoform 3 of Mediator of RNA polymerase II transcription subunit 16
0.989	1.082	1.071	1.078	1.035	0.977	0.990	1.060	1.108	1.096	1.046	1.083	Q71SY5-3	MED25	Isoform 3 of Mediator of RNA polymerase II transcription subunit 25
1.076	1.016	0.981	0.986	0.984	1.047	1.006	0.931	1.079	1.032	1.006	1.067	Q2M296-3	MTHFSD	Isoform 3 of Methenyltetrahydrofolate synthase domain-containing protein
1.049	1.017	1.038	1.045	0.993	1.067	1.085	1.007	0.933	1.071	1.037	1.076	Q9H7H0-3	METTL17	Isoform 3 of Methyltransferase-like protein 17, mitochondrial
0.993	0.967	1.001	0.966	0.939	0.986	1.017	0.946	1.018	1.017	0.996	1.064	Q9UPN3-3	MACF1	Isoform 3 of Microtubule-actin cross-linking factor 1, isoforms 1/2/3/5
1.021	0.976	1.006	0.955	1.028	1.018	1.012	0.961	1.051	1.023	1.001	1.065	A9UHW6-3	MIF4GD	Isoform 3 of MIF4G domain-containing protein
1.233	0.897	1.488										Q96C03-3	MIEF2	Isoform 3 of Mitochondrial dynamics protein MID49
1.003	1.006	1.018	0.997	0.959	1.028	1.041	1.118	1.085	1.015	0.975	1.052	Q8IXI2-3	RHOT1	Isoform 3 of Mitochondrial Rho GTPase 1
1.009	1.005	1.012	0.995	0.917	0.920	1.065	1.095	1.030	0.732	0.772	0.948	O60566-3	BUB1B	Isoform 3 of Mitotic checkpoint serine/threonine-protein kinase BUB1 beta
1.023	1.101	1.120	1.001	0.961	0.934	0.981	1.077	0.981				Q9ULH7-3	MKL2	Isoform 3 of MKL/myocardin-like protein 2
0.936	1.034	0.984	0.978	0.971	1.065	1.069	1.001	0.999	1.079	0.995	1.084	Q70IA6-3	MOB2	Isoform 3 of MOB kinase activator 2
1.170	1.023	1.119	1.112	0.891	0.934	1.031	0.905	0.986	0.949	1.012	1.066	Q8N5Y8-3	PARP16	Isoform 3 of Mono [ADP-ribose] polymerase PARP16
									0.784	0.998	1.001	Q9UJG1-3	MOSPD1	Isoform 3 of Motile sperm domain-containing protein 1
1.101	0.903	0.913	0.988	1.032	0.941	0.937	1.050	0.997	1.026	1.061	1.077	P58340-3	MLF1	Isoform 3 of Myeloid leukemia factor 1
						1.004	1.507	1.050	1.016	1.045	0.941	Q9NZM1-3	MYOF	Isoform 3 of Myoferlin
1.047	0.975	1.130	1.065	0.959	0.979	1.042	0.996	1.001	1.032	1.066	1.181	P35580-3	MYH10	Isoform 3 of Myosin-10
0.966	0.996	1.059	0.990	1.018	1.012	0.971	0.996	1.014	1.023	1.028	1.026	Q9Y303-3	AMDHD2	Isoform 3 of N-acetylglucosamine-6-phosphate deacetylase
			0.944	0.930	0.997	0.951	0.964	0.991				P15559-3	NQO1	Isoform 3 of NAD(P)H dehydrogenase [quinone] 1
0.938	1.000	0.913	0.974	0.954	0.954	1.056	1.043	0.988	1.100	1.048	1.068	P00387-3	CYB5R3	Isoform 3 of NADH-cytochrome b5 reductase 3
0.804	1.055	1.048	1.270	1.097	1.080	1.120	1.017	1.145	0.995	0.903	1.084	Q9NQS3-3	NECTIN3	Isoform 3 of Nectin-3

Abundance Ratio														
1 d M1/C1	1 d M2/C2	1 d M3/C3	4 d M1/C1	4 d M2/C2	4 d M3/C3	7 d M1/C1	7 d M2/C2	7 d M3/C3	10 d M1/C1	10 d M2/C2	10 d M3/C3	Accession ID	Gene name	Protein Description
1.037	1.045	1.040	0.980	0.962	0.945	0.924	0.803	1.043	1.048	0.934	1.187	Q9ULJ8-3	PPP1R9A	Isoform 3 of Neurabin-1
1.053	1.020	1.049	1.009	0.981	1.007	0.922	1.167	1.065	0.999	0.999	1.111	Q9UBB6-3	NCDN	Isoform 3 of Neurochondrin
1.001	0.893	0.988	1.059	0.977	1.022	1.008	1.105	1.066	1.004	0.974	1.012	Q9GZT8-3	NIF3L1	Isoform 3 of NIF3-like protein 1
1.163	1.004	1.013	1.064	1.005	0.950	0.827	1.130	1.123	1.076	0.976	1.138	Q9NPJ8-3	NXT2	Isoform 3 of NTF2-related export protein 2
0.973	1.014	1.009	1.005	0.983	0.982	1.000	1.036	0.983	1.001	1.019	1.003	P49790-3	NUP153	Isoform 3 of Nuclear pore complex protein Nup153
1.000	1.072	1.020	1.001	1.012	1.020	1.055	1.003	0.945				P35658-3	NUP214	Isoform 3 of Nuclear pore complex protein Nup214
									1.269	1.124	1.008	Q92570-3	NR4A3	Isoform 3 of Nuclear receptor subfamily 4 group A member 3
0.937	0.946	1.050	0.959	0.815	1.016	0.897	1.150	0.926	0.946	0.976	1.016	Q14978-3	NOLC1	Isoform 3 of Nucleolar and coiled-body phosphoprotein 1
1.078	0.949	1.003	1.002	0.951	0.998	0.986	1.058	1.016	0.969	0.994	1.035	O60936-3	NOL3	Isoform 3 of Nucleolar protein 3
1.023	0.992	1.013	1.029	0.971	1.011	1.059	0.992	0.981	1.032	0.981	1.025	Q9BUP3-3	HTATIP2	Isoform 3 of Oxidoreductase HTATIP2
0.940	0.973	1.007	0.960	0.903	0.963	0.988	0.994	0.982	1.017	0.957	1.045	Q06710-3	PAX8	Isoform 3 of Paired box protein Pax-8
0.955	1.015	0.985	0.982	1.006	1.001	0.947	0.980	0.999	0.975	1.030	0.988	O60664-3	PLIN3	Isoform 3 of Perilipin-3
1.051	0.916	1.068	1.032	0.949	1.152							Q9UKG9-3	CROT	Isoform 3 of Peroxisomal carnitine O-octanoyltransferase
1.090	0.995	1.137	0.956	0.871	0.985	0.762	0.966	1.222	0.936	0.967	1.047	Q96BD5-3	PHF21A	Isoform 3 of PHD finger protein 21A
1.026	1.023	1.033	0.965	0.936	0.988	1.012	1.084	0.994	0.956	1.005	0.956	Q96BW9-3	TAMM41	Isoform 3 of Phosphatidate cytidylyltransferase, mitochondrial
0.978	0.985	0.984	1.010	0.981	0.993	1.014	1.064	1.043	1.056	1.042	1.033	P48739-3	PITPNB	Isoform 3 of Phosphatidylinositol transfer protein beta isoform
0.985	0.989	1.031	0.989	1.038	0.997	0.998	0.956	0.998	0.991	0.966	1.038	O95394-4	PGM3	Isoform 3 of Phosphoacetylglucosamine mutase
1.028	0.935	1.041	0.898	0.916	1.049	0.993	0.854	1.001	1.060	1.003	1.117	Q9Y2Q0-3	ATP8A1	Isoform 3 of Phospholipid-transporting ATPase IA
0.884	0.997	1.022	1.006	0.872	0.943	0.919	1.127	0.869				P98198-3	ATP8B2	Isoform 3 of Phospholipid-transporting ATPase ID
1.046	1.005	1.031	1.031	0.944	1.002	1.002	1.160	1.011	1.000	1.084	0.994	Q93100-3	PHKB	Isoform 3 of Phosphorylase b kinase regulatory subunit beta
1.072	1.020	0.994	0.971	0.989	0.799	1.011	1.297	0.940	1.052	1.064	1.310	Q8NC51-3	SERBP1	Isoform 3 of Plasminogen activator inhibitor 1 RNA-binding protein
0.970	0.957	1.039	1.006	0.894	0.989	0.996	0.994	1.002	1.100	0.965	1.080	Q15149-3	PLEC	Isoform 3 of Plectin
			0.995	1.210	1.019	1.486	1.542	1.056	1.019	0.922	1.057	Q7Z5L7-3	PODN	Isoform 3 of Podocan
1.099	0.936	1.013	1.145	1.335	1.070	1.027	1.049	1.150	0.913	0.992	1.036	Q15366-3	PCBP2	Isoform 3 of Poly(rC)-binding protein 2
1.024	1.006	0.999	0.989	0.979	0.992	0.978	1.029	0.994	0.992	1.017	0.977	Q13310-3	PABPC4	Isoform 3 of Polyadenylate-binding protein 4
0.809	0.851	1.095	0.818	1.107	1.029	0.635	0.415	0.918	0.856	0.991	0.966	P26599-3	PTBP1	Isoform 3 of Polypyrimidine tract-binding protein 1

Abundance Ratio														
1 d M1/C1	1 d M2/C2	1 d M3/C3	4 d M1/C1	4 d M2/C2	4 d M3/C3	7 d M1/C1	7 d M2/C2	7 d M3/C3	10 d M1/C1	10 d M2/C2	10 d M3/C3	Accession ID	Gene name	Protein Description
1.005	1.015	1.068	0.927	1.018	1.070	0.919	0.899	0.991	0.990	0.927	1.030	Q6UN15-3	FIP1L1	Isoform 3 of Pre-mRNA 3'-end-processing factor FIP1
1.009	1.000	0.990	0.978	0.962	1.027	0.993	1.003	0.964	1.022	1.008	1.001	O75400-3	PRPF40A	Isoform 3 of Pre-mRNA-processing factor 40 homolog A
1.007	0.979	0.954	0.934	0.930	0.936	0.953	1.103	0.960	0.982	0.971	0.940	Q96B36-3	AKT1S1	Isoform 3 of Proline-rich AKT1 substrate 1
0.917	0.909	0.983	1.017	0.971	1.044	0.905	0.776	1.107	1.183	0.816	1.034	Q9ULL5-3	PRR12	Isoform 3 of Proline-rich protein 12
1.036	1.092	1.080	1.154	0.888	1.092	0.850	1.037	0.993	1.039	1.191	1.039	O95905-3	ECD	Isoform 3 of Protein ecdysoneless homolog
1.360	0.965	1.009	1.256	1.044	1.463							Q3B820-3	FAM161A	Isoform 3 of Protein FAM161A
1.033	0.938	0.999	1.063	1.005	1.037	0.995	0.944	1.111	0.952	0.996	0.950	Q8TAV0-3	FAM76A	Isoform 3 of Protein FAM76A
1.012	0.960	0.977	1.008	1.063	1.040	1.033	1.069	1.039	1.022	1.001	1.082	Q9Y316-3	MEMO1	Isoform 3 of Protein MEMO1
1.007	0.989	0.989	0.975	0.968	0.999	1.013	0.903	0.987	1.035	0.990	1.065	Q8NHV4-3	NEDD1	Isoform 3 of Protein NEDD1
1.037	0.960	1.016	1.032	0.942	1.056	0.990	1.022	0.980	1.053	1.036	1.043	P35813-3	PPM1A	Isoform 3 of Protein phosphatase 1A
			0.993	1.006	0.953							Q7L099-3	RUFY3	Isoform 3 of Protein RUFY3
1.055	0.996	1.006	1.016	1.058	0.987	1.036	0.917	1.039	1.036	1.003	1.072	P55735-3	SEC13	Isoform 3 of Protein SEC13 homolog
1.072	1.150	1.291										Q9BVV6-3	KIAA0586	Isoform 3 of Protein TALPID3
0.989	1.010	1.001	0.978	0.967	0.998	0.966	1.076	0.994	0.971	0.982	0.991	O95487-3	SEC24B	Isoform 3 of Protein transport protein Sec24B
									0.735	1.269	0.984	Q70J99-3	UNC13D	Isoform 3 of Protein unc-13 homolog D
1.025	1.009	1.031	0.976	0.958	0.963	1.010	1.021	1.011	1.044	1.095	0.970	Q969M3-3	YIPF5	Isoform 3 of Protein YIPF5
						0.925	1.509	1.567				Q5T4F4-3	ZFYVE27	Isoform 3 of Protrudin
1.013	1.016	1.137	1.019	0.979	1.080	1.024	0.971	1.078	1.084	1.012	1.033	Q8TB72-3	PUM2	Isoform 3 of Pumilio homolog 2
1.051	0.968	0.976	1.018	0.977	1.037	0.960	1.033	1.086	1.043	1.040	1.030	Q17R31-3	TATDN3	Isoform 3 of Putative deoxyribonuclease TATDN3
1.001	1.007	0.986	0.996	1.032	0.959	0.971	0.964	0.997	0.949	0.978	0.952	P32322-3	PYCR1	Isoform 3 of Pyrroline-5-carboxylate reductase 1, mitochondrial
0.990	0.980	0.970	0.975	0.875	0.896	1.276	0.893	0.971	1.084	0.892	1.183	Q15032-3	R3HDM1	Isoform 3 of R3H domain-containing protein 1
			0.938	0.927	0.912	1.172	1.037	1.043				O75154-3	RAB11FIP3	Isoform 3 of Rab11 family-interacting protein 3
0.946	1.037	1.082	0.998	0.973	1.046	1.029	1.020	1.079	1.118	1.114	1.244	Q13905-3	RAPGEF1	Isoform 3 of Rap guanine nucleotide exchange factor 1
1.021	0.987	1.025	1.014	0.982	1.036	0.977	1.000	0.992	0.998	0.981	1.024	Q6R327-3	RICTOR	Isoform 3 of Rapamycin-insensitive companion of mTOR
									1.075	0.974	1.267	Q5VT52-3	RPRD2	Isoform 3 of Regulation of nuclear pre-mRNA domain-containing protein 2
0.990	1.010	1.026	0.982	1.006	1.035	0.943	1.084	1.061	0.937	0.989	1.038	O43665-3	RGS10	Isoform 3 of Regulator of G-protein signaling 10
1.053	1.008	1.043	1.007	1.150	0.949	1.005	0.957	1.068	0.995	1.000	0.985	P15927-3	RPA2	Isoform 3 of Replication protein A 32 kDa subunit
0.942	0.968	1.036	0.910	0.999	1.068	0.955	0.784	1.057	1.003	0.942	1.034	Q9P2K3-3	RCOR3	Isoform 3 of REST corepressor 3
1.054	0.976	1.013	1.065	0.959	1.002	1.076	1.210	1.025	0.896	0.879	0.981	Q86XN8-3	MEX3D	Isoform 3 of RNA-binding protein MEX3D
1.028	0.988	1.015	0.963	1.014	0.991	1.011	0.871	1.010	1.053	1.038	1.048	Q15424-3	SAFB	Isoform 3 of Scaffold attachment factor B1

Abundance Ratio														
1 d M1/C1	1 d M2/C2	1 d M3/C3	4 d M1/C1	4 d M2/C2	4 d M3/C3	7 d M1/C1	7 d M2/C2	7 d M3/C3	10 d M1/C1	10 d M2/C2	10 d M3/C3	Accession ID	Gene name	Protein Description
1.057	0.968	0.948	0.951	0.895	1.035	1.017	1.036	0.950	1.007	1.060	0.982	Q96PY6-3	NEK1	Isoform 3 of Serine/threonine-protein kinase Nek1
0.935	1.019	1.151	0.962	0.898	0.952	0.948	1.103	1.050	1.052	1.053	1.047	P48454-3	PPP3CC	Isoform 3 of Serine/threonine-protein phosphatase 2B catalytic subunit gamma isoform
1.003	0.998	1.023	1.021	1.014	0.997	0.987	0.993	0.988	1.008	0.971	0.995	O00743-3	PPP6C	Isoform 3 of Serine/threonine-protein phosphatase 6 catalytic subunit
1.036	0.991	1.013	0.984	0.955	0.992	0.998	1.042	1.007	0.957	0.973	1.047	O15084-1	ANKRD28	Isoform 3 of Serine/threonine-protein phosphatase 6 regulatory ankyrin repeat subunit A
1.012	0.945	1.018	0.994	0.988	1.043	1.011	0.940	1.028	1.009	1.030	1.057	Q9UPX8-3	SHANK2	Isoform 3 of SH3 and multiple ankyrin repeat domains protein 2
									0.973	0.866	1.159	Q9NQQ7-3	SLC35C2	Isoform 3 of Solute carrier family 35 member C2
1.008	1.006	1.048	0.986	0.975	1.070	1.039	1.076	1.011	1.060	0.974	1.009	Q9UNH6-3	SNX7	Isoform 3 of Sorting nexin-7
0.979	1.034	0.966	1.051	1.035	0.901	1.026	1.055	1.021	1.054	1.007	1.109	Q13813-3	SPTAN1	Isoform 3 of Spectrin alpha chain, non-erythrocytic 1
									1.189	1.098	0.994	O75602-3	SPAG6	Isoform 3 of Sperm-associated antigen 6
1.004	0.968	1.028	1.012	0.931	1.095	1.048	1.034	1.126	1.013	1.045	1.119	Q9NRL3-3	STRN4	Isoform 3 of Striatin-4
0.812	0.792	1.069	0.919	0.908	1.072	1.001	0.885	1.082	0.980	0.918	1.027	Q9HAC7-3	SUGCT	Isoform 3 of Succinate--hydroxymethylglutarate CoA-transferase
1.159	1.029	1.091	1.117	1.007	1.231	1.166	1.308	1.038	1.084	0.935	1.008	P04179-3	SOD2	Isoform 3 of Superoxide dismutase [Mn], mitochondrial
0.995	1.005	1.028	1.031	0.996	1.062	0.995	1.034	1.012	0.982	1.012	1.010	Q8TC07-3	TBC1D15	Isoform 3 of TBC1 domain family member 15
0.764	0.960	0.985	0.934	0.952	1.036	1.068	0.945	0.997	1.157	1.074	1.068	Q96BZ9-3	TBC1D20	Isoform 3 of TBC1 domain family member 20
0.988	0.948	1.102	0.915	0.930	1.072	1.007	0.972	0.946	0.970	0.948	1.094	Q5JTV8-3	TOR1AIP1	Isoform 3 of Torsin-1A-interacting protein 1
1.020	1.033	0.977	1.066	1.066	1.027	1.048	1.156	0.997	1.033	1.050	1.000	P0DI81-3	TRAPPC2	Isoform 3 of Trafficking protein particle complex subunit 2
1.044	0.798	1.131										Q99081-3	TCF12	Isoform 3 of Transcription factor 12
0.933	0.981	0.951	0.974	1.046	1.001	0.831	1.010	1.171	0.850	0.953	0.997	Q8NEM7-3	SUPT20H	Isoform 3 of Transcription factor SPT20 homolog
1.031	0.973	1.059	1.037	0.958	0.965	0.990	1.065	0.956	0.939	1.018	1.005	Q86YP4-3	GATAD2A	Isoform 3 of Transcriptional repressor p66-alpha
1.140	0.920	1.063	1.019	0.879	0.945	1.180	1.012	1.102				Q9NXG6-3	P4HTM	Isoform 3 of Transmembrane prolyl 4-hydroxylase
0.920	0.967	1.258										Q96Q45-3	TMEM237	Isoform 3 of Transmembrane protein 237
0.970	0.953	0.981	0.946	0.966	0.992	0.960	0.857	0.929	1.002	0.970	1.004	Q13428-3	TCOF1	Isoform 3 of Treacle protein
0.902	1.001	0.961	0.935	0.960	0.936	0.978	0.866	0.948	0.960	0.992	0.913	P07951-3	TPM2	Isoform 3 of Tropomyosin beta chain
0.967	0.936	1.010	1.001	0.942	1.038	0.940	0.983	1.027	1.032	1.007	1.094	Q9H7E2-3	TDRD3	Isoform 3 of Tudor domain-containing protein 3
1.044	0.993	1.051	0.982	0.983	1.048	0.974	0.982	1.044	1.029	1.055	1.038	Q9ULQ1-3	TPCN1	Isoform 3 of Two pore calcium channel protein 1
0.991	0.989	0.973				1.021	0.875	0.923				Q9Y4E8-3	USP15	Isoform 3 of Ubiquitin carboxyl-terminal hydrolase 15

Abundance Ratio														
1 d M1/C1	1 d M2/C2	1 d M3/C3	4 d M1/C1	4 d M2/C2	4 d M3/C3	7 d M1/C1	7 d M2/C2	7 d M3/C3	10 d M1/C1	10 d M2/C2	10 d M3/C3	Accession ID	Gene name	Protein Description
1.040	1.081	0.994	0.980	0.989	1.052	0.941	1.032	0.961	1.034	0.966	0.983	Q9NVE5-3	USP40	Isoform 3 of Ubiquitin carboxyl-terminal hydrolase 40
0.870	0.968	1.047	0.938	0.788	0.908	0.777	0.701	0.952	1.298	1.053	1.280	Q8N5J2-3	MINDY1	Isoform 3 of Ubiquitin carboxyl-terminal hydrolase MINDY-1
0.944	0.941	1.017	0.970	1.038	1.027	0.941	0.765	0.967				O00159-3	MYO1C	Isoform 3 of Unconventional myosin-Ic
1.058	0.989	1.025	1.036	0.951	1.025	1.003	1.036	1.033	0.992	0.964	0.976	Q9H3H3-3	C11orf68	Isoform 3 of UPF0696 protein C11orf68
1.002	0.986	1.004	1.003	0.976	1.001	1.015	1.076	1.052	0.953	0.954	1.045	Q8N3P4-3	VPS8	Isoform 3 of Vacuolar protein sorting-associated protein 8 homolog
0.979	0.995	0.998	0.973	0.987	0.987	0.965	0.984	0.963	0.959	0.907	0.977	P49748-3	ACADVL	Isoform 3 of Very long-chain specific acyl-CoA dehydrogenase, mitochondrial
0.986	1.006	0.991	1.020	0.867	1.058	1.055	1.058	1.025	1.104	1.077	1.023	Q93050-3	ATP6V0A1	Isoform 3 of V-type proton ATPase 116 kDa subunit a isoform 1
1.069	1.033	1.049	1.112	0.922	1.046	1.118	1.043	1.048	1.202	0.998	1.097	Q9Y484-3	WDR45	Isoform 3 of WD repeat domain phosphoinositide-interacting protein 4
0.999	0.998	1.038	0.980	0.987	1.037	1.002	0.991	0.989	1.010	1.042	1.059	Q7Z5K2-3	WAPL	Isoform 3 of Wings apart-like protein homolog
0.976	1.001	0.993	0.998	0.986	1.012	0.982	1.032	1.019	0.967	0.979	1.057	Q9NQW7-3	XPNPEP1	Isoform 3 of Xaa-Pro aminopeptidase 1
1.088	0.919	1.068	1.010	1.083	1.018	0.926	1.585	1.139				Q8TBC5-3	ZSCAN18	Isoform 3 of Zinc finger and SCAN domain-containing protein 18
1.002	1.066	0.977				0.947	0.916	0.983				O15231-3	ZNF185	Isoform 3 of Zinc finger protein 185
0.969	1.038	0.940	1.091	0.959	0.998	1.097	0.983	0.998				Q96JM2-3	ZNF462	Isoform 3 of Zinc finger protein 462
0.979	0.926	0.928	0.908	0.913	1.169	0.951	0.949	0.927				Q5VV52-3	ZNF691	Isoform 3 of Zinc finger protein 691
						0.966	0.878	0.816				P17010-3	ZFX	Isoform 3 of Zinc finger X-chromosomal protein
									1.209	1.152	1.308	Q9ULB1-3	NRXN1	Isoform 3a of Neurexin-1
1.022	1.004	1.024	1.015	0.997	0.990	0.996	1.034	1.020	1.013	1.015	0.990	Q8TDZ2-4	MICAL1	Isoform 4 of [F-actin]-methionine sulfoxide oxidase MICAL1
0.986	0.970	0.976	0.978	0.960	1.017	0.989	0.998	0.994	1.060	0.999	1.045	Q8N983-4	MRPL43	Isoform 4 of 39S ribosomal protein L43, mitochondrial
0.951	1.013	0.950	0.981	1.021	1.011	0.992	0.951	0.999	1.010	1.057	0.963	P62847-4	RPS24	Isoform 4 of 40S ribosomal protein S24
0.972	1.002	0.996	1.052	0.999	1.031	1.022	1.136	1.055	0.968	1.047	0.992	P08195-4	SLC3A2	Isoform 4 of 4F2 cell-surface antigen heavy chain
0.975	0.995	1.004	1.013	0.944	0.996	1.012	1.098	1.040	1.027	1.024	1.069	Q13085-4	ACACA	Isoform 4 of Acetyl-CoA carboxylase 1
			1.015	0.976	0.961	0.967	1.059	0.989	0.913	0.904	0.927	Q8N4X5-4	AFAP1L2	Isoform 4 of Actin filament-associated protein 1-like 2
1.138	0.972	0.998				1.071	1.296	0.956	0.989	1.237	0.947	P36896-4	ACVR1B	Isoform 4 of Activin receptor type-1B
0.991	1.017	1.011	0.986	0.973	1.032	1.037	0.974	1.003	1.025	0.944	1.063	Q9Y2D5-6	AKAP2	Isoform 4 of A-kinase anchor protein 2
0.966	1.000	1.091	1.043	0.903	0.958	0.946	1.111	1.165	1.053	0.994	1.151	Q96BT7-4	ALKBH8	Isoform 4 of Alkylated DNA repair protein alkB homolog 8
			1.064	1.131	0.975	1.031	1.170	0.963	0.910	1.023	0.966	Q9NTJ4-4	MAN2C1	Isoform 4 of Alpha-mannosidase 2C1
1.035	0.832	1.191	0.948	0.979	1.076	0.983	0.897	1.011	1.012	0.971	1.008	Q9UHK6-5	AMACR	Isoform 4 of Alpha-methylacyl-CoA racemase

Abundance Ratio														
1 d M1/C1	1 d M2/C2	1 d M3/C3	4 d M1/C1	4 d M2/C2	4 d M3/C3	7 d M1/C1	7 d M2/C2	7 d M3/C3	10 d M1/C1	10 d M2/C2	10 d M3/C3	Accession ID	Gene name	Protein Description
			0.884	0.899	0.988	0.997	1.034	1.174	1.177	1.052	1.128	Q92870-4	APBB2	Isoform 4 of Amyloid beta A4 precursor protein-binding family B member 2
0.925	0.963	1.046	0.868	0.929	0.938	1.036	1.030	1.008	1.023	0.999	0.838	Q9Y2J4-4	AMOTL2	Isoform 4 of Angiomotin-like protein 2
0.959	0.978	0.994	0.961	0.995	0.999	0.975	0.919	1.000	0.998	0.993	1.014	Q9P0K7-4	RAI14	Isoform 4 of Ankycorbin
1.117	0.978	1.104										Q9UKV3-5	ACIN1	Isoform 4 of Apoptotic chromatin condensation inducer in the nucleus
1.011	0.993	1.021	0.999	1.035	1.033	1.000	0.926	1.005	1.025	1.028	1.072	P52594-4	AGFG1	Isoform 4 of Arf-GAP domain and FG repeat-containing protein 1
0.960	1.023	1.007	0.996	1.007	1.014	0.991	1.078	0.975	0.995	1.001	1.036	Q96P47-4	AGAP3	Isoform 4 of Arf-GAP with GTPase, ANK repeat and PH domain-containing protein 3
0.957	1.013	0.953	1.049	0.980	1.040	1.073	0.974	1.020	1.168	1.007	1.150	Q9NW81-4	ATP5SL	Isoform 4 of ATP synthase subunit s-like protein
0.970	0.999	1.020	1.005	0.971	1.031	0.986	1.047	1.060	1.022	1.014	0.999	Q9HCM4-4	EPB41L5	Isoform 4 of Band 4.1-like protein 5
1.007	1.028	1.006	1.025	0.999	0.992	0.997	1.110	0.983	1.023	0.994	1.062	P32121-4	ARRB2	Isoform 4 of Beta-arrestin-2
0.990	0.978	1.025	1.018	1.003	0.955	1.083	0.955	1.074	0.959	0.938	1.073	P55290-4	CDH13	Isoform 4 of Cadherin-13
1.691	0.880	0.992	2.278	0.859	0.973	2.177	0.814	1.005	1.017	1.031	1.110	Q13137-4	CALCOCO2	Isoform 4 of Calcium-binding and coiled-coil domain-containing protein 2
0.956	0.997	0.953	1.027	0.997	1.036	1.002	1.086	1.021	1.021	0.936	1.046	O43852-4	CALU	Isoform 4 of Calumenin
1.054	0.977	1.017	1.064	0.946	1.099	1.100	1.108	1.036	1.091	1.015	1.071	O15438-4	ABCC3	Isoform 4 of Canalicular multispecific organic anion transporter 2
1.020	0.992	1.002	1.006	1.006	1.042	1.015	1.019	1.025	1.036	1.022	1.020	Q96JB5-4	CDK5RAP3	Isoform 4 of CDK5 regulatory subunit-associated protein 3
									1.090	1.113	1.063	P62633-4	CNBP	Isoform 4 of Cellular nucleic acid-binding protein
0.953	1.046	1.035	0.976	0.929	1.097	1.070	1.131	0.987	0.962	0.951	1.117	O14578-4	CIT	Isoform 4 of Citron Rho-interacting kinase
0.994	1.020	0.988	0.897	1.101	0.995	1.113	1.197	1.024	1.012	1.015	1.133	O60271-4	SPAG9	Isoform 4 of C-Jun-amino-terminal kinase-interacting protein 4
1.037	1.034	1.118	1.189	1.136	0.989	1.155	0.769	0.818	0.998	0.888	1.113	Q7Z460-4	CLASP1	Isoform 4 of CLIP-associating protein 1
1.006	0.994	1.000	1.049	1.017	1.064	1.056	1.037	1.034	1.036	1.045	1.050	O75534-4	CSDE1	Isoform 4 of Cold shock domain-containing protein E1
1.003	0.970	1.062	1.109	0.882	0.980	1.059	1.190	1.089	0.870	0.896	0.936	Q6IBW4-4	NCAPH2	Isoform 4 of Condensin-2 complex subunit H2
1.034	1.028	1.020	0.968	0.957	0.979	0.995	1.084	1.029	0.923	0.994	0.993	O75909-4	CCNK	Isoform 4 of Cyclin-K
0.934	1.013	1.026	1.002	1.020	0.989	1.051	1.141	1.022	0.843	0.844	1.029	P11388-4	TOP2A	Isoform 4 of DNA topoisomerase 2-alpha
0.982	0.998	1.001	0.998	0.986	1.008	1.003	0.971	0.991	0.991	1.003	0.998	P55265-4	ADAR	Isoform 4 of Double-stranded RNA-specific adenosine deaminase
0.965	0.993	1.002	0.988	0.891	0.892	1.044	1.016	1.046	1.123	1.212	1.317	O00429-2	DNM1L	Isoform 4 of Dynamin-1-like protein
			0.919	1.011	1.012	0.883	1.005	1.103	0.873	0.916	1.100	P50570-4	DNM2	Isoform 4 of Dynamin-2
1.030	1.028	1.018	1.030	0.989	1.031	1.061	0.998	1.062	0.987	0.961	1.071	P46934-4	NEDD4	Isoform 4 of E3 ubiquitin-protein ligase NEDD4
1.048	1.105	1.053	1.090	1.010	1.015	1.156	1.171	0.998				Q6ZT12-4	UBR3	Isoform 4 of E3 ubiquitin-protein ligase UBR3

Abundance Ratio														
1 d M1/C1	1 d M2/C2	1 d M3/C3	4 d M1/C1	4 d M2/C2	4 d M3/C3	7 d M1/C1	7 d M2/C2	7 d M3/C3	10 d M1/C1	10 d M2/C2	10 d M3/C3	Accession ID	Gene name	Protein Description
0.987	0.978	1.015	0.992	0.937	0.953	1.003	1.155	1.076	1.004	1.014	0.949	Q8TE02-4	ELP5	Isoform 4 of Elongator complex protein 5
1.063	1.008	1.101	1.003	0.908	0.967	1.037	1.214	0.993	1.064	0.997	1.071	Q08345-5	DDR1	Isoform 4 of Epithelial discoidin domain-containing receptor 1
0.995	0.962	1.030	0.975	0.978	1.003	0.973	1.046	1.081	0.990	1.003	1.104	Q96ME1-4	FBXL18	Isoform 4 of F-box/LRR-repeat protein 18
0.998	0.905	1.009	1.088	0.974	1.057	0.966	1.045	1.094	0.937	0.919	0.969	Q96C11-3	FGGY	Isoform 4 of FGGY carbohydrate kinase domain-containing protein
0.945	1.028	1.225	1.043	0.852	1.060	0.939	1.100	0.906				Q2V2M9-4	FHOD3	Isoform 4 of FH1/FH2 domain-containing protein 3
0.999	0.956	0.970	0.996	0.965	1.004	0.993	1.001	1.007	0.986	0.963	1.011	Q5T0N5-4	FNBP1L	Isoform 4 of Formin-binding protein 1-like
0.988	0.988	0.979	0.987	0.986	0.948	0.982	0.912	0.965	0.959	0.933	0.981	Q13642-4	FHL1	Isoform 4 of Four and a half LIM domains protein 1
0.954	0.986	1.032	1.022	0.943	1.034	1.040	0.990	1.000	1.026	0.986	1.052	Q9BQS8-4	FYCO1	Isoform 4 of FYVE and coiled-coil domain-containing protein 1
0.962	0.989	1.007	1.035	0.916	1.077	0.969	0.949	0.998	1.008	1.022	0.982	P63092-4	GNAS	Isoform 4 of Guanine nucleotide-binding protein G(s) subunit alpha isoforms short
			0.927	1.069	0.993							Q9NQG7-4	HPS4	Isoform 4 of Hermansky-Pudlak syndrome 4 protein
1.014	1.145	1.308				1.006	0.690	1.206	1.031	1.118	1.402	Q14103-4	HNRNPD	Isoform 4 of Heterogeneous nuclear ribonucleoprotein D0
0.972	1.005	0.996	0.982	0.939	0.984	0.981	0.989	0.968	0.994	0.969	0.973	Q70UQ0-4	IKBIP	Isoform 4 of Inhibitor of nuclear factor kappa-B kinase-interacting protein
1.007	1.046	1.067	0.798	1.070	1.156	1.237	1.266	0.959				Q86Y91-4	KIF18B	Isoform 4 of Kinesin-like protein KIF18B
0.963	0.965	1.020	0.969	0.987	1.021	0.934	0.868	0.976	1.022	1.016	1.048	Q7Z4S6-4	KIF21A	Isoform 4 of Kinesin-like protein KIF21A
0.927	0.987	1.051	0.962	0.967	1.017	0.995	1.034	1.018	1.009	0.983	1.042	Q71RC2-4	LARP4	Isoform 4 of La-related protein 4
0.835	0.904	0.990	1.077	0.990	1.073	1.035	1.019	1.056	0.999	0.913	1.157	Q32MZ4-4	LRRFIP1	Isoform 4 of Leucine-rich repeat flightless-interacting protein 1
1.021	0.990	1.022	1.044	0.985	0.990	0.991	0.935	0.987	1.075	1.045	1.071	Q9Y608-4	LRRFIP2	Isoform 4 of Leucine-rich repeat flightless-interacting protein 2
0.968	1.015	0.987	0.974	0.941	1.069	0.963	1.032	1.056	1.552	1.061	1.358	Q8IWI9-4	MGA	Isoform 4 of MAX gene-associated protein
0.990	1.000	1.037	1.024	1.031	0.929	0.984	1.087	1.074	0.955	0.938	1.016	Q16891-4	IMMT	Isoform 4 of MICOS complex subunit MIC60
1.047	1.089	1.042	0.957	0.870	0.920	0.934	0.974	1.082	1.051	0.942	1.136	P30305-4	CDC25B	Isoform 4 of M-phase inducer phosphatase 2
0.987	0.983	1.026	0.975	0.969	1.001	0.970	0.975	0.990	0.992	0.974	1.019	P35580-4	MYH10	Isoform 4 of Myosin-10
1.032	0.997	1.072	1.002	0.949	1.026	1.028	1.060	1.040	0.967	0.980	1.127	O95248-4	SBF1	Isoform 4 of Myotubularin-related protein 5
0.848	0.993	1.071	0.874	0.718	0.711	1.133	0.850	0.961	0.948	0.914	0.945	P49281-4	SLC11A2	Isoform 4 of Natural resistance-associated macrophage protein 2
			1.085	0.880	1.017	1.032	1.161	0.993				O15259-4	NPHP1	Isoform 4 of Nephrocystin-1
0.960	0.977	0.997	0.943	0.921	0.984	0.967	0.963	0.987	1.006	0.957	1.028	Q8IY17-4	PNPLA6	Isoform 4 of Neuropathy target esterase
									0.820	0.899	1.074	Q13772-4	NCOA4	Isoform 4 of Nuclear receptor coactivator 4

Abundance Ratio														
1 d M1/C1	1 d M2/C2	1 d M3/C3	4 d M1/C1	4 d M2/C2	4 d M3/C3	7 d M1/C1	7 d M2/C2	7 d M3/C3	10 d M1/C1	10 d M2/C2	10 d M3/C3	Accession ID	Gene name	Protein Description
1.073	1.022	1.019	1.059	0.986	0.990	1.066	1.197	1.062	1.056	1.057	1.090	Q16656-4	NRF1	Isoform 4 of Nuclear respiratory factor 1
1.078	1.116	0.995	1.160	1.033	0.968	0.961	0.750	1.028	1.041	1.072	1.139	O75167-4	PHACTR2	Isoform 4 of Phosphatase and actin regulator 2
			0.950	1.012	1.046	1.010	0.988	1.034				Q8IZ21-4	PHACTR4	Isoform 4 of Phosphatase and actin regulator 4
0.907	0.956	1.075	0.972	0.975	1.032	0.987	0.944	0.980	1.032	0.984	1.080	P27986-4	PIK3R1	Isoform 4 of Phosphatidylinositol 3-kinase regulatory subunit alpha
0.991	1.008	1.012	1.006	0.955	1.024	0.994	0.925	0.965	1.047	1.061	1.068	Q9HAU0-4	PLEKHA5	Isoform 4 of Pleckstrin homology domain-containing family A member 5
0.978	1.086	1.063	1.028	0.761	0.974	1.041	1.132	1.062	1.152	0.956	1.061	Q15149-4	PLEC	Isoform 4 of Plectin
1.037	1.033	1.038	0.997	1.122	0.971	0.976	0.975	1.014	1.010	0.954	0.998	O95758-4	PTBP3	Isoform 4 of Polypyrimidine tract-binding protein 3
0.999	0.985	0.992	0.973	0.990	1.011	0.991	0.979	1.012	1.000	1.017	1.010	P46087-4	NOP2	Isoform 4 of Probable 28S rRNA (cytosine(4447)-C(5))-methyltransferase
1.035	1.080	1.011	1.001	0.923	1.100	1.100	1.015	1.050	1.042	0.938	1.114	Q7Z333-4	SETX	Isoform 4 of Probable helicase senataxin
1.013	1.016	1.102	1.038	0.972	1.016							O43822-4	C21orf2	Isoform 4 of Protein C21orf2
0.941	0.968	0.996	0.917	0.890	0.977	0.978	0.991	0.977	1.072	1.038	1.067	Q6P4E1-4	CASC4	Isoform 4 of Protein CASC4
1.021	1.039	1.054	1.023	0.996	1.011	1.012	1.031	0.994	1.002	0.986	1.035	Q6ZS17-4	FAM65A	Isoform 4 of Protein FAM65A
0.947	0.971	0.990	0.990	0.918	0.991	1.013	1.007	1.015	0.996	1.027	1.027	Q9C0E8-4	LNP	Isoform 4 of Protein lunapark
0.971	1.008	1.021	0.943	0.992	1.071	1.010	0.935	1.029	1.096	1.000	1.106	Q9BZL4-4	PPP1R12C	Isoform 4 of Protein phosphatase 1 regulatory subunit 12C
1.265	1.111	1.067	1.004	0.981	0.971	0.971	1.038	0.973	1.019	1.097	1.033	Q92540-4	SMG7	Isoform 4 of Protein SMG7
0.897	0.919	1.031	1.110	0.896	1.214	1.311	0.952	1.133	1.037	1.029	1.418	Q9C0H2-4	TTYH3	Isoform 4 of Protein tweety homolog 3
1.029	1.003	1.033	1.021	1.071	1.104	0.921	0.906	1.077	0.933	1.007	1.095	Q08623-4	PUDP	Isoform 4 of Pseudouridine-5'-phosphatase
0.959	0.983	0.969	0.979	0.995	0.999	1.004	0.948	0.964	0.993	0.981	0.982	P08559-4	PDHA1	Isoform 4 of Pyruvate dehydrogenase E1 component subunit alpha, somatic form, mitochondrial
1.016	1.018	1.042	0.959	1.008	1.021	0.994	1.080	1.014	1.028	1.027	1.043	Q9H974-4	QTRT2	Isoform 4 of Queuine tRNA-ribosyltransferase accessory subunit 2
1.007	1.010	1.039	0.996	1.000	0.995	1.006	1.042	1.012	0.935	0.932	1.018	Q9P2R3-4	ANKFY1	Isoform 4 of Rabankyrin-5
			0.918	1.013	1.014	1.014	0.866	0.968	1.080	1.039	0.946	P47736-4	RAP1GAP	Isoform 4 of Rap1 GTPase-activating protein 1
			0.932	0.664	0.934							Q96PV0-4	SYNGAP1	Isoform 4 of Ras/Rap GTPase-activating protein SynGAP
0.868	0.987	1.019	0.960	0.889	1.004	0.964	0.919	0.982	1.032	0.998	1.104	P62070-4	RRAS2	Isoform 4 of Ras-related protein R-Ras2
						0.954	1.115	1.032	0.845	1.079	0.942	O43236-4	4-Sep	Isoform 4 of Septin-4
1.032	1.129	1.063	0.956	0.922	0.992	0.976	1.093	0.867				Q13362-4	PPP2R5C	Isoform 4 of Serine/threonine-protein phosphatase 2A 56 kDa regulatory subunit gamma isoform
0.987	0.947	1.096	1.061	0.889	1.023	0.999	1.169	1.134	0.905	1.013	1.006	P16298-4	PPP3CB	Isoform 4 of Serine/threonine-protein phosphatase 2B catalytic subunit beta isoform

Abundance Ratio														
1 d M1/C1	1 d M2/C2	1 d M3/C3	4 d M1/C1	4 d M2/C2	4 d M3/C3	7 d M1/C1	7 d M2/C2	7 d M3/C3	10 d M1/C1	10 d M2/C2	10 d M3/C3	Accession ID	Gene name	Protein Description
1.004	0.970	1.017	1.033	0.974	0.971	1.021	1.139	0.980	0.982	1.029	1.067	Q96C92-4	SDCCAG3	Isoform 4 of Serologically defined colon cancer antigen 3
			1.140	0.909	1.070	1.823	0.984	1.096	1.223	0.975	1.073	P78314-4	SH3BP2	Isoform 4 of SH3 domain-binding protein 2
1.100	0.799	1.001	1.275	1.317	1.168							P24821-4	TNC	Isoform 4 of Tenascin
1.239	1.155	1.147	1.211	0.848	1.012	1.156	1.253	1.047				Q63HR2-4	TNS2	Isoform 4 of Tensin-2
1.082	1.042	1.040	1.044	1.050	1.052	1.066	0.996	1.011	1.029	1.034	1.045	Q8NFU3-4	TSTD1	Isoform 4 of Thiosulfate sulfurtransferase/rhodanese-like domain-containing protein 1
1.131	0.952	1.102	0.976	1.012	1.055	1.108	0.881	0.966	1.093	1.010	1.126	A5PLN9-5	TRAPPC13	Isoform 4 of Trafficking protein particle complex subunit 13
1.046	0.990	1.113	0.959	0.982	0.993	0.961	1.066	1.003	1.015	0.967	1.071	P21675-4	TAF1	Isoform 4 of Transcription initiation factor TFIID subunit 1
1.077	1.102	1.068	0.966	0.936	0.993	1.005	1.172	1.050	1.031	1.022	0.896	Q6ICL3-4	TANGO2	Isoform 4 of Transport and Golgi organization protein 2 homolog
1.112	1.067	1.068	1.064	1.395	1.119	1.089	1.012	0.996	1.171	0.833	0.944	O95379-4	TNFAIP8	Isoform 4 of Tumor necrosis factor alpha-induced protein 8
			0.912	0.809	1.005							Q12923-4	PTPN13	Isoform 4 of Tyrosine-protein phosphatase non-receptor type 13
1.029	1.022	1.006	1.015	0.978	0.988	0.991	1.028	1.013	1.017	0.999	1.031	Q96K76-4	USP47	Isoform 4 of Ubiquitin carboxyl-terminal hydrolase 47
0.998	0.992	1.033	0.997	0.938	1.028	1.041	1.023	1.034	1.032	0.999	1.098	O95155-4	UBE4B	Isoform 4 of Ubiquitin conjugation factor E4 B
0.989	0.961	1.128	0.988	0.968	1.018	0.957	1.109	1.006	0.946	0.993	0.990	Q9NZ09-4	UBAP1	Isoform 4 of Ubiquitin-associated protein 1
0.946	0.968	0.840	0.941	0.915	1.159	0.991	1.028	1.045	0.992	0.928	1.175	P78381-4	SLC35A2	Isoform 4 of UDP-galactose translocator
1.155	1.353	1.111	1.091	1.626	1.070	1.174	1.438	1.115	1.027	1.496	0.955	Q5SQH8-4	C6orf136	Isoform 4 of Uncharacterized protein C6orf136
1.191	1.054	1.112	0.957	0.929	0.980	1.062	0.960	0.882	0.909	0.794	1.132	B2RTY4-4	MYO9A	Isoform 4 of Unconventional myosin-IXa
			1.036	1.010	0.893							Q9HA47-4	UCK1	Isoform 4 of Uridine-cytidine kinase 1
0.996	1.010	1.012	0.985	1.014	1.003	1.005	1.068	1.030	0.995	0.995	1.047	Q5VIR6-4	VPS53	Isoform 4 of Vacuolar protein sorting-associated protein 53 homolog
0.954	1.049	0.990	0.964	1.026	1.028	1.007	1.110	1.036	0.932	0.958	1.022	O94967-4	WDR47	Isoform 4 of WD repeat-containing protein 47
0.952	1.070	1.051	1.016	0.949	0.964	0.992	1.103	1.096	0.941	0.935	0.985	O43379-4	WDR62	Isoform 4 of WD repeat-containing protein 62
1.013	0.994	1.003	0.999	0.980	1.002	1.023	0.964	0.976	1.042	0.993	1.042	P49750-4	YLPM1	Isoform 4 of YLP motif-containing protein 1
1.151	0.958	1.011	0.914	0.935	1.047	1.000	1.046	1.049	0.990	0.949	1.062	A7E2V4-4	ZSWIM8	Isoform 4 of Zinc finger SWIM domain-containing protein 8
0.893	0.939	1.253	1.002	0.984	0.939							Q9C0C7-5	AMBRA1	Isoform 5 of Activating molecule in BECN1-regulated autophagy protein 1
0.989	1.013	0.952	0.973	0.956	0.899	0.974	1.023	0.940	0.966	1.011	0.887	P07108-5	DBI	Isoform 5 of Acyl-CoA-binding protein
0.995	1.013	1.013	1.015	0.961	1.022	1.004	1.028	1.013	1.022	1.009	1.030	P55196-5	AFDN	Isoform 5 of Afadin
1.019	1.016	1.023	1.004	0.990	1.004	1.003	1.016	1.045	0.987	1.006	0.995	O14617-5	AP3D1	Isoform 5 of AP-3 complex subunit delta-1

Abundance Ratio														
1 d M1/C1	1 d M2/C2	1 d M3/C3	4 d M1/C1	4 d M2/C2	4 d M3/C3	7 d M1/C1	7 d M2/C2	7 d M3/C3	10 d M1/C1	10 d M2/C2	10 d M3/C3	Accession ID	Gene name	Protein Description
0.993	0.984	0.996	1.089	0.970	0.972	1.110	1.102	1.177	0.975	0.962	0.945	Q96GD4-5	AURKB	Isoform 5 of Aurora kinase B
1.078	0.864	1.072	1.014	0.892	0.947	1.067	1.083	1.074	0.937	1.012	1.070	O75143-5	ATG13	Isoform 5 of Autophagy-related protein 13
0.921	1.045	0.855										P54687-5	BCAT1	Isoform 5 of Branched-chain-amino-acid aminotransferase, cytosolic
0.982	0.997	1.001	0.964	0.949	0.967	0.990	0.955	0.957	0.990	0.953	1.016	Q05682-5	CALD1	Isoform 5 of Caldesmon
			0.958	0.871	1.031							Q9Y2G2-5	CARD8	Isoform 5 of Caspase recruitment domain-containing protein 8
1.013	1.061	0.976	0.979	0.912	0.985	1.076	1.019	0.944	1.144	1.121	1.046	Q8TCZ2-5	CD99L2	Isoform 5 of CD99 antigen-like protein 2
			1.174	1.177	0.939	1.116	0.980	1.107				P42771-4	CDKN2A	Isoform 5 of Cyclin-dependent kinase inhibitor 2A
0.922	1.032	0.971	1.024	1.089	1.000	1.102	0.734	0.967	1.084	1.043	1.079	Q6UXH1-5	CRELD2	Isoform 5 of Cysteine-rich with EGF-like domain protein 2
1.000	1.081	1.004	1.004	0.985	0.993	0.964	1.080	0.995	1.015	0.983	0.970	Q7Z5Q1-6	CPEB2	Isoform 5 of Cytoplasmic polyadenylation element-binding protein 2
1.056	1.048	1.031	0.970	0.991	1.011	0.901	1.041	1.031	0.946	1.019	0.984	Q9H2P9-5	DPH5	Isoform 5 of Diphthine methyl ester synthase
1.057	1.049	1.057	0.981	0.938	1.034	0.986	1.077	1.011	0.993	1.013	1.008	P78563-5	ADARB1	Isoform 5 of Double-stranded RNA-specific editase 1
1.125	0.998	1.028	0.901	0.907	1.044				1.025	0.910	1.109	Q86XK2-5	FBXO11	Isoform 5 of F-box only protein 11
0.990	0.996	1.004	1.018	0.936	1.070	0.993	0.861	0.976	0.983	0.980	1.092	Q9HCN4-5	GPN1	Isoform 5 of GPN-loop GTPase 1
1.014	1.061	0.958										Q6NT76-5	HMBOX1	Isoform 5 of Homeobox-containing protein 1
1.097	0.968	1.021	1.046	1.004	1.026	0.952	1.134	1.027	0.789	0.843	1.010	Q9P2N7-5	KLHL13	Isoform 5 of Kelch-like protein 13
						1.125	1.620	0.919				Q9BVG8-6	KIFC3	Isoform 5 of Kinesin-like protein KIFC3
0.947	0.984	0.952	0.935	0.914	1.123	1.031	0.873	0.927	1.133	1.065	1.146	P27816-5	MAP4	Isoform 5 of Microtubule-associated protein 4
0.978	1.022	0.996	0.976	0.969	0.998	0.997	1.013	0.974	1.099	1.017	1.090	Q9ULH7-5	MKL2	Isoform 5 of MKL/myocardin-like protein 2
0.989	1.002	1.019	0.979	0.954	1.012	0.951	1.049	1.005	1.019	0.995	1.030	Q96T76-8	MMS19	Isoform 5 of MMS19 nucleotide excision repair protein homolog
1.321	1.497	1.285	0.787	0.969	0.486	0.790	1.206	0.918				Q9NR56-5	MBNL1	Isoform 5 of Muscleblind-like protein 1
1.009	1.017	1.007	1.010	0.945	1.022	1.034	1.010	0.963	1.002	1.007	1.014	Q8NDF8-5	PAPD5	Isoform 5 of Non-canonical poly(A) RNA polymerase PAPD5
1.027	1.083	1.106	1.003	0.938	1.066	0.979	1.002	1.119	0.986	0.929	1.026	Q9BZF3-5	OSBPL6	Isoform 5 of Oxysterol-binding protein-related protein 6
1.037	1.023	0.999	1.044	1.122	0.982	1.049	0.945	1.010	1.020	1.009	1.028	Q96JY6-5	PDLIM2	Isoform 5 of PDZ and LIM domain protein 2
1.078	1.028	1.127										P19021-5	PAM	Isoform 5 of Peptidyl-glycine alpha-amidating monooxygenase
1.033	1.020	1.029	0.998	0.963	1.004	1.014	1.040	1.026	1.031	0.994	1.066	Q8IXK0-5	PHC2	Isoform 5 of Polyhomeotic-like protein 2
									1.031	0.805	1.082	Q9Y6V0-5	PCLO	Isoform 5 of Protein piccolo
1.085	1.022	1.149	1.003	0.969	1.059	0.984	0.970	1.075	1.042	1.043	1.050	Q5R372-5	RABGAP1L	Isoform 5 of Rab GTPase-activating protein 1-like
0.995	0.989	1.010	0.986	0.987	1.002	0.952	0.912	0.982	1.005	1.013	1.036	P35241-5	RDX	Isoform 5 of Radixin

Abundance Ratio														
1 d M1/C1	1 d M2/C2	1 d M3/C3	4 d M1/C1	4 d M2/C2	4 d M3/C3	7 d M1/C1	7 d M2/C2	7 d M3/C3	10 d M1/C1	10 d M2/C2	10 d M3/C3	Accession ID	Gene name	Protein Description
1.003	1.003	1.022	0.995	0.981	1.005	1.026	1.045	1.034	1.022	0.966	1.055	P52306-5	RAP1GDS1	Isoform 5 of Rap1 GTPase-GDP dissociation stimulator 1
0.943	0.923	1.006	1.011	0.929	0.986	1.086	1.026	0.903	1.038	0.983	1.106	O76081-6	RGS20	Isoform 5 of Regulator of G-protein signaling 20
									1.026	0.920	1.104	Q9NQC3-5	RTN4	Isoform 5 of Reticulon-4
1.000	1.020	0.998	0.994	1.007	1.000	0.980	1.014	1.011	1.037	1.025	1.010	Q14155-5	ARHGEF7	Isoform 5 of Rho guanine nucleotide exchange factor 7
1.003	1.037	1.001	1.018	1.076	1.004	1.015	0.998	1.010	1.008	1.037	1.066	Q01844-5	EWSR1	Isoform 5 of RNA-binding protein EWS
0.769	1.026	1.018	1.066	1.037	1.032							Q8IWQ3-5	BRSK2	Isoform 5 of Serine/threonine-protein kinase BRSK2
1.009	1.046	1.068	1.020	0.938	1.048	1.026	1.085	1.048	1.042	0.970	1.049	O75170-5	PPP6R2	Isoform 5 of Serine/threonine-protein phosphatase 6 regulatory subunit 2
0.962	0.986	0.988	0.970	0.944	1.025	1.008	1.060	1.027	0.972	0.960	1.063	Q5H9R7-5	PPP6R3	Isoform 5 of Serine/threonine-protein phosphatase 6 regulatory subunit 3
			0.891	0.750	1.118	0.981	0.910	0.921	1.034	0.963	1.019	Q8TDB8-5	SLC2A14	Isoform 5 of Solute carrier family 2, facilitated glucose transporter member 14
						0.915	0.943	1.248	1.035	1.143	0.878	Q9NRA0-5	SPHK2	Isoform 5 of Sphingosine kinase 2
1.012	1.021	0.979	1.007	1.122	0.964	1.007	0.952	1.009	1.004	1.040	1.007	Q15637-5	SF1	Isoform 5 of Splicing factor 1
1.060	1.044	0.984	0.990	0.960	0.985	1.048	1.060	1.058	1.179	1.120	1.290	Q06787-6	FMR1	Isoform 5 of Synaptic functional regulator FMR1
1.017	0.983	0.986	0.901	0.952	0.974	0.962	1.037	0.980	0.962	0.989	1.018	P06753-5	TPM3	Isoform 5 of Tropomyosin alpha-3 chain
1.046	1.031	1.046	0.967	0.996	1.040	0.986	1.055	1.061	1.059	0.993	1.040	O94966-5	USP19	Isoform 5 of Ubiquitin carboxyl-terminal hydrolase 19
0.999	1.003	0.993	0.988	0.987	0.984	0.967	0.948	0.993	0.992	1.070	1.007	Q14157-5	UBAP2L	Isoform 5 of Ubiquitin-associated protein 2-like
0.953	1.084	0.888	1.032	0.919	0.948	1.031	1.102	0.990				Q9BUV8-5	C20orf24	Isoform 5 of Uncharacterized protein C20orf24
			0.855	1.112	1.019							Q86VK4-5	ZNF410	Isoform 5 of Zinc finger protein 410
			0.817	0.772	0.803							Q96HQ0-5	ZNF419	Isoform 5 of Zinc finger protein 419
0.834	0.919	1.228	0.878	0.637	1.057				1.267	0.981	1.283	P26367-2	PAX6	Isoform 5a of Paired box protein Pax-6
						1.209	0.768	0.972				P07108-6	DBI	Isoform 6 of Acyl-CoA-binding protein
0.993	1.014	1.040	1.037	0.981	1.005	1.146	1.031	1.123	1.076	1.108	1.038	O95490-6	ADGRL2	Isoform 6 of Adhesion G protein-coupled receptor L2
1.049	1.039	1.060	1.081	1.021	1.101	1.088	1.107	1.054	1.128	1.004	1.127	O00468-6	AGRN	Isoform 6 of Agrin
1.002	0.988	1.029	1.004	0.963	1.053	1.028	0.937	1.000	1.027	0.966	1.053	Q99996-6	AKAP9	Isoform 6 of A-kinase anchor protein 9
1.018	0.997	1.034	1.013	0.996	1.003	1.023	1.023	1.026	0.980	0.946	1.038	Q8IWZ3-6	ANKHD1	Isoform 6 of Ankyrin repeat and KH domain-containing protein 1
0.991	1.004	0.982	0.996	0.953	1.008	1.003	1.053	0.962	0.972	0.973	1.005	P56945-6	BCAR1	Isoform 6 of Breast cancer anti-estrogen resistance protein 1
1.000	1.006	1.006	0.963	0.983	0.987	0.933	0.990	0.955	0.996	0.955	1.014	Q13555-6	CAMK2G	Isoform 6 of Calcium/calmodulin-dependent protein kinase type II subunit gamma
0.956	0.982	0.992	0.951	1.003	0.975	0.918	0.791	0.943	1.005	1.013	1.021	P20810-6	CAST	Isoform 6 of Calpastatin

Abundance Ratio														
1 d M1/C1	1 d M2/C2	1 d M3/C3	4 d M1/C1	4 d M2/C2	4 d M3/C3	7 d M1/C1	7 d M2/C2	7 d M3/C3	10 d M1/C1	10 d M2/C2	10 d M3/C3	Accession ID	Gene name	Protein Description
1.001	1.007	1.021	0.996	0.945	0.991	0.978	1.079	1.010	0.995	1.005	1.004	Q9H9A5-6	CNOT10	Isoform 6 of CCR4-NOT transcription complex subunit 10
1.058	0.963	1.038	0.998	1.000	1.001	0.974	1.104	1.046	1.005	1.006	1.077	O95628-6	CNOT4	Isoform 6 of CCR4-NOT transcription complex subunit 4
1.029	1.010	1.017	1.023	1.057	0.991	1.019	1.044	1.035	0.979	0.985	1.022	Q6IA86-6	ELP2	Isoform 6 of Elongator complex protein 2
0.980	1.029	1.030	0.991	0.966	1.002	0.969	1.063	1.008	0.979	1.008	1.016	Q14C86-6	GAPVD1	Isoform 6 of GTPase-activating protein and VPS9 domain-containing protein 1
0.983	0.953	1.006	1.006	0.928	1.023	1.036	1.006	1.024	1.104	1.047	1.052	Q13308-6	PTK7	Isoform 6 of Inactive tyrosine-protein kinase 7
0.996	0.951	1.032	1.013	0.957	0.976	1.014	1.144	1.008	1.026	1.018	1.055	P20839-6	IMPDH1	Isoform 6 of Inosine-5'-monophosphate dehydrogenase 1
1.081	1.099	1.065	0.984	0.960	1.007	1.087	1.170	1.062	0.991	0.980	1.099	Q9HBG6-6	IFT122	Isoform 6 of Intraflagellar transport protein 122 homolog
1.032	1.003	1.085	0.985	0.991	1.004	0.988	0.975	0.973	1.086	1.036	1.079	O95819-6	MAP4K4	Isoform 6 of Mitogen-activated protein kinase kinase kinase kinase 4
1.255	0.921	0.770										Q8NEY8-6	PPHLN1	Isoform 6 of Periphilin-1
1.003	0.933	0.912	1.072	1.041	1.009	0.990	1.019	0.957	1.074	0.991	1.045	O94827-6	PLEKHG5	Isoform 6 of Pleckstrin homology domain-containing family G member 5
1.102	1.022	1.007	1.034	1.204	0.955	1.068	0.960	1.031	0.978	1.017	0.975	Q15366-6	PCBP2	Isoform 6 of Poly(rC)-binding protein 2
1.013	1.027	1.031	1.017	0.948	1.037	0.996	1.133	0.974	0.965	1.081	1.000	P14859-6	POU2F1	Isoform 6 of POU domain, class 2, transcription factor 1
1.012	1.037	0.959	1.032	0.962	1.012	1.018	1.072	0.969	1.108	1.095	1.063	Q9NQV6-6	PRDM10	Isoform 6 of PR domain zinc finger protein 10
1.064	0.993	1.029	0.834	0.950	0.997	0.850	0.704	1.010	0.980	0.978	1.076	Q6AWC2-6	WWC2	Isoform 6 of Protein WWC2
1.044	1.017	1.054	0.982	0.947	1.072	1.007	0.980	1.014	1.008	0.967	1.055	Q8N1W1-6	ARHGEF28	Isoform 6 of Rho guanine nucleotide exchange factor 28
1.038	1.047	0.998	1.005	0.917	1.024	0.990	1.044	1.008	1.053	1.013	1.032	Q7LG56-6	RRM2B	Isoform 6 of Ribonucleoside-diphosphate reductase subunit M2 B
0.999	0.931	1.019	0.973	0.928	0.982	1.003	1.006	1.017	1.044	0.907	1.054	Q5VT25-6	CDC42BPA	Isoform 6 of Serine/threonine-protein kinase MRCK alpha
0.994	1.006	1.019	0.997	0.981	1.013	1.026	1.009	0.992	1.035	1.010	1.046	Q9H4A3-7	WNK1	Isoform 6 of Serine/threonine-protein kinase WNK1
1.056	1.008	1.016	1.038	0.981	1.005	1.016	1.051	0.985	0.967	1.048	0.967	P29353-6	SHC1	Isoform 6 of SHC-transforming protein 1
1.012	1.029	1.200	0.967	0.962	1.034							Q9HB58-6	SP110	Isoform 6 of Sp110 nuclear body protein
			1.101	0.956	0.918	0.964	1.196	0.918	0.983	1.170	0.938	Q9NWM0-6	SMOX	Isoform 6 of Spermine oxidase
1.004	1.160	0.997	1.142	1.516	0.896	1.054	1.071	1.167	1.023	1.053	0.964	Q15637-6	SF1	Isoform 6 of Splicing factor 1
			1.028	1.021	1.181							P24821-6	TNC	Isoform 6 of Tenascin
0.984	0.933	1.031	0.931	0.952	1.022	0.932	1.021	1.011	1.000	0.958	1.008	O00142-6	TK2	Isoform 6 of Thymidine kinase 2, mitochondrial
1.035	0.983	1.044	0.977	0.955	0.992	0.956	0.896	0.980	0.989	0.997	1.003	P06753-6	TPM3	Isoform 6 of Tropomyosin alpha-3 chain

Abundance Ratio												Accession ID	Gene name	Protein Description
1 d M1/C1	1 d M2/C2	1 d M3/C3	4 d M1/C1	4 d M2/C2	4 d M3/C3	7 d M1/C1	7 d M2/C2	7 d M3/C3	10 d M1/C1	10 d M2/C2	10 d M3/C3			
0.999	1.036	0.999	1.072	1.052	1.032	1.010	1.029	0.943	0.822	0.837	1.041	Q7Z589-7	EMSY	Isoform 7 of BRCA2-interacting transcriptional repressor EMSY
0.935	1.013	1.096				1.074	1.197	1.261				P38398-7	BRCA1	Isoform 7 of Breast cancer type 1 susceptibility protein
0.986	0.990	0.998	0.978	1.004	0.997	0.981	0.975	0.998	0.972	1.016	0.963	Q12906-7	ILF3	Isoform 7 of Interleukin enhancer-binding factor 3
0.973	0.930	0.990	0.960	0.890	1.011	1.082	1.076	0.997	1.026	0.996	1.040	Q6P1Q0-7	LETMD1	Isoform 7 of LETM1 domain-containing protein 1
1.028	0.999	0.990	1.009	0.999	0.984	0.966	1.021	0.983	0.990	0.988	1.006	P22570-7	FDXR	Isoform 7 of NADPH:adrenodoxin oxidoreductase, mitochondrial
1.106	1.086	1.291	1.044	1.158	1.054	1.088	0.974	1.004	1.001	1.073	1.059	Q14693-7	LPIN1	Isoform 7 of Phosphatidate phosphatase LPIN1
1.056	0.984	1.013	0.961	1.099	1.049							Q8NDX5-7	PHC3	Isoform 7 of Polyhomeotic-like protein 3
1.061	1.114	1.116	0.945	1.130	1.016	1.062	1.174	1.123	1.015	0.949	1.183	Q9Y2Z2-6	MTO1	Isoform 7 of Protein MTO1 homolog, mitochondrial
1.074	1.023	0.980	0.976	1.052	0.964	1.057	0.991	0.962	1.087	1.104	1.194	Q13438-7	OS9	Isoform 7 of Protein OS-9
0.991	1.014	1.003	0.985	0.990	0.995	0.983	0.912	0.969	1.020	1.014	1.048	Q9Y520-7	PRRC2C	Isoform 7 of Protein PRRC2C
0.953	1.027	1.056	1.038	0.932	1.080	1.089	1.086	1.018	1.082	1.048	1.132	Q9Y6M7-7	SLC4A7	Isoform 7 of Sodium bicarbonate cotransporter 3
0.984	0.994	0.965	0.996	1.004	0.977	0.978	1.001	1.009	0.950	0.972	0.985	O43399-7	TPD52L2	Isoform 7 of Tumor protein D54
1.087	0.954	0.811										O75771-8	RAD51D	Isoform 8 of DNA repair protein RAD51 homolog 4
0.981	1.015	1.035	0.996	0.940	1.043	1.004	0.958	1.056	1.056	0.997	1.139	Q96RT1-8	ERBIN	Isoform 8 of Erbin
0.900	0.972	0.979	0.957	1.091	1.079	1.009	1.026	1.075	1.122	1.014	1.095	Q04637-8	EIF4G1	Isoform 8 of Eukaryotic translation initiation factor 4 gamma 1
0.988	0.995	0.989	0.988	1.020	0.960	0.964	0.990	0.975	0.937	0.963	0.932	O75369-8	FLNB	Isoform 8 of Filamin-B
1.102	0.965	1.054							1.045	0.993	0.987	Q15149-8	PLEC	Isoform 8 of Plectin
0.943	0.984	0.977	0.963	0.982	1.052	0.975	1.011	1.026	1.012	1.039	1.093	O43251-8	RBFOX2	Isoform 8 of RNA binding protein fox-1 homolog 2
1.036	1.028	1.023	1.010	0.961	1.006	0.952	0.989	0.981	0.991	1.042	0.994	Q06787-8	FMR1	Isoform 8 of Synaptic functional regulator FMR1
1.054	1.014	1.013	0.978	1.000	1.028	0.978	1.055	1.006	0.956	0.933	0.995	Q9NVH6-8	TMLHE	Isoform 8 of Trimethyllysine dioxygenase, mitochondrial
1.012	1.004	1.013	1.008	0.918	0.981	1.035	1.039	1.017	1.010	0.963	1.058	Q86UV5-8	USP48	Isoform 8 of Ubiquitin carboxyl-terminal hydrolase 48
1.011	0.991	1.014	1.026	1.061	0.997	1.039	0.997	1.052	1.000	0.964	1.019	Q14790-9	CASP8	Isoform 9 of Caspase-8
0.971	1.005	1.025	0.996	0.979	1.017	0.994	0.993	1.008	0.952	1.010	1.000	Q04637-9	EIF4G1	Isoform 9 of Eukaryotic translation initiation factor 4 gamma 1
1.047	1.008	1.051	0.981	1.019	1.020	1.014	0.921	1.007	1.008	1.070	1.102	Q9UIS9-9	MBD1	Isoform 9 of Methyl-CpG-binding domain protein 1
0.995	0.980	1.013	1.015	0.901	1.014	0.982	1.067	1.027	0.994	1.024	1.078	P33527-9	ABCC1	Isoform 9 of Multidrug resistance-associated protein 1
0.936	0.962	0.982	0.950	0.962	1.041	0.938	0.899	0.953	1.006	0.944	1.064	O96017-9	CHEK2	Isoform 9 of Serine/threonine-protein kinase Chk2

Abundance Ratio														
1 d M1/C1	1 d M2/C2	1 d M3/C3	4 d M1/C1	4 d M2/C2	4 d M3/C3	7 d M1/C1	7 d M2/C2	7 d M3/C3	10 d M1/C1	10 d M2/C2	10 d M3/C3	Accession ID	Gene name	Protein Description
1.000	0.985	1.040	0.976	0.904	1.043	0.982	1.047	0.981	1.053	1.041	1.017	O94901-9	SUN1	Isoform 9 of SUN domain-containing protein 1
0.997	1.016	0.988	0.992	0.990	1.012	1.000	1.041	1.002	1.017	1.007	1.000	P46937-9	YAP1	Isoform 9 of Transcriptional coactivator YAP1
1.083	1.027	1.212				1.073	1.081	1.123				P53367-2	ARFIP1	Isoform A of Arfaptin-1
1.029	0.987	1.047	1.002	0.978	1.017	0.979	1.078	1.042	0.953	0.995	1.005	O60888-2	CUTA	Isoform A of Protein CutA
1.020	0.985	1.071	0.990	0.905	1.045	0.925	0.999	1.015	0.939	0.928	1.086	O14662-2	STX16	Isoform A of Syntaxin-16
			1.238	0.982	0.487	1.877	1.508	0.695				P09651-2	HNRNPA1	Isoform A1-A of Heterogeneous nuclear ribonucleoprotein A1
1.016	1.051	0.995	1.076	1.036	1.026	0.985	1.059	1.054	0.932	0.953	1.009	P55210-3	CASP7	Isoform Alpha' of Caspase-7
						1.055	1.650	0.933				Q92692-2	NECTIN2	Isoform Alpha of Nectin-2
1.058	1.054	0.959	1.122	1.189	1.069	1.268	1.151	1.291	1.095	0.692	0.971	Q13033-2	STRN3	Isoform Alpha of Striatin-3
1.010	1.017	1.063	0.936	1.022	0.968	0.959	1.076	1.005	0.941	0.949	1.033	P04150-3	NR3C1	Isoform Alpha-2 of Glucocorticoid receptor
			1.121	0.981	1.104							P09471-2	GNAO1	Isoform Alpha-2 of Guanine nucleotide-binding protein G(o) subunit alpha
									0.948	0.856	1.041	Q01196-8	RUNX1	Isoform AML-1G of Runt-related transcription factor 1
0.995	0.977	0.790	0.913	0.990	1.142	1.271	0.911	0.967	1.066	1.093	1.177	O15265-2	ATXN7	Isoform b of Ataxin-7
1.036	0.995	1.027	1.069	1.061	1.040	1.084	1.010	1.048	1.087	1.009	1.065	O95340-2	PAPSS2	Isoform B of Bifunctional 3'-phosphoadenosine 5'-phosphosulfate synthase 2
1.067	1.045	0.958	1.078	1.016	1.003	1.048	0.997	0.938	1.017	0.889	0.995	O75190-2	DNAJB6	Isoform B of DnaJ homolog subfamily B member 6
0.912	0.968	0.941	0.972	0.979	1.013	1.096	0.944	1.033	1.095	1.073	1.104	P15529-2	CD46	Isoform B of Membrane cofactor protein
0.995	1.008	1.023	0.978	0.986	1.036	0.996	0.979	0.984	1.040	1.026	1.016	P51608-2	MECP2	Isoform B of Methyl-CpG-binding protein 2
1.032	0.996	1.028	1.012	1.074	1.019	0.984	0.962	1.033	1.021	1.004	1.014	Q96EE3-1	SEH1L	Isoform B of Nucleoporin SEH1
0.953	0.987	0.933	0.982	0.882	0.992	0.977	1.084	1.001	0.998	1.062	0.917	Q00325-2	SLC25A3	Isoform B of Phosphate carrier protein, mitochondrial
									0.834	0.953	1.021	Q9UN86-2	G3BP2	Isoform B of Ras GTPase-activating protein-binding protein 2
1.130	1.256	1.236	1.091	0.981	0.897							P61812-2	TGFB2	Isoform B of Transforming growth factor beta-2
0.947	0.957	1.085	1.039	0.946	0.982	1.007	0.960	0.963	0.925	0.888	1.114	P53814-5	SMTN	Isoform B2 of Smoothelin
0.902	1.025	0.968	0.972	1.061	1.021	0.953	1.159	1.003	1.013	1.202	1.053	Q07812-2	BAX	Isoform Beta of Apoptosis regulator BAX
			0.909	0.898	0.847							Q9UPN9-2	TRIM33	Isoform Beta of E3 ubiquitin-protein ligase TRIM33
1.012	0.970	1.010	0.953	0.985	1.028	0.977	0.866	0.940	0.941	1.021	0.977	Q14978-2	NOLC1	Isoform Beta of Nucleolar and coiled-body phosphoprotein 1
1.022	1.003	0.995	0.997	1.047	1.010	1.028	1.008	1.011	1.004	0.973	1.008	Q02880-2	TOP2B	Isoform Beta-1 of DNA topoisomerase 2-beta
			1.176	0.844	1.015							O75688-2	PPM1B	Isoform Beta-2 of Protein phosphatase 1B
2.173	1.016	1.110	1.146	2.090	0.801	0.990	0.829	1.046	0.924	1.043	0.829	Q10567-3	AP1B1	Isoform C of AP-1 complex subunit beta-1
0.881	0.799	1.192	1.012	0.972	0.998	0.950	0.945	0.893				P02545-2	LMNA	Isoform C of Prelamin-A/C

Abundance Ratio														
1 d M1/C1	1 d M2/C2	1 d M3/C3	4 d M1/C1	4 d M2/C2	4 d M3/C3	7 d M1/C1	7 d M2/C2	7 d M3/C3	10 d M1/C1	10 d M2/C2	10 d M3/C3	Accession ID	Gene name	Protein Description
1.153	1.149	0.991	0.971	0.934	1.023	1.044	1.179	1.059	1.032	1.023	0.997	Q9NS23-4	RASSF1	Isoform C of Ras association domain-containing protein 1
1.064	1.032	0.967	1.004	0.997	0.969	1.011	1.143	1.000	1.021	1.030	0.960	Q93062-3	RBPMS	Isoform C of RNA-binding protein with multiple splicing
1.047	0.991	1.016	0.974	0.953	1.010	0.951	0.955	0.979	0.976	1.007	0.957	P07910-2	HNRNPC	Isoform C1 of Heterogeneous nuclear ribonucleoproteins C1/C2
1.052	1.012	1.053	1.044	1.037	1.002	1.025	1.014	1.000	1.000	0.937	1.034	Q7Z6K5-2	ARPIN	Isoform C15orf38-AP3S2 of Arpin
1.484	1.086	0.966	1.112	0.915	0.899	0.941	1.010	0.955	0.838	1.006	0.741	P46108-2	CRK	Isoform Crk-I of Adapter molecule crk
1.070	1.019	0.991	0.978	0.936	0.912	1.093	1.152	0.998	1.002	0.967	1.019	Q16539-2	MAPK14	Isoform CSBP1 of Mitogen-activated protein kinase 14
0.954	0.975	0.984	0.935	0.966	0.976	0.928	0.902	0.966	1.002	0.979	1.030	O94916-4	NFAT5	Isoform D of Nuclear factor of activated T-cells 5
0.993	0.985	1.013	1.001	0.979	1.039	1.005	0.999	1.012	1.030	0.989	1.049	P18583-5	SON	Isoform D of Protein SON
1.318	1.017	1.019	0.780	1.279	0.962	0.949	0.913	1.084				O00273-2	DFFA	Isoform DFF35 of DNA fragmentation factor subunit alpha
0.938	0.930	0.968	0.993	1.033	0.941							O94916-5	NFAT5	Isoform E of Nuclear factor of activated T-cells 5
0.985	1.135	0.832	0.860	0.877	1.049	0.806	0.995	1.102				P18583-6	SON	Isoform E of Protein SON
1.025	1.022	1.000	1.019	0.974	0.973	1.010	1.039	1.006	1.042	1.029	1.017	Q9NZB2-6	FAM120A	Isoform F of Constitutive coactivator of PPAR-gamma-like protein 1
1.090	0.976	1.202	0.917	1.063	1.061	0.780	0.757	1.052	1.045	0.908	1.124	Q07866-3	KLC1	Isoform G of Kinesin light chain 1
0.945	0.896	1.041	0.914	0.902	1.024	1.004	0.832	0.941	1.134	1.035	1.138	P42167-2	TMPO	Isoform Gamma of Lamina-associated polypeptide 2, isoforms beta/gamma
0.919	0.948	0.919				0.905	1.031	1.068	1.093	0.973	1.056	Q13362-2	PPP2R5C	Isoform Gamma-1 of Serine/threonine-protein phosphatase 2A 56 kDa regulatory subunit gamma isoform
0.975	1.002	1.024	1.032	0.932	1.086	1.028	1.031	1.065	1.022	1.006	1.023	P36873-2	PPP1CC	Isoform Gamma-2 of Serine/threonine-protein phosphatase PP1-gamma catalytic subunit
1.088	0.787	0.879	0.919	0.870	0.984							P63092-2	GNAS	Isoform Gnas-2 of Guanine nucleotide-binding protein G(s) subunit alpha isoforms short
			0.906	1.000	1.417	0.872	0.794	0.894	1.033	1.049	1.124	P43250-2	GRK6	Isoform GRK6B of G protein-coupled receptor kinase 6
0.973	1.009	0.972	0.986	1.013	0.998	0.997	0.983	0.996	1.000	0.986	0.979	P36542-2	ATP5C1	Isoform Heart of ATP synthase subunit gamma, mitochondrial
1.019	0.922	0.987	0.746	0.853	0.975	0.808	0.626	0.785	1.120	0.798	0.751	P17096-2	HMGA1	Isoform HMG-Y of High mobility group protein HMG-I/HMG-Y
1.018	0.972	0.970	0.990	0.932	1.044	0.899	0.953	1.015	0.802	1.251	0.867	P00519-2	ABL1	Isoform IB of Tyrosine-protein kinase ABL1
1.090	0.976	1.019	0.991	0.929	1.024	0.965	1.167	1.026	1.064	1.060	1.007	Q9P2H3-3	IFT80	Isoform IFT80-L of Intraflagellar transport protein 80 homolog
0.998	0.988	0.994	0.992	1.005	0.998	0.988	1.000	1.001	1.014	0.968	1.045	O00499-2	BIN1	Isoform IIB of Myc box-dependent-interacting protein 1

Abundance Ratio														
1 d M1/C1	1 d M2/C2	1 d M3/C3	4 d M1/C1	4 d M2/C2	4 d M3/C3	7 d M1/C1	7 d M2/C2	7 d M3/C3	10 d M1/C1	10 d M2/C2	10 d M3/C3	Accession ID	Gene name	Protein Description
0.977	1.002	0.983	0.986	1.041	0.974	1.051	1.024	1.016	1.025	1.024	1.027	Q14195-2	DPYSL3	Isoform LCRMP-4 of Dihydropyrimidinase-related protein 3
1.016	1.009	0.975	1.014	1.032	0.998	1.010	1.019	0.997	1.056	1.011	0.993	P11413-2	G6PD	Isoform Long of Glucose-6-phosphate 1-dehydrogenase
0.995	1.002	0.986	1.000	0.992	0.976	0.982	1.004	0.985	1.003	0.992	0.996	P25786-2	PSMA1	Isoform Long of Proteasome subunit alpha type-1
0.988	0.986	1.009	0.961	0.965	0.960	0.965	0.988	0.960	1.006	0.998	1.044	Q92890-1	UFD1	Isoform Long of Ubiquitin recognition factor in ER-associated degradation protein 1
1.028	0.876	1.018	0.949	0.880	1.085	1.171	1.168	1.356	1.025	0.847	1.011	P14618-2	PKM	Isoform M1 of Pyruvate kinase PKM
0.670	0.939	0.915	1.002	0.930	1.111	0.991	0.880	0.989	0.979	1.028	0.937	P06733-2	ENO1	Isoform MBP-1 of Alpha-enolase
1.067	0.972	1.007	0.944	0.954	1.099	0.974	1.029	1.060	1.087	1.146	1.085	Q14814-4	MEF2D	Isoform MEF2DA0 of Myocyte-specific enhancer factor 2D
0.983	0.911	1.002	0.969	0.956	1.028	1.076	1.059	1.025	0.989	1.012	0.976	P54136-2	RARS	Isoform Monomeric of Arginine--tRNA ligase, cytoplasmic
0.995	0.996	0.988	1.026	1.053	1.011	1.017	1.021	1.021	1.021	1.021	1.001	Q07866-6	KLC1	Isoform N of Kinesin light chain 1
1.038	0.991	0.997	0.994	1.079	0.991	0.993	0.882	1.014	1.017	0.993	0.987	P09496-2	CLTA	Isoform Non-brain of Clathrin light chain A
0.986	0.977	1.022	0.989	0.967	1.029	0.989	0.902	1.008	0.980	1.027	1.008	P09497-2	CLTB	Isoform Non-brain of Clathrin light chain B
0.999	1.017	1.067	1.027	0.984	1.027	1.013	1.022	0.994	1.005	0.968	1.026	Q9Y446-2	PKP3	Isoform PKP3b of Plakophilin-3
									1.094	0.994	1.216	P29590-3	PML	Isoform PML-8 of Protein PML
1.011	1.003	1.012	1.001	0.999	0.989	1.009	1.018	1.026	1.039	0.971	1.056	Q12979-2	ABR	Isoform Short of Active breakpoint cluster region-related protein
1.059	1.001	1.017	1.058	1.133	0.989	1.051	0.970	1.098	1.022	1.004	1.041	Q15056-2	EIF4H	Isoform Short of Eukaryotic translation initiation factor 4H
0.693	0.847	1.105	0.588	1.344	1.045	0.928	0.395	1.063	1.182	0.849	1.430	Q00839-2	HNRNPU	Isoform Short of Heterogeneous nuclear ribonucleoprotein U
1.270	1.102	0.698										P23246-2	SFPQ	Isoform Short of Splicing factor, proline- and glutamine-rich
1.004	0.988	0.995	1.003	1.059	1.010	1.024	0.990	1.017	0.980	1.010	0.966	P14678-3	SNRPB	Isoform SM-B1 of Small nuclear ribonucleoprotein-associated proteins B and B'
0.969	1.002	1.011	0.989	0.983	0.977	1.005	0.992	0.975	0.995	0.959	1.017	O95425-3	SVIL	Isoform SV3 of Supervillin
1.024	0.976	0.955	1.012	1.026	0.971	1.023	1.000	1.243				P10636-5	MAPT	Isoform Tau-C of Microtubule-associated protein tau
						0.938	1.560	1.357	1.126	0.883	0.822	Q9BX59-7	TAPBPL	Isoform zeta of Tapasin-related protein
0.992	0.997	1.016	0.994	0.968	0.982	0.980	1.025	0.996	0.957	0.977	0.954	P41252	IARS	Isoleucine--tRNA ligase, cytoplasmic
0.988	0.998	0.978	0.988	1.007	0.970	0.984	1.017	0.984	0.991	0.987	0.962	Q9NSE4	IARS2	Isoleucine--tRNA ligase, mitochondrial
0.955	1.004	0.988	1.015	0.963	1.010	1.016	1.032	0.978	1.082	0.987	1.013	A0A0A0MT83	IVD	Isovaleryl-CoA dehydrogenase, mitochondrial
1.067	0.740	0.923	1.043	1.028	0.959	0.857	1.171	1.139	0.993	1.043	0.980	P0C870	JMJD7	JmjC domain-containing protein 7
			0.920	0.916	0.987	0.970	0.964	1.087	1.178	0.984	0.957	Q8N157	AHI1	Jouberin
1.053	0.952	1.087	1.053	0.958	1.010	0.957	1.081	1.011	1.197	0.900	1.146	Q86XJ5	JRK	JRK protein

Abundance Ratio														
1 d M1/C1	1 d M2/C2	1 d M3/C3	4 d M1/C1	4 d M2/C2	4 d M3/C3	7 d M1/C1	7 d M2/C2	7 d M3/C3	10 d M1/C1	10 d M2/C2	10 d M3/C3	Accession ID	Gene name	Protein Description
0.939	1.004	1.011	0.983	0.996	0.964	0.999	1.045	0.996	0.975	0.973	1.000	P14923	JUP	Junction plakoglobin
0.947	0.978	0.980	0.964	0.894	0.991	0.989	1.116	1.016	1.067	1.001	1.070	Q9Y624	F11R	Junctional adhesion molecule A
0.873	0.914	1.144	0.996	0.930	0.948	0.945	0.861	0.980	1.000	0.960	1.089	Q8N9B5	JMY	Junction-mediating and -regulatory protein
1.061	0.999	1.037	0.965	1.010	1.003	0.974	1.057	1.021	0.972	0.995	1.003	Q9BWU0	SLC4A1AP	Kanadaptin
1.179	1.022	1.063	0.934	1.101	1.033	0.860	1.163	1.020	0.814	0.990	1.052	Q9Y664	KPTN	Kaptin
1.048	0.956	1.106							1.024	0.891	1.025	A0A0G2JNT7	KANSL1	KAT8 regulatory NSL complex subunit 1
1.016	1.115	0.880	1.462	0.834	1.027	1.192	0.841	1.306	1.056	1.070	1.113	F8VX10	KANSL2	KAT8 regulatory NSL complex subunit 2
0.922	1.053	1.041	0.996	0.853	0.879	1.075	1.038	1.016				Q9P2N6	KANSL3	KAT8 regulatory NSL complex subunit 3
1.015	1.028	1.003	1.045	1.061	0.974	1.155	0.901	0.930	0.944	0.978	0.869	O75449	KATNA1	Katanin p60 ATPase-containing subunit A1
1.039	0.993	0.952	1.051	0.846	0.912	0.883	1.123	1.145	0.971	0.971	1.079	Q9BW62	KATNAL1	Katanin p60 ATPase-containing subunit A-like 1
0.951	0.961	1.027	1.025	1.010	1.040	1.011	1.082	1.080	0.977	0.998	1.048	Q9BVA0	KATNB1	Katanin p80 WD40 repeat-containing subunit B1
0.988	0.907	0.975	0.952	0.886	1.015	0.932	0.906	1.066				Q6UW63	KDELC1	KDEL motif-containing protein 1
1.005	1.010	1.037	1.047	0.992	1.007	0.991	1.086	1.017	0.961	0.982	0.976	Q7Z4H8	KDELC2	KDEL motif-containing protein 2
									0.984	0.893	1.177	G3V5V5	KLHDC1	Kelch domain-containing protein 1 (Fragment)
1.172	0.987	1.044	0.989	0.929	0.976	0.926	1.156	0.892				Q6PID8	KLHDC10	Kelch domain-containing protein 10
						0.720	0.755	1.189	0.900	0.891	1.684	Q9Y2U9	KLHDC2	Kelch domain-containing protein 2
1.126	1.035	0.993				0.914	1.008	1.068	0.869	1.093	0.953	E7ERR0	KLHDC3	Kelch domain-containing protein 3
0.972	1.008	0.979	0.987	0.925	1.015	1.043	1.027	0.985	1.120	1.044	1.061	Q8TBB5	KLHDC4	Kelch domain-containing protein 4
0.988	0.995	0.992	1.060	0.941	1.081	1.010	1.131	0.997	0.970	1.010	0.982	Q8IY47	KBTBD2	Kelch repeat and BTB domain-containing protein 2
1.065	1.015	1.004	0.996	0.892	1.003	0.985	1.027	0.973	0.941	0.970	0.967	A0A075B6T4	KBTBD4	Kelch repeat and BTB domain-containing protein 4
1.032	1.000	0.968	0.999	0.939	0.990	1.009	1.121	1.056	1.035	1.022	0.934	Q14145	KEAP1	Kelch-like ECH-associated protein 1
0.970	1.015	0.915	1.088	0.972	1.035	1.020	1.120	1.340	0.900	0.957	0.930	Q9NVR0	KLHL11	Kelch-like protein 11
1.094	1.032	1.190	0.962	0.897	0.888	0.886	0.933	0.800				Q9P2G3	KLHL14	Kelch-like protein 14
									0.950	0.986	0.960	O94889	KLHL18	Kelch-like protein 18
									0.925	1.171	1.039	Q9Y2M5	KLHL20	Kelch-like protein 20
			0.844	0.971	0.963	0.720	0.593	1.377	1.413	1.352	1.206	Q96CT2	KLHL29	Kelch-like protein 29
1.221	1.407	1.202	0.935	0.964	1.018	0.971	1.087	0.924	1.057	0.991	1.127	Q8N4N3	KLHL36	Kelch-like protein 36
			0.973	1.039	1.020							Q9P2K6	KLHL42	Kelch-like protein 42
1.095	1.019	1.254	1.292	1.449	1.117	0.826	1.030	1.195	0.990	0.996	1.160	Q8IXQ5	KLHL7	Kelch-like protein 7
			1.305	0.830	1.231	1.031	1.140	1.040	1.034	1.139	1.044	A0A1B0GVI3	KRT10	Keratin, type I cytoskeletal 10 (Fragment)
1.249	0.706	1.061	1.099	1.023	0.481	0.690	0.647	0.492	0.656	1.335	0.812	P02533	KRT14	Keratin, type I cytoskeletal 14
			0.935	0.898	0.949	1.057	0.943	0.982	1.129	1.052	1.122	P19012	KRT15	Keratin, type I cytoskeletal 15
0.989	0.994	1.052	0.981	1.099	1.007	1.066	1.011	1.066	1.015	1.146	1.027	Q04695	KRT17	Keratin, type I cytoskeletal 17

Abundance Ratio														
1 d M1/C1	1 d M2/C2	1 d M3/C3	4 d M1/C1	4 d M2/C2	4 d M3/C3	7 d M1/C1	7 d M2/C2	7 d M3/C3	10 d M1/C1	10 d M2/C2	10 d M3/C3	Accession ID	Gene name	Protein Description
0.973	0.997	1.000	0.987	0.962	0.982	0.967	0.980	0.993	0.969	1.005	0.979	P05783	KRT18	Keratin, type I cytoskeletal 18
0.942	0.987	0.976	1.130	1.444	0.676	0.694	0.777	0.583	0.925	1.349	0.542	P35527	KRT9	Keratin, type I cytoskeletal 9
0.962	1.013	0.983	1.088	1.216	0.711	0.730	0.803	0.580	0.868	1.310	0.603	P04264	KRT1	Keratin, type II cytoskeletal 1
1.055	1.072	0.987	1.237	1.134	0.961	0.864	0.835	0.481	0.870	1.186	0.618	P35908	KRT2	Keratin, type II cytoskeletal 2 epidermal
0.949	0.871	1.082	1.037	1.306	0.695	0.825	0.746	0.553	0.811	1.377	0.764	P13647	KRT5	Keratin, type II cytoskeletal 5
1.626	0.766	1.172	0.874	1.244	0.509	0.852	0.499	0.440	0.620	1.296	0.873	P04259	KRT6B	Keratin, type II cytoskeletal 6B
0.968	0.967	1.030	0.959	0.999	1.016	1.031	1.058	1.052	0.955	0.966	1.033	Q6KB66	KRT80	Keratin, type II cytoskeletal 80
0.883	0.775	1.054	0.974	0.946	1.051	0.979	0.978	1.017	1.014	0.877	1.078	Q8NC54	KCT2	Keratinocyte-associated transmembrane protein 2
1.007	1.020	1.058	0.998	1.019	1.014	0.958	0.917	1.030	1.015	1.018	1.051	Q9HA64	FN3KRP	Ketosamine-3-kinase
1.009	0.989	1.000	0.985	0.962	0.979	0.957	0.943	0.970	1.019	1.045	0.976	Q07666	KHDRBS1	KH domain-containing, RNA-binding, signal transduction-associated protein 1
1.079	1.056	1.060	0.987	0.936	1.033	1.028	1.017	0.974	0.976	1.009	1.092	O75525	KHDRBS3	KH domain-containing, RNA-binding, signal transduction-associated protein 3
0.974	1.006	1.016	0.986	0.976	1.000	1.004	1.059	1.034	0.996	1.014	1.049	J3KN16	KIAA0368	KIAA0368
			0.941	1.023	0.983	0.999	1.136	0.960	1.060	1.030	1.047	Q5SVS4	SLC25A30	Kidney mitochondrial carrier protein 1
0.981	1.009	0.978	0.985	0.953	0.989	1.026	1.022	1.047	1.003	0.957	1.067	A0A1B0GUA3	KIF1BP	KIF1-binding protein
1.009	1.012	1.042	1.061	0.828	1.054	1.015	1.126	1.068	0.996	1.108	1.048	Q9ULH0	KIDINS220	Kinase D-interacting substrate of 220 kDa
1.124	1.018	0.961	1.068	0.905	0.976	1.029	1.105	0.944	1.086	1.051	1.033	Q8IVT5	KSR1	Kinase suppressor of Ras 1
0.958	1.001	1.008	0.986	0.973	0.996	1.005	0.978	0.985	1.049	1.040	1.020	Q86UP2	KTN1	Kinectin
1.068	1.054	0.986	1.008	0.870	1.018	1.003	1.219	1.405				O60282	KIF5C	Kinesin heavy chain isoform 5C
1.028	0.971	1.022	1.003	0.986	1.021	1.018	1.022	0.989	1.103	0.993	1.043	Q9H0B6	KLC2	Kinesin light chain 2
0.989	0.988	1.010	0.996	1.010	1.021	0.990	0.992	1.026	1.000	1.018	1.037	P33176	KIF5B	Kinesin-1 heavy chain
0.996	1.010	1.030	1.005	0.973	1.007	1.018	1.060	0.964	1.006	1.000	0.986	Q92845	KIFAP3	Kinesin-associated protein 3
0.947	0.978	1.015	1.018	1.022	1.010	1.105	1.209	1.127	0.949	0.901	1.080	P52732	KIF11	Kinesin-like protein KIF11
1.059	0.846	1.304	0.949	1.005	0.991							Q96FN5	KIF12	Kinesin-like protein KIF12
1.028	0.998	1.027	0.992	0.952	0.980	1.025	1.058	0.981	1.027	0.951	1.025	Q9NQT8	KIF13B	Kinesin-like protein KIF13B
1.003	1.045	1.124	1.036	0.921	1.099	0.992	1.117	1.007	0.931	0.948	1.098	Q15058	KIF14	Kinesin-like protein KIF14
0.947	1.001	1.028	0.917	0.974	1.028	0.572	0.633	0.740	0.942	0.978	0.939	Q9NS87	KIF15	Kinesin-like protein KIF15
0.955	0.987	1.121	0.989	0.949	1.034	1.017	1.004	1.005	1.010	0.980	1.044	Q96L93	KIF16B	Kinesin-like protein KIF16B
1.371	1.211	1.157				0.936	1.096	1.138				Q8NI77	KIF18A	Kinesin-like protein KIF18A
0.996	0.978	0.996	1.031	0.948	1.053	1.065	1.066	1.042	1.010	1.015	1.032	O43896	KIF1C	Kinesin-like protein KIF1C
1.007	1.035	1.020							1.070	0.753	1.223	O95235	KIF20A	Kinesin-like protein KIF20A
0.990	0.952	0.980	0.943	0.873	0.977	1.103	1.061	0.945	0.981	0.994	1.012	H0YI78	KIF21A	Kinesin-like protein KIF21A (Fragment)
0.898	1.020	1.081							0.876	1.003	0.998	Q14807	KIF22	Kinesin-like protein KIF22

Abundance Ratio														
1 d M1/C1	1 d M2/C2	1 d M3/C3	4 d M1/C1	4 d M2/C2	4 d M3/C3	7 d M1/C1	7 d M2/C2	7 d M3/C3	10 d M1/C1	10 d M2/C2	10 d M3/C3	Accession ID	Gene name	Protein Description
0.935	1.013	1.014	1.026	0.964	0.924	1.078	1.225	1.044	0.817	0.840	1.024	Q99661	KIF2C	Kinesin-like protein KIF2C
1.020	1.007	1.003	1.050	0.969	1.008	0.982	0.956	0.987	1.045	0.987	1.038	O15066	KIF3B	Kinesin-like protein KIF3B
0.901	0.972	1.043	0.969	0.944	1.043	0.971	0.880	0.976	1.107	1.143	1.256	Q2M1P5	KIF7	Kinesin-like protein KIF7
0.966	0.976	1.031	1.024	0.992	0.991	1.017	1.004	0.927	0.861	0.951	0.963	Q9BW19	KIFC1	Kinesin-like protein KIFC1
1.004	0.988	0.998	1.007	0.968	1.023	0.978	1.062	1.017	0.997	0.996	1.047	E9PES4	KIF3A	Kinesin-like protein
0.973	1.023	0.982	1.032	0.994	1.077	1.020	0.966	0.961	1.009	0.984	0.993	O14777	NDC80	Kinetochore protein NDC80 homolog
0.951	0.991	1.034	1.036	0.900	0.940				0.950	0.912	1.207	Q9BZD4	NUF2	Kinetochore protein Nuf2
0.996	0.961	0.970	0.991	1.165	1.116	0.925	1.185	1.090	0.928	0.953	0.995	K7EMX1	SPC24	Kinetochore protein Spc24 (Fragment)
1.001	1.017	0.985	1.147	4.181	1.082	0.939	1.432	1.025	0.987	1.250	1.118	Q9HBM1	SPC25	Kinetochore protein Spc25
1.018	1.030	1.054										Q8NG31	KNL1	Kinetochore scaffold 1
1.025	0.970	1.015	1.011	0.919	1.018	1.053	1.107	0.994	1.013	0.965	1.035	P50748	KNTC1	Kinetochore-associated protein 1
1.020	0.995	0.978	0.973	0.932	1.056	1.017	1.035	1.011	1.056	0.955	1.028	Q9H410	DSN1	Kinetochore-associated protein DSN1 homolog
1.029	0.985	0.957	1.046	0.911	1.029	0.994	1.097	1.099	1.022	0.994	0.993	Q96IY1	NSL1	Kinetochore-associated protein NSL1 homolog
0.895	0.970	1.074	0.972	0.865	1.065	0.989	1.039	0.966	1.098	0.995	1.219	Q14678	KANK1	KN motif and ankyrin repeat domain-containing protein 1
0.821	0.902	0.959	0.896	0.740	0.936							O00522	KRIT1	Krev interaction trapped protein 1
0.989	1.001	0.970	0.984	0.952	1.026	1.006	0.998	1.042	0.972	1.018	0.972	Q13601	KRR1	KRR1 small subunit processome component homolog
1.501	0.986	0.953	1.008	1.012	1.067	1.024	0.921	0.954	1.138	1.013	1.055	Q9BXK1	KLF16	Krueppel-like factor 16
			0.956	0.865	0.924	0.729	0.491	0.880	0.997	1.119	1.010	O43474	KLF4	Krueppel-like factor 4
			0.896	0.925	1.027				1.010	0.968	1.014	Q99612	KLF6	Krueppel-like factor 6
0.928	0.974	1.019	1.125	0.995	1.223	1.101	1.019	1.049	1.095	1.037	1.073	O43291	SPINT2	Kunitz-type protease inhibitor 2
1.018	0.918	0.994	0.919	0.896	1.051	1.012	1.172	1.035				Q6PIL6	KCNIP4	Kv channel-interacting protein 4
0.871	1.196	1.046	1.004	0.936	1.025	1.145	1.033	1.047				M0QY77	KXD1	KxDL motif-containing protein 1
1.075	1.093	1.325	1.088	1.123	1.181	1.090	1.238	1.245	0.990	1.015	1.355	Q16719	KYNU	Kynureninase
1.021	0.996	1.009	1.008	0.978	0.997	1.009	1.057	0.987	1.030	1.015	1.006	Q6YP21	KYAT3	Kynurenine--oxoglutarate transaminase 3
1.028	1.002	1.024	0.989	0.962	0.987	0.975	1.039	0.974	0.939	0.994	0.973	Q9H9P8	L2HGDH	L-2-hydroxyglutarate dehydrogenase, mitochondrial
1.031	1.050	1.080	1.116	1.159	1.068	1.023	1.109	1.135	1.115	1.033	1.148	Q08431	MFGE8	Lactadherin
1.052	0.887	1.025	0.942	1.029	1.079	1.057	1.152	1.019	1.004	0.949	1.092	Q8WV93	LACE1	Lactation elevated protein 1
1.125	0.864	1.117	0.892	0.714	1.063							Q9NPC4	A4GALT	Lactosylceramide 4-alpha-galactosyltransferase
						0.874	0.543	0.544				P02788	LTF	Lactotransferrin
1.027	1.010	0.960	1.009	1.072	0.997	1.013	1.055	1.017	0.984	1.013	0.974	Q04760	GLO1	Lactoylglutathione lyase
1.031	0.945	0.983	0.964	0.926	1.005	0.992	1.077	1.028	0.998	0.991	1.038	Q9Y2S2	CRYL1	Lambda-crystallin homolog
0.991	0.999	0.996	0.984	0.934	0.949	0.997	1.023	0.968	0.999	1.040	0.985	P42166	TMPO	Lamina-associated polypeptide 2, isoform alpha

Abundance Ratio														
1 d M1/C1	1 d M2/C2	1 d M3/C3	4 d M1/C1	4 d M2/C2	4 d M3/C3	7 d M1/C1	7 d M2/C2	7 d M3/C3	10 d M1/C1	10 d M2/C2	10 d M3/C3	Accession ID	Gene name	Protein Description
0.841	1.111	1.110	0.916	0.996	1.037	0.894	0.920	0.946	1.068	1.371	1.286	H0YJH7	TMPO	Lamina-associated polypeptide 2, isoforms beta/gamma (Fragment)
0.961	0.996	1.000	0.971	0.946	0.992	1.008	1.034	0.995	1.017	1.037	0.986	P42167	TMPO	Lamina-associated polypeptide 2, isoforms beta/gamma
0.946	0.989	0.994	0.992	0.955	0.999	0.995	1.022	0.969	1.097	1.072	1.024	Q14739	LBR	Lamin-B receptor
0.992	0.991	0.995	0.980	0.979	1.001	0.972	0.978	0.971	0.977	0.991	0.975	P20700	LMNB1	Lamin-B1
1.002	0.995	1.001	0.995	0.970	0.993	0.988	0.962	0.987	0.991	0.998	0.989	Q03252	LMNB2	Lamin-B2
1.072	1.045	1.030				1.058	1.169	1.083	0.900	1.004	1.060	P25391	LAMA1	Laminin subunit alpha-1
1.032	1.041	1.037	1.046	1.070	1.079	1.079	1.043	1.049	1.044	1.051	1.035	O15230	LAMA5	Laminin subunit alpha-5
									1.207	1.096	1.125	E7EPA6	LAMB1	Laminin subunit beta-1
1.009	1.010	1.040	1.046	1.045	1.057	1.078	1.051	1.056	1.070	1.059	1.065	G3XAI2	LAMB1	Laminin subunit beta-1
1.004	1.002	1.038	1.023	0.985	1.042	0.953	1.071	0.992	1.102	0.999	1.091	P55268	LAMB2	Laminin subunit beta-2
			1.128	0.833	1.033	1.032	0.981	0.992	0.986	0.886	1.122	Q13751	LAMB3	Laminin subunit beta-3
0.995	1.013	1.062	1.037	1.052	1.075	1.091	1.027	1.064	1.123	1.058	1.153	P11047	LAMC1	Laminin subunit gamma-1
1.272	1.118	1.101	1.037	0.924	0.938	1.090	0.860	0.920	0.943	0.785	1.087	Q13753	LAMC2	Laminin subunit gamma-2
1.015	0.989	1.007	1.007	1.019	1.018	0.995	1.041	1.031	0.985	0.971	0.990	Q9NRN7	AASDHPPT	L-aminoadipate-semialdehyde dehydrogenase-phosphopantetheinyl transferase
1.029	0.978	0.974	1.028	1.094	0.989	1.015	0.986	1.012	1.012	0.997	0.942	O43813	LANCL1	LanC-like protein 1
0.967	0.985	0.985	0.991	0.951	1.082	1.042	1.062	1.004	1.043	1.007	1.077	Q9NS86	LANCL2	LanC-like protein 2
1.004	1.041	1.071	1.093	1.020	1.090	1.101	1.123	1.056	1.073	1.019	1.069	A0A0C4DFL7	CYP51A1	Lanosterol 14-alpha demethylase
0.981	1.011	1.021	1.019	0.984	1.002	1.045	1.062	0.988	1.092	1.025	1.033	P48449	LSS	Lanosterol synthase
1.025	1.015	1.013	1.026	0.997	1.019	1.008	1.053	1.005	1.015	1.039	0.999	Q6PKG0	LARP1	La-related protein 1
0.977	0.969	1.066	1.076	0.850	1.066	1.038	0.961	1.018	1.102	0.989	1.084	Q659C4	LARP1B	La-related protein 1B
1.035	0.963	1.020	0.957	1.002	1.054	1.005	1.073	1.034	1.019	1.018	1.058	Q92615	LARP4B	La-related protein 4B
1.035	1.065	1.034	1.058	0.945	1.014	0.957	1.101	1.034	0.844	0.855	0.964	Q9BRS8	LARP6	La-related protein 6
0.929	0.964	0.950	1.017	0.901	1.087	1.112	1.065	1.038	1.127	1.119	1.092	Q01650	SLC7A5	Large neutral amino acids transporter small subunit 1
0.967	0.981	0.996	0.964	0.989	1.049	1.006	0.994	1.018	0.973	1.016	1.042	Q9H089	LSG1	Large subunit GTPase 1 homolog
1.021	1.019	0.974	1.038	0.995	1.049	1.031	1.089	0.999	1.020	0.992	1.015	Q9UK59	DBR1	Lariat debranching enzyme
1.073	1.024	1.050	1.001	0.974	1.017	0.960	1.079	1.032	1.038	0.975	0.997	Q8NBF6	AVL9	Late secretory pathway protein AVL9 homolog
1.032	1.044	1.010	0.973	0.929	0.961	0.980	1.017	0.986	1.114	0.986	1.047	Q8N2S1	LTBP4	Latent-transforming growth factor beta-binding protein 4
0.963	1.038	1.036	1.017	0.950	1.001	1.027	1.113	0.953	1.024	1.081	1.083	O75845	SC5D	Lathosterol oxidase
0.994	0.985	0.980	1.019	0.991	1.018	1.018	0.985	0.987	1.046	1.020	1.005	Q14696	MESDC2	LDLR chaperone MESD
0.929	0.973	0.959	1.114	1.077	1.114	1.301	1.282	1.319	1.521	1.505	1.472	Q99538	LGMN	Legumain
0.982	0.995	1.041	0.963	0.939	1.042	0.954	0.885	0.975	1.026	1.009	1.043	Q8NC56	LEMD2	LEM domain-containing protein 2

Abundance Ratio														
1 d M1/C1	1 d M2/C2	1 d M3/C3	4 d M1/C1	4 d M2/C2	4 d M3/C3	7 d M1/C1	7 d M2/C2	7 d M3/C3	10 d M1/C1	10 d M2/C2	10 d M3/C3	Accession ID	Gene name	Protein Description
1.011	0.987	1.015	1.031	1.032	0.989	1.004	1.037	1.012	1.026	1.026	1.019	Q15334	LLGL1	Lethal(2) giant larvae protein homolog 1
0.985	1.014	1.042	1.005	1.000	0.974	1.024	1.057	1.011	1.080	1.020	1.089	Q6P1M3	LLGL2	Lethal(2) giant larvae protein homolog 2
0.943	0.975	0.982	1.091	0.989	1.098	1.000	1.057	0.984	0.934	0.958	1.074	Q969R5	L3MBTL2	Lethal(3)malignant brain tumor-like protein 2
1.041	0.979	1.061	1.077	0.924	1.016	1.002	1.057	1.076	1.024	1.160	1.047	Q96JM7	L3MBTL3	Lethal(3)malignant brain tumor-like protein 3
0.983	0.981	1.016	0.977	0.901	0.987	1.018	1.000	1.008	0.997	1.003	0.991	O95202	LETM1	LETM1 and EF-hand domain-containing protein 1, mitochondrial
1.014	1.012	1.036	0.970	1.001	1.033	1.007	0.976	1.000	1.065	0.976	1.081	Q86V48	LUZP1	Leucine zipper protein 1
0.898	0.979	1.334	0.900	0.663	1.390	0.818	0.769	0.973				Q9BRK4	LZTS2	Leucine zipper putative tumor suppressor 2
1.113	0.903	1.071	1.100	0.952	1.176	1.056	1.166	1.012				O60299	LZTS3	Leucine zipper putative tumor suppressor 3
1.034	0.979	1.016	0.995	1.000	0.999	0.999	0.975	1.020	0.994	1.015	0.996	Q9NQ48	LZTFL1	Leucine zipper transcription factor-like protein 1
0.981	1.001	1.004	1.014	1.022	1.001	1.016	0.992	1.029	1.017	0.995	0.985	P42704	LRPPRC	Leucine-rich PPR motif-containing protein, mitochondrial
0.982	0.973	1.003	0.999	0.938	1.013	0.986	0.962	1.020	0.980	0.970	1.039	Q96II8	LRCH3	Leucine-rich repeat and calponin homology domain-containing protein 3
									0.989	0.999	0.885	O75427	LRCH4	Leucine-rich repeat and calponin homology domain-containing protein 4
1.034	1.013	1.032	0.970	0.927	1.015	0.951	1.065	0.981	0.914	0.962	0.967	Q9UFC0	LRWD1	Leucine-rich repeat and WD repeat-containing protein 1
0.972	0.962	1.046	1.033	0.972	1.056	1.038	1.061	1.120	1.028	1.075	1.073	Q8WUT4	LRRN4	Leucine-rich repeat neuronal protein 4
1.018	1.019	1.024	0.998	0.951	1.031	1.004	1.078	1.004	1.010	0.994	1.058	Q9UQ13	SHOC2	Leucine-rich repeat protein SHOC-2
0.955	1.016	1.071	1.068	1.119	1.079	1.145	1.176	0.928	0.941	1.046	1.242	Q9BXB1	LGR4	Leucine-rich repeat-containing G-protein coupled receptor 4
			0.935	0.758	1.141	1.028	2.030	1.008	1.196	0.949	1.346	O75473	LGR5	Leucine-rich repeat-containing G-protein coupled receptor 5
1.025	0.981	1.195	0.948	1.015	1.052	0.999	1.039	0.994	0.964	0.967	1.084	Q9BTT6	LRRC1	Leucine-rich repeat-containing protein 1
0.979	1.069	1.013	0.971	0.931	1.028	1.065	1.043	1.010	1.006	1.031	0.969	Q15048	LRRC14	Leucine-rich repeat-containing protein 14
1.108	1.038	0.962	0.997	0.916	0.896	1.021	1.182	1.027	0.985	0.974	0.996	Q8TCA0	LRRC20	Leucine-rich repeat-containing protein 20
1.056	1.078	0.995	0.917	0.951	1.015	0.967	1.125	1.067	1.025	0.961	1.005	Q6UY01	LRRC31	Leucine-rich repeat-containing protein 31
1.048	0.985	1.019	1.004	0.926	1.001	1.006	1.063	1.023	1.010	0.946	1.019	Q9H9A6	LRRC40	Leucine-rich repeat-containing protein 40
1.009	1.061	1.053	0.961	0.978	1.130	1.032	1.055	1.081	1.304	1.104	1.107	Q15345	LRRC41	Leucine-rich repeat-containing protein 41
0.947	1.059	0.986	0.942	0.915	1.047	0.821	1.137	1.104	0.843	0.879	0.962	Q9Y546	LRRC42	Leucine-rich repeat-containing protein 42
1.005	0.998	1.007	1.012	1.004	1.012	1.042	0.994	1.003	1.015	0.986	1.030	Q8N1G4	LRRC47	Leucine-rich repeat-containing protein 47
1.000	0.961	0.981	0.991	0.936	1.058	0.998	1.020	0.969	0.987	1.020	0.995	Q8N9N7	LRRC57	Leucine-rich repeat-containing protein 57
			1.142	0.917	0.936	1.117	0.963	0.985	1.020	0.968	0.935	Q96CX6	LRRC58	Leucine-rich repeat-containing protein 58
1.002	0.995	1.020	0.998	1.047	0.999	1.007	0.985	1.034	0.978	1.034	1.010	Q96AG4	LRRC59	Leucine-rich repeat-containing protein 59
1.260	0.795	0.903										Q96NW7	LRRC7	Leucine-rich repeat-containing protein 7

Abundance Ratio														
1 d M1/C1	1 d M2/C2	1 d M3/C3	4 d M1/C1	4 d M2/C2	4 d M3/C3	7 d M1/C1	7 d M2/C2	7 d M3/C3	10 d M1/C1	10 d M2/C2	10 d M3/C3	Accession ID	Gene name	Protein Description
			0.912	0.911	0.934							Q8N4P6	LRRC71	Leucine-rich repeat-containing protein 71
0.961	0.991	0.990	0.986	1.012	0.994	0.979	0.972	0.992	0.968	0.981	0.997	Q9P2J5	LARS	Leucine--tRNA ligase, cytoplasmic
1.078	1.060	1.077	0.924	0.952	1.020	1.044	1.070	1.118	1.017	1.027	1.045	Q8N653	LZTR1	Leucine-zipper-like transcriptional regulator 1
1.028	1.024	1.017	1.049	1.000	1.057	1.079	1.126	1.078	1.077	1.048	1.054	Q9UIQ6	LNPEP	Leucyl-cystinyl aminopeptidase
						1.262	0.859	1.169	0.938	0.882	1.187	P15018	LIF	Leukemia inhibitory factor
0.999	1.001	0.976	0.989	0.989	0.990	0.999	1.037	0.978	1.009	0.970	0.998	P30740	SERPINB1	Leukocyte elastase inhibitor
1.088	1.019	1.097				1.032	1.004	0.891	1.036	1.049	1.049	Q96BZ8	LENG1	Leukocyte receptor cluster member 1
1.011	0.996	1.074	1.131	0.947	0.978	1.168	1.090	1.126	1.171	1.032	1.071	Q08722	CD47	Leukocyte surface antigen CD47
1.011	0.996	0.999	0.993	1.019	0.974	0.973	0.986	0.994	1.004	1.004	0.973	P09960	LTA4H	Leukotriene A-4 hydrolase
0.845	0.894	0.945	1.036	0.992	0.978	0.953	0.909	0.907	1.006	1.020	0.947	Q9UNZ5	C19orf53	Leydig cell tumor 10 kDa protein homolog
1.078	1.066	1.071	1.022	1.149	1.061	0.996	0.917	0.993	1.110	1.101	1.179	Q5T3J3	LRIF1	Ligand-dependent nuclear receptor-interacting factor 1
1.040	0.942	1.026	0.971	0.967	0.960	1.039	0.917	1.011	0.917	1.000	1.003	Q9NZU5	LMCD1	LIM and cysteine-rich domains protein 1
1.048	1.002	1.009	0.987	1.073	0.974	0.992	0.869	0.985	0.998	0.988	0.995	Q14847	LASP1	LIM and SH3 domain protein 1
1.011	0.980	1.002	0.988	1.027	1.017	1.001	0.952	0.998	1.012	1.002	1.012	Q9UHB6	LIMA1	LIM domain and actin-binding protein 1
1.006	1.130	0.765	1.057	0.990	1.069	1.063	1.220	1.165	0.948	0.838	1.026	E9PC47	LIMK1	LIM domain kinase 1
0.997	0.991	1.008	0.964	0.964	1.026	1.056	0.942	0.999	1.003	0.945	1.065	F8WD26	LMO7	LIM domain only protein 7
1.050	1.004	1.043	1.003	1.001	1.001	1.029	1.009	1.006	0.949	1.011	1.039	Q9UGP4	LIMD1	LIM domain-containing protein 1
1.239	1.254	0.702	1.009	1.053	1.058	1.060	0.969	1.062	0.967	0.841	1.099	Q9BT23	LIMD2	LIM domain-containing protein 2
			0.912	0.927	1.152	0.987	0.887	0.987	1.009	0.994	1.088	Q96IF1	AJUBA	LIM domain-containing protein ajuba
1.031	0.973	0.989	1.046	1.098	0.946	1.111	1.120	0.983				Q9UN81	L1RE1	LINE-1 retrotransposable element ORF1 protein
0.978	1.007	1.035	0.903	0.857	1.213	1.061	1.190	0.941	0.996	1.020	1.367	Q9GZY6	LAT2	Linker for activation of T-cells family member 2
0.716	0.881	1.125	1.076	0.931	1.071	0.958	0.828	0.916	0.681	1.189	1.022	Q96S06	LMF1	Lipase maturation factor 1
0.963	0.990	0.973	0.966	0.911	1.036	1.099	1.085	1.074	1.091	1.008	1.088	Q9BU23	LMF2	Lipase maturation factor 2
1.003	1.018	1.027	1.009	0.974	1.030	1.029	1.068	0.991	1.012	0.998	1.092	B5MDU6	LDAH	Lipid droplet-associated hydrolase
1.026	1.005	0.998	1.019	0.986	1.009	1.005	1.035	1.006	1.014	0.998	0.978	P11182	DBT	Lipoamide acyltransferase component of branched-chain alpha-keto acid dehydrogenase complex, mitochondrial
1.021	1.056	1.014	1.087	0.972	1.107	1.141	1.000	1.055	1.172	1.052	1.120	Q86X29	LSR	Lipolysis-stimulated lipoprotein receptor
1.061	0.984	0.991	0.968	0.970	0.991	0.967	1.059	0.930	0.944	0.942	0.913	C9J2R5	LPP	Lipoma-preferred partner (Fragment)
1.023	1.032	1.014	0.993	1.179	0.976	1.047	0.862	1.001	1.019	1.042	1.034	Q93052	LPP	Lipoma-preferred partner
1.093	0.903	1.117	0.967	0.827	0.696	0.654	0.889	0.814	0.466	1.105	0.564	P18428	LBP	Lipopolysaccharide-binding protein
0.903	1.056	1.010	0.994	0.912	1.093	1.136	1.033	1.058	1.046	0.954	0.995	Q99732	LITAF	Lipopolysaccharide-induced tumor necrosis factor-alpha factor
0.998	1.103	0.962				1.003	1.091	0.991				F5GZB4	LOXHD1	Lipoxygenase homology domain-containing protein 1

Abundance Ratio														
1 d M1/C1	1 d M2/C2	1 d M3/C3	4 d M1/C1	4 d M2/C2	4 d M3/C3	7 d M1/C1	7 d M2/C2	7 d M3/C3	10 d M1/C1	10 d M2/C2	10 d M3/C3	Accession ID	Gene name	Protein Description
1.251	0.889	1.122	1.021	0.893	1.081	0.974	1.115	1.186	1.051	1.008	1.011	O43766	LIAS	Lipoyl synthase, mitochondrial
1.095	0.956	0.960	0.923	0.808	0.995				1.281	0.956	0.744	Q9Y234	LIPT1	Lipoyltransferase 1, mitochondrial
0.986	0.969	1.016	0.968	0.989	1.034	1.013	0.955	1.002	1.022	1.006	1.058	Q13136	PPFIA1	Liprin-alpha-1
1.099	1.187	1.123	1.135	0.752	1.161							O75145	PPFIA3	Liprin-alpha-3
									0.915	1.066	0.968	B1APN9	PPFIA4	Liprin-alpha-4
0.972	0.967	1.037	0.963	0.919	0.966	0.981	0.931	0.984	1.005	0.977	1.051	Q86W92	PPFIBP1	Liprin-beta-1
						0.866	0.958	0.831	0.832	0.904	1.116	Q8ND30	PPFIBP2	Liprin-beta-2
									0.976	0.977	1.002	A0A075B768	KIAA1468	LisH domain and HEAT repeat-containing protein KIAA1468 (Fragment)
0.991	0.949	0.992	0.947	1.099	1.030	1.112	1.030	1.037	1.012	0.988	1.107	A0A087X1I8	ARMC9	LisH domain-containing protein ARMC9
1.002	0.981	0.954	1.010	0.933	0.946	0.889	0.833	1.004	0.974	1.007	1.049	Q9Y2F5	ICE1	Little elongation complex subunit 1
1.060	1.033	0.989	1.034	1.036	0.911	0.858	1.329	1.157	0.891	0.945	1.089	Q659A1	ICE2	Little elongation complex subunit 2
1.016	1.052	1.208	0.876	0.956	1.037	0.945	0.910	1.020	0.936	0.911	1.216	Q8IVB5	LIX1L	LIX1-like protein
0.982	0.993	0.981	1.011	0.989	1.007	1.011	1.049	0.996	1.007	1.009	0.982	P07195	LDHB	L-lactate dehydrogenase B chain
			0.887	1.257	0.730							P07864	LDHC	L-lactate dehydrogenase C chain
1.155	1.076	0.993	1.101	1.053	1.020	1.070	0.951	1.078	1.141	1.060	1.017	Q68DH5	LMBRD2	LMBR1 domain-containing protein 2
1.075	1.063	0.988	1.008	0.962	1.019	0.987	1.044	1.057	0.941	0.984	1.072	Q86WA8	LONP2	Lon protease homolog 2, peroxisomal
0.985	1.013	1.003	0.998	0.993	1.020	1.008	1.042	0.997	0.987	0.980	1.021	P36776	LONP1	Lon protease homolog, mitochondrial
1.019	0.986	0.978	0.999	1.015	0.995	1.019	1.074	1.018	1.033	1.005	0.971	Q6PCB7	SLC27A1	Long-chain fatty acid transport protein 1
0.975	0.991	1.019	0.996	0.927	1.002	1.019	1.041	1.012	1.035	1.056	1.020	Q6P1M0	SLC27A4	Long-chain fatty acid transport protein 4
1.029	1.012	1.008	1.038	1.036	1.029	1.021	1.041	1.056	1.041	1.017	1.065	P33121	ACSL1	Long-chain-fatty-acid--CoA ligase 1
0.974	1.025	0.979	0.988	0.958	1.006	1.004	1.015	1.007	0.977	1.041	1.029	O95573	ACSL3	Long-chain-fatty-acid--CoA ligase 3
1.054	1.044	1.022	1.069	1.087	1.008	1.056	1.040	1.022	1.014	1.036	1.028	O60488	ACSL4	Long-chain-fatty-acid--CoA ligase 4
0.988	0.992	1.025	0.981	0.996	1.072	0.944	0.890	0.959	1.008	0.944	1.067	Q5SW96	LDLRAP1	Low density lipoprotein receptor adapter protein 1
1.045	0.992	1.000	1.018	0.938	0.994	0.968	1.055	0.984	0.956	1.001	0.977	P24666	ACP1	Low molecular weight phosphotyrosine protein phosphatase
0.958	1.077	1.000	1.068	0.967	1.084	1.041	1.211	1.102	1.063	1.110	1.129	J3KMZ9	LDLR	Low-density lipoprotein receptor (Fragment)
1.009	1.134	1.124	1.034	0.882	1.294	1.147	1.197	1.510	1.177	0.967	1.134	Q7Z4F1	LRP10	Low-density lipoprotein receptor-related protein 10
						1.236	1.333	1.141	1.338	1.134	1.254	Q14114	LRP8	Low-density lipoprotein receptor-related protein 8
1.015	0.997	1.001	0.980	0.975	1.028	0.974	1.026	1.022	0.993	1.024	0.981	P05455	SSB	Lupus La protein
1.007	1.044	1.016	0.994	1.008	1.026	1.030	1.068	1.009	1.004	0.976	1.051	Q7Z4W1	DCXR	L-xylulose reductase
0.886	0.924	1.222				1.253	1.064	1.116	1.057	1.147	1.114	Q17RY6	LY6K	Lymphocyte antigen 6K
0.981	0.870	1.088	0.980	0.908	0.929	1.065	1.139	0.948	1.167	1.158	1.107	P19256	CD58	Lymphocyte function-associated antigen 3

Abundance Ratio														
1 d M1/C1	1 d M2/C2	1 d M3/C3	4 d M1/C1	4 d M2/C2	4 d M3/C3	7 d M1/C1	7 d M2/C2	7 d M3/C3	10 d M1/C1	10 d M2/C2	10 d M3/C3	Accession ID	Gene name	Protein Description
0.804	0.993	1.075	0.992	0.928	1.020	1.138	0.990	1.019	1.139	0.957	1.153	O43325	LYRM1	LYR motif-containing protein 1
0.931	1.010	0.960	1.027	1.034	0.965	1.013	0.961	0.876	1.026	1.011	1.026	Q9NU23	LYRM2	LYR motif-containing protein 2
0.976	0.971	1.004	0.977	0.941	0.993	1.017	1.030	0.977	1.055	1.010	0.992	Q9HD34	LYRM4	LYR motif-containing protein 4
0.921	0.963	0.958	1.006	1.002	1.017	0.969	0.968	0.993	1.018	0.947	1.036	P46736	BRCC3	Lys-63-specific deubiquitinase BRCC36
0.972	0.981	1.038	1.027	0.986	1.066	1.002	0.995	0.907	1.050	1.043	1.038	Q1ED39	KNOP1	Lysine-rich nucleolar protein 1
1.010	1.025	1.023	0.991	0.895	0.914	1.026	1.081	0.987	0.969	0.964	1.052	Q9Y2K7	KDM2A	Lysine-specific demethylase 2A
									0.809	0.673	0.987	Q8NHM5	KDM2B	Lysine-specific demethylase 2B
0.957	0.916	1.036	0.957	0.918	1.000	0.995	1.021	1.015	0.981	0.960	0.994	F5H070	KDM3A	Lysine-specific demethylase 3A
0.992	0.982	0.983	0.965	0.916	0.991	1.027	1.080	1.007	0.987	0.995	1.030	Q7LBC6	KDM3B	Lysine-specific demethylase 3B
0.954	1.038	1.050							1.008	0.923	1.066	O75164	KDM4A	Lysine-specific demethylase 4A
			0.976	0.779	1.125							F5GX28	KDM4B	Lysine-specific demethylase 4B
0.970	1.003	1.019	0.972	0.937	1.049	0.846	0.774	1.078	0.949	1.175	1.140	P29375	KDM5A	Lysine-specific demethylase 5A
0.945	0.935	1.014	1.019	0.897	1.021	0.962	1.081	1.023	0.954	0.890	1.093	P41229	KDM5C	Lysine-specific demethylase 5C
0.983	0.990	1.012	0.955	0.939	1.043	1.036	0.951	1.040	0.992	1.019	1.095	O75151	PHF2	Lysine-specific demethylase PHF2
1.005	1.013	0.985	1.015	0.973	0.991	1.032	1.084	0.983	0.987	0.988	1.013	O60341	KDM1A	Lysine-specific histone demethylase 1A
1.114	1.017	1.037	1.069	1.011	0.928	0.947	1.256	0.922	0.945	1.087	1.000	Q8NB78	KDM1B	Lysine-specific histone demethylase 1B
0.959	1.009	0.993	0.999	1.002	1.013	0.998	1.003	0.994	0.983	1.026	0.986	Q15046	KARS	Lysine--tRNA ligase
1.073	0.940	1.188	1.082	1.139	1.040	0.994	0.767	1.069	1.116	0.972	1.057	Q8IV50	LYSMD2	LysM and putative peptidoglycan-binding domain-containing protein 2
0.880	0.924	0.893	1.080	1.063	0.858	1.078	1.237	1.033	1.021	1.047	0.934	Q7Z3D4	LYSMD3	LysM and putative peptidoglycan-binding domain-containing protein 3
0.978	0.980	1.041	1.003	0.961	1.034	1.024	0.994	1.000	1.062	1.009	1.054	Q6UWP7	LCLAT1	Lysocardiolipin acyltransferase 1
1.106	1.016	1.022	1.135	1.111	1.177	1.045	1.198	1.024	1.026	1.095	1.090	Q9NPH0	ACP6	Lysophosphatidic acid phosphatase type 6
1.032	0.991	1.034	0.955	0.900	1.012	0.960	0.984	0.980	0.997	1.011	1.059	Q8NF37	LPCAT1	Lysophosphatidylcholine acyltransferase 1
0.960	0.961	1.029	0.940	0.911	1.025	1.000	1.003	1.005	1.031	0.977	1.036	Q7L5N7	LPCAT2	Lysophosphatidylcholine acyltransferase 2
1.062	1.013	0.999	1.003	0.976	0.999	0.997	1.105	0.985	1.022	0.966	0.997	Q5VWZ2	LYPLAL1	Lysophospholipase-like protein 1
1.046	1.044	1.017	1.120	0.961	0.926				1.111	0.998	1.126	Q6ZNC8	MBOAT1	Lysophospholipid acyltransferase 1
			1.131	0.885	1.140	1.018	0.819	1.119				Q6ZWT7	MBOAT2	Lysophospholipid acyltransferase 2
0.941	0.979	0.994	0.949	0.835	1.050	1.035	1.189	1.033	1.129	1.025	1.110	Q6P1A2	LPCAT3	Lysophospholipid acyltransferase 5
0.912	0.986	0.984	0.953	0.861	1.002	1.023	1.012	0.982	1.142	1.072	1.058	Q96N66	MBOAT7	Lysophospholipid acyltransferase 7
0.972	0.961	1.003	0.955	0.894	1.036	0.976	1.060	1.027	0.964	0.996	1.001	Q643R3	LPCAT4	Lysophospholipid acyltransferase LPCAT4
1.118	0.925	1.041	1.343	1.058	1.112	1.566	1.365	1.452	1.657	1.301	1.477	P38571	LIPA	Lysosomal acid lipase/cholesteryl ester hydrolase
1.047	1.020	1.039	1.003	0.952	1.025	1.035	0.996	1.031	1.060	1.021	1.018	P11117	ACP2	Lysosomal acid phosphatase
1.068	1.013	1.035	1.008	0.952	0.951	0.987	1.073	0.987	0.961	0.962	0.991	P10253	GAA	Lysosomal alpha-glucosidase

Abundance Ratio														
1 d M1/C1	1 d M2/C2	1 d M3/C3	4 d M1/C1	4 d M2/C2	4 d M3/C3	7 d M1/C1	7 d M2/C2	7 d M3/C3	10 d M1/C1	10 d M2/C2	10 d M3/C3	Accession ID	Gene name	Protein Description
1.108	0.977	1.031	1.068	0.955	1.028	1.059	0.949	1.020	1.103	1.055	1.044	O00754	MAN2B1	Lysosomal alpha-mannosidase
1.001	0.988	1.011	1.053	1.066	1.053	1.096	1.067	1.085	1.145	1.105	1.098	P42785	PRCP	Lysosomal Pro-X carboxypeptidase
0.927	1.020	0.938	1.062	1.029	1.054	1.073	1.231	1.064	1.085	1.077	1.002	A0A140T8X7	PPT2	Lysosomal thioesterase PPT2
									1.497	1.490	1.527	Q15012	LAPTM4A	Lysosomal-associated transmembrane protein 4A
1.071	1.060	0.921	0.976	0.979	1.043	1.029	1.125	0.988	1.022	1.102	1.023	Q99698	LYST	Lysosomal-trafficking regulator
0.948	0.978	0.990	0.975	0.887	1.017	1.018	1.001	1.017	1.077	1.054	0.998	Q14108	SCARB2	Lysosome membrane protein 2
1.024	1.022	0.990	1.043	0.975	0.979	0.947	1.051	1.092	0.980	1.048	0.886	P11279	LAMP1	Lysosome-associated membrane glycoprotein 1
1.034	1.009	1.048	1.008	0.995	1.106	1.071	1.086	1.103	1.134	1.073	1.061	P13473	LAMP2	Lysosome-associated membrane glycoprotein 2
0.844	0.834	0.679	1.097	1.262	0.917	0.691	0.760	1.196	4.121	1.119	0.718	P61626	LYZ	Lysozyme C
0.944	1.075	1.020	1.020	0.951	1.010	1.220	0.938	1.105	0.908	1.005	1.119	Q9Y4K0	LOXL2	Lysyl oxidase homolog 2
0.911	0.971	1.048	0.945	1.024	1.010	0.998	1.061	1.053	1.033	1.061	0.897	Q8IU60	DCP2	m7GpppN-mRNA hydrolase
1.009	0.996	0.973	0.989	0.992	1.025	0.988	1.028	0.994	0.984	1.043	1.001	Q96C86	DCPS	m7GpppX diphosphatase
1.017	1.007	1.029	1.022	1.091	1.098	1.045	1.072	1.081	1.057	0.994	1.091	Q8WWC4	MAIP1	m-AAA protease-interacting protein 1, mitochondrial
0.852	1.147	1.009	1.029	0.817	0.791	1.003	0.918	1.093				Q8N5G2	TMEM57	Macoilin
1.021	0.953	1.198										P09603	CSF1	Macrophage colony-stimulating factor 1
1.003	0.939	1.060	1.015	0.881	0.983	1.061	1.062	1.055	1.072	1.015	1.023	E7ESC7	MAEA	Macrophage erythroblast attacher
1.094	0.998	1.079	1.009	1.263	0.976	0.926	0.777	1.081	0.925	0.994	0.993	P14174	MIF	Macrophage migration inhibitory factor
1.003	0.988	0.960	0.988	0.959	0.956	0.981	0.980	0.916	0.966	0.924	0.940	P40121	CAPG	Macrophage-capping protein
0.839	1.045	1.013	0.947	0.971	1.080	1.123	0.841	1.065	0.979	0.981	1.132	Q04912	MST1R	Macrophage-stimulating protein receptor
1.039	1.050	1.039	0.991	0.978	1.036	0.992	1.029	0.987	0.984	0.897	1.011	Q8NDA8	MROH1	Maestro heat-like repeat-containing protein family member 1
0.973	0.969	1.014	0.986	0.842	1.045	1.125	1.099	1.048	1.164	1.087	1.098	Q9HD23	MRS2	Magnesium transporter MRS2 homolog, mitochondrial
						0.945	1.113	0.812				Q7RTP0	NIPA1	Magnesium transporter NIPA1
1.012	0.983	1.018	0.952	0.938	1.015	1.025	0.880	1.025	1.053	0.990	0.988	A0A087WU53	MAGT1	Magnesium transporter protein 1
1.018	0.982	1.059	0.994	0.925	1.016	1.043	1.065	0.994	1.010	1.018	1.021	Q86V88	MDP1	Magnesium-dependent phosphatase 1
1.000	1.022	0.996	0.979	0.969	1.029	0.991	1.041	1.028	0.958	0.942	0.972	Q8N3R9	MPP5	MAGUK p55 subfamily member 5
1.016	0.944	1.032	1.055	1.020	1.050	1.068	1.082	0.984	0.995	1.027	1.049	Q9NZW5	MPP6	MAGUK p55 subfamily member 6
1.020	0.938	0.998	1.021	0.981	1.053	0.992	0.970	0.938	0.951	0.892	1.030	P07199	CENPB	Major centromere autoantigen B
0.875	0.991	0.973	0.933	0.859	1.036	1.048	1.040	0.961	1.184	1.037	1.129	Q14728	MFSD10	Major facilitator superfamily domain-containing protein 10
			0.850	0.629	0.887	1.061	1.054	1.022	1.156	0.903	0.986	Q96ES6	MFSD3	Major facilitator superfamily domain-containing protein 3
			1.055	0.791	1.066							Q6ZSS7	MFSD6	Major facilitator superfamily domain-containing protein 6

Abundance Ratio														
1 d M1/C1	1 d M2/C2	1 d M3/C3	4 d M1/C1	4 d M2/C2	4 d M3/C3	7 d M1/C1	7 d M2/C2	7 d M3/C3	10 d M1/C1	10 d M2/C2	10 d M3/C3	Accession ID	Gene name	Protein Description
1.204	0.946	0.874	1.043	0.915	1.001	1.141	1.041	1.054	1.075	1.037	1.043	Q8NHS3	MFSD8	Major facilitator superfamily domain-containing protein 8
0.893	0.985	0.959	1.140	0.981	1.146	1.214	0.964	1.088	1.189	0.996	1.309	P04156	PRNP	Major prion protein
1.009	0.998	1.013	1.006	1.003	1.011	1.014	1.023	1.009	1.032	1.000	1.022	Q14764	MVP	Major vault protein
1.030	1.008	0.988	1.011	1.107	0.998	1.007	0.975	1.018	1.004	1.005	0.964	P40926	MDH2	Malate dehydrogenase, mitochondrial
0.962	0.973	0.996	0.996	0.924	0.990	0.988	1.043	0.977	1.051	1.015	1.021	Q14165	MLEC	Malectin
0.986	1.001	0.951	1.022	1.057	0.969	0.948	1.166	1.027	1.189	0.998	1.148	Q16626	MEA1	Male-enhanced antigen 1
0.946	1.041	0.926	0.976	0.867	0.940	0.981	0.991	1.030	1.026	1.079	1.132	Q68DK7	MSL1	Male-specific lethal 1 homolog
1.138	0.960	1.031	0.901	1.019	1.026	0.959	1.021	1.021	1.066	0.780	1.274	Q8N5Y2	MSL3	Male-specific lethal 3 homolog
1.011	0.946	0.980	0.984	0.957	1.002	1.017	1.072	1.029	1.021	0.996	0.993	G3V4T6	GSTZ1	Maleylacetoacetate isomerase
1.070	1.150	0.972	1.012	0.912	1.060	0.968	1.061	1.050	1.024	0.943	1.036	O95822	MLYCD	Malonyl-CoA decarboxylase, mitochondrial
1.013	1.034	1.012	1.015	0.998	1.010	1.003	1.070	1.043	0.985	0.958	1.050	Q8IVS2	MCAT	Malonyl-CoA-acyl carrier protein transacylase, mitochondrial
0.986	0.986	1.006	0.992	0.909	1.003	1.028	1.070	1.036	1.070	1.011	1.066	Q9HD20	ATP13A1	Manganese-transporting ATPase 13A1
1.054	1.008	0.987	0.996	0.980	1.013	0.978	1.031	1.028	0.987	1.007	0.963	P34949	MPI	Mannose-6-phosphate isomerase
1.004	1.002	0.958	0.946	0.869	1.016	1.037	1.124	1.053	1.041	1.001	1.063	O75352	MPDU1	Mannose-P-dolichol utilization defect 1 protein
0.985	1.117	1.053				1.125	0.820	1.132				P33908	MAN1A1	Mannosyl-oligosaccharide 1,2-alpha-mannosidase IA
1.014	0.971	1.088	1.008	0.883	0.966	0.931	0.996	0.990	0.903	0.883	0.919	O60476	MAN1A2	Mannosyl-oligosaccharide 1,2-alpha-mannosidase IB
0.979	1.010	1.042	0.984	0.992	1.021	1.003	1.072	1.003	1.017	0.986	0.998	Q13724	MOGS	Mannosyl-oligosaccharide glucosidase
1.014	1.050	0.960	0.980	0.980	1.057	0.987	0.848	0.993	1.046	1.064	1.107	A0A087WZ62	3 SV	Mannosyltransferase
1.001	1.008	0.981	1.025	1.021	0.996	1.044	1.050	1.026	1.089	1.031	1.091	P49137	MAPKAPK2	MAP kinase-activated protein kinase 2
0.976	1.002	0.973	0.992	0.980	1.003	1.039	1.007	0.954	1.091	0.982	1.061	Q16644	MAPKAPK3	MAP kinase-activated protein kinase 3
1.069	0.989	1.014	0.948	0.944	0.923	0.921	1.016	1.073	1.030	0.947	1.131	Q9BUB5	MKNK1	MAP kinase-interacting serine/threonine-protein kinase 1
1.352	0.782	1.268										Q9HBH9	MKNK2	MAP kinase-interacting serine/threonine-protein kinase 2
1.146	0.922	1.147	0.832	0.877	1.222	0.898	0.928	0.965	1.020	0.966	1.114	Q9NS73	MBIP	MAP3K12-binding inhibitory protein 1
1.001	0.991	1.067	0.985	0.958	1.010	0.972	0.928	1.032	1.032	0.963	1.045	Q3KQU3	MAP7D1	MAP7 domain-containing protein 1
0.861	0.968	1.065	0.969	1.002	0.984	1.024	0.833	0.933	0.999	0.980	1.071	Q8IWC1	MAP7D3	MAP7 domain-containing protein 3
1.004	0.945	0.904	1.021	0.951	1.090	0.896	1.058	1.040	1.012	1.032	1.177	Q9BUT9	MCRIP2	MAPK regulated corepressor interacting protein 2
0.964	0.947	0.994	0.887	0.976	1.024	0.950	0.817	0.925	1.023	1.027	0.954	P49006	MARCKSL1	MARCKS-related protein
1.024	0.999	1.030	1.049	0.951	1.004	1.036	1.051	1.068	1.119	1.052	1.007	Q9NZD8	SPG21	Maspardin
0.964	1.056	1.154										Q14680	MELK	Maternal embryonic leucine zipper kinase
1.134	1.082	1.031	0.929	0.971	0.890				0.879	0.860	0.923	A0A0R4J2E8	MATR3	Matrin-3

Abundance Ratio												Accession ID	Gene name	Protein Description
1 d M1/C1	1 d M2/C2	1 d M3/C3	4 d M1/C1	4 d M2/C2	4 d M3/C3	7 d M1/C1	7 d M2/C2	7 d M3/C3	10 d M1/C1	10 d M2/C2	10 d M3/C3			
0.975	0.989	0.981	0.984	1.005	0.967	0.984	0.905	0.969	1.037	1.027	1.041	A8MXP9	MATR3	Matrin-3
1.019	0.990	1.025	1.011	0.955	1.035	1.033	1.069	0.992	1.069	0.962	1.028	P50281	MMP14	Matrix metalloproteinase-14
						0.992	0.746	1.011	1.095	1.334	1.292	Q9Y5R2	MMP24	Matrix metalloproteinase-24
			1.098	0.977	1.055							Q8N3F0	MTURN	Maturin
1.032	0.994	1.021	0.964	1.004	0.986	0.949	1.038	1.107	0.979	0.947	0.968	Q9Y6X3	MAU2	MAU2 chromatid cohesion factor homolog
0.934	1.156	0.901	0.964	0.988	1.015							H3BU53	MGA	MAX gene-associated protein (Fragment)
1.175	1.004	1.064	0.954	1.065	0.973				0.991	0.985	1.074	Q99583	MNT	Max-binding protein MNT
						1.104	1.025	0.962				Q9UH92	MLX	Max-like protein X
1.036	1.135	1.163	1.002	0.957	1.074	0.844	1.400	1.081	1.034	1.023	1.079	Q05BQ5	MBTD1	MBT domain-containing protein 1
0.996	0.813	1.097	0.893	0.921	0.997							Q96DY7	MTBP	Mdm2-binding protein
0.978	0.967	1.030	0.996	0.974	1.030	1.001	1.016	1.008	1.000	1.026	1.038	Q14676	MDC1	Mediator of DNA damage checkpoint protein 1
1.006	1.026	1.031	0.961	0.977	0.998	0.976	0.979	0.988	0.985	0.985	1.011	Q15648	MED1	Mediator of RNA polymerase II transcription subunit 1
1.058	0.942	1.006	0.993	0.871	0.999	1.033	1.073	1.054	1.039	0.982	1.061	Q9BTT4	MED10	Mediator of RNA polymerase II transcription subunit 10
0.763	0.947	1.020	0.920	1.073	1.136	0.911	0.923	1.086	1.145	0.941	1.167	Q9P086	MED11	Mediator of RNA polymerase II transcription subunit 11
1.127	1.107	1.017	0.998	0.913	0.970	0.946	1.118	1.077	1.042	1.082	1.069	Q9UHV7	MED13	Mediator of RNA polymerase II transcription subunit 13
0.803	0.976	0.986	1.025	0.961	1.056	0.989	1.371	1.007	0.911	1.244	0.881	Q71F56	MED13L	Mediator of RNA polymerase II transcription subunit 13-like
0.995	0.996	1.055	1.053	0.894	0.997	1.054	1.041	1.051	0.976	0.986	1.015	O60244	MED14	Mediator of RNA polymerase II transcription subunit 14
1.038	0.951	1.041	0.998	0.954	1.024	0.980	1.022	0.992	0.946	0.980	1.022	Q96RN5	MED15	Mediator of RNA polymerase II transcription subunit 15
0.988	1.027	1.018	0.977	0.975	1.032	0.945	1.000	0.983	0.984	1.017	1.008	Q9NVC6	MED17	Mediator of RNA polymerase II transcription subunit 17
1.049	0.937	0.980	0.970	1.011	0.997	0.967	1.026	0.983	0.996	1.048	1.058	Q9BUE0	MED18	Mediator of RNA polymerase II transcription subunit 18
0.703	1.114	1.243	1.114	1.177	1.004	0.933	1.128	1.026	0.737	1.061	0.964	A0JLT2	MED19	Mediator of RNA polymerase II transcription subunit 19
0.998	1.010	1.002	1.010	1.001	1.016	0.994	1.097	1.020	1.011	1.039	0.989	Q9H944	MED20	Mediator of RNA polymerase II transcription subunit 20
1.082	1.041	0.965	0.992	1.069	1.011	0.929	1.043	1.241	0.916	0.809	1.037	F5H872	MED21	Mediator of RNA polymerase II transcription subunit 21
0.968	0.908	0.983	0.924	0.995	0.981	1.115	1.056	1.162	1.159	1.014	1.089	Q15528	MED22	Mediator of RNA polymerase II transcription subunit 22

Abundance Ratio														
1 d M1/C1	1 d M2/C2	1 d M3/C3	4 d M1/C1	4 d M2/C2	4 d M3/C3	7 d M1/C1	7 d M2/C2	7 d M3/C3	10 d M1/C1	10 d M2/C2	10 d M3/C3	Accession ID	Gene name	Protein Description
0.978	0.987	1.034	0.963	0.897	1.090	0.968	1.028	1.065	0.987	0.995	1.039	Q5JWT2	MED23	Mediator of RNA polymerase II transcription subunit 23
1.002	0.996	1.024	1.006	0.945	1.028	0.959	1.131	1.050	0.982	0.989	1.003	A0A0B4J1W0	MED24	Mediator of RNA polymerase II transcription subunit 24
1.034	1.059	1.028	1.028	0.951	1.003	1.051	1.042	0.970	0.960	1.052	1.034	Q6P2C8	MED27	Mediator of RNA polymerase II transcription subunit 27
1.105	1.038	1.001	1.043	0.971	0.923	1.005	1.158	0.926	1.006	1.002	1.126	Q9H204	MED28	Mediator of RNA polymerase II transcription subunit 28
1.021	1.033	1.063	0.985	0.985	1.084	1.026	0.943	1.079	0.991	1.037	1.003	Q96HR3	MED30	Mediator of RNA polymerase II transcription subunit 30
0.905	0.937	1.013	0.966	0.879	1.058	0.957	1.090	1.073	1.035	0.973	0.980	Q9Y3C7	MED31	Mediator of RNA polymerase II transcription subunit 31
1.018	0.999	0.991	0.921	0.986	1.037	1.015	1.032	0.996	0.990	0.999	1.022	Q9NPJ6	MED4	Mediator of RNA polymerase II transcription subunit 4
1.016	0.997	0.981	0.971	1.046	1.074	1.070	0.986	0.999	1.058	1.031	1.034	A0A087WYL7	MED6	Mediator of RNA polymerase II transcription subunit 6
			1.087	1.014	0.733							O43513	MED7	Mediator of RNA polymerase II transcription subunit 7
0.729	0.881	0.958				0.833	0.971	1.377				Q9NWA0	MED9	Mediator of RNA polymerase II transcription subunit 9
1.088	0.952	1.071	0.945	0.900	1.024	0.971	0.828	1.011	1.167	1.019	2.024	Q9Y4F3	KIAA0430	Meiosis arrest female protein 1
									0.874	0.446	0.752	A0A087WXM9	MEIKIN	Meiosis-specific kinetochore protein
1.012	1.041	1.043	1.112	1.043	1.020							Q9BWT6	MND1	Meiotic nuclear division protein 1 homolog
1.067	0.990	1.061	1.023	0.931	1.092	0.989	1.048	0.987	1.027	0.988	0.953	Q96PC5	MIA2	Melanoma inhibitory activity protein 2
0.985	0.975	1.023	1.000	0.959	1.027	1.012	0.966	1.002	1.052	1.019	1.063	Q5JRA6	MIA3	Melanoma inhibitory activity protein 3
0.955	0.989	0.995	0.943	0.954	0.984	0.942	0.940	0.974	0.982	0.929	1.019	Q9UNF1	MAGED2	Melanoma-associated antigen D2
0.989	0.956	0.997	1.080	0.945	0.982	1.272	0.988	0.956	1.058	0.948	1.055	P08582	MELTF	Melanotransferrin
0.947	0.973	0.993	0.956	0.939	1.030	1.097	1.064	0.993	1.084	0.961	1.159	Q4ZIN3	TMEM259	Membralin
0.963	0.982	0.989	1.030	1.058	0.847	0.963	1.088	0.958	1.019	1.139	1.028	Q8N4V1	MMGT1	Membrane magnesium transporter 1
			1.017	0.996	1.041							Q96QZ7	MAGI1	Membrane-associated guanylate kinase, WW and PDZ domain-containing protein 1
1.153	1.026	1.027	0.992	0.986	1.061	0.958	1.107	0.974				Q5TCQ9	MAGI3	Membrane-associated guanylate kinase, WW and PDZ domain-containing protein 3
1.054	1.053	1.017	0.999	0.984	0.967	1.033	1.106	0.964	0.967	1.008	1.053	O00562	PITPNM1	Membrane-associated phosphatidylinositol transfer protein 1
0.956	1.010	0.985	0.995	0.959	0.994	0.985	1.000	0.979	1.017	1.025	1.027	O00264	PGRMC1	Membrane-associated progesterone receptor component 1
0.928	1.017	1.098										B4DZM6	PKMYT1	Membrane-associated tyrosine- and threonine-specific cdc2-inhibitory kinase

Abundance Ratio														
1 d M1/C1	1 d M2/C2	1 d M3/C3	4 d M1/C1	4 d M2/C2	4 d M3/C3	7 d M1/C1	7 d M2/C2	7 d M3/C3	10 d M1/C1	10 d M2/C2	10 d M3/C3	Accession ID	Gene name	Protein Description
1.089	1.550	1.002	0.772	0.658	1.105							Q14703	MBTPS1	Membrane-bound transcription factor site-1 protease
									1.076	0.935	1.120	O43462	MBTPS2	Membrane-bound transcription factor site-2 protease
1.041	1.006	1.000	0.987	0.980	1.009	1.039	0.992	0.824	1.072	1.025	1.101	O00255	MEN1	Menin
0.961	0.983	0.994	0.976	0.955	1.006	1.003	0.989	1.014	1.036	0.994	1.054	P35240	NF2	Merlin
1.001	1.025	0.980	1.008	1.020	1.021	1.005	0.953	1.028	0.979	0.987	0.932	A8K878	MANF	Mesencephalic astrocyte-derived neurotrophic factor
0.911	0.992	1.092	0.950	1.152	0.991	1.093	0.807	0.940				Q14872	MTF1	Metal regulatory transcription factor 1
0.872	1.148	1.127										Q9H8M5	CNNM2	Metal transporter CNNM2
1.011	1.015	1.149	1.051	0.980	1.064	1.062	1.011	0.959	0.980	1.065	1.070	Q8NE01	CNNM3	Metal transporter CNNM3
0.949	1.046	0.967	1.048	0.923	1.000	1.039	1.180	1.021	1.032	0.985	1.013	Q6P4Q7	CNNM4	Metal transporter CNNM4
1.014	0.976	1.115	0.990	0.939	1.084	1.012	0.963	1.020	1.078	0.976	1.031	Q68D91	MBLAC2	Metallo-beta-lactamase domain-containing protein 2
1.023	0.965	1.031	0.985	0.887	1.046	0.980	1.039	1.021	0.965	0.969	0.981	Q96E52	OMA1	Metalloendopeptidase OMA1, mitochondrial
1.181	0.959	0.984	0.928	0.947	1.143	1.097	0.877	1.409	0.946	0.966	1.258	Q53F39	MPPE1	Metallophosphoesterase 1
0.977	0.779	1.179	0.826	0.763	1.033	0.968	1.033	0.964	1.035	0.954	1.070	P01033	TIMP1	Metalloproteinase inhibitor 1
									1.066	0.837	1.103	P04731	MT1A	Metallothionein-1A
4.002	0.750	1.797	1.433	7.537	0.796	1.755	0.117	1.322	1.246	0.918	1.024	P04732	MT1E	Metallothionein-1E
						2.200	0.187	1.644	1.218	1.056	1.273	P04733	MT1F	Metallothionein-1F
						2.236	0.341	0.607	1.407	2.097	1.284	P80294	MT1H	Metallothionein-1H
									1.087	1.129	1.372	Q93083	MT1L	Metallothionein-1L
									1.070	0.914	1.215	Q8N339	MT1M	Metallothionein-1M
									1.059	1.129	1.127	P80297	MT1X	Metallothionein-1X
3.778	0.737	1.635	1.307	6.530	0.781	1.983	0.163	1.353	1.441	0.889	1.085	P02795	MT2A	Metallothionein-2
0.978	0.964	0.978							1.134	1.197	0.974	Q9Y483	MTF2	Metal-response element-binding transcription factor 2
1.009	1.009	1.025	1.031	0.990	1.020	0.994	1.024	1.018	1.040	1.064	1.006	Q13330	MTA1	Metastasis-associated protein MTA1
1.009	0.982	1.010	0.989	0.957	0.997	0.982	0.996	1.003	0.948	0.970	1.002	O94776	MTA2	Metastasis-associated protein MTA2
0.930	0.985	1.038	0.953	0.991	1.062	1.067	0.981	1.023	1.022	1.020	1.041	Q9BTC8	MTA3	Metastasis-associated protein MTA3
1.040	1.009	1.051	1.033	0.923	1.005	1.073	1.030	0.993	1.024	1.044	1.041	A0A0A0MRK6	MTX1	Metaxin 1, isoform CRA_b
1.039	1.033	0.986	1.027	1.099	0.959	1.037	0.973	1.044	1.005	0.998	1.013	O75431	MTX2	Metaxin-2
0.976	1.024	0.957	1.071	0.987	1.062	1.038	0.977	1.020	0.997	1.024	0.991	Q5HYI7	MTX3	Metaxin-3
									1.130	1.128	1.084	Q2M296	MTHFSD	Methenyltetrahydrofolate synthase domain-containing protein
1.006	0.984	1.011	1.015	1.022	0.998	1.007	1.012	1.037	1.003	1.008	1.032	Q9NZL9	MAT2B	Methionine adenosyltransferase 2 subunit beta

Abundance Ratio														
1 d M1/C1	1 d M2/C2	1 d M3/C3	4 d M1/C1	4 d M2/C2	4 d M3/C3	7 d M1/C1	7 d M2/C2	7 d M3/C3	10 d M1/C1	10 d M2/C2	10 d M3/C3	Accession ID	Gene name	Protein Description
1.043	0.996	0.988	1.026	1.277	0.956	1.098	0.894	1.079	1.019	1.006	0.979	P53582	METAP1	Methionine aminopeptidase 1
1.180	0.992	0.977	0.969	0.931	1.085	1.028	1.057	0.985	1.056	0.968	1.121	Q6UB28	METAP1D	Methionine aminopeptidase 1D, mitochondrial
0.947	0.944	0.981	0.967	0.983	0.995	0.971	0.908	0.992	1.016	1.029	1.067	F8VQZ7	METAP2	Methionine aminopeptidase 2
0.903	0.930	0.981	0.921	0.949	0.947	0.980	0.826	0.964	1.059	1.067	1.134	P50579	METAP2	Methionine aminopeptidase 2
1.000	0.998	1.021	1.004	1.038	1.015	0.991	1.061	1.057	1.035	0.911	1.058	Q99707	MTR	Methionine synthase
0.853	0.990	1.026	0.961	0.918	1.069	1.016	1.060	0.992	1.195	0.953	1.174	Q9UBK8	MTRR	Methionine synthase reductase
1.064	1.000	1.011	0.965	0.981	1.072	1.020	0.924	1.098	1.041	0.912	0.996	Q9Y3D2	MSRB2	Methionine-R-sulfoxide reductase B2, mitochondrial
0.992	0.998	0.997	0.992	0.993	0.993	0.973	0.982	1.002	0.975	0.993	0.977	P56192	MARS	Methionine--tRNA ligase, cytoplasmic
1.114	1.060	1.042	0.986	0.949	1.014	0.997	1.074	1.021	0.947	0.995	1.040	Q96GW9	MARS2	Methionine--tRNA ligase, mitochondrial
1.024	0.947	1.039	1.019	0.870	0.967	1.071	1.131	1.002	1.095	1.008	1.148	Q96DP5	MTFMT	Methionyl-tRNA formyltransferase, mitochondrial
1.002	0.970	0.969	1.001	1.011	1.008	1.010	0.944	1.064	1.043	1.029	1.018	P16455	MGMT	Methylated-DNA--protein-cysteine methyltransferase
1.040	0.994	0.979	1.021	0.931	1.032	1.034	1.076	1.044	0.988	1.024	0.976	Q9UBB5	MBD2	Methyl-CpG-binding domain protein 2
0.936	0.991	1.005	0.957	1.094	0.992	0.976	1.198	1.249	0.964	0.914	1.039	O95983	MBD3	Methyl-CpG-binding domain protein 3
0.991	0.995	0.985	0.997	0.971	0.965	1.016	1.038	0.971	1.025	1.003	0.995	Q9HCC0	MCCC2	Methylcrotonoyl-CoA carboxylase beta chain, mitochondrial
0.967	0.977	1.057	1.005	0.978	1.004	0.985	1.011	1.014	1.004	0.970	1.031	Q96RQ3	MCCC1	Methylcrotonoyl-CoA carboxylase subunit alpha, mitochondrial
1.046	0.996	1.018	1.025	0.994	1.015	1.073	1.035	1.023	1.070	0.989	1.042	Q13825	AUH	Methylglutaconyl-CoA hydratase, mitochondrial
0.949	1.020	0.966	1.004	1.005	0.998	1.002	1.046	0.993	1.031	0.983	1.016	Q02252	ALDH6A1	Methylmalonate-semialdehyde dehydrogenase [acylating], mitochondrial
1.014	1.054	1.367	0.864	1.064	1.200	0.904	0.864	1.151	0.909	0.905	1.008	Q9Y4U1	MMACHC	Methylmalonic aciduria and homocystinuria type C protein
1.205	0.966	1.039	1.096	0.935	1.020	1.015	0.979	1.028	0.966	1.032	0.995	Q96PE7	MCEE	Methylmalonyl-CoA epimerase, mitochondrial
0.993	1.011	1.036	0.971	0.989	1.014	0.998	1.009	1.013	1.033	0.988	1.050	P22033	MUT	Methylmalonyl-CoA mutase, mitochondrial
1.032	0.999	0.997	0.998	0.953	0.987	0.949	1.061	1.039	0.952	0.978	0.953	Q9BQA1	WDR77	Methylosome protein 50
1.047	1.020	1.027	1.021	0.929	0.957	1.002	1.096	0.974	0.975	1.015	1.022	P54105	CLNS1A	Methylosome subunit pICln
0.891	1.047	0.885	0.803	0.773	0.611	1.226	1.241	0.889	1.256	1.119	1.220	Q15800	MSMO1	Methylsterol monooxygenase 1
0.998	1.025	0.961	0.998	0.959	0.999	1.017	1.088	1.005	1.002	0.979	1.030	Q9BV20	MRI1	Methylthioribose-1-phosphate isomerase
0.971	0.984	1.043	1.018	1.005	0.998	0.992	1.039	0.996	1.017	1.023	0.987	Q96GX9	APIP	Methylthioribulose-1-phosphate dehydratase
0.998	1.002	1.010	1.048	1.016	1.086	1.041	1.064	1.045	1.068	1.076	1.073	Q96S19	METTL26	Methyltransferase-like 26
0.954	0.999	1.084	0.958	0.964	1.028	1.064	1.031	1.031	1.027	0.968	1.080	Q8N6R0	METTL13	Methyltransferase-like protein 13
1.059	0.966	1.050	1.052	1.002	0.977	1.016	1.039	1.001	1.050	1.002	1.030	Q86W50	METTL16	Methyltransferase-like protein 16
1.007	0.997	0.958	0.976	0.984	0.962	1.042	0.968	1.009	1.009	0.995	1.102	Q6P1Q9	METTL2B	Methyltransferase-like protein 2B
1.026	1.004	1.001	1.052	0.961	1.061	1.046	1.064	1.020	0.948	0.912	1.189	B8ZZC8	METTL5	Methyltransferase-like protein 5

Abundance Ratio														
1 d M1/C1	1 d M2/C2	1 d M3/C3	4 d M1/C1	4 d M2/C2	4 d M3/C3	7 d M1/C1	7 d M2/C2	7 d M3/C3	10 d M1/C1	10 d M2/C2	10 d M3/C3	Accession ID	Gene name	Protein Description
0.996	0.945	0.955	0.955	0.891	0.951	0.984	0.933	1.071	1.006	0.955	1.053	B3KW44	METTL8	Methyltransferase-like protein 8
0.953	1.157	1.057	1.021	0.955	1.048							Q9H1A3	METTL9	Methyltransferase-like protein 9
1.023	1.029	1.000	0.982	0.954	1.022	1.032	1.079	1.016	1.087	1.042	1.037	Q03426	MVK	Mevalonate kinase
0.865	1.106	1.120				1.016	0.923	1.035				Q6NT16	SLC18B1	MFS-type transporter SLC18B1
1.201	0.986	1.039	0.981	0.896	1.070	1.075	1.067	1.046	0.962	1.087	1.102	Q29983	MICA	MHC class I polypeptide-related sequence A
			1.107	0.927	1.076				1.074	1.130	1.279	A0A0G2JH58	MICB	MHC class I polypeptide-related sequence B
1.117	1.040	1.038	0.997	0.986	1.063	0.997	1.058	1.033	0.960	1.026	1.078	P22670	RFX1	MHC class II regulatory factor RFX1
0.961	0.979	0.983	1.022	1.011	1.000	1.020	0.977	1.000	0.999	1.003	1.083	Q8N3F8	MICALL1	MICAL-like protein 1
1.220	1.360	1.073	0.914	1.034	0.971	0.940	1.051	0.895	0.938	0.962	1.005	Q8IY33	MICALL2	MICAL-like protein 2
1.810	0.840	1.277	1.206	2.098	0.970	1.238	0.687	1.293	1.079	1.025	0.987	Q5TGZ0	MINOS1	MICOS complex subunit MIC10
0.984	0.946	0.963	0.979	0.950	0.993	0.998	0.845	0.978	1.074	1.018	1.045	A0A140TA86	C19orf70	MICOS complex subunit MIC13
1.017	0.936	1.041	1.015	0.958	1.018	1.024	1.091	1.036	1.056	1.036	1.014	Q9BUR5	APOO	MICOS complex subunit MIC26
1.024	1.021	1.009	0.985	0.949	0.961	0.998	1.096	0.998	1.023	1.021	0.992	A0A087WYF7	APOOL	MICOS complex subunit
1.009	0.991	0.975	0.973	0.943	0.977	1.004	0.913	0.956	1.083	1.022	1.010	C9JRZ6	CHCHD3	MICOS complex subunit
0.937	0.991	0.997	1.047	0.985	1.082	1.060	0.869	1.032	1.184	1.085	1.118	J3QTA6	CHCHD6	MICOS complex subunit
1.017	1.043	1.040	0.999	0.956	1.023	1.016	0.955	0.980	1.087	1.052	1.063	P55081	MFAP1	Microfibrillar-associated protein 1
						1.176	1.019	0.933				O75030	MITF	Microphthalmia-associated transcription factor
0.972	1.033	1.035	0.936	0.950	1.057	1.024	1.013	1.113	0.989	0.936	0.835	Q8WYQ5	DGCR8	Microprocessor complex subunit DGCR8
1.112	0.983	1.035	1.077	1.309	0.976	1.037	0.937	1.184	1.044	1.040	1.016	P10620	MGST1	Microsomal glutathione S-transferase 1
1.084	0.949	1.027	1.086	0.873	1.071							Q99735	MGST2	Microsomal glutathione S-transferase 2
0.836	0.932	0.924	0.894	0.847	0.965	0.951	0.862	0.974	1.140	1.032	1.029	Q5VV89	MGST3	Microsomal glutathione S-transferase 3
0.940	0.960	1.106	0.994	0.907	1.046	0.939	0.829	1.029	0.964	0.999	1.015	Q9Y4B5	MTCL1	Microtubule cross-linking factor 1
0.988	0.922	0.976	0.967	0.806	1.064	1.020	1.014	1.043	0.998	0.888	1.207	E9PLY5	MACF1	Microtubule-actin cross-linking factor 1, isoforms 1/2/3/5 (Fragment)
									1.009	1.135	1.085	H0YD69	MACF1	Microtubule-actin cross-linking factor 1, isoforms 1/2/3/5 (Fragment)
0.927	1.006	1.031	0.960	0.907	1.020	1.025	1.022	1.031	1.045	0.970	1.104	A0A0A6YYJ5	MACF1	Microtubule-actin cross-linking factor 1, isoforms 1/2/3/5
0.962	0.989	1.015	0.985	0.968	1.004	0.996	0.989	1.006	1.021	0.986	1.040	Q9UPN3	MACF1	Microtubule-actin cross-linking factor 1, isoforms 1/2/3/5
						0.964	1.059	0.955				E9PGC8	MAP1A	Microtubule-associated protein 1A
0.984	1.019	1.013	0.984	0.973	0.998	0.982	0.963	0.976	1.003	1.007	1.018	P46821	MAP1B	Microtubule-associated protein 1B
0.993	1.011	1.037	0.985	0.955	1.002	0.998	1.041	1.021	1.014	0.981	1.042	Q66K74	MAP1S	Microtubule-associated protein 1S
						1.085	0.896	1.157	1.023	1.250	1.688	P27816	MAP4	Microtubule-associated protein 4
0.952	0.981	0.984	0.969	0.989	0.987	0.961	0.931	0.976	0.997	1.013	1.001	E7EVA0	MAP4	Microtubule-associated protein

Abundance Ratio														
1 d M1/C1	1 d M2/C2	1 d M3/C3	4 d M1/C1	4 d M2/C2	4 d M3/C3	7 d M1/C1	7 d M2/C2	7 d M3/C3	10 d M1/C1	10 d M2/C2	10 d M3/C3	Accession ID	Gene name	Protein Description
			1.085	0.672	1.104							A0A0G2JMX7	MAPT	Microtubule-associated protein
1.018	0.992	0.991	0.978	0.986	0.991	0.992	1.050	1.014	0.993	0.979	0.996	Q15691	MAPRE1	Microtubule-associated protein RP/EB family member 1
1.051	1.033	0.984	1.028	0.980	0.984	0.951	1.032	0.909	0.909	0.937	0.984	Q15555	MAPRE2	Microtubule-associated protein RP/EB family member 2
1.021	0.999	0.940	1.039	0.965	1.034	1.053	1.065	1.003	1.046	0.993	1.060	Q9UPY8	MAPRE3	Microtubule-associated protein RP/EB family member 3
0.997	0.985	0.977	1.049	1.002	1.076	1.109	1.096	1.084	1.010	1.115	1.011	A6NCE7	MAP1LC3B2	Microtubule-associated proteins 1A/1B light chain 3 beta 2
1.015	0.931	1.255	1.022	0.605	1.022							Q6P0Q8	MAST2	Microtubule-associated serine/threonine-protein kinase 2
						0.993	1.009	0.996				Q9NPA3	MID1IP1	Mid1-interacting protein 1
0.992	0.975	1.040	0.983	0.928	1.019	0.999	1.017	1.017	0.974	0.977	1.030	Q9NU22	MDN1	Midasin
			1.009	1.887	0.908	1.275	0.519	1.169	1.115	1.132	1.089	E9PLM6	MDK	Midkine
0.963	0.971	1.042	1.002	0.904	1.078	1.040	1.050	0.932	1.107	1.033	1.075	Q9BRT3	MIEN1	Migration and invasion enhancer 1
1.011	0.987	0.968	0.981	0.973	0.984	0.997	0.930	0.931	1.067	0.988	1.016	Q8N183	NDUFAF2	Mimitin, mitochondrial
1.040	1.001	1.019	1.015	1.014	0.992	1.063	1.067	1.048	1.015	1.003	1.016	Q9BTE3	MCMBP	Mini-chromosome maintenance complex-binding protein
0.869	0.999	0.988	0.910	0.842	1.034	1.030	1.003	1.005	1.125	1.011	1.061	Q8TCT9	HM13	Minor histocompatibility antigen H13
0.915	1.049	1.048	1.011	0.922	1.037	0.962	1.053	1.011	0.942	0.940	1.033	Q9H5X1	FAM96A	MIP18 family protein FAM96A
			0.939	0.882	0.988	0.900	1.246	1.009				Q8TD10	MIPOL1	Mirror-image polydactyly gene 1 protein
1.024	1.006	0.922										Q6P0N0	MIS18BP1	Mis18-binding protein 1
1.026	1.009	1.020	1.012	0.959	0.977	0.983	1.088	1.109	0.965	1.017	1.100	P54278	PMS2	Mismatch repair endonuclease PMS2
1.045	1.027	1.002	1.038	0.989	1.002	1.034	1.030	1.001	1.052	1.011	1.041	Q8N4C8	MINK1	Misshapen-like kinase 1
1.094	1.025	1.006	0.989	0.947	1.012	0.976	1.099	0.986	0.906	1.029	1.123	Q8WV92	MITD1	MIT domain-containing protein 1
0.984	0.995	1.021	0.994	0.961	0.996	1.020	1.007	1.002	0.997	0.967	1.066	Q3SY69	ALDH1L2	Mitochondrial 10-formyltetrahydrofolate dehydrogenase
1.011	0.989	1.043	0.974	0.900	0.987	1.027	1.075	0.943	1.050	1.008	1.022	Q9BQT8	SLC25A21	Mitochondrial 2-oxodicarboxylate carrier
1.025	1.004	1.005	0.993	0.942	0.977	0.963	1.032	0.991	0.939	0.980	0.931	Q02978	SLC25A11	Mitochondrial 2-oxoglutarate/malate carrier protein
0.962	1.002	0.968	1.001	0.938	1.051	1.059	1.096	1.020	1.195	1.037	1.098	Q969Z3	2-Mar	Mitochondrial amidoxime reducing component 2
0.968	1.057	1.002	0.972	1.002	0.960	1.045	1.044	0.998	1.078	1.010	1.058	Q7Z434	MAVS	Mitochondrial antiviral-signaling protein
0.966	0.980	0.931	1.025	1.005	1.004	1.013	1.049	1.009	1.256	0.956	1.020	Q96EH3	MALSU1	Mitochondrial assembly of ribosomal large subunit protein 1
0.998	0.985	1.020	1.004	0.938	1.065	1.035	0.925	0.955	1.068	0.953	0.963	Q96AQ8	MCUR1	Mitochondrial calcium uniporter regulator 1
0.997	0.998	0.990	1.001	0.975	1.031	1.069	1.053	0.975	1.038	0.977	0.998	O43772	SLC25A20	Mitochondrial carnitine/acylcarnitine carrier protein

Abundance Ratio														
1 d M1/C1	1 d M2/C2	1 d M3/C3	4 d M1/C1	4 d M2/C2	4 d M3/C3	7 d M1/C1	7 d M2/C2	7 d M3/C3	10 d M1/C1	10 d M2/C2	10 d M3/C3	Accession ID	Gene name	Protein Description
1.003	1.000	0.998	0.996	0.901	1.005	1.017	1.084	1.003	1.100	1.092	1.021	Q9NZJ7	MTCH1	Mitochondrial carrier homolog 1
1.020	0.943	1.022	0.960	0.960	1.115	0.963	1.049	0.923	1.010	1.025	0.947	E9PIE4	MTCH2	Mitochondrial carrier homolog 2 (Fragment)
1.049	0.992	1.002	0.983	0.892	0.961	1.074	1.052	0.987	1.051	1.035	1.073	Q9Y6C9	MTCH2	Mitochondrial carrier homolog 2
1.010	1.003	1.005	1.015	0.956	1.027	1.014	1.062	0.988	0.990	0.979	0.984	Q9Y276	BCS1L	Mitochondrial chaperone BCS1
			0.912	1.017	0.979	0.926	1.089	1.016	0.905	1.342	0.932	Q86VD7	SLC25A42	Mitochondrial coenzyme A transporter SLC25A42
0.968	0.965	1.082	0.960	0.953	1.091	1.011	0.936	0.945	1.043	1.037	1.090	B0QY95	MIEF1	Mitochondrial dynamics protein MID51
0.899	1.008	0.997	1.070	1.067	1.161	1.134	0.994	1.113	1.020	1.011	1.080	Q7L5Y1	ENOSF1	Mitochondrial enolase superfamily member 1
1.009	0.973	1.047	1.009	1.055	1.052	1.055	0.994	1.090	1.075	1.021	1.090	Q9Y3D6	FIS1	Mitochondrial fission 1 protein
1.038	1.021	1.000	0.985	0.934	1.026	0.979	1.050	1.008	0.945	0.970	0.888	Q9UDX5	MTFP1	Mitochondrial fission process protein 1
0.898	0.933	1.041	0.960	0.966	1.023	0.881	0.820	1.040	0.948	1.023	1.009	Q15390	MTFR1	Mitochondrial fission regulator 1
0.973	1.012	1.062	0.995	0.989	1.113	0.959	0.953	1.002	1.007	0.973	1.009	Q9H019	MTFR1L	Mitochondrial fission regulator 1-like
0.986	1.000	0.992	1.017	0.937	1.010	0.964	1.055	0.983	0.953	0.994	1.025	Q9H2D1	SLC25A32	Mitochondrial folate transporter/carrier
0.972	1.042	1.007	0.976	0.949	0.999	1.020	1.008	0.982	1.005	0.977	1.058	Q9BQP7	MGME1	Mitochondrial genome maintenance exonuclease 1
0.988	0.993	1.009	0.990	0.951	1.039	1.004	1.044	0.980	0.999	1.002	1.028	Q9H936	SLC25A22	Mitochondrial glutamate carrier 1
1.020	0.988	0.999	0.896	0.942	1.067	0.997	0.914	0.995	1.118	1.049	1.094	Q9Y5J6	TIMM10B	Mitochondrial import inner membrane translocase subunit Tim10 B
0.997	0.905	1.053	0.964	1.012	0.967	0.974	0.802	0.998	1.033	1.026	1.051	P62072	TIMM10	Mitochondrial import inner membrane translocase subunit Tim10
0.960	0.933	0.954	0.962	0.984	1.015	1.006	1.029	1.010	0.937	1.014	0.918	Q9Y5L4	TIMM13	Mitochondrial import inner membrane translocase subunit Tim13
1.030	1.001	0.988	1.016	1.001	0.984	1.021	1.048	0.972	1.057	1.030	0.978	Q96DA6	DNAJC19	Mitochondrial import inner membrane translocase subunit TIM14
0.980	1.104	1.070	0.980	1.130	0.986	1.018	0.947	1.071				Q99595	TIMM17A	Mitochondrial import inner membrane translocase subunit Tim17-A
1.036	1.009	1.027	0.979	1.029	1.027	1.020	0.871	0.991	1.099	1.013	1.062	O60830	TIMM17B	Mitochondrial import inner membrane translocase subunit Tim17-B
0.946	1.011	0.974	0.971	0.930	0.998	1.047	0.967	0.979	1.102	1.008	1.055	Q9BVV7	TIMM21	Mitochondrial import inner membrane translocase subunit Tim21
0.919	0.984	1.017	1.074	1.017	0.912	1.050	1.026	1.070	1.086	1.028	1.083	Q9Y584	TIMM22	Mitochondrial import inner membrane translocase subunit Tim22
0.935	1.003	1.030	1.078	1.029	1.051	0.904	1.154	1.085	0.973	0.920	1.022	O14925	TIMM23	Mitochondrial import inner membrane translocase subunit Tim23
1.130	0.920	1.091	0.998	0.920	1.002	1.077	1.064	1.063	1.080	1.005	1.086	Q9BSF4	TIMM29	Mitochondrial import inner membrane translocase subunit Tim29
0.981	0.969	1.007	0.973	0.965	1.002	0.956	0.907	0.977	0.975	0.972	0.996	O43615	TIMM44	Mitochondrial import inner membrane translocase subunit TIM44

Abundance Ratio														
1 d M1/C1	1 d M2/C2	1 d M3/C3	4 d M1/C1	4 d M2/C2	4 d M3/C3	7 d M1/C1	7 d M2/C2	7 d M3/C3	10 d M1/C1	10 d M2/C2	10 d M3/C3	Accession ID	Gene name	Protein Description
1.009	0.982	0.994	1.013	0.964	0.980	0.970	1.097	1.000	0.978	0.994	0.978	O60220	TIMM8A	Mitochondrial import inner membrane translocase subunit Tim8 A
1.085	0.980	1.029	0.943	1.129	0.979	1.023	0.876	0.981	1.096	0.953	1.091	G3XAN8	TIMM8B	Mitochondrial import inner membrane translocase subunit Tim8 B
0.994	0.990	0.946	0.981	1.044	0.952	0.989	0.949	0.941	1.011	1.005	0.998	Q9Y5J7	TIMM9	Mitochondrial import inner membrane translocase subunit Tim9
1.013	0.986	1.051	0.999	0.999	0.973	1.073	0.962	1.027	1.162	0.999	1.105	Q15388	TOMM20	Mitochondrial import receptor subunit TOM20 homolog
0.924	1.035	1.011	0.986	0.966	0.983	0.992	1.045	1.014	0.975	1.013	1.008	Q9NS69	TOMM22	Mitochondrial import receptor subunit TOM22 homolog
0.986	0.984	1.013	1.001	1.023	0.999	1.012	0.972	1.041	1.047	0.993	1.046	Q15785	TOMM34	Mitochondrial import receptor subunit TOM34
0.914	0.997	0.983	0.965	0.913	1.018	0.981	1.027	0.937	1.015	1.019	0.996	O96008	TOMM40	Mitochondrial import receptor subunit TOM40 homolog
1.054	0.970	0.988	0.970	0.813	0.984	1.033	0.842	1.047	1.096	1.098	0.914	Q969M1	TOMM40L	Mitochondrial import receptor subunit TOM40B
			0.944	0.940	1.112				1.046	0.945	1.218	Q8N4H5	TOMM5	Mitochondrial import receptor subunit TOM5 homolog
0.849	0.765	1.092	0.896	1.000	0.997	0.962	0.986	1.001	0.915	1.000	1.169	Q96B49	TOMM6	Mitochondrial import receptor subunit TOM6 homolog
0.967	1.004	0.998	1.014	0.998	0.996	1.007	1.000	0.969	1.024	1.023	0.993	O94826	TOMM70	Mitochondrial import receptor subunit TOM70
1.108	1.005	0.980	0.974	0.971	0.987	1.028	1.027	0.989	0.952	1.011	0.944	Q9Y6H3	ATP23	Mitochondrial inner membrane protease ATP23 homolog
1.027	1.042	1.133	0.900	0.880	1.038	0.914	1.079	0.981	1.010	0.997	1.077	Q96LU5	IMMP1L	Mitochondrial inner membrane protease subunit 1
1.101	1.118	1.299	1.073	0.807	0.904	0.867	1.566	0.709	0.873	1.026	0.987	Q96T52	IMMP2L	Mitochondrial inner membrane protease subunit 2
0.951	0.990	1.001	1.015	0.921	1.040	1.066	1.047	0.981	1.099	1.037	1.020	J3KNA0	OXA1L	Mitochondrial inner membrane protein OXA1L
0.959	0.897	0.957	0.893	0.841	0.891	0.910	0.879	0.912	0.934	0.938	0.979	Q99797	MIPEP	Mitochondrial intermediate peptidase
1.027	0.980	1.007	0.982	0.952	0.941	1.022	1.117	1.010	0.958	1.061	0.957	Q9Y619	SLC25A15	Mitochondrial ornithine transporter 1
1.086	1.027	0.990	1.039	1.025	1.001	1.018	1.014	0.920	1.103	0.966	1.033	Q9UJ68	MSRA	Mitochondrial peptide methionine sulfoxide reductase
1.004	1.013	0.999	1.005	1.058	0.990	0.986	1.127	1.022	0.932	0.977	0.980	O95563	MPC2	Mitochondrial pyruvate carrier 2
									1.512	1.019	0.837	A0A087WVZ0	MPC1	Mitochondrial pyruvate carrier
1.011	0.995	1.024	1.000	0.924	0.952	1.017	1.094	1.003	1.016	1.010	1.001	Q8IXI1	RHOT2	Mitochondrial Rho GTPase 2
0.994	1.003	1.011	1.004	0.999	1.002	1.017	1.044	1.022	1.023	1.009	1.035	Q7L0Y3	TRMT10C	Mitochondrial ribonuclease P protein 1
1.018	1.000	1.040	0.924	0.968	0.969	0.971	1.000	0.997	0.927	0.938	1.021	O15091	KIAA0391	Mitochondrial ribonuclease P protein 3
0.950	0.995	1.038	0.986	0.941	1.074	1.046	0.978	0.979	1.066	0.988	0.950	Q9BT17	MTG1	Mitochondrial ribosome-associated GTPase 1
0.852	0.869	0.903	0.897	0.841	0.935	1.008	1.006	0.965	1.031	0.969	1.046	Q9H4K7	MTG2	Mitochondrial ribosome-associated GTPase 2
0.995	0.993	1.023	0.999	0.945	1.033	1.033	1.049	0.980	1.022	1.011	1.023	Q9HC21	SLC25A19	Mitochondrial thiamine pyrophosphate carrier

Abundance Ratio														
1 d M1/C1	1 d M2/C2	1 d M3/C3	4 d M1/C1	4 d M2/C2	4 d M3/C3	7 d M1/C1	7 d M2/C2	7 d M3/C3	10 d M1/C1	10 d M2/C2	10 d M3/C3	Accession ID	Gene name	Protein Description
1.036	1.001	1.016	1.005	0.947	0.971	0.994	1.144	0.960	1.013	0.986	0.955	O75648	TRMU	Mitochondrial tRNA-specific 2-thiouridylase 1
0.915	1.012	1.001	0.978	0.912	1.005	1.025	0.983	0.960	1.116	1.021	1.055	Q969V5	MUL1	Mitochondrial ubiquitin ligase activator of NFKB 1
1.003	0.952	0.980	0.953	0.948	0.934	0.956	0.860	0.958	0.949	0.893	0.928	Q10713	PMPCA	Mitochondrial-processing peptidase subunit alpha
0.970	0.968	0.972	0.948	0.914	0.927	0.950	0.963	0.911	0.982	0.930	0.955	O75439	PMPCB	Mitochondrial-processing peptidase subunit beta
0.911	1.057	0.940	1.193	0.910	0.947	1.092	1.249	0.910	1.200	1.082	1.165	Q9NYZ2	SLC25A37	Mitoferrin-1
						1.076	0.925	1.180				Q96A46	SLC25A28	Mitoferrin-2
1.053	1.007	1.021	1.034	0.968	1.033	1.037	1.075	1.033	1.061	1.067	1.041	A0A0C4DFN1	MFN1	Mitofusin-1
1.043	1.005	1.048	1.021	0.982	1.048	1.043	1.050	1.007	1.057	1.013	1.078	O95140	MFN2	Mitofusin-2
0.986	0.996	0.995	1.017	1.002	1.012	1.031	1.067	1.029	1.021	0.991	1.027	P28482	MAPK1	Mitogen-activated protein kinase 1
1.030	1.039	1.058	1.063	0.988	1.065	1.097	1.009	0.977	1.203	1.020	0.719	Q15759	MAPK11	Mitogen-activated protein kinase 11
1.042	1.029	1.015	0.982	0.895	1.102	0.994	1.093	1.069	1.012	1.060	0.986	P53778	MAPK12	Mitogen-activated protein kinase 12
1.134	0.899	0.910	0.940	1.004	1.059	1.060	1.205	1.035				O15264	MAPK13	Mitogen-activated protein kinase 13
0.990	1.004	1.004	0.995	0.990	1.035	1.003	1.084	1.036	0.979	0.959	0.992	Q16539	MAPK14	Mitogen-activated protein kinase 14
0.965	0.992	0.999	1.011	0.984	1.051	1.022	1.034	1.010	1.049	1.025	1.026	P27361	MAPK3	Mitogen-activated protein kinase 3
1.039	0.969	1.006	1.014	0.926	1.016	1.062	0.897	1.059	0.967	0.974	1.035	Q13164	MAPK7	Mitogen-activated protein kinase 7
1.021	0.977	0.989	0.958	1.071	1.062	1.059	0.952	0.981	0.940	1.064	1.118	P45983	MAPK8	Mitogen-activated protein kinase 8
0.979	1.013	1.030	0.997	0.982	1.032	1.033	1.045	0.969	1.004	1.021	1.009	P45984	MAPK9	Mitogen-activated protein kinase 9
1.408	0.985	0.849										Q13233	MAP3K1	Mitogen-activated protein kinase kinase kinase 1
			0.933	0.792	0.971				1.011	0.951	1.040	Q16584	MAP3K11	Mitogen-activated protein kinase kinase kinase 11
1.091	1.017	1.024	1.009	0.955	0.994	1.033	1.005	1.007	1.005	0.960	0.989	Q9Y2U5	MAP3K2	Mitogen-activated protein kinase kinase kinase 2
1.025	1.040	1.036	1.007	0.986	1.044	1.046	1.091	1.044	0.999	0.970	1.014	Q9NYL2	MAP3K20	Mitogen-activated protein kinase kinase kinase 20
1.020	1.105	1.053	1.016	0.926	0.943	1.092	1.082	1.046	1.023	0.950	0.999	Q9Y6R4	MAP3K4	Mitogen-activated protein kinase kinase kinase 4
0.916	1.096	0.901	0.953	0.934	1.023	1.072	1.044	0.977	0.996	0.903	1.118	Q99683	MAP3K5	Mitogen-activated protein kinase kinase kinase 5
1.002	1.068	0.989	1.008	0.951	1.049	0.982	1.102	1.078	1.044	1.013	0.966	O95382	MAP3K6	Mitogen-activated protein kinase kinase kinase 6
0.933	0.956	0.982	1.011	0.986	0.973	1.025	1.067	0.930	0.953	1.011	1.034	Q12851	MAP4K2	Mitogen-activated protein kinase kinase kinase kinase 2
1.013	1.021	1.056	1.041	0.886	1.168	1.087	1.130	1.034	1.012	0.983	0.925	Q8IVH8	MAP4K3	Mitogen-activated protein kinase kinase kinase kinase 3
0.921	1.044	1.084	0.902	1.013	1.104	1.005	0.695	1.378				O95819	MAP4K4	Mitogen-activated protein kinase kinase kinase kinase 4
0.980	1.026	1.020	0.988	0.929	1.000	1.038	1.036	0.975	1.035	0.951	0.997	A0A0A0MQR1	MAP4K5	Mitogen-activated protein kinase kinase kinase kinase
1.111	1.006	1.097	1.070	0.899	1.084	0.945	1.151	1.091	0.824	0.940	1.025	F8W7S1	MIGA1	Mitoguardin 1

Abundance Ratio														
1 d M1/C1	1 d M2/C2	1 d M3/C3	4 d M1/C1	4 d M2/C2	4 d M3/C3	7 d M1/C1	7 d M2/C2	7 d M3/C3	10 d M1/C1	10 d M2/C2	10 d M3/C3	Accession ID	Gene name	Protein Description
1.000	0.997	0.988	0.994	0.974	0.980	1.010	1.039	1.016	1.004	1.001	1.016	O43684	BUB3	Mitotic checkpoint protein BUB3
1.019	0.959	1.101										O43683	BUB1	Mitotic checkpoint serine/threonine-protein kinase BUB1
									1.050	1.106	1.088	Q8IVT2	MISP	Mitotic interactor and substrate of PLK1
1.005	0.958	1.048	0.968	0.989	1.070	0.958	0.955	0.966	0.996	0.990	1.045	Q9Y6D9	MAD1L1	Mitotic spindle assembly checkpoint protein MAD1
1.014	1.026	0.986	1.040	0.975	1.031	1.053	1.046	0.979	0.906	0.945	0.952	Q13257	MAD2L1	Mitotic spindle assembly checkpoint protein MAD2A
0.908	0.972	1.313	1.238	1.024	1.252	0.961	0.770	1.016	1.159	1.046	1.229	B1AK44	MAD2L2	Mitotic spindle assembly checkpoint protein MAD2B
1.008	0.935	1.019	0.999	0.946	0.988	0.989	0.977	1.103	1.015	0.960	1.159	Q9Y3D0	FAM96B	Mitotic spindle-associated MMXD complex subunit MIP18
									0.928	0.940	0.993	Q08AG7	MZT1	Mitotic-spindle organizing protein 1
			1.081	1.121	1.163	1.105	1.394	1.247	0.753	1.131	1.119	Q6P582	MZT2A	Mitotic-spindle organizing protein 2A
1.040	0.970	1.040	0.952	1.077	1.064	0.942	0.925	1.012	0.873	0.959	1.048	B8ZZ87	MZT2B	Mitotic-spindle organizing protein 2B
0.991	1.018	1.015	0.975	0.944	0.963	1.021	1.011	0.977	1.043	0.870	1.082	Q8NB16	MLKL	Mixed lineage kinase domain-like protein
1.028	0.998	0.978	1.031	0.946	1.011	1.020	1.035	1.028	1.038	0.992	1.020	Q9BYG3	NIFK	MKI67 FHA domain-interacting nucleolar phosphoprotein
1.030	0.992	1.030	0.996	0.950	1.008	0.995	1.081	0.998	0.947	0.955	1.068	W0Z7M9	mkl1	MKL/myocardin-like protein 1
			1.015	0.955	0.992	0.965	1.166	0.928				O95772	STARD3NL	MLN64 N-terminal domain homolog
0.975	0.977	0.903	1.089	1.063	0.954				1.030	1.067	0.994	Q9HAP2	MLXIP	MLX-interacting protein
0.961	1.089	1.044	0.984	0.963	1.082	1.041	1.047	1.015	1.013	0.964	1.048	Q495G5	MMAA	MMAA protein
						0.848	0.883	1.832				A0A0U1RRL7	MMP24-AS1	MMP24 antisense RNA 1
1.096	1.035	0.992	1.090	0.807	0.976	1.053	0.853	0.953	1.212	0.759	1.016	Q96BX8	MOB3A	MOB kinase activator 3A
0.875	0.823	1.334	1.148	0.969	0.984	1.120	1.149	1.013				X6R3L3	MOB3C	MOB kinase activator 3C
1.032	0.953	1.038	1.035	1.080	1.045	1.031	0.826	1.101	1.004	1.000	1.057	Q9Y3A3	MOB4	MOB-like protein phocein
0.933	0.999	0.996	0.870	0.949	0.964	0.901	1.377	1.101				Q9BWK5	MRI	Modulator of retrovirus infection homolog
0.999	1.004	0.993	0.994	0.973	1.003	0.958	0.992	0.976	0.951	0.987	0.949	P26038	MSN	Moesin
0.996	1.000	1.046	0.955	0.901	0.988	1.002	1.038	0.996	0.995	0.933	1.086	Q9NZB8	MOCS1	Molybdenum cofactor biosynthesis protein 1
1.037	0.974	1.013	1.058	0.972	1.006	1.081	1.144	1.212	1.030	1.000	1.026	Q96EN8	MOCOS	Molybdenum cofactor sulfurase
1.025	1.012	1.069	1.019	0.917	1.002	1.007	1.108	1.040	1.021	0.975	1.037	O96007	MOCS2	Molybdopterin synthase catalytic subunit
0.934	0.915	0.970	0.941	0.944	1.013	1.028	0.971	1.012	1.051	0.990	1.047	O96033	MOCS2	Molybdopterin synthase sulfur carrier subunit
1.182	1.184	1.068	1.019	1.035	1.277	0.950	1.097	1.040	1.127	0.854	1.042	X6R3V9	MON1A	MON1 homolog A (Yeast), isoform CRA_b
1.028	1.075	0.952	0.973	0.899	1.091	1.006	1.104	0.964	0.983	0.960	0.951	Q9BV23	ABHD6	Monoacylglycerol lipase ABHD6
0.924	0.989	1.012	0.982	0.912	0.994	0.966	0.868	1.037	1.064	1.113	1.121	P53985	SLC16A1	Monocarboxylate transporter 1
0.906	0.961	1.029	0.962	0.932	1.199	0.976	0.912	1.015	1.110	1.036	1.068	O60669	SLC16A7	Monocarboxylate transporter 2

Abundance Ratio														
1 d M1/C1	1 d M2/C2	1 d M3/C3	4 d M1/C1	4 d M2/C2	4 d M3/C3	7 d M1/C1	7 d M2/C2	7 d M3/C3	10 d M1/C1	10 d M2/C2	10 d M3/C3	Accession ID	Gene name	Protein Description
1.003	1.016	0.982	1.028	0.899	1.137	1.086	1.152	1.045	1.103	1.131	1.043	O15427	SLC16A3	Monocarboxylate transporter 4
						0.728	0.649	0.824				P36021	SLC16A2	Monocarboxylate transporter 8
1.004	0.871	1.034										P08571	CD14	Monocyte differentiation antigen CD14
0.889	1.045	0.992	1.005	1.127	1.009	1.069	1.005	1.140	1.179	1.018	1.079	Q6UB35	MTHFD1L	Monofunctional C1-tetrahydrofolate synthase, mitochondrial
1.057	1.056	1.051	1.008	0.979	0.969	1.011	1.049	0.939	0.931	0.902	0.942	A0A0C4DFN3	MGLL	Monoglyceride lipase
1.046	1.025	1.009	0.970	0.992	1.042	0.935	0.897	0.932	1.055	0.998	1.039	Q9Y6X9	MORC2	MORC family CW-type zinc finger protein 2
1.028	0.975	1.041	0.974	0.960	1.037	0.982	1.015	1.006	0.983	0.989	1.080	Q14149	MORC3	MORC family CW-type zinc finger protein 3
			0.970	0.889	0.832							Q8TE76	MORC4	MORC family CW-type zinc finger protein 4
									0.801	0.750	1.184	Q9Y605	MRFAP1	MORF4 family-associated protein 1
1.042	1.001	0.991	1.023	0.998	0.971	1.048	1.092	0.981	1.054	1.006	1.013	B3KTM8	MORF4L1	Mortality factor 4-like protein 1
0.986	0.974	0.995	0.982	0.900	1.029	1.003	1.052	0.950	1.041	0.975	1.039	Q15014	MORF4L2	Mortality factor 4-like protein 2
0.897	1.009	0.976	1.029	1.030	1.074	1.072	0.859	0.997	1.086	1.031	1.074	Q15796	SMAD2	Mothers against decapentaplegic homolog 2
0.933	0.952	0.945	0.948	0.915	0.966	1.051	1.030	0.980	1.026	0.976	1.056	P84022	SMAD3	Mothers against decapentaplegic homolog 3
1.020	1.018	1.015	0.982	1.011	0.946	1.037	0.996	1.014	1.036	0.920	0.976	Q13485	SMAD4	Mothers against decapentaplegic homolog 4
1.063	1.007	1.062	1.003	1.039	1.031	1.025	0.917	1.035	1.199	1.048	1.091	Q99717	SMAD5	Mothers against decapentaplegic homolog 5
1.054	1.048	1.034	1.014	0.966	1.002	0.990	1.074	1.029	0.975	1.003	1.052	Q8NHP6	MOSPD2	Motile sperm domain-containing protein 2
1.144	0.983	1.261				0.847	0.975	0.932	0.881	0.817	1.210	P50219	MNX1	Motor neuron and pancreas homeobox protein 1
1.078	1.005	1.084										P30307	CDC25C	M-phase inducer phosphatase 3
0.988	1.047	1.104	0.993	1.007	1.111	1.025	1.068	1.165	1.008	1.041	1.143	Q99547	MPHOSPH6	M-phase phosphoprotein 6
1.029	0.989	1.017	0.985	0.929	1.016	1.005	1.004	1.024	0.963	0.979	1.011	Q99549	MPHOSPH8	M-phase phosphoprotein 8
1.105	1.069	1.045	1.016	0.931	0.986	1.034	1.231	1.023	1.045	0.959	1.003	Q567V2	MPV17L2	Mpv17-like protein 2
1.039	0.941	1.027	0.979	0.910	0.998	0.912	1.013	1.023	0.946	0.951	1.049	Q9NV56	MRGBP	MRG/MORF4L-binding protein
0.982	0.918	1.041	0.965	0.852	1.008	0.858	1.148	1.069	0.906	1.099	0.940	Q6NTE8	MRNIP	MRN complex-interacting protein
			0.918	0.919	0.930	1.104	1.254	0.893	1.091	1.124	1.179	M0R0H3	ZFP36	mRNA decay activator protein ZFP36 (Fragment)
0.980	1.014	1.049	0.964	1.006	0.960	0.981	0.947	0.972	1.051	1.039	1.076	Q07352	ZFP36L1	mRNA decay activator protein ZFP36L1
1.020	1.038	1.060	1.024	1.046	1.019	0.977	0.998	0.988	1.004	1.073	1.126	P47974	ZFP36L2	mRNA decay activator protein ZFP36L2
1.017	1.016	1.018	1.018	1.037	0.968	1.002	0.991	1.016	0.989	1.031	0.983	P78406	RAE1	mRNA export factor
0.988	0.998	1.011	1.005	0.995	1.009	1.030	1.070	1.041	0.990	1.017	1.013	Q9UKD2	MRTO4	mRNA turnover protein 4 homolog
1.017	1.008	1.011	1.017	0.993	1.031	1.010	0.969	1.002	0.969	0.890	1.053	O60942	RNGTT	mRNA-capping enzyme
1.114	1.020	1.070	1.043	0.930	1.011	1.035	1.137	1.027	1.039	1.017	1.033	Q9NPI6	DCP1A	mRNA-decapping enzyme 1A
						1.092	1.186	1.526				Q8IZD4	DCP1B	mRNA-decapping enzyme 1B
0.997	1.001	1.006	0.955	0.925	1.009	0.976	1.014	0.998	1.005	0.980	1.039	Q96T58	SPEN	Msx2-interacting protein
0.984	0.874	0.968	0.949	0.910	0.980	0.992	0.972	0.959	0.988	1.014	1.038	E9PNP3	AAMDC	Mth938 domain-containing protein

Abundance Ratio														
1 d M1/C1	1 d M2/C2	1 d M3/C3	4 d M1/C1	4 d M2/C2	4 d M3/C3	7 d M1/C1	7 d M2/C2	7 d M3/C3	10 d M1/C1	10 d M2/C2	10 d M3/C3	Accession ID	Gene name	Protein Description
0.986	0.998	1.002	0.995	0.977	1.005	1.019	1.019	1.002	1.026	1.017	1.032	B7ZM99	MTHFD1L	MTHFD1L protein
0.900	1.091	1.207	0.973	0.913	1.286				1.018	0.933	1.204	Q9GZU1	MCOLN1	Mucolipin-1
0.998	0.996	0.999	1.014	0.969	0.998	1.028	0.991	1.048	1.017	0.963	1.078	Q9UDY8	MALT1	Mucosa-associated lymphoid tissue lymphoma translocation protein 1
0.955	0.987	1.025	0.966	0.889	1.026	1.083	0.956	1.051	1.025	0.992	1.081	P08183	ABCB1	Multidrug resistance protein 1
0.962	0.995	1.017	0.998	0.918	1.043	1.017	0.992	1.031	1.060	1.041	1.042	O15439	ABCC4	Multidrug resistance-associated protein 4
0.804	0.821	1.000							1.065	0.858	0.837	O15440	ABCC5	Multidrug resistance-associated protein 5
1.020	0.989	0.993	0.989	1.114	0.999	1.001	1.041	1.093	1.030	1.037	1.068	Q9UI30	TRMT112	Multifunctional methyltransferase subunit TRM112-like protein
0.978	0.971	0.996	0.998	1.034	0.985	0.998	1.011	0.976	0.962	0.980	0.982	P22234	PAICS	Multifunctional protein ADE2
1.029	1.027	0.965	1.021	0.967	0.927	1.003	1.083	0.937	1.033	1.050	0.887	Q8NI22	MCFD2	Multiple coagulation factor deficiency protein 2
			1.128	0.995	1.092	1.073	1.231	1.102	1.156	1.174	1.142	Q9H1U4	MEGF9	Multiple epidermal growth factor-like domains protein 9
0.968	1.006	1.017	1.049	0.951	1.012	1.008	1.038	1.006	1.034	0.973	1.043	Q9UNW1	MINPP1	Multiple inositol polyphosphate phosphatase 1
1.038	0.988	0.970	1.031	1.025	0.952	1.005	1.031	1.007	1.028	1.015	1.026	Q9BU76	MMTAG2	Multiple myeloma tumor-associated protein 2
1.018	0.996	1.039	0.985	0.911	0.939	1.020	0.999	1.021	1.005	1.030	1.086	F5H1U9	MPDZ	Multiple PDZ domain protein
1.043	0.939	1.133	0.973	1.125	1.053	1.067	1.094	1.032	0.985	0.986	1.031	Q96EY5	MVB12A	Multivesicular body subunit 12A
			0.917	0.966	1.059	1.020	0.978	1.045				F6XAJ2	MUSK	Muscle, skeletal receptor tyrosine-protein kinase (Fragment)
1.083	1.028	1.009	1.039	1.207	0.993	1.040	0.916	1.029	0.995	0.999	1.033	A0A0A0MQX8	MBNL1	Muscleblind-like protein 1
1.102	1.076	0.935	1.025	0.875	0.981	1.115	1.065	1.044	1.069	0.847	0.953	Q5VZF2	MBNL2	Muscleblind-like protein 2
1.028	1.041	0.933	1.105	0.859	0.958	1.225	1.009	0.928	1.052	0.972	1.055	Q9UL63	MKLN1	Muskelin
			0.988	0.942	0.930							Q6P1R3	MSANTD2	Myb/SANT-like DNA-binding domain-containing protein 2
1.030	0.987	0.988	0.847	0.947	1.031	0.888	0.550	0.910	1.124	0.932	1.165	Q96H12	MSANTD3	Myb/SANT-like DNA-binding domain-containing protein 3
1.019	0.988	0.997	1.016	0.995	1.015	1.005	1.036	1.023	0.986	0.997	0.974	Q9NUJ1	ABHD10	Mycophenolic acid acyl-glucuronide esterase, mitochondrial
1.042	1.004	0.965	0.926	0.934	1.069	0.988	0.905	0.965	1.079	1.038	1.095	P02686	MBP	Myelin basic protein
0.967	0.987	1.025	1.060	1.003	1.107	1.061	1.094	1.050	1.087	1.024	1.157	O95297	MPZL1	Myelin protein zero-like protein 1
1.022	1.003	1.014	1.007	1.038	1.009	1.023	1.002	1.028	1.011	0.978	1.008	A0A0A0MST0	MYD88	Myeloid differentiation primary response protein MyD88
0.951	1.002	1.106	0.951	0.931	1.017	1.003	0.866	1.012	1.108	0.989	1.147	Q15773	MLF2	Myeloid leukemia factor 2
0.936	1.073	0.955	0.953	0.891	1.000	1.048	1.144	1.057	1.091	1.093	1.044	Q96S97	MYADM	Myeloid-associated differentiation marker
1.010	1.017	0.984	1.002	0.987	0.987	0.940	1.073	0.952	0.952	0.976	0.918	Q969H8	MYDGF	Myeloid-derived growth factor
0.989	0.997	1.022	1.018	0.973	1.016	1.037	1.027	1.043	1.037	1.058	1.019	Q9NZM1	MYOF	Myoferlin
0.962	0.907	1.119	1.014	0.959	1.000	0.834	1.037	0.942				P02144	MB	Myoglobin

Abundance Ratio														
1 d M1/C1	1 d M2/C2	1 d M3/C3	4 d M1/C1	4 d M2/C2	4 d M3/C3	7 d M1/C1	7 d M2/C2	7 d M3/C3	10 d M1/C1	10 d M2/C2	10 d M3/C3	Accession ID	Gene name	Protein Description
0.882	0.948	1.005	0.878	0.944	1.072	0.944	0.949	0.978	0.968	0.940	1.039	E9PL24	PDE4DIP	Myomegalin
0.996	0.987	1.105	0.970	1.007	1.000	1.020	1.055	1.095	0.998	0.995	1.068	P52179	MYOM1	Myomesin-1
0.987	0.973	0.992	1.013	0.954	1.055	1.043	0.956	0.999	1.054	0.930	1.107	P14649	MYL6B	Myosin light chain 6B
									0.856	0.680	0.610	G8JLA2	MYL6	Myosin light polypeptide 6
0.966	0.995	0.945	1.010	0.991	0.982	0.980	0.991	0.979	0.971	0.986	0.962	G3V1V0	MYL6	Myosin light polypeptide 6
1.024	0.911	1.054	1.058	0.880	1.024	0.963	0.997	0.978	1.037	0.918	0.898	H0Y2S9	MPRIP	Myosin phosphatase Rho-interacting protein (Fragment)
0.984	0.993	1.010	0.999	0.943	0.949	0.923	0.988	0.989	0.903	1.007	0.895	J3QRS3	MYL12A	Myosin regulatory light chain 12A
0.995	1.015	1.005	1.026	0.993	0.938	1.027	1.004	0.985	0.962	0.968	0.969	P24844	MYL9	Myosin regulatory light polypeptide 9
0.969	1.002	0.990	0.996	1.029	0.967	0.998	0.975	0.996	0.971	0.978	0.981	P35579	MYH9	Myosin-9
0.941	0.991	0.952	0.998	0.971	0.999	1.004	1.004	0.976	1.010	0.972	0.993	P58546	MTPN	Myotrophin
1.063	1.063	1.049	0.982	0.966	0.993	0.998	1.002	1.020	0.965	0.980	1.017	Q13496	MTM1	Myotubularin
1.016	1.006	1.060	0.989	0.970	1.064	1.047	1.010	1.012	0.995	1.005	1.104	F8WA39	MTMR1	Myotubularin-related protein 1
1.084	1.096	1.002	0.977	0.966	0.982	1.018	1.073	1.017	1.011	1.011	0.972	Q9NXD2	MTMR10	Myotubularin-related protein 10
1.023	0.998	1.050	1.009	0.970	1.007	1.003	1.006	1.028	1.024	0.999	1.083	Q9C0I1	MTMR12	Myotubularin-related protein 12
0.912	0.970	1.026	0.961	0.974	0.928	0.965	0.950	1.100				Q86WG5	SBF2	Myotubularin-related protein 13
1.076	0.979	1.010	1.029	1.018	0.990	1.029	0.976	1.000	0.986	1.003	0.981	Q8NCE2	MTMR14	Myotubularin-related protein 14
1.087	1.049	1.084	1.039	0.941	0.976	0.970	1.042	0.996	0.946	0.984	1.007	Q13614	MTMR2	Myotubularin-related protein 2
1.112	0.929	1.047	1.136	0.920	0.978	1.172	0.922	0.978	0.973	1.022	1.069	Q13615	MTMR3	Myotubularin-related protein 3
			0.849	0.879	1.091							Q9NYA4	MTMR4	Myotubularin-related protein 4
0.952	1.005	1.124	0.988	0.936	1.044	0.964	1.020	1.072	1.051	1.036	1.058	Q9Y217	MTMR6	Myotubularin-related protein 6
1.097	0.919	1.117	0.951	0.950	1.007	0.963	0.994	1.073	1.017	1.023	1.046	Q96QG7	MTMR9	Myotubularin-related protein 9
1.046	1.054	1.040	1.005	1.031	1.106	1.127	0.989	0.982	1.153	1.031	1.147	P29966	MARCKS	Myristoylated alanine-rich C-kinase substrate
1.046	0.968	1.001	1.019	1.021	1.012	1.015	0.988	0.989	1.088	1.047	1.025	P20933	AGA	N(4)-(beta-N-acetylglucosaminyl)-L-asparaginase
0.984	0.991	0.976	0.979	0.972	0.983	0.991	0.943	0.996	1.012	0.979	1.014	O94760	DDAH1	N(G),N(G)-dimethylarginine dimethylaminohydrolase 1
1.003	0.992	0.994	0.988	0.987	0.983	0.971	0.958	0.983	0.982	0.969	1.007	O95865	DDAH2	N(G),N(G)-dimethylarginine dimethylaminohydrolase 2
1.031	0.976	1.001	0.993	0.931	1.007	1.017	1.026	1.040	0.985	0.946	1.030	Q86U44	METTL3	N6-adenosine-methyltransferase 70 kDa subunit
0.970	0.987	1.099	0.993	1.043	1.063	1.055	0.988	1.023	1.023	0.952	1.041	Q9HCE5	METTL14	N6-adenosine-methyltransferase subunit METTL14
0.974	0.985	0.976	0.986	0.983	1.022	0.999	1.015	1.021	1.025	0.987	1.045	O14745	SLC9A3R1	Na(+)/H(+) exchange regulatory cofactor NHE-RF1
1.003	0.880	1.078	1.033	0.976	1.011	1.004	0.991	0.987	1.032	0.984	1.041	Q15599	SLC9A3R2	Na(+)/H(+) exchange regulatory cofactor NHE-RF2
			1.014	0.978	0.975	1.027	0.880	0.973	1.209	1.062	1.067	Q5T2W1	PDZK1	Na(+)/H(+) exchange regulatory cofactor NHE-RF3
0.998	0.985	1.026	0.997	0.979	0.996	0.962	0.934	1.025	1.013	1.014	1.045	Q01415	GALK2	N-acetylgalactosamine kinase

Abundance Ratio														
1 d M1/C1	1 d M2/C2	1 d M3/C3	4 d M1/C1	4 d M2/C2	4 d M3/C3	7 d M1/C1	7 d M2/C2	7 d M3/C3	10 d M1/C1	10 d M2/C2	10 d M3/C3	Accession ID	Gene name	Protein Description
1.016	0.982	1.009	1.055	0.989	1.010	0.985	1.005	0.996	1.005	1.007	0.997	P34059	GALNS	N-acetylgalactosamine-6-sulfatase
1.134	1.050	0.978	0.930	0.925	1.079	0.922	0.935	0.810	1.054	0.877	0.838	E9PBY3	GALNT7	N-acetylgalactosaminyltransferase 7
0.996	1.002	1.009	0.983	0.897	0.979	1.001	0.959	0.951	0.980	0.972	0.977	Q86SF2	GALNT7	N-acetylgalactosaminyltransferase 7
1.005	1.012	1.211	0.977	1.089	1.167							Q9UK23	NAGPA	N-acetylglucosamine-1-phosphodiester alpha-N-acetylglucosaminidase
1.004	0.966	0.912	1.033	0.896	1.215	1.255	0.917	0.996	1.068	0.965	1.005	Q9UJJ9	GNPTG	N-acetylglucosamine-1-phosphotransferase subunit gamma
0.995	1.013	1.112	0.999	0.970	0.982				1.012	1.119	1.172	Q9Y303	AMDHD2	N-acetylglucosamine-6-phosphate deacetylase
1.002	1.009	1.003	1.015	1.053	1.038	1.072	1.070	1.063	1.094	1.082	1.015	P15586	GNS	N-acetylglucosamine-6-sulfatase
									0.932	1.067	0.793	Q9Y2B2	PIGL	N-acetylglucosaminyl-phosphatidylinositol de-N-acetylase
									0.897	0.768	1.048	Q9NY97	B3GNT2	N-acetyllactosaminide beta-1,3-N-acetylglucosaminyltransferase 2
1.024	0.973	1.021	1.037	1.101	1.011	1.021	0.932	0.979	1.051	1.044	1.125	O95671	ASMTL	N-acetylserotonin O-methyltransferase-like protein
0.981	0.863	0.882	0.980	0.900	1.051	0.979	1.156	0.940	1.085	1.098	1.155	Q8WUY8	NAT14	N-acetyltransferase 14
			1.041	0.806	1.131	1.133	1.143	1.133				J3KT72	NAT9	N-acetyltransferase 9
1.028	0.997	0.976	1.033	0.987	1.086	1.089	1.217	1.152	1.149	1.037	1.145	Q02083	NAAA	N-acylethanolamine-hydrolyzing acid amidase
1.018	0.997	1.049	0.999	0.993	1.017	0.978	1.022	0.974	0.994	0.980	0.989	Q8NFW8	CMAS	N-acylneuraminate cytidylyltransferase
0.976	0.970	1.036	0.926	0.997	1.171				1.068	0.964	1.081	Q8TBE9	NANP	N-acylneuraminate-9-phosphatase
1.035	0.978	1.006	0.994	1.011	1.009	1.000	0.935	1.020	1.029	1.021	1.034	Q4G0N4	NADK2	NAD kinase 2, mitochondrial
0.986	0.993	0.991	0.986	0.916	0.973	1.007	1.019	0.968	1.035	0.986	0.981	Q13423	NNT	NAD(P) transhydrogenase, mitochondrial
1.055	1.005	0.993	1.034	0.997	1.003	0.974	1.016	0.988	1.007	1.044	0.921	P15559	NQO1	NAD(P)H dehydrogenase [quinone] 1
0.984	0.984	1.003	0.990	0.981	0.995	0.994	1.077	1.008	0.967	0.997	0.968	Q8NCW5	NAXE	NAD(P)H-hydrate epimerase
1.013	0.980	1.012	1.010	0.996	0.992	1.028	1.004	1.009	0.998	0.996	0.992	P23368	ME2	NAD-dependent malic enzyme, mitochondrial
0.993	1.000	1.007	0.986	0.933	1.087	1.007	1.028	1.019	1.009	1.028	1.049	Q96EB6	SIRT1	NAD-dependent protein deacetylase sirtuin-1
1.069	1.026	1.030	1.046	0.951	1.023	1.030	1.126	1.001	0.971	1.017	0.983	A0A087WYM3	SIRT2	NAD-dependent protein deacetylase sirtuin-2
1.014	1.117	1.027	0.967	1.079	1.066	1.055	1.021	1.048	1.060	0.977	1.015	Q9NTG7	SIRT3	NAD-dependent protein deacetylase sirtuin-3, mitochondrial
0.958	0.860	1.005	1.032	0.980	0.957	0.981	1.153	0.985	1.082	0.883	1.154	Q8N6T7	SIRT6	NAD-dependent protein deacetylase sirtuin-6
1.036	1.032	0.981	0.746	0.712	1.606				0.946	1.024	1.206	Q9NRC8	SIRT7	NAD-dependent protein deacetylase sirtuin-7
1.007	0.969	1.062	1.024	1.133	0.957	1.087	1.024	0.957	1.106	0.987	1.018	Q9NXA8	SIRT5	NAD-dependent protein deacylase sirtuin-5, mitochondrial
1.090	0.961	1.026	1.026	1.088	1.107	0.991	1.067	1.121	0.996	0.976	1.041	Q330K2	NDUFAF6	NADH dehydrogenase (ubiquinone) complex I, assembly factor 6
0.978	1.006	1.017	1.012	0.957	1.017	1.008	1.053	0.966	1.064	1.015	1.049	Q9BU61	NDUFAF3	NADH dehydrogenase [ubiquinone] 1 alpha subcomplex assembly factor 3

Abundance Ratio														
1 d M1/C1	1 d M2/C2	1 d M3/C3	4 d M1/C1	4 d M2/C2	4 d M3/C3	7 d M1/C1	7 d M2/C2	7 d M3/C3	10 d M1/C1	10 d M2/C2	10 d M3/C3	Accession ID	Gene name	Protein Description
1.062	0.967	0.974	0.995	0.966	1.025	1.036	0.974	0.990	1.104	0.994	0.987	Q9P032	NDUFAF4	NADH dehydrogenase [ubiquinone] 1 alpha subcomplex assembly factor 4
									1.091	1.051	1.091	A1L188	NDUFAF8	NADH dehydrogenase [ubiquinone] 1 alpha subcomplex assembly factor 8
1.019	1.014	0.981	1.021	1.092	0.997	1.030	0.997	1.018	1.072	1.047	0.999	E7ESZ7	NDUFA10	NADH dehydrogenase [ubiquinone] 1 alpha subcomplex subunit 10, mitochondrial
1.021	0.991	0.949	0.996	0.970	0.981	0.990	1.093	0.959	1.042	1.028	0.966	Q9UI09	NDUFA12	NADH dehydrogenase [ubiquinone] 1 alpha subcomplex subunit 12
0.963	0.997	1.013	1.021	1.122	1.020	1.050	0.878	1.031	0.983	0.976	1.042	O43678	NDUFA2	NADH dehydrogenase [ubiquinone] 1 alpha subcomplex subunit 2
0.973	0.999	0.915	1.001	1.066	0.923	1.135	1.021	1.011	1.140	1.039	0.985	O95167	NDUFA3	NADH dehydrogenase [ubiquinone] 1 alpha subcomplex subunit 3
0.994	0.978	0.980	0.957	0.991	1.025	1.014	1.027	1.014	1.017	0.985	1.038	Q16718	NDUFA5	NADH dehydrogenase [ubiquinone] 1 alpha subcomplex subunit 5
1.011	0.973	1.004	0.931	0.944	1.038	0.938	1.004	0.996	0.990	0.952	1.023	P56556	NDUFA6	NADH dehydrogenase [ubiquinone] 1 alpha subcomplex subunit 6
1.069	0.984	1.015	1.012	1.039	0.991	0.993	0.898	0.943	1.065	1.021	0.986	O95182	NDUFA7	NADH dehydrogenase [ubiquinone] 1 alpha subcomplex subunit 7
0.905	0.995	0.842	1.027	0.961	1.007	0.977	1.005	1.088	1.032	0.995	0.974	P51970	NDUFA8	NADH dehydrogenase [ubiquinone] 1 alpha subcomplex subunit 8
1.037	1.009	1.009	0.998	0.984	1.027	1.025	1.087	0.999	1.034	0.967	0.974	Q16795	NDUFA9	NADH dehydrogenase [ubiquinone] 1 alpha subcomplex subunit 9, mitochondrial
0.988	1.041	1.035	0.995	1.075	1.004	1.008	1.036	1.031	1.123	0.994	1.100	O75438	NDUFB1	NADH dehydrogenase [ubiquinone] 1 beta subcomplex subunit 1
1.016	0.994	0.998	0.994	0.975	1.007	1.006	0.987	1.001	1.075	1.015	1.050	O96000	NDUFB10	NADH dehydrogenase [ubiquinone] 1 beta subcomplex subunit 10
1.209	0.861	1.329	0.696	1.023	1.039	0.867	0.602	0.855	1.079	1.041	1.308	O95178	NDUFB2	NADH dehydrogenase [ubiquinone] 1 beta subcomplex subunit 2, mitochondrial
0.990	0.951	0.969	0.945	0.953	0.949	1.009	1.003	0.996	1.010	0.960	0.991	O43676	NDUFB3	NADH dehydrogenase [ubiquinone] 1 beta subcomplex subunit 3
1.015	0.974	0.995	1.009	0.984	0.969	1.069	1.006	1.009	1.063	1.033	1.028	O95168	NDUFB4	NADH dehydrogenase [ubiquinone] 1 beta subcomplex subunit 4
0.992	0.969	0.994	1.021	0.945	1.035	1.048	0.952	1.003	1.102	0.990	1.006	H0Y886	NDUFB5	NADH dehydrogenase [ubiquinone] 1 beta subcomplex subunit 5, mitochondrial (Fragment)
0.965	0.978	0.987	0.999	1.019	1.017	1.119	0.971	0.978	1.092	1.030	0.983	O95139	NDUFB6	NADH dehydrogenase [ubiquinone] 1 beta subcomplex subunit 6
0.901	0.907	0.875	0.913	0.972	1.046	1.025	0.883	0.966	1.133	1.011	0.983	P17568	NDUFB7	NADH dehydrogenase [ubiquinone] 1 beta subcomplex subunit 7
1.010	0.990	0.965	1.010	0.884	0.999	1.057	1.046	0.937	1.087	1.013	0.961	O95169	NDUFB8	NADH dehydrogenase [ubiquinone] 1 beta subcomplex subunit 8, mitochondrial

Abundance Ratio												Accession ID	Gene name	Protein Description
1 d M1/C1	1 d M2/C2	1 d M3/C3	4 d M1/C1	4 d M2/C2	4 d M3/C3	7 d M1/C1	7 d M2/C2	7 d M3/C3	10 d M1/C1	10 d M2/C2	10 d M3/C3			
1.018	1.007	0.956	0.978	0.961	0.979	0.975	1.025	0.991	1.028	1.034	0.984	Q9Y6M9	NDUFB9	NADH dehydrogenase [ubiquinone] 1 beta subcomplex subunit 9
0.943	0.878	1.083	1.052	1.015	1.043	1.142	0.778	0.880	1.103	1.011	1.207	O43677	NDUFC1	NADH dehydrogenase [ubiquinone] 1 subunit C1, mitochondrial
1.022	1.006	0.991	1.028	1.013	1.003	1.043	1.015	0.986	1.167	1.019	0.972	O95298	NDUFC2	NADH dehydrogenase [ubiquinone] 1 subunit C2
1.030	1.004	1.000	0.999	0.978	1.001	1.028	1.023	1.001	1.042	0.989	1.013	P49821	NDUFV1	NADH dehydrogenase [ubiquinone] flavoprotein 1, mitochondrial
1.031	0.985	1.015	0.980	0.962	0.987	0.987	0.996	1.014	1.049	1.005	1.052	E7EPT4	NDUFV2	NADH dehydrogenase [ubiquinone] flavoprotein 2, mitochondrial
1.041	1.068	0.961	0.979	0.954	0.900	0.976	1.020	0.939	1.046	1.057	1.008	P56181	NDUFV3	NADH dehydrogenase [ubiquinone] flavoprotein 3, mitochondrial
1.026	0.999	1.005	1.010	0.962	0.954	1.014	1.054	0.982	1.029	0.994	1.009	O75306	NDUFS2	NADH dehydrogenase [ubiquinone] iron-sulfur protein 2, mitochondrial
1.042	0.997	1.002	1.007	0.967	0.983	1.009	1.019	1.000	1.025	0.992	1.008	O75489	NDUFS3	NADH dehydrogenase [ubiquinone] iron-sulfur protein 3, mitochondrial
0.998	0.990	0.969	0.981	0.974	1.009	1.027	0.996	0.996	1.098	1.025	1.061	O43181	NDUFS4	NADH dehydrogenase [ubiquinone] iron-sulfur protein 4, mitochondrial
1.055	0.841	0.966	1.010	0.920	1.010	1.058	1.005	1.009	1.084	0.754	1.049	O43920	NDUFS5	NADH dehydrogenase [ubiquinone] iron-sulfur protein 5
1.011	0.989	1.010	0.981	1.267	0.983	1.064	0.861	0.999	1.071	0.991	1.009	O75380	NDUFS6	NADH dehydrogenase [ubiquinone] iron-sulfur protein 6, mitochondrial
0.985	1.007	0.975	1.034	0.965	1.025	1.021	0.998	1.001	0.969	0.981	0.946	O75251	NDUFS7	NADH dehydrogenase [ubiquinone] iron-sulfur protein 7, mitochondrial
1.016	1.035	0.973	0.970	1.018	1.022	1.007	0.993	0.985	1.013	1.012	0.983	O00217	NDUFS8	NADH dehydrogenase [ubiquinone] iron-sulfur protein 8, mitochondrial
0.999	1.032	1.033	1.011	1.033	1.017	1.032	1.047	1.016	1.030	1.004	1.046	Q9UHQ9	CYB5R1	NADH-cytochrome b5 reductase 1
0.933	1.005	0.978	0.957	0.828	0.976	0.911	1.160	1.065	1.192	1.124	1.029	P03886	MT-ND1	NADH-ubiquinone oxidoreductase chain 1
0.892	0.904	0.934	0.948	0.708	1.032	0.994	1.009	0.910	1.167	1.196	1.051	P03891	MT-ND2	NADH-ubiquinone oxidoreductase chain 2
0.910	1.008	0.929	0.958	0.747	1.006	1.102	1.119	0.968	1.317	1.190	0.973	P03905	MT-ND4	NADH-ubiquinone oxidoreductase chain 4
0.885	0.979	0.956	0.851	0.786	1.028	1.003	1.193	1.109	1.221	1.102	0.919	P03915	MT-ND5	NADH-ubiquinone oxidoreductase chain 5
0.986	1.001	0.999	0.992	0.966	1.021	1.004	0.975	1.036	1.046	0.985	1.054	P48163	ME1	NADP-dependent malic enzyme
1.018	1.004	1.004	0.991	0.936	0.974	1.003	1.021	1.001	1.068	0.977	1.044	Q16798	ME3	NADP-dependent malic enzyme, mitochondrial
0.891	0.974	1.094	1.075	0.960	1.044				1.011	1.033	1.099	J3QQX3	FDXR	NADPH:adrenodoxin oxidoreductase, mitochondrial
0.986	1.004	0.999	1.011	0.941	0.970	1.014	1.050	0.984	1.012	1.023	0.982	P16435	POR	NADPH--cytochrome P450 reductase
1.026	0.995	0.948	1.001	0.988	1.018	1.034	1.019	1.000	1.052	1.057	1.013	P41227	NAA10	N-alpha-acetyltransferase 10
			1.009	1.321	1.183	1.063	0.824	1.148	0.923	1.108	1.037	Q9BXJ9	NAA15	N-alpha-acetyltransferase 15, NatA auxiliary subunit

Abundance Ratio														
1 d M1/C1	1 d M2/C2	1 d M3/C3	4 d M1/C1	4 d M2/C2	4 d M3/C3	7 d M1/C1	7 d M2/C2	7 d M3/C3	10 d M1/C1	10 d M2/C2	10 d M3/C3	Accession ID	Gene name	Protein Description
1.000	1.008	1.012	1.021	1.030	1.015	1.003	1.007	1.019	0.986	1.032	1.005	A0A0B4J1W3	NAA15	N-alpha-acetyltransferase 15, NatA auxiliary subunit
1.024	0.980	1.075	1.010	1.039	1.024	1.045	1.010	1.012	0.958	0.985	1.046	Q6N069	NAA16	N-alpha-acetyltransferase 16, NatA auxiliary subunit
1.212	0.882	1.053	1.084	0.858	1.041	1.000	1.028	1.064	1.028	0.996	1.023	P61599	NAA20	N-alpha-acetyltransferase 20
0.973	0.988	1.017	1.006	0.943	1.046	0.986	1.065	1.068	0.957	1.056	1.025	Q14CX7	NAA25	N-alpha-acetyltransferase 25, NatB auxiliary subunit
0.989	0.957	1.068	0.966	0.979	1.047	0.961	0.991	1.053	1.013	1.024	1.165	Q147X3	NAA30	N-alpha-acetyltransferase 30
0.955	0.976	1.002	1.010	0.939	1.041	1.026	1.001	1.020	0.993	1.012	1.053	Q5VZE5	NAA35	N-alpha-acetyltransferase 35, NatC auxiliary subunit
0.983	1.007	0.986	0.976	0.943	0.967	0.943	1.054	1.081	0.940	0.908	1.034	Q86UY6	NAA40	N-alpha-acetyltransferase 40
1.016	0.982	0.984	0.999	0.951	0.996	0.984	1.040	0.999	0.994	0.986	1.015	E7EQ69	NAA50	N-alpha-acetyltransferase 50
1.012	0.990	1.017	1.012	0.955	1.002	0.994	1.054	1.041	1.009	0.963	1.007	B1AKJ5	NRDC	Nardilysin
1.059	1.047	0.984	1.051	1.041	1.010	1.012	1.103	1.018	1.015	1.026	0.975	E9PAV3	NACA	Nascent polypeptide-associated complex subunit alpha, muscle-specific form
1.136	0.909	0.912	1.254	0.963	1.017	1.312	1.144	1.031	1.472	1.030	1.390	Q9H009	NACA2	Nascent polypeptide-associated complex subunit alpha-2
0.990	0.704	0.920	1.183	0.886	1.047				1.081	1.115	0.931	Q68D85	NCR3LG1	Natural cytotoxicity triggering receptor 3 ligand 1
1.096	1.025	1.071	0.862	0.865	1.048	1.046	1.058	1.041	1.092	0.939	0.903	P15882	CHN1	N-chimaerin
1.022	0.903	1.029	0.985	1.159	0.937	0.966	1.074	1.104				Q9HCH0	NCKAP5L	Nck-associated protein 5-like
0.933	0.940	1.047	0.923	0.889	1.030	1.008	0.926	1.026	0.982	0.874	1.123	Q9NZQ3	NCKIPSD	NCK-interacting protein with SH3 domain
1.013	1.060	0.871	1.106	0.965	0.873	1.112	1.197	0.916	1.204	1.056	1.113	A0A087X1N7	NEB	Nebulin
			0.945	1.031	1.036	1.058	1.052	0.949	0.969	0.860	0.947	Q15223	NECTIN1	Nectin-1
0.967	0.998	1.026	1.010	0.920	1.075	1.022	1.098	1.053	1.030	1.020	1.088	Q92692	NECTIN2	Nectin-2
0.880	1.118	0.974	0.959	1.112	1.167							Q9NQS3	NECTIN3	Nectin-3
0.966	0.991	0.949	0.972	0.952	1.011	1.041	1.032	1.032	1.051	1.005	1.104	O75113	N4BP1	NEDD4-binding protein 1
0.991	1.158	1.732				0.854	0.743	0.524				Q86UW6	N4BP2	NEDD4-binding protein 2
0.354	0.609	2.044	0.414	0.650	1.794	0.427	0.724	1.492	0.822	0.989	1.347	Q5TBK1	N4BP2L1	NEDD4-binding protein 2-like 1
1.079	1.116	1.207	0.967	0.973	0.927	1.147	0.703	0.925	1.266	1.238	1.074	D6R968	N4BP2L2	NEDD4-binding protein 2-like 2 (Fragment)
			1.092	0.876	1.151				0.975	0.937	0.950	Q9H0M0	WWP1	NEDD4-like E3 ubiquitin-protein ligase WWP1
0.997	1.032	1.031	1.097	0.959	1.061	0.993	1.132	1.076	0.952	0.968	1.108	O00308	WWP2	NEDD4-like E3 ubiquitin-protein ligase WWP2
0.953	0.983	0.975	0.956	0.950	1.049	0.946	0.906	1.008	1.042	0.984	1.054	Q15843	NEDD8	NEDD8
1.031	0.991	1.016	1.010	0.963	0.986	0.993	1.014	1.044	1.009	0.955	1.040	H3BM74	NUB1	NEDD8 ultimate buster 1
1.032	0.996	1.006	1.017	1.024	0.996	0.995	1.012	1.004	0.958	0.990	0.993	Q8TBC4	UBA3	NEDD8-activating enzyme E1 catalytic subunit
1.014	1.011	1.006	1.001	1.005	0.969	0.993	1.036	1.003	0.956	0.985	0.960	Q13564	NAE1	NEDD8-activating enzyme E1 regulatory subunit
1.033	0.970	1.022	0.972	1.109	1.041	0.993	0.845	1.015	1.028	1.070	1.018	P61081	UBE2M	NEDD8-conjugating enzyme Ubc12

Abundance Ratio														
1 d M1/C1	1 d M2/C2	1 d M3/C3	4 d M1/C1	4 d M2/C2	4 d M3/C3	7 d M1/C1	7 d M2/C2	7 d M3/C3	10 d M1/C1	10 d M2/C2	10 d M3/C3	Accession ID	Gene name	Protein Description
1.025	0.963	1.015	0.966	0.965	1.015	1.021	1.018	0.983	1.010	0.983	1.047	A0A0C4DFX9	NELFA	Negative elongation factor A
1.005	0.984	1.034	0.975	0.939	1.056	0.984	0.970	1.017	1.039	1.011	1.042	A0A0X1KG71	NELFB	Negative elongation factor B
0.993	1.002	1.029	0.986	1.015	1.053	1.006	1.101	1.058	0.981	0.967	1.045	H0UI80	TH1L	Negative elongation factor C/D
0.997	0.968	1.008	0.973	0.961	1.030	0.990	0.978	1.010	1.043	1.030	1.040	P18615	NELFE	Negative elongation factor E
1.048	0.974	1.049	1.069	0.852	1.025	0.872	0.734	0.911	0.972	1.001	1.038	A0A0U1RRE5	NBDY	Negative regulator of P-body association
1.196	0.808	0.893										Q92859	NEO1	Neogenin
1.046	1.054	1.037	0.917	0.837	1.011	1.057	0.966	1.050	1.078	1.094	0.967	Q7Z494	NPHP3	Nephrocystin-3
0.953	0.995	1.030	0.978	0.963	0.987	1.014	0.913	0.974	1.038	0.962	0.974	Q6UXI9	NPNT	Nephronectin
1.068	1.046	1.020	1.109	1.384	0.952							Q8NF91	SYNE1	Nesprin-1
1.153	1.183	1.076	1.016	0.963	1.037	0.933	1.094	1.109	0.940	1.035	1.210	O95631	NTN1	Netrin-1
1.022	1.001	0.996	0.973	0.998	0.985	1.025	1.054	1.011	1.069	1.049	1.004	Q9UMX5	NENF	Neudesin
0.898	1.016	0.971	0.956	0.846	0.992	0.975	0.951	1.050	0.943	0.956	1.101	Q9NPE2	NGRN	Neugrin
0.993	0.946	1.074	0.999	0.961	1.045	1.025	1.004	0.995	1.050	1.017	1.044	D3DTX6	PPP1R9B	Neurabin-2
0.985	0.983	1.033	1.010	1.005	0.999	1.025	0.984	1.058	1.031	1.013	1.104	O00401	WASL	Neural Wiskott-Aldrich syndrome protein
						0.962	0.969	1.106	1.128	1.012	0.952	Q96JN8	NEURL4	Neuralized-like protein 4
1.026	1.021	1.086	1.019	0.932	1.026	1.035	1.200	1.052	0.928	0.991	1.012	Q6ZS30	NBEAL1	Neurobeachin-like protein 1
0.993	1.002	1.050	0.980	0.950	0.885	1.082	1.297	1.005				Q6ZNJ1	NBEAL2	Neurobeachin-like protein 2
0.906	0.986	0.965	0.958	0.943	0.962	0.984	1.003	0.970	0.971	0.957	0.975	Q09666	AHNAK	Neuroblast differentiation-associated protein AHNAK
1.011	1.016	1.049	1.005	0.972	1.004	0.996	1.015	0.981	1.015	0.995	1.031	A2RRP1	NBAS	Neuroblastoma-amplified sequence
0.809	1.002	1.054	1.314	0.885	0.959							P29120	PCSK1	Neuroendocrine convertase 1
1.000	1.001	1.022	1.002	0.972	1.042	0.998	1.099	1.067	0.990	0.997	1.024	P21359	NF1	Neurofibromin
0.760	0.758	0.774				1.126	1.150	0.951	1.121	1.181	1.228	P46531	NOTCH1	Neurogenic locus notch homolog protein 1
1.036	0.978	1.009	1.023	0.984	1.047	0.998	1.072	0.968	1.060	1.007	1.069	Q04721	NOTCH2	Neurogenic locus notch homolog protein 2
0.953	0.934	0.935	0.881	1.010	0.967	1.028	0.826	0.913	1.117	0.860	1.061	Q92686	NRGN	Neurogranin
1.004	1.014	1.030	0.993	0.982	1.040	1.042	1.038	0.974	1.005	1.012	1.047	Q8NEJ9	NGDN	Neuroguidin
1.000	0.997	0.996	0.992	0.979	0.983	0.981	1.043	0.992	1.013	0.987	0.994	Q9BYT8	NLN	Neurolysin, mitochondrial
1.038	0.905	0.836										Q8NEY1	NAV1	Neuron navigator 1
						1.148	0.779	0.978	1.048	1.082	1.068	Q8IVL0	NAV3	Neuron navigator 3
0.987	0.937	1.324	1.008	0.953	1.205	1.000	1.044	1.400	1.013	1.039	1.341	P62166	NCS1	Neuronal calcium sensor 1
									1.215	1.270	1.011	Q9UH03	3-Sep	Neuronal-specific septin-3
									1.031	1.815	0.929	M0R2C2	PNPLA6	Neuropathy target esterase (Fragment)
0.838	0.984	0.948	0.943	0.862	0.946	1.008	0.974	0.927	1.015	0.929	1.019	M0R2H4	PNPLA6	Neuropathy target esterase (Fragment)
1.090	0.956	1.030	1.151	0.892	1.017	1.253	0.943	0.914	1.321	0.950	1.172	Q9Y639	NPTN	Neuroplastin

Abundance Ratio														
1 d M1/C1	1 d M2/C2	1 d M3/C3	4 d M1/C1	4 d M2/C2	4 d M3/C3	7 d M1/C1	7 d M2/C2	7 d M3/C3	10 d M1/C1	10 d M2/C2	10 d M3/C3	Accession ID	Gene name	Protein Description
1.003	1.011	1.008	0.992	0.978	0.997	1.011	1.008	0.996	1.019	0.982	1.009	Q14697	GANAB	Neutral alpha-glucosidase AB
1.089	1.035	0.970	1.015	0.978	1.019	1.022	0.981	1.009	1.111	0.921	1.038	Q8TET4	GANC	Neutral alpha-glucosidase C
0.980	1.013	1.025	1.048	0.992	1.111	1.058	1.096	1.036	1.032	0.997	1.135	Q15758	SLC1A5	Neutral amino acid transporter B(0)
1.136	1.113	1.077	1.069	0.924	0.976	1.052	1.171	0.966	0.934	0.870	1.085	X6R8F3	LCN2	Neutrophil gelatinase-associated lipocalin
			1.051	0.771	0.710							Q0ZGT2	NEXN	Nexilin
0.938	0.887	1.066	1.076	0.963	0.898	1.071	0.928	0.988	1.078	1.136	1.050	Q14596	NBR1	Next to BRCA1 gene 1 protein
0.998	0.980	0.990	0.982	0.933	0.994	1.009	1.167	1.039	0.958	0.908	1.006	Q8NCF5	NFATC2IP	NFATC2-interacting protein
1.205	1.162	1.157	1.044	1.074	0.847	0.891	0.959	1.061	0.924	1.233	0.777	P25963	NFKBIA	NF-kappa-B inhibitor alpha
0.999	0.989	1.084	0.994	0.995	0.977	0.822	0.743	1.027	1.001	1.028	1.102	Q15653	NFKBIB	NF-kappa-B inhibitor beta
2.330	0.623	0.790	0.940	0.975	0.922	0.944	1.019	1.132	0.933	1.149	1.073	O00221	NFKBIE	NF-kappa-B inhibitor epsilon
0.915	0.773	0.874	0.976	0.858	0.945	1.253	1.879	1.418				G5E9P3	NKIRAS1	NF-kappa-B inhibitor-interacting Ras-like protein 1
1.100	1.009	1.053	1.000	1.009	1.005	0.995	1.093	1.010	0.972	1.024	1.042	H7BXP1	NKIRAS2	NF-kappa-B inhibitor-interacting Ras-like protein 2
						0.933	1.037	1.429				Q9UBC1	NFKBIL1	NF-kappa-B inhibitor-like protein 1
0.967	0.900	1.020	0.930	1.014	1.174	1.218	0.955	1.022	1.286	0.947	1.231	Q8N5F7	NKAP	NF-kappa-B-activating protein
1.019	0.978	1.010	0.998	1.022	0.981	1.019	1.019	1.042	1.046	0.954	1.081	Q9UMS0	NFU1	NFU1 iron-sulfur cluster scaffold homolog, mitochondrial
0.909	1.003	1.015	0.947	0.957	1.027	1.003	0.860	0.979	1.076	1.008	1.056	Q6ZNB6	NFXL1	NF-X1-type zinc finger protein NFXL1
1.047	1.045	1.022	0.956	0.907	0.991	1.004	1.022	1.032	0.968	0.977	1.029	Q9P2E3	ZNFX1	NFX1-type zinc finger-containing protein 1
1.126	1.052	0.987	0.985	0.891	0.913	0.940	1.107	1.064	0.955	0.996	1.008	Q13506	NAB1	NGFI-A-binding protein 1
1.064	1.049	1.073	0.993	0.955	1.017	0.994	1.048	0.998	1.070	1.017	1.011	Q15742	NAB2	NGFI-A-binding protein 2
1.014	0.996	0.985	1.044	0.961	1.001	0.993	1.089	1.000	0.999	1.024	0.985	Q8NBF2	NHLRC2	NHL repeat-containing protein 2
1.032	0.966	0.971	0.955	0.915	0.938	0.998	0.948	0.951	1.001	0.974	0.966	Q5JS37	NHLRC3	NHL repeat-containing protein 3
1.040	1.016	1.024	1.068	1.420	0.974	0.995	0.893	1.051	0.941	1.016	0.903	B1AHD1	SNU13	NHP2-like protein 1
0.941	0.865	1.050	1.000	0.928	1.006	0.963	1.097	0.947	1.145	1.048	1.040	H0YDF6	NHSL1	NHS-like protein 1 (Fragment)
0.902	1.031	1.162	0.893	0.917	1.091	0.926	1.110	0.953	0.882	0.812	1.044	Q5SYE7	NHSL1	NHS-like protein 1
0.970	0.989	1.004	1.044	1.011	1.056	1.000	1.056	1.002	1.012	1.010	0.995	Q96TA1	FAM129B	Niban-like protein 1
1.022	1.003	1.010	1.006	0.967	0.996	1.015	1.054	0.985	1.005	1.011	1.021	O60934	NBN	Nibrin
0.990	0.986	1.016	0.995	0.945	0.999	0.993	1.014	0.976	1.012	1.003	0.985	Q969V3	NCLN	Nicalin
0.987	1.087	0.971	1.052	0.965	1.014							Q5T205	NCSTN	Nicastrin (Fragment)
0.995	1.025	1.017	1.095	1.048	1.142	1.118	1.198	1.114	1.117	1.073	1.097	Q92542	NCSTN	Nicastrin
1.044	1.005	0.989	1.067	1.261	0.996	1.104	0.963	1.098	1.032	1.037	0.985	P40261	NNMT	Nicotinamide N-methyltransferase
1.086	1.032	1.022	1.139	1.167	1.117	1.100	1.153	1.091	1.057	1.057	1.025	P43490	NAMPT	Nicotinamide phosphoribosyltransferase
1.214	1.111	1.018	0.961	0.961	0.956	1.014	1.125	1.082	0.952	1.093	0.901	Q9NWW6	NMRK1	Nicotinamide riboside kinase 1

Abundance Ratio														
1 d M1/C1	1 d M2/C2	1 d M3/C3	4 d M1/C1	4 d M2/C2	4 d M3/C3	7 d M1/C1	7 d M2/C2	7 d M3/C3	10 d M1/C1	10 d M2/C2	10 d M3/C3	Accession ID	Gene name	Protein Description
1.060	1.003	0.985	1.003	1.038	0.997	1.067	1.030	1.040	1.051	0.988	1.073	Q9HAN9	NMNAT1	Nicotinamide/nicotinic acid mononucleotide adenylyltransferase 1
1.018	1.023	1.011	1.001	1.010	1.002	1.003	1.018	1.020	1.049	0.987	1.032	Q6XQN6	NAPRT	Nicotinate phosphoribosyltransferase
0.913	1.011	1.028	1.034	1.071	1.022	1.053	0.964	0.995	1.084	0.933	1.103	Q15274	QPRT	Nicotinate-nucleotide pyrophosphorylase [carboxylating]
1.116	1.119	1.070	1.071	0.996	1.058	1.094	1.053	1.053	1.102	1.328	1.090	P14543	NID1	Nidogen-1
0.952	0.994	0.964	0.999	0.864	1.073	1.118	0.993	1.021	1.107	1.099	1.033	O15118	NPC1	Niemann-Pick C1 protein
1.022	0.988	1.017	0.986	1.011	1.013	0.986	1.040	1.010	0.994	0.978	1.003	Q9GZT8	NIF3L1	NIF3-like protein 1
1.007	1.001	1.032	1.007	0.960	1.015	1.014	0.993	1.029	1.004	0.980	1.075	Q6KC79	NIPBL	Nipped-B-like protein
1.070	1.028	1.023	0.997	0.955	1.092	1.037	1.084	1.019	1.015	0.992	1.013	Q9Y2I1	NISCH	Nischarin
1.038	1.019	1.028	0.988	0.946	0.967	1.026	1.057	1.014	0.997	0.966	1.012	Q9Y314	NOSIP	Nitric oxide synthase-interacting protein
0.995	0.961	1.035	0.963	0.863	0.984	0.923	0.891	0.987	0.957	0.886	1.000	Q8NC60	NOA1	Nitric oxide-associated protein 1
0.999	1.005	1.017	1.008	1.012	1.021	1.050	0.952	0.973	1.002	1.043	0.978	Q86X76	NIT1	Nitrilase homolog 1
			0.955	0.863	1.013				0.968	0.979	1.025	Q8WTW4	NPRL2	Nitrogen permease regulator 2-like protein
0.898	1.088	0.921	0.907	0.948	1.090	1.063	0.998	1.034	1.091	1.041	1.079	Q12980	NPRL3	Nitrogen permease regulator 3-like protein
0.989	1.074	1.051										Q9BZM4	ULBP3	NKG2D ligand 3
1.022	0.973	1.095	1.107	0.903	0.995	1.563	0.844	0.901	0.961	0.906	1.105	P30414	NKTR	NK-tumor recognition protein
0.989	0.988	1.105	0.973	0.912	1.130	1.044	1.103	1.065	1.092	1.006	1.182	Q86UT6	NLRX1	NLR family member X1
									0.953	1.246	1.336	Q8TBK2	SETD6	N-lysine methyltransferase SETD6
1.288	0.888	1.092	0.987	0.896	1.065	0.928	1.051	1.028				Q9NRG4	SMYD2	N-lysine methyltransferase SMYD2
1.008	1.000	1.025	1.045	1.014	1.077	1.064	1.069	1.079	1.084	0.966	1.028	Q9HBL8	NMRAL1	NmrA-like family domain-containing protein 1
0.992	1.018	1.007	0.979	0.943	0.968	0.970	0.978	0.981	1.021	0.957	1.057	Q13287	NMI	N-myc-interactor
			0.908	0.818	0.783				1.267	0.810	1.007	Q9UK39	NOCT	Nocturnin
0.962	1.037	0.934	0.963	0.946	0.957	1.068	0.992	1.044	1.136	1.039	1.094	A0A087X117	NOMO1	Nodal modulator 1
0.975	0.990	1.008	0.992	0.949	1.029	0.987	0.981	1.022	1.020	0.996	1.018	Q5JPE7	NOMO2	Nodal modulator 2
1.225	1.163	0.993	1.083	1.092	0.828				0.959	0.945	0.855	A0A0X1KG68	PAPD7	Non-canonical poly(A) RNA polymerase PAPD7
1.001	0.871	0.898	0.643	0.797	1.034	0.899	0.518	0.699	1.327	0.900	0.723	P05114	HMGN1	Non-histone chromosomal protein HMG-14
0.855	0.862	0.830	0.548	0.793	1.061	0.744	0.367	0.608	1.133	0.781	0.635	P05204	HMGN2	Non-histone chromosomal protein HMG-17
0.984	1.015	1.009	1.000	0.988	0.999	1.011	1.019	0.994	1.002	1.001	1.031	Q9H9Q4	NHEJ1	Non-homologous end-joining factor 1
						1.019	0.768	1.003	1.052	1.046	1.060	Q9HCG7	GBA2	Non-lysosomal glucosylceramidase
0.952	0.998	0.994	1.012	0.982	0.999	1.008	0.970	0.976	0.959	1.017	0.956	Q15233	NONO	Non-POU domain-containing octamer-binding protein
1.148	1.051	1.016	0.914	0.781	1.064							Q13470	TNK1	Non-receptor tyrosine-protein kinase TNK1
1.018	1.042	1.094	1.044	0.754	0.924	0.949	1.233	0.842	1.056	0.890	1.100	P29597	TYK2	Non-receptor tyrosine-protein kinase TYK2
0.965	1.010	0.967	0.988	0.981	0.961	0.974	0.982	0.972	0.944	0.944	0.941	P22307	SCP2	Non-specific lipid-transfer protein

Abundance Ratio														
1 d M1/C1	1 d M2/C2	1 d M3/C3	4 d M1/C1	4 d M2/C2	4 d M3/C3	7 d M1/C1	7 d M2/C2	7 d M3/C3	10 d M1/C1	10 d M2/C2	10 d M3/C3	Accession ID	Gene name	Protein Description
1.059	0.997	1.089	0.993	0.963	1.054	1.029	0.907	0.998	0.982	1.099	1.109	A0A0A0MT23	MARK3	Non-specific serine/threonine protein kinase
0.949	0.931	0.955	0.956	0.944	1.053	0.951	1.055	1.030	0.986	0.992	1.058	Q8WV22	NSMCE1	Non-structural maintenance of chromosomes element 1 homolog
1.053	0.979	1.066	0.997	0.960	1.008	1.015	0.995	0.989	1.023	0.971	1.074	Q96MG7	NSMCE3	Non-structural maintenance of chromosomes element 3 homolog
1.053	1.039	1.083	0.948	0.853	1.088	0.957	1.092	1.008	1.029	0.930	1.026	Q9NXX6	NSMCE4A	Non-structural maintenance of chromosomes element 4 homolog A
1.021	1.046	1.036	1.023	0.914	1.043	1.019	1.052	1.004	0.985	0.970	1.058	O60443	DFNA5	Non-syndromic hearing impairment protein 5
1.115	0.999	1.022	0.965	0.988	0.924	0.944	1.015	0.935	0.943	0.939	0.944	Q9C002	NMES1	Normal mucosa of esophagus-specific gene 1 protein
1.009	0.987	1.036	1.022	0.923	1.022	1.037	1.038	1.018	0.990	1.017	1.026	Q9NVX2	NLE1	Notchless protein homolog 1
1.019	0.996	1.004	0.991	0.987	0.992	0.991	0.979	0.990	0.956	0.968	1.013	Q9UNZ2	NSFL1C	NSFL1 cofactor p47
1.009	1.018	0.976	1.002	0.978	0.992	0.993	0.995	0.969	1.047	0.957	0.972	P51688	SGSH	N-sulphoglucosamine sulphohydrolase
1.239	1.189	1.112							1.132	0.958	1.071	Q96P71	NECAB3	N-terminal EF-hand calcium-binding protein 3
0.999	1.014	0.996	1.010	0.959	1.021	1.026	1.060	1.013	1.007	1.034	1.062	Q96KG9	SCYL1	N-terminal kinase-like protein
1.015	0.992	0.962	1.048	1.028	1.069	1.032	1.018	0.995	1.042	1.015	1.059	Q9BV86	NTMT1	N-terminal Xaa-Pro-Lys N-methyltransferase 1
1.068	0.989	1.013	0.933	1.094	1.013	1.089	0.707	1.207	1.074	1.055	1.202	Q9UKK6	NXT1	NTF2-related export protein 1
			0.942	1.145	1.040	0.922	1.031	0.986	0.773	0.846	1.056	Q9H093	NUAK2	NUAK family SNF1-like kinase 2
1.103	0.989	1.035	0.980	0.896	1.041	0.922	0.979	0.948	0.884	0.967	0.837	P23497	SP100	Nuclear autoantigen Sp-100
0.958	0.989	0.961	0.966	0.970	0.991	0.983	0.936	0.957	0.970	0.988	1.024	P49321	NASP	Nuclear autoantigenic sperm protein
1.008	0.983	1.040	0.964	0.961	1.036	1.008	1.011	1.025	0.985	1.014	0.973	Q09161	NCBP1	Nuclear cap-binding protein subunit 1
0.976	1.017	1.008	1.028	1.054	1.035	1.058	0.938	0.984	1.085	1.053	1.041	P52298	NCBP2	Nuclear cap-binding protein subunit 2
1.002	1.015	0.994	1.034	0.978	1.031	1.042	1.024	1.026	1.069	1.025	1.041	Q53F19	NCBP3	Nuclear cap-binding protein subunit 3
0.987	0.963	1.053	0.994	1.009	1.000	0.895	0.956	0.954	0.971	0.949	1.016	Q9NXR1	NDE1	Nuclear distribution protein nudE homolog 1
0.949	0.975	1.027	0.917	0.882	1.072	1.027	1.061	0.973	1.017	0.949	1.047	O14524	NEMP1	Nuclear envelope integral membrane protein 1
1.064	1.052	1.008	1.088	0.969	0.907	0.977	1.175	1.046	0.986	1.075	0.937	Q96HA1	POM121	Nuclear envelope pore membrane protein POM 121
1.053	1.007	1.017	1.024	0.999	0.991	1.020	0.964	0.970	0.977	1.088	1.006	A0A075B7F8	POM121C	Nuclear envelope pore membrane protein POM 121C
0.977	0.986	1.004	0.976	0.957	0.996	1.006	1.015	1.008	1.029	1.026	1.039	O60524	NEMF	Nuclear export mediator factor NEMF
1.015	1.001	0.977	0.999	0.925	0.986	0.980	1.023	1.048	1.069	1.055	1.054	P08651	NFIC	Nuclear factor 1 C-type
1.035	1.049	0.984	0.973	0.964	0.940	0.986	1.063	0.990	0.984	0.938	0.908	Q5VW26	NFIB	Nuclear factor 1
1.271	1.039	1.097	0.933	0.895	0.975				1.427	1.110	1.405	Q14938	NFIX	Nuclear factor 1 X-type
			0.941	0.864	1.288							Q16649	NFIL3	Nuclear factor interleukin-3-regulated protein
1.004	1.038	1.042	0.998	0.984	1.028	1.023	1.047	1.008	1.006	0.995	1.039	Q00653	NFKB2	Nuclear factor NF-kappa-B p100 subunit
1.042	1.076	1.036	0.877	0.920	1.080	1.021	1.120	1.060	0.972	1.105	1.030	O95644	NFATC1	Nuclear factor of activated T-cells, cytoplasmic 1

Abundance Ratio														
1 d M1/C1	1 d M2/C2	1 d M3/C3	4 d M1/C1	4 d M2/C2	4 d M3/C3	7 d M1/C1	7 d M2/C2	7 d M3/C3	10 d M1/C1	10 d M2/C2	10 d M3/C3	Accession ID	Gene name	Protein Description
1.019	1.010	1.025				0.967	1.069	0.826	1.858	0.345	0.953	Q9UHK0	NUFIP1	Nuclear fragile X mental retardation-interacting protein 1
1.019	1.067	1.089	0.993	0.992	1.010	0.987	0.953	1.011	0.983	1.045	1.047	Q7Z417	NUFIP2	Nuclear fragile X mental retardation-interacting protein 2
1.039	1.018	1.018	0.999	0.960	0.982	1.010	1.071	0.988	0.932	1.046	0.944	Q12972	PPP1R8	Nuclear inhibitor of protein phosphatase 1
0.966	0.999	0.992	0.988	0.966	0.981	0.980	0.973	0.961	0.973	0.989	0.990	Q9Y266	NUDC	Nuclear migration protein nudC
0.982	1.000	1.003	0.970	0.951	1.011	0.996	0.986	0.998	0.981	0.987	1.044	Q14980	NUMA1	Nuclear mitotic apparatus protein 1
1.104	0.997	1.025	0.976	0.946	1.101	1.020	1.070	1.066	0.988	0.995	1.047	J3KPZ4	C1D	Nuclear nucleic acid-binding protein C1D
1.002	0.999	1.024	0.985	0.949	1.019	0.974	1.042	1.006	0.935	0.999	0.971	P57740	NUP107	Nuclear pore complex protein Nup107
1.014	1.007	1.010	0.994	1.011	0.998	0.991	1.076	1.024	0.982	1.002	1.005	Q8WUM0	NUP133	Nuclear pore complex protein Nup133
1.008	1.001	0.995	1.015	0.985	1.023	1.014	1.053	1.000	0.977	0.976	0.986	O75694	NUP155	Nuclear pore complex protein Nup155
0.998	0.979	1.035	0.992	0.967	1.027	1.009	1.047	1.034	0.962	0.996	0.961	Q12769	NUP160	Nuclear pore complex protein Nup160
0.999	1.001	1.020	0.976	0.941	1.028	1.004	1.022	1.028	0.967	1.007	0.972	Q92621	NUP205	Nuclear pore complex protein Nup205
0.980	1.008	1.000	1.014	0.988	1.035	0.989	1.022	1.013	1.029	1.023	1.052	P35658	NUP214	Nuclear pore complex protein Nup214
0.999	1.002	1.015	1.015	0.968	0.968	1.017	1.054	0.988	0.984	1.003	1.000	Q9UKX7	NUP50	Nuclear pore complex protein Nup50
1.018	1.002	1.045	1.023	0.944	1.004	0.997	1.073	0.986	0.972	1.043	1.008	Q9BW27	NUP85	Nuclear pore complex protein Nup85
1.022	0.994	1.018	0.977	0.977	1.005	0.992	1.011	0.995	1.006	1.002	1.031	Q99567	NUP88	Nuclear pore complex protein Nup88
0.977	0.996	0.991	0.983	0.964	0.996	0.996	1.031	0.983	0.975	1.000	0.989	Q8N1F7	NUP93	Nuclear pore complex protein Nup93
1.003	1.016	0.977	1.002	0.988	1.005	1.013	1.036	0.996	1.009	1.011	1.001	P52948	NUP98	Nuclear pore complex protein Nup98-Nup96
1.031	0.999	1.030	0.962	0.962	1.045	0.982	1.045	1.014	0.959	1.002	1.054	P37198	NUP62	Nuclear pore glycoprotein p62
0.968	0.976	0.991	0.986	0.940	0.998	1.029	1.071	0.996	1.014	1.029	1.006	Q8TEM1	NUP210	Nuclear pore membrane glycoprotein 210
0.956	0.973	1.045	1.427	1.072	1.358							A0A088AWN8	NARF	Nuclear prelamin A recognition factor
1.082	1.051	1.108	1.025	1.319	1.076	1.033	0.963	0.914				Q15596	NCOA2	Nuclear receptor coactivator 2
0.954	0.973	1.031	0.953	1.022	1.051	1.009	0.997	1.004	0.973	0.977	1.127	Q9Y6Q9	NCOA3	Nuclear receptor coactivator 3
1.035	1.010	0.997	0.975	0.964	1.002	1.033	0.953	0.985	1.081	1.014	1.083	Q9HCD5	NCOA5	Nuclear receptor coactivator 5
1.103	1.059	0.999	0.986	0.948	1.015	0.981	1.016	1.079				Q14686	NCOA6	Nuclear receptor coactivator 6
1.015	0.986	1.000	0.999	0.918	1.007	1.019	0.965	1.005	1.042	1.000	1.087	O75376	NCOR1	Nuclear receptor corepressor 1
0.970	0.950	0.991	0.966	0.973	1.042	1.057	1.041	0.976	1.095	0.997	1.016	Q9Y618	NCOR2	Nuclear receptor corepressor 2
									1.097	1.025	1.562	P13056	NR2C1	Nuclear receptor subfamily 2 group C member 1
			0.819	1.007	0.855							P49116	NR2C2	Nuclear receptor subfamily 2 group C member 2
1.016	0.853	0.940	0.968	1.061	0.965	0.993	0.966	0.992	1.038	0.950	1.229	P10588	NR2F6	Nuclear receptor subfamily 2 group F member 6
			0.919	0.886	0.979	1.104	1.148	1.015	0.935	1.058	1.222	Q96F24	NRBF2	Nuclear receptor-binding factor 2
1.058	1.058	1.028	0.998	0.995	1.050	0.968	1.073	1.063	0.990	0.998	1.018	Q9NSY0	NRBP2	Nuclear receptor-binding protein 2
0.977	0.983	0.974	0.970	0.961	0.982	0.969	1.067	1.096	0.999	0.973	1.041	Q9UHY1	NRBP1	Nuclear receptor-binding protein
1.010	1.005	0.991	0.980	0.955	0.994	0.966	1.033	0.984	0.991	0.981	1.032	Q9UBU9	NXF1	Nuclear RNA export factor 1

Abundance Ratio														
1 d M1/C1	1 d M2/C2	1 d M3/C3	4 d M1/C1	4 d M2/C2	4 d M3/C3	7 d M1/C1	7 d M2/C2	7 d M3/C3	10 d M1/C1	10 d M2/C2	10 d M3/C3	Accession ID	Gene name	Protein Description
1.032	1.010	1.018	1.014	1.014	1.047	1.008	0.967	0.942	1.017	1.003	1.098	Q9H0G5	NSRP1	Nuclear speckle splicing regulatory protein 1
			1.018	0.849	1.079	0.968	1.138	0.851	0.878	0.957	0.964	P23511	NFYA	Nuclear transcription factor Y subunit alpha
1.006	0.990	0.992	1.015	0.945	0.988	1.027	1.061	0.955	0.932	1.052	0.968	P25208	NFYB	Nuclear transcription factor Y subunit beta
0.988	1.011	1.031	0.988	0.943	1.026	1.022	1.027	1.028	0.975	0.974	0.978	Q13952	NFYC	Nuclear transcription factor Y subunit gamma
0.933	0.999	0.966	0.986	1.022	1.003	0.970	1.041	1.007	0.963	1.003	0.952	P61970	NUTF2	Nuclear transport factor 2
0.986	0.881	0.916	0.730	0.968	1.036	0.876	0.598	0.835	1.144	0.900	0.825	Q9H1E3	NUCKS1	Nuclear ubiquitous casein and cyclin-dependent kinase substrate 1
1.057	1.015	1.006	0.991	0.914	1.007	1.011	1.091	1.127	0.987	0.989	1.045	O15381	NVL	Nuclear valosin-containing protein-like
0.968	0.999	1.029	0.955	1.015	1.000	0.935	0.913	1.028	0.965	1.009	1.080	Q86WB0	ZC3HC1	Nuclear-interacting partner of ALK
0.936	0.970	0.936	0.936	0.942	0.959	1.038	0.914	0.996	1.024	1.020	1.043	Q9Y2C4	EXOG	Nuclease EXOG, mitochondrial
1.055	1.047	0.994	1.005	0.972	0.948	1.026	0.981	0.963	1.027	1.090	1.083	P67809	YBX1	Nuclease-sensitive element-binding protein 1
1.029	1.054	1.012	1.024	0.955	1.027	1.075	0.983	1.029	1.008	0.988	1.219	Q13686	ALKBH1	Nucleic acid dioxygenase ALKBH1
0.974	1.000	1.018	0.990	0.989	1.025	1.021	1.033	1.036	1.037	0.988	1.031	A0A087WSV8	NUCB2	Nucleobindin 2, isoform CRA_b
0.963	0.967	0.988	1.000	0.981	0.992	0.961	0.906	0.978	1.024	1.029	0.992	Q02818	NUCB1	Nucleobindin-1
0.977	1.021	0.990	1.036	0.982	0.923	0.958	1.018	0.936	0.984	0.902	1.072	Q9BXS6	NUSAP1	Nucleolar and spindle-associated protein 1
0.988	0.985	1.012	0.966	0.980	1.024	1.004	0.914	0.992	0.998	1.035	1.038	Q9Y3T9	NOC2L	Nucleolar complex protein 2 homolog
0.979	0.981	0.988	0.988	0.954	1.004	0.978	0.974	0.992	1.006	1.035	1.018	Q8WTT2	NOC3L	Nucleolar complex protein 3 homolog
1.051	0.961	1.045	0.994	0.912	1.059	1.104	1.109	1.014	1.023	1.017	1.016	Q9BVI4	NOC4L	Nucleolar complex protein 4 homolog
0.987	0.993	0.989	1.002	0.972	1.040	1.019	1.041	1.029	1.012	1.026	1.030	Q9BZE4	GTPBP4	Nucleolar GTP-binding protein 1
1.021	0.975	1.056	1.021	1.027	1.070	1.000	0.934	1.003	1.121	1.060	1.082	Q13823	GNL2	Nucleolar GTP-binding protein 2
0.992	0.965	1.025	0.963	0.953	1.074	1.008	1.015	0.976	0.950	0.996	0.969	Q5C9Z4	NOM1	Nucleolar MIF4G domain-containing protein 1
1.013	0.988	1.021	0.973	0.956	1.047	0.994	1.082	1.070	1.034	1.021	1.044	O60287	URB1	Nucleolar pre-ribosomal-associated protein 1
1.053	1.000	1.021	1.023	0.950	1.019	0.976	1.031	1.020	0.999	0.986	0.927	Q9BSC4	NOL10	Nucleolar protein 10
0.995	1.019	1.026	0.988	0.951	0.979	1.002	1.036	1.009	0.961	1.013	0.979	Q9H8H0	NOL11	Nucleolar protein 11
0.965	0.945	0.893	1.286	0.997	1.141	1.031	1.059	1.188	1.170	1.044	1.032	Q9UGY1	NOL12	Nucleolar protein 12
1.003	0.996	1.015	0.980	0.954	1.052	1.008	0.993	0.989	1.012	1.054	0.999	P78316	NOP14	Nucleolar protein 14
1.007	0.992	0.983	1.030	0.977	1.035	1.014	1.044	1.020	1.026	1.062	0.990	Q9Y3C1	NOP16	Nucleolar protein 16
0.995	0.994	0.994	1.011	0.987	1.015	1.009	1.039	1.013	1.010	1.019	1.000	O00567	NOP56	Nucleolar protein 56
1.018	0.997	1.009	0.996	0.996	1.015	1.013	1.004	0.994	1.007	1.023	0.990	Q9Y2X3	NOP58	Nucleolar protein 58
1.025	0.993	1.036	1.009	1.010	1.043	1.038	1.061	1.052	0.984	1.009	1.029	Q9H6R4	NOL6	Nucleolar protein 6
1.003	1.015	1.006	0.963	0.962	0.984	0.971	1.005	0.985	1.009	1.043	1.012	Q9UMY1	NOL7	Nucleolar protein 7
0.969	0.985	1.028	0.994	0.995	1.017	0.983	0.961	0.977	1.006	0.966	1.057	Q76FK4	NOL8	Nucleolar protein 8
0.996	1.007	0.991	0.994	0.921	1.065	1.022	1.076	1.046	0.982	0.996	1.035	Q86U38	NOP9	Nucleolar protein 9
1.043	1.035	1.001	0.959	0.944	0.964	1.096	0.980	1.019	0.945	0.887	1.066	A0A087WXF8	ZCCHC17	Nucleolar protein of 40 kDa

Abundance Ratio														
1 d M1/C1	1 d M2/C2	1 d M3/C3	4 d M1/C1	4 d M2/C2	4 d M3/C3	7 d M1/C1	7 d M2/C2	7 d M3/C3	10 d M1/C1	10 d M2/C2	10 d M3/C3	Accession ID	Gene name	Protein Description
0.985	1.003	0.986	1.012	1.003	1.024	1.031	1.039	1.018	1.000	1.046	1.018	Q9NR30	DDX21	Nucleolar RNA helicase 2
0.968	0.995	0.997	0.975	0.965	1.002	0.972	0.952	0.982	0.995	1.024	1.010	P17480	UBTF	Nucleolar transcription factor 1
0.881	1.254	0.926	0.926	0.943	1.105	0.983	0.955	0.967	0.992	0.895	1.030	E9PKP7	UBTF	Nucleolar transcription factor 1
1.052	0.977	0.988	0.971	0.983	1.006	0.959	0.924	0.977	0.950	1.013	0.901	P19338	NCL	Nucleolin
1.030	1.009	1.081	1.089	0.945	1.012	1.034	1.147	0.944	1.101	1.109	1.176	Q6NW34	NEPRO	Nucleolus and neural progenitor protein
1.040	1.016	0.959	0.966	1.053	1.022	0.987	0.926	0.995	1.001	1.027	1.026	P31483	TIA1	Nucleolysin TIA-1 isoform p40
0.964	0.961	0.999	0.944	0.952	1.007	0.926	0.871	0.959	0.950	1.040	0.929	P06748	NPM1	Nucleophosmin
0.987	1.016	0.938	1.027	0.963	1.047	1.011	1.038	1.004	1.049	1.021	1.074	O75607	NPM3	Nucleoplasmin-3
0.978	0.963	1.000	1.013	0.969	1.042	1.016	1.055	1.058	1.001	0.997	1.063	Q53GS7	GLE1	Nucleoporin GLE1
0.979	1.023	0.997	1.005	0.928	0.945	1.007	1.081	0.947	0.993	1.025	0.973	Q9BTX1	NDC1	Nucleoporin NDC1
1.017	0.997	1.015	0.992	0.956	1.032	1.037	1.061	1.035	1.004	1.004	1.001	Q5SRE5	NUP188	Nucleoporin NUP188 homolog
1.009	0.968	1.035	0.998	1.038	1.030	0.985	1.037	1.042	0.952	0.959	0.986	Q8NFH4	NUP37	Nucleoporin Nup37
0.966	0.996	0.980	0.957	0.929	1.021	0.942	1.096	1.028	0.938	0.981	0.964	Q8NFH3	NUP43	Nucleoporin Nup43
1.000	1.017	0.991	0.996	0.950	0.956	0.976	0.998	0.953	0.959	1.039	0.945	Q8NFH5	NUP35	Nucleoporin NUP53
0.986	0.997	1.013	0.969	0.983	1.008	0.950	0.972	0.989	0.968	1.002	1.012	Q7Z3B4	NUP54	Nucleoporin p54
0.999	0.985	1.023	0.945	0.955	1.026	0.921	0.854	1.012	1.008	1.016	1.013	Q9BVL2	NUP58	Nucleoporin p58/p45
1.005	0.996	0.961	1.007	0.944	1.034	1.045	1.053	1.040	0.980	1.001	1.064	O15504	NUPL2	Nucleoporin-like protein 2
0.983	0.992	1.013	0.989	0.977	1.011	0.975	0.981	0.984	0.993	1.006	1.017	P12270	TPR	Nucleoprotein TPR
0.989	0.986	0.985	1.035	0.964	1.012	1.014	1.092	1.021	1.001	1.025	1.048	Q6DKJ4	NXN	Nucleoredoxin
0.987	0.982	1.010	1.000	0.935	1.051	1.009	1.047	1.053	1.066	1.031	1.102	Q13232	NME3	Nucleoside diphosphate kinase 3
1.053	1.051	1.037	1.037	0.997	1.040	1.029	1.141	1.031	1.074	1.016	1.124	Q9Y5B8	NME7	Nucleoside diphosphate kinase 7
1.016	1.017	0.989	1.009	1.022	0.991	0.992	1.071	1.003	0.990	1.016	0.974	Q32Q12	NME1-NME2	Nucleoside diphosphate kinase
0.950	0.913	0.913	0.873	0.890	0.883	0.926	0.872	0.863	0.895	0.887	0.886	F2Z2X0	NME4	Nucleoside diphosphate kinase
0.932	1.027	1.052	1.002	0.897	1.060	1.009	1.074	1.062	1.037	1.046	1.062	A0A0C4DG91	NME6	Nucleoside diphosphate kinase
1.088	0.942	1.052	1.000	0.972	1.010	1.024	1.038	1.034	1.001	0.958	1.021	A8MXV4	NUDT19	Nucleoside diphosphate-linked moiety X motif 19
						0.980	1.107	0.893				Q9BRQ3	NUDT22	Nucleoside diphosphate-linked moiety X motif 22
			1.024	0.988	1.021	1.022	1.031	0.936	0.931	1.018	1.092	Q8WV74	NUDT8	Nucleoside diphosphate-linked moiety X motif 8
0.991	0.999	0.980	0.969	1.015	0.958	1.005	1.071	1.031	0.957	0.993	0.977	P55209	NAP1L1	Nucleosome assembly protein 1-like 1
0.674	1.131	1.138	0.917	0.719	0.874	0.994	0.882	1.165	0.777	1.193	1.043	F8VV59	NAP1L1	Nucleosome assembly protein 1-like 1
0.981	1.006	1.026	0.992	0.974	0.988	0.957	0.999	1.006	1.002	1.015	1.023	Q12830	BPTF	Nucleosome-remodeling factor subunit BPTF
0.911	0.971	0.973	1.079	1.064	1.038	1.004	1.073	1.042	0.963	0.949	1.003	Q9H173	SIL1	Nucleotide exchange factor SIL1
1.010	1.075	1.079	1.091	0.908	1.025	1.090	1.058	1.059	1.029	1.006	1.024	Q9NV35	NUDT15	Nucleotide triphosphate diphosphatase NUDT15
0.964	0.996	1.045	0.928	0.965	1.107	1.083	0.980	1.018	1.011	0.993	1.155	Q96RE7	NACC1	Nucleus accumbens-associated protein 1
0.989	1.033	1.029	0.911	0.968	1.149	1.033	1.064	0.960	1.007	1.382	0.937	Q96BF6	NACC2	Nucleus accumbens-associated protein 2

Abundance Ratio														
1 d M1/C1	1 d M2/C2	1 d M3/C3	4 d M1/C1	4 d M2/C2	4 d M3/C3	7 d M1/C1	7 d M2/C2	7 d M3/C3	10 d M1/C1	10 d M2/C2	10 d M3/C3	Accession ID	Gene name	Protein Description
0.981	0.974	0.993	0.986	0.960	1.004	0.967	0.962	1.023	0.963	0.965	1.016	Q96RS6	NUDCD1	NudC domain-containing protein 1
1.063	1.001	0.993	0.984	1.020	0.981	0.992	0.928	0.978	1.053	0.993	1.060	Q8WVJ2	NUDCD2	NudC domain-containing protein 2
0.981	1.000	1.046	1.034	1.154	1.015	0.968	1.006	1.018	1.049	1.029	1.001	Q8IVD9	NUDCD3	NudC domain-containing protein 3
0.994	1.024	1.045	0.982	1.020	0.997	0.995	0.971	1.013	1.013	1.069	1.137	Q9Y6R0	NUMBL	Numb-like protein
1.021	0.989	0.926	1.023	0.991	0.980	1.067	1.103	0.983	0.920	0.946	0.960	Q8IXM6	NRM	Nurim
1.008	1.035	0.992	0.986	0.979	0.978	0.952	1.157	1.020	0.996	0.988	1.000	Q9Y530	OARD1	O-acetyl-ADP-ribose deacetylase 1
1.013	0.980	0.994	0.967	1.007	0.985	1.025	0.936	1.011	1.060	0.987	1.016	Q9BQ69	MACROD1	O-acetyl-ADP-ribose deacetylase MACROD1
1.054	0.968	1.083	0.861	0.894	0.990	0.980	1.144	0.902	1.188	0.990	0.861	A1Z1Q3	MACROD2	O-acetyl-ADP-ribose deacetylase MACROD2
0.969	0.995	0.969	0.999	1.033	0.988	0.988	1.025	1.028	1.000	0.993	0.996	J3KQ32	OLA1	Obg-like ATPase 1
0.952	0.919	0.974										O75147	OBSL1	Obscurin-like protein 1
1.045	1.013	1.025	1.107	0.966	0.969	1.123	0.893	1.071	1.119	0.945	1.141	A0A0G2JMZ8	OCLN	Occludin
			0.901	0.910	0.880	1.137	1.174	0.920	1.052	0.944	1.097	D6RDK6	OCIAD1	OCIA domain-containing protein 1 (Fragment)
1.023	0.991	1.026	0.998	0.970	0.960	1.058	1.021	0.975	1.049	1.003	1.076	Q9NX40	OCIAD1	OCIA domain-containing protein 1
1.082	0.991	1.026	0.998	1.018	0.986	1.017	0.847	0.983	1.077	0.894	1.077	Q56VL3	OCIAD2	OCIA domain-containing protein 2
0.889	1.056	0.942	1.157	1.136	1.127	1.071	1.152	1.073	0.928	1.013	0.955	Q8NGA1	OR1M1	Olfactory receptor 1M1
			1.186	0.802	0.967							Q8NGY6	OR6N2	Olfactory receptor 6N2
0.982	1.032	1.057	0.958	1.039	1.018	1.015	1.050	1.046	0.951	1.016	1.104	O60890	OPHN1	Oligophrenin-1
1.037	0.926	1.000	1.098	0.860	0.984							F5GYG5	REXO2	Oligoribonuclease, mitochondrial
1.004	0.975	0.960	1.004	0.968	1.014	0.991	1.025	0.981	1.022	1.009	0.989	Q9Y3B8	REXO2	Oligoribonuclease, mitochondrial
1.001	1.010	0.990	0.996	1.047	0.999	0.998	1.038	1.010	1.010	1.007	1.022	Q9NQR4	NIT2	Omega-amidase NIT2
1.021	1.082	1.016	1.070	0.983	0.941	1.036	1.198	1.039	1.079	0.922	1.112	Q99650	OSMR	Oncostatin-M-specific receptor subunit beta
1.019	1.013	1.148	1.060	0.965	1.098	0.938	1.037	0.959	1.012	1.003	1.038	Q9HD40	SEPSECS	O-phosphoseryl-tRNA(Sec) selenium transferase
1.010	1.011	1.032	0.980	0.950	0.997	0.985	0.958	1.009	0.966	0.982	1.025	Q9NZT2	OGFR	Opioid growth factor receptor
1.024	0.995	1.013	1.021	0.942	1.036	1.080	1.145	1.038	1.082	1.008	1.068	Q5TC84	OGFRL1	Opioid growth factor receptor-like protein 1
1.005	0.954	1.005	1.012	1.063	1.075	1.026	0.825	1.100	1.037	0.990	1.012	Q9H6K4	OPA3	Optic atrophy 3 protein
1.006	1.005	1.028	1.016	1.029	1.035	1.014	0.960	1.011	1.082	1.040	1.037	Q96CV9	OPTN	Optineurin
									0.949	0.895	0.991	O75665	OFD1	Oral-facial-digital syndrome 1 protein
1.008	1.036	1.136				0.983	1.165	1.044	0.858	0.882	1.052	Q13415	ORC1	Origin recognition complex subunit 1
1.021	0.994	0.977	1.022	0.951	0.986	1.057	1.059	0.989	1.037	1.039	1.063	Q13416	ORC2	Origin recognition complex subunit 2
0.981	0.986	1.025	1.030	0.946	1.027	1.013	1.044	1.008	0.938	0.945	0.986	Q9UBD5	ORC3	Origin recognition complex subunit 3
0.976	1.007	1.013	0.971	0.987	1.068	1.047	1.057	1.026	1.017	0.977	1.047	O43929	ORC4	Origin recognition complex subunit 4
1.029	1.007	1.007	1.036	0.920	1.126	1.049	1.100	1.000	0.979	1.057	0.994	O43913	ORC5	Origin recognition complex subunit 5
0.897	0.892	1.150	1.182	1.049	1.063	1.171	1.150	1.249				Q9Y5N6	ORC6	Origin recognition complex subunit 6
0.915	0.918	1.068	1.024	0.968	1.082	1.036	1.041	0.984	1.004	1.054	1.171	Q8N138	ORMDL3	ORM1-like protein 3

Abundance Ratio														
1 d M1/C1	1 d M2/C2	1 d M3/C3	4 d M1/C1	4 d M2/C2	4 d M3/C3	7 d M1/C1	7 d M2/C2	7 d M3/C3	10 d M1/C1	10 d M2/C2	10 d M3/C3	Accession ID	Gene name	Protein Description
0.987	0.994	0.971	0.980	0.945	0.954	0.971	1.037	0.970	0.951	0.906	0.955	P04181	OAT	Ornithine aminotransferase, mitochondrial
			0.940	0.853	1.052	1.201	1.119	1.081	1.021	0.939	1.278	P00480	OTC	Ornithine carbamoyltransferase, mitochondrial
1.011	1.020	1.009	0.968	0.977	0.990	0.917	0.970	1.003	0.959	0.990	1.001	Q92882	OSTF1	Osteoclast-stimulating factor 1
0.926	1.010	0.835	1.022	0.936	0.990	1.009	1.171	1.022	0.923	1.059	0.856	Q86WC4	OSTM1	Osteopetrosis-associated transmembrane protein 1
1.024	1.021	1.037	1.034	0.935	1.043	0.992	1.053	1.031	0.992	0.983	0.983	A0A087X0W9	OTUD6B	OTU domain containing 6B, isoform CRA_b
0.997	1.021	1.070	0.994	0.961	0.998	1.018	1.019	1.089	1.013	0.984	1.071	Q01804	OTUD4	OTU domain-containing protein 4
0.866	1.122	1.206	0.970	0.853	1.088	1.071	0.965	1.053	1.048	1.125	1.049	Q96G74	OTUD5	OTU domain-containing protein 5
0.969	1.011	0.993	1.051	0.930	1.063	1.011	0.989	0.930	1.015	1.008	1.093	Q6GQQ9	OTUD7B	OTU domain-containing protein 7B
1.066	1.015	1.057	1.049	1.127	1.033	0.937	0.916	1.088	0.945	1.030	1.172	Q5BJF6	ODF2	Outer dense fiber protein 2
1.029	0.971	0.992	1.033	0.984	0.989	1.060	0.985	0.987	1.013	1.045	0.954	Q8N573	OXR1	Oxidation resistance protein 1
						0.944	1.092	1.173				Q9UJX0	OSGIN1	Oxidative stress-induced growth inhibitor 1
0.855	0.842	1.134	1.144	0.980	1.073	1.004	1.178	1.077	1.017	0.971	1.131	C9JLB7	OXNAD1	Oxidoreductase NAD-binding domain-containing protein 1
0.905	0.951	1.080	1.152	1.052	1.220	1.061	0.980	1.137	1.134	1.089	1.136	Q5BKU9	OXLD1	Oxidoreductase-like domain-containing protein 1
1.003	1.003	0.989	0.986	0.968	0.987	1.024	1.019	1.005	1.032	0.981	1.030	P36551	CPOX	Oxygen-dependent coproporphyrinogen-III oxidase, mitochondrial
1.009	1.005	1.017	1.034	1.032	1.008	1.003	1.020	1.006	1.006	0.982	1.025	P22059	OSBP	Oxysterol-binding protein 1
			1.021	1.018	1.077							Q969R2	OSBP2	Oxysterol-binding protein 2
0.990	1.017	0.981	0.962	0.946	0.962	0.901	0.982	1.235	0.855	0.984	0.872	Q9BXW6	OSBPL1A	Oxysterol-binding protein-related protein 1
0.996	1.016	1.026	0.993	0.977	1.008	0.976	1.031	1.020	1.003	0.941	1.027	Q9BXB5	OSBPL10	Oxysterol-binding protein-related protein 10
1.105	1.045	1.024	1.057	1.027	0.886	1.054	1.043	0.786	0.999	1.003	1.051	Q9BXB4	OSBPL11	Oxysterol-binding protein-related protein 11
1.024	0.999	1.035	0.996	0.946	0.999	0.985	1.029	1.035	0.984	0.955	1.050	Q9H1P3	OSBPL2	Oxysterol-binding protein-related protein 2
1.003	0.998	1.000	0.955	0.993	1.041	1.015	0.996	1.019	0.975	0.969	1.087	Q9H4L5	OSBPL3	Oxysterol-binding protein-related protein 3
1.122	1.009	1.120	0.980	0.983	1.038				1.253	0.938	0.957	Q9H0X9	OSBPL5	Oxysterol-binding protein-related protein 5
						0.973	1.253	0.942				Q9BZF2	OSBPL7	Oxysterol-binding protein-related protein 7
0.983	1.017	1.015	1.000	0.941	0.996	1.008	1.058	1.024	0.984	1.009	0.962	Q9BZF1	OSBPL8	Oxysterol-binding protein-related protein 8
1.005	1.023	1.031	0.967	0.999	1.047	0.986	1.053	1.027	1.009	0.995	1.004	Q96SU4	OSBPL9	Oxysterol-binding protein-related protein 9
0.942	1.021	1.217	0.928	0.893	1.212	0.929	1.064	0.951				E9PLL4	NR1H3	Oxysterols receptor LXR-alpha
1.028	0.995	0.980	1.018	0.923	0.979	1.008	1.046	0.985	0.996	1.030	0.990	Q9NWT1	PAK1IP1	p21-activated protein kinase-interacting protein 1
						0.972	1.199	1.032				O15547	P2RX6	P2X purinoceptor 6
1.002	1.009	0.992	1.066	0.917	1.030	1.011	1.132	1.000	0.988	1.068	1.037	Q9NUG6	PDRG1	p53 and DNA damage-regulated protein 1
									1.418	0.902	1.343	Q96FX8	PERP	p53 apoptosis effector related to PMP-22
1.089	0.987	1.074	1.052	1.051	1.070	0.984	1.111	1.099	0.940	0.983	1.048	Q504Q3	PAN2	PAB-dependent poly(A)-specific ribonuclease subunit PAN2

Abundance Ratio														
1 d M1/C1	1 d M2/C2	1 d M3/C3	4 d M1/C1	4 d M2/C2	4 d M3/C3	7 d M1/C1	7 d M2/C2	7 d M3/C3	10 d M1/C1	10 d M2/C2	10 d M3/C3	Accession ID	Gene name	Protein Description
1.190	0.958	1.064	0.904	1.021	1.038	1.031	0.987	0.979	0.995	0.981	0.992	Q58A45	PAN3	PAB-dependent poly(A)-specific ribonuclease subunit PAN3
1.000	1.005	1.006	0.992	0.968	1.002	0.997	1.090	1.004	0.939	0.987	0.979	Q15645	TRIP13	Pachytene checkpoint protein 2 homolog
1.009	1.029	1.008	0.977	0.976	1.023	0.976	1.054	1.040	0.972	0.982	1.047	Q96ST3	SIN3A	Paired amphipathic helix protein Sin3a
			1.188	0.996	1.054							Q02962	PAX2	Paired box protein Pax-2
1.077	0.947	1.017	0.987	0.957	1.190	0.991	0.898	1.036	1.166	0.973	1.168	Q99453	PHOX2B	Paired mesoderm homeobox protein 2B
1.051	1.026	1.017	1.027	0.936	0.957	1.034	1.091	0.977	1.079	1.044	1.033	Q8WX93	PALLD	Palladin
1.074	1.020	0.973	1.125	1.097	1.042	1.198	1.265	1.131	1.288	1.280	1.133	P50897	PPT1	Palmitoyl-protein thioesterase 1
						0.971	1.159	0.712	1.151	0.924	1.199	F8W6M3	ZDHHC3	Palmitoyltransferase
0.727	1.023	0.981	1.465	1.005	1.102	1.088	1.014	0.990				Q8IUH4	ZDHHC13	Palmitoyltransferase ZDHHC13
									1.137	0.951	1.176	Q8IUH5	ZDHHC17	Palmitoyltransferase ZDHHC17
			1.217	1.017	0.884	1.220	1.070	1.084				Q9NUE0	ZDHHC18	Palmitoyltransferase ZDHHC18
			1.061	1.836	0.907	1.046	0.909	1.106	0.956	0.860	0.828	Q8IVQ6	ZDHHC21	Palmitoyltransferase ZDHHC21
1.077	0.985	1.022	1.012	0.939	1.036	1.137	0.978	1.010	1.168	1.008	1.103	Q9C0B5	ZDHHC5	Palmitoyltransferase ZDHHC5
1.030	0.838	1.120										X6R7I6	PPDPF	Pancreatic progenitor cell differentiation and proliferation factor
0.968	1.051	0.952	1.020	0.875	1.085	1.052	1.191	1.089	1.066	1.037	1.082	Q96RD7	PANX1	Pannexin-1
1.113	1.110	0.975	1.071	0.975	1.026	1.051	1.118	0.985	1.055	0.952	1.063	Q9BZ23	PANK2	Pantothenate kinase 2, mitochondrial
1.106	1.009	1.044	1.115	0.939	0.984	1.036	1.115	1.139	0.966	1.000	1.004	Q9H999	PANK3	Pantothenate kinase 3
1.007	1.012	1.013	1.012	0.945	0.988	0.974	1.084	1.005	0.969	0.972	1.013	Q9NVE7	PANK4	Pantothenate kinase 4
1.289	0.851	0.958	1.030	1.078	1.867	0.845	0.598	0.938				O95428	PAPLN	Papilin
1.023	0.983	1.006	0.993	0.970	1.026	1.023	1.065	1.006	0.999	1.007	1.041	Q6P1J9	CDC73	Parafibromin
0.994	0.885	1.148	0.908	0.964	1.021				1.517	1.490	0.897	O75781	PALM	Paralemmin-1
0.927	1.237	0.759										A6NDB9	PALM3	Paralemmin-3
1.458	1.050	1.002	1.195	1.250	1.338	1.800	1.060	0.874	1.127	0.893	1.125	Q8ND90	PNMA1	Paraneoplastic antigen Ma1
1.012	1.009	1.039	1.028	1.078	1.036	1.041	1.072	1.044	1.029	0.976	1.052	A0A0J9YXF2	PON2	Paraoxonase 2, isoform CRA_a
1.010	1.025	1.058	1.039	0.918	1.028	1.053	1.067	0.979	1.083	0.998	1.062	Q9UQ90	SPG7	Paraplegin
1.003	1.005	0.987	0.991	0.992	0.984	1.002	1.044	0.990	0.980	1.014	0.990	Q8WXF1	PSPC1	Paraspeckle component 1
1.046	0.973	0.968	0.861	0.919	1.012	1.018	0.859	0.885	1.149	1.030	1.031	P20962	PTMS	Parathymosin
0.964	1.014	0.988	0.993	0.941	1.046	1.059	1.037	1.025	1.048	0.999	1.110	Q8TEW0	PARD3	Partitioning defective 3 homolog
1.026	1.045	1.022	1.034	0.976	0.995	1.009	1.017	0.982	0.993	1.007	0.973	Q9BYG5	PARD6B	Partitioning defective 6 homolog beta
1.039	0.966	0.977	1.006	0.992	0.954	1.011	0.983	0.973	1.082	1.025	1.031	Q9BRP8	PYM1	Partner of Y14 and mago
1.063	1.019	1.027	0.990	0.931	0.939	0.943	1.070	0.949	0.956	0.977	0.990	Q96AD5	PNPLA2	Patatin-like phospholipase domain-containing protein 2
1.068	1.024	1.019	0.984	0.941	1.010	1.038	1.090	1.010	0.974	0.950	1.004	Q9Y5B6	PAXBP1	PAX3- and PAX7-binding protein 1

Abundance Ratio														
1 d M1/C1	1 d M2/C2	1 d M3/C3	4 d M1/C1	4 d M2/C2	4 d M3/C3	7 d M1/C1	7 d M2/C2	7 d M3/C3	10 d M1/C1	10 d M2/C2	10 d M3/C3	Accession ID	Gene name	Protein Description
0.930	0.971	0.971	0.954	1.067	0.994	0.955	0.746	0.976	0.947	0.984	1.025	A0A1B0GTU4	PXN	Paxillin
1.062	1.044	1.018	0.917	0.933	1.047	1.009	1.103	1.102	0.935	0.937	1.035	Q6ZW49	PAXIP1	PAX-interacting protein 1
1.009	0.987	1.144	0.988	1.120	1.139	1.097	0.923	1.114	1.204	1.017	1.284	Q9BTK6	PAGR1	PAXIP1-associated glutamate-rich protein 1
0.994	1.008	0.988	0.954	0.965	0.994	0.979	0.946	0.982	0.997	1.010	0.981	O75475	PSIP1	PC4 and SFRS1-interacting protein
0.995	0.969	1.016	0.991	0.991	1.081	1.031	0.985	1.013	0.988	1.052	1.060	Q5JVF3	PCID2	PCI domain-containing protein 2
1.061	1.002	1.007	1.023	0.956	0.959	1.012	1.073	1.023	1.081	0.961	1.060	Q9Y365	STARD10	PCTP-like protein
1.008	0.987	0.986	0.998	1.072	0.963	1.009	0.964	0.982	0.977	0.968	1.005	O00151	PDLIM1	PDZ and LIM domain protein 1
1.022	1.075	1.031	1.043	1.022	1.041	1.055	1.059	0.979	0.970	0.947	1.037	P50479	PDLIM4	PDZ and LIM domain protein 4
1.018	1.000	1.002	1.003	1.036	0.925	1.034	0.964	0.956	0.998	0.961	0.993	Q96HC4	PDLIM5	PDZ and LIM domain protein 5
									1.040	0.897	1.153	D6RH06	PDLIM7	PDZ and LIM domain protein 7 (Fragment)
0.788	0.852	1.089	0.753	1.316	1.114	0.924	0.494	1.114	1.099	1.137	1.342	D6RAN1	PDLIM7	PDZ and LIM domain protein 7 (Fragment)
1.021	1.007	1.007	0.974	1.037	0.973	1.037	0.925	1.006	0.971	0.942	1.026	Q9NR12	PDLIM7	PDZ and LIM domain protein 7
0.969	0.998	0.966	1.002	0.878	1.044	0.956	1.119	1.035	0.945	1.042	0.976	Q8NEN9	PDZD8	PDZ domain-containing protein 8
1.054	0.964	1.047	0.976	0.959	1.021	1.059	0.924	1.010	1.011	0.929	1.072	O14908	GIPC1	PDZ domain-containing protein GIPC1
0.958	0.961	1.188				0.985	1.094	1.010	1.064	0.946	1.135	Q13113	PDZK1IP1	PDZK1-interacting protein 1
1.049	0.933	1.068	0.947	0.918	1.027	0.974	1.262	1.075	0.926	0.922	0.999	Q9H6A9	PCNX3	Pecanex-like protein 3
1.077	1.032	1.022	1.058	1.138	1.049	0.947	1.019	0.954	0.920	1.009	0.919	Q9UBV8	PEF1	Peflin
1.016	0.994	1.025	0.965	0.967	1.023	0.995	1.005	0.983	0.985	0.994	1.002	Q96EY7	PTCD3	Pentatricopeptide repeat domain-containing protein 3, mitochondrial
0.993	0.974	0.957	1.035	0.995	1.007	1.012	0.861	0.812	1.057	0.981	1.002	Q8WV60	PTCD2	Pentatricopeptide repeat-containing protein 2, mitochondrial
						1.539	0.922	1.198				Q96A99	PTX4	Pentraxin-4
			0.986	0.846	1.347	0.902	0.910	0.972	1.029	1.024	1.022	P26022	PTX3	Pentraxin-related protein PTX3
0.970	0.991	1.003	0.993	0.956	1.002	1.072	1.004	0.974	0.998	0.949	1.068	Q8IYS1	PM20D2	Peptidase M20 domain-containing protein 2
0.991	0.941	0.984	1.172	1.059	1.046	1.169	1.036	0.961				O75570	MTRF1	Peptide chain release factor 1, mitochondrial
0.895	1.338	1.172	1.100	1.035	1.047	0.924	0.866	1.057	1.086	1.010	1.031	Q9UGC7	MTRF1L	Peptide chain release factor 1-like, mitochondrial
0.765	1.007	1.011	1.030	1.040	0.960	1.010	1.081	1.093	0.971	0.990	1.058	Q9HBH1	PDF	Peptide deformylase, mitochondrial
1.005	0.956	1.017	0.976	0.926	1.050	1.024	1.052	1.024	1.006	1.010	1.073	Q96IV0	NGLY1	Peptide-N(4)-(N-acetyl-beta-glucosaminyl)asparagine amidase
1.038	0.992	1.042	1.040	0.962	0.996	0.993	1.061	1.018	1.062	1.024	0.994	H7BZ14	PPIL3	Peptidyl-prolyl cis-trans isomerase (Fragment)
1.023	0.979	0.977	1.015	1.061	1.006	1.018	0.917	1.019	0.993	0.978	1.009	P62937	PPIA	Peptidyl-prolyl cis-trans isomerase A
1.058	0.992	1.010	1.075	1.038	0.990	1.037	0.955	1.006	0.982	1.013	0.961	P23284	PPIB	Peptidyl-prolyl cis-trans isomerase B
1.029	1.040	0.992	1.001	0.984	1.009	0.993	1.082	1.017	1.000	0.944	1.050	P45877	PPIC	Peptidyl-prolyl cis-trans isomerase C
0.997	0.971	1.007	0.993	0.946	0.974	0.993	1.013	0.994	0.997	1.002	1.046	Q6UX04	CWC27	Peptidyl-prolyl cis-trans isomerase CWC27 homolog

Abundance Ratio														
1 d M1/C1	1 d M2/C2	1 d M3/C3	4 d M1/C1	4 d M2/C2	4 d M3/C3	7 d M1/C1	7 d M2/C2	7 d M3/C3	10 d M1/C1	10 d M2/C2	10 d M3/C3	Accession ID	Gene name	Protein Description
0.999	0.987	0.992	0.999	0.957	0.998	0.999	1.063	1.018	0.994	0.962	1.006	Q08752	PPID	Peptidyl-prolyl cis-trans isomerase D
1.028	0.998	1.018	0.954	0.960	1.024	1.010	1.056	1.050	1.028	0.975	1.066	Q9UNP9	PPIE	Peptidyl-prolyl cis-trans isomerase E
1.017	0.986	0.962	1.022	0.972	1.007	0.997	1.034	0.941	0.976	0.945	0.947	P30405	PPIF	Peptidyl-prolyl cis-trans isomerase F, mitochondrial
0.990	1.013	0.971	1.024	1.019	1.034	1.017	1.040	0.994	1.040	1.021	1.016	Q96AY3	FKBP10	Peptidyl-prolyl cis-trans isomerase FKBP10
									0.908	0.954	0.958	H0YHM7	FKBP11	Peptidyl-prolyl cis-trans isomerase FKBP11 (Fragment)
1.001	0.951	1.013	0.977	0.967	0.986	1.012	1.121	0.975	0.990	0.965	0.967	Q9NYL4	FKBP11	Peptidyl-prolyl cis-trans isomerase FKBP11
1.069	0.935	1.000	0.962	0.880	0.988	0.959	1.082	0.975	0.981	1.004	0.979	Q9NWM8	FKBP14	Peptidyl-prolyl cis-trans isomerase FKBP14
0.970	0.994	1.020	0.984	0.931	0.994	0.945	0.953	0.997	0.932	0.972	0.983	P62942	FKBP1A	Peptidyl-prolyl cis-trans isomerase FKBP1A
1.026	0.986	1.027	0.982	1.044	0.992	0.975	1.041	1.025	0.971	1.019	0.987	P26885	FKBP2	Peptidyl-prolyl cis-trans isomerase FKBP2
1.019	0.981	0.978	0.960	0.976	0.968	0.945	0.921	0.957	0.974	0.971	0.962	Q00688	FKBP3	Peptidyl-prolyl cis-trans isomerase FKBP3
1.004	0.999	0.990	1.017	1.072	1.001	1.013	1.031	1.020	0.960	1.007	0.978	Q02790	FKBP4	Peptidyl-prolyl cis-trans isomerase FKBP4
0.987	1.001	0.974	1.018	1.032	1.000	0.997	1.008	0.983	0.988	1.001	0.990	Q13451	FKBP5	Peptidyl-prolyl cis-trans isomerase FKBP5
1.023	0.986	0.981	1.021	0.993	0.995	1.060	1.034	1.021	1.068	1.014	0.968	O95302	FKBP9	Peptidyl-prolyl cis-trans isomerase FKBP9
0.941	0.968	0.982	0.976	1.063	1.010	0.964	0.777	1.008	0.997	0.991	1.022	Q13427	PPIG	Peptidyl-prolyl cis-trans isomerase G
1.033	0.997	1.008	0.977	0.938	1.013	0.998	1.053	0.983	0.980	1.001	1.017	O43447	PPIH	Peptidyl-prolyl cis-trans isomerase H
1.011	0.984	0.953	1.003	1.061	1.026	1.027	0.992	1.043	1.027	0.991	1.062	Q13526	PIN1	Peptidyl-prolyl cis-trans isomerase NIMA-interacting 1
			1.095	1.717	0.805	0.955	1.075	1.003				F8WE65	PPIA	Peptidyl-prolyl cis-trans isomerase
1.010	1.007	0.969	1.045	1.112	0.978	1.024	0.966	0.992	1.037	1.069	1.035	Q9Y3C6	PPIL1	Peptidyl-prolyl cis-trans isomerase-like 1
0.990	1.022	1.019	1.009	0.970	1.009	1.010	1.009	0.980	1.073	0.951	1.067	Q13356	PPIL2	Peptidyl-prolyl cis-trans isomerase-like 2
1.050	0.961	1.005	0.984	0.967	1.003	1.043	0.977	1.050	0.994	1.008	1.020	Q8WUA2	PPIL4	Peptidyl-prolyl cis-trans isomerase-like 4
0.985	0.991	1.015	1.005	0.968	1.037	1.008	0.938	0.996	0.993	0.980	1.057	A0A0A0MT60	FKBP15	Peptidylprolyl isomerase (Fragment)
0.994	0.967	1.005	0.983	1.012	1.024	1.005	0.962	1.014	1.010	0.999	1.001	Q96BP3	PPWD1	Peptidylprolyl isomerase domain and WD repeat-containing protein 1
0.940	1.005	1.005	0.965	0.965	0.994	1.005	0.987	1.042	0.994	1.051	0.996	J3KQ48	PTRH2	Peptidyl-tRNA hydrolase 2, mitochondrial
0.974	1.004	0.983	1.006	1.046	0.989	1.017	0.947	1.024	1.091	1.004	1.088	Q14197	MRPL58	Peptidyl-tRNA hydrolase ICT1, mitochondrial
1.157	1.067	0.993	1.007	1.042	1.181	1.051	1.041	0.946	1.154	1.115	1.006	P55201	BRPF1	Peregrin
0.982	0.965	1.070	1.018	0.948	1.044	1.030	0.973	0.992	0.972	1.038	1.191	O95613	PCNT	Pericentrin
0.936	0.977	0.986	1.012	1.015	1.033	1.036	0.995	1.036	1.043	1.011	1.130	Q15154	PCM1	Pericentriolar material 1 protein
0.988	1.000	0.999	0.939	0.937	0.956	0.897	0.914	0.936	0.921	0.944	0.927	Q99541	PLIN2	Perilipin-2
0.993	1.067	1.068	0.974	0.971	0.912	1.041	0.946	0.960	1.039	1.015	1.207	O60664	PLIN3	Perilipin-3
1.060	1.018	1.028	1.002	1.029	0.938	1.037	1.038	1.067	0.978	0.998	1.030	Q13610	PWP1	Periodic tryptophan protein 1 homolog
1.033	0.966	1.024	0.993	0.977	1.051	1.006	1.063	1.043	1.016	1.014	1.009	Q15269	PWP2	Periodic tryptophan protein 2 homolog

Abundance Ratio												Accession ID	Gene name	Protein Description
1 d M1/C1	1 d M2/C2	1 d M3/C3	4 d M1/C1	4 d M2/C2	4 d M3/C3	7 d M1/C1	7 d M2/C2	7 d M3/C3	10 d M1/C1	10 d M2/C2	10 d M3/C3			
0.980	0.996	0.999	0.984	0.968	0.993	1.010	1.058	0.989	1.041	0.995	1.047	O14936	CASK	Peripheral plasma membrane protein CASK
1.078	1.040	0.968	1.001	0.999	0.980	1.008	1.013	0.954	1.066	1.067	1.006	F8W0Q9	PPHLN1	Periphilin-1
0.872	0.938	1.029	0.975	0.953	1.046	0.925	0.959	1.028	0.971	1.031	1.180	Q8NEY8	PPHLN1	Periphilin-1
0.981	0.935	0.994	0.942	0.901	0.947	0.942	0.831	0.957	1.064	0.970	1.016	O60437	PPL	Periplakin
0.981	1.001	0.954	0.996	1.013	0.975	1.012	1.007	1.015	0.994	0.981	0.976	Q06830	PRDX1	Peroxiredoxin-1
1.044	1.002	1.032	0.995	0.988	1.014	0.997	1.039	1.006	0.999	0.997	1.032	P32119	PRDX2	Peroxiredoxin-2
1.046	1.006	1.002	1.090	0.941	1.036	1.036	1.078	0.970	1.031	0.992	1.000	Q13162	PRDX4	Peroxiredoxin-4
1.029	0.996	0.997	1.004	1.034	1.011	1.011	1.068	1.066	1.003	0.996	1.008	P30044	PRDX5	Peroxiredoxin-5, mitochondrial
0.991	0.997	1.004	1.011	1.010	1.018	0.990	1.041	1.029	0.966	1.008	0.944	P30041	PRDX6	Peroxiredoxin-6
1.025	1.072	1.050	0.981	1.038	1.018	1.000	1.002	1.003	1.057	1.031	1.057	Q9NUI1	DECR2	Peroxisomal 2,4-dienoyl-CoA reductase
1.057	1.006	1.012	1.002	0.973	1.034	1.021	1.082	1.088	0.943	1.030	0.979	Q15067	ACOX1	Peroxisomal acyl-coenzyme A oxidase 1
1.492	0.954	0.959	1.319	0.898	1.004	1.267	1.004	1.018	1.163	0.931	1.030	O15254	ACOX3	Peroxisomal acyl-coenzyme A oxidase 3
1.022	0.988	1.034	0.996	0.981	0.979	1.001	1.046	1.010	1.026	0.980	1.041	Q08426	EHHADH	Peroxisomal bifunctional enzyme
1.091	1.042	1.059	0.986	0.965	1.010	0.978	1.008	0.990	1.050	1.020	1.040	P40855	PEX19	Peroxisomal biogenesis factor 19
0.984	0.935	1.013	0.989	0.867	1.008	0.991	1.096	1.017	0.975	1.009	1.015	P56589	PEX3	Peroxisomal biogenesis factor 3
			0.846	1.013	0.953							P0C024	NUDT7	Peroxisomal coenzyme A diphosphatase NUDT7
1.077	0.993	1.013	1.116	0.931	1.042	1.088	0.860	1.094				O75192	PEX11A	Peroxisomal membrane protein 11A
0.914	0.965	0.992	0.990	0.930	1.077	1.031	0.961	1.038	1.086	1.044	1.074	O96011	PEX11B	Peroxisomal membrane protein 11B
0.961	1.046	1.028	0.990	0.964	1.029	1.010	1.042	1.028	1.133	0.993	1.062	Q9NR77	PXMP2	Peroxisomal membrane protein 2
1.158	1.032	1.078	1.026	0.912	0.940	1.039	0.871	0.969	0.892	1.043	0.818	Q9Y6I8	PXMP4	Peroxisomal membrane protein 4
1.013	0.995	1.013	1.014	0.950	1.010				1.073	1.021	1.021	Q92968	PEX13	Peroxisomal membrane protein PEX13
0.978	1.004	0.999	0.940	0.964	0.997	0.983	0.972	0.980	1.049	0.987	1.041	O75381	PEX14	Peroxisomal membrane protein PEX14
1.012	0.963	1.056	1.017	0.894	0.952	1.044	1.103	0.985	1.076	1.130	0.945	O43808	SLC25A17	Peroxisomal membrane protein PMP34
0.984	0.997	0.989	0.991	0.984	0.982	0.994	0.997	0.978	0.961	0.938	0.989	P51659	HSD17B4	Peroxisomal multifunctional enzyme type 2
1.340	1.036	1.296	1.010	1.175	0.952	1.007	1.250	1.091	1.124	0.964	1.124	Q6QHF9	PAOX	Peroxisomal N(1)-acetyl-spermine/spermidine oxidase
1.031	1.003	1.022	0.978	1.003	1.002	1.055	0.887	0.997	1.067	1.087	1.064	Q9BQG2	NUDT12	Peroxisomal NADH pyrophosphatase NUDT12
0.989	0.968	1.012	1.046	0.961	1.055	1.073	1.049	1.049	1.150	0.968	1.096	B4E0T2	PEX5	Peroxisomal targeting signal 1 receptor
1.081	1.014	1.037	1.084	0.911	1.004	1.109	1.095	1.424	1.079	0.996	1.034	O00628	PEX7	Peroxisomal targeting signal 2 receptor
1.005	0.996	1.029	0.970	0.981	1.014	1.015	1.027	0.972	0.994	0.971	1.007	Q9BY49	PECR	Peroxisomal trans-2-enoyl-CoA reductase
						1.182	1.040	1.034				Q13608	PEX6	Peroxisome assembly factor 2
1.031	0.914	1.018	1.021	1.010	1.017	1.077	1.104	1.052	1.050	0.998	1.062	O43933	PEX1	Peroxisome biogenesis factor 1
			1.005	0.839	1.117							P28328	PEX2	Peroxisome biogenesis factor 2
1.156	0.987	1.049	1.036	1.308	0.999	1.066	0.931	1.130	1.004	1.019	1.001	M0QXB5	ETHE1	Persulfide dioxygenase ETHE1, mitochondrial

Abundance Ratio														
1 d M1/C1	1 d M2/C2	1 d M3/C3	4 d M1/C1	4 d M2/C2	4 d M3/C3	7 d M1/C1	7 d M2/C2	7 d M3/C3	10 d M1/C1	10 d M2/C2	10 d M3/C3	Accession ID	Gene name	Protein Description
1.022	1.004	1.044	1.013	1.012	1.041	1.021	0.935	1.027	1.034	1.026	1.032	O00541	PES1	Pescadillo homolog
1.015	0.983	0.949	0.962	0.966	1.005	0.967	0.959	0.973	1.008	1.014	0.990	Q8WW12	PCNP	PEST proteolytic signal-containing nuclear protein
0.959	0.966	1.084	0.990	0.942	1.017	1.094	1.007	1.055	1.119	1.019	1.108	Q9NYI0	PSD3	PH and SEC7 domain-containing protein 3
1.057	1.026	1.091	1.003	1.188	1.089	1.051	0.494	0.953	1.007	1.116	1.208	Q9P1Y6	PHRF1	PHD and RING finger domain-containing protein 1
1.081	1.025	0.949	0.954	0.972	1.040	0.973	1.034	1.019	1.029	0.983	1.024	O43189	PHF1	PHD finger protein 1
1.011	1.024	0.999	0.960	0.930	0.945	0.991	1.130	1.049	0.987	1.007	1.069	Q8WUB8	PHF10	PHD finger protein 10
0.962	1.272	0.952	0.987	0.872	0.924				1.262	1.050	1.274	Q96QT6	PHF12	PHD finger protein 12
0.983	0.998	0.998	0.959	0.932	1.015	1.015	0.987	0.957	0.978	1.003	0.981	A0A0U1RRH6	PHF14	PHD finger protein 14
									1.475	1.019	1.042	G3XAA4	JADE2	PHD finger protein 15, isoform CRA_d
1.266	2.802	1.154										Q9BVI0	PHF20	PHD finger protein 20
0.971	1.001	1.023	1.053	0.992	1.041	0.985	0.943	0.955	1.139	1.153	1.193	F8W9L8	PHF20L1	PHD finger protein 20-like protein 1
1.052	1.009	0.975	0.994	0.930	0.999	1.042	0.990	0.973	1.059	1.013	1.033	Q9BUL5	PHF23	PHD finger protein 23
1.008	0.979	1.055	0.996	0.951	1.020	0.983	0.914	1.005	1.067	1.006	1.028	Q92576	PHF3	PHD finger protein 3
0.993	0.960	0.966	0.959	0.983	0.982	1.014	0.911	0.981	1.041	0.993	1.094	A0A0D9SGE8	PHF6	PHD finger protein 6
1.027	0.979	0.960	0.989	1.174	0.993	1.065	0.912	0.991	1.051	1.053	1.004	Q7RTV0	PHF5A	PHD finger-like domain-containing protein 5A
1.121	0.768	0.830	0.907	1.042	1.121	0.833	1.078	1.094	0.937	0.942	1.012	P30039	PBLD	Phenazine biosynthesis-like domain-containing protein
0.987	0.987	1.005	0.998	0.990	1.016	0.992	1.002	1.026	1.004	1.011	1.036	K7ER00	FARSA	Phenylalanine--tRNA ligase alpha subunit
1.038	0.990	1.008	1.034	1.009	1.028	1.031	1.000	0.982	0.997	0.998	0.997	Q9NSD9	FARSB	Phenylalanine--tRNA ligase beta subunit
1.003	0.949	0.988	0.994	0.972	1.000	1.027	1.021	1.051	1.075	0.975	1.075	O95363	FARS2	Phenylalanine--tRNA ligase, mitochondrial
1.019	1.010	0.999	0.963	0.941	0.978	1.000	0.993	0.983	0.936	0.941	1.029	Q8WWQ0	PHIP	PH-interacting protein
			1.078	1.020	1.064	1.050	0.897	1.022	1.094	1.103	1.160	Q13794	PMAIP1	Phorbol-12-myristate-13-acetate-induced protein 1
1.039	0.988	1.011	1.004	0.976	1.048	0.999	1.116	1.065	0.969	1.065	1.027	Q9H2J4	PDCL3	Phosducin-like protein 3
1.002	1.026	0.987	1.017	0.962	0.972	0.977	1.133	0.997	0.958	0.986	0.986	Q13371	PDCL	Phosducin-like protein
0.952	1.041	1.016	0.978	0.970	1.075	1.038	0.994	1.000	1.194	1.043	1.111	O95674	CDS2	Phosphatidate cytidylyltransferase 2
1.085	0.941	0.967	1.191	1.553	1.029	1.068	0.930	1.147	1.124	1.046	1.019	Q9UKL6	PCTP	Phosphatidylcholine transfer protein
1.355	1.267	0.936										Q86VZ5	SGMS1	Phosphatidylcholine:ceramide cholinephosphotransferase 1
0.993	1.006	0.952	0.958	0.989	0.900	0.961	0.974	0.940	0.982	0.968	0.903	P30086	PEBP1	Phosphatidylethanolamine-binding protein 1
0.977	1.030	1.053	0.978	0.990	1.012	1.019	0.986	1.025	1.024	0.984	1.041	Q8WUK0	PTPMT1	Phosphatidylglycerophosphatase and protein-tyrosine phosphatase 1
0.967	0.997	1.001	0.982	0.929	0.977	0.947	1.040	0.969	0.981	0.993	0.972	Q9NTJ5	SACM1L	Phosphatidylinositide phosphatase SAC1
1.042	0.992	1.012	0.981	1.048	1.057	1.052	0.965	0.965	1.013	1.041	1.104	Q9Y2H2	INPP5F	Phosphatidylinositide phosphatase SAC2

Abundance Ratio														
1 d M1/C1	1 d M2/C2	1 d M3/C3	4 d M1/C1	4 d M2/C2	4 d M3/C3	7 d M1/C1	7 d M2/C2	7 d M3/C3	10 d M1/C1	10 d M2/C2	10 d M3/C3	Accession ID	Gene name	Protein Description
1.081	0.998	0.996	1.015	1.033	1.001	1.014	0.964	1.030				A0A0U1RR63	PTEN	Phosphatidylinositol 3,4,5-trisphosphate 3-phosphatase and dual-specificity protein phosphatase PTEN
1.021	0.983	1.043	0.987	0.930	1.010	0.986	1.049	0.995	1.040	0.977	1.055	O15357	INPPL1	Phosphatidylinositol 3,4,5-trisphosphate 5-phosphatase 2
0.999	0.984	1.005	0.974	0.956	1.037	0.997	1.044	0.997	0.988	0.973	1.055	Q8NEB9	PIK3C3	Phosphatidylinositol 3-kinase catalytic subunit type 3
1.051	1.016	1.000	1.041	0.984	1.054	0.974	1.034	1.037	1.031	0.966	1.055	O00459	PIK3R2	Phosphatidylinositol 3-kinase regulatory subunit beta
3.487	1.190	0.655										F6TDL0	PIK3R3	Phosphatidylinositol 3-kinase regulatory subunit gamma
1.000	0.998	0.977	0.934	0.939	1.095	0.934	1.156	1.055	0.903	0.932	1.020	P42336	PIK3CA	Phosphatidylinositol 4,5-bisphosphate 3-kinase catalytic subunit alpha isoform
0.999	1.139	1.130	1.016	0.893	0.932	0.876	1.174	1.070	1.008	1.017	1.064	P42338	PIK3CB	Phosphatidylinositol 4,5-bisphosphate 3-kinase catalytic subunit beta isoform
1.041	1.036	1.050	0.973	0.963	1.059	1.039	1.114	1.017	0.949	0.943	1.042	F8W9P4	PIK3CD	Phosphatidylinositol 4,5-bisphosphate 3-kinase catalytic subunit delta isoform
0.986	0.997	1.052	0.990	0.949	1.014	0.985	1.032	1.017	0.988	1.019	1.008	P42356	PI4KA	Phosphatidylinositol 4-kinase alpha
1.018	0.999	1.065	1.037	0.969	1.026	1.051	1.103	1.000	1.022	0.916	1.051	A0A0B4J1S8	PI4KB	Phosphatidylinositol 4-kinase beta
1.045	0.996	1.017	1.047	0.895	1.019	1.074	1.080	1.016	1.035	1.010	1.028	Q9BTU6	PI4K2A	Phosphatidylinositol 4-kinase type 2-alpha
1.155	1.015	1.180	1.027	0.966	1.103	1.015	1.203	1.033	0.857	0.995	1.298	Q8TCG2	PI4K2B	Phosphatidylinositol 4-kinase type 2-beta
1.004	0.963	1.044	1.005	0.940	1.033	0.997	1.085	0.998	0.999	0.940	1.034	O00443	PIK3C2A	Phosphatidylinositol 4-phosphate 3-kinase C2 domain-containing subunit alpha
						0.946	0.893	0.955				O00750	PIK3C2B	Phosphatidylinositol 4-phosphate 3-kinase C2 domain-containing subunit beta
0.974	0.979	1.038	0.959	0.958	0.953	0.965	1.191	0.992	0.908	1.020	0.916	Q99755	PIP5K1A	Phosphatidylinositol 4-phosphate 5-kinase type-1 alpha
0.959	0.979	1.053	1.011	0.952	1.068	1.037	0.990	1.053	1.057	1.042	1.066	P48426	PIP4K2A	Phosphatidylinositol 5-phosphate 4-kinase type-2 alpha
1.009	1.000	1.039	0.988	0.954	1.036	1.019	0.990	0.989	1.051	1.005	1.022	P78356	PIP4K2B	Phosphatidylinositol 5-phosphate 4-kinase type-2 beta
1.006	0.991	1.022	0.997	0.982	1.031	1.019	0.976	0.996	1.044	0.986	1.060	Q8TBX8	PIP4K2C	Phosphatidylinositol 5-phosphate 4-kinase type-2 gamma
0.881	0.955	1.065	0.970	0.878	1.086	1.051	1.009	1.116	1.069	0.967	1.023	Q9H490	PIGU	Phosphatidylinositol glycan anchor biosynthesis class U protein
						0.852	1.091	1.031	0.977	0.900	1.034	P37287	PIGA	Phosphatidylinositol N-acetylglucosaminyltransferase subunit A
1.022	1.002	0.992	0.988	1.098	1.010	1.039	0.934	1.008	1.026	1.011	1.042	Q00169	PITPNA	Phosphatidylinositol transfer protein alpha isoform

Abundance Ratio														
1 d M1/C1	1 d M2/C2	1 d M3/C3	4 d M1/C1	4 d M2/C2	4 d M3/C3	7 d M1/C1	7 d M2/C2	7 d M3/C3	10 d M1/C1	10 d M2/C2	10 d M3/C3	Accession ID	Gene name	Protein Description
1.194	0.993	1.023	0.980	1.040	0.741	0.812	1.234	0.939	0.817	1.028	0.961	H0YEH1	PICALM	Phosphatidylinositol-binding clathrin assembly protein (Fragment)
1.027	1.017	0.998	1.000	1.015	0.995	0.992	1.093	0.984	0.968	0.967	0.967	Q13492	PICALM	Phosphatidylinositol-binding clathrin assembly protein
			0.909	0.721	0.974				1.095	0.820	1.203	Q7Z7B1	PIGW	Phosphatidylinositol-glycan biosynthesis class W protein
0.986	0.976	0.989	0.995	0.891	0.965	1.020	0.981	0.980	1.034	0.956	1.056	Q9UG56	PISD	Phosphatidylserine decarboxylase proenzyme, mitochondrial
0.903	0.995	0.984	0.886	0.912	1.074	0.908	0.763	1.045	1.084	0.986	1.126	P48651	PTDSS1	Phosphatidylserine synthase 1
1.085	1.119	1.017	0.953	0.921	0.981	0.962	1.080	1.015	1.140	1.040	0.987	Q9BVG9	PTDSS2	Phosphatidylserine synthase 2
1.186	1.073	1.139	0.979	0.929	1.152	0.907	1.006	1.107	1.111	1.101	1.160	H0YM31	PCK2	Phosphoenolpyruvate carboxykinase [GTP], mitochondrial
1.016	0.992	1.013	1.017	1.020	1.015	1.008	0.997	1.040	1.000	0.982	1.008	Q16822	PCK2	Phosphoenolpyruvate carboxykinase [GTP], mitochondrial
1.018	0.991	0.994	1.028	0.987	1.009	1.026	1.013	1.014	1.049	1.000	1.056	Q6VY07	PACS1	Phosphofurin acidic cluster sorting protein 1
1.021	1.009	1.028	1.006	0.993	1.009	0.979	1.013	0.990	1.002	0.966	0.999	P36871	PGM1	Phosphoglucomutase-1
1.003	0.996	0.994	1.028	0.974	1.016	0.990	1.031	0.993	1.014	1.012	1.000	Q96G03	PGM2	Phosphoglucomutase-2
0.983	0.980	1.063	0.923	0.957	1.020	0.946	1.004	0.996	0.949	1.001	0.966	Q15124	PGM5	Phosphoglucomutase-like protein 5
1.006	1.005	0.982	0.997	1.036	1.003	0.968	1.004	1.000	0.923	0.999	0.904	P00558	PGK1	Phosphoglycerate kinase 1
0.982	0.999	1.020	0.960	0.991	1.086	0.947	0.916	1.021	1.063	0.959	1.018	P07205	PGK2	Phosphoglycerate kinase 2
0.985	0.984	1.000	0.975	1.084	0.985	0.937	0.858	0.989	0.961	0.998	0.988	P18669	PGAM1	Phosphoglycerate mutase 1
1.009	1.006	1.037	1.015	0.916	1.074	0.991	0.988	1.000	0.966	0.995	1.013	Q99570	PIK3R4	Phosphoinositide 3-kinase regulatory subunit 4
									1.040	0.962	1.025	Q53H76	PLA1A	Phospholipase A1 member A
0.984	0.995	1.013	1.009	1.015	0.995	1.014	1.003	1.000	0.994	0.996	1.002	Q9Y263	PLAA	Phospholipase A-2-activating protein
			0.939	0.881	0.967							Q6P1J6	PLB1	Phospholipase B1, membrane-associated
1.113	1.092	1.102	1.036	0.927	1.006	1.078	1.095	1.031	1.031	0.996	1.078	Q13393	PLD1	Phospholipase D1
0.990	0.984	0.999	0.966	0.902	1.024	0.995	1.050	1.036	1.027	0.944	1.072	O14939	PLD2	Phospholipase D2
1.019	1.068	1.061	1.111	1.117	1.178	1.065	1.145	1.093	1.071	0.982	1.116	Q8IV08	PLD3	Phospholipase D3
						1.016	0.993	1.108	0.992	0.872	1.001	Q8NEL9	DDHD1	Phospholipase DDHD1
0.997	0.987	1.027	0.992	1.004	0.978	1.018	1.043	1.027	0.980	1.075	1.019	O94830	DDHD2	Phospholipase DDHD2
0.896	0.981	0.909	1.139	0.824	0.905	1.084	1.851	0.946	1.382	1.250	1.297	O14494	PLPP1	Phospholipid phosphatase 1
0.932	1.021	0.969	1.004	0.889	1.147	1.050	0.992	1.109	1.056	1.050	1.081	O15162	PLSCR1	Phospholipid scramblase 1
0.990	0.815	1.069	1.133	0.933	1.230	1.238	0.996	0.858	1.115	1.085	1.169	Q9NRY6	PLSCR3	Phospholipid scramblase 3
1.011	0.961	1.032	0.978	0.875	1.046	1.007	0.964	1.041	1.014	1.025	1.076	Q8NB49	ATP11C	Phospholipid-transporting ATPase IG
0.993	1.042	0.877	0.882	0.946	1.107	0.925	1.049	1.207	1.097	1.069	1.025	E9PEJ6	ATP11A	Phospholipid-transporting ATPase

Abundance Ratio														
1 d M1/C1	1 d M2/C2	1 d M3/C3	4 d M1/C1	4 d M2/C2	4 d M3/C3	7 d M1/C1	7 d M2/C2	7 d M3/C3	10 d M1/C1	10 d M2/C2	10 d M3/C3	Accession ID	Gene name	Protein Description
1.121	0.925	1.056	1.048	0.945	1.023	0.984	1.065	1.026	0.959	1.000	0.939	Q9H008	LHPP	Phospholysine phosphohistidine inorganic pyrophosphate phosphatase
0.923	1.009	0.992	0.941	0.962	1.076	0.992	1.006	1.052	0.982	0.989	1.020	Q92871	PMM1	Phosphomannomutase 1
0.984	0.988	0.982	0.999	0.950	1.004	0.996	1.041	0.988	1.008	0.987	0.937	O15305	PMM2	Phosphomannomutase 2
1.013	1.004	1.011	1.014	0.986	1.024	1.018	1.041	1.007	1.026	0.983	1.012	Q15126	PMVK	Phosphomevalonate kinase
1.003	0.991	0.989	0.990	0.945	0.997	0.992	1.026	0.975	0.953	0.951	0.999	Q9HAB8	PPCS	Phosphopantothenate--cysteine ligase
0.923	1.013	1.048	1.001	0.932	1.014	1.017	1.103	1.098	0.958	0.990	1.051	Q96CD2	PPCDC	Phosphopantothenoylcysteine decarboxylase
1.013	1.011	1.023	0.985	0.953	0.999	0.969	1.054	1.010	0.976	0.972	1.011	O60256	PRPSAP2	Phosphoribosyl pyrophosphate synthase-associated protein 2
0.997	0.987	1.030	1.000	1.016	1.013	1.011	0.957	1.016	1.029	1.003	1.030	O15067	PFAS	Phosphoribosylformylglycinamidine synthase
1.094	0.978	1.053	1.009	0.911	1.053	1.010	1.005	1.029	0.993	0.905	1.109	Q9NRG1	PRTFDC1	Phosphoribosyltransferase domain-containing protein 1
1.011	1.006	1.029	0.982	0.950	1.124	0.980	1.112	0.943	1.070	0.978	0.980	J3KNN3	PHKG2	Phosphorylase b kinase gamma catalytic chain, liver/testis isoform
			0.992	0.891	1.058	0.900	1.001	1.134				P46019	PHKA2	Phosphorylase b kinase regulatory subunit alpha, liver isoform
0.946	1.024	0.963	0.947	1.055	1.045	1.029	1.108	1.006	0.990	1.038	1.051	A6NMN0	PHKA1	Phosphorylase b kinase regulatory subunit alpha, skeletal muscle isoform
1.031	1.022	1.055	1.051	1.009	1.025	1.041	1.004	1.030	0.983	1.042	1.012	Q9H814	PHAX	Phosphorylated adapter RNA export protein
1.037	1.002	1.046	1.015	1.046	1.115	0.957	1.176	1.053	1.038	1.015	1.026	Q9H4Z3	PCIF1	Phosphorylated CTD-interacting factor 1
0.966	0.991	0.974	0.969	1.003	0.938	0.991	0.983	0.965	0.952	0.969	0.970	Q9Y617	PSAT1	Phosphoserine aminotransferase
1.012	1.003	0.988	0.997	0.975	0.984	1.019	1.029	0.975	1.032	0.962	1.030	P78330	PSPH	Phosphoserine phosphatase
1.000	0.987	0.997	0.957	0.948	1.017	1.023	1.045	0.987	0.983	0.956	1.021	Q96BW5	PTER	Phosphotriesterase-related protein
0.993	0.993	1.003	0.999	1.009	1.021	1.023	1.000	1.026	1.052	1.026	1.077	Q6NYC8	PPP1R18	Phostensin
1.010	0.986	1.046	1.016	0.982	1.045	1.018	0.990	0.947	0.992	0.973	1.003	O14832	PHYH	Phytanoyl-CoA dioxygenase, peroxisomal
1.010	1.008	0.977	1.000	0.922	1.068	1.082	1.030	1.035	1.136	1.017	1.126	Q92508	PIEZO1	Piezo-type mechanosensitive ion channel component 1
0.994	0.986	1.038	0.981	0.974	1.004	0.990	1.040	0.983	0.956	0.944	1.028	Q9NWS0	PIH1D1	PIH1 domain-containing protein 1
0.970	0.996	1.193	0.954	1.007	0.957	0.961	0.994	1.093	1.192	1.010	1.472	Q96BK5	PINX1	PIN2/TERF1-interacting telomerase inhibitor 1
0.985	0.993	0.997	0.975	0.957	0.996	0.988	0.984	0.963	0.998	1.027	1.031	Q9H307	PNN	Pinin
0.971	0.978	1.022	0.970	1.018	1.046	1.008	1.056	1.063	1.009	1.022	1.043	O00625	PIR	Pirin
1.004	0.986	1.051	0.989	1.005	1.029	0.989	1.019	1.028	0.958	1.019	1.020	Q9GZP4	PITHD1	PITH domain-containing protein 1
0.935	1.028	1.099	1.070	0.949	1.094	1.137	0.959	1.025	1.328	1.259	1.287	P53801	PTTG1IP	Pituitary tumor-transforming gene 1 protein-interacting protein
1.059	1.016	0.999	1.046	0.995	1.000	1.079	1.020	1.018	1.080	0.983	1.069	A0A0D9SF60	PKP4	Plakophilin-4
1.046	0.977	1.065	1.018	1.028	1.044	1.062	1.122	1.050	1.094	1.005	1.067	Q9BTY2	FUCA2	Plasma alpha-L-fucosidase

Abundance Ratio														
1 d M1/C1	1 d M2/C2	1 d M3/C3	4 d M1/C1	4 d M2/C2	4 d M3/C3	7 d M1/C1	7 d M2/C2	7 d M3/C3	10 d M1/C1	10 d M2/C2	10 d M3/C3	Accession ID	Gene name	Protein Description
1.017	0.976	0.997	1.025	0.961	1.074	1.046	1.059	1.043	1.057	1.060	1.096	P20020	ATP2B1	Plasma membrane calcium-transporting ATPase 1
			0.837	0.781	1.212	1.150	1.140	1.125	0.860	0.980	1.063	P23634	ATP2B4	Plasma membrane calcium-transporting ATPase 4
1.085	1.026	1.048	1.052	1.017	1.090	1.069	1.230	0.976	0.979	1.074	1.027	P05121	SERPINE1	Plasminogen activator inhibitor 1
1.006	0.997	0.977	0.981	0.961	0.986	1.012	0.975	0.970	1.050	1.028	1.027	Q8NC51	SERBP1	Plasminogen activator inhibitor 1 RNA-binding protein
0.976	0.914	1.065	1.058	0.846	1.070	1.048	1.092	1.015	1.018	1.056	1.014	Q9HBL7	PLGRKT	Plasminogen receptor (KT)
0.651	1.016	0.703	0.983	0.822	1.117	1.323	0.687	0.822	1.339	1.049	1.008	Q9Y342	PLLP	Plasmolipin
0.985	0.997	0.991	0.975	0.965	0.973	0.997	1.013	1.021	0.972	0.944	1.029	Q14651	PLS1	Plastin-1
1.000	0.998	0.991	1.006	1.061	0.992	1.009	1.002	1.013	0.968	0.954	0.977	P13797	PLS3	Plastin-3
0.938	0.958	0.954	0.984	0.862	0.941	1.011	1.219	1.044	1.006	1.004	0.989	Q99487	PAFAH2	Platelet-activating factor acetylhydrolase 2, cytoplasmic
1.011	0.982	1.000	1.019	1.084	0.983	1.000	0.991	1.006	1.003	0.986	0.972	P43034	PAFAH1B1	Platelet-activating factor acetylhydrolase IB subunit alpha
1.032	1.048	0.992	0.939	0.960	1.082	0.920	1.010	1.011	0.922	1.000	0.944	P68402	PAFAH1B2	Platelet-activating factor acetylhydrolase IB subunit beta
1.034	0.984	1.036	1.002	1.094	0.991	0.985	0.932	1.022	1.040	0.976	1.015	Q15102	PAFAH1B3	Platelet-activating factor acetylhydrolase IB subunit gamma
									0.870	1.149	0.995	P04085	PDGFA	Platelet-derived growth factor subunit A
0.679	0.843	1.042	0.576	0.899	0.686	0.553	0.286	0.865	0.930	0.926	0.967	P01127	PDGFB	Platelet-derived growth factor subunit B
1.015	1.043	1.130	0.925	0.901	0.992	0.925	0.975	1.028	1.054	1.070	1.108	Q5RGS4	PLEKHA1	Pleckstrin homology domain-containing family A member 1
0.952	0.995	1.018	1.030	1.001	0.965	0.995	1.156	1.092	0.988	0.984	0.962	Q9HB20	PLEKHA3	Pleckstrin homology domain-containing family A member 3
						0.854	0.952	1.246	1.026	1.048	0.832	J3KQS5	PLEKHA8	Pleckstrin homology domain-containing family A member 8 (Fragment)
0.904	0.943	1.081	0.958	0.920	1.068	0.903	0.886	0.988	0.988	0.924	1.039	Q96S99	PLEKHF1	Pleckstrin homology domain-containing family F member 1
0.988	1.001	1.008	0.975	0.937	0.998	1.037	0.946	1.069	0.947	1.017	1.005	Q9H8W4	PLEKHF2	Pleckstrin homology domain-containing family F member 2
0.981	1.004	1.046	0.958	0.937	1.025	0.929	0.814	0.921	0.927	0.970	0.956	Q9H7P9	PLEKHG2	Pleckstrin homology domain-containing family G member 2
7.619	0.118	0.889	7.237	0.112	0.672	1.075	1.052	0.906				A1L390	PLEKHG3	Pleckstrin homology domain-containing family G member 3
						0.933	1.339	0.912				Q7Z736	PLEKHH3	Pleckstrin homology domain-containing family H member 3
			0.901	1.079	0.899	1.559	1.044	0.933	0.899	0.990	0.981	Q9Y4G2	PLEKHM1	Pleckstrin homology domain-containing family M member 1

Abundance Ratio														
1 d M1/C1	1 d M2/C2	1 d M3/C3	4 d M1/C1	4 d M2/C2	4 d M3/C3	7 d M1/C1	7 d M2/C2	7 d M3/C3	10 d M1/C1	10 d M2/C2	10 d M3/C3	Accession ID	Gene name	Protein Description
1.046	0.969	1.005	0.997	0.964	1.062	1.005	1.164	1.065	1.061	1.022	1.072	Q8IWE5	PLEKHM2	Pleckstrin homology domain-containing family M member 2
			1.073	1.034	1.050	1.162	1.042	1.111				Q494U1	PLEKHN1	Pleckstrin homology domain-containing family N member 1
0.972	1.107	1.060	0.928	0.994	1.008	1.039	0.968	0.989	0.919	0.990	1.057	Q8TD55	PLEKHO2	Pleckstrin homology domain-containing family O member 2
1.013	1.007	1.007	1.019	0.994	0.983	1.001	1.084	0.999	1.012	0.922	0.988	Q8WV24	PHLDA1	Pleckstrin homology-like domain family A member 1
1.019	1.053	1.022	1.087	0.934	0.979	1.087	1.149	1.013	0.932	1.107	1.098	Q53GA4	PHLDA2	Pleckstrin homology-like domain family A member 2
1.060	1.064	1.009	0.988	0.923	1.002	0.986	0.954	1.001	1.089	0.931	1.064	Q86SQ0	PHLDB2	Pleckstrin homology-like domain family B member 2
1.059	0.968	0.992	1.038	0.930	0.993	1.045	1.175	0.985	0.957	0.865	1.077	Q9NYT0	PLEK2	Pleckstrin-2
0.965	1.000	1.009	0.981	0.978	0.997	0.975	0.977	0.996	0.986	1.003	0.997	Q15149	PLEC	Plectin
0.984	0.995	1.004	0.993	1.015	1.030	1.021	1.030	0.987	1.014	1.007	1.024	O43660	PLRG1	Pleiotropic regulator 1
1.001	1.058	1.052	0.977	0.955	1.045	1.018	1.014	0.992	1.046	1.066	1.056	Q9UIW2	PLXNA1	Plexin-A1
0.996	0.915	0.952	1.032	1.060	1.027	1.219	0.843	1.007	1.337	0.860	1.089	O43157	PLXNB1	Plexin-B1
1.004	0.988	1.031	1.035	0.927	1.042	1.055	1.040	1.027	1.044	0.985	1.046	O15031	PLXNB2	Plexin-B2
1.055	1.017	1.014	0.990	0.899	0.920	0.938	1.090	0.921	0.931	0.976	0.980	P54277	PMS1	PMS1 protein homolog 1
0.979	0.958	0.868	0.811	1.028	1.187	1.106	0.798	1.099				Q8TC44	POC1B	POC1 centriolar protein homolog B
0.968	1.027	0.994	0.969	0.949	1.000	1.006	1.110	0.990	0.959	0.859	1.044	O00592	PODXL	Podocalyxin
0.931	1.019	1.125	0.961	0.912	1.000	0.967	0.908	1.043	0.952	1.053	1.065	Q9P215	POGK	Pogo transposable element with KRAB domain
1.027	1.010	1.020	0.977	0.941	0.981	0.992	1.014	0.983	1.012	1.010	1.018	Q7Z3K3	POGZ	Pogo transposable element with ZNF domain
0.874	0.968	0.977	1.007	0.934	0.989	1.057	1.121	0.961	1.107	1.048	1.063	A0A0C4DG49	PVR	Poliovirus receptor
0.987	1.014	0.997	1.002	1.056	0.989	1.002	0.957	0.994	1.001	0.976	1.001	P09874	PARP1	Poly [ADP-ribose] polymerase 1
1.040	1.069	1.035	1.003	0.962	0.991	1.003	1.064	0.986	1.021	0.998	1.050	Q9H0J9	PARP12	Poly [ADP-ribose] polymerase 12
0.994	0.998	1.025	0.918	0.929	1.003	0.918	1.026	1.003	1.065	1.028	1.032	Q460N5	PARP14	Poly [ADP-ribose] polymerase 14
0.972	0.919	0.967	1.031	0.977	0.984	1.007	1.060	0.928	0.892	1.017	1.001	Q9UGN5	PARP2	Poly [ADP-ribose] polymerase 2
1.028	1.022	1.028	1.016	0.962	1.010	1.026	1.006	1.027	1.025	0.966	1.058	Q9UKK3	PARP4	Poly [ADP-ribose] polymerase 4
1.009	0.968	1.111	1.055	0.996	0.947	1.042	1.117	0.987	1.044	1.040	1.002	Q8IXQ6	PARP9	Poly [ADP-ribose] polymerase 9
1.061	0.995	1.037	0.981	0.898	0.958	0.979	0.984	0.978	0.996	0.950	1.003	E9PNI7	PARP10	Poly [ADP-ribose] polymerase
1.021	0.968	1.025	0.966	0.956	1.020	0.987	1.082	1.048	0.948	0.955	1.039	P51003	PAPOLA	Poly(A) polymerase alpha
0.978	0.961	0.967	0.993	0.920	1.086	1.031	0.909	0.986	1.128	1.030	1.115	Q9BWT3	PAPOLG	Poly(A) polymerase gamma
1.007	0.886	1.065	0.820	0.944	0.912	1.079	1.127	1.024	0.988	1.235	1.007	Q6PIY7	PAPD4	Poly(A) RNA polymerase GLD2
0.959	0.991	0.992	1.024	0.976	1.071	1.072	0.966	1.031	1.106	1.069	1.078	Q9NVV4	MTPAP	Poly(A) RNA polymerase, mitochondrial
0.980	0.992	0.983	0.996	0.974	0.995	0.970	1.028	1.002	1.006	1.004	1.034	O95453	PARN	Poly(A)-specific ribonuclease PARN

Abundance Ratio														
1 d M1/C1	1 d M2/C2	1 d M3/C3	4 d M1/C1	4 d M2/C2	4 d M3/C3	7 d M1/C1	7 d M2/C2	7 d M3/C3	10 d M1/C1	10 d M2/C2	10 d M3/C3	Accession ID	Gene name	Protein Description
0.986	1.010	0.985	1.000	0.966	1.011	0.998	1.069	1.031	1.007	0.990	0.995	Q9NX46	ADPRHL2	Poly(ADP-ribose) glycohydrolase ARH3
1.072	1.027	1.042	1.019	0.993	1.020	1.016	1.046	1.063	0.983	0.996	1.010	Q86W56	PARG	Poly(ADP-ribose) glycohydrolase
0.961	0.999	0.964	0.989	1.024	0.986	1.029	0.965	0.992	0.997	1.015	0.994	Q15365	PCBP1	Poly(rC)-binding protein 1
									1.182	1.114	6.949	F8W0G4	PCBP2	Poly(rC)-binding protein 2 (Fragment)
0.932	0.713	0.919	1.025	0.967	0.978	1.182	1.161	1.011	0.993	1.005	1.017	Q15366	PCBP2	Poly(rC)-binding protein 2
0.946	0.958	1.170	0.899	0.901	1.046	1.019	0.858	0.946	1.139	0.996	1.139	P57723	PCBP4	Poly(rC)-binding protein 4
1.043	1.010	0.950	0.962	0.921	1.028	1.088	1.281	1.037	0.904	0.923	0.985	H0YAR2	PABPC1	Polyadenylate-binding protein 1 (Fragment)
0.858	0.826	0.946	0.794	0.893	1.194	0.986	1.083	1.152	0.957	1.037	1.108	E5RJM8	PABPC1	Polyadenylate-binding protein 1 (Fragment)
1.006	1.020	0.980	1.016	1.067	0.970	1.005	1.010	1.018	0.982	1.035	0.976	P11940	PABPC1	Polyadenylate-binding protein 1
0.903	1.046	1.210	0.959	0.944	0.787	1.007	1.059	1.100	0.799	1.005	1.209	Q86U42	PABPN1	Polyadenylate-binding protein 2
1.028	0.980	1.005	0.948	0.998	1.005	0.971	0.948	1.037	1.023	1.009	1.042	Q9H074	PAIP1	Polyadenylate-binding protein-interacting protein 1
1.038	0.944	1.005	0.989	0.994	1.057	0.941	0.873	1.037	1.100	0.924	1.166	Q9BPZ3	PAIP2	Polyadenylate-binding protein-interacting protein 2
1.069	1.036	1.021	1.040	0.978	0.994	0.979	0.965	0.977	0.955	0.896	0.991	Q6P1K2	PMF1	Polyamine-modulated factor 1
1.031	0.982	1.009	0.965	0.933	1.021	0.922	1.086	0.895	0.948	0.956	1.035	P35227	PCGF2	Polycomb group RING finger protein 2
			0.781	0.873	1.189				0.934	0.904	1.030	Q86SE9	PCGF5	Polycomb group RING finger protein 5
									0.908	0.904	1.139	Q9BYE7	PCGF6	Polycomb group RING finger protein 6
1.055	1.030	0.983	1.015	1.006	1.015	1.044	1.065	0.961	1.135	0.973	1.016	Q15022	SUZ12	Polycomb protein SUZ12
0.986	0.921	1.205	0.959	0.953	1.110	0.918	0.881	1.046	1.048	1.065	1.006	Q13563	PKD2	Polycystin-2
1.005	0.979	0.986	0.976	0.970	0.965	0.950	1.009	0.969	0.962	0.978	0.952	O60828	PQBP1	Polyglutamine-binding protein 1
1.118	1.076	1.009	0.920	1.014	0.853	1.124	1.044	0.977	0.821	1.194	1.110	P78364	PHC1	Polyhomeotic-like protein 1
1.005	1.011	1.043	1.000	0.975	1.059	0.975	1.057	1.040	0.999	1.009	1.015	Q9Y2S7	POLDIP2	Polymerase delta-interacting protein 2
1.013	0.983	1.004	1.000	0.979	0.998	1.020	1.004	0.973	1.032	1.015	1.033	Q9BY77	POLDIP3	Polymerase delta-interacting protein 3
0.923	0.994	0.982	0.954	0.967	0.987	0.988	0.994	0.999	0.975	0.917	1.025	Q6NZI2	PTRF	Polymerase I and transcript release factor
1.022	1.017	1.008	1.002	0.942	0.956	0.991	1.044	0.996	0.973	1.010	0.967	Q5SY16	NOL9	Polynucleotide 5'-hydroxyl-kinase NOL9
0.894	0.900	0.907	0.859	0.770	0.822	0.973	1.084	0.910	1.057	1.019	1.038	Q10472	GALNT1	Polypeptide N-acetylgalactosaminyltransferase 1
0.979	1.008	1.050	1.023	0.928	1.093	1.048	1.114	1.007	0.995	1.121	0.990	Q8NCW6	GALNT11	Polypeptide N-acetylgalactosaminyltransferase 11
0.976	0.988	1.099	0.960	0.970	1.056	1.043	0.968	1.126	1.042	0.973	1.087	Q96FL9	GALNT14	Polypeptide N-acetylgalactosaminyltransferase 14
0.976	1.013	1.004	1.050	0.962	1.000	1.042	1.051	1.034	1.069	0.993	1.059	Q10471	GALNT2	Polypeptide N-acetylgalactosaminyltransferase 2
0.992	0.890	1.010	0.970	0.912	0.957	1.041	1.072	0.957	1.151	1.010	0.985	Q8N4A0	GALNT4	Polypeptide N-acetylgalactosaminyltransferase 4
0.741	0.884	0.922	0.921	1.144	1.145	1.002	0.925	0.793				J3KNN1	GALNT9	Polypeptide N-acetylgalactosaminyltransferase
0.985	1.019	0.947	0.998	0.961	1.012	0.924	1.105	0.922	1.026	0.965	1.029	Q92562	FIG4	Polyphosphoinositide phosphatase

Abundance Ratio														
1 d M1/C1	1 d M2/C2	1 d M3/C3	4 d M1/C1	4 d M2/C2	4 d M3/C3	7 d M1/C1	7 d M2/C2	7 d M3/C3	10 d M1/C1	10 d M2/C2	10 d M3/C3	Accession ID	Gene name	Protein Description
						1.178	1.000	1.047				Q9H8P0	SRD5A3	Polyprenol reductase
1.034	1.034	1.023	0.985	1.003	1.035	0.963	1.042	1.016	0.938	1.012	0.960	A0A0U1RRM4	PTBP1	Polypyrimidine tract-binding protein 1
1.056	0.980	1.022	1.042	0.979	0.993	1.012	0.996	1.002	0.981	1.077	0.985	E9PL17	CLP1	Polyribonucleotide 5'-hydroxyl-kinase Clp1
1.002	1.008	1.009	0.994	0.979	0.999	1.010	1.011	0.992	1.011	1.005	1.012	Q8TCS8	PNPT1	Polyribonucleotide nucleotidyltransferase 1, mitochondrial
1.211	1.114	1.071	1.002	1.167	1.040	1.048	1.150	0.973	1.078	1.160	1.148	F5H265	UBC	Polyubiquitin-C (Fragment)
1.050	0.995	1.019	1.019	0.910	1.006	1.007	1.026	0.994	0.996	0.990	1.018	P08397	HMBS	Porphobilinogen deaminase
1.033	0.976	0.840	1.010	0.847	1.002	1.001	1.189	0.975				Q9HB15	KCNK12	Potassium channel subfamily K member 12
0.917	0.883	1.396				0.767	1.303	1.407	0.845	0.752	1.053	A8MYU2	KCNU1	Potassium channel subfamily U member 1
1.124	0.971	0.916	0.777	0.678	0.834	1.110	0.879	1.084				Q14721	KCNB1	Potassium voltage-gated channel subfamily B member 1
0.832	0.946	0.983	1.072	1.077	1.086	0.958	1.040	1.110				Q8TAE7	KCNG3	Potassium voltage-gated channel subfamily G member 3
						1.127	1.052	1.116				Q8TDN1	KCNG4	Potassium voltage-gated channel subfamily G member 4
									0.890	0.934	0.973	A5A3E0	POTEF	POTE ankyrin domain family member F
0.962	1.031	0.983				0.954	0.963	0.894	0.954	1.020	1.022	P0CG38	POTEI	POTE ankyrin domain family member I
1.721	0.978	1.226	1.189	1.932	0.784	1.167	0.841	1.234	0.969	0.986	1.070	P0CG39	POTEJ	POTE ankyrin domain family member J
1.079	0.942	0.883	0.963	0.967	0.963	0.889	1.114	1.087	1.000	0.966	1.039	Q9HBE1	PATZ1	POZ-, AT hook-, and zinc finger-containing protein 1
1.099	1.016	0.931	0.944	0.947	1.112	0.921	1.257	0.942	1.043	1.019	0.885	Q8N755	PQLC3	PQ-loop repeat-containing protein 3
									0.905	0.928	1.109	Q9H4Q3	PRDM13	PR domain zinc finger protein 13
1.066	1.111	1.075	0.955	0.946	1.037	1.146	1.144	0.994				P57071	PRDM15	PR domain zinc finger protein 15
0.983	0.985	0.992	0.991	0.918	1.017	1.022	1.074	1.053	1.015	0.987	1.057	O60831	PRAF2	PRA1 family protein 2
0.938	0.977	0.963	0.968	0.916	0.984	1.030	1.039	1.007	1.080	0.968	1.068	O75915	ARL6IP5	PRA1 family protein 3
			0.926	0.848	0.922	1.306	1.209	0.991	1.227	0.969	1.445	A6NFR9	PRAMEF14	PRAME family member 14
1.075	1.085	0.995	1.167	1.130	1.329	0.976	1.326	1.040	1.334	1.095	1.663	P40425	PBX2	Pre-B-cell leukemia transcription factor 2
			0.782	0.747	1.036				0.834	0.961	0.786	Q9BYU1	PBX4	Pre-B-cell leukemia transcription factor 4
0.962	0.982	1.043	0.979	0.933	1.018	0.974	1.005	0.979	0.977	0.963	1.033	Q96AQ6	PBXIP1	Pre-B-cell leukemia transcription factor-interacting protein 1
0.996	0.995	1.028	0.970	1.002	1.033	0.982	0.981	1.001	1.015	0.994	1.058	O60925	PFDN1	Prefoldin subunit 1
1.036	0.994	1.005	0.985	0.932	1.017	0.988	1.052	1.008	0.969	1.006	0.988	Q9UHV9	PFDN2	Prefoldin subunit 2
1.000	1.010	0.989	1.011	1.032	1.035	1.012	1.012	1.002	0.987	1.021	1.036	P61758	VBP1	Prefoldin subunit 3
1.019	0.992	1.012	0.957	0.923	1.036	0.952	1.073	1.023	0.979	0.981	1.013	Q9NQP4	PFDN4	Prefoldin subunit 4
1.041	1.014	0.984	1.040	1.039	0.983	1.010	1.025	1.001	0.986	1.007	0.984	Q99471	PFDN5	Prefoldin subunit 5
0.944	0.982	0.970	0.975	1.036	1.012	0.968	0.951	0.986	0.953	0.993	0.963	O15212	PFDN6	Prefoldin subunit 6

Abundance Ratio														
1 d M1/C1	1 d M2/C2	1 d M3/C3	4 d M1/C1	4 d M2/C2	4 d M3/C3	7 d M1/C1	7 d M2/C2	7 d M3/C3	10 d M1/C1	10 d M2/C2	10 d M3/C3	Accession ID	Gene name	Protein Description
0.728	1.125	0.986										Q00888	PSG4	Pregnancy-specific beta-1-glycoprotein 4
0.770	0.976	0.747	1.026	1.110	1.008	1.113	1.103	1.070	1.058	0.969	0.905	Q00887	PSG9	Pregnancy-specific beta-1-glycoprotein 9
1.005	1.000	0.994	0.992	0.992	0.987	0.961	0.974	0.972	0.979	1.019	0.969	P02545	LMNA	Prelamin-A/C
0.908	0.987	0.904				1.337	1.525	1.301				Q9Y3B1	PRELID3B	PRELI domain containing protein 3B
						1.014	0.965	1.066				Q9Y255	PRELID1	PRELI domain-containing protein 1, mitochondrial
0.998	0.992	1.015	0.968	0.979	0.979	1.001	1.012	1.010	0.983	0.975	1.016	Q9C0J8	WDR33	pre-mRNA 3' end processing protein WDR33
1.005	1.023	1.038	0.963	0.962	1.025	0.970	0.965	1.009	0.991	1.013	1.045	Q6UN15	FIP1L1	Pre-mRNA 3'-end-processing factor FIP1
0.989	0.987	1.044	0.968	0.971	1.015	1.020	1.038	1.050	1.034	1.016	1.063	O94913	PCF11	Pre-mRNA cleavage complex 2 protein Pcf11
1.005	0.950	1.003	0.996	0.952	1.048	1.037	0.997	1.017	1.025	0.983	1.040	O60508	CDC40	Pre-mRNA-processing factor 17
1.065	1.004	1.028	0.987	0.962	0.990	0.967	0.996	0.961	0.939	1.028	0.965	Q9UMS4	PRPF19	Pre-mRNA-processing factor 19
0.991	0.988	0.995	1.009	0.965	1.027	1.022	1.020	1.036	0.986	0.995	1.034	Q86UA1	PRPF39	Pre-mRNA-processing factor 39
1.037	0.985	1.025	1.024	0.996	0.871	1.053	0.997	1.035	1.021	0.995	0.937	O75400	PRPF40A	Pre-mRNA-processing factor 40 homolog A
0.988	0.986	1.017	0.998	0.989	1.031	0.981	0.995	1.000	0.977	0.989	1.009	O94906	PRPF6	Pre-mRNA-processing factor 6
0.994	1.001	0.997	0.995	0.974	1.018	0.999	1.059	1.006	0.984	1.025	0.972	Q6P2Q9	PRPF8	Pre-mRNA-processing-splicing factor 8
0.990	1.053	1.115	1.026	0.839	1.054	0.972	1.125	1.030	0.905	0.992	1.032	Q99633	PRPF18	Pre-mRNA-splicing factor 18
1.026	1.014	1.026	1.007	1.038	1.023	1.022	1.064	0.999	1.038	1.009	1.010	Q8NAV1	PRPF38A	Pre-mRNA-splicing factor 38A
0.995	0.972	1.022	0.959	0.894	1.014	0.945	0.982	0.974	0.961	0.950	0.945	Q5VTL8	PRPF38B	Pre-mRNA-splicing factor 38B
0.998	1.018	1.011	1.019	1.049	1.008	1.023	1.047	1.048	1.001	1.031	1.020	O43143	DHX15	Pre-mRNA-splicing factor ATP-dependent RNA helicase DHX15
1.038	0.991	1.012	0.993	0.962	1.023	1.036	1.038	1.036	0.992	1.021	0.992	Q92620	DHX38	Pre-mRNA-splicing factor ATP-dependent RNA helicase PRP16
1.024	0.988	1.053	0.976	1.014	1.029	0.979	1.055	1.014	0.961	1.009	1.053	Q9HCG8	CWC22	Pre-mRNA-splicing factor CWC22 homolog
1.004	1.012	0.986	1.068	1.100	1.095	1.232	1.017	1.109	0.993	1.024	0.879	Q9NXE8	CWC25	Pre-mRNA-splicing factor CWC25 homolog
0.967	1.004	0.956	0.994	1.071	0.991	1.015	0.880	1.001	1.061	1.019	1.058	Q9NW64	RBM22	Pre-mRNA-splicing factor RBM22
1.011	1.019	1.024	0.995	0.953	1.035	1.026	1.038	1.064	1.040	1.029	1.031	O95391	SLU7	Pre-mRNA-splicing factor SLU7
0.993	0.998	0.982	1.013	0.980	1.027	1.025	1.056	0.986	1.025	1.046	1.023	O75934	BCAS2	Pre-mRNA-splicing factor SPF27
0.990	0.989	1.029	1.026	0.970	1.028	1.023	1.037	1.006	1.015	1.000	1.041	Q9HCS7	XAB2	Pre-mRNA-splicing factor SYF1
1.063	0.709	0.810	0.961	1.005	1.087	1.050	0.953	0.998	1.008	1.062	0.987	O95926	SYF2	Pre-mRNA-splicing factor SYF2
1.021	1.008	1.023	1.000	1.007	1.025	1.026	1.051	1.008	1.040	1.013	1.055	Q15007	WTAP	Pre-mRNA-splicing regulator WTAP
0.998	1.040	1.041	1.027	0.938	0.932	1.019	1.113	1.002	1.015	1.040	1.039	Q9UI14	RABAC1	Prenylated Rab acceptor protein 1
1.027	1.002	1.004	1.001	1.016	0.947	0.953	0.983	0.978	0.981	0.978	0.931	Q9UHG3	PCYOX1	Prenylcysteine oxidase 1
0.998	1.073	1.022										Q8NBM8	PCYOX1L	Prenylcysteine oxidase-like
0.990	0.989	1.017	0.991	0.955	1.024	0.976	0.997	1.008	1.002	1.010	1.046	Q8IY81	FTSJ3	pre-rRNA processing protein FTSJ3
0.987	0.996	1.018	1.010	1.026	1.020	1.009	1.051	0.988	0.981	1.014	1.016	Q2NL82	TSR1	Pre-rRNA-processing protein TSR1 homolog

Abundance Ratio														
1 d M1/C1	1 d M2/C2	1 d M3/C3	4 d M1/C1	4 d M2/C2	4 d M3/C3	7 d M1/C1	7 d M2/C2	7 d M3/C3	10 d M1/C1	10 d M2/C2	10 d M3/C3	Accession ID	Gene name	Protein Description
0.965	0.964	0.917	0.990	1.008	1.096	1.047	0.939	1.005	0.947	1.048	1.110	Q969E8	TSR2	Pre-rRNA-processing protein TSR2 homolog
1.018	0.871	1.001	0.855	0.898	1.053				0.915	0.861	0.854	B1AP22	PSEN2	Presenilin
0.943	1.015	1.048	0.982	0.889	1.035	1.019	0.958	1.066	1.084	0.997	1.161	P49768	PSEN1	Presenilin-1
1.075	0.968	1.008	0.986	0.894	0.993	1.033	1.017	1.084	1.105	1.035	1.023	Q9H300	PARL	Presenilins-associated rhomboid-like protein, mitochondrial
0.964	1.181	1.135	0.815	0.995	0.771	0.903	0.787	0.847	1.143	0.947	1.192	O43900	PRICKLE3	Prickle-like protein 3
1.014	0.971	0.984	1.011	0.999	1.007	1.034	1.046	1.023	1.008	1.017	1.056	Q96IZ0	PAWR	PRKC apoptosis WT1 regulator protein
1.049	1.080	1.081	0.961	0.890	0.969	1.070	1.237	1.036	0.944	0.995	1.155	Q9NRD5	PICK1	PRKCA-binding protein
0.984	0.978	1.011	1.015	0.972	1.069	0.996	0.939	1.005	1.049	1.019	1.078	Q9H875	PRKRIP1	PRKR-interacting protein 1
0.964	1.022	1.026	0.977	0.917	1.047	0.986	1.123	1.036	0.835	0.963	1.116	O43709	BUD23	Probable 18S rRNA (guanine-N(7))-methyltransferase
1.025	0.974	1.016	1.019	0.941	0.967	1.024	0.960	0.981	1.066	0.908	0.994	Q96HY7	DHTKD1	Probable 2-oxoglutarate dehydrogenase E1 component DHKTD1, mitochondrial
1.067	1.027	0.977	1.019	1.023	1.000	1.017	1.072	0.993	1.107	0.972	1.042	Q8NDH3	NPEPL1	Probable aminopeptidase NPEPL1
0.996	0.983	1.025	0.979	0.909	1.021	0.987	0.995	1.006	0.978	0.984	1.056	Q5T160	RARS2	Probable arginine--tRNA ligase, mitochondrial
0.981	0.994	1.012	0.982	1.015	0.977	0.995	0.985	0.985	0.978	0.955	0.969	Q96I59	NARS2	Probable asparagine--tRNA ligase, mitochondrial
1.036	1.024	1.051	1.020	0.955	1.025	0.996	1.078	0.977	0.985	1.011	1.000	Q13206	DDX10	Probable ATP-dependent RNA helicase DDX10
1.016	0.997	0.997	0.984	1.008	0.997	0.994	0.997	0.982	0.992	1.000	0.976	Q92841	DDX17	Probable ATP-dependent RNA helicase DDX17
1.007	1.010	1.021	0.991	0.966	1.018	0.996	1.046	1.000	0.969	0.978	1.063	Q9UHI6	DDX20	Probable ATP-dependent RNA helicase DDX20
0.971	1.000	0.992	0.982	0.977	1.027	0.981	1.007	0.998	0.995	0.992	1.014	Q9BUQ8	DDX23	Probable ATP-dependent RNA helicase DDX23
0.982	0.991	1.002	1.010	1.005	1.045	1.001	1.010	1.012	0.986	1.016	1.002	Q96GQ7	DDX27	Probable ATP-dependent RNA helicase DDX27
0.980	0.998	1.027	0.968	0.969	1.023	1.016	0.987	1.004	1.026	1.001	1.122	Q9NUL7	DDX28	Probable ATP-dependent RNA helicase DDX28
1.048	1.021	1.043	1.001	0.973	0.998	1.019	1.009	1.027	1.030	0.998	1.032	Q9H8H2	DDX31	Probable ATP-dependent RNA helicase DDX31
1.016	0.991	0.992	1.020	0.962	1.015	0.978	1.070	0.996	0.975	1.000	0.974	J3KNN5	DDX41	Probable ATP-dependent RNA helicase DDX41 (Fragment)
0.988	0.998	0.997	1.011	0.911	1.059	1.018	1.124	1.076	1.031	0.989	0.864	A0A0C4DG89	DDX46	Probable ATP-dependent RNA helicase DDX46
0.975	0.997	0.997	0.991	0.979	0.999	1.003	0.974	0.981	1.008	1.000	1.036	Q7L014	DDX46	Probable ATP-dependent RNA helicase DDX46
0.981	1.015	1.003	1.015	0.971	1.030	0.985	1.117	1.007	0.962	1.022	0.968	Q9H0S4	DDX47	Probable ATP-dependent RNA helicase DDX47
1.096	0.998	1.056	1.071	0.967	1.053	1.010	1.197	0.985	1.251	0.752	1.018	Q9Y6V7	DDX49	Probable ATP-dependent RNA helicase DDX49
1.008	1.001	0.997	1.006	0.993	1.006	1.019	1.027	1.038	1.009	0.980	1.014	P17844	DDX5	Probable ATP-dependent RNA helicase DDX5
0.968	1.084	0.994	0.983	0.941	1.063	1.020	1.019	1.003	1.047	1.018	1.019	Q9Y2R4	DDX52	Probable ATP-dependent RNA helicase DDX52
1.010	0.995	1.044	1.024	0.973	1.028	0.993	1.014	1.052	0.986	1.035	1.039	Q9NY93	DDX56	Probable ATP-dependent RNA helicase DDX56
1.011	1.010	1.032	1.010	0.960	0.994	1.014	1.008	0.984	1.040	0.942	1.101	O95786	DDX58	Probable ATP-dependent RNA helicase DDX58
1.034	1.033	0.987	1.007	0.959	0.972	1.063	1.150	1.051	0.941	0.988	1.020	Q5T1V6	DDX59	Probable ATP-dependent RNA helicase DDX59
1.009	1.006	1.001	0.999	1.050	1.007	1.001	0.992	1.028	0.981	1.059	1.006	P26196	DDX6	Probable ATP-dependent RNA helicase DDX6

Abundance Ratio												Accession ID	Gene name	Protein Description
1 d M1/C1	1 d M2/C2	1 d M3/C3	4 d M1/C1	4 d M2/C2	4 d M3/C3	7 d M1/C1	7 d M2/C2	7 d M3/C3	10 d M1/C1	10 d M2/C2	10 d M3/C3			
1.041	1.013	1.052	0.909	0.895	1.008	0.906	1.134	1.071	1.020	0.958	1.107	Q8IY21	DDX60	Probable ATP-dependent RNA helicase DDX60
1.024	0.997	1.090	1.002	1.010	1.070							Q14147	DHX34	Probable ATP-dependent RNA helicase DHX34
0.975	1.010	0.977	0.975	0.997	1.035	0.958	1.030	1.129	0.913	1.021	1.067	Q9H5Z1	DHX35	Probable ATP-dependent RNA helicase DHX35
1.020	1.006	1.056	0.999	1.022	0.992	0.972	1.023	1.005	0.939	1.031	0.998	Q8IY37	DHX37	Probable ATP-dependent RNA helicase DHX37
0.984	0.999	0.982	1.003	1.007	1.023	0.990	1.077	1.071	0.981	0.946	1.032	Q8IX18	DHX40	Probable ATP-dependent RNA helicase DHX40
1.011	1.006	1.004	1.004	0.997	1.018	1.029	1.067	1.028	1.033	0.980	1.024	Q9H6S0	YTHDC2	Probable ATP-dependent RNA helicase YTHDC2
1.105	0.903	1.127	1.048	0.904	1.017	0.979	1.193	1.077	0.998	1.117	0.924	Q9H7F0	ATP13A3	Probable cation-transporting ATPase 13A3
0.989	1.049	1.032	0.991	0.964	0.972	1.063	1.054	0.971	1.073	1.053	1.056	A0A1B0GW05	DPY19L1	Probable C-mannosyltransferase DPY19L1
			0.945	0.951	0.975							Q7Z388	DPY19L4	Probable C-mannosyltransferase DPY19L4
1.127	1.092	1.038	0.994	0.940	1.027	0.997	1.104	1.048	1.057	0.949	1.079	Q9HA77	CARS2	Probable cysteine--tRNA ligase, mitochondrial
1.001	0.957	1.057	1.036	1.168	0.989	1.075	0.976	1.031	0.968	1.015	1.027	O76071	CIAO1	Probable cytosolic iron-sulfur protein assembly protein CIAO1
1.036	1.015	1.001	1.009	1.002	1.020	1.026	1.109	1.012	0.971	1.044	0.985	Q9UNQ2	DIMT1	Probable dimethyladenosine transferase
0.883	0.915	1.009	1.000	0.812	1.181	1.041	1.102	1.127	1.126	1.043	1.080	Q9BVK2	ALG8	Probable dolichyl pyrophosphate Glc1Man9GlcNAc2 alpha-1,3-glucosyltransferase
			0.790	1.059	0.959							Q96FN9	DTD2	Probable D-tyrosyl-tRNA(Tyr) deacylase 2
1.080	1.039	1.013	1.007	0.964	0.981	0.942	1.042	0.930	0.935	0.953	0.953	J3KPF0	HECTD4	Probable E3 ubiquitin-protein ligase HECTD4
1.016	1.099	1.008	1.000	1.027	0.975	1.056	1.185	0.938				Q15751	HERC1	Probable E3 ubiquitin-protein ligase HERC1
0.991	0.988	1.016	0.996	0.963	1.004	1.019	1.104	1.035	1.005	0.983	1.012	Q5GLZ8	HERC4	Probable E3 ubiquitin-protein ligase HERC4
0.936	0.958	0.917	0.965	0.952	1.134	0.997	1.047	1.012	1.017	1.052	0.937	Q8IVU3	HERC6	Probable E3 ubiquitin-protein ligase HERC6
1.071	0.998	1.029	1.059	1.190	1.067	1.157	0.940	1.055	1.071	0.990	1.033	Q9H000	MKRN2	Probable E3 ubiquitin-protein ligase makorin-2
1.366	0.949	1.554				0.944	0.834	0.962	1.006	1.194	1.224	Q9UJV3	MID2	Probable E3 ubiquitin-protein ligase MID2
1.026	1.000	1.072	1.001	0.896	1.012	1.115	1.054	1.049	1.091	0.934	1.124	J3KNI5	TRIML2	Probable E3 ubiquitin-protein ligase TRIML2 (Fragment)
1.076	1.044	1.154	1.039	0.885	0.806	0.855	1.123	1.053	0.980	0.986	0.840	Q9UKR5	C14orf1	Probable ergosterol biosynthetic protein 28
0.993	0.985	1.005	0.980	0.982	0.965	0.988	0.996	0.982	1.019	0.969	1.019	B7ZLQ5	SMARCA1	Probable global transcription activator SNF2L1
0.958	0.902	1.022	1.071	1.010	0.931	0.981	1.104	1.035	1.058	0.927	1.077	P51531	SMARCA2	Probable global transcription activator SNF2L2
0.982	0.976	0.953	0.913	0.935	1.011	0.930	1.105	0.943	0.925	1.043	1.023	Q8TED1	GPX8	Probable glutathione peroxidase 8
1.020	1.016	1.042	0.986	0.914	1.019	1.032	1.202	1.040	1.048	1.047	1.137	J3QS41	HELZ	Probable helicase with zinc finger domain
0.958	0.984	1.008	1.016	0.977	1.010	1.003	0.981	1.004	0.988	0.934	1.053	P49590	HARS2	Probable histidine--tRNA ligase, mitochondrial
1.115	0.858	1.073	0.997	0.934	1.027	1.111	0.909	1.040	1.052	1.032	1.121	Q8N490	PNKD	Probable hydrolase PNKD
1.066	0.980	0.990	1.030	0.872	1.039	1.121	0.873	0.915	0.964	0.987	0.950	Q15652	JMJD1C	Probable JmjC domain-containing histone demethylation protein 2C
1.003	0.989	0.987	0.972	0.972	0.977	0.971	0.973	0.958	1.003	0.957	1.003	Q15031	LARS2	Probable leucine--tRNA ligase, mitochondrial
0.941	0.923	0.949	0.906	0.921	1.130	1.074	1.098	1.151	1.095	0.960	1.130	Q9NUN5	LMBRD1	Probable lysosomal cobalamin transporter

Abundance Ratio														
1 d M1/C1	1 d M2/C2	1 d M3/C3	4 d M1/C1	4 d M2/C2	4 d M3/C3	7 d M1/C1	7 d M2/C2	7 d M3/C3	10 d M1/C1	10 d M2/C2	10 d M3/C3	Accession ID	Gene name	Protein Description
			0.852	0.859	1.087	0.868	1.086	1.043	1.102	1.023	1.258	Q13395	TARBP1	Probable methyltransferase TARBP1
0.964	0.999	0.972	0.959	1.009	1.014	1.037	0.922	1.022	1.058	0.979	1.103	A6NJ78	METTL15	Probable methyltransferase-like protein 15
1.070	1.020	0.998	0.924	1.171	0.962	1.089	0.968	1.086	0.864	1.110	0.985	Q5W0Z9	ZDHHC20	Probable palmitoyltransferase ZDHHC20
									1.372	0.981	0.947	Q9NPG8	ZDHHC4	Probable palmitoyltransferase ZDHHC4
			1.261	1.168	1.034				1.027	1.011	1.045	Q9H3J6	C12orf65	Probable peptide chain release factor C12orf65, mitochondrial
1.062	0.997	1.050	0.990	0.927	0.972	1.016	0.961	1.016	1.018	1.016	1.059	Q86Y79	PTRH1	Probable peptidyl-tRNA hydrolase
0.982	1.074	1.023	1.195	1.173	1.147	0.823	0.672	1.059	0.876	0.993	1.267	Q9Y2G3	ATP11B	Probable phospholipid-transporting ATPase IF
0.962	1.021	0.940				0.769	0.987	1.009	1.168	0.821	1.183	O75110	ATP9A	Probable phospholipid-transporting ATPase IIA
0.963	1.025	1.071	0.977	0.945	1.016	0.935	0.913	0.943	1.009	0.970	1.046	O43861	ATP9B	Probable phospholipid-transporting ATPase IIB
1.038	1.010	0.964	0.971	0.941	1.024	0.970	1.095	1.039	1.010	0.948	1.011	Q7L3T8	PARS2	Probable proline--tRNA ligase, mitochondrial
1.007	1.009	0.958	1.020	0.987	1.087	0.972	1.025	0.971	0.995	1.059	1.041	Q9UHA3	RSL24D1	Probable ribosome biogenesis protein RLP24
1.084	0.835	1.000	0.909	0.972	1.126	0.986	0.735	1.171	1.041	1.084	3.110	Q96CW6	SLC7A6OS	Probable RNA polymerase II nuclear localization protein SLC7A6OS
1.083	1.084	0.892	0.997	1.001	1.076	1.112	1.122	1.011	1.092	1.017	1.006	Q96H35	RBM18	Probable RNA-binding protein 18
1.005	0.978	0.982	1.011	0.936	1.028	1.024	1.022	0.983	1.033	1.021	1.046	Q9Y4C8	RBM19	Probable RNA-binding protein 19
1.001	0.990	0.912	0.911	0.945	1.010	0.977	1.094	0.965				Q86U06	RBM23	Probable RNA-binding protein 23
0.848	1.093	0.835	1.005	1.013	1.008	1.018	1.059	0.975	0.945	0.944	1.036	Q8N9N8	EIF1AD	Probable RNA-binding protein EIF1AD
0.988	0.928	0.870	1.110	0.939	1.123	0.916	0.849	1.140	1.070	0.921	1.033	A2RTX5	TARSL2	Probable threonine--tRNA ligase 2, cytoplasmic
0.995	0.976	0.991	1.001	0.980	0.981	0.969	1.030	1.035	0.981	1.022	1.081	Q9NPF4	OSGEP	Probable tRNA N6-adenosine threonylcarbamoyltransferase
1.008	1.041	0.981	1.093	0.990	1.071	1.009	1.066	1.010	1.050	1.026	1.010	Q9H4B0	OSGEPL1	Probable tRNA N6-adenosine threonylcarbamoyltransferase, mitochondrial
1.075	1.010	1.004	0.993	0.949	1.005	1.020	1.056	0.938	0.977	0.985	1.005	Q8WWH5	TRUB1	Probable tRNA pseudouridine synthase 1
0.989	0.980	1.032	0.992	0.867	1.043	1.141	1.108	0.993	0.932	0.983	1.188	O95900	TRUB2	Probable tRNA pseudouridine synthase 2
1.031	0.992	1.024	1.039	1.008	0.989	0.998	1.172	0.983	1.062	1.029	0.966	Q9NWX6	THG1L	Probable tRNA(His) guanylyltransferase
1.013	0.991	1.006	0.992	0.906	1.023	1.008	1.025	1.011	1.038	0.993	0.990	Q9Y3A2	UTP11	Probable U3 small nucleolar RNA-associated protein 11
0.977	0.999	1.013	0.996	0.984	1.021	1.015	1.030	1.013	0.981	0.946	1.033	Q93008	USP9X	Probable ubiquitin carboxyl-terminal hydrolase FAF-X
1.029	1.005	0.985	0.968	0.956	0.993	0.995	1.007	1.017	1.023	0.991	1.023	Q9NQH7	XPNPEP3	Probable Xaa-Pro aminopeptidase 3
1.181	1.037	1.066	1.023	0.948	1.012	0.959	1.148	0.961	0.911	1.019	0.869	P09668	CTSH	Pro-cathepsin H
0.975	0.995	1.007	0.972	0.998	1.039	0.992	1.028	1.017	0.975	0.986	1.015	Q8NBJ5	COLGALT1	Procollagen galactosyltransferase 1
0.987	0.985	1.009	0.995	1.009	1.028	0.972	1.026	1.003	0.986	0.976	1.006	O60568	PLOD3	Procollagen-lysine,2-oxoglutarate 5-dioxygenase 3
0.935	1.111	0.973	0.973	0.963	1.266	1.141	1.120	1.183	1.138	1.060	1.161	O14944	EREG	Proepiregulin

Abundance Ratio														
1 d M1/C1	1 d M2/C2	1 d M3/C3	4 d M1/C1	4 d M2/C2	4 d M3/C3	7 d M1/C1	7 d M2/C2	7 d M3/C3	10 d M1/C1	10 d M2/C2	10 d M3/C3	Accession ID	Gene name	Protein Description
0.845	0.935	1.107	0.952	0.839	1.042	0.940	1.045	1.112	1.066	0.977	1.230	I3L3D5	PFN1	Profilin (Fragment)
1.005	1.036	0.949	0.993	0.966	0.991	0.978	1.011	0.967	1.004	1.026	0.975	G5E9Q6	PFN2	Profilin
1.016	1.000	0.959	1.039	0.943	1.005	1.020	1.038	0.995	0.992	0.977	0.956	P07737	PFN1	Profilin-1
0.930	1.061	0.904	1.039	0.986	1.098	0.937	1.013	1.045	0.997	1.064	1.078	P35080	PFN2	Profilin-2
1.133	1.106	1.039	1.026	1.076	0.966				0.933	0.912	1.096	Q8WXW3	PIBF1	Progesterone-induced-blocking factor 1
			0.886	0.968	1.249							Q6TCH7	PAQR3	Progestin and adipoQ receptor family member 3
1.012	1.010	1.024	1.009	0.998	1.013	1.004	1.019	1.006	1.009	0.994	1.000	Q8WUM4	PDCD6IP	Programmed cell death 6-interacting protein
1.026	1.012	1.018	0.984	0.967	1.027	0.975	1.045	1.017	0.973	1.002	1.021	Q9BUL8	PDCD10	Programmed cell death protein 10
1.078	1.049	1.057	0.990	0.975	1.052	0.985	1.020	0.986	1.010	1.065	1.048	Q16342	PDCD2	Programmed cell death protein 2
0.867	1.146	1.022	1.968	0.792	1.073	1.000	1.003	1.059	0.848	1.081	1.022	Q9BRP1	PDCD2L	Programmed cell death protein 2-like
1.028	0.977	0.999	0.992	0.940	1.012	0.988	1.040	1.005	1.009	0.950	1.047	Q53EL6	PDCD4	Programmed cell death protein 4
0.991	1.026	0.956	0.988	0.982	0.990	0.990	1.051	0.990	0.959	0.985	0.985	O14737	PDCD5	Programmed cell death protein 5
0.993	1.024	0.947	1.081	0.993	0.997	1.097	1.108	0.976	1.086	1.025	0.961	O75340	PDCD6	Programmed cell death protein 6
			1.061	1.106	0.794							Q8N8D1	PDCD7	Programmed cell death protein 7
1.034	0.996	1.024	0.993	1.004	1.015	0.942	1.005	1.013	0.925	0.985	0.940	P35232	PHB	Prohibitin
0.999	0.998	0.995	0.982	0.973	1.024	0.992	0.989	1.008	0.995	1.008	1.021	Q99623	PHB2	Prohibitin-2
0.963	0.994	0.988	1.006	0.894	0.948	0.946	0.983	0.972	1.030	1.065	0.997	Q9HCU5	PREB	Prolactin regulatory element-binding protein
0.938	0.998	0.969	0.993	1.018	0.971	1.073	1.048	0.994	0.968	0.971	1.064	P12004	PCNA	Proliferating cell nuclear antigen
0.942	0.994	1.014	0.994	0.928	0.988	1.050	1.113	1.030	0.907	0.899	1.059	P46013	MKI67	Proliferation marker protein Ki-67
0.967	1.002	0.973	1.000	1.001	1.004	0.977	1.019	1.000	0.975	1.026	0.969	Q9UQ80	PA2G4	Proliferation-associated protein 2G4
0.913	0.986	1.159										Q86XN7	PROSER1	Proline and serine-rich protein 1
1.002	1.028	1.010	1.005	1.148	1.005	0.990	0.918	1.045	1.047	0.946	1.163	Q86WR7	PROSER2	Proline and serine-rich protein 2
1.005	0.993	0.985	1.003	0.980	0.995	1.033	1.025	1.022	1.011	1.011	1.037	O94903	PROSC	Proline synthase co-transcribed bacterial homolog protein
1.000	1.018	1.003	0.999	1.016	0.998	1.034	1.033	0.992	0.992	0.993	0.979	C9JFV4	PELP1	Proline-, glutamic acid- and leucine-rich protein 1
1.041	1.010	1.089										Q96HE9	PRR11	Proline-rich protein 11
1.152	0.982	1.143	1.090	0.944	1.112	0.986	0.887	1.058	0.850	0.973	1.093	Q9BWN1	PRR14	Proline-rich protein 14
1.060	0.979	1.026	1.035	1.008	1.017	1.235	1.034	1.020	0.928	0.971	0.964	P79522	PRR3	Proline-rich protein 3
1.001	1.004	0.978	1.010	0.971	1.012	0.989	1.011	0.988	1.086	1.005	1.065	Q92733	PRCC	Proline-rich protein PRCC
1.003	0.993	1.013	1.026	0.950	1.012	1.026	1.136	1.046	1.059	0.960	1.178	Q07954	LRP1	Prolow-density lipoprotein receptor-related protein 1
0.996	0.996	0.988	1.022	0.979	1.000	0.967	0.989	1.004	1.007	0.954	1.038	Q32P28	P3H1	Prolyl 3-hydroxylase 1
0.973	0.947	1.010	0.933	0.828	0.935							Q8IVL5	P3H2	Prolyl 3-hydroxylase 2
0.938	0.976	0.974	1.003	0.958	1.014	1.034	0.989	0.974	0.989	0.964	1.079	Q8N543	OGFOD1	Prolyl 3-hydroxylase OGFOD1

Abundance Ratio														
1 d M1/C1	1 d M2/C2	1 d M3/C3	4 d M1/C1	4 d M2/C2	4 d M3/C3	7 d M1/C1	7 d M2/C2	7 d M3/C3	10 d M1/C1	10 d M2/C2	10 d M3/C3	Accession ID	Gene name	Protein Description
1.011	1.015	1.033	0.983	0.946	0.965	1.024	1.001	0.981	1.041	0.883	1.061	O15460	P4HA2	Prolyl 4-hydroxylase subunit alpha-2
0.973	1.000	1.007	1.007	1.027	1.002	0.972	0.970	1.004	0.990	0.974	1.000	P48147	PREP	Prolyl endopeptidase
1.037	0.997	1.025	0.990	0.992	0.973	0.982	0.996	0.999	0.995	1.011	1.013	Q4J6C6	PREPL	Prolyl endopeptidase-like
1.075	1.014	1.051	0.999	0.965	1.016	0.984	1.035	0.998	1.046	1.008	1.049	P05165	PCCA	Propionyl-CoA carboxylase alpha chain, mitochondrial
1.021	1.034	1.047	0.988	0.939	1.015	1.041	1.051	1.028	1.045	0.997	1.063	E7EX59	PCCB	Propionyl-CoA carboxylase beta chain, mitochondrial
0.758	0.907	0.883	1.007	1.153	1.039	1.232	0.958	1.021	1.261	1.145	1.201	P07602	PSAP	Prosaposin
			1.040	0.968	0.972	1.067	1.233	1.022	1.128	1.142	0.961	Q13258	PTGDR	Prostaglandin D2 receptor
1.015	1.012	0.970	0.982	0.981	0.958	0.991	1.014	0.980	1.027	0.969	0.973	Q9H7Z7	PTGES2	Prostaglandin E synthase 2
1.121	0.995	0.977	1.003	1.221	0.961	1.022	0.864	1.029	0.974	0.982	0.970	Q15185	PTGES3	Prostaglandin E synthase 3
			0.868	0.945	0.986	0.981	0.994	1.030	0.928	1.079	1.150	O14684	PTGES	Prostaglandin E synthase
0.973	0.996	0.981	0.981	0.998	0.982	0.950	1.005	0.999	1.001	0.974	0.979	Q14914	PTGR1	Prostaglandin reductase 1
1.012	1.018	0.993	0.991	0.929	0.939	0.921	1.056	1.039	0.977	1.023	0.979	Q8N8N7	PTGR2	Prostaglandin reductase 2
1.090	1.057	1.010	1.041	0.987	1.065	1.094	1.046	0.975	1.029	0.989	1.031	Q8N4Q0	ZADH2	Prostaglandin reductase 3
0.991	1.048	0.957	0.989	0.954	1.009	0.974	1.063	1.009	0.973	1.031	1.027	A0A0A0MT35	FAM213B	Prostamide/prostaglandin F synthase
0.989	0.965	1.045	0.992	0.947	0.969	1.039	0.932	0.896	1.024	0.990	1.063	Q86YD1	PTOV1	Prostate tumor-overexpressed gene 1 protein
						1.085	1.236	0.924				Q9BSG0	PRADC1	Protease-associated domain-containing protein 1
1.138	0.965	0.995	1.128	0.988	1.000	1.243	0.974	0.988	1.081	0.995	1.027	Q9BRP4	PAAF1	Proteasomal ATPase-associated factor 1
0.916	0.992	0.959	0.978	1.076	1.000	1.016	0.866	0.979	1.019	1.026	1.085	Q16186	ADRM1	Proteasomal ubiquitin receptor ADRM1
1.088	1.020	1.007	1.007	1.053	0.985	0.985	0.996	0.994	0.954	0.985	0.933	Q06323	PSME1	Proteasome activator complex subunit 1
1.133	0.848	0.861										H0YM70	PSME2	Proteasome activator complex subunit 2
1.040	1.015	1.042	1.020	1.093	1.033	0.957	1.023	1.016	0.964	1.011	0.996	A0A087X1Z3	PSME2	Proteasome activator complex subunit 2
1.020	1.016	1.016	0.989	0.920	1.028	1.004	1.103	1.083	0.970	0.989	0.984	Q14997	PSME4	Proteasome activator complex subunit 4
0.982	0.996	0.984	0.981	1.018	1.031	0.953	1.026	1.035	0.928	0.973	0.983	O95456	PSMG1	Proteasome assembly chaperone 1
0.899	1.027	0.969	1.089	1.053	0.980	1.068	1.056	1.045	0.991	0.973	0.986	Q969U7	PSMG2	Proteasome assembly chaperone 2
1.040	1.010	1.015	0.997	0.963	1.036	0.971	1.129	1.011	0.951	0.984	1.074	Q9BT73	PSMG3	Proteasome assembly chaperone 3
1.062	1.045	1.035	0.995	0.988	0.973	0.975	1.119	1.004	0.972	1.077	0.998	Q92530	PSMF1	Proteasome inhibitor PI31 subunit
0.949	0.991	1.010	0.950	0.914	0.945	0.919	1.031	0.971	0.907	0.933	1.020	Q9Y244	POMP	Proteasome maturation protein
1.045	1.024	0.980	1.003	1.002	1.002	0.995	1.109	1.018	0.977	0.990	0.943	A0A024RA52	PSMA2	Proteasome subunit alpha type
1.010	0.994	0.999	1.005	1.159	0.994	1.035	0.946	1.029	1.009	0.984	0.966	G3V5Z7	PSMA6	Proteasome subunit alpha type
1.005	0.999	0.955	1.003	0.968	0.988	1.002	1.003	0.966	0.998	0.981	0.947	P25788	PSMA3	Proteasome subunit alpha type-3
1.012	1.008	1.015	0.988	1.024	0.987	0.974	0.935	0.994	0.978	0.990	0.974	P25789	PSMA4	Proteasome subunit alpha type-4
1.021	1.020	0.987	1.007	0.993	0.989	0.995	1.073	0.984	0.998	1.010	1.004	P28066	PSMA5	Proteasome subunit alpha type-5
1.037	1.016	1.005	0.984	0.970	0.988	0.982	1.045	0.987	0.972	0.976	0.988	O14818	PSMA7	Proteasome subunit alpha type-7

Abundance Ratio														
1 d M1/C1	1 d M2/C2	1 d M3/C3	4 d M1/C1	4 d M2/C2	4 d M3/C3	7 d M1/C1	7 d M2/C2	7 d M3/C3	10 d M1/C1	10 d M2/C2	10 d M3/C3	Accession ID	Gene name	Protein Description
1.043	1.008	0.977	1.033	1.037	0.976	0.988	1.023	0.981	0.996	1.010	0.956	P20618	PSMB1	Proteasome subunit beta type-1
1.003	1.007	0.977	0.984	0.938	0.984	0.987	1.011	0.999	0.977	0.933	0.982	P40306	PSMB10	Proteasome subunit beta type-10
0.986	1.019	0.967	1.021	1.072	0.957	0.926	0.993	0.968	0.958	1.007	0.928	P49721	PSMB2	Proteasome subunit beta type-2
1.009	1.013	0.950	1.009	1.016	0.968	0.992	1.050	0.991	0.973	1.020	0.941	P49720	PSMB3	Proteasome subunit beta type-3
0.952	1.005	0.994	0.968	0.955	1.000	0.994	1.072	1.024	0.974	0.993	0.994	P28070	PSMB4	Proteasome subunit beta type-4
1.046	1.009	0.975	1.057	0.932	0.958	0.979	1.009	0.946	1.001	1.007	0.949	P28074	PSMB5	Proteasome subunit beta type-5
1.006	0.977	0.989	0.979	0.960	1.009	0.974	1.009	0.942	1.023	1.022	0.976	P28072	PSMB6	Proteasome subunit beta type-6
1.007	1.006	0.954	1.013	0.951	0.954	0.969	1.030	0.930	0.991	1.031	0.944	Q99436	PSMB7	Proteasome subunit beta type-7
1.042	1.008	1.009	1.046	1.030	0.997	1.020	0.977	0.980	0.964	0.947	0.956	P28062	PSMB8	Proteasome subunit beta type-8
1.008	0.999	0.976	1.002	0.970	0.993	0.979	1.044	0.992	0.974	0.964	1.001	P28065	PSMB9	Proteasome subunit beta type-9
0.967	0.896	0.966				1.255	1.268	1.020	0.950	0.913	0.965	Q9NUX5	POT1	Protection of telomeres protein 1
1.000	0.974	1.082	0.985	0.928	0.997	1.026	1.055	1.019	0.999	0.946	1.041	P11171	EPB41	Protein 4.1
1.067	0.970	1.054	0.981	0.984	1.091	0.991	1.007	1.077	0.978	0.971	1.052	Q9Y312	AAR2	Protein AAR2 homolog
1.068	0.972	1.010	0.969	0.954	0.993	0.973	1.040	1.012	0.975	1.018	1.018	Q9NY61	AATF	Protein AATF
0.991	0.993	1.005	0.971	0.968	0.988	0.996	1.019	0.993	1.015	0.933	1.027	Q8NFV4	ABHD11	Protein ABHD11
1.090	1.016	0.986	1.005	0.973	0.954	0.965	1.044	0.970	0.987	0.982	0.906	Q96IU4	ABHD14B	Protein ABHD14B
						1.115	1.451	1.035				Q6UXT9	ABHD15	Protein ABHD15
1.316	1.316	1.189	1.187	1.084	1.144	0.972	1.090	1.062				Q8TB40	ABHD4	Protein ABHD4
0.943	1.080	1.094	0.945	0.997	0.970	0.958	0.952	0.991	1.015	0.973	1.119	A0A087WW39	MLLT6	Protein AF-17
1.043	0.906	0.988										Q13015	MLLT11	Protein AF1q
0.949	0.977	0.985	0.994	0.972	1.025	0.988	0.993	0.992	1.041	1.021	1.018	Q8IVF2	AHNAK2	Protein AHNAK2
						1.017	1.080	1.050	1.013	0.969	0.974	Q8IY45	AMN1	Protein AMN1 homolog
			0.699	1.003	1.513				1.038	0.988	1.099	Q9UNK9	ANGEL1	Protein angel homolog 1
0.918	0.931	1.040	1.005	1.058	1.003	1.003	1.034	1.077	0.996	1.048	1.097	Q5VTE6	ANGEL2	Protein angel homolog 2
0.979	0.958	1.073	1.031	0.953	1.035	0.842	0.921	0.960	1.062	0.979	1.141	A8K0B5	ZBTB8OS	Protein archease
0.986	1.008	1.022	0.994	1.010	1.008	1.022	1.009	0.998	1.008	0.995	1.007	Q7L592	NDUFAF7	Protein arginine methyltransferase NDUFAF7, mitochondrial
1.011	0.996	0.988	1.013	1.037	0.989	1.033	1.001	1.049	1.004	1.020	0.997	H7C2I1	PRMT1	Protein arginine N-methyltransferase 1
1.006	1.038	1.029	1.037	0.905	1.054							P55345	PRMT2	Protein arginine N-methyltransferase 2
1.003	0.979	1.008	0.984	0.977	1.039	1.025	1.054	1.007	0.984	0.999	1.016	O60678	PRMT3	Protein arginine N-methyltransferase 3
1.047	0.996	0.999	1.005	0.979	0.987	1.026	1.008	0.990	1.004	0.977	0.957	O14744	PRMT5	Protein arginine N-methyltransferase 5
0.997	1.105	0.891	0.996	0.969	1.000	0.925	1.158	1.015	0.989	1.083	1.043	Q96LA8	PRMT6	Protein arginine N-methyltransferase 6
1.118	1.052	0.929										Q9NVM4	PRMT7	Protein arginine N-methyltransferase 7
1.030	1.051	0.975	1.013	0.970	0.976	1.006	1.043	0.998	0.962	1.003	1.007	Q9UL18	AGO1	Protein argonaute-1

Abundance Ratio														
1 d M1/C1	1 d M2/C2	1 d M3/C3	4 d M1/C1	4 d M2/C2	4 d M3/C3	7 d M1/C1	7 d M2/C2	7 d M3/C3	10 d M1/C1	10 d M2/C2	10 d M3/C3	Accession ID	Gene name	Protein Description
1.038	1.014	1.002	1.037	0.970	0.989	1.019	1.092	1.016	1.022	1.055	1.036	Q9UKV8	AGO2	Protein argonaute-2
0.944	0.994	1.039	1.012	0.969	0.943	0.990	1.216	0.910	0.934	0.971	1.023	Q9H9G7	AGO3	Protein argonaute-3
1.024	1.038	1.041	1.003	0.945	1.018	0.993	1.002	1.028	1.012	1.000	1.063	Q9NVM9	ASUN	Protein asunder homolog
1.076	0.931	1.022	0.936	0.948	0.992	1.065	1.022	0.925	1.105	0.997	0.984	Q8N9N5	BANP	Protein BANP
0.978	0.979	0.993	0.994	0.965	1.028	1.005	0.965	1.004	1.049	0.955	1.073	Q9H694	BICC1	Protein bicaudal C homolog 1
0.862	1.059	0.829	0.907	0.937	0.978	1.040	0.880	1.068	0.722	0.690	1.096	Q96G01	BICD1	Protein bicaudal D homolog 1
1.052	1.061	0.973	1.037	1.136	1.028	1.053	1.025	1.050	1.041	1.013	0.992	P41223	BUD31	Protein BUD31 homolog
0.943	0.944	1.006	1.010	1.027	1.035	0.900	1.029	1.009	1.002	1.044	1.017	Q99622	C12orf57	Protein C10
1.083	0.987	1.057	0.994	1.370	0.965	0.981	0.933	1.155				Q9NQ89	C12orf4	Protein C12orf4
1.047	1.003	1.036	1.154	0.955	1.045	1.107	0.869	1.082	1.033	1.013	1.155	Q96NL8	C8orf37	Protein C8orf37
1.056	1.006	0.996	1.024	0.902	1.025	0.989	0.943	1.008	1.024	0.993	0.989	Q9BT09	CNPY3	Protein canopy homolog 3
0.938	1.032	0.974	0.988	0.919	1.073	1.000	0.903	0.979	0.937	0.735	1.091	Q8N129	CNPY4	Protein canopy homolog 4
1.061	1.038	1.039	0.919	0.949	0.970	0.905	0.918	1.021	1.108	0.969	1.114	I3L2J0	CIC	Protein capicua homolog
1.013	0.988	1.035	1.020	1.029	1.050	0.996	0.923	1.035	1.060	1.038	1.209	O15234	CASC3	Protein CASC3
0.969	0.995	1.023	1.003	0.942	0.987	1.033	1.043	0.970	1.087	1.022	1.121	Q13948	CUX1	Protein CASP
1.046	0.945	1.035	0.976	0.841	0.832	0.851	1.042	0.916	0.880	1.114	0.968	A0A0A0MSU1	RUNX1T1	Protein CBFA2T1
			0.914	0.814	1.178	1.520	1.102	0.937	1.067	1.214	0.938	Q4G0I0	CCSMST1	Protein CCSMST1
0.969	0.989	0.978	0.987	0.964	0.943	1.009	1.029	1.007	1.002	0.993	1.048	Q9UKY7	CDV3	Protein CDV3 homolog
			0.982	0.937	1.054	0.979	1.011	0.970	1.118	1.048	1.227	A8MTT3	CEBPZOS	Protein CEBPZOS
0.981	0.958	0.990	1.050	0.923	1.052	0.990	0.911	0.994	1.028	0.994	1.091	Q96SW2	CRBN	Protein cereblon
1.040	1.052	1.077	1.018	0.959	1.006	1.004	1.141	0.953	0.962	0.997	0.966	P14921	ETS1	Protein C-ets-1
0.922	0.983	0.979	1.004	1.116	1.055	1.084	0.931	1.020	1.040	1.004	1.080	Q8WUH1	CHURC1	Protein Churchill
0.977	1.044	1.024	1.007	0.947	0.989	1.095	0.941	1.006	0.933	1.092	1.290	A0A087X211	KIAA1524	Protein CIP2A
1.016	0.984	1.055	0.979	0.900	0.888	0.951	0.711	1.118	1.156	1.009	1.032	Q2KHT3	CLEC16A	Protein CLEC16A
1.098	0.992	1.065	0.874	0.981	0.929	0.999	1.143	0.986	1.045	1.025	1.028	Q9UBY8	CLN8	Protein CLN8
1.016	1.006	0.976	1.043	1.110	1.105	1.083	1.103	1.055	1.047	1.053	1.046	Q9BQ75	CMSS1	Protein CMSS1
0.922	0.929	0.933	0.992	0.884	1.065	0.946	1.158	1.017	0.937	1.053	1.068	Q9P003	CNIH4	Protein cornichon homolog 4
1.018	1.481	0.984	0.991	0.721	1.180	1.101	1.275	1.079				O75629	CREG1	Protein CREG1
0.973	0.906	0.901	0.934	0.913	0.920	0.934	0.946	0.891	0.813	0.958	0.784	O00622	CYR61	Protein CYR61
1.174	0.969	1.110	1.018	0.824	1.012	0.782	0.926	0.874	0.683	0.825	0.571	Q8NFT6	DBF4B	Protein DBF4 homolog B
1.021	0.953	1.014	1.017	0.963	0.988	0.975	0.997	1.054	1.005	1.004	1.085	Q5TDH0	DDI2	Protein DDI1 homolog 2
0.994	0.979	0.975	0.972	0.927	0.931	0.942	0.992	0.934	0.934	0.971	0.912	P35659	DEK	Protein DEK
1.095	1.110	1.087	1.351	0.696	1.082	9.736	0.093	0.873	0.966	0.950	1.050	Q8IWF6	DENND6A	Protein DENND6A
0.973	0.941	1.020	1.089	1.193	1.057	1.021	0.945	1.022	1.010	1.067	1.094	Q96DF8	DGCR14	Protein DGCR14

Abundance Ratio														
1 d M1/C1	1 d M2/C2	1 d M3/C3	4 d M1/C1	4 d M2/C2	4 d M3/C3	7 d M1/C1	7 d M2/C2	7 d M3/C3	10 d M1/C1	10 d M2/C2	10 d M3/C3	Accession ID	Gene name	Protein Description
0.959	0.978	0.966	1.011	0.892	1.182	0.994	0.827	1.107	1.037	1.026	1.103	Q14129	DGCR6	Protein DGCR6
0.999	1.010	1.012	1.037	0.992	1.009	1.012	0.967	0.994	1.003	0.892	1.047	A0A0G2JH68	DIAPH1	Protein diaphanous homolog 1
			1.168	1.304	0.974	1.305	0.792	0.896	1.048	1.059	1.110	C9J6U3	DIAPH2	Protein diaphanous homolog 2
0.931	0.983	0.923				1.099	1.251	1.238	0.846	0.894	1.229	Q9NSV4	DIAPH3	Protein diaphanous homolog 3
1.008	1.026	0.996	0.976	0.970	0.925	0.985	1.056	0.964	0.974	0.981	0.989	H7BZJ3	PDIA3	Protein disulfide-isomerase A3 (Fragment)
1.024	1.021	0.990	1.010	0.983	0.987	0.969	1.011	0.964	0.968	0.996	0.942	P30101	PDIA3	Protein disulfide-isomerase A3
0.983	1.020	0.979	1.006	0.973	0.993	0.982	1.029	0.972	0.976	1.008	0.940	P13667	PDIA4	Protein disulfide-isomerase A4
1.018	1.010	1.006	1.001	0.998	0.981	0.996	1.044	1.017	0.988	0.972	1.022	Q14554	PDIA5	Protein disulfide-isomerase A5
0.998	1.007	1.000	1.003	1.007	1.026	0.995	1.031	0.999	0.959	0.979	0.986	P07237	P4HB	Protein disulfide-isomerase
0.910	0.976	0.981	0.973	0.923	1.006	1.014	1.020	1.003	1.023	1.042	1.010	Q96JJ7	TMX3	Protein disulfide-isomerase TMX3
1.055	0.994	0.982	1.022	1.117	0.989	1.008	0.943	0.996	0.967	1.015	0.925	Q99497	PARK7	Protein DJ-1
1.145	0.967	1.028	1.050	0.942	0.994	1.039	1.087	1.169	1.028	0.926	1.055	Q9Y3R5	DOPEY2	Protein dopey-2
1.039	0.971	1.047	1.019	0.891	1.061	1.038	1.026	0.991	1.083	1.006	1.041	Q9BVM2	DPCD	Protein DPCD
1.002	1.121	1.029	0.945	1.048	1.015	0.866	0.982	0.948	0.960	0.978	0.887	Q9C005	DPY30	Protein dpy-30 homolog
1.013	0.960	0.994	1.021	1.019	1.042	0.969	1.105	1.076	0.980	1.019	1.010	Q01658	DR1	Protein Dr1
0.980	1.161	1.175	0.985	0.949	1.044	1.026	1.089	1.067	0.957	0.946	0.897	Q9H8V3	ECT2	Protein ECT2
0.937	1.004	1.051	0.993	0.913	1.048	1.002	1.071	0.989	0.979	0.980	1.060	A0A1B0GUZ7	EFR3A	Protein EFR3 homolog A
0.869	1.129	1.082	1.070	1.109	1.178	1.126	1.024	1.113				P0C7U0	ELFN1	Protein ELFN1
1.117	0.771	0.935										A0A075B6E5	ENAH	Protein enabled homolog (Fragment)
1.012	1.016	0.998	0.991	0.991	1.004	0.965	0.947	0.978	0.963	1.026	0.998	A0A0U1RRM6	ENAH	Protein enabled homolog
1.002	1.052	0.981	0.967	1.039	1.028	0.959	0.930	0.961	1.086	1.037	1.063	Q03111	MLLT1	Protein ENL
0.940	1.010	0.994	1.006	0.954	0.988	0.980	1.022	0.989	1.009	1.023	0.991	P49257	LMAN1	Protein ERGIC-53
			0.910	1.017	1.070							Q9H8M9	EVA1A	Protein eva-1 homolog A
			1.223	0.822	0.938							Q9NVM1	EVA1B	Protein eva-1 homolog B
1.066	1.086	1.091							1.192	0.975	0.948	Q5T1H1	EYS	Protein eyes shut homolog
1.059	1.022	1.017	1.052	0.924	0.914	1.053	1.113	1.018	0.944	0.876	1.026	Q96PZ2	FAM111A	Protein FAM111A
0.999	0.881	1.131	1.402	0.683	1.490	1.137	0.992	0.973	1.077	1.009	1.117	Q6SJ93	FAM111B	Protein FAM111B
1.031	1.010	1.034	0.989	0.998	1.022	0.983	1.009	1.025	0.986	1.040	1.034	Q9NRY5	FAM114A2	Protein FAM114A2
0.969	0.915	1.073	1.030	0.916	1.015	0.960	1.023	1.017	0.931	0.983	1.041	Q6P1L5	FAM117B	Protein FAM117B
1.021	1.043	0.997	0.990	1.000	0.994	1.062	0.977	1.085	1.037	1.072	1.051	Q9NWS6	FAM118A	Protein FAM118A
1.004	0.982	1.016	0.959	0.949	1.012	0.953	1.035	1.025	1.064	1.043	1.023	Q9BPY3	FAM118B	Protein FAM118B
			0.962	0.753	1.063	0.955	1.185	0.988				Q96E09	FAM122A	Protein FAM122A
1.157	0.972	1.081	0.965	0.993	1.247	1.050	1.086	1.078	0.987	0.975	1.036	G1UD79	SPACIA2	Protein FAM122B
			1.051	0.786	1.030							Q8IXS8	FAM126B	Protein FAM126B

Abundance Ratio														
1 d M1/C1	1 d M2/C2	1 d M3/C3	4 d M1/C1	4 d M2/C2	4 d M3/C3	7 d M1/C1	7 d M2/C2	7 d M3/C3	10 d M1/C1	10 d M2/C2	10 d M3/C3	Accession ID	Gene name	Protein Description
1.145	1.051	1.006	1.021	1.046	0.988	0.957	1.061	0.936	0.967	1.082	0.911	A6ZKI3	FAM127A	Protein FAM127A
									0.764	1.129	0.809	Q9BWD3	FAM127B	Protein FAM127B
1.046	0.989	0.962	1.008	0.960	1.008	1.009	0.975	1.013	0.886	0.955	1.083	Q5BKY9	FAM133B	Protein FAM133B
1.005	0.844	1.035	1.003	0.862	0.973	0.993	0.845	0.998	1.109	0.980	1.050	Q8NC44	FAM134A	Protein FAM134A
1.038	0.992	1.078	1.036	0.903	1.115	1.058	1.015	1.004	1.136	1.004	1.090	Q86VR2	FAM134C	Protein FAM134C
0.898	0.991	1.106	0.995	0.799	1.176							Q9P2D6	FAM135A	Protein FAM135A
0.901	0.951	0.998	0.947	1.073	0.999	1.013	0.725	0.964	1.021	0.980	1.018	Q96C01	FAM136A	Protein FAM136A
						1.000	1.105	1.137	0.959	0.911	0.981	Q05DH4	FAM160A1	Protein FAM160A1
1.043	1.034	1.072	0.949	0.919	1.036	0.974	1.105	1.040	0.943	0.979	0.983	Q5W0V3	FAM160B1	Protein FAM160B1
1.002	0.929	1.067				0.935	1.041	1.111				Q86V87	FAM160B2	Protein FAM160B2
1.092	0.996	1.001	0.959	0.952	0.938	0.949	1.032	0.951	0.883	1.014	0.901	Q96A26	FAM162A	Protein FAM162A
1.063	1.036	1.014	1.082	1.032	1.014	1.037	0.959	0.878	1.167	1.020	0.887	Q92567	FAM168A	Protein FAM168A
			0.952	0.852	1.037							Q5VUB5	FAM171A1	Protein FAM171A1
1.015	1.016	0.998	0.994	0.994	1.019	1.004	1.006	1.129	1.048	0.996	1.014	Q8WUF8	FAM172A	Protein FAM172A
0.831	0.726	0.891	1.001	0.923	0.939	1.029	0.961	1.104				Q6P4H8	FAM173B	Protein FAM173B
1.020	1.029	1.006	1.040	1.040	1.057	1.089	0.989	1.060	1.073	1.000	1.082	Q9GZU8	FAM192A	Protein FAM192A
									1.010	1.066	0.907	A0A1B0GVL4	FAM193A	Protein FAM193A
0.866	1.034	1.206										Q96PV7	FAM193B	Protein FAM193B
1.380	0.687	1.057										Q6PEV8	FAM199X	Protein FAM199X
1.000	1.019	1.032	1.025	0.967	1.071	0.968	1.080	1.000	0.981	1.027	1.027	Q9NSI2	FAM207A	Protein FAM207A
0.916	1.015	1.280	1.006	0.935	1.067	1.061	1.023	0.906	1.073	1.142	0.963	Q5VWN6	FAM208B	Protein FAM208B
1.122	1.104	0.966	1.012	0.942	0.934	0.983	1.135	0.979	0.955	0.982	0.993	Q96ND0	FAM210A	Protein FAM210A
1.064	1.026	0.935	1.064	0.859	1.043	1.076	1.140	0.972	1.065	1.090	0.967	Q96KR6	FAM210B	Protein FAM210B
1.009	0.996	1.009	1.055	0.953	1.094	1.086	1.013	0.996	1.146	1.017	1.178	Q9H0X4	FAM234A	Protein FAM234A
			1.066	0.964	1.106	1.170	1.040	1.096	1.147	1.068	1.192	A2RU67	FAM234B	Protein FAM234B
1.193	0.965	1.030	0.869	1.005	1.139	1.028	1.095	0.986	1.175	0.911	1.028	Q9Y421	FAM32A	Protein FAM32A
0.926	0.996	0.948	0.967	0.935	1.034	0.993	1.108	0.963	0.986	1.027	0.980	Q92520	FAM3C	Protein FAM3C
1.053	0.939	1.041	1.050	0.914	0.986	1.003	1.083	0.999	0.995	0.964	0.977	Q8TCE6	FAM45A	Protein FAM45A
0.993	1.003	1.012	0.996	0.886	1.014	0.993	0.998	0.968	1.115	1.071	1.045	Q9NUQ9	FAM49B	Protein FAM49B
0.924	0.998	0.975	0.972	0.964	1.006	1.003	1.029	1.008	0.980	1.006	0.986	Q14320	FAM50A	Protein FAM50A
1.027	1.037	1.038	1.007	1.082	1.136	1.065	0.955	1.061	1.175	1.053	1.104	Q8TBR7	FAM57A	Protein FAM57A
0.763	1.754	1.026	1.189	0.915	0.868	0.832	1.094	0.991	0.971	0.932	1.019	Q9NP50	FAM60A	Protein FAM60A
									1.031	0.999	1.333	Q8TAV0	FAM76A	Protein FAM76A
1.023	0.925	1.037	1.000	0.902	1.136	0.954	0.880	0.958	1.084	0.942	1.108	Q5HYJ3	FAM76B	Protein FAM76B

Abundance Ratio														
1 d M1/C1	1 d M2/C2	1 d M3/C3	4 d M1/C1	4 d M2/C2	4 d M3/C3	7 d M1/C1	7 d M2/C2	7 d M3/C3	10 d M1/C1	10 d M2/C2	10 d M3/C3	Accession ID	Gene name	Protein Description
0.943	1.024	0.956	0.975	1.005	1.108	0.978	0.892	1.019	1.015	0.921	1.114	Q5T0W9	FAM83B	Protein FAM83B
0.927	0.964	1.192	0.875	0.941	0.994	1.030	0.895	0.998	1.068	0.925	1.033	A6ND36	FAM83G	Protein FAM83G
1.043	1.020	1.002	1.154	0.990	0.999	0.989	1.079	1.005	0.994	0.911	1.055	Q6ZRV2	FAM83H	Protein FAM83H
1.072	1.043	1.012	1.011	0.950	0.998	1.029	1.096	0.982	1.008	0.996	1.013	Q96KN1	FAM84B	Protein FAM84B
1.017	1.054	1.004	0.886	0.860	0.958	0.890	1.020	0.952	0.896	0.901	1.152	Q96GI7	FAM89A	Protein FAM89A
1.080	1.023	1.027	1.001	0.918	1.105	0.991	0.989	1.055	1.265	1.374	1.040	Q8N5H3	FAM89B	Protein FAM89B
0.857	0.964	0.960	0.980	0.862	1.074	1.015	1.088	0.859	1.260	1.044	1.041	Q9UBU6	FAM8A1	Protein FAM8A1
1.020	0.973	1.030	0.975	1.034	1.017	1.003	0.965	1.013	1.045	0.971	1.034	Q658Y4	FAM91A1	Protein FAM91A1
1.313	0.871	0.836				0.999	1.115	1.167				A1XBS5	FAM92A	Protein FAM92A
1.032	0.973	1.022	1.001	1.022	0.994	1.002	1.030	0.977	0.991	1.028	1.040	Q8NCA5	FAM98A	Protein FAM98A
0.935	1.007	1.037	0.970	0.974	1.101	1.030	1.004	1.072	1.028	0.989	1.047	Q17RN3	FAM98C	Protein FAM98C
1.037	0.980	1.039	0.986	1.080	0.956	0.986	0.985	1.059	0.892	1.097	0.953	Q92636	NSMAF	Protein FAN
1.081	0.947	1.243										Q68CZ1	RPGRIP1L	Protein fantom
1.054	1.032	1.011	1.057	1.005	0.967	1.005	1.099	0.976	1.028	1.002	0.984	P49356	FNTB	Protein farnesyltransferase subunit beta
1.024	0.979	0.998	0.983	1.015	1.076	1.029	1.048	1.044	1.029	0.963	1.053	P49354	FNTA	Protein farnesyltransferase/geranylgeranyltransferase type-1 subunit alpha
0.935	0.996	1.147	0.917	1.035	1.132	1.028	0.834	0.959				Q9UK73	FEM1B	Protein fem-1 homolog B
1.081	0.966	1.064	1.061	0.995	0.978	1.038	1.346	0.928	0.966	1.015	0.971	J3KS54	FLII	Protein flightless-1 homolog
0.987	1.000	1.006	0.999	0.991	1.018	1.012	1.015	1.020	1.014	0.983	1.043	Q13045	FLII	Protein flightless-1 homolog
1.053	1.058	1.078	1.041	1.023	1.012	1.025	0.966	0.902	1.078	1.035	1.126	Q96HJ9	FMC1	Protein FMC1 homolog
			1.279	1.009	0.893				1.033	1.153	1.093	Q70Z53	FRA10AC1	Protein FRA10AC1
1.042	0.984	0.993	1.033	1.045	1.006	1.042	0.966	1.016	1.025	1.041	1.024	Q14331	FRG1	Protein FRG1
1.004	0.989	1.009	0.970	0.964	1.000	0.973	1.048	0.993	0.997	0.970	1.066	O94915	FRYL	Protein furry homolog-like
0.994	0.962	1.015	0.991	0.945	1.048	0.990	0.861	0.986	1.026	1.004	1.022	O94992	HEXIM1	Protein HEXIM1
1.027	0.993	1.040	1.014	0.905	0.978	1.064	1.060	1.072	1.047	1.036	1.055	Q9BTY7	HGH1	Protein HGH1 homolog
			0.999	0.980	0.945							Q8IV36	HID1	Protein HID1
1.017	1.012	1.008	1.036	1.036	0.964	1.033	1.112	1.026	1.015	1.017	1.010	Q53FT3	HIKESHI	Protein Hikeshi
1.076	0.970	1.064	0.963	0.928	1.105	1.032	0.998	0.987				P54198	HIRA	Protein HIRA
1.055	0.969	1.058	1.023	0.984	1.004	0.974	1.009	1.016	0.990	0.975	1.075	Q9UJC3	HOOK1	Protein Hook homolog 1
1.018	1.005	1.043	0.974	0.968	1.036	0.999	1.010	1.013	1.047	1.007	1.054	Q86VS8	HOOK3	Protein Hook homolog 3
0.988	0.978	1.065	1.038	0.929	1.036	0.969	1.114	1.024	1.083	1.014	1.139	Q9P2X3	IMPACT	Protein IMPACT
0.986	1.001	1.012	0.977	0.971	1.005	0.988	0.995	0.986	0.987	1.002	1.049	Q96ST2	IWS1	Protein IWS1 homolog
1.144	1.027	1.089	1.050	0.954	1.089	1.002	0.988	1.142	0.969	1.095	1.039	Q6IE81	JADE1	Protein Jade-1
0.951	1.055	0.634	0.780	1.075	1.302				1.091	0.908	1.097	Q92613	JADE3	Protein Jade-3

Abundance Ratio														
1 d M1/C1	1 d M2/C2	1 d M3/C3	4 d M1/C1	4 d M2/C2	4 d M3/C3	7 d M1/C1	7 d M2/C2	7 d M3/C3	10 d M1/C1	10 d M2/C2	10 d M3/C3	Accession ID	Gene name	Protein Description
1.156	1.279	1.045							0.866	0.835	0.889	P78504	JAG1	Protein jagged-1
0.911	0.982	1.048	0.968	0.899	1.022	0.975	0.973	1.032	1.077	1.028	1.103	Q8N5M9	JAGN1	Protein jagunal homolog 1
1.010	1.323	1.055										Q9H799	C5orf42	Protein JBTS17
						1.334	0.874	1.087	1.123	1.019	0.954	O76095	JTB	Protein JTB
1.044	0.968	1.100	1.072	0.958	1.032	1.027	1.072	1.014	1.073	1.049	1.064	O15037	KHNYN	Protein KHNYN
1.088	1.097	1.116	0.937	0.956	1.041	0.953	0.979	0.991	0.939	0.965	1.071	Q14667	KIAA0100	Protein KIAA0100
			1.119	0.957	0.971							O60303	KIAA0556	Protein KIAA0556
0.959	1.001	1.027	0.984	0.956	1.039	0.928	0.942	0.996	0.999	1.041	0.985	P17252	PRKCA	Protein kinase C alpha type
0.951	0.986	0.991	0.991	0.989	0.975	0.995	0.962	0.988	1.013	0.955	1.029	Q9UNF0	PACSIN2	Protein kinase C and casein kinase substrate in neurons protein 2
1.032	0.969	1.035	1.008	0.957	0.987	0.984	0.994	1.022	1.007	0.976	1.045	Q9UKS6	PACSIN3	Protein kinase C and casein kinase substrate in neurons protein 3
0.980	1.005	1.017	0.977	0.975	0.997	0.979	0.990	1.000	1.003	0.964	0.971	Q05655	PRKCD	Protein kinase C delta type
1.020	0.920	0.825	0.934	0.855	1.097	0.946	0.897	1.063	1.087	0.934	1.190	Q02156	PRKCE	Protein kinase C epsilon type
1.043	0.858	1.034	1.032	0.979	1.014	1.002	0.996	0.982	1.032	1.007	1.029	P41743	PRKCI	Protein kinase C iota type
0.965	1.017	1.042	0.999	0.941	0.978	1.003	1.000	1.055	1.021	0.978	1.049	Q05513	PRKCZ	Protein kinase C zeta type
0.962	0.948	0.997	0.993	0.871	1.060	0.901	1.217	0.920	0.963	1.045	1.036	Q9NVR5	DNAAF2	Protein kintoun
0.783	0.995	0.895	0.876	0.872	1.037	0.978	1.075	0.999	0.928	0.902	0.947	Q8TBQ9	TMEM167A	Protein kish-A
1.006	1.050	1.055	1.029	0.908	0.996	1.074	1.085	1.053	0.990	1.036	1.002	Q9NRX6	TMEM167B	Protein kish-B
1.022	0.981	1.009	0.976	0.943	1.010	1.010	0.998	1.023	1.018	1.007	1.071	Q8N9T8	KRI1	Protein KRI1 homolog
1.064	1.018	0.970	1.035	0.963	1.046	1.050	1.103	0.972	1.088	0.999	1.005	Q96EK9	KTI12	Protein KTI12 homolog
1.001	0.828	1.070	1.029	1.039	1.090				0.822	0.574	1.063	A4D1U4	LCHN	Protein LCHN
0.992	1.013	1.021	1.028	0.933	0.999	1.074	1.045	1.033	1.053	1.152	1.095	Q969X1	TMBIM1	Protein lifeguard 3
									1.029	1.053	1.197	Q96GY3	LIN37	Protein lin-37 homolog
			1.095	0.960	1.117							Q52LA3	LIN52	Protein lin-52 homolog
1.094	1.082	0.981	1.040	0.943	0.912	1.120	1.083	1.046	0.910	0.974	0.967	Q6MZP7	LIN54	Protein lin-54 homolog
0.962	0.946	0.996	0.964	0.982	1.019	1.012	0.957	1.025	1.028	1.004	1.030	Q9NUP9	LIN7C	Protein lin-7 homolog C
1.063	0.934	1.142	1.097	1.054	1.054	1.140	1.047	1.136	0.855	1.024	0.931	H0Y322	LIN9	Protein lin-9 homolog (Fragment)
1.022	1.050	1.043	1.154	1.097	1.112	1.282	1.537	1.200	1.200	1.175	1.125	Q9BRT6	LLPH	Protein LLP homolog
1.000	0.998	1.007	0.995	0.972	0.984	1.000	0.971	1.017	1.107	1.053	1.021	Q8ND56	LSM14A	Protein LSM14 homolog A
0.961	1.035	0.915	1.026	1.069	1.015	0.998	1.141	1.029	0.939	1.007	1.035	Q5TBP9	LSM14B	Protein LSM14 homolog B (Fragment)
1.013	1.027	1.008	1.005	1.004	1.103	1.009	1.069	1.085	1.026	1.064	1.128	Q9BX40	LSM14B	Protein LSM14 homolog B
0.984	0.979	0.987	0.988	0.984	1.029	0.952	1.021	1.039	1.006	1.045	1.093	Q96GA3	LTV1	Protein LTV1 homolog
0.950	0.982	1.017	0.980	0.927	0.990	1.003	1.000	0.981	1.060	1.038	1.047	Q86UE4	MTDH	Protein LYRIC
1.065	0.919	1.002	1.066	0.919	1.066	1.047	1.002	0.993	1.073	0.979	0.966	Q96A72	MAGOHB	Protein mago nashi homolog 2

Abundance Ratio														
1 d M1/C1	1 d M2/C2	1 d M3/C3	4 d M1/C1	4 d M2/C2	4 d M3/C3	7 d M1/C1	7 d M2/C2	7 d M3/C3	10 d M1/C1	10 d M2/C2	10 d M3/C3	Accession ID	Gene name	Protein Description
1.012	1.002	0.990	1.037	1.011	1.003	1.040	1.063	1.039	1.067	1.060	0.969	Q9BXY0	MAK16	Protein MAK16 homolog
1.036	0.916	1.067	0.990	0.851	1.035	1.000	0.908	0.870	1.094	1.075	1.062	Q9NQG1	MANBAL	Protein MANBAL
1.111	0.980	1.045	1.108	0.938	1.033	1.068	0.868	0.875	0.759	0.735	0.797	P61244	MAX	Protein max
1.030	1.020	1.095	0.988	0.869	1.118	1.297	0.909	1.204				Q8IYB1	MB21D2	Protein MB21D2
1.059	0.978	1.060	0.984	0.862	1.091	1.084	1.058	0.952	0.988	1.058	0.978	Q9H081	MIS12	Protein MIS12 homolog
1.015	1.020	1.010	0.960	0.972	1.057	1.003	1.002	1.034	1.168	0.930	1.171	Q9BUK6	MSTO1	Protein misato homolog 1
0.998	0.967	0.902	0.879	0.941	0.978	1.024	0.974	1.182	0.874	0.801	0.892	Q6ZRQ5	MMS22L	Protein MMS22-like
1.005	0.972	1.009	1.003	0.921	1.060	0.997	1.102	1.021	0.972	0.988	1.004	Q7Z3U7	MON2	Protein MON2 homolog
0.978	0.956	1.008	0.982	0.979	1.018	0.980	0.964	0.972	1.091	1.049	1.054	P39210	MPV17	Protein Mpv17
1.095	0.960	0.912	1.040	0.958	1.017	1.175	1.315	1.343	0.884	0.946	1.667	Q8N6N6	NATD1	Protein NATD1
0.983	1.062	0.984	1.022	0.989	0.956	0.969	1.049	0.925	0.881	0.977	0.902	Q92597	NDRG1	Protein NDRG1
1.023	1.002	0.986	0.992	0.958	0.929	0.988	1.118	0.969	0.966	0.974	1.000	Q9UGV2	NDRG3	Protein NDRG3
0.974	1.022	1.015	1.015	0.997	1.018	1.033	1.032	1.033	1.002	0.992	1.028	Q9BZQ8	FAM129A	Protein Niban
1.024	1.028	0.978	0.998	1.005	0.952	0.972	1.013	0.942	0.986	1.015	0.928	Q9BPW8	NIPSNAP1	Protein NipSnap homolog 1
1.008	0.997	0.977	1.002	0.974	0.984	0.994	0.989	0.995	1.010	0.998	0.951	O75323	GBAS	Protein NipSnap homolog 2
0.998	1.003	1.002	0.985	1.025	0.995	0.995	1.019	1.028	1.011	1.001	0.971	Q9UFN0	NIPSNAP3A	Protein NipSnap homolog 3A
1.056	0.969	1.038	0.980	1.000	1.029	0.975	0.959	1.023	1.005	1.026	1.068	J9JIC5	C17orf75	Protein Njmu-R1
1.012	1.002	1.031	0.993	0.962	0.976	0.942	0.989	0.980	0.947	0.960	1.033	Q8IWE2	FAM114A1	Protein NOXP20
1.182	1.063	1.061	1.057	1.236	0.900	0.993	1.089	1.029	1.081	0.984	0.998	Q9H7Z3	NRDE2	Protein NRDE2 homolog
0.989	1.003	1.000	0.963	0.892	1.045	1.035	1.049	1.059	1.062	0.948	1.171	Q96AB6	NTAN1	Protein N-terminal asparagine amidohydrolase
			0.818	0.983	1.042							Q96HA8	WDYHV1	Protein N-terminal glutamine amidohydrolase
1.013	1.007	1.012	0.982	0.983	1.015	1.048	1.035	0.997	1.075	0.988	1.077	P49757	NUMB	Protein numb homolog
1.138	0.964	1.021	1.011	0.934	1.058	1.021	0.891	0.933	1.111	1.012	1.069	Q5SWX8	ODR4	Protein odr-4 homolog
1.012	1.009	0.993	0.988	1.045	1.027	0.998	1.000	1.059	0.999	1.010	1.047	O60502	MGEA5	Protein O-GlcNAcase
1.045	1.026	1.013	1.018	0.984	1.057	1.044	1.105	1.096	1.049	0.987	1.103	Q8NBL1	POGLUT1	Protein O-glucosyltransferase 1
0.990	0.969	0.992	0.997	0.931	0.980	0.967	1.082	0.963	0.970	1.016	1.001	Q8WZA1	POMGNT1	Protein O-linked-mannose beta-1,2-N-acetylglucosaminyltransferase 1
1.042	1.034	0.984	1.017	0.948	1.015	1.015	1.148	0.995	0.935	0.988	1.005	Q8NAT1	POMGNT2	Protein O-linked-mannose beta-1,4-N-acetylglucosaminyltransferase 2
0.977	0.997	0.918	1.017	0.906	0.934	0.985	1.069	1.052	1.061	0.867	0.877	Q9H5K3	POMK	Protein O-mannose kinase
						1.171	0.766	0.889	1.379	1.018	1.196	Q9Y6A1	POMT1	Protein O-mannosyl-transferase 1
			1.023	0.911	1.032	1.089	1.049	0.923				Q9UKY4	POMT2	Protein O-mannosyl-transferase 2
1.016	0.994	1.029	0.987	0.996	1.054	0.950	0.871	1.024	1.016	1.037	1.070	Q13438	OS9	Protein OS-9
1.139	1.236	1.183	0.998	0.901	0.996	1.089	1.012	1.083	0.815	1.040	0.746	Q8WVF1	OSCP1	Protein OSCP1
1.027	1.034	1.057	1.024	0.982	1.031	0.996	1.105	0.997	0.992	1.024	1.013	Q86TB9	PATL1	Protein PAT1 homolog 1

Abundance Ratio														
1 d M1/C1	1 d M2/C2	1 d M3/C3	4 d M1/C1	4 d M2/C2	4 d M3/C3	7 d M1/C1	7 d M2/C2	7 d M3/C3	10 d M1/C1	10 d M2/C2	10 d M3/C3	Accession ID	Gene name	Protein Description
1.015	0.973	1.015	0.994	0.979	1.056	1.037	0.960	1.033	1.028	1.004	1.081	Q9BUH6	C9orf142	Protein PAXX
1.014	0.987	1.018	0.970	0.952	0.999	0.982	0.971	0.997	0.998	1.011	1.031	Q9BVG4	PBDC1	Protein PBDC1
1.015	0.967	1.025	1.010	0.929	1.022	1.038	0.905	0.986	1.068	0.938	1.064	Q9BRX2	PELO	Protein pelota homolog
0.962	0.946	1.018	0.904	0.943	1.145	1.012	0.973	0.991	1.012	0.947	1.056	P0DJ07	PET100	Protein PET100 homolog, mitochondrial
						0.741	0.839	1.182	0.953	0.944	0.975	Q6UWS5	PET117	Protein PET117 homolog, mitochondrial
0.956	1.059	1.066	0.819	1.152	0.900				1.059	0.857	1.099	O60927	PPP1R11	Protein phosphatase 1 regulatory subunit 11
0.995	0.995	1.008	0.984	0.980	0.997	0.992	0.937	0.989	1.029	0.997	1.033	O14974	PPP1R12A	Protein phosphatase 1 regulatory subunit 12A
0.985	0.964	1.054	0.884	0.895	0.976	1.105	0.910	0.929	1.038	1.126	1.154	Q9BZL4	PPP1R12C	Protein phosphatase 1 regulatory subunit 12C
0.994	1.005	1.003	0.982	1.186	0.979	1.130	0.968	1.158	0.996	1.111	0.961	Q96C90	PPP1R14B	Protein phosphatase 1 regulatory subunit 14B
0.871	0.831	0.985	1.018	0.835	1.001	1.169	0.891	0.979	1.200	1.243	1.424	Q96I34	PPP1R16A	Protein phosphatase 1 regulatory subunit 16A
0.963	0.986	1.025	0.983	0.969	1.051	1.003	1.000	1.029	1.034	0.980	1.087	Q6ZMI0	PPP1R21	Protein phosphatase 1 regulatory subunit 21
			1.120	1.087	1.062	0.850	1.013	0.909	0.944	1.005	1.010	O75864	PPP1R37	Protein phosphatase 1 regulatory subunit 37
0.964	1.028	1.019	0.949	0.994	0.980	0.929	0.996	1.041	1.047	1.010	1.062	O95685	PPP1R3D	Protein phosphatase 1 regulatory subunit 3D
1.041	0.989	1.007	0.994	1.033	1.007	1.008	1.061	1.028	0.993	0.969	0.992	Q15435	PPP1R7	Protein phosphatase 1 regulatory subunit 7
0.973	0.969	0.960	1.012	0.990	0.967	1.052	1.034	0.990	1.054	1.040	1.025	O75688	PPM1B	Protein phosphatase 1B
1.124	0.961	1.047	1.063	0.944	1.034	1.072	0.898	1.030	1.008	0.988	1.046	P49593	PPM1F	Protein phosphatase 1F
0.988	0.985	1.007	1.005	1.031	1.007	1.034	0.981	1.010	1.040	0.976	1.077	O15355	PPM1G	Protein phosphatase 1G
									0.717	0.993	0.907	Q5SGD2	PPM1L	Protein phosphatase 1L
1.015	0.955	1.022	1.023	0.965	1.026	0.980	1.021	1.003	0.996	0.933	1.035	P41236	PPP1R2	Protein phosphatase inhibitor 2
0.986	0.991	0.976	0.980	1.009	0.967	1.002	0.973	1.030	1.010	0.995	1.024	Q9Y570	PPME1	Protein phosphatase methylesterase 1
1.076	0.929	1.124	1.009	1.058	0.986	1.041	0.886	1.008	1.283	1.233	1.151	Q8NI37	PPTC7	Protein phosphatase PTC7 homolog
0.957	0.923	1.127	1.063	0.936	0.976	1.051	1.009	1.068	0.956	1.020	1.016	Q8WYL5	SSH1	Protein phosphatase Slingshot homolog 1
1.024	1.000	0.992	0.994	0.978	0.992	1.028	1.061	0.963	1.063	0.981	1.058	Q8TE77	SSH3	Protein phosphatase Slingshot homolog 3
0.946	0.968	1.010	1.021	0.873	0.833	1.053	1.076	1.062	0.991	0.986	1.136	Q9BSJ6	PIMREG	Protein PIMREG
1.004	1.017	1.037	1.045	1.118	1.016	1.079	0.915	1.009	1.052	0.958	1.100	P29590	PML	Protein PML
1.001	1.003	1.003	0.997	0.969	0.997	1.025	0.994	0.980	1.032	1.026	1.022	Q86U86	PBRM1	Protein polybromo-1
1.118	1.021	1.027	0.985	0.942	1.117	0.948	1.112	1.078	0.935	1.021	1.005	Q7Z6K3	PTAR1	Protein prenyltransferase alpha subunit repeat-containing protein 1
0.925	0.899	1.061	0.917	0.905	0.978	0.849	0.689	0.933	1.034	0.980	1.025	Q96I23	PYURF	Protein preY, mitochondrial
0.978	1.002	1.008	0.990	1.000	1.011	0.951	1.003	1.002	0.953	0.951	0.998	Q96M27	PRRC1	Protein PRRC1
0.996	0.993	1.019	0.999	0.931	0.994	1.015	0.940	0.982	1.039	0.995	1.075	P48634	PRRC2A	Protein PRRC2A
0.977	0.989	1.023	1.044	0.988	0.996	1.021	1.057	1.064	1.094	1.136	1.112	Q5JSZ5	PRRC2B	Protein PRRC2B
1.083	1.095	1.029	1.081	0.872	1.132	1.081	1.073	0.968	0.901	1.243	0.857	Q3SYG4	BBS9	Protein PTHB1
1.095	1.064	1.062	1.005	0.947	0.958	0.937	1.216	0.951	0.888	1.078	0.888	Q96PU8	QKI	Protein quaking

Abundance Ratio														
1 d M1/C1	1 d M2/C2	1 d M3/C3	4 d M1/C1	4 d M2/C2	4 d M3/C3	7 d M1/C1	7 d M2/C2	7 d M3/C3	10 d M1/C1	10 d M2/C2	10 d M3/C3	Accession ID	Gene name	Protein Description
0.963	0.987	0.975	0.978	1.030	1.000	0.928	0.904	0.997	0.973	1.006	0.992	Q9P258	RCC2	Protein RCC2
0.993	0.991	0.983	0.992	0.957	1.039	1.023	0.995	0.974	1.013	1.013	1.016	Q13123	IK	Protein Red
0.942	1.038	1.045	1.077	1.019	1.090	1.057	1.193	1.186	0.940	0.942	1.151	O43663	PRC1	Protein regulator of cytokinesis 1
0.976	0.966	0.938	0.986	0.884	0.985	1.026	1.071	1.066	1.026	1.005	0.881	O15258	RER1	Protein RER1
0.950	0.986	0.999	0.999	0.910	1.075	1.022	1.148	1.030	1.092	0.990	1.009	Q96AA3	RFT1	Protein RFT1 homolog
1.118	1.001	1.012	1.000	0.973	1.047	0.993	1.064	1.067	0.991	0.985	1.037	Q9H871	RMND5A	Protein RMD5 homolog A
1.088	0.806	1.471										Q96G75	RMND5B	Protein RMD5 homolog B
			0.990	0.976	1.064	0.882	1.335	1.043				Q9GZN7	ROGDI	Protein rogdi homolog
						1.107	1.511	1.044				Q96FB5	RRNAD1	Protein RRNAD1
0.995	1.013	1.015	0.997	0.949	1.005	0.979	1.034	1.005	1.002	1.032	1.019	Q14690	PDCD11	Protein RRP5 homolog
0.989	0.995	1.010	0.988	1.020	1.069	0.975	0.943	0.977	1.016	1.061	1.071	A0A0A0MQR2	RTFDC1	Protein RTF2 homolog
1.083	1.020	1.050	1.042	1.099	1.077	1.009	0.961	1.036	1.003	1.012	1.001	P60903	S100A10	Protein S100-A10
0.970	1.026	0.959	1.030	1.251	1.043	1.001	0.998	1.106	0.991	1.005	0.999	P31949	S100A11	Protein S100-A11
1.011	0.988	0.978	0.975	0.950	0.969	0.990	1.057	0.975	1.038	1.025	0.969	Q99584	S100A13	Protein S100-A13
1.013	1.021	1.037	0.990	1.031	1.044	0.942	1.093	1.019	1.019	1.017	1.066	Q96FQ6	S100A16	Protein S100-A16
						0.961	0.949	0.999				P29034	S100A2	Protein S100-A2
0.983	0.919	0.970	0.991	0.918	0.997	0.975	0.719	0.906	1.072	0.959	0.947	P06703	S100A6	Protein S100-A6
			1.116	0.902	1.026	2.334	1.017	0.688	0.976	0.884	0.971	P31151	S100A7	Protein S100-A7
			0.963	0.980	1.102	2.767	0.963	0.940	1.056	0.976	1.078	P05109	S100A8	Protein S100-A8
1.081	0.963	0.858	1.084	0.931	1.388	1.349	1.073	0.900	1.041	0.922	1.031	P06702	S100A9	Protein S100-A9
0.996	0.948	0.994	1.039	0.864	1.089	1.045	0.998	0.999	0.977	1.049	1.034	Q96ER3	SAAL1	Protein SAAL1
0.974	1.043	1.171	1.012	0.903	0.949	0.895	0.874	1.104	0.988	1.033	1.028	Q9H4B6	SAV1	Protein salvador homolog 1
0.985	0.969	1.011	0.965	0.994	1.024	0.988	0.943	0.971	1.003	0.984	1.043	Q99590	SCAF11	Protein SCAF11
1.043	1.009	0.997	0.973	0.963	1.011	1.030	1.086	1.014	0.981	1.008	1.024	A0A0A0MT33	SCAF8	Protein SCAF8
1.122	1.022	1.052	1.030	0.987	0.980	0.984	1.010	0.938	1.015	1.086	1.013	O75880	SCO1	Protein SCO1 homolog, mitochondrial
1.020	1.002	0.971	1.273	0.920	0.997	1.110	0.957	0.995	1.197	0.957	1.065	O43819	SCO2	Protein SCO2 homolog, mitochondrial
0.980	1.027	1.061	0.993	0.969	1.067							H0YDF9	SCRIB	Protein scribble homolog (Fragment)
						0.840	1.056	0.922				A0A0G2JPP5	SCRIB	Protein scribble homolog
0.958	0.994	1.018	0.990	0.934	1.011	1.004	1.002	1.000	1.032	1.012	1.064	A0A0G2JNZ2	SCRIB	Protein scribble homolog
1.036	1.022	1.034	1.053	0.970	1.029	1.020	1.021	1.027	0.974	1.041	1.004	Q9NVU7	SDAD1	Protein SDA1 homolog
1.000	0.984	1.026	0.991	0.944	1.017	1.004	1.006	1.007	1.005	1.005	1.031	Q6IQ49	SDE2	Protein SDE2 homolog
0.997	1.037	0.967	1.003	0.942	0.981	1.007	1.015	0.957	1.100	1.013	1.027	Q9UBV2	SEL1L	Protein sel-1 homolog 1
0.953	0.834	1.049	0.980	0.877	1.016	1.002	1.089	1.022	0.966	0.944	0.985	Q96JX3	SERAC1	Protein SERAC1
1.025	0.993	0.982	1.008	0.986	0.994	1.011	1.048	1.001	1.042	1.030	0.988	Q01105	SET	Protein SET

Abundance Ratio														
1 d M1/C1	1 d M2/C2	1 d M3/C3	4 d M1/C1	4 d M2/C2	4 d M3/C3	7 d M1/C1	7 d M2/C2	7 d M3/C3	10 d M1/C1	10 d M2/C2	10 d M3/C3	Accession ID	Gene name	Protein Description
1.031	1.010	0.995	1.017	0.982	0.971	0.991	1.016	1.035	0.991	1.011	0.993	Q9Y2Z0	SUGT1	Protein SGT1 homolog
			0.752	0.798	1.212	1.218	0.818	0.954				Q6UWI4	SHISA2	Protein shisa-2 homolog
1.284	1.262	1.062	0.934	0.945	0.966	0.943	0.908	0.990	1.096	1.011	1.144	Q2M3G4	SHROOM1	Protein Shroom1
1.166	1.095	1.065				1.149	0.994	0.971	0.999	0.988	1.041	Q8TF72	SHROOM3	Protein Shroom3
			1.138	1.001	1.002							A0A087WTQ6	SDK1	Protein sidekick-1
1.025	0.926	1.001				0.864	1.044	0.994	1.040	1.144	1.184	Q9NX38	FAM206A	Protein Simiate
1.029	1.029	1.085	0.996	0.926	0.967	0.989	1.074	1.010	1.104	1.015	1.082	Q9UPU9	SAMD4A	Protein Smaug homolog 1
1.038	1.047	1.005	0.987	1.028	1.043	1.002	1.127	1.078	0.970	0.991	1.027	Q5PRF9	SAMD4B	Protein Smaug homolog 2
0.904	0.946	1.032	0.954	0.952	1.040	0.964	1.023	1.048	0.964	0.994	1.007	Q9UPR3	SMG5	Protein SMG5
1.150	1.008	1.008	0.977	0.976	0.954	0.981	1.085	0.941	1.032	1.072	1.017	Q9H0W8	SMG9	Protein SMG9
0.978	0.944	1.040	0.894	0.829	0.742	1.052	1.004	0.989	1.071	1.018	1.062	Q5TF21	SOGA3	Protein SOGA3
0.982	0.950	0.969	1.091	0.829	1.035	1.045	0.956	1.034	1.106	1.028	1.053	H3BMF4	SPNS1	Protein spinster homolog 1
			0.976	0.934	0.932				1.078	1.067	1.393	O43597	SPRY2	Protein sprouty homolog 2
1.002	1.015	1.073	0.941	0.891	0.985	1.049	1.016	1.073	1.041	1.020	1.066	A0A0C4DFS6	SPRY4	Protein sprouty homolog 4
1.002	1.043	1.021	1.055	0.980	1.022	1.028	1.066	0.961	1.064	1.026	1.157	Q68D10	SPTY2D1	Protein SPT2 homolog
1.007	0.971	0.965	0.968	0.946	0.958	1.028	1.041	1.043	1.169	1.009	1.106	Q15532	SS18	Protein SSXT
1.011	0.997	1.047	0.990	0.977	1.021	0.973	1.027	1.012	0.988	1.019	1.046	A3KN83	SBNO1	Protein strawberry notch homolog 1
1.053	1.158	1.054	0.973	1.107	1.187	1.077	1.181	0.770	1.353	1.118	1.142	Q9Y2G9	SBNO2	Protein strawberry notch homolog 2
1.008	1.052	0.995	0.983	0.891	0.977	1.119	1.166	1.009	1.149	1.055	1.062	Q8N2H4	SYS1	Protein SYS1 homolog
1.066	1.077	1.156	1.010	0.961	1.009	1.020	1.073	0.952	0.971	0.971	1.079	Q5T011	SZT2	Protein SZT2
0.950	1.047	1.051	0.940	1.003	1.019	1.010	1.000	1.017	0.995	0.987	1.088	Q9C0D5	TANC1	Protein TANC1
1.036	1.025	1.065	0.981	0.957	0.995	1.015	1.049	1.028	1.040	0.986	1.037	Q9UK61	FAM208A	Protein TASOR
0.997	0.985	1.014	1.008	0.970	0.989	0.999	1.027	1.002	1.013	0.991	0.983	Q969Z0	TBRG4	Protein TBRG4
1.024	0.997	1.002	0.971	0.947	0.944	0.904	0.925	0.966	0.929	1.023	1.007	Q92734	TFG	Protein TFG
0.895	0.917	1.035	0.912	0.909	1.210	1.031	0.998	0.969	1.146	0.999	1.077	Q8WUY1	THEM6	Protein THEM6
1.037	1.075	1.097	0.982	0.930	1.020	0.933	1.116	1.010	0.975	0.981	0.985	Q5TEJ8	THEMIS2	Protein THEMIS2
0.948	0.960	1.018	1.051	0.870	0.842	1.151	1.201	0.946	0.850	0.858	0.979	Q9UNS1	TIMELESS	Protein timeless homolog
1.125	1.072	1.045	1.037	0.989	1.086	1.048	1.072	1.073	1.106	1.008	1.078	Q6PL24	TMED8	Protein TMED8
						0.700	1.228	0.877				F1T0I1	SEC16A	Protein transport protein Sec16A
0.965	1.004	0.997	1.006	0.958	1.002	1.000	1.029	0.988	1.030	0.973	1.041	J3KNL6	SEC16A	Protein transport protein Sec16A
0.978	1.001	0.982	0.981	1.037	1.012	0.975	0.965	1.011	0.984	0.983	0.986	Q15436	SEC23A	Protein transport protein Sec23A
0.976	0.982	1.038	1.020	0.957	1.062	1.017	1.074	1.000	0.966	0.987	1.004	Q15437	SEC23B	Protein transport protein Sec23B
1.019	1.006	1.016	1.009	0.992	1.053	0.994	1.126	1.040	1.001	1.025	1.002	O95486	SEC24A	Protein transport protein Sec24A
1.023	1.036	1.034	1.015	1.014	1.051	0.985	1.058	1.029	0.966	1.011	0.983	P53992	SEC24C	Protein transport protein Sec24C

Abundance Ratio														
1 d M1/C1	1 d M2/C2	1 d M3/C3	4 d M1/C1	4 d M2/C2	4 d M3/C3	7 d M1/C1	7 d M2/C2	7 d M3/C3	10 d M1/C1	10 d M2/C2	10 d M3/C3	Accession ID	Gene name	Protein Description
			1.013	1.025	0.950	1.048	0.962	1.002				H0YAB3	SEC31A	Protein transport protein Sec31A (Fragment)
1.011	1.017	1.006	0.997	0.990	0.970	0.989	1.020	0.984	0.975	0.993	0.960	D6REX3	SEC31A	Protein transport protein Sec31A
0.940	0.997	0.932	0.944	0.848	1.042	1.067	1.019	0.957	1.199	1.033	0.942	B4DR61	SEC61A1	Protein transport protein Sec61 subunit alpha isoform 1
									1.373	0.811	0.806	Q9H9S3	SEC61A2	Protein transport protein Sec61 subunit alpha isoform 2
0.900	0.986	0.979	0.960	0.938	1.052	0.990	0.954	0.960	1.015	1.012	0.965	P60468	SEC61B	Protein transport protein Sec61 subunit beta
1.213	0.818	1.171	0.917	0.862	0.944	0.856	1.322	0.977	0.885	0.931	1.014	P60059	SEC61G	Protein transport protein Sec61 subunit gamma
0.929	1.044	0.985	0.991	0.997	1.020	1.019	0.970	0.996	0.960	0.996	0.990	Q9Y5U2	TSSC4	Protein TSSC4
1.019	1.002	1.063	1.034	0.992	0.995	1.036	1.068	0.983	0.998	1.000	1.043	Q93096	PTP4A1	Protein tyrosine phosphatase type IVA 1
			1.139	1.831	0.997	1.168	0.744	1.068	1.053	1.011	1.050	Q12974	PTP4A2	Protein tyrosine phosphatase type IVA 2
0.931	0.905	1.030	0.903	0.885	1.139				1.062	0.996	1.105	Q13432	UNC119	Protein unc-119 homolog A
1.037	1.045	1.015	1.018	0.962	0.980	0.979	0.998	0.998	0.970	1.040	0.995	A6NIH7	UNC119B	Protein unc-119 homolog B
1.037	1.025	1.041	0.978	0.970	1.013	0.967	0.994	1.055	1.016	0.989	1.022	A0A1B0GUS7	UNC13B	Protein unc-13 homolog B
0.982	1.016	1.019	0.969	0.992	1.043	0.994	1.025	1.027	1.007	1.007	1.046	Q9H3U1	UNC45A	Protein unc-45 homolog A
0.952	0.954	0.943	0.935	0.823	0.974	1.036	1.085	0.997	1.118	1.025	1.045	Q9H1C4	UNC93B1	Protein unc-93 homolog B1
1.002	0.953	0.997	0.979	0.943	1.044	1.030	1.005	0.983	0.991	1.007	1.034	A0A0C4DFR8	UXT	Protein UXT
1.030	1.022	1.018	1.033	0.960	1.047	1.026	1.045	1.029	0.999	1.029	0.973	Q08AM6	VAC14	Protein VAC14 homolog
1.029	1.014	1.059	0.978	0.957	1.020	0.994	1.064	1.053	1.028	0.988	1.032	Q69YN4	KIAA1429	Protein virilizer homolog
1.030	0.996	1.032	1.025	0.989	0.977	1.000	1.034	0.951	1.015	1.024	1.019	M0QXA7	WIZ	Protein Wiz
									1.224	0.999	0.991	Q9H1J7	WNT5B	Protein Wnt-5b
0.947	1.004	0.979	0.996	0.933	1.012	0.999	0.984	0.980	1.017	1.029	1.087	O75695	RP2	Protein XRP2
0.956	1.057	0.929	0.949	0.942	1.012	0.943	1.079	0.979	0.975	1.022	0.907	O95070	YIF1A	Protein YIF1A
0.950	1.250	1.016	0.825	0.830	1.082	1.036	1.058	1.005				Q5BJH7	YIF1B	Protein YIF1B
0.984	0.980	0.996	1.006	0.950	0.930	1.159	1.095	1.043	1.031	1.094	0.943	Q9Y548	YIPF1	Protein YIPF1
0.867	0.976	0.979	1.000	0.868	1.023	1.031	1.024	1.000	1.014	1.022	1.113	E7EQR8	YIPF3	Protein YIPF3
0.964	0.969	1.020	0.919	0.936	1.035	1.042	1.074	0.907	0.951	0.982	1.001	Q9BSR8	YIPF4	Protein YIPF4
0.949	0.995	1.031	1.042	0.951	1.045	1.071	1.007	1.016	1.075	0.965	1.151	Q96EC8	YIPF6	Protein YIPF6
1.049	0.988	1.002	1.087	0.924	1.025	1.044	1.010	1.101	1.044	0.983	1.032	P62699	YPEL5	Protein yippee-like 5
0.830	0.970	0.894	0.913	1.005	1.208	0.822	0.961	1.008	0.629	0.875	1.049	Q7Z7L7	ZER1	Protein zer-1 homolog
1.039	0.994	1.015	0.978	0.925	1.014	1.029	1.043	0.990	0.927	0.954	1.037	Q9H900	ZWILCH	Protein zwilch homolog
0.988	0.933	1.078	0.872	0.893	1.044	0.960	1.128	1.074	0.923	0.969	1.071	Q9C0D3	ZYG11B	Protein zyg-11 homolog B
			0.996	1.250	0.849							Q9ULC6	PADI1	Protein-arginine deiminase type-1
0.955	0.991	0.956	1.022	1.080	1.030	1.051	1.067	1.069	1.043	1.104	1.005	Q9Y2J8	PADI2	Protein-arginine deiminase type-2

Abundance Ratio														
1 d M1/C1	1 d M2/C2	1 d M3/C3	4 d M1/C1	4 d M2/C2	4 d M3/C3	7 d M1/C1	7 d M2/C2	7 d M3/C3	10 d M1/C1	10 d M2/C2	10 d M3/C3	Accession ID	Gene name	Protein Description
1.167	1.213	1.063	1.349	0.971	0.777	1.302	1.116	0.902	1.267	1.128	1.423	Q8IZE3	SCYL3	Protein-associating with the carboxyl-terminal domain of ezrin
1.026	1.027	1.074	1.008	0.932	1.021	1.002	1.178	0.999	1.006	1.035	1.006	E7EMA9	PGGHG	Protein-glucosylgalactosylhydroxylysine glucosidase
1.004	0.989	0.975	1.000	0.991	1.024	0.980	1.035	1.008	1.007	0.970	1.024	Q9H993	ARMT1	Protein-glutamate O-methyltransferase
1.035	1.019	0.986	0.993	1.111	0.979	0.990	0.950	1.003	0.967	0.983	0.977	P21980	TGM2	Protein-glutamine gamma-glutamyltransferase 2
									0.941	1.089	1.168	Q96MG8	PCMTD1	Protein-L-isoaspartate O-methyltransferase domain-containing protein 1
1.107	1.002	1.072	0.934	0.922	1.200	1.033	1.024	1.003				Q9NV79	PCMTD2	Protein-L-isoaspartate O-methyltransferase domain-containing protein 2
0.992	0.979	0.997	0.976	0.990	0.969	0.998	0.949	0.978	0.984	0.985	0.998	H7BY58	PCMT1	Protein-L-isoaspartate O-methyltransferase
1.118	1.094	0.971	1.095	0.829	0.965	1.176	1.118	1.183	0.944	1.046	1.056	O60725	ICMT	Protein-S-isoprenylcysteine O-methyltransferase
			1.110	0.754	1.070	0.883	1.094	1.047				O60704	TPST2	Protein-tyrosine sulfotransferase 2
0.987	1.385	1.375				0.881	1.148	0.994				Q92954	PRG4	Proteoglycan 4
0.972	0.881	1.051	1.592	1.406	1.103	1.064	0.853	0.858	1.122	1.015	1.045	Q04941	PLP2	Proteolipid protein 2
1.109	1.037	0.946	0.971	0.922	1.056	1.037	0.921	0.954	1.188	1.165	1.167	H7C2N1	PTMA	Prothymosin alpha (Fragment)
1.060	0.991	1.072	0.985	0.929	1.047	1.025	1.130	0.863	0.997	1.062	1.160	A0A087WVP1	FAT1	Protocadherin Fat 1
0.969	0.562	0.980	1.051	0.701	0.943							Q6V0I7	FAT4	Protocadherin Fat 4
									1.146	1.068	1.105	Q9UN70	PCDHGC3	Protocadherin gamma-C3
1.049	0.947	1.077										Q8TAB3	PCDH19	Protocadherin-19
1.121	0.908	0.974	0.931	0.970	1.024	1.025	1.000	0.784	1.131	0.892	0.935	A0A096LNH0	DCHS2	Protocadherin-23 (Fragment)
			0.833	0.703	1.166	0.777	0.912	1.215	1.002	1.215	1.075	Q96QE2	SLC2A13	Proton myo-inositol cotransporter
			1.108	0.875	1.257	0.948	1.107	1.223	1.281	1.238	1.306	Q7Z2H8	SLC36A1	Proton-coupled amino acid transporter 1
0.990	1.059	1.063	1.073	0.900	1.051	1.062	1.074	1.115	1.023	0.957	1.161	Q6YBV0	SLC36A4	Proton-coupled amino acid transporter 4
1.043	0.992	1.016	0.961	0.996	0.989	1.010	1.109	0.991	0.997	1.026	1.026	Q04864	REL	Proto-oncogene c-Rel
1.043	1.057	1.011	1.002	0.988	1.023	0.980	0.971	1.025	1.031	0.981	1.045	P50336	PPOX	Protoporphyrinogen oxidase
0.973	1.023	1.091	0.997	0.916	1.129	1.121	1.153	1.060	0.853	0.942	0.856	F8VU11	PRPF40B	PRP40 pre-mRNA processing factor 40 homolog B (Yeast), isoform CRA_a
1.148	0.674	0.865	0.806	0.917	1.099	0.956	1.002	1.007	0.869	0.933	1.126	B1AHC4	PRR5-ARHGAP8	PRR5-ARHGAP8 readthrough
1.040	1.014	0.998	1.009	0.997	0.943	0.994	1.068	0.959	0.962	0.798	1.029	Q9H792	PEAK1	Pseudopodium-enriched atypical kinase 1
1.016	1.010	0.949	0.995	0.959	0.979	0.997	1.109	1.005	0.982	0.974	0.997	Q96PZ0	PUS7	Pseudouridylate synthase 7 homolog
0.987	1.054	1.023	0.950	0.907	0.999	0.986	1.224	1.067	0.970	0.967	0.977	Q9H0K6	PUS7L	Pseudouridylate synthase 7 homolog-like protein
1.026	0.951	1.051	1.008	1.038	1.013	1.037	0.991	0.946	1.016	0.987	1.009	Q9UBP9	GULP1	PTB domain-containing engulfment adapter protein 1
1.073	1.015	1.070	0.939	0.961	0.989	1.116	1.065	1.026	1.037	0.937	1.039	Q9H0N5	PCBD2	Pterin-4-alpha-carbinolamine dehydratase 2
1.011	0.998	0.971	0.988	0.930	0.973	1.017	1.050	0.985	1.060	0.990	1.000	P61457	PCBD1	Pterin-4-alpha-carbinolamine dehydratase

Abundance Ratio												Accession ID	Gene name	Protein Description
1 d M1/C1	1 d M2/C2	1 d M3/C3	4 d M1/C1	4 d M2/C2	4 d M3/C3	7 d M1/C1	7 d M2/C2	7 d M3/C3	10 d M1/C1	10 d M2/C2	10 d M3/C3			
1.007	1.008	1.000	0.982	0.994	0.989	0.996	0.982	0.991	1.007	1.007	1.011	Q5T1Z8	PUM1	Pumilio homolog 1
			1.063	0.912	1.081	1.002	0.941	1.091	0.904	0.981	1.137	Q14671	PUM1	Pumilio homolog 1
0.983	1.020	1.035	0.997	0.999	1.046	1.022	1.077	1.056	1.001	1.033	1.035	Q15397	PUM3	Pumilio homolog 3
0.952	1.007	1.002	0.997	1.029	0.998	0.991	1.041	0.999	0.989	0.977	0.999	P00491	PNP	Purine nucleoside phosphorylase
0.976	1.010	0.981	0.988	0.986	0.990	0.983	1.023	0.994	0.970	0.961	0.980	P55786	NPEPPS	Puromycin-sensitive aminopeptidase
0.852	0.949	1.412	0.772	0.942	1.339							A6NEC2	NPEPPSL1	Puromycin-sensitive aminopeptidase-like protein
1.038	0.990	1.008	0.981	0.836	0.870	1.110	1.061	0.944	0.893	0.772	1.155	Q96GX2	ATXN7L3B	Putative ataxin-7-like protein 3B
1.052	1.067	1.119	0.982	1.081	0.975	0.966	1.333	1.149				Q92771	DDX12P	Putative ATP-dependent RNA helicase DDX12
0.962	0.928	0.943	1.069	0.937	1.020	1.097	1.088	0.924	1.018	1.045	1.030	Q9H6R0	DHX33	Putative ATP-dependent RNA helicase DHX33
1.021	1.003	1.027	0.950	0.940	1.027	0.971	1.060	1.076	0.992	0.985	1.040	Q6P158	DHX57	Putative ATP-dependent RNA helicase DHX57
									0.884	1.086	1.046	Q9NP73	ALG13	Putative bifunctional UDP-N-acetylglucosamine transferase and deubiquitinase ALG13
1.010	1.001	0.996	1.023	1.027	1.026	1.016	0.995	0.981	0.988	0.994	0.987	Q6P1N9	TATDN1	Putative deoxyribonuclease TATDN1
1.070	1.028	1.066	1.066	0.996	0.919							Q93075	TATDN2	Putative deoxyribonuclease TATDN2
0.953	0.974	1.018	1.028	0.981	1.039	0.978	0.961	1.004	0.952	0.929	1.127	Q8N806	UBR7	Putative E3 ubiquitin-protein ligase UBR7
1.233	1.006	1.043	1.026	0.845	0.807							E9PDK2	UNKL	Putative E3 ubiquitin-protein ligase UNKL
0.979	0.980	0.999	0.970	0.982	0.944	0.857	0.838	0.953	0.917	1.031	0.886	Q5VTE0	EEF1A1P5	Putative elongation factor 1-alpha-like 3
1.051	1.036	1.012	0.983	1.116	1.055	0.947	1.028	1.003	1.034	0.934	1.027	Q8WUX2	CHAC2	Putative glutathione-specific gamma-glutamylcyclotransferase 2
1.076	0.973	1.025	1.103	0.929	1.052	1.106	0.969	1.054	1.166	1.025	1.052	H0Y2S1	GTPBP6	Putative GTP-binding protein 6
0.824	0.986	0.876	0.966	0.881	1.071	0.975	1.124	0.906	1.085	1.016	1.010	Q58FG1	HSP90AA4P	Putative heat shock protein HSP 90-alpha A4
			0.974	0.880	1.220				1.135	1.046	1.266	Q58FF8	HSP90AB2P	Putative heat shock protein HSP 90-beta 2
1.014	0.987	0.923				1.077	0.989	1.010	0.997	0.645	1.082	Q58FF7	HSP90AB3P	Putative heat shock protein HSP 90-beta-3
0.995	1.029	0.995	1.005	0.958	0.948	0.990	1.089	1.007	0.974	0.993	1.024	Q9HCE1	MOV10	Putative helicase MOV-10
1.086	1.008	1.224	0.993	1.032	0.958	1.055	1.010	1.011	1.013	0.931	1.142	Q2TB90	HKDC1	Putative hexokinase HKDC1
1.105	1.048	0.985	1.078	1.105	0.913	1.024	1.022	0.974	1.026	1.038	0.976	O75884	RBBP9	Putative hydrolase RBBP9
0.963	1.035	1.051	0.972	0.952	1.026	1.033	1.122	1.059	0.995	0.973	1.112	Q5T013	HYI	Putative hydroxypyruvate isomerase
1.024	1.020	1.015	1.004	0.978	1.046	1.022	1.068	1.080	0.987	1.028	1.009	Q5T280	SPOUT1	Putative methyltransferase C9orf114
1.013	1.033	1.064										Q8TEA1	NSUN6	Putative methyltransferase NSUN6
1.089	1.087	0.986	1.029	0.953	1.021	1.013	0.983	1.026	1.018	1.017	0.997	Q96A73	KIAA1191	Putative monooxygenase p33MONOX
			0.968	0.858	0.903	1.023	1.230	0.849	1.108	1.033	1.078	Q9BZK3	NACAP1	Putative nascent polypeptide-associated complex subunit alpha-like protein
0.968	1.001	0.955	1.013	0.880	1.055	1.119	1.119	1.010	1.201	1.061	1.196	O60361	NME2P1	Putative nucleoside diphosphate kinase
1.004	0.970	0.988	0.981	1.016	0.999	1.014	0.963	0.989	1.056	1.000	1.001	Q6GMV3	PTRHD1	Putative peptidyl-tRNA hydrolase PTRHD1
1.012	1.007	0.985	1.032	1.037	1.041	1.073	1.020	1.057	1.138	1.085	1.109	Q8NHP8	PLBD2	Putative phospholipase B-like 2

Abundance Ratio														
1 d M1/C1	1 d M2/C2	1 d M3/C3	4 d M1/C1	4 d M2/C2	4 d M3/C3	7 d M1/C1	7 d M2/C2	7 d M3/C3	10 d M1/C1	10 d M2/C2	10 d M3/C3	Accession ID	Gene name	Protein Description
1.001	1.036	0.978	0.925	1.028	1.053	0.996	1.061	1.065	0.949	1.140	1.048	Q8IXJ9	ASXL1	Putative Polycomb group protein ASXL1
1.015	1.007	1.029	0.999	0.976	1.021	0.970	1.021	1.003	0.967	1.014	1.027	O60231	DHX16	Putative pre-mRNA-splicing factor ATP-dependent RNA helicase DHX16
1.070	0.803	1.042	0.927	1.087	1.032	1.043	1.015	1.061	1.000	0.980	1.045	Q7L7V1	DHX32	Putative pre-mRNA-splicing factor ATP-dependent RNA helicase DHX32
1.088	0.986	1.073	1.033	0.994	1.038	1.045	1.094	1.039	0.951	0.996	0.926	Q6P2P2	PRMT9	Putative protein arginine N-methyltransferase 9
						1.038	1.012	1.251				Q8IZP2	ST13P4	Putative protein FAM10A4
1.051	0.982	1.064	0.953	0.911	1.116				1.017	0.889	1.174	B3EWG4	FAM25BP	Putative protein FAM25BP
1.007	0.992	1.018	1.089	1.079	1.195	1.003	0.994	1.060	1.087	1.133	1.135	P58557	YBEY	Putative ribonuclease
0.946	0.894	1.069	0.986	1.067	1.013	0.983	1.034	1.023	1.020	0.935	1.023	Q8N0V3	RBFA	Putative ribosome-binding factor A, mitochondrial
0.956	0.989	1.024	0.969	0.949	1.055	0.974	0.963	1.016	0.977	0.965	1.063	Q8IXW5	RPAP2	Putative RNA polymerase II subunit B1 CTD phosphatase RPAP2
1.032	1.008	0.987	1.010	0.952	0.998	1.014	1.002	0.966	1.048	1.021	1.023	Q96T37	RBM15	Putative RNA-binding protein 15
1.073	1.006	1.071	0.998	0.943	1.018	0.995	1.033	1.047	1.000	1.005	1.051	Q8NDT2	RBM15B	Putative RNA-binding protein 15B
1.048	0.935	1.058	1.017	0.906	1.087	1.192	0.955	1.114	1.006	0.969	1.126	Q9NQ29	LUC7L	Putative RNA-binding protein Luc7-like 1
0.986	1.009	0.980	0.997	1.021	1.019	1.020	0.994	0.989	1.012	0.996	1.027	Q9Y383	LUC7L2	Putative RNA-binding protein Luc7-like 2
0.978	0.968	1.019	0.988	0.958	1.093	0.965	0.984	1.065	0.997	0.994	0.981	A8MWD9	SNRPGP15	Putative small nuclear ribonucleoprotein G-like protein 15
			0.997	0.834	0.951	0.841	0.927	0.986				Q9HBR0	SLC38A10	Putative sodium-coupled neutral amino acid transporter 10
			1.006	0.601	1.106	1.067	1.048	1.041	0.979	1.110	1.006	Q9NVC3	SLC38A7	Putative sodium-coupled neutral amino acid transporter 7
1.046	1.056	0.987	1.014	1.018	0.996	1.030	1.013	1.022	1.003	0.984	1.022	Q5T440	IBA57	Putative transferase CAF17, mitochondrial
1.050	0.970	0.993	1.024	0.979	0.994	1.031	1.006	0.944	1.015	0.903	0.960	Q9UET6	FTSJ1	Putative tRNA (cytidine(32)/guanosine(34)-2'-O)-methyltransferase
1.050	0.928	1.133	0.979	0.997	0.975	0.911	1.044	1.008	1.014	1.033	1.065	Q3MIT2	PUS10	Putative tRNA pseudouridine synthase Pus10
			0.844	0.759	1.149							Q5JXB2	UBE2NL	Putative ubiquitin-conjugating enzyme E2 N-like
			1.001	0.976	1.168							Q7Z3I0	DKFZp313E1411	Putative uncharacterized protein DKFZp313E1411
									0.866	1.012	0.898	A1L4Q6	5 SV	Putative uncharacterized protein FLJ41423
0.970	0.927	1.011	0.937	0.902	1.024	1.024	1.139	1.078	1.050	1.051	1.077	Q53S08	RAB6C	Putative uncharacterized protein RAB6C
1.194	0.924	1.063	1.110	0.885	0.990	1.189	1.080	1.060	1.039	1.045	0.887	Q96N64	PWWP2A	PWWP domain-containing protein 2A
0.945	0.903	1.033				1.136	1.046	1.217				J3KNX4	MUM1	PWWP domain-containing protein MUM1
			1.011	0.975	1.121	1.052	1.063	1.040	0.969	0.940	1.036	Q5TGL8	PXDC1	PX domain-containing protein 1
0.983	1.005	0.972	1.045	0.944	1.030	1.045	1.137	1.006	0.963	0.999	1.075	Q7Z7A4	PXK	PX domain-containing protein kinase-like protein
			0.936	1.180	1.220	0.678	0.722	0.657				Q9Y3Y4	PYGO1	Pygopus homolog 1

Abundance Ratio														
1 d M1/C1	1 d M2/C2	1 d M3/C3	4 d M1/C1	4 d M2/C2	4 d M3/C3	7 d M1/C1	7 d M2/C2	7 d M3/C3	10 d M1/C1	10 d M2/C2	10 d M3/C3	Accession ID	Gene name	Protein Description
1.155	1.025	1.095	0.929	0.893	1.118	0.937	1.095	1.122	0.963	1.013	0.892	Q9BRQ0	PYGO2	Pygopus homolog 2
						0.968	0.859	0.815				Q8WU10	PYROXD1	Pyridine nucleotide-disulfide oxidoreductase domain-containing protein 1
1.015	0.998	1.020	1.010	1.057	0.988	0.975	1.018	1.031	1.003	1.002	1.005	O00764	PDXK	Pyridoxal kinase
0.988	1.031	0.976	0.997	0.971	1.030	1.013	1.054	1.036	0.970	1.012	0.992	Q96GD0	PDXP	Pyridoxal phosphate phosphatase
0.992	0.980	1.015	0.977	0.946	0.979	1.003	1.027	1.018	0.968	0.935	0.998	Q6P996	PDXDC1	Pyridoxal-dependent decarboxylase domain-containing protein 1
1.051	1.019	1.096	1.049	1.121	1.021	1.020	1.058	1.058	1.044	1.017	1.028	Q9NVS9	PNPO	Pyridoxine-5'-phosphate oxidase
1.011	0.991	0.937	0.944	0.865	0.967	0.903	1.069	1.028	0.938	0.964	0.957	Q9NXJ5	PGPEP1	Pyroglutamyl-peptidase 1
0.959	1.048	1.013	1.002	0.968	0.999	0.968	1.057	0.982	0.985	0.998	0.987	Q96C36	PYCR2	Pyrroline-5-carboxylate reductase 2
1.001	1.002	1.019	0.989	0.964	0.974	0.989	1.058	0.967	1.026	0.991	1.037	A0A0A0MQS1	PYCRL	Pyrroline-5-carboxylate reductase
0.983	1.004	1.039	1.001	0.970	1.001	0.974	0.963	0.992	1.028	0.977	1.014	P11498	PC	Pyruvate carboxylase, mitochondrial
0.989	1.007	0.982	1.001	1.000	0.992	1.017	1.036	0.977	1.018	1.000	0.986	P11177	PDHB	Pyruvate dehydrogenase E1 component subunit beta, mitochondrial
1.003	1.004	0.995	0.992	1.005	0.991	0.998	0.982	0.989	1.040	0.986	1.015	Q8NCN5	PDPR	Pyruvate dehydrogenase phosphatase regulatory subunit, mitochondrial
0.976	0.989	1.025	0.990	0.952	1.020	1.000	1.013	0.995	1.016	0.983	1.062	O00330	PDHX	Pyruvate dehydrogenase protein X component, mitochondrial
1.022	0.998	1.008	1.001	1.074	0.978	0.975	0.963	1.014	0.931	0.974	0.944	P14618	PKM	Pyruvate kinase PKM
1.037	1.016	1.040	1.001	0.990	0.995	0.993	1.073	0.993	1.005	1.006	1.030	Q9BXR0	QTRT1	Queuine tRNA-ribosyltransferase catalytic subunit 1
1.031	1.005	0.985	0.999	0.991	0.968	0.993	1.012	0.983	1.035	0.979	0.973	Q08257	CRYZ	Quinone oxidoreductase
1.015	1.011	0.990	1.028	0.995	0.984	1.004	1.004	0.988	1.027	1.005	0.949	Q53FA7	TP53I3	Quinone oxidoreductase PIG3
1.002	1.010	1.011	1.015	0.972	0.973	0.964	1.028	1.010	0.964	0.988	0.959	O95825	CRYZL1	Quinone oxidoreductase-like protein 1
1.036	1.009	1.137	1.036	1.022	0.934	0.935	1.101	0.948	0.935	1.091	0.855	A0A0R4J2E2	R3HCC1	R3H and coiled-coil domain-containing protein 1
			1.025	0.657	0.917	0.993	1.130	1.278				B5MCG9	R3HDM2	R3H domain-containing protein 2
1.036	1.044	1.062							0.870	0.859	1.056	Q96D70	R3HDM4	R3H domain-containing protein 4
			0.852	0.873	1.023	0.914	1.062	0.951				Q5SX86	GDI2	Rab GDP dissociation inhibitor (Fragment)
1.025	1.000	1.005	1.009	1.068	0.981	0.986	0.994	1.023	0.980	0.999	0.993	P31150	GDI1	Rab GDP dissociation inhibitor alpha
1.020	1.011	0.985	1.008	1.082	0.998	1.009	1.045	1.026	0.994	1.017	0.996	P50395	GDI2	Rab GDP dissociation inhibitor beta
1.022	0.953	1.045	1.145	0.930	1.003	1.053	0.953	1.001	1.161	0.909	1.029	Q9Y3P9	RABGAP1	Rab GTPase-activating protein 1
1.034	0.990	1.016	0.979	0.975	1.009	1.022	1.035	1.020	1.017	1.035	1.015	Q5R372	RABGAP1L	Rab GTPase-activating protein 1-like
1.024	0.983	1.021	1.017	0.982	1.054	1.005	0.971	1.027	1.068	0.990	1.058	Q15276	RABEP1	Rab GTPase-binding effector protein 1
1.013	0.997	1.027	1.029	1.004	1.074	1.055	1.030	0.978	1.044	1.011	1.082	Q9H5N1	RABEP2	Rab GTPase-binding effector protein 2
1.023	0.988	1.006	0.989	0.908	1.000	0.972	1.076	1.033	1.021	0.997	1.031	P24386	CHM	Rab proteins geranylgeranyltransferase component A 1

Abundance Ratio												Accession ID	Gene name	Protein Description
1 d M1/C1	1 d M2/C2	1 d M3/C3	4 d M1/C1	4 d M2/C2	4 d M3/C3	7 d M1/C1	7 d M2/C2	7 d M3/C3	10 d M1/C1	10 d M2/C2	10 d M3/C3			
0.983	0.952	0.944	1.020	0.894	1.008	0.930	1.148	1.006	0.935	0.947	0.961	P26374	CHML	Rab proteins geranylgeranyltransferase component A 2
1.024	1.053	1.025	0.988	0.977	1.048	1.016	1.070	1.031	0.974	1.007	1.042	Q6WKZ4	RAB11FIP1	Rab11 family-interacting protein 1
1.039	0.989	1.038	0.990	0.997	1.025	1.009	1.001	1.033	1.058	1.018	1.109	A0A1B0GTL5	RAB11FIP5	Rab11 family-interacting protein 5
0.994	1.016	1.013	1.007	0.976	1.026	1.004	1.010	1.005	1.003	0.995	1.032	Q15042	RAB3GAP1	Rab3 GTPase-activating protein catalytic subunit
1.025	0.977	1.022	1.014	0.967	1.023	1.019	1.052	1.016	1.021	1.004	1.025	Q9H2M9	RAB3GAP2	Rab3 GTPase-activating protein non-catalytic subunit
1.143	1.067	1.046	1.012	1.002	1.089	1.038	1.020	0.974	1.040	1.006	1.108	Q96QF0	RAB3IP	Rab-3A-interacting protein
1.033	0.996	1.007	1.019	1.026	1.068	1.053	1.012	1.011	1.042	0.980	1.017	Q9UJ41	RABGEF1	Rab5 GDP/GTP exchange factor
0.951	0.913	1.540	1.075	1.003	1.437							Q4ADV7	RIC1	RAB6A-GEF complex partner protein 1
									0.998	0.918	0.792	Q92546	RGP1	RAB6A-GEF complex partner protein 2
0.925	0.933	1.208	0.953	0.948	1.078	1.006	0.923	0.925				Q5T7V8	GORAB	RAB6-interacting golgin
1.156	1.016	1.062	1.038	1.051	1.008	1.023	0.973	0.991	1.013	1.023	1.108	Q7Z6M1	RABEPK	Rab9 effector protein with kelch motifs
1.141	0.988	1.202	1.012	0.957	1.050	1.042	1.039	1.071	0.909	1.024	1.019	Q9H1K0	RBSN	Rabenosyn-5
						1.072	1.238	0.925	1.034	1.011	0.984	Q96NA2	RILP	Rab-interacting lysosomal protein
1.066	1.040	1.029	1.002	0.940	0.952	1.010	1.066	0.981	1.024	0.989	1.002	Q5HYI8	RABL3	Rab-like protein 3
1.047	0.963	0.991	0.995	0.964	0.980	1.021	0.949	0.982	1.033	0.932	1.000	Q3YEC7	RABL6	Rab-like protein 6
0.981	1.049	1.062	1.025	1.083	1.058	1.104	1.012	1.009	1.056	1.039	1.147	Q9H0H5	RACGAP1	Rac GTPase-activating protein 1
1.015	1.023	1.027	0.972	0.982	1.061	1.061	1.088	1.036	1.019	1.064	1.043	P31749	AKT1	RAC-alpha serine/threonine-protein kinase
0.964	0.990	0.960	1.014	0.975	1.052	1.012	1.066	1.050	0.982	1.007	1.039	P31751	AKT2	RAC-beta serine/threonine-protein kinase
1.078	1.058	0.988	1.018	0.956	1.035	0.983	1.115	1.038	0.987	1.019	1.089	Q9Y243	AKT3	RAC-gamma serine/threonine-protein kinase
1.010	1.018	1.044	0.992	0.943	1.027	1.062	1.064	1.078	1.008	1.031	1.007	Q6NUQ1	RINT1	RAD50-interacting protein 1
0.933	1.044	1.116	1.063	0.926	1.045	2.402	1.261	1.541	1.580	1.055	1.181	Q9HA92	RSAD1	Radical S-adenosyl methionine domain-containing protein 1, mitochondrial
0.917	1.016	1.041	0.982	0.988	1.052	1.050	0.905	1.042	1.097	1.041	1.142	Q14699	RFTN1	Raftlin
1.001	1.037	1.007	1.050	0.914	1.005	1.003	1.025	1.031	1.024	0.967	0.970	Q6IAA8	LAMTOR1	Ragulator complex protein LAMTOR1
1.006	1.018	0.994	1.015	1.031	1.016	0.999	1.053	1.035	1.005	0.994	1.023	Q9Y2Q5	LAMTOR2	Ragulator complex protein LAMTOR2
1.051	0.955	1.014	0.978	0.960	0.943	0.905	1.101	1.003	0.919	1.012	0.971	Q9UHA4	LAMTOR3	Ragulator complex protein LAMTOR3
0.891	1.004	0.914	0.996	0.990	1.033	0.997	1.065	0.913	0.989	1.026	1.028	C9JXA7	LAMTOR4	Ragulator complex protein LAMTOR4
1.015	0.969	0.995	0.999	1.010	1.004	0.944	1.019	0.942	0.923	1.016	0.899	O43504	LAMTOR5	Ragulator complex protein LAMTOR5
0.994	0.979	1.015	0.974	1.007	1.061	0.981	1.042	1.029	0.991	0.983	1.049	A0A1B0GUI1	RALGAPA1	Ral GTPase-activating protein subunit alpha-1
1.106	1.011	1.078	0.983	0.969	1.012	0.985	1.023	1.030	0.912	0.998	0.966	Q2PPJ7	RALGAPA2	Ral GTPase-activating protein subunit alpha-2
1.029	1.019	1.016	0.993	0.987	1.015	1.018	1.058	0.999	0.993	1.007	1.003	Q86X10	RALGAPB	Ral GTPase-activating protein subunit beta
0.923	1.046	0.953	0.981	0.950	0.987	0.980	1.025	1.029	1.054	0.985	1.053	O15211	RGL2	Ral guanine nucleotide dissociation stimulator-like 2

Abundance Ratio														
1 d M1/C1	1 d M2/C2	1 d M3/C3	4 d M1/C1	4 d M2/C2	4 d M3/C3	7 d M1/C1	7 d M2/C2	7 d M3/C3	10 d M1/C1	10 d M2/C2	10 d M3/C3	Accession ID	Gene name	Protein Description
1.029	0.918	1.076	1.009	0.958	1.015	1.090	1.107	1.056	1.057	1.051	1.078	Q15311	RALBP1	RalA-binding protein 1
1.017	1.016	1.025	1.031	0.944	0.980	0.973	1.051	0.986	1.007	1.044	0.996	Q96D71	REPS1	RalBP1-associated Eps domain-containing protein 1
0.966	1.013	1.017	0.988	0.997	0.991	1.037	1.049	1.008	0.990	0.968	1.010	P46060	RANGAP1	Ran GTPase-activating protein 1
			0.928	0.805	0.970	0.950	0.974	1.027	1.096	0.931	1.101	Q9HD47	RANGRF	Ran guanine nucleotide release factor
1.077	1.080	1.058	1.017	0.919	0.986	0.963	1.150	0.947	1.063	0.985	1.020	R4GMX8	RANBP10	Ran-binding protein 10
						0.999	1.333	1.101	1.329	1.003	0.934	Q9H2T7	RANBP17	Ran-binding protein 17
									1.081	1.029	0.938	Q9H6Z4	RANBP3	Ran-binding protein 3
1.061	0.970	1.043	1.013	0.946	0.989	0.993	1.068	1.120	0.902	1.009	1.039	O60518	RANBP6	Ran-binding protein 6
1.111	1.053	1.038	0.987	0.892	1.014	0.966	1.149	1.017	0.873	1.031	1.039	Q96S59	RANBP9	Ran-binding protein 9
									1.284	1.126	1.083	F8VYC4	RGPD1	RANBP2-like and GRIP domain-containing protein 1
1.059	0.956	0.976	1.098	0.937	1.035	1.115	1.094	1.074	0.860	1.021	0.927	O14715	RGPD8	RANBP2-like and GRIP domain-containing protein 8
0.923	0.974	1.003	0.946	0.910	1.000	0.926	0.850	0.983	0.995	0.974	1.030	Q9BYM8	RBCK1	RanBP-type and C3HC4-type zinc finger-containing protein 1
0.991	0.957	0.991	0.971	1.072	0.954	0.979	0.991	1.003	0.919	0.975	0.921	F6WQW2	RANBP1	Ran-specific GTPase-activating protein
0.932	1.067	1.066	1.107	0.954	0.862	1.024	1.047	1.064	0.996	0.954	0.957	Q9Y4G8	RAPGEF2	Rap guanine nucleotide exchange factor 2
0.714	0.770	1.554										O95398	RAPGEF3	Rap guanine nucleotide exchange factor 3
			0.914	0.724	1.117				0.948	1.035	1.203	A8MQ07	RAPGEF5	Rap guanine nucleotide exchange factor 5
0.899	0.908	1.008				1.047	1.150	0.923	0.986	1.091	1.341	Q8IZ41	RASEF	Ras and EF-hand domain-containing protein
1.097	1.095	1.051	1.011	0.987	0.978	1.021	1.014	1.002	0.991	0.992	1.034	Q13671	RIN1	Ras and Rab interactor 1
			0.977	0.757	0.708							Q8TB24	RIN3	Ras and Rab interactor 3
1.046	1.012	0.975	1.008	0.988	0.940	1.016	1.014	1.013	1.036	0.990	1.201	P50749	RASSF2	Ras association domain-containing protein 2
0.968	0.965	1.081	1.007	0.939	1.108	1.030	1.048	0.959	1.063	1.148	1.107	Q86WH2	RASSF3	Ras association domain-containing protein 3
1.048	0.909	1.169	1.022	0.838	1.024				0.983	1.014	1.068	A0A0G2JMW4	RASSF7	Ras association domain-containing protein 7
			0.884	0.827	1.067	0.933	0.977	0.938				Q8NHQ8	RASSF8	Ras association domain-containing protein 8
1.003	1.000	1.017	0.985	0.974	1.035	0.992	1.065	1.035	0.986	1.041	1.021	P20936	RASA1	Ras GTPase-activating protein 1
1.142	0.915	1.032	1.032	0.953	1.038	1.036	1.039	1.033	0.956	0.979	1.041	A0A0A0MSJ9	RASA2	Ras GTPase-activating protein 2
1.028	0.973	1.021	1.039	0.987	1.060	1.037	1.096	1.013	0.971	1.004	1.039	Q14644	RASA3	Ras GTPase-activating protein 3
1.014	1.030	1.014	1.001	1.022	0.969	1.012	1.103	1.032	0.984	1.040	1.057	Q13283	G3BP1	Ras GTPase-activating protein-binding protein 1
0.990	0.998	0.994	0.976	0.987	0.989	1.005	1.010	0.994	1.039	1.022	1.076	Q9UN86	G3BP2	Ras GTPase-activating protein-binding protein 2
0.960	1.003	0.974	0.997	0.996	0.990	0.977	1.044	0.999	0.986	0.994	0.976	P46940	IQGAP1	Ras GTPase-activating-like protein IQGAP1
1.004	1.026	1.009	1.028	0.982	1.000	1.062	1.129	1.060	0.966	0.921	1.108	Q86VI3	IQGAP3	Ras GTPase-activating-like protein IQGAP3
0.989	0.988	0.995	0.975	0.988	0.997	1.029	1.006	1.012	0.982	0.953	1.030	Q15404	RSU1	Ras suppressor protein 1

Abundance Ratio														
1 d M1/C1	1 d M2/C2	1 d M3/C3	4 d M1/C1	4 d M2/C2	4 d M3/C3	7 d M1/C1	7 d M2/C2	7 d M3/C3	10 d M1/C1	10 d M2/C2	10 d M3/C3	Accession ID	Gene name	Protein Description
1.045	1.034	1.058	1.034	0.955	0.970	1.020	1.005	0.974	0.980	0.942	1.045	Q70E73	RAPH1	Ras-associated and pleckstrin homology domains-containing protein 1
1.058	1.048	0.974	1.032	1.077	0.933	1.055	0.975	1.051	0.989	1.023	0.928	P63000	RAC1	Ras-related C3 botulinum toxin substrate 1
1.198	1.089	1.140	0.959	0.903	0.997	0.961	1.302	0.930	0.892	1.008	0.897	P60763	RAC3	Ras-related C3 botulinum toxin substrate 3
1.045	0.983	0.995	0.990	0.978	0.887	0.895	0.887	1.016	0.906	1.090	0.906	Q7L523	RRAGA	Ras-related GTP-binding protein A
1.006	1.004	1.012	0.972	0.963	1.013	0.928	0.966	1.030	0.908	0.976	0.934	Q9HB90	RRAGC	Ras-related GTP-binding protein C
									1.040	1.098	0.983	Q9NQL2	RRAGD	Ras-related GTP-binding protein D
0.983	0.962	1.011	0.986	0.912	1.034	1.006	1.046	1.042	1.040	1.038	1.057	P61026	RAB10	Ras-related protein Rab-10
0.981	1.001	0.980	0.987	0.940	1.008	1.014	1.063	1.011	1.027	1.015	0.987	Q15907	RAB11B	Ras-related protein Rab-11B
0.960	0.951	1.104	1.031	0.895	0.981	1.018	1.069	1.008	0.987	1.053	1.037	Q6IQ22	RAB12	Ras-related protein Rab-12
0.964	0.998	0.982	1.010	0.944	1.055	1.049	1.055	1.040	1.094	1.104	1.068	P51153	RAB13	Ras-related protein Rab-13
0.958	0.998	0.975	1.014	0.941	1.015	1.000	1.027	1.013	1.005	1.010	0.981	P61106	RAB14	Ras-related protein Rab-14
0.933	0.997	0.976	0.980	0.945	1.069	1.009	1.056	1.012	1.029	0.987	1.024	Q9NP72	RAB18	Ras-related protein Rab-18
0.971	0.986	0.973	0.980	0.895	1.000	0.989	1.027	1.000	1.000	1.031	0.954	P62820	RAB1A	Ras-related protein Rab-1A
0.887	1.038	0.977	0.957	0.928	0.984	0.949	0.921	0.943	0.945	1.059	0.956	Q9H0U4	RAB1B	Ras-related protein Rab-1B
									0.940	1.067	1.021	Q9NX57	RAB20	Ras-related protein Rab-20
1.034	1.033	0.960	1.070	1.087	1.058	1.125	0.937	1.097	1.026	1.017	1.014	Q9UL25	RAB21	Ras-related protein Rab-21
0.974	0.986	1.030	0.963	0.908	1.055	0.988	1.056	1.085	0.986	1.019	1.047	Q9UL26	RAB22A	Ras-related protein Rab-22A
0.938	0.964	1.029	1.030	0.945	1.120	0.996	0.996	0.980	1.002	0.965	1.037	Q9ULC3	RAB23	Ras-related protein Rab-23
1.042	1.042	1.032	1.002	0.849	1.001	1.008	1.056	0.980	1.008	1.085	1.004	Q969Q5	RAB24	Ras-related protein Rab-24
1.029	0.988	1.012	1.010	0.941	1.026	1.038	0.965	0.975	1.083	0.987	1.074	P51159	RAB27A	Ras-related protein Rab-27A
0.961	0.899	1.027	1.042	0.858	1.014	1.010	1.035	0.996	1.077	1.112	1.173	O00194	RAB27B	Ras-related protein Rab-27B
0.945	1.219	1.423	0.840	0.906	1.168	1.054	0.884	1.016	0.978	1.090	1.062	P51157	RAB28	Ras-related protein Rab-28
0.979	0.985	1.001	0.965	0.907	0.998	0.984	1.033	1.012	0.973	1.017	0.985	P61019	RAB2A	Ras-related protein Rab-2A
1.020	0.999	0.981	0.929	0.905	0.942	0.981	0.952	0.979	1.004	1.075	1.055	Q8WUD1	RAB2B	Ras-related protein Rab-2B
1.107	0.957	1.050	0.881	0.896	1.141							Q15771	RAB30	Ras-related protein Rab-30
1.005	0.957	0.997	1.023	0.879	1.003	1.004	1.041	1.033	0.982	1.013	0.985	Q13636	RAB31	Ras-related protein Rab-31
0.987	0.987	1.002	0.944	0.892	1.018	0.953	1.053	1.023	1.015	0.996	0.970	Q13637	RAB32	Ras-related protein Rab-32
0.992	0.961	1.123	0.966	0.955	1.006	0.995	0.956	1.011	1.125	1.004	1.092	Q9H082	RAB33B	Ras-related protein Rab-33B
0.979	0.986	0.992	1.009	0.939	1.026	1.028	1.096	1.075	1.038	1.026	1.047	Q9BZG1	RAB34	Ras-related protein Rab-34
1.232	0.757	1.011	0.823	0.937	0.795				0.987	1.028	1.244	A0A1B0GTQ2	RAB34	Ras-related protein Rab-34, isoform NARR
0.955	1.012	0.957	1.028	0.938	0.995	1.058	1.047	0.977	1.122	1.083	1.069	Q15286	RAB35	Ras-related protein Rab-35
0.916	1.103	1.171	1.004	0.854	1.082	1.002	1.266	1.127	1.341	1.058	1.077	Q14964	RAB39A	Ras-related protein Rab-39A
0.922	0.962	1.016	1.013	0.939	1.067	1.016	1.011	1.055	0.942	0.975	1.028	P20337	RAB3B	Ras-related protein Rab-3B

Abundance Ratio														
1 d M1/C1	1 d M2/C2	1 d M3/C3	4 d M1/C1	4 d M2/C2	4 d M3/C3	7 d M1/C1	7 d M2/C2	7 d M3/C3	10 d M1/C1	10 d M2/C2	10 d M3/C3	Accession ID	Gene name	Protein Description
1.020	1.006	0.983	0.989	0.929	0.964	0.993	1.065	1.022	1.026	1.045	0.943	O95716	RAB3D	Ras-related protein Rab-3D
1.127	1.143	1.026	1.024	0.903	0.990	0.991	1.110	0.962	1.002	1.015	0.950	Q86YS6	RAB43	Ras-related protein Rab-43
1.006	0.979	0.971	1.009	0.876	1.005	1.029	1.099	1.009	1.000	1.045	0.974	P20338	RAB4A	Ras-related protein Rab-4A
0.993	0.985	0.970	0.981	0.884	0.988	1.000	0.999	0.960	1.031	1.060	1.071	P20339	RAB5A	Ras-related protein Rab-5A
0.960	1.006	0.979	0.994	0.941	1.028	0.984	1.028	1.010	1.012	1.073	1.060	P61020	RAB5B	Ras-related protein Rab-5B
0.924	0.985	0.973	0.965	0.898	1.003	1.005	1.013	1.013	0.988	1.028	1.010	P20340	RAB6A	Ras-related protein Rab-6A
0.971	0.985	1.026	0.956	0.933	1.051	1.028	1.037	1.029	1.124	1.076	0.977	Q9NRW1	RAB6B	Ras-related protein Rab-6B
1.026	1.012	1.005	1.005	0.971	0.991	1.016	1.026	1.002	1.011	1.051	0.944	P51149	RAB7A	Ras-related protein Rab-7a
0.949	1.006	0.989	0.994	0.910	1.008	1.026	1.072	1.034	1.029	1.000	1.027	O14966	RAB29	Ras-related protein Rab-7L1
0.992	1.010	0.971	1.011	0.879	1.027	1.048	1.135	0.972	1.108	1.097	1.092	P61006	RAB8A	Ras-related protein Rab-8A
1.008	1.005	0.987	0.999	1.082	0.995	1.107	1.135	1.004	0.866	1.133	0.977	Q92930	RAB8B	Ras-related protein Rab-8B
0.998	0.987	1.057	1.026	0.984	1.038	1.085	1.092	1.074	1.069	1.020	1.065	P51151	RAB9A	Ras-related protein Rab-9A
0.949	0.967	0.954	0.979	0.952	1.035	1.008	1.008	0.961	1.013	1.007	0.982	P11233	RALA	Ras-related protein Ral-A
0.881	0.937	0.999	1.052	0.975	1.013	1.027	1.027	1.001	1.015	1.051	1.007	P62834	RAP1A	Ras-related protein Rap-1A
1.037	0.982	0.986	1.031	1.021	1.014	1.015	1.024	0.987	1.035	1.023	0.980	P61224	RAP1B	Ras-related protein Rap-1b
0.907	1.020	1.035	0.987	0.949	1.029	1.011	1.116	1.026	1.018	0.993	1.111	P61225	RAP2B	Ras-related protein Rap-2b
0.980	1.010	0.934	1.006	0.922	1.036	1.045	1.102	1.004	1.033	1.066	0.994	Q9Y3L5	RAP2C	Ras-related protein Rap-2c
0.923	0.993	1.005	0.953	0.893	1.026	0.998	1.077	0.994	1.058	1.002	1.050	P10301	RRAS	Ras-related protein R-Ras
1.036	1.023	0.950	1.011	0.970	1.040	1.123	0.980	1.213	1.097	0.956	1.027	Q86X27	RALGPS2	Ras-specific guanine nucleotide-releasing factor RalGPS2
0.905	0.860	1.297	0.962	0.731	1.082	1.038	0.973	1.026	1.029	0.996	1.107	Q8TDY2	RB1CC1	RB1-inducible coiled-coil protein 1
			1.008	0.894	0.955	1.066	1.248	1.129	1.169	0.930	1.162	Q8NDN9	RCBTB1	RCC1 and BTB domain-containing protein 1
1.031	0.938	0.977	0.968	0.955	1.004	1.024	1.051	1.079				B4DWG0	RCBTB2	RCC1 and BTB domain-containing protein 2
1.031	1.042	0.925	1.012	0.870	1.031	0.900	0.833	0.889				A6NED2	RCCD1	RCC1 domain-containing protein 1
0.933	0.941	1.013	0.975	1.037	1.036	1.040	1.028	1.085	1.024	1.032	0.982	Q96I51	RCC1L	RCC1-like G exchanging factor-like protein
						0.970	1.123	0.781				Q13127	REST	RE1-silencing transcription factor
0.916	0.923	1.014	1.093	0.806	1.070	1.025	1.108	1.031	1.004	1.018	1.045	P60602	ROMO1	Reactive oxygen species modulator 1
1.024	1.018	0.996	0.997	1.004	0.953	1.013	1.019	0.953	1.002	1.032	0.970	Q6NUK4	REEP3	Receptor expression-enhancing protein 3
0.914	1.005	1.072	1.032	0.942	1.042	0.890	0.831	1.009	0.998	1.035	1.133	Q9H6H4	REEP4	Receptor expression-enhancing protein 4
1.008	0.963	1.058	1.016	0.953	0.988	1.041	0.965	0.984	0.995	1.031	1.007	Q00765	REEP5	Receptor expression-enhancing protein 5
0.824	0.962	0.998	0.963	0.896	1.090	0.918	0.915	1.049	1.029	0.974	1.094	Q96HR9	REEP6	Receptor expression-enhancing protein 6
1.060	0.997	1.015	1.045	1.268	0.990	1.040	0.947	1.074	0.999	1.008	0.987	P63244	RACK1	Receptor of activated protein C kinase 1
0.992	0.979	1.013	0.953	0.887	1.068				1.145	1.004	1.130	P04626	ERBB2	Receptor tyrosine-protein kinase erbB-2
0.997	1.348	1.443	1.085	0.874	0.806	0.757	1.329	0.992	1.000	0.894	0.950	P21860	ERBB3	Receptor tyrosine-protein kinase erbB-3

Abundance Ratio														
1 d M1/C1	1 d M2/C2	1 d M3/C3	4 d M1/C1	4 d M2/C2	4 d M3/C3	7 d M1/C1	7 d M2/C2	7 d M3/C3	10 d M1/C1	10 d M2/C2	10 d M3/C3	Accession ID	Gene name	Protein Description
0.988	0.992	0.998	0.998	0.972	0.997	1.031	1.024	0.984	1.011	0.980	1.024	Q13546	RIPK1	Receptor-interacting serine/threonine-protein kinase 1
0.995	0.981	1.063	1.008	0.954	1.002	0.983	1.017	1.014	0.955	0.982	1.028	O43353	RIPK2	Receptor-interacting serine/threonine-protein kinase 2
1.180	1.139	1.039	1.051	1.010	1.022	1.031	1.095	1.032	1.025	0.922	1.008	P57078	RIPK4	Receptor-interacting serine/threonine-protein kinase 4
1.014	0.973	1.009	1.022	0.906	1.041	1.033	1.058	1.030	1.072	1.048	1.019	P18433	PTPRA	Receptor-type tyrosine-protein phosphatase alpha
0.900	1.072	0.918	1.334	1.418	1.453	1.520	1.148	0.881	0.959	1.031	1.090	P23469	PTPRE	Receptor-type tyrosine-protein phosphatase epsilon
0.970	0.998	1.051	1.019	0.945	1.016	1.037	1.104	1.021	1.068	0.974	1.112	A0A087WVC6	PTPRJ	Receptor-type tyrosine-protein phosphatase eta
0.966	0.985	0.992	0.972	0.922	0.954	0.999	1.061	1.009	1.021	0.977	1.033	P10586	PTPRF	Receptor-type tyrosine-protein phosphatase F
0.984	1.059	1.045	0.995	0.898	1.017	0.972	1.029	1.047	1.000	1.081	1.032	P23470	PTPRG	Receptor-type tyrosine-protein phosphatase gamma
1.064	1.003	1.075	0.995	0.982	1.030	1.001	1.094	1.045	1.053	1.011	1.105	E9PGC5	PTPRK	Receptor-type tyrosine-protein phosphatase kappa
1.099	1.041	0.991	1.032	1.015	0.958							Q13332	PTPRS	Receptor-type tyrosine-protein phosphatase S
1.467	1.080	1.254				0.870	1.352	1.148				Q92729	PTPRU	Receptor-type tyrosine-protein phosphatase U
1.045	1.019	1.012	0.975	0.946	0.915	0.975	1.010	0.920	0.997	0.985	1.037	Q06330	RBPJ	Recombining binding protein suppressor of hairless
0.991	0.987	1.018	1.001	0.977	1.019	1.023	1.056	1.001	1.027	0.967	0.975	Q9BRX8	FAM213A	Redox-regulatory protein FAM213A
1.017	1.003	0.953	1.017	1.011	1.102	1.083	1.139	1.084	0.995	0.993	1.013	Q8N5W9	RFLNB	Refilin-B
0.986	0.996	1.009	0.977	0.973	1.068	0.994	1.036	1.041	1.073	0.975	1.095	Q96P16	RPRD1A	Regulation of nuclear pre-mRNA domain-containing protein 1A
1.065	1.000	0.991	1.004	0.973	0.989	0.993	1.076	1.015	0.982	1.006	0.961	Q9NQG5	RPRD1B	Regulation of nuclear pre-mRNA domain-containing protein 1B
1.032	1.013	1.024	1.051	0.912	0.995	1.056	1.014	1.031	1.055	0.967	1.036	Q5VT52	RPRD2	Regulation of nuclear pre-mRNA domain-containing protein 2
									1.040	1.319	0.877	C9JW69	RCC1	Regulator of chromosome condensation (Fragment)
1.121	1.088	1.278										O14924	RGS12	Regulator of G-protein signaling 12
									0.913	0.938	1.058	P49795	RGS19	Regulator of G-protein signaling 19
1.039	1.003	0.983	1.010	1.007	0.994	1.010	1.014	0.972	1.047	0.997	0.981	Q96DB5	RMDN1	Regulator of microtubule dynamics protein 1
1.099	0.969	1.127	0.938	0.858	0.990	1.007	0.996	1.016	0.973	1.005	1.032	Q96LZ7	RMDN2	Regulator of microtubule dynamics protein 2
0.984	0.991	1.045	1.013	0.941	1.031	1.013	1.047	0.999	1.058	0.989	1.011	Q96TC7	RMDN3	Regulator of microtubule dynamics protein 3
1.014	1.021	0.998	1.023	1.017	0.980	1.014	1.066	1.034	0.973	1.009	1.001	Q92900	UPF1	Regulator of nonsense transcripts 1
1.033	1.001	1.026	1.013	0.955	1.031	0.995	1.047	1.037	1.020	0.966	1.050	Q9HAU5	UPF2	Regulator of nonsense transcripts 2
			0.647	0.875	0.953	1.022	0.607	0.734	1.226	1.280	1.449	Q9H1J1	UPF3A	Regulator of nonsense transcripts 3A

Abundance Ratio														
1 d M1/C1	1 d M2/C2	1 d M3/C3	4 d M1/C1	4 d M2/C2	4 d M3/C3	7 d M1/C1	7 d M2/C2	7 d M3/C3	10 d M1/C1	10 d M2/C2	10 d M3/C3	Accession ID	Gene name	Protein Description
0.986	1.014	1.027	1.000	0.976	1.033	1.011	1.035	0.999	1.037	0.979	1.035	Q9BZI7	UPF3B	Regulator of nonsense transcripts 3B
0.929	0.922	1.641				0.906	0.755	1.150				F6WH68	RTEL1-TNFRSF6B	Regulator of telomere elongation helicase 1
1.038	1.077	1.029	0.952	0.975	0.930	1.059	1.071	1.033	0.852	1.099	0.996	O00287	RFXAP	Regulatory factor X-associated protein
0.972	0.973	1.025	0.977	0.941	1.041	1.054	1.090	1.022	1.041	0.989	1.067	Q8N122	RPTOR	Regulatory-associated protein of mTOR
1.038	1.007	1.001	1.000	0.958	0.998	1.015	1.003	0.974	1.009	0.982	1.034	Q8WUF5	PPP1R13L	RelA-associated inhibitor
0.814	1.101	0.925							1.677	1.167	1.623	Q8TDU9	RXFP4	Relaxin-3 receptor 2
0.937	1.035	0.955	0.771	0.839	1.046	0.983	0.439	1.033	0.922	0.924	1.146	Q8IUW5	RELL1	RELT-like protein 1
1.007	0.966	1.013	0.983	0.965	1.032	1.010	0.966	1.017	1.010	1.029	1.066	Q96T23	RSF1	Remodeling and spacing factor 1
			0.953	0.973	0.815	0.847	1.118	0.694	0.904	0.968	0.950	A0A1B0GTB0	ATP6AP2	Renin receptor (Fragment)
0.960	0.989	0.966	1.023	0.908	0.997	1.010	1.083	1.020	1.094	1.072	1.050	O75787	ATP6AP2	Renin receptor
0.972	0.985	1.010	1.048	0.948	1.035	1.025	1.038	1.008	1.107	1.033	1.099	P35251	RFC1	Replication factor C subunit 1
0.989	0.990	0.992	0.998	0.967	0.982	1.014	1.089	1.018	0.958	0.973	0.999	P35250	RFC2	Replication factor C subunit 2
1.035	1.000	1.015	0.963	0.949	0.991	1.049	0.971	1.036	0.997	0.972	1.020	P40938	RFC3	Replication factor C subunit 3
1.014	1.006	1.007	0.980	0.987	0.982	0.997	1.064	0.995	0.969	1.004	0.981	P35249	RFC4	Replication factor C subunit 4
1.018	1.006	1.009	0.984	0.982	0.967	0.991	0.978	1.041	0.966	0.968	0.981	P40937	RFC5	Replication factor C subunit 5
1.040	1.017	1.035	0.996	1.065	0.986	1.007	1.037	1.025	0.910	1.007	0.940	P35244	RPA3	Replication protein A 14 kDa subunit
1.006	0.992	0.977	1.004	1.065	0.974	0.982	0.958	1.004	0.970	0.999	0.961	P27694	RPA1	Replication protein A 70 kDa DNA-binding subunit
0.999	0.993	1.062	1.370	1.491	0.960	1.032	1.012	1.045	1.140	0.881	1.127	E9PSH4	MAF1	Repressor of RNA polymerase III transcription MAF1 homolog
1.059	1.008	1.002	1.003	0.941	1.036	1.040	0.947	0.984	1.047	0.973	0.990	Q9NUL5	RYDEN	Repressor of yield of DENV protein
0.987	0.974	0.992	1.019	0.886	1.036	1.008	1.046	1.049	1.008	1.058	1.004	Q9NWS8	RMND1	Required for meiotic nuclear division protein 1 homolog
1.002	0.974	0.990	1.011	0.944	0.984	0.990	1.082	1.010	0.991	0.948	0.973	Q9UKL0	RCOR1	REST corepressor 1
1.017	1.008	0.984	0.983	0.958	0.995	0.984	1.018	0.967	0.977	0.970	0.967	Q15293	RCN1	Reticulocalbin-1
1.104	0.922	0.988	0.892	0.889	1.149	1.007	0.920	0.916	0.929	0.953	1.051	Q96D15	RCN3	Reticulocalbin-3
0.938	1.003	0.982	0.990	0.939	0.981	0.998	1.074	1.021	1.074	1.017	1.045	O95197	RTN3	Reticulon-3
1.018	1.009	0.987	0.993	0.969	1.005	1.008	1.004	0.978	1.013	0.973	1.012	Q8WWV3	RTN4IP1	Reticulon-4-interacting protein 1, mitochondrial
			1.024	0.820	1.105							P00352	ALDH1A1	Retinal dehydrogenase 1
1.036	0.971	1.033	0.858	0.967	1.102	1.017	0.878	1.004	0.826	1.039	1.053	O94788	ALDH1A2	Retinal dehydrogenase 2
1.081	0.922	1.059	0.973	1.095	0.980	1.012	0.988	1.079	1.001	1.002	1.044	O43924	PDE6D	Retinal rod rhodopsin-sensitive cGMP 3',5'-cyclic phosphodiesterase subunit delta
			1.013	0.856	0.892	1.061	1.139	0.640	1.032	0.932	0.781	Q8TA86	RP9	Retinitis pigmentosa 9 protein
1.011	1.016	1.009	0.976	0.900	0.989	1.029	1.095	1.032	0.954	0.977	1.073	P06400	RB1	Retinoblastoma-associated protein
0.989	0.993	1.000	1.002	0.952	0.992	1.026	1.008	1.000	1.043	0.968	1.060	Q15291	RBBP5	Retinoblastoma-binding protein 5

Abundance Ratio														
1 d M1/C1	1 d M2/C2	1 d M3/C3	4 d M1/C1	4 d M2/C2	4 d M3/C3	7 d M1/C1	7 d M2/C2	7 d M3/C3	10 d M1/C1	10 d M2/C2	10 d M3/C3	Accession ID	Gene name	Protein Description
0.946	1.066	0.976	0.834	0.917	0.915	0.724	1.303	0.905				P28749	RBL1	Retinoblastoma-like protein 1
1.191	1.106	1.074	0.981	0.941	1.061	1.023	1.164	0.987	1.126	0.970	1.040	Q08999	RBL2	Retinoblastoma-like protein 2
						1.071	1.075	0.903				A8MUP8	RARA	Retinoic acid receptor alpha
0.993	0.932	0.996	0.885	0.813	0.907	1.155	1.229	0.947	1.083	1.077	1.141	P19793	RXRA	Retinoic acid receptor RXR-alpha
0.952	1.007	0.980	0.999	1.009	0.993	0.997	0.969	0.979	0.992	1.003	1.057	Q7Z5J4	RAI1	Retinoic acid-induced protein 1
1.014	1.006	0.989	1.042	1.023	1.033	1.071	1.154	1.076	1.147	1.142	1.072	Q9HB40	SCPEP1	Retinoid-inducible serine carboxypeptidase
									0.956	0.943	1.017	Q8IZV5	RDH10	Retinol dehydrogenase 10
			0.962	0.800	1.200							G3V2G6	RDH11	Retinol dehydrogenase 11 (Fragment)
0.942	0.975	0.956	0.944	0.857	0.963	0.996	1.054	0.955	1.013	1.041	1.043	Q8TC12	RDH11	Retinol dehydrogenase 11
1.027	1.044	1.033	0.995	0.989	1.003	0.964	1.038	0.997	1.052	0.991	1.038	Q8NBN7	RDH13	Retinol dehydrogenase 13
0.982	1.002	1.018	1.001	0.948	1.014	1.033	1.063	0.984	1.008	0.980	1.056	Q9HBH5	RDH14	Retinol dehydrogenase 14
1.022	1.007	1.244	0.997	0.893	1.018	1.308	0.973	1.987				A0A087WX23	PEG10	Retrotransposon-derived protein PEG10
1.025	1.008	0.993	1.015	0.963	0.978	0.973	1.017	0.963	0.982	1.045	0.930	P52565	ARHGDIA	Rho GDP-dissociation inhibitor 1
						1.021	0.815	0.828				P52566	ARHGDIB	Rho GDP-dissociation inhibitor 2
1.001	1.021	1.023	1.005	0.981	1.006	1.025	1.045	1.037	1.014	0.981	1.039	Q07960	ARHGAP1	Rho GTPase-activating protein 1
0.944	0.922	1.020	1.048	0.993	1.004	1.005	0.966	1.015	0.925	0.875	0.993	A1A4S6	ARHGAP10	Rho GTPase-activating protein 10
1.067	1.123	1.168										Q6P4F7	ARHGAP11A	Rho GTPase-activating protein 11A
1.039	0.981	1.031	1.002	0.986	1.017	0.997	0.993	1.018	1.006	0.994	1.056	Q68EM7	ARHGAP17	Rho GTPase-activating protein 17
0.974	0.970	0.988	0.986	0.988	0.958	0.987	0.950	0.997	1.009	0.995	1.055	Q8N392	ARHGAP18	Rho GTPase-activating protein 18
1.072	0.981	1.021	0.996	0.948	1.012	1.031	1.119	0.970	1.021	0.991	1.094	Q5T5U3	ARHGAP21	Rho GTPase-activating protein 21
0.937	0.965	1.069	0.992	0.942	0.984	0.966	0.855	0.997	0.859	1.136	1.199	A0A0G2JNY3	ARHGAP27	Rho GTPase-activating protein 27
0.971	0.982	0.996	1.004	0.997	0.994	1.055	1.043	1.041	1.008	0.997	1.060	Q52LW3	ARHGAP29	Rho GTPase-activating protein 29
			0.911	0.958	1.011							A7KAX9	ARHGAP32	Rho GTPase-activating protein 32
1.033	1.005	1.005	1.040	0.972	1.040	1.026	1.086	0.990	1.035	0.956	1.038	Q9NRY4	ARHGAP35	Rho GTPase-activating protein 35
0.956	1.014	0.941	1.015	0.946	1.030	1.081	1.205	1.128	0.987	0.904	1.285	Q5TG30	ARHGAP40	Rho GTPase-activating protein 40
1.028	0.961	1.018	1.005	0.992	1.058	1.027	1.043	1.016	1.006	1.007	1.048	Q92619	ARHGAP45	Rho GTPase-activating protein 45
1.020	1.016	1.011	0.987	0.970	1.015	0.963	1.039	1.018	0.995	0.986	1.051	Q13017	ARHGAP5	Rho GTPase-activating protein 5
1.159	1.091	1.114	1.040	0.956	1.013	1.022	1.077	1.088	1.051	1.132	0.997	Q96QB1	DLC1	Rho GTPase-activating protein 7
1.092	1.126	0.962	1.010	0.918	1.038	0.987	1.169	1.032	1.023	0.937	0.993	Q6ZW31	SYDE1	Rho GTPase-activating protein SYDE1
0.990	0.997	1.006	1.009	0.938	1.015	1.032	1.023	1.014	1.006	0.946	1.055	M0QZR4	ARHGEF1	Rho guanine nucleotide exchange factor 1
0.980	0.969	1.011	0.996	0.957	1.049	1.010	1.039	1.070	0.976	0.914	0.961	O15013	ARHGEF10	Rho guanine nucleotide exchange factor 10
1.125	1.096	1.013	0.990	0.962	1.149	1.087	1.017	1.046	0.933	0.847	1.136	Q9HCE6	ARHGEF10L	Rho guanine nucleotide exchange factor 10-like protein
1.007	0.998	1.018	0.996	1.028	0.999	1.018	1.017	1.018	1.068	0.994	1.077	Q9NZN5	ARHGEF12	Rho guanine nucleotide exchange factor 12

Abundance Ratio														
1 d M1/C1	1 d M2/C2	1 d M3/C3	4 d M1/C1	4 d M2/C2	4 d M3/C3	7 d M1/C1	7 d M2/C2	7 d M3/C3	10 d M1/C1	10 d M2/C2	10 d M3/C3	Accession ID	Gene name	Protein Description
0.983	0.976	0.993	1.014	0.962	1.016	0.978	1.043	1.008	0.979	0.936	1.044	Q5VV41	ARHGEF16	Rho guanine nucleotide exchange factor 16
0.903	1.075	1.005	1.015	0.892	1.039	1.072	0.515	1.144	0.933	1.002	0.995	Q96PE2	ARHGEF17	Rho guanine nucleotide exchange factor 17
1.007	1.004	0.995	0.931	0.889	0.981	0.983	1.084	1.080	0.938	0.978	1.049	Q6ZSZ5	ARHGEF18	Rho guanine nucleotide exchange factor 18
1.026	0.975	1.024	1.018	0.972	1.055	1.011	0.995	1.000	0.986	0.954	1.033	V9GYM8	ARHGEF2	Rho guanine nucleotide exchange factor 2
			1.133	0.901	0.975	0.901	1.301	1.069	1.054	0.762	1.223	E7EV07	ARHGEF4	Rho guanine nucleotide exchange factor 4
1.071	0.976	1.030	0.989	1.050	0.981	1.010	1.121	1.051	0.979	0.946	1.061	Q8TER5	ARHGEF40	Rho guanine nucleotide exchange factor 40
1.029	0.994	1.008	0.984	0.960	1.018	0.991	0.953	0.988	1.035	0.979	1.086	Q12774	ARHGEF5	Rho guanine nucleotide exchange factor 5
0.982	0.997	1.031	1.019	0.989	1.048	1.024	0.962	1.022	1.055	0.997	1.069	Q13464	ROCK1	Rho-associated protein kinase 1
1.016	1.009	1.011	0.996	0.966	1.000	1.002	0.944	1.001	1.021	0.983	1.079	O75116	ROCK2	Rho-associated protein kinase 2
1.001	0.864	1.061	1.075	0.936	1.003	0.929	1.065	1.063	1.022	0.883	1.092	Q8TEB9	RHBDD1	Rhomboid-related protein 4
0.991	1.014	1.001	0.966	0.931	0.993	1.024	1.042	0.977	1.003	0.960	1.000	Q8IUC4	RHPN2	Rhophilin-2
			0.920	1.009	1.179							O94955	RHOBTB3	Rho-related BTB domain-containing protein 3
1.053	1.183	1.149	1.036	0.964	1.048	1.043	0.946	1.104	1.065	1.169	1.050	P62745	RHOB	Rho-related GTP-binding protein RhoB
1.179	0.987	1.075	1.080	1.437	0.961	1.072	0.908	1.054	0.935	1.020	0.914	P08134	RHOC	Rho-related GTP-binding protein RhoC
0.970	0.961	0.971	1.290	1.033	0.958				0.923	1.072	0.952	P61587	RND3	Rho-related GTP-binding protein RhoE
0.969	1.048	1.041	0.997	0.853	1.038	1.058	1.161	1.012	1.038	1.034	1.027	Q9HBH0	RHOF	Rho-related GTP-binding protein RhoF
1.010	0.993	1.002	1.039	1.064	0.998	1.054	0.909	1.033	1.061	1.016	0.996	P84095	RHOG	Rho-related GTP-binding protein RhoG
0.795	1.062	1.343	1.086	0.960	1.122	1.110	1.018	1.052	1.053	0.934	1.081	P17081	RHOQ	Rho-related GTP-binding protein RhoQ
0.968	0.974	1.033	0.996	1.003	1.010	1.020	1.126	1.071	1.016	1.098	1.016	Q969G6	RFK	Riboflavin kinase
1.002	0.975	1.009	0.971	1.061	1.018	1.062	0.975	0.989	1.092	1.023	1.099	Q9H477	RBKS	Ribokinase
0.944	1.048	1.029	0.932	0.925	0.882	0.916	0.884	1.147	0.984	0.966	1.027	Q9NRR4	DROSHA	Ribonuclease 3
1.121	0.892	1.075	0.919	0.825	1.205	1.063	1.169	1.040				O60930	RNASEH1	Ribonuclease H1
1.056	1.016	1.049	0.954	0.966	1.017	1.031	1.083	0.992	1.004	1.030	1.048	O75792	RNASEH2A	Ribonuclease H2 subunit A
1.036	1.032	0.987	0.968	0.919	0.996	1.041	1.085	1.001	0.980	1.008	1.070	Q5TBB1	RNASEH2B	Ribonuclease H2 subunit B
1.166	0.934	0.994	1.016	0.917	1.046	1.277	0.922	1.005	0.953	0.904	1.076	E9PN81	RNASEH2C	Ribonuclease H2 subunit C
									1.488	1.530	1.907	H0YCR7	RNH1	Ribonuclease inhibitor (Fragment)
1.042	0.976	1.020	1.017	1.203	1.014	1.124	0.870	1.079	1.004	0.969	0.996	P13489	RNH1	Ribonuclease inhibitor
1.051	1.077	1.157	1.032	0.933	1.051	0.953	1.197	0.978	0.956	0.927	1.115	O95059	RPP14	Ribonuclease P protein subunit p14
1.030	1.042	1.067	1.040	1.005	0.918	1.009	1.017	0.977	1.005	1.023	0.974	O75817	POP7	Ribonuclease P protein subunit p20
			0.885	1.109	1.001	0.894	0.852	0.868	0.819	1.066	1.000	A0A0G2JIT5	RPP21	Ribonuclease P protein subunit p21
1.053	0.990	0.953	0.988	0.931	1.046	0.967	1.059	1.003	0.902	1.007	1.012	Q9BUL9	RPP25	Ribonuclease P protein subunit p25
1.025	0.998	1.091	0.988	1.052	1.138	0.972	1.018	1.093	1.032	0.980	0.942	Q8N5L8	RPP25L	Ribonuclease P protein subunit p25-like protein
0.959	1.001	1.025	0.978	1.030	1.022	0.951	0.980	0.979	0.961	0.989	1.156	O95707	POP4	Ribonuclease P protein subunit p29
1.048	1.006	1.058	1.022	1.014	0.978	1.001	1.068	0.979	1.103	1.023	1.057	P78345	RPP38	Ribonuclease P protein subunit p38

Abundance Ratio														
1 d M1/C1	1 d M2/C2	1 d M3/C3	4 d M1/C1	4 d M2/C2	4 d M3/C3	7 d M1/C1	7 d M2/C2	7 d M3/C3	10 d M1/C1	10 d M2/C2	10 d M3/C3	Accession ID	Gene name	Protein Description
1.010	0.982	0.998	0.961	0.945	1.047	0.956	1.126	0.991	0.978	0.993	1.010	O75818	RPP40	Ribonuclease P protein subunit p40
1.324	1.141	0.867	1.018	0.997	1.166	1.005	1.210	1.008	0.888	0.890	1.122	Q969H6	POP5	Ribonuclease P/MRP protein subunit POP5
1.017	0.957	0.948	0.996	0.998	1.021	1.078	1.107	0.992	1.081	0.963	1.097	A0A087WZM2	RNASET2	Ribonuclease T2
0.958	1.050	1.008	0.976	0.958	1.009	0.951	1.011	1.036	0.994	0.994	1.007	P52758	HRSP12	Ribonuclease UK114
0.993	0.996	1.008	0.988	0.991	0.998	0.963	1.063	0.984	0.977	0.997	1.017	Q99575	POP1	Ribonucleases P/MRP protein subunit POP1
1.031	1.012	1.021	1.030	0.998	1.022	1.019	1.058	1.051	1.011	1.017	1.036	E9PAU2	RAVER1	Ribonucleoprotein PTB-binding 1
1.068	0.948	1.011	0.931	0.904	0.937	0.818	0.759	1.008	0.913	0.977	0.959	Q9HCJ3	RAVER2	Ribonucleoprotein PTB-binding 2
0.987	0.979	1.022	1.052	1.007	0.990	1.080	1.006	0.856	0.947	0.956	1.057	P23921	RRM1	Ribonucleoside-diphosphate reductase large subunit
1.011	1.079	1.014	0.999	0.992	1.034	0.988	1.029	1.006	0.974	0.990	1.004	P49247	RPIA	Ribose-5-phosphate isomerase
			1.038	0.900	0.936							B1ALA9	PRPS1	Ribose-phosphate pyrophosphokinase 1
0.920	0.976	0.983	0.976	1.045	1.012	1.002	0.974	1.006	0.940	0.979	1.006	P60891	PRPS1	Ribose-phosphate pyrophosphokinase 1
1.011	0.980	0.995	0.980	1.055	1.023	0.986	1.020	1.064	0.954	0.932	0.994	P11908	PRPS2	Ribose-phosphate pyrophosphokinase 2
1.006	0.993	1.030	0.989	0.987	1.002	1.002	0.928	1.022	1.004	0.987	1.054	Q9Y4W2	LAS1L	Ribosomal biogenesis protein LAS1L
0.984	0.998	0.984	0.984	1.071	1.015	0.957	0.936	0.995	1.016	1.056	1.012	O76021	RSL1D1	Ribosomal L1 domain-containing protein 1
1.048	1.023	0.971	0.957	0.933	1.017	1.046	1.031	1.017	1.112	1.039	1.100	Q9H6W3	RIOX1	Ribosomal oxygenase 1
1.020	0.986	0.950	1.000	0.923	0.972	1.024	1.105	0.983	1.029	1.018	0.987	Q8IUF8	RIOX2	Ribosomal oxygenase 2
1.000	1.005	1.009	0.950	0.945	1.054	0.964	1.038	0.968	1.028	0.983	1.059	Q9BQC6	MRPL57	Ribosomal protein 63, mitochondrial
0.966	1.013	0.998	1.036	0.958	0.990	1.019	1.045	1.002	0.998	0.967	1.052	P51812	RPS6KA3	Ribosomal protein S6 kinase alpha-3
1.012	0.977	1.043	1.003	0.983	1.010	1.009	0.972	0.996	0.997	0.961	0.999	O75676	RPS6KA4	Ribosomal protein S6 kinase alpha-4
1.049	0.995	0.969	0.975	1.012	1.019	0.977	1.053	0.967	0.924	0.971	0.899	O75582	RPS6KA5	Ribosomal protein S6 kinase alpha-5
1.084	0.963	1.039	1.056	0.924	0.967	1.024	1.068	1.034	1.067	1.005	1.062	P23443	RPS6KB1	Ribosomal protein S6 kinase beta-1
1.030	1.106	1.018	1.119	0.998	1.152	1.057	1.082	1.030	1.052	1.064	1.116	Q9UBS0	RPS6KB2	Ribosomal protein S6 kinase beta-2
									0.940	0.866	1.145	Q96S38	RPS6KC1	Ribosomal protein S6 kinase delta-1
0.988	0.948	1.029	0.965	0.970	1.049	1.005	0.958	1.001	1.019	0.989	1.049	P56182	RRP1	Ribosomal RNA processing protein 1 homolog A
0.977	1.006	1.028	0.998	0.952	1.002	0.995	1.035	0.965	0.976	1.009	1.053	Q14684	RRP1B	Ribosomal RNA processing protein 1 homolog B
0.894	0.938	1.062	0.931	1.077	1.054	0.959	0.556	1.015	0.974	1.046	0.992	Q96EU6	RRP36	Ribosomal RNA processing protein 36 homolog
1.054	1.007	1.015	1.014	1.009	1.038	0.987	1.092	1.023	0.941	0.997	0.956	Q92979	EMG1	Ribosomal RNA small subunit methyltransferase NEP1
1.010	1.026	1.024	1.019	1.158	1.024	1.037	1.012	1.041	0.992	1.029	1.114	Q9Y3A4	RRP7A	Ribosomal RNA-processing protein 7 homolog A
1.030	1.012	1.000	1.014	0.973	1.083	1.049	1.119	0.976	1.000	1.028	1.066	O43159	RRP8	Ribosomal RNA-processing protein 8
1.004	0.996	1.001	0.984	0.939	0.986	1.010	1.021	0.984	1.000	1.019	1.039	Q14692	BMS1	Ribosome biogenesis protein BMS1 homolog
0.981	0.994	1.001	0.985	0.951	1.009	0.991	1.000	0.997	0.993	1.008	1.027	Q14137	BOP1	Ribosome biogenesis protein BOP1
1.013	1.006	1.024	1.026	1.051	1.022	0.996	1.036	1.035	0.975	1.034	0.976	Q8TDN6	BRIX1	Ribosome biogenesis protein BRX1 homolog
1.025	1.009	1.009	1.067	0.967	1.013	1.006	1.064	0.993	0.970	1.028	0.972	O95478	NSA2	Ribosome biogenesis protein NSA2 homolog

Abundance Ratio														
1 d M1/C1	1 d M2/C2	1 d M3/C3	4 d M1/C1	4 d M2/C2	4 d M3/C3	7 d M1/C1	7 d M2/C2	7 d M3/C3	10 d M1/C1	10 d M2/C2	10 d M3/C3	Accession ID	Gene name	Protein Description
1.032	1.019	1.074	0.975	1.001	0.964	1.032	0.921	1.021	1.042	1.084	1.119	Q9UJK0	TSR3	Ribosome biogenesis protein TSR3 homolog
1.019	0.979	1.021	1.006	1.062	1.028	0.990	0.981	1.017	0.956	1.018	1.012	Q9GZL7	WDR12	Ribosome biogenesis protein WDR12
0.967	0.979	0.994	0.951	0.969	1.048	0.968	0.898	1.035	1.010	1.027	1.028	Q15050	RRS1	Ribosome biogenesis regulatory protein homolog
1.043	1.010	0.998	1.006	1.053	1.011	1.004	0.964	1.019	1.024	0.982	1.003	A0A087X020	SBDS	Ribosome maturation protein SBDS
1.024	0.948	1.035	0.942	0.968	1.008	0.934	1.004	1.035	1.021	0.958	0.929	Q9H9Y2	RPF1	Ribosome production factor 1
1.052	0.984	1.011	0.987	1.004	0.996	0.942	0.921	0.941	0.990	1.059	0.968	Q9H7B2	RPF2	Ribosome production factor 2 homolog
0.926	0.966	1.029	0.938	0.981	1.027	0.933	0.769	0.950	1.044	0.998	1.063	Q9P2E9	RRBP1	Ribosome-binding protein 1
1.009	0.986	1.008	0.983	0.954	0.986	1.034	0.957	0.977	1.052	0.983	1.054	Q96E11	MRRF	Ribosome-recycling factor, mitochondrial
1.007	0.996	1.068	0.979	0.985	0.983	1.041	1.026	1.018	0.985	0.954	1.038	Q969S9	GFM2	Ribosome-releasing factor 2, mitochondrial
0.930	0.997	1.148	1.021	0.968	1.061	0.943	1.068	1.049	1.015	1.048	1.053	P16083	NQO2	Ribosyldihydronicotinamide dehydrogenase [quinone]
1.060	0.967	0.996	0.990	0.992	0.985	1.004	0.928	1.092	0.975	1.032	0.997	Q96AT9	RPE	Ribulose-phosphate 3-epimerase
0.968	0.959	1.042	0.958	0.975	1.100	1.023	1.018	1.037	1.024	0.978	1.046	Q5EBL4	RILPL1	RILP-like protein 1
0.956	0.750	0.803	0.808	1.004	1.157	1.130	0.754	0.819				Q969X0	RILPL2	RILP-like protein 2
			0.930	0.798	1.150	1.052	0.860	0.977	0.869	1.123	1.632	A6NJZ7	RIMBP3C	RIMS-binding protein 3C
									1.060	1.041	1.043	Q96PM5	RCHY1	RING finger and CHY zinc finger domain-containing protein 1
1.021	1.005	0.961	0.982	0.875	0.986	1.083	0.948	1.106	1.012	0.929	0.944	Q96DX4	RSPRY1	RING finger and SPRY domain-containing protein 1
0.922	0.924	0.937	1.151	1.087	1.192	0.984	0.990	1.082				Q9Y3C5	RNF11	RING finger protein 11
0.960	1.010	0.947	1.041	1.158	1.082	1.048	0.947	1.029	1.048	0.988	1.038	O15541	RNF113A	RING finger protein 113A
1.045	1.201	0.930	0.877	1.032	1.149	1.134	0.933	0.861	0.942	1.077	1.027	Q96A37	RNF166	RING finger protein 166
0.983	0.986	0.986	0.905	0.790	1.250	0.958	0.957	1.005	0.917	1.036	0.889	Q9BXT8	RNF17	RING finger protein 17
									1.243	1.103	1.030	Q8N4F7	RNF175	RING finger protein 175
0.982	0.987	1.017	0.995	0.993	1.005	1.024	0.957	0.983	1.048	1.040	1.066	Q8ND24	RNF214	RING finger protein 214
1.163	0.856	1.093	0.889	1.079	0.963	0.858	0.838	1.225	0.988	1.103	1.129	Q5W0B1	RNF219	RING finger protein 219
1.057	0.906	1.067							0.889	1.024	0.822	O94941	UBOX5	RING finger protein 37
1.041	0.983	1.040	1.035	0.970	1.022	1.028	0.999	0.951	1.094	1.025	1.089	Q9C0B0	UNK	RING finger protein unkempt homolog
1.023	0.997	1.007	1.004	0.949	1.040	0.991	0.952	1.009	1.005	1.115	1.073	Q8N488	RYBP	RING1 and YY1-binding protein
						1.088	0.806	1.024	1.025	0.977	1.031	Q9UBF6	RNF7	RING-box protein 2
1.084	0.989	1.051	1.056	0.917	0.972	1.015	0.981	0.995	0.970	1.022	1.311	Q15633	TARBP2	RISC-loading complex subunit TARBP2
0.979	0.986	1.044	0.983	0.980	1.000	0.987	1.058	1.001	1.007	0.992	0.949	Q9Y2P8	RCL1	RNA 3'-terminal phosphate cyclase-like protein
0.993	1.002	0.994	0.990	1.011	1.014	1.018	0.942	1.018	1.054	1.060	1.065	A0A0A0MR66	RBM10	RNA binding motif protein 10, isoform CRA_d
1.002	0.986	0.999	0.983	1.011	1.061	0.953	0.975	1.035	0.973	1.021	0.970	J3KPD3	RBM7	RNA binding motif protein 7, isoform CRA_c
1.040	1.042	1.035	1.002	1.042	1.027	0.994	0.874	1.110	0.990	0.986	1.013	Q96E39	RBMXL1	RNA binding motif protein, X-linked-like-1

Abundance Ratio														
1 d M1/C1	1 d M2/C2	1 d M3/C3	4 d M1/C1	4 d M2/C2	4 d M3/C3	7 d M1/C1	7 d M2/C2	7 d M3/C3	10 d M1/C1	10 d M2/C2	10 d M3/C3	Accession ID	Gene name	Protein Description
0.996	0.994	1.010	1.008	1.017	1.029	1.008	1.030	0.998	0.964	1.012	0.968	Q9H0A0	NAT10	RNA cytidine acetyltransferase
1.090	1.030	1.004	1.289	0.854	1.032	1.092	1.064	1.129	1.015	1.008	1.019	Q8N1G1	REXO1	RNA exonuclease 1 homolog
1.008	1.051	1.000	0.977	0.965	1.041	0.982	1.076	1.039	1.000	1.068	0.993	Q9GZR2	REXO4	RNA exonuclease 4
1.115	1.128	1.052	1.048	0.950	0.937	1.052	1.015	0.990	1.164	1.067	1.097	P55199	ELL	RNA polymerase II elongation factor ELL
1.009	1.009	1.045	0.998	0.973	1.050	1.022	0.966	1.025	1.050	1.032	1.075	Q9Y5B0	CTDP1	RNA polymerase II subunit A C-terminal domain phosphatase
1.075	0.974	1.007	1.044	1.084	0.920	1.043	1.091	1.037	0.997	1.017	1.001	Q9NP77	SSU72	RNA polymerase II subunit A C-terminal domain phosphatase SSU72
0.982	0.993	1.002	0.967	0.940	1.007	0.994	1.025	0.974	0.971	1.011	1.043	Q8N7H5	PAF1	RNA polymerase II-associated factor 1 homolog
0.853	0.937	1.026	1.039	1.011	1.060	1.030	1.141	0.995	0.945	0.983	0.820	Q9BWH6	RPAP1	RNA polymerase II-associated protein 1
0.987	0.984	1.014	0.957	0.962	0.987	0.987	0.995	0.995	1.015	0.980	1.043	Q9H6T3	RPAP3	RNA polymerase II-associated protein 3
1.134	1.001	1.070	0.984	0.949	1.090	1.007	1.198	1.015	0.896	0.979	1.130	Q9NYV6	RRN3	RNA polymerase I-specific transcription initiation factor RRN3
1.032	1.006	0.971	1.007	0.963	0.986	1.039	1.130	1.004	1.008	0.986	1.053	Q6PD62	CTR9	RNA polymerase-associated protein CTR9 homolog
1.006	1.019	0.965	0.943	0.955	0.955	0.955	1.041	0.979	0.951	1.067	0.990	Q8WVC0	LEO1	RNA polymerase-associated protein LEO1
1.012	0.997	1.021	1.011	1.009	1.028	1.036	1.046	0.990	0.993	1.024	1.024	Q92541	RTF1	RNA polymerase-associated protein RTF1 homolog
1.130	0.968	1.219	0.940	1.008	1.038	1.013	0.737	1.058	1.444	0.938	1.042	Q9UJJ7	RPUSD1	RNA pseudouridylate synthase domain-containing protein 1
1.114	1.047	1.045	0.976	0.893	1.042	1.121	0.962	0.993	1.129	1.004	1.128	Q8IZ73	RPUSD2	RNA pseudouridylate synthase domain-containing protein 2
0.966	0.940	1.032	0.997	0.927	1.026	0.981	1.049	1.003	0.993	0.981	1.067	Q6P087	RPUSD3	RNA pseudouridylate synthase domain-containing protein 3
1.036	1.073	0.908	1.120	1.012	1.021	1.148	0.971	0.940	1.235	0.994	1.039	Q96CM3	RPUSD4	RNA pseudouridylate synthase domain-containing protein 4
1.014	1.062	1.061	1.030	0.980	1.070	1.129	1.107	1.004	0.981	1.061	1.141	O75319	DUSP11	RNA/RNP complex-1-interacting phosphatase
0.865	0.954	0.881	0.838	0.990	1.058	1.075	0.948	0.998	1.293	0.786	1.205	Q5U5Q3	MEX3C	RNA-binding E3 ubiquitin-protein ligase MEX3C
1.022	0.993	1.015	0.955	0.974	1.001	0.943	0.856	0.989	1.002	1.031	0.990	P38159	RBMX	RNA-binding motif protein, X chromosome
1.012	0.996	1.038	0.980	0.840	1.008	0.995	1.090	1.052	0.938	0.916	1.183	Q9Y388	RBMX2	RNA-binding motif protein, X-linked 2
0.958	0.968	1.025	0.938	1.022	0.958	0.943	0.798	0.960	0.983	1.000	1.016	P29558	RBMS1	RNA-binding motif, single-stranded-interacting protein 1
0.951	1.001	0.984	0.990	1.172	0.997	1.061	0.941	0.999	1.046	1.003	1.003	Q15434	RBMS2	RNA-binding motif, single-stranded-interacting protein 2
1.000	0.995	0.971	0.998	0.958	1.035	1.001	1.070	0.998	0.977	1.000	0.995	Q9NTZ6	RBM12	RNA-binding protein 12
1.020	0.999	1.035	1.001	0.956	1.055	0.991	0.981	1.005	1.045	1.023	1.042	Q8IXT5	RBM12B	RNA-binding protein 12B
1.007	1.029	0.980	1.023	0.988	0.968	1.053	1.004	0.966	1.038	1.019	0.969	Q96PK6	RBM14	RNA-binding protein 14
0.973	0.993	0.999	0.963	0.961	1.002	0.990	0.896	0.975	1.020	0.995	1.017	P49756	RBM25	RNA-binding protein 25

Abundance Ratio														
1 d M1/C1	1 d M2/C2	1 d M3/C3	4 d M1/C1	4 d M2/C2	4 d M3/C3	7 d M1/C1	7 d M2/C2	7 d M3/C3	10 d M1/C1	10 d M2/C2	10 d M3/C3	Accession ID	Gene name	Protein Description
1.034	1.004	1.035	0.991	0.983	1.029	0.993	1.006	0.976	1.006	1.001	1.033	Q5T8P6	RBM26	RNA-binding protein 26
1.028	1.003	1.004	0.970	0.952	1.017	0.944	0.927	0.978	0.979	1.043	1.029	Q9P2N5	RBM27	RNA-binding protein 27
1.014	0.993	1.014	0.996	0.977	1.040	0.986	1.049	1.023	1.009	1.042	1.027	Q9NW13	RBM28	RNA-binding protein 28
1.123	1.038	0.995	1.049	1.063	0.909	1.067	0.842	0.922	1.174	1.099	0.976	P98179	RBM3	RNA-binding protein 3
1.057	1.025	1.048	0.972	0.913	0.996	1.018	0.997	1.005	0.986	1.031	1.045	Q96EV2	RBM33	RNA-binding protein 33
1.011	1.006	1.025	1.027	0.984	1.001	1.016	1.086	1.024	1.032	1.061	1.022	P42696	RBM34	RNA-binding protein 34
1.021	1.000	0.939				1.067	0.906	1.043				Q9H0Z9	RBM38	RNA-binding protein 38
1.040	0.975	0.990	1.010	1.035	0.999	1.051	0.975	0.998	1.044	1.021	1.016	Q9BWF3	RBM4	RNA-binding protein 4
			1.005	0.883	0.998	1.076	1.053	0.989				Q96LT9	RNPC3	RNA-binding protein 40
			1.067	0.931	0.850	0.892	0.997	1.015				Q96IZ5	RBM41	RNA-binding protein 41
1.069	1.024	1.037	1.015	0.974	1.011	0.983	1.098	1.013	0.973	1.005	0.973	Q9BTD8	RBM42	RNA-binding protein 42
0.953	0.851	0.853										Q6ZSC3	RBM43	RNA-binding protein 43
0.970	1.037	0.982	1.026	0.946	1.060	0.985	1.097	1.038	0.907	1.094	0.905	Q8IUH3	RBM45	RNA-binding protein 45
0.969	0.931	1.406	1.019	0.725	1.320	0.809	0.827	1.344	0.797	0.928	1.025	Q9BQ04	RBM4B	RNA-binding protein 4B
1.079	1.021	1.019	1.075	1.011	1.104	1.149	1.154	1.084	1.059	1.021	1.163	P52756	RBM5	RNA-binding protein 5
1.020	0.978	1.050	0.989	0.992	1.056	1.012	0.846	1.038	1.003	1.015	1.104	P78332	RBM6	RNA-binding protein 6
1.075	0.986	0.981	0.976	0.971	0.989	1.012	1.031	0.964	1.001	1.027	1.014	Q9Y5S9	RBM8A	RNA-binding protein 8A
									0.925	1.074	0.894	H7BY36	EWSR1	RNA-binding protein EWS (Fragment)
0.893	1.032	0.902	1.023	1.012	0.912	1.039	0.998	0.943	1.078	1.048	1.027	H3BPE7	FUS	RNA-binding protein FUS
1.044	1.017	1.008	0.995	1.012	1.002	1.033	1.032	1.030	0.988	1.013	0.992	Q96DH6	MSI2	RNA-binding protein Musashi homolog 2
1.012	0.978	1.000	1.060	1.011	1.012	1.035	0.960	1.028	1.047	1.042	1.095	Q9ULX3	NOB1	RNA-binding protein NOB1
0.996	0.937	0.948	0.969	0.945	0.987	0.999	1.003	0.971	0.989	0.964	0.986	Q9UNW9	NOVA2	RNA-binding protein Nova-2
0.995	0.981	1.011	1.007	0.978	1.060	1.045	1.031	1.055	1.041	1.082	1.067	Q9NRX1	PNO1	RNA-binding protein PNO1
0.996	0.988	0.966	0.975	0.963	0.979	0.980	0.981	0.968	1.007	1.013	1.000	Q9UKM9	RALY	RNA-binding protein Raly
0.981	1.020	0.957	1.012	0.979	1.000	1.039	1.079	0.989	0.998	1.025	0.987	Q15287	RNPS1	RNA-binding protein with serine-rich domain 1
1.054	1.022	1.004	1.002	0.911	0.996	0.964	0.965	0.961	1.041	0.918	1.066	Q9BTL3	FAM103A1	RNMT-activating mini protein
0.988	0.988	1.025	0.991	0.932	1.055	1.025	0.953	0.994	1.075	1.022	1.072	Q5TZA2	CROCC	Rootletin
1.052	1.124	1.068	0.949	0.903	0.933	0.942	0.830	1.075	1.076	1.133	1.132	Q5TC82	RC3H1	Roquin-1
1.044	0.926	1.064	0.836	0.826	1.008	0.911	0.983	1.003	0.992	0.998	1.003	Q5VWQ0	RSBN1	Round spermatid basic protein 1
0.995	1.017	0.953	1.013	0.956	1.050	1.068	0.997	0.989	1.035	0.957	1.158	Q6PCB5	RSBN1L	Round spermatid basic protein 1-like protein
									1.038	1.059	0.870	Q96MS0	ROBO3	Roundabout homolog 3
0.893	0.993	1.055	0.928	0.902	1.047	0.993	1.226	1.066	0.980	0.981	1.039	A0A0A0MSE7	RPAIN	RPA-interacting protein
1.089	1.022	1.015	1.008	0.986	0.991	0.986	1.015	0.990	0.970	1.009	0.934	P22087	FBL	rRNA 2'-O-methyltransferase fibrillarin
1.033	0.961	0.983	0.979	1.032	0.985	1.068	0.944	1.000	1.072	0.995	1.034	Q6IN84	MRM1	rRNA methyltransferase 1, mitochondrial

Abundance Ratio														
1 d M1/C1	1 d M2/C2	1 d M3/C3	4 d M1/C1	4 d M2/C2	4 d M3/C3	7 d M1/C1	7 d M2/C2	7 d M3/C3	10 d M1/C1	10 d M2/C2	10 d M3/C3	Accession ID	Gene name	Protein Description
1.249	0.933	1.027	0.945	0.856	1.086	1.258	0.946	0.959	1.158	1.085	1.144	Q9UI43	MRM2	rRNA methyltransferase 2, mitochondrial
0.903	0.972	1.023	0.910	0.943	1.038	0.943	0.958	1.030	1.021	0.983	1.060	Q9HC36	MRM3	rRNA methyltransferase 3, mitochondrial
1.038	1.098	1.114	0.939	0.769	0.945	0.954	1.195	0.951	0.926	1.132	0.973	A6NHQ2	FBLL1	rRNA/tRNA 2'-O-methyltransferase fibrillarin-like protein 1
			0.986	1.174	1.024	1.167	0.803	1.261	1.130	0.972	1.020	Q9Y324	FCF1	rRNA-processing protein FCF1 homolog
1.073	1.051	0.993	1.056	1.048	1.128	0.988	0.936	1.152	1.133	0.878	1.047	Q9BRU9	UTP23	rRNA-processing protein UTP23 homolog
1.008	0.988	1.011	1.015	0.979	1.026	1.009	1.091	0.992	0.999	1.047	1.009	Q5JTH9	RRP12	RRP12-like protein
1.081	1.015	1.021	1.006	0.985	1.017	0.984	1.055	0.980	0.980	1.038	0.989	Q9Y3B9	RRP15	RRP15-like protein
0.975	0.994	1.022	0.978	0.959	1.037	1.005	0.958	1.041	1.011	0.964	1.061	Q96T51	RUFY1	RUN and FYVE domain-containing protein 1
1.023	0.985	1.043	1.001	0.911	1.023	1.018	1.044	1.039	0.997	0.969	1.029	Q8WXA3	RUFY2	RUN and FYVE domain-containing protein 2
						1.080	1.252	0.914	1.106	0.971	0.976	Q9BVN2	RUSC1	RUN and SH3 domain-containing protein 1
0.942	1.121	0.835	0.906	0.908	0.929	1.063	1.064	0.913	0.989	1.036	1.020	Q92622	RUBCN	Run domain Beclin-1-interacting and cysteine-rich domain-containing protein
			0.786	0.887	1.194	1.740	1.447	1.196	0.934	0.999	1.463	Q96C34	RUNDC1	RUN domain-containing protein 1
0.964	0.990	1.063	0.958	0.917	1.035	1.025	1.028	1.032	1.020	1.028	1.032	Q96GQ5	C16orf58	RUS1 family protein C16orf58
0.989	0.985	0.984	0.974	0.985	0.978	0.942	0.987	0.995	0.967	0.993	0.977	Q9Y265	RUVBL1	RuvB-like 1
0.997	0.995	0.987	0.998	0.990	0.999	0.994	1.004	0.991	0.977	0.999	0.950	Q9Y230	RUVBL2	RuvB-like 2
1.008	0.960	0.975	0.957	0.931	0.988	0.957	1.020	0.985	0.993	0.951	1.007	Q9H446	RWDD1	RWD domain-containing protein 1
1.079	1.186	1.125										P57060	RWDD2B	RWD domain-containing protein 2B
1.102	0.994	0.905	0.931	0.990	1.051	0.913	1.078	0.972	0.870	0.966	0.922	Q9Y3V2	RWDD3	RWD domain-containing protein 3
1.073	0.977	0.908	0.970	0.937	0.925	0.970	1.086	1.011	0.998	0.999	1.091	Q6NW29	RWDD4	RWD domain-containing protein 4
0.979	0.888	1.141	0.926	0.917	1.063	0.841	1.154	0.853	0.710	0.999	1.061	Q9BY12	SCAPER	S phase cyclin A-associated protein in the endoplasmic reticulum
0.986	0.992	1.043	1.008	1.008	1.034	1.037	1.044	1.034	0.999	1.003	1.085	Q8N5C6	SRBD1	S1 RNA-binding domain-containing protein 1
0.830	0.907	1.424	0.843	0.779	0.995							A0A096LPE2	SAA2-SAA4	SAA2-SAA4 readthrough
			2.165	0.993	0.927	3.249	0.694	1.741				A6NKF1	SAC3D1	SAC3 domain-containing protein 1
1.054	1.006	0.992	1.057	0.904	0.976	1.039	1.089	0.985	1.010	1.042	0.939	Q8NBX0	SCCPDH	Saccharopine dehydrogenase-like oxidoreductase
0.927	1.061	0.851	1.018	1.121	1.000	1.026	1.032	1.201	1.014	1.063	1.071	Q9NZJ4	SACS	Sacsin
0.981	1.004	0.975	1.035	1.066	1.020	1.019	0.994	1.043	1.024	1.025	1.009	O43865	AHCYL1	S-adenosylhomocysteine hydrolase-like protein 1
1.017	0.921	1.129	0.823	0.994	1.085	1.110	0.755	1.270				Q9NV66	TYW1	S-adenosyl-L-methionine-dependent tRNA 4-demethylwyosine synthase
1.055	0.992	1.267	1.056	1.095	1.093	1.072	1.120	1.039	0.967	1.091	1.060	P17707	AMD1	S-adenosylmethionine decarboxylase proenzyme
1.043	0.992	1.076	0.987	0.847	1.049	0.848	0.873	0.971	1.133	0.948	0.987	F8WAB8	SLC25A26	S-adenosylmethionine mitochondrial carrier protein
1.034	0.994	0.998	1.006	1.038	1.020	0.985	0.998	1.025	0.984	1.023	0.973	P31153	MAT2A	S-adenosylmethionine synthase isoform type-2
0.984	0.981	0.994	0.991	0.958	0.961	0.946	0.841	0.934	1.023	1.033	1.022	Q9NWH9	SLTM	SAFB-like transcription modulator

Abundance Ratio														
1 d M1/C1	1 d M2/C2	1 d M3/C3	4 d M1/C1	4 d M2/C2	4 d M3/C3	7 d M1/C1	7 d M2/C2	7 d M3/C3	10 d M1/C1	10 d M2/C2	10 d M3/C3	Accession ID	Gene name	Protein Description
0.993	1.020	0.942	0.962	0.928	0.984	0.966	1.087	1.036	0.978	0.987	1.033	Q96ES7	SGF29	SAGA-associated factor 29
0.927	0.922	0.941	0.925	0.852	0.920	0.876	0.752	1.018	0.925	0.996	1.135	O94885	SASH1	SAM and SH3 domain-containing protein 1
1.023	1.021	0.989	1.005	0.976	1.043	0.989	1.093	1.028	1.015	1.005	1.057	J3QQJ0	SAP30BP	SAP30-binding protein (Fragment)
0.965	0.981	1.011	0.992	0.921	1.000	0.964	0.858	0.975	1.030	1.016	1.118	Q14BN4	SLMAP	Sarcolemmal membrane-associated protein
0.919	1.009	0.980	0.998	0.976	1.011	1.028	1.017	1.013	1.057	1.053	1.006	P16615	ATP2A2	Sarcoplasmic/endoplasmic reticulum calcium ATPase 2
1.014	0.990	1.041	0.955	0.942	1.007	0.988	0.934	0.980	0.972	0.970	0.990	Q14151	SAFB2	Scaffold attachment factor B2
1.041	0.964	1.071	1.218	1.003	1.152	1.269	1.274	1.045	1.265	1.112	1.330	Q8WTV0	SCARB1	Scavenger receptor class B member 1
0.949	0.979	0.986	1.004	0.934	0.982	1.009	1.062	1.000	0.976	0.948	1.017	Q7Z7L1	SLFN11	Schlafen family member 11
0.968	0.994	1.018	0.994	0.994	1.035							Q68D06	SLFN13	Schlafen family member 13
1.029	1.022	1.037	0.996	1.004	1.021	1.035	1.039	1.012	0.989	0.955	1.005	Q08AF3	SLFN5	Schlafen family member 5
									1.138	1.090	1.018	Q9UHJ3	SFMBT1	Scm-like with four MBT domains protein 1
1.024	1.004	1.012	0.986	0.978	1.034	0.998	1.105	1.045	0.988	1.027	1.048	Q6P3W7	SCYL2	SCY1-like protein 2
0.984	0.995	1.022	0.967	1.025	1.028	0.971	0.980	1.017	1.016	0.993	1.014	Q8WVM8	SCFD1	Sec1 family domain-containing protein 1
0.997	0.978	1.053	0.964	0.946	1.009	1.002	1.006	1.119	0.937	0.975	0.993	Q8WU76	SCFD2	Sec1 family domain-containing protein 2
1.015	1.003	1.014	0.976	0.956	0.999	0.981	1.049	0.997	0.905	1.012	0.996	O76054	SEC14L2	SEC14-like protein 2
0.917	0.957	0.950	0.977	0.974	1.035	1.044	1.024	1.053	0.996	0.994	1.065	Q9UDX4	SEC14L3	SEC14-like protein 3
1.007	0.962	0.970	1.006	0.967	1.019	1.005	1.006	0.991	1.019	0.931	0.980	Q9Y6Y8	SEC23IP	SEC23-interacting protein
0.945	0.985	1.000	0.983	0.969	1.059	1.024	0.987	1.083	1.022	1.026	1.034	J3QL71	SCRN2	Secernin-2
0.984	1.005	1.020	0.992	1.016	1.011	1.012	0.994	1.013	0.987	0.937	1.056	Q0VDG4	SCRN3	Secernin-3
0.896	1.018	1.109	0.890	0.822	1.101	0.973	0.798	1.055	1.131	0.821	1.238	Q8WVN6	SECTM1	Secreted and transmembrane protein 1
									0.980	1.064	1.043	Q9UGK8	SERGEF	Secretion-regulating guanine nucleotide exchange factor
			0.979	0.825	0.970	1.041	1.158	0.977				H3BUB6	SCAMP2	Secretory carrier-associated membrane protein (Fragment)
0.960	0.986	1.000	0.992	0.955	1.027	0.980	0.999	1.005	1.094	1.056	1.075	O15126	SCAMP1	Secretory carrier-associated membrane protein 1
1.010	1.011	1.074	1.010	0.966	0.996	1.028	1.019	1.120	1.071	1.040	1.033	O15127	SCAMP2	Secretory carrier-associated membrane protein 2
0.945	0.993	0.989	0.971	0.963	1.085	1.022	0.970	1.022	1.131	1.035	1.089	O14828	SCAMP3	Secretory carrier-associated membrane protein 3
0.947	0.978	1.028	0.961	0.923	1.052	1.005	0.961	1.018	1.061	1.051	1.075	Q969E2	SCAMP4	Secretory carrier-associated membrane protein 4
1.082	1.007	1.091	1.020	0.973	1.009	1.091	1.038	0.961				Q13018	PLA2R1	Secretory phospholipase A2 receptor
0.892	0.931	1.045										O95997	PTTG1	Securin
1.081	1.007	1.031	1.030	0.980	0.948	0.946	1.043	1.058	1.005	1.019	1.096	O14640	DVL1	Segment polarity protein dishevelled homolog DVL-1
0.997	0.974	1.010	0.984	1.002	1.020	0.978	0.977	1.012	1.016	1.048	1.029	O14641	DVL2	Segment polarity protein dishevelled homolog DVL-2

Abundance Ratio														
1 d M1/C1	1 d M2/C2	1 d M3/C3	4 d M1/C1	4 d M2/C2	4 d M3/C3	7 d M1/C1	7 d M2/C2	7 d M3/C3	10 d M1/C1	10 d M2/C2	10 d M3/C3	Accession ID	Gene name	Protein Description
0.951	0.871	0.932	1.021	0.923	1.072	1.009	0.987	1.165	1.007	1.018	1.236	Q92997	DVL3	Segment polarity protein dishevelled homolog DVL-3
0.901	0.899	0.988	0.775	0.736	0.956							A0A087WYL5	SEZ6L2	Seizure 6-like protein 2
1.009	0.991	0.984	1.003	0.958	0.972	0.984	1.080	0.975	0.972	1.004	0.963	P49903	SEPHS1	Selenide, water dikinase 1
			1.043	0.902	1.010	0.968	1.059	1.039	1.015	1.031	1.016	Q99611	SEPHS2	Selenide, water dikinase 2
0.986	1.084	0.978	0.828	0.705	1.096				0.820	1.286	1.451	Q96T21	SECISBP2	Selenocysteine insertion sequence-binding protein 2
0.949	0.981	1.072	0.977	0.855	1.075				0.937	1.050	1.144	Q93073	SECISBP2L	Selenocysteine insertion sequence-binding protein 2-like
0.985	1.007	0.970	1.006	1.051	1.004	0.981	1.050	1.033	0.967	1.016	1.019	A0A0A0MQU4	SCLY	Selenocysteine lyase
0.938	1.007	1.027	1.029	0.994	1.009	1.057	0.914	1.007	1.122	1.028	1.182	P57772	EEFSEC	Selenocysteine-specific elongation factor
1.017	0.960	0.979	0.959	0.996	1.034	0.964	1.074	1.009	1.001	0.952	1.031	A0A0B4J1S4	SELENOF	Selenoprotein F
1.023	0.953	1.050	0.943	0.899	1.072							Q8IZQ5	SELENOH	Selenoprotein H
0.989	0.926	0.914	0.935	0.710	1.239				0.999	1.105	1.034	Q9Y6D0	SELENOK	Selenoprotein K
1.033	1.008	1.090	0.931	0.890	0.918	1.065	1.115	1.135				Q8WWX9	SELENOM	Selenoprotein M
0.964	1.018	1.089	0.889	1.041	0.990							Q9NZV5	SELENON	Selenoprotein N
1.065	1.009	0.996	1.058	0.922	1.034	1.009	0.960	0.969	1.112	1.004	1.064	Q9BVL4	SELENOO	Selenoprotein O
1.059	1.054	1.059	0.996	0.953	0.949	1.026	0.762	0.923				E9PN30	SELENOS	Selenoprotein S (Fragment)
0.973	1.161	1.064				1.134	1.168	1.110				A0A087WVA1	SELENOT	Selenoprotein T
0.940	0.955	1.097	0.849	0.887	1.002	0.916	0.647	0.900				Q14563	SEMA3A	Semaphorin-3A
1.008	0.976	0.997	0.938	0.852	0.973	0.974	1.007	0.979	1.078	0.939	1.025	Q99985	SEMA3C	Semaphorin-3C
1.015	1.019	1.033	1.015	0.988	1.054	1.117	1.104	0.917	0.915	1.026	0.998	J3KNP4	SEMA4B	Semaphorin-4B
1.035	1.070	0.965	0.982	0.915	1.126	1.078	0.921	1.049	1.137	0.969	1.108	Q9C0C4	SEMA4C	Semaphorin-4C
1.027	0.899	0.882										Q92854	SEMA4D	Semaphorin-4D
1.061	1.017	1.065	1.040	1.002	0.994	1.005	1.053	1.014	1.027	1.010	0.985	Q9P0U3	SENP1	Sentrin-specific protease 1
0.970	0.887	1.124										Q9HC62	SENP2	Sentrin-specific protease 2
1.028	1.000	1.025	1.033	0.950	1.010	1.012	1.064	1.011	1.046	1.005	1.018	Q9H4L4	SENP3	Sentrin-specific protease 3
1.026	1.112	1.195										Q96HI0	SENP5	Sentrin-specific protease 5
0.887	0.928	1.104	0.951	1.166	0.935	0.989	0.934	0.890	0.837	1.088	0.838	Q9GZR1	SENP6	Sentrin-specific protease 6
1.056	0.983	1.214	0.829	0.950	1.167				0.865	0.944	1.033	Q96LD8	SENP8	Sentrin-specific protease 8
1.069	1.421	1.093										Q14674	ESPL1	Separin
1.023	1.003	1.021	0.988	1.067	1.014	0.973	0.999	1.044	0.967	0.976	0.988	P35270	SPR	Sepiapterin reductase
0.994	0.973	0.979	0.969	0.969	0.956	0.985	1.035	1.018	0.998	1.001	1.016	B5ME97	10-Sep	Septin 10, isoform CRA_c
			0.980	0.880	1.086	1.036	1.336	1.045	1.349	0.898	0.977	J3KNL2	1-Sep	Septin-1
0.827	1.026	0.957	0.984	0.909	1.093	0.996	1.119	1.033	0.948	0.924	1.015	C9IY94	2-Sep	Septin-2 (Fragment)

Abundance Ratio														
1 d M1/C1	1 d M2/C2	1 d M3/C3	4 d M1/C1	4 d M2/C2	4 d M3/C3	7 d M1/C1	7 d M2/C2	7 d M3/C3	10 d M1/C1	10 d M2/C2	10 d M3/C3	Accession ID	Gene name	Protein Description
1.037	1.027	1.008	1.010	0.958	0.962	1.007	1.076	1.002	0.939	1.003	1.023	Q14141	6-Sep	Septin-6
0.740	1.089	0.856	1.055	1.026	0.988	1.063	0.889	1.102	1.091	1.249	0.805	Q16181	7-Sep	Septin-7
1.059	1.039	1.009	0.882	1.057	0.876	1.131	1.297	1.028	1.139	1.067	0.936	F8W8I8	8-Sep	Septin-8
1.033	1.000	1.002	1.010	0.969	0.953	1.015	1.004	0.972	1.056	0.985	1.053	Q92599	8-Sep	Septin-8
0.972	0.980	0.985	0.983	0.993	0.991	0.977	0.961	0.979	0.997	1.025	1.001	Q9UHD8	9-Sep	Septin-9
									1.034	0.898	0.847	D6RBF1	SQSTM1	Sequestosome-1
1.070	1.082	1.013	1.069	1.075	1.026	1.060	1.073	1.010	1.008	1.041	1.051	Q13501	SQSTM1	Sequestosome-1
1.007	0.978	0.984	0.981	1.002	0.995	0.999	1.048	1.042	0.995	0.978	1.047	P83111	LACTB	Serine beta-lactamase-like protein LACTB, mitochondrial
1.023	0.922	1.080	0.994	0.956	0.994	1.012	1.110	1.038	1.039	1.006	1.097	Q96GA7	SDSL	Serine dehydratase-like
1.008	1.007	0.991	1.022	1.074	0.995	1.060	1.002	1.016	1.018	0.981	0.987	P34896	SHMT1	Serine hydroxymethyltransferase, cytosolic
0.985	0.993	0.998	1.001	1.021	0.981	0.985	0.999	0.997	0.987	0.975	0.998	P34897	SHMT2	Serine hydroxymethyltransferase, mitochondrial
1.004	0.996	1.107	1.001	0.914	1.173	1.050	0.928	1.158	1.112	1.103	1.190	Q9NRX5	SERINC1	Serine incorporator 1
1.082	0.925	1.364							1.101	1.119	1.026	Q13530	SERINC3	Serine incorporator 3
0.979	0.979	1.065	1.005	0.983	1.004	1.009	0.978	1.011	1.062	1.015	1.041	O15269	SPTLC1	Serine palmitoyltransferase 1
0.990	0.961	0.984	1.014	0.928	0.920	1.014	1.082	0.959	1.052	1.024	1.046	O15270	SPTLC2	Serine palmitoyltransferase 2
0.967	1.016	0.951	0.983	0.910	0.945	0.906	1.083	1.214	1.063	1.012	1.013	O95084	PRSS23	Serine protease 23
						0.852	1.171	0.988				Q92743	HTRA1	Serine protease HTRA1
0.993	0.986	1.044	0.970	0.963	1.017	0.961	1.019	0.993	1.008	0.999	1.019	O43464	HTRA2	Serine protease HTRA2, mitochondrial
0.970	1.011	0.994	0.996	1.010	1.058	1.031	0.936	1.012	1.046	0.952	1.015	Q9GZT4	SRR	Serine racemase
1.030	1.004	1.051	0.995	1.077	0.966	0.995	0.919	0.997	0.919	0.995	0.950	A9Z1X7	SRRM1	Serine/arginine repetitive matrix protein 1
0.993	0.988	1.005	0.985	0.956	0.978	0.959	1.001	0.996	0.988	1.030	1.015	Q9UQ35	SRRM2	Serine/arginine repetitive matrix protein 2
0.928	1.014	1.063	0.991	0.932	1.030	0.930	0.984	1.013	0.996	0.979	1.102	Q96IZ7	RSRC1	Serine/Arginine-related protein 53
1.021	0.993	0.974	0.969	0.986	0.963	0.971	1.003	0.993	0.967	0.986	0.977	O75494	SRSF10	Serine/arginine-rich splicing factor 10
1.011	0.985	0.995	0.973	0.961	1.010	0.994	1.084	1.017	1.048	1.000	1.014	Q05519	SRSF11	Serine/arginine-rich splicing factor 11
1.196	1.161	1.066				0.842	0.549	1.141	0.887	0.927	1.148	Q8WXF0	SRSF12	Serine/arginine-rich splicing factor 12
0.952	0.968	0.907	0.959	0.922	0.968	0.981	0.901	0.921	1.057	0.965	0.962	Q01130	SRSF2	Serine/arginine-rich splicing factor 2
1.079	1.018	0.955	1.028	1.117	0.995	1.081	0.938	1.001	1.036	1.022	1.012	P84103	SRSF3	Serine/arginine-rich splicing factor 3
1.054	0.979	0.999	0.977	1.029	1.018	1.015	0.933	0.973	1.025	1.011	1.002	Q08170	SRSF4	Serine/arginine-rich splicing factor 4
1.002	0.994	0.975	1.004	0.975	1.022	0.992	1.000	0.974	1.008	1.036	0.985	Q13243	SRSF5	Serine/arginine-rich splicing factor 5
1.045	0.984	0.978	1.000	0.953	1.001	0.995	0.916	0.968	1.064	1.018	0.951	Q13247	SRSF6	Serine/arginine-rich splicing factor 6
1.034	0.997	0.993	0.999	1.057	0.978	1.033	0.906	0.975	1.013	1.007	1.002	Q16629	SRSF7	Serine/arginine-rich splicing factor 7
1.080	1.094	0.907	1.025	0.986	1.069	0.934	0.799	0.973	0.960	0.931	1.184	Q9BRL6	SRSF8	Serine/arginine-rich splicing factor 8
1.007	0.994	0.984	0.994	1.018	0.993	1.018	1.038	1.004	1.020	1.038	1.004	Q13242	SRSF9	Serine/arginine-rich splicing factor 9

Abundance Ratio														
1 d M1/C1	1 d M2/C2	1 d M3/C3	4 d M1/C1	4 d M2/C2	4 d M3/C3	7 d M1/C1	7 d M2/C2	7 d M3/C3	10 d M1/C1	10 d M2/C2	10 d M3/C3	Accession ID	Gene name	Protein Description
1.011	0.988	0.990	0.987	1.018	0.985	0.985	0.994	1.011	0.954	1.003	0.968	J3KTL2	SRSF1	Serine/arginine-rich-splicing factor 1
0.967	0.986	1.020	0.971	0.989	1.029	1.038	0.967	1.022	1.041	0.985	1.083	O94804	STK10	Serine/threonine-protein kinase 10
0.967	0.976	0.993	0.999	0.924	1.016	1.023	1.018	1.045	0.998	0.997	1.010	Q8N1F8	STK11IP	Serine/threonine-protein kinase 11-interacting protein
0.974	1.172	1.036	0.799	0.944	1.148	0.937	1.138	1.154	0.956	1.004	1.192	B8ZZN3	STK16	Serine/threonine-protein kinase 16
0.933	0.988	1.058	0.746	0.996	0.756	0.768	0.961	1.198				O94768	STK17B	Serine/threonine-protein kinase 17B
0.972	0.973	1.021	0.962	0.969	1.019	1.018	1.032	1.017	1.003	0.967	1.048	Q9Y6E0	STK24	Serine/threonine-protein kinase 24
1.052	1.013	0.984	1.014	1.074	1.010	1.031	1.006	1.062	1.000	0.998	1.007	O00506	STK25	Serine/threonine-protein kinase 25
1.011	0.999	1.014	0.995	0.967	0.999	0.988	1.034	0.975	0.974	0.966	0.967	B4E0Y9	STK26	Serine/threonine-protein kinase 26
1.036	0.988	1.061	0.963	0.921	1.010	0.948	0.890	1.044	0.980	1.003	0.998	Q86UX6	STK32C	Serine/threonine-protein kinase 32C
1.070	1.004	0.953	0.918	1.082	1.069	1.001	0.988	0.975	0.825	1.084	0.984	Q8TDR2	STK35	Serine/threonine-protein kinase 35
0.948	0.932	0.835	0.941	0.868	1.045	1.056	1.009	1.019	1.093	1.174	1.353	Q9NRP7	STK36	Serine/threonine-protein kinase 36
0.962	0.997	1.001	0.984	0.994	0.994	0.988	1.002	1.010	0.971	0.966	1.040	Q15208	STK38	Serine/threonine-protein kinase 38
0.981	1.079	1.081	1.005	0.921	1.084	0.992	1.000	1.083	0.946	0.921	1.019	Q9Y2H1	STK38L	Serine/threonine-protein kinase 38-like
1.008	0.996	0.982	1.011	0.992	1.036	1.027	1.099	1.014	1.019	1.008	1.008	Q13043	STK4	Serine/threonine-protein kinase 4
0.981	0.999	1.023	0.976	1.072	1.025	1.023	1.044	0.993	1.484	0.846	0.832	Q13535	ATR	Serine/threonine-protein kinase ATR
0.859	1.189	1.037	0.936	0.958	0.939	0.928	1.249	0.885	0.977	1.080	0.963	P15056	BRAF	Serine/threonine-protein kinase B-raf
0.992	1.015	1.040	0.998	0.960	1.062	0.969	1.110	0.938	0.899	0.930	1.015	E7EPP6	CHEK1	Serine/threonine-protein kinase Chk1
0.945	0.987	1.005	0.990	0.969	1.001	0.961	0.987	1.029	0.972	0.852	1.040	Q15139	PRKD1	Serine/threonine-protein kinase D1
0.838	1.032	1.117	0.922	0.975	1.197	1.214	0.951	1.136				O94806	PRKD3	Serine/threonine-protein kinase D3
1.091	1.055	1.085				1.046	0.846	1.474	1.118	1.099	1.254	Q96GX5	MASTL	Serine/threonine-protein kinase greatwall
0.915	1.111	1.121	0.961	1.007	1.026	0.956	0.901	0.962	1.228	1.040	1.054	O95835	LATS1	Serine/threonine-protein kinase LATS1
			0.831	1.051	1.029	1.030	0.980	0.870	1.224	0.974	1.172	Q8IWU2	LMTK2	Serine/threonine-protein kinase LMTK2
1.000	1.009	0.986	0.991	0.964	1.005	0.997	1.034	1.103	1.016	1.012	1.035	Q7KZI7	MARK2	Serine/threonine-protein kinase MARK2
0.999	0.973	1.022	0.997	1.009	0.998	1.008	0.995	1.029	1.034	0.974	1.047	Q9Y5S2	CDC42BPB	Serine/threonine-protein kinase MRCK beta
1.004	0.991	1.026	1.004	0.975	1.038	1.006	1.042	1.022	1.013	0.988	1.026	P42345	MTOR	Serine/threonine-protein kinase mTOR
0.980	0.995	1.009	0.993	1.000	0.995	1.004	1.027	1.024	0.992	0.980	1.045	Q16513	PKN2	Serine/threonine-protein kinase N2
0.980	1.001	0.976	0.939	1.069	1.202	1.009	1.179	1.023	0.831	0.721	0.967	Q6P5Z2	PKN3	Serine/threonine-protein kinase N3
0.976	0.950	0.988	1.086	0.909	1.161	1.086	0.940	1.070	1.197	0.999	1.184	P51956	NEK3	Serine/threonine-protein kinase Nek3
1.035	1.058	0.956	1.080	0.926	1.077							P51957	NEK4	Serine/threonine-protein kinase Nek4
1.016	0.988	0.984	1.024	0.988	1.023	1.065	1.073	1.024	1.003	0.987	1.026	Q8TDX7	NEK7	Serine/threonine-protein kinase Nek7
			0.958	0.836	1.167	1.447	0.596	0.972				Q86SG6	NEK8	Serine/threonine-protein kinase Nek8
1.008	1.009	1.033	0.990	0.991	0.993	1.013	1.060	1.083	1.042	1.031	1.043	Q8TD19	NEK9	Serine/threonine-protein kinase Nek9
1.105	1.035	1.054	0.950	0.987	0.985	0.990	1.082	1.003	1.009	1.014	1.002	B4DTS2	PRKD2	Serine/threonine-protein kinase

Abundance Ratio														
1 d M1/C1	1 d M2/C2	1 d M3/C3	4 d M1/C1	4 d M2/C2	4 d M3/C3	7 d M1/C1	7 d M2/C2	7 d M3/C3	10 d M1/C1	10 d M2/C2	10 d M3/C3	Accession ID	Gene name	Protein Description
1.040	0.991	1.017	0.999	1.007	0.977	0.990	1.031	1.007	0.970	0.986	1.011	O95747	OXSR1	Serine/threonine-protein kinase OSR1
0.983	1.002	0.974	1.009	0.957	0.984	1.013	1.053	0.987	1.008	1.017	0.985	Q13153	PAK1	Serine/threonine-protein kinase PAK 1
0.987	1.004	0.988	1.006	0.979	0.985	1.009	1.041	1.015	0.991	1.017	0.999	Q13177	PAK2	Serine/threonine-protein kinase PAK 2
0.983	0.995	1.019	1.021	0.978	1.015	1.010	1.021	0.997	1.018	1.037	1.078	O96013	PAK4	Serine/threonine-protein kinase PAK 4
2.680	2.469	2.635	1.103	0.728	1.010	1.169	1.107	1.048	1.082	1.139	1.162	P53350	PLK1	Serine/threonine-protein kinase PLK1
1.014	0.985	1.004	0.988	0.977	1.008	1.019	0.996	0.986	1.031	0.989	0.999	Q13523	PRPF4B	Serine/threonine-protein kinase PRP4 homolog
0.997	1.043	0.978	0.952	1.011	1.092	1.062	1.130	1.049	0.987	0.936	1.046	Q9BRS2	RIOK1	Serine/threonine-protein kinase RIO1
1.014	0.898	1.017	1.009	1.002	0.956	1.073	1.023	0.981	1.122	1.054	1.112	Q9BVS4	RIOK2	Serine/threonine-protein kinase RIO2
0.992	1.000	1.011	1.033	0.995	1.038	1.004	1.073	1.008	1.112	1.055	1.098	O14730	RIOK3	Serine/threonine-protein kinase RIO3
1.017	0.955	1.015	1.049	1.048	1.194	1.110	1.210	0.868	0.969	0.935	1.047	H0Y4E8	SIK3	Serine/threonine-protein kinase SIK3 (Fragment)
1.024	0.982	1.032	0.970	0.933	1.042	0.981	1.040	1.019	0.993	0.999	1.006	Q96Q15	SMG1	Serine/threonine-protein kinase SMG1
1.019	0.970	1.049	1.010	0.899	1.109	0.922	1.138	1.136	1.020	0.912	1.034	Q15831	STK11	Serine/threonine-protein kinase STK11
0.944	1.000	0.975	0.974	0.975	1.048	1.010	1.010	1.039	1.047	1.070	1.150	Q7L7X3	TAOK1	Serine/threonine-protein kinase TAO1
0.982	1.009	1.029	0.888	0.959	1.150	0.854	0.976	1.041	1.361	1.152	0.777	Q9UL54	TAOK2	Serine/threonine-protein kinase TAO2
1.062	0.968	1.036	1.020	0.950	1.032	0.986	0.917	0.951	1.018	1.021	1.045	Q9H2K8	TAOK3	Serine/threonine-protein kinase TAO3
0.995	0.985	1.018	1.004	0.974	1.061	1.001	1.042	1.015	0.976	1.007	1.013	Q9UHD2	TBK1	Serine/threonine-protein kinase TBK1
0.915	1.042	1.120	1.000	0.984	1.100	0.999	1.191	0.953	0.988	0.899	0.979	O75385	ULK1	Serine/threonine-protein kinase ULK1
1.045	1.008	1.067	1.092	0.955	1.013	1.065	1.041	0.983	1.077	0.973	1.038	Q6PHR2	ULK3	Serine/threonine-protein kinase ULK3
1.047	0.978	1.018	1.028	0.949	1.023	1.057	1.011	1.015	0.991	1.009	1.028	Q99986	VRK1	Serine/threonine-protein kinase VRK1
0.979	0.981	1.001	1.040	0.936	1.012	1.051	1.087	1.014	1.156	1.053	1.089	Q86Y07	VRK2	Serine/threonine-protein kinase VRK2
			1.051	0.880	1.254							Q9Y3S1	WNK2	Serine/threonine-protein kinase WNK2
1.011	1.010	0.978	0.902	0.964	0.839							A0A0U1RRC7	ERN2	Serine/threonine-protein kinase/endoribonuclease IRE2
1.033	1.035	1.011	0.971	0.999	1.009	0.968	0.904	0.977	1.013	1.059	0.994	Q96QC0	PPP1R10	Serine/threonine-protein phosphatase 1 regulatory subunit 10
1.051	0.950	1.050	0.977	1.046	0.794							Q66LE6	PPP2R2D	Serine/threonine-protein phosphatase 2A 55 kDa regulatory subunit B delta isoform
1.017	0.975	0.973	0.971	0.976	1.012	1.015	0.985	0.966	1.052	0.967	0.943	Q15172	PPP2R5A	Serine/threonine-protein phosphatase 2A 56 kDa regulatory subunit alpha isoform
1.141	1.038	1.039	1.001	0.845	0.991	1.023	1.280	1.089	1.061	0.971	0.777	Q15173	PPP2R5B	Serine/threonine-protein phosphatase 2A 56 kDa regulatory subunit beta isoform
0.984	0.997	0.992	0.969	0.967	0.973	0.994	1.046	1.001	0.974	0.990	0.945	Q14738	PPP2R5D	Serine/threonine-protein phosphatase 2A 56 kDa regulatory subunit delta isoform
1.120	0.937	1.072	0.976	0.916	1.019	1.009	1.057	0.996	0.959	0.965	0.909	Q16537	PPP2R5E	Serine/threonine-protein phosphatase 2A 56 kDa regulatory subunit epsilon isoform
0.972	1.000	1.008	0.980	1.005	0.999	0.985	1.022	1.011	0.973	0.961	0.998	P30153	PPP2R1A	Serine/threonine-protein phosphatase 2A 65 kDa regulatory subunit A alpha isoform

Abundance Ratio														
1 d M1/C1	1 d M2/C2	1 d M3/C3	4 d M1/C1	4 d M2/C2	4 d M3/C3	7 d M1/C1	7 d M2/C2	7 d M3/C3	10 d M1/C1	10 d M2/C2	10 d M3/C3	Accession ID	Gene name	Protein Description
1.004	0.955	1.059	0.999	0.939	1.043	0.964	0.963	0.972	1.000	0.971	0.999	A6PVN8	PTPA	Serine/threonine-protein phosphatase 2A activator (Fragment)
0.991	1.000	1.003	0.968	0.961	0.998	0.967	1.058	1.014	0.958	0.993	0.988	Q15257	PTPA	Serine/threonine-protein phosphatase 2A activator
1.049	0.975	0.999	1.053	1.184	1.000	1.066	0.857	1.113	1.029	0.977	0.999	P67775	PPP2CA	Serine/threonine-protein phosphatase 2A catalytic subunit alpha isoform
0.961	0.998	1.006	1.010	1.133	0.981	1.001	0.968	1.031	0.982	0.981	0.966	P62714	PPP2CB	Serine/threonine-protein phosphatase 2A catalytic subunit beta isoform
1.036	0.929	0.876	0.916	0.892	1.221				0.994	0.963	1.077	Q06190	PPP2R3A	Serine/threonine-protein phosphatase 2A regulatory subunit B'' subunit alpha
1.006	1.018	0.997	1.009	0.966	1.106	1.008	1.076	1.018	0.949	0.958	0.970	P60510	PPP4C	Serine/threonine-protein phosphatase 4 catalytic subunit
1.030	1.001	1.061	1.008	0.959	1.043	1.063	0.998	0.961	0.958	0.958	1.047	Q8TF05	PPP4R1	Serine/threonine-protein phosphatase 4 regulatory subunit 1
0.948	0.978	1.023	0.961	0.981	1.025	0.910	0.790	0.955	0.936	0.995	0.983	Q9NY27	PPP4R2	Serine/threonine-protein phosphatase 4 regulatory subunit 2
1.047	1.000	1.030	1.029	0.935	1.033	1.016	1.058	1.048	1.001	0.989	1.015	Q6IN85	PPP4R3A	Serine/threonine-protein phosphatase 4 regulatory subunit 3A
1.107	0.961	1.194	0.987	0.937	0.837				0.968	1.057	1.230	Q6NUP7	PPP4R4	Serine/threonine-protein phosphatase 4 regulatory subunit 4
0.966	0.999	0.998	1.001	1.003	1.002	1.001	0.989	1.001	0.995	1.003	1.036	P53041	PPP5C	Serine/threonine-protein phosphatase 5
1.190	1.056	1.224	1.017	0.841	0.962							Q8NB46	ANKRD52	Serine/threonine-protein phosphatase 6 regulatory ankyrin repeat subunit C
1.032	0.986	0.989	0.960	0.990	1.076	1.029	1.017	1.027	1.077	0.962	1.092	Q9UPN7	PPP6R1	Serine/threonine-protein phosphatase 6 regulatory subunit 1
1.078	1.000	1.034	1.022	1.019	1.014	1.044	1.019	1.031	1.005	0.996	1.038	Q9BRF8	CPPED1	Serine/threonine-protein phosphatase CPPED1
0.998	0.991	1.003	0.972	0.948	1.008	1.030	1.034	1.029	1.007	1.007	1.039	Q96HS1	PGAM5	Serine/threonine-protein phosphatase PGAM5, mitochondrial
1.045	0.999	1.020	1.011	1.036	1.003	1.044	1.049	1.037	1.022	1.026	1.014	P62136	PPP1CA	Serine/threonine-protein phosphatase PP1-alpha catalytic subunit
1.063	1.042	1.017	1.019	1.249	0.987	1.053	0.967	1.086	0.995	0.974	0.983	P62140	PPP1CB	Serine/threonine-protein phosphatase PP1-beta catalytic subunit
1.015	0.984	1.025	0.991	0.930	1.002	0.986	1.058	1.041	0.984	0.992	1.024	Q13315	ATM	Serine-protein kinase ATM
1.004	0.993	1.019	1.005	1.101	0.996	0.966	0.947	1.039	0.991	0.998	0.980	Q5T5C7	SARS	Serine--tRNA ligase, cytoplasmic
1.003	1.015	1.036	0.964	0.961	1.014	0.998	0.992	1.037	1.028	1.001	1.025	M0QWZ7	SARS2	Serine--tRNA ligase, mitochondrial
									0.977	1.071	1.214	Q86SQ7	SDCCAG8	Serologically defined colon cancer antigen 8
									1.085	1.004	1.068	P29508	SERPINB3	Serpin B3
0.971	0.996	0.997	1.006	1.012	0.998	0.995	1.050	1.022	1.004	1.016	0.996	A0A087X1N8	SERPINB6	Serpin B6
1.227	1.059	1.059	0.901	1.015	0.861	1.002	1.232	1.113	1.262	1.040	0.997	O75635	SERPINB7	Serpin B7

Abundance Ratio														
1 d M1/C1	1 d M2/C2	1 d M3/C3	4 d M1/C1	4 d M2/C2	4 d M3/C3	7 d M1/C1	7 d M2/C2	7 d M3/C3	10 d M1/C1	10 d M2/C2	10 d M3/C3	Accession ID	Gene name	Protein Description
1.008	1.036	1.010	1.053	1.049	1.007	1.037	1.011	1.025	0.973	0.944	1.028	P50452	SERPINB8	Serpin B8
									1.014	0.875	1.014	P50453	SERPINB9	Serpin B9
1.001	1.003	0.977	0.971	0.983	0.954	0.979	0.997	1.001	0.998	0.976	0.997	P50454	SERPINH1	Serpin H1
1.024	1.001	1.012	1.001	1.061	0.980	1.001	0.952	1.015	1.019	1.015	1.048	Q9BXP5	SRRT	Serrate RNA effector molecule homolog
1.183	1.101	1.087	1.246	1.170	1.353	1.307	1.158	1.227	1.213	1.076	1.125	P0DJI8	SAA1	Serum amyloid A-1 protein
			0.973	0.734	0.975							P0DJI9	SAA2	Serum amyloid A-2 protein
			0.856	0.851	1.004							P27169	PON1	Serum paraoxonase/arylesterase 1
1.073	0.982	1.076	0.922	0.950	1.051	0.967	0.988	0.934	1.031	1.056	1.007	P11831	SRF	Serum response factor
1.086	0.941	1.010	1.024	0.983	1.069	1.031	1.024	0.964	1.087	1.042	0.997	Q8NEF9	SRFBP1	Serum response factor-binding protein 1
						1.135	1.060	1.031				P58004	SESN2	Sestrin-2
1.209	1.023	1.071	1.245	1.088	1.047							Q8IYR2	SMYD4	SET and MYND domain-containing protein 4
1.036	1.026	1.004	0.996	1.096	1.082	1.069	1.055	1.208	1.011	0.975	1.059	Q6GMV2	SMYD5	SET and MYND domain-containing protein 5
0.997	0.976	1.113	1.000	0.826	1.049	0.994	0.991	1.131				Q8NE22	SETD9	SET domain-containing protein 9
1.005	0.998	1.004	1.007	0.960	1.003	1.040	1.033	0.971	1.007	1.004	1.006	Q9UBL3	ASH2L	Set1/Ash2 histone methyltransferase complex subunit ASH2
1.063	1.003	1.015	0.979	0.941	0.941	0.933	1.143	1.018	0.959	0.991	1.054	Q9UQR0	SCML2	Sex comb on midleg-like protein 2
1.035	1.003	1.008	1.017	1.088	0.969	1.031	1.027	1.015	1.020	0.974	0.958	P10768	ESD	S-formylglutathione hydrolase
1.042	1.043	1.047	0.869	1.100	0.844	1.076	0.953	1.101	1.042	0.972	1.053	Q15464	SHB	SH2 domain-containing adapter protein B
0.961	0.967	1.002				1.085	1.199	0.923				Q9BRG2	SH2D3A	SH2 domain-containing protein 3A
1.011	0.998	1.026	0.948	0.944	1.004	1.025	0.895	1.004	1.026	0.964	1.117	Q9H788	SH2D4A	SH2 domain-containing protein 4A
									0.975	0.960	1.010	Q6ZV89	SH2D5	SH2 domain-containing protein 5
1.091	0.913	1.151	0.964	0.927	0.977	0.925	1.089	1.018	1.042	1.049	1.064	Q9NRF2	SH2B1	SH2B adapter protein 1
0.970	1.085	0.994	0.952	0.896	0.988	0.962	1.071	1.148	0.948	0.984	1.016	Q9UQQ2	SH2B3	SH2B adapter protein 3
			0.922	0.771	0.880	1.034	1.140	0.942				H9KV90	SHANK1	SH3 and multiple ankyrin repeat domains protein 1
0.995	0.995	1.003	1.003	0.971	0.973	0.989	0.959	0.944	0.977	0.982	1.026	A1X283	SH3PXD2B	SH3 and PX domain-containing protein 2B
6.314	6.870	6.533	2.957	3.561	4.761				3.622	4.115	3.693	Q8TF17	SH3TC2	SH3 domain and tetratricopeptide repeat-containing protein 2
0.965	0.967	0.995	0.979	0.971	0.942	0.941	0.904	0.938	1.011	0.960	1.049	Q9H299	SH3BGRL3	SH3 domain-binding glutamic acid-rich-like protein 3
1.089	0.987	0.984	0.974	0.958	0.990	0.997	0.927	0.977	1.049	0.998	0.974	O75368	SH3BGRL	SH3 domain-binding glutamic acid-rich-like protein
1.008	1.004	1.003	0.999	0.984	1.018	1.029	1.050	0.891	0.989	1.005	1.074	Q9Y3L3	SH3BP1	SH3 domain-binding protein 1
1.031	1.024	1.051	1.017	0.967	1.037	0.996	1.135	0.990	0.989	0.983	1.064	Q9P0V3	SH3BP4	SH3 domain-binding protein 4
0.985	1.035	0.988	0.962	0.925	0.979	0.952	0.989	0.965	0.982	0.887	1.040	Q96B97	SH3KBP1	SH3 domain-containing kinase-binding protein 1
1.021	1.020	1.012	0.985	1.026	1.074	1.062	0.908	1.002	1.119	1.010	1.055	A0A0U1RQE4	SH3D19	SH3 domain-containing protein 19

Abundance Ratio												Accession ID	Gene name	Protein Description
1 d M1/C1	1 d M2/C2	1 d M3/C3	4 d M1/C1	4 d M2/C2	4 d M3/C3	7 d M1/C1	7 d M2/C2	7 d M3/C3	10 d M1/C1	10 d M2/C2	10 d M3/C3			
1.103	1.077	1.059	0.961	1.035	1.019	1.030	1.514	1.258	0.887	1.176	1.119	Q8TBC3	SHKBP1	SH3KBP1-binding protein 1
1.038	0.932	0.992	0.940	1.047	1.017	0.992	1.101	1.019	0.944	1.021	1.014	Q9H0F6	SHARPIN	Sharpin
1.005	0.987	0.993	0.992	0.994	0.979	1.014	0.937	0.991	1.021	0.990	1.046	A0MZ66	SHTN1	Shootin-1
0.948	1.052	1.117	0.921	0.862	1.166	0.941	1.079	0.997	0.946	0.933	1.063	Q9UIL1	SCOC	Short coiled-coil protein
1.036	1.003	1.058	0.986	0.831	0.986	0.949	1.252	1.038	0.965	0.920	0.997	Q8TEL6	TRPC4AP	Short transient receptor potential channel 4-associated protein
1.061	1.017	1.034	1.038	0.951	0.972	1.030	1.069	0.989	1.057	0.986	1.021	P45954	ACADSB	Short/branched chain specific acyl-CoA dehydrogenase, mitochondrial
0.977	0.993	1.010	0.991	0.820	0.927	0.987	1.114	1.028				O75911	DHRS3	Short-chain dehydrogenase/reductase 3
0.996	0.998	1.022	1.035	1.015	1.001	1.030	1.020	1.010	1.007	0.963	1.012	P16219	ACADS	Short-chain specific acyl-CoA dehydrogenase, mitochondrial
1.072	0.840	1.125							0.885	0.872	1.035	Q5FBB7	SGO1	Shugoshin 1
0.920	0.945	1.164										Q562F6	SGO2	Shugoshin 2
1.046	0.983	0.966	0.982	0.934	0.954	1.025	0.967	0.984	1.099	0.964	1.000	Q9HAT2	SIAE	Sialate O-acetylesterase
0.981	0.998	0.972	0.989	1.074	0.997	0.986	0.943	1.006	0.980	1.009	0.959	Q9NR45	NANS	Sialic acid synthase
1.110	1.075	1.081	1.020	1.049	1.005	1.029	0.971	0.917	1.057	0.943	1.028	Q99519	NEU1	Sialidase-1
0.919	0.948	1.009	0.889	0.823	1.023	0.920	0.834	0.941	1.043	1.056	1.036	Q9NRA2	SLC17A5	Sialin
									1.016	0.868	0.939	Q5T5P2	KIAA1217	Sickle tail protein homolog
0.979	1.006	0.963	0.998	0.991	1.002	1.011	1.067	1.013	1.022	0.975	1.044	A0A1P0AYU5	SFXN3	Sideroflexin
0.990	0.979	1.039	0.982	0.938	1.015	0.943	1.011	1.006	0.998	1.002	1.023	Q9H9B4	SFXN1	Sideroflexin-1
1.086	0.982	1.063	0.905	0.965	1.020	1.030	1.002	1.002	1.143	1.066	1.142	Q96NB2	SFXN2	Sideroflexin-2
0.921	0.894	1.085	1.063	0.961	1.056	0.937	1.104	0.991	0.937	0.935	1.026	Q6P4A7	SFXN4	Sideroflexin-4
			1.070	1.071	0.980	0.972	0.994	0.896	1.355	1.225	1.136	Q8TD22	SFXN5	Sideroflexin-5
0.981	0.938	1.072	1.000	0.823	1.010	0.987	0.993	1.022	0.923	0.993	0.864	Q99720	SIGMAR1	Sigma non-opioid intracellular receptor 1
0.948	0.932	0.994	0.977	0.883	1.093	1.025	0.932	0.998	1.091	1.019	1.026	P67812	SEC11A	Signal peptidase complex catalytic subunit SEC11A
0.999	0.902	1.049	0.919	0.992	1.093	0.997	0.963	1.007	1.108	0.987	1.129	Q9BY50	SEC11C	Signal peptidase complex catalytic subunit SEC11C
0.969	0.920	0.980	0.955	0.788	0.965	0.991	0.762	0.975	1.048	0.794	1.006	X6R2S6	SPCS1	Signal peptidase complex subunit 1
0.943	0.957	1.021	0.985	0.854	1.026	0.965	0.924	0.961	0.990	0.964	0.945	E9PI68	SPCS2	Signal peptidase complex subunit 2
0.990	0.988	1.009	0.982	0.908	1.006	0.978	1.024	0.997	0.953	0.990	0.983	P61009	SPCS3	Signal peptidase complex subunit 3
1.064	1.105	1.166	1.105	0.907	1.040	0.954	1.126	0.997	1.094	1.232	0.978	Q8TCT8	SPPL2A	Signal peptide peptidase-like 2A
0.998	1.031	0.914	0.986	0.950	0.972	0.992	1.060	0.953	1.046	1.047	0.972	P37108	SRP14	Signal recognition particle 14 kDa protein
0.911	0.977	0.915	1.016	0.944	1.034	1.061	0.990	0.973	0.979	1.013	0.982	P09132	SRP19	Signal recognition particle 19 kDa protein
0.931	1.003	0.975	0.992	0.995	1.017	1.003	0.998	1.019	0.996	0.982	1.011	P61011	SRP54	Signal recognition particle 54 kDa protein
0.939	0.936	0.942	0.957	1.131	0.972	1.001	0.683	1.032	1.011	1.021	0.986	P49458	SRP9	Signal recognition particle 9 kDa protein

Abundance Ratio														
1 d M1/C1	1 d M2/C2	1 d M3/C3	4 d M1/C1	4 d M2/C2	4 d M3/C3	7 d M1/C1	7 d M2/C2	7 d M3/C3	10 d M1/C1	10 d M2/C2	10 d M3/C3	Accession ID	Gene name	Protein Description
0.920	1.009	1.031	1.029	0.951	1.037	1.003	1.026	0.982	0.985	1.005	1.002	P08240	SRPRA	Signal recognition particle receptor subunit alpha
0.965	1.000	1.011	0.988	0.969	0.990	1.043	1.030	0.964	1.011	1.012	0.986	Q9Y5M8	SRPRB	Signal recognition particle receptor subunit beta
0.999	0.996	0.998	1.006	0.996	1.022	0.984	0.986	1.002	0.991	1.015	1.010	Q9UHB9	SRP68	Signal recognition particle subunit SRP68
0.983	1.004	0.999	0.990	1.005	1.012	0.976	0.992	0.976	0.994	1.012	1.016	O76094	SRP72	Signal recognition particle subunit SRP72
0.981	1.009	1.004	1.027	1.015	0.996	1.063	1.017	1.031	1.057	0.986	1.038	P42224	STAT1	Signal transducer and activator of transcription 1-alpha/beta
1.037	1.010	1.031	1.011	1.013	1.031	0.991	1.049	1.022	1.094	0.969	1.001	P52630	STAT2	Signal transducer and activator of transcription 2
0.966	1.000	1.004	0.984	0.939	0.965	0.988	1.009	0.980	0.994	0.956	0.991	P40763	STAT3	Signal transducer and activator of transcription 3
1.012	1.015	0.992	0.996	0.954	1.032	0.990	1.036	1.010	1.051	1.022	1.067	P51692	STAT5B	Signal transducer and activator of transcription 5B
1.016	1.026	1.021	1.031	1.034	1.039	1.000	1.011	0.992	0.987	0.942	0.983	P42226	STAT6	Signal transducer and activator of transcription 6
			1.140	0.918	0.962	1.142	1.038	1.078				J3KPM9	STAT1	Signal transducer and activator of transcription
0.987	1.002	0.985	0.979	0.995	1.059	0.988	0.990	1.029	0.986	1.016	1.016	Q92783	STAM	Signal transducing adapter molecule 1
0.990	0.981	0.985	0.967	0.968	0.997	0.970	0.987	1.009	0.989	0.996	1.028	O75886	STAM2	Signal transducing adapter molecule 2
1.064	1.027	1.054	0.986	0.959	1.016	0.972	1.026	0.987	0.973	0.995	1.064	O43166	SIPA1L1	Signal-induced proliferation-associated 1-like protein 1
1.020	0.993	1.060	0.975	0.865	0.969	1.068	1.120	1.031	0.966	0.949	1.085	O60292	SIPA1L3	Signal-induced proliferation-associated 1-like protein 3
0.999	0.946	1.036	0.941	0.914	1.046	1.007	0.960	0.958	0.985	0.977	1.016	Q96FS4	SIPA1	Signal-induced proliferation-associated protein 1
1.052	1.030	1.053	1.047	1.021	0.973	1.049	1.022	0.993	0.934	0.953	1.018	Q9H7L9	SUDS3	Sin3 histone deacetylase corepressor complex component SDS3
			0.802	0.857	1.133	1.203	0.984	0.907				A2RU48	SMCO3	Single-pass membrane and coiled-coil domain-containing protein 3
0.913	0.983	1.027	0.965	0.981	1.042	1.024	1.171	1.012	1.091	1.050	1.198	Q53HV7	SMUG1	Single-strand selective monofunctional uracil DNA glycosylase
			1.083	0.928	0.984	1.008	1.078	0.992	0.977	0.914	0.960	Q9BWW4	SSBP3	Single-stranded DNA-binding protein 3
0.984	0.997	0.959	0.988	0.953	0.986	0.989	1.039	0.977	1.010	1.007	0.983	Q04837	SSBP1	Single-stranded DNA-binding protein, mitochondrial
0.991	0.982	1.005	1.012	0.928	0.990	0.993	1.129	0.993	0.920	0.959	1.020	Q9BVC3	DSCC1	Sister chromatid cohesion protein DCC1
0.990	1.000	1.009	1.010	0.958	1.022	1.007	0.999	1.011	0.961	0.983	1.012	Q29RF7	PDS5A	Sister chromatid cohesion protein PDS5 homolog A
1.017	1.006	1.048	1.000	0.949	1.019	0.981	1.002	1.012	0.964	0.972	0.994	Q9NTI5	PDS5B	Sister chromatid cohesion protein PDS5 homolog B
0.994	0.907	1.011	1.250	1.794	1.566	0.676	1.045	1.268				O43805	SSNA1	Sjoegren syndrome nuclear autoantigen 1

Abundance Ratio														
1 d M1/C1	1 d M2/C2	1 d M3/C3	4 d M1/C1	4 d M2/C2	4 d M3/C3	7 d M1/C1	7 d M2/C2	7 d M3/C3	10 d M1/C1	10 d M2/C2	10 d M3/C3	Accession ID	Gene name	Protein Description
1.013	1.013	1.002	0.997	1.055	1.031	1.120	0.887	1.020	1.014	1.023	1.015	O60232	SSSCA1	Sjoegren syndrome/scleroderma autoantigen 1
0.942	1.002	1.028	0.976	0.907	1.022	0.978	1.131	1.054	0.968	0.980	1.040	B1PBA3	SMPD4	SKNY protein
			1.255	1.000	1.080							D6RIF6	SLAIN2	SLAIN motif-containing protein 2
0.987	0.995	1.037	1.035	0.973	0.981	0.963	0.916	0.975	1.032	1.007	1.027	Q9P270	SLAIN2	SLAIN motif-containing protein 2
1.041	1.007	1.034	1.032	0.986	0.955	0.997	1.054	1.020	1.037	1.121	1.035	L0R6Q1	SLC35A4	SLC35A4 upstream open reading frame protein
									0.936	0.983	0.735	Q8IW52	SLITRK4	SLIT and NTRK-like protein 4
0.930	1.003	1.082	0.955	0.942	1.022	0.961	1.039	0.955	1.076	1.014	1.032	O75093	SLIT1	Slit homolog 1 protein
0.948	0.992	1.016	0.983	0.961	1.021	0.983	1.001	1.015	0.986	0.957	1.049	A2RUF3	SRGAP2	SLIT-ROBO Rho GTPase activating protein 2
0.971	1.005	1.052	0.974	0.928	1.024	1.042	0.987	1.007	1.003	0.997	1.025	Q7Z6B7	SRGAP1	SLIT-ROBO Rho GTPase-activating protein 1
1.172	1.045	1.377	0.914	0.933	0.973	0.941	0.959	1.078	1.101	0.920	1.109	O43295	SRGAP3	SLIT-ROBO Rho GTPase-activating protein 3
1.143	0.961	1.011	0.923	0.979	1.033	1.017	0.791	0.971	1.034	1.085	1.013	Q8TAD8	SNIP1	Smad nuclear-interacting protein 1
0.982	1.005	1.002	0.939	0.970	0.984	0.938	0.855	0.947	0.996	0.970	0.990	O00193	SMAP	Small acidic protein
0.933	0.962	1.059	0.858	0.885	0.975	0.954	0.668	0.885	1.153	0.876	1.002	P84101	SERF2	Small EDRK-rich factor 2
0.936	0.998	0.925	0.984	1.054	0.997	1.002	0.906	0.983	0.987	1.044	0.974	O43765	SGTA	Small glutamine-rich tetratricopeptide repeat-containing protein alpha
1.049	1.003	1.004	1.012	0.956	1.014	0.996	1.046	0.949	1.045	1.014	0.946	Q96EQ0	SGTB	Small glutamine-rich tetratricopeptide repeat-containing protein beta
									0.996	1.008	1.122	B2RUZ4	SMIM1	Small integral membrane protein 1
1.157	0.583	1.111										P58511	SMIM11A	Small integral membrane protein 11A
1.084	0.970	1.015	1.022	0.993	0.993	0.969	0.973	0.964	1.121	0.952	0.976	Q96EX1	SMIM12	Small integral membrane protein 12
0.924	0.989	0.860	0.858	0.928	0.935				0.955	1.057	1.024	Q7Z3B0	SMIM15	Small integral membrane protein 15
1.069	1.062	1.160				0.963	0.984	0.819				Q8WVI0	SMIM4	Small integral membrane protein 4
1.024	1.068	0.952				1.151	1.112	0.997				Q9Y448	KNSTRN	Small kinetochore-associated protein
1.024	1.014	1.014	0.962	0.956	1.009	0.985	1.057	1.046	0.910	1.002	0.943	P62304	SNRPE	Small nuclear ribonucleoprotein E
1.051	1.077	0.964	1.080	1.399	0.979	1.070	0.966	1.016	1.063	1.072	0.991	P62306	SNRPF	Small nuclear ribonucleoprotein F
1.090	1.004	1.064	1.015	0.993	1.111	1.043	1.131	1.051	1.028	1.057	1.046	P62314	SNRPD1	Small nuclear ribonucleoprotein Sm D1
1.003	0.995	1.002	1.025	1.100	1.019	1.000	0.980	1.017	0.999	1.042	0.957	P62316	SNRPD2	Small nuclear ribonucleoprotein Sm D2
0.945	0.999	0.942	0.970	0.999	1.031	0.997	1.071	1.003	0.984	1.004	0.983	P62318	SNRPD3	Small nuclear ribonucleoprotein Sm D3
1.014	0.986	1.021	1.016	0.925	1.035	1.001	1.046	1.020	0.987	1.057	0.999	O75691	UTP20	Small subunit processome component 20 homolog
0.952	1.033	0.997	0.993	0.920	1.046	0.975	0.871	0.968	1.010	0.953	1.042	B8ZZN6	SUMO1	Small ubiquitin-related modifier 1
0.943	0.939	0.995	0.923	0.912	0.950	0.970	0.886	0.962	1.032	0.958	1.046	P61956	SUMO2	Small ubiquitin-related modifier 2
0.943	0.979	0.948	0.957	0.950	0.977	0.931	0.935	0.941	0.975	0.950	0.961	A8MU27	SUMO3	Small ubiquitin-related modifier 3
0.872	1.025	1.098	0.990	0.818	1.079	1.101	0.948	0.913	1.008	0.947	1.007	Q8NHG7	SVIP	Small VCP/p97-interacting protein
0.997	0.972	1.022	0.966	0.922	0.988	0.985	1.010	0.982	1.002	0.981	1.118	Q9HBD4	SMARCA4	SMARCA4 isoform 2

Abundance Ratio														
1 d M1/C1	1 d M2/C2	1 d M3/C3	4 d M1/C1	4 d M2/C2	4 d M3/C3	7 d M1/C1	7 d M2/C2	7 d M3/C3	10 d M1/C1	10 d M2/C2	10 d M3/C3	Accession ID	Gene name	Protein Description
0.972	0.999	1.047	0.967	0.990	0.990	0.934	1.046	1.051	0.966	0.972	0.992	B4DUC8	MTAP	S-methyl-5'-thioadenosine phosphorylase
0.984	1.025	0.995	0.939	0.949	0.979	0.987	1.014	0.920	1.019	0.953	1.000	F8WES2	MTAP	S-methyl-5'-thioadenosine phosphorylase
0.909	1.057	0.963	0.968	0.892	1.089	1.079	1.024	1.096	1.064	0.924	1.100	Q8NCG7	DAGLB	Sn1-specific diacylglycerol lipase beta
1.041	1.012	0.966	1.026	0.997	1.004	1.012	1.077	1.033	1.049	1.021	1.019	O95295	SNAPIN	SNARE-associated protein Snapin
									1.042	0.962	1.058	A0A0B4J2F2	LOC102724428	SNF1-like kinase, isoform CRA_a
1.055	1.135	0.953	0.997	1.053	0.985	1.169	0.859	0.932				Q9NRH2	SNRK	SNF-related serine/threonine-protein kinase
			1.034	1.019	1.111				1.055	0.988	1.069	Q16533	SNAPC1	snRNA-activating protein complex subunit 1
0.950	1.012	1.199				1.098	1.074	1.267				Q92966	SNAPC3	snRNA-activating protein complex subunit 3
0.927	1.063	1.061	1.236	1.512	1.106	1.076	0.946	0.942	1.029	1.000	1.129	O95149	SNUPN	Snurportin-1
1.012	1.004	0.994	0.998	0.983	0.999	1.017	0.995	0.961	1.026	1.053	1.013	Q13573	SNW1	SNW domain-containing protein 1
			1.044	0.896	1.028	0.995	0.976	1.052	1.048	1.149	1.060	P48029	SLC6A8	Sodium- and chloride-dependent creatine transporter 1
0.864	0.959	0.937										Q96NL6	SCLT1	Sodium channel and clathrin linker 1
									0.941	1.092	1.072	Q9BWG6	SCNM1	Sodium channel modifier 1
1.171	0.949	1.022	1.128	0.942	0.981							P32418	SLC8A1	Sodium/calcium exchanger 1
1.107	1.263	1.354	1.032	1.332	1.115	1.235	2.620	0.989				P19634	SLC9A1	Sodium/hydrogen exchanger 1
0.938	0.993	0.975	1.005	0.922	1.148	1.129	1.027	1.065	1.133	1.041	1.174	A0A087WXD1	SLC9A7	Sodium/hydrogen exchanger
0.983	1.004	1.014	0.884	0.763	0.924	0.985	0.992	0.902	1.039	0.795	1.039	P53794	SLC5A3	Sodium/myo-inositol cotransporter
			1.001	0.899	1.007				1.065	1.022	1.006	Q6J4K2	SLC8B1	Sodium/potassium/calcium exchanger 6, mitochondrial
0.948	0.982	0.981	0.987	0.907	1.005	1.010	0.993	1.016	1.059	1.011	1.014	P05023	ATP1A1	Sodium/potassium-transporting ATPase subunit alpha-1
1.146	0.935	1.071	0.940	0.762	1.092	1.078	0.934	1.134	1.040	1.196	1.085	P50993	ATP1A2	Sodium/potassium-transporting ATPase subunit alpha-2
1.023	1.005	0.983	0.978	0.998	1.067	1.006	1.057	0.909				A0A0A0MT26	ATP1A3	Sodium/potassium-transporting ATPase subunit alpha-3
0.950	1.005	0.987	1.012	0.913	0.958	1.008	0.994	0.954	1.085	0.997	0.963	P05026	ATP1B1	Sodium/potassium-transporting ATPase subunit beta-1
1.009	1.001	0.993	1.019	0.922	0.988	1.051	1.111	1.016	1.029	1.039	0.991	P54709	ATP1B3	Sodium/potassium-transporting ATPase subunit beta-3
0.873	1.014	0.955	0.989	0.828	1.141	1.154	1.061	1.026	1.150	1.051	1.113	F8VX04	SLC38A1	Sodium-coupled neutral amino acid transporter 1
0.992	1.015	1.054	0.992	0.784	1.067	1.059	1.148	1.126	1.128	0.946	1.041	Q96QD8	SLC38A2	Sodium-coupled neutral amino acid transporter 2
									0.924	1.076	1.405	Q8NBW4	SLC38A9	Sodium-coupled neutral amino acid transporter 9
1.122	0.980	1.033	0.879	0.865	0.909	1.015	1.205	0.967	1.075	1.127	1.078	Q9Y289	SLC5A6	Sodium-dependent multivitamin transporter
			1.103	1.226	1.164	1.125	1.155	1.018	1.060	1.094	1.204	Q8WUM9	SLC20A1	Sodium-dependent phosphate transporter 1
			1.039	0.928	0.964	0.899	1.277	1.072				Q86WA9	SLC26A11	Sodium-independent sulfate anion transporter

Abundance Ratio														
1 d M1/C1	1 d M2/C2	1 d M3/C3	4 d M1/C1	4 d M2/C2	4 d M3/C3	7 d M1/C1	7 d M2/C2	7 d M3/C3	10 d M1/C1	10 d M2/C2	10 d M3/C3	Accession ID	Gene name	Protein Description
0.986	0.931	1.022	0.959	0.819	0.923	1.051	1.236	1.033	1.052	0.923	1.141	Q8WVQ1	CANT1	Soluble calcium-activated nucleotidase 1
0.993	0.998	1.048	0.963	0.996	0.973	0.986	1.043	0.960	1.008	1.004	1.016	Q9Y6X4	FAM169A	Soluble lamin-associated protein of 75 kDa
1.014	1.013	1.026	1.051	1.058	1.124	1.056	1.146	1.078	1.048	1.035	1.134	P55011	SLC12A2	Solute carrier family 12 member 2
1.009	1.049	1.021	0.996	0.977	1.085	1.054	1.120	1.113	0.999	0.922	1.035	Q9UP95	SLC12A4	Solute carrier family 12 member 4
0.987	0.979	1.018	1.044	0.945	1.095	1.053	1.081	1.029	1.074	0.995	1.072	A0A0G2JNW7	SLC12A7	Solute carrier family 12 member 7
0.996	0.918	1.049	0.994	0.845	1.044	1.079	1.031	1.110	1.083	1.018	1.056	Q9BXP2	SLC12A9	Solute carrier family 12 member 9
1.146	1.179	1.072	1.025	0.823	1.053	1.091	1.027	1.069	1.110	1.049	1.027	Q8N697	SLC15A4	Solute carrier family 15 member 4
0.885	0.938	0.952	0.875	0.821	1.122	0.971	1.087	1.006	1.113	0.903	1.091	P11166	SLC2A1	Solute carrier family 2, facilitated glucose transporter member 1
0.993	0.967	1.020	1.024	1.045	1.039	1.033	1.035	1.272	1.163	1.040	1.110	A0A024RCG2	SLC22A18	Solute carrier family 22 (Organic cation transporter), member 18, isoform CRA_a
1.022	1.194	0.816	0.771	0.769	0.876							Q9UGH3	SLC23A2	Solute carrier family 23 member 2
1.105	0.962	0.982	0.906	0.888	0.999	1.024	1.180	0.962	0.931	0.889	0.999	Q9BSK2	SLC25A33	Solute carrier family 25 member 33
0.938	0.933	0.961	0.860	0.914	1.021	0.998	1.106	0.966	1.152	1.423	0.912	Q3KQZ1	SLC25A35	Solute carrier family 25 member 35
0.946	1.160	0.888	1.039	1.010	0.972	0.933	1.120	1.140	0.999	0.966	0.973	Q9BZJ4	SLC25A39	Solute carrier family 25 member 39
0.973	0.988	1.030	0.998	0.822	1.014	0.954	0.958	1.007	0.979	0.966	1.005	Q8TBP6	SLC25A40	Solute carrier family 25 member 40
			0.950	0.937	1.105	1.024	1.219	0.926				E9PGQ0	SLC25A44	Solute carrier family 25 member 44
1.123	0.957	1.062	0.967	0.815	1.114	1.084	1.091	0.968	1.163	1.076	1.051	Q96AG3	SLC25A46	Solute carrier family 25 member 46
1.124	1.044	1.091	1.164	1.063	0.996	1.193	1.122	0.915	1.093	1.028	0.979	Q9H1U9	SLC25A51	Solute carrier family 25 member 51
						1.071	0.943	1.312	1.057	0.905	1.253	Q9BXS9	SLC26A6	Solute carrier family 26 member 6
			0.994	1.027	1.038	0.934	0.774	1.238				Q96K37	SLC35E1	Solute carrier family 35 member E1
									1.280	1.022	0.934	Q6ICL7	SLC35E4	Solute carrier family 35 member E4
0.965	1.033	1.042	0.966	0.888	0.980	0.971	1.017	0.963	1.112	1.073	0.971	Q8IXU6	SLC35F2	Solute carrier family 35 member F2
0.939	1.024	1.073	0.914	0.930	1.139	1.028	0.777	0.959	1.359	1.117	1.070	Q8N357	SLC35F6	Solute carrier family 35 member F6
0.975	1.399	1.974	0.947	1.194	1.329	1.060	1.081	0.951				Q96JW4	SLC41A2	Solute carrier family 41 member 2
0.969	0.961	1.245				1.051	1.188	1.070	1.070	1.029	0.967	Q96GZ6	SLC41A3	Solute carrier family 41 member 3
1.022	1.029	1.023	0.871	0.816	1.072	1.036	1.109	1.039	1.144	1.036	0.971	Q9HAB3	SLC52A2	Solute carrier family 52, riboflavin transporter, member 2
0.859	1.024	1.024	0.958	0.889	1.067	1.177	0.985	1.013	1.197	0.971	1.215	Q96BD0	SLCO4A1	Solute carrier organic anion transporter family member 4A1
1.000	0.997	1.011	0.962	0.979	1.030	1.039	1.014	0.972	1.012	1.016	1.039	Q9NQZ2	UTP3	Something about silencing protein 10
1.033	0.964	1.020	1.028	0.927	1.010	1.017	1.051	1.057	1.021	0.926	1.092	Q07889	SOS1	Son of sevenless homolog 1
0.995	1.015	1.021	0.917	0.820	0.960	0.973	0.887	0.956	1.030	0.924	1.069	Q15465	SHH	Sonic hedgehog protein
1.017	0.997	1.010	0.990	0.986	1.025	0.998	0.971	1.032	1.016	0.997	1.007	Q00796	SORD	Sorbitol dehydrogenase
1.485	1.013	1.090	1.364	3.250	0.818	1.304	0.777	1.130	1.274	1.035	1.024	E9PG82	SRI	Sorcin

Abundance Ratio														
1 d M1/C1	1 d M2/C2	1 d M3/C3	4 d M1/C1	4 d M2/C2	4 d M3/C3	7 d M1/C1	7 d M2/C2	7 d M3/C3	10 d M1/C1	10 d M2/C2	10 d M3/C3	Accession ID	Gene name	Protein Description
0.970	1.004	0.957	0.999	1.128	1.022	1.022	0.986	1.009	0.973	0.987	0.946	P30626	SRI	Sorcin
0.883	0.991	1.000										B5MBX0	CDCA5	Sororin
0.971	0.924	1.002	0.998	0.912	1.042				1.017	1.007	1.255	Q99523	SORT1	Sortilin
1.002	1.006	0.982	1.009	1.000	0.962	1.031	1.066	1.009	1.028	1.006	0.999	Q9Y512	SAMM50	Sorting and assembly machinery component 50 homolog
0.979	0.974	0.979	0.988	1.003	1.014	1.003	0.997	1.002	1.017	0.985	1.018	A0A0A0MRI2	SNX6	Sorting nexin
1.017	0.958	0.979	1.030	0.946	0.964	1.077	0.954	0.996	1.134	0.853	1.008	Q13596	SNX1	Sorting nexin-1
						1.053	1.344	0.995				A0A087WUJ3	SNX10	Sorting nexin-10
1.000	0.918	1.112	0.902	0.858	1.063	1.615	0.968	1.082				Q9Y5W9	SNX11	Sorting nexin-11
1.030	1.001	0.972	1.024	0.980	1.005	0.999	1.092	1.000	1.029	0.998	0.974	A0A087X0R6	SNX12	Sorting nexin-12
1.040	1.118	1.038	0.961	0.925	1.035	0.942	1.095	1.029	1.020	1.018	1.042	Q9Y5W8	SNX13	Sorting nexin-13
0.886	1.070	1.142	0.978	0.941	1.033	1.011	1.069	0.935	0.883	0.889	1.052	Q9Y5W7	SNX14	Sorting nexin-14
1.030	0.999	0.996	1.018	0.945	1.010	1.018	1.017	1.022	1.028	0.940	1.025	Q9NRS6	SNX15	Sorting nexin-15
1.085	1.002	1.087	1.145	1.060	0.911	0.948	1.103	0.992	0.953	1.050	0.951	P57768	SNX16	Sorting nexin-16
1.046	1.016	1.005	0.999	1.008	1.039	1.015	1.083	1.035	0.986	0.930	1.035	Q15036	SNX17	Sorting nexin-17
0.982	0.993	0.992	1.003	0.997	1.011	0.984	1.049	0.993	1.002	0.990	0.999	O60749	SNX2	Sorting nexin-2
1.068	0.926	0.977	1.009	0.947	1.052	0.941	1.132	1.036	1.046	0.953	1.111	Q969T3	SNX21	Sorting nexin-21
1.085	0.992	0.993	1.080	0.988	1.132	0.959	1.179	1.037	0.975	0.946	1.024	A8MXB7	SNX24	Sorting nexin-24
1.011	0.992	1.019	1.005	0.918	1.001	0.999	1.060	0.987	1.041	0.975	1.044	Q96L92	SNX27	Sorting nexin-27
0.934	1.022	1.015	1.013	0.975	0.959	0.984	0.933	1.002	1.004	0.913	1.085	Q8TEQ0	SNX29	Sorting nexin-29
1.041	0.952	1.005	1.007	0.979	1.017	0.991	0.937	1.027	1.010	0.940	1.006	O60493	SNX3	Sorting nexin-3
1.039	1.125	1.101	1.026	0.933	1.010	1.047	1.021	0.995	0.934	0.945	1.168	Q5VWJ9	SNX30	Sorting nexin-30
1.011	0.907	1.030	1.237	0.850	1.223	1.054	1.007	1.084	0.993	0.927	1.106	Q8WV41	SNX33	Sorting nexin-33
1.038	0.985	0.989	0.985	0.976	1.005	0.995	0.989	1.038	1.014	0.996	1.038	O95219	SNX4	Sorting nexin-4
1.000	0.984	0.977	1.026	0.994	1.019	1.014	1.009	1.060	1.017	1.007	1.003	Q9Y5X3	SNX5	Sorting nexin-5
1.010	1.010	1.032	0.986	0.958	1.015	0.971	1.087	1.008	0.938	1.011	0.985	Q9Y5X2	SNX8	Sorting nexin-8
1.016	0.988	1.003	1.003	1.048	0.996	0.975	1.010	1.043	0.983	1.017	0.972	Q9Y5X1	SNX9	Sorting nexin-9
1.053	0.969	0.990	1.078	1.008	1.028	1.021	1.106	1.084	1.080	1.069	1.015	Q9BQ15	NABP2	SOSS complex subunit B1
1.041	0.989	1.047	0.962	0.993	0.985	1.021	1.120	1.019	1.056	0.949	1.060	Q9NRY2	INIP	SOSS complex subunit C
1.101	1.025	1.046	1.058	1.025	1.040	1.031	0.992	1.026	1.005	1.009	1.080	Q8N0X7	SPG20	Spartin
1.011	1.006	1.073	0.991	0.958	1.062	1.009	0.987	1.023	1.010	0.916	1.057	Q9UBP0	SPAST	Spastin
0.995	1.005	0.991	0.987	0.947	1.049	0.996	1.056	1.035	0.980	1.028	1.037	Q96JI7	SPG11	Spatacsin
0.956	1.038	0.924	1.090	0.920	0.942	0.935	0.966	0.964	0.969	1.023	1.040	F5H0R1	TUT1	Speckle targeted PIP5K1A-regulated poly(A) polymerase
									1.092	1.008	1.124	A0A0D9SF54	SPTAN1	Spectrin alpha chain, non-erythrocytic 1

Abundance Ratio														
1 d M1/C1	1 d M2/C2	1 d M3/C3	4 d M1/C1	4 d M2/C2	4 d M3/C3	7 d M1/C1	7 d M2/C2	7 d M3/C3	10 d M1/C1	10 d M2/C2	10 d M3/C3	Accession ID	Gene name	Protein Description
0.975	1.001	0.994	0.976	0.986	0.988	0.969	0.974	0.999	0.987	0.959	1.002	Q13813	SPTAN1	Spectrin alpha chain, non-erythrocytic 1
1.311	1.105	1.112	0.999	1.164	1.013	1.042	1.034	0.959				C9JY79	SPTBN4	Spectrin beta chain
0.967	1.002	1.007	0.980	0.984	0.986	0.975	0.954	0.993	0.990	0.974	1.018	Q01082	SPTBN1	Spectrin beta chain, non-erythrocytic 1
1.251	1.023	0.957	0.986	0.994	1.101	0.868	0.877	1.081	1.187	1.127	1.231	O15020	SPTBN2	Spectrin beta chain, non-erythrocytic 2
1.113	1.224	1.258	0.944	0.744	0.976	1.565	1.426	1.582				Q15506	SPA17	Sperm surface protein Sp17
1.028	0.918	1.062	0.970	0.946	1.001	0.966	0.952	1.055	0.921	0.924	1.059	Q07617	SPAG1	Sperm-associated antigen 1
1.048	1.044	0.897	0.844	1.004	0.968	1.035	0.912	0.955	0.993	0.976	1.038	Q6Q759	SPAG17	Sperm-associated antigen 17
0.969	1.034	1.109	1.051	0.737	0.914	0.895	0.863	1.011	1.213	0.965	1.087	Q96R06	SPAG5	Sperm-associated antigen 5
1.026	0.993	1.037	1.006	0.993	1.041	1.010	0.937	0.975	1.030	0.982	1.015	O75391	SPAG7	Sperm-associated antigen 7
1.028	1.015	1.001	0.999	0.984	0.988	1.052	1.013	1.012	1.009	1.034	1.037	Q96SI9	STRBP	Spermatid perinuclear RNA-binding protein
			0.914	0.893	1.251	0.760	1.394	1.222	1.240	1.141	1.366	Q9UM82	SPATA2	Spermatogenesis-associated protein 2
1.199	1.071	0.958				1.115	1.141	0.903				J3KRC8	SPATA33	Spermatogenesis-associated protein 33 (Fragment)
1.021	0.993	0.986	1.006	0.977	1.004	0.981	1.035	0.984	0.979	0.997	1.008	Q8NB90	SPATA5	Spermatogenesis-associated protein 5
1.029	0.982	1.037	1.007	1.023	1.022	1.017	1.059	1.052	0.951	0.971	1.036	Q9BVQ7	SPATA5L1	Spermatogenesis-associated protein 5-like protein 1
1.031	1.024	1.013	1.007	1.042	1.001	1.060	0.966	0.976	1.136	1.030	1.119	Q86XZ4	SPATS2	Spermatogenesis-associated serine-rich protein 2
1.018	1.008	0.971	1.026	1.001	0.999	0.996	1.082	1.036	1.032	1.019	1.003	G3V4K3	VIPAS39	Spermatogenesis-defective protein 39 homolog
0.986	1.008	0.985	1.022	0.990	0.990	1.023	1.084	1.012	0.976	1.035	0.979	P19623	SRM	Spermidine synthase
0.974	0.978	1.009	1.007	1.062	0.997	0.995	0.976	1.020	0.985	0.975	1.051	P52788	SMS	Spermine synthase
0.986	0.969	1.021	1.027	0.976	1.012	1.056	0.967	0.980	1.084	0.987	1.115	P28290	SSFA2	Sperm-specific antigen 2
1.051	0.999	1.033	1.026	1.176	0.980	1.029	0.939	1.052	1.014	0.992	1.018	P63208	SKP1	S-phase kinase-associated protein 1
0.933	0.959	1.011	0.942	0.839	0.995	0.972	1.026	0.954	0.942	0.930	0.986	O15121	DEGS1	Sphingolipid delta(4)-desaturase DES1
			0.811	0.690	1.190				0.934	1.061	0.921	O60906	SMPD2	Sphingomyelin phosphodiesterase 2
1.048	0.929	0.932	1.152	0.919	0.991	1.032	0.977	1.126	1.141	1.191	0.929	P17405	SMPD1	Sphingomyelin phosphodiesterase
0.985	1.006	0.979	1.001	0.936	0.997	1.036	1.095	0.985	1.014	1.024	0.989	O95470	SGPL1	Sphingosine-1-phosphate lyase 1
0.958	0.968	1.030	0.915	1.031	1.112	1.062	0.914	0.997	0.971	1.036	0.950	Q9BX95	SGPP1	Sphingosine-1-phosphate phosphatase 1
			0.870	0.805	1.077							Q8N0Z3	SPICE1	Spindle and centriole-associated protein 1
0.972	1.013	1.002	1.026	0.878	0.944	1.145	1.009	1.001				Q96BD8	SKA1	Spindle and kinetochore-associated protein 1
0.990	1.026	1.014										Q8WVK7	SKA2	Spindle and kinetochore-associated protein 2
0.919	0.868	0.944	0.882	1.075	0.698							Q8IX90	SKA3	Spindle and kinetochore-associated protein 3
1.017	0.900	0.913										Q6UVJ0	SASS6	Spindle assembly abnormal protein 6 homolog
1.002	1.030	0.993	0.979	0.985	1.010	1.005	1.095	1.021	1.014	0.952	1.106	Q9Y657	SPIN1	Spindlin-1
									0.931	0.909	0.950	F6R6M7	DDX39B	Spliceosome RNA helicase DDX39B (Fragment)
0.943	0.837	0.962										F6WLT2	DDX39B	Spliceosome RNA helicase DDX39B (Fragment)

Abundance Ratio														
1 d M1/C1	1 d M2/C2	1 d M3/C3	4 d M1/C1	4 d M2/C2	4 d M3/C3	7 d M1/C1	7 d M2/C2	7 d M3/C3	10 d M1/C1	10 d M2/C2	10 d M3/C3	Accession ID	Gene name	Protein Description
0.998	1.013	1.004	1.019	1.036	1.013	0.971	1.006	0.986	0.989	1.020	0.958	Q13838	DDX39B	Spliceosome RNA helicase DDX39B
1.051	0.921	0.979	1.017	0.897	0.994	1.064	0.796	1.009	1.025	0.984	1.054	Q9P013	CWC15	Spliceosome-associated protein CWC15 homolog
1.058	1.086	1.031	1.027	0.970	1.014	1.078	0.999	0.965	1.078	1.121	1.141	H7C0N4	SF1	Splicing factor 1 (Fragment)
0.990	1.008	0.992	0.985	0.979	1.011	1.004	1.020	0.986	1.005	1.020	1.021	Q15459	SF3A1	Splicing factor 3A subunit 1
0.967	1.005	0.983	0.978	0.972	0.994	0.985	1.022	0.981	1.031	1.014	1.053	Q15428	SF3A2	Splicing factor 3A subunit 2
1.018	1.013	0.985	0.989	1.047	0.997	0.974	0.974	1.023	0.952	1.029	0.985	Q12874	SF3A3	Splicing factor 3A subunit 3
1.004	1.018	1.007	0.994	0.952	0.992	0.999	1.045	0.990	0.987	1.034	1.001	O75533	SF3B1	Splicing factor 3B subunit 1
1.421	1.404	1.152										H0YCG1	SF3B2	Splicing factor 3B subunit 2 (Fragment)
0.962	0.978	0.984	0.974	0.979	0.993	0.978	0.972	0.981	1.008	1.025	1.022	Q13435	SF3B2	Splicing factor 3B subunit 2
1.006	0.998	1.003	0.991	0.974	0.980	0.982	1.040	0.987	0.964	1.006	0.965	Q15393	SF3B3	Splicing factor 3B subunit 3
1.009	1.060	0.981	0.996	0.973	0.990	0.986	1.102	1.012	0.968	1.052	0.976	Q15427	SF3B4	Splicing factor 3B subunit 4
1.001	0.991	0.980	0.995	1.053	0.999	0.971	1.013	1.000	1.018	1.024	1.027	Q9BWJ5	SF3B5	Splicing factor 3B subunit 5
0.944	0.988	0.976	0.992	0.995	1.030	0.998	1.053	1.017	1.032	1.008	1.028	Q9Y3B4	SF3B6	Splicing factor 3B subunit 6
1.008	0.978	1.008	0.962	0.992	0.986	1.012	0.985	1.004	1.016	0.963	1.052	Q96I25	RBM17	Splicing factor 45
1.222	1.011	1.035	1.052	1.462	0.939	1.074	0.851	1.027	0.927	0.988	0.889	Q01081	U2AF1	Splicing factor U2AF 35 kDa subunit
0.986	0.980	0.995	0.982	1.021	0.975	0.958	0.995	1.013	0.952	1.028	0.984	P26368	U2AF2	Splicing factor U2AF 65 kDa subunit
1.009	0.976	1.026	0.967	0.959	1.007	0.943	0.845	0.982	1.007	1.046	1.051	O95104	SCAF4	Splicing factor, arginine/serine-rich 15
0.916	0.998	1.011	0.990	0.979	1.060	0.936	0.975	1.032	1.017	1.007	1.084	Q9H7N4	SCAF1	Splicing factor, arginine/serine-rich 19
0.949	0.996	0.960	0.977	0.990	0.989	0.992	0.982	0.980	1.015	1.005	0.999	P23246	SFPQ	Splicing factor, proline- and glutamine-rich
									1.008	0.841	1.008	P57052	RBM11	Splicing regulator RBM11
1.053	0.893	1.109										Q7Z699	SPRED1	Sprouty-related, EVH1 domain-containing protein 1
1.035	0.792	1.135										F8VWW7	SPRYD3	SPRY domain-containing protein 3
0.983	0.961	0.995	1.012	0.996	1.008	1.030	1.035	1.009	1.057	0.985	1.013	Q8WW59	SPRYD4	SPRY domain-containing protein 4
1.107	1.045	1.051	0.964	1.026	1.014	1.022	1.103	1.028	1.029	1.020	0.993	Q5W111	SPRYD7	SPRY domain-containing protein 7
0.945	0.968	1.074	0.997	0.918	0.977	1.024	1.108	1.001	1.012	0.992	1.020	Q14534	SQLE	Squalene monooxygenase
0.893	1.031	1.042	0.941	0.873	0.937	1.086	1.050	1.144	1.030	1.121	1.065	P37268	FDFT1	Squalene synthase
						1.079	0.976	1.019	0.980	1.032	1.258	F8VV04	SART3	Squamous cell carcinoma antigen recognized by T-cells 3 (Fragment)
1.010	1.004	1.034	1.014	1.092	1.026	1.003	0.962	1.007	1.003	1.010	1.005	Q15020	SART3	Squamous cell carcinoma antigen recognized by T-cells 3
1.037	0.988	1.024	1.015	1.089	0.937	0.988	0.946	1.008	0.992	1.011	0.983	Q9GZT3	SLIRP	SRA stem-loop-interacting RNA-binding protein, mitochondrial
0.980	1.010	0.979	0.986	1.013	0.979	0.980	0.928	0.977	1.043	1.012	1.031	Q14247	CTTN	Src substrate cortactin
1.031	1.030	1.040	0.992	1.011	1.038	0.969	1.088	1.052	0.927	0.987	0.975	Q96SB4	SRPK1	SRSF protein kinase 1

Abundance Ratio														
1 d M1/C1	1 d M2/C2	1 d M3/C3	4 d M1/C1	4 d M2/C2	4 d M3/C3	7 d M1/C1	7 d M2/C2	7 d M3/C3	10 d M1/C1	10 d M2/C2	10 d M3/C3	Accession ID	Gene name	Protein Description
0.921	0.979	1.011	0.952	0.921	1.199	1.083	1.117	1.109				Q9NY15	STAB1	Stabilin-1
1.068	1.100	1.087	1.046	0.901	0.969	1.085	1.098	0.897	1.050	0.944	0.999	O94864	SUPT7L	STAGA complex 65 subunit gamma
1.042	1.013	0.990	1.063	1.035	1.007	1.004	1.007	1.054	1.063	1.010	1.028	O95630	STAMBP	STAM-binding protein
1.086	0.974	0.919	0.930	0.883	1.024	1.051	1.088	0.864	0.958	0.923	1.000	O76061	STC2	Stanniocalcin-2
0.986	0.998	0.989	1.006	1.016	0.985	1.007	1.015	1.010	1.020	1.005	1.019	Q7KZF4	SND1	Staphylococcal nuclease domain-containing protein 1
1.019	1.023	0.926	0.969	0.936	0.926	1.034	1.170	0.999	1.018	0.993	1.042	O95210	STBD1	Starch-binding domain-containing protein 1
0.926	0.989	0.977	1.057	1.253	0.997	1.125	0.996	1.010	1.012	0.967	1.141	Q9Y3M8	STARD13	StAR-related lipid transfer protein 13
			0.975	1.016	1.001				1.040	1.109	1.029	J3QLM1	STARD3	StAR-related lipid transfer protein 3
			1.190	0.873	1.065	1.080	1.131	1.045	1.000	0.974	1.034	Q96DR4	STARD4	StAR-related lipid transfer protein 4
						0.976	1.171	1.028	0.835	0.899	1.179	Q9NSY2	STARD5	StAR-related lipid transfer protein 5
1.027	1.024	0.985	0.974	0.945	0.974	1.009	0.943	0.951	0.886	0.852	1.016	Q9NQZ5	STARD7	StAR-related lipid transfer protein 7, mitochondrial
0.986	0.977	0.980	0.949	0.939	0.968	0.967	0.877	0.954	0.957	0.911	0.983	P16949	STMN1	Stathmin
0.999	0.965	1.001	0.970	0.967	1.084	0.998	0.822	0.974	1.134	1.028	1.122	A0A0J9YW36	STMN3	Stathmin-3 (Fragment)
1.002	0.958	0.985	0.954	0.960	1.007	0.966	0.980	1.013	0.999	0.985	1.083	Q9UEW8	STK39	STE20/SPS1-related proline-alanine-rich protein kinase
1.077	1.037	1.064	1.052	0.977	1.012	1.072	1.094	1.041	1.126	1.074	1.058	Q7RTN6	STRADA	STE20-related kinase adapter protein alpha
			0.939	0.597	2.165							Q7RTU9	STRC	Stereocilin
1.087	0.983	1.015	0.991	1.216	1.136	0.904	1.189	1.063	0.988	0.986	1.166	Q6SZW1	SARM1	Sterile alpha and TIR motif-containing protein 1
1.336	1.198	1.070	0.937	0.856	0.891							Q5K651	SAMD9	Sterile alpha motif domain-containing protein 9
			1.258	0.577	1.036	0.971	1.150	0.961	1.013	1.045	0.830	P11474	ESRRA	Steroid hormone receptor ERR1
1.038	1.018	1.009	0.966	0.959	1.062	0.975	1.030	1.013	1.030	1.071	1.116	Q9HD15	SRA1	Steroid receptor RNA activator 1
0.978	0.974	0.982	0.897	0.903	0.897	0.998	0.921	0.936	1.024	0.868	1.077	Q02318	CYP27A1	Sterol 26-hydroxylase, mitochondrial
0.958	1.023	0.979	0.968	0.904	1.009	0.995	1.009	0.974	1.040	0.981	1.009	P35610	SOAT1	Sterol O-acyltransferase 1
0.984	0.884	1.064	0.967	0.965	1.028	0.872	0.514	0.818	1.308	1.056	1.273	Q12770	SCAP	Sterol regulatory element-binding protein cleavage-activating protein
0.937	1.000	0.999	0.982	0.984	0.993	1.035	1.033	1.040	1.030	1.020	1.056	Q15738	NSDHL	Sterol-4-alpha-carboxylate 3-dehydrogenase, decarboxylating
1.032	0.974	1.012	0.976	0.945	1.001	1.052	1.073	1.044	1.020	1.007	1.067	G0XQ39	STIM1	STIM1L
1.026	0.994	1.019	0.994	0.963	1.025	1.001	1.013	0.998	1.015	0.994	1.008	Q9UJZ1	STOML2	Stomatin-like protein 2, mitochondrial
0.996	1.005	1.005	0.982	0.983	0.968	0.967	0.955	0.955	0.961	1.001	0.967	P38646	HSPA9	Stress-70 protein, mitochondrial
0.976	0.989	0.996	1.011	0.983	1.018	1.018	1.051	0.985	0.985	0.997	1.006	O43815	STRN	Striatin
0.914	1.121	1.008							0.675	0.757	0.922	H0YJT2	STRN3	Striatin-3 (Fragment)
0.968	0.962	0.984	1.039	0.999	1.052	0.996	1.011	1.028	1.027	1.052	1.023	Q13033	STRN3	Striatin-3
1.014	1.025	0.992	1.020	0.961	1.032	1.031	1.093	1.008	1.017	0.993	1.008	Q5VSL9	STRIP1	Striatin-interacting protein 1

Abundance Ratio														
1 d M1/C1	1 d M2/C2	1 d M3/C3	4 d M1/C1	4 d M2/C2	4 d M3/C3	7 d M1/C1	7 d M2/C2	7 d M3/C3	10 d M1/C1	10 d M2/C2	10 d M3/C3	Accession ID	Gene name	Protein Description
1.055	0.939	0.993	0.897	1.005	0.992	0.971	0.797	0.940	0.971	1.063	1.072	Q99470	SDF2	Stromal cell-derived factor 2
1.002	1.015	1.031	0.996	0.941	0.996	0.983	0.939	1.022	1.017	0.995	1.008	Q9HCN8	SDF2L1	Stromal cell-derived factor 2-like protein 1
1.064	1.011	1.106	0.997	0.928	0.975	1.123	1.036	1.050	1.040	1.025	1.062	Q8WU79	SMAP2	Stromal membrane-associated protein 2
1.020	1.016	1.007	1.007	0.975	1.005	1.020	1.048	0.998	1.007	1.003	1.028	A6NHR9	SMCHD1	Structural maintenance of chromosomes flexible hinge domain-containing protein 1
0.975	0.997	0.991	0.981	0.975	1.011	0.992	0.958	0.974	1.017	0.994	1.034	Q14683	SMC1A	Structural maintenance of chromosomes protein 1A
0.964	0.990	1.000	0.982	0.952	0.987	1.026	1.016	0.994	0.951	0.927	1.071	O95347	SMC2	Structural maintenance of chromosomes protein 2
0.995	1.002	0.995	0.989	0.975	1.005	0.995	0.975	0.992	0.991	0.977	1.019	Q9UQE7	SMC3	Structural maintenance of chromosomes protein 3
0.950	0.985	1.016	0.982	0.974	1.000	1.016	1.029	1.010	0.969	0.938	1.087	Q9NTJ3	SMC4	Structural maintenance of chromosomes protein 4
1.007	1.017	1.035	1.014	0.941	1.063	1.043	1.045	0.986	1.009	1.050	1.048	Q8IY18	SMC5	Structural maintenance of chromosomes protein 5
0.866	0.961	0.932	1.006	1.001	1.042	1.066	1.058	1.120	1.052	0.980	0.944	O14521	SDHD	Succinate dehydrogenase [ubiquinone] cytochrome b small subunit, mitochondrial
0.991	1.014	0.974	1.029	1.083	0.997	1.055	0.970	1.037	1.056	1.013	1.030	P31040	SDHA	Succinate dehydrogenase [ubiquinone] flavoprotein subunit, mitochondrial
1.172	0.830	1.055	1.389	0.859	1.114	1.294	0.879	1.082	1.300	0.792	1.108	A0A087WWT1	SDHB	Succinate dehydrogenase [ubiquinone] iron-sulfur subunit, mitochondrial (Fragment)
1.041	1.024	1.014	1.062	1.058	1.006	1.059	1.011	1.024	1.039	1.046	0.987	P21912	SDHB	Succinate dehydrogenase [ubiquinone] iron-sulfur subunit, mitochondrial
0.802	0.835	0.856	0.889	0.913	0.976	0.852	0.744	0.890	0.974	0.968	1.205	Q9NX18	SDHAF2	Succinate dehydrogenase assembly factor 2, mitochondrial
1.130	0.925	0.834	0.970	1.036	1.015	0.837	0.705	1.091	1.239	0.980	1.140	Q9NRP4	SDHAF3	Succinate dehydrogenase assembly factor 3, mitochondrial
1.002	0.965	1.020	0.964	0.927	0.963	0.901	0.869	0.902	1.010	0.995	0.966	Q5VUM1	SDHAF4	Succinate dehydrogenase assembly factor 4, mitochondrial
0.954	0.997	0.973	0.978	0.879	1.095	1.093	1.136	1.074	1.168	1.130	1.027	Q99643	SDHC	Succinate dehydrogenase cytochrome b560 subunit, mitochondrial
1.031	1.015	0.939	0.990	1.111	0.964	0.995	0.978	1.000	1.023	0.982	0.976	P53597	SUCLG1	Succinate--CoA ligase [ADP/GDP-forming] subunit alpha, mitochondrial
1.030	1.060	1.067	0.986	0.968	1.000	0.994	0.990	0.988	1.011	0.964	1.007	Q9P2R7	SUCLA2	Succinate--CoA ligase [ADP-forming] subunit beta, mitochondrial
0.982	0.987	0.985	0.971	0.989	0.990	1.000	0.987	0.988	0.994	0.994	0.980	Q96I99	SUCLG2	Succinate--CoA ligase [GDP-forming] subunit beta, mitochondrial
0.988	1.006	1.016	1.003	1.022	1.026	1.011	1.015	1.042	1.000	1.004	0.972	P55809	OXCT1	Succinyl-CoA:3-ketoacid coenzyme A transferase 1, mitochondrial
			0.972	0.688	1.084	1.287	1.116	0.977				Q8NCC5	SLC37A3	Sugar phosphate exchanger 3

Abundance Ratio														
1 d M1/C1	1 d M2/C2	1 d M3/C3	4 d M1/C1	4 d M2/C2	4 d M3/C3	7 d M1/C1	7 d M2/C2	7 d M3/C3	10 d M1/C1	10 d M2/C2	10 d M3/C3	Accession ID	Gene name	Protein Description
1.024	0.954	0.984	0.917	0.884	0.951	0.866	0.783	0.942	1.065	0.916	0.972	Q8NBK3	SUMF1	Sulfatase-modifying factor 1
1.031	0.990	0.978	0.982	1.017	1.031	1.023	1.042	1.039	1.092	1.043	1.049	J3KQJ1	SUMF2	Sulfatase-modifying factor 2
0.997	0.985	0.988	1.038	0.886	1.031	1.099	1.095	1.064	1.128	1.056	1.079	P50443	SLC26A2	Sulfate transporter
0.963	1.024	0.973										O00391	QSOX1	Sulfhydryl oxidase 1
0.972	1.014	1.026	0.996	0.968	1.019	0.996	1.072	1.028	0.984	1.024	1.025	Q6ZRP7	QSOX2	Sulfhydryl oxidase 2
1.102	1.025	1.019	1.036	1.104	0.987	1.024	1.031	1.044	1.005	1.004	0.971	Q9Y6N5	SQRDL	Sulfide:quinone oxidoreductase, mitochondrial
1.062	1.019	1.011	1.037	1.045	1.048	0.984	1.026	1.006	0.901	1.041	0.970	Q9BYN0	SRXN1	Sulfiredoxin-1
1.007	0.980	1.001	0.951	0.963	1.024	0.997	0.860	0.955	1.093	1.001	1.108	P51687	SUOX	Sulfite oxidase, mitochondrial
1.027	0.877	1.017										M0R286	SAE1	SUMO-activating enzyme subunit 1 (Fragment)
0.995	1.002	0.989	0.992	1.008	1.006	0.998	1.026	1.009	0.981	1.012	0.971	Q9UBE0	SAE1	SUMO-activating enzyme subunit 1
1.006	0.987	0.997	0.991	0.993	1.027	0.990	0.988	1.019	0.995	1.030	0.991	Q9UBT2	UBA2	SUMO-activating enzyme subunit 2
1.002	1.005	0.972	1.003	1.007	0.983	0.984	1.073	0.994	0.991	0.990	0.980	P63279	UBE2I	SUMO-conjugating enzyme UBC9
0.973	0.985	1.010	0.952	0.926	1.000	0.986	0.954	0.997	1.031	0.996	0.976	Q9UH99	SUN2	SUN domain-containing protein 2
0.996	0.998	1.009	0.995	0.993	1.020	0.973	0.970	1.008	0.952	0.986	1.009	P42285	SKIV2L2	Superkiller viralicidic activity 2-like 2
									0.961	0.836	1.026	F5H3C5	SOD2	Superoxide dismutase (Fragment)
1.056	1.003	1.019	0.979	0.968	1.008	0.927	0.840	0.965	0.984	0.983	0.952	P00441	SOD1	Superoxide dismutase [Cu-Zn]
1.128	1.105	1.104	1.157	1.095	1.085	1.091	1.109	1.082	1.156	0.976	1.096	P04179	SOD2	Superoxide dismutase [Mn], mitochondrial
1.045	0.957	1.090	0.972	0.915	1.077							Q86UD0	SAPCD2	Suppressor APC domain-containing protein 2
1.076	1.070	1.016				0.979	0.832	1.012	1.031	0.968	0.989	O14508	SOCS2	Suppressor of cytokine signaling 2
1.015	1.106	1.006							0.913	0.988	0.967	O14544	SOCS6	Suppressor of cytokine signaling 6
			0.916	0.951	0.900							Q6UWP8	SBSN	Suprabasin
0.947	0.978	1.003	1.015	0.972	1.024	1.055	0.999	0.998	1.068	0.985	1.065	Q15526	SURF1	Surfeit locus protein 1
1.057	0.901	0.903	0.945	1.013	1.174	1.152	1.070	1.046	1.064	1.020	1.235	Q15527	SURF2	Surfeit locus protein 2
0.900	1.029	0.939	0.971	0.925	1.067	1.054	1.177	1.027	1.269	1.064	1.149	O15260	SURF4	Surfeit locus protein 4
1.013	1.026	1.043	0.987	1.000	1.033	1.018	1.043	1.021	1.059	1.030	1.115	O75683	SURF6	Surfeit locus protein 6
1.026	0.980	1.029	1.053	0.928	1.007	1.047	1.033	1.052	1.053	1.057	1.060	Q8IWZ8	SUGP1	SURP and G-patch domain-containing protein 1
0.959	0.981	0.977	0.960	0.946	0.998	1.019	1.016	1.050	0.995	0.954	1.056	M0R2Z9	SUGP2	SURP and G-patch domain-containing protein 2
0.987	0.974	0.981	0.989	0.922	0.953	0.930	1.002	0.987	0.965	1.002	0.942	E7EQZ4	SMN1	Survival motor neuron protein
1.032	0.996	0.996	0.983	1.001	1.009	0.976	1.023	0.973	0.956	1.023	1.032	O75940	SMNDC1	Survival of motor neuron-related-splicing factor 30
1.020	0.884	1.253	1.202	1.035	0.960	1.148	1.050	1.076	1.302	1.070	1.129	Q9UGT4	SUSD2	Sushi domain-containing protein 2
0.977	0.968	1.012	0.990	0.875	1.011	1.007	1.122	1.022	1.129	0.999	0.946	P78539	SRPX	Sushi repeat-containing protein SRPX
0.999	0.981	0.953	1.025	0.957	1.062	1.041	0.970	1.041	1.203	1.050	1.175	Q7Z422	SZRD1	SUZ domain-containing protein 1
0.988	0.972	1.020	1.001	0.955	1.015	1.033	0.985	1.017	1.034	1.087	1.049	Q92922	SMARCC1	SWI/SNF complex subunit SMARCC1

Abundance Ratio														
1 d M1/C1	1 d M2/C2	1 d M3/C3	4 d M1/C1	4 d M2/C2	4 d M3/C3	7 d M1/C1	7 d M2/C2	7 d M3/C3	10 d M1/C1	10 d M2/C2	10 d M3/C3	Accession ID	Gene name	Protein Description
0.964	1.003	1.023	1.145	0.975	0.966	0.964	1.031	0.995	0.922	1.011	0.933	Q8TAQ2	SMARCC2	SWI/SNF complex subunit SMARCC2
0.953	0.995	0.982	0.977	0.966	1.009	0.956	0.909	0.984	1.009	0.979	1.054	F8VXC8	SMARCC2	SWI/SNF complex subunit SMARCC2
0.992	0.977	1.009	0.975	0.949	0.967	1.015	1.052	0.972	0.955	0.991	1.022	G5E975	SMARCB1	SWI/SNF related, matrix associated, actin dependent regulator of chromatin, subfamily b, member 1, isoform CRA_c
1.000	0.999	1.005	0.990	1.026	0.993	1.020	1.008	1.012	0.976	0.979	1.018	O60264	SMARCA5	SWI/SNF-related matrix-associated actin-dependent regulator of chromatin subfamily A member 5
0.990	0.957	1.068	0.983	0.905	1.075	1.045	1.058	1.031	0.976	0.969	0.974	Q9NZC9	SMARCAL1	SWI/SNF-related matrix-associated actin-dependent regulator of chromatin subfamily A-like protein 1
1.014	1.064	1.006	0.982	0.951	0.981	1.017	1.079	1.005	1.032	1.041	1.016	Q96GM5	SMARCD1	SWI/SNF-related matrix-associated actin-dependent regulator of chromatin subfamily D member 1
1.012	1.012	1.015	0.973	0.940	0.987	0.984	1.003	1.013	1.027	1.004	1.057	Q92925	SMARCD2	SWI/SNF-related matrix-associated actin-dependent regulator of chromatin subfamily D member 2
1.136	1.072	1.012	1.014	0.972	0.933	0.909	1.109	0.877	0.985	1.040	0.962	Q6STE5	SMARCD3	SWI/SNF-related matrix-associated actin-dependent regulator of chromatin subfamily D member 3
0.960	0.967	0.982	0.986	0.974	1.034	0.961	0.911	0.984	0.978	1.000	1.027	Q969G3	SMARCE1	SWI/SNF-related matrix-associated actin-dependent regulator of chromatin subfamily E member 1
1.035	0.988	1.085	0.990	0.969	0.994	1.011	0.989	0.976	0.962	1.019	1.023	Q9P0W2	HMG20B	SWI/SNF-related matrix-associated actin-dependent regulator of chromatin subfamily E member 1-related
1.012	1.001	1.043	1.004	0.952	1.013	1.014	0.931	1.005	1.049	0.987	1.044	Q9UH65	SWAP70	Switch-associated protein 70
1.017	1.001	1.026	1.004	0.973	1.029	0.992	1.053	1.035	0.992	0.986	1.034	Q92797	SYMPK	Symplekin
0.988	0.988	1.024	0.980	0.978	0.990	1.002	1.061	0.971	1.027	1.006	1.056	Q96A49	SYAP1	Synapse-associated protein 1
1.001	1.008	0.998	0.999	1.074	0.994	1.038	1.056	1.036	1.027	1.030	0.991	Q99536	VAT1	Synaptic vesicle membrane protein VAT-1 homolog
0.984	1.006	1.021	0.993	0.917	1.013	1.007	1.034	1.088	1.004	1.012	1.072	O15498	YKT6	Synaptobrevin homolog YKT6
1.026	1.145	0.995										O43759	SYNGR1	Synaptogyrin-1
0.976	0.973	1.064	0.834	0.929	1.021	0.951	0.900	1.049				O43761	SYNGR3	Synaptogyrin-3
0.993	1.004	1.046	0.999	0.969	1.055	0.946	1.047	1.026	1.059	0.991	1.078	J3KQV8	SYNJ1	Synaptojanin-1
0.992	1.016	1.047	0.971	0.946	0.968	0.966	0.940	0.999	0.943	0.915	1.005	O15056	SYNJ2	Synaptojanin-2
0.924	1.004	1.017	1.244	1.557	0.947							Q92791	P3H4	Synaptonemal complex protein SC65
1.050	1.032	1.025	1.077	1.128	1.041				1.271	0.970	1.197	P08247	SYP	Synaptophysin

Abundance Ratio												Accession ID	Gene name	Protein Description
1 d M1/C1	1 d M2/C2	1 d M3/C3	4 d M1/C1	4 d M2/C2	4 d M3/C3	7 d M1/C1	7 d M2/C2	7 d M3/C3	10 d M1/C1	10 d M2/C2	10 d M3/C3			
0.982	0.960	0.917	0.967	0.927	1.026	1.164	1.173	1.032	1.117	1.029	1.009	Q16563	SYPL1	Synaptophysin-like protein 1
0.953	0.966	1.010	1.025	0.933	1.015	1.027	0.992	0.977	1.051	1.032	1.096	O00161	SNAP23	Synaptosomal-associated protein 23
1.002	0.984	1.047	0.987	1.003	1.007	0.975	0.922	1.003	1.069	0.998	1.024	O95721	SNAP29	Synaptosomal-associated protein 29
1.114	0.824	1.088							0.969	1.548	0.636	Q5SQN1	SNAP47	Synaptosomal-associated protein 47
1.054	0.993	0.890				1.013	1.164	1.158				Q96C24	SYTL4	Synaptotagmin-like protein 4
1.028	1.043	1.056	1.132	1.160	1.107	1.095	1.169	1.126	1.090	1.074	1.136	G5EA09	SDCBP	Syndecan binding protein (Syntenin), isoform CRA_a
0.942	0.941	1.008	0.997	1.053	1.020	0.962	0.974	0.940	1.017	1.036	1.095	P18827	SDC1	Syndecan-1
0.930	1.003	0.978	0.989	0.914	1.045	1.086	0.979	0.958	1.198	1.006	1.128	P31431	SDC4	Syndecan-4
1.038	0.981	1.048	1.023	0.970	1.030	1.001	0.974	1.030	0.996	1.016	1.023	Q96JG6	VPS50	Syndetin
0.982	1.033	1.016	0.984	0.989	0.995	0.987	1.013	0.992	1.011	0.984	1.021	Q9NPQ8	RIC8A	Synembryn-A
						1.083	0.900	1.072	0.927	1.053	1.054	B7WPL0	RIC8B	Synembryn-B
1.006	1.030	1.069	0.988	0.950	0.978	1.010	1.014	0.997	1.015	1.014	1.062	Q9UMZ2	SYNRG	Synergin gamma
1.090	0.974	0.990	0.949	0.989	0.984	0.999	0.892	0.901	1.099	1.046	1.084	A0A087WUM0	SYNJ2BP-COX16	SYNJ2BP-COX16 readthrough (Fragment)
1.047	0.971	0.988	0.978	0.902	1.007	1.024	1.021	0.979	0.987	0.981	1.044	A0A087WYV9	SYNJ2BP-COX16	SYNJ2BP-COX16 readthrough
0.899	1.161	0.900	1.303	0.951	1.267	0.737	0.725	1.140				A0A0C4DG86	SYBU	Syntabulin
0.923	1.011	0.969	0.992	0.865	1.017							O60499	STX10	Syntaxin-10
1.060	0.934	1.044	0.994	0.916	1.071	0.984	1.031	1.002	1.163	1.162	1.232	O75558	STX11	Syntaxin-11
0.927	1.003	1.029	0.983	0.935	1.055	0.988	1.024	1.041	1.016	1.029	1.057	Q86Y82	STX12	Syntaxin-12
1.063	1.011	1.016	0.994	0.928	0.980	0.957	0.998	1.016	0.981	0.964	1.033	P56962	STX17	Syntaxin-17
0.975	0.999	1.027	0.991	1.038	1.006	1.052	1.061	1.082	1.061	1.047	1.094	Q9P2W9	STX18	Syntaxin-18
0.990	0.989	1.044	0.970	0.946	1.045	1.024	0.968	0.999	1.074	1.004	1.129	P32856	STX2	Syntaxin-2
0.993	0.945	1.090	1.083	0.900	1.063	1.067	1.046	1.094	1.155	0.995	1.062	Q13277	STX3	Syntaxin-3
0.968	0.950	1.037	0.999	0.956	1.051	1.080	0.963	1.042	1.088	1.039	1.032	Q12846	STX4	Syntaxin-4
0.955	0.974	1.048	0.967	0.880	1.024	0.957	1.011	1.018	0.994	1.004	0.999	Q13190	STX5	Syntaxin-5
1.032	0.972	1.000	1.057	0.948	0.990	1.054	1.039	1.030	1.040	0.986	1.019	O43752	STX6	Syntaxin-6
0.976	1.006	1.044	0.982	0.962	1.024	0.994	1.025	1.022	1.038	1.020	1.019	O15400	STX7	Syntaxin-7
0.971	0.983	1.045	1.007	0.917	1.015	0.979	0.996	0.974	0.961	0.984	0.987	Q9UNK0	STX8	Syntaxin-8
0.997	1.028	1.015	1.007	0.988	1.016	1.004	1.048	0.993	1.005	0.986	1.011	Q15833	STXBP2	Syntaxin-binding protein 2
0.973	1.002	1.007	0.992	1.001	1.009	1.007	1.027	1.011	0.960	0.999	1.022	O00186	STXBP3	Syntaxin-binding protein 3
1.090	0.925	1.149							1.244	1.142	1.265	Q6ZWJ1	STXBP4	Syntaxin-binding protein 4
1.008	1.052	0.978	0.933	0.916	0.955	0.992	1.008	1.024	0.911	1.015	0.923	Q5T5C0	STXBP5	Syntaxin-binding protein 5
1.065	0.938	0.810				0.616	1.407	0.770	0.855	1.156	0.999	O00560	SDCBP	Syntenin-1
1.141	0.861	0.969										Q9H190	SDCBP2	Syntenin-2

Abundance Ratio														
1 d M1/C1	1 d M2/C2	1 d M3/C3	4 d M1/C1	4 d M2/C2	4 d M3/C3	7 d M1/C1	7 d M2/C2	7 d M3/C3	10 d M1/C1	10 d M2/C2	10 d M3/C3	Accession ID	Gene name	Protein Description
1.012	1.028	1.028	0.895	1.100	0.918	0.978	1.012	1.012	0.992	0.965	1.113	O75529	TAF5L	TAF5-like RNA polymerase II p300/CBP-associated factor-associated factor 65 kDa subunit 5L
1.032	1.069	1.125	1.022	0.992	0.992	0.976	0.933	1.000	1.031	1.027	1.123	Q9Y6J9	TAF6L	TAF6-like RNA polymerase II p300/CBP-associated factor-associated factor 65 kDa subunit 6L
0.779	0.973	0.996	0.831	1.085	0.925	1.023	0.665	0.925	1.069	1.041	1.078	O00258	WRB	Tail-anchored protein insertion receptor WRB
0.968	0.999	0.996	0.978	1.006	0.992	0.987	0.975	0.997	0.976	0.981	0.999	Q9Y490	TLN1	Talin-1
0.939	0.937	1.025	1.006	1.061	1.012	0.886	0.736	0.914	0.939	0.994	1.004	Q9Y4G6	TLN2	Talin-2
1.053	1.365	0.904	1.065	1.016	1.181	0.894	1.438	1.330	0.971	1.238	1.073	O95271	TNKS	Tankyrase-1
0.979	1.020	1.019	0.983	1.009	0.992	1.019	1.043	0.997	0.959	0.991	0.924	A0A0A0MSV9	TAPBP	Tapasin
0.991	0.968	1.062	0.992	0.984	1.036	0.998	0.985	0.992	1.050	1.031	1.036	Q4KMQ1	TPRN	Taperin
0.983	1.002	0.962	0.968	0.996	0.994	0.980	1.028	0.980	0.983	1.039	0.984	Q13148	TARDBP	TAR DNA-binding protein 43
1.012	1.012	1.064	0.985	0.972	1.021	0.952	0.942	1.027	0.958	0.973	1.102	Q96GM8	TOE1	Target of EGR1 protein 1
1.005	0.978	1.074	0.986	0.977	1.072	1.006	1.009	1.038	1.032	0.988	1.028	O60784	TOM1	Target of Myb protein 1
1.030	0.999	1.015	1.017	0.955	1.056	0.997	0.918	0.953	1.030	1.024	1.023	Q9BPZ7	MAPKAP1	Target of rapamycin complex 2 subunit MAPKAP1
1.012	0.959	1.068	0.946	1.101	1.006	0.979	0.877	0.977	1.010	1.017	1.035	Q9BVC4	MLST8	Target of rapamycin complex subunit LST8
0.932	1.005	0.993	1.021	0.918	0.926	1.134	1.130	1.024	0.905	0.871	1.045	Q9ULW0	TPX2	Targeting protein for Xklp2
0.817	1.008	1.111	0.963	0.877	0.904	0.897	1.265	1.181	0.694	0.860	1.139	F8W130	TROAP	Tastin
						0.841	1.163	0.827	0.952	1.117	0.981	Q15573	TAF1A	TATA box-binding protein-associated factor RNA polymerase I subunit A
			0.940	0.888	1.247	1.026	1.162	0.983	0.963	1.034	1.250	Q15572	TAF1C	TATA box-binding protein-associated factor RNA polymerase I subunit C
1.024	0.996	0.986	1.006	0.984	1.000	0.874	1.108	1.116	0.985	1.112	1.308	P62380	TBPL1	TATA box-binding protein-like protein 1
1.026	1.040	1.021	0.992	0.989	1.028	1.043	1.113	1.014	1.010	0.999	1.011	A0A0A0MTH9	BTAF1	TATA-binding protein-associated factor 172
0.983	0.982	1.029	0.940	1.024	0.984	0.900	0.821	0.968	0.982	1.011	1.023	Q92804	TAF15	TATA-binding protein-associated factor 2N
0.944	0.968	1.046	1.023	0.969	1.007	0.990	0.983	0.936	1.008	0.995	0.884	P20226	TBP	TATA-box-binding protein
1.034	1.111	1.037	1.107	1.050	0.995	1.003	1.041	1.057	0.935	0.920	1.041	B8ZZD4	TAX1BP1	Tax1-binding protein 1
1.020	1.008	0.979	0.961	0.905	0.946	0.972	1.050	0.981	1.018	0.990	1.017	O14907	TAX1BP3	Tax1-binding protein 3
1.129	1.066	1.197	0.972	1.012	0.987	1.027	0.989	0.958	0.890	1.023	1.166	Q8TEA7	TBCK	TBC domain-containing protein kinase-like protein
0.991	0.999	1.038	1.002	0.961	0.976	0.933	0.972	1.014	0.973	0.980	1.086	Q86TI0	TBC1D1	TBC1 domain family member 1
1.020	0.960	1.004	0.979	0.940	1.025	0.986	1.069	1.035	1.019	1.038	1.062	Q9BXI6	TBC1D10A	TBC1 domain family member 10A
0.965	0.973	1.019	1.010	0.996	1.014	1.001	0.952	0.977	0.999	1.017	1.020	Q4KMP7	TBC1D10B	TBC1 domain family member 10B
1.019	1.003	1.004	1.001	0.969	1.030	0.989	1.060	1.019	0.987	0.988	0.969	Q9NVG8	TBC1D13	TBC1 domain family member 13
0.985	0.882	1.048	0.912	0.951	1.109	1.014	1.245	1.090	1.096	1.198	1.177	Q9P2M4	TBC1D14	TBC1 domain family member 14

Abundance Ratio														
1 d M1/C1	1 d M2/C2	1 d M3/C3	4 d M1/C1	4 d M2/C2	4 d M3/C3	7 d M1/C1	7 d M2/C2	7 d M3/C3	10 d M1/C1	10 d M2/C2	10 d M3/C3	Accession ID	Gene name	Protein Description
1.129	0.923	1.075	1.068	0.984	1.157				1.338	1.109	1.430	Q8TBP0	TBC1D16	TBC1 domain family member 16
1.001	1.062	1.048	0.982	0.952	1.029	0.992	0.983	0.978	1.053	1.072	1.027	Q9HA65	TBC1D17	TBC1 domain family member 17
1.068	0.971	1.028	1.092	1.084	1.112	1.009	1.063	1.034	1.002	0.972	1.089	Q8WUA7	TBC1D22A	TBC1 domain family member 22A
			1.116	0.935	0.922							Q9NU19	TBC1D22B	TBC1 domain family member 22B
0.988	1.003	1.006	0.998	0.956	1.024	0.985	1.059	1.037	1.012	0.988	1.064	Q9NUY8	TBC1D23	TBC1 domain family member 23
0.972	0.952	0.923	1.007	0.897	1.011	1.038	1.057	1.013	1.008	0.982	1.046	Q9ULP9	TBC1D24	TBC1 domain family member 24
0.957	0.888	1.141	1.016	1.014	1.016	1.007	1.078	1.025	0.989	0.988	0.990	Q3MII6	TBC1D25	TBC1 domain family member 25
0.971	1.011	1.023	1.015	0.943	1.094	0.977	1.040	1.055	0.970	0.986	1.018	Q9BYX2	TBC1D2	TBC1 domain family member 2A
0.932	1.007	0.965	0.978	0.961	1.046	1.127	1.058	0.976	1.000	0.941	1.339	Q9UPU7	TBC1D2B	TBC1 domain family member 2B
1.067	1.013	1.049	1.018	0.957	0.998	0.987	1.010	1.012	0.988	0.993	1.080	O60343	TBC1D4	TBC1 domain family member 4
1.064	1.101	0.981	0.956	1.032	1.042	0.958	1.015	1.095	0.955	0.978	1.034	Q9P0N9	TBC1D7	TBC1 domain family member 7
0.998	1.014	1.069	0.980	0.845	1.139							J3KQ40	TBC1D8	TBC1 domain family member 8
0.999	0.985	0.979	0.974	0.982	0.982	0.938	1.067	0.981	0.937	0.967	0.940	Q0IIM8	TBC1D8B	TBC1 domain family member 8B
0.977	0.960	1.064	0.972	0.945	1.012	1.034	1.027	1.028	1.069	0.986	1.022	Q66K14	TBC1D9B	TBC1 domain family member 9B
1.022	0.954	0.989	0.982	0.931	1.085	1.033	1.200	1.145	1.056	0.946	1.007	Q8TB96	ITFG1	T-cell immunomodulatory protein
1.000	1.004	1.011	1.003	1.091	0.989	1.001	1.012	1.034	0.953	1.003	0.991	P17987	TCP1	T-complex protein 1 subunit alpha
0.962	0.994	0.968	0.999	0.988	1.009	0.971	1.028	1.021	0.957	1.001	0.973	P78371	CCT2	T-complex protein 1 subunit beta
1.028	0.988	1.009	1.009	1.168	0.988	1.020	0.949	1.049	0.959	1.001	0.973	P50991	CCT4	T-complex protein 1 subunit delta
0.964	1.003	0.968	1.017	1.042	1.016	0.993	1.009	1.018	0.953	0.987	0.974	P48643	CCT5	T-complex protein 1 subunit epsilon
1.028	0.995	1.008	1.024	1.061	0.984	1.012	1.029	1.016	0.965	0.983	0.981	Q99832	CCT7	T-complex protein 1 subunit eta
0.996	0.986	0.988	1.004	1.106	0.976	1.005	0.997	1.027	0.948	1.003	0.994	P49368	CCT3	T-complex protein 1 subunit gamma
0.960	0.999	0.984	0.997	0.990	1.003	1.002	1.029	0.988	0.993	1.020	0.990	P50990	CCT8	T-complex protein 1 subunit theta
0.994	1.001	0.998	1.012	0.991	1.027	0.988	1.045	1.021	0.979	1.010	0.999	P40227	CCT6A	T-complex protein 1 subunit zeta
0.954	0.890	1.042	0.958	0.896	0.954	0.942	0.984	1.006	0.800	1.012	0.992	Q92526	CCT6B	T-complex protein 1 subunit zeta-2
0.994	0.982	1.038	0.975	0.922	1.007	1.014	1.025	1.031	1.050	1.032	1.052	Q9NUJ3	TCP11L1	T-complex protein 11-like protein 1
0.985	0.889	1.008	0.880	0.852	0.867							Q8N4U5	TCP11L2	T-complex protein 11-like protein 2
1.115	1.139	1.176	0.906	0.942	0.928	0.850	1.176	1.045	0.987	0.968	1.039	O15040	TECPR2	Tectonin beta-propeller repeat-containing protein 2
1.023	0.959	1.035	0.961	0.935	1.000	1.006	1.080	1.063	0.908	0.980	1.010	O43156	TTI1	TELO2-interacting protein 1 homolog
1.005	0.968	1.034	0.974	0.924	0.980	0.983	1.159	0.948	0.981	0.996	1.058	Q6NXR4	TTI2	TELO2-interacting protein 2
0.962	0.973	1.057	0.912	0.980	0.977	0.952	0.857	1.001	0.959	0.958	1.077	Q9BUR4	WRAP53	Telomerase Cajal body protein 1
			0.949	1.012	1.010	1.006	1.046	0.981	1.066	0.963	1.022	Q99973	TEP1	Telomerase protein component 1
1.033	0.994	1.058	1.008	0.957	0.993	1.025	1.040	1.051	1.006	0.975	1.034	Q86US8	SMG6	Telomerase-binding protein EST1A
1.029	0.972	0.997	0.957	0.931	0.999	0.980	0.988	1.061	0.969	0.963	1.010	Q9Y4R8	TELO2	Telomere length regulation protein TEL2 homolog

Abundance Ratio														
1 d M1/C1	1 d M2/C2	1 d M3/C3	4 d M1/C1	4 d M2/C2	4 d M3/C3	7 d M1/C1	7 d M2/C2	7 d M3/C3	10 d M1/C1	10 d M2/C2	10 d M3/C3	Accession ID	Gene name	Protein Description
1.018	1.019	1.080	0.988	0.957	1.018	0.995	1.101	1.061	1.003	0.954	1.048	Q5UIP0	RIF1	Telomere-associated protein RIF1
1.013	1.034	1.005	1.005	0.996	1.009	1.023	1.049	1.024	1.100	1.017	1.027	Q15554	TERF2	Telomeric repeat-binding factor 2
1.042	0.987	1.009	0.981	0.952	1.055	0.981	0.958	0.980	1.016	1.041	1.030	Q9NYB0	TERF2IP	Telomeric repeat-binding factor 2-interacting protein 1
1.050	0.981	1.052	1.014	0.968	1.010	1.065	0.927	0.978	1.133	0.939	1.055	P24821	TNC	Tenascin
0.979	1.003	1.020	1.032	1.020	1.012	1.044	0.984	1.022	1.060	0.957	1.122	Q68CZ2	TNS3	Tensin-3
1.050	0.990	1.028	0.932	0.892	1.076	1.044	1.007	1.062	1.081	1.064	0.899	Q9BSI4	TINF2	TERF1-interacting nuclear factor 2
1.031	1.018	1.030	1.004	0.911	1.013	0.932	1.053	1.026	0.966	0.908	1.032	A0A0C4DFM7	ZCCHC11	Terminal uridylyltransferase 4
1.061	1.009	1.045	1.039	0.958	1.029	1.031	1.123	1.053	1.022	0.982	1.055	Q5VYS8	ZCCHC6	Terminal uridylyltransferase 7
0.988	0.984	0.986	1.000	1.023	0.978	1.024	0.939	0.971	1.015	0.987	0.997	Q9UGI8	TES	Testin
			0.776	1.006	1.128				1.470	1.073	1.687	Q8N9V7	TOPAZ1	Testis- and ovary-specific PAZ domain-containing protein 1
1.006	0.990	1.029	0.990	0.912	1.010	1.005	1.038	0.992	0.980	1.014	0.978	Q9NXF1	TEX10	Testis-expressed protein 10
0.935	0.999	1.068	0.992	0.981	1.069	0.996	1.044	0.992	1.157	0.962	1.060	Q9Y6I9	TEX264	Testis-expressed protein 264
1.041	1.060	1.063	1.130	0.890	0.944							Q5JUR7	TEX30	Testis-expressed protein 30
0.976	1.168	0.928	1.066	1.077	1.007	0.959	0.999	0.986				Q9H0U9	TSPYL1	Testis-specific Y-encoded-like protein 1
1.011	0.861	1.116				0.794	1.219	1.000				Q9H2G4	TSPYL2	Testis-specific Y-encoded-like protein 2
0.978	0.986	0.984	0.949	0.870	1.020	1.010	1.009	1.007	0.924	1.026	0.954	G8JLH6	CD9	Tetraspanin (Fragment)
1.034	0.962	1.085	1.107	0.854	0.950	1.078	1.051	1.046	1.078	1.061	1.060	A6NMH8	CD81	Tetraspanin
1.077	1.206	1.225							0.903	0.886	0.927	J3KQ42	TSPAN4	Tetraspanin
									1.087	0.961	1.034	O95857	TSPAN13	Tetraspanin-13
1.054	1.131	1.219	1.061	1.119	1.355	1.079	1.057	1.123	1.078	1.044	1.082	Q8NG11	TSPAN14	Tetraspanin-14
0.874	0.881	1.215	1.207	1.016	1.517	1.324	1.092	1.159	1.346	1.320	1.359	O60637	TSPAN3	Tetraspanin-3
									1.115	1.205	1.347	Q12999	TSPAN31	Tetraspanin-31
			1.092	0.913	1.037	1.465	0.866	0.918	1.367	1.168	1.146	O43657	TSPAN6	Tetraspanin-6
0.965	1.059	1.160	0.949	0.957	1.076	1.159	0.889	1.026	0.921	0.971	1.029	A0A0C4DGX9	TTC8	Tetratricopeptide repeat domain 8 isoform 1
1.076	1.028	0.972	1.015	1.016	0.963	0.976	1.019	0.950	0.954	0.984	1.010	Q99614	TTC1	Tetratricopeptide repeat protein 1
0.949	0.988	1.165	1.002	0.936	0.930	0.998	1.063	1.046	0.949	0.821	0.913	Q8NBP0	TTC13	Tetratricopeptide repeat protein 13
0.932	0.912	0.891	0.884	0.842	1.161	0.821	1.171	1.290				Q96N46	TTC14	Tetratricopeptide repeat protein 14
1.066	0.938	1.115	1.083	0.945	1.053	1.000	0.949	0.977	1.074	0.980	0.876	Q96AE7	TTC17	Tetratricopeptide repeat protein 17
1.003	0.998	0.997	0.991	0.976	0.995	1.045	1.004	0.998	1.072	0.996	1.016	Q6DKK2	TTC19	Tetratricopeptide repeat protein 19, mitochondrial
1.100	0.905	1.043	0.982	0.945	1.027	0.964	0.990	1.113	1.045	0.970	1.128	Q7Z4L5	TTC21B	Tetratricopeptide repeat protein 21B
1.000	1.020	1.070	1.023	0.908	1.020	0.995	1.082	1.010	0.927	0.955	1.092	Q6P3X3	TTC27	Tetratricopeptide repeat protein 27
1.037	1.051	1.013	1.028	0.973	1.005	1.055	1.101	0.998	1.016	1.001	1.053	Q96AY4	TTC28	Tetratricopeptide repeat protein 28

Abundance Ratio 1 d M1/C1	1 d M2/C2	1 d M3/C3	4 d M1/C1	4 d M2/C2	4 d M3/C3	7 d M1/C1	7 d M2/C2	7 d M3/C3	10 d M1/C1	10 d M2/C2	10 d M3/C3	Accession ID	Gene name	Protein Description
0.813	0.817	0.888	1.102	1.029	1.062	1.041	0.961	1.513	1.076	1.112	0.894	Q86WT1	TTC30A	Tetratricopeptide repeat protein 30A
0.981	0.950	0.986	0.963	0.930	1.010	0.942	1.007	1.002	0.978	0.967	1.021	Q6PID6	TTC33	Tetratricopeptide repeat protein 33
1.020	0.986	0.994	1.009	0.962	1.008	1.018	1.023	1.006	1.038	0.991	1.021	Q6PGP7	TTC37	Tetratricopeptide repeat protein 37
1.042	0.983	1.034	1.001	0.935	1.006	0.958	1.095	1.023	1.093	0.952	1.099	Q5R3I4	TTC38	Tetratricopeptide repeat protein 38
			1.054	0.997	1.030	1.314	1.085	1.021	1.011	0.997	1.066	Q5SRH9	TTC39A	Tetratricopeptide repeat protein 39A
1.087	0.984	1.063	0.926	0.915	1.146	0.926	0.997	1.071	0.855	1.000	0.952	Q5VTQ0	TTC39B	Tetratricopeptide repeat protein 39B
1.000	0.983	0.983	1.023	0.974	1.006	1.085	1.082	0.944	0.933	0.909	1.122	Q8N584	TTC39C	Tetratricopeptide repeat protein 39C
1.093	1.074	1.050	0.984	1.051	0.971	0.985	1.096	1.016	0.982	1.008	0.979	O95801	TTC4	Tetratricopeptide repeat protein 4
0.954	0.982	0.990	1.054	1.112	1.022	1.010	1.056	1.098	0.995	1.150	1.118	Q8N0Z6	TTC5	Tetratricopeptide repeat protein 5
1.079	1.128	1.016	1.001	1.004	1.079	1.030	1.080	1.054	0.990	1.015	1.038	Q9ULT0	TTC7A	Tetratricopeptide repeat protein 7A
1.068	1.046	1.004	0.948	0.957	0.967	0.970	1.047	0.961	0.979	0.986	0.998	Q8N5M4	TTC9C	Tetratricopeptide repeat protein 9C
0.993	1.002	1.025	1.002	0.950	1.053	0.988	1.034	1.029	0.978	0.973	1.049	P19447	ERCC3	TFIIH basal transcription factor complex helicase XPB subunit
1.042	1.013	1.040	1.019	1.008	1.040	1.027	1.059	1.004	0.984	0.981	1.053	P18074	ERCC2	TFIIH basal transcription factor complex helicase XPD subunit
0.995	0.970	1.012	0.964	0.978	1.027	1.043	1.055	1.034	0.974	0.989	1.029	Q15750	TAB1	TGF-beta-activated kinase 1 and MAP3K7-binding protein 1
0.918	0.974	0.977	1.027	0.869	1.020	0.937	0.986	1.076	0.928	0.798	1.118	Q9NYJ8	TAB2	TGF-beta-activated kinase 1 and MAP3K7-binding protein 2
1.025	0.989	1.068	0.964	0.989	1.108	0.885	0.960	1.147	0.792	1.069	1.250	Q8N5C8	TAB3	TGF-beta-activated kinase 1 and MAP3K7-binding protein 3
1.153	1.020	0.986	0.955	1.022	0.990	1.014	1.155	0.894	1.008	0.975	0.934	Q96EK4	THAP11	THAP domain-containing protein 11
									1.075	0.940	0.855	Q9H3S4	TPK1	Thiamin pyrophosphokinase 1
									1.047	1.113	0.973	O60779	SLC19A2	Thiamine transporter 1
1.135	0.980	1.111	1.113	0.911	0.821	0.902	1.102	1.066	0.840	0.933	0.981	Q9BU02	THTPA	Thiamine-triphosphatase
1.026	0.996	1.009	0.987	0.954	1.011	0.989	1.054	1.022	1.006	0.976	1.003	P52888	THOP1	Thimet oligopeptidase
1.037	1.017	0.979	0.980	1.031	1.007	1.036	1.025	1.066	0.990	0.969	1.026	P51580	TPMT	Thiopurine S-methyltransferase
0.991	0.998	0.986	1.006	1.110	0.997	1.005	0.947	1.019	0.981	1.018	0.968	O95881	TXNDC12	Thioredoxin domain-containing protein 12
1.141	0.993	1.122										Q9P2K2	TXNDC16	Thioredoxin domain-containing protein 16
1.125	1.030	1.006	1.042	1.063	0.979	1.012	0.995	0.986	0.955	0.988	0.931	Q9BRA2	TXNDC17	Thioredoxin domain-containing protein 17
1.016	0.989	0.992	1.006	1.016	1.018	0.993	1.004	1.001	0.978	0.994	0.974	Q8NBS9	TXNDC5	Thioredoxin domain-containing protein 5
0.987	0.993	1.008	1.012	0.995	1.007	1.033	1.054	1.009	0.947	0.995	0.975	O14530	TXNDC9	Thioredoxin domain-containing protein 9
1.030	1.004	0.935	1.038	1.235	1.002	1.076	0.964	1.072	1.022	1.084	0.971	P10599	TXN	Thioredoxin
1.012	0.995	0.980	1.006	1.009	1.026	0.989	1.046	1.025	0.982	1.021	0.973	Q16881	TXNRD1	Thioredoxin reductase 1, cytoplasmic
0.988	0.969	1.020	0.979	1.010	1.002	1.022	0.952	1.006	1.086	0.971	1.056	Q9NNW7	TXNRD2	Thioredoxin reductase 2, mitochondrial
			0.964	0.942	1.056	0.981	1.108	1.220	0.901	0.981	1.034	H0YBQ0	TXNRD3	Thioredoxin reductase 3

Abundance Ratio														
1 d M1/C1	1 d M2/C2	1 d M3/C3	4 d M1/C1	4 d M2/C2	4 d M3/C3	7 d M1/C1	7 d M2/C2	7 d M3/C3	10 d M1/C1	10 d M2/C2	10 d M3/C3	Accession ID	Gene name	Protein Description
1.082	0.995	0.991	0.966	0.973	0.997	0.969	0.991	1.026	0.986	0.993	0.922	Q99757	TXN2	Thioredoxin, mitochondrial
1.011	0.996	0.989	0.981	0.959	1.005	1.000	1.031	0.995	0.986	0.992	0.980	P30048	PRDX3	Thioredoxin-dependent peroxide reductase, mitochondrial
0.981	1.061	1.051										Q9H3M7	TXNIP	Thioredoxin-interacting protein
0.978	1.008	0.965	0.982	1.075	0.968	1.013	0.992	0.991	1.014	1.010	1.037	O43396	TXNL1	Thioredoxin-like protein 1
0.945	1.024	0.981	1.054	1.103	1.055	1.019	1.081	1.013	0.997	1.038	1.013	P83876	TXNL4A	Thioredoxin-like protein 4A
1.002	0.985	0.941	0.978	0.961	1.017	1.108	1.008	1.017				Q9NX01	TXNL4B	Thioredoxin-like protein 4B
0.966	1.033	1.070	1.045	0.962	0.967	0.939	1.125	0.983	0.691	0.704	1.039	Q7RTV5	AAED1	Thioredoxin-like protein AAED1
0.974	1.012	0.999	0.979	0.962	1.005	1.035	1.041	0.999	1.027	1.024	0.973	Q9H3N1	TMX1	Thioredoxin-related transmembrane protein 1
0.977	1.005	1.016	1.085	0.967	1.061	1.059	1.060	1.047	1.080	1.049	1.079	Q9Y320	TMX2	Thioredoxin-related transmembrane protein 2
0.981	0.991	1.023	0.972	0.939	0.973	1.005	1.062	1.009	1.070	0.992	1.048	Q9H1E5	TMX4	Thioredoxin-related transmembrane protein 4
1.019	1.010	1.022	1.002	1.023	1.073	1.022	1.010	1.012	1.118	0.998	1.057	Q16762	TST	Thiosulfate sulfurtransferase
1.009	0.986	1.019	0.998	1.042	0.976	1.028	1.005	1.017	1.030	1.029	1.016	Q96FV9	THOC1	THO complex subunit 1
0.960	1.001	1.006	0.972	0.964	1.021	0.971	0.961	0.989	1.000	1.001	1.015	Q8NI27	THOC2	THO complex subunit 2
0.946	0.949	0.973	0.972	0.948	0.996	0.985	1.038	0.979	1.004	1.018	1.041	Q96J01	THOC3	THO complex subunit 3
0.977	1.003	0.987	0.977	0.946	0.986	0.956	1.013	0.978	0.983	1.050	0.965	E9PB61	ALYREF	THO complex subunit 4
0.992	1.007	0.950	0.957	0.932	0.987	0.924	0.901	0.960	1.023	0.994	1.016	Q13769	THOC5	THO complex subunit 5 homolog
1.018	1.028	1.001	0.971	0.982	0.996	0.998	1.045	0.998	0.968	1.002	0.976	Q86W42	THOC6	THO complex subunit 6 homolog
1.094	0.975	1.048	1.037	0.914	1.015	0.978	0.888	0.928	0.987	0.976	0.975	Q6I9Y2	THOC7	THO complex subunit 7 homolog
1.062	1.010	1.019	1.034	0.957	0.991	0.993	1.174	1.019	0.910	0.961	1.028	Q9H6P5	TASP1	Threonine aspartase 1
1.012	1.159	0.999	1.005	0.877	0.955	1.027	0.941	1.066	0.967	0.821	1.001	Q8IYQ7	THNSL1	Threonine synthase-like 1
0.963	1.006	0.978	0.979	1.015	0.944	0.980	0.949	0.990	0.967	0.943	0.952	P26639	TARS	Threonine--tRNA ligase, cytoplasmic
1.008	1.003	1.025	1.029	0.989	1.049	1.014	1.042	1.055	1.036	0.988	1.037	Q9BW92	TARS2	Threonine--tRNA ligase, mitochondrial
1.005	1.006	1.069	1.023	0.970	1.015	1.035	1.042	1.015	1.041	1.014	1.113	Q5VV42	CDKAL1	Threonylcarbamoyladenosine tRNA methylthiotransferase
0.935	0.942	1.046	0.960	0.959	0.995	1.030	1.017	1.051	1.096	1.056	1.050	Q6ZMP0	THSD4	Thrombospondin type-1 domain-containing protein 4
0.940	1.020	1.107	1.070	1.074	1.151	1.038	1.140	1.013	1.071	1.252	1.107	P07996	THBS1	Thrombospondin-1
1.026	1.009	0.997	0.994	1.088	0.988	1.006	1.001	1.011	0.990	1.039	0.976	Q9NXG2	THUMPD1	THUMP domain-containing protein 1
1.109	1.006	1.195	0.908	0.942	1.009	1.147	0.994	0.972	0.787	0.906	0.989	Q9BTF0	THUMPD2	THUMP domain-containing protein 2
1.013	0.995	1.012	0.960	0.987	1.005	1.011	1.088	1.029	0.995	1.023	1.025	Q9BV44	THUMPD3	THUMP domain-containing protein 3
0.915	0.997	0.987	0.928	0.947	0.932	1.029	1.115	0.994	0.884	0.841	1.077	P04183	TK1	Thymidine kinase, cytosolic
1.068	1.022	1.082	1.012	1.061	1.069	1.017	1.066	1.064	1.039	1.011	1.065	P19971	TYMP	Thymidine phosphorylase
1.028	1.020	1.007	0.994	1.021	1.001	0.979	1.016	1.020	0.968	0.963	0.944	P23919	DTYMK	Thymidylate kinase
0.949	1.012	1.008	1.003	0.810	1.153				0.776	0.968	1.093	P04818	TYMS	Thymidylate synthase

Abundance Ratio														
1 d M1/C1	1 d M2/C2	1 d M3/C3	4 d M1/C1	4 d M2/C2	4 d M3/C3	7 d M1/C1	7 d M2/C2	7 d M3/C3	10 d M1/C1	10 d M2/C2	10 d M3/C3	Accession ID	Gene name	Protein Description
1.023	0.964	1.013	0.956	0.997	1.024	0.958	0.901	0.964	1.010	0.959	0.994	Q9P016	THYN1	Thymocyte nuclear protein 1
0.954	0.868	0.941	0.924	0.904	0.919	1.084	0.561	0.883	1.259	0.912	1.196	P63313	TMSB10	Thymosin beta-10
1.002	1.052	0.989	1.010	0.959	1.022	1.029	1.150	1.028	0.962	0.941	1.041	Q6YHU6	THADA	Thyroid adenoma-associated protein
0.981	0.998	0.979	0.989	0.976	0.993	0.989	0.929	0.960	1.051	1.019	1.030	Q9Y2W1	THRAP3	Thyroid hormone receptor-associated protein 3
0.964	0.965	1.041	0.989	0.966	1.019	1.004	0.990	0.988	1.033	0.992	1.116	Q15643	TRIP11	Thyroid receptor-interacting protein 11
0.938	0.991	1.063	0.985	1.052	0.989	0.952	0.881	0.984	0.987	1.002	1.056	Q15654	TRIP6	Thyroid receptor-interacting protein 6
0.983	0.976	1.033	1.084	0.960	0.990	0.977	1.039	0.984	0.973	1.042	0.993	Q9P031	CCDC59	Thyroid transcription factor 1-associated protein 26
0.992	1.000	0.994	0.987	0.971	1.002	1.040	0.990	0.972	1.044	0.985	1.058	G5E9E7	TJP1	Tight junction protein 1 (Zona occludens 1), isoform CRA_e
1.008	1.039	1.012	1.043	0.999	1.024	1.090	1.058	0.999	1.065	0.940	1.126	A0A1B0GTW1	TJP2	Tight junction protein ZO-2
0.989	0.929	1.036	0.952	0.943	1.023	0.965	0.927	0.999	1.014	0.992	1.092	Q5JTD0	TJAP1	Tight junction-associated protein 1
0.998	0.972	0.993	1.092	1.034	1.135	1.053	1.033	1.026	1.041	1.067	1.196	Q9BVW5	TIPIN	TIMELESS-interacting protein
1.057	0.994	1.017	1.033	1.061	0.996	1.066	0.965	0.978	1.052	1.025	0.988	O75663	TIPRL	TIP41-like protein
1.045	0.997	1.026	0.953	0.984	1.105							Q8IUC6	TICAM1	TIR domain-containing adapter molecule 1
1.006	0.996	0.993	1.001	1.008	0.976	0.997	1.017	0.982	1.005	0.971	0.959	P04066	FUCA1	Tissue alpha-L-fucosidase
1.415	0.839	1.106	1.049	1.452	0.890	1.066	0.706	1.002	0.840	0.976	0.821	P48307	TFPI2	Tissue factor pathway inhibitor 2
0.990	0.941	0.942	0.897	0.866	0.897	1.014	1.036	0.862	0.904	0.864	0.954	P10646	TFPI	Tissue factor pathway inhibitor
0.912	0.949	0.850	0.997	0.827	1.095	1.041	1.020	0.965	1.105	0.986	1.059	A0A0A0MTS7	TTN	Titin
						1.119	1.207	1.384				A6NGC4	TLCD2	TLC domain-containing protein 2
0.969	0.934	1.118	1.042	0.888	1.060	0.904	0.725	0.957	1.085	0.984	1.093	Q6P9B6	TLDC1	TLD domain-containing protein 1
									1.109	1.099	1.150	J3KPA2	TM2D1	TM2 domain containing 1, isoform CRA_c
						0.843	1.088	1.170				Q13114	TRAF3	TNF receptor-associated factor 3
0.923	1.013	0.999	1.023	0.997	1.008	1.017	0.994	1.038	1.189	1.031	0.896	Q9Y4K3	TRAF6	TNF receptor-associated factor 6
0.979	0.950	1.126	0.995	0.946	1.056	1.103	1.083	1.059	0.829	0.804	1.153	Q15025	TNIP1	TNFAIP3-interacting protein 1
1.042	0.978	1.019	1.008	1.004	1.057	1.041	1.013	1.019	1.027	0.980	1.042	Q9H0E2	TOLLIP	Toll-interacting protein
1.014	1.003	1.063	0.982	0.899	0.999	1.035	0.915	1.074	1.026	0.929	1.058	O60603	TLR2	Toll-like receptor 2
1.070	1.077	1.034	0.949	0.984	1.046	1.027	1.135	1.017	1.015	1.030	1.052	O15455	TLR3	Toll-like receptor 3
1.123	0.954	0.988	1.023	0.977	1.022	1.047	1.080	1.012	1.047	1.019	1.021	O75674	TOM1L1	TOM1-like protein 1
1.026	0.983	0.994	1.004	0.967	1.015	1.010	1.075	1.024	1.019	0.990	1.023	Q6ZVM7	TOM1L2	TOM1-like protein 2
0.962	1.012	1.025	1.000	1.012	1.028	1.019	0.865	0.952	1.010	0.972	1.033	Q96HA7	TONSL	Tonsoku-like protein
0.959	0.990	1.011	1.020	0.969	1.055	1.042	1.056	0.987	1.050	0.980	1.014	O14656	TOR1A	Torsin-1A
0.963	0.991	1.020	0.988	1.036	0.984	0.995	0.915	1.001	1.036	1.000	1.048	Q5JTV8	TOR1AIP1	Torsin-1A-interacting protein 1
0.968	0.986	1.032	0.969	0.985	1.029	1.013	0.963	1.029	1.052	0.989	1.105	Q8NFQ8	TOR1AIP2	Torsin-1A-interacting protein 2
1.062	0.906	1.004										Q9H496	TOR1AIP2	Torsin-1A-interacting protein 2, isoform IFRG15

Abundance Ratio														
1 d M1/C1	1 d M2/C2	1 d M3/C3	4 d M1/C1	4 d M2/C2	4 d M3/C3	7 d M1/C1	7 d M2/C2	7 d M3/C3	10 d M1/C1	10 d M2/C2	10 d M3/C3	Accession ID	Gene name	Protein Description
1.036	1.053	1.033	1.032	0.990	1.019	1.067	1.145	1.024	0.964	1.000	1.046	O14657	TOR1B	Torsin-1B
0.936	1.152	0.948	0.950	0.903	1.160	0.927	1.071	0.920	0.923	0.912	1.110	Q5JU69	TOR2A	Torsin-2A
1.023	1.006	1.045	1.014	1.011	1.034	1.129	1.118	1.089	1.236	1.041	1.184	Q9H497	TOR3A	Torsin-3A
1.042	1.022	1.045	1.034	0.921	1.014	1.064	1.111	1.033	1.068	1.000	1.069	Q9NXH8	TOR4A	Torsin-4A
1.056	1.005	0.995	1.002	0.978	0.950	1.003	1.101	1.016	0.993	1.040	0.994	O94842	TOX4	TOX high mobility group box family member 4
0.878	0.939	0.908	0.940	0.993	0.969	0.992	0.949	1.043	1.036	1.133	1.029	O43715	TRIAP1	TP53-regulated inhibitor of apoptosis 1
1.057	1.037	1.056	1.012	1.058	1.046	1.022	0.983	1.037	0.965	0.995	1.025	Q96S44	TP53RK	TP53-regulating kinase
0.981	0.980	0.969	0.996	0.952	1.041	1.041	0.965	1.005	0.985	0.968	1.026	J3KPT4	TRABD	TraB domain-containing protein
1.057	1.015	1.044	0.948	0.958	1.014	0.963	0.969	0.955	0.980	0.963	0.965	Q92844	TANK	TRAF family member-associated NF-kappa-B activator
1.082	0.972	1.026	0.848	0.931	1.022	1.013	0.965	1.082	1.035	1.014	1.171	Q8TDR0	TRAF3IP1	TRAF3-interacting protein 1
1.156	1.040	1.133				0.892	1.003	0.748				O60296	TRAK2	Trafficking kinesin-binding protein 2
1.051	0.997	0.957	1.105	1.047	0.914	0.949	1.079	1.024	0.916	1.024	0.918	Q9Y5R8	TRAPPC1	Trafficking protein particle complex subunit 1
1.038	1.019	1.036	0.962	0.924	1.020	0.945	1.015	1.041	1.009	0.988	1.114	P48553	TRAPPC10	Trafficking protein particle complex subunit 10
1.052	1.024	1.041	0.995	0.942	1.002	0.935	1.048	0.992	0.947	0.986	0.964	Q7Z392	TRAPPC11	Trafficking protein particle complex subunit 11
1.020	0.972	1.081	1.007	0.975	1.042	0.995	1.013	1.041	1.000	0.994	1.041	Q8WVT3	TRAPPC12	Trafficking protein particle complex subunit 12
0.974	1.003	0.981	1.013	0.993	1.032	1.030	1.051	0.983	1.103	1.029	0.995	H3BP13	TRAPPC2L	Trafficking protein particle complex subunit 2-like protein
0.993	0.942	1.006	0.994	0.846	1.156	1.066	1.003	1.035	1.092	1.115	1.077	O43617	TRAPPC3	Trafficking protein particle complex subunit 3
1.060	0.989	1.022	1.026	0.995	1.040	1.015	1.119	1.031	0.977	0.978	1.005	Q9Y296	TRAPPC4	Trafficking protein particle complex subunit 4
0.981	1.011	1.027	1.034	1.028	1.041	1.005	1.051	0.993	1.011	1.045	1.017	Q8IUR0	TRAPPC5	Trafficking protein particle complex subunit 5
1.032	1.105	1.070	0.966	1.011	1.088	1.016	1.080	1.102	0.993	0.999	1.013	Q86SZ2	TRAPPC6B	Trafficking protein particle complex subunit 6B
1.032	0.992	1.000	1.038	1.016	1.057	1.049	0.998	1.051	1.035	0.992	1.037	Q9Y2L5	TRAPPC8	Trafficking protein particle complex subunit 8
									1.235	0.972	1.429	Q96CG3	TIFA	TRAF-interacting protein with FHA domain-containing protein A
1.008	0.981	1.144	1.034	1.004	0.862	0.856	0.611	0.802	1.088	1.099	1.103	O14545	TRAFD1	TRAF-type zinc finger domain-containing protein 1
0.989	1.007	1.086	0.985	0.962	1.025	1.002	0.994	1.034	1.040	0.976	1.065	Q96EM0	L3HYPDH	Trans-3-hydroxy-L-proline dehydratase
0.991	1.004	0.994	1.015	1.026	1.037	1.004	1.001	1.021	1.051	0.990	0.996	P37837	TALDO1	Transaldolase
1.048	0.998	1.002	0.937	0.968	0.998	1.017	1.038	0.994	0.975	0.995	0.954	Q9NPA8	ENY2	Transcription and mRNA export factor ENY2
0.952	1.007	0.950	1.003	1.118	1.207	0.679	1.296	0.971	0.891	1.282	1.118	G5E9M7	VGLL4	Transcription cofactor vestigial-like protein 4
									0.943	1.003	1.023	K7EMV4	ELOF1	Transcription elongation factor 1 homolog
0.990	1.005	0.995	1.001	1.050	1.023	1.017	0.938	1.027	0.993	0.983	1.062	P23193	TCEA1	Transcription elongation factor A protein 1
0.960	0.924	1.011										Q15560	TCEA2	Transcription elongation factor A protein 2
0.998	0.977	1.048	0.958	0.974	0.999	0.878	0.696	0.944	1.006	1.056	1.134	Q969E4	TCEAL3	Transcription elongation factor A protein-like 3

Abundance Ratio														
1 d M1/C1	1 d M2/C2	1 d M3/C3	4 d M1/C1	4 d M2/C2	4 d M3/C3	7 d M1/C1	7 d M2/C2	7 d M3/C3	10 d M1/C1	10 d M2/C2	10 d M3/C3	Accession ID	Gene name	Protein Description
1.072	1.006	0.925				0.904	1.312	1.081				Q9UHQ7	TCEAL9	Transcription elongation factor A protein-like 9
1.660	1.074	1.241	1.174	1.353	0.828	0.855	0.867	0.955	0.776	0.964	0.775	P63272	SUPT4H1	Transcription elongation factor SPT4
1.001	1.007	1.016	0.982	0.957	1.015	0.990	0.995	0.984	0.969	0.984	0.997	O00267	SUPT5H	Transcription elongation factor SPT5
1.032	1.012	1.021	1.037	0.988	1.018	1.024	1.047	1.010	0.979	0.988	1.029	Q7KZ85	SUPT6H	Transcription elongation factor SPT6
0.982	0.951	1.020	0.966	0.912	1.014	0.992	0.981	0.958	0.987	0.987	0.958	Q96QE5	TEFM	Transcription elongation factor, mitochondrial
1.042	1.009	0.998	1.000	0.980	1.005	1.006	1.024	0.990	1.014	1.007	0.980	O14776	TCERG1	Transcription elongation regulator 1
1.299	1.069	0.888										A0A0G2JIJ7	TCF19	Transcription factor 19
0.966	1.024	1.010	0.970	0.944	1.080	0.967	0.965	0.985	1.018	0.998	1.066	Q9UGU0	TCF20	Transcription factor 20
1.002	1.004	1.043	0.994	1.076	0.994	1.006	0.960	1.021	1.052	0.973	1.061	Q9BQ70	TCF25	Transcription factor 25
1.035	0.993	0.983	1.007	0.967	0.993	0.974	0.913	0.931	0.986	0.990	0.948	Q00059	TFAM	Transcription factor A, mitochondrial
0.904	1.057	0.996	1.020	0.854	0.965	1.043	0.960	1.053	1.052	0.947	1.105	P05412	JUN	Transcription factor AP-1
1.011	1.006	1.009	0.999	0.981	1.004	1.000	1.056	0.989	0.999	1.047	1.024	Q96SH1	TFAP2A	Transcription factor AP-2 alpha (Activating enhancer binding protein 2 alpha), isoform CRA_c
						0.972	1.006	0.990	1.026	0.969	1.039	Q92754	TFAP2C	Transcription factor AP-2 gamma
			0.900	0.825	1.045				1.070	1.045	0.979	Q7Z6R9	TFAP2D	Transcription factor AP-2-delta
0.900	0.941	0.997	0.900	0.875	0.991	0.891	0.865	0.983	1.072	1.013	0.880	Q01664	TFAP4	Transcription factor AP-4
1.010	0.999	1.000	1.002	0.937	0.990	0.950	1.040	0.972	0.846	0.989	0.845	Q96K17	BTF3L4	Transcription factor BTF3 homolog 4
1.023	0.985	1.018	0.990	0.948	1.007	0.957	1.024	0.985	0.925	1.019	0.929	P20290	BTF3	Transcription factor BTF3
0.989	0.992	1.000	0.908	0.848	1.077	0.979	1.093	1.058	1.003	0.976	1.069	Q14186	TFDP1	Transcription factor Dp-1
0.938	0.817	0.963	0.923	1.284	0.894	1.389	1.014	0.734				X6REB3	TCF3	Transcription factor E2-alpha
0.960	0.771	1.131	0.871	0.896	1.104	1.010	1.104	1.218	0.915	0.972	1.181	O00716	E2F3	Transcription factor E2F3
1.092	1.017	0.989	1.046	1.061	1.000	1.018	0.924	1.070	1.018	0.959	1.049	Q16254	E2F4	Transcription factor E2F4
0.983	1.035	1.088	1.021	0.978	1.002	0.986	0.905	1.009	1.081	1.010	1.114	P19532	TFE3	Transcription factor E3
0.979	1.098	1.026	1.034	1.091	0.953	0.965	1.297	0.977	0.895	1.036	0.926	P41212	ETV6	Transcription factor ETV6
1.070	0.837	1.030										Q9Y543	HES2	Transcription factor HES-2
			0.997	0.787	1.137	0.944	1.093	1.075	0.946	1.002	0.943	P31629	HIVEP2	Transcription factor HIVEP2
						0.860	0.650	1.147				Q9HAW0	BRF2	Transcription factor IIIB 50 kDa subunit
0.938	1.039	0.975	1.083	0.923	0.971				1.133	1.112	1.300	Q92994	BRF1	Transcription factor IIIB 90 kDa subunit
						1.003	1.077	1.023	1.046	1.229	0.861	P17275	JUNB	Transcription factor jun-B
1.716	0.846	0.942	1.280	0.906	1.011	1.431	1.083	0.987	1.253	0.969	1.008	P17535	JUND	Transcription factor jun-D
0.967	1.009	1.071	1.051	0.880	1.072	1.013	1.253	1.021	0.924	0.963	0.948	Q9ULX9	MAFF	Transcription factor MafF
0.954	1.003	1.030	1.033	0.918	1.077	1.173	1.135	1.022	0.969	1.035	0.997	O15525	MAFG	Transcription factor MafG
			1.043	0.901	1.069	1.055	1.055	0.975	0.960	1.065	1.050	O60675	MAFK	Transcription factor MafK
1.029	1.000	0.986	1.018	0.959	1.014	1.014	1.006	1.020	1.025	0.992	1.040	Q04206	RELA	Transcription factor p65

Abundance Ratio														
1 d M1/C1	1 d M2/C2	1 d M3/C3	4 d M1/C1	4 d M2/C2	4 d M3/C3	7 d M1/C1	7 d M2/C2	7 d M3/C3	10 d M1/C1	10 d M2/C2	10 d M3/C3	Accession ID	Gene name	Protein Description
1.048	1.106	1.058	1.013	0.904	0.994	1.040	1.132	1.022	1.043	0.997	1.048	Q01201	RELB	Transcription factor RelB
			0.721	1.561	1.070	0.926	0.636	0.960				P35716	SOX11	Transcription factor SOX-11
1.105	0.878	1.172	1.014	0.907	1.147	0.988	0.858	1.043	1.155	1.037	1.158	Q9Y651	SOX21	Transcription factor SOX-21
0.770	1.312	0.906										P48436	SOX9	Transcription factor SOX-9
0.930	0.956	0.955	0.951	0.921	0.980	1.174	0.763	1.091	1.069	1.016	1.111	P08047	SP1	Transcription factor Sp1
			1.068	1.123	1.261	0.925	1.163	1.238				Q02086	SP2	Transcription factor Sp2
1.004	1.037	0.999	1.022	0.936	0.956	1.026	1.023	0.967	1.101	1.001	1.129	Q02447	SP3	Transcription factor Sp3
									1.133	1.003	1.185	Q02446	SP4	Transcription factor Sp4
0.829	1.016	0.820	1.051	0.618	1.356	1.395	1.183	1.071	1.192	1.139	0.822	A0A0G2JNU3	BDP1	Transcription factor TFIIIB component B'' homolog
0.980	1.039	1.069				1.257	0.845	1.096	0.990	0.936	1.171	P52655	GTF2A1	Transcription initiation factor IIA subunit 1
0.986	0.997	0.979	1.021	0.954	1.109	0.927	1.036	0.998	0.964	1.020	1.085	P52657	GTF2A2	Transcription initiation factor IIA subunit 2
1.027	0.976	1.023	1.021	0.953	1.036	1.059	1.037	1.047	1.020	1.005	1.026	Q00403	GTF2B	Transcription initiation factor IIB
1.045	0.962	1.013	1.008	0.978	1.039	1.024	1.050	1.034	1.011	0.999	1.027	P29084	GTF2E2	Transcription initiation factor IIE subunit beta
1.150	0.997	1.055	1.026	0.981	0.999	1.038	0.980	0.993	0.973	0.991	0.973	Q12962	TAF10	Transcription initiation factor TFIID subunit 10
0.997	0.972	1.070	0.781	1.089	1.111	0.953	0.957	1.003				Q16514	TAF12	Transcription initiation factor TFIID subunit 12
0.997	1.019	1.073	0.972	1.143	1.077	1.008	1.045	1.091	1.002	1.036	0.949	Q6P1X5	TAF2	Transcription initiation factor TFIID subunit 2
						1.059	0.915	0.902				Q5VWG9	TAF3	Transcription initiation factor TFIID subunit 3
1.001	0.993	1.057	0.998	1.091	1.008	0.986	1.117	1.056	0.995	0.961	1.096	O00268	TAF4	Transcription initiation factor TFIID subunit 4
1.045	1.016	1.019	0.944	0.967	1.034	0.912	1.088	1.021	0.990	0.910	1.011	Q15542	TAF5	Transcription initiation factor TFIID subunit 5
0.976	0.957	1.018	1.021	0.895	1.035	0.953	1.089	1.048	1.017	1.031	1.067	J3KR72	TAF6	Transcription initiation factor TFIID subunit 6
1.028	0.979	0.957	0.999	0.901	1.068	0.992	1.068	1.072	0.975	0.968	1.018	Q15545	TAF7	Transcription initiation factor TFIID subunit 7
			0.920	0.954	0.992	0.966	1.177	1.002	0.826	0.972	0.902	Q16594	TAF9	Transcription initiation factor TFIID subunit 9
1.003	1.028	1.048	0.992	0.889	1.116	1.083	1.131	1.051	1.001	0.983	1.099	Q9HBM6	TAF9B	Transcription initiation factor TFIID subunit 9B
0.946	0.980	0.962	0.935	0.929	0.902	1.047	1.127	0.906				O75486	SUPT3H	Transcription initiation protein SPT3 homolog
1.031	1.012	0.985	0.956	0.876	1.044	0.991	0.981	1.018	1.032	1.004	1.063	O15164	TRIM24	Transcription intermediary factor 1-alpha
1.015	0.970	1.072	0.930	1.076	1.109	0.854	0.952	1.032	1.026	1.090	1.108	M0R0K9	TRIM28	Transcription intermediary factor 1-beta (Fragment)
1.014	0.998	1.004	0.985	1.020	0.985	0.983	0.943	0.987	0.988	1.010	1.012	Q13263	TRIM28	Transcription intermediary factor 1-beta
0.966	1.003	1.122	0.877	0.925	1.179	1.104	1.138	0.998	0.995	1.000	0.840	O14867	BACH1	Transcription regulator protein BACH1
0.986	1.000	1.049	0.717	1.023	1.181	0.841	1.228	0.954				Q15361	TTF1	Transcription termination factor 1
1.149	1.004	1.014	1.089	1.175	1.243							Q99551	MTERF1	Transcription termination factor 1, mitochondrial
1.019	1.027	1.026	1.046	0.955	1.020	1.031	1.091	0.993	0.966	0.941	1.034	Q9UNY4	TTF2	Transcription termination factor 2

Abundance Ratio														
1 d M1/C1	1 d M2/C2	1 d M3/C3	4 d M1/C1	4 d M2/C2	4 d M3/C3	7 d M1/C1	7 d M2/C2	7 d M3/C3	10 d M1/C1	10 d M2/C2	10 d M3/C3	Accession ID	Gene name	Protein Description
1.065	0.999	1.060	1.004	0.957	1.020	1.066	1.061	0.961	1.036	0.989	1.060	Q96E29	MTERF3	Transcription termination factor 3, mitochondrial
1.068	0.999	1.067	0.943	0.957	1.012	0.973	0.963	0.915	1.114	1.066	1.050	Q7Z6M4	MTERF4	Transcription termination factor 4, mitochondrial
1.040	1.018	0.977	1.006	0.978	0.965	1.010	1.039	0.983	1.044	0.988	0.969	Q00577	PURA	Transcriptional activator protein Pur-alpha
1.041	0.986	0.993	1.019	1.017	1.054	0.999	1.001	1.024	1.076	1.030	1.047	Q96QR8	PURB	Transcriptional activator protein Pur-beta
0.995	0.967	1.056	1.010	1.054	0.954	0.986	1.126	1.013	1.004	0.917	1.232	Q96BN2	TADA1	Transcriptional adapter 1
1.025	0.988	1.021	1.028	0.877	0.908	0.978	1.115	1.033	1.015	1.033	1.003	Q86TJ2	TADA2B	Transcriptional adapter 2-beta
1.014	1.006	1.034	0.968	0.986	0.990	0.988	0.869	1.023	1.261	0.967	1.212	O75528	TADA3	Transcriptional adapter 3
1.025	0.982	1.041	1.012	0.891	0.999	0.924	1.036	0.976	0.940	0.957	1.024	H0YE88	TEAD1	Transcriptional enhancer factor TEF-1
1.148	1.032	1.045	1.169	1.514	1.281				0.899	1.144	0.910	Q99594	TEAD3	Transcriptional enhancer factor TEF-5
1.010	0.973	1.040	1.045	0.955	0.930	0.948	1.174	1.047	0.964	0.973	0.919	Q5T5J6	SWT1	Transcriptional protein SWT1
0.994	0.995	1.005	0.952	0.954	1.004	1.028	1.004	0.967	1.099	1.021	1.147	P46100	ATRX	Transcriptional regulator ATRX
0.937	0.955	1.033	1.013	1.011	1.016	0.988	0.902	1.026	1.064	1.029	1.145	Q86T24	ZBTB33	Transcriptional regulator Kaiso
0.923	1.022	0.968	1.025	1.056	0.979	1.072	0.934	0.969	1.035	0.985	1.004	P49711	CTCF	Transcriptional repressor CTCF
1.105	0.984	1.032	1.006	1.011	0.964	1.051	0.995	0.989	1.074	1.010	1.087	Q12986	NFX1	Transcriptional repressor NF-X1
1.038	1.010	1.029	0.999	0.992	0.976	1.038	1.076	0.997	1.013	1.000	1.063	Q8WXI9	GATAD2B	Transcriptional repressor p66-beta
1.072	1.047	0.995	1.040	1.003	0.952	1.151	1.054	0.982	1.092	1.031	1.050	P25490	YY1	Transcriptional repressor protein YY1
1.004	0.998	0.999	1.011	1.022	1.029	1.011	0.982	1.033	1.051	1.061	1.105	Q9Y4P3	TBL2	Transducin beta-like protein 2
1.015	0.997	1.056	0.973	0.973	1.015	0.996	0.997	1.052	0.972	1.020	0.995	Q12788	TBL3	Transducin beta-like protein 3
1.001	1.038	1.063							1.029	0.894	1.026	Q04724	TLE1	Transducin-like enhancer protein 1
0.977	1.002	0.975	0.967	0.951	1.017	1.037	1.038	1.038	1.018	1.021	1.004	H0YL70	TLE3	Transducin-like enhancer protein 3
0.782	0.810	0.802	0.605	0.562	0.594	0.641	0.720	0.648	0.630	0.693	0.672	P02786	TFRC	Transferrin receptor protein 1
1.021	1.004	1.036	1.001	0.937	1.027	0.955	1.120	1.007	1.006	0.994	0.991	Q9Y4A5	TRRAP	Transformation/transcription domain-associated protein
1.108	1.052	0.971	1.013	0.964	0.952	0.971	1.022	0.981	0.977	1.017	0.954	Q13595	TRA2A	Transformer-2 protein homolog alpha
1.061	0.994	1.027	0.987	0.924	0.951	0.943	0.965	0.961	0.933	1.011	0.931	P62995	TRA2B	Transformer-2 protein homolog beta
1.019	1.006	1.052	0.994	0.972	0.985	1.021	0.980	0.966	1.017	0.987	1.055	O75410	TACC1	Transforming acidic coiled-coil-containing protein 1
0.988	0.941	1.057	0.961	0.987	1.017	0.974	0.997	1.006	0.982	0.930	1.056	O95359	TACC2	Transforming acidic coiled-coil-containing protein 2
0.953	1.005	0.997	0.970	0.819	0.958	1.104	1.043	0.998	0.926	0.845	1.009	Q9Y6A5	TACC3	Transforming acidic coiled-coil-containing protein 3
0.877	0.831	0.940										Q03167	TGFBR3	Transforming growth factor beta receptor type 3
0.981	0.836	0.898	1.236	1.329	0.858	0.951	0.960	1.018	0.861	0.885	0.991	P01137	TGFB1	Transforming growth factor beta-1

Abundance Ratio														
1 d M1/C1	1 d M2/C2	1 d M3/C3	4 d M1/C1	4 d M2/C2	4 d M3/C3	7 d M1/C1	7 d M2/C2	7 d M3/C3	10 d M1/C1	10 d M2/C2	10 d M3/C3	Accession ID	Gene name	Protein Description
0.976	1.077	0.996	1.003	1.160	0.973	1.041	0.811	1.024	1.033	0.976	1.039	O43294	TGFB1I1	Transforming growth factor beta-1-induced transcript 1 protein
1.044	1.008	1.056	0.999	0.920	1.052	1.025	1.045	1.054	1.015	1.004	1.044	Q8WUH2	TGFBRAP1	Transforming growth factor-beta receptor-associated protein 1
1.039	1.051	0.994	0.925	0.915	0.967	1.040	0.969	0.990	0.748	0.806	0.808	Q15582	TGFBI	Transforming growth factor-beta-induced protein ig-h3
0.945	0.980	0.964	1.065	1.020	0.954	1.055	0.978	0.951	1.015	1.103	0.930	P61586	RHOA	Transforming protein RhoA
0.844	0.958	1.187	0.895	0.919	1.033	1.098	1.159	1.034	1.002	0.895	1.078	Q01995	TAGLN	Transgelin
0.872	0.815	0.963	1.014	0.972	1.163	0.945	1.076	1.008	1.063	0.971	1.080	Q9UI15	TAGLN3	Transgelin-3
0.863	0.933	0.983	0.844	0.899	0.949	0.917	0.795	0.971	0.997	0.938	1.072	O43493	TGOLN2	Trans-Golgi network integral membrane protein 2
1.009	1.025	1.011	1.015	1.018	0.955	1.049	1.215	0.986	1.086	1.142	1.094	Q8TD43	TRPM4	Transient receptor potential cation channel subfamily M member 4
0.954	0.995	1.032	0.997	0.939	0.997	1.014	0.995	0.994	0.998	1.031	1.093	Q9Y5S1	TRPV2	Transient receptor potential cation channel subfamily V member 2
0.997	1.018	0.993	1.002	1.124	0.997	1.019	1.009	1.054	0.993	1.014	1.009	P55072	VCP	Transitional endoplasmic reticulum ATPase
0.769	1.023	0.798	1.038	1.141	0.911	1.215	1.196	0.940	1.396	1.062	1.106	A0A0B4J1R6	TKT	Transketolase
0.966	0.963	1.028	0.946	0.934	1.035	0.950	1.137	1.041	0.962	1.017	1.093	Q8N442	GUF1	Translation factor GUF1, mitochondrial
1.034	0.983	0.985	1.002	0.995	1.029	1.014	0.996	1.010	0.992	0.962	0.978	Q14232	EIF2B1	Translation initiation factor eIF-2B subunit alpha
1.039	0.973	1.039	0.969	0.942	1.029	0.948	0.954	1.017	0.976	0.969	1.048	P49770	EIF2B2	Translation initiation factor eIF-2B subunit beta
1.038	1.016	1.011	0.973	0.993	1.013	0.984	0.976	0.988	1.047	1.000	1.032	E7ERK9	EIF2B4	Translation initiation factor eIF-2B subunit delta
0.980	0.967	1.036	1.000	0.881	0.991	0.943	0.981	0.992	0.926	0.952	1.012	Q13144	EIF2B5	Translation initiation factor eIF-2B subunit epsilon
0.993	1.020	0.990	1.002	1.026	0.993	1.017	1.051	1.011	1.015	1.010	1.005	Q9NR50	EIF2B3	Translation initiation factor eIF-2B subunit gamma
0.992	0.975	1.010	0.967	1.007	0.984	0.975	0.882	1.013	0.998	0.997	1.007	P46199	MTIF2	Translation initiation factor IF-2, mitochondrial
1.047	1.008	1.025	0.976	0.956	0.993	1.027	0.935	0.989	0.996	1.071	1.061	Q9H2K0	MTIF3	Translation initiation factor IF-3, mitochondrial
1.033	0.997	1.003	1.012	0.984	1.023	1.062	1.084	0.999	1.001	1.035	0.987	H0Y9X1	TMA16	Translation machinery-associated protein 16 (Fragment)
0.911	0.901	0.952	0.857	0.979	1.001	0.877	0.628	0.869	1.015	0.976	0.975	Q9Y2S6	TMA7	Translation machinery-associated protein 7
0.943	1.008	1.045	0.959	0.912	0.939	0.958	0.902	0.946	0.989	0.928	0.979	Q9BSH4	TACO1	Translational activator of cytochrome c oxidase 1
0.985	1.011	0.990	1.021	0.994	1.016	0.951	0.993	1.003	0.943	1.023	0.942	A0A0B4J2C3	TPT1	Translationally-controlled tumor protein
1.016	0.973	0.995	0.987	0.941	1.028	1.013	1.012	1.049	1.042	1.027	0.971	Q15631	TSN	Translin
0.964	0.974	0.991	1.000	1.001	1.033	0.986	0.911	1.019	0.994	1.016	1.021	Q99598	TSNAX	Translin-associated protein X
0.974	1.020	0.992	1.012	0.992	0.996	1.080	1.064	1.012	1.159	1.062	1.089	Q15629	TRAM1	Translocating chain-associated membrane protein 1
0.964	1.015	1.037	0.963	0.925	1.000	0.971	0.912	0.978	1.064	1.043	1.069	Q99442	SEC62	Translocation protein SEC62

Abundance Ratio														
1 d M1/C1	1 d M2/C2	1 d M3/C3	4 d M1/C1	4 d M2/C2	4 d M3/C3	7 d M1/C1	7 d M2/C2	7 d M3/C3	10 d M1/C1	10 d M2/C2	10 d M3/C3	Accession ID	Gene name	Protein Description
0.982	1.012	1.015	0.980	0.940	0.978	0.993	1.080	1.000	1.003	1.016	0.996	Q9UGP8	SEC63	Translocation protein SEC63 homolog
0.967	0.904	0.990	0.954	0.844	1.100	1.055	0.950	0.948	1.108	0.971	0.959	P30536	TSPO	Translocator protein
0.965	0.991	0.990	0.981	0.982	1.037	1.022	1.090	0.982	0.998	1.018	0.973	P43307	SSR1	Translocon-associated protein subunit alpha
1.012	1.006	1.024	0.983	0.976	0.946	0.985	1.021	0.956	0.915	1.017	0.920	P51571	SSR4	Translocon-associated protein subunit delta
									1.029	0.941	0.997	Q96CE8	TM4SF18	Transmembrane 4 L6 family member 18
1.057	0.965	1.005	1.162	1.179	1.174	1.368	1.225	1.223				Q9NS93	TM7SF3	Transmembrane 7 superfamily member 3
0.955	0.993	0.994	0.958	0.887	0.989	1.028	1.005	1.001	1.055	1.040	1.037	Q99805	TM9SF2	Transmembrane 9 superfamily member 2
1.087	1.004	0.990	1.073	0.989	0.983	1.080	1.022	1.050	1.086	1.054	0.956	Q9HD45	TM9SF3	Transmembrane 9 superfamily member 3
0.933	1.000	0.987	0.966	0.918	1.018	1.011	1.001	1.016	1.018	1.028	1.014	Q92544	TM9SF4	Transmembrane 9 superfamily member 4
0.971	0.975	1.000	0.991	0.937	1.015	1.029	1.028	1.015	1.099	1.062	1.063	E9PSI1	3 SV	Transmembrane 9 superfamily member
0.943	1.057	0.947	1.005	0.971	1.109	0.895	1.029	1.226	0.886	0.954	1.124	Q6UWJ1	TMCO3	Transmembrane and coiled-coil domain-containing protein 3
			0.949	0.770	1.170							Q5TGY1	TMCO4	Transmembrane and coiled-coil domain-containing protein 4
			1.112	0.869	1.042							Q8N394	TMTC2	Transmembrane and TPR repeat-containing protein 2
0.936	0.973	1.012	1.004	0.947	1.016	1.040	1.045	1.009	1.095	1.008	1.007	Q6ZXV5	TMTC3	Transmembrane and TPR repeat-containing protein 3
0.887	0.971	0.986	0.899	1.052	1.157	1.013	1.120	1.048	0.996	1.004	1.209	Q9BVT8	TMUB1	Transmembrane and ubiquitin-like domain-containing protein 1
0.896	0.992	0.933	0.829	0.995	0.983	0.823	1.269	1.089	0.947	0.948	0.901	Q71RG4	TMUB2	Transmembrane and ubiquitin-like domain-containing protein 2
			1.110	0.902	0.908	0.921	1.092	1.249	0.910	0.742	0.943	Q6NXT6	TAPT1	Transmembrane anterior posterior transformation protein 1 homolog
0.922	0.954	0.983	1.011	0.899	1.012	1.066	0.922	0.976	1.005	0.976	1.031	Q13445	TMED1	Transmembrane emp24 domain-containing protein 1
0.961	0.973	0.992	0.979	1.008	1.033	0.990	0.977	1.009	0.967	1.002	0.974	P49755	TMED10	Transmembrane emp24 domain-containing protein 10
0.884	0.983	0.962	0.979	0.934	1.053	1.022	0.980	0.971	1.062	1.043	1.045	Q15363	TMED2	Transmembrane emp24 domain-containing protein 2
									0.884	0.951	1.164	Q9Y3Q3	TMED3	Transmembrane emp24 domain-containing protein 3
0.983	0.970	0.999	1.027	0.973	1.026	1.018	1.101	1.029	1.024	1.012	1.005	Q7Z7H5	TMED4	Transmembrane emp24 domain-containing protein 4
0.893	0.971	0.965	0.984	0.908	1.033	1.045	1.057	1.004	1.009	0.978	1.020	Q9Y3A6	TMED5	Transmembrane emp24 domain-containing protein 5
0.960	1.005	1.017	0.988	0.964	0.994	0.961	0.952	0.990	0.981	0.983	0.960	Q9BVK6	TMED9	Transmembrane emp24 domain-containing protein 9

Abundance Ratio														
1 d M1/C1	1 d M2/C2	1 d M3/C3	4 d M1/C1	4 d M2/C2	4 d M3/C3	7 d M1/C1	7 d M2/C2	7 d M3/C3	10 d M1/C1	10 d M2/C2	10 d M3/C3	Accession ID	Gene name	Protein Description
			1.161	1.188	0.924	1.040	1.034	1.136	1.140	1.047	0.985	Q96IK0	TMEM101	Transmembrane protein 101
			0.934	0.824	1.116	0.958	1.030	0.997				Q8N9M5	TMEM102	Transmembrane protein 102
1.032	1.023	0.943	1.092	1.009	1.006	1.136	1.015	1.002	1.170	1.188	1.064	Q9NUM4	TMEM106B	Transmembrane protein 106B
0.915	0.963	0.978	0.944	0.907	1.002	1.003	0.944	0.986	1.117	1.002	1.075	Q9BVC6	TMEM109	Transmembrane protein 109
1.038	0.953	1.014	1.016	1.026	0.969	1.024	0.826	1.025	1.074	1.043	1.012	P17152	TMEM11	Transmembrane protein 11, mitochondrial
0.983	0.938	1.003	0.959	0.915	0.951	1.005	0.884	0.893	0.995	0.964	1.043	Q12893	TMEM115	Transmembrane protein 115
1.078	1.019	1.031	1.032	0.892	0.930	0.937	1.031	0.993	1.111	1.029	1.124	A0A087X266	TMEM120A	Transmembrane protein 120A
			0.981	1.287	0.865	1.026	1.063	1.037	1.106	1.071	1.163	A0PK00	TMEM120B	Transmembrane protein 120B
0.999	0.969	1.023	1.024	0.924	1.044	1.011	1.067	0.999	0.970	1.023	0.985	Q9H061	TMEM126A	Transmembrane protein 126A
0.958	0.990	1.226	0.867	0.854	1.079	0.915	0.678	1.099				Q92545	TMEM131	Transmembrane protein 131
0.934	1.066	0.894	0.962	0.857	1.066	1.039	1.181	0.983	1.046	1.077	1.068	Q9H6X4	TMEM134	Transmembrane protein 134
0.970	1.060	0.981	1.015	0.953	1.004	0.960	1.131	1.050	1.052	1.084	1.087	Q86UB9	TMEM135	Transmembrane protein 135
									1.108	1.058	1.100	Q96I45	TMEM141	Transmembrane protein 141
0.858	1.229	0.908				1.361	1.008	1.137	1.402	0.897	1.105	Q9BVK8	TMEM147	Transmembrane protein 147
1.116	0.991	1.028	1.116	0.981	0.950	0.919	1.060	1.013	0.944	0.980	0.922	Q9P0S9	TMEM14C	Transmembrane protein 14C
1.439	0.894	1.271	0.932	0.923	1.090	0.994	1.019	1.044				A8MWL7	TMEM14DP	Transmembrane protein 14DP
0.948	0.941	1.043	0.969	0.915	0.929	0.926	0.954	0.902	1.017	0.973	1.004	Q9NX00	TMEM160	Transmembrane protein 160
			0.934	0.890	0.950	0.946	1.068	1.023				Q9NX61	TMEM161A	Transmembrane protein 161A
1.035	0.983	1.009	0.989	0.921	1.004	1.065	1.074	1.011	1.082	1.046	1.110	Q9HC07	TMEM165	Transmembrane protein 165
0.896	1.119	1.018	1.005	1.033	0.959	1.020	1.197	1.059	0.925	1.062	0.884	Q9H0V1	TMEM168	Transmembrane protein 168
1.046	1.105	1.050	1.020	0.975	0.975	1.016	1.054	0.974	1.063	1.140	1.037	Q53S58	TMEM177	Transmembrane protein 177
1.014	0.967	1.074	1.003	1.008	1.118	1.121	0.886	1.091	1.290	1.103	1.133	Q7Z7N9	TMEM179B	Transmembrane protein 179B
1.002	1.061	1.036	1.103	1.042	1.058	1.097	1.116	1.070	1.068	1.107	1.102	Q9P2C4	TMEM181	Transmembrane protein 181
1.010	0.931	1.062	0.960	0.894	1.025	0.938	0.810	1.040	1.126	1.221	1.273	Q9Y519	TMEM184B	Transmembrane protein 184B
1.025	0.924	1.096	0.887	0.898	1.016	1.010	0.980	1.089	1.076	1.063	1.117	Q9NVA4	TMEM184C	Transmembrane protein 184C
						1.118	1.080	0.997				Q8NFB2	TMEM185A	Transmembrane protein 185A
1.159	1.078	1.069	0.917	0.804	1.066	0.960	1.074	1.005	1.028	0.926	0.985	Q96B77	TMEM186	Transmembrane protein 186
0.973	0.942	0.932	0.991	0.901	0.922	1.051	0.976	0.997	1.054	1.020	1.044	Q96HH6	TMEM19	Transmembrane protein 19
0.975	0.992	1.023	0.946	0.879	1.038	1.046	1.077	1.045	1.048	1.022	0.984	Q8IY95	TMEM192	Transmembrane protein 192
1.158	0.841	0.909	1.020	0.932	1.063	1.062	0.839	1.112	1.051	1.046	1.116	Q8N511	TMEM199	Transmembrane protein 199
1.349	1.155	1.075	1.106	1.134	0.834	1.189	0.996	0.951				Q9UHN6	TMEM2	Transmembrane protein 2
0.954	1.031	1.038	0.951	0.881	0.993	1.031	1.118	1.022	1.013	1.009	0.977	Q5SNT2	TMEM201	Transmembrane protein 201
0.977	1.031	0.942	0.973	0.940	1.028	1.031	1.157	1.020	1.098	1.078	1.029	Q6UW68	TMEM205	Transmembrane protein 205
						1.201	1.006	0.941				Q9BTX3	TMEM208	Transmembrane protein 208

Abundance Ratio														
1 d M1/C1	1 d M2/C2	1 d M3/C3	4 d M1/C1	4 d M2/C2	4 d M3/C3	7 d M1/C1	7 d M2/C2	7 d M3/C3	10 d M1/C1	10 d M2/C2	10 d M3/C3	Accession ID	Gene name	Protein Description
0.947	1.002	0.943	1.118	0.988	0.984	1.068	1.072	1.014	1.123	1.133	1.076	Q96SK2	TMEM209	Transmembrane protein 209
0.965	1.016	1.000	0.994	0.906	0.975	1.031	1.148	1.014	1.038	1.023	1.037	Q6NUQ4	TMEM214	Transmembrane protein 214
1.110	1.004	1.099	0.979	0.785	1.033	0.942	1.125	1.050	1.067	1.144	0.955	Q9H0R3	TMEM222	Transmembrane protein 222
0.974	0.861	0.985	0.999	0.931	1.029	1.071	1.047	1.121	1.094	0.911	1.033	A0PJW6	TMEM223	Transmembrane protein 223
			0.862	0.744	1.288							C9JI98	TMEM238	Transmembrane protein 238
						0.934	0.911	0.778	0.988	1.019	1.044	C9JY04	TMEM243	Transmembrane protein 243
0.836	0.853	1.115										Q9BRR3	TMEM246	Transmembrane protein 246
			0.909	0.999	0.931	0.829	1.086	1.028				Q8N2U0	TMEM256	Transmembrane protein 256
			1.035	0.875	1.176							P61165	TMEM258	Transmembrane protein 258
			1.024	0.703	1.059							Q9NX78	TMEM260	Transmembrane protein 260
1.010	0.995	0.976	0.968	0.932	0.939	0.926	1.104	0.974	0.927	0.964	0.938	Q8WUH6	TMEM263	Transmembrane protein 263
0.952	0.927	0.823	0.786	0.987	1.171	1.279	1.108	1.222	1.175	0.981	1.050	Q0VDI3	TMEM267	Transmembrane protein 267
0.913	0.959	0.940	0.933	0.884	1.034	0.991	1.068	0.995	1.069	1.030	0.965	P57088	TMEM33	Transmembrane protein 33
0.952	0.786	1.012	0.728	0.697	1.324	1.067	0.820	1.051	0.944	0.920	1.198	Q8NCS4	TMEM35B	Transmembrane protein 35B
1.002	0.897	0.945	1.029	0.973	0.964	1.090	1.088	1.033	1.027	1.023	1.125	Q8WWA1	TMEM40	Transmembrane protein 40
0.894	0.976	1.058	0.870	0.859	1.161	1.022	0.899	1.086	1.099	0.943	1.168	Q96HV5	TMEM41A	Transmembrane protein 41A
1.094	0.844	1.115				1.177	1.155	1.008				Q5BJD5	TMEM41B	Transmembrane protein 41B
0.973	0.986	1.015	0.974	0.897	0.984	0.998	1.031	0.988	1.029	1.016	0.977	Q9BTV4	TMEM43	Transmembrane protein 43
0.713	1.051	1.028	1.074	0.913	0.992							Q9BQJ4	TMEM47	Transmembrane protein 47
			0.837	1.035	0.940	0.885	0.865	0.963	0.965	1.017	0.989	Q9Y2B1	TMEM5	Transmembrane protein 5
									1.245	1.047	1.046	O95807	TMEM50A	Transmembrane protein 50A
									0.904	0.935	1.443	Q9BXS4	TMEM59	Transmembrane protein 59
1.006	1.033	0.934	1.078	0.926	1.056	1.003	1.200	1.327	1.056	1.008	1.013	Q0P6H9	TMEM62	Transmembrane protein 62
0.926	0.967	1.005	0.987	0.928	1.091	1.050	1.008	1.021	1.208	0.991	1.099	Q6PI78	TMEM65	Transmembrane protein 65
						1.030	1.057	0.861	0.960	1.064	1.012	Q96MH6	TMEM68	Transmembrane protein 68
1.035	0.991	1.011	1.038	1.112	0.981	1.060	1.074	0.992	1.000	0.993	0.968	Q9BUB7	TMEM70	Transmembrane protein 70, mitochondrial
1.002	0.995	1.004	0.995	0.928	1.027	0.984	1.079	1.067	1.007	0.995	1.029	Q8NBN3	TMEM87A	Transmembrane protein 87A
0.991	1.053	1.036	0.907	0.829	0.976	0.898	0.894	0.941	1.015	0.932	0.997	Q96K49	TMEM87B	Transmembrane protein 87B
1.025	1.031	0.964	1.112	0.996	0.934	1.066	1.319	1.003	1.183	1.121	1.184	Q9HCN3	TMEM8A	Transmembrane protein 8A
0.964	0.946	0.996	0.990	0.984	1.085	0.943	0.681	0.988	1.079	1.061	1.098	B1ALM5	TMEM9	Transmembrane protein 9
0.946	0.962	0.735	0.950	0.958	1.019	1.160	0.973	1.069	1.278	1.100	1.172	Q5BJF2	TMEM97	Transmembrane protein 97
0.985	1.149	1.185	1.028	1.040	1.138	1.150	0.914	1.096	1.193	1.131	1.257	Q9NQ34	TMEM9B	Transmembrane protein 9B
									1.203	0.909	1.145	Q86W33	TPRA1	Transmembrane protein adipocyte-associated 1

Abundance Ratio														
1 d M1/C1	1 d M2/C2	1 d M3/C3	4 d M1/C1	4 d M2/C2	4 d M3/C3	7 d M1/C1	7 d M2/C2	7 d M3/C3	10 d M1/C1	10 d M2/C2	10 d M3/C3	Accession ID	Gene name	Protein Description
1.018	1.033	1.063	0.988	1.057	0.975	1.025	1.058	0.989	0.935	1.062	1.099	Q9C0B7	TANGO6	Transport and Golgi organization protein 6 homolog
0.989	1.002	1.012	0.993	1.020	1.008	1.003	1.045	1.030	0.947	1.008	0.987	Q92973	TNPO1	Transportin-1
0.994	1.002	1.016	0.989	1.016	1.053	1.050	1.042	1.062	1.014	0.979	1.037	Q9Y5L0	TNPO3	Transportin-3
0.891	1.260	0.874	0.999	0.877	1.082	1.113	1.215	1.028	1.424	1.020	1.200	H0YA99	TCOF1	Treacle protein (Fragment)
1.042	1.098	1.009										E7ETY2	TCOF1	Treacle protein
						1.044	1.032	1.087	0.841	0.983	1.146	J3KR25	TRIB3	Tribbles homolog 3
0.964	0.991	0.997	1.007	1.120	1.002	1.014	0.948	1.000	1.047	1.046	1.031	P53007	SLC25A1	Tricarboxylate transport protein, mitochondrial
0.908	1.029	1.046	0.932	0.822	0.985	1.048	0.973	1.068	1.005	0.853	1.051	Q9BT92	TCHP	Trichoplein keratin filament-binding protein
0.976	1.004	0.985	0.996	1.009	1.008	1.004	0.990	0.995	1.003	0.993	0.988	P40939	HADHA	Trifunctional enzyme subunit alpha, mitochondrial
0.995	0.987	0.976	0.990	0.974	0.998	0.985	1.004	0.979	0.974	0.962	0.954	P55084	HADHB	Trifunctional enzyme subunit beta, mitochondrial
1.005	0.985	1.023	0.988	1.052	1.003	0.972	0.966	1.042	0.948	1.008	0.995	P22102	GART	Trifunctional purine biosynthetic protein adenosine-3
0.892	0.974	0.881	1.095	1.048	1.094	1.046	1.169	1.015	1.021	1.155	0.978	Q9NVV0	TMEM38B	Trimeric intracellular cation channel type B
1.027	0.918	0.879	1.044	0.869	0.970	1.001	1.110	0.972	1.187	0.894	0.959	Q96RS0	TGS1	Trimethylguanosine synthase
									1.078	0.925	1.027	H9KVB4	TNRC18	Trinucleotide repeat-containing gene 18 protein
1.019	1.064	1.015	1.013	0.936	1.027	1.020	1.062	1.023	1.000	0.984	1.103	Q8NDV7	TNRC6A	Trinucleotide repeat-containing gene 6A protein
0.987	1.047	1.026	0.987	1.011	1.030	1.003	0.867	1.026	1.094	1.054	1.118	Q9UPQ9	TNRC6B	Trinucleotide repeat-containing gene 6B protein
1.198	1.026	0.940	0.843	0.876	0.890	1.058	1.165	0.826	1.268	1.028	1.026	A0A1B0GU24	TNRC6C	Trinucleotide repeat-containing gene 6C protein
1.025	0.995	1.014	0.997	0.962	0.989	0.995	1.003	0.980	1.044	1.014	1.010	Q9H2D6	TRIOBP	TRIO and F-actin-binding protein
0.983	0.995	1.025	0.979	0.976	1.016	0.986	1.011	1.022	1.031	1.014	1.026	Q3LXA3	TKFC	Triokinase/FMN cyclase
1.143	0.986	1.004	1.042	1.180	0.955	0.996	0.954	1.026	0.967	0.980	0.934	P60174	TPI1	Triosephosphate isomerase
1.034	0.947	1.012	0.912	0.904	1.069	1.030	1.154	1.098	0.941	0.722	0.888	Q14142	TRIM14	Tripartite motif-containing protein 14
1.043	1.062	1.120	0.939	1.034	1.110	1.118	1.010	0.895				O95361	TRIM16	Tripartite motif-containing protein 16
1.026	0.944	1.028	0.999	0.860	0.948	0.963	1.028	1.022	1.023	0.951	1.115	Q12899	TRIM26	Tripartite motif-containing protein 26
0.985	0.994	0.973	0.958	0.931	0.990	1.045	1.083	0.967	0.998	1.017	1.043	O75382	TRIM3	Tripartite motif-containing protein 3
			1.006	1.069	0.906							Q96DX7	TRIM44	Tripartite motif-containing protein 44
1.023	1.003	1.011	1.026	0.951	1.027	1.011	0.952	1.014	1.059	0.970	1.084	Q96LD4	TRIM47	Tripartite motif-containing protein 47
1.056	1.065	1.021	1.070	0.959	1.056	1.166	0.871	1.018	1.203	1.105	1.063	Q9C035	TRIM5	Tripartite motif-containing protein 5
0.985	1.012	1.050	0.991	0.997	1.050	0.926	0.902	1.025	1.105	1.018	1.099	Q6PJ69	TRIM65	Tripartite motif-containing protein 65
1.018	0.996	1.023	0.960	0.990	0.986	0.939	0.929	0.969	0.983	1.032	0.978	O14773	TPP1	Tripeptidyl-peptidase 1
1.033	1.014	1.004	1.014	0.983	0.983	1.010	1.068	0.999	1.023	0.996	1.001	P29144	TPP2	Tripeptidyl-peptidase 2
0.962	0.989	1.004	0.959	0.959	0.997	1.012	0.969	1.002	0.987	0.976	1.044	O75962	TRIO	Triple functional domain protein
									0.988	1.122	0.970	Q629K1	TRIQK	Triple QxxK/R motif-containing protein

Abundance Ratio														
1 d M1/C1	1 d M2/C2	1 d M3/C3	4 d M1/C1	4 d M2/C2	4 d M3/C3	7 d M1/C1	7 d M2/C2	7 d M3/C3	10 d M1/C1	10 d M2/C2	10 d M3/C3	Accession ID	Gene name	Protein Description
1.048	1.031	0.994	1.041	1.004	1.002	1.019	1.050	0.977	1.023	1.025	1.008	Q7Z2T5	TRMT1L	TRMT1-like protein
1.073	1.004	1.034	1.006	0.981	1.007	0.998	0.980	1.021	1.038	1.065	0.994	Q96FX7	TRMT61A	tRNA (adenine(58)-N(1))-methyltransferase catalytic subunit TRMT61A
0.993	1.007	1.024	1.017	0.942	1.015	0.988	1.032	1.005	1.020	1.071	1.034	Q9UJA5	TRMT6	tRNA (adenine(58)-N(1))-methyltransferase non-catalytic subunit TRM6
1.064	1.015	1.105	0.972	0.945	1.009	1.051	0.984	1.114	1.039	1.051	1.056	Q9BVS5	TRMT61B	tRNA (adenine(58)-N(1))-methyltransferase, mitochondrial
0.998	1.003	0.989	0.996	1.003	0.967	1.011	1.030	0.992	0.987	0.997	1.010	Q08J23	NSUN2	tRNA (cytosine(34)-C(5))-methyltransferase
0.852	0.978	1.081							0.857	0.979	1.417	Q9H649	NSUN3	tRNA (cytosine(34)-C(5))-methyltransferase, mitochondrial
0.993	0.978	1.008	1.027	0.958	1.062	0.995	1.127	1.009	1.003	1.039	1.006	Q7Z4G4	TRMT11	tRNA (guanine(10)-N2)-methyltransferase homolog
0.942	0.985	1.006	0.948	0.982	1.057	1.022	0.956	0.999	1.034	0.993	1.109	Q9NXH9	TRMT1	tRNA (guanine(26)-N(2))-dimethyltransferase
1.069	0.986	1.004	1.055	0.964	1.030	1.066	1.062	1.016	1.048	1.000	1.041	Q32P41	TRMT5	tRNA (guanine(37)-N1)-methyltransferase
1.019	0.987	1.037	0.995	0.972	0.996	1.024	1.057	1.027	1.033	0.992	1.022	P57081	WDR4	tRNA (guanine-N(7)-)-methyltransferase non-catalytic subunit WDR4
1.102	1.047	1.026	1.040	1.018	1.029	0.965	1.065	1.001	1.032	1.147	1.052	Q9UBP6	METTL1	tRNA (guanine-N(7)-)-methyltransferase
			0.845	0.929	0.792							Q96GJ1	TRMT2B	tRNA (uracil(54)-C(5))-methyltransferase homolog
0.975	0.992	1.054	0.991	0.981	1.042	1.021	1.141	1.038	1.033	1.037	1.041	F2Z2W7	TRMT2A	tRNA (uracil-5-)-methyltransferase homolog A
1.040	1.023	1.084	0.956	0.932	1.065	0.942	0.948	1.040	1.053	1.014	1.086	Q86TN4	TRPT1	tRNA 2'-phosphotransferase 1
1.049	0.984	1.012	0.980	0.969	1.007	0.935	0.913	1.006	0.998	0.985	1.030	Q9H3H1	TRIT1	tRNA dimethylallyltransferase, mitochondrial
1.017	0.990	1.031	0.984	0.976	1.035	1.000	0.966	1.005	1.021	0.979	1.016	Q9Y606	PUS1	tRNA pseudouridine synthase A, mitochondrial
1.031	1.065	1.026	1.023	0.929	0.982	1.040	1.076	1.074	0.976	0.904	1.102	Q8N0Z8	PUSL1	tRNA pseudouridine synthase-like 1
1.000	0.967	0.930										Q9BZE2	PUS3	tRNA pseudouridine(38/39) synthase
1.091	1.018	1.031	1.060	0.984	1.022	1.019	1.019	0.940	0.966	0.963	1.025	Q9NX07	TRNAU1AP	tRNA selenocysteine 1-associated protein 1
0.953	0.971	1.031	0.963	0.937	1.035	1.106	1.023	0.965	1.008	0.911	0.974	Q6IPR3	TYW3	tRNA wybutosine-synthesizing protein 3 homolog
			1.016	1.044	0.992							A2RUC4	TYW5	tRNA wybutosine-synthesizing protein 5
1.104	0.940	0.956	0.974	0.929	0.994	1.100	1.064	1.275	1.229	1.085	1.128	Q9NUP7	TRMT13	tRNA:m(4)X modification enzyme TRM13 homolog
0.982	0.975	1.031	1.092	0.967	1.056	1.043	1.112	1.079	1.026	1.096	1.070	Q6P1R4	DUS1L	tRNA-dihydrouridine(16/17) synthase [NAD(P)(+)]-like
1.014	1.005	1.048	0.994	0.953	1.027	1.011	0.978	1.031	1.001	1.006	1.098	Q9NX74	DUS2	tRNA-dihydrouridine(20) synthase [NAD(P)+]-like
1.043	1.030	1.002	1.009	0.969	1.035	1.062	1.076	0.968	1.001	1.027	1.022	Q96G46	DUS3L	tRNA-dihydrouridine(47) synthase [NAD(P)(+)]-like
1.027	1.036	0.944	1.030	0.835	1.052	0.952	0.916	1.098				Q9BUB4	ADAT1	tRNA-specific adenosine deaminase 1
0.936	0.959	1.061	0.841	1.040	1.072	0.845	0.755	0.958	1.034	1.015	1.065	Q7Z6V5	ADAT2	tRNA-specific adenosine deaminase 2
0.820	0.780	0.833	0.910	1.126	0.961	1.068	0.889	1.052	0.963	1.029	1.200	Q8WW01	TSEN15	tRNA-splicing endonuclease subunit Sen15
1.212	1.043	0.959	1.003	0.938	0.959	0.911	1.122	1.072	0.999	1.008	1.027	Q8NCE0	TSEN2	tRNA-splicing endonuclease subunit Sen2

Abundance Ratio														
1 d M1/C1	1 d M2/C2	1 d M3/C3	4 d M1/C1	4 d M2/C2	4 d M3/C3	7 d M1/C1	7 d M2/C2	7 d M3/C3	10 d M1/C1	10 d M2/C2	10 d M3/C3	Accession ID	Gene name	Protein Description
1.062	1.029	1.016	1.003	0.919	1.036	1.056	1.075	1.034	1.024	0.965	1.044	Q9BSV6	TSEN34	tRNA-splicing endonuclease subunit Sen34
0.961	0.970	0.958	1.014	0.919	1.046	0.902	1.046	1.009	0.963	1.068	1.048	Q7Z6J9	TSEN54	tRNA-splicing endonuclease subunit Sen54
0.940	1.003	0.965	0.996	0.981	0.983	0.982	1.027	0.997	0.986	1.017	0.985	Q9Y3I0	RTCB	tRNA-splicing ligase RtcB homolog
1.005	1.018	1.023	1.034	0.882	1.024	0.979	1.011	1.018	1.017	0.982	1.062	Q13641	TPBG	Trophoblast glycoprotein
0.980	0.967	1.002	1.058	0.918	0.983	0.915	0.982	1.021	1.024	0.922	1.088	P28289	TMOD1	Tropomodulin-1
1.034	0.958	0.835	0.851	0.953	1.136	1.115	0.957	1.063	1.009	0.955	1.036	Q9NZR1	TMOD2	Tropomodulin-2
1.008	1.006	0.993	0.978	0.968	1.004	0.968	0.932	0.981	0.958	0.995	0.998	Q9NYL9	TMOD3	Tropomodulin-3
1.007	1.009	1.024	0.984	0.953	0.997	1.001	0.940	0.999	0.960	0.965	0.973	Q6ZN40	TPM1	Tropomyosin 1 (Alpha), isoform CRA_f
1.037	0.979	1.029	1.007	0.953	0.989	0.944	0.875	1.005	1.013	1.013	1.010	H0YK48	TPM1	Tropomyosin alpha-1 chain
1.033	0.976	1.025	0.964	0.995	1.000	1.023	0.838	0.954	1.012	1.007	1.019	J3KN67	TPM3	Tropomyosin alpha-3 chain
1.009	1.005	1.026	0.982	0.936	0.957	0.944	0.920	0.971	0.943	0.981	0.980	P67936	TPM4	Tropomyosin alpha-4 chain
0.992	0.992	0.993	0.964	0.973	0.987	0.958	0.962	0.981	0.928	0.978	0.917	Q5TCU3	TPM2	Tropomyosin beta chain
0.992	1.014	1.014	1.012	0.919	0.991	0.988	1.090	1.002	0.968	0.985	1.021	Q9Y4C2	TCAF1	TRPM8 channel-associated factor 1
0.989	1.010	1.001	1.029	1.072	1.020	1.007	0.982	1.033	0.985	0.976	0.985	P23381	WARS	Tryptophan--tRNA ligase, cytoplasmic
1.006	1.010	1.046	0.968	0.965	1.027	0.986	1.015	0.999	1.006	1.021	1.036	Q9UGM6	WARS2	Tryptophan--tRNA ligase, mitochondrial
0.856	1.022	1.015	0.906	1.079	0.975	0.904	0.956	0.990	0.922	0.927	1.072	Q15714	TSC22D1	TSC22 domain family protein 1
1.010	1.201	0.741	1.022	0.865	0.971	0.991	0.967	1.000	1.221	0.837	1.000	O75157	TSC22D2	TSC22 domain family protein 2
0.998	1.012	0.993	0.961	0.953	0.975	0.987	1.031	1.014	1.043	0.965	1.037	Q9Y3Q8	TSC22D4	TSC22 domain family protein 4
1.081	1.109	1.077	1.054	0.836	1.057	1.051	1.014	1.035	1.056	0.911	1.087	Q8WUA8	TSKU	Tsukushin
0.996	1.013	1.042	1.003	0.996	1.015	1.044	1.064	1.052	1.005	1.027	1.026	P49815	TSC2	Tuberin
1.048	0.915	1.189				0.968	0.736	1.453	0.939	0.881	1.159	F8VQQ4	TUBA1A	Tubulin alpha chain (Fragment)
0.946	0.911	0.957	0.869	1.062	1.350	1.109	0.957	1.192	1.275	0.900	1.147	C9JJQ8	TUBA4A	Tubulin alpha chain (Fragment)
1.009	1.015	0.977	1.001	1.031	0.983	1.024	1.051	1.022	0.978	0.957	0.957	F5H5D3	TUBA1C	Tubulin alpha chain
0.903	0.981	0.952	0.989	0.960	1.004	1.064	1.118	0.992	1.015	0.997	1.133	A6NHL2	TUBAL3	Tubulin alpha chain-like 3
1.104	1.009	1.049	1.020	1.204	0.933	1.000	1.013	1.030	0.952	1.005	0.979	Q71U36	TUBA1A	Tubulin alpha-1A chain
1.186	0.998	1.048	1.056	1.604	0.970	1.105	0.890	1.191	0.981	0.925	0.994	P68366	TUBA4A	Tubulin alpha-4A chain
1.562	1.023	1.082	1.068	0.938	1.063	1.107	0.942	1.157	1.177	0.999	1.175	G3V2A3	TUBB3	Tubulin beta chain (Fragment)
						0.972	1.159	1.056	0.898	0.893	1.179	K7ES63	TUBB6	Tubulin beta chain (Fragment)
0.960	1.010	0.988	1.007	0.973	0.993	0.976	1.064	0.983	0.959	0.974	0.967	P07437	TUBB	Tubulin beta chain
0.991	1.004	0.972	1.058	0.944	0.891	1.040	1.029	0.889	1.051	0.975	0.960	Q13885	TUBB2A	Tubulin beta-2A chain
0.875	1.030	0.940	0.986	0.987	1.003	0.958	1.060	1.022	0.956	1.000	1.010	Q9BVA1	TUBB2B	Tubulin beta-2B chain
0.967	1.020	0.989	1.006	0.995	1.011	0.989	1.060	0.999	0.961	1.005	1.000	Q13509	TUBB3	Tubulin beta-3 chain
0.960	1.008	1.076	1.056	0.899	1.058	1.049	1.203	0.994	1.082	0.945	1.177	M0QYM7	TUBB4A	Tubulin beta-4A chain (Fragment)
0.978	1.003	0.952	0.967	0.951	0.988	1.018	0.989	0.945	1.016	0.946	1.010	P04350	TUBB4A	Tubulin beta-4A chain

Abundance Ratio												Accession ID	Gene name	Protein Description
1 d M1/C1	1 d M2/C2	1 d M3/C3	4 d M1/C1	4 d M2/C2	4 d M3/C3	7 d M1/C1	7 d M2/C2	7 d M3/C3	10 d M1/C1	10 d M2/C2	10 d M3/C3			
0.988	1.016	0.982	1.033	1.084	0.976	1.002	1.015	1.021	0.952	0.997	0.974	P68371	TUBB4B	Tubulin beta-4B chain
0.950	1.008	0.969	1.047	0.994	0.986	1.020	1.038	1.017	1.020	0.959	1.002	Q9BUF5	TUBB6	Tubulin beta-6 chain
									0.957	1.091	0.958	Q9UJT0	TUBE1	Tubulin epsilon chain
0.977	0.998	1.004	1.115	0.905	1.048	1.056	0.959	0.987	1.031	0.982	1.001	P23258	TUBG1	Tubulin gamma-1 chain
0.964	1.233	0.811				0.915	1.009	0.830	0.947	1.042	0.881	Q9NRH3	TUBG2	Tubulin gamma-2 chain
1.022	0.969	1.013	0.961	1.043	1.001	0.980	1.045	1.012	1.036	0.970	1.074	O94811	TPPP	Tubulin polymerization-promoting protein
1.007	0.995	0.999	0.981	1.006	1.018	1.014	0.966	1.035	1.042	1.056	1.019	Q99426	TBCB	Tubulin-folding cofactor B
1.002	0.991	0.963	1.005	0.986	1.017	1.028	1.035	0.974	1.071	1.047	0.995	E5RJD8	TBCA	Tubulin-specific chaperone A
1.115	0.962	1.037	1.009	0.949	1.035	1.099	0.864	1.021	1.007	0.975	0.963	Q15814	TBCC	Tubulin-specific chaperone C
0.963	0.993	1.000	0.949	0.998	1.052	1.016	1.075	1.129	0.818	0.853	1.025	Q5QJ74	TBCEL	Tubulin-specific chaperone cofactor E-like protein
1.000	1.026	0.996	0.994	0.990	1.048	1.006	1.079	1.070	1.003	1.016	1.053	J3KR97	TBCD	Tubulin-specific chaperone D
0.973	1.007	1.015	0.993	1.023	1.011	0.997	1.018	1.056	0.967	0.984	1.033	Q15813	TBCE	Tubulin-specific chaperone E
1.052	1.047	1.049	0.981	0.956	1.031	1.036	1.023	1.009	0.989	1.024	1.010	Q8NG68	TTL	Tubulin--tyrosine ligase
1.005	0.998	1.005	0.996	0.980	0.986	1.000	1.058	1.048	1.004	1.005	1.016	Q14166	TTLL12	Tubulin--tyrosine ligase-like protein 12
1.033	0.902	1.010	1.022	1.013	1.007	1.110	1.025	1.041	1.115	1.100	1.063	F6TB26	TDRKH	Tudor and KH domain-containing protein
0.997	1.016	1.017	0.963	0.961	0.958	1.019	1.075	1.015	0.978	0.929	1.055	Q8NHU6	TDRD7	Tudor domain-containing protein 7
1.023	1.025	1.004	1.008	0.954	1.045	1.050	1.119	1.075	1.017	0.974	1.052	Q9BRJ7	NUDT16L1	Tudor-interacting repair regulator protein
1.016	1.017	0.992	0.988	1.000	1.188	1.175	1.206	1.280				Q9NNX1	TUFT1	Tuftelin
1.047	1.001	1.056	1.015	0.981	1.033	0.998	0.982	0.994	1.045	0.977	1.010	Q9UBB9	TFIP11	Tuftelin-interacting protein 11
			1.091	0.897	0.867	0.936	1.142	1.034				H0YKR7	TNFAIP2	Tumor necrosis factor alpha-induced protein 2 (Fragment)
1.031	1.063	1.068	1.023	1.060	1.030	1.113	1.129	1.044	1.000	0.949	1.091	Q03169	TNFAIP2	Tumor necrosis factor alpha-induced protein 2
0.985	1.030	0.985	0.949	1.013	0.999	0.976	0.869	0.963	1.019	1.001	1.066	P21580	TNFAIP3	Tumor necrosis factor alpha-induced protein 3
			0.968	0.990	1.028	0.968	1.056	0.961	0.863	1.021	0.952	P41273	TNFSF9	Tumor necrosis factor ligand superfamily member 9
			1.089	0.840	1.033	1.158	1.053	1.126	1.234	1.037	1.002	O00220	TNFRSF10A	Tumor necrosis factor receptor superfamily member 10A
			0.985	1.001	1.178	1.004	1.022	1.061	1.040	1.042	1.040	O14763	TNFRSF10B	Tumor necrosis factor receptor superfamily member 10B
1.076	1.184	0.915	1.048	0.831	1.012	1.021	1.011	0.983	1.037	0.852	1.110	O00300	TNFRSF11B	Tumor necrosis factor receptor superfamily member 11B
						1.081	1.134	0.906	1.222	0.894	1.190	Q9NP84	TNFRSF12A	Tumor necrosis factor receptor superfamily member 12A
			1.049	1.836	1.030	1.278	0.586	1.119	1.009	0.955	1.123	P25942	CD40	Tumor necrosis factor receptor superfamily member 5

Abundance Ratio														
1 d M1/C1	1 d M2/C2	1 d M3/C3	4 d M1/C1	4 d M2/C2	4 d M3/C3	7 d M1/C1	7 d M2/C2	7 d M3/C3	10 d M1/C1	10 d M2/C2	10 d M3/C3	Accession ID	Gene name	Protein Description
1.002	1.057	1.033	1.039	1.040	1.028	1.093	1.028	1.042	1.143	1.046	1.154	P25445	FAS	Tumor necrosis factor receptor superfamily member 6
0.994	1.008	0.998	0.992	0.913	1.057	1.026	0.954	1.023	1.051	0.963	1.122	Q15628	TRADD	Tumor necrosis factor receptor type 1-associated DEATH domain protein
1.056	0.910	1.008	0.984	0.909	0.934	0.965	0.965	0.987	1.000	0.951	1.027	J3KNE7	TPD52L1	Tumor protein D53
0.760	1.307	1.038	0.885	1.043	1.066	0.962	0.615	0.922				Q8IXH6	TP53INP2	Tumor protein p53-inducible nuclear protein 2
0.991	0.969	0.976	1.005	0.970	1.024	1.034	1.140	1.066	1.032	1.016	1.017	Q5T0D9	TPRG1L	Tumor protein p63-regulated gene 1-like protein
0.919	0.964	0.897	1.017	1.065	1.280	1.244	0.868	0.964	1.156	1.077	1.216	Q8N726	CDKN2A	Tumor suppressor ARF
1.001	0.864	0.994	1.003	0.873	1.031	1.087	1.097	1.021	1.071	1.023	1.023	O75896	TUSC2	Tumor suppressor candidate 2
0.847	1.103	0.965	0.881	0.924	1.049	1.008	1.033	1.010	0.985	1.003	1.081	Q13454	TUSC3	Tumor suppressor candidate 3
0.902	0.970	1.010	1.007	0.989	1.226	1.064	1.087	0.991	1.015	1.057	1.087	Q2TAM9	TUSC1	Tumor suppressor candidate gene 1 protein
1.043	1.006	1.018	1.011	0.992	1.002	1.010	1.058	1.005	0.998	1.001	1.035	Q99816	TSG101	Tumor susceptibility gene 101 protein
1.016	0.991	0.979	0.994	1.007	0.976	1.009	1.039	1.035	1.004	0.977	1.022	A8MUM1	TSSC1	Tumor-suppressing subtransferable candidate 1
1.029	1.004	0.997	1.021	1.007	1.000	1.027	1.075	1.009	0.983	1.009	0.996	Q12792	TWF1	Twinfilin-1
0.999	0.996	0.979	1.000	0.973	0.989	0.997	1.050	0.988	0.943	0.973	0.925	Q6IBS0	TWF2	Twinfilin-2
0.899	0.989	1.123	0.993	0.783	0.933	0.983	1.082	1.046	0.959	0.886	1.032	Q96RR1	TWNK	Twinkle protein, mitochondrial
0.922	1.001	1.092				1.295	0.724	1.068	1.076	0.946	1.080	Q9GZX9	TWSG1	Twisted gastrulation protein homolog 1
0.923	0.968	1.118	1.022	0.881	1.144	1.018	0.939	1.032	1.116	1.022	1.063	Q8N4L2	TMEM55A	Type 2 phosphatidylinositol 4,5-bisphosphate 4-phosphatase
1.033	0.946	1.081	1.311	0.874	1.215	0.868	0.865	0.921	0.957	1.014	0.980	Q14642	INPP5A	Type I inositol 1,4,5-trisphosphate 5-phosphatase
1.078	1.008	1.092	0.977	0.915	0.988	1.062	1.202	0.981	1.020	0.981	1.061	Q96PE3	INPP4A	Type I inositol 3,4-bisphosphate 4-phosphatase
1.044	0.956	1.027	1.003	0.915	1.044	1.026	0.980	0.990	0.992	1.031	1.048	P32019	INPP5B	Type II inositol 1,4,5-trisphosphate 5-phosphatase
									1.100	0.975	1.151	Q6RW13	AGTRAP	Type-1 angiotensin II receptor-associated protein
1.000	0.988	1.003	0.997	0.984	1.007	1.020	1.013	1.016	1.014	1.004	1.051	Q9UIG0	BAZ1B	Tyrosine-protein kinase BAZ1B
0.948	0.964	0.997	0.963	0.935	1.029	0.969	1.028	1.030	0.951	0.912	1.054	P41240	CSK	Tyrosine-protein kinase CSK
1.020	1.015	1.016	0.972	0.864	1.075	1.075	1.076	0.991	0.977	0.979	1.094	P16591	FER	Tyrosine-protein kinase Fer
1.002	1.029	1.045	1.027	1.033	1.058	1.075	1.004	1.061	1.069	0.979	1.186	P23458	JAK1	Tyrosine-protein kinase JAK1
1.004	1.000	1.012	1.017	0.944	1.015	1.087	1.107	1.003	1.016	1.018	1.000	P07948	LYN	Tyrosine-protein kinase Lyn
1.000	1.086	1.104	0.996	0.964	1.033	1.026	1.053	1.013	1.132	1.091	1.037	J3QRU1	YES1	Tyrosine-protein kinase
1.053	1.037	0.981	1.004	0.932	0.981	1.021	1.093	1.062	0.997	0.968	1.090	P30530	AXL	Tyrosine-protein kinase receptor UFO
1.029	1.111	1.060	1.013	0.877	1.019	1.035	1.082	1.002	0.981	0.999	0.989	Q86YV5	SGK223	Tyrosine-protein kinase SgK223
0.961	0.977	1.018	0.988	0.959	1.001	1.005	0.952	0.981	1.020	0.986	1.041	P18031	PTPN1	Tyrosine-protein phosphatase non-receptor type 1

Abundance Ratio														
1 d M1/C1	1 d M2/C2	1 d M3/C3	4 d M1/C1	4 d M2/C2	4 d M3/C3	7 d M1/C1	7 d M2/C2	7 d M3/C3	10 d M1/C1	10 d M2/C2	10 d M3/C3	Accession ID	Gene name	Protein Description
0.932	0.963	0.950	0.954	0.992	1.100	0.866	1.021	1.111	1.020	0.958	1.149	Q06124	PTPN11	Tyrosine-protein phosphatase non-receptor type 11
1.024	1.016	1.027	1.016	1.008	1.011	1.042	1.000	1.021	1.030	0.992	1.083	Q05209	PTPN12	Tyrosine-protein phosphatase non-receptor type 12
0.991	1.032	0.978	1.022	0.999	0.981	0.998	1.017	1.014	1.042	0.966	1.028	Q15678	PTPN14	Tyrosine-protein phosphatase non-receptor type 14
1.095	1.259	1.002				1.105	0.810	0.893				Q99952	PTPN18	Tyrosine-protein phosphatase non-receptor type 18
1.010	1.026	1.015	1.007	0.983	1.017	1.034	1.068	1.055	0.967	0.985	1.029	P17706	PTPN2	Tyrosine-protein phosphatase non-receptor type 2
1.000	0.992	1.007	1.004	0.994	1.043	0.995	1.036	0.992	1.006	0.990	1.014	Q9H3S7	PTPN23	Tyrosine-protein phosphatase non-receptor type 23
1.052	0.986	0.970	1.000	0.865	1.046	1.041	1.131	1.004	1.117	0.985	1.054	P26045	PTPN3	Tyrosine-protein phosphatase non-receptor type 3
0.973	1.077	1.005	1.008	0.921	0.961	1.088	0.901	0.924	1.039	1.032	0.931	P43378	PTPN9	Tyrosine-protein phosphatase non-receptor type 9
1.012	1.023	1.016	1.040	0.992	1.046	1.074	1.005	1.012	1.090	1.032	1.110	P78324	SIRPA	Tyrosine-protein phosphatase non-receptor type substrate 1
0.983	1.002	0.998	1.003	1.035	1.001	0.966	0.994	1.008	0.958	1.009	0.950	P54577	YARS	Tyrosine--tRNA ligase, cytoplasmic
0.986	0.987	1.001	0.982	0.980	1.004	0.990	1.050	0.986	1.000	0.959	0.984	Q9Y2Z4	YARS2	Tyrosine--tRNA ligase, mitochondrial
1.070	1.003	0.961	0.998	0.960	0.994	0.994	1.120	1.013	1.037	1.003	1.033	Q9NUW8	TDP1	Tyrosyl-DNA phosphodiesterase 1
1.172	0.985	0.993	0.960	0.945	0.961							P08621	SNRNP70	U1 small nuclear ribonucleoprotein 70 kDa
0.984	0.989	1.011	0.958	0.972	0.976	0.932	0.889	0.957	0.955	1.021	0.968	P09012	SNRPA	U1 small nuclear ribonucleoprotein A
0.952	0.973	0.933	0.961	1.005	0.979	0.958	0.966	0.964	1.014	0.996	0.972	A0A0A0MRR7	SNRPC	U1 small nuclear ribonucleoprotein C
			0.950	1.019	1.143				0.977	0.870	0.978	Q6IEG0	SNRNP48	U11/U12 small nuclear ribonucleoprotein 48 kDa protein
1.015	0.991	1.016	0.982	0.967	1.024	0.950	0.997	0.987	0.978	1.012	0.981	P09661	SNRPA1	U2 small nuclear ribonucleoprotein A'
0.981	0.966	0.969	1.005	1.007	1.029	1.000	0.937	0.959	1.052	1.048	1.042	P08579	SNRPB2	U2 small nuclear ribonucleoprotein B''
1.040	1.012	1.034	1.012	0.981	1.002	0.996	1.017	1.002	0.979	0.974	0.997	O15042	U2SURP	U2 snRNP-associated SURP motif-containing protein
1.008	0.978	1.004	0.982	0.938	1.002	0.963	1.048	1.041	0.967	1.027	1.000	Q9NV31	IMP3	U3 small nucleolar ribonucleoprotein protein IMP3
1.012	1.044	1.072	0.996	1.086	1.180	0.989	1.040	1.065	1.008	1.056	1.278	Q96G21	IMP4	U3 small nucleolar ribonucleoprotein protein IMP4
0.996	0.996	0.968	1.013	0.962	0.995	1.055	1.062	1.013	1.042	0.984	1.002	O00566	MPHOSPH10	U3 small nucleolar ribonucleoprotein protein MPP10
1.007	0.984	1.001	0.995	0.956	1.025	0.996	1.031	1.024	1.016	1.025	1.021	Q9BVJ6	UTP14A	U3 small nucleolar RNA-associated protein 14 homolog A

Abundance Ratio												Accession ID	Gene name	Protein Description
1 d M1/C1	1 d M2/C2	1 d M3/C3	4 d M1/C1	4 d M2/C2	4 d M3/C3	7 d M1/C1	7 d M2/C2	7 d M3/C3	10 d M1/C1	10 d M2/C2	10 d M3/C3			
1.068	1.003	1.029	1.022	0.949	0.969	1.029	1.077	1.029	0.981	1.022	1.016	Q8TED0	UTP15	U3 small nucleolar RNA-associated protein 15 homolog
0.990	1.032	0.994	1.009	0.958	1.009	0.996	1.026	1.013	1.017	1.045	1.016	Q9Y5J1	UTP18	U3 small nucleolar RNA-associated protein 18 homolog
1.019	1.006	1.005	0.985	0.963	1.005	1.008	1.071	1.002	1.011	1.008	0.993	Q969X6	UTP4	U3 small nucleolar RNA-associated protein 4 homolog
0.986	0.969	1.050	0.969	0.900	1.001	0.987	0.981	0.989	0.991	1.017	1.010	Q9NYH9	UTP6	U3 small nucleolar RNA-associated protein 6 homolog
1.019	1.026	1.018	1.007	1.054	1.024	1.046	0.999	1.084	0.993	1.031	1.039	O43818	RRP9	U3 small nucleolar RNA-interacting protein 2
1.025	0.996	1.052	0.984	0.978	1.057	0.996	0.971	1.038	0.994	1.033	1.038	O43395	PRPF3	U4/U6 small nuclear ribonucleoprotein Prp3
0.965	1.013	0.971	0.993	1.006	1.021	0.991	1.044	0.983	1.013	1.034	1.009	Q8WWY3	PRPF31	U4/U6 small nuclear ribonucleoprotein Prp31
0.996	1.016	1.004	0.979	0.996	1.008	1.005	1.083	1.007	1.002	0.997	1.017	O43172	PRPF4	U4/U6 small nuclear ribonucleoprotein Prp4
1.069	1.017	0.999	1.029	0.950	0.963	0.996	1.120	0.934	1.015	1.035	0.982	Q8WVK2	SNRNP27	U4/U6.U5 small nuclear ribonucleoprotein 27 kDa protein
1.011	0.998	1.010	0.977	0.974	0.988	0.997	0.995	0.991	1.002	0.997	1.011	O43290	SART1	U4/U6.U5 tri-snRNP-associated protein 1
1.016	0.986	1.024	1.005	1.001	1.009	1.006	0.994	1.026	1.035	0.981	1.027	Q53GS9	USP39	U4/U6.U5 tri-snRNP-associated protein 2
0.996	0.999	1.012	0.997	0.999	1.025	0.998	1.048	1.004	0.974	1.000	0.981	O75643	SNRNP200	U5 small nuclear ribonucleoprotein 200 kDa helicase
1.015	1.023	0.976	0.986	1.007	0.990	0.971	1.075	1.003	0.994	1.014	1.016	Q96DI7	SNRNP40	U5 small nuclear ribonucleoprotein 40 kDa protein
1.120	1.105	1.004	1.042	0.984	0.987	1.008	1.222	1.047	0.907	0.927	0.987	Q9BQ65	USB1	U6 snRNA phosphodiesterase
1.108	0.963	1.055	0.930	0.950	1.039	0.880	1.020	1.123	0.937	0.983	0.998	O15116	LSM1	U6 snRNA-associated Sm-like protein LSm1
1.030	1.006	0.994	0.992	0.971	0.950	0.937	1.073	0.994	0.908	1.023	0.914	Q9Y333	LSM2	U6 snRNA-associated Sm-like protein LSm2
1.071	0.986	1.015	1.031	0.931	0.951	0.965	1.060	1.013	0.865	0.975	0.946	P62310	LSM3	U6 snRNA-associated Sm-like protein LSm3
0.978	0.999	1.021	1.005	1.144	1.005	1.003	0.991	1.020	0.999	1.023	0.996	V9GZ56	LSM4	U6 snRNA-associated Sm-like protein LSm4 (Fragment)
0.883	0.729	1.018	1.013	0.978	1.084	0.906	0.929	1.044	0.808	0.961	1.106	Q9Y4Y9	LSM5	U6 snRNA-associated Sm-like protein LSm5
1.049	0.972	0.973	1.001	0.989	1.006	0.982	1.032	0.919	0.991	1.007	0.974	P62312	LSM6	U6 snRNA-associated Sm-like protein LSm6
0.983	0.921	0.963	0.985	1.064	1.032	1.058	0.995	1.049	0.959	0.989	0.998	Q9UK45	LSM7	U6 snRNA-associated Sm-like protein LSm7
0.997	1.003	1.034	1.006	1.022	1.107	0.980	1.073	1.057	1.030	1.069	0.990	O95777	LSM8	U6 snRNA-associated Sm-like protein LSm8
			0.917	0.724	1.134							Q969L4	LSM10	U7 snRNA-associated Sm-like protein LSm10
1.028	0.990	1.050	0.998	0.963	1.095	0.944	1.048	1.106	1.005	0.979	1.042	Q96DE0	NUDT16	U8 snoRNA-decapping enzyme
1.050	1.005	0.978	1.007	0.958	1.032	0.991	1.043	0.991	0.973	0.997	0.995	Q96QD9	FYTTD1	UAP56-interacting factor
0.999	0.942	1.018	0.933	0.948	1.054	1.013	1.168	1.057	0.979	1.021	1.047	Q9Y5Z9	UBIAD1	UbiA prenyltransferase domain-containing protein 1
1.045	0.983	1.020	0.916	0.900	0.931							Q9NPG3	UBN1	Ubinuclein-1
0.974	1.011	0.966	0.946	0.936	0.985	0.973	1.148	1.021	0.952	1.059	0.947	Q9UMX0	UBQLN1	Ubiquilin-1

Abundance Ratio														
1 d M1/C1	1 d M2/C2	1 d M3/C3	4 d M1/C1	4 d M2/C2	4 d M3/C3	7 d M1/C1	7 d M2/C2	7 d M3/C3	10 d M1/C1	10 d M2/C2	10 d M3/C3	Accession ID	Gene name	Protein Description
0.988	1.009	0.978	1.009	0.986	0.984	0.966	1.099	0.993	0.956	1.036	1.000	Q9UHD9	UBQLN2	Ubiquilin-2
1.076	1.021	0.995	0.998	0.920	0.972	1.026	1.114	1.012	0.997	1.021	1.002	Q9NRR5	UBQLN4	Ubiquilin-4
0.963	1.007	0.955	0.970	1.013	1.018	1.007	1.009	1.015	0.992	1.004	0.976	Q9NVA1	UQCC1	Ubiquinol-cytochrome-c reductase complex assembly factor 1
1.059	1.035	0.955	1.016	1.051	0.990	1.053	0.980	0.978	1.082	0.970	0.979	Q9BRT2	UQCC2	Ubiquinol-cytochrome-c reductase complex assembly factor 2
1.015	0.949	1.081	0.882	0.833	0.915	0.879	0.891	1.025	0.891	1.113	0.897	Q6UW78	UQCC3	Ubiquinol-cytochrome-c reductase complex assembly factor 3
0.966	0.930	1.093	0.877	1.013	1.020	0.921	0.996	1.039	0.892	0.925	1.079	Q9Y2Z9	COQ6	Ubiquinone biosynthesis monooxygenase COQ6, mitochondrial
0.960	0.934	0.982	0.969	0.956	1.017	1.009	1.014	1.098	0.969	0.967	1.018	Q9NZJ6	COQ3	Ubiquinone biosynthesis O-methyltransferase, mitochondrial
1.125	1.047	1.127										Q9Y3A0	COQ4	Ubiquinone biosynthesis protein COQ4 homolog, mitochondrial
1.007	0.987	1.026	0.964	0.939	1.016	0.959	0.951	0.964	0.987	0.979	1.004	O75208	COQ9	Ubiquinone biosynthesis protein COQ9, mitochondrial
			1.092	0.850	0.932							O94782	USP1	Ubiquitin carboxyl-terminal hydrolase 1
1.019	1.001	1.070	1.049	0.920	1.016	1.021	0.957	1.041	1.119	0.954	1.056	P51784	USP11	Ubiquitin carboxyl-terminal hydrolase 11
1.068	1.033	1.063	1.053	1.053	1.047	0.899	0.928	1.137	0.920	1.065	1.053	O75317	USP12	Ubiquitin carboxyl-terminal hydrolase 12
0.980	1.009	1.036	0.980	0.938	1.102	1.185	1.088	1.026	1.109	1.254	1.276	Q92995	USP13	Ubiquitin carboxyl-terminal hydrolase 13
0.997	0.987	1.004	0.999	0.984	1.025	0.987	0.979	0.959	1.010	0.999	1.003	P54578	USP14	Ubiquitin carboxyl-terminal hydrolase 14
1.004	1.006	0.994	1.008	0.987	1.019	0.995	1.061	1.019	0.983	0.992	0.982	Q9Y4E8	USP15	Ubiquitin carboxyl-terminal hydrolase 15
			0.926	0.964	1.227	0.954	1.010	1.067	1.014	0.982	0.997	Q9Y5T5	USP16	Ubiquitin carboxyl-terminal hydrolase 16
									0.988	1.465	1.291	O75604	USP2	Ubiquitin carboxyl-terminal hydrolase 2
			0.957	0.909	0.797							Q9Y2K6	USP20	Ubiquitin carboxyl-terminal hydrolase 20
0.995	0.988	1.012	1.027	1.176	1.072	1.004	0.999	1.034	1.045	0.964	1.068	Q9UPT9	USP22	Ubiquitin carboxyl-terminal hydrolase 22
0.981	1.020	1.025	1.001	0.964	1.001	1.013	1.036	1.042	1.000	1.013	1.040	Q9UPU5	USP24	Ubiquitin carboxyl-terminal hydrolase 24
1.009	0.974	1.041	0.999	1.007	1.003	0.978	1.032	0.961	1.005	1.011	1.066	Q9UHP3	USP25	Ubiquitin carboxyl-terminal hydrolase 25
1.007	0.924	1.020	0.968	0.911	1.017	0.989	0.946	1.010	0.960	0.943	0.977	Q96RU2	USP28	Ubiquitin carboxyl-terminal hydrolase 28
1.027	0.980	0.991	1.050	1.367	0.956	1.156	0.897	1.098	0.971	0.958	0.933	Q9Y6I4	USP3	Ubiquitin carboxyl-terminal hydrolase 3
0.948	0.937	0.980	1.329	0.781	1.113	0.948	1.517	1.028	1.118	1.112	1.072	Q70CQ3	USP30	Ubiquitin carboxyl-terminal hydrolase 30
			1.048	1.022	1.138	0.916	1.003	1.110	1.148	1.042	0.951	Q70CQ4	USP31	Ubiquitin carboxyl-terminal hydrolase 31
1.074	1.026	1.081	1.056	0.878	0.915	1.050	1.060	0.947	0.920	1.024	1.031	Q8NFA0	USP32	Ubiquitin carboxyl-terminal hydrolase 32
1.008	1.009	1.071	1.024	1.092	0.910	1.130	1.017	1.064				Q8TEY7	USP33	Ubiquitin carboxyl-terminal hydrolase 33
1.021	1.034	1.012	1.019	0.958	1.034	1.019	1.099	1.001	1.037	0.998	1.109	Q70CQ2	USP34	Ubiquitin carboxyl-terminal hydrolase 34
			1.104	0.967	1.157							Q9P2H5	USP35	Ubiquitin carboxyl-terminal hydrolase 35

Abundance Ratio												Accession ID	Gene name	Protein Description
1 d M1/C1	1 d M2/C2	1 d M3/C3	4 d M1/C1	4 d M2/C2	4 d M3/C3	7 d M1/C1	7 d M2/C2	7 d M3/C3	10 d M1/C1	10 d M2/C2	10 d M3/C3			
0.927	0.888	1.183				1.156	0.925	1.049	1.069	1.174	1.133	Q86T82	USP37	Ubiquitin carboxyl-terminal hydrolase 37
1.011	0.996	1.000	1.005	0.962	1.053	0.965	1.110	1.030	1.009	0.982	1.025	Q13107	USP4	Ubiquitin carboxyl-terminal hydrolase 4
1.009	1.000	1.003	0.999	0.996	0.995	0.985	1.004	1.011	1.012	0.991	1.013	P45974	USP5	Ubiquitin carboxyl-terminal hydrolase 5
0.990	1.013	1.012	1.002	0.975	1.027	1.008	1.049	0.996	0.999	0.994	1.008	Q93009	USP7	Ubiquitin carboxyl-terminal hydrolase 7
1.064	0.998	1.014	0.973	0.954	0.982	1.004	1.040	0.998	1.012	1.038	1.041	P40818	USP8	Ubiquitin carboxyl-terminal hydrolase 8
0.994	0.981	1.004	0.995	0.935	1.047	1.011	1.051	1.043	1.020	0.967	1.040	Q92560	BAP1	Ubiquitin carboxyl-terminal hydrolase BAP1
1.048	0.976	0.936	1.061	1.029	1.069	1.099	0.909	1.198	0.953	0.982	1.063	Q9NQC7	CYLD	Ubiquitin carboxyl-terminal hydrolase CYLD
1.022	0.979	1.002	1.015	0.948	1.012	0.967	1.029	0.997	1.008	0.986	1.010	P15374	UCHL3	Ubiquitin carboxyl-terminal hydrolase isozyme L3
1.007	1.058	1.088	1.032	1.002	1.017	1.090	1.067	1.018	1.049	0.988	1.098	Q9H8M7	MINDY3	Ubiquitin carboxyl-terminal hydrolase MINDY-3
1.002	1.001	1.013	1.023	0.992	1.017	0.997	1.029	0.999	0.942	0.994	0.954	Q5LJA5	UCHL5	Ubiquitin carboxyl-terminal hydrolase
0.996	1.026	1.045	1.037	0.907	1.005	0.959	1.101	1.035	0.942	1.007	1.032	Q8WUN7	UBTD2	Ubiquitin domain-containing protein 2
0.989	0.993	0.989	0.983	0.942	1.016	1.064	1.036	0.996	0.963	0.975	0.990	O14562	UBFD1	Ubiquitin domain-containing protein UBFD1
1.048	1.004	1.012	1.020	0.985	0.976	0.995	1.026	0.987	1.012	0.998	0.964	J3KR44	OTUB1	Ubiquitin thioesterase
						0.957	1.039	1.276				Q5VVQ6	YOD1	Ubiquitin thioesterase OTU1
1.034	0.963	1.008	0.838	0.982	1.163	1.211	1.404	1.269				Q96DC9	OTUB2	Ubiquitin thioesterase OTUB2
0.954	0.984	0.998	0.998	0.971	1.010	1.025	0.987	0.967	1.007	0.994	1.034	Q96BN8	OTULIN	Ubiquitin thioesterase otulin
1.018	1.042	0.971	1.012	0.924	0.957	1.004	1.063	0.961	1.001	0.958	1.060	O14933	UBE2L6	Ubiquitin/ISG15-conjugating enzyme E2 L6
1.036	0.988	0.975	1.033	1.010	1.002	1.057	0.976	0.985	1.017	0.991	0.967	P62979	RPS27A	Ubiquitin-40S ribosomal protein S27a
1.417	1.042	1.136	1.068	2.605	0.789	1.219	0.497	1.100	1.057	0.795	1.041	P62987	UBA52	Ubiquitin-60S ribosomal protein L40
1.030	1.049	1.013	1.060	1.053	0.973	1.047	0.986	1.033	1.061	1.007	1.038	Q8TF42	UBASH3B	Ubiquitin-associated and SH3 domain-containing protein B
0.973	0.953	1.026	0.976	0.986	1.067	0.989	1.097	0.993	0.905	1.004	1.040	Q9BSL1	UBAC1	Ubiquitin-associated domain-containing protein 1
1.062	1.022	0.947	1.039	0.957	0.975	1.045	1.080	1.042	1.165	1.129	1.030	Q8NBM4	UBAC2	Ubiquitin-associated domain-containing protein 2
0.956	1.006	0.993	1.002	1.000	1.032	1.027	1.084	0.997	1.056	1.050	1.101	Q5T6F2	UBAP2	Ubiquitin-associated protein 2
1.034	1.037	1.036	1.018	1.008	0.938	1.081	1.174	0.975	0.993	0.994	1.073	P49459	UBE2A	Ubiquitin-conjugating enzyme E2 A
			1.121	0.988	0.963	1.029	1.102	1.222	1.099	0.976	0.997	P63146	UBE2B	Ubiquitin-conjugating enzyme E2 B
0.961	1.009	0.975	0.998	0.893	0.985	1.058	1.128	1.054	0.919	0.897	1.048	O00762	UBE2C	Ubiquitin-conjugating enzyme E2 C
1.015	0.994	0.959	0.992	0.978	0.946	1.015	1.108	0.986	0.986	1.091	1.013	P51668	UBE2D1	Ubiquitin-conjugating enzyme E2 D1
1.032	1.004	0.903	0.989	1.020	0.954	0.996	1.108	0.973	0.969	1.004	0.972	A0A0A0MQU3	UBE2D2	Ubiquitin-conjugating enzyme E2 D2
1.050	1.018	1.002	1.021	0.945	1.015	1.042	1.033	0.998	1.024	0.946	0.978	P51965	UBE2E1	Ubiquitin-conjugating enzyme E2 E1
0.553	0.978	1.413	0.829	1.047	1.073	1.086	0.970	0.984	1.490	1.080	1.039	Q96LR5	UBE2E2	Ubiquitin-conjugating enzyme E2 E2
0.927	0.910	1.074	0.781	1.339	1.079	0.852	0.467	0.898	1.250	1.026	1.149	Q969T4	UBE2E3	Ubiquitin-conjugating enzyme E2 E3

Abundance Ratio														
1 d M1/C1	1 d M2/C2	1 d M3/C3	4 d M1/C1	4 d M2/C2	4 d M3/C3	7 d M1/C1	7 d M2/C2	7 d M3/C3	10 d M1/C1	10 d M2/C2	10 d M3/C3	Accession ID	Gene name	Protein Description
1.012	0.999	1.001	1.026	1.004	1.034	1.012	1.051	1.020	1.018	0.973	0.982	P62253	UBE2G1	Ubiquitin-conjugating enzyme E2 G1
1.035	0.988	1.073	0.978	0.986	1.016	0.979	1.077	0.949	0.977	0.955	1.065	P60604	UBE2G2	Ubiquitin-conjugating enzyme E2 G2
1.038	0.978	0.970	1.032	0.937	1.002	1.026	1.111	1.033	1.072	1.007	1.160	P62256	UBE2H	Ubiquitin-conjugating enzyme E2 H
0.985	0.964	1.041	1.001	0.863	0.976	0.923	0.710	0.944	1.007	0.949	1.175	Q9Y385	UBE2J1	Ubiquitin-conjugating enzyme E2 J1
1.075	0.774	0.988	0.987	1.109	1.081				1.088	1.012	1.022	Q8N2K1	UBE2J2	Ubiquitin-conjugating enzyme E2 J2
1.016	0.974	0.994	1.011	0.990	0.999	1.009	1.073	1.002	0.970	0.981	1.019	P61086	UBE2K	Ubiquitin-conjugating enzyme E2 K
1.048	1.012	0.972	1.012	0.999	0.985	0.992	1.091	1.010	1.005	1.012	0.978	P68036	UBE2L3	Ubiquitin-conjugating enzyme E2 L3
1.012	1.010	1.015	1.003	1.070	0.997	1.015	0.986	1.048	0.983	1.022	0.984	P61088	UBE2N	Ubiquitin-conjugating enzyme E2 N
1.007	0.996	1.012	1.011	0.982	1.093	0.997	0.978	1.032	1.007	1.057	1.056	Q7Z7E8	UBE2Q1	Ubiquitin-conjugating enzyme E2 Q1
1.008	1.016	1.004	1.008	0.949	1.041	1.028	1.097	1.025	0.984	0.988	1.058	P49427	CDC34	Ubiquitin-conjugating enzyme E2 R1
0.998	0.981	0.981	1.001	0.923	0.952	0.976	1.170	0.948	1.018	0.978	1.026	Q712K3	UBE2R2	Ubiquitin-conjugating enzyme E2 R2
0.958	1.064	1.008	1.118	1.000	1.069	1.082	1.159	1.073	0.907	0.970	1.064	Q16763	UBE2S	Ubiquitin-conjugating enzyme E2 S
1.044	0.938	1.050	0.975	1.041	0.970	1.090	0.977	1.008				Q9NPD8	UBE2T	Ubiquitin-conjugating enzyme E2 T
1.031	0.969	1.027	1.012	0.970	0.994	1.053	1.014	1.019	1.051	1.004	1.004	Q15819	UBE2V2	Ubiquitin-conjugating enzyme E2 variant 2
1.056	1.015	1.010	0.985	0.927	0.945	1.029	1.169	1.016	0.979	0.965	1.061	Q8IX04	UEVLD	Ubiquitin-conjugating enzyme E2 variant 3
1.013	1.020	0.998	1.011	1.132	0.947	1.069	1.017	1.036	0.957	0.994	0.985	Q9H832	UBE2Z	Ubiquitin-conjugating enzyme E2 Z
1.015	0.992	1.012	0.970	0.993	0.982	0.914	1.006	0.988	0.926	1.017	0.940	P61960	UFM1	Ubiquitin-fold modifier 1
1.014	0.981	1.003	1.009	1.064	1.034	1.074	1.008	1.040	1.018	1.026	1.019	Q9Y3C8	UFC1	Ubiquitin-fold modifier-conjugating enzyme 1
1.004	0.999	1.004	1.005	0.951	1.024	1.034	1.016	1.031	1.017	1.019	1.022	Q8WVY7	UBLCP1	Ubiquitin-like domain-containing CTD phosphatase 1
									1.243	1.084	1.288	Q5JRS2	UBA1	Ubiquitin-like modifier-activating enzyme 1 (Fragment)
0.973	0.964	0.971	1.047	0.934	1.110	1.004	0.901	1.103	1.043	1.004	1.053	Q5JRS3	UBA1	Ubiquitin-like modifier-activating enzyme 1 (Fragment)
1.008	1.008	0.998	0.994	1.035	0.988	0.972	1.015	1.007	0.956	0.984	0.954	P22314	UBA1	Ubiquitin-like modifier-activating enzyme 1
1.051	0.997	1.046	1.027	0.930	1.014	0.974	1.037	1.013	1.003	1.043	0.996	Q9GZZ9	UBA5	Ubiquitin-like modifier-activating enzyme 5
1.004	1.010	0.997	1.005	1.024	1.004	0.999	1.052	1.029	0.995	1.008	1.011	A0AVT1	UBA6	Ubiquitin-like modifier-activating enzyme 6
0.947	1.006	1.145	0.986	1.020	1.082				1.028	0.935	1.088	P41226	UBA7	Ubiquitin-like modifier-activating enzyme 7
0.997	1.002	1.022	1.023	1.041	1.074	1.003	1.095	1.054	0.967	0.986	1.008	O95352	ATG7	Ubiquitin-like modifier-activating enzyme ATG7
						1.002	1.241	1.000				O95164	UBL3	Ubiquitin-like protein 3
1.019	1.014	1.042	1.013	0.972	1.002	1.059	1.082	1.029	0.993	1.047	1.072	P11441	UBL4A	Ubiquitin-like protein 4A
1.141	1.001	1.047	1.065	1.190	1.004	0.966	0.904	1.010	0.896	0.979	0.883	Q9BZL1	UBL5	Ubiquitin-like protein 5
1.021	0.980	1.057	0.958	0.932	0.950	1.075	1.007	0.994	1.034	1.035	0.897	Q96S82	UBL7	Ubiquitin-like protein 7
1.027	1.006	0.977	0.992	0.968	1.031	0.959	1.094	0.993	0.911	0.966	0.956	O94817	ATG12	Ubiquitin-like protein ATG12
1.016	1.039	0.972	0.994	1.003	1.012	1.026	1.080	0.961	1.004	0.978	1.050	P05161	ISG15	Ubiquitin-like protein ISG15

Abundance Ratio														
1 d M1/C1	1 d M2/C2	1 d M3/C3	4 d M1/C1	4 d M2/C2	4 d M3/C3	7 d M1/C1	7 d M2/C2	7 d M3/C3	10 d M1/C1	10 d M2/C2	10 d M3/C3	Accession ID	Gene name	Protein Description
1.011	1.107	0.988	1.101	0.858	0.971	0.957	0.878	1.025	0.808	0.735	0.953	Q9H0Y0	ATG10	Ubiquitin-like-conjugating enzyme ATG10
1.025	1.003	1.016	0.999	0.980	1.033	1.031	1.049	1.022	1.039	0.974	1.025	Q9NT62	ATG3	Ubiquitin-like-conjugating enzyme ATG3
1.008	1.002	1.010	1.031	0.980	1.004	1.008	1.005	1.008	0.968	1.004	0.998	Q05086	UBE3A	Ubiquitin-protein ligase E3A
1.094	1.029	1.097	1.169	1.028	0.997	1.111	1.067	1.005	0.970	1.016	1.041	Q7Z3V4	UBE3B	Ubiquitin-protein ligase E3B
1.001	1.014	1.032	0.995	0.986	1.027	1.005	1.061	1.048	0.961	0.976	0.989	Q15386	UBE3C	Ubiquitin-protein ligase E3C
0.990	1.004	1.022	0.976	0.993	1.056	1.017	0.992	1.022	1.044	1.045	1.046	Q04323	UBXN1	UBX domain-containing protein 1
1.042	1.177	1.026				0.901	1.290	0.997	0.898	1.115	1.231	P68543	UBXN2A	UBX domain-containing protein 2A
0.903	1.038	1.020	0.897	0.981	0.998	1.006	1.048	1.010				Q14CS0	UBXN2B	UBX domain-containing protein 2B
1.021	0.994	1.037	0.987	0.859	0.974	0.944	0.965	0.977	0.946	0.965	0.965	Q92575	UBXN4	UBX domain-containing protein 4
1.003	0.999	0.997	1.025	1.002	1.039	1.024	1.082	1.062	1.072	1.024	1.033	Q9BZV1	UBXN6	UBX domain-containing protein 6
1.008	0.968	0.988	1.009	0.979	1.036	0.997	1.071	1.064	0.941	1.007	1.017	O94888	UBXN7	UBX domain-containing protein 7
1.025	1.151	1.132	1.061	1.082	0.911	1.263	1.007	0.828	1.119	1.050	1.019	Q67FW5	B3GNTL1	UDP-GlcNAc:betaGal beta-1,3-N-acetylglucosaminyltransferase-like protein 1
0.993	0.988	0.979	1.014	1.013	1.000	1.005	0.917	0.969	0.995	0.977	1.043	Q14376	GALE	UDP-glucose 4-epimerase
1.020	1.001	1.011	1.041	1.064	1.050	1.006	1.003	1.056	1.045	1.016	0.997	O60701	UGDH	UDP-glucose 6-dehydrogenase
0.979	0.996	1.006	0.994	0.991	1.002	0.990	1.036	0.998	0.980	0.973	0.987	Q9NYU2	UGGT1	UDP-glucose:glycoprotein glucosyltransferase 1
1.023	1.013	1.087	1.017	0.989	0.958	1.004	1.085	1.085	0.958	1.001	1.057	Q9NYU1	UGGT2	UDP-glucose:glycoprotein glucosyltransferase 2
			1.426	0.651	0.657	1.206	0.821	0.545				Q9NTN3	SLC35D1	UDP-glucuronic acid/UDP-N-acetylgalactosamine transporter
0.989	1.016	1.161	0.917	0.780	1.063	1.089	0.813	1.032	1.210	1.013	1.099	Q9H3H5	DPAGT1	UDP-N-acetylglucosamine--dolichyl-phosphate N-acetylglucosaminephosphotransferase
0.979	0.987	1.000	0.987	0.968	0.994	1.025	1.031	1.007	0.990	0.941	1.009	O15294	OGT	UDP-N-acetylglucosamine--peptide N-acetylglucosaminyltransferase 110 kDa subunit
0.966	0.999	0.983	0.996	0.997	0.996	0.977	1.047	1.026	0.925	0.949	1.005	Q16222	UAP1	UDP-N-acetylhexosamine pyrophosphorylase
1.011	0.988	0.991	1.002	1.048	1.058	1.004	0.943	1.043	1.052	1.048	1.038	Q3KQV9	UAP1L1	UDP-N-acetylhexosamine pyrophosphorylase-like protein 1
1.139	1.258	1.656				1.107	1.225	0.925				Q969S0	SLC35B4	UDP-xylose and UDP-N-acetylglucosamine transporter
1.008	0.959	1.016	0.989	0.991	1.026	0.983	1.060	1.030	0.955	0.977	0.958	Q9NUQ7	UFSP2	Ufm1-specific protease 2
0.961	0.975	1.064	0.938	0.945	0.968	1.041	1.043	0.999	1.061	0.995	1.071	Q6BDS2	UHRF1BP1	UHRF1-binding protein 1
0.916	0.842	1.145	1.068	0.959	1.019	1.123	1.110	1.060	1.109	1.038	1.020	A0JNW5	UHRF1BP1L	UHRF1-binding protein 1-like
1.003	1.020	0.994	1.023	0.988	1.029	0.957	1.015	0.972	1.026	1.016	1.011	P30085	CMPK1	UMP-CMP kinase
									0.962	1.042	1.041	Q96GV9	C5orf30	UNC119-binding protein C5orf30
			0.969	0.769	1.214	0.971	1.007	0.952				O43934	MFSD11	UNC93-like protein MFSD11
						0.983	0.917	1.122				Q86TW2	ADCK1	Uncharacterized aarF domain-containing protein kinase 1

Abundance Ratio												Accession ID	Gene name	Protein Description
1 d M1/C1	1 d M2/C2	1 d M3/C3	4 d M1/C1	4 d M2/C2	4 d M3/C3	7 d M1/C1	7 d M2/C2	7 d M3/C3	10 d M1/C1	10 d M2/C2	10 d M3/C3			
0.890	0.891	0.996	0.958	0.901	1.061	1.052	1.070	1.082	1.162	0.754	1.000	Q3MIX3	ADCK5	Uncharacterized aarF domain-containing protein kinase 5
1.030	1.102	1.076							1.054	0.920	1.390	Q6NSJ0	KIAA1161	Uncharacterized family 31 glucosidase KIAA1161
1.002	0.979	1.015	0.994	0.967	1.006	0.974	0.951	0.972	1.007	0.940	0.994	F8W031	1 SV	Uncharacterized protein (Fragment)
0.988	0.998	0.938	0.983	0.925	0.946	0.939	1.029	0.947	0.952	0.993	0.912	H0YHG0	1 SV	Uncharacterized protein (Fragment)
1.017	1.000	1.059	0.940	0.979	1.003	0.974	1.026	0.987	1.040	0.944	1.048	H0YIV9	3 SV	Uncharacterized protein (Fragment)
									1.058	1.002	1.232	M0R296	4 SV	Uncharacterized protein (Fragment)
0.975	1.004	0.966	0.954	1.039	0.947	0.986	0.955	0.977	0.891	1.035	1.002	L7N2F9	4 SV	Uncharacterized protein (Fragment)
0.957	0.983	0.971	0.888	0.877	1.069							H0Y3Z8	4 SV	Uncharacterized protein (Fragment)
1.080	1.012	1.048	1.004	0.947	1.065	0.971	1.040	0.915	1.022	1.030	1.051	H7C0S8	4 SV	Uncharacterized protein (Fragment)
0.957	1.000	0.992	0.985	0.992	0.974	1.009	0.962	0.980	0.982	0.991	1.017	A0A096LPI6	4 SV	Uncharacterized protein (Fragment)
0.887	0.990	0.980	0.971	1.000	1.024	0.989	0.924	1.013	0.958	1.011	0.979	H3BN98	4 SV	Uncharacterized protein (Fragment)
1.120	1.213	1.066	1.060	1.207	1.218	0.938	1.227	1.223	0.858	1.034	0.999	Q9H8K7	C10orf88	Uncharacterized protein C10orf88
1.066	0.953	1.068	0.994	1.024	1.080	1.019	1.081	1.076	0.987	1.017	1.165	Q9BUA3	C11orf84	Uncharacterized protein C11orf84
1.054	1.005	1.024	1.022	0.979	1.116	1.052	0.812	1.024	0.978	0.841	1.014	Q8N999	C12orf29	Uncharacterized protein C12orf29
1.027	0.961	0.994	0.951	0.991	0.969	1.023	1.054	0.940	0.932	0.922	1.083	A0A087WT30	C12orf45	Uncharacterized protein C12orf45
			1.050	1.079	1.119	1.002	1.065	1.074	0.988	1.036	1.028	Q69YU5	C12orf73	Uncharacterized protein C12orf73
0.914	0.930	0.964	0.985	1.014	1.156	0.992	0.940	1.050	0.950	0.842	1.053	Q9NWQ9	C14orf119	Uncharacterized protein C14orf119
0.935	0.980	1.033	0.977	0.972	1.048	0.962	0.823	0.995	1.087	0.949	1.129	Q6ZUT6	C15orf52	Uncharacterized protein C15orf52
			1.104	0.849	0.984	0.796	1.061	1.126	0.884	1.089	1.270	A6NNL5	C15orf61	Uncharacterized protein C15orf61
			0.785	0.902	1.080	0.847	1.026	0.917				Q8N3J3	C17orf53	Uncharacterized protein C17orf53
0.987	0.960	1.005	1.015	0.899	1.096	1.125	1.103	1.042	1.052	0.980	1.091	Q9BQA9	C17orf62	Uncharacterized protein C17orf62
0.985	1.018	1.067				1.036	1.021	0.903	1.035	1.046	1.022	Q9BSJ5	C17orf80	Uncharacterized protein C17orf80
0.995	0.984	1.016	0.950	0.923	1.031	1.000	1.133	1.035	0.994	0.987	1.032	Q96DM3	C18orf8	Uncharacterized protein C18orf8
0.981	1.014	0.930	0.980	0.926	0.959	0.953	0.990	0.931	1.007	1.014	1.005	Q9BQ61	C19orf43	Uncharacterized protein C19orf43
1.022	1.013	1.084	0.996	0.912	1.072	1.147	0.951	0.952	1.008	0.977	1.092	Q8N9M1	C19orf47	Uncharacterized protein C19orf47
1.009	0.966	1.006	1.154	1.130	1.251							A0A087WWL5	C19orf68	Uncharacterized protein C19orf68
0.960	0.963	1.059	0.942	0.864	0.903	0.956	1.268	0.954	0.999	0.872	1.057	Q9NX04	C1orf109	Uncharacterized protein C1orf109
1.034	1.134	1.018	1.129	0.959	0.986	1.009	0.842	0.945	0.957	0.915	1.077	Q6ZSJ8	C1orf122	Uncharacterized protein C1orf122
1.009	1.005	0.938	0.990	0.949	0.956	0.986	1.046	0.957	1.051	0.978	1.017	Q8NDD1	C1orf131	Uncharacterized protein C1orf131
0.982	0.935	0.959	0.938	0.930	0.975	0.921	1.057	1.035	0.977	0.988	1.011	Q9H425	C1orf198	Uncharacterized protein C1orf198
0.995	0.867	1.029	1.117	0.930	0.988	1.007	0.891	0.947	1.041	0.871	1.039	Q9BWL3	C1orf43	Uncharacterized protein C1orf43
0.969	0.994	0.995	1.012	1.021	1.085	0.966	1.025	0.982	0.921	1.006	0.951	Q9BV19	C1orf50	Uncharacterized protein C1orf50
			1.111	1.101	1.053							Q8N0U7	C1orf87	Uncharacterized protein C1orf87

Abundance Ratio														
1 d M1/C1	1 d M2/C2	1 d M3/C3	4 d M1/C1	4 d M2/C2	4 d M3/C3	7 d M1/C1	7 d M2/C2	7 d M3/C3	10 d M1/C1	10 d M2/C2	10 d M3/C3	Accession ID	Gene name	Protein Description
1.002	0.983	1.016	0.973	0.984	1.187	1.116	0.904	0.975	1.043	0.996	1.128	Q5JPI3	C3orf38	Uncharacterized protein C3orf38
1.195	0.947	1.037	0.944	1.012	0.958	1.155	0.915	0.847				Q8N8J7	C4orf32	Uncharacterized protein C4orf32
			1.097	1.018	0.996				1.193	1.119	1.087	Q9H6K1	C6orf106	Uncharacterized protein C6orf106
0.868	0.914	1.023	1.099	0.953	1.043	1.094	0.884	1.049	1.201	1.044	1.098	A0A0D9SEI0	C6orf203	Uncharacterized protein C6orf203
0.906	0.919	1.324	0.969	1.248	1.086				1.333	1.018	1.178	O95873	C6orf47	Uncharacterized protein C6orf47
1.090	1.005	1.168	1.002	0.925	1.022	1.009	1.195	0.987	0.947	1.050	0.987	Q96N11	C7orf26	Uncharacterized protein C7orf26
			2.783	0.803	1.048							Q8WVR3	C7orf43	Uncharacterized protein C7orf43
0.995	0.961	1.036	0.963	1.006	1.040	1.009	1.031	1.045	0.966	1.024	1.048	Q9BRJ6	C7orf50	Uncharacterized protein C7orf50
1.037	1.003	1.087	1.031	0.950	1.064	1.019	1.058	1.039	0.889	1.105	1.103	Q8N0T1	C8orf59	Uncharacterized protein C8orf59
0.841	0.700	1.175	0.857	0.874	1.269	1.245	1.290	1.117	0.965	1.014	1.125	Q96K31	C8orf76	Uncharacterized protein C8orf76
1.020	0.989	1.013	0.994	0.941	0.993	1.029	0.955	1.007	1.106	1.020	1.057	Q9NZ63	C9orf78	Uncharacterized protein C9orf78
1.031	0.988	1.044	0.995	1.002	1.006	1.003	1.043	1.056	1.020	1.017	1.056	Q8TB03	CXorf38	Uncharacterized protein CXorf38
			1.124	0.797	1.041	1.098	1.018	0.948	1.010	1.004	1.097	O60268	KIAA0513	Uncharacterized protein KIAA0513
1.051	1.011	1.071	1.016	0.994	1.011	1.024	1.069	1.055	0.969	1.000	1.090	Q6ICG6	KIAA0930	Uncharacterized protein KIAA0930
0.689	0.757	0.880	1.142	1.004	1.122							Q2LD37	KIAA1109	Uncharacterized protein KIAA1109
1.007	1.022	1.012	1.032	0.982	1.058	1.013	0.943	0.946	1.077	1.061	1.094	Q96AT1	KIAA1143	Uncharacterized protein KIAA1143
0.873	1.202	1.183	0.988	1.020	0.924	1.067	1.146	1.155	0.995	0.985	1.052	Q6ZU35	KIAA1211	Uncharacterized protein KIAA1211
0.959	0.942	1.084	0.933	0.923	0.931	1.004	1.012	1.004	0.835	0.836	1.016	Q9HCM1	KIAA1551	Uncharacterized protein KIAA1551
0.987	0.983	1.074	0.966	1.006	1.046	1.015	0.880	0.936	0.959	0.978	1.063	Q9BY89	KIAA1671	Uncharacterized protein KIAA1671
0.814	1.002	1.202	0.910	0.931	0.927	0.871	0.726	1.076	0.924	0.981	1.087	Q69YL0	NCBP2-AS2	Uncharacterized protein NCBP2-AS2
0.927	1.012	0.961	1.015	1.008	0.982	1.022	1.018	0.985	1.013	0.982	1.007	A0A0B4J2A0	1 SV	Uncharacterized protein
									1.026	0.958	1.093	J3KTA2	3 SV	Uncharacterized protein
			0.998	0.818	0.858	0.904	1.123	1.272				E9PBE3	4 SV	Uncharacterized protein
1.020	0.953	0.994	1.050	1.001	1.021	1.064	1.025	1.034	1.045	1.005	1.037	G3V3G9	4 SV	Uncharacterized protein
1.015	1.005	1.016	1.007	0.998	0.998	1.000	0.952	0.990	1.037	1.001	1.001	F5H5P2	4 SV	Uncharacterized protein
1.006	0.975	1.028	0.962	0.986	0.981	1.000	1.176	1.024	1.001	1.052	1.027	E9PCH4	4 SV	Uncharacterized protein
1.002	0.952	1.054	0.990	0.888	1.090	1.017	1.028	0.950	1.134	0.925	1.052	U3KQK5	4 SV	Uncharacterized protein
0.967	0.983	0.930	0.970	1.012	1.014	0.988	1.009	0.988	0.932	1.003	0.970	A0A087WV05	4 SV	Uncharacterized protein
0.897	0.893	1.175	0.833	1.719	1.037							A0A0G2JPF8	4 SV	Uncharacterized protein
1.015	1.013	0.973	0.963	0.967	0.999	0.972	1.039	1.016	0.963	1.014	0.973	H0YC42	4 SV	Uncharacterized protein
1.011	1.018	1.032	1.038	1.006	1.039	1.038	1.064	1.020	0.981	0.963	1.023	O43795	MYO1B	Unconventional myosin-Ib
0.977	1.008	0.999	0.989	1.013	0.993	1.011	1.016	1.033	1.006	0.980	1.024	O00159	MYO1C	Unconventional myosin-Ic
1.042	1.009	1.030	0.988	0.996	0.992	0.988	1.082	1.040	0.996	0.971	1.037	O94832	MYO1D	Unconventional myosin-Id
0.963	0.985	0.986	0.959	0.981	0.991	0.990	1.046	1.009	1.002	0.974	1.023	Q12965	MYO1E	Unconventional myosin-Ie

Abundance Ratio														
1 d M1/C1	1 d M2/C2	1 d M3/C3	4 d M1/C1	4 d M2/C2	4 d M3/C3	7 d M1/C1	7 d M2/C2	7 d M3/C3	10 d M1/C1	10 d M2/C2	10 d M3/C3	Accession ID	Gene name	Protein Description
1.021	1.022	1.018	1.008	0.986	0.977	1.037	1.066	1.019	1.012	0.972	1.036	M0R0P8	MYO9B	Unconventional myosin-IXb
0.977	0.995	1.018	0.986	0.942	1.037	0.996	0.988	1.006	1.022	1.003	1.063	Q9Y4I1	MYO5A	Unconventional myosin-Va
0.953	1.096	1.168	0.911	0.877	0.955	0.989	0.936	1.023	1.011	0.974	1.052	Q9ULV0	MYO5B	Unconventional myosin-Vb
0.993	0.999	1.007	0.990	0.974	1.007	0.986	1.020	0.986	0.973	0.984	1.000	A0A0A0MRM8	MYO6	Unconventional myosin-VI
0.997	0.981	1.024	0.985	0.932	0.995	0.926	0.774	0.986	0.834	0.905	0.932	A0A0A0MQX1	MYO10	Unconventional myosin-X
1.065	1.033	1.024	0.960	0.882	0.968	1.062	0.980	0.966	1.048	0.977	1.091	Q96H55	MYO19	Unconventional myosin-XIX
0.995	1.006	1.046	0.981	0.930	1.032	1.012	1.020	1.013	1.000	0.970	1.068	Q92614	MYO18A	Unconventional myosin-XVIIIa
0.973	0.995	1.015	1.044	0.922	1.009	1.022	1.073	0.994	0.988	1.046	1.059	O94763	URI1	Unconventional prefoldin RPB5 interactor 1
0.997	1.031	0.986	1.013	0.935	1.034	1.031	1.057	1.020	0.980	1.026	1.008	Q14146	URB2	Unhealthy ribosome biogenesis protein 2 homolog
1.013	1.010	1.004	1.022	1.020	0.996	1.005	1.058	1.023	0.980	1.019	0.978	Q9HB07	C12orf10	UPF0160 protein MYG1, mitochondrial
			1.023	1.041	1.108	1.040	0.806	1.135	1.020	0.982	1.101	Q9BSU1	C16orf70	UPF0183 protein C16orf70
1.088	0.940	1.083	1.073	0.997	1.159	1.020	0.911	1.007	1.038	0.818	1.014	Q9BUW7	C9orf16	UPF0184 protein C9orf16
0.978	1.005	0.937	1.036	0.975	0.969	1.016	1.001	0.996	0.903	0.936	1.085	Q8WUR7	C15orf40	UPF0235 protein C15orf40
0.993	0.968	1.003	1.007	0.934	1.043	0.999	1.001	0.958	1.038	1.082	1.056	Q9H5V9	CXorf56	UPF0428 protein CXorf56
1.006	1.015	1.022	0.979	0.951	1.031	1.059	1.016	0.993	0.998	1.021	1.092	Q9UFG5	C19orf25	UPF0449 protein C19orf25
1.067	1.044	1.015	1.056	0.975	1.001	0.975	0.947	0.935	1.027	1.093	0.993	Q7Z6I8	C5orf24	UPF0461 protein C5orf24
1.066	0.882	1.008	0.963	0.962	1.140	1.087	1.128	1.003				Q8N1A6	C4orf33	UPF0462 protein C4orf33
1.028	1.039	1.054	0.977	0.869	0.943	0.958	1.013	0.948	1.025	1.044	1.038	Q7Z7F0	KIAA0907	UPF0469 protein KIAA0907
1.018	1.006	1.010	1.004	0.976	1.066	1.004	0.922	0.997	1.042	1.051	1.124	Q9H7E9	C8orf33	UPF0488 protein C8orf33
0.986	0.990	1.016	0.992	0.916	1.028	0.970	1.035	0.991	0.952	0.942	1.083	Q49AR2	C5orf22	UPF0489 protein C5orf22
0.990	0.991	1.011	0.988	0.985	1.020	0.992	1.068	1.035	0.998	1.000	1.032	E7EWW0	C16orf62	UPF0505 protein C16orf62
1.028	0.924	1.066	0.942	0.943	1.039							J3KNH0	C12orf66	UPF0536 protein C12orf66
1.045	0.960	0.977	0.968	0.960	0.992	0.959	0.872	1.005	1.107	1.403	1.101	Q3KRA6	C2orf76	UPF0538 protein C2orf76
0.961	0.976	0.929				1.095	0.890	1.094	1.079	0.989	1.129	Q6P5X5	C22orf39	UPF0545 protein C22orf39
1.051	1.053	1.079				0.903	1.042	1.037				Q6PH81	C16orf87	UPF0547 protein C16orf87
1.006	1.001	1.024	0.996	1.035	1.033	1.008	0.993	1.062	1.014	0.975	1.024	Q5T6V5	C9orf64	UPF0553 protein C9orf64
0.944	0.894	0.942	0.990	0.934	1.028	1.086	1.099	0.986	1.057	1.013	1.127	Q8N8R5	C2orf69	UPF0565 protein C2orf69
1.012	0.987	1.017	0.989	0.972	1.027	1.000	1.018	1.023	0.981	0.991	0.973	Q9Y224	C14orf166	UPF0568 protein C14orf166
0.977	0.983	0.983	0.984	0.981	0.971	1.053	0.938	0.994	1.045	1.027	1.030	Q9NWV4	C1orf123	UPF0587 protein C1orf123
1.045	0.993	0.999	1.012	1.215	0.967	1.024	0.888	1.031	0.982	0.975	0.989	Q6P1X6	C8orf82	UPF0598 protein C8orf82
1.046	0.959	1.021	0.984	0.990	1.028	1.069	0.956	1.056	1.039	1.019	1.057	A6NDU8	C5orf51	UPF0600 protein C5orf51
1.002	0.997	1.032	1.022	0.905	0.976	0.997	1.080	1.037	0.981	0.956	1.004	Q5T2E6	C10orf76	UPF0668 protein C10orf76
0.922	0.942	1.107	1.060	0.967	0.812	0.899	0.910	1.038	0.866	1.118	0.911	Q7Z4R8	C6orf120	UPF0669 protein C6orf120

Abundance Ratio														
1 d M1/C1	1 d M2/C2	1 d M3/C3	4 d M1/C1	4 d M2/C2	4 d M3/C3	7 d M1/C1	7 d M2/C2	7 d M3/C3	10 d M1/C1	10 d M2/C2	10 d M3/C3	Accession ID	Gene name	Protein Description
0.963	0.892	0.916	0.853	0.814	1.017	0.986	0.930	1.031	0.990	0.939	1.035	Q8IYL3	C1orf174	UPF0688 protein C1orf174
1.103	1.062	1.069	1.118	1.035	0.888	0.925	1.117	0.949	1.031	0.986	1.010	Q8N6N3	C1orf52	UPF0690 protein C1orf52
			1.018	1.092	1.125	0.959	0.792	1.003	1.039	0.724	1.033	Q32NC0	C18orf21	UPF0711 protein C18orf21
0.843	0.928	0.977	0.970	0.890	1.058	0.934	0.732	0.892	0.985	0.943	1.039	Q8TCD1	C18orf32	UPF0729 protein C18orf32
0.961	0.941	0.964	0.993	0.943	1.036	0.994	1.039	1.052	1.023	1.019	1.060	P57076	C21orf59	UPF0769 protein C21orf59
0.989	1.038	0.937	0.955	0.957	0.984	1.064	1.062	0.967	1.147	1.047	0.951	Q96IX5	USMG5	Up-regulated during skeletal muscle growth protein 5
0.977	1.083	1.048	0.972	0.974	1.015	1.049	1.157	1.007	1.066	0.911	0.875	Q8TCY9	URGCP	Up-regulator of cell proliferation
1.150	0.939	1.485										P22415	USF1	Upstream stimulatory factor 1
1.040	0.942	1.021	0.984	0.984	0.997	0.878	0.868	0.993	1.007	1.056	0.987	Q15853	USF2	Upstream stimulatory factor 2
1.060	1.036	1.119	0.994	0.937	0.949	0.939	1.074	0.962	0.928	0.943	1.052	Q9NZI7	UBP1	Upstream-binding protein 1
1.070	0.910	1.010	0.948	0.900	0.760	1.018	1.208	0.897				Q96BW1	UPRT	Uracil phosphoribosyltransferase homolog
0.977	1.065	1.052	0.991	0.952	1.009	0.997	0.967	0.957	0.907	1.007	1.053	P13051	UNG	Uracil-DNA glycosylase
0.981	0.985	0.991	1.018	0.971	1.017	1.008	1.022	1.029	0.980	0.983	1.025	P11172	UMPS	Uridine 5'-monophosphate synthase
1.098	1.093	0.993	0.990	0.999	0.932	0.957	1.100	1.010	0.993	1.013	0.993	O95848	NUDT14	Uridine diphosphate glucose pyrophosphatase
1.089	1.032	1.014	1.037	1.099	1.026	1.032	1.062	1.011	0.973	1.034	0.992	Q16831	UPP1	Uridine phosphorylase 1
0.996	1.007	1.051	1.003	0.934	0.982	1.027	1.065	1.006	1.035	0.998	1.020	Q9BZX2	UCK2	Uridine-cytidine kinase 2
0.984	0.961	1.027	0.982	0.866	0.885	0.953	1.135	1.041	0.941	1.095	1.089	Q9NWZ5	UCKL1	Uridine-cytidine kinase-like 1
0.763	1.023	0.852	1.144	1.065	1.333	1.249	1.102	1.280	0.937	0.990	1.074	Q03405	PLAUR	Urokinase plasminogen activator surface receptor
0.989	0.933	0.995	0.952	0.937	0.992	1.064	0.945	0.944	1.019	1.000	1.001	E7ET40	PLAU	Urokinase-type plasminogen activator
1.044	1.046	1.046	1.026	1.065	0.993	1.045	1.015	1.029	1.001	1.022	1.039	P06132	UROD	Uroporphyrinogen decarboxylase
1.023	0.975	0.997	0.957	1.009	0.925	1.000	1.079	1.070	1.019	1.064	1.109	P10746	UROS	Uroporphyrinogen-III synthase
1.044	1.160	1.207	1.089	0.912	1.095	0.779	0.831	1.011	0.811	0.786	1.410	X6RAB3	USP6NL	USP6 N-terminal-like protein
1.034	1.004	1.012	0.997	0.993	0.994	0.995	1.047	1.008	0.995	0.973	1.015	Q16851	UGP2	UTP--glucose-1-phosphate uridylyltransferase
0.969	0.980	0.990	0.987	0.968	0.982	1.016	0.972	1.002	1.021	0.993	1.044	P46939	UTRN	Utrophin
0.929	1.080	0.987	0.980	0.989	0.994	0.959	1.022	0.999	1.058	1.051	1.008	P54725	RAD23A	UV excision repair protein RAD23 homolog A
1.030	0.999	1.008	0.978	1.003	1.025	0.981	0.994	1.002	0.950	0.991	0.959	P54727	RAD23B	UV excision repair protein RAD23 homolog B
1.053	1.007	1.013	0.973	0.997	1.041	1.004	1.007	1.038	0.974	0.975	1.055	Q9P2Y5	UVRAG	UV radiation resistance-associated gene protein
1.014	1.032	1.023	1.023	0.974	1.011	1.037	1.029	1.052	1.001	0.987	1.083	Q9BZF9	UACA	Uveal autoantigen with coiled-coil domains and ankyrin repeats
0.989	0.971	1.006	0.980	0.923	1.006	1.037	1.150	1.064	1.114	1.071	1.140	Q3ZAQ7	VMA21	Vacuolar ATPase assembly integral membrane protein VMA21
1.001	1.030	0.985	0.988	0.915	0.983	1.031	1.096	1.027	1.036	0.995	1.011	P86790	CCZ1B	Vacuolar fusion protein CCZ1 homolog B
1.028	1.013	1.002	0.970	0.917	1.013	1.015	0.983	0.994	0.935	0.918	1.061	Q7L1V2	MON1B	Vacuolar fusion protein MON1 homolog B

Abundance Ratio														
1 d M1/C1	1 d M2/C2	1 d M3/C3	4 d M1/C1	4 d M2/C2	4 d M3/C3	7 d M1/C1	7 d M2/C2	7 d M3/C3	10 d M1/C1	10 d M2/C2	10 d M3/C3	Accession ID	Gene name	Protein Description
1.011	0.981	0.988	1.009	1.014	1.016	1.000	1.008	1.011	1.016	0.985	1.034	A0A087WXL6	VPS11	Vacuolar protein sorting 11 (Yeast), isoform CRA_a
1.036	0.955	1.027	1.005	0.949	0.988	1.025	1.060	0.987	1.036	0.989	1.032	Q96RL7	VPS13A	Vacuolar protein sorting-associated protein 13A
1.004	1.078	1.073	0.965	1.114	0.987							Q7Z7G8	VPS13B	Vacuolar protein sorting-associated protein 13B
0.987	1.008	1.018	1.016	0.954	1.012	1.030	1.064	1.014	1.001	0.995	1.027	Q709C8	VPS13C	Vacuolar protein sorting-associated protein 13C
1.103	1.038	1.019	0.991	0.963	0.997	1.003	1.078	1.034	1.024	0.974	1.046	Q5THJ4	VPS13D	Vacuolar protein sorting-associated protein 13D
1.022	1.008	1.040	1.005	0.929	0.979	1.000	0.982	1.028	0.998	0.947	1.037	Q9H269	VPS16	Vacuolar protein sorting-associated protein 16 homolog
0.977	0.998	1.000	0.988	0.974	1.038	0.993	1.034	1.014	1.038	0.994	1.070	Q9P253	VPS18	Vacuolar protein sorting-associated protein 18 homolog
1.006	0.995	0.991	1.004	0.950	1.018	0.990	1.022	1.008	0.999	0.984	0.992	O75436	VPS26A	Vacuolar protein sorting-associated protein 26A
1.073	1.004	1.008	1.057	0.980	1.018	1.030	1.019	1.036	1.096	1.030	1.006	Q4G0F5	VPS26B	Vacuolar protein sorting-associated protein 26B
0.930	0.903	1.009	1.002	0.940	1.059	1.066	1.041	0.981	0.974	0.956	0.999	Q9UK41	VPS28	Vacuolar protein sorting-associated protein 28 homolog
1.036	0.998	1.030	1.004	1.078	1.003	1.029	1.001	1.011	1.029	0.966	0.992	F8VXU5	VPS29	Vacuolar protein sorting-associated protein 29
0.887	1.063	1.277	0.867	0.929	0.998	0.965	1.091	1.096	1.005	0.894	1.044	Q9UBQ0	VPS29	Vacuolar protein sorting-associated protein 29
1.036	0.983	1.032	0.982	0.959	0.973	1.020	1.028	1.023	0.969	0.968	0.990	Q96AX1	VPS33A	Vacuolar protein sorting-associated protein 33A
1.011	1.002	0.990	0.993	0.985	1.010	1.020	1.077	1.007	0.995	0.989	1.012	Q9H267	VPS33B	Vacuolar protein sorting-associated protein 33B
1.013	1.014	1.022	0.993	1.065	0.998	0.997	1.005	1.039	0.975	0.991	1.012	Q96QK1	VPS35	Vacuolar protein sorting-associated protein 35
0.984	1.000	0.982	0.967	0.971	0.999	0.987	1.077	1.005	0.943	0.937	0.969	Q8NEZ2	VPS37A	Vacuolar protein sorting-associated protein 37A
1.095	1.006	1.029	1.094	0.999	1.073	1.071	1.099	1.077	1.065	1.036	1.050	Q9H9H4	VPS37B	Vacuolar protein sorting-associated protein 37B
0.979	0.885	0.987	0.981	1.044	0.998	1.015	1.013	1.108	0.960	1.002	1.084	A5D8V6	VPS37C	Vacuolar protein sorting-associated protein 37C
1.036	1.036	1.058	1.017	0.973	1.013	1.076	1.155	1.012	1.037	1.004	0.917	P49754	VPS41	Vacuolar protein sorting-associated protein 41 homolog
1.029	1.016	1.011	1.047	0.973	1.038	1.024	0.995	1.044	1.018	0.982	1.021	Q9NRW7	VPS45	Vacuolar protein sorting-associated protein 45
1.029	1.000	1.027	1.005	0.958	1.011	1.001	1.064	1.015	1.006	1.015	1.038	Q9UN37	VPS4A	Vacuolar protein sorting-associated protein 4A
1.017	1.016	1.005	1.003	0.952	0.974	0.991	1.051	1.000	0.989	1.008	0.990	O75351	VPS4B	Vacuolar protein sorting-associated protein 4B
1.006	0.997	1.011	1.013	1.026	1.015	1.002	1.105	1.050	0.926	1.026	0.993	Q9UID3	VPS51	Vacuolar protein sorting-associated protein 51 homolog
1.021	1.011	1.015	1.015	0.975	1.030	1.002	1.028	1.063	1.003	0.991	1.038	Q8N1B4	VPS52	Vacuolar protein sorting-associated protein 52 homolog
1.132	1.064	1.250	0.861	1.058	1.051							Q9P1Q0	VPS54	Vacuolar protein sorting-associated protein 54
0.910	0.997	0.946	1.018	1.058	0.951	1.010	1.050	0.955	1.004	1.048	0.965	Q9NP79	VTA1	Vacuolar protein sorting-associated protein VTA1 homolog
1.071	0.997	1.021	1.021	1.001	1.018	1.018	1.094	1.027	1.001	0.999	1.018	Q9BRG1	VPS25	Vacuolar protein-sorting-associated protein 25
0.992	0.999	1.051	1.034	0.958	1.028	1.020	0.979	1.015	1.047	1.001	1.065	Q86VN1	VPS36	Vacuolar protein-sorting-associated protein 36
1.019	0.978	1.006	1.039	0.931	1.042	1.027	1.003	1.002	1.060	0.990	1.063	Q96H20	SNF8	Vacuolar-sorting protein SNF8

Abundance Ratio														
1 d M1/C1	1 d M2/C2	1 d M3/C3	4 d M1/C1	4 d M2/C2	4 d M3/C3	7 d M1/C1	7 d M2/C2	7 d M3/C3	10 d M1/C1	10 d M2/C2	10 d M3/C3	Accession ID	Gene name	Protein Description
1.011	0.984	0.900	0.875	0.969	1.111	1.155	1.183	1.123	1.029	1.043	1.053	Q96GC9	VMP1	Vacuole membrane protein 1
1.021	0.975	0.981	0.986	0.972	1.017	1.002	0.935	1.071	1.028	0.963	1.004	Q86WA6	BPHL	Valacyclovir hydrolase
0.985	0.997	1.003	0.996	1.022	1.016	1.004	1.038	1.022	1.000	1.001	1.005	P26640	VARS	Valine--tRNA ligase
1.011	1.027	1.034	1.026	0.988	1.003	1.031	1.096	1.058	1.024	1.011	1.054	A0A0G2JL52	VARS2	Valine--tRNA ligase, mitochondrial
1.008	0.950	1.040	1.017	0.987	1.027	0.976	1.118	1.097	1.011	0.985	1.001	Q96JC1	VPS39	Vam6/Vps39-like protein
1.014	1.110	1.078	0.942	0.952	1.077	1.015	0.986	0.881				Q8TAA9	VANGL1	Vang-like protein 1
						0.860	0.845	1.045	1.055	1.031	1.051	J3KPA4	VEGFA	Vascular endothelial growth factor A
0.983	1.039	1.012	1.069	1.013	0.978	1.105	0.787	1.076	1.015	1.041	0.971	Q14119	VEZF1	Vascular endothelial zinc finger 1
1.025	0.990	1.025	0.982	0.960	1.004	0.941	1.005	1.004	0.911	1.001	0.927	P50552	VASP	Vasodilator-stimulated phosphoprotein
1.064	0.979	1.066	1.028	1.086	0.984	1.062	0.824	0.996	1.000	0.993	1.063	P13611	VCAN	Versican core protein
0.933	0.952	0.983	1.060	1.043	1.181							Q68DQ2	CRYBG3	Very large A-kinase anchor protein
0.795	0.923	1.006	0.901	0.930	1.045	0.830	0.606	1.045	1.038	1.085	1.075	Q6Y1H2	HACD2	Very-long-chain (3R)-3-hydroxyacyl-CoA dehydratase 2
0.950	0.998	0.985	0.966	0.900	0.978	0.999	1.113	1.001	1.041	1.024	1.011	Q9P035	HACD3	Very-long-chain (3R)-3-hydroxyacyl-CoA dehydratase 3
1.011	0.990	1.044	1.001	0.948	1.011	1.005	1.059	0.977	0.957	1.044	0.912	Q53GQ0	HSD17B12	Very-long-chain 3-oxoacyl-CoA reductase
0.948	0.980	0.974	0.967	0.884	1.022	1.037	0.959	0.992	1.331	1.072	1.083	Q9NZ01	TECR	Very-long-chain enoyl-CoA reductase
0.958	0.996	1.038	0.977	0.962	0.997	1.054	1.081	0.993	0.982	0.950	1.175	Q9Y3E0	GOLT1B	Vesicle transport protein GOT1B
0.962	0.977	0.996	0.963	0.910	1.030	1.005	1.110	1.012	0.978	1.025	1.066	Q12981	BNIP1	Vesicle transport protein SEC20
						1.056	0.761	0.898				Q8WV19	SFT2D1	Vesicle transport protein SFT2A
						1.107	0.880	0.931				O95562	SFT2D2	Vesicle transport protein SFT2B
0.793	0.881	1.167	0.946	0.790	1.017	0.929	1.071	0.884				Q587I9	SFT2D3	Vesicle transport protein SFT2C
1.012	1.025	1.091	0.978	0.907	1.015	1.013	0.966	0.933	1.051	1.053	1.075	Q9NZ43	USE1	Vesicle transport protein USE1
1.031	0.570	1.097	0.748	1.071	1.164	0.920	0.551	0.983	1.057	1.024	1.036	Q96AJ9	VTI1A	Vesicle transport through interaction with t-SNAREs homolog 1A
0.947	0.930	1.043	0.976	0.944	1.015	0.995	0.926	0.970	1.060	0.994	1.086	Q9UEU0	VTI1B	Vesicle transport through interaction with t-SNAREs homolog 1B
0.893	0.888	0.999	0.969	1.013	0.993	0.969	0.955	0.997	0.995	0.993	0.948	Q15836	VAMP3	Vesicle-associated membrane protein 3
0.863	0.994	1.152	0.920	1.056	0.865	0.954	0.964	1.136	0.884	1.073	1.174	O75379	VAMP4	Vesicle-associated membrane protein 4
0.943	0.976	1.011	1.000	0.900	1.015	1.026	1.113	1.069	1.057	1.063	1.068	P51809	VAMP7	Vesicle-associated membrane protein 7
1.014	0.976	1.018	0.899	1.172	1.125	1.097	0.853	0.944	1.083	1.020	1.064	Q9BV40	VAMP8	Vesicle-associated membrane protein 8
0.869	0.973	0.941	0.972	0.896	0.983	1.004	1.004	0.953	1.005	1.028	0.971	Q9P0L0	VAPA	Vesicle-associated membrane protein-associated protein A
0.968	1.006	0.997	0.983	0.975	0.979	0.995	1.016	0.967	1.026	1.023	1.018	O95292	VAPB	Vesicle-associated membrane protein-associated protein B/C
0.987	1.005	1.014	0.994	1.021	0.997	0.994	1.006	1.010	0.978	0.966	1.005	P46459	NSF	Vesicle-fusing ATPase

Abundance Ratio												Accession ID	Gene name	Protein Description
1 d M1/C1	1 d M2/C2	1 d M3/C3	4 d M1/C1	4 d M2/C2	4 d M3/C3	7 d M1/C1	7 d M2/C2	7 d M3/C3	10 d M1/C1	10 d M2/C2	10 d M3/C3			
			1.030	0.951	0.872	1.191	1.021	0.976	0.990	1.064	0.861	Q96IW7	SEC22A	Vesicle-trafficking protein SEC22a
0.953	1.000	0.993	0.995	0.953	0.996	1.042	1.083	0.993	1.059	1.037	1.044	O75396	SEC22B	Vesicle-trafficking protein SEC22b
0.948	0.995	0.970	0.951	0.934	0.943	1.007	1.025	0.991	1.074	0.999	0.962	Q12907	LMAN2	Vesicular integral-membrane protein VIP36
1.086	1.035	1.176	0.963	0.890	1.156	1.137	0.985	0.977	1.002	1.101	1.101	Q9HBM0	VEZT	Vezatin
1.049	1.020	1.015	1.017	0.979	1.000	0.984	0.990	0.989	0.995	0.977	0.972	P08670	VIM	Vimentin
0.957	1.009	0.984	0.992	1.018	0.969	0.992	0.971	0.978	0.992	0.979	1.005	P18206	VCL	Vinculin
1.018	1.028	0.980	1.000	0.916	1.009	1.029	1.032	0.981	1.084	0.966	1.054	O60504	SORBS3	Vinexin
0.820	0.920	1.043	0.824	0.946	0.939	0.776	0.601	0.889	1.020	1.015	1.033	Q9BQB6	VKORC1	Vitamin K epoxide reductase complex subunit 1
0.883	0.948	0.921	1.014	0.850	1.026	1.042	0.952	1.107	1.105	1.054	1.026	Q8N0U8	VKORC1L1	Vitamin K epoxide reductase complex subunit 1-like protein 1
1.011	1.015	1.003	0.893	0.788	1.109	0.904	1.127	1.274	1.112	1.107	1.038	P38435	GGCX	Vitamin K-dependent gamma-carboxylase
			0.962	0.942	0.921							P07225	PROS1	Vitamin K-dependent protein S
1.051	1.000	1.014	1.006	1.024	0.969	0.982	0.997	0.983	0.979	1.005	0.949	P21796	VDAC1	Voltage-dependent anion-selective channel protein 1
1.026	1.013	0.966	0.998	1.006	0.936	0.989	1.008	1.002	1.012	1.018	0.944	Q9Y277	VDAC3	Voltage-dependent anion-selective channel protein 3
0.709	1.248	1.842	0.918	1.190	1.284	0.930	1.239	1.551	1.049	0.736	1.201	A0A140LJL2	CBARP	Voltage-dependent calcium channel beta subunit-associated regulatory protein
			1.032	0.907	1.094							P54289	CACNA2D1	Voltage-dependent calcium channel subunit alpha-2/delta-1
0.686	0.863	1.478	0.760	0.740	1.447	0.707	0.931	1.794	0.688	0.817	1.567	Q8IZS8	CACNA2D3	Voltage-dependent calcium channel subunit alpha-2/delta-3
1.062	0.944	1.302	0.855	0.927	1.015	0.643	0.435	0.902	1.003	1.147	1.184	O60840	CACNA1F	Voltage-dependent L-type calcium channel subunit alpha-1F
0.971	1.015	1.013	0.918	0.989	0.964	1.013	1.075	1.027	1.049	0.823	1.140	P54284	CACNB3	Voltage-dependent L-type calcium channel subunit beta-3
1.059	1.029	1.023	1.024	0.922	1.017	0.976	1.156	0.980	1.043	1.014	1.030	Q8IWT6	LRRC8A	Volume-regulated anion channel subunit LRRC8A
1.074	0.918	0.928	1.014	1.142	1.061	1.155	1.322	0.999				Q6P9F7	LRRC8B	Volume-regulated anion channel subunit LRRC8B
0.941	0.999	1.006	1.011	0.985	1.030	1.075	1.026	1.069	1.043	1.118	1.061	Q8TDW0	LRRC8C	Volume-regulated anion channel subunit LRRC8C
0.937	0.967	1.116	1.043	0.915	1.058				0.954	1.030	1.615	Q7L1W4	LRRC8D	Volume-regulated anion channel subunit LRRC8D
1.048	0.941	0.931	1.083	0.671	1.023	0.841	0.909	1.010				Q6NSJ5	LRRC8E	Volume-regulated anion channel subunit LRRC8E
1.042	0.993	1.024	0.983	0.916	0.978	0.992	1.058	0.967	0.986	0.981	0.986	P40337	VHL	Von Hippel-Lindau disease tumor suppressor
1.016	0.819	1.041	1.060	0.980	0.939	1.049	1.324	1.109	1.075	0.981	0.960	O00534	VWA5A	von Willebrand factor A domain-containing protein 5A
1.003	1.021	1.027	1.004	0.944	1.025	1.006	1.094	1.024	0.993	1.009	0.987	A3KMH1	VWA8	von Willebrand factor A domain-containing protein 8
									1.042	0.983	1.111	Q8N2E2	VWDE	von Willebrand factor D and EGF domain-containing protein

Abundance Ratio														
1 d M1/C1	1 d M2/C2	1 d M3/C3	4 d M1/C1	4 d M2/C2	4 d M3/C3	7 d M1/C1	7 d M2/C2	7 d M3/C3	10 d M1/C1	10 d M2/C2	10 d M3/C3	Accession ID	Gene name	Protein Description
0.975	0.924	1.135				0.795	0.698	0.830	1.429	1.404	1.243	Q8N0Z9	VSIG10	V-set and immunoglobulin domain-containing protein 10
									1.053	1.195	1.169	Q9H7M9	VSIR	V-type immunoglobulin domain-containing suppressor of T-cell activation
0.948	1.042	1.020	0.995	0.928	1.066	0.985	1.117	1.090	0.979	1.015	1.000	Q9Y487	ATP6V0A2	V-type proton ATPase 116 kDa subunit a isoform 2
1.020	0.996	1.032	0.963	0.875	1.023	0.997	1.007	1.031	1.036	0.984	1.004	Q13488	TCIRG1	V-type proton ATPase 116 kDa subunit a isoform 3
0.833	1.035	0.928	1.060	0.940	0.955	0.996	1.050	1.001	1.040	1.217	0.918	P27449	ATP6V0C	V-type proton ATPase 16 kDa proteolipid subunit
1.009	1.010	0.989	1.011	1.003	1.039	1.016	1.019	1.017	1.014	0.994	1.018	P38606	ATP6V1A	V-type proton ATPase catalytic subunit A
1.007	1.006	1.025	1.007	1.011	1.037	1.007	1.033	1.022	1.019	1.017	1.025	P21281	ATP6V1B2	V-type proton ATPase subunit B, brain isoform
0.988	0.976	1.008	1.015	1.004	1.080	1.038	1.023	1.052	1.006	0.993	1.022	P21283	ATP6V1C1	V-type proton ATPase subunit C 1
1.011	1.000	1.005	1.020	1.020	1.069	1.060	1.068	1.019	1.038	1.007	1.033	F5GYQ1	ATP6V0D1	V-type proton ATPase subunit d 1
									0.747	0.828	1.538	Q8N8Y2	ATP6V0D2	V-type proton ATPase subunit d 2
1.017	0.998	1.030	1.008	0.994	1.048	1.000	0.959	1.042	1.052	0.997	1.051	Q9Y5K8	ATP6V1D	V-type proton ATPase subunit D
0.982	0.989	0.994	0.997	0.997	1.030	0.972	0.991	1.014	1.015	1.027	1.025	P36543	ATP6V1E1	V-type proton ATPase subunit E 1
1.147	1.038	1.116	1.034	1.182	1.052	1.058	0.818	1.110	1.052	1.017	1.072	O75348	ATP6V1G1	V-type proton ATPase subunit G 1
1.009	0.995	1.006	1.017	0.922	1.027	1.013	1.009	0.977	1.013	1.004	1.023	Q9UI12	ATP6V1H	V-type proton ATPase subunit H
0.955	0.964	1.006	1.025	0.965	1.041	1.042	1.100	1.032	1.006	1.057	1.003	Q15904	ATP6AP1	V-type proton ATPase subunit S1
			1.092	0.852	1.103	1.099	1.219	0.899	1.099	1.063	1.031	Q5VU97	CACHD1	VWFA and cache domain-containing protein 1
									1.258	0.990	1.077	Q14508	WFDC2	WAP four-disulfide core domain protein 2
1.018	0.974	1.004	0.987	0.960	0.992	1.015	1.015	1.026	1.014	0.988	1.039	Q6VEQ5	WASH2P	WAS protein family homolog 2
1.091	0.954	0.969	1.051	1.672	1.016	1.116	0.833	1.024	1.257	1.030	1.347	Q9NQA3	WASH6P	WAS protein family homolog 6
1.031	1.004	1.062	0.984	0.940	1.019	0.982	1.039	1.010	1.026	0.990	1.054	Q8TF74	WIPF2	WAS/WASL-interacting protein family member 2
1.004	0.990	1.011	0.991	0.975	1.026	1.003	0.960	1.038	1.020	0.999	1.053	Q641Q2	WASHC2A	WASH complex subunit 2A
1.018	1.013	1.020	1.003	0.987	1.002	1.018	0.973	1.082	1.085	1.001	1.099	A0A096LPC5	WASHC2C	WASH complex subunit 2C
1.019	0.998	1.013	0.978	0.979	1.000	1.039	1.011	1.028	1.031	0.981	1.028	Q9Y3C0	WASHC3	WASH complex subunit 3
1.008	0.987	1.021	0.989	0.961	1.048	1.015	1.021	1.047	0.981	1.004	1.005	A0A087X256	WASHC4	WASH complex subunit 4
1.040	1.002	1.028	1.017	0.930	1.038	1.029	1.088	1.027	0.994	1.027	0.997	Q12768	WASHC5	WASH complex subunit 5
1.060	1.045	0.965	1.006	0.913	1.065	0.995	0.964	1.000	1.191	0.954	1.074	Q8N5D0	WDTC1	WD and tetratricopeptide repeats protein 1
0.688	1.211	1.020	1.135	0.872	1.011	1.134	0.947	0.947	1.025	1.000	1.096	Q9H6R7	WDCP	WD repeat and coiled-coil-containing protein
0.977	1.024	1.007	1.027	1.115	0.980	1.015	0.828	1.049	0.958	0.983	0.992	Q8IWB7	WDFY1	WD repeat and FYVE domain-containing protein 1
0.986	1.053	0.881	0.875	0.864	1.084	0.852	1.069	1.934	0.960	1.106	1.196	Q96P53	WDFY2	WD repeat and FYVE domain-containing protein 2
1.055	1.044	1.042	0.959	0.913	0.939	0.928	1.067	1.059	0.983	0.886	0.897	Q8IZQ1	WDFY3	WD repeat and FYVE domain-containing protein 3
0.977	1.000	1.015	0.960	0.948	0.988	1.058	0.994	1.022	0.941	0.941	1.081	O75717	WDHD1	WD repeat and HMG-box DNA-binding protein 1

Abundance Ratio														
1 d M1/C1	1 d M2/C2	1 d M3/C3	4 d M1/C1	4 d M2/C2	4 d M3/C3	7 d M1/C1	7 d M2/C2	7 d M3/C3	10 d M1/C1	10 d M2/C2	10 d M3/C3	Accession ID	Gene name	Protein Description
1.062	0.988	1.085	1.031	0.929	1.038	1.034	0.975	1.001	0.956	1.037	0.990	Q5MNZ9	WIPI1	WD repeat domain phosphoinositide-interacting protein 1
1.027	0.990	0.958	1.008	0.921	0.932	0.989	1.035	0.985	1.000	0.983	1.008	Q9Y4P8	WIPI2	WD repeat domain phosphoinositide-interacting protein 2
1.037	1.012	1.003	1.039	0.977	0.948	0.992	1.073	0.990	0.993	1.029	1.009	Q5MNZ6	WDR45B	WD repeat domain phosphoinositide-interacting protein 3
						1.071	0.985	0.989				Q9BRX9	WDR83	WD repeat domain-containing protein 83
1.068	0.962	1.018	0.983	1.012	1.059	1.016	0.933	0.997	0.993	1.002	1.227	Q8N9V3	WDSUB1	WD repeat, SAM and U-box domain-containing protein 1
1.025	0.989	0.992	1.013	1.024	0.993	0.992	0.962	1.023	0.998	0.980	1.013	O75083	WDR1	WD repeat-containing protein 1
1.000	0.965	0.994	0.995	0.988	1.027	1.001	1.010	1.034	1.024	0.999	1.066	Q9BZH6	WDR11	WD repeat-containing protein 11
1.443	0.820	0.801	1.039	0.943	1.149	1.125	1.008	0.967	1.029	1.066	1.021	A0A087X091	WDR13	WD repeat-containing protein 13 (Fragment)
1.038	1.019	1.056	1.019	0.987	1.023	1.039	1.049	1.002	1.043	1.006	1.044	Q9H1Z4	WDR13	WD repeat-containing protein 13
1.009	1.000	1.025	0.995	0.967	1.034	0.988	1.024	0.986	0.993	0.972	0.984	Q9BV38	WDR18	WD repeat-containing protein 18
1.040	1.006	0.992	1.013	0.951	1.026	0.965	1.017	1.078	1.061	0.965	1.053	Q8NEZ3	WDR19	WD repeat-containing protein 19
			1.027	1.169	1.105				1.040	1.007	1.030	A0A088AWN2	WDR20	WD repeat-containing protein 20
0.902	0.902	1.098	0.944	0.891	1.013	0.956	1.029	1.037	1.097	0.956	1.070	Q96S15	WDR24	WD repeat-containing protein 24
0.918	1.624	0.541				0.663	1.475	0.499	0.733	1.263	0.672	Q64LD2	WDR25	WD repeat-containing protein 25
1.035	1.014	1.018	0.990	0.945	1.063	1.021	1.095	1.021	0.937	0.989	0.998	Q9H7D7	WDR26	WD repeat-containing protein 26
1.047	0.987	1.022	0.993	0.961	1.041	1.026	1.080	1.020	1.019	1.010	1.004	Q9UNX4	WDR3	WD repeat-containing protein 3
1.052	1.009	1.032	0.965	0.908	0.937	0.910	0.999	1.046	0.888	0.962	0.980	Q96EX3	WDR34	WD repeat-containing protein 34
1.038	1.046	1.077	1.034	0.965	1.020	0.956	1.086	1.015	0.999	1.055	1.037	Q9P2L0	WDR35	WD repeat-containing protein 35
1.015	1.010	1.028	0.989	0.952	1.003	1.003	1.074	1.001	0.990	1.035	0.986	Q8NI36	WDR36	WD repeat-containing protein 36
1.056	1.018	1.054	0.968	0.974	1.051	0.971	0.986	1.084	0.966	1.001	1.025	Q9HAD4	WDR41	WD repeat-containing protein 41
0.952	1.015	1.024	0.970	0.968	1.022	0.984	1.054	0.983	0.995	1.008	1.047	Q15061	WDR43	WD repeat-containing protein 43
0.993	0.993	1.007	0.997	0.943	0.999	0.998	0.991	1.002	1.005	0.968	1.034	Q5JSH3	WDR44	WD repeat-containing protein 44
0.900	0.955	1.009	0.890	0.875	0.987	0.931	1.002	1.057	1.042	1.152	1.025	A0A0G2JJL1	WDR46	WD repeat-containing protein 46 (Fragment)
1.109	0.976	1.009	0.989	0.987	1.058	1.051	0.974	1.012	1.040	1.044	1.043	O15213	WDR46	WD repeat-containing protein 46
0.976	0.983	1.022	1.020	0.976	0.998	1.053	1.038	1.027	1.037	0.976	1.126	Q8TAF3	WDR48	WD repeat-containing protein 48
1.026	0.990	0.988	0.971	0.984	0.990	0.949	0.983	0.997	0.939	0.982	0.992	P61964	WDR5	WD repeat-containing protein 5
0.937	1.019	1.093										Q7Z5U6	WDR53	WD repeat-containing protein 53
1.028	0.964	1.058	1.005	0.993	1.294	1.012	1.083	1.087	0.965	0.913	1.081	Q9H977	WDR54	WD repeat-containing protein 54
0.961	1.021	1.018	0.978	0.932	0.998	1.027	1.025	0.983	1.047	0.966	1.076	Q9H6Y2	WDR55	WD repeat-containing protein 55
0.960	1.028	1.034	1.016	0.938	1.014	0.953	1.058	0.997	1.019	0.986	1.012	Q6PJI9	WDR59	WD repeat-containing protein 59
1.012	0.987	1.067	0.979	0.887	0.992	0.928	0.935	0.972	1.067	0.893	1.034	A0A087X295	WDR6	WD repeat-containing protein 6

Abundance Ratio												Accession ID	Gene name	Protein Description
1 d M1/C1	1 d M2/C2	1 d M3/C3	4 d M1/C1	4 d M2/C2	4 d M3/C3	7 d M1/C1	7 d M2/C2	7 d M3/C3	10 d M1/C1	10 d M2/C2	10 d M3/C3			
1.226	0.886	1.185	0.979	0.914	1.018	0.940	0.952	0.999	1.053	0.855	0.950	Q8WVS4	WDR60	WD repeat-containing protein 60
1.032	0.994	1.006	1.014	0.972	0.965	0.987	1.060	1.006	0.954	0.980	0.945	Q9GZS3	WDR61	WD repeat-containing protein 61
0.997	1.024	0.969	0.967	0.926	1.023	0.995	1.067	1.003	0.998	0.959	1.052	Q9Y4E6	WDR7	WD repeat-containing protein 7
1.010	0.994	1.005	1.011	0.970	0.999	1.051	0.999	0.983	1.028	0.978	1.050	Q9NW82	WDR70	WD repeat-containing protein 70
1.012	0.979	1.015	0.995	0.978	0.981	0.999	1.081	1.072	0.970	1.001	1.038	Q6RFH5	WDR74	WD repeat-containing protein 74
1.024	1.006	0.973	0.992	1.003	1.011	0.965	1.027	1.060	0.913	1.008	0.960	Q8IWA0	WDR75	WD repeat-containing protein 75
1.088	0.985	1.034	0.997	0.970	0.864	1.121	1.585	0.953	0.939	0.964	0.929	Q9H967	WDR76	WD repeat-containing protein 76
0.990	1.007	1.044	1.015	0.972	1.019	1.039	1.065	1.012	1.052	0.975	1.074	Q562E7	WDR81	WD repeat-containing protein 81
1.019	1.012	0.997	1.045	1.020	0.959	1.047	0.969	0.995	1.038	1.014	1.024	Q6UXN9	WDR82	WD repeat-containing protein 82
									1.064	1.013	1.158	E7ESW6	WDR87	WD repeat-containing protein 87
0.906	0.938	0.951	0.928	0.946	1.087	1.035	1.009	1.046	1.038	1.034	1.115	Q96FK6	WDR89	WD repeat-containing protein 89
0.990	0.992	0.978	1.004	0.956	1.016	1.003	1.003	1.010	1.046	0.959	1.025	A4D1P6	WDR91	WD repeat-containing protein 91
1.034	0.987	0.977	1.004	1.008	1.010	1.122	0.980	0.983	1.044	1.039	1.041	Q96MX6	WDR92	WD repeat-containing protein 92
1.025	0.967	1.003	0.996	0.992	0.991	1.073	1.028	1.039	1.044	1.007	1.054	Q9NXC5	MIOS	WD repeat-containing protein mio
0.960	0.965	1.175	0.956	0.787	1.132	0.894	1.249	1.030	1.031	1.083	0.986	Q9P2S5	WRAP73	WD repeat-containing protein WRAP73
1.006	1.009	1.004	1.022	1.091	0.976	1.024	0.981	1.017	0.964	1.011	0.993	Q2TAY7	SMU1	WD40 repeat-containing protein SMU1
						0.979	1.120	1.123				P30291	WEE1	Wee1-like protein kinase
0.911	0.911	0.944	1.040	0.942	1.041	0.984	1.054	1.049	1.037	0.999	1.029	Q14191	WRN	Werner syndrome ATP-dependent helicase
1.030	1.024	1.013	1.070	0.918	1.040	1.059	1.005	0.970	0.992	1.033	1.159	Q92558	WASF1	Wiskott-Aldrich syndrome protein family member 1
1.000	1.013	1.051	0.986	0.963	1.013	0.935	0.930	0.987	1.014	0.992	1.019	Q9Y6W5	WASF2	Wiskott-Aldrich syndrome protein family member 2
			1.108	0.714	1.005	4.444	0.810	1.388	2.772	0.960	1.768	Q9UPY6	WASF3	Wiskott-Aldrich syndrome protein family member 3
0.965	0.960	1.017	0.923	0.948	0.992	1.015	1.049	1.059	1.060	1.012	1.096	O76024	WFS1	Wolframin
0.985	0.978	1.003	1.008	0.972	1.013	0.987	0.973	0.989	1.021	1.023	1.008	Q9Y2W2	WBP11	WW domain-binding protein 11
1.073	1.089	1.024	1.077	1.025	1.035	1.035	1.139	0.982	1.072	1.059	0.975	Q969T9	WBP2	WW domain-binding protein 2
1.049	0.945	0.977	1.039	0.994	0.971	0.936	0.884	1.001	1.027	1.011	1.050	O75554	WBP4	WW domain-binding protein 4
0.988	1.084	0.989	1.054	1.068	1.019	1.047	1.063	0.995	1.007	1.026	1.119	Q9BTA9	WAC	WW domain-containing adapter protein with coiled-coil
1.075	1.001	0.999	1.026	0.937	0.977	1.023	0.947	1.007	1.202	0.954	1.039	Q9NZC7	WWOX	WW domain-containing oxidoreductase
0.996	0.984	1.051	1.013	0.857	0.832	1.032	0.957	1.006	0.988	0.946	0.919	Q9GZV5	WWTR1	WW domain-containing transcription regulator protein 1
1.000	0.995	0.983	0.976	1.029	0.982	1.019	0.915	1.017	0.978	0.956	1.042	P12955	PEPD	Xaa-Pro dipeptidase
1.085	0.985	1.073	0.935	0.861	1.026				1.301	1.258	1.191	Q9UBH6	XPR1	Xenotropic and polytropic retrovirus receptor 1
			1.045	0.938	0.890	1.023	0.950	0.989	0.991	1.009	1.199	Q92834	RPGR	X-linked retinitis pigmentosa GTPase regulator

Abundance Ratio														
1 d M1/C1	1 d M2/C2	1 d M3/C3	4 d M1/C1	4 d M2/C2	4 d M3/C3	7 d M1/C1	7 d M2/C2	7 d M3/C3	10 d M1/C1	10 d M2/C2	10 d M3/C3	Accession ID	Gene name	Protein Description
0.986	1.003	0.994	0.998	1.040	1.020	0.998	1.029	1.022	0.957	1.023	0.959	P13010	XRCC5	X-ray repair cross-complementing protein 5
1.009	1.009	0.986	0.991	0.995	0.987	0.972	1.023	0.997	0.970	1.017	0.952	P12956	XRCC6	X-ray repair cross-complementing protein 6
			0.958	1.031	1.085				0.676	0.915	0.943	Q9H1B5	XYLT2	Xylosyltransferase 2
1.052	0.996	0.986	0.962	0.954	0.981	1.012	1.004	0.981	0.995	1.008	1.021	O75191	XYLB	Xylulose kinase
0.885	1.111	1.072										Q92536	SLC7A6	Y+L amino acid transporter 2
0.998	0.992	0.993	0.994	1.006	1.027	1.042	0.917	0.986	1.094	1.057	1.115	P16989	YBX3	Y-box-binding protein 3
1.054	1.016	1.076	0.991	0.996	0.996	0.989	1.113	1.062	0.957	0.958	1.159	Q9ULM3	YEATS2	YEATS domain-containing protein 2
1.188	1.128	1.045	1.016	0.950	0.957	0.960	1.036	1.086	0.920	0.949	0.846	O95619	YEATS4	YEATS domain-containing protein 4
1.045	0.984	1.010	1.005	0.941	1.073	1.011	1.157	1.101	1.006	1.033	1.008	Q86U90	YRDC	YrdC domain-containing protein, mitochondrial
0.999	1.014	1.018	0.999	0.990	1.045	1.002	0.930	1.000	1.034	1.009	1.108	Q9BYJ9	YTHDF1	YTH domain-containing family protein 1
1.014	0.992	0.990	1.014	1.007	1.022	1.030	0.975	0.987	1.087	1.031	1.080	Q9Y5A9	YTHDF2	YTH domain-containing family protein 2
1.007	0.989	0.955	0.999	0.927	1.019	1.007	1.069	1.019	1.012	1.027	1.038	A0A087WY31	YTHDF3	YTH domain-containing family protein 3
1.014	1.121	0.940										J3QR07	YTHDC1	YTH domain-containing protein 1
1.000	0.975	0.984	1.010	1.045	1.020	1.010	0.966	1.012	1.007	1.046	1.030	Q96MU7	YTHDC1	YTH domain-containing protein 1
1.148	1.066	1.170	0.851	0.942	1.052	1.150	0.743	0.801	1.001	1.079	0.974	Q9Y2K1	ZBTB1	Zinc finger and BTB domain-containing protein 1
1.020	1.010	0.873	1.014	0.978	1.062	0.963	1.119	1.087	0.856	1.105	1.070	Q96DT7	ZBTB10	Zinc finger and BTB domain-containing protein 10
1.006	0.978	1.108	0.991	0.936	1.097	0.937	0.928	1.033	1.159	0.985	1.115	O95625	ZBTB11	Zinc finger and BTB domain-containing protein 11
			1.147	1.204	1.104				1.048	1.039	0.823	O43829	ZBTB14	Zinc finger and BTB domain-containing protein 14
			1.003	0.789	1.051							Q05516	ZBTB16	Zinc finger and BTB domain-containing protein 16
1.032	0.892	1.095	1.080	0.989	0.864	1.024	1.008	1.004	0.937	0.960	1.056	Q9HC78	ZBTB20	Zinc finger and BTB domain-containing protein 20
1.068	0.952	1.061				0.959	0.988	0.998	0.922	1.265	1.107	Q9ULJ3	ZBTB21	Zinc finger and BTB domain-containing protein 21
						1.048	1.028	1.062	0.955	0.953	1.043	O43167	ZBTB24	Zinc finger and BTB domain-containing protein 24
			1.045	0.986	0.845							A0A0C4DFQ2	ZBTB34	Zinc finger and BTB domain-containing protein 34
			1.048	0.925	0.972							Q9P1Z0	ZBTB4	Zinc finger and BTB domain-containing protein 4
0.937	0.929	0.933	0.693	1.030	1.155	0.907	0.946	0.962	0.939	0.990	0.995	Q9NUA8	ZBTB40	Zinc finger and BTB domain-containing protein 40
			1.145	0.760	0.898				0.945	0.992	0.952	O43298	ZBTB43	Zinc finger and BTB domain-containing protein 43
1.042	0.769	0.809	1.148	1.074	1.010							H0YEM9	ZBTB44	Zinc finger and BTB domain-containing protein 44 (Fragment)
			1.055	1.107	1.106							Q96K62	ZBTB45	Zinc finger and BTB domain-containing protein 45
0.951	1.025	1.008	1.109	1.255	1.127				1.021	1.020	1.025	O95365	ZBTB7A	Zinc finger and BTB domain-containing protein 7A
			1.105	1.018	1.167							Q9Y5A6	ZSCAN21	Zinc finger and SCAN domain-containing protein 21
0.953	0.956	0.995				1.078	0.774	0.924				A0A024RCN4	ZNF187	Zinc finger and SCAN domain-containing protein 26
1.118	1.077	1.056	1.119	1.109	1.215	0.997	1.165	1.167	0.883	1.108	1.167	O96006	ZBED1	Zinc finger BED domain-containing protein 1

Abundance Ratio														
1 d M1/C1	1 d M2/C2	1 d M3/C3	4 d M1/C1	4 d M2/C2	4 d M3/C3	7 d M1/C1	7 d M2/C2	7 d M3/C3	10 d M1/C1	10 d M2/C2	10 d M3/C3	Accession ID	Gene name	Protein Description
						0.967	1.010	1.010				P86452	ZBED6	Zinc finger BED domain-containing protein 6
1.032	1.004	1.014	0.990	0.975	1.003	1.014	1.011	0.999	1.056	1.032	1.054	Q96GY0	ZC2HC1A	Zinc finger C2HC domain-containing protein 1A
1.106	0.995	1.152	0.972	0.886	0.996	1.059	1.053	1.002	1.002	1.006	1.051	O60293	ZFC3H1	Zinc finger C3H1 domain-containing protein
									1.369	1.381	1.299	Q96K80	ZC3H10	Zinc finger CCCH domain-containing protein 10
0.987	0.988	0.998	0.969	0.914	0.990	0.991	0.962	1.009	1.018	0.988	1.053	O75152	ZC3H11A	Zinc finger CCCH domain-containing protein 11A
1.030	0.997	1.037	0.980	0.999	1.043	0.993	0.870	0.954	1.047	0.994	1.050	Q5T200	ZC3H13	Zinc finger CCCH domain-containing protein 13
1.010	1.021	1.018	0.963	0.979	0.978	0.993	0.964	1.006	1.022	0.991	1.030	Q6PJT7	ZC3H14	Zinc finger CCCH domain-containing protein 14
0.973	0.990	1.002	1.032	0.958	1.015	1.017	0.934	1.024	1.003	1.003	1.016	Q8WU90	ZC3H15	Zinc finger CCCH domain-containing protein 15
0.982	0.991	1.011	0.959	0.967	1.020	0.935	0.862	0.982	1.036	1.009	1.050	E7ERS3	ZC3H18	Zinc finger CCCH domain-containing protein 18
1.491	1.109	1.210										Q8IXZ2	ZC3H3	Zinc finger CCCH domain-containing protein 3
1.006	1.000	0.994	1.000	0.971	0.989	0.970	0.974	1.004	0.994	1.046	1.054	Q9UPT8	ZC3H4	Zinc finger CCCH domain-containing protein 4
1.032	1.027	1.031	1.022	1.000	0.975	1.016	1.012	1.052	1.034	1.000	1.122	Q8IWR0	ZC3H7A	Zinc finger CCCH domain-containing protein 7A
1.077	1.058	1.039	1.035	0.969	0.991	1.062	1.026	1.026	1.061	0.992	1.065	Q9UGR2	ZC3H7B	Zinc finger CCCH domain-containing protein 7B
0.774	0.981	0.965										A0A0C4DGZ1	ZC3H8	Zinc finger CCCH domain-containing protein 8
0.981	0.998	1.014	0.988	0.983	1.006	0.983	0.977	0.979	0.999	1.042	1.006	Q7Z2W4	ZC3HAV1	Zinc finger CCCH-type antiviral protein 1
1.017	1.025	1.032	0.991	1.018	0.979	1.018	0.856	1.008	1.053	1.029	1.050	Q96H79	ZC3HAV1L	Zinc finger CCCH-type antiviral protein 1-like
			1.018	0.934	0.906							Q8TBK6	ZCCHC10	Zinc finger CCHC domain-containing protein 10
0.999	1.017	0.974	0.989	0.932	1.028	1.028	1.071	1.049	1.013	0.957	1.001	Q9NUD5	ZCCHC3	Zinc finger CCHC domain-containing protein 3
0.883	0.806	0.978				0.893	1.155	0.987	0.845	0.971	0.914	Q9H5U6	ZCCHC4	Zinc finger CCHC domain-containing protein 4
1.013	1.051	1.082	0.937	0.898	0.980	0.951	1.022	0.981				Q8N3Z6	ZCCHC7	Zinc finger CCHC domain-containing protein 7
1.054	1.010	0.996	0.985	0.947	0.969	1.018	1.067	1.016	1.004	1.003	1.036	Q6NZY4	ZCCHC8	Zinc finger CCHC domain-containing protein 8
1.037	1.047	1.194	1.004	1.045	0.996	0.972	0.898	1.071	1.056	1.003	1.161	Q8N567	ZCCHC9	Zinc finger CCHC domain-containing protein 9
1.065	1.158	1.110	0.963	1.126	0.964	1.006	1.159	0.961	0.966	1.144	1.056	Q8TBF4	ZCRB1	Zinc finger CCHC-type and RNA-binding motif-containing protein 1
0.974	0.980	1.042	0.904	1.024	1.050	0.864	1.010	1.055	0.912	1.179	0.882	G3V5N8	ZFYVE1	Zinc finger FYVE domain-containing protein 1
0.978	0.986	0.992	0.980	0.907	1.003	1.000	1.065	0.983	1.007	0.996	1.050	Q7Z3T8	ZFYVE16	Zinc finger FYVE domain-containing protein 16
1.038	1.007	1.012	0.985	0.966	1.039	1.017	1.086	1.064	0.972	0.974	1.024	Q68DK2	ZFYVE26	Zinc finger FYVE domain-containing protein 26
1.135	1.205	0.897	1.351	0.729	0.842	0.922	1.225	1.124				O95405	ZFYVE9	Zinc finger FYVE domain-containing protein 9
0.964	0.986	1.097	1.052	1.018	1.147	1.114	0.966	1.053	1.202	1.114	1.128	Q9UHR6	ZNHIT2	Zinc finger HIT domain-containing protein 2
1.055	0.986	0.989	1.020	0.883	1.074	0.978	0.915	0.990	1.015	0.992	1.002	Q96NC0	ZMAT2	Zinc finger matrin-type protein 2
1.014	1.036	1.249	1.023	1.064	1.032	0.953	0.942	1.044	0.996	1.003	1.142	Q9ULJ6	ZMIZ1	Zinc finger MIZ domain-containing protein 1
0.990	1.006	1.022				0.965	1.169	1.052				A0A087X127	ZMIZ2	Zinc finger MIZ domain-containing protein 2
1.025	1.045	1.039	1.073	0.916	1.044	0.898	1.133	1.132	0.882	1.009	1.060	Q5SVZ6	ZMYM1	Zinc finger MYM-type protein 1
1.060	0.954	1.016	0.885	0.880	0.991	0.872	0.923	1.051	0.995	0.985	1.041	Q9UBW7	ZMYM2	Zinc finger MYM-type protein 2

Abundance Ratio														
1 d M1/C1	1 d M2/C2	1 d M3/C3	4 d M1/C1	4 d M2/C2	4 d M3/C3	7 d M1/C1	7 d M2/C2	7 d M3/C3	10 d M1/C1	10 d M2/C2	10 d M3/C3	Accession ID	Gene name	Protein Description
0.994	0.972	1.014	0.979	1.041	0.995	0.969	1.064	1.005	0.977	0.998	0.992	A6NHB5	ZMYM3	Zinc finger MYM-type protein 3
0.986	0.974	1.029	0.936	0.926	0.948	0.916	0.899	0.941	0.937	1.032	1.002	Q5VZL5	ZMYM4	Zinc finger MYM-type protein 4
1.005	0.937	1.058	1.019	0.958	1.017	1.024	0.918	0.882	0.969	1.014	1.002	Q15326	ZMYND11	Zinc finger MYND domain-containing protein 11
1.062	1.231	1.344										Q9H2Y7	ZNF106	Zinc finger protein 106
						1.047	1.000	0.933	1.248	1.025	1.244	Q8NC26	ZNF114	Zinc finger protein 114
1.420	0.818	0.949	1.341	0.823	1.149	1.354	1.001	1.268	1.108	1.006	0.969	P58317	ZNF121	Zinc finger protein 121
1.024	0.953	1.036	0.997	0.936	1.031	0.982	0.934	1.046	1.035	0.976	0.975	P52739	ZNF131	Zinc finger protein 131
0.949	0.989	1.000	1.027	1.162	1.022	1.063	1.069	0.941	1.001	1.040	1.088	P52747	ZNF143	Zinc finger protein 143
1.041	1.001	1.036	1.021	0.939	1.022	1.035	0.975	1.007	1.013	1.040	1.032	Q9UQR1	ZNF148	Zinc finger protein 148
			1.412	1.175	1.282							Q99676	ZNF184	Zinc finger protein 184
1.028	0.995	0.980	1.011	0.989	0.950	1.008	0.862	0.930	1.039	0.983	1.021	O15231	ZNF185	Zinc finger protein 185
1.034	0.942	0.998	1.005	1.060	1.023	1.281	0.972	0.938	1.021	0.917	1.061	O75362	ZNF217	Zinc finger protein 217
0.922	1.001	1.361	0.812	1.003	0.996							P17026	ZNF22	Zinc finger protein 22
0.960	0.993	0.994	1.035	0.918	0.931	1.014	1.034	1.033	1.034	1.024	1.021	P17028	ZNF24	Zinc finger protein 24
1.213	0.930	1.064	1.252	0.973	1.002	0.822	1.111	1.176	0.944	1.005	1.013	O43296	ZNF264	Zinc finger protein 264
			0.961	0.821	1.108	0.963	0.965	0.958				Q14587	ZNF268	Zinc finger protein 268
						0.933	1.159	1.165	1.026	0.979	0.952	Q9NRM2	ZNF277	Zinc finger protein 277
0.945	1.098	0.990				0.771	0.683	0.495				P17035	ZNF28	Zinc finger protein 28
1.031	1.013	1.003	1.033	0.967	1.012	1.048	0.953	1.025	1.010	1.021	1.031	Q8ND82	ZNF280C	Zinc finger protein 280C
0.971	0.985	1.005										Q6N043	ZNF280D	Zinc finger protein 280D
1.028	0.991	1.019	1.016	0.981	1.013	1.001	1.033	0.972	0.981	1.043	0.977	Q9Y2X9	ZNF281	Zinc finger protein 281
0.988	0.959	1.066	0.951	0.977	0.892	0.867	0.898	0.896	0.999	1.100	1.069	O60281	ZNF292	Zinc finger protein 292
1.027	1.078	1.027	0.917	0.867	1.214	0.861	1.083	1.133	1.128	0.717	1.090	A0A0D9SFG5	ZNF302	Zinc finger protein 302
1.263	0.970	1.039	1.041	0.991	1.097	1.013	0.853	1.033	1.174	1.048	1.184	A6NFI3	ZNF316	Zinc finger protein 316
1.041	0.971	1.042	0.998	0.957	0.978	1.054	1.201	0.944	1.053	0.996	1.062	Q5VUA4	ZNF318	Zinc finger protein 318
						0.776	0.746	0.983	1.159	0.794	1.156	A2RRD8	ZNF320	Zinc finger protein 320
0.823	1.068	1.199	0.942	0.989	1.134	1.136	0.861	1.102	1.003	1.021	1.044	Q9Y3S2	ZNF330	Zinc finger protein 330
						0.899	1.020	1.049				Q9H4Z2	ZNF335	Zinc finger protein 335
1.638	0.706	1.096										A0A0A0MR11	ZNF33A	Zinc finger protein 33A
0.894	1.076	0.994	0.993	0.962	0.992	0.980	1.003	0.946	0.943	1.045	0.975	Q9UL40	ZNF346	Zinc finger protein 346
1.006	0.935	1.103										Q96PM9	ZNF385A	Zinc finger protein 385A
						1.172	1.130	0.917				Q9H8N7	ZNF395	Zinc finger protein 395
			1.060	0.915	0.983				0.895	1.081	0.905	C9JLG1	HIVEP1	Zinc finger protein 40 (Fragment)
									0.983	0.946	1.000	P15822	HIVEP1	Zinc finger protein 40

Abundance Ratio														
1 d M1/C1	1 d M2/C2	1 d M3/C3	4 d M1/C1	4 d M2/C2	4 d M3/C3	7 d M1/C1	7 d M2/C2	7 d M3/C3	10 d M1/C1	10 d M2/C2	10 d M3/C3	Accession ID	Gene name	Protein Description
1.049	0.899	0.908				1.353	0.708	1.230	0.922	0.906	1.120	M0QXZ5	ZNF428	Zinc finger protein 428 (Fragment)
1.126	1.038	1.004										P59923	ZNF445	Zinc finger protein 445
1.047	0.929	1.104				1.072	1.262	1.265				Q9NWS9	ZNF446	Zinc finger protein 446
1.024	0.896	1.117	0.903	0.976	1.372							Q14592	ZNF460	Zinc finger protein 460
						0.963	1.003	0.930				Q8NCK3	ZNF485	Zinc finger protein 485
0.966	1.014	1.026	1.008	0.998	1.011	1.061	0.925	0.942	1.116	0.962	1.067	Q96ME7	ZNF512	Zinc finger protein 512
1.098	1.084	1.078	1.014	0.974	0.922	0.944	0.976	0.969	0.965	1.113	0.918	Q96KM6	ZNF512B	Zinc finger protein 512B
0.932	0.858	0.942	1.188	0.944	1.540	1.071	1.098	1.159	0.906	1.157	1.100	Q92618	ZNF516	Zinc finger protein 516
						1.361	0.943	0.869	1.049	1.114	0.968	Q96C55	ZNF524	Zinc finger protein 524
1.103	0.826	1.550										Q8TF50	ZNF526	Zinc finger protein 526
0.990	1.060	0.938	0.992	1.066	0.995	1.049	1.134	1.016	1.053	1.027	0.891	Q9HCE3	ZNF532	Zinc finger protein 532
			0.830	1.083	1.079							Q86YE8	ZNF573	Zinc finger protein 573
0.928	1.114	1.031										Q96N58	ZNF578	Zinc finger protein 578
1.050	0.924	1.033	1.024	0.919	0.952	0.989	0.864	0.943	1.079	0.946	1.050	Q8NAF0	ZNF579	Zinc finger protein 579
0.729	1.058	1.137										Q9UK33	ZNF580	Zinc finger protein 580
									1.117	1.258	1.441	Q96SQ5	ZNF587	Zinc finger protein 587
1.065	0.978	0.979	1.028	0.968	0.945				0.926	0.939	1.033	Q92610	ZNF592	Zinc finger protein 592
1.006	0.950	1.046	0.970	1.004	1.049	1.027	1.061	0.996	1.034	1.069	1.030	O00488	ZNF593	Zinc finger protein 593
1.032	0.943	1.193	1.013	0.916	1.028	1.026	1.008	0.988	1.135	1.079	1.034	O15014	ZNF609	Zinc finger protein 609
1.302	0.786	0.955				0.779	0.533	1.020				Q08AN1	ZNF616	Zinc finger protein 616
1.033	0.994	1.140	1.078	0.954	0.954	0.974	0.965	0.870	1.217	0.998	0.994	Q5T7W0	ZNF618	Zinc finger protein 618
									1.135	1.072	0.857	Q6ZSS3	ZNF621	Zinc finger protein 621
1.019	0.991	1.000	0.988	0.968	1.006	1.022	0.949	0.976	1.013	1.007	1.073	Q969S3	ZNF622	Zinc finger protein 622
0.957	1.138	0.999	1.014	0.876	1.015	0.971	1.408	1.065	0.936	1.173	0.803	Q9UEG4	ZNF629	Zinc finger protein 629
1.032	0.993	1.022	0.983	0.950	0.960	0.973	0.935	0.980	1.000	0.999	1.027	Q14966	ZNF638	Zinc finger protein 638
									0.887	1.002	1.073	Q9UID6	ZNF639	Zinc finger protein 639
0.925	0.858	1.083	0.977	0.905	0.813				0.700	1.181	1.007	Q9NPA5	ZFP64	Zinc finger protein 64 homolog, isoforms 1 and 2
0.980	1.079	0.946	0.968	1.044	0.976	1.103	0.991	0.950	1.033	1.157	0.999	Q9NTW7	ZFP64	Zinc finger protein 64 homolog, isoforms 3 and 4
1.135	0.995	1.145	1.001	0.894	1.082	0.979	1.192	0.986	1.049	1.201	0.903	Q9H582	ZNF644	Zinc finger protein 644
1.027	0.996	1.042	1.286	0.852	1.074	1.047	1.038	1.011	1.034	0.914	1.038	A0A1B0GWA0	ZNF654	Zinc finger protein 654
									0.822	1.004	0.876	Q96BR6	ZNF669	Zinc finger protein 669
1.002	1.021	1.066	0.969	0.975	1.115	1.020	1.081	1.005	1.064	0.976	1.108	Q8N1G0	ZNF687	Zinc finger protein 687
			0.958	1.010	0.966							Q9H7X3	ZNF696	Zinc finger protein 696
1.898	0.951	1.030	1.135	1.725	0.854	1.114	0.698	1.014	0.967	0.979	0.910	Q9Y5V0	ZNF706	Zinc finger protein 706

Abundance Ratio														
1 d M1/C1	1 d M2/C2	1 d M3/C3	4 d M1/C1	4 d M2/C2	4 d M3/C3	7 d M1/C1	7 d M2/C2	7 d M3/C3	10 d M1/C1	10 d M2/C2	10 d M3/C3	Accession ID	Gene name	Protein Description
			0.876	1.032	0.955	1.109	1.115	0.663				Q8NDX6	ZNF740	Zinc finger protein 740
1.126	1.012	0.998	1.042	1.049	0.957	0.954	0.750	0.877	1.050	0.991	1.009	Q9H5H4	ZNF768	Zinc finger protein 768
1.041	1.025	0.921	1.042	0.978	1.073	1.084	0.983	1.092	1.159	1.146	1.188	A0A087WUD1	ZNF787	Zinc finger protein 787
0.924	0.928	1.064	0.846	1.101	0.984	0.950	0.687	0.936	1.205	0.943	1.015	P17098	ZNF8	Zinc finger protein 8
0.994	1.044	0.983	0.989	0.965	1.029	0.984	0.938	0.994	1.023	0.999	1.063	Q2TB10	ZNF800	Zinc finger protein 800
									0.990	0.913	0.996	Q5CZA5	ZNF805	Zinc finger protein 805
0.988	1.008	0.998	0.953	0.932	1.001	1.026	0.884	1.038	1.095	1.045	1.118	Q6ZN06	ZNF813	Zinc finger protein 813
1.166	1.416	0.871										Q17R98	ZNF827	Zinc finger protein 827
0.960	0.977	1.036	0.983	0.896	1.002	0.990	0.992	1.034	0.927	1.040	1.099	Q96NB3	ZNF830	Zinc finger protein 830
0.931	0.906	1.077	0.836	1.192	1.015	0.864	0.582	0.965	0.959	1.059	1.054	Q96IR2	ZNF845	Zinc finger protein 845
			1.083	0.865	0.951							P0CJ78	ZNF865	Zinc finger protein 865
1.009	0.957	0.882	0.925	0.935	0.982				1.144	1.137	1.030	P0CJ79	ZNF888	Zinc finger protein 888
0.988	1.041	1.200				1.161	1.023	1.096	1.059	0.983	0.994	P35789	ZNF93	Zinc finger protein 93
						1.070	1.393	1.127	1.100	0.976	1.048	Q92784	DPF3	Zinc finger protein DPF3
									0.914	0.868	1.067	Q15072	ZNF146	Zinc finger protein OZF
1.019	0.900	1.009	0.983	0.936	1.041	1.070	1.070	1.008	1.005	0.954	1.022	P14373	TRIM27	Zinc finger protein RFP
1.055	0.854	0.923	1.199	1.020	0.974							Q13129	RLF	Zinc finger protein Rlf
0.971	0.977	0.947	0.992	0.986	0.970	1.018	0.988	0.979	1.025	1.007	1.053	J3KMZ8	DPF2	Zinc finger protein ubi-d4
1.075	1.045	0.988	1.074	1.043	1.160	0.988	1.063	1.031	1.052	1.135	1.058	P17029	ZKSCAN1	Zinc finger protein with KRAB and SCAN domains 1
			1.037	0.903	1.109	0.915	1.030	1.003				Q969J2	ZKSCAN4	Zinc finger protein with KRAB and SCAN domains 4
1.178	1.013	1.099	1.051	0.974	1.025	1.056	1.061	0.938	1.102	1.011	1.085	Q15776	ZKSCAN8	Zinc finger protein with KRAB and SCAN domains 8
			0.947	0.889	1.041							Q8IX07	ZFPM1	Zinc finger protein ZFPM1
1.032	0.997	1.014	1.014	0.992	1.084	1.028	1.068	1.044	1.035	1.027	1.058	O75312	ZPR1	Zinc finger protein ZPR1
			0.954	1.320	0.808	1.044	1.049	1.004	0.949	1.050	0.984	O95159	ZFPL1	Zinc finger protein-like 1
1.063	1.006	1.001	1.023	1.305	0.975	1.056	0.806	1.033	1.026	1.015	1.051	O95218	ZRANB2	Zinc finger Ran-binding domain-containing protein 2
1.001	1.019	1.010	1.003	1.002	0.997	0.984	0.984	1.000	1.011	1.017	1.000	Q96KR1	ZFR	Zinc finger RNA-binding protein
1.048	1.004	0.995	1.004	0.952	1.002	1.049	1.033	1.004	1.009	1.035	1.040	O43149	ZZEF1	Zinc finger ZZ-type and EF-hand domain-containing protein 1
1.022	0.988	1.025	0.876	0.851	1.340							Q9UKY1	ZHX1	Zinc fingers and homeoboxes protein 1
1.103	1.103	0.902	1.015	1.123	1.020	1.057	0.932	1.049				Q9Y6X8	ZHX2	Zinc fingers and homeoboxes protein 2
0.915	0.748	1.307	1.139	1.172	1.456				1.007	0.999	0.833	Q9H4I2	ZHX3	Zinc fingers and homeoboxes protein 3
0.978	1.029	1.023	1.074	1.041	1.098	1.070	1.021	1.065	1.045	1.016	1.061	Q9BQ52	ELAC2	Zinc phosphodiesterase ELAC protein 2

Abundance Ratio														
1 d M1/C1	1 d M2/C2	1 d M3/C3	4 d M1/C1	4 d M2/C2	4 d M3/C3	7 d M1/C1	7 d M2/C2	7 d M3/C3	10 d M1/C1	10 d M2/C2	10 d M3/C3	Accession ID	Gene name	Protein Description
1.008	0.977	1.005	1.031	0.958	1.070	1.063	1.062	1.101	1.036	1.013	1.088	Q9Y6M5	SLC30A1	Zinc transporter 1
0.941	1.028	0.985	0.931	0.856	0.973	0.941	1.176	0.961	1.026	1.053	0.985	Q8TAD4	SLC30A5	Zinc transporter 5
1.098	0.807	0.989	1.112	0.874	1.086	1.103	0.959	1.027	1.143	1.092	0.988	Q8NEW0	SLC30A7	Zinc transporter 7
0.928	0.960	0.994	0.983	0.839	0.985	1.003	1.023	1.006	1.074	0.979	1.088	Q6PML9	SLC30A9	Zinc transporter 9
0.973	1.038	0.965	0.985	0.894	0.953	0.862	0.798	1.027	1.006	1.012	0.981	Q92504	SLC39A7	Zinc transporter SLC39A7
1.009	0.835	1.066	0.953	0.896	1.160	1.074	0.934	1.083	1.333	1.181	1.232	Q9NY26	SLC39A1	Zinc transporter ZIP1
1.106	0.956	1.146	1.102	0.830	1.276	0.962	1.155	0.963				Q9ULF5	SLC39A10	Zinc transporter ZIP10
0.962	0.964	0.954	0.981	0.940	1.032	1.026	0.929	1.040	1.017	0.882	1.071	Q8N1S5	SLC39A11	Zinc transporter ZIP11
1.001	1.045	1.079	1.127	0.899	1.194				1.260	1.072	0.766	Q15043	SLC39A14	Zinc transporter ZIP14
0.931	0.999	0.909	0.930	0.956	1.088				0.980	0.998	1.078	Q13433	SLC39A6	Zinc transporter ZIP6
						1.095	1.149	0.970	1.143	1.002	1.337	H7BY64	ZNF511-PRAP1	ZNF511-PRAP1 readthrough (Fragment)
1.002	1.042	1.051							1.017	0.976	1.026	O95229	ZWINT	ZW10 interactor
1.272	1.131	1.044	1.012	0.888	0.761	0.890	1.125	0.943	0.875	0.953	0.834	H0Y2Y8	ZYX	Zyxin (Fragment)
1.027	1.034	0.995	1.007	1.095	0.938	1.031	0.991	0.974	0.989	0.996	1.014	Q15942	ZYX	Zyxin
0.677	0.936	0.928	0.951	0.868	1.038				0.781	1.106	1.007	Q8IYH5	ZZZ3	ZZ-type zinc finger-containing protein 3

C, control cell; M, MAG-treated cell.
