# Supplementary material for: Bacterial Magnetosomes Release Iron Ions and Induce Regulation of Iron Homeostasis in Endothelial Cells
Source: Nanomaterials (Basel). 2022 Nov 13;12(22):3995. doi: 10.3390/nano12223995 (PMC9695978; doi:10.3390/nano12223995)
Supplement: Supplementary file 1 [file nanomaterials-12-03995-s001.zip › Table S6.pdf]

Table S6. The ratios of 158 differential expressed proteins identified by LC-MS/MS from MAG-treated ECs at 4 time point.

1 d log(2)Avg. (M/C)	4 d log(2)Avg. (M/C)	7 d log(2)Avg. (M/C)	10 d log(2)Avg. (M/C)	Gene name	Protein Description
0.347	0.186	0.057		ABHD4	Protein ABHD4
0.269				ANTXR2	Anthrax toxin receptor 2
0.431	0.313	-0.073	-0.108	AP1B1	Isoform C of AP-1 complex subunit beta-1
0.329			0.098	AP4M1	AP-4 complex subunit mu-1
	0.350	0.410		APOC1	Apolipoprotein C-I
0.343				AREL1	Apoptosis-resistant E3 ubiquitin protein ligase 1
-0.023	0.299	-0.252	0.074	ATRAID	Isoform 3 of All-trans retinoic acid-induced differentiation factor
1.175	1.002	1.500		BAZ2B	Bromodomain adjacent to zinc finger domain protein 2B (Fragment)
-0.272	-0.063	0.239		BCL2L12	Bcl-2-like protein 12
0.005	-0.029	0.090	0.293	C12orf43	Chromosome 12 open reading frame 43, isoform CRA_a
-0.177	-0.024	0.281	0.046	C8orf76	Uncharacterized protein C8orf76
	-0.014	-0.288	0.182	C9orf72	Guanine nucleotide exchange C9orf72
0.604	-0.200	-0.580	-1.389	CANX	Calnexin (Fragment)
0.065	0.305	0.132	0.043	CCL20	C-C motif chemokine 20
	0.329	-0.085	0.038	CD40	Tumor necrosis factor receptor superfamily member 5
-0.112	0.131	0.213	0.301	CD63	CD63 antigen
0.103	0.269			CEBPD	CCAAT/enhancer-binding protein delta
-0.265	0.229	0.305		CEP78	Isoform 2 of Centrosomal protein of 78 kDa
0.244	-0.019	-0.529		CFAP97	Cilia- and flagella-associated protein 97
-0.014	0.035	0.167	0.273	CLCN5	Isoform 2 of H(+)/Cl(-) exchange transporter 5
		-0.302	0.064	CLEC4E	C-type lectin domain family 4 member E
0.130	0.376	-0.183	0.066	CNBP	Cellular nucleic acid-binding protein
-0.321				CNTRL	Centriolin
-0.057	0.247	0.138	0.387	CPQ	Carboxypeptidase Q
0.112	-0.079	-0.220	-0.545	DBF4B	Protein DBF4 homolog B
0.009	0.120	0.217	0.286	DNASE2	Deoxyribonuclease-2-alpha
-0.020	-0.116	0.050	0.281	DNM1L	Isoform 4 of Dynamin-1-like protein
0.144	0.122	0.188	0.290	DPYD	Dihydropyrimidine dehydrogenase [NADP(+)]

1 d log(2)Avg. (M/C)	4 d log(2)Avg. (M/C)	7 d log(2)Avg. (M/C)	10 d log(2)Avg. (M/C)	Gene name	Protein Description
	0.412	0.077	0.045	EIF1B	Eukaryotic translation initiation factor 1b
0.305	-0.207			ELF2	ETS-related transcription factor Elf-2
-0.185	-0.005	0.153	0.296	EMP3	Epithelial membrane protein 3
0.319	-0.129	-0.001	-0.079	ERBB3	Receptor tyrosine-protein kinase erbB-3
0.135	0.313			FAM161A	Isoform 3 of Protein FAM161A
-0.299	-0.068	0.042		FAM173B	Protein FAM173B
-0.136	-0.019	0.194	0.267	FBRSL1	Fibrosin-1-like protein
0.271		0.069	0.099	FBXO28	F-box only protein 28
0.081	0.141	0.285	0.024	FEZ1	Fasciculation and elongation protein zeta-1
0.046	-0.274	0.044	-0.060	FKRP	Fukutin-related protein
-0.085	0.147	0.146	0.496	FOXH1	Forkhead box protein H1
1.241	1.591	1.581	1.410	FTH1	Ferritin heavy chain
1.212	1.553	1.516	1.349	FTL	Ferritin light chain
-0.151	-0.293	-0.020	0.054	GALNT1	Polypeptide N-acetylgalactosaminyltransferase 1
-0.053	-0.295	-0.216	-0.189	GBA	Glucosylceramidase
0.192	0.301	0.280	0.192	GLT8D1	Glycosyltransferase 8 domain-containing protein 1
0.088	0.438	-0.079	-0.061	H3F3A	Histone H3.3
0.429	0.012	-0.275	-0.348	HIGD1A	Isoform 2 of HIG1 domain family member 1A, mitochondrial
0.137	0.022	-0.283	-0.152	HIGD2A	HIG1 domain family member 2A, mitochondrial
0.321	0.476	0.013	-0.169	HIST2H3A	Histone H3.2
-0.036	-0.230	-0.444	-0.192	HMGA1	Isoform HMG-Y of High mobility group protein HMG-I/HMG-Y
-0.236	-0.372	-0.864	-0.277	HMGN2	Non-histone chromosomal protein HMG-17
-0.149	-0.479	-0.831	-0.340	HMGN4	High mobility group nucleosome-binding domain-containing protein 4
0.070	0.149	0.198	0.358	HPSE	Heparanase
-0.121	-0.288	-0.462	0.265	HRNR	Hornerin
-0.112	0.026	0.134	0.294	HSPA8	Heat shock cognate 71 kDa protein (Fragment)
	0.324	-0.134		ING2	Inhibitor of growth protein 2
-0.352	-0.279	0.211	-0.070	IREB2	Iron-responsive element-binding protein 2
			0.324	ITGB8	Integrin beta-8

1 d log(2)Avg. (M/C)	4 d log(2)Avg. (M/C)	7 d log(2)Avg. (M/C)	10 d log(2)Avg. (M/C)	Gene name	Protein Description
-0.012	-0.041	-0.633	-0.070	KIF15	Kinesin-like protein KIF15
0.349	-0.041	-0.012	0.080	KLHL36	Kelch-like protein 36
0.162	0.355	0.008	0.065	KLHL7	Kelch-like protein 7
			0.589	LAPTM4A	Lysosomal-associated transmembrane protein 4A
0.047	-0.282	-0.360	-0.595	LBP	Lipopolysaccharide-binding protein
-0.069	0.140	0.379	0.584	LGMN	Legumain
	-0.102	0.358	0.204	LGR5	Leucine-rich repeat-containing G-protein coupled receptor 5
0.035	0.220	0.545	0.557	LIPA	Lysosomal acid lipase/cholesteryl ester hydrolase
0.054	0.164	0.414	0.222	LLPH	Protein LLP homolog
0.332	-0.023		0.088	LOC401307	HCG19535
		0.304	0.309	LRP8	Low-density lipoprotein receptor-related protein 8
		-0.651		LTF	Lactotransferrin
		0.057	0.370	MAP4	Microtubule-associated protein 4
0.062	0.034	-0.101	-0.390	MAX	Protein max
0.448	-0.477	-0.064		MBNL1	Isoform 5 of Muscleblind-like protein 1
0.253	-0.278			MBTPS1	Membrane-bound transcription factor site-1 protease
		-0.304		MCM9	DNA helicase MCM9
0.277	-0.041	-0.059	-0.047	MICALL2	MICAL-like protein 2
0.319	0.432	0.046	0.042	MINOS1	MICOS complex subunit MIC10
-0.111	-0.369		0.005	MPP1	55 kDa erythrocyte membrane protein
0.017	-0.076	0.041	0.320	MYH14	Isoform 2 of Myosin-14
			-0.498	MYL6	Myosin light polypeptide 6
	0.165	0.314	-0.023	MZT2A	Mitotic-spindle organizing protein 2A
-0.029	0.099	0.210	0.359	NACA2	Nascent polypeptide-associated complex subunit alpha-2
-0.020	0.006	0.351	0.160	NATD1	Protein NATD1
0.156	-0.145	-0.388	0.185	NDUFB2	NADH dehydrogenase [ubiquinone] 1 beta subcomplex subunit 2, mitochondrial
0.178	-0.099		0.385	NFIX	Nuclear factor 1 X-type
-0.388		0.100	0.234	NOTCH1	Neurogenic locus notch homolog protein 1
			0.288	NRXN1	Isoform 3a of Neurexin-1

1 d log(2)Avg. (M/C)	4 d log(2)Avg. (M/C)	7 d log(2)Avg. (M/C)	10 d log(2)Avg. (M/C)	Gene name	Protein Description
0.301				NSFL1C	Isoform 2 of NSFL1 cofactor p47
	-0.240	-0.450		NUPR1	Isoform 2 of Nuclear protein 1
1.508	2.295	2.324	2.209	ODAPH	Isoform 2 of odontogenesis associated phosphoprotein
0.002	-0.021	0.370		OTUB2	Ubiquitin thioesterase OTUB2
-0.006	0.309	0.063	0.087	PCTP	Phosphatidylcholine transfer protein
-0.021	0.090	0.094	-0.262	PKN3	Serine/threonine-protein kinase N3
1.375	-0.078	0.148	0.173	PLK1	Serine/threonine-protein kinase PLK1
-0.051	0.435	-0.120	0.084	PLP2	Proteolipid protein 2
-0.108	-0.079	0.308	0.388	PLPP1	Phospholipid phosphatase 1
0.458	0.236		0.063	PLPP2	Isoform 2 of Phospholipid phosphatase 2
0.206	0.333	0.246	0.060	PNMA1	Paraneoplastic antigen Ma1
	0.098	0.425	-0.003	PODN	Isoform 3 of Podocan
-0.163	-0.078	0.009	0.362	PPP1R16A	Protein phosphatase 1 regulatory subunit 16A
0.031	0.121	0.259	0.300	PPT1	Palmitoyl-protein thioesterase 1
0.056	0.025	-0.035	0.288	PPTC7	Protein phosphatase PTC7 homolog
-0.101		0.469		PRELID3B	PRELI domain containing protein 3B
0.303		0.003		PRG4	Proteoglycan 4
-0.240	0.090	0.090	0.265	PSAP	Prosaposin
0.026	0.051	0.053	0.369	PTTG1IP	Pituitary tumor-transforming gene 1 protein-interacting protein
	0.143	-0.546		PYGO1	Pygopus homolog 1
-0.020	-0.395	-0.051	0.004	RABIF	Guanine nucleotide exchange factor MSS4
-0.095	-0.139	-0.414	-0.345	RBBP5	Isoform 2 of Retinoblastoma-binding protein 5
0.379	0.010	0.361		RBM26	Isoform 2 of RNA-binding protein 26
0.475	0.320	-0.096	-0.078	RBX1	E3 ubiquitin-protein ligase RBX1
-0.044	0.129	0.147	0.310	RNF13	E3 ubiquitin-protein ligase RNF13
			0.706	RNH1	Ribonuclease inhibitor (Fragment)
0.212	0.348	-0.108	-0.177	RPS29	Isoform 2 of 40S ribosomal protein S29
0.167	0.327	0.298	0.185	SAA1	Serum amyloid A-1 protein
0.035	0.164	0.252	0.301	SCARB1	Scavenger receptor class B member 1
0.214	0.325	0.070	0.113	SDF4	45 kDa calcium-binding protein

1 d log(2)Avg. (M/C)	4 d log(2)Avg. (M/C)	7 d log(2)Avg. (M/C)	10 d log(2)Avg. (M/C)	Gene name	Protein Description
-0.268	-0.112	-0.275	0.061	SDHAF2	Succinate dehydrogenase assembly factor 2, mitochondrial
2.716	1.883		1.927	SH3TC2	SH3 domain and tetratricopeptide repeat-containing protein 2
0.110	0.006	0.324	0.074	SHKBP1	SH3KBP1-binding protein 1
0.280	-0.006	0.206	-0.116	SIX4	Homeobox protein SIX4
-0.050	-0.388	-0.037	-0.096	SLC11A2	Isoform 4 of Natural resistance-associated macrophage protein 2
0.416		0.109		SLC35B4	UDP-xylose and UDP-N-acetylglucosamine transporter
	-0.238	-0.297		SLC35D1	UDP-glucuronic acid/UDP-N-acetylgalactosamine transporter
	0.095	0.120	0.350	SLC36A1	Proton-coupled amino acid transporter 1
-0.052	-0.005	0.040	0.319	SLC39A1	Zinc transporter ZIP1
-0.020	-0.288	0.021	0.070	SOGA3	Protein SOGA3
0.259	-0.182	0.607		SPA17	Sperm surface protein Sp17
	0.010	0.124	0.317	SPATA2	Spermatogenesis-associated protein 2
0.238	0.619	0.065	0.144	SRI	Sorcin
-0.045	0.604	-0.053		SSNA1	Sjoegren syndrome nuclear autoantigen 1
0.041	0.706	0.040	-0.121	SUGT1	Isoform 2 of Protein SGT1 homolog
0.382	0.132	-0.166	-0.262	SUPT4H1	Transcription elongation factor SPT4
		0.330	-0.097	TAPBPL	Isoform zeta of Tapasin-related protein
0.055	0.094		0.362	TBC1D16	TBC1 domain family member 16
0.103	0.394		-0.032	TEAD3	Transcriptional enhancer factor TEF-5
-0.326	-0.769	-0.580	-0.590	TFRC	Transferrin receptor protein 1
0.039	-0.096	-0.001	-0.346	TGFBI	Transforming growth factor-beta-induced protein ig-h3
0.012	0.229	0.345		TM7SF3	Transmembrane 7 superfamily member 3
-0.154	-0.046	0.264	0.092	TMEM267	Transmembrane protein 267
0.534	0.042	0.132	0.342	TPH1	Isoform 2 of Tryptophan 5-hydroxylase 1
0.059	-0.053	-0.418	0.133	TRAFD1	TRAF-type zinc finger domain-containing protein 1
-0.303	-0.007	-0.001	0.083	TSEN15	tRNA-splicing endonuclease subunit Sen15
-0.032	0.299	0.248	0.424	TSPAN3	Tetraspanin-3
			0.285	TSPAN31	Tetraspanin-31
0.012	0.077	0.286		TUFT1	Tuftelin
0.249	0.378	-0.195	-0.064	UBA52	Ubiquitin-60S ribosomal protein L40

1 d log(2)Avg. (M/C)	4 d log(2)Avg. (M/C)	7 d log(2)Avg. (M/C)	10 d log(2)Avg. (M/C)	Gene name	Protein Description
	-0.297	-0.378	0.395	UPF3A	Regulator of nonsense transcripts 3A
0.320	0.394	-0.166	0.073	UQCRH	Cytochrome b-c1 complex subunit 6, mitochondrial
0.012	0.006	0.135	0.276	USP13	Ubiquitin carboxyl-terminal hydrolase 13
0.011		-0.373	0.439	VSIG10	V-set and immunoglobulin domain-containing protein 10
0.004	0.279	-0.024	0.267	WASH6P	WAS protein family homolog 6
-0.043	-0.096	0.272	0.115	WDFY2	WD repeat and FYVE domain-containing protein 2
0.066	-0.092		0.321	XPR1	Xenotropic and polytropic retrovirus receptor 1
			0.432	ZC3H10	Zinc finger CCCH domain-containing protein 10
0.334				ZC3H3	Zinc finger CCCH domain-containing protein 3
0.013		-0.647		ZNF28	Zinc finger protein 28
	-0.327			ZNF419	Isoform 5 of Zinc finger protein 419
0.298	0.247	-0.114	-0.072	ZNF706	Zinc finger protein 706

C, control cell; M, MAG-treated cell.
