# Supplementary material for: Bacterial Magnetosomes Release Iron Ions and Induce Regulation of Iron Homeostasis in Endothelial Cells
Source: Nanomaterials (Basel). 2022 Nov 13;12(22):3995. doi: 10.3390/nano12223995 (PMC9695978; doi:10.3390/nano12223995)
Supplement: Supplementary file 1 [file nanomaterials-12-03995-s001.zip › Table S7.pdf]

Table S7. Cellular Component enrichment of the differential expressed proteins from 4 time points of ECs under MAGs treatment.

ID	Term	Ontology Source	P Value*	% Associated Genes	Nr. Genes	Associated Genes Found
GO:0005768	endosome	GO_CellularComponent-EBI-UniProt	9.14E-05	2.02	22	[ANTXR2, AP1B1, AP4M1, C9orf72, CD63, CLCN5, HSPA8, LAPTM4A, LGMN, LTF, MICALL2, PLPP2, PSAP, RNF13, SDF4, SH3TC2, SLC11A2, TBC1D16, TFRC, UBA52, WASH6P, WDFY2]
GO:1903561	extracellular vesicle	GO_CellularComponent-EBI-UniProt	3.56E-04	1.47	34	[AP4M1, CANX, CD40, CD63, CPQ, DNASE2, FTH1, FTL, GBA, H3-3A, HRNR, HSPA8, ITGB8, LBP, LGMN, LTF, MYH14, MYL6, PLPP1, PPT1, PSAP, PTTG1IP, RNH1, RPS29, SAA1, SCARB1, SDF4, SLC11A2, SRI, TFRC, TGFBI, TM7SF3, TSPAN3, UBA52]
GO:0005764	lysosome	GO_CellularComponent-EBI-UniProt	6.60E-08	3.00	24	[AP1B1, ATRAID, C9orf72, CD63, CLCN5, CPQ, DNASE2, FTH1, FTL, GBA, HPSE, HRNR, HSPA8, LAPTM4A, LGMN, LIPA, PPT1, PSAP, RNF13, SCARB1, SHKBP1, SLC11A2, SLC36A1, UBA52]
GO:0005765	lysosomal membrane	GO_CellularComponent-EBI-UniProt	6.60E-08	3.19	14	[AP1B1, ATRAID, CD63, CLCN5, GBA, HPSE, HSPA8, LAPTM4A, PSAP, RNF13, SCARB1, SLC11A2, SLC36A1, UBA52]
GO:0005775	vacuolar lumen	GO_CellularComponent-EBI-UniProt	8.05E-05	4.57	9	[FTL, GBA, HPSE, HRNR, HSPA8, LGMN, LIPA, PPT1, PSAP]
GO:0043202	lysosomal lumen	GO_CellularComponent-EBI-UniProt	8.05E-05	6.48	7	[GBA, HPSE, HSPA8, LGMN, LIPA, PPT1, PSAP]

* *p* -Value was corrected with Bonferroni step down
