# Supplementary material for: Bacterial Magnetosomes Release Iron Ions and Induce Regulation of Iron Homeostasis in Endothelial Cells
Source: Nanomaterials (Basel). 2022 Nov 13;12(22):3995. doi: 10.3390/nano12223995 (PMC9695978; doi:10.3390/nano12223995)
Supplement: Supplementary file 1 [file nanomaterials-12-03995-s001.zip › Table S8.pdf]

Table S8. Function and pathway enrichment of the differential expressed proteins from 4 time points of ECs under MAGs treatment.

ID	Database	Term	P Value*	% Associated Genes	Nr. Genes	Associated Genes Found
KEGG:04142	KEGG	Lysosome	0.00	8.59	11	[AP1B1, AP4M1, CD63, DNASE2, GBA, LAPTM4A, LGMN, LIPA, PPT1, PSAP, SLC11A2]
WP:2007	WikiPathways	Iron metabolism in placenta	0.00	33.33	4	[FTH1, IREB2, SLC11A2, TFRC]
R-HSA:199992	REACTOME_Pathways	trans-Golgi Network Vesicle Budding	0.00	9.72	7	[AP1B1, AP4M1, DNASE2, FTH1, FTL, HSPA8, TFRC]
R-HSA:917937	REACTOME_Pathways	Iron uptake and transport	0.00	10.34	6	[FTH1, FTL, IREB2, SLC11A2, TFRC, LTF, UBA52]
R-HSA:432722	REACTOME_Pathways	Golgi Associated Vesicle Biogenesis	0.01	8.93	5	[AP1B1, FTH1, FTL, HSPA8, TFRC]
R-HSA:432720	REACTOME_Pathways	Lysosome Vesicle Biogenesis	0.01	11.43	4	[AP1B1, AP4M1, DNASE2, HSPA8]
GO:0106235	GO_MolecularFunction-EBI-UniProt	ceramide-1-phosphate phosphatase activity	0.01	66.67	2	[PLPP1, PLPP2]
R-HSA:2173782	REACTOME_Pathways	Binding and Uptake of Ligands by Scavenger Receptors	0.01	9.52	4	[FTH1, FTL, SAA1, SCARB1]
KEGG:04216	KEGG	Ferroptosis	0.01	9.76	4	[FTH1, FTL, SLC11A2, TFRC]
WP:4313	WikiPathways	Ferroptosis	0.01	10.00	4	[FTH1, FTL, SLC11A2, TFRC]
R-HSA:382551	REACTOME_Pathways	Transport of small molecules	0.02	2.20	16	[APOC1, CLCN5, FTH1, FTL, IREB2, LIPA, MBTPS1, SCARB1, SLC11A2, SLC35B4, SLC35D1, SLC36A1, SLC39A1, SRI, TFRC, UBA52]
R-HSA:977225	REACTOME_Pathways	Amyloid fiber formation	0.02	5.50	6	[H3-3A, H3C15, LTF, SAA1, TGFBI, UBA52]
R-HSA:380270	REACTOME_Pathways	Recruitment of mitotic centrosome proteins and complexes	0.02	6.10	5	[CEP78, CNTRL, MZT2A, PLK1, SSNA1]
R-HSA:380287	REACTOME_Pathways	Centrosome maturation	0.02	6.10	5	[CEP78, CNTRL, MZT2A, PLK1, SSNA1]
R-HSA:2644605	REACTOME_Pathways	FBXW7 Mutants and NOTCH1 in Cancer	0.02	40.00	2	[NOTCH1, RBX1]
R-HSA:2644607	REACTOME_Pathways	Loss of Function of FBXW7 in Cancer and NOTCH1 Signaling	0.02	40.00	2	[NOTCH1, RBX1]
R-HSA:2565942	REACTOME_Pathways	Regulation of PLK1 Activity at G2/M Transition	0.02	5.68	5	[CEP78, CNTRL, PLK1, SSNA1, UBA52]
GO:0042392	GO_MolecularFunction-EBI-UniProt	sphingosine-1-phosphate phosphatase activity	0.03	40.00	2	[PLPP1, PLPP2]
R-HSA:3000471	REACTOME_Pathways	Scavenging by Class B Receptors	0.03	33.33	2	[SAA1, SCARB1]
R-HSA:5625740	REACTOME_Pathways	RHO GTPases activate PKNs	0.03	5.26	5	[H3-3A, H3C15, MYH14, MYL6, PKN3]

ID	Database	Term	P Value*	% Associated Genes	Nr. Genes	Associated Genes Found
R-HSA:69275	REACTOME_Pathways	G2/M Transition	0.03	3.57	7	[CEP78, CNTRL, MZT2A, PLK1, RBX1, SSNA1, UBA52]
R-HSA:453274	REACTOME_Pathways	Mitotic G2-G2/M phases	0.03	3.54	7	[CEP78, CNTRL, MZT2A, PLK1, RBX1, SSNA1, UBA52]
R-HSA:380320	REACTOME_Pathways	Recruitment of NuMA to mitotic centrosomes	0.03	5.32	5	[CEP78, CNTRL, MZT2A, PLK1, SSNA1]
GO:0030021	GO_MolecularFunction-EBI-UniProt	extracellular matrix structural constituent conferring compression resistance	0.03	12.00	3	[PODN, PRG4, TUFT1]
WP:2361	WikiPathways	Gastric Cancer Network 1	0.03	10.34	3	[H3-3A, KIF15, NOTCH1]
R-HSA:5653656	REACTOME_Pathways	Vesicle-mediated transport	0.04	2.08	13	[AP1B1, AP4M1, DNASE2, FTH1, FTL, GALNT1, HSPA8, KIF15, SAA1, SCARB1, TBC1D16, TFRC, UBA52]
R-HSA:174824	REACTOME_Pathways	Plasma lipoprotein assembly, remodeling, and clearance	0.04	5.80	4	[APOC1, LIPA, MBTPS1, SCARB1]
R-HSA:380259	REACTOME_Pathways	Loss of Nlp from mitotic centrosomes	0.04	5.71	4	[CEP78, CNTRL, PLK1, SSNA1]
R-HSA:380284	REACTOME_Pathways	Loss of proteins required for interphase microtubule organization from the centrosome	0.04	5.71	4	[CEP78, CNTRL, PLK1, SSNA1]
R-HSA:727802	REACTOME_Pathways	Transport of nucleotide sugars	0.04	22.22	2	[SLC35B4, SLC35D1]
R-HSA:8964043	REACTOME_Pathways	Plasma lipoprotein clearance	0.04	9.09	3	[APOC1, LIPA, SCARB1]
R-HSA:201681	REACTOME_Pathways	TCF dependent signaling in response to WNT	0.05	3.00	7	[H3-3A, H3C15, LGR5, PYGO1, RBBP5, RBX1, UBA52]
R-HSA:8854518	REACTOME_Pathways	AURKA Activation by TPX2	0.05	5.48	4	[CEP78, CNTRL, PLK1, SSNA1]

* *p* -Value was corrected with Benjamini-Hochberg
